# A novel prognostic signature of immune-related lncRNA pairs in lung adenocarcinoma

**DOI:** 10.1038/s41598-021-96236-4

**Published:** 2021-08-18

**Authors:** Yang Liu, Qiuhong Wu, Xuejiao Fan, Wen Li, Xiaogang Li, Hui Zhu, Qinghua Zhou, Jinming Yu

**Affiliations:** 1 Lung Cancer Center, West China Hospital, Sichuan University, Chengdu, 610041 Sichuan China; 2 Department of Radiation Oncology, Shandong Cancer Hospital and Institute, Shandong First Medical University and Shandong Academy of Medical Sciences, Jinan, 250117 Shandong China; 3 Department of Rheumatology and Immunology, West China Hospital, Sichuan University, Chengdu, 610041 Sichuan China; 4 Clinical Research Management Department, West China Hospital, Sichuan University, Chengdu, 610041 Sichuan China

**Keywords:** Non-small-cell lung cancer, Prognostic markers, Cancer epigenetics

## Abstract

Lung adenocarcinoma (LUAD) is the most common subtype of lung cancer, but the prognosis of LUAD patients remains unsatisfactory. Here, we retrieved the RNA-seq data of LUAD cohort from The Cancer Genome Atlas (TCGA) database and then identified differentially expressed immune-related lncRNAs (DEirlncRNAs) between LUAD and normal controls. Based on a new method of cyclically single pairing along with a 0-or-1 matrix, we constructed a novel prognostic signature of 8 DEirlncRNA pairs in LUAD with no dependence upon specific expression levels of lncRNAs. This prognostic model exhibited significant power in distinguishing good or poor prognosis of LUAD patients and the values of the area under the curve (AUC) were all over 0.70 in 1, 3, 5 years receiver operating characteristic (ROC) curves. Moreover, the risk score of the model could serve as an independent prognostic factor for patients with LUAD. In addition, the risk model was significantly associated with clinicopathological characteristics, tumor-infiltrating immune cells, immune-related molecules and sensitivity of anti-tumor drugs. This novel signature of DEirlncRNA pairs in LUAD, which did not require specific expression levels of lncRNAs, might be used to guide the administration of patients with LUAD in clinical practice.

## Introduction

Lung cancer remains the leading cause of cancer-related deaths worldwide. Non-small-cell lung cancer (NSCLC) accounts for 80% of lung cancer cases, of which lung adenocarcinoma (LUAD) is the most common subtype^1,2^. LUAD patients are often diagnosed at advanced disease stage. Despite advances have been achieved in the treatment of advanced LUAD, such as molecularly targeted therapy and immunotherapy, the overall survival (OS) of advanced LUAD patients is still unsatisfactory, with 5-year OS rate less than 20%^3–5^. Therefore, it is urgent to develop efficient biomarkers to accurately predict the prognosis of LUAD patients.

In recent years, tumor microenvironment (TME) has been proved to be involved in the occurrence and development of malignant tumors^6,7^. And cancer cells possess various immune resistance mechanisms to escape the surveillance and elimination of anti-tumor immunity, namely immunoediting, such as downregulation of costimulatory molecules and major histocompatibility complex (MHC), upregulation of immunosuppressive ligands, and lack of appropriate inflammatory cytokines to attract anti-tumor immune cells^8^. In addition, there are also some inhibitory immune cells in the TME, such as regulatory T cells (Tregs) and myelogenic suppressor cells (MDSCs), which are also the reasons for cancer cells to escape immune surveillance^9,10^. Therefore, evaluating the proportion of tumor-infiltrating immune cells in the TME can not only improve our understanding of the pathogenesis of cancers, but also help us to develop novel biomarkers to predict the effectiveness of immunotherapy.

Long non-coding RNAs (lncRNAs), longer than 200 nucleotides in length, are a subtype of non-coding RNAs (ncRNAs) that can regulate gene expression at both transcriptional and post-transcriptional levels. Accumulating evidences have suggested that lncRNAs can participate in the occurrence and development of cancers^11,12^. In addition, lncRNAs can regulate the TME and play an essential role in tumor immunity^13,14^. For instance, Li et al. systematically analyzed immune-related lncRNAs (irlncRNAs) in 33 cancer types and found that several lncRNAs were significantly associated with immune cell infiltration^15^. Furthermore, recent studies have indicated that signatures of irlncRNAs could serve as prognostic biomarkers of the survival of cancers, such as breast cancer, colon cancer, pancreatic cancer as well as LUAD^16–20^. By using the gene expression data and clinical data in The Cancer Genome Atlas (TCGA), Miao et al. constructed a signature of immune-related six-lncRNAs to predict the OS of patients with LUAD and this signature could act as an independent prognostic factor^18^. Similarly, Li et al. identified a seven irlncRNA signature for predicting OS of LUAD patients and this prognostic model could well distinguish good or poor survival of patients with LUAD^20^. However, the majority of these lncRNA signatures were based on the quantitative expression levels of lncRNAs. Since different experimental platforms and batches may lead to the heterogeneity of gene expression data, it is inappropriate to apply one gene signature from specific platform to another gene expression data without normalization and correction, limiting the clinical application values of these lncRNA signatures.

In this study, we downloaded the RNA-seq data and corresponding clinical information from TCGA database and constructed a novel signature of irlncRNA pairs in LUAD. Then, the predictive value of this prognostic prediction model was estimated. Considering that it only needed the relative expression levels between the two in each pair, this irlncRNA pair signature was not affected by the heterogeneity of different experimental platforms, making it more valuable for clinical application. Meanwhile, we also evaluated the correlation between this risk model and tumor-infiltrating immune cells or immune-related molecules, as well as the relationship between the model and chemotherapeutic efficacy of LUAD.

## Methods and materials

### Data download and processing

The transcriptome profiling (RNA-seq) data and corresponding clinical information of LUAD samples were downloaded from TCGA database (https://portal.gdc.cancer.gov/). Patients without corresponding RNA-seq data or clinical information or with a survival time < 30 days were excluded. GTF files were retrieved from Ensembl dataset (http://asia.ensembl.org), which were used to transform the Ensembl ID of genes into homologous gene symbols and to distinguish mRNAs and lncRNAs. And the gene list of immune-related genes (ir-genes) was obtained from the ImmPort database (http://www.immport.org).

### irlncRNAs identification and differential expression analysis

Pearson correlation analysis was used to investigate the correlation between ir-genes and lncRNAs, and the absolute value of correlation coefficient > 0.5 and P < 0.001 were used as the criteria to identify irlncRNAs. Then, differentially expressed irlncRNAs (DEirlncRNAs) between LUAD and normal controls were identified by “limma” package of R software (v.4.0.3)^21^. The Benjamini-Hochberg (BH) false discovery rate (FDR) method was conducted to adjust P values^22^. Those irlncRNAs that meet the screening criteria (|log fold change (FC)|> 2, and adjusted P value < 0.05) were identified as DEirlncRNAs. Then, the “ggplot2” and “pheatmap” packages of R software were used to depict the volcano plot and heatmap of DEirlncRNAs.

### Establishment of DEirlncRNA pairs

We cyclically single paired the DEirlncRNAs and constructed a 0-or-1 matrix. For one DEirlncRNA pair (lncRNA A|lncRNA B), if the expression level of lncRNA A was higher than lncRNA B, the value of this pair was defined as 1. On the contrary, the value of lncRNA A|lncRNA B was defined as 0 if the expression level of lncRNA A was lower than lncRNA B. Then, those DEirlncRNA pairs with 0-or-1 less than 20% or more than 80% of total pairs were excluded, since only pairs with a certain rank were closely associated with the prognosis of patients.

### Identification of survival-related DEirlncRNA pairs and construction of prognostic prediction model

We conducted univariate Cox regression analysis to identify survival-related DEirlncRNAs (P < 0.05) by using “survival” and “survminer” R packages. Then, by using "glmnet" R package, we performed the least absolute shrinkage and selection operator (LASSO) regression analysis to select the most significant DEirlncRNA pairs out of all OS-related DEirlncRNA pairs. This process was performed to avoid model overfitting, and several optimal DEirlncRNA pairs with non-zero coefficients were used as candidates to construct the prognosis predictive model by multivariate Cox regression analysis^23^. In the risk model, the risk score of each LUAD patients was calculated based on the value of lncRNA A|lncRNA B (0 or 1) and the corresponding multivariate Cox regression coefficient. The formula was as follows: $\mathrm{Riskscore}={\sum }_{i}^{n}\left(\mathrm{lncRNA A}|\mathrm{lncRNA B}\right)\mathrm{i}\beta i$, where *β* represented the regression coefficient.

### Evaluation of prognostic prediction model

The 1-, 3-, and 5-year receiver operating characteristic (ROC) curve analyses were conducted and the values of the area under the curve (AUC) were calculated by using “survival ROC” R package to estimate the specificity and sensitivity of the model. The point of maximum Youden Index in the 1-year ROC curve were defined as the cut-off point, and its corresponding risk score was used to divide patients with LUAD into high-risk and low-risk subgroups^24^. The formula was as follows: $\mathrm{Youden Index}=\mathrm{Sensitivity}+\mathrm{Specificity}-1$. The Kaplan-Meier (K-M) analysis and log-rank test were conducted to compare the survival difference between high-risk and low-risk patients. Then, LUAD samples were reordered based on the risk score, and the risk score curve and the survival status distribution were plotted. Furthermore, the independent prognostic roles of risk score and clinicopathological characteristics, such as age, gender, and TNM stage, were assessed by univariate and multivariate Cox regression analysis. In addition, the relationship between risk model and clinicopathological characteristics was assessed by chi-square test, which was visualized by heatmap. And Wilcoxon rank-sum test was used to compare the difference of risk scores among patients with different TNM stages.

### Comprehensive analysis of tumor-infiltrating immune cells and immune-related molecules

The status of immune cell infiltration of LUAD samples was estimated using the online platform of Tumor Immune Estimation Resource 2.0 (TIMER2.0, http://timer.cistrome.org/), which integrated seven quantification methods of immune infiltration estimations including TIMER, xCell, quanTIseq, MCP-counter, EPIC, CIBERSORT-ABS, and CIBERSORT^25–27^. Then, we performed Spearman correlation analysis to explore the relationship between risk score and tumor-infiltrating immune cells, and P < 0.05 was considered statistically significant. Furthermore, the correlation between risk model and the expression levels of genes associated with immune checkpoint inhibitors (ICIs), including cytotoxic T-lymphocyte associated protein 4 (*CTLA4*), programmed cell death 1 (*PDCD1*), lymphocyte activating 3 (*LAG3*), and hepatitis A virus cellular receptor 2 (*HAVCR2*), was evaluated by the Wilcoxon rank-sum test and visualized by "ggpubr" package in R software.

### Sensitivity evaluation of anti-tumor drugs

We obtained the Immunophenoscores (IPS) of patients with LUAD from the Cancer Immunome Database (TCIA, https://tcia.at/home). And the relationship between IPS and the risk signature was investigated by the Wilcoxon rank-sum test. Furthermore, the half inhibitory concentration (IC50) of common anti-tumor drugs was analyzed by "pRRophetic" package in R software. To explore the clinical value of this risk model in the treatment of LUAD, the Wilcoxon rank-sum test was conducted to estimate the difference of IC50 of anti-tumor drugs, including paclitaxel, docetaxel, gemcitabine, vinorelbine, etoposide, cisplatin, gefitinib, and erlotinib, between the high-risk and low-risk subgroups.

## Results

### Identification of DEirlncRNAs

**Figure 1:**
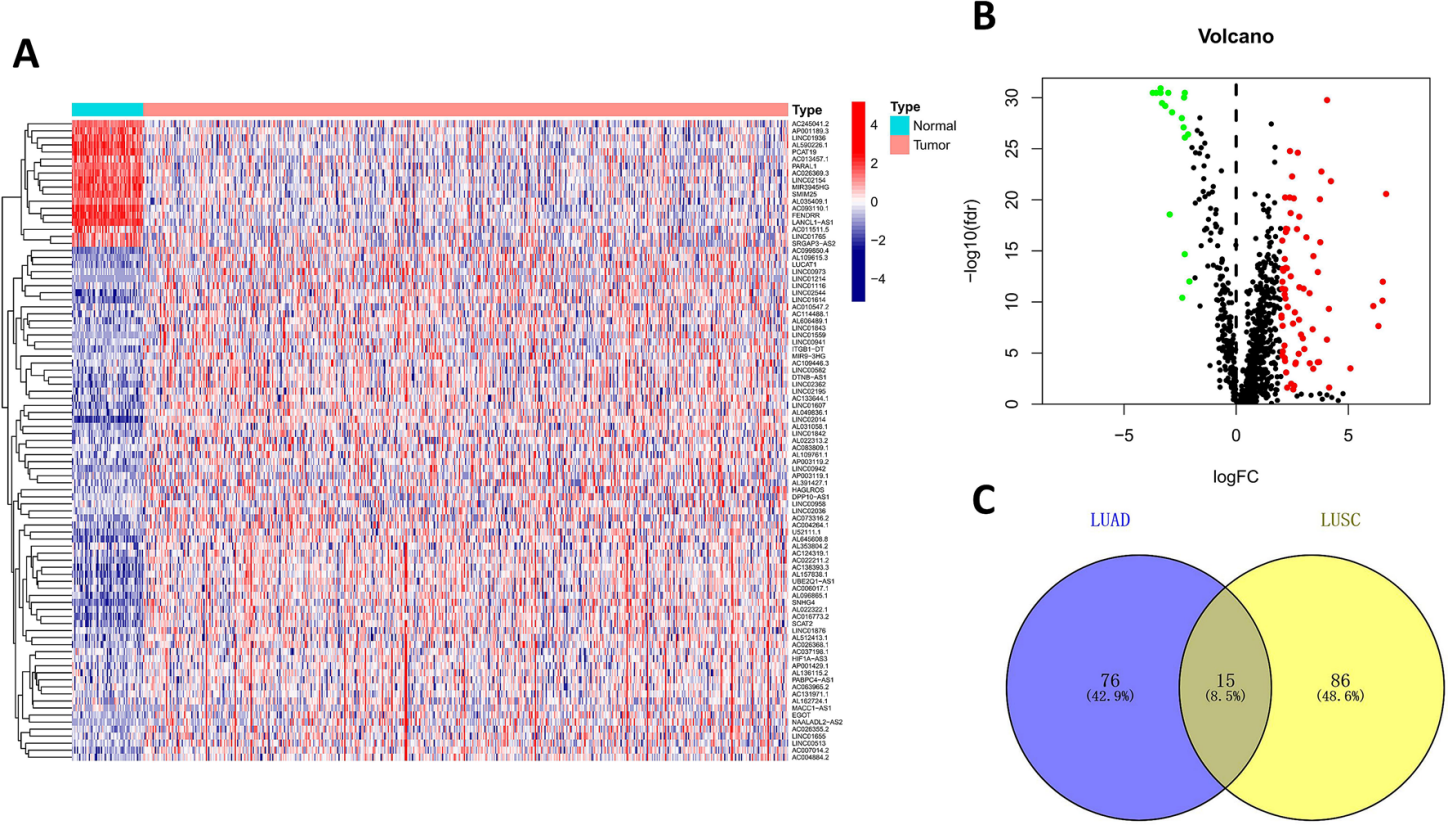
DEirlncRNAs between LUAD samples and normal controls. (**A**) The heat map of DEirlncRNAs. (**B**) The volcano plot of DEirlncRNAs. (**C**) The intersection of DEirlncRNAs between LUAD and LUSC. DEirlncRNAs, differentially expressed immune-related lncRNAs; LUAD, lung adenocarcinoma; LUSC, lung squamous cell carcinoma.

Totally, this study included 490 LUAD samples and 59 normal controls from TCGA database, and the characteristics of 490 patients with LUAD (228 men and 262 women) were shown in Table 1. The majority of patients with LUAD in TCGA were in early stages, including stage I in 263 cases (53.67%), stage II in 115 cases (23.47%), stage III in 79 cases (16.12%) and stage IV in 25 cases (5.10%). According to the co-expression analysis between lncRNAs and ir-genes, we identified 1035 irlncRNAs (Supplementary Table S1). Among them, 91 DEirlncRNAs were identified between LUAD and normal controls, of which 73 DEirlncRNAs were upregulated and 18 DEirlncRNAs were downregulated (Fig. 1A and 1B and Supplementary Table S2). Besides, we also analyzed the irlncRNAs in lung squamous cell carcinoma (LUSC) and identified 101 DEirlncRNAs between LUSC and normal controls including 62 upregulated and 39 downregulated DEirlncRNAs (Supplementary Figure S1 and Supplementary Table S3 and S4). In addition, the intersection of DEirlncRNAs between LUAD and LUSC was analyzed using the Venny 2.1.0 online database (https://bioinfogp.cnb.csic.es/tools/venny/index.html), and there were only 15 overlapping DEirlncRNAs between LUAD and LUSC (Fig. 1C and Supplementary Table S5). These results indicated that the DEirlncRNAs of LUAD identified in this study had high specificity in LUAD.

**Table 1:** Characteristics of patients with LUAD from TCGA database.

Characteristics	No. of patients	%
**Age at diagnosis (years)**		
≤ 65	231	47.14
> 65	249	50.82
Unknown	10	2.04
**Sex**		
Male	228	46.53
Female	262	53.47
**Stage**		
I	263	53.67
II	115	23.47
III	79	16.12
IV	25	5.10
Unknown	8	1.63
**T category**		
T1	163	33.27
T2	263	53.67
T3	43	8.78
T4	18	3.67
Tx	3	0.61
**N category**		
N0	317	64.69
N1	92	18.78
N2	68	13.88
N3	2	0.41
Nx	11	2.24
**M category**		
M0	324	66.12
M1	24	4.90
Mx	142	28.98

LUAD, lung adenocarcinoma; TCGA, The Cancer Genome Atlas.

### Prognostic prediction model of LUAD based on survival-related DEirlncRNA pairs

Totally, 3440 valid DEirlncRNA pairs were identified by the method of cyclically single pairing along with a 0-or-1 matrix. Then 619 survival-related DEirlncRNA pairs were determined by univariate Cox regression analysis (Supplementary Table S3). LASSO regression analysis was conducted to screen the optimal survival-related DEirlncRNA pairs, which were used as candidates to construct the prognosis predictive model by multivariate Cox regression analysis. Finally, a total of 8 DEirlncRNA pairs were selected to construct the prediction model of LUAD (Table 2 and Supplementary Table S4).

**Table 2:** Immune-related lncRNA pairs used for construction of prognostic model.

lncRNA pairs	Coef.	HR	HR.95L	HR.95H	p value
LINC00958|HIF1A-AS3	− 0.559056064	0.571748503	0.404824948	0.807500506	0.00150468
ITGB1-DT|FENDRR	0.372948657	1.452009787	1.060841449	1.9874152	0.019871749
AC004264.1|LINC02036	0.39121285	1.478773237	1.076738936	2.030919672	0.015661584
AC026355.2|AL049836.1	− 0.42262269	0.655325847	0.480477131	0.893803134	0.00760824
LINC02195|LINC01116	− 0.513869512	0.598176444	0.437208026	0.81840917	0.00131407
LINC02362|LINC00941	− 0.534861917	0.585750167	0.411859423	0.833059143	0.002916854
LINC01116|LINC02154	0.621835544	1.86234332	1.229948538	2.819892489	0.003305756
AL606489.1|AC006017.1	0.443770725	1.558573103	1.084414844	2.240056129	0.016491365

**Figure 2:**
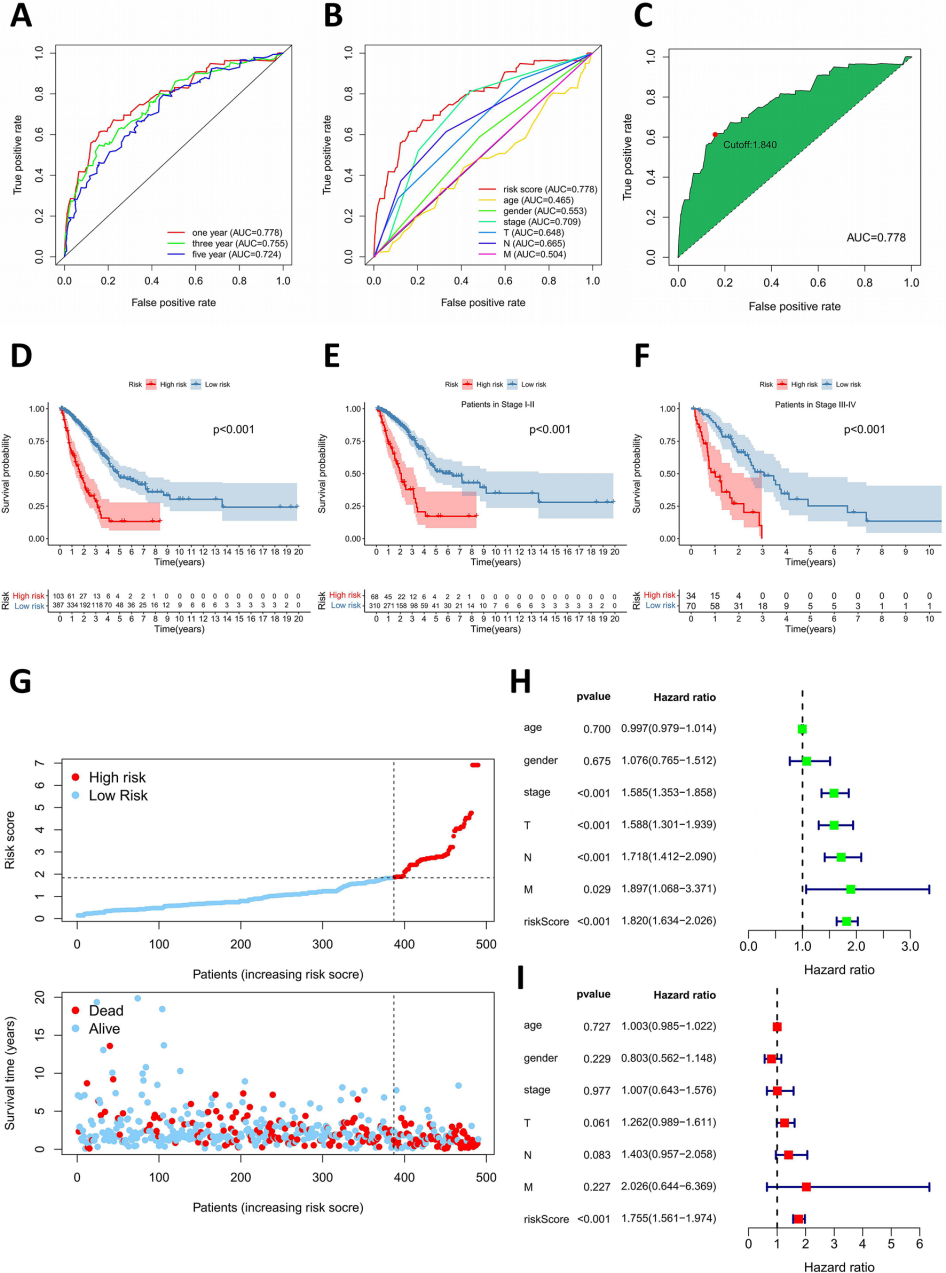
The prognostic prediction model based on survival-related DEirlncRNA pairs in LUAD. (**A**) The 1-, 3-, and 5-year ROC curves of the risk model with AUC values. (**B**) The 1-year ROC curve of the risk model and other clinicopathological characteristics. (**C**) The cut-off risk score identified by Youden Index. (**D**) The Kaplan-Meier survival analysis for all LUAD patients. (**E**) The Kaplan-Meier survival analysis for LUAD patients in Stage I-II. (**F**) The Kaplan-Meier survival analysis for LUAD patients in Stage III-IV. (**G**) The risk score curve and the distribution of survival status of LUAD patients. (**H**, **I**) The univariate and multivariate Cox regression analysis to evaluate the independent prognostic value. DEirlncRNAs, differentially expressed immune-related lncRNAs; LUAD, lung adenocarcinoma; ROC, receiver operating characteristic; AUC, area under the curve.

To validate the accuracy of the model, the 1-, 3-, and 5-year ROC curves were plotted, and they revealed that this model was efficient in predicting the prognosis of LUAD patients since AUC values were all over 0.70 (Fig. 2A). When compared with other clinicopathological characteristics, this risk model possessed the greatest prognostic power with the maximum AUC value of 0.778 in 1-year ROC curve (Fig. 2B). According to the cut-off risk score identified by Youden Index, LUAD patients were divided into high-risk and low-risk subgroups (Fig. 2C). The K-M analysis indicated that this prediction model could efficiently distinguish good or poor survival of patients with LUAD (P < 0.001) (Fig. 2D). Subgroup analysis was also performed, which showed that this risk model exhibited significant power in distinguishing good or poor survival of LUAD patients not only in Stage I-II subgroup but also in Stage III-IV subgroup (Fig. 2E and 2F). And the risk score curve and the distribution of survival status revealed that high-risk patients had a relatively worse clinical outcome (Fig. 2G). In addition, the univariate Cox regression analysis demonstrated that clinical stage (p < 0.001), T stage (p < 0.001), N stage (p < 0.001), M stage (p = 0.029) and riskScore (p < 0.001) were significantly associated with poor prognosis, whereas only riskScore (p < 0.001) was statistically different in multivariate Cox regression analysis, implying that only the risk score of the model could serve as an independent prognostic factor for patients with LUAD (Fig. 2H and 2I).

### Clinical significance of prognostic prediction model

**Figure 3:**
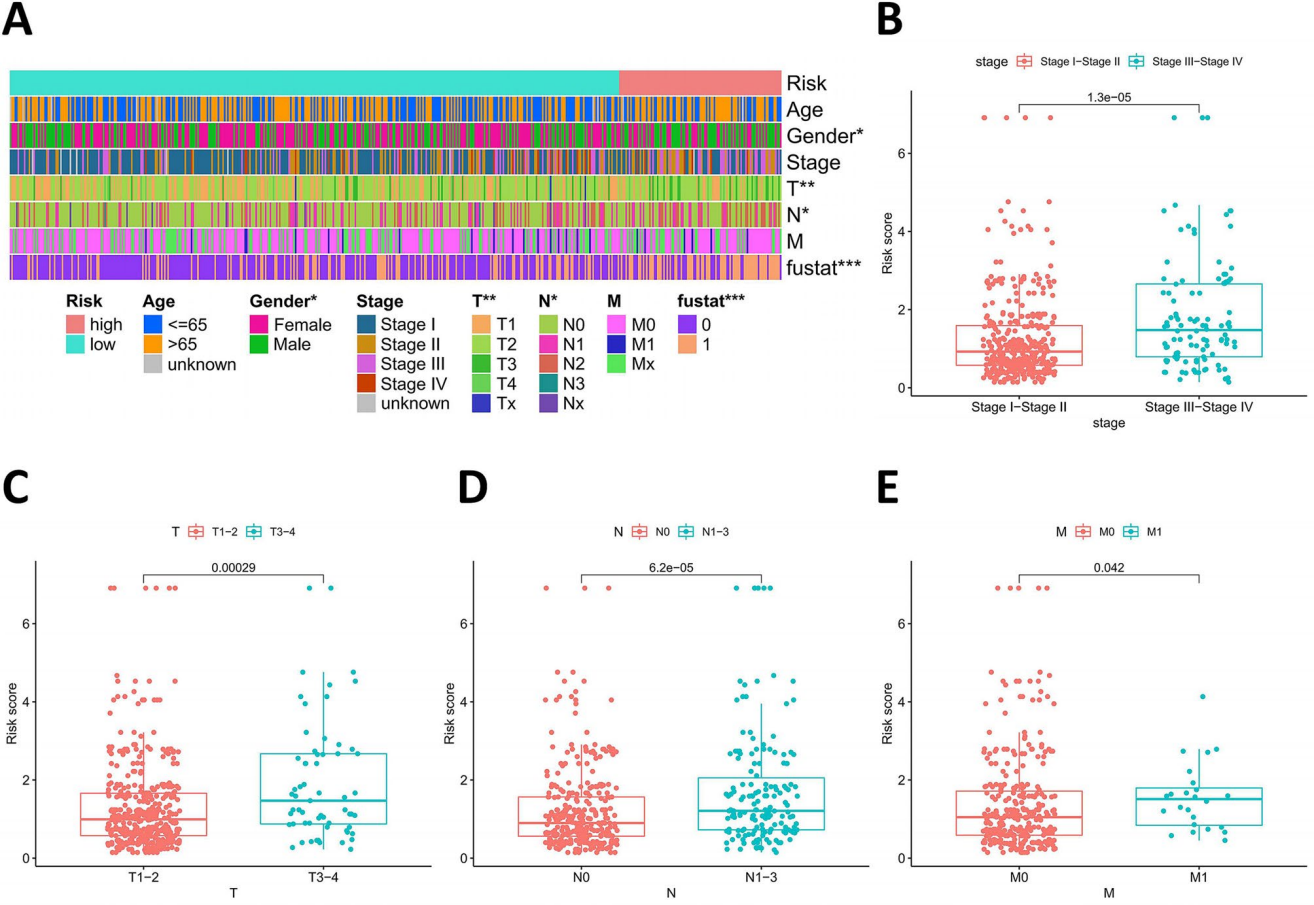
The clinical correlation between risk model and clinicopathological characteristics of LUAD patients. (**A**) The chi-square test showed that gender (P < 0.05), T stage (P < 0.01), N stage (P < 0.05), and survival status (P < 0.001) were significantly related to the risk model. (**B-E**) The Wilcoxon rank-sum test showed that the risk scores of LUAD patients were significantly related to clinical stage (**B**), status of primary tumor (**C**), status of lymph node metastasis (**D**), and status of distant metastasis (**E**). LUAD, lung adenocarcinoma.

To estimate the correlation between risk model and clinicopathological characteristics of LUAD patients, we performed chi-square test, which showed that gender (P < 0.05), T stage (P < 0.01), N stage (P < 0.05), and survival status (P < 0.001) were significantly related to the risk model (Fig. 3A). Furthermore, according to the Wilcoxon rank-sum test, the risk scores of LUAD patients were significantly related to clinical stage, status of primary tumor, status of lymph node metastasis, and status of distant metastasis (all P < 0.05) (Fig. 3B-3E).

### Correlation between risk model and tumor-infiltrating immune cells or immune-related molecules

**Figure 4:**
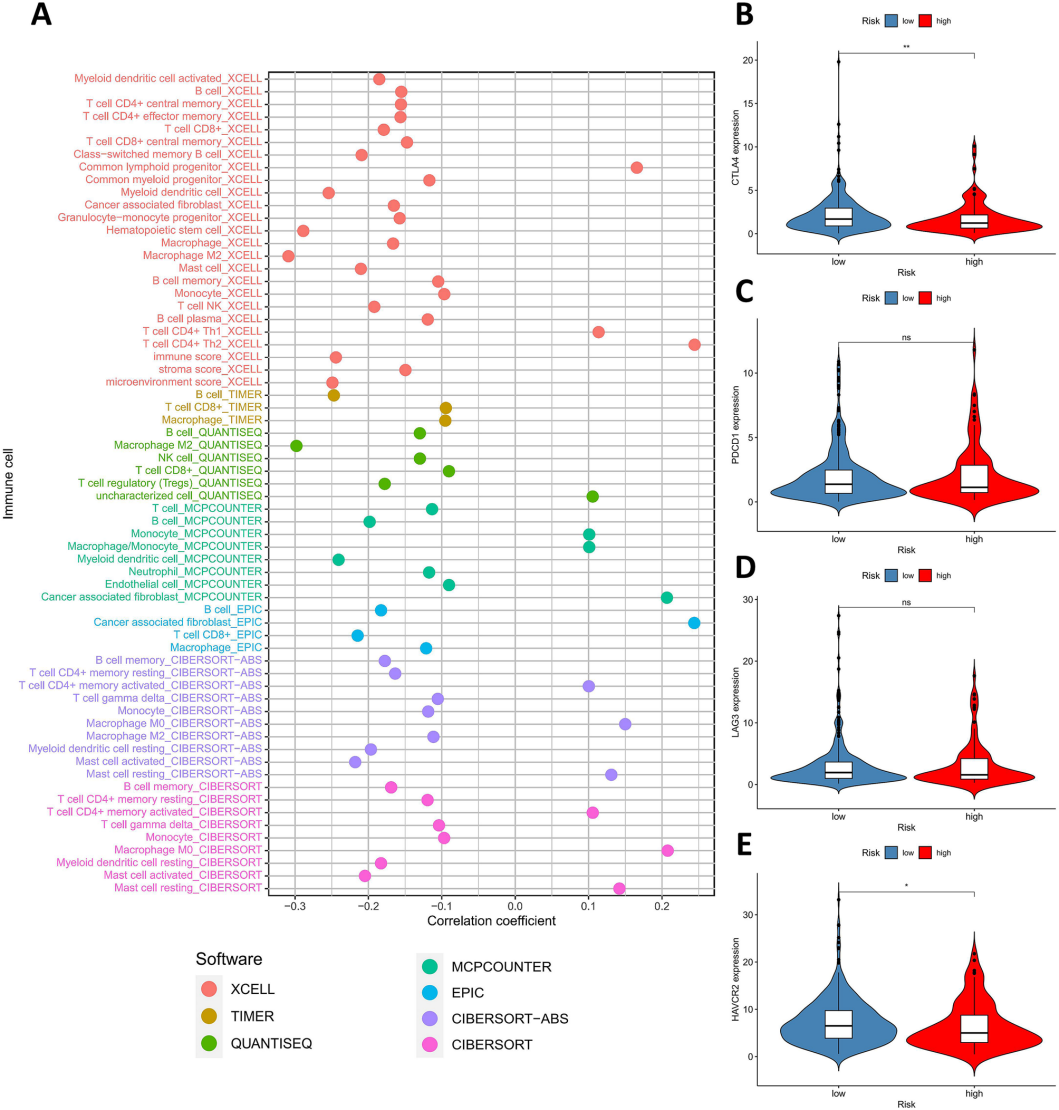
The immune significance of the risk model in LUAD. (**A**) The correlation between risk score and tumor-infiltrating immune cells, which were analyzed by seven different quantification methods of immune infiltration estimations including TIMER, xCell, quanTIseq, MCP-counter, EPIC, CIBERSORT-ABS, and CIBERSORT. (**B-E**) The relationship between risk model and the expression levels of immune-related molecules, including *CTLA4* (**B**), *PDCD1* (**C**), *LAG3* (**D**), and *HAVCR2* (**E**). LUAD, lung adenocarcinoma; *CTLA4*, cytotoxic T-lymphocyte associated protein 4; *PDCD1*, programmed cell death 1; *LAG3*, lymphocyte activating 3; *HAVCR2*, hepatitis A virus cellular receptor 2.

The correlation between risk score and tumor-infiltrating immune cells was estimated via Spearman correlation analysis, which showed that the risk scores of LUAD patients were more negatively associated with the tumor-infiltrating immune cells, such as B cells, CD8 + T cells, and monocytes. However, some other immune cells, such as M0 macrophages, CD4 + T cells, and cancer associated fibroblasts, were positively related to the risk scores of patients with LUAD (Fig. 4A). We also investigated the relationship between the risk model and immune-related molecules and found that the expression levels of *CTLA4* gene (P < 0.01) and *HAVCR2* gene (P < 0.05) were significantly correlated with the risk model (Fig. 4B-4E).

### Relationship between risk model and sensitivity of anti-tumor drugs

**Figure 5:**
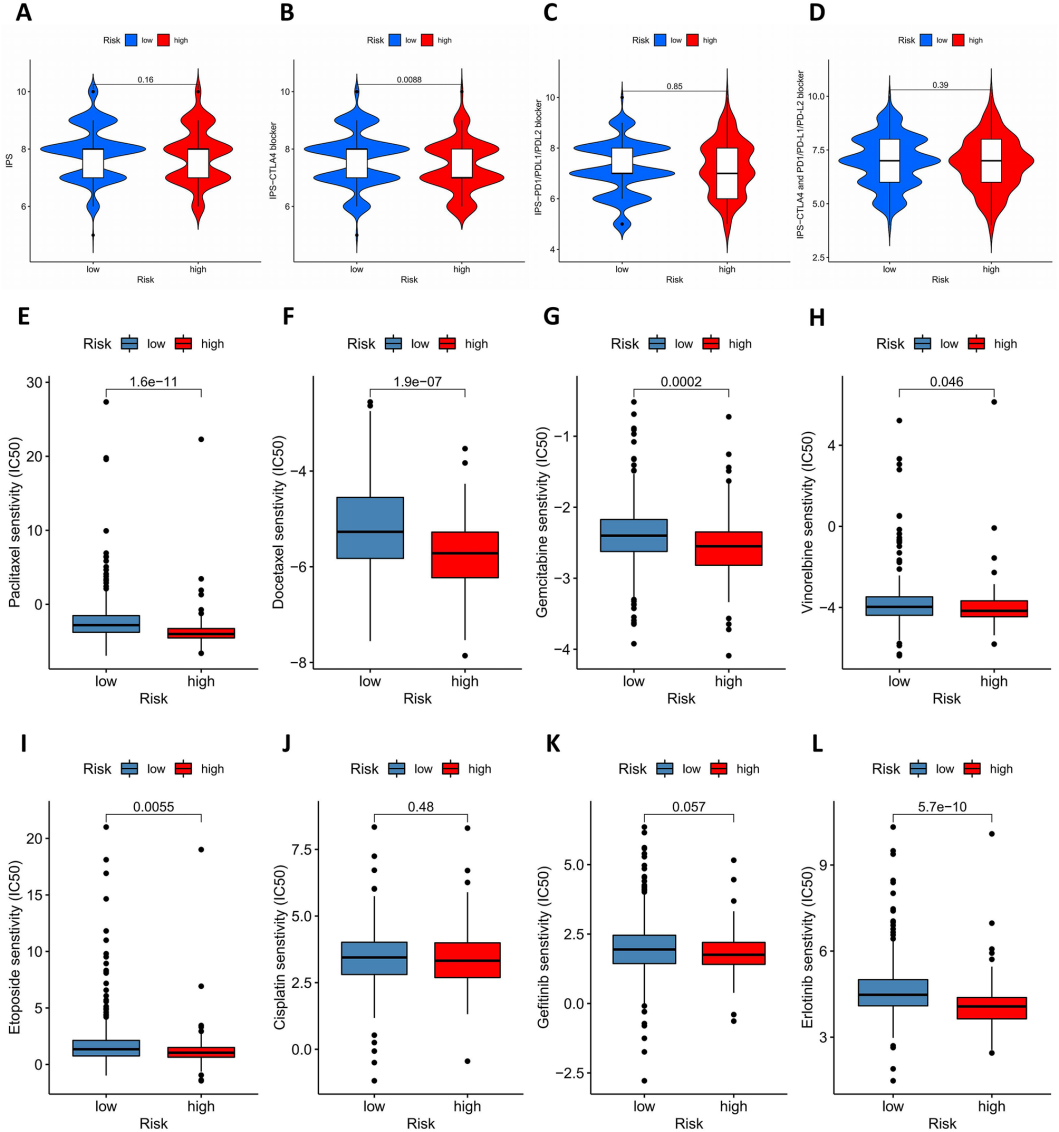
The Relationship between risk model and sensitivity of anti-tumor drugs. (**A-D**) The association between IPS and the immune-related risk signature of LUAD patients. (**E-L**) The difference of IC50 of anti-tumor drugs, including paclitaxel (**E**), docetaxel (**F**), gemcitabine (**G**), vinorelbine (**H**), etoposide (**I**), cisplatin (**J**), gefitinib (**K**), and erlotinib (**L**), between the high-risk and low-risk subgroups. IPS, Immunophenoscore; LUAD, lung adenocarcinoma; IC50, the half inhibitory concentration.

We used the IPS, IPS-CTLA4 blocker, IPS-PD1/PD-L1/PD-L2 blocker, and IPS-CTLA4 and PD1/PD-L1/PD-L2 blocker to assess the potential application values of ICIs for LUAD. The IPS-CTLA4 blocker was significantly higher in the low-risk group (P < 0.01), implying that patients in low-risk subgroup might have a better opportunity for ICIs treatment, especially for the anti-CTLA4 treatment (Fig. 5A-5D). In addition, the sensitivity of anti-tumor drugs (IC50) was calculated and the difference of IC50 between high-risk and low-risk LUAD patients was evaluated. High-risk subgroup was significantly related to higher sensitivity (lower IC50) of anti-tumor drugs including paclitaxel (P < 0.001), docetaxel (P < 0.001), gemcitabine (P < 0.001), vinorelbine (P < 0.05), etoposide (P < 0.01), and erlotinib (P < 0.001), indicating that this risk model might be used as a biomarker to guide the selection of anti-tumor drugs (Fig. 5E-5L).

## Discussion

LUAD accounts for nearly 40% of all lung cancer cases, and the prognosis of LUAD patients remains unsatisfactory^2,5^. Therefore, developing effective biomarkers with high specificity and sensitivity is of significance to improve the survival of LUAD patients, especially in the era of immunotherapy. Nowadays, with the development of the technology of high-throughput sequencing, many studies have indicated the prognostic value of genome-wide biomarkers in malignant tumors, such as signatures of messenger RNAs (mRNAs), microRNAs (miRNAs), and lncRNAs^28–30^. In particular, immune-related signatures have been proved to have effectively predictive values in the treatment and prognosis of cancers including LUAD^16–20^. However, most of these signatures depend on the exact expression levels of transcripts, which weakens their clinical application values due to the heterogeneity of gene expression data.

In this study, we innovatively conducted a new method of cyclically single pairing along with a 0-or-1 matrix to construct a novel prognostic signature of irlncRNA pairs in LUAD. This novel signature does not require quantitative expression levels of lncRNAs, but only needs to detect the higher or lower expression level of the two lncRNAs in each lncRNA pair, which improves its clinical practicability. Totally, 8 DEirlncRNA pairs were selected to construct the prediction model of LUAD, which was proved to be efficient to predict the survival of LUAD patients. Among these DEirlncRNAs included in the model, some have been revealed to be related to the development of cancers, such as *LINC00958*, FOXF1 adjacent non-coding developmental regulatory RNA (*FENDRR*), *LINC01116*, and *LINC00941*.

LncRNA *LINC00958* was initially identified as an oncogene in bladder cancer^31^, and subsequent studies revealed the overexpression of *LINC00958* in many other malignant tumors, such as hepatocellular carcinoma, pancreatic cancer, gastric cancer, glioma, and cervical cancer^32–36^. In NSCLC, Luo et al. demonstrated that *LINC00958* was highly expressed in both LUAD and LUSC cell lines and it could facilitate the proliferation and migration of NSCLC cells, which was mediated by JNK/c-JUN signaling pathway^37^.

LncRNA *FENDRR*, as a potential tumor suppressor, has been revealed to be downregulated in different cancers, such as gastric cancer, breast cancer, hepatocellular carcinoma as well as NSCLC^38–41^. Zhang et al. demonstrated that *FENDRR* was downregulated in both NSCLC cells and tissues and was negatively related to the prognosis of NSCLC patients. Up-expression of *FENDRR* could inhibit the aggressiveness phenotypes of NSCLC cells, such as proliferation, migration and invasion, via directly binding to miR-761 and regulating the expression of tissue inhibitor of metalloproteinases 2 (*TIMP2*)^41^. Besides, Munteanu et al. indicated that *FENDRR* might also regulate the immune response in macrophages. In detail, the overexpression of *FENDRR* could enhance interferon γ (IFN γ) induced M1 macrophage polarization by modulating signal transducer and activator of transcription 1 (STAT1) activation pathway^42^.

LncRNA *LINC01116* was found to be dysregulated in various human cancers, such as glioma, prostate cancer, breast cancer, and osteosarcoma^43–46^. Recent studies also suggested that *LINC01116* played an oncogenic role in lung cancer. For instance, Zeng et al. demonstrated the upregulation of *LINC01116* in LUAD tissues and cell lines, and short interfering RNAs (siRNAs) induced *LINC01116* knockdown could inhibit the cell proliferation, migration, and epithelial-mesenchymal transition (EMT) of LUAD cells^47^. And Wang et al. found that *LINC0116* overexpression contributed to cisplatin resistance in LUAD^48^. Besides, *LINC01116* also played a significant role in gefitinib resistance of NSCLC via regulating the expression of interferon-induced protein 4 (*IFI4*)^49^.

LncRNA *LINC00941*, also known as MSC upregulated factor (*lncRNA-MUF*), was first identified as an oncogene in gastric cancer by Luo et al. They found that *LINC00941* was overexpression in the tissues of gastric cancer compared with adjacent normal tissues and its aberrant expression was related to invasion depth, TNM stage, and lymphatic metastasis^50^. Consistently, Liu et al. indicated that silence of *LINC00941* could inhibit the proliferation, migration, and invasion of gastric cancer cells^51^. Meanwhile, Wang et al. found that *LINC00941* could act as a competing endogenous RNA (ceRNA) by sponging miR-335-5p to regulate ROCK1-mediated LIMK1/Cofilin-1 signaling, which contributed to the proliferation, migration, invasion, and EMT of pancreatic cancer cells^52^. And in LUAD, *LINC0094* could regulate focal adhesion and PI3K-AKT signaling pathway, and its elevated expression level was related to decreased survival of LUAD patients^53^. However, in this study, the potential regulatory mechanisms of these lncRNAs were not clearly elucidated. LncRNAs play significant roles in maintaining physiological functions by regulating gene expression at both the transcriptional and post-transcriptional levels. Nowadays, Salmena et al. put forward a competing endogenous RNA (ceRNA) hypothesis, in which lncRNAs, mRNAs, and other RNAs were able to compete with each other as ceRNAs to bind to miRNAs through sharing one or more miRNA response elements (MREs)^54^. LncRNAs can serve as endogenous molecular sponges for miRNAs to indirectly regulate the expression of mRNAs. For instance, Zhao et al. have indicated that lncRNA HOMEOBOX A11 antisense RNA (*HOXA11-AS*), acting as a ceRNA by sponging miR-454-3p, could regulate the expression of signal transducer and activator of transcription 3 (*STAT3*), and the HOXA11-AS/miR-454-3p/Stat3 axis was associated with the cisplatin resistance of human LUAD cells^55^. Uncovering the potential regulatory roles of lncRNAs, including its regulation on immune system and other pathological and physiological processes, may provide new insights into the pathological mechanisms in LUAD.

To evaluate the efficacy and accuracy of this prediction model, we performed 1-, 3-, and 5-year ROC curve analysis and the results showed that this model was efficient in predicting the prognosis of LUAD patients since AUC values were all over 0.70. And based on the optimal cut-off risk score identified by Youden Index, LUAD patients were divided into high-risk and low-risk subgroups, and K-M analysis revealed that this risk model exhibited great power in distinguishing good or poor survival of LUAD patients. In addition, the risk score of the model was found to be an independent prognostic factor for LUAD patients. Moreover, in order to estimate the clinical significance of the model, we performed chi-square test and Wilcoxon rank-sum test to explore the correlation between risk model and clinicopathological characteristics of LUAD patients, which indicated that the risk model was significantly associated with the clinical stage of patients including T stage, N stage, and M stage. All these results implied that this risk model performed well in predicting the prognosis of LUAD patients and these survival-related irlncRNAs included in the model might be used as novel therapeutic targets for LUAD treatment in the future.

In recent years, the treatment of lung cancer has entered the era of immunotherapy. However, not all patients with lung cancer can benefit from the treatment of immunotherapeutic agents, and the response rate of LUAD patients to immunotherapy remains unsatisfactory^5^. Tumor infiltrating immune cells in TME participate in various biological processes of malignant tumors, and the interaction between infiltrating immune cells and cancer cells can influence the malignant phenotypes of cancers^56,57^. In this study, we comprehensively estimated the tumor-infiltrating immune cells of LUAD samples by using seven acceptable methods including xCell, TIMER, quanTIseq, MCP-counter, EPIC, CIBERSORT-ABS, and CIBERSORT^25–27^, and then analyzed the relationship between tumor-infiltrating immune cells and the risk model. The correlation analysis indicated that the high-risk subgroup was more negatively related to tumor-infiltrating immune cells, such as CD8 + T cells and monocytes. CD8 + T cells are key effectors in anti-tumor immunity, and the frequency of CD8 + T cells is positively associated with the survival of patients with lung cancer, melanoma, and breast cancer^58,59^. In addition, the infiltration of CD8 + T cells in TME is related to improved responses of cancer patients treated with ICIs. For instance, Wong et al. demonstrated that melanoma patients with high CD8 + T cell count experienced prolonged survival when treated with anti-PD-1 therapy^60^. In addition, monocytes are a subtype of innate immune cells, which also play significant roles in anti-tumor immunity by various mechanisms, such as phagocytosis, apoptosis, and cell contact-mediated antibody-dependent cellular cytotoxicity (ADCC)^61,62^. The role of CD8 + T cells and monocytes in anti-tumor immunity was consistent with our results of correlation analysis, which indicated that LUAD patients in low-risk subgroup have more CD8 + T cells and monocytes infiltration. Besides, we also found that the risk scores of LUAD patients were significantly related to the expression levels of *CTLA4* gene and *HAVCR2* gene, which have been proved to be potential biomarkers associated with the treatment of ICIs^63,64^.

To further evaluate the clinical application value of the risk model in the treatment of LUAD, we calculated the IC50 of several common anti-tumor drugs and compared the differences of drug sensitivity between patients with high-risk and low-risk subgroups. We found that high-risk LUAD patients have higher sensitivity (lower IC50) of anti-tumor drugs including paclitaxel, docetaxel, gemcitabine, vinorelbine, etoposide, and erlotinib. This relationship between risk model and drug sensitivity might be used to guide the selection and administration of anti-tumor drugs in clinical practice, which needs to be further investigated in the future.

However, there are some shortcomings in this study. First, the RNA-seq data of LUAD cohort was only downloaded from TCGA database. Although we performed various methods to validate the accuracy and efficiency of our prognostic prediction model, additional external cohorts are needed to confirm it in the future. In addition, the expression of these irlncRNAs in lung tissues of LUAD patients need to be further investigated, such as RNA fluorescence in situ hybridization (FISH) and histological staining. And the potential regulatory mechanisms of lncRNAs and the relationship between lncRNAs and immune system need to be further studied. Our team will perform biological experiments and clinical studies to explore the downstream signaling pathways of these lncRNAs in the future. Second, the tumor-infiltrating immune cells of LUAD samples were estimated by different quantification methods based on the RNA-seq data, which need to be experimentally validated. Finally, the clinical application value of our risk model, such as its relationship with anti-tumor drug sensitivity, has not been clinically verified. Further studies with larger sample sizes of LUAD patients are required to confirm our results in the future.

In conclusion, we innovatively constructed a novel signature of DEirlncRNA pairs in LUAD, which did not depend on specific expression levels of lncRNAs. This signature performed well in predicting the prognosis of LUAD patients and might be used to guide the administration of patients with LUAD in clinical practice. Future studies, preferably with a large sample size, are needed to verify our findings.

## Supplementary Information

Supplementary Information 1.

Supplementary Information 2.
