# Supplementary figures and images for: A novel prognostic signature of immune-related lncRNA pairs in lung adenocarcinoma
Source: Sci Rep. 2021 Aug 18;11:16794. doi: 10.1038/s41598-021-96236-4 (PMC8373953; doi:10.1038/s41598-021-96236-4)

Supplementary Figure S1. DElncRNAs between LUSC samples and normal controls.

A

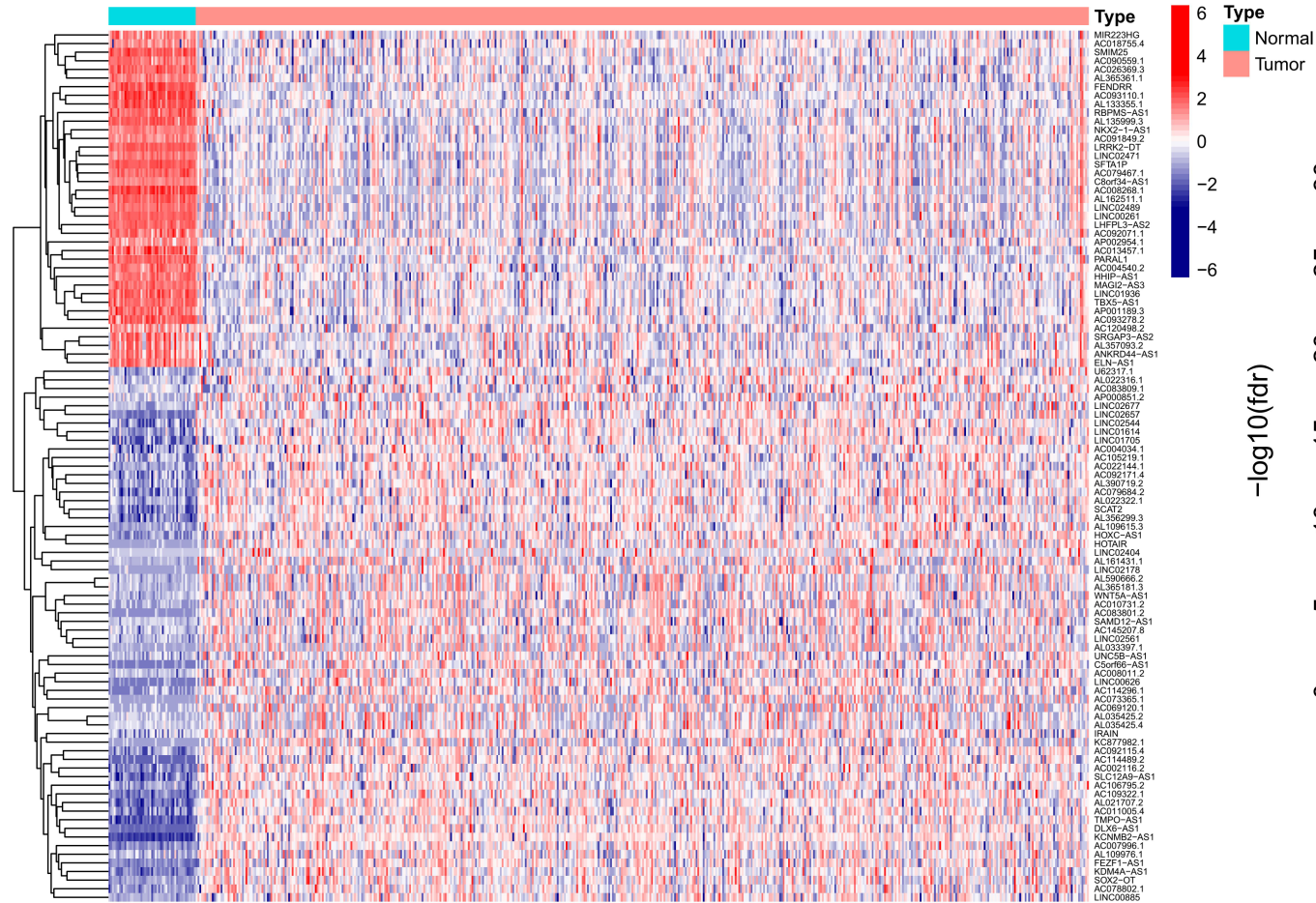

B

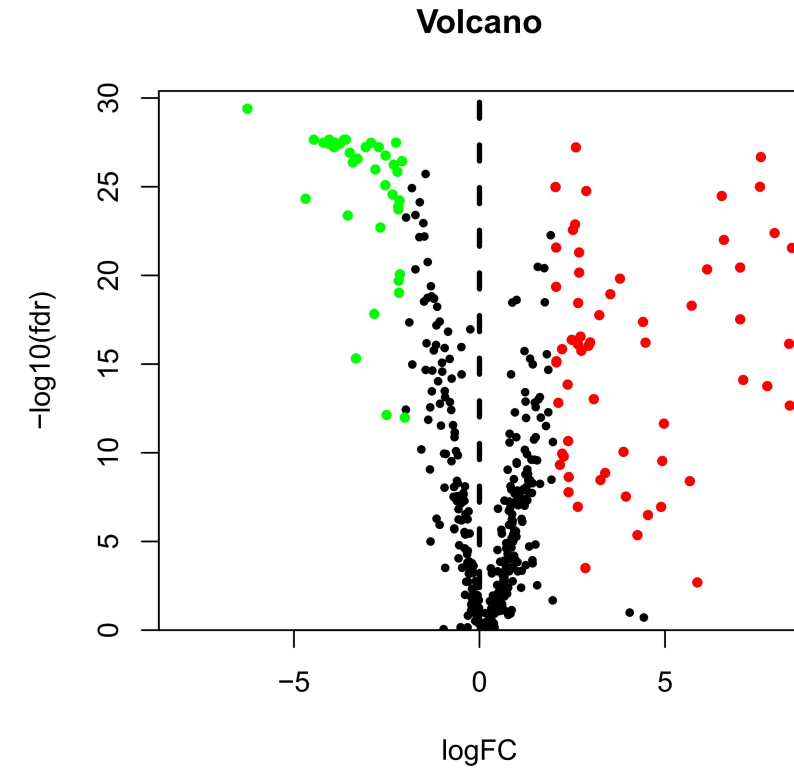

Supplement: Supplementary file 1 — Supplementary Information 1. [file 41598_2021_96236_MOESM1_ESM.pdf]
