# Supplementary material for: A novel prognostic signature of immune-related lncRNA pairs in lung adenocarcinoma
Source: Sci Rep. 2021 Aug 18;11:16794. doi: 10.1038/s41598-021-96236-4 (PMC8373953; doi:10.1038/s41598-021-96236-4)
Supplement: Supplementary file 2 — Supplementary Information 2. [file 41598_2021_96236_MOESM2_ESM.pdf]

A Novel Prognostic Signature of Immune-related lncRNA Pairs in Lung Adenocarcinoma

Yang Liu[1,2], Qiuhong Wu[3], Xuejiao Fan[4], Wen Li[1], Xiaogang Li[1,2], Hui Zhu[2], Qinghua Zhou[1]*, Jinming Yu[2]*

[1]Lung Cancer Center, West China Hospital, Sichuan University, Chengdu 610041, Sichuan, China

[2]Department of Radiation Oncology, Shandong Cancer Hospital and Institute, Shandong First Medical University and Shandong Academy of Medical Sciences, Jinan 250117, Shandong, China

[3]Department of Rheumatology and Immunology, West China Hospital, Sichuan University, Chengdu 610041, Sichuan, China

[4]Clinical Research Management Department, West China Hospital, Sichuan University, Chengdu 610041, Sichuan, China

Corresponding authors: Qinghua Zhou and Jinming Yu

Supplementary Table S1. Coexpression analysis between lncRNAs and immune-related genes in LUAD.

immuneGene	lncRNA	cor	pvalue	Regulation
NFAT5	AL512413.1	0.525338207	2.75E-39	postive
GNRH1	AL512413.1	0.504076089	8.02E-36	postive
CREB1	FLNB-AS1	0.685072081	2.34E-75	postive
UBR1	FLNB-AS1	0.609004974	1.30E-55	postive
ZC3HAV1	FLNB-AS1	0.502540057	1.40E-35	postive
CYLD	FLNB-AS1	0.529416258	5.58E-40	postive
MAPK8	FLNB-AS1	0.536121809	3.86E-41	postive
SP1	FLNB-AS1	0.521145138	1.39E-38	postive
IREB2	FLNB-AS1	0.558707602	3.03E-45	postive
DDX17	FLNB-AS1	0.656114091	3.65E-67	postive
JAK2	FLNB-AS1	0.525946136	2.17E-39	postive
TXK	FLNB-AS1	0.585383783	1.61E-50	postive
NFAT5	FLNB-AS1	0.722933247	1.24E-87	postive
PIK3R1	FLNB-AS1	0.503127062	1.13E-35	postive
PIK3CA	FLNB-AS1	0.573185508	4.78E-48	postive
ROBO2	FLNB-AS1	0.583190193	4.57E-50	postive
GNRH1	FLNB-AS1	0.800874658	8.67E-121	postive
RABEP1	FLNB-AS1	0.515547504	1.16E-37	postive
ACVR2A	FLNB-AS1	0.61124914	4.05E-56	postive
ANGPTL1	FLNB-AS1	0.610766439	5.20E-56	postive
BMPR1A	FLNB-AS1	0.52868625	7.43E-40	postive
BMPR2	FLNB-AS1	0.662895122	5.32E-69	postive
CRLF3	FLNB-AS1	0.509960326	9.32E-37	postive
NR1D2	FLNB-AS1	0.510656069	7.21E-37	postive
NR2C1	FLNB-AS1	0.578366505	4.39E-49	postive
NR2C2	FLNB-AS1	0.735431949	3.80E-92	postive
RORA	FLNB-AS1	0.695878186	1.15E-78	postive
SOS1	FLNB-AS1	0.62178119	1.50E-58	postive
BRAF	FLNB-AS1	0.682934226	1.01E-74	postive
CBL	FLNB-AS1	0.538566768	1.43E-41	postive
CBLB	FLNB-AS1	0.617263738	1.70E-57	postive
TRAJ1	FLNB-AS1	0.561562193	8.69E-46	postive
TRAJ2	FLNB-AS1	0.542917529	2.42E-42	postive
TRAJ3	FLNB-AS1	0.589559004	2.18E-51	postive
TRAJ5	FLNB-AS1	0.573346284	4.44E-48	postive
TRAJ6	FLNB-AS1	0.592541746	5.11E-52	postive
TRAJ8	FLNB-AS1	0.554727889	1.69E-44	postive
TRAJ10	FLNB-AS1	0.561048694	1.09E-45	postive
TRAJ12	FLNB-AS1	0.52633084	1.87E-39	postive
TRAJ13	FLNB-AS1	0.575186391	1.91E-48	postive
TRAJ14	FLNB-AS1	0.529856394	4.69E-40	postive
TRAJ16	FLNB-AS1	0.579991456	2.06E-49	postive
TRAJ17	FLNB-AS1	0.53431042	7.98E-41	postive
TRAJ18	FLNB-AS1	0.508234576	1.76E-36	postive
TRAJ21	FLNB-AS1	0.577807412	5.69E-49	postive
TRAJ31	FLNB-AS1	0.613303685	1.38E-56	postive
TRAJ37	FLNB-AS1	0.584233243	2.79E-50	postive
TRAJ38	FLNB-AS1	0.641797579	1.94E-63	postive
TRAJ39	FLNB-AS1	0.581126839	1.21E-49	postive
ACKR4	AL139246.3	0.665188675	1.24E-69	postive
FAM3D	AL139246.3	0.721299238	4.62E-87	postive
MIA	AL139246.3	0.762167499	1.01E-102	postive
HNF4A	AL139246.3	0.61417841	8.71E-57	postive
IL1R2	AL139246.3	0.675348308	1.68E-72	postive
IL22RA1	AL139246.3	0.591600625	8.09E-52	postive
DDX17	AC109460.2	0.66576645	8.58E-70	postive
IRF9	AC109460.2	0.567405618	6.52E-47	postive
LTB4R2	AC109460.2	0.597318705	4.86E-53	postive
GNRH1	AC109460.2	0.650736197	9.67E-66	postive
NR2C1	AC109460.2	0.580350844	1.74E-49	postive
NR2C2	AC109460.2	0.661523803	1.26E-68	postive
LMBR1L	AL022328.2	0.539349974	1.04E-41	postive

IRF3	AL022328.2	0.510965196	6.43E-37	postive
TYK2	AL022328.2	0.60794804	2.24E-55	postive
IRF9	AL022328.2	0.636331062	4.55E-62	postive
PLXNB1	AL022328.2	0.532794541	1.46E-40	postive
LRSAM1	AL022328.2	0.513211023	2.79E-37	postive
EPOR	AL022328.2	0.537720846	2.02E-41	postive
GIPR	AL022328.2	0.565379684	1.61E-46	postive
IL11RA	AL022328.2	0.53460477	7.10E-41	postive
TNFRSF14	AL022328.2	0.557318059	5.53E-45	postive
TNFRSF25	AL022328.2	0.501449905	2.07E-35	postive
CREB1	AC068790.3	0.789055466	7.18E-115	postive
RFXAP	AC068790.3	0.551158351	7.73E-44	postive
UBR1	AC068790.3	0.658089162	1.08E-67	postive
ZC3HAV1	AC068790.3	0.558551434	3.24E-45	postive
ZC3HAV1L	AC068790.3	0.510977936	6.40E-37	postive
IL15	AC068790.3	0.569139504	2.99E-47	postive
CYLD	AC068790.3	0.617682205	1.36E-57	postive
EIF2AK2	AC068790.3	0.524021663	4.58E-39	postive
MAPK8	AC068790.3	0.570745194	1.45E-47	postive
LMBR1	AC068790.3	0.572626081	6.16E-48	postive
IREB2	AC068790.3	0.618426261	9.11E-58	postive
DDX17	AC068790.3	0.621470732	1.77E-58	postive
PIK3CG	AC068790.3	0.511091601	6.13E-37	postive
JAK2	AC068790.3	0.619004173	6.69E-58	postive
TXK	AC068790.3	0.548322009	2.56E-43	postive
PPP3CB	AC068790.3	0.505257368	5.22E-36	postive
NFAT5	AC068790.3	0.909239208	5.11E-205	postive
NFATC3	AC068790.3	0.559530157	2.11E-45	postive
MALT1	AC068790.3	0.531150933	2.81E-40	postive
PIK3R1	AC068790.3	0.614547956	7.16E-57	postive
PIK3CA	AC068790.3	0.65960278	4.20E-68	postive
AKT3	AC068790.3	0.572977157	5.25E-48	postive
RASGRP3	AC068790.3	0.508476548	1.61E-36	postive
IGHD4-4	AC068790.3	0.536207219	3.73E-41	postive
IGHD6-6	AC068790.3	0.520241668	1.96E-38	postive
GNRH1	AC068790.3	0.776452271	5.77E-109	postive
IL6ST	AC068790.3	0.612294895	2.34E-56	postive
RABEP1	AC068790.3	0.58865928	3.36E-51	postive
ACVR2A	AC068790.3	0.66423897	2.27E-69	postive
ANGPTL1	AC068790.3	0.733397993	2.14E-91	postive
BMPR1A	AC068790.3	0.572919573	5.39E-48	postive
BMPR2	AC068790.3	0.706993558	3.17E-82	postive
CRLF3	AC068790.3	0.673715931	4.94E-72	postive
NR1D2	AC068790.3	0.505442088	4.88E-36	postive
NR2C1	AC068790.3	0.503984028	8.29E-36	postive
NR2C2	AC068790.3	0.713940603	1.55E-84	postive
RORA	AC068790.3	0.831383572	4.15E-138	postive
SOS1	AC068790.3	0.695526244	1.48E-78	postive
SOS2	AC068790.3	0.597935668	3.58E-53	postive
BRAF	AC068790.3	0.815863332	6.79E-129	postive
CBL	AC068790.3	0.600797419	8.55E-54	postive
CBLB	AC068790.3	0.657964469	1.16E-67	postive
RASGRP1	AC068790.3	0.512766654	3.29E-37	postive
PDK1	AC068790.3	0.559895332	1.80E-45	postive
TRAJ1	AC068790.3	0.83583444	6.31E-141	postive
TRAJ2	AC068790.3	0.806314217	1.20E-123	postive
TRAJ3	AC068790.3	0.877851901	1.52E-172	postive
TRAJ5	AC068790.3	0.853512638	5.17E-153	postive
TRAJ6	AC068790.3	0.895228039	3.01E-189	postive
TRAJ8	AC068790.3	0.886765409	9.01E-181	postive
TRAJ10	AC068790.3	0.871687552	3.17E-167	postive
TRAJ12	AC068790.3	0.801538519	3.92E-121	postive
TRAJ13	AC068790.3	0.886942941	6.08E-181	postive
TRAJ14	AC068790.3	0.868412352	1.65E-164	postive
TRAJ16	AC068790.3	0.86951083	2.07E-165	postive

TRAJ17	AC068790.3	0.843048692	1.12E-145	postive
TRAJ18	AC068790.3	0.793528731	4.59E-117	postive
TRAJ21	AC068790.3	0.894469364	1.85E-188	postive
TRAJ31	AC068790.3	0.908285439	7.26E-204	postive
TRAJ37	AC068790.3	0.911734852	4.28E-208	postive
TRAJ38	AC068790.3	0.919577235	2.15E-218	postive
TRAJ39	AC068790.3	0.913866822	8.51E-211	postive
CREB1	AL359715.3	0.588176124	4.24E-51	postive
UBR1	AL359715.3	0.503491467	9.91E-36	postive
MAPK8	AL359715.3	0.513110381	2.90E-37	postive
IREB2	AL359715.3	0.52236996	8.67E-39	postive
NFAT5	AL359715.3	0.585524921	1.51E-50	postive
PIK3CA	AL359715.3	0.519974134	2.17E-38	postive
GNRH1	AL359715.3	0.530940183	3.05E-40	postive
ACVR2A	AL359715.3	0.534367181	7.80E-41	postive
ANGPTL1	AL359715.3	0.560653167	1.30E-45	postive
BMPR1A	AL359715.3	0.508123465	1.83E-36	postive
BMPR2	AL359715.3	0.550454147	1.04E-43	postive
CRLF3	AL359715.3	0.528068161	9.47E-40	postive
NR2C2	AL359715.3	0.542922219	2.41E-42	postive
RORA	AL359715.3	0.543434253	1.95E-42	postive
SOS1	AL359715.3	0.58975049	1.98E-51	postive
BRAF	AL359715.3	0.65306749	2.36E-66	postive
CBLB	AL359715.3	0.5102287	8.44E-37	postive
TRAJ2	AL359715.3	0.529215931	6.03E-40	postive
TRAJ3	AL359715.3	0.526306355	1.89E-39	postive
TRAJ5	AL359715.3	0.53275395	1.49E-40	postive
TRAJ6	AL359715.3	0.547955094	2.98E-43	postive
TRAJ8	AL359715.3	0.585939329	1.24E-50	postive
TRAJ13	AL359715.3	0.562590786	5.53E-46	postive
TRAJ14	AL359715.3	0.555148534	1.41E-44	postive
TRAJ16	AL359715.3	0.550953807	8.42E-44	postive
TRAJ21	AL359715.3	0.560882996	1.17E-45	postive
TRAJ31	AL359715.3	0.575243173	1.86E-48	postive
TRAJ37	AL359715.3	0.550560387	9.95E-44	postive
TRAJ38	AL359715.3	0.608454478	1.73E-55	postive
TRAJ39	AL359715.3	0.576009128	1.31E-48	postive
CREB1	AP001429.1	0.755194057	7.82E-100	postive
UBR1	AP001429.1	0.612787963	1.81E-56	postive
ZC3HAV1	AP001429.1	0.562723241	5.22E-46	postive
IL15	AP001429.1	0.538759979	1.33E-41	postive
CYLD	AP001429.1	0.565015592	1.89E-46	postive
EIF2AK2	AP001429.1	0.552903982	3.68E-44	postive
MAPK8	AP001429.1	0.565646145	1.43E-46	postive
LMBR1	AP001429.1	0.595405392	1.25E-52	postive
LIMS1	AP001429.1	0.509078134	1.29E-36	postive
IREB2	AP001429.1	0.597449276	4.56E-53	postive
DDX17	AP001429.1	0.564524675	2.35E-46	postive
PIK3CG	AP001429.1	0.52181454	1.07E-38	postive
JAK2	AP001429.1	0.604781679	1.14E-54	postive
TXK	AP001429.1	0.590200782	1.60E-51	postive
NFAT5	AP001429.1	0.793104484	7.45E-117	postive
MALT1	AP001429.1	0.525950521	2.17E-39	postive
PIK3R1	AP001429.1	0.645815191	1.83E-64	postive
PIK3CA	AP001429.1	0.669847287	6.20E-71	postive
PIK3CB	AP001429.1	0.510245522	8.39E-37	postive
AKT3	AP001429.1	0.594878376	1.63E-52	postive
RASGRP3	AP001429.1	0.500375054	3.04E-35	postive
GNRH1	AP001429.1	0.689251873	1.28E-76	postive
IL6ST	AP001429.1	0.645449626	2.27E-64	postive
RABEP1	AP001429.1	0.540775877	5.83E-42	postive
ACVR2A	AP001429.1	0.592801967	4.50E-52	postive
ANGPTL1	AP001429.1	0.802770617	8.94E-122	postive
BMPR1A	AP001429.1	0.515339235	1.26E-37	postive
BMPR2	AP001429.1	0.719332052	2.23E-86	postive

CRLF3	AP001429.1	0.661100642	1.65E-68	postive
LIFR	AP001429.1	0.512994509	3.02E-37	postive
NR2C2	AP001429.1	0.627550191	6.35E-60	postive
RORA	AP001429.1	0.82350482	2.56E-133	postive
SOS1	AP001429.1	0.67288437	8.53E-72	postive
SOS2	AP001429.1	0.558446789	3.39E-45	postive
BRAF	AP001429.1	0.834993126	2.18E-140	postive
ITK	AP001429.1	0.545216607	9.34E-43	postive
CBL	AP001429.1	0.61322125	1.44E-56	postive
CBLB	AP001429.1	0.741825446	1.48E-94	postive
PDK1	AP001429.1	0.582513653	6.29E-50	postive
TRAJ1	AP001429.1	0.684726119	2.97E-75	postive
TRAJ2	AP001429.1	0.713681681	1.89E-84	postive
TRAJ3	AP001429.1	0.738623029	2.43E-93	postive
TRAJ5	AP001429.1	0.802987727	6.88E-122	postive
TRAJ6	AP001429.1	0.745016273	8.72E-96	postive
TRAJ8	AP001429.1	0.765829823	2.79E-104	postive
TRAJ10	AP001429.1	0.703374499	4.77E-81	postive
TRAJ12	AP001429.1	0.646768274	1.04E-64	postive
TRAJ13	AP001429.1	0.810105079	1.07E-125	postive
TRAJ14	AP001429.1	0.764852731	7.31E-104	postive
TRAJ16	AP001429.1	0.73067344	2.13E-90	postive
TRAJ17	AP001429.1	0.702092902	1.23E-80	postive
TRAJ18	AP001429.1	0.653648124	1.65E-66	postive
TRAJ21	AP001429.1	0.812900924	3.10E-127	postive
TRAJ31	AP001429.1	0.815170023	1.67E-128	postive
TRAJ37	AP001429.1	0.782609105	8.43E-112	postive
TRAJ38	AP001429.1	0.829304152	8.05E-137	postive
TRAJ39	AP001429.1	0.816049427	5.33E-129	postive
CD4	AC090559.1	0.837567727	4.79E-142	postive
CD74	AC090559.1	0.574027706	3.25E-48	postive
FCER1G	AC090559.1	0.645053418	2.87E-64	postive
HLA-DMB	AC090559.1	0.578402551	4.31E-49	postive
HLA-DOA	AC090559.1	0.570711083	1.47E-47	postive
HLA-DPA1	AC090559.1	0.619232227	5.92E-58	postive
HLA-DPB1	AC090559.1	0.591780019	7.41E-52	postive
HLA-DQA1	AC090559.1	0.625035337	2.54E-59	postive
HLA-DRA	AC090559.1	0.580210196	1.86E-49	postive
HLA-DRB1	AC090559.1	0.524986315	3.15E-39	postive
CIITA	AC090559.1	0.564937134	1.96E-46	postive
TLR4	AC090559.1	0.560419289	1.43E-45	postive
NOD2	AC090559.1	0.524964122	3.18E-39	postive
CYBB	AC090559.1	0.699572124	7.87E-80	postive
TLR7	AC090559.1	0.675140288	1.93E-72	postive
TLR8	AC090559.1	0.663836011	2.93E-69	postive
MSR1	AC090559.1	0.591522021	8.40E-52	postive
MARCO	AC090559.1	0.547775425	3.21E-43	postive
CD14	AC090559.1	0.601191303	7.01E-54	postive
CCR5	AC090559.1	0.585551547	1.49E-50	postive
CCL23	AC090559.1	0.500773164	2.63E-35	postive
CCR1	AC090559.1	0.585727416	1.37E-50	postive
FGR	AC090559.1	0.693636597	5.74E-78	postive
CD86	AC090559.1	0.692062093	1.76E-77	postive
OLR1	AC090559.1	0.512681901	3.40E-37	postive
SYK	AC090559.1	0.501385806	2.11E-35	postive
BTK	AC090559.1	0.742566043	7.70E-95	postive
VAV1	AC090559.1	0.553459178	2.90E-44	postive
RAC2	AC090559.1	0.505800445	4.29E-36	postive
PIK3R5	AC090559.1	0.683065936	9.27E-75	postive
PIK3CD	AC090559.1	0.517627449	5.30E-38	postive
INPP5D	AC090559.1	0.521435017	1.24E-38	postive
PTPN6	AC090559.1	0.531034054	2.94E-40	postive
LILRB3	AC090559.1	0.671707009	1.85E-71	postive
C5AR1	AC090559.1	0.55652047	7.80E-45	postive
CMKLR1	AC090559.1	0.66796096	2.10E-70	postive

CX3CR1	AC090559.1	0.500896926	2.52E-35	postive
FPR1	AC090559.1	0.546702559	5.03E-43	postive
PTAFR	AC090559.1	0.598421076	2.81E-53	postive
ADA2	AC090559.1	0.554931007	1.55E-44	postive
IL16	AC090559.1	0.565761946	1.36E-46	postive
TNFSF12	AC090559.1	0.525278017	2.81E-39	postive
C3AR1	AC090559.1	0.746288341	2.79E-96	postive
CSF1R	AC090559.1	0.806695724	7.49E-124	postive
CSF2RA	AC090559.1	0.507770434	2.09E-36	postive
CSF2RB	AC090559.1	0.709549928	4.55E-83	postive
IL10RA	AC090559.1	0.677780895	3.32E-73	postive
IL12RB1	AC090559.1	0.544631189	1.19E-42	postive
IL17RA	AC090559.1	0.594838441	1.66E-52	postive
IL21R	AC090559.1	0.541792186	3.84E-42	postive
PTGER4	AC090559.1	0.532925026	1.39E-40	postive
TNFRSF1B	AC090559.1	0.502310878	1.52E-35	postive
ITGAL	AC090559.1	0.620469551	3.04E-58	postive
ITGB2	AC090559.1	0.717479731	9.66E-86	postive
TYROBP	AC090559.1	0.564373926	2.52E-46	postive
FCGR3A	AC090559.1	0.625312054	2.18E-59	postive
LCP2	AC090559.1	0.684804872	2.81E-75	postive
PTPRC	AC090559.1	0.54199508	3.54E-42	postive
CREB1	RAB30-DT	0.542403377	2.99E-42	postive
MAPK8	RAB30-DT	0.509668713	1.04E-36	postive
EED	RAB30-DT	0.620283889	3.36E-58	postive
NFAT5	RAB30-DT	0.642987539	9.68E-64	postive
GNRH1	RAB30-DT	0.583002308	5.00E-50	postive
ANGPTL1	RAB30-DT	0.518734251	3.48E-38	postive
CRLF3	RAB30-DT	0.518003065	4.60E-38	postive
NR2C2	RAB30-DT	0.533719894	1.01E-40	postive
RORA	RAB30-DT	0.613363513	1.34E-56	postive
SOS1	RAB30-DT	0.520784355	1.59E-38	postive
BRAF	RAB30-DT	0.637668192	2.12E-62	postive
CBLB	RAB30-DT	0.503078808	1.15E-35	postive
PDK1	RAB30-DT	0.55977679	1.90E-45	postive
TRAJ1	RAB30-DT	0.581619686	9.59E-50	postive
TRAJ2	RAB30-DT	0.588403208	3.80E-51	postive
TRAJ3	RAB30-DT	0.611826157	2.99E-56	postive
TRAJ5	RAB30-DT	0.597343562	4.80E-53	postive
TRAJ6	RAB30-DT	0.619973744	3.97E-58	postive
TRAJ8	RAB30-DT	0.60583167	6.65E-55	postive
TRAJ10	RAB30-DT	0.577079975	7.97E-49	postive
TRAJ12	RAB30-DT	0.565939593	1.25E-46	postive
TRAJ13	RAB30-DT	0.615829072	3.64E-57	postive
TRAJ14	RAB30-DT	0.61568524	3.93E-57	postive
TRAJ16	RAB30-DT	0.591448617	8.71E-52	postive
TRAJ17	RAB30-DT	0.557000097	6.34E-45	postive
TRAJ18	RAB30-DT	0.544548329	1.23E-42	postive
TRAJ21	RAB30-DT	0.627523679	6.45E-60	postive
TRAJ31	RAB30-DT	0.615023282	5.57E-57	postive
TRAJ37	RAB30-DT	0.628599291	3.55E-60	postive
TRAJ38	RAB30-DT	0.657031375	2.07E-67	postive
TRAJ39	RAB30-DT	0.636064931	5.30E-62	postive
CREB1	AC067817.2	0.629991404	1.64E-60	postive
UBR1	AC067817.2	0.519134818	2.99E-38	postive
CYLD	AC067817.2	0.510582921	7.41E-37	postive
DDX17	AC067817.2	0.578518552	4.09E-49	postive
TXK	AC067817.2	0.58661692	8.96E-51	postive
NFAT5	AC067817.2	0.638499724	1.31E-62	postive
MALT1	AC067817.2	0.512816179	3.23E-37	postive
PIK3R1	AC067817.2	0.653077462	2.34E-66	postive
PIK3CA	AC067817.2	0.564421992	2.46E-46	postive
GNRH1	AC067817.2	0.619516834	5.08E-58	postive
IL6ST	AC067817.2	0.648019447	4.94E-65	postive
ACVR2A	AC067817.2	0.560002564	1.72E-45	postive

ANGPTL1	AC067817.2	0.712330339	5.39E-84	postive
BMPR2	AC067817.2	0.691960121	1.89E-77	postive
NR2C2	AC067817.2	0.575539253	1.62E-48	postive
RORA	AC067817.2	0.798003061	2.58E-119	postive
SOS1	AC067817.2	0.566338478	1.05E-46	postive
BRAF	AC067817.2	0.705652912	8.69E-82	postive
ITK	AC067817.2	0.586819682	8.13E-51	postive
CBLB	AC067817.2	0.652234032	3.91E-66	postive
TRAJ2	AC067817.2	0.563709246	3.38E-46	postive
TRAJ3	AC067817.2	0.560873779	1.18E-45	postive
TRAJ5	AC067817.2	0.589814358	1.92E-51	postive
TRAJ6	AC067817.2	0.507354305	2.43E-36	postive
TRAJ8	AC067817.2	0.517546037	5.46E-38	postive
TRAJ10	AC067817.2	0.503034843	1.17E-35	postive
TRAJ13	AC067817.2	0.55045818	1.04E-43	postive
TRAJ14	AC067817.2	0.517978561	4.64E-38	postive
TRAJ16	AC067817.2	0.523549879	5.50E-39	postive
TRAJ17	AC067817.2	0.514271226	1.88E-37	postive
TRAJ18	AC067817.2	0.514148723	1.96E-37	postive
TRAJ21	AC067817.2	0.583372667	4.19E-50	postive
TRAJ31	AC067817.2	0.622031457	1.31E-58	postive
TRAJ37	AC067817.2	0.588488229	3.65E-51	postive
TRAJ38	AC067817.2	0.635685557	6.58E-62	postive
TRAJ39	AC067817.2	0.595483694	1.21E-52	postive
DDX17	AL159169.2	0.517780928	5.00E-38	postive
LTB4R2	AL159169.2	0.510089791	8.89E-37	postive
GNRH1	AL159169.2	0.609388618	1.06E-55	postive
ANGPTL1	AL159169.2	0.507400069	2.39E-36	postive
NR2C2	AL159169.2	0.645290703	2.50E-64	postive
SOS1	AL159169.2	0.504766472	6.24E-36	postive
BRAF	AL159169.2	0.66227374	7.87E-69	postive
TRAJ1	AL159169.2	0.569098126	3.05E-47	postive
TRAJ3	AL159169.2	0.579797098	2.25E-49	postive
TRAJ5	AL159169.2	0.62897182	2.89E-60	postive
TRAJ6	AL159169.2	0.542549136	2.82E-42	postive
TRAJ8	AL159169.2	0.626147524	1.38E-59	postive
TRAJ10	AL159169.2	0.524745165	3.46E-39	postive
TRAJ12	AL159169.2	0.56120052	1.02E-45	postive
TRAJ13	AL159169.2	0.590191996	1.60E-51	postive
TRAJ14	AL159169.2	0.603809037	1.87E-54	postive
TRAJ16	AL159169.2	0.568626692	3.77E-47	postive
TRAJ17	AL159169.2	0.607946767	2.24E-55	postive
TRAJ21	AL159169.2	0.595376388	1.27E-52	postive
TRAJ31	AL159169.2	0.613649662	1.15E-56	postive
TRAJ37	AL159169.2	0.549769919	1.39E-43	postive
TRAJ38	AL159169.2	0.597932438	3.58E-53	postive
TRAJ39	AL159169.2	0.607217471	3.27E-55	postive
NFATC3	MID1IP1-AS1	0.583100861	4.77E-50	postive
CREB1	AC141002.1	0.636617241	3.87E-62	postive
UBR1	AC141002.1	0.563281058	4.08E-46	postive
ZC3HAV1	AC141002.1	0.519399366	2.70E-38	postive
ZC3HAV1L	AC141002.1	0.51908026	3.05E-38	postive
MAPK8	AC141002.1	0.53051796	3.61E-40	postive
IREB2	AC141002.1	0.532153845	1.89E-40	postive
DDX17	AC141002.1	0.6226271	9.45E-59	postive
TXK	AC141002.1	0.514429277	1.77E-37	postive
NFAT5	AC141002.1	0.672747972	9.33E-72	postive
PIK3R1	AC141002.1	0.51841168	3.94E-38	postive
PIK3CA	AC141002.1	0.51495798	1.45E-37	postive
LTB4R2	AC141002.1	0.500832403	2.58E-35	postive
GNRH1	AC141002.1	0.7718403	6.70E-107	postive
IL6ST	AC141002.1	0.534355106	7.84E-41	postive
ACVR2A	AC141002.1	0.555637007	1.14E-44	postive
ANGPTL1	AC141002.1	0.618147363	1.06E-57	postive
BMPR2	AC141002.1	0.594811914	1.68E-52	postive

CRLF3	AC141002.1	0.540863167	5.62E-42	postive
NR2C1	AC141002.1	0.532289727	1.79E-40	postive
NR2C2	AC141002.1	0.738394412	2.96E-93	postive
RORA	AC141002.1	0.699105867	1.11E-79	postive
SOS1	AC141002.1	0.600369565	1.06E-53	postive
BRAF	AC141002.1	0.757482739	9.03E-101	postive
CBL	AC141002.1	0.516566513	7.91E-38	postive
CBLB	AC141002.1	0.642166279	1.56E-63	postive
PDK1	AC141002.1	0.502312081	1.52E-35	postive
TRAJ1	AC141002.1	0.542700194	2.65E-42	postive
TRAJ2	AC141002.1	0.56245041	5.88E-46	postive
TRAJ3	AC141002.1	0.607345391	3.06E-55	postive
TRAJ5	AC141002.1	0.626487303	1.14E-59	postive
TRAJ6	AC141002.1	0.577730798	5.89E-49	postive
TRAJ8	AC141002.1	0.594321147	2.14E-52	postive
TRAJ10	AC141002.1	0.54170771	3.98E-42	postive
TRAJ13	AC141002.1	0.603887123	1.79E-54	postive
TRAJ14	AC141002.1	0.568627944	3.77E-47	postive
TRAJ16	AC141002.1	0.572249725	7.32E-48	postive
TRAJ17	AC141002.1	0.540011982	7.96E-42	postive
TRAJ21	AC141002.1	0.610836278	5.02E-56	postive
TRAJ31	AC141002.1	0.627801719	5.53E-60	postive
TRAJ37	AC141002.1	0.612041119	2.68E-56	postive
TRAJ38	AC141002.1	0.643863243	5.79E-64	postive
TRAJ39	AC141002.1	0.644084596	5.08E-64	postive
CREB1	NUTM2B-AS1	0.767191766	7.23E-105	postive
RFXAP	NUTM2B-AS1	0.552941196	3.62E-44	postive
UBR1	NUTM2B-AS1	0.654875457	7.81E-67	postive
ZC3HAV1	NUTM2B-AS1	0.532256902	1.81E-40	postive
IL15	NUTM2B-AS1	0.568012295	4.97E-47	postive
CYLD	NUTM2B-AS1	0.595271542	1.34E-52	postive
EIF2AK2	NUTM2B-AS1	0.504134682	7.85E-36	postive
MAPK8	NUTM2B-AS1	0.625721168	1.74E-59	postive
LMBR1	NUTM2B-AS1	0.548985006	1.93E-43	postive
IREB2	NUTM2B-AS1	0.620686839	2.70E-58	postive
DDX17	NUTM2B-AS1	0.635298271	8.21E-62	postive
JAK2	NUTM2B-AS1	0.57873673	3.69E-49	postive
TXK	NUTM2B-AS1	0.591229725	9.69E-52	postive
PPP3CB	NUTM2B-AS1	0.536539638	3.26E-41	postive
NFAT5	NUTM2B-AS1	0.874003348	3.43E-169	postive
NFATC3	NUTM2B-AS1	0.529746688	4.90E-40	postive
MALT1	NUTM2B-AS1	0.538023679	1.79E-41	postive
PIK3R1	NUTM2B-AS1	0.651788301	5.12E-66	postive
PIK3CA	NUTM2B-AS1	0.632079875	5.07E-61	postive
AKT3	NUTM2B-AS1	0.524821999	3.36E-39	postive
IGHD4-4	NUTM2B-AS1	0.541928993	3.63E-42	postive
IGHD6-6	NUTM2B-AS1	0.537169776	2.53E-41	postive
GNRH1	NUTM2B-AS1	0.741595982	1.81E-94	postive
IL6ST	NUTM2B-AS1	0.663885476	2.84E-69	postive
RABEP1	NUTM2B-AS1	0.564125348	2.81E-46	postive
ACVR2A	NUTM2B-AS1	0.640034626	5.41E-63	postive
ANGPTL1	NUTM2B-AS1	0.739359497	1.28E-93	postive
BMPR1A	NUTM2B-AS1	0.60140941	6.28E-54	postive
BMPR2	NUTM2B-AS1	0.720835221	6.70E-87	postive
CRLF3	NUTM2B-AS1	0.603679531	1.99E-54	postive
NR1D2	NUTM2B-AS1	0.510123835	8.78E-37	postive
NR2C2	NUTM2B-AS1	0.68773898	3.68E-76	postive
RORA	NUTM2B-AS1	0.879956798	1.98E-174	postive
SOS1	NUTM2B-AS1	0.666935784	4.06E-70	postive
SOS2	NUTM2B-AS1	0.554331036	2.00E-44	postive
BRAF	NUTM2B-AS1	0.801325004	5.06E-121	postive
ITK	NUTM2B-AS1	0.520012787	2.14E-38	postive
CBL	NUTM2B-AS1	0.574980731	2.10E-48	postive
CBLB	NUTM2B-AS1	0.688983668	1.54E-76	postive
RASGRP1	NUTM2B-AS1	0.508585778	1.55E-36	postive

PDK1	NUTM2B-AS1	0.570762	1.44E-47	postive
TRAJ1	NUTM2B-AS1	0.697188517	4.46E-79	postive
TRAJ2	NUTM2B-AS1	0.753666799	3.26E-99	postive
TRAJ3	NUTM2B-AS1	0.796667913	1.23E-118	postive
TRAJ5	NUTM2B-AS1	0.763746017	2.17E-103	postive
TRAJ6	NUTM2B-AS1	0.781024971	4.61E-111	postive
TRAJ8	NUTM2B-AS1	0.73612279	2.10E-92	postive
TRAJ10	NUTM2B-AS1	0.773113016	1.82E-107	postive
TRAJ12	NUTM2B-AS1	0.705005436	1.41E-81	postive
TRAJ13	NUTM2B-AS1	0.767113607	7.81E-105	postive
TRAJ14	NUTM2B-AS1	0.746853764	1.68E-96	postive
TRAJ16	NUTM2B-AS1	0.743454133	3.51E-95	postive
TRAJ17	NUTM2B-AS1	0.716954265	1.46E-85	postive
TRAJ18	NUTM2B-AS1	0.745376693	6.32E-96	postive
TRAJ21	NUTM2B-AS1	0.802209811	1.76E-121	postive
TRAJ31	NUTM2B-AS1	0.822417204	1.12E-132	postive
TRAJ37	NUTM2B-AS1	0.844709728	8.34E-147	postive
TRAJ38	NUTM2B-AS1	0.842635498	2.13E-145	postive
TRAJ39	NUTM2B-AS1	0.821447985	4.17E-132	postive
SCTR	BCRP3	0.501423644	2.09E-35	postive
CREB1	EBLN3P	0.58470264	2.23E-50	postive
MAPK8	EBLN3P	0.53586274	4.28E-41	postive
PIK3CA	EBLN3P	0.540203804	7.36E-42	postive
CRLF3	EBLN3P	0.502416713	1.46E-35	postive
NR2C2	EBLN3P	0.55122553	7.51E-44	postive
SOS1	EBLN3P	0.557532105	5.04E-45	postive
SOS2	EBLN3P	0.515686515	1.10E-37	postive
BRAF	EBLN3P	0.580522391	1.60E-49	postive
PDK1	EBLN3P	0.502645657	1.34E-35	postive
TRAJ5	EBLN3P	0.51025128	8.37E-37	postive
TRAJ38	EBLN3P	0.501935634	1.74E-35	postive
GNRH2	AC036214.2	0.677424139	4.21E-73	postive
CREB1	AC026124.2	0.780163049	1.16E-110	postive
UBR1	AC026124.2	0.622169111	1.21E-58	postive
ZC3HAV1	AC026124.2	0.560197473	1.58E-45	postive
IL15	AC026124.2	0.567524845	6.18E-47	postive
CYLD	AC026124.2	0.578363524	4.39E-49	postive
EIF2AK2	AC026124.2	0.559603237	2.05E-45	postive
MAPK8	AC026124.2	0.577742862	5.86E-49	postive
TLR1	AC026124.2	0.509169012	1.25E-36	postive
LMBR1	AC026124.2	0.571825453	8.87E-48	postive
LIMS1	AC026124.2	0.509525932	1.09E-36	postive
IREB2	AC026124.2	0.612794405	1.80E-56	postive
DDX17	AC026124.2	0.558743976	2.98E-45	postive
PIK3CG	AC026124.2	0.513316677	2.68E-37	postive
JAK2	AC026124.2	0.638430211	1.37E-62	postive
TXK	AC026124.2	0.553684289	2.64E-44	postive
NFAT5	AC026124.2	0.810293802	8.48E-126	postive
MALT1	AC026124.2	0.537255388	2.44E-41	postive
PIK3R1	AC026124.2	0.582509964	6.30E-50	postive
PIK3CA	AC026124.2	0.698969052	1.22E-79	postive
PIK3CB	AC026124.2	0.542263622	3.17E-42	postive
AKT3	AC026124.2	0.537602935	2.12E-41	postive
RASGRP3	AC026124.2	0.514971826	1.44E-37	postive
GNRH1	AC026124.2	0.696137546	9.54E-79	postive
IL6ST	AC026124.2	0.59834807	2.91E-53	postive
RABEP1	AC026124.2	0.555785494	1.07E-44	postive
ACVR2A	AC026124.2	0.639376285	7.91E-63	postive
ANGPTL1	AC026124.2	0.786025911	2.05E-113	postive
BMPR1A	AC026124.2	0.543773049	1.70E-42	postive
BMPR2	AC026124.2	0.700400797	4.29E-80	postive
CRLF3	AC026124.2	0.683442088	7.17E-75	postive
NR2C2	AC026124.2	0.640852695	3.36E-63	postive
RORA	AC026124.2	0.798001706	2.59E-119	postive
SOS1	AC026124.2	0.680698922	4.64E-74	postive

SOS2	AC026124.2	0.571626271	9.72E-48	postive
BRAF	AC026124.2	0.782063556	1.52E-111	postive
CBL	AC026124.2	0.583483414	3.98E-50	postive
CBLB	AC026124.2	0.719681589	1.68E-86	postive
PDK1	AC026124.2	0.590717879	1.24E-51	postive
TRAJ1	AC026124.2	0.746270152	2.83E-96	postive
TRAJ2	AC026124.2	0.710333578	2.50E-83	postive
TRAJ3	AC026124.2	0.758373402	3.87E-101	postive
TRAJ5	AC026124.2	0.822980808	5.23E-133	postive
TRAJ6	AC026124.2	0.801722668	3.15E-121	postive
TRAJ8	AC026124.2	0.789461631	4.56E-115	postive
TRAJ10	AC026124.2	0.782201472	1.31E-111	postive
TRAJ12	AC026124.2	0.712558124	4.52E-84	postive
TRAJ13	AC026124.2	0.825275983	2.25E-134	postive
TRAJ14	AC026124.2	0.797145144	7.04E-119	postive
TRAJ16	AC026124.2	0.805449308	3.46E-123	postive
TRAJ17	AC026124.2	0.770246117	3.38E-106	postive
TRAJ18	AC026124.2	0.713059291	3.06E-84	postive
TRAJ21	AC026124.2	0.83281132	5.28E-139	postive
TRAJ31	AC026124.2	0.853844876	2.96E-153	postive
TRAJ37	AC026124.2	0.799610205	3.89E-120	postive
TRAJ38	AC026124.2	0.86928368	3.18E-165	postive
TRAJ39	AC026124.2	0.83265425	6.63E-139	postive
CREB1	AL139407.1	0.796677247	1.21E-118	postive
RFXAP	AL139407.1	0.529853502	4.69E-40	postive
UBR1	AL139407.1	0.649238095	2.38E-65	postive
ZC3HAV1	AL139407.1	0.572782347	5.74E-48	postive
ZC3HAV1L	AL139407.1	0.518159084	4.33E-38	postive
IL15	AL139407.1	0.644120209	4.98E-64	postive
CYLD	AL139407.1	0.62449502	3.41E-59	postive
EIF2AK2	AL139407.1	0.563458485	3.77E-46	postive
MAPK8	AL139407.1	0.579889631	2.16E-49	postive
TLR1	AL139407.1	0.510539769	7.52E-37	postive
NFKBIZ	AL139407.1	0.509129612	1.27E-36	postive
LMBR1	AL139407.1	0.588884335	3.02E-51	postive
LIMS1	AL139407.1	0.530364569	3.84E-40	postive
IREB2	AL139407.1	0.61437136	7.86E-57	postive
DDX17	AL139407.1	0.627870334	5.32E-60	postive
PIK3CG	AL139407.1	0.561058299	1.08E-45	postive
JAK2	AL139407.1	0.683573168	6.55E-75	postive
TXK	AL139407.1	0.604373229	1.40E-54	postive
PPP3CB	AL139407.1	0.502007841	1.69E-35	postive
NFAT5	AL139407.1	0.836727984	1.68E-141	postive
MALT1	AL139407.1	0.553294587	3.11E-44	postive
PIK3R1	AL139407.1	0.649609165	1.91E-65	postive
PIK3CA	AL139407.1	0.700554302	3.83E-80	postive
PIK3CB	AL139407.1	0.542586276	2.77E-42	postive
AKT3	AL139407.1	0.543535865	1.87E-42	postive
RASGRP3	AL139407.1	0.556098239	9.36E-45	postive
PLXNC1	AL139407.1	0.52670827	1.61E-39	postive
GNRH1	AL139407.1	0.74003984	7.08E-94	postive
IL6ST	AL139407.1	0.609688152	9.12E-56	postive
RABEP1	AL139407.1	0.576416202	1.08E-48	postive
ACVR2A	AL139407.1	0.610521637	5.91E-56	postive
ANGPTL1	AL139407.1	0.813738202	1.06E-127	postive
BMPR1A	AL139407.1	0.545901716	7.03E-43	postive
BMPR2	AL139407.1	0.728834183	9.85E-90	postive
CRLF3	AL139407.1	0.661285497	1.47E-68	postive
LIFR	AL139407.1	0.537514865	2.20E-41	postive
NR1D2	AL139407.1	0.5259595	2.16E-39	postive
NR2C2	AL139407.1	0.712045866	6.71E-84	postive
RORA	AL139407.1	0.819694582	4.37E-131	postive
SOS1	AL139407.1	0.705209017	1.21E-81	postive
SOS2	AL139407.1	0.594330099	2.13E-52	postive
BRAF	AL139407.1	0.822303904	1.31E-132	postive

ITK	AL139407.1	0.543425869	1.96E-42	postive
CBL	AL139407.1	0.648100091	4.70E-65	postive
CBLB	AL139407.1	0.800845976	8.97E-121	postive
RASGRP1	AL139407.1	0.519943389	2.20E-38	postive
PDK1	AL139407.1	0.608922753	1.36E-55	postive
TRAJ1	AL139407.1	0.72519002	1.98E-88	postive
TRAJ2	AL139407.1	0.767807301	3.91E-105	postive
TRAJ3	AL139407.1	0.803871094	2.37E-122	postive
TRAJ5	AL139407.1	0.765434237	4.12E-104	postive
TRAJ6	AL139407.1	0.799076362	7.31E-120	postive
TRAJ8	AL139407.1	0.734452102	8.76E-92	postive
TRAJ10	AL139407.1	0.774285572	5.46E-108	postive
TRAJ12	AL139407.1	0.734650767	7.40E-92	postive
TRAJ13	AL139407.1	0.76510456	5.71E-104	postive
TRAJ14	AL139407.1	0.780687197	6.62E-111	postive
TRAJ16	AL139407.1	0.769260736	9.12E-106	postive
TRAJ17	AL139407.1	0.720386633	9.60E-87	postive
TRAJ18	AL139407.1	0.755518566	5.77E-100	postive
TRAJ21	AL139407.1	0.804996934	6.01E-123	postive
TRAJ31	AL139407.1	0.809301361	2.95E-125	postive
TRAJ37	AL139407.1	0.787598449	3.62E-114	postive
TRAJ38	AL139407.1	0.87380552	5.07E-169	postive
TRAJ39	AL139407.1	0.798549213	1.36E-119	postive
CREB1	AC092801.1	0.799755533	3.28E-120	postive
RFXAP	AC092801.1	0.525776853	2.32E-39	postive
UBR1	AC092801.1	0.687884544	3.33E-76	postive
ZC3HAV1	AC092801.1	0.578698407	3.76E-49	postive
ZC3HAV1L	AC092801.1	0.530950382	3.04E-40	postive
IL15	AC092801.1	0.583187188	4.58E-50	postive
CYLD	AC092801.1	0.626045083	1.46E-59	postive
EIF2AK2	AC092801.1	0.554681105	1.72E-44	postive
MAPK8	AC092801.1	0.591323059	9.26E-52	postive
LMBR1	AC092801.1	0.605449438	8.09E-55	postive
LIMS1	AC092801.1	0.514822477	1.53E-37	postive
IREB2	AC092801.1	0.640796727	3.47E-63	postive
DDX17	AC092801.1	0.608724333	1.50E-55	postive
PIK3CG	AC092801.1	0.563323539	4.00E-46	postive
JAK2	AC092801.1	0.650340818	1.23E-65	postive
TXK	AC092801.1	0.608154776	2.02E-55	postive
PPP3CB	AC092801.1	0.509418732	1.14E-36	postive
NFAT5	AC092801.1	0.883914698	4.56E-178	postive
NFATC3	AC092801.1	0.519764575	2.35E-38	postive
MALT1	AC092801.1	0.551868645	5.72E-44	postive
PIK3R1	AC092801.1	0.67454207	2.86E-72	postive
PIK3CA	AC092801.1	0.680933638	3.96E-74	postive
PIK3CB	AC092801.1	0.513876841	2.17E-37	postive
AKT3	AC092801.1	0.576587288	1.00E-48	postive
RASGRP3	AC092801.1	0.532576091	1.60E-40	postive
IGHD4-4	AC092801.1	0.55576099	1.08E-44	postive
IGHD6-6	AC092801.1	0.540640853	6.16E-42	postive
PLXNC1	AC092801.1	0.506078403	3.87E-36	postive
GNRH1	AC092801.1	0.752219483	1.25E-98	postive
IL6ST	AC092801.1	0.663199838	4.39E-69	postive
RABEP1	AC092801.1	0.589486085	2.26E-51	postive
ACVR2A	AC092801.1	0.653965405	1.36E-66	postive
ANGPTL1	AC092801.1	0.774647244	3.76E-108	postive
BMPR1A	AC092801.1	0.579478275	2.61E-49	postive
BMPR2	AC092801.1	0.740684491	4.03E-94	postive
CRLF3	AC092801.1	0.677611558	3.71E-73	postive
LIFR	AC092801.1	0.517439541	5.69E-38	postive
NR1D2	AC092801.1	0.534457929	7.53E-41	postive
NR2C2	AC092801.1	0.696792588	5.94E-79	postive
NR3C1	AC092801.1	0.509135308	1.26E-36	postive
RORA	AC092801.1	0.874459892	1.39E-169	postive
SOS1	AC092801.1	0.681859292	2.11E-74	postive

SOS2	AC092801.1	0.587502256	5.86E-51	postive
BRAF	AC092801.1	0.843286544	7.73E-146	postive
ITK	AC092801.1	0.514341849	1.83E-37	postive
TEC	AC092801.1	0.512380931	3.80E-37	postive
CD28	AC092801.1	0.501835941	1.80E-35	postive
CBL	AC092801.1	0.598946356	2.16E-53	postive
CBLB	AC092801.1	0.724531457	3.39E-88	postive
RASGRP1	AC092801.1	0.515896885	1.02E-37	postive
PDK1	AC092801.1	0.610925033	4.79E-56	postive
TRAJ1	AC092801.1	0.754092639	2.19E-99	postive
TRAJ2	AC092801.1	0.795884325	3.05E-118	postive
TRAJ3	AC092801.1	0.826020461	8.04E-135	postive
TRAJ5	AC092801.1	0.813563511	1.33E-127	postive
TRAJ6	AC092801.1	0.828927717	1.37E-136	postive
TRAJ8	AC092801.1	0.804428969	1.20E-122	postive
TRAJ10	AC092801.1	0.800471223	1.40E-120	postive
TRAJ12	AC092801.1	0.739453585	1.18E-93	postive
TRAJ13	AC092801.1	0.820940102	8.25E-132	postive
TRAJ14	AC092801.1	0.807894231	1.70E-124	postive
TRAJ16	AC092801.1	0.799066384	7.39E-120	postive
TRAJ17	AC092801.1	0.759109248	1.92E-101	postive
TRAJ18	AC092801.1	0.752108426	1.38E-98	postive
TRAJ21	AC092801.1	0.84484465	6.74E-147	postive
TRAJ31	AC092801.1	0.860374505	3.85E-158	postive
TRAJ37	AC092801.1	0.859223927	2.91E-157	postive
TRAJ38	AC092801.1	0.893189896	3.84E-187	postive
TRAJ39	AC092801.1	0.870909906	1.42E-166	postive
CIITA	MIR3142HG	0.519916744	2.22E-38	postive
CREB1	ANKRD44-IT1	0.776515975	5.40E-109	postive
RFXAP	ANKRD44-IT1	0.506913814	2.86E-36	postive
UBR1	ANKRD44-IT1	0.641516157	2.29E-63	postive
ZC3HAV1	ANKRD44-IT1	0.573807837	3.59E-48	postive
IL15	ANKRD44-IT1	0.580665221	1.50E-49	postive
CYLD	ANKRD44-IT1	0.658402086	8.87E-68	postive
EIF2AK2	ANKRD44-IT1	0.572764232	5.79E-48	postive
MAPK8	ANKRD44-IT1	0.556855936	6.75E-45	postive
LMBR1	ANKRD44-IT1	0.574710767	2.38E-48	postive
LIMS1	ANKRD44-IT1	0.522037453	9.85E-39	postive
IREB2	ANKRD44-IT1	0.595440545	1.23E-52	postive
DDX17	ANKRD44-IT1	0.597376996	4.72E-53	postive
PIK3CG	ANKRD44-IT1	0.571588933	9.88E-48	postive
JAK2	ANKRD44-IT1	0.64654041	1.19E-64	postive
TXK	ANKRD44-IT1	0.6159397	3.43E-57	postive
NFAT5	ANKRD44-IT1	0.868806824	7.84E-165	postive
NFATC3	ANKRD44-IT1	0.505078952	5.57E-36	postive
MALT1	ANKRD44-IT1	0.552698613	4.02E-44	postive
PIK3R1	ANKRD44-IT1	0.702266874	1.08E-80	postive
PIK3CA	ANKRD44-IT1	0.700938996	2.89E-80	postive
PIK3CB	ANKRD44-IT1	0.5189979	3.15E-38	postive
AKT3	ANKRD44-IT1	0.535949705	4.13E-41	postive
RASGRP3	ANKRD44-IT1	0.537566086	2.15E-41	postive
IGHD6-6	ANKRD44-IT1	0.551692687	6.16E-44	postive
PLXNC1	ANKRD44-IT1	0.512926583	3.10E-37	postive
GNRH1	ANKRD44-IT1	0.676206687	9.48E-73	postive
IL6ST	ANKRD44-IT1	0.701179385	2.42E-80	postive
RABEP1	ANKRD44-IT1	0.537322359	2.38E-41	postive
ACVR2A	ANKRD44-IT1	0.62598483	1.51E-59	postive
ANGPTL1	ANKRD44-IT1	0.783633889	2.78E-112	postive
BMPR1A	ANKRD44-IT1	0.531498414	2.45E-40	postive
BMPR2	ANKRD44-IT1	0.744036381	2.09E-95	postive
CRLF3	ANKRD44-IT1	0.639357444	8.00E-63	postive
LIFR	ANKRD44-IT1	0.51396862	2.10E-37	postive
NR1D2	ANKRD44-IT1	0.528026493	9.63E-40	postive
NR2C2	ANKRD44-IT1	0.657912837	1.20E-67	postive
RORA	ANKRD44-IT1	0.904984894	5.68E-200	postive

SOS1	ANKRD44-IT1	0.668251035	1.74E-70	postive
SOS2	ANKRD44-IT1	0.576471284	1.06E-48	postive
BRAF	ANKRD44-IT1	0.83055159	1.37E-137	postive
ITK	ANKRD44-IT1	0.585662414	1.41E-50	postive
CD28	ANKRD44-IT1	0.54687928	4.67E-43	postive
CBL	ANKRD44-IT1	0.586473472	9.60E-51	postive
CBLB	ANKRD44-IT1	0.730455875	2.55E-90	postive
RASGRP1	ANKRD44-IT1	0.528461497	8.12E-40	postive
PDK1	ANKRD44-IT1	0.575642729	1.55E-48	postive
TRAJ1	ANKRD44-IT1	0.746831227	1.71E-96	postive
TRAJ2	ANKRD44-IT1	0.795118269	7.39E-118	postive
TRAJ3	ANKRD44-IT1	0.825098685	2.88E-134	postive
TRAJ5	ANKRD44-IT1	0.841676852	9.35E-145	postive
TRAJ6	ANKRD44-IT1	0.818611645	1.84E-130	postive
TRAJ8	ANKRD44-IT1	0.809391174	2.63E-125	postive
TRAJ10	ANKRD44-IT1	0.798207892	2.03E-119	postive
TRAJ12	ANKRD44-IT1	0.754887996	1.04E-99	postive
TRAJ13	ANKRD44-IT1	0.841629537	1.01E-144	postive
TRAJ14	ANKRD44-IT1	0.822393271	1.16E-132	postive
TRAJ16	ANKRD44-IT1	0.825245073	2.35E-134	postive
TRAJ17	ANKRD44-IT1	0.791763111	3.42E-116	postive
TRAJ18	ANKRD44-IT1	0.800455467	1.43E-120	postive
TRAJ21	ANKRD44-IT1	0.876761859	1.39E-171	postive
TRAJ31	ANKRD44-IT1	0.880713214	4.09E-175	postive
TRAJ37	ANKRD44-IT1	0.868725947	9.14E-165	postive
TRAJ38	ANKRD44-IT1	0.902256569	7.35E-197	postive
TRAJ39	ANKRD44-IT1	0.887970608	6.16E-182	postive
CREB1	LINC-PINT	0.748306156	4.51E-97	postive
UBR1	LINC-PINT	0.621020648	2.26E-58	postive
ZC3HAV1	LINC-PINT	0.522527156	8.16E-39	postive
IL15	LINC-PINT	0.605124472	9.55E-55	postive
CYLD	LINC-PINT	0.597242336	5.05E-53	postive
EIF2AK2	LINC-PINT	0.511478704	5.31E-37	postive
MAPK8	LINC-PINT	0.54579021	7.36E-43	postive
LMBR1	LINC-PINT	0.571506195	1.03E-47	postive
IREB2	LINC-PINT	0.566083851	1.18E-46	postive
DDX17	LINC-PINT	0.609136608	1.21E-55	postive
PIK3CG	LINC-PINT	0.526737786	1.59E-39	postive
JAK2	LINC-PINT	0.6150068	5.62E-57	postive
TXK	LINC-PINT	0.594714473	1.76E-52	postive
NFAT5	LINC-PINT	0.791671427	3.80E-116	postive
MALT1	LINC-PINT	0.521381628	1.27E-38	postive
PIK3R1	LINC-PINT	0.661703356	1.13E-68	postive
PIK3CA	LINC-PINT	0.637699332	2.08E-62	postive
PIK3CB	LINC-PINT	0.519781987	2.34E-38	postive
AKT3	LINC-PINT	0.506068649	3.89E-36	postive
RASGRP3	LINC-PINT	0.517082517	6.51E-38	postive
GNRH1	LINC-PINT	0.711798732	8.12E-84	postive
IL6ST	LINC-PINT	0.669617407	7.20E-71	postive
RABEP1	LINC-PINT	0.509191589	1.24E-36	postive
ACVR2A	LINC-PINT	0.593003649	4.08E-52	postive
ANGPTL1	LINC-PINT	0.777428213	2.08E-109	postive
BMPR1A	LINC-PINT	0.524922601	3.23E-39	postive
BMPR2	LINC-PINT	0.703518061	4.28E-81	postive
CRLF3	LINC-PINT	0.616617146	2.39E-57	postive
NR1D2	LINC-PINT	0.517014782	6.68E-38	postive
NR2C2	LINC-PINT	0.683239782	8.23E-75	postive
NR3C1	LINC-PINT	0.50845945	1.62E-36	postive
RORA	LINC-PINT	0.858649247	7.94E-157	postive
SOS1	LINC-PINT	0.626134322	1.39E-59	postive
SOS2	LINC-PINT	0.535144857	5.71E-41	postive
BRAF	LINC-PINT	0.832386179	9.78E-139	postive
ITK	LINC-PINT	0.591422926	8.82E-52	postive
CD28	LINC-PINT	0.507079849	2.69E-36	postive
CBL	LINC-PINT	0.542756236	2.59E-42	postive

CBLB	LINC-PINT	0.701438382	2.00E-80	postive
RASGRP1	LINC-PINT	0.525319663	2.77E-39	postive
PDK1	LINC-PINT	0.585712746	1.38E-50	postive
TRAJ1	LINC-PINT	0.667266402	3.28E-70	postive
TRAJ2	LINC-PINT	0.70029744	4.63E-80	postive
TRAJ3	LINC-PINT	0.747572664	8.76E-97	postive
TRAJ5	LINC-PINT	0.782839496	6.57E-112	postive
TRAJ6	LINC-PINT	0.735653868	3.14E-92	postive
TRAJ8	LINC-PINT	0.718185394	5.53E-86	postive
TRAJ10	LINC-PINT	0.722774112	1.41E-87	postive
TRAJ12	LINC-PINT	0.687826517	3.46E-76	postive
TRAJ13	LINC-PINT	0.760955037	3.26E-102	postive
TRAJ14	LINC-PINT	0.738908987	1.90E-93	postive
TRAJ16	LINC-PINT	0.722463497	1.81E-87	postive
TRAJ17	LINC-PINT	0.712852593	3.60E-84	postive
TRAJ18	LINC-PINT	0.698937979	1.25E-79	postive
TRAJ21	LINC-PINT	0.789275498	5.61E-115	postive
TRAJ31	LINC-PINT	0.812736304	3.83E-127	postive
TRAJ37	LINC-PINT	0.780323753	9.74E-111	postive
TRAJ38	LINC-PINT	0.832787922	5.47E-139	postive
TRAJ39	LINC-PINT	0.790743135	1.08E-115	postive
CRABP1	AL513318.2	0.576759456	9.24E-49	postive
CREB1	AL132780.1	0.628918106	2.97E-60	postive
RFXAP	AL132780.1	0.550054602	1.23E-43	postive
UBR1	AL132780.1	0.527494143	1.19E-39	postive
ZC3HAV1	AL132780.1	0.501470802	2.05E-35	postive
ZC3HAV1L	AL132780.1	0.50266926	1.33E-35	postive
IREB2	AL132780.1	0.50416782	7.76E-36	postive
DDX17	AL132780.1	0.670415675	4.28E-71	postive
NFAT5	AL132780.1	0.661729644	1.11E-68	postive
PIK3R1	AL132780.1	0.500156361	3.29E-35	postive
PIK3CA	AL132780.1	0.50943778	1.13E-36	postive
LTB4R2	AL132780.1	0.570964517	1.31E-47	postive
GNRH1	AL132780.1	0.704296221	2.40E-81	postive
IL6ST	AL132780.1	0.50061301	2.79E-35	postive
ACVR2A	AL132780.1	0.547978954	2.95E-43	postive
ANGPTL1	AL132780.1	0.633574657	2.18E-61	postive
BMPR2	AL132780.1	0.526326954	1.87E-39	postive
CRLF3	AL132780.1	0.599873568	1.36E-53	postive
NR2C1	AL132780.1	0.556505352	7.85E-45	postive
NR2C2	AL132780.1	0.70117245	2.43E-80	postive
RORA	AL132780.1	0.639779873	6.26E-63	postive
SOS1	AL132780.1	0.579950924	2.10E-49	postive
SOS2	AL132780.1	0.554924645	1.55E-44	postive
BRAF	AL132780.1	0.747005768	1.46E-96	postive
CBL	AL132780.1	0.534995161	6.07E-41	postive
CBLB	AL132780.1	0.565126634	1.80E-46	postive
PDK1	AL132780.1	0.510172892	8.62E-37	postive
TRAJ1	AL132780.1	0.622337941	1.11E-58	postive
TRAJ2	AL132780.1	0.637020135	3.07E-62	postive
TRAJ3	AL132780.1	0.669131735	9.86E-71	postive
TRAJ5	AL132780.1	0.641813924	1.92E-63	postive
TRAJ6	AL132780.1	0.627341334	7.13E-60	postive
TRAJ8	AL132780.1	0.656782678	2.42E-67	postive
TRAJ10	AL132780.1	0.592013185	6.61E-52	postive
TRAJ12	AL132780.1	0.520898351	1.52E-38	postive
TRAJ13	AL132780.1	0.647530512	6.61E-65	postive
TRAJ14	AL132780.1	0.632995529	3.03E-61	postive
TRAJ16	AL132780.1	0.606035285	5.99E-55	postive
TRAJ17	AL132780.1	0.592091566	6.37E-52	postive
TRAJ18	AL132780.1	0.527090711	1.39E-39	postive
TRAJ21	AL132780.1	0.654516676	9.73E-67	postive
TRAJ31	AL132780.1	0.632106324	5.00E-61	postive
TRAJ37	AL132780.1	0.625506147	1.96E-59	postive
TRAJ38	AL132780.1	0.660287411	2.74E-68	postive

TRAJ39	AL132780.1	0.673925506	4.30E-72	postive
CREB1	AC007684.2	0.789851654	2.95E-115	postive
UBR1	AC007684.2	0.649119641	2.56E-65	postive
ZC3HAV1	AC007684.2	0.549881618	1.33E-43	postive
ZC3HAV1L	AC007684.2	0.514629598	1.64E-37	postive
IL15	AC007684.2	0.563347399	3.96E-46	postive
CYLD	AC007684.2	0.568344692	4.28E-47	postive
EIF2AK2	AC007684.2	0.54950072	1.56E-43	postive
MAPK8	AC007684.2	0.577000917	8.27E-49	postive
TLR1	AC007684.2	0.507802102	2.06E-36	postive
LMBR1	AC007684.2	0.603294014	2.42E-54	postive
LIMS1	AC007684.2	0.522652863	7.77E-39	postive
IREB2	AC007684.2	0.623392182	6.23E-59	postive
DDX17	AC007684.2	0.570078685	1.96E-47	postive
PIK3CG	AC007684.2	0.525723836	2.37E-39	postive
JAK2	AC007684.2	0.642052157	1.67E-63	postive
TXK	AC007684.2	0.587493183	5.89E-51	postive
PPP3CB	AC007684.2	0.507123413	2.64E-36	postive
NFAT5	AC007684.2	0.796726154	1.15E-118	postive
MALT1	AC007684.2	0.528247705	8.83E-40	postive
PIK3R1	AC007684.2	0.627569022	6.29E-60	postive
PIK3CA	AC007684.2	0.686204968	1.07E-75	postive
PIK3CB	AC007684.2	0.521706912	1.12E-38	postive
AKT3	AC007684.2	0.589108018	2.71E-51	postive
RASGRP3	AC007684.2	0.512212803	4.04E-37	postive
GNRH1	AC007684.2	0.724257295	4.23E-88	postive
IL6ST	AC007684.2	0.612377846	2.24E-56	postive
RABEP1	AC007684.2	0.557238201	5.72E-45	postive
ACVR2A	AC007684.2	0.623424835	6.12E-59	postive
ANGPTL1	AC007684.2	0.800850492	8.92E-121	postive
BMPR1A	AC007684.2	0.568697848	3.65E-47	postive
BMPR2	AC007684.2	0.747627259	8.34E-97	postive
CRLF3	AC007684.2	0.678199145	2.51E-73	postive
LIFR	AC007684.2	0.510373441	8.00E-37	postive
NR1D2	AC007684.2	0.52215167	9.42E-39	postive
NR2C2	AC007684.2	0.646236737	1.43E-64	postive
RORA	AC007684.2	0.79755328	4.37E-119	postive
SOS1	AC007684.2	0.686934523	6.44E-76	postive
SOS2	AC007684.2	0.561883271	7.55E-46	postive
BRAF	AC007684.2	0.820918575	8.49E-132	postive
CBL	AC007684.2	0.599991229	1.28E-53	postive
CBLB	AC007684.2	0.769109388	1.06E-105	postive
PDK1	AC007684.2	0.597190799	5.18E-53	postive
TRAJ1	AC007684.2	0.670443959	4.21E-71	postive
TRAJ2	AC007684.2	0.729355779	6.39E-90	postive
TRAJ3	AC007684.2	0.730110281	3.40E-90	postive
TRAJ5	AC007684.2	0.74178503	1.53E-94	postive
TRAJ6	AC007684.2	0.746552819	2.20E-96	postive
TRAJ8	AC007684.2	0.734094579	1.19E-91	postive
TRAJ10	AC007684.2	0.703556891	4.16E-81	postive
TRAJ12	AC007684.2	0.656038339	3.82E-67	postive
TRAJ13	AC007684.2	0.761008612	3.10E-102	postive
TRAJ14	AC007684.2	0.738010904	4.13E-93	postive
TRAJ16	AC007684.2	0.728578092	1.22E-89	postive
TRAJ17	AC007684.2	0.67180746	1.73E-71	postive
TRAJ18	AC007684.2	0.64588476	1.76E-64	postive
TRAJ21	AC007684.2	0.769747855	5.59E-106	postive
TRAJ31	AC007684.2	0.789711173	3.45E-115	postive
TRAJ37	AC007684.2	0.747698554	7.82E-97	postive
TRAJ38	AC007684.2	0.836900622	1.30E-141	postive
TRAJ39	AC007684.2	0.76657943	1.33E-104	postive
CREB1	ACTA2-AS1	0.635424391	7.64E-62	postive
CYLD	ACTA2-AS1	0.521934158	1.02E-38	postive
PDGFRA	ACTA2-AS1	0.60681222	4.02E-55	postive
NFKBIZ	ACTA2-AS1	0.504501996	6.87E-36	postive

LIMS1	ACTA2-AS1	0.518253853	4.18E-38	postive
DDX17	ACTA2-AS1	0.537503706	2.21E-41	postive
JAK2	ACTA2-AS1	0.621589369	1.66E-58	postive
TXK	ACTA2-AS1	0.512831971	3.21E-37	postive
NFAT5	ACTA2-AS1	0.669998335	5.62E-71	postive
PIK3R1	ACTA2-AS1	0.547934959	3.01E-43	postive
PIK3CA	ACTA2-AS1	0.570821483	1.40E-47	postive
RASGRP3	ACTA2-AS1	0.554866947	1.59E-44	postive
PLXNC1	ACTA2-AS1	0.50742414	2.37E-36	postive
FGF7	ACTA2-AS1	0.56907414	3.08E-47	postive
GNRH1	ACTA2-AS1	0.646890018	9.67E-65	postive
INHBA	ACTA2-AS1	0.539981778	8.06E-42	postive
ACVR2A	ACTA2-AS1	0.517487397	5.59E-38	postive
ANGPTL1	ACTA2-AS1	0.655519142	5.26E-67	postive
BMPR2	ACTA2-AS1	0.619359263	5.53E-58	postive
NR2C2	ACTA2-AS1	0.592401073	5.47E-52	postive
RORA	ACTA2-AS1	0.700762697	3.29E-80	postive
SOS1	ACTA2-AS1	0.562304252	6.28E-46	postive
BRAF	ACTA2-AS1	0.602011687	4.64E-54	postive
CBL	ACTA2-AS1	0.507333503	2.45E-36	postive
CBLB	ACTA2-AS1	0.694132373	4.03E-78	postive
PDK1	ACTA2-AS1	0.51310507	2.90E-37	postive
TRAJ1	ACTA2-AS1	0.581704347	9.21E-50	postive
TRAJ2	ACTA2-AS1	0.527162916	1.35E-39	postive
TRAJ3	ACTA2-AS1	0.547858239	3.10E-43	postive
TRAJ5	ACTA2-AS1	0.645611795	2.06E-64	postive
TRAJ6	ACTA2-AS1	0.588345339	3.91E-51	postive
TRAJ8	ACTA2-AS1	0.50385225	8.69E-36	postive
TRAJ10	ACTA2-AS1	0.591899363	6.99E-52	postive
TRAJ12	ACTA2-AS1	0.530123157	4.22E-40	postive
TRAJ13	ACTA2-AS1	0.562649936	5.39E-46	postive
TRAJ14	ACTA2-AS1	0.553849533	2.46E-44	postive
TRAJ16	ACTA2-AS1	0.581393398	1.07E-49	postive
TRAJ17	ACTA2-AS1	0.525303288	2.79E-39	postive
TRAJ18	ACTA2-AS1	0.554079002	2.23E-44	postive
TRAJ21	ACTA2-AS1	0.584331499	2.66E-50	postive
TRAJ31	ACTA2-AS1	0.628709398	3.34E-60	postive
TRAJ37	ACTA2-AS1	0.592524534	5.15E-52	postive
TRAJ38	ACTA2-AS1	0.663683606	3.23E-69	postive
TRAJ39	ACTA2-AS1	0.611046946	4.50E-56	postive
DDX17	CBR3-AS1	0.527429964	1.22E-39	postive
NR2C2	CBR3-AS1	0.518699477	3.53E-38	postive
RORA	CBR3-AS1	0.503460305	1.00E-35	postive
BRAF	CBR3-AS1	0.58221497	7.24E-50	postive
CREB1	AL136115.2	0.815680005	8.62E-129	postive
RFXAP	AL136115.2	0.50088929	2.53E-35	postive
UBR1	AL136115.2	0.654105678	1.25E-66	postive
ZC3HAV1	AL136115.2	0.566555429	9.53E-47	postive
ZC3HAV1L	AL136115.2	0.505527575	4.73E-36	postive
IL15	AL136115.2	0.607592196	2.69E-55	postive
CYLD	AL136115.2	0.621762858	1.51E-58	postive
EIF2AK2	AL136115.2	0.5616937	8.21E-46	postive
MAPK8	AL136115.2	0.585400111	1.60E-50	postive
TLR1	AL136115.2	0.512606082	3.49E-37	postive
LMBR1	AL136115.2	0.604319228	1.44E-54	postive
LIMS1	AL136115.2	0.538463169	1.50E-41	postive
IREB2	AL136115.2	0.627324925	7.20E-60	postive
DDX17	AL136115.2	0.597054413	5.54E-53	postive
PIK3CG	AL136115.2	0.539768811	8.79E-42	postive
JAK2	AL136115.2	0.667596328	2.65E-70	postive
TXK	AL136115.2	0.59471945	1.76E-52	postive
NFAT5	AL136115.2	0.864351783	3.06E-161	postive
MALT1	AL136115.2	0.552006166	5.39E-44	postive
PIK3R1	AL136115.2	0.638594365	1.24E-62	postive
PIK3CA	AL136115.2	0.735266497	4.37E-92	postive

PIK3CB	AL136115.2	0.556656393	7.36E-45	postive
AKT3	AL136115.2	0.547507035	3.60E-43	postive
RASGRP3	AL136115.2	0.545197776	9.42E-43	postive
PLXNC1	AL136115.2	0.509471509	1.12E-36	postive
GNRH1	AL136115.2	0.748721652	3.09E-97	postive
IL6ST	AL136115.2	0.633323025	2.52E-61	postive
IL7	AL136115.2	0.504253605	7.52E-36	postive
RABEP1	AL136115.2	0.561866044	7.61E-46	postive
ACVR2A	AL136115.2	0.659784641	3.75E-68	postive
ANGPTL1	AL136115.2	0.805730204	2.45E-123	postive
BMPR1A	AL136115.2	0.567454669	6.38E-47	postive
BMPR2	AL136115.2	0.765105022	5.71E-104	postive
CRLF3	AL136115.2	0.669914631	5.93E-71	postive
NR1D2	AL136115.2	0.522460021	8.37E-39	postive
NR2C2	AL136115.2	0.6880366	2.99E-76	postive
RORA	AL136115.2	0.856473799	3.41E-155	postive
SOS1	AL136115.2	0.711576186	9.63E-84	postive
SOS2	AL136115.2	0.598933017	2.18E-53	postive
BRAF	AL136115.2	0.805526912	3.15E-123	postive
ITK	AL136115.2	0.523852791	4.89E-39	postive
CBL	AL136115.2	0.612759351	1.84E-56	postive
CBLB	AL136115.2	0.756922806	1.54E-100	postive
RASGRP1	AL136115.2	0.505452904	4.86E-36	postive
PDK1	AL136115.2	0.612524625	2.08E-56	postive
TRAJ1	AL136115.2	0.751749773	1.93E-98	postive
TRAJ2	AL136115.2	0.743562202	3.19E-95	postive
TRAJ3	AL136115.2	0.794889767	9.62E-118	postive
TRAJ5	AL136115.2	0.826562278	3.79E-135	postive
TRAJ6	AL136115.2	0.819740051	4.11E-131	postive
TRAJ8	AL136115.2	0.792042083	2.49E-116	postive
TRAJ10	AL136115.2	0.811688162	1.45E-126	postive
TRAJ12	AL136115.2	0.733337262	2.26E-91	postive
TRAJ13	AL136115.2	0.824184198	1.01E-133	postive
TRAJ14	AL136115.2	0.794359434	1.77E-117	postive
TRAJ16	AL136115.2	0.826997765	2.06E-135	postive
TRAJ17	AL136115.2	0.788228513	1.80E-114	postive
TRAJ18	AL136115.2	0.751013573	3.80E-98	postive
TRAJ21	AL136115.2	0.842684069	1.97E-145	postive
TRAJ31	AL136115.2	0.873958271	3.75E-169	postive
TRAJ37	AL136115.2	0.83302095	3.90E-139	postive
TRAJ38	AL136115.2	0.895283544	2.63E-189	postive
TRAJ39	AL136115.2	0.848870341	1.09E-149	postive
NENF	TP53TG1	0.558141006	3.87E-45	postive
TYK2	AC138028.4	0.515397353	1.23E-37	postive
DDX17	AC138028.4	0.512417245	3.75E-37	postive
IRF9	AC138028.4	0.583441896	4.06E-50	postive
LTB4R2	AC138028.4	0.548289913	2.59E-43	postive
DDX17	AL021878.2	0.639111449	9.22E-63	postive
NFAT5	AL021878.2	0.564698065	2.18E-46	postive
LTB4R2	AL021878.2	0.537821328	1.94E-41	postive
GNRH1	AL021878.2	0.624063028	4.32E-59	postive
NR2C1	AL021878.2	0.504685772	6.43E-36	postive
NR2C2	AL021878.2	0.593879057	2.66E-52	postive
RORA	AL021878.2	0.548086861	2.82E-43	postive
BRAF	AL021878.2	0.541292012	4.72E-42	postive
TRAJ3	AL021878.2	0.521533603	1.20E-38	postive
TRAJ6	AL021878.2	0.501056751	2.38E-35	postive
TRAJ38	AL021878.2	0.523892635	4.82E-39	postive
LMBR1L	STAG3L5P-PVRIG2P-PI	0.527788554	1.06E-39	postive
TYK2	STAG3L5P-PVRIG2P-PI	0.511605184	5.07E-37	postive
IRF9	STAG3L5P-PVRIG2P-PI	0.561370873	9.46E-46	postive
CREB1	AC253576.2	0.747165091	1.27E-96	postive
RFXAP	AC253576.2	0.55376669	2.55E-44	postive
UBR1	AC253576.2	0.596984912	5.74E-53	postive
ZC3HAV1	AC253576.2	0.53113994	2.82E-40	postive

ZC3HAV1L	AC253576.2	0.533003968	1.35E-40	postive
IL15	AC253576.2	0.629818793	1.80E-60	postive
CYLD	AC253576.2	0.534350227	7.86E-41	postive
EIF2AK2	AC253576.2	0.524918302	3.24E-39	postive
MAPK8	AC253576.2	0.54038608	6.84E-42	postive
NFKBIZ	AC253576.2	0.500053429	3.41E-35	postive
LMBR1	AC253576.2	0.546394905	5.72E-43	postive
IREB2	AC253576.2	0.594741999	1.74E-52	postive
DDX17	AC253576.2	0.578374929	4.37E-49	postive
JAK2	AC253576.2	0.579447718	2.65E-49	postive
TXK	AC253576.2	0.514344108	1.83E-37	postive
NFAT5	AC253576.2	0.783391555	3.62E-112	postive
MALT1	AC253576.2	0.518345103	4.04E-38	postive
PIK3R1	AC253576.2	0.572565745	6.34E-48	postive
PIK3CA	AC253576.2	0.632649169	3.68E-61	postive
AKT3	AC253576.2	0.522312825	8.86E-39	postive
RASGRP3	AC253576.2	0.503634403	9.41E-36	postive
IGHD4-4	AC253576.2	0.551271133	7.37E-44	postive
LTB4R2	AC253576.2	0.521567752	1.18E-38	postive
GNRH1	AC253576.2	0.736430885	1.61E-92	postive
IL6ST	AC253576.2	0.568444627	4.09E-47	postive
RABEP1	AC253576.2	0.531743915	2.22E-40	postive
ACVR2A	AC253576.2	0.640556772	3.99E-63	postive
ANGPTL1	AC253576.2	0.72156852	3.72E-87	postive
BMPR1A	AC253576.2	0.545938689	6.92E-43	postive
BMPR2	AC253576.2	0.670565948	3.89E-71	postive
CRLF3	AC253576.2	0.626204569	1.34E-59	postive
NR2C2	AC253576.2	0.694725379	2.63E-78	postive
RORA	AC253576.2	0.744771472	1.09E-95	postive
SOS1	AC253576.2	0.661254686	1.49E-68	postive
SOS2	AC253576.2	0.586634708	8.88E-51	postive
BRAF	AC253576.2	0.769147417	1.02E-105	postive
CBL	AC253576.2	0.569667744	2.36E-47	postive
CBLB	AC253576.2	0.655492953	5.35E-67	postive
PDK1	AC253576.2	0.569749265	2.27E-47	postive
TRAJ1	AC253576.2	0.732981657	3.05E-91	postive
TRAJ2	AC253576.2	0.730273829	2.97E-90	postive
TRAJ3	AC253576.2	0.80452456	1.07E-122	postive
TRAJ5	AC253576.2	0.7682497	2.52E-105	postive
TRAJ6	AC253576.2	0.792695385	1.19E-116	postive
TRAJ8	AC253576.2	0.793680848	3.86E-117	postive
TRAJ10	AC253576.2	0.783521505	3.14E-112	postive
TRAJ12	AC253576.2	0.705490468	9.81E-82	postive
TRAJ13	AC253576.2	0.773214315	1.64E-107	postive
TRAJ14	AC253576.2	0.778694405	5.48E-110	postive
TRAJ16	AC253576.2	0.779360355	2.71E-110	postive
TRAJ17	AC253576.2	0.770952862	1.65E-106	postive
TRAJ18	AC253576.2	0.695227004	1.84E-78	postive
TRAJ21	AC253576.2	0.794729597	1.16E-117	postive
TRAJ31	AC253576.2	0.813023143	2.65E-127	postive
TRAJ37	AC253576.2	0.793612882	4.17E-117	postive
TRAJ38	AC253576.2	0.837266437	7.51E-142	postive
TRAJ39	AC253576.2	0.820336436	1.85E-131	postive
CREB1	AL133445.2	0.780466809	8.37E-111	postive
UBR1	AL133445.2	0.61248185	2.12E-56	postive
ZC3HAV1	AL133445.2	0.568955052	3.25E-47	postive
IL15	AL133445.2	0.637455078	2.39E-62	postive
CYLD	AL133445.2	0.614968145	5.74E-57	postive
EIF2AK2	AL133445.2	0.565882855	1.29E-46	postive
MAPK8	AL133445.2	0.550884241	8.68E-44	postive
TLR1	AL133445.2	0.519208592	2.91E-38	postive
LMBR1	AL133445.2	0.590402985	1.45E-51	postive
LIMS1	AL133445.2	0.545581915	8.03E-43	postive
IREB2	AL133445.2	0.588983684	2.87E-51	postive
DDX17	AL133445.2	0.564031713	2.93E-46	postive

PIK3CG	AL133445.2	0.564784103	2.10E-46	postive
JAK2	AL133445.2	0.661877824	1.01E-68	postive
TXK	AL133445.2	0.588131044	4.33E-51	postive
NFAT5	AL133445.2	0.808320177	1.00E-124	postive
MALT1	AL133445.2	0.55483887	1.61E-44	postive
PIK3R1	AL133445.2	0.650286961	1.27E-65	postive
PIK3CA	AL133445.2	0.740462127	4.90E-94	postive
PIK3CB	AL133445.2	0.56492782	1.97E-46	postive
AKT3	AL133445.2	0.554010512	2.29E-44	postive
RASGRP3	AL133445.2	0.546040598	6.63E-43	postive
IGHD6-6	AL133445.2	0.508468903	1.61E-36	postive
PLXNC1	AL133445.2	0.52128081	1.32E-38	postive
GNRH1	AL133445.2	0.674276465	3.41E-72	postive
IL6ST	AL133445.2	0.631891461	5.64E-61	postive
IL7	AL133445.2	0.512948407	3.08E-37	postive
RABEP1	AL133445.2	0.534325092	7.94E-41	postive
ACVR2A	AL133445.2	0.606849718	3.95E-55	postive
ANGPTL1	AL133445.2	0.827359389	1.24E-135	postive
BMPR1A	AL133445.2	0.506722917	3.06E-36	postive
BMPR2	AL133445.2	0.73251625	4.52E-91	postive
CRLF3	AL133445.2	0.646540631	1.19E-64	postive
IL18R1	AL133445.2	0.529543397	5.30E-40	postive
NR2C2	AL133445.2	0.65293859	2.55E-66	postive
RORA	AL133445.2	0.827438839	1.11E-135	postive
SOS1	AL133445.2	0.687122029	5.65E-76	postive
SOS2	AL133445.2	0.5939513	2.56E-52	postive
BRAF	AL133445.2	0.79726018	6.16E-119	postive
ITK	AL133445.2	0.561699585	8.19E-46	postive
CD28	AL133445.2	0.50686834	2.90E-36	postive
CBL	AL133445.2	0.62634215	1.24E-59	postive
CBLB	AL133445.2	0.751222716	3.13E-98	postive
RASGRP1	AL133445.2	0.5246681	3.57E-39	postive
PDK1	AL133445.2	0.61232781	2.30E-56	postive
TRAJ1	AL133445.2	0.742247624	1.02E-94	postive
TRAJ2	AL133445.2	0.727980656	2.00E-89	postive
TRAJ3	AL133445.2	0.806719976	7.27E-124	postive
TRAJ5	AL133445.2	0.828475145	2.60E-136	postive
TRAJ6	AL133445.2	0.805779131	2.31E-123	postive
TRAJ8	AL133445.2	0.794382871	1.72E-117	postive
TRAJ10	AL133445.2	0.796897468	9.40E-119	postive
TRAJ12	AL133445.2	0.724845909	2.62E-88	postive
TRAJ13	AL133445.2	0.819121434	9.36E-131	postive
TRAJ14	AL133445.2	0.809454414	2.43E-125	postive
TRAJ16	AL133445.2	0.828543274	2.36E-136	postive
TRAJ17	AL133445.2	0.809837071	1.50E-125	postive
TRAJ18	AL133445.2	0.7670371	8.43E-105	postive
TRAJ21	AL133445.2	0.856985084	1.42E-155	postive
TRAJ31	AL133445.2	0.853088612	1.05E-152	postive
TRAJ37	AL133445.2	0.803901362	2.28E-122	postive
TRAJ38	AL133445.2	0.8833478	1.54E-177	postive
TRAJ39	AL133445.2	0.860139262	5.83E-158	postive
TYK2	TNRC6C-AS1	0.525544285	2.54E-39	postive
CREB1	AC007849.1	0.666335402	5.96E-70	postive
UBR1	AC007849.1	0.613127008	1.51E-56	postive
IREB2	AC007849.1	0.5554902	1.22E-44	postive
DDX17	AC007849.1	0.501033881	2.40E-35	postive
TXK	AC007849.1	0.515568271	1.15E-37	postive
NFAT5	AC007849.1	0.713247284	2.65E-84	postive
PIK3CA	AC007849.1	0.530148407	4.18E-40	postive
ROBO2	AC007849.1	0.711796749	8.13E-84	postive
GNRH1	AC007849.1	0.704600185	1.91E-81	postive
ACVR2A	AC007849.1	0.647645134	6.17E-65	postive
ANGPTL1	AC007849.1	0.522240624	9.11E-39	postive
BMPR1A	AC007849.1	0.599008078	2.10E-53	postive
BMPR2	AC007849.1	0.676691568	6.87E-73	postive

NR1D2	AC007849.1	0.552496319	4.38E-44	postive
NR2C2	AC007849.1	0.586991769	7.49E-51	postive
RORA	AC007849.1	0.671032296	2.87E-71	postive
SOS1	AC007849.1	0.547779739	3.21E-43	postive
BRAF	AC007849.1	0.599343577	1.77E-53	postive
TRAJ1	AC007849.1	0.547709907	3.30E-43	postive
TRAJ2	AC007849.1	0.513153235	2.85E-37	postive
TRAJ3	AC007849.1	0.578322754	4.48E-49	postive
TRAJ5	AC007849.1	0.554011024	2.29E-44	postive
TRAJ6	AC007849.1	0.612885453	1.72E-56	postive
TRAJ8	AC007849.1	0.569420126	2.64E-47	postive
TRAJ10	AC007849.1	0.594047765	2.44E-52	postive
TRAJ12	AC007849.1	0.622885625	8.21E-59	postive
TRAJ13	AC007849.1	0.571506268	1.03E-47	postive
TRAJ14	AC007849.1	0.535737597	4.50E-41	postive
TRAJ16	AC007849.1	0.57585351	1.40E-48	postive
TRAJ17	AC007849.1	0.563964827	3.02E-46	postive
TRAJ18	AC007849.1	0.546981463	4.48E-43	postive
TRAJ21	AC007849.1	0.577801432	5.70E-49	postive
TRAJ31	AC007849.1	0.661674651	1.15E-68	postive
TRAJ37	AC007849.1	0.641858189	1.87E-63	postive
TRAJ38	AC007849.1	0.646855624	9.87E-65	postive
TRAJ39	AC007849.1	0.587979423	4.66E-51	postive
CD1D	LINC01781	0.501730347	1.87E-35	postive
HLA-DOB	LINC01781	0.504544298	6.77E-36	postive
LTA	LINC01781	0.562918433	4.79E-46	postive
CXCL13	LINC01781	0.780251982	1.05E-110	postive
PTGDS	LINC01781	0.632741832	3.49E-61	postive
RBP5	LINC01781	0.669199277	9.43E-71	postive
CD40LG	LINC01781	0.561068093	1.08E-45	postive
CCL19	LINC01781	0.678999775	1.46E-73	postive
CCR7	LINC01781	0.675487274	1.53E-72	postive
CXCR4	LINC01781	0.715432258	4.83E-85	postive
CD79A	LINC01781	0.66278939	5.69E-69	postive
CD79B	LINC01781	0.735927759	2.48E-92	postive
CD19	LINC01781	0.780354862	9.43E-111	postive
CR2	LINC01781	0.7697084	5.81E-106	postive
CD22	LINC01781	0.713023646	3.15E-84	postive
PLCG2	LINC01781	0.560625237	1.31E-45	postive
PRKCB	LINC01781	0.540100321	7.68E-42	postive
ACKR1	LINC01781	0.506028	3.95E-36	postive
IL16	LINC01781	0.650910192	8.71E-66	postive
IL24	LINC01781	0.5901432	1.64E-51	postive
LTB	LINC01781	0.543935955	1.59E-42	postive
TNFSF8	LINC01781	0.611725336	3.16E-56	postive
TNFRSF13C	LINC01781	0.803523629	3.60E-122	postive
NCR3	LINC01781	0.777538812	1.85E-109	postive
CD48	LINC01781	0.600363947	1.06E-53	postive
CD3E	LINC01781	0.5555854	1.17E-44	postive
ITK	LINC01781	0.511265989	5.75E-37	postive
TRAC	LINC01781	0.511888521	4.56E-37	postive
TRBC2	LINC01781	0.505121616	5.49E-36	postive
TRBV5-1	LINC01781	0.581877691	8.49E-50	postive
TRBV18	LINC01781	0.539483806	9.88E-42	postive
TRBV20-1	LINC01781	0.533193905	1.25E-40	postive
TRBV28	LINC01781	0.534082161	8.75E-41	postive
LTA	COPDA1	0.591560565	8.25E-52	postive
CXCL13	COPDA1	0.539071861	1.17E-41	postive
PTGDS	COPDA1	0.528235313	8.87E-40	postive
RBP5	COPDA1	0.602035146	4.58E-54	postive
CCL19	COPDA1	0.563809774	3.23E-46	postive
CCR7	COPDA1	0.593342498	3.45E-52	postive
CD79A	COPDA1	0.601512423	5.96E-54	postive
CD79B	COPDA1	0.684631665	3.17E-75	postive
CD19	COPDA1	0.730995124	1.62E-90	postive

CR2	COPDA1	0.517103594	6.46E-38	postive
CD22	COPDA1	0.65592151	4.11E-67	postive
PLCG2	COPDA1	0.560191528	1.58E-45	postive
IL16	COPDA1	0.61988803	4.16E-58	postive
LTB	COPDA1	0.514518569	1.71E-37	postive
PNOC	COPDA1	0.510116501	8.80E-37	postive
TNFRSF13C	COPDA1	0.693273801	7.44E-78	postive
NCR3	COPDA1	0.624653064	3.13E-59	postive
ZAP70	COPDA1	0.509506553	1.10E-36	postive
CD3E	COPDA1	0.503319833	1.05E-35	postive
TRAC	COPDA1	0.505343884	5.06E-36	postive
TRAV9-2	COPDA1	0.502305208	1.52E-35	postive
TRBC1	COPDA1	0.505672705	4.49E-36	postive
TRBV5-1	COPDA1	0.506890776	2.88E-36	postive
CREB1	AC008966.2	0.699653949	7.41E-80	postive
UBR1	AC008966.2	0.554571989	1.80E-44	postive
IL15	AC008966.2	0.555159485	1.40E-44	postive
CYLD	AC008966.2	0.551156263	7.73E-44	postive
EIF2AK2	AC008966.2	0.505536337	4.72E-36	postive
NFKBIZ	AC008966.2	0.522517377	8.19E-39	postive
LMBR1	AC008966.2	0.530382882	3.81E-40	postive
LIMS1	AC008966.2	0.502820622	1.26E-35	postive
IREB2	AC008966.2	0.518894767	3.28E-38	postive
DDX17	AC008966.2	0.571333193	1.11E-47	postive
JAK2	AC008966.2	0.589136138	2.67E-51	postive
TXK	AC008966.2	0.58961341	2.12E-51	postive
NFAT5	AC008966.2	0.710139974	2.90E-83	postive
MALT1	AC008966.2	0.514211062	1.92E-37	postive
PIK3R1	AC008966.2	0.623049896	7.51E-59	postive
PIK3CA	AC008966.2	0.638544633	1.28E-62	postive
PIK3CB	AC008966.2	0.511903302	4.54E-37	postive
GNRH1	AC008966.2	0.667574566	2.69E-70	postive
IL6ST	AC008966.2	0.657031206	2.07E-67	postive
ACVR2A	AC008966.2	0.525646929	2.44E-39	postive
ANGPTL1	AC008966.2	0.756506439	2.28E-100	postive
BMPR2	AC008966.2	0.695326567	1.71E-78	postive
CRLF3	AC008966.2	0.561462993	9.08E-46	postive
NR2C2	AC008966.2	0.609142476	1.21E-55	postive
RORA	AC008966.2	0.804056684	1.89E-122	postive
SOS1	AC008966.2	0.57880661	3.57E-49	postive
SOS2	AC008966.2	0.506600322	3.20E-36	postive
BRAF	AC008966.2	0.742833695	6.08E-95	postive
ITK	AC008966.2	0.535046459	5.94E-41	postive
CBL	AC008966.2	0.548523501	2.35E-43	postive
CBLB	AC008966.2	0.758047362	5.28E-101	postive
RASGRP1	AC008966.2	0.501778585	1.84E-35	postive
PDK1	AC008966.2	0.553159457	3.30E-44	postive
TRAJ1	AC008966.2	0.516302071	8.74E-38	postive
TRAJ2	AC008966.2	0.576433101	1.07E-48	postive
TRAJ3	AC008966.2	0.605035397	9.99E-55	postive
TRAJ5	AC008966.2	0.649924923	1.58E-65	postive
TRAJ6	AC008966.2	0.585458857	1.56E-50	postive
TRAJ8	AC008966.2	0.529675802	5.03E-40	postive
TRAJ10	AC008966.2	0.595493577	1.20E-52	postive
TRAJ12	AC008966.2	0.512917857	3.11E-37	postive
TRAJ13	AC008966.2	0.596410979	7.63E-53	postive
TRAJ14	AC008966.2	0.570376308	1.71E-47	postive
TRAJ16	AC008966.2	0.589756086	1.98E-51	postive
TRAJ17	AC008966.2	0.556869928	6.71E-45	postive
TRAJ18	AC008966.2	0.579788703	2.26E-49	postive
TRAJ21	AC008966.2	0.640829825	3.41E-63	postive
TRAJ31	AC008966.2	0.672771572	9.19E-72	postive
TRAJ37	AC008966.2	0.628415462	3.93E-60	postive
TRAJ38	AC008966.2	0.70448621	2.08E-81	postive
TRAJ39	AC008966.2	0.647743725	5.82E-65	postive

BIRC5	TMPO-AS1	0.564419907	2.47E-46	postive
LTB4R2	AC018653.3	0.509455863	1.12E-36	postive
IL15	AL157871.2	0.558240685	3.71E-45	postive
JAK2	AL157871.2	0.519623883	2.48E-38	postive
IL18RAP	AL157871.2	0.51169996	4.89E-37	postive
SOS1	AL157871.2	0.50474238	6.30E-36	postive
TRAJ1	AL157871.2	0.665658127	9.20E-70	postive
TRAJ3	AL157871.2	0.607671585	2.59E-55	postive
TRAJ5	AL157871.2	0.656823076	2.36E-67	postive
TRAJ6	AL157871.2	0.597786581	3.85E-53	postive
TRAJ8	AL157871.2	0.622479145	1.02E-58	postive
TRAJ10	AL157871.2	0.574832894	2.25E-48	postive
TRAJ12	AL157871.2	0.661722989	1.11E-68	postive
TRAJ13	AL157871.2	0.616199199	2.99E-57	postive
TRAJ14	AL157871.2	0.674977382	2.15E-72	postive
TRAJ16	AL157871.2	0.611468313	3.61E-56	postive
TRAJ17	AL157871.2	0.650108501	1.41E-65	postive
TRAJ18	AL157871.2	0.596150315	8.68E-53	postive
TRAJ21	AL157871.2	0.639945235	5.69E-63	postive
TRAJ31	AL157871.2	0.596937509	5.87E-53	postive
TRAJ37	AL157871.2	0.519914937	2.22E-38	postive
TRAJ38	AL157871.2	0.609289186	1.12E-55	postive
TRAJ39	AL157871.2	0.599954975	1.30E-53	postive
NFAT5	JPX	0.525997788	2.13E-39	postive
TRAJ31	JPX	0.508857108	1.40E-36	postive
TRAJ37	JPX	0.514295329	1.86E-37	postive
TRAJ38	JPX	0.51858109	3.69E-38	postive
FOS	NR4A1AS	0.621677786	1.58E-58	postive
NR4A1	NR4A1AS	0.500052861	3.41E-35	postive
CREB1	ALMS1-IT1	0.605879489	6.49E-55	postive
ZC3HAV1L	ALMS1-IT1	0.518940574	3.22E-38	postive
EIF2AK2	ALMS1-IT1	0.543709961	1.74E-42	postive
MAPK8	ALMS1-IT1	0.526611462	1.67E-39	postive
LMBR1	ALMS1-IT1	0.522307846	8.88E-39	postive
LIMS1	ALMS1-IT1	0.507203859	2.57E-36	postive
IREB2	ALMS1-IT1	0.541413443	4.49E-42	postive
NFAT5	ALMS1-IT1	0.630998626	9.31E-61	postive
PIK3CA	ALMS1-IT1	0.620321264	3.29E-58	postive
GSK3B	ALMS1-IT1	0.546335803	5.86E-43	postive
GNRH1	ALMS1-IT1	0.520919786	1.51E-38	postive
ANGPTL1	ALMS1-IT1	0.584176865	2.86E-50	postive
BMPR2	ALMS1-IT1	0.549676067	1.45E-43	postive
CRLF3	ALMS1-IT1	0.579402751	2.71E-49	postive
NR2C2	ALMS1-IT1	0.507534602	2.27E-36	postive
RORA	ALMS1-IT1	0.600034573	1.25E-53	postive
SOS1	ALMS1-IT1	0.657972613	1.16E-67	postive
BRAF	ALMS1-IT1	0.680759076	4.46E-74	postive
CBL	ALMS1-IT1	0.632267229	4.57E-61	postive
CBLB	ALMS1-IT1	0.613571661	1.20E-56	postive
PDK1	ALMS1-IT1	0.531860168	2.12E-40	postive
TRAJ1	ALMS1-IT1	0.527444555	1.21E-39	postive
TRAJ2	ALMS1-IT1	0.565544539	1.50E-46	postive
TRAJ3	ALMS1-IT1	0.590813463	1.19E-51	postive
TRAJ5	ALMS1-IT1	0.610088935	7.40E-56	postive
TRAJ6	ALMS1-IT1	0.579337566	2.79E-49	postive
TRAJ8	ALMS1-IT1	0.58548673	1.54E-50	postive
TRAJ10	ALMS1-IT1	0.554342698	1.99E-44	postive
TRAJ13	ALMS1-IT1	0.61088551	4.89E-56	postive
TRAJ14	ALMS1-IT1	0.586386909	1.00E-50	postive
TRAJ16	ALMS1-IT1	0.57909187	3.13E-49	postive
TRAJ17	ALMS1-IT1	0.548191993	2.70E-43	postive
TRAJ18	ALMS1-IT1	0.500878696	2.54E-35	postive
TRAJ21	ALMS1-IT1	0.61944469	5.28E-58	postive
TRAJ31	ALMS1-IT1	0.629874823	1.75E-60	postive
TRAJ37	ALMS1-IT1	0.601873963	4.97E-54	postive

TRAJ38	ALMS1-IT1	0.642237279	1.50E-63	postive
TRAJ39	ALMS1-IT1	0.629846275	1.77E-60	postive
IGLV3-16	AC015722.2	0.539016384	1.19E-41	postive
CREB1	AL133342.1	0.72601114	1.01E-88	postive
UBR1	AL133342.1	0.559628753	2.03E-45	postive
ZC3HAV1	AL133342.1	0.505739185	4.38E-36	postive
TLR4	AL133342.1	0.554635348	1.76E-44	postive
IL15	AL133342.1	0.673324361	6.39E-72	postive
CYLD	AL133342.1	0.592425791	5.41E-52	postive
EIF2AK2	AL133342.1	0.515137884	1.36E-37	postive
TLR1	AL133342.1	0.573202737	4.74E-48	postive
LMBR1	AL133342.1	0.53243451	1.69E-40	postive
LIMS1	AL133342.1	0.563426242	3.83E-46	postive
IREB2	AL133342.1	0.55189454	5.65E-44	postive
DDX17	AL133342.1	0.505386728	4.98E-36	postive
TANK	AL133342.1	0.510852236	6.70E-37	postive
PIK3CG	AL133342.1	0.594685776	1.79E-52	postive
JAK2	AL133342.1	0.69373523	5.35E-78	postive
TXK	AL133342.1	0.553272906	3.14E-44	postive
NFAT5	AL133342.1	0.744578796	1.29E-95	postive
MALT1	AL133342.1	0.535351549	5.26E-41	postive
PIK3R1	AL133342.1	0.586854125	8.00E-51	postive
PIK3CA	AL133342.1	0.721866489	2.93E-87	postive
PIK3CB	AL133342.1	0.528907076	6.81E-40	postive
RASGRP3	AL133342.1	0.569106726	3.04E-47	postive
PLXNC1	AL133342.1	0.549738997	1.41E-43	postive
GNRH1	AL133342.1	0.647414464	7.08E-65	postive
IL6ST	AL133342.1	0.53661867	3.16E-41	postive
IL7	AL133342.1	0.571730561	9.27E-48	postive
RABEP1	AL133342.1	0.506562481	3.25E-36	postive
ACVR2A	AL133342.1	0.566869151	8.29E-47	postive
ANGPTL1	AL133342.1	0.765799072	2.88E-104	postive
BMPR2	AL133342.1	0.657891809	1.22E-67	postive
CRLF3	AL133342.1	0.654455476	1.01E-66	postive
IL18R1	AL133342.1	0.563457375	3.77E-46	postive
NR2C2	AL133342.1	0.61108122	4.42E-56	postive
RORA	AL133342.1	0.742452969	8.51E-95	postive
SOS1	AL133342.1	0.624821861	2.85E-59	postive
SOS2	AL133342.1	0.551029688	8.16E-44	postive
BRAF	AL133342.1	0.683501156	6.88E-75	postive
PTPRC	AL133342.1	0.534764543	6.65E-41	postive
ITK	AL133342.1	0.552525184	4.32E-44	postive
CD28	AL133342.1	0.517615685	5.32E-38	postive
CBL	AL133342.1	0.583266517	4.41E-50	postive
CBLB	AL133342.1	0.698819693	1.36E-79	postive
RASGRP1	AL133342.1	0.500179134	3.26E-35	postive
PDK1	AL133342.1	0.638535908	1.28E-62	postive
TRAJ1	AL133342.1	0.717569333	9.00E-86	postive
TRAJ2	AL133342.1	0.663164168	4.49E-69	postive
TRAJ3	AL133342.1	0.767870507	3.67E-105	postive
TRAJ5	AL133342.1	0.758434859	3.65E-101	postive
TRAJ6	AL133342.1	0.769224146	9.47E-106	postive
TRAJ8	AL133342.1	0.719774462	1.56E-86	postive
TRAJ10	AL133342.1	0.772491427	3.45E-107	postive
TRAJ12	AL133342.1	0.686701945	7.57E-76	postive
TRAJ13	AL133342.1	0.733655601	1.72E-91	postive
TRAJ14	AL133342.1	0.740914207	3.29E-94	postive
TRAJ16	AL133342.1	0.794324788	1.84E-117	postive
TRAJ17	AL133342.1	0.772226098	4.52E-107	postive
TRAJ18	AL133342.1	0.742816897	6.17E-95	postive
TRAJ21	AL133342.1	0.779706438	1.88E-110	postive
TRAJ31	AL133342.1	0.781061041	4.44E-111	postive
TRAJ37	AL133342.1	0.729027339	8.39E-90	postive
TRAJ38	AL133342.1	0.820058693	2.69E-131	postive
TRAJ39	AL133342.1	0.794332616	1.83E-117	postive

GAL	BANCR	0.536334307	3.54E-41	postive
OSGIN1	AP003119.2	0.510383765	7.97E-37	postive
GNRH2	AC009686.2	0.833946995	1.01E-139	postive
VGF	AC009686.2	0.699928212	6.06E-80	postive
CREB1	AC007216.4	0.74036872	5.31E-94	postive
RFXAP	AC007216.4	0.550545568	1.00E-43	postive
UBR1	AC007216.4	0.6601814	2.93E-68	postive
ZC3HAV1	AC007216.4	0.569010182	3.17E-47	postive
ZC3HAV1L	AC007216.4	0.546510297	5.45E-43	postive
IL15	AC007216.4	0.546553128	5.36E-43	postive
CYLD	AC007216.4	0.566485529	9.84E-47	postive
EIF2AK2	AC007216.4	0.507957321	1.95E-36	postive
MAPK8	AC007216.4	0.566576616	9.44E-47	postive
LMBR1	AC007216.4	0.540256969	7.21E-42	postive
IREB2	AC007216.4	0.608627674	1.58E-55	postive
DDX17	AC007216.4	0.666759596	4.54E-70	postive
PIK3CG	AC007216.4	0.515277154	1.29E-37	postive
JAK2	AC007216.4	0.56460979	2.27E-46	postive
TXK	AC007216.4	0.585510399	1.52E-50	postive
NFAT5	AC007216.4	0.781839196	1.93E-111	postive
MALT1	AC007216.4	0.537277841	2.42E-41	postive
PIK3R1	AC007216.4	0.645590511	2.09E-64	postive
PIK3CA	AC007216.4	0.64075702	3.55E-63	postive
PIK3CB	AC007216.4	0.516467447	8.21E-38	postive
AKT3	AC007216.4	0.535434412	5.09E-41	postive
IGHD4-4	AC007216.4	0.543429624	1.96E-42	postive
IGHD6-6	AC007216.4	0.548555222	2.32E-43	postive
LTB4R2	AC007216.4	0.525130714	2.98E-39	postive
GNRH1	AC007216.4	0.768382139	2.20E-105	postive
IL6ST	AC007216.4	0.645915743	1.72E-64	postive
RABEP1	AC007216.4	0.534763321	6.66E-41	postive
ACVR2A	AC007216.4	0.651668598	5.50E-66	postive
ANGPTL1	AC007216.4	0.730648765	2.17E-90	postive
BMPR1A	AC007216.4	0.573525982	4.09E-48	postive
BMPR2	AC007216.4	0.701672549	1.68E-80	postive
CRLF3	AC007216.4	0.59623468	8.32E-53	postive
NR2C1	AC007216.4	0.546116689	6.43E-43	postive
NR2C2	AC007216.4	0.756003844	3.65E-100	postive
NR3C1	AC007216.4	0.504222302	7.60E-36	postive
RORA	AC007216.4	0.817034696	1.47E-129	postive
SOS1	AC007216.4	0.643909865	5.63E-64	postive
SOS2	AC007216.4	0.576574805	1.01E-48	postive
BRAF	AC007216.4	0.819729721	4.17E-131	postive
ITK	AC007216.4	0.531611427	2.34E-40	postive
CBL	AC007216.4	0.569754168	2.27E-47	postive
CBLB	AC007216.4	0.644666757	3.61E-64	postive
RASGRP1	AC007216.4	0.508598265	1.54E-36	postive
PDK1	AC007216.4	0.580098159	1.96E-49	postive
TRAJ1	AC007216.4	0.647569093	6.46E-65	postive
TRAJ2	AC007216.4	0.663196671	4.40E-69	postive
TRAJ3	AC007216.4	0.736325512	1.77E-92	postive
TRAJ5	AC007216.4	0.72419459	4.45E-88	postive
TRAJ6	AC007216.4	0.69390789	4.73E-78	postive
TRAJ8	AC007216.4	0.693466932	6.48E-78	postive
TRAJ10	AC007216.4	0.68473841	2.94E-75	postive
TRAJ12	AC007216.4	0.609282848	1.12E-55	postive
TRAJ13	AC007216.4	0.701646633	1.71E-80	postive
TRAJ14	AC007216.4	0.67299143	7.95E-72	postive
TRAJ16	AC007216.4	0.681099439	3.54E-74	postive
TRAJ17	AC007216.4	0.683649205	6.22E-75	postive
TRAJ18	AC007216.4	0.628815576	3.15E-60	postive
TRAJ21	AC007216.4	0.728930282	9.09E-90	postive
TRAJ31	AC007216.4	0.739643664	1.00E-93	postive
TRAJ37	AC007216.4	0.722189719	2.26E-87	postive
TRAJ38	AC007216.4	0.756516647	2.25E-100	postive

TRAJ39	AC007216.4	0.757149374	1.24E-100	postive
EPOR	AC020558.2	0.537963212	1.83E-41	postive
CREB1	AC092123.1	0.500935979	2.49E-35	postive
DDX17	AC092123.1	0.622663728	9.27E-59	postive
NFAT5	AC092123.1	0.649711756	1.79E-65	postive
GNRH1	AC092123.1	0.704553833	1.98E-81	postive
ANGPTL1	AC092123.1	0.510198433	8.54E-37	postive
NR2C2	AC092123.1	0.643194919	8.57E-64	postive
RORA	AC092123.1	0.589343735	2.42E-51	postive
BRAF	AC092123.1	0.556307997	8.55E-45	postive
CBLB	AC092123.1	0.522394816	8.58E-39	postive
TRAJ1	AC092123.1	0.533780329	9.87E-41	postive
TRAJ2	AC092123.1	0.545332652	8.90E-43	postive
TRAJ3	AC092123.1	0.566706797	8.91E-47	postive
TRAJ5	AC092123.1	0.541238355	4.82E-42	postive
TRAJ6	AC092123.1	0.564727783	2.15E-46	postive
TRAJ8	AC092123.1	0.503013869	1.18E-35	postive
TRAJ10	AC092123.1	0.541115621	5.07E-42	postive
TRAJ13	AC092123.1	0.528012075	9.68E-40	postive
TRAJ14	AC092123.1	0.514784765	1.55E-37	postive
TRAJ16	AC092123.1	0.531926247	2.07E-40	postive
TRAJ18	AC092123.1	0.514118964	1.99E-37	postive
TRAJ21	AC092123.1	0.552009347	5.38E-44	postive
TRAJ31	AC092123.1	0.543637345	1.80E-42	postive
TRAJ37	AC092123.1	0.552499505	4.37E-44	postive
TRAJ38	AC092123.1	0.584642231	2.30E-50	postive
TRAJ39	AC092123.1	0.566559697	9.52E-47	postive
CREB1	AL049869.3	0.801016663	7.32E-121	postive
RFXAP	AL049869.3	0.529793898	4.81E-40	postive
UBR1	AL049869.3	0.649530146	2.00E-65	postive
ZC3HAV1	AL049869.3	0.580649538	1.51E-49	postive
ZC3HAV1L	AL049869.3	0.516740282	7.41E-38	postive
IL15	AL049869.3	0.614898343	5.95E-57	postive
CYLD	AL049869.3	0.634203853	1.53E-61	postive
EIF2AK2	AL049869.3	0.553800337	2.51E-44	postive
MAPK8	AL049869.3	0.562150421	6.71E-46	postive
LMBR1	AL049869.3	0.581799161	8.81E-50	postive
IREB2	AL049869.3	0.612972127	1.64E-56	postive
DDX17	AL049869.3	0.635906064	5.80E-62	postive
PIK3CG	AL049869.3	0.522239779	9.11E-39	postive
JAK2	AL049869.3	0.642662533	1.17E-63	postive
TXK	AL049869.3	0.566567551	9.48E-47	postive
NFAT5	AL049869.3	0.886058391	4.29E-180	postive
NFATC3	AL049869.3	0.520473941	1.79E-38	postive
MALT1	AL049869.3	0.538541384	1.45E-41	postive
PIK3R1	AL049869.3	0.626247635	1.30E-59	postive
PIK3CA	AL049869.3	0.694142598	4.00E-78	postive
PIK3CB	AL049869.3	0.53992128	8.26E-42	postive
AKT3	AL049869.3	0.54993235	1.30E-43	postive
RASGRP3	AL049869.3	0.531093018	2.88E-40	postive
PLXNC1	AL049869.3	0.515612575	1.13E-37	postive
GNRH1	AL049869.3	0.756433436	2.44E-100	postive
IL6ST	AL049869.3	0.607759723	2.47E-55	postive
RABEP1	AL049869.3	0.578343736	4.43E-49	postive
ACVR2A	AL049869.3	0.667582688	2.68E-70	postive
ANGPTL1	AL049869.3	0.775667353	1.31E-108	postive
BMPR1A	AL049869.3	0.548827995	2.07E-43	postive
BMPR2	AL049869.3	0.716529678	2.04E-85	postive
CRLF3	AL049869.3	0.648753869	3.18E-65	postive
NR1D2	AL049869.3	0.503573769	9.62E-36	postive
NR2C2	AL049869.3	0.727710226	2.50E-89	postive
RORA	AL049869.3	0.828915012	1.40E-136	postive
SOS1	AL049869.3	0.715888301	3.38E-85	postive
SOS2	AL049869.3	0.629797985	1.82E-60	postive
BRAF	AL049869.3	0.814847427	2.54E-128	postive

ITK	AL049869.3	0.515803473	1.06E-37	postive
CBL	AL049869.3	0.632253971	4.60E-61	postive
CBLB	AL049869.3	0.708865357	7.67E-83	postive
RASGRP1	AL049869.3	0.526679515	1.63E-39	postive
PDK1	AL049869.3	0.568871741	3.38E-47	postive
TRAJ1	AL049869.3	0.840043845	1.14E-143	postive
TRAJ2	AL049869.3	0.777963609	1.18E-109	postive
TRAJ3	AL049869.3	0.867716079	6.11E-164	postive
TRAJ5	AL049869.3	0.884496227	1.30E-178	postive
TRAJ6	AL049869.3	0.886406023	1.99E-180	postive
TRAJ8	AL049869.3	0.872845533	3.34E-168	postive
TRAJ10	AL049869.3	0.873349696	1.24E-168	postive
TRAJ12	AL049869.3	0.822836979	6.36E-133	postive
TRAJ13	AL049869.3	0.89469305	1.08E-188	postive
TRAJ14	AL049869.3	0.878536025	3.74E-173	postive
TRAJ16	AL049869.3	0.873242185	1.53E-168	postive
TRAJ17	AL049869.3	0.866188326	1.05E-162	postive
TRAJ18	AL049869.3	0.817037808	1.46E-129	postive
TRAJ21	AL049869.3	0.909674426	1.51E-205	postive
TRAJ31	AL049869.3	0.91711417	4.75E-215	postive
TRAJ37	AL049869.3	0.892443554	2.21E-186	postive
TRAJ38	AL049869.3	0.925649322	4.06E-227	postive
TRAJ39	AL049869.3	0.909421206	3.07E-205	postive
SLPI	AC129507.3	0.521102177	1.41E-38	postive
RABEP2	AC009065.5	0.538962951	1.22E-41	postive
TNFRSF14	AC009065.5	0.514530518	1.70E-37	postive
DDX17	AC004148.1	0.630991458	9.35E-61	postive
LTB4R2	AC004148.1	0.513766184	2.27E-37	postive
GNRH1	AC004148.1	0.654338869	1.08E-66	postive
NR2C1	AC004148.1	0.535540678	4.87E-41	postive
NR2C2	AC004148.1	0.626164765	1.36E-59	postive
CREB1	LINC00513	0.717579266	8.93E-86	postive
UBR1	LINC00513	0.61240844	2.21E-56	postive
ZC3HAV1	LINC00513	0.520600673	1.71E-38	postive
IL15	LINC00513	0.556650829	7.37E-45	postive
CYLD	LINC00513	0.548368363	2.51E-43	postive
MAPK8	LINC00513	0.556219593	8.88E-45	postive
LMBR1	LINC00513	0.541320107	4.66E-42	postive
IREB2	LINC00513	0.574393241	2.75E-48	postive
DDX17	LINC00513	0.55545899	1.23E-44	postive
JAK2	LINC00513	0.558689235	3.05E-45	postive
TXK	LINC00513	0.548475932	2.40E-43	postive
NFAT5	LINC00513	0.780464527	8.39E-111	postive
PIK3R1	LINC00513	0.590608099	1.31E-51	postive
PIK3CA	LINC00513	0.637138886	2.87E-62	postive
PIK3CB	LINC00513	0.5052577	5.22E-36	postive
GNRH1	LINC00513	0.673263836	6.65E-72	postive
IL6ST	LINC00513	0.579981331	2.07E-49	postive
RABEP1	LINC00513	0.507966276	1.94E-36	postive
ACVR2A	LINC00513	0.584246676	2.77E-50	postive
ANGPTL1	LINC00513	0.734490586	8.48E-92	postive
BMPR1A	LINC00513	0.541232761	4.83E-42	postive
BMPR2	LINC00513	0.66556802	9.74E-70	postive
CRLF3	LINC00513	0.571057722	1.26E-47	postive
NR2C2	LINC00513	0.640642535	3.80E-63	postive
NR3C1	LINC00513	0.507319269	2.46E-36	postive
RORA	LINC00513	0.795961551	2.79E-118	postive
SOS1	LINC00513	0.626892101	9.14E-60	postive
SOS2	LINC00513	0.543665901	1.78E-42	postive
BRAF	LINC00513	0.767618382	4.73E-105	postive
CBL	LINC00513	0.520341232	1.89E-38	postive
CBLB	LINC00513	0.642899668	1.02E-63	postive
PDK1	LINC00513	0.564557832	2.32E-46	postive
TRAJ1	LINC00513	0.666366198	5.85E-70	postive
TRAJ2	LINC00513	0.68053106	5.20E-74	postive

TRAJ3	LINC00513	0.746939574	1.55E-96	postive
TRAJ5	LINC00513	0.751448681	2.54E-98	postive
TRAJ6	LINC00513	0.750567252	5.72E-98	postive
TRAJ8	LINC00513	0.721262656	4.76E-87	postive
TRAJ10	LINC00513	0.737459955	6.65E-93	postive
TRAJ12	LINC00513	0.688252204	2.57E-76	postive
TRAJ13	LINC00513	0.746723325	1.89E-96	postive
TRAJ14	LINC00513	0.735200369	4.63E-92	postive
TRAJ16	LINC00513	0.732596245	4.22E-91	postive
TRAJ17	LINC00513	0.723178598	1.02E-87	postive
TRAJ18	LINC00513	0.700052713	5.53E-80	postive
TRAJ21	LINC00513	0.782931818	5.95E-112	postive
TRAJ31	LINC00513	0.795429392	5.16E-118	postive
TRAJ37	LINC00513	0.769745195	5.60E-106	postive
TRAJ38	LINC00513	0.816531021	2.84E-129	postive
TRAJ39	LINC00513	0.789198312	6.12E-115	postive
CREB1	HECW2-AS1	0.515955814	9.96E-38	postive
PDGFRA	HECW2-AS1	0.513928622	2.13E-37	postive
LIMS1	HECW2-AS1	0.580727257	1.46E-49	postive
PIK3CG	HECW2-AS1	0.563352237	3.95E-46	postive
JAK2	HECW2-AS1	0.545518975	8.24E-43	postive
PLXNC1	HECW2-AS1	0.617802642	1.27E-57	postive
CRLF3	HECW2-AS1	0.515095446	1.38E-37	postive
RORA	HECW2-AS1	0.500029898	3.44E-35	postive
PTPRC	HECW2-AS1	0.509011972	1.32E-36	postive
CD28	HECW2-AS1	0.558698202	3.04E-45	postive
CBLB	HECW2-AS1	0.504357657	7.24E-36	postive
PDK1	HECW2-AS1	0.518030588	4.55E-38	postive
TRAJ3	HECW2-AS1	0.501962457	1.72E-35	postive
TRAJ38	HECW2-AS1	0.504724995	6.34E-36	postive
CREB1	AC010536.2	0.716838429	1.60E-85	postive
RFXAP	AC010536.2	0.525700601	2.39E-39	postive
UBR1	AC010536.2	0.643293	8.09E-64	postive
ZC3HAV1	AC010536.2	0.513831505	2.21E-37	postive
IL15	AC010536.2	0.543138195	2.21E-42	postive
CYLD	AC010536.2	0.587231722	6.67E-51	postive
MAPK8	AC010536.2	0.548659134	2.22E-43	postive
LMBR1	AC010536.2	0.503599423	9.53E-36	postive
IREB2	AC010536.2	0.574895856	2.18E-48	postive
DDX17	AC010536.2	0.621636661	1.62E-58	postive
JAK2	AC010536.2	0.556879558	6.68E-45	postive
TXK	AC010536.2	0.587635632	5.50E-51	postive
NFAT5	AC010536.2	0.877893829	1.39E-172	postive
NFATC3	AC010536.2	0.561217273	1.01E-45	postive
MALT1	AC010536.2	0.513203955	2.80E-37	postive
PIK3R1	AC010536.2	0.579286917	2.86E-49	postive
PIK3CA	AC010536.2	0.612004264	2.73E-56	postive
IGHD4-4	AC010536.2	0.550564217	9.93E-44	postive
IGHD6-6	AC010536.2	0.59797231	3.51E-53	postive
GNRH1	AC010536.2	0.797949491	2.75E-119	postive
IL6ST	AC010536.2	0.584582579	2.36E-50	postive
RABEP1	AC010536.2	0.550027807	1.25E-43	postive
ACVR2A	AC010536.2	0.604351369	1.42E-54	postive
ANGPTL1	AC010536.2	0.656761351	2.45E-67	postive
BMPR1A	AC010536.2	0.530971198	3.02E-40	postive
BMPR2	AC010536.2	0.66647329	5.46E-70	postive
CRLF3	AC010536.2	0.56963799	2.39E-47	postive
NR2C2	AC010536.2	0.686452149	9.00E-76	postive
RORA	AC010536.2	0.830035464	2.85E-137	postive
SOS1	AC010536.2	0.600466857	1.01E-53	postive
SOS2	AC010536.2	0.510841468	6.73E-37	postive
BRAF	AC010536.2	0.706305263	5.32E-82	postive
TEC	AC010536.2	0.513460051	2.54E-37	postive
CBL	AC010536.2	0.546311309	5.92E-43	postive
CBLB	AC010536.2	0.604715085	1.18E-54	postive

PDK1	AC010536.2	0.575796675	1.44E-48	postive
TRAJ1	AC010536.2	0.673707925	4.96E-72	postive
TRAJ2	AC010536.2	0.696182321	9.24E-79	postive
TRAJ3	AC010536.2	0.768940304	1.26E-105	postive
TRAJ5	AC010536.2	0.680671323	4.73E-74	postive
TRAJ6	AC010536.2	0.751773236	1.89E-98	postive
TRAJ8	AC010536.2	0.674480724	2.98E-72	postive
TRAJ10	AC010536.2	0.756738531	1.83E-100	postive
TRAJ12	AC010536.2	0.631487201	7.08E-61	postive
TRAJ13	AC010536.2	0.686949225	6.38E-76	postive
TRAJ14	AC010536.2	0.6579513	1.17E-67	postive
TRAJ16	AC010536.2	0.720022602	1.28E-86	postive
TRAJ17	AC010536.2	0.680459045	5.46E-74	postive
TRAJ18	AC010536.2	0.703479735	4.41E-81	postive
TRAJ21	AC010536.2	0.729956162	3.87E-90	postive
TRAJ31	AC010536.2	0.75186416	1.73E-98	postive
TRAJ37	AC010536.2	0.792142858	2.22E-116	postive
TRAJ38	AC010536.2	0.771315951	1.14E-106	postive
TRAJ39	AC010536.2	0.772716792	2.74E-107	postive
CREB1	AC025165.4	0.534248602	8.18E-41	postive
DDX17	AC025165.4	0.519207751	2.91E-38	postive
NFAT5	AC025165.4	0.594542296	1.92E-52	postive
LTB4R2	AC025165.4	0.554757211	1.67E-44	postive
GNRH1	AC025165.4	0.598643962	2.51E-53	postive
ANGPTL1	AC025165.4	0.511515146	5.24E-37	postive
NR2C2	AC025165.4	0.595247717	1.35E-52	postive
RORA	AC025165.4	0.577825549	5.64E-49	postive
BRAF	AC025165.4	0.51783012	4.91E-38	postive
CBLB	AC025165.4	0.523146028	6.43E-39	postive
TRAJ1	AC025165.4	0.551717679	6.09E-44	postive
TRAJ2	AC025165.4	0.509892021	9.56E-37	postive
TRAJ3	AC025165.4	0.585146767	1.81E-50	postive
TRAJ5	AC025165.4	0.549268566	1.72E-43	postive
TRAJ6	AC025165.4	0.564252253	2.66E-46	postive
TRAJ8	AC025165.4	0.540413717	6.76E-42	postive
TRAJ10	AC025165.4	0.569406211	2.65E-47	postive
TRAJ12	AC025165.4	0.506086888	3.86E-36	postive
TRAJ13	AC025165.4	0.536026977	4.01E-41	postive
TRAJ14	AC025165.4	0.536016739	4.02E-41	postive
TRAJ16	AC025165.4	0.596669493	6.71E-53	postive
TRAJ17	AC025165.4	0.563085871	4.45E-46	postive
TRAJ18	AC025165.4	0.539857977	8.48E-42	postive
TRAJ21	AC025165.4	0.560595511	1.33E-45	postive
TRAJ31	AC025165.4	0.578146117	4.86E-49	postive
TRAJ37	AC025165.4	0.551422189	6.91E-44	postive
TRAJ38	AC025165.4	0.601950899	4.78E-54	postive
TRAJ39	AC025165.4	0.578550227	4.03E-49	postive
CREB1	TPT1-AS1	0.568078968	4.82E-47	postive
RFXAP	TPT1-AS1	0.580655659	1.51E-49	postive
UBR1	TPT1-AS1	0.509102063	1.28E-36	postive
DDX17	TPT1-AS1	0.685243555	2.08E-75	postive
TXK	TPT1-AS1	0.60012131	1.20E-53	postive
NFAT5	TPT1-AS1	0.654834282	8.01E-67	postive
PIK3CA	TPT1-AS1	0.504339251	7.29E-36	postive
ROBO2	TPT1-AS1	0.506283852	3.59E-36	postive
GNRH1	TPT1-AS1	0.793784307	3.43E-117	postive
ACVR2A	TPT1-AS1	0.618372418	9.38E-58	postive
ANGPTL1	TPT1-AS1	0.606215899	5.46E-55	postive
BMPR2	TPT1-AS1	0.588955158	2.91E-51	postive
NR2C1	TPT1-AS1	0.568524708	3.95E-47	postive
NR2C2	TPT1-AS1	0.698581384	1.62E-79	postive
RORA	TPT1-AS1	0.66867688	1.32E-70	postive
SOS1	TPT1-AS1	0.5503648	1.08E-43	postive
BRAF	TPT1-AS1	0.592563906	5.06E-52	postive
CBLB	TPT1-AS1	0.569380479	2.68E-47	postive

TRAJ1	TPT1-AS1	0.534847718	6.44E-41	postive
TRAJ2	TPT1-AS1	0.522444462	8.42E-39	postive
TRAJ3	TPT1-AS1	0.561044434	1.09E-45	postive
TRAJ5	TPT1-AS1	0.557574601	4.95E-45	postive
TRAJ6	TPT1-AS1	0.551301957	7.27E-44	postive
TRAJ8	TPT1-AS1	0.518647707	3.60E-38	postive
TRAJ10	TPT1-AS1	0.535437544	5.08E-41	postive
TRAJ12	TPT1-AS1	0.52471309	3.51E-39	postive
TRAJ13	TPT1-AS1	0.530449074	3.71E-40	postive
TRAJ14	TPT1-AS1	0.507837754	2.04E-36	postive
TRAJ16	TPT1-AS1	0.54883915	2.06E-43	postive
TRAJ17	TPT1-AS1	0.517728172	5.10E-38	postive
TRAJ18	TPT1-AS1	0.523938768	4.73E-39	postive
TRAJ21	TPT1-AS1	0.560564613	1.35E-45	postive
TRAJ31	TPT1-AS1	0.585914406	1.25E-50	postive
TRAJ37	TPT1-AS1	0.570265425	1.80E-47	postive
TRAJ38	TPT1-AS1	0.603840983	1.84E-54	postive
TRAJ39	TPT1-AS1	0.570500696	1.62E-47	postive
FABP5	AC010136.1	0.519730716	2.38E-38	postive
CREB1	AL122035.1	0.62446044	3.48E-59	postive
UBR1	AL122035.1	0.544566428	1.22E-42	postive
ZC3HAV1	AL122035.1	0.515998827	9.80E-38	postive
CYLD	AL122035.1	0.574395173	2.75E-48	postive
IREB2	AL122035.1	0.517543361	5.47E-38	postive
PIK3CG	AL122035.1	0.585404072	1.60E-50	postive
JAK2	AL122035.1	0.550314409	1.10E-43	postive
NFAT5	AL122035.1	0.626108306	1.41E-59	postive
PIK3R1	AL122035.1	0.638649906	1.20E-62	postive
PIK3CA	AL122035.1	0.571740246	9.23E-48	postive
AKT3	AL122035.1	0.545450487	8.48E-43	postive
PLXNC1	AL122035.1	0.502863339	1.24E-35	postive
GNRH1	AL122035.1	0.506010407	3.97E-36	postive
IL6ST	AL122035.1	0.562675637	5.33E-46	postive
RABEP1	AL122035.1	0.525916283	2.20E-39	postive
ACVR2A	AL122035.1	0.523814603	4.96E-39	postive
ANGPTL1	AL122035.1	0.606373973	5.04E-55	postive
BMPR2	AL122035.1	0.611196379	4.16E-56	postive
NR1D2	AL122035.1	0.508116127	1.84E-36	postive
NR2C2	AL122035.1	0.554196758	2.12E-44	postive
RORA	AL122035.1	0.6519601	4.61E-66	postive
SOS1	AL122035.1	0.567513086	6.21E-47	postive
SOS2	AL122035.1	0.597216842	5.11E-53	postive
BRAF	AL122035.1	0.608058159	2.12E-55	postive
CD28	AL122035.1	0.513065723	2.94E-37	postive
CBL	AL122035.1	0.560153894	1.61E-45	postive
CBLB	AL122035.1	0.527730728	1.08E-39	postive
TRAJ1	AL122035.1	0.531524473	2.42E-40	postive
TRAJ2	AL122035.1	0.587089485	7.15E-51	postive
TRAJ3	AL122035.1	0.610236163	6.86E-56	postive
TRAJ5	AL122035.1	0.54900912	1.91E-43	postive
TRAJ6	AL122035.1	0.567548639	6.11E-47	postive
TRAJ8	AL122035.1	0.554199603	2.12E-44	postive
TRAJ10	AL122035.1	0.51902766	3.11E-38	postive
TRAJ12	AL122035.1	0.501295976	2.18E-35	postive
TRAJ13	AL122035.1	0.548812195	2.08E-43	postive
TRAJ14	AL122035.1	0.552080055	5.22E-44	postive
TRAJ16	AL122035.1	0.528228986	8.89E-40	postive
TRAJ17	AL122035.1	0.514134842	1.97E-37	postive
TRAJ18	AL122035.1	0.527439236	1.21E-39	postive
TRAJ21	AL122035.1	0.571516065	1.02E-47	postive
TRAJ31	AL122035.1	0.547624716	3.42E-43	postive
TRAJ37	AL122035.1	0.55784431	4.40E-45	postive
TRAJ38	AL122035.1	0.593642707	2.98E-52	postive
TRAJ39	AL122035.1	0.591315585	9.29E-52	postive
CREB1	SCAANT1	0.577347993	7.04E-49	postive

EIF2AK2	SCAANT1	0.506874952	2.90E-36	postive
NFAT5	SCAANT1	0.602606169	3.43E-54	postive
PIK3CA	SCAANT1	0.54405851	1.51E-42	postive
GNRH1	SCAANT1	0.618420215	9.14E-58	postive
ACVR2A	SCAANT1	0.520586029	1.72E-38	postive
ANGPTL1	SCAANT1	0.549013801	1.91E-43	postive
BMPR2	SCAANT1	0.519739556	2.37E-38	postive
CRLF3	SCAANT1	0.509186126	1.24E-36	postive
NR2C2	SCAANT1	0.68002818	7.31E-74	postive
RORA	SCAANT1	0.560112992	1.64E-45	postive
SOS1	SCAANT1	0.561976552	7.25E-46	postive
BRAF	SCAANT1	0.658654747	7.58E-68	postive
CBLB	SCAANT1	0.522667832	7.73E-39	postive
TRAJ1	SCAANT1	0.686301108	1.00E-75	postive
TRAJ2	SCAANT1	0.55179238	5.90E-44	postive
TRAJ3	SCAANT1	0.675825877	1.22E-72	postive
TRAJ5	SCAANT1	0.714912527	7.25E-85	postive
TRAJ6	SCAANT1	0.676023527	1.07E-72	postive
TRAJ8	SCAANT1	0.717342873	1.08E-85	postive
TRAJ10	SCAANT1	0.661669592	1.15E-68	postive
TRAJ12	SCAANT1	0.68682826	6.94E-76	postive
TRAJ13	SCAANT1	0.683009684	9.64E-75	postive
TRAJ14	SCAANT1	0.699727231	7.03E-80	postive
TRAJ16	SCAANT1	0.708680172	8.83E-83	postive
TRAJ17	SCAANT1	0.733359353	2.22E-91	postive
TRAJ18	SCAANT1	0.623105969	7.28E-59	postive
TRAJ21	SCAANT1	0.704400062	2.22E-81	postive
TRAJ31	SCAANT1	0.707176391	2.76E-82	postive
TRAJ37	SCAANT1	0.654840074	7.98E-67	postive
TRAJ38	SCAANT1	0.702968431	6.44E-81	postive
TRAJ39	SCAANT1	0.722587341	1.64E-87	postive
CREB1	SDCBP2-AS1	0.756761087	1.79E-100	postive
RFXAP	SDCBP2-AS1	0.557365953	5.41E-45	postive
UBR1	SDCBP2-AS1	0.611760016	3.10E-56	postive
ZC3HAV1	SDCBP2-AS1	0.56210021	6.86E-46	postive
ZC3HAV1L	SDCBP2-AS1	0.500917943	2.50E-35	postive
IL15	SDCBP2-AS1	0.55812947	3.89E-45	postive
CYLD	SDCBP2-AS1	0.58015517	1.90E-49	postive
EIF2AK2	SDCBP2-AS1	0.546493763	5.49E-43	postive
MAPK8	SDCBP2-AS1	0.588887058	3.01E-51	postive
LMBR1	SDCBP2-AS1	0.521317286	1.30E-38	postive
IREB2	SDCBP2-AS1	0.58775625	5.19E-51	postive
DDX17	SDCBP2-AS1	0.657622665	1.44E-67	postive
JAK2	SDCBP2-AS1	0.571677826	9.49E-48	postive
TXK	SDCBP2-AS1	0.558552222	3.24E-45	postive
NFAT5	SDCBP2-AS1	0.815682194	8.59E-129	postive
MALT1	SDCBP2-AS1	0.530629329	3.45E-40	postive
PIK3R1	SDCBP2-AS1	0.627901886	5.23E-60	postive
PIK3CA	SDCBP2-AS1	0.667966564	2.09E-70	postive
PIK3CB	SDCBP2-AS1	0.500959631	2.46E-35	postive
AKT3	SDCBP2-AS1	0.520063356	2.10E-38	postive
IGHD6-6	SDCBP2-AS1	0.528305751	8.63E-40	postive
GNRH1	SDCBP2-AS1	0.75315662	5.24E-99	postive
IL6ST	SDCBP2-AS1	0.606401549	4.97E-55	postive
RABEP1	SDCBP2-AS1	0.557320369	5.52E-45	postive
ACVR2A	SDCBP2-AS1	0.654091889	1.26E-66	postive
ANGPTL1	SDCBP2-AS1	0.785053991	5.94E-113	postive
BMPR1A	SDCBP2-AS1	0.550254353	1.13E-43	postive
BMPR2	SDCBP2-AS1	0.678348402	2.27E-73	postive
CRLF3	SDCBP2-AS1	0.601236297	6.85E-54	postive
NR2C2	SDCBP2-AS1	0.745871273	4.06E-96	postive
RORA	SDCBP2-AS1	0.823408847	2.92E-133	postive
SOS1	SDCBP2-AS1	0.692833255	1.02E-77	postive
SOS2	SDCBP2-AS1	0.587742694	5.22E-51	postive
BRAF	SDCBP2-AS1	0.783946897	1.98E-112	postive

ITK	SDCBP2-AS1	0.512449452	3.70E-37	postive
CBL	SDCBP2-AS1	0.587961261	4.70E-51	postive
CBLB	SDCBP2-AS1	0.682828627	1.09E-74	postive
PDK1	SDCBP2-AS1	0.581526862	1.00E-49	postive
TRAJ1	SDCBP2-AS1	0.720187092	1.13E-86	postive
TRAJ2	SDCBP2-AS1	0.694674941	2.73E-78	postive
TRAJ3	SDCBP2-AS1	0.781017686	4.65E-111	postive
TRAJ5	SDCBP2-AS1	0.79010347	2.22E-115	postive
TRAJ6	SDCBP2-AS1	0.771274861	1.19E-106	postive
TRAJ8	SDCBP2-AS1	0.757245736	1.13E-100	postive
TRAJ10	SDCBP2-AS1	0.760509054	5.01E-102	postive
TRAJ12	SDCBP2-AS1	0.703724296	3.67E-81	postive
TRAJ13	SDCBP2-AS1	0.77748593	1.96E-109	postive
TRAJ14	SDCBP2-AS1	0.761475098	1.97E-102	postive
TRAJ16	SDCBP2-AS1	0.770145604	3.74E-106	postive
TRAJ17	SDCBP2-AS1	0.763601424	2.49E-103	postive
TRAJ18	SDCBP2-AS1	0.719687842	1.68E-86	postive
TRAJ21	SDCBP2-AS1	0.809367043	2.71E-125	postive
TRAJ31	SDCBP2-AS1	0.808317236	1.01E-124	postive
TRAJ37	SDCBP2-AS1	0.786467313	1.26E-113	postive
TRAJ38	SDCBP2-AS1	0.831174222	5.60E-138	postive
TRAJ39	SDCBP2-AS1	0.821391008	4.50E-132	postive
CREB1	AC135050.5	0.568634272	3.76E-47	postive
UBR1	AC135050.5	0.511703065	4.89E-37	postive
DDX17	AC135050.5	0.674217482	3.55E-72	postive
NFAT5	AC135050.5	0.639335058	8.10E-63	postive
LTB4R2	AC135050.5	0.530611063	3.48E-40	postive
GNRH1	AC135050.5	0.585827537	1.31E-50	postive
IL6ST	AC135050.5	0.525375976	2.71E-39	postive
ANGPTL1	AC135050.5	0.541784445	3.86E-42	postive
NR2C1	AC135050.5	0.511567724	5.14E-37	postive
NR2C2	AC135050.5	0.694453086	3.20E-78	postive
RORA	AC135050.5	0.673713004	4.95E-72	postive
SOS1	AC135050.5	0.554770137	1.66E-44	postive
BRAF	AC135050.5	0.670596899	3.81E-71	postive
CBLB	AC135050.5	0.621437429	1.80E-58	postive
TRAJ1	AC135050.5	0.519309578	2.80E-38	postive
TRAJ2	AC135050.5	0.561393328	9.36E-46	postive
TRAJ3	AC135050.5	0.566920989	8.10E-47	postive
TRAJ5	AC135050.5	0.58052731	1.60E-49	postive
TRAJ6	AC135050.5	0.532294998	1.78E-40	postive
TRAJ8	AC135050.5	0.518869782	3.31E-38	postive
TRAJ10	AC135050.5	0.509248712	1.21E-36	postive
TRAJ13	AC135050.5	0.55631152	8.54E-45	postive
TRAJ14	AC135050.5	0.549402409	1.62E-43	postive
TRAJ16	AC135050.5	0.537279077	2.42E-41	postive
TRAJ17	AC135050.5	0.502296252	1.52E-35	postive
TRAJ18	AC135050.5	0.525108624	3.01E-39	postive
TRAJ21	AC135050.5	0.578659048	3.83E-49	postive
TRAJ31	AC135050.5	0.568350073	4.27E-47	postive
TRAJ37	AC135050.5	0.571238314	1.16E-47	postive
TRAJ38	AC135050.5	0.608640833	1.57E-55	postive
TRAJ39	AC135050.5	0.576900809	8.66E-49	postive
NGFR	LINC00958	0.697276728	4.19E-79	postive
CREB1	Z68871.1	0.815848238	6.93E-129	postive
RFXAP	Z68871.1	0.517947477	4.69E-38	postive
AP3B1	Z68871.1	0.512420416	3.74E-37	postive
UBR1	Z68871.1	0.767149578	7.54E-105	postive
ZC3HAV1	Z68871.1	0.631178439	8.42E-61	postive
ZC3HAV1L	Z68871.1	0.574386614	2.76E-48	postive
IFNAR1	Z68871.1	0.50514305	5.44E-36	postive
IL15	Z68871.1	0.523751859	5.09E-39	postive
CYLD	Z68871.1	0.705143	1.27E-81	postive
EIF2AK2	Z68871.1	0.592135006	6.23E-52	postive
MAPK8	Z68871.1	0.549691052	1.44E-43	postive

TLR1	Z68871.1	0.536659586	3.10E-41	postive
LMBR1	Z68871.1	0.621399873	1.84E-58	postive
LIMS1	Z68871.1	0.505646222	4.53E-36	postive
IREB2	Z68871.1	0.677822619	3.22E-73	postive
DDX17	Z68871.1	0.600180647	1.17E-53	postive
PIK3CG	Z68871.1	0.633497423	2.28E-61	postive
JAK2	Z68871.1	0.701859577	1.46E-80	postive
TXK	Z68871.1	0.571662087	9.56E-48	postive
NFAT5	Z68871.1	0.864589806	1.98E-161	postive
NFATC3	Z68871.1	0.637931667	1.82E-62	postive
MALT1	Z68871.1	0.548379776	2.49E-43	postive
PIK3R1	Z68871.1	0.659749469	3.83E-68	postive
PIK3CA	Z68871.1	0.718055973	6.13E-86	postive
PIK3CB	Z68871.1	0.538880911	1.26E-41	postive
AKT3	Z68871.1	0.507797467	2.07E-36	postive
GSK3B	Z68871.1	0.512151364	4.14E-37	postive
RASGRP3	Z68871.1	0.520326871	1.90E-38	postive
PLXNC1	Z68871.1	0.539072867	1.17E-41	postive
GNRH1	Z68871.1	0.670328828	4.53E-71	postive
IL6ST	Z68871.1	0.627283099	7.36E-60	postive
RABEP1	Z68871.1	0.615702052	3.89E-57	postive
ACVR2A	Z68871.1	0.615775044	3.74E-57	postive
ANGPTL1	Z68871.1	0.652191984	4.01E-66	postive
BMPR1A	Z68871.1	0.592415671	5.44E-52	postive
BMPR2	Z68871.1	0.711588729	9.54E-84	postive
CRLF3	Z68871.1	0.64972258	1.78E-65	postive
LIFR	Z68871.1	0.515308136	1.27E-37	postive
NR1D2	Z68871.1	0.624638149	3.16E-59	postive
NR2C2	Z68871.1	0.722083386	2.46E-87	postive
NR3C1	Z68871.1	0.513688705	2.33E-37	postive
RORA	Z68871.1	0.815792326	7.45E-129	postive
SOS1	Z68871.1	0.678270705	2.39E-73	postive
SOS2	Z68871.1	0.604768034	1.15E-54	postive
BRAF	Z68871.1	0.784078958	1.72E-112	postive
TEC	Z68871.1	0.538744983	1.33E-41	postive
CBL	Z68871.1	0.603332219	2.38E-54	postive
CBLB	Z68871.1	0.66072664	2.08E-68	postive
PDK1	Z68871.1	0.562584352	5.55E-46	postive
TRAJ1	Z68871.1	0.706169828	5.89E-82	postive
TRAJ2	Z68871.1	0.748601416	3.45E-97	postive
TRAJ3	Z68871.1	0.766629864	1.26E-104	postive
TRAJ5	Z68871.1	0.695856284	1.17E-78	postive
TRAJ6	Z68871.1	0.774782592	3.27E-108	postive
TRAJ8	Z68871.1	0.685382741	1.89E-75	postive
TRAJ10	Z68871.1	0.753123117	5.41E-99	postive
TRAJ12	Z68871.1	0.715345492	5.17E-85	postive
TRAJ13	Z68871.1	0.702126679	1.20E-80	postive
TRAJ14	Z68871.1	0.709861804	3.59E-83	postive
TRAJ16	Z68871.1	0.739795681	8.76E-94	postive
TRAJ17	Z68871.1	0.679367591	1.14E-73	postive
TRAJ18	Z68871.1	0.73208622	6.49E-91	postive
TRAJ21	Z68871.1	0.736948538	1.03E-92	postive
TRAJ31	Z68871.1	0.749075651	2.24E-97	postive
TRAJ37	Z68871.1	0.756516515	2.25E-100	postive
TRAJ38	Z68871.1	0.825919646	9.24E-135	postive
TRAJ39	Z68871.1	0.750390649	6.73E-98	postive
GNRH1	AC005899.7	0.543512054	1.89E-42	postive
NR2C2	AC005899.7	0.539315853	1.06E-41	postive
GNRH1	AC067838.1	0.583212151	4.52E-50	postive
DDX17	AC139795.2	0.581278101	1.13E-49	postive
NFAT5	AC139795.2	0.550371405	1.08E-43	postive
LTB4R2	AC139795.2	0.530668502	3.40E-40	postive
GNRH1	AC139795.2	0.610350357	6.46E-56	postive
NR2C2	AC139795.2	0.604519164	1.30E-54	postive
RORA	AC139795.2	0.561583907	8.61E-46	postive

TRAJ3	AC139795.2	0.504849251	6.06E-36	postive
ZC3HAV1L	AC091057.1	0.61841398	9.17E-58	postive
EED	AC091057.1	0.515425991	1.22E-37	postive
IREB2	AC091057.1	0.51440379	1.78E-37	postive
CBL	AC091057.1	0.552587257	4.21E-44	postive
PDK1	AC091057.1	0.532847767	1.43E-40	postive
CHGA	U91328.1	0.533295793	1.20E-40	postive
GRP	U91328.1	0.503330627	1.05E-35	postive
NFKBIZ	AP000442.1	0.527253036	1.30E-39	postive
DDX17	AP000442.1	0.585546859	1.49E-50	postive
NFAT5	AP000442.1	0.517757051	5.04E-38	postive
GNRH1	AP000442.1	0.631389218	7.48E-61	postive
NR2C1	AP000442.1	0.525773524	2.32E-39	postive
NR2C2	AP000442.1	0.588966195	2.90E-51	postive
DDX17	AC009118.3	0.616851499	2.11E-57	postive
GNRH1	AC009118.3	0.618288425	9.81E-58	postive
NR2C1	AC009118.3	0.505954768	4.05E-36	postive
NR2C2	AC009118.3	0.541957277	3.59E-42	postive
DDX17	AL031673.1	0.535010526	6.03E-41	postive
NFAT5	AL031673.1	0.625746931	1.72E-59	postive
GNRH1	AL031673.1	0.592005859	6.64E-52	postive
NR2C2	AL031673.1	0.604064712	1.64E-54	postive
RORA	AL031673.1	0.598170521	3.18E-53	postive
BRAF	AL031673.1	0.605165399	9.35E-55	postive
TRAJ1	AL031673.1	0.535582665	4.79E-41	postive
TRAJ2	AL031673.1	0.56528729	1.68E-46	postive
TRAJ3	AL031673.1	0.583950208	3.19E-50	postive
TRAJ5	AL031673.1	0.542427014	2.96E-42	postive
TRAJ6	AL031673.1	0.571929263	8.47E-48	postive
TRAJ8	AL031673.1	0.589789837	1.95E-51	postive
TRAJ10	AL031673.1	0.542426387	2.96E-42	postive
TRAJ12	AL031673.1	0.530314709	3.91E-40	postive
TRAJ13	AL031673.1	0.58388373	3.29E-50	postive
TRAJ14	AL031673.1	0.573540629	4.06E-48	postive
TRAJ16	AL031673.1	0.570571983	1.57E-47	postive
TRAJ17	AL031673.1	0.535573963	4.81E-41	postive
TRAJ18	AL031673.1	0.525532279	2.55E-39	postive
TRAJ21	AL031673.1	0.59341006	3.34E-52	postive
TRAJ31	AL031673.1	0.594241223	2.22E-52	postive
TRAJ37	AL031673.1	0.613170676	1.48E-56	postive
TRAJ38	AL031673.1	0.605474451	7.98E-55	postive
TRAJ39	AL031673.1	0.603933033	1.75E-54	postive
TYK2	AL031714.1	0.540555101	6.38E-42	postive
IRF9	AL031714.1	0.601628589	5.63E-54	postive
RABEP2	AL031714.1	0.505906631	4.12E-36	postive
GIPR	AL031714.1	0.508010806	1.91E-36	postive
CREB1	ACAP2-IT1	0.813169301	2.20E-127	postive
RFXAP	ACAP2-IT1	0.515723607	1.09E-37	postive
UBR1	ACAP2-IT1	0.644013788	5.30E-64	postive
ZC3HAV1	ACAP2-IT1	0.569841206	2.18E-47	postive
ZC3HAV1L	ACAP2-IT1	0.509822281	9.81E-37	postive
IL15	ACAP2-IT1	0.589925834	1.82E-51	postive
CYLD	ACAP2-IT1	0.625465905	2.00E-59	postive
EIF2AK2	ACAP2-IT1	0.560577121	1.34E-45	postive
MAPK8	ACAP2-IT1	0.577795762	5.72E-49	postive
TLR1	ACAP2-IT1	0.543489777	1.91E-42	postive
LMBR1	ACAP2-IT1	0.605629915	7.37E-55	postive
LIMS1	ACAP2-IT1	0.558851855	2.84E-45	postive
IREB2	ACAP2-IT1	0.625894954	1.58E-59	postive
DDX17	ACAP2-IT1	0.597861693	3.71E-53	postive
PIK3CG	ACAP2-IT1	0.558918454	2.76E-45	postive
JAK2	ACAP2-IT1	0.684378553	3.77E-75	postive
TXK	ACAP2-IT1	0.59504854	1.49E-52	postive
NFAT5	ACAP2-IT1	0.847295845	1.37E-148	postive
MALT1	ACAP2-IT1	0.556309391	8.55E-45	postive

PIK3R1	ACAP2-IT1	0.637068142	2.99E-62	postive
PIK3CA	ACAP2-IT1	0.75538982	6.51E-100	postive
PIK3CB	ACAP2-IT1	0.551411584	6.94E-44	postive
AKT3	ACAP2-IT1	0.557024698	6.27E-45	postive
GSK3B	ACAP2-IT1	0.505819946	4.26E-36	postive
RASGRP3	ACAP2-IT1	0.546743617	4.95E-43	postive
PLXNC1	ACAP2-IT1	0.511586606	5.10E-37	postive
GNRH1	ACAP2-IT1	0.739948375	7.67E-94	postive
IL6ST	ACAP2-IT1	0.619751794	4.47E-58	postive
RABEP1	ACAP2-IT1	0.576217188	1.19E-48	postive
ACVR2A	ACAP2-IT1	0.639572168	7.06E-63	postive
ANGPTL1	ACAP2-IT1	0.799523821	4.31E-120	postive
BMPR1A	ACAP2-IT1	0.549185266	1.78E-43	postive
BMPR2	ACAP2-IT1	0.759856681	9.38E-102	postive
CRLF3	ACAP2-IT1	0.678350667	2.26E-73	postive
NR1D2	ACAP2-IT1	0.523480678	5.65E-39	postive
NR2C2	ACAP2-IT1	0.684510731	3.44E-75	postive
RORA	ACAP2-IT1	0.842786502	1.68E-145	postive
SOS1	ACAP2-IT1	0.709739503	3.94E-83	postive
SOS2	ACAP2-IT1	0.59526107	1.35E-52	postive
BRAF	ACAP2-IT1	0.788460328	1.39E-114	postive
ITK	ACAP2-IT1	0.516635904	7.71E-38	postive
CBL	ACAP2-IT1	0.633530608	2.24E-61	postive
CBLB	ACAP2-IT1	0.768207807	2.62E-105	postive
PDK1	ACAP2-IT1	0.628842867	3.10E-60	postive
TRAJ1	ACAP2-IT1	0.723739874	6.44E-88	postive
TRAJ2	ACAP2-IT1	0.732489225	4.62E-91	postive
TRAJ3	ACAP2-IT1	0.762146223	1.03E-102	postive
TRAJ5	ACAP2-IT1	0.78157893	2.55E-111	postive
TRAJ6	ACAP2-IT1	0.787590569	3.65E-114	postive
TRAJ8	ACAP2-IT1	0.749013379	2.37E-97	postive
TRAJ10	ACAP2-IT1	0.772186953	4.71E-107	postive
TRAJ12	ACAP2-IT1	0.681369371	2.95E-74	postive
TRAJ13	ACAP2-IT1	0.778432389	7.23E-110	postive
TRAJ14	ACAP2-IT1	0.753147292	5.29E-99	postive
TRAJ16	ACAP2-IT1	0.800880027	8.61E-121	postive
TRAJ17	ACAP2-IT1	0.740250495	5.89E-94	postive
TRAJ18	ACAP2-IT1	0.716031142	3.02E-85	postive
TRAJ21	ACAP2-IT1	0.797345511	5.57E-119	postive
TRAJ31	ACAP2-IT1	0.826303105	5.43E-135	postive
TRAJ37	ACAP2-IT1	0.78608256	1.93E-113	postive
TRAJ38	ACAP2-IT1	0.865843535	1.98E-162	postive
TRAJ39	ACAP2-IT1	0.808502145	7.99E-125	postive
CREB1	AL590723.1	0.80620421	1.37E-123	postive
RFXAP	AL590723.1	0.519429507	2.67E-38	postive
UBR1	AL590723.1	0.651394882	6.50E-66	postive
ZC3HAV1	AL590723.1	0.560252912	1.54E-45	postive
ZC3HAV1L	AL590723.1	0.535322446	5.32E-41	postive
IL15	AL590723.1	0.620923886	2.38E-58	postive
CYLD	AL590723.1	0.591252775	9.58E-52	postive
EIF2AK2	AL590723.1	0.528697787	7.40E-40	postive
MAPK8	AL590723.1	0.591070673	1.05E-51	postive
TLR1	AL590723.1	0.500615629	2.79E-35	postive
LMBR1	AL590723.1	0.609535787	9.87E-56	postive
LIMS1	AL590723.1	0.530630054	3.45E-40	postive
IREB2	AL590723.1	0.633174104	2.74E-61	postive
DDX17	AL590723.1	0.570451516	1.65E-47	postive
PIK3CG	AL590723.1	0.5394526	1.00E-41	postive
JAK2	AL590723.1	0.633842503	1.88E-61	postive
TXK	AL590723.1	0.571030193	1.27E-47	postive
PPP3CB	AL590723.1	0.500459076	2.95E-35	postive
NFAT5	AL590723.1	0.842947246	1.31E-145	postive
MALT1	AL590723.1	0.551679335	6.19E-44	postive
PIK3R1	AL590723.1	0.629126473	2.65E-60	postive
PIK3CA	AL590723.1	0.715524376	4.50E-85	postive

PIK3CB	AL590723.1	0.539952616	8.16E-42	postive
AKT3	AL590723.1	0.576100516	1.25E-48	postive
RASGRP3	AL590723.1	0.519903887	2.23E-38	postive
IGHD4-4	AL590723.1	0.510157312	8.67E-37	postive
IGHD6-6	AL590723.1	0.517436557	5.70E-38	postive
GNRH1	AL590723.1	0.743219076	4.32E-95	postive
IL6ST	AL590723.1	0.619414835	5.36E-58	postive
RABEP1	AL590723.1	0.564259369	2.65E-46	postive
ACVR2A	AL590723.1	0.642874253	1.03E-63	postive
ANGPTL1	AL590723.1	0.792454925	1.56E-116	postive
BMPR1A	AL590723.1	0.57543674	1.70E-48	postive
BMPR2	AL590723.1	0.752355187	1.10E-98	postive
CRLF3	AL590723.1	0.669033027	1.05E-70	postive
NR1D2	AL590723.1	0.508022685	1.90E-36	postive
NR2C2	AL590723.1	0.68068229	4.70E-74	postive
RORA	AL590723.1	0.825370414	1.98E-134	postive
SOS1	AL590723.1	0.686957923	6.34E-76	postive
SOS2	AL590723.1	0.597102463	5.41E-53	postive
BRAF	AL590723.1	0.806123319	1.51E-123	postive
ITK	AL590723.1	0.506116035	3.82E-36	postive
CBL	AL590723.1	0.618804042	7.44E-58	postive
CBLB	AL590723.1	0.723206128	9.93E-88	postive
PDK1	AL590723.1	0.62652957	1.12E-59	postive
TRAJ1	AL590723.1	0.714502637	9.99E-85	postive
TRAJ2	AL590723.1	0.735637273	3.19E-92	postive
TRAJ3	AL590723.1	0.801501432	4.10E-121	postive
TRAJ5	AL590723.1	0.76739227	5.92E-105	postive
TRAJ6	AL590723.1	0.797236978	6.33E-119	postive
TRAJ8	AL590723.1	0.771499057	9.48E-107	postive
TRAJ10	AL590723.1	0.791241443	6.17E-116	postive
TRAJ12	AL590723.1	0.685400899	1.86E-75	postive
TRAJ13	AL590723.1	0.779217827	3.15E-110	postive
TRAJ14	AL590723.1	0.76089413	3.46E-102	postive
TRAJ16	AL590723.1	0.790602982	1.27E-115	postive
TRAJ17	AL590723.1	0.764536707	9.98E-104	postive
TRAJ18	AL590723.1	0.714338129	1.14E-84	postive
TRAJ21	AL590723.1	0.804951222	6.36E-123	postive
TRAJ31	AL590723.1	0.828035421	4.82E-136	postive
TRAJ37	AL590723.1	0.803309003	4.67E-122	postive
TRAJ38	AL590723.1	0.859577132	1.57E-157	postive
TRAJ39	AL590723.1	0.825039194	3.12E-134	postive
DDX17	ARHGAP27P1-BPTFP1-	0.531741391	2.22E-40	postive
LTB4R2	ARHGAP27P1-BPTFP1-	0.503836997	8.74E-36	postive
GNRH1	ARHGAP27P1-BPTFP1-	0.525684365	2.40E-39	postive
NFAT5	AC027117.1	0.518177958	4.30E-38	postive
GNRH1	AC027117.1	0.515800254	1.06E-37	postive
NR2C2	AC027117.1	0.560517419	1.37E-45	postive
TRAJ3	AC027117.1	0.519130869	2.99E-38	postive
TRAJ37	AC027117.1	0.516809301	7.22E-38	postive
TRAJ38	AC027117.1	0.513625366	2.39E-37	postive
TRAJ39	AC027117.1	0.501953089	1.72E-35	postive
CREB1	KANSL1L-AS1	0.817890338	4.77E-130	postive
RFXAP	KANSL1L-AS1	0.564052162	2.90E-46	postive
UBR1	KANSL1L-AS1	0.697369311	3.92E-79	postive
ZC3HAV1	KANSL1L-AS1	0.572453018	6.67E-48	postive
ZC3HAV1L	KANSL1L-AS1	0.510639629	7.25E-37	postive
IL15	KANSL1L-AS1	0.568315498	4.33E-47	postive
CYLD	KANSL1L-AS1	0.647128465	8.39E-65	postive
EIF2AK2	KANSL1L-AS1	0.546043116	6.63E-43	postive
MAPK8	KANSL1L-AS1	0.573976235	3.33E-48	postive
LMBR1	KANSL1L-AS1	0.577182654	7.60E-49	postive
IREB2	KANSL1L-AS1	0.641566111	2.22E-63	postive
DDX17	KANSL1L-AS1	0.661182759	1.56E-68	postive
PIK3CG	KANSL1L-AS1	0.544820155	1.10E-42	postive
JAK2	KANSL1L-AS1	0.653287428	2.06E-66	postive

TXK	KANSL1L-AS1	0.591909887	6.96E-52	postive
PPP3CB	KANSL1L-AS1	0.500030332	3.44E-35	postive
NFAT5	KANSL1L-AS1	0.887073074	4.55E-181	postive
NFATC3	KANSL1L-AS1	0.521510088	1.21E-38	postive
MALT1	KANSL1L-AS1	0.539109474	1.15E-41	postive
PIK3R1	KANSL1L-AS1	0.647597361	6.35E-65	postive
PIK3CA	KANSL1L-AS1	0.682802708	1.11E-74	postive
PIK3CB	KANSL1L-AS1	0.515135738	1.36E-37	postive
AKT3	KANSL1L-AS1	0.553183032	3.27E-44	postive
RASGRP3	KANSL1L-AS1	0.543465746	1.93E-42	postive
IGHD4-4	KANSL1L-AS1	0.503250587	1.08E-35	postive
IGHD6-6	KANSL1L-AS1	0.517837386	4.89E-38	postive
PLXNC1	KANSL1L-AS1	0.525421542	2.66E-39	postive
ROBO2	KANSL1L-AS1	0.502680319	1.33E-35	postive
GNRH1	KANSL1L-AS1	0.789726293	3.39E-115	postive
IL6ST	KANSL1L-AS1	0.631500229	7.03E-61	postive
RABEP1	KANSL1L-AS1	0.592975132	4.14E-52	postive
ACVR2A	KANSL1L-AS1	0.692546605	1.25E-77	postive
ANGPTL1	KANSL1L-AS1	0.745374232	6.33E-96	postive
BMPR1A	KANSL1L-AS1	0.597510585	4.42E-53	postive
BMPR2	KANSL1L-AS1	0.757976954	5.65E-101	postive
CRLF3	KANSL1L-AS1	0.675795961	1.25E-72	postive
NR1D2	KANSL1L-AS1	0.565861071	1.30E-46	postive
NR2C1	KANSL1L-AS1	0.516715732	7.48E-38	postive
NR2C2	KANSL1L-AS1	0.737975349	4.26E-93	postive
RORA	KANSL1L-AS1	0.845799966	1.49E-147	postive
SOS1	KANSL1L-AS1	0.707627913	1.96E-82	postive
SOS2	KANSL1L-AS1	0.59729999	4.91E-53	postive
BRAF	KANSL1L-AS1	0.825754682	1.16E-134	postive
CBL	KANSL1L-AS1	0.592310099	5.72E-52	postive
CBLB	KANSL1L-AS1	0.722609084	1.61E-87	postive
RASGRP1	KANSL1L-AS1	0.512179805	4.09E-37	postive
PDK1	KANSL1L-AS1	0.575514134	1.64E-48	postive
TRAJ1	KANSL1L-AS1	0.769095713	1.08E-105	postive
TRAJ2	KANSL1L-AS1	0.810626829	5.57E-126	postive
TRAJ3	KANSL1L-AS1	0.828870378	1.49E-136	postive
TRAJ5	KANSL1L-AS1	0.778419299	7.33E-110	postive
TRAJ6	KANSL1L-AS1	0.834763032	3.06E-140	postive
TRAJ8	KANSL1L-AS1	0.81030632	8.34E-126	postive
TRAJ10	KANSL1L-AS1	0.797623088	4.03E-119	postive
TRAJ12	KANSL1L-AS1	0.756939898	1.51E-100	postive
TRAJ13	KANSL1L-AS1	0.802352061	1.48E-121	postive
TRAJ14	KANSL1L-AS1	0.798392748	1.63E-119	postive
TRAJ16	KANSL1L-AS1	0.820703152	1.13E-131	postive
TRAJ17	KANSL1L-AS1	0.759407758	1.44E-101	postive
TRAJ18	KANSL1L-AS1	0.747001586	1.47E-96	postive
TRAJ21	KANSL1L-AS1	0.818274471	2.88E-130	postive
TRAJ31	KANSL1L-AS1	0.84924009	5.97E-150	postive
TRAJ37	KANSL1L-AS1	0.839595236	2.25E-143	postive
TRAJ38	KANSL1L-AS1	0.89520222	3.20E-189	postive
TRAJ39	KANSL1L-AS1	0.83854789	1.10E-142	postive
CREB1	MAST4-AS1	0.649359535	2.21E-65	postive
UBR1	MAST4-AS1	0.548345242	2.53E-43	postive
IL15	MAST4-AS1	0.543106843	2.24E-42	postive
LMBR1	MAST4-AS1	0.510930032	6.51E-37	postive
IREB2	MAST4-AS1	0.546050711	6.60E-43	postive
NFAT5	MAST4-AS1	0.733384159	2.17E-91	postive
PIK3R1	MAST4-AS1	0.552661772	4.08E-44	postive
PIK3CA	MAST4-AS1	0.552758161	3.92E-44	postive
IGHD4-4	MAST4-AS1	0.587707117	5.31E-51	postive
GNRH1	MAST4-AS1	0.606532739	4.64E-55	postive
IL6ST	MAST4-AS1	0.557420061	5.29E-45	postive
ACVR2A	MAST4-AS1	0.591525025	8.39E-52	postive
ANGPTL1	MAST4-AS1	0.616260259	2.89E-57	postive
BMPR1A	MAST4-AS1	0.519586322	2.52E-38	postive

BMPR2	MAST4-AS1	0.599501947	1.64E-53	postive
CRLF3	MAST4-AS1	0.539239534	1.09E-41	postive
NR2C2	MAST4-AS1	0.570570162	1.57E-47	postive
RORA	MAST4-AS1	0.722169945	2.29E-87	postive
SOS1	MAST4-AS1	0.548440935	2.43E-43	postive
SOS2	MAST4-AS1	0.51816744	4.32E-38	postive
BRAF	MAST4-AS1	0.692810649	1.03E-77	postive
CBLB	MAST4-AS1	0.511979986	4.41E-37	postive
PDK1	MAST4-AS1	0.502628168	1.35E-35	postive
TRAJ1	MAST4-AS1	0.632118276	4.96E-61	postive
TRAJ2	MAST4-AS1	0.664368938	2.09E-69	postive
TRAJ3	MAST4-AS1	0.724314692	4.04E-88	postive
TRAJ5	MAST4-AS1	0.68596871	1.26E-75	postive
TRAJ6	MAST4-AS1	0.705834971	7.58E-82	postive
TRAJ8	MAST4-AS1	0.715207062	5.76E-85	postive
TRAJ10	MAST4-AS1	0.7279756	2.01E-89	postive
TRAJ12	MAST4-AS1	0.627615353	6.13E-60	postive
TRAJ13	MAST4-AS1	0.69811302	2.28E-79	postive
TRAJ14	MAST4-AS1	0.684455194	3.58E-75	postive
TRAJ16	MAST4-AS1	0.68613116	1.12E-75	postive
TRAJ17	MAST4-AS1	0.703262558	5.18E-81	postive
TRAJ18	MAST4-AS1	0.636224167	4.84E-62	postive
TRAJ21	MAST4-AS1	0.71225934	5.69E-84	postive
TRAJ31	MAST4-AS1	0.756819278	1.69E-100	postive
TRAJ37	MAST4-AS1	0.763039652	4.32E-103	postive
TRAJ38	MAST4-AS1	0.751415213	2.62E-98	postive
TRAJ39	MAST4-AS1	0.752971767	6.22E-99	postive
CD4	LINC01150	0.661087539	1.66E-68	postive
FCER1G	LINC01150	0.645271875	2.52E-64	postive
HLA-DMB	LINC01150	0.574807671	2.27E-48	postive
HLA-DPA1	LINC01150	0.527130154	1.37E-39	postive
HLA-DPB1	LINC01150	0.549123832	1.82E-43	postive
HLA-DQA1	LINC01150	0.51248704	3.65E-37	postive
HLA-DRA	LINC01150	0.597266932	4.99E-53	postive
CYBB	LINC01150	0.504321342	7.34E-36	postive
MSR1	LINC01150	0.584352316	2.64E-50	postive
CD14	LINC01150	0.56348228	3.73E-46	postive
CCR1	LINC01150	0.505233364	5.27E-36	postive
IL10	LINC01150	0.533087587	1.30E-40	postive
FGR	LINC01150	0.520175494	2.01E-38	postive
CD86	LINC01150	0.629870549	1.75E-60	postive
BTK	LINC01150	0.635307457	8.17E-62	postive
LILRB3	LINC01150	0.501291171	2.19E-35	postive
GMFG	LINC01150	0.607094959	3.48E-55	postive
TNFSF12	LINC01150	0.528292031	8.68E-40	postive
C3AR1	LINC01150	0.618623783	8.20E-58	postive
CSF1R	LINC01150	0.628192162	4.45E-60	postive
ITGB2	LINC01150	0.525896802	2.21E-39	postive
TYROBP	LINC01150	0.653695649	1.61E-66	postive
FCGR3A	LINC01150	0.541037256	5.24E-42	postive
LCP2	LINC01150	0.506682165	3.11E-36	postive
BMPR2	AP001330.4	0.521069082	1.43E-38	postive
DDX17	AC092119.2	0.617170638	1.78E-57	postive
GNRH1	AC092119.2	0.644516676	3.94E-64	postive
NR2C2	AC092119.2	0.563510027	3.69E-46	postive
CREB1	AP006621.2	0.511006802	6.33E-37	postive
RFXAP	AP006621.2	0.515023144	1.41E-37	postive
DDX17	AP006621.2	0.687076306	5.84E-76	postive
NFAT5	AP006621.2	0.629746853	1.87E-60	postive
IGHD6-6	AP006621.2	0.528895551	6.84E-40	postive
LTB4R2	AP006621.2	0.618404358	9.22E-58	postive
GNRH1	AP006621.2	0.762579924	6.75E-103	postive
ANGPTL1	AP006621.2	0.517554436	5.45E-38	postive
NR2C1	AP006621.2	0.505996937	3.99E-36	postive
NR2C2	AP006621.2	0.697213807	4.38E-79	postive

RORA	AP006621.2	0.588866746	3.04E-51	postive
BRAF	AP006621.2	0.639278798	8.37E-63	postive
CBLB	AP006621.2	0.511324136	5.63E-37	postive
TRAJ1	AP006621.2	0.620956066	2.34E-58	postive
TRAJ2	AP006621.2	0.586432066	9.79E-51	postive
TRAJ3	AP006621.2	0.665380192	1.10E-69	postive
TRAJ5	AP006621.2	0.598789632	2.34E-53	postive
TRAJ6	AP006621.2	0.603873027	1.81E-54	postive
TRAJ8	AP006621.2	0.6227434	8.87E-59	postive
TRAJ10	AP006621.2	0.559186581	2.46E-45	postive
TRAJ12	AP006621.2	0.539618116	9.35E-42	postive
TRAJ13	AP006621.2	0.599040353	2.06E-53	postive
TRAJ14	AP006621.2	0.600193916	1.16E-53	postive
TRAJ16	AP006621.2	0.589898396	1.85E-51	postive
TRAJ17	AP006621.2	0.576500649	1.04E-48	postive
TRAJ18	AP006621.2	0.537300882	2.40E-41	postive
TRAJ21	AP006621.2	0.620502677	2.99E-58	postive
TRAJ31	AP006621.2	0.593962841	2.55E-52	postive
TRAJ37	AP006621.2	0.60621273	5.47E-55	postive
TRAJ38	AP006621.2	0.615975144	3.37E-57	postive
TRAJ39	AP006621.2	0.64833487	4.09E-65	postive
CREB1	IRF1-AS1	0.709407213	5.07E-83	postive
CIITA	IRF1-AS1	0.651327715	6.77E-66	postive
RFXAP	IRF1-AS1	0.52302971	6.72E-39	postive
UBR1	IRF1-AS1	0.562410822	5.99E-46	postive
ZC3HAV1	IRF1-AS1	0.538848102	1.28E-41	postive
IL15	IRF1-AS1	0.715050827	6.51E-85	postive
CYLD	IRF1-AS1	0.728459883	1.34E-89	postive
EIF2AK2	IRF1-AS1	0.530693161	3.37E-40	postive
TLR1	IRF1-AS1	0.507803503	2.06E-36	postive
DDX17	IRF1-AS1	0.680974109	3.85E-74	postive
TRIM22	IRF1-AS1	0.532300188	1.78E-40	postive
PIK3CG	IRF1-AS1	0.610706113	5.37E-56	postive
JAK2	IRF1-AS1	0.746116447	3.25E-96	postive
TXK	IRF1-AS1	0.595174296	1.40E-52	postive
NFAT5	IRF1-AS1	0.811755407	1.34E-126	postive
NFATC2	IRF1-AS1	0.502933844	1.21E-35	postive
NFATC3	IRF1-AS1	0.509707745	1.02E-36	postive
MALT1	IRF1-AS1	0.50704759	2.72E-36	postive
PIK3R1	IRF1-AS1	0.625519739	1.95E-59	postive
PIK3CA	IRF1-AS1	0.609272863	1.13E-55	postive
RASGRP3	IRF1-AS1	0.664661087	1.74E-69	postive
IGHD6-6	IRF1-AS1	0.566767251	8.67E-47	postive
LTB4R2	IRF1-AS1	0.511207598	5.88E-37	postive
PLXNC1	IRF1-AS1	0.63569829	6.54E-62	postive
GNRH1	IRF1-AS1	0.745577819	5.28E-96	postive
IL6ST	IRF1-AS1	0.562163426	6.68E-46	postive
IL7	IRF1-AS1	0.567529397	6.17E-47	postive
RABEP1	IRF1-AS1	0.530263108	3.99E-40	postive
TNFSF8	IRF1-AS1	0.501521436	2.01E-35	postive
ACVR2A	IRF1-AS1	0.579294085	2.85E-49	postive
ANGPTL1	IRF1-AS1	0.687898883	3.29E-76	postive
BMPR2	IRF1-AS1	0.61789654	1.21E-57	postive
CRLF3	IRF1-AS1	0.634142501	1.58E-61	postive
IL18RAP	IRF1-AS1	0.529328939	5.77E-40	postive
NR2C2	IRF1-AS1	0.717217017	1.19E-85	postive
RORA	IRF1-AS1	0.791778825	3.36E-116	postive
SOS1	IRF1-AS1	0.637577282	2.23E-62	postive
SOS2	IRF1-AS1	0.551313074	7.24E-44	postive
BRAF	IRF1-AS1	0.708823238	7.92E-83	postive
PTPRC	IRF1-AS1	0.605010984	1.01E-54	postive
ITK	IRF1-AS1	0.652859806	2.67E-66	postive
CD28	IRF1-AS1	0.619168592	6.12E-58	postive
ICOS	IRF1-AS1	0.556773442	6.99E-45	postive
CBL	IRF1-AS1	0.605262248	8.90E-55	postive

CBLB	IRF1-AS1	0.65920439	5.39E-68	postive
RASGRP1	IRF1-AS1	0.577449871	6.71E-49	postive
PDK1	IRF1-AS1	0.562280242	6.34E-46	postive
TRAJ1	IRF1-AS1	0.840700796	4.18E-144	postive
TRAJ2	IRF1-AS1	0.766804618	1.06E-104	postive
TRAJ3	IRF1-AS1	0.852703889	2.00E-152	postive
TRAJ5	IRF1-AS1	0.833073467	3.61E-139	postive
TRAJ6	IRF1-AS1	0.831362445	4.27E-138	postive
TRAJ8	IRF1-AS1	0.775914258	1.01E-108	postive
TRAJ10	IRF1-AS1	0.811771942	1.31E-126	postive
TRAJ12	IRF1-AS1	0.799194766	6.36E-120	postive
TRAJ13	IRF1-AS1	0.788039687	2.22E-114	postive
TRAJ14	IRF1-AS1	0.806570558	8.74E-124	postive
TRAJ16	IRF1-AS1	0.820174167	2.30E-131	postive
TRAJ17	IRF1-AS1	0.797576082	4.26E-119	postive
TRAJ18	IRF1-AS1	0.825285341	2.22E-134	postive
TRAJ21	IRF1-AS1	0.823741422	1.85E-133	postive
TRAJ31	IRF1-AS1	0.825666838	1.31E-134	postive
TRAJ37	IRF1-AS1	0.818406923	2.41E-130	postive
TRAJ38	IRF1-AS1	0.85551752	1.75E-154	postive
TRAJ39	IRF1-AS1	0.840858938	3.28E-144	postive
PGF	LINC01088	0.602576254	3.49E-54	postive
CREB1	RBMS3-AS3	0.635252491	8.42E-62	postive
UBR1	RBMS3-AS3	0.572220604	7.41E-48	postive
IREB2	RBMS3-AS3	0.523178022	6.35E-39	postive
DDX17	RBMS3-AS3	0.514139513	1.97E-37	postive
TXK	RBMS3-AS3	0.564271991	2.63E-46	postive
NFAT5	RBMS3-AS3	0.70706394	3.00E-82	postive
PIK3R1	RBMS3-AS3	0.641490495	2.32E-63	postive
PIK3CA	RBMS3-AS3	0.519154282	2.97E-38	postive
IGHD4-4	RBMS3-AS3	0.549288776	1.70E-43	postive
IGHD6-6	RBMS3-AS3	0.508648536	1.51E-36	postive
GNRH1	RBMS3-AS3	0.672066028	1.46E-71	postive
IL6ST	RBMS3-AS3	0.614952032	5.79E-57	postive
ACVR2A	RBMS3-AS3	0.566396013	1.02E-46	postive
ANGPTL1	RBMS3-AS3	0.590343742	1.49E-51	postive
BMPR1A	RBMS3-AS3	0.542040619	3.47E-42	postive
BMPR2	RBMS3-AS3	0.661457484	1.32E-68	postive
NR1D2	RBMS3-AS3	0.502127309	1.62E-35	postive
NR2C2	RBMS3-AS3	0.594922629	1.59E-52	postive
NR3C2	RBMS3-AS3	0.502100044	1.64E-35	postive
RORA	RBMS3-AS3	0.749587698	1.40E-97	postive
SOS1	RBMS3-AS3	0.510563323	7.46E-37	postive
BRAF	RBMS3-AS3	0.677655784	3.61E-73	postive
CBLB	RBMS3-AS3	0.553166676	3.29E-44	postive
TRAJ1	RBMS3-AS3	0.510715123	7.05E-37	postive
TRAJ2	RBMS3-AS3	0.533036887	1.33E-40	postive
TRAJ3	RBMS3-AS3	0.592487622	5.25E-52	postive
TRAJ5	RBMS3-AS3	0.596627895	6.85E-53	postive
TRAJ6	RBMS3-AS3	0.574476656	2.64E-48	postive
TRAJ8	RBMS3-AS3	0.557461001	5.20E-45	postive
TRAJ10	RBMS3-AS3	0.558977486	2.69E-45	postive
TRAJ12	RBMS3-AS3	0.537489977	2.22E-41	postive
TRAJ13	RBMS3-AS3	0.567968369	5.07E-47	postive
TRAJ14	RBMS3-AS3	0.536313421	3.57E-41	postive
TRAJ16	RBMS3-AS3	0.54280656	2.53E-42	postive
TRAJ17	RBMS3-AS3	0.536857843	2.87E-41	postive
TRAJ18	RBMS3-AS3	0.541055143	5.20E-42	postive
TRAJ21	RBMS3-AS3	0.603842191	1.83E-54	postive
TRAJ31	RBMS3-AS3	0.637847668	1.91E-62	postive
TRAJ37	RBMS3-AS3	0.656006542	3.90E-67	postive
TRAJ38	RBMS3-AS3	0.632163331	4.84E-61	postive
TRAJ39	RBMS3-AS3	0.649429782	2.12E-65	postive
CREB1	AC107027.3	0.640540843	4.03E-63	postive
UBR1	AC107027.3	0.563766748	3.29E-46	postive

ZC3HAV1	AC107027.3	0.560611531	1.32E-45	postive
CYLD	AC107027.3	0.519081523	3.05E-38	postive
EIF2AK2	AC107027.3	0.577666628	6.07E-49	postive
SP1	AC107027.3	0.511134061	6.04E-37	postive
IREB2	AC107027.3	0.568043863	4.90E-47	postive
NFAT5	AC107027.3	0.608731231	1.50E-55	postive
PIK3R1	AC107027.3	0.551892385	5.66E-44	postive
PIK3CA	AC107027.3	0.649080409	2.62E-65	postive
PIK3CB	AC107027.3	0.651728178	5.31E-66	postive
GNRH1	AC107027.3	0.502586196	1.37E-35	postive
IL6ST	AC107027.3	0.546436455	5.62E-43	postive
ACVR2A	AC107027.3	0.558089077	3.96E-45	postive
ANGPTL1	AC107027.3	0.603740308	1.93E-54	postive
BMPR2	AC107027.3	0.593861036	2.68E-52	postive
NR2C2	AC107027.3	0.660598	2.26E-68	postive
RORA	AC107027.3	0.637389436	2.48E-62	postive
SOS1	AC107027.3	0.633098762	2.86E-61	postive
SOS2	AC107027.3	0.503205566	1.10E-35	postive
BRAF	AC107027.3	0.678989357	1.47E-73	postive
CBLB	AC107027.3	0.565384731	1.61E-46	postive
RASGRP1	AC107027.3	0.526614377	1.67E-39	postive
TRAJ1	AC107027.3	0.649316448	2.27E-65	postive
TRAJ2	AC107027.3	0.501997439	1.70E-35	postive
TRAJ3	AC107027.3	0.611887457	2.90E-56	postive
TRAJ5	AC107027.3	0.738227107	3.43E-93	postive
TRAJ6	AC107027.3	0.638563567	1.26E-62	postive
TRAJ8	AC107027.3	0.664395893	2.05E-69	postive
TRAJ10	AC107027.3	0.618764992	7.60E-58	postive
TRAJ12	AC107027.3	0.663933809	2.76E-69	postive
TRAJ13	AC107027.3	0.66818104	1.82E-70	postive
TRAJ14	AC107027.3	0.671193531	2.58E-71	postive
TRAJ16	AC107027.3	0.665317953	1.14E-69	postive
TRAJ17	AC107027.3	0.687727495	3.71E-76	postive
TRAJ18	AC107027.3	0.622990592	7.76E-59	postive
TRAJ21	AC107027.3	0.690680327	4.68E-77	postive
TRAJ31	AC107027.3	0.711976478	7.08E-84	postive
TRAJ37	AC107027.3	0.639367862	7.95E-63	postive
TRAJ38	AC107027.3	0.6850788	2.33E-75	postive
TRAJ39	AC107027.3	0.706326358	5.24E-82	postive
SLC11A1	AC026369.3	0.505539443	4.71E-36	postive
MARCO	AC026369.3	0.616592765	2.42E-57	postive
AGER	AC026369.3	0.532817695	1.45E-40	postive
CCL23	AC026369.3	0.616174523	3.03E-57	postive
FGR	AC026369.3	0.540343571	6.96E-42	postive
BTK	AC026369.3	0.541270102	4.76E-42	postive
AGRP	AC026369.3	0.658540599	8.14E-68	postive
GMFG	AC026369.3	0.522039609	9.84E-39	postive
RETN	AC026369.3	0.563675117	3.43E-46	postive
TYROBP	AC026369.3	0.546623327	5.20E-43	postive
CYLD	AC060766.7	0.530909132	3.09E-40	postive
RORA	AC060766.7	0.525574112	2.51E-39	postive
KRAS	RASSF8-AS1	0.523568431	5.46E-39	postive
BPIFB1	LINC02166	0.682813424	1.10E-74	postive
DES	LINC02166	0.693115404	8.33E-78	postive
GIPR	LINC02166	0.54694427	4.55E-43	postive
CREB1	AP002336.2	0.764997611	6.34E-104	postive
RFXAP	AP002336.2	0.501715502	1.88E-35	postive
UBR1	AP002336.2	0.611697657	3.20E-56	postive
ZC3HAV1	AP002336.2	0.556560748	7.67E-45	postive
IL15	AP002336.2	0.57845983	4.20E-49	postive
CYLD	AP002336.2	0.589004369	2.85E-51	postive
EIF2AK2	AP002336.2	0.521073945	1.43E-38	postive
MAPK8	AP002336.2	0.555610575	1.15E-44	postive
LMBR1	AP002336.2	0.589436144	2.31E-51	postive
IREB2	AP002336.2	0.586886233	7.88E-51	postive

DDX17	AP002336.2	0.572924057	5.38E-48	postive
PIK3CG	AP002336.2	0.50910333	1.28E-36	postive
JAK2	AP002336.2	0.627001684	8.60E-60	postive
TXK	AP002336.2	0.554024647	2.28E-44	postive
NFAT5	AP002336.2	0.841875781	6.88E-145	postive
MALT1	AP002336.2	0.534792411	6.58E-41	postive
PIK3R1	AP002336.2	0.608830271	1.42E-55	postive
PIK3CA	AP002336.2	0.689234518	1.29E-76	postive
PIK3CB	AP002336.2	0.503055847	1.16E-35	postive
AKT3	AP002336.2	0.541381399	4.55E-42	postive
RASGRP3	AP002336.2	0.509907188	9.51E-37	postive
GNRH1	AP002336.2	0.718338197	4.90E-86	postive
IL6ST	AP002336.2	0.600912872	8.07E-54	postive
RABEP1	AP002336.2	0.555795646	1.07E-44	postive
ACVR2A	AP002336.2	0.608347883	1.82E-55	postive
ANGPTL1	AP002336.2	0.794353093	1.78E-117	postive
BMPR1A	AP002336.2	0.51772577	5.10E-38	postive
BMPR2	AP002336.2	0.708316606	1.16E-82	postive
CRLF3	AP002336.2	0.640724998	3.62E-63	postive
NR2C2	AP002336.2	0.657607219	1.45E-67	postive
RORA	AP002336.2	0.827614648	8.71E-136	postive
SOS1	AP002336.2	0.670511345	4.03E-71	postive
SOS2	AP002336.2	0.57387663	3.48E-48	postive
BRAF	AP002336.2	0.801895943	2.56E-121	postive
CBL	AP002336.2	0.61278107	1.82E-56	postive
CBLB	AP002336.2	0.742289264	9.83E-95	postive
PDK1	AP002336.2	0.5874275	6.08E-51	postive
TRAJ1	AP002336.2	0.725780323	1.22E-88	postive
TRAJ2	AP002336.2	0.722902215	1.27E-87	postive
TRAJ3	AP002336.2	0.782573922	8.75E-112	postive
TRAJ5	AP002336.2	0.8050005	5.99E-123	postive
TRAJ6	AP002336.2	0.802269214	1.63E-121	postive
TRAJ8	AP002336.2	0.7682431	2.53E-105	postive
TRAJ10	AP002336.2	0.794137378	2.29E-117	postive
TRAJ12	AP002336.2	0.706228178	5.64E-82	postive
TRAJ13	AP002336.2	0.806052171	1.65E-123	postive
TRAJ14	AP002336.2	0.784824099	7.63E-113	postive
TRAJ16	AP002336.2	0.802471396	1.28E-121	postive
TRAJ17	AP002336.2	0.767107679	7.86E-105	postive
TRAJ18	AP002336.2	0.736917804	1.06E-92	postive
TRAJ21	AP002336.2	0.829388234	7.15E-137	postive
TRAJ31	AP002336.2	0.847282476	1.40E-148	postive
TRAJ37	AP002336.2	0.815986801	5.78E-129	postive
TRAJ38	AP002336.2	0.872173379	1.24E-167	postive
TRAJ39	AP002336.2	0.835334066	1.32E-140	postive
IRF3	AL021707.6	0.51832655	4.06E-38	postive
TYK2	AL021707.6	0.52528195	2.81E-39	postive
DDX17	AL021707.6	0.508973429	1.34E-36	postive
IRF9	AL021707.6	0.559890912	1.81E-45	postive
CREB1	AC022400.6	0.679344725	1.16E-73	postive
RFXAP	AC022400.6	0.52879045	7.13E-40	postive
UBR1	AC022400.6	0.614891178	5.98E-57	postive
ZC3HAV1	AC022400.6	0.547664789	3.37E-43	postive
CYLD	AC022400.6	0.529432462	5.54E-40	postive
EIF2AK2	AC022400.6	0.515318451	1.27E-37	postive
MAPK8	AC022400.6	0.603784213	1.89E-54	postive
IREB2	AC022400.6	0.541205134	4.89E-42	postive
DDX17	AC022400.6	0.716384394	2.29E-85	postive
JAK2	AC022400.6	0.504370047	7.21E-36	postive
TXK	AC022400.6	0.543638816	1.80E-42	postive
PPP3CB	AC022400.6	0.514916818	1.47E-37	postive
NFAT5	AC022400.6	0.744250898	1.73E-95	postive
PIK3R1	AC022400.6	0.548494711	2.38E-43	postive
PIK3CA	AC022400.6	0.511592949	5.09E-37	postive
LTB4R2	AC022400.6	0.520703671	1.64E-38	postive

GNRH1	AC022400.6	0.761633786	1.69E-102	postive
IL6ST	AC022400.6	0.586649648	8.82E-51	postive
RABEP1	AC022400.6	0.539179606	1.12E-41	postive
ACVR2A	AC022400.6	0.56705993	7.61E-47	postive
ANGPTL1	AC022400.6	0.619567882	4.94E-58	postive
BMPR1A	AC022400.6	0.533514365	1.10E-40	postive
BMPR2	AC022400.6	0.598233984	3.08E-53	postive
CRLF3	AC022400.6	0.550913011	8.57E-44	postive
NR2C1	AC022400.6	0.603281612	2.44E-54	postive
NR2C2	AC022400.6	0.785797236	2.63E-113	postive
RORA	AC022400.6	0.727283262	3.55E-89	postive
SOS1	AC022400.6	0.631247224	8.10E-61	postive
BRAF	AC022400.6	0.800040095	2.34E-120	postive
CBL	AC022400.6	0.505889942	4.15E-36	postive
CBLB	AC022400.6	0.618819997	7.38E-58	postive
TRAJ1	AC022400.6	0.595892922	9.85E-53	postive
TRAJ2	AC022400.6	0.618764032	7.61E-58	postive
TRAJ3	AC022400.6	0.647176056	8.16E-65	postive
TRAJ5	AC022400.6	0.627352194	7.09E-60	postive
TRAJ6	AC022400.6	0.622965022	7.87E-59	postive
TRAJ8	AC022400.6	0.596871522	6.07E-53	postive
TRAJ10	AC022400.6	0.590359366	1.48E-51	postive
TRAJ12	AC022400.6	0.560927879	1.15E-45	postive
TRAJ13	AC022400.6	0.61801276	1.14E-57	postive
TRAJ14	AC022400.6	0.598173563	3.18E-53	postive
TRAJ16	AC022400.6	0.590408977	1.44E-51	postive
TRAJ17	AC022400.6	0.554847005	1.60E-44	postive
TRAJ18	AC022400.6	0.566934906	8.05E-47	postive
TRAJ21	AC022400.6	0.634471047	1.31E-61	postive
TRAJ31	AC022400.6	0.64417117	4.83E-64	postive
TRAJ37	AC022400.6	0.647789964	5.66E-65	postive
TRAJ38	AC022400.6	0.673587858	5.37E-72	postive
TRAJ39	AC022400.6	0.647544772	6.55E-65	postive
TINAGL1	AC114488.1	0.598951668	2.16E-53	postive
ACKR4	AC114488.1	0.571648058	9.62E-48	postive
MIA	AC114488.1	0.516277379	8.83E-38	postive
IL1R2	AC114488.1	0.512759193	3.30E-37	postive
CREB1	TRAF3IP2-AS1	0.793366728	5.52E-117	postive
RFXAP	TRAF3IP2-AS1	0.632589339	3.81E-61	postive
UBR1	TRAF3IP2-AS1	0.626765609	9.80E-60	postive
ZC3HAV1	TRAF3IP2-AS1	0.508303326	1.72E-36	postive
IL15	TRAF3IP2-AS1	0.532549656	1.61E-40	postive
CYLD	TRAF3IP2-AS1	0.629422552	2.25E-60	postive
MAPK8	TRAF3IP2-AS1	0.59031446	1.51E-51	postive
LMBR1	TRAF3IP2-AS1	0.575462387	1.68E-48	postive
LIMS1	TRAF3IP2-AS1	0.533809303	9.76E-41	postive
IREB2	TRAF3IP2-AS1	0.611733423	3.14E-56	postive
DDX17	TRAF3IP2-AS1	0.637077388	2.97E-62	postive
PIK3CG	TRAF3IP2-AS1	0.555223604	1.36E-44	postive
JAK2	TRAF3IP2-AS1	0.619744339	4.49E-58	postive
TXK	TRAF3IP2-AS1	0.594515568	1.94E-52	postive
PPP3CB	TRAF3IP2-AS1	0.512732448	3.33E-37	postive
NFAT5	TRAF3IP2-AS1	0.877599031	2.54E-172	postive
NFATC3	TRAF3IP2-AS1	0.550911495	8.58E-44	postive
MALT1	TRAF3IP2-AS1	0.548239077	2.65E-43	postive
PIK3R1	TRAF3IP2-AS1	0.640702857	3.67E-63	postive
PIK3CA	TRAF3IP2-AS1	0.673527296	5.59E-72	postive
AKT3	TRAF3IP2-AS1	0.619425935	5.33E-58	postive
RASGRP3	TRAF3IP2-AS1	0.5067366	3.05E-36	postive
IGHD6-6	TRAF3IP2-AS1	0.534576639	7.18E-41	postive
PLXNC1	TRAF3IP2-AS1	0.500098248	3.35E-35	postive
GNRH1	TRAF3IP2-AS1	0.749113317	2.16E-97	postive
IL6ST	TRAF3IP2-AS1	0.622732474	8.93E-59	postive
RABEP1	TRAF3IP2-AS1	0.614574212	7.07E-57	postive
ACVR2A	TRAF3IP2-AS1	0.677898756	3.06E-73	postive

ANGPTL1	TRAF3IP2-AS1	0.738657485	2.36E-93	postive
BMPR1A	TRAF3IP2-AS1	0.582568366	6.13E-50	postive
BMPR2	TRAF3IP2-AS1	0.713840404	1.67E-84	postive
CRLF3	TRAF3IP2-AS1	0.703104426	5.82E-81	postive
NR1D2	TRAF3IP2-AS1	0.52348704	5.63E-39	postive
NR2C1	TRAF3IP2-AS1	0.540814117	5.74E-42	postive
NR2C2	TRAF3IP2-AS1	0.679863518	8.17E-74	postive
RORA	TRAF3IP2-AS1	0.853788067	3.26E-153	postive
SOS1	TRAF3IP2-AS1	0.67114904	2.66E-71	postive
SOS2	TRAF3IP2-AS1	0.57912099	3.09E-49	postive
BRAF	TRAF3IP2-AS1	0.78062883	7.04E-111	postive
ITK	TRAF3IP2-AS1	0.513348011	2.65E-37	postive
CD28	TRAF3IP2-AS1	0.529861328	4.68E-40	postive
CBL	TRAF3IP2-AS1	0.610580878	5.73E-56	postive
CBLB	TRAF3IP2-AS1	0.667446941	2.92E-70	postive
PDK1	TRAF3IP2-AS1	0.619007301	6.68E-58	postive
TRAJ1	TRAF3IP2-AS1	0.742452357	8.51E-95	postive
TRAJ2	TRAF3IP2-AS1	0.794250325	2.01E-117	postive
TRAJ3	TRAF3IP2-AS1	0.800419817	1.49E-120	postive
TRAJ5	TRAF3IP2-AS1	0.762476813	7.47E-103	postive
TRAJ6	TRAF3IP2-AS1	0.808583091	7.22E-125	postive
TRAJ8	TRAF3IP2-AS1	0.789960496	2.61E-115	postive
TRAJ10	TRAF3IP2-AS1	0.773648862	1.05E-107	postive
TRAJ12	TRAF3IP2-AS1	0.689327002	1.21E-76	postive
TRAJ13	TRAF3IP2-AS1	0.796272633	1.94E-118	postive
TRAJ14	TRAF3IP2-AS1	0.767070183	8.16E-105	postive
TRAJ16	TRAF3IP2-AS1	0.802683629	9.93E-122	postive
TRAJ17	TRAF3IP2-AS1	0.735147734	4.84E-92	postive
TRAJ18	TRAF3IP2-AS1	0.718565059	4.09E-86	postive
TRAJ21	TRAF3IP2-AS1	0.805191361	4.74E-123	postive
TRAJ31	TRAF3IP2-AS1	0.812918237	3.03E-127	postive
TRAJ37	TRAF3IP2-AS1	0.83080264	9.54E-138	postive
TRAJ38	TRAF3IP2-AS1	0.857330358	7.82E-156	postive
TRAJ39	TRAF3IP2-AS1	0.828351914	3.09E-136	postive
NR2C2	AL122010.1	0.505313705	5.12E-36	postive
CREB1	TBILA	0.526494961	1.75E-39	postive
UBR1	TBILA	0.515247536	1.30E-37	postive
TCF7L2	TBILA	0.51162439	5.03E-37	postive
DDX17	TBILA	0.549964564	1.28E-43	postive
TXK	TBILA	0.515363128	1.25E-37	postive
NFAT5	TBILA	0.510158461	8.66E-37	postive
ROBO2	TBILA	0.666153082	6.70E-70	postive
GNRH1	TBILA	0.696697677	6.37E-79	postive
BMPR2	TBILA	0.573812538	3.59E-48	postive
NR2C2	TBILA	0.593589326	3.06E-52	postive
RORA	TBILA	0.532988782	1.35E-40	postive
BRAF	TBILA	0.536460159	3.36E-41	postive
CBLB	TBILA	0.549654369	1.46E-43	postive
CREB1	ARHGAP31-AS1	0.514438158	1.76E-37	postive
NFAT5	ARHGAP31-AS1	0.610234876	6.86E-56	postive
PIK3CA	ARHGAP31-AS1	0.555535766	1.19E-44	postive
PIK3CB	ARHGAP31-AS1	0.504989079	5.76E-36	postive
NR2C2	ARHGAP31-AS1	0.53440381	7.69E-41	postive
RORA	ARHGAP31-AS1	0.566221167	1.11E-46	postive
SOS1	ARHGAP31-AS1	0.541340578	4.63E-42	postive
BRAF	ARHGAP31-AS1	0.566208333	1.11E-46	postive
CBLB	ARHGAP31-AS1	0.504399548	7.13E-36	postive
TRAJ1	ARHGAP31-AS1	0.549614622	1.48E-43	postive
TRAJ3	ARHGAP31-AS1	0.591871013	7.09E-52	postive
TRAJ5	ARHGAP31-AS1	0.574701488	2.39E-48	postive
TRAJ6	ARHGAP31-AS1	0.591816265	7.28E-52	postive
TRAJ8	ARHGAP31-AS1	0.559367512	2.27E-45	postive
TRAJ10	ARHGAP31-AS1	0.581860998	8.56E-50	postive
TRAJ12	ARHGAP31-AS1	0.570974921	1.31E-47	postive
TRAJ13	ARHGAP31-AS1	0.565910805	1.27E-46	postive

TRAJ14	ARHGAP31-AS1	0.571899011	8.58E-48	postive
TRAJ16	ARHGAP31-AS1	0.593583863	3.07E-52	postive
TRAJ17	ARHGAP31-AS1	0.579542006	2.54E-49	postive
TRAJ18	ARHGAP31-AS1	0.585981664	1.21E-50	postive
TRAJ21	ARHGAP31-AS1	0.604915903	1.06E-54	postive
TRAJ31	ARHGAP31-AS1	0.593707839	2.89E-52	postive
TRAJ37	ARHGAP31-AS1	0.595187255	1.40E-52	postive
TRAJ38	ARHGAP31-AS1	0.604844222	1.10E-54	postive
TRAJ39	ARHGAP31-AS1	0.617399317	1.58E-57	postive
CREB1	AC011815.1	0.60004997	1.24E-53	postive
UBR1	AC011815.1	0.54376543	1.70E-42	postive
ZC3HAV1L	AC011815.1	0.531842869	2.14E-40	postive
IREB2	AC011815.1	0.552282532	4.79E-44	postive
NFAT5	AC011815.1	0.584616534	2.33E-50	postive
AKT3	AC011815.1	0.517273032	6.06E-38	postive
GNRH1	AC011815.1	0.581505239	1.01E-49	postive
ANGPTL1	AC011815.1	0.536516216	3.29E-41	postive
BMPR2	AC011815.1	0.511148366	6.01E-37	postive
CRLF3	AC011815.1	0.544110839	1.48E-42	postive
NR2C2	AC011815.1	0.559057155	2.60E-45	postive
RORA	AC011815.1	0.522654948	7.77E-39	postive
SOS1	AC011815.1	0.574000168	3.29E-48	postive
BRAF	AC011815.1	0.663796946	3.01E-69	postive
PDK1	AC011815.1	0.503465716	1.00E-35	postive
TRAJ1	AC011815.1	0.541828131	3.79E-42	postive
TRAJ2	AC011815.1	0.565454901	1.56E-46	postive
TRAJ3	AC011815.1	0.605751508	6.93E-55	postive
TRAJ5	AC011815.1	0.554543128	1.83E-44	postive
TRAJ6	AC011815.1	0.581024	1.27E-49	postive
TRAJ8	AC011815.1	0.615571828	4.17E-57	postive
TRAJ10	AC011815.1	0.535850767	4.30E-41	postive
TRAJ13	AC011815.1	0.590350901	1.48E-51	postive
TRAJ14	AC011815.1	0.576880023	8.74E-49	postive
TRAJ16	AC011815.1	0.539932035	8.23E-42	postive
TRAJ17	AC011815.1	0.532437487	1.69E-40	postive
TRAJ21	AC011815.1	0.589843942	1.90E-51	postive
TRAJ31	AC011815.1	0.564665246	2.21E-46	postive
TRAJ37	AC011815.1	0.566763545	8.69E-47	postive
TRAJ38	AC011815.1	0.597768771	3.89E-53	postive
TRAJ39	AC011815.1	0.61049847	5.98E-56	postive
CREB1	AC004466.3	0.713658649	1.93E-84	postive
UBR1	AC004466.3	0.565864077	1.30E-46	postive
IL15	AC004466.3	0.536618161	3.16E-41	postive
CYLD	AC004466.3	0.617309609	1.66E-57	postive
NFKBIZ	AC004466.3	0.532916318	1.39E-40	postive
DDX17	AC004466.3	0.673441927	5.91E-72	postive
JAK2	AC004466.3	0.617254186	1.70E-57	postive
TXK	AC004466.3	0.563616175	3.52E-46	postive
NFAT5	AC004466.3	0.843180356	9.12E-146	postive
MALT1	AC004466.3	0.511139739	6.03E-37	postive
PIK3R1	AC004466.3	0.579848619	2.20E-49	postive
PIK3CA	AC004466.3	0.616525136	2.51E-57	postive
GNRH1	AC004466.3	0.758610536	3.09E-101	postive
IL6ST	AC004466.3	0.596543935	7.14E-53	postive
RABEP1	AC004466.3	0.507638597	2.19E-36	postive
ACVR2A	AC004466.3	0.563543409	3.63E-46	postive
ANGPTL1	AC004466.3	0.704031225	2.92E-81	postive
BMPR2	AC004466.3	0.636610479	3.88E-62	postive
CRLF3	AC004466.3	0.585747307	1.36E-50	postive
NR2C1	AC004466.3	0.516978009	6.77E-38	postive
NR2C2	AC004466.3	0.701880906	1.44E-80	postive
RORA	AC004466.3	0.821459914	4.10E-132	postive
SOS1	AC004466.3	0.601374805	6.39E-54	postive
SOS2	AC004466.3	0.54052425	6.46E-42	postive
BRAF	AC004466.3	0.710484475	2.23E-83	postive

ITK	AC004466.3	0.512022649	4.34E-37	postive
CBL	AC004466.3	0.581891756	8.43E-50	postive
CBLB	AC004466.3	0.686635851	7.93E-76	postive
PDK1	AC004466.3	0.504292083	7.41E-36	postive
TRAJ1	AC004466.3	0.693506716	6.30E-78	postive
TRAJ2	AC004466.3	0.675139662	1.93E-72	postive
TRAJ3	AC004466.3	0.718698409	3.68E-86	postive
TRAJ5	AC004466.3	0.753563812	3.59E-99	postive
TRAJ6	AC004466.3	0.740496813	4.75E-94	postive
TRAJ8	AC004466.3	0.667698482	2.49E-70	postive
TRAJ10	AC004466.3	0.758373306	3.87E-101	postive
TRAJ12	AC004466.3	0.653766012	1.54E-66	postive
TRAJ13	AC004466.3	0.737226482	8.13E-93	postive
TRAJ14	AC004466.3	0.696279199	8.61E-79	postive
TRAJ16	AC004466.3	0.736053066	2.23E-92	postive
TRAJ17	AC004466.3	0.690101576	7.04E-77	postive
TRAJ18	AC004466.3	0.72619805	8.67E-89	postive
TRAJ21	AC004466.3	0.759898306	9.01E-102	postive
TRAJ31	AC004466.3	0.781186604	3.88E-111	postive
TRAJ37	AC004466.3	0.777239344	2.53E-109	postive
TRAJ38	AC004466.3	0.792978813	8.60E-117	postive
TRAJ39	AC004466.3	0.759092487	1.95E-101	postive
CREB1	FMR1-IT1	0.667062118	3.74E-70	postive
RFXAP	FMR1-IT1	0.501441094	2.07E-35	postive
UBR1	FMR1-IT1	0.519362227	2.74E-38	postive
ZC3HAV1	FMR1-IT1	0.504195814	7.68E-36	postive
IL15	FMR1-IT1	0.507445628	2.35E-36	postive
CYLD	FMR1-IT1	0.576031248	1.29E-48	postive
NFKBIZ	FMR1-IT1	0.522611638	7.90E-39	postive
DDX17	FMR1-IT1	0.650622123	1.04E-65	postive
JAK2	FMR1-IT1	0.559814661	1.87E-45	postive
NFAT5	FMR1-IT1	0.772240932	4.45E-107	postive
PIK3R1	FMR1-IT1	0.532929546	1.39E-40	postive
PIK3CA	FMR1-IT1	0.572155347	7.64E-48	postive
LTB4R2	FMR1-IT1	0.549940406	1.29E-43	postive
GNRH1	FMR1-IT1	0.715556102	4.39E-85	postive
IL6ST	FMR1-IT1	0.514900592	1.48E-37	postive
ACVR2A	FMR1-IT1	0.573011973	5.17E-48	postive
ANGPTL1	FMR1-IT1	0.662660188	6.17E-69	postive
BMPR2	FMR1-IT1	0.554162573	2.15E-44	postive
CRLF3	FMR1-IT1	0.588040093	4.53E-51	postive
NR2C1	FMR1-IT1	0.544416478	1.30E-42	postive
NR2C2	FMR1-IT1	0.732458603	4.75E-91	postive
RORA	FMR1-IT1	0.716652525	1.85E-85	postive
SOS1	FMR1-IT1	0.651618835	5.67E-66	postive
SOS2	FMR1-IT1	0.574198203	3.00E-48	postive
BRAF	FMR1-IT1	0.743669123	2.90E-95	postive
CBL	FMR1-IT1	0.570296221	1.78E-47	postive
CBLB	FMR1-IT1	0.681384567	2.92E-74	postive
TRAJ1	FMR1-IT1	0.77622297	7.32E-109	postive
TRAJ2	FMR1-IT1	0.735462603	3.70E-92	postive
TRAJ3	FMR1-IT1	0.77137404	1.08E-106	postive
TRAJ5	FMR1-IT1	0.793913723	2.95E-117	postive
TRAJ6	FMR1-IT1	0.783489599	3.25E-112	postive
TRAJ8	FMR1-IT1	0.786999048	7.03E-114	postive
TRAJ10	FMR1-IT1	0.757531246	8.63E-101	postive
TRAJ12	FMR1-IT1	0.733776659	1.56E-91	postive
TRAJ13	FMR1-IT1	0.801820393	2.80E-121	postive
TRAJ14	FMR1-IT1	0.799357829	5.24E-120	postive
TRAJ16	FMR1-IT1	0.786443255	1.30E-113	postive
TRAJ17	FMR1-IT1	0.750260902	7.58E-98	postive
TRAJ18	FMR1-IT1	0.718745899	3.55E-86	postive
TRAJ21	FMR1-IT1	0.803001351	6.77E-122	postive
TRAJ31	FMR1-IT1	0.796840134	1.00E-118	postive
TRAJ37	FMR1-IT1	0.779959673	1.44E-110	postive

TRAJ38	FMR1-IT1	0.826831236	2.60E-135	postive
TRAJ39	FMR1-IT1	0.791994089	2.63E-116	postive
CREB1	AL021578.1	0.735005365	5.47E-92	postive
RFXAP	AL021578.1	0.5047333	6.32E-36	postive
UBR1	AL021578.1	0.655171617	6.51E-67	postive
ZC3HAV1	AL021578.1	0.500089753	3.36E-35	postive
IL15	AL021578.1	0.531038006	2.94E-40	postive
CYLD	AL021578.1	0.631218177	8.23E-61	postive
EIF2AK2	AL021578.1	0.516646246	7.68E-38	postive
IREB2	AL021578.1	0.562622288	5.46E-46	postive
DDX17	AL021578.1	0.601454128	6.14E-54	postive
PIK3CG	AL021578.1	0.515615632	1.13E-37	postive
JAK2	AL021578.1	0.627067402	8.30E-60	postive
TXK	AL021578.1	0.610578542	5.74E-56	postive
NFAT5	AL021578.1	0.764631593	9.09E-104	postive
MALT1	AL021578.1	0.504040939	8.12E-36	postive
PIK3R1	AL021578.1	0.63163106	6.53E-61	postive
PIK3CA	AL021578.1	0.673931284	4.28E-72	postive
PIK3CB	AL021578.1	0.516565227	7.92E-38	postive
RASGRP3	AL021578.1	0.511445523	5.38E-37	postive
ROBO2	AL021578.1	0.528021801	9.65E-40	postive
GNRH1	AL021578.1	0.669393591	8.32E-71	postive
IL6ST	AL021578.1	0.605844016	6.61E-55	postive
ACVR2A	AL021578.1	0.582453253	6.47E-50	postive
ANGPTL1	AL021578.1	0.691592268	2.46E-77	postive
BMPR1A	AL021578.1	0.504326194	7.32E-36	postive
BMPR2	AL021578.1	0.730730569	2.03E-90	postive
CRLF3	AL021578.1	0.545689143	7.68E-43	postive
NR1D2	AL021578.1	0.565795455	1.34E-46	postive
NR2C2	AL021578.1	0.678262784	2.40E-73	postive
NR3C2	AL021578.1	0.500441989	2.97E-35	postive
RORA	AL021578.1	0.780865172	5.47E-111	postive
SOS1	AL021578.1	0.617480915	1.51E-57	postive
SOS2	AL021578.1	0.514498536	1.72E-37	postive
BRAF	AL021578.1	0.730954427	1.68E-90	postive
CBL	AL021578.1	0.525096822	3.02E-39	postive
CBLB	AL021578.1	0.698735142	1.45E-79	postive
TRAJ1	AL021578.1	0.620219635	3.48E-58	postive
TRAJ2	AL021578.1	0.674623545	2.71E-72	postive
TRAJ3	AL021578.1	0.706527981	4.50E-82	postive
TRAJ5	AL021578.1	0.632639493	3.70E-61	postive
TRAJ6	AL021578.1	0.689208944	1.32E-76	postive
TRAJ8	AL021578.1	0.636017025	5.45E-62	postive
TRAJ10	AL021578.1	0.662379188	7.37E-69	postive
TRAJ12	AL021578.1	0.669008978	1.07E-70	postive
TRAJ13	AL021578.1	0.631948268	5.46E-61	postive
TRAJ14	AL021578.1	0.657637013	1.43E-67	postive
TRAJ16	AL021578.1	0.713929233	1.56E-84	postive
TRAJ17	AL021578.1	0.651513951	6.04E-66	postive
TRAJ18	AL021578.1	0.692478917	1.31E-77	postive
TRAJ21	AL021578.1	0.68761481	4.01E-76	postive
TRAJ31	AL021578.1	0.702134655	1.19E-80	postive
TRAJ37	AL021578.1	0.675933908	1.14E-72	postive
TRAJ38	AL021578.1	0.778288995	8.41E-110	postive
TRAJ39	AL021578.1	0.697770157	2.93E-79	postive
CREB1	MCM3AP-AS1	0.726305442	7.94E-89	postive
RFXAP	MCM3AP-AS1	0.570690199	1.49E-47	postive
UBR1	MCM3AP-AS1	0.635414463	7.68E-62	postive
ZC3HAV1	MCM3AP-AS1	0.599176206	1.93E-53	postive
ZC3HAV1L	MCM3AP-AS1	0.65159293	5.76E-66	postive
CYLD	MCM3AP-AS1	0.512584061	3.52E-37	postive
EIF2AK2	MCM3AP-AS1	0.532134699	1.90E-40	postive
MAPK8	MCM3AP-AS1	0.580672272	1.50E-49	postive
LMBR1	MCM3AP-AS1	0.568483772	4.02E-47	postive
IREB2	MCM3AP-AS1	0.607492113	2.84E-55	postive

DDX17	MCM3AP-AS1	0.659508688	4.46E-68	postive
JAK2	MCM3AP-AS1	0.523178974	6.35E-39	postive
TXK	MCM3AP-AS1	0.516775677	7.31E-38	postive
NFAT5	MCM3AP-AS1	0.782488435	9.60E-112	postive
MALT1	MCM3AP-AS1	0.51700691	6.70E-38	postive
PIK3R1	MCM3AP-AS1	0.535232018	5.52E-41	postive
PIK3CA	MCM3AP-AS1	0.567938881	5.13E-47	postive
AKT3	MCM3AP-AS1	0.559900435	1.80E-45	postive
IGHD4-4	MCM3AP-AS1	0.550800347	8.99E-44	postive
LTB4R2	MCM3AP-AS1	0.525644865	2.44E-39	postive
GNRH1	MCM3AP-AS1	0.769252648	9.20E-106	postive
IL6ST	MCM3AP-AS1	0.537028513	2.68E-41	postive
RABEP1	MCM3AP-AS1	0.595255431	1.35E-52	postive
ACVR2A	MCM3AP-AS1	0.572612446	6.20E-48	postive
ACVR2B	MCM3AP-AS1	0.504738419	6.31E-36	postive
ANGPTL1	MCM3AP-AS1	0.672627495	1.01E-71	postive
BMPR1A	MCM3AP-AS1	0.535896162	4.22E-41	postive
BMPR2	MCM3AP-AS1	0.611418073	3.71E-56	postive
CRLF3	MCM3AP-AS1	0.668902995	1.14E-70	postive
LIFR	MCM3AP-AS1	0.500088235	3.37E-35	postive
NR2C1	MCM3AP-AS1	0.559600796	2.05E-45	postive
NR2C2	MCM3AP-AS1	0.754444149	1.58E-99	postive
RORA	MCM3AP-AS1	0.703713499	3.70E-81	postive
SOS1	MCM3AP-AS1	0.687677696	3.84E-76	postive
SOS2	MCM3AP-AS1	0.56810173	4.77E-47	postive
BRAF	MCM3AP-AS1	0.835105881	1.85E-140	postive
TEC	MCM3AP-AS1	0.516118948	9.37E-38	postive
CBL	MCM3AP-AS1	0.639310559	8.22E-63	postive
CBLB	MCM3AP-AS1	0.65566922	4.80E-67	postive
PDK1	MCM3AP-AS1	0.549415371	1.61E-43	postive
TRAJ1	MCM3AP-AS1	0.681847916	2.13E-74	postive
TRAJ2	MCM3AP-AS1	0.733234362	2.46E-91	postive
TRAJ3	MCM3AP-AS1	0.753479484	3.88E-99	postive
TRAJ5	MCM3AP-AS1	0.679650724	9.43E-74	postive
TRAJ6	MCM3AP-AS1	0.72726201	3.62E-89	postive
TRAJ8	MCM3AP-AS1	0.728827972	9.90E-90	postive
TRAJ10	MCM3AP-AS1	0.676417542	8.24E-73	postive
TRAJ12	MCM3AP-AS1	0.606508143	4.70E-55	postive
TRAJ13	MCM3AP-AS1	0.712151291	6.18E-84	postive
TRAJ14	MCM3AP-AS1	0.706436687	4.82E-82	postive
TRAJ16	MCM3AP-AS1	0.685105626	2.28E-75	postive
TRAJ17	MCM3AP-AS1	0.637153824	2.84E-62	postive
TRAJ18	MCM3AP-AS1	0.599324891	1.79E-53	postive
TRAJ21	MCM3AP-AS1	0.718364539	4.80E-86	postive
TRAJ31	MCM3AP-AS1	0.712309143	5.47E-84	postive
TRAJ37	MCM3AP-AS1	0.725838395	1.16E-88	postive
TRAJ38	MCM3AP-AS1	0.760426428	5.42E-102	postive
TRAJ39	MCM3AP-AS1	0.744124575	1.93E-95	postive
DDX17	AC060780.1	0.59013423	1.65E-51	postive
NFAT5	AC060780.1	0.506440879	3.39E-36	postive
LTB4R2	AC060780.1	0.507539354	2.27E-36	postive
GNRH1	AC060780.1	0.573392962	4.34E-48	postive
NR2C1	AC060780.1	0.517827222	4.91E-38	postive
NR2C2	AC060780.1	0.617365015	1.61E-57	postive
BRAF	AC060780.1	0.520444673	1.81E-38	postive
CYLD	AF131215.6	0.555981147	9.84E-45	postive
DDX17	AF131215.6	0.51717521	6.29E-38	postive
NFAT5	AF131215.6	0.631844731	5.79E-61	postive
ROBO2	AF131215.6	0.579722985	2.33E-49	postive
GNRH1	AF131215.6	0.635299235	8.20E-62	postive
ACVR2A	AF131215.6	0.516063419	9.57E-38	postive
RORA	AF131215.6	0.592061736	6.46E-52	postive
TRAJ1	AF131215.6	0.550266344	1.13E-43	postive
TRAJ2	AF131215.6	0.520369184	1.87E-38	postive
TRAJ3	AF131215.6	0.52777163	1.06E-39	postive

TRAJ6	AF131215.6	0.567012446	7.77E-47	postive
TRAJ10	AF131215.6	0.548166398	2.73E-43	postive
TRAJ12	AF131215.6	0.546897381	4.64E-43	postive
TRAJ13	AF131215.6	0.526078266	2.06E-39	postive
TRAJ16	AF131215.6	0.556465446	7.99E-45	postive
TRAJ17	AF131215.6	0.503209404	1.10E-35	postive
TRAJ18	AF131215.6	0.560184969	1.59E-45	postive
TRAJ21	AF131215.6	0.525159924	2.95E-39	postive
TRAJ31	AF131215.6	0.568073645	4.83E-47	postive
TRAJ37	AF131215.6	0.576745403	9.30E-49	postive
TRAJ38	AF131215.6	0.565158647	1.78E-46	postive
TRAJ39	AF131215.6	0.519074508	3.06E-38	postive
IRF9	AC093752.3	0.502545161	1.39E-35	postive
MUC5AC	AC010998.3	0.646741923	1.06E-64	postive
IL33	AC010998.3	0.613880105	1.02E-56	postive
MIA	AC010998.3	0.55409322	2.21E-44	postive
CRABP1	SOCS2-AS1	0.626830698	9.45E-60	postive
GPER1	SOCS2-AS1	0.539768692	8.80E-42	postive
IL17RD	SOCS2-AS1	0.596523668	7.21E-53	postive
GNRH1	AC079210.1	0.527382593	1.24E-39	postive
TRAJ1	AC079210.1	0.514920573	1.47E-37	postive
TRAJ3	AC079210.1	0.512544153	3.58E-37	postive
TRAJ5	AC079210.1	0.524649124	3.59E-39	postive
TRAJ6	AC079210.1	0.507642406	2.19E-36	postive
TRAJ8	AC079210.1	0.545075356	9.91E-43	postive
TRAJ12	AC079210.1	0.507762352	2.09E-36	postive
TRAJ13	AC079210.1	0.518875838	3.30E-38	postive
TRAJ14	AC079210.1	0.537834547	1.93E-41	postive
TRAJ16	AC079210.1	0.522862239	7.17E-39	postive
TRAJ17	AC079210.1	0.530703309	3.35E-40	postive
TRAJ21	AC079210.1	0.525182864	2.92E-39	postive
TRAJ31	AC079210.1	0.53980038	8.68E-42	postive
TRAJ38	AC079210.1	0.536988672	2.72E-41	postive
TRAJ39	AC079210.1	0.544269351	1.38E-42	postive
CREB1	Z98884.2	0.684221383	4.20E-75	postive
RFXAP	Z98884.2	0.521597477	1.17E-38	postive
UBR1	Z98884.2	0.551483085	6.73E-44	postive
ZC3HAV1	Z98884.2	0.52502499	3.11E-39	postive
ZC3HAV1L	Z98884.2	0.524535516	3.75E-39	postive
CYLD	Z98884.2	0.507504974	2.30E-36	postive
EIF2AK2	Z98884.2	0.533063922	1.31E-40	postive
MAPK8	Z98884.2	0.502345981	1.50E-35	postive
LMBR1	Z98884.2	0.515584609	1.15E-37	postive
IREB2	Z98884.2	0.554606053	1.78E-44	postive
DDX17	Z98884.2	0.649067969	2.64E-65	postive
JAK2	Z98884.2	0.533350378	1.17E-40	postive
TXK	Z98884.2	0.581456299	1.04E-49	postive
NFAT5	Z98884.2	0.716406846	2.25E-85	postive
MALT1	Z98884.2	0.50122063	2.24E-35	postive
PIK3R1	Z98884.2	0.503219	1.09E-35	postive
PIK3CA	Z98884.2	0.626237034	1.31E-59	postive
LTB4R2	Z98884.2	0.53698548	2.72E-41	postive
GNRH1	Z98884.2	0.794363031	1.76E-117	postive
RABEP1	Z98884.2	0.542386711	3.01E-42	postive
ACVR2A	Z98884.2	0.586332953	1.03E-50	postive
ANGPTL1	Z98884.2	0.655841189	4.32E-67	postive
BMPR2	Z98884.2	0.649829292	1.67E-65	postive
CRLF3	Z98884.2	0.541788468	3.85E-42	postive
NR2C1	Z98884.2	0.596621507	6.87E-53	postive
NR2C2	Z98884.2	0.726478233	6.89E-89	postive
RORA	Z98884.2	0.689771389	8.87E-77	postive
SOS1	Z98884.2	0.667721405	2.45E-70	postive
SOS2	Z98884.2	0.539862311	8.47E-42	postive
BRAF	Z98884.2	0.726825342	5.18E-89	postive
CBL	Z98884.2	0.626172778	1.36E-59	postive

CBLB	Z98884.2	0.653356248	1.98E-66	postive
PDK1	Z98884.2	0.513272316	2.73E-37	postive
TRAJ1	Z98884.2	0.604018803	1.68E-54	postive
TRAJ2	Z98884.2	0.570717245	1.47E-47	postive
TRAJ3	Z98884.2	0.648012044	4.96E-65	postive
TRAJ5	Z98884.2	0.626489108	1.14E-59	postive
TRAJ6	Z98884.2	0.619889843	4.15E-58	postive
TRAJ8	Z98884.2	0.600168862	1.17E-53	postive
TRAJ10	Z98884.2	0.600671194	9.11E-54	postive
TRAJ12	Z98884.2	0.563614946	3.52E-46	postive
TRAJ13	Z98884.2	0.61286609	1.74E-56	postive
TRAJ14	Z98884.2	0.591486013	8.55E-52	postive
TRAJ16	Z98884.2	0.612013728	2.71E-56	postive
TRAJ17	Z98884.2	0.590081621	1.69E-51	postive
TRAJ18	Z98884.2	0.557662023	4.76E-45	postive
TRAJ21	Z98884.2	0.633636332	2.11E-61	postive
TRAJ31	Z98884.2	0.649725973	1.78E-65	postive
TRAJ37	Z98884.2	0.625886505	1.59E-59	postive
TRAJ38	Z98884.2	0.669480333	7.86E-71	postive
TRAJ39	Z98884.2	0.639479141	7.46E-63	postive
CREB1	RRN3P2	0.724361358	3.89E-88	postive
CIITA	RRN3P2	0.545892912	7.05E-43	postive
RFXAP	RRN3P2	0.509986648	9.23E-37	postive
UBR1	RRN3P2	0.57764875	6.12E-49	postive
TLR4	RRN3P2	0.563093753	4.43E-46	postive
IL15	RRN3P2	0.666549299	5.20E-70	postive
CYLD	RRN3P2	0.708245929	1.23E-82	postive
TLR1	RRN3P2	0.563337791	3.98E-46	postive
LIMS1	RRN3P2	0.524042051	4.55E-39	postive
IREB2	RRN3P2	0.523057553	6.65E-39	postive
IL7R	RRN3P2	0.538982678	1.21E-41	postive
DDX17	RRN3P2	0.62596678	1.52E-59	postive
PIK3CG	RRN3P2	0.649470775	2.07E-65	postive
JAK2	RRN3P2	0.740181495	6.26E-94	postive
TXK	RRN3P2	0.600187054	1.16E-53	postive
PPP3CC	RRN3P2	0.503136373	1.13E-35	postive
NFAT5	RRN3P2	0.791728564	3.56E-116	postive
NFATC2	RRN3P2	0.517120079	6.42E-38	postive
MALT1	RRN3P2	0.531209288	2.75E-40	postive
PIK3R1	RRN3P2	0.657747537	1.33E-67	postive
PIK3CA	RRN3P2	0.651210497	7.26E-66	postive
AKT3	RRN3P2	0.534302937	8.01E-41	postive
RASGRP3	RRN3P2	0.662387247	7.33E-69	postive
IGHD6-6	RRN3P2	0.56139123	9.37E-46	postive
LTB4R2	RRN3P2	0.501283482	2.19E-35	postive
PLXNC1	RRN3P2	0.62759487	6.20E-60	postive
FGF7	RRN3P2	0.504661495	6.48E-36	postive
GNRH1	RRN3P2	0.705264293	1.16E-81	postive
IL6ST	RRN3P2	0.606850843	3.94E-55	postive
IL7	RRN3P2	0.553607246	2.73E-44	postive
RABEP1	RRN3P2	0.538861732	1.27E-41	postive
TNFSF8	RRN3P2	0.542637135	2.71E-42	postive
ACVR2A	RRN3P2	0.585602852	1.45E-50	postive
ANGPTL1	RRN3P2	0.751866445	1.73E-98	postive
BMPR2	RRN3P2	0.68536434	1.91E-75	postive
CRLF3	RRN3P2	0.643187089	8.61E-64	postive
IL18R1	RRN3P2	0.571274658	1.14E-47	postive
IL18RAP	RRN3P2	0.515477024	1.19E-37	postive
NR2C2	RRN3P2	0.653852603	1.46E-66	postive
RORA	RRN3P2	0.812522565	5.03E-127	postive
SOS1	RRN3P2	0.617838712	1.25E-57	postive
SOS2	RRN3P2	0.543243149	2.11E-42	postive
BRAF	RRN3P2	0.722094533	2.44E-87	postive
PTPRC	RRN3P2	0.626707539	1.01E-59	postive
ITK	RRN3P2	0.681889136	2.07E-74	postive

CD28	RRN3P2	0.627772959	5.62E-60	postive
CBL	RRN3P2	0.592616561	4.93E-52	postive
CBLB	RRN3P2	0.696831719	5.78E-79	postive
RASGRP1	RRN3P2	0.542365525	3.04E-42	postive
PDK1	RRN3P2	0.568491235	4.01E-47	postive
TRAJ1	RRN3P2	0.731151118	1.43E-90	postive
TRAJ2	RRN3P2	0.756066093	3.45E-100	postive
TRAJ3	RRN3P2	0.796469497	1.55E-118	postive
TRAJ5	RRN3P2	0.748628762	3.36E-97	postive
TRAJ6	RRN3P2	0.770140353	3.76E-106	postive
TRAJ8	RRN3P2	0.723600808	7.21E-88	postive
TRAJ10	RRN3P2	0.763365993	3.14E-103	postive
TRAJ12	RRN3P2	0.683633107	6.29E-75	postive
TRAJ13	RRN3P2	0.7362135	1.94E-92	postive
TRAJ14	RRN3P2	0.732835491	3.45E-91	postive
TRAJ16	RRN3P2	0.777976759	1.17E-109	postive
TRAJ17	RRN3P2	0.737116193	8.95E-93	postive
TRAJ18	RRN3P2	0.751726619	1.97E-98	postive
TRAJ21	RRN3P2	0.772126984	5.00E-107	postive
TRAJ31	RRN3P2	0.776495909	5.51E-109	postive
TRAJ37	RRN3P2	0.759237566	1.70E-101	postive
TRAJ38	RRN3P2	0.824545332	6.16E-134	postive
TRAJ39	RRN3P2	0.784202777	1.50E-112	postive
GNRH1	AC106028.3	0.520920875	1.51E-38	postive
UBR1	AC025171.2	0.50183199	1.80E-35	postive
DDX17	AC025171.2	0.566893374	8.20E-47	postive
NFAT5	AC025171.2	0.55288101	3.72E-44	postive
PIK3R1	AC025171.2	0.50518349	5.36E-36	postive
LTB4R2	AC025171.2	0.518740687	3.47E-38	postive
GNRH1	AC025171.2	0.569359223	2.71E-47	postive
IL6ST	AC025171.2	0.553723001	2.59E-44	postive
NR2C2	AC025171.2	0.664541141	1.87E-69	postive
NR3C2	AC025171.2	0.518076851	4.47E-38	postive
RORA	AC025171.2	0.562006608	7.15E-46	postive
BRAF	AC025171.2	0.569690715	2.33E-47	postive
CREB1	CHROMR	0.652360149	3.62E-66	postive
RFXAP	CHROMR	0.545655343	7.79E-43	postive
IL15	CHROMR	0.525952305	2.17E-39	postive
CYLD	CHROMR	0.528224441	8.91E-40	postive
IREB2	CHROMR	0.507128412	2.64E-36	postive
DDX17	CHROMR	0.577562608	6.37E-49	postive
JAK2	CHROMR	0.524749055	3.46E-39	postive
NFAT5	CHROMR	0.667216312	3.39E-70	postive
PIK3CA	CHROMR	0.536367901	3.49E-41	postive
AKT3	CHROMR	0.556320061	8.51E-45	postive
GNRH1	CHROMR	0.654596104	9.27E-67	postive
RABEP1	CHROMR	0.50752072	2.29E-36	postive
ACVR2A	CHROMR	0.567398705	6.54E-47	postive
ANGPTL1	CHROMR	0.579527651	2.55E-49	postive
BMPR2	CHROMR	0.608100078	2.07E-55	postive
CRLF3	CHROMR	0.523224947	6.23E-39	postive
NR2C1	CHROMR	0.526339303	1.86E-39	postive
NR2C2	CHROMR	0.557193542	5.83E-45	postive
RORA	CHROMR	0.673633398	5.21E-72	postive
SOS1	CHROMR	0.535272661	5.43E-41	postive
BRAF	CHROMR	0.576706807	9.47E-49	postive
CBLB	CHROMR	0.524622133	3.63E-39	postive
PDK1	CHROMR	0.503360201	1.04E-35	postive
TRAJ1	CHROMR	0.564867299	2.02E-46	postive
TRAJ2	CHROMR	0.619248977	5.86E-58	postive
TRAJ3	CHROMR	0.631951611	5.45E-61	postive
TRAJ5	CHROMR	0.56157542	8.64E-46	postive
TRAJ6	CHROMR	0.605683001	7.18E-55	postive
TRAJ8	CHROMR	0.572454099	6.67E-48	postive
TRAJ10	CHROMR	0.581410875	1.06E-49	postive

TRAJ12	CHROMR	0.521753067	1.10E-38	postive
TRAJ13	CHROMR	0.575493353	1.66E-48	postive
TRAJ14	CHROMR	0.561889582	7.53E-46	postive
TRAJ16	CHROMR	0.589298984	2.47E-51	postive
TRAJ17	CHROMR	0.544815169	1.10E-42	postive
TRAJ18	CHROMR	0.543029238	2.31E-42	postive
TRAJ21	CHROMR	0.591882539	7.05E-52	postive
TRAJ31	CHROMR	0.586371091	1.01E-50	postive
TRAJ37	CHROMR	0.603280913	2.44E-54	postive
TRAJ38	CHROMR	0.633866305	1.85E-61	postive
TRAJ39	CHROMR	0.613286735	1.39E-56	postive
PTGER2	AC012511.1	0.527726298	1.08E-39	postive
CREB1	AL157786.1	0.660775792	2.02E-68	postive
UBR1	AL157786.1	0.635712374	6.48E-62	postive
CYLD	AL157786.1	0.525394394	2.69E-39	postive
EIF2AK2	AL157786.1	0.502310278	1.52E-35	postive
MAPK8	AL157786.1	0.501448782	2.07E-35	postive
IREB2	AL157786.1	0.532869145	1.42E-40	postive
DDX17	AL157786.1	0.689919806	8.00E-77	postive
JAK2	AL157786.1	0.534293368	8.04E-41	postive
TXK	AL157786.1	0.622568786	9.76E-59	postive
NFAT5	AL157786.1	0.724163928	4.57E-88	postive
PIK3R1	AL157786.1	0.58362806	3.72E-50	postive
PIK3CA	AL157786.1	0.551874051	5.70E-44	postive
IGHD6-6	AL157786.1	0.55331958	3.08E-44	postive
GNRH1	AL157786.1	0.769438418	7.63E-106	postive
IL6ST	AL157786.1	0.577959222	5.30E-49	postive
RABEP1	AL157786.1	0.517327035	5.94E-38	postive
ACVR2A	AL157786.1	0.559418869	2.22E-45	postive
ANGPTL1	AL157786.1	0.598091027	3.31E-53	postive
BMPR2	AL157786.1	0.645312646	2.46E-64	postive
CRLF3	AL157786.1	0.536186806	3.76E-41	postive
NR2C2	AL157786.1	0.760082194	7.55E-102	postive
NR3C2	AL157786.1	0.518911538	3.25E-38	postive
RORA	AL157786.1	0.736562879	1.44E-92	postive
SOS1	AL157786.1	0.593867283	2.67E-52	postive
BRAF	AL157786.1	0.721248394	4.81E-87	postive
TEC	AL157786.1	0.6225729	9.74E-59	postive
CBLB	AL157786.1	0.627618774	6.12E-60	postive
RASGRP1	AL157786.1	0.518229585	4.22E-38	postive
TRAJ1	AL157786.1	0.596096366	8.91E-53	postive
TRAJ2	AL157786.1	0.558548859	3.24E-45	postive
TRAJ3	AL157786.1	0.634836564	1.07E-61	postive
TRAJ5	AL157786.1	0.620095968	3.72E-58	postive
TRAJ6	AL157786.1	0.608287884	1.88E-55	postive
TRAJ8	AL157786.1	0.553488234	2.87E-44	postive
TRAJ10	AL157786.1	0.561679876	8.26E-46	postive
TRAJ12	AL157786.1	0.580023677	2.03E-49	postive
TRAJ13	AL157786.1	0.556441552	8.07E-45	postive
TRAJ14	AL157786.1	0.562283799	6.33E-46	postive
TRAJ16	AL157786.1	0.589846813	1.89E-51	postive
TRAJ17	AL157786.1	0.542096288	3.39E-42	postive
TRAJ18	AL157786.1	0.589337687	2.42E-51	postive
TRAJ21	AL157786.1	0.612275569	2.37E-56	postive
TRAJ31	AL157786.1	0.613452762	1.28E-56	postive
TRAJ37	AL157786.1	0.615870473	3.56E-57	postive
TRAJ38	AL157786.1	0.653933261	1.39E-66	postive
TRAJ39	AL157786.1	0.658715892	7.30E-68	postive
CD4	AC093278.2	0.512006239	4.37E-37	postive
CIITA	AC093278.2	0.508745474	1.46E-36	postive
A2M	AC093278.2	0.66695318	4.01E-70	postive
CETP	AC093278.2	0.545948406	6.89E-43	postive
IL7R	AC093278.2	0.518628985	3.62E-38	postive
CD40LG	AC093278.2	0.60005322	1.24E-53	postive
AGER	AC093278.2	0.550346132	1.09E-43	postive

CCL14	AC093278.2	0.639729611	6.45E-63	postive
PTK2B	AC093278.2	0.518751066	3.46E-38	postive
BTK	AC093278.2	0.523077323	6.60E-39	postive
PIK3R5	AC093278.2	0.581853499	8.59E-50	postive
RASGRP3	AC093278.2	0.515216302	1.32E-37	postive
PLCG2	AC093278.2	0.535908704	4.20E-41	postive
PRKCB	AC093278.2	0.583763636	3.48E-50	postive
SEMA3G	AC093278.2	0.538095169	1.74E-41	postive
SLIT2	AC093278.2	0.621474787	1.77E-58	postive
CMKLR1	AC093278.2	0.502608939	1.36E-35	postive
ACKR1	AC093278.2	0.51642134	8.36E-38	postive
EDNRB	AC093278.2	0.63670103	3.69E-62	postive
ADA2	AC093278.2	0.503556412	9.68E-36	postive
VEGFD	AC093278.2	0.59162849	7.98E-52	postive
IL16	AC093278.2	0.572413369	6.79E-48	postive
ACVRL1	AC093278.2	0.580096773	1.96E-49	postive
CALCRL	AC093278.2	0.673371723	6.19E-72	postive
CSF2RB	AC093278.2	0.538258221	1.63E-41	postive
IL3RA	AC093278.2	0.529349205	5.73E-40	postive
S1PR1	AC093278.2	0.74092729	3.26E-94	postive
TEK	AC093278.2	0.690927122	3.93E-77	postive
TGFBR2	AC093278.2	0.509381082	1.15E-36	postive
TIE1	AC093278.2	0.53190866	2.08E-40	postive
ITGAL	AC093278.2	0.517698805	5.16E-38	postive
CREB1	ATP13A4-AS1	0.540129415	7.59E-42	postive
CYLD	ATP13A4-AS1	0.543384479	2.00E-42	postive
DDX17	ATP13A4-AS1	0.51976329	2.35E-38	postive
NFAT5	ATP13A4-AS1	0.674593373	2.77E-72	postive
PIK3R1	ATP13A4-AS1	0.560118228	1.64E-45	postive
IL6ST	ATP13A4-AS1	0.591552224	8.28E-52	postive
ANGPTL1	ATP13A4-AS1	0.519457097	2.64E-38	postive
BMPR2	ATP13A4-AS1	0.550442191	1.05E-43	postive
RORA	ATP13A4-AS1	0.741788103	1.53E-94	postive
BRAF	ATP13A4-AS1	0.574987066	2.09E-48	postive
CBLB	ATP13A4-AS1	0.502662061	1.34E-35	postive
TRAJ1	ATP13A4-AS1	0.501352667	2.14E-35	postive
TRAJ2	ATP13A4-AS1	0.637695502	2.08E-62	postive
TRAJ3	ATP13A4-AS1	0.579813368	2.24E-49	postive
TRAJ5	ATP13A4-AS1	0.548888363	2.01E-43	postive
TRAJ6	ATP13A4-AS1	0.570037552	2.00E-47	postive
TRAJ8	ATP13A4-AS1	0.541238239	4.82E-42	postive
TRAJ10	ATP13A4-AS1	0.562238193	6.46E-46	postive
TRAJ12	ATP13A4-AS1	0.551877973	5.69E-44	postive
TRAJ13	ATP13A4-AS1	0.573170279	4.81E-48	postive
TRAJ14	ATP13A4-AS1	0.563401583	3.87E-46	postive
TRAJ16	ATP13A4-AS1	0.56402759	2.93E-46	postive
TRAJ17	ATP13A4-AS1	0.519551367	2.55E-38	postive
TRAJ18	ATP13A4-AS1	0.605389083	8.34E-55	postive
TRAJ21	ATP13A4-AS1	0.605649036	7.30E-55	postive
TRAJ31	ATP13A4-AS1	0.624401773	3.59E-59	postive
TRAJ37	ATP13A4-AS1	0.655209627	6.36E-67	postive
TRAJ38	ATP13A4-AS1	0.651972089	4.58E-66	postive
TRAJ39	ATP13A4-AS1	0.601558005	5.83E-54	postive
CREB1	AL031717.1	0.567983238	5.03E-47	postive
CIITA	AL031717.1	0.510753834	6.95E-37	postive
CYLD	AL031717.1	0.502014503	1.69E-35	postive
NFKBIZ	AL031717.1	0.556958931	6.46E-45	postive
DDX17	AL031717.1	0.653558488	1.75E-66	postive
NFAT5	AL031717.1	0.643076562	9.18E-64	postive
LTB4R2	AL031717.1	0.595647122	1.11E-52	postive
GNRH1	AL031717.1	0.679599626	9.77E-74	postive
ANGPTL1	AL031717.1	0.548643915	2.23E-43	postive
NR2C1	AL031717.1	0.515642908	1.12E-37	postive
NR2C2	AL031717.1	0.704018609	2.95E-81	postive
RORA	AL031717.1	0.624963748	2.64E-59	postive

BRAF	AL031717.1	0.594347588	2.11E-52	postive
CBL	AL031717.1	0.507487462	2.31E-36	postive
CBLB	AL031717.1	0.562711921	5.24E-46	postive
TRAJ1	AL031717.1	0.55414667	2.16E-44	postive
TRAJ3	AL031717.1	0.59177959	7.41E-52	postive
TRAJ5	AL031717.1	0.567231013	7.05E-47	postive
TRAJ6	AL031717.1	0.565263453	1.70E-46	postive
TRAJ10	AL031717.1	0.573468242	4.20E-48	postive
TRAJ12	AL031717.1	0.50403828	8.13E-36	postive
TRAJ13	AL031717.1	0.516057979	9.59E-38	postive
TRAJ14	AL031717.1	0.51821763	4.24E-38	postive
TRAJ16	AL031717.1	0.543056892	2.28E-42	postive
TRAJ17	AL031717.1	0.531906283	2.08E-40	postive
TRAJ18	AL031717.1	0.554256082	2.06E-44	postive
TRAJ21	AL031717.1	0.555151278	1.41E-44	postive
TRAJ31	AL031717.1	0.552690934	4.03E-44	postive
TRAJ37	AL031717.1	0.54596538	6.84E-43	postive
TRAJ38	AL031717.1	0.591687782	7.75E-52	postive
TRAJ39	AL031717.1	0.562434841	5.92E-46	postive
CREB1	PCBP1-AS1	0.678477758	2.08E-73	postive
RFXAP	PCBP1-AS1	0.627203453	7.70E-60	postive
UBR1	PCBP1-AS1	0.576268451	1.16E-48	postive
IL15	PCBP1-AS1	0.504797552	6.17E-36	postive
CYLD	PCBP1-AS1	0.551077346	8.00E-44	postive
MAPK8	PCBP1-AS1	0.540912911	5.51E-42	postive
IREB2	PCBP1-AS1	0.57138342	1.08E-47	postive
DDX17	PCBP1-AS1	0.711782132	8.22E-84	postive
JAK2	PCBP1-AS1	0.561554117	8.73E-46	postive
TXK	PCBP1-AS1	0.556827908	6.83E-45	postive
NFAT5	PCBP1-AS1	0.750893044	4.24E-98	postive
PIK3R1	PCBP1-AS1	0.547201059	4.09E-43	postive
PIK3CA	PCBP1-AS1	0.568304016	4.36E-47	postive
RASGRP3	PCBP1-AS1	0.514862995	1.50E-37	postive
IGHD6-6	PCBP1-AS1	0.543624136	1.81E-42	postive
GNRH1	PCBP1-AS1	0.785430222	3.94E-113	postive
IL6ST	PCBP1-AS1	0.533654501	1.04E-40	postive
RABEP1	PCBP1-AS1	0.518088459	4.45E-38	postive
ACVR2A	PCBP1-AS1	0.638517331	1.30E-62	postive
ANGPTL1	PCBP1-AS1	0.673576382	5.41E-72	postive
BMPR1A	PCBP1-AS1	0.510744585	6.98E-37	postive
BMPR2	PCBP1-AS1	0.614285944	8.23E-57	postive
CRLF3	PCBP1-AS1	0.571000342	1.29E-47	postive
NR2C1	PCBP1-AS1	0.566574777	9.45E-47	postive
NR2C2	PCBP1-AS1	0.75780307	6.66E-101	postive
RORA	PCBP1-AS1	0.746473311	2.36E-96	postive
SOS1	PCBP1-AS1	0.636115927	5.15E-62	postive
SOS2	PCBP1-AS1	0.546373384	5.77E-43	postive
BRAF	PCBP1-AS1	0.696767051	6.06E-79	postive
CBL	PCBP1-AS1	0.510798344	6.84E-37	postive
CBLB	PCBP1-AS1	0.60568381	7.17E-55	postive
PDK1	PCBP1-AS1	0.540091747	7.71E-42	postive
TRAJ1	PCBP1-AS1	0.698605991	1.59E-79	postive
TRAJ2	PCBP1-AS1	0.67865966	1.84E-73	postive
TRAJ3	PCBP1-AS1	0.739840822	8.43E-94	postive
TRAJ5	PCBP1-AS1	0.698299431	1.99E-79	postive
TRAJ6	PCBP1-AS1	0.71442173	1.06E-84	postive
TRAJ8	PCBP1-AS1	0.697660196	3.17E-79	postive
TRAJ10	PCBP1-AS1	0.685716141	1.50E-75	postive
TRAJ12	PCBP1-AS1	0.656277423	3.30E-67	postive
TRAJ13	PCBP1-AS1	0.680175148	6.62E-74	postive
TRAJ14	PCBP1-AS1	0.685389252	1.88E-75	postive
TRAJ16	PCBP1-AS1	0.724529394	3.39E-88	postive
TRAJ17	PCBP1-AS1	0.68377534	5.70E-75	postive
TRAJ18	PCBP1-AS1	0.662194891	8.27E-69	postive
TRAJ21	PCBP1-AS1	0.716320504	2.41E-85	postive

TRAJ31	PCBP1-AS1	0.712177879	6.06E-84	postive
TRAJ37	PCBP1-AS1	0.710365736	2.44E-83	postive
TRAJ38	PCBP1-AS1	0.753201922	5.03E-99	postive
TRAJ39	PCBP1-AS1	0.744882251	9.83E-96	postive
CREB1	PABPC4-AS1	0.747226241	1.20E-96	postive
RFXAP	PABPC4-AS1	0.519692143	2.42E-38	postive
UBR1	PABPC4-AS1	0.626614579	1.07E-59	postive
ZC3HAV1	PABPC4-AS1	0.560160476	1.61E-45	postive
ZC3HAV1L	PABPC4-AS1	0.558827023	2.87E-45	postive
IL15	PABPC4-AS1	0.52740415	1.23E-39	postive
CYLD	PABPC4-AS1	0.549481909	1.57E-43	postive
EIF2AK2	PABPC4-AS1	0.518376305	3.99E-38	postive
MAPK8	PABPC4-AS1	0.564143989	2.79E-46	postive
LMBR1	PABPC4-AS1	0.573987187	3.31E-48	postive
IREB2	PABPC4-AS1	0.617517892	1.48E-57	postive
DDX17	PABPC4-AS1	0.578001812	5.20E-49	postive
PIK3CG	PABPC4-AS1	0.542985219	2.35E-42	postive
JAK2	PABPC4-AS1	0.576262089	1.16E-48	postive
TXK	PABPC4-AS1	0.539776822	8.77E-42	postive
NFAT5	PABPC4-AS1	0.762335653	8.56E-103	postive
MALT1	PABPC4-AS1	0.518867956	3.31E-38	postive
PIK3R1	PABPC4-AS1	0.609945566	7.98E-56	postive
PIK3CA	PABPC4-AS1	0.632614087	3.76E-61	postive
AKT3	PABPC4-AS1	0.61787303	1.23E-57	postive
IGHD4-4	PABPC4-AS1	0.517138065	6.38E-38	postive
IGHD6-6	PABPC4-AS1	0.524597578	3.67E-39	postive
GNRH1	PABPC4-AS1	0.69008527	7.12E-77	postive
IL6ST	PABPC4-AS1	0.608516622	1.67E-55	postive
RABEP1	PABPC4-AS1	0.558701172	3.03E-45	postive
ACVR2A	PABPC4-AS1	0.596250728	8.25E-53	postive
ANGPTL1	PABPC4-AS1	0.737248266	7.98E-93	postive
BMPR1A	PABPC4-AS1	0.546439067	5.62E-43	postive
BMPR2	PABPC4-AS1	0.687572692	4.13E-76	postive
CRLF3	PABPC4-AS1	0.663411012	3.84E-69	postive
LIFR	PABPC4-AS1	0.511720714	4.86E-37	postive
NR2C2	PABPC4-AS1	0.675113701	1.96E-72	postive
RORA	PABPC4-AS1	0.758467488	3.54E-101	postive
SOS1	PABPC4-AS1	0.675048927	2.05E-72	postive
SOS2	PABPC4-AS1	0.563706324	3.38E-46	postive
BRAF	PABPC4-AS1	0.834453009	4.83E-140	postive
CBL	PABPC4-AS1	0.592612149	4.94E-52	postive
CBLB	PABPC4-AS1	0.707385718	2.36E-82	postive
PDK1	PABPC4-AS1	0.581195714	1.17E-49	postive
TRAJ1	PABPC4-AS1	0.668304306	1.68E-70	postive
TRAJ2	PABPC4-AS1	0.769025043	1.16E-105	postive
TRAJ3	PABPC4-AS1	0.764157403	1.45E-103	postive
TRAJ5	PABPC4-AS1	0.708487461	1.02E-82	postive
TRAJ6	PABPC4-AS1	0.728542973	1.25E-89	postive
TRAJ8	PABPC4-AS1	0.769618206	6.37E-106	postive
TRAJ10	PABPC4-AS1	0.673228708	6.80E-72	postive
TRAJ12	PABPC4-AS1	0.609321644	1.10E-55	postive
TRAJ13	PABPC4-AS1	0.741056847	2.91E-94	postive
TRAJ14	PABPC4-AS1	0.738548924	2.59E-93	postive
TRAJ16	PABPC4-AS1	0.720364024	9.77E-87	postive
TRAJ17	PABPC4-AS1	0.668330697	1.65E-70	postive
TRAJ18	PABPC4-AS1	0.60366432	2.01E-54	postive
TRAJ21	PABPC4-AS1	0.746123365	3.23E-96	postive
TRAJ31	PABPC4-AS1	0.745376303	6.32E-96	postive
TRAJ37	PABPC4-AS1	0.735330646	4.14E-92	postive
TRAJ38	PABPC4-AS1	0.807649201	2.31E-124	postive
TRAJ39	PABPC4-AS1	0.774923795	2.82E-108	postive
NR4A3	AC073316.2	0.568576542	3.86E-47	postive
CREB1	AC020915.2	0.711190322	1.30E-83	postive
RFXAP	AC020915.2	0.517402714	5.77E-38	postive
UBR1	AC020915.2	0.583788036	3.44E-50	postive

ZC3HAV1	AC020915.2	0.584733249	2.20E-50	postive
ZC3HAV1L	AC020915.2	0.536598351	3.18E-41	postive
CYLD	AC020915.2	0.546036415	6.64E-43	postive
EIF2AK2	AC020915.2	0.560784494	1.22E-45	postive
MAPK8	AC020915.2	0.552299315	4.76E-44	postive
LMBR1	AC020915.2	0.520408726	1.84E-38	postive
IREB2	AC020915.2	0.593242503	3.63E-52	postive
DDX17	AC020915.2	0.646560887	1.18E-64	postive
JAK2	AC020915.2	0.585398177	1.60E-50	postive
TXK	AC020915.2	0.58666194	8.77E-51	postive
NFAT5	AC020915.2	0.796778695	1.08E-118	postive
MALT1	AC020915.2	0.510125684	8.77E-37	postive
PIK3R1	AC020915.2	0.552383974	4.59E-44	postive
PIK3CA	AC020915.2	0.644234158	4.65E-64	postive
IGHD6-6	AC020915.2	0.518150978	4.34E-38	postive
GNRH1	AC020915.2	0.764309691	1.25E-103	postive
IL6ST	AC020915.2	0.563137799	4.35E-46	postive
RABEP1	AC020915.2	0.517439972	5.69E-38	postive
ACVR2A	AC020915.2	0.598990953	2.11E-53	postive
ANGPTL1	AC020915.2	0.660405881	2.54E-68	postive
BMPR1A	AC020915.2	0.512185523	4.09E-37	postive
BMPR2	AC020915.2	0.651192128	7.34E-66	postive
CRLF3	AC020915.2	0.587016087	7.40E-51	postive
NR2C1	AC020915.2	0.571753188	9.17E-48	postive
NR2C2	AC020915.2	0.745202222	7.39E-96	postive
RORA	AC020915.2	0.798015882	2.54E-119	postive
SOS1	AC020915.2	0.680474451	5.41E-74	postive
SOS2	AC020915.2	0.553031996	3.48E-44	postive
BRAF	AC020915.2	0.769085885	1.09E-105	postive
CBL	AC020915.2	0.602351351	3.91E-54	postive
CBLB	AC020915.2	0.676139115	9.92E-73	postive
PDK1	AC020915.2	0.575987532	1.32E-48	postive
TRAJ1	AC020915.2	0.676918831	5.90E-73	postive
TRAJ2	AC020915.2	0.646188267	1.47E-64	postive
TRAJ3	AC020915.2	0.710923709	1.59E-83	postive
TRAJ5	AC020915.2	0.733804992	1.52E-91	postive
TRAJ6	AC020915.2	0.703447129	4.51E-81	postive
TRAJ8	AC020915.2	0.672221631	1.32E-71	postive
TRAJ10	AC020915.2	0.684904601	2.62E-75	postive
TRAJ12	AC020915.2	0.60838204	1.79E-55	postive
TRAJ13	AC020915.2	0.701464122	1.96E-80	postive
TRAJ14	AC020915.2	0.664749404	1.64E-69	postive
TRAJ16	AC020915.2	0.701046649	2.67E-80	postive
TRAJ17	AC020915.2	0.656836667	2.34E-67	postive
TRAJ18	AC020915.2	0.650357838	1.21E-65	postive
TRAJ21	AC020915.2	0.720886288	6.43E-87	postive
TRAJ31	AC020915.2	0.74801791	5.85E-97	postive
TRAJ37	AC020915.2	0.734315094	9.84E-92	postive
TRAJ38	AC020915.2	0.75328416	4.66E-99	postive
TRAJ39	AC020915.2	0.75298487	6.15E-99	postive
CREB1	AL359962.2	0.668551789	1.43E-70	postive
UBR1	AL359962.2	0.568814334	3.46E-47	postive
ZC3HAV1	AL359962.2	0.514208557	1.92E-37	postive
IL15	AL359962.2	0.541740418	3.93E-42	postive
CYLD	AL359962.2	0.592265899	5.85E-52	postive
EIF2AK2	AL359962.2	0.549003651	1.92E-43	postive
IREB2	AL359962.2	0.53263809	1.56E-40	postive
DDX17	AL359962.2	0.551902212	5.63E-44	postive
JAK2	AL359962.2	0.5962375	8.31E-53	postive
TXK	AL359962.2	0.539402592	1.02E-41	postive
NFAT5	AL359962.2	0.783688725	2.62E-112	postive
PIK3R1	AL359962.2	0.58031457	1.77E-49	postive
PIK3CA	AL359962.2	0.655514844	5.28E-67	postive
PIK3CB	AL359962.2	0.521774142	1.09E-38	postive
GNRH1	AL359962.2	0.643863717	5.79E-64	postive

IL6ST	AL359962.2	0.563501141	3.70E-46	postive
ACVR2A	AL359962.2	0.5484836	2.39E-43	postive
ANGPTL1	AL359962.2	0.707896441	1.60E-82	postive
BMPR2	AL359962.2	0.633045158	2.94E-61	postive
CRLF3	AL359962.2	0.569048977	3.12E-47	postive
NR2C2	AL359962.2	0.604053963	1.65E-54	postive
RORA	AL359962.2	0.781585782	2.53E-111	postive
SOS1	AL359962.2	0.617185538	1.77E-57	postive
SOS2	AL359962.2	0.514759822	1.56E-37	postive
BRAF	AL359962.2	0.698751113	1.43E-79	postive
CBL	AL359962.2	0.507718213	2.13E-36	postive
CBLB	AL359962.2	0.637283575	2.64E-62	postive
PDK1	AL359962.2	0.504007576	8.22E-36	postive
TRAJ1	AL359962.2	0.745120642	7.95E-96	postive
TRAJ2	AL359962.2	0.693555487	6.08E-78	postive
TRAJ3	AL359962.2	0.775839646	1.09E-108	postive
TRAJ5	AL359962.2	0.817441588	8.62E-130	postive
TRAJ6	AL359962.2	0.795052503	7.98E-118	postive
TRAJ8	AL359962.2	0.776692483	4.49E-109	postive
TRAJ10	AL359962.2	0.792148242	2.21E-116	postive
TRAJ12	AL359962.2	0.742600591	7.47E-95	postive
TRAJ13	AL359962.2	0.809537421	2.19E-125	postive
TRAJ14	AL359962.2	0.795651894	3.99E-118	postive
TRAJ16	AL359962.2	0.824990722	3.34E-134	postive
TRAJ17	AL359962.2	0.806969373	5.34E-124	postive
TRAJ18	AL359962.2	0.785710448	2.90E-113	postive
TRAJ21	AL359962.2	0.840138091	9.87E-144	postive
TRAJ31	AL359962.2	0.848615745	1.64E-149	postive
TRAJ37	AL359962.2	0.807700568	2.16E-124	postive
TRAJ38	AL359962.2	0.848175132	3.35E-149	postive
TRAJ39	AL359962.2	0.844227517	1.78E-146	postive
CREB1	AL031716.1	0.629234768	2.49E-60	postive
UBR1	AL031716.1	0.504073789	8.02E-36	postive
CYLD	AL031716.1	0.529795522	4.80E-40	postive
DDX17	AL031716.1	0.595051285	1.49E-52	postive
TXK	AL031716.1	0.560627854	1.31E-45	postive
NFAT5	AL031716.1	0.719573818	1.84E-86	postive
PIK3R1	AL031716.1	0.645558832	2.13E-64	postive
PIK3CA	AL031716.1	0.552324777	4.71E-44	postive
IGHD6-6	AL031716.1	0.501004384	2.43E-35	postive
GNRH1	AL031716.1	0.633380759	2.44E-61	postive
IL6ST	AL031716.1	0.687464016	4.46E-76	postive
ACVR2A	AL031716.1	0.542344468	3.06E-42	postive
ANGPTL1	AL031716.1	0.688112938	2.84E-76	postive
BMPR2	AL031716.1	0.641244814	2.68E-63	postive
CRLF3	AL031716.1	0.505628446	4.56E-36	postive
NR2C2	AL031716.1	0.579549549	2.53E-49	postive
RORA	AL031716.1	0.851206064	2.39E-151	postive
SOS1	AL031716.1	0.540887472	5.57E-42	postive
BRAF	AL031716.1	0.740735999	3.85E-94	postive
ITK	AL031716.1	0.56521167	1.73E-46	postive
CBLB	AL031716.1	0.615214295	5.04E-57	postive
TRAJ1	AL031716.1	0.541172647	4.95E-42	postive
TRAJ2	AL031716.1	0.606627855	4.42E-55	postive
TRAJ3	AL031716.1	0.616355707	2.75E-57	postive
TRAJ5	AL031716.1	0.715425151	4.86E-85	postive
TRAJ6	AL031716.1	0.586005459	1.20E-50	postive
TRAJ8	AL031716.1	0.619510897	5.09E-58	postive
TRAJ10	AL031716.1	0.582679269	5.82E-50	postive
TRAJ12	AL031716.1	0.504280956	7.44E-36	postive
TRAJ13	AL031716.1	0.678278835	2.37E-73	postive
TRAJ14	AL031716.1	0.603602182	2.07E-54	postive
TRAJ16	AL031716.1	0.601224696	6.89E-54	postive
TRAJ17	AL031716.1	0.593040004	4.01E-52	postive
TRAJ18	AL031716.1	0.570511044	1.61E-47	postive

TRAJ21	AL031716.1	0.690264702	6.27E-77	postive
TRAJ31	AL031716.1	0.721283092	4.68E-87	postive
TRAJ37	AL031716.1	0.711195733	1.29E-83	postive
TRAJ38	AL031716.1	0.689523756	1.06E-76	postive
TRAJ39	AL031716.1	0.717021906	1.39E-85	postive
CREB1	AP001469.2	0.638593294	1.24E-62	postive
RFXAP	AP001469.2	0.505504421	4.77E-36	postive
UBR1	AP001469.2	0.56709003	7.51E-47	postive
ZC3HAV1	AP001469.2	0.553037605	3.48E-44	postive
ZC3HAV1L	AP001469.2	0.638896152	1.04E-62	postive
MAPK8	AP001469.2	0.517579176	5.40E-38	postive
LMBR1	AP001469.2	0.513744204	2.28E-37	postive
IREB2	AP001469.2	0.549156241	1.80E-43	postive
DDX17	AP001469.2	0.6162177	2.96E-57	postive
NFAT5	AP001469.2	0.681972803	1.96E-74	postive
PIK3CA	AP001469.2	0.505404065	4.95E-36	postive
LTB4R2	AP001469.2	0.508410823	1.65E-36	postive
GNRH1	AP001469.2	0.681884712	2.08E-74	postive
RABEP1	AP001469.2	0.533818751	9.72E-41	postive
ANGPTL1	AP001469.2	0.566636911	9.19E-47	postive
BMPR2	AP001469.2	0.527794445	1.05E-39	postive
CRLF3	AP001469.2	0.585682903	1.40E-50	postive
NR2C1	AP001469.2	0.516565294	7.92E-38	postive
NR2C2	AP001469.2	0.721918898	2.81E-87	postive
RORA	AP001469.2	0.586615626	8.97E-51	postive
SOS1	AP001469.2	0.634415412	1.36E-61	postive
SOS2	AP001469.2	0.506423455	3.42E-36	postive
BRAF	AP001469.2	0.771373731	1.08E-106	postive
CBL	AP001469.2	0.60163585	5.60E-54	postive
CBLB	AP001469.2	0.573420871	4.29E-48	postive
TRAJ1	AP001469.2	0.596342365	7.89E-53	postive
TRAJ2	AP001469.2	0.659834484	3.64E-68	postive
TRAJ3	AP001469.2	0.67420453	3.58E-72	postive
TRAJ5	AP001469.2	0.565743052	1.37E-46	postive
TRAJ6	AP001469.2	0.628706153	3.35E-60	postive
TRAJ8	AP001469.2	0.633127662	2.81E-61	postive
TRAJ10	AP001469.2	0.575748855	1.47E-48	postive
TRAJ12	AP001469.2	0.51229363	3.93E-37	postive
TRAJ13	AP001469.2	0.603174387	2.57E-54	postive
TRAJ14	AP001469.2	0.606913165	3.82E-55	postive
TRAJ16	AP001469.2	0.590145665	1.64E-51	postive
TRAJ17	AP001469.2	0.537542047	2.17E-41	postive
TRAJ18	AP001469.2	0.512340238	3.86E-37	postive
TRAJ21	AP001469.2	0.610465422	6.09E-56	postive
TRAJ31	AP001469.2	0.597854979	3.72E-53	postive
TRAJ37	AP001469.2	0.616582137	2.44E-57	postive
TRAJ38	AP001469.2	0.651656962	5.54E-66	postive
TRAJ39	AP001469.2	0.63324037	2.64E-61	postive
CREB1	MECOM-AS1	0.687562902	4.16E-76	postive
UBR1	MECOM-AS1	0.610762956	5.21E-56	postive
MAPK8	MECOM-AS1	0.505227323	5.28E-36	postive
IREB2	MECOM-AS1	0.567415339	6.49E-47	postive
DDX17	MECOM-AS1	0.51788784	4.80E-38	postive
NFAT5	MECOM-AS1	0.742839536	6.04E-95	postive
PIK3R1	MECOM-AS1	0.514677008	1.61E-37	postive
PIK3CA	MECOM-AS1	0.547106394	4.25E-43	postive
ROBO2	MECOM-AS1	0.672660972	9.88E-72	postive
GNRH1	MECOM-AS1	0.703837901	3.38E-81	postive
IL6ST	MECOM-AS1	0.503981034	8.30E-36	postive
ACVR2A	MECOM-AS1	0.661679485	1.14E-68	postive
ANGPTL1	MECOM-AS1	0.558207326	3.76E-45	postive
BMPR1A	MECOM-AS1	0.601059793	7.49E-54	postive
BMPR2	MECOM-AS1	0.689712061	9.25E-77	postive
NR1D2	MECOM-AS1	0.551124937	7.84E-44	postive
NR2C2	MECOM-AS1	0.612524426	2.08E-56	postive

RORA	MECOM-AS1	0.698937677	1.25E-79	postive
SOS1	MECOM-AS1	0.579017873	3.24E-49	postive
BRAF	MECOM-AS1	0.632715888	3.55E-61	postive
TRAJ1	MECOM-AS1	0.5915366	8.34E-52	postive
TRAJ2	MECOM-AS1	0.557410319	5.31E-45	postive
TRAJ3	MECOM-AS1	0.623805369	4.98E-59	postive
TRAJ5	MECOM-AS1	0.604949519	1.04E-54	postive
TRAJ6	MECOM-AS1	0.656370417	3.12E-67	postive
TRAJ8	MECOM-AS1	0.630975765	9.43E-61	postive
TRAJ10	MECOM-AS1	0.642900917	1.02E-63	postive
TRAJ12	MECOM-AS1	0.655552005	5.16E-67	postive
TRAJ13	MECOM-AS1	0.627767187	5.63E-60	postive
TRAJ14	MECOM-AS1	0.591038029	1.06E-51	postive
TRAJ16	MECOM-AS1	0.626535433	1.11E-59	postive
TRAJ17	MECOM-AS1	0.617944558	1.18E-57	postive
TRAJ18	MECOM-AS1	0.583526353	3.90E-50	postive
TRAJ21	MECOM-AS1	0.628487701	3.78E-60	postive
TRAJ31	MECOM-AS1	0.710597877	2.04E-83	postive
TRAJ37	MECOM-AS1	0.695701596	1.31E-78	postive
TRAJ38	MECOM-AS1	0.691698583	2.28E-77	postive
TRAJ39	MECOM-AS1	0.640705607	3.66E-63	postive
CREB1	AC087752.4	0.578763719	3.65E-49	postive
MAPK8	AC087752.4	0.509310613	1.18E-36	postive
DDX17	AC087752.4	0.608074684	2.10E-55	postive
NFAT5	AC087752.4	0.592472051	5.29E-52	postive
GNRH1	AC087752.4	0.68121285	3.28E-74	postive
ANGPTL1	AC087752.4	0.567960838	5.08E-47	postive
CRLF3	AC087752.4	0.552843907	3.77E-44	postive
NR2C1	AC087752.4	0.560243627	1.55E-45	postive
NR2C2	AC087752.4	0.653487479	1.82E-66	postive
RORA	AC087752.4	0.596904293	5.97E-53	postive
SOS1	AC087752.4	0.53385719	9.57E-41	postive
BRAF	AC087752.4	0.675829529	1.22E-72	postive
CBLB	AC087752.4	0.569829931	2.19E-47	postive
TRAJ1	AC087752.4	0.551505547	6.67E-44	postive
TRAJ2	AC087752.4	0.542102976	3.38E-42	postive
TRAJ3	AC087752.4	0.563577927	3.58E-46	postive
TRAJ5	AC087752.4	0.609429566	1.04E-55	postive
TRAJ6	AC087752.4	0.567481691	6.30E-47	postive
TRAJ8	AC087752.4	0.601930874	4.83E-54	postive
TRAJ10	AC087752.4	0.528693042	7.41E-40	postive
TRAJ13	AC087752.4	0.607332431	3.08E-55	postive
TRAJ14	AC087752.4	0.577797814	5.71E-49	postive
TRAJ16	AC087752.4	0.575242877	1.86E-48	postive
TRAJ17	AC087752.4	0.541302956	4.70E-42	postive
TRAJ21	AC087752.4	0.60260827	3.43E-54	postive
TRAJ31	AC087752.4	0.596641943	6.80E-53	postive
TRAJ37	AC087752.4	0.567720539	5.66E-47	postive
TRAJ38	AC087752.4	0.607140332	3.40E-55	postive
TRAJ39	AC087752.4	0.621730371	1.54E-58	postive
LMBR1L	AC012615.6	0.529216494	6.03E-40	postive
TYK2	AC012615.6	0.629379906	2.30E-60	postive
NFKBIZ	AC012615.6	0.501902717	1.76E-35	postive
LTB4R	AC012615.6	0.53142974	2.52E-40	postive
DDX17	AC012615.6	0.56730892	6.81E-47	postive
IRF9	AC012615.6	0.658917745	6.44E-68	postive
LTB4R2	AC012615.6	0.656583785	2.73E-67	postive
GNRH1	AC012615.6	0.604950985	1.04E-54	postive
GIPR	AC012615.6	0.50042957	2.98E-35	postive
IL11RA	AC012615.6	0.534825994	6.49E-41	postive
NR2C2	AC012615.6	0.569509258	2.53E-47	postive
SH3BP2	AC012615.6	0.50085585	2.56E-35	postive
CD8A	TRBV11-2	0.522144675	9.45E-39	postive
LTA	TRBV11-2	0.588863729	3.05E-51	postive
APOBEC3G	TRBV11-2	0.519364794	2.74E-38	postive

CCL5	TRBV11-2	0.51687413	7.05E-38	postive
CD40LG	TRBV11-2	0.506600403	3.20E-36	postive
CCL19	TRBV11-2	0.548265209	2.62E-43	postive
CCR7	TRBV11-2	0.526393769	1.82E-39	postive
CCR5	TRBV11-2	0.546601249	5.25E-43	postive
CXCR4	TRBV11-2	0.518891358	3.28E-38	postive
CXCR6	TRBV11-2	0.529981983	4.46E-40	postive
CD79B	TRBV11-2	0.515158981	1.34E-37	postive
BTK	TRBV11-2	0.512290864	3.93E-37	postive
RAC2	TRBV11-2	0.534802871	6.55E-41	postive
GMFG	TRBV11-2	0.515281818	1.28E-37	postive
IL16	TRBV11-2	0.584297691	2.71E-50	postive
TNFSF13B	TRBV11-2	0.518656167	3.59E-38	postive
IL10RA	TRBV11-2	0.526147729	2.01E-39	postive
IL12RB1	TRBV11-2	0.516541705	7.99E-38	postive
IL2RB	TRBV11-2	0.523424049	5.77E-39	postive
IL21R	TRBV11-2	0.501168789	2.29E-35	postive
IL2RG	TRBV11-2	0.517796513	4.97E-38	postive
ITGAL	TRBV11-2	0.553548036	2.80E-44	postive
LCK	TRBV11-2	0.55714017	5.97E-45	postive
NCR3	TRBV11-2	0.592806014	4.49E-52	postive
CD247	TRBV11-2	0.646862628	9.83E-65	postive
CD48	TRBV11-2	0.577890165	5.47E-49	postive
SH2D1A	TRBV11-2	0.623866609	4.81E-59	postive
CD3D	TRBV11-2	0.644503106	3.97E-64	postive
CD3E	TRBV11-2	0.679197977	1.28E-73	postive
CD3G	TRBV11-2	0.624903055	2.73E-59	postive
ICOS	TRBV11-2	0.511942517	4.47E-37	postive
TRAC	TRBV11-2	0.675373765	1.65E-72	postive
TRAV4	TRBV11-2	0.552814082	3.82E-44	postive
TRAV8-2	TRBV11-2	0.55982593	1.86E-45	postive
TRAV8-4	TRBV11-2	0.64231839	1.43E-63	postive
TRAV8-6	TRBV11-2	0.6058603	6.55E-55	postive
TRAV9-2	TRBV11-2	0.580324942	1.76E-49	postive
TRAV12-2	TRBV11-2	0.56595777	1.24E-46	postive
TRAV12-3	TRBV11-2	0.514112942	1.99E-37	postive
TRAV13-2	TRBV11-2	0.556561344	7.67E-45	postive
TRAV16	TRBV11-2	0.648064456	4.80E-65	postive
TRAV17	TRBV11-2	0.529644391	5.10E-40	postive
TRAV29DV5	TRBV11-2	0.591406153	8.89E-52	postive
TRBC1	TRBV11-2	0.616728738	2.26E-57	postive
TRBC2	TRBV11-2	0.6142189	8.52E-57	postive
TRBV2	TRBV11-2	0.607994811	2.19E-55	postive
TRBV3-1	TRBV11-2	0.550136546	1.19E-43	postive
TRBV5-1	TRBV11-2	0.664565375	1.84E-69	postive
TRBV5-4	TRBV11-2	0.562924789	4.77E-46	postive
TRBV6-1	TRBV11-2	0.597826433	3.78E-53	postive
TRBV6-5	TRBV11-2	0.595345288	1.29E-52	postive
TRBV7-9	TRBV11-2	0.583887929	3.29E-50	postive
TRBV9	TRBV11-2	0.520273746	1.94E-38	postive
TRBV10-3	TRBV11-2	0.541079751	5.15E-42	postive
TRBV11-2	TRBV11-2	1	0	postive
TRBV12-4	TRBV11-2	0.533692556	1.02E-40	postive
TRBV18	TRBV11-2	0.629494781	2.16E-60	postive
TRBV20-1	TRBV11-2	0.626745269	9.91E-60	postive
TRBV28	TRBV11-2	0.665564627	9.76E-70	postive
CREB1	SAP30L-AS1	0.761649891	1.67E-102	postive
UBR1	SAP30L-AS1	0.644697853	3.54E-64	postive
ZC3HAV1	SAP30L-AS1	0.55196808	5.48E-44	postive
IL15	SAP30L-AS1	0.516642271	7.69E-38	postive
CYLD	SAP30L-AS1	0.67592844	1.14E-72	postive
EIF2AK2	SAP30L-AS1	0.501734658	1.87E-35	postive
LMBR1	SAP30L-AS1	0.561400245	9.34E-46	postive
IREB2	SAP30L-AS1	0.556283173	8.64E-45	postive
DDX17	SAP30L-AS1	0.605493122	7.91E-55	postive

PIK3CG	SAP30L-AS1	0.547931351	3.01E-43	postive
JAK2	SAP30L-AS1	0.634564612	1.25E-61	postive
TXK	SAP30L-AS1	0.557641959	4.80E-45	postive
NFAT5	SAP30L-AS1	0.888334818	2.72E-182	postive
NFATC3	SAP30L-AS1	0.57259433	6.25E-48	postive
MALT1	SAP30L-AS1	0.521249258	1.33E-38	postive
PIK3R1	SAP30L-AS1	0.645084235	2.82E-64	postive
PIK3CA	SAP30L-AS1	0.650939564	8.55E-66	postive
AKT3	SAP30L-AS1	0.507489289	2.31E-36	postive
RASGRP3	SAP30L-AS1	0.5124464	3.71E-37	postive
IGHD6-6	SAP30L-AS1	0.502597273	1.37E-35	postive
GNRH1	SAP30L-AS1	0.706666263	4.05E-82	postive
IL6ST	SAP30L-AS1	0.67605612	1.05E-72	postive
RABEP1	SAP30L-AS1	0.541502685	4.33E-42	postive
ACVR2A	SAP30L-AS1	0.613287862	1.39E-56	postive
ANGPTL1	SAP30L-AS1	0.688738965	1.83E-76	postive
BMPR1A	SAP30L-AS1	0.521868319	1.05E-38	postive
BMPR2	SAP30L-AS1	0.709177438	6.05E-83	postive
CRLF3	SAP30L-AS1	0.642766459	1.10E-63	postive
NR1D2	SAP30L-AS1	0.53215198	1.89E-40	postive
NR2C2	SAP30L-AS1	0.672966052	8.09E-72	postive
RORA	SAP30L-AS1	0.858037644	2.30E-156	postive
SOS1	SAP30L-AS1	0.627117145	8.07E-60	postive
SOS2	SAP30L-AS1	0.552103771	5.17E-44	postive
BRAF	SAP30L-AS1	0.771387278	1.06E-106	postive
CD28	SAP30L-AS1	0.534893913	6.32E-41	postive
CBL	SAP30L-AS1	0.564669435	2.21E-46	postive
CBLB	SAP30L-AS1	0.656738758	2.48E-67	postive
RASGRP1	SAP30L-AS1	0.512645046	3.44E-37	postive
PDK1	SAP30L-AS1	0.506243299	3.65E-36	postive
TRAJ1	SAP30L-AS1	0.742672265	7.01E-95	postive
TRAJ2	SAP30L-AS1	0.771361273	1.09E-106	postive
TRAJ3	SAP30L-AS1	0.796552614	1.40E-118	postive
TRAJ5	SAP30L-AS1	0.784004648	1.86E-112	postive
TRAJ6	SAP30L-AS1	0.805184669	4.78E-123	postive
TRAJ8	SAP30L-AS1	0.769750019	5.57E-106	postive
TRAJ10	SAP30L-AS1	0.792345882	1.77E-116	postive
TRAJ12	SAP30L-AS1	0.707111635	2.90E-82	postive
TRAJ13	SAP30L-AS1	0.797464535	4.85E-119	postive
TRAJ14	SAP30L-AS1	0.760949967	3.28E-102	postive
TRAJ16	SAP30L-AS1	0.790944977	8.62E-116	postive
TRAJ17	SAP30L-AS1	0.741207993	2.55E-94	postive
TRAJ18	SAP30L-AS1	0.741162746	2.65E-94	postive
TRAJ21	SAP30L-AS1	0.805049947	5.64E-123	postive
TRAJ31	SAP30L-AS1	0.843429842	6.19E-146	postive
TRAJ37	SAP30L-AS1	0.843557886	5.07E-146	postive
TRAJ38	SAP30L-AS1	0.855156884	3.23E-154	postive
TRAJ39	SAP30L-AS1	0.83539048	1.22E-140	postive
TRAJ1	AC109347.2	0.555797968	1.07E-44	postive
TRAJ3	AC109347.2	0.503468374	9.99E-36	postive
TRAJ5	AC109347.2	0.580558148	1.58E-49	postive
TRAJ6	AC109347.2	0.515435759	1.21E-37	postive
TRAJ8	AC109347.2	0.541518355	4.30E-42	postive
TRAJ10	AC109347.2	0.506200496	3.70E-36	postive
TRAJ12	AC109347.2	0.530926956	3.07E-40	postive
TRAJ13	AC109347.2	0.551110286	7.88E-44	postive
TRAJ14	AC109347.2	0.549384002	1.63E-43	postive
TRAJ16	AC109347.2	0.544836191	1.09E-42	postive
TRAJ17	AC109347.2	0.562492374	5.78E-46	postive
TRAJ18	AC109347.2	0.504093975	7.97E-36	postive
TRAJ21	AC109347.2	0.564246038	2.66E-46	postive
TRAJ31	AC109347.2	0.575159178	1.93E-48	postive
TRAJ37	AC109347.2	0.520947495	1.50E-38	postive
TRAJ38	AC109347.2	0.553093281	3.39E-44	postive
TRAJ39	AC109347.2	0.571338873	1.11E-47	postive

B2M	LINC01094	0.535805654	4.38E-41	postive
CD4	LINC01094	0.532088891	1.94E-40	postive
CTSB	LINC01094	0.536842861	2.88E-41	postive
CTSS	LINC01094	0.518688854	3.54E-38	postive
FCER1G	LINC01094	0.682443596	1.42E-74	postive
HLA-DMB	LINC01094	0.614930728	5.85E-57	postive
HLA-DRA	LINC01094	0.590195009	1.60E-51	postive
CXCL10	LINC01094	0.572265503	7.26E-48	postive
CCL13	LINC01094	0.543988695	1.55E-42	postive
CCL8	LINC01094	0.578998927	3.27E-49	postive
IFNGR1	LINC01094	0.503313833	1.06E-35	postive
TLR4	LINC01094	0.646902005	9.60E-65	postive
CYBB	LINC01094	0.698716804	1.47E-79	postive
TLR7	LINC01094	0.572071659	7.93E-48	postive
TLR8	LINC01094	0.643712466	6.32E-64	postive
TLR1	LINC01094	0.601333466	6.53E-54	postive
MSR1	LINC01094	0.793895737	3.01E-117	postive
CD14	LINC01094	0.678623695	1.88E-73	postive
TNFSF4	LINC01094	0.506528412	3.29E-36	postive
CCL2	LINC01094	0.500626645	2.78E-35	postive
CCL3	LINC01094	0.563728827	3.35E-46	postive
CCR1	LINC01094	0.584826079	2.10E-50	postive
IL10	LINC01094	0.609861457	8.33E-56	postive
CD86	LINC01094	0.789332806	5.26E-115	postive
RNASE2	LINC01094	0.52112063	1.40E-38	postive
BTK	LINC01094	0.608220548	1.95E-55	postive
FCGR2B	LINC01094	0.725181754	1.99E-88	postive
FPR1	LINC01094	0.604834527	1.11E-54	postive
GMFG	LINC01094	0.604275205	1.47E-54	postive
SPP1	LINC01094	0.538869089	1.27E-41	postive
TNFSF13B	LINC01094	0.682993253	9.74E-75	postive
C3AR1	LINC01094	0.706011185	6.64E-82	postive
CSF1R	LINC01094	0.581538552	9.96E-50	postive
CSF2RA	LINC01094	0.504062622	8.06E-36	postive
TYROBP	LINC01094	0.648309685	4.15E-65	postive
FCGR3A	LINC01094	0.7620609	1.12E-102	postive
LCP2	LINC01094	0.576540972	1.02E-48	postive
PTPRC	LINC01094	0.521648673	1.14E-38	postive
RFXAP	ZNF32-AS2	0.501264966	2.21E-35	postive
DDX17	ZNF32-AS2	0.625590222	1.87E-59	postive
NFAT5	ZNF32-AS2	0.586404329	9.92E-51	postive
LTB4R2	ZNF32-AS2	0.619677198	4.66E-58	postive
GNRH1	ZNF32-AS2	0.647006773	9.02E-65	postive
NR2C1	ZNF32-AS2	0.544373199	1.33E-42	postive
NR2C2	ZNF32-AS2	0.729432561	5.99E-90	postive
RORA	ZNF32-AS2	0.553619783	2.71E-44	postive
SOS1	ZNF32-AS2	0.515813119	1.05E-37	postive
BRAF	ZNF32-AS2	0.602670263	3.32E-54	postive
TRAJ1	ZNF32-AS2	0.564253855	2.65E-46	postive
TRAJ3	ZNF32-AS2	0.552386424	4.59E-44	postive
TRAJ5	ZNF32-AS2	0.564837606	2.05E-46	postive
TRAJ6	ZNF32-AS2	0.532730332	1.50E-40	postive
TRAJ8	ZNF32-AS2	0.525933906	2.18E-39	postive
TRAJ10	ZNF32-AS2	0.526732428	1.60E-39	postive
TRAJ13	ZNF32-AS2	0.53031667	3.91E-40	postive
TRAJ14	ZNF32-AS2	0.524730693	3.48E-39	postive
TRAJ16	ZNF32-AS2	0.559599585	2.05E-45	postive
TRAJ17	ZNF32-AS2	0.543143527	2.20E-42	postive
TRAJ18	ZNF32-AS2	0.509236807	1.22E-36	postive
TRAJ21	ZNF32-AS2	0.548458519	2.41E-43	postive
TRAJ31	ZNF32-AS2	0.54629307	5.97E-43	postive
TRAJ37	ZNF32-AS2	0.535497776	4.96E-41	postive
TRAJ38	ZNF32-AS2	0.555479764	1.22E-44	postive
TRAJ39	ZNF32-AS2	0.56530431	1.66E-46	postive
CREB1	AC008035.1	0.518881583	3.29E-38	postive

IL15	AC008035.1	0.514290526	1.86E-37	postive
NFAT5	AC008035.1	0.531958309	2.04E-40	postive
GNRH1	AC008035.1	0.50908044	1.29E-36	postive
IL7	AC008035.1	0.500986578	2.44E-35	postive
ACVR2A	AC008035.1	0.501065287	2.37E-35	postive
ANGPTL1	AC008035.1	0.61183354	2.98E-56	postive
RORA	AC008035.1	0.550500076	1.02E-43	postive
BRAF	AC008035.1	0.548758937	2.13E-43	postive
PDK1	AC008035.1	0.507437473	2.36E-36	postive
TRAJ1	AC008035.1	0.580742506	1.45E-49	postive
TRAJ3	AC008035.1	0.596411547	7.62E-53	postive
TRAJ5	AC008035.1	0.6520955	4.25E-66	postive
TRAJ6	AC008035.1	0.613294435	1.39E-56	postive
TRAJ8	AC008035.1	0.630552702	1.20E-60	postive
TRAJ10	AC008035.1	0.619791809	4.38E-58	postive
TRAJ12	AC008035.1	0.588778767	3.17E-51	postive
TRAJ13	AC008035.1	0.627343957	7.12E-60	postive
TRAJ14	AC008035.1	0.633981942	1.73E-61	postive
TRAJ16	AC008035.1	0.624226741	3.95E-59	postive
TRAJ17	AC008035.1	0.660625699	2.22E-68	postive
TRAJ18	AC008035.1	0.5739198	3.41E-48	postive
TRAJ21	AC008035.1	0.662131657	8.61E-69	postive
TRAJ31	AC008035.1	0.65414778	1.22E-66	postive
TRAJ37	AC008035.1	0.584898176	2.03E-50	postive
TRAJ38	AC008035.1	0.655443722	5.51E-67	postive
TRAJ39	AC008035.1	0.66506969	1.34E-69	postive
CREB1	AC090181.2	0.756938027	1.51E-100	postive
RFXAP	AC090181.2	0.512928543	3.10E-37	postive
UBR1	AC090181.2	0.674428289	3.08E-72	postive
ZC3HAV1	AC090181.2	0.529047083	6.45E-40	postive
IL15	AC090181.2	0.52944796	5.51E-40	postive
CYLD	AC090181.2	0.543258828	2.10E-42	postive
EIF2AK2	AC090181.2	0.564407697	2.48E-46	postive
MAPK8	AC090181.2	0.560687329	1.28E-45	postive
LMBR1	AC090181.2	0.547124084	4.22E-43	postive
IREB2	AC090181.2	0.642977102	9.73E-64	postive
DDX17	AC090181.2	0.602203492	4.21E-54	postive
JAK2	AC090181.2	0.60363172	2.04E-54	postive
TXK	AC090181.2	0.632261436	4.58E-61	postive
NFAT5	AC090181.2	0.74030375	5.62E-94	postive
MALT1	AC090181.2	0.507294061	2.48E-36	postive
PIK3R1	AC090181.2	0.634944842	1.00E-61	postive
PIK3CA	AC090181.2	0.655089282	6.85E-67	postive
PIK3CB	AC090181.2	0.532750841	1.49E-40	postive
RASGRP3	AC090181.2	0.517256629	6.10E-38	postive
ROBO2	AC090181.2	0.51501227	1.42E-37	postive
GNRH1	AC090181.2	0.741371027	2.21E-94	postive
IL6ST	AC090181.2	0.593104369	3.88E-52	postive
RABEP1	AC090181.2	0.554602895	1.78E-44	postive
ACVR2A	AC090181.2	0.628457953	3.84E-60	postive
ANGPTL1	AC090181.2	0.721534072	3.83E-87	postive
BMPR1A	AC090181.2	0.563757072	3.31E-46	postive
BMPR2	AC090181.2	0.745916311	3.90E-96	postive
CRLF3	AC090181.2	0.597083809	5.46E-53	postive
NR1D2	AC090181.2	0.552646585	4.11E-44	postive
NR2C2	AC090181.2	0.70733583	2.45E-82	postive
RORA	AC090181.2	0.770099841	3.92E-106	postive
SOS1	AC090181.2	0.681109782	3.52E-74	postive
SOS2	AC090181.2	0.536228431	3.69E-41	postive
BRAF	AC090181.2	0.793940836	2.86E-117	postive
TEC	AC090181.2	0.543427368	1.96E-42	postive
CBL	AC090181.2	0.546341653	5.85E-43	postive
CBLB	AC090181.2	0.746996469	1.47E-96	postive
PDK1	AC090181.2	0.554035999	2.27E-44	postive
TRAJ1	AC090181.2	0.623519734	5.81E-59	postive

TRAJ2	AC090181.2	0.644199591	4.75E-64	postive
TRAJ3	AC090181.2	0.684413875	3.68E-75	postive
TRAJ5	AC090181.2	0.667894775	2.19E-70	postive
TRAJ6	AC090181.2	0.675244071	1.80E-72	postive
TRAJ8	AC090181.2	0.643044095	9.36E-64	postive
TRAJ10	AC090181.2	0.62178372	1.49E-58	postive
TRAJ12	AC090181.2	0.678596593	1.92E-73	postive
TRAJ13	AC090181.2	0.636616354	3.87E-62	postive
TRAJ14	AC090181.2	0.661919765	9.84E-69	postive
TRAJ16	AC090181.2	0.657578509	1.48E-67	postive
TRAJ17	AC090181.2	0.609978952	7.84E-56	postive
TRAJ18	AC090181.2	0.626087546	1.42E-59	postive
TRAJ21	AC090181.2	0.678825813	1.64E-73	postive
TRAJ31	AC090181.2	0.71259232	4.40E-84	postive
TRAJ37	AC090181.2	0.673371278	6.19E-72	postive
TRAJ38	AC090181.2	0.769970569	4.46E-106	postive
TRAJ39	AC090181.2	0.699185858	1.04E-79	postive
LEPR	AC083809.1	0.528730128	7.30E-40	postive
CREB1	AL117336.1	0.60587231	6.51E-55	postive
IL15	AL117336.1	0.578317977	4.49E-49	postive
DDX17	AL117336.1	0.509420778	1.14E-36	postive
JAK2	AL117336.1	0.533026831	1.33E-40	postive
TXK	AL117336.1	0.501803691	1.82E-35	postive
NFAT5	AL117336.1	0.618073969	1.10E-57	postive
PIK3R1	AL117336.1	0.514027067	2.06E-37	postive
PIK3CA	AL117336.1	0.571834335	8.84E-48	postive
RASGRP3	AL117336.1	0.51613541	9.31E-38	postive
GNRH1	AL117336.1	0.587505241	5.85E-51	postive
IL6ST	AL117336.1	0.507402257	2.39E-36	postive
ANGPTL1	AL117336.1	0.697456589	3.68E-79	postive
BMPR2	AL117336.1	0.565850683	1.31E-46	postive
CRLF3	AL117336.1	0.523484601	5.64E-39	postive
NR2C2	AL117336.1	0.566933417	8.05E-47	postive
RORA	AL117336.1	0.701095392	2.57E-80	postive
SOS1	AL117336.1	0.566604516	9.33E-47	postive
BRAF	AL117336.1	0.646369872	1.32E-64	postive
ITK	AL117336.1	0.562716187	5.23E-46	postive
CBL	AL117336.1	0.518697719	3.53E-38	postive
CBLB	AL117336.1	0.60414322	1.57E-54	postive
PDK1	AL117336.1	0.545668426	7.74E-43	postive
TRAJ1	AL117336.1	0.590616288	1.30E-51	postive
TRAJ2	AL117336.1	0.524330601	4.07E-39	postive
TRAJ3	AL117336.1	0.621017061	2.26E-58	postive
TRAJ5	AL117336.1	0.711977147	7.07E-84	postive
TRAJ6	AL117336.1	0.606941091	3.77E-55	postive
TRAJ8	AL117336.1	0.616427841	2.65E-57	postive
TRAJ10	AL117336.1	0.630212339	1.45E-60	postive
TRAJ12	AL117336.1	0.557252218	5.69E-45	postive
TRAJ13	AL117336.1	0.650773917	9.45E-66	postive
TRAJ14	AL117336.1	0.624547859	3.32E-59	postive
TRAJ16	AL117336.1	0.652085138	4.28E-66	postive
TRAJ17	AL117336.1	0.671194917	2.58E-71	postive
TRAJ18	AL117336.1	0.58247493	6.41E-50	postive
TRAJ21	AL117336.1	0.678821749	1.65E-73	postive
TRAJ31	AL117336.1	0.68890787	1.63E-76	postive
TRAJ37	AL117336.1	0.63543954	7.57E-62	postive
TRAJ38	AL117336.1	0.686654064	7.83E-76	postive
TRAJ39	AL117336.1	0.688741185	1.83E-76	postive
CREB1	AC008770.3	0.811687625	1.46E-126	postive
RFXAP	AC008770.3	0.558456692	3.37E-45	postive
UBR1	AC008770.3	0.690092502	7.08E-77	postive
ZC3HAV1	AC008770.3	0.612786851	1.81E-56	postive
ZC3HAV1L	AC008770.3	0.52931759	5.80E-40	postive
IL15	AC008770.3	0.61345951	1.27E-56	postive
CYLD	AC008770.3	0.67343853	5.93E-72	postive

EIF2AK2	AC008770.3	0.557173721	5.88E-45	postive
MAPK8	AC008770.3	0.54917734	1.78E-43	postive
LMBR1	AC008770.3	0.576853835	8.85E-49	postive
IREB2	AC008770.3	0.633275366	2.59E-61	postive
DDX17	AC008770.3	0.676773791	6.50E-73	postive
PIK3CG	AC008770.3	0.593468927	3.25E-52	postive
JAK2	AC008770.3	0.695578859	1.43E-78	postive
TXK	AC008770.3	0.641677293	2.08E-63	postive
NFAT5	AC008770.3	0.881074606	1.92E-175	postive
NFATC3	AC008770.3	0.557601347	4.89E-45	postive
MALT1	AC008770.3	0.572241101	7.35E-48	postive
PIK3R1	AC008770.3	0.691510812	2.60E-77	postive
PIK3CA	AC008770.3	0.687225099	5.26E-76	postive
PIK3CB	AC008770.3	0.538455685	1.50E-41	postive
AKT3	AC008770.3	0.559454255	2.19E-45	postive
RASGRP3	AC008770.3	0.578695508	3.76E-49	postive
IGHD4-4	AC008770.3	0.519706042	2.40E-38	postive
IGHD6-6	AC008770.3	0.534221712	8.27E-41	postive
PLXNC1	AC008770.3	0.556975723	6.41E-45	postive
GNRH1	AC008770.3	0.79708047	7.59E-119	postive
IL6ST	AC008770.3	0.666177212	6.60E-70	postive
IL7	AC008770.3	0.511320974	5.63E-37	postive
RABEP1	AC008770.3	0.621317276	1.92E-58	postive
ACVR2A	AC008770.3	0.666296297	6.11E-70	postive
ANGPTL1	AC008770.3	0.775771567	1.17E-108	postive
BMPR1A	AC008770.3	0.5569371	6.52E-45	postive
BMPR2	AC008770.3	0.726474164	6.91E-89	postive
CRLF3	AC008770.3	0.684521008	3.42E-75	postive
LIFR	AC008770.3	0.503412606	1.02E-35	postive
NR1D2	AC008770.3	0.545192894	9.44E-43	postive
NR2C1	AC008770.3	0.527794781	1.05E-39	postive
NR2C2	AC008770.3	0.771492374	9.55E-107	postive
NR3C1	AC008770.3	0.515367635	1.24E-37	postive
RORA	AC008770.3	0.853556196	4.81E-153	postive
SOS1	AC008770.3	0.688824227	1.72E-76	postive
SOS2	AC008770.3	0.61891429	7.02E-58	postive
BRAF	AC008770.3	0.836267253	3.32E-141	postive
PTPRC	AC008770.3	0.503703265	9.18E-36	postive
ITK	AC008770.3	0.559519206	2.13E-45	postive
TEC	AC008770.3	0.520450227	1.81E-38	postive
CD28	AC008770.3	0.539517489	9.74E-42	postive
CBL	AC008770.3	0.649015603	2.72E-65	postive
CBLB	AC008770.3	0.726214534	8.55E-89	postive
RASGRP1	AC008770.3	0.591324349	9.25E-52	postive
PDK1	AC008770.3	0.581729346	9.10E-50	postive
TRAJ1	AC008770.3	0.79607852	2.44E-118	postive
TRAJ2	AC008770.3	0.77378838	9.12E-108	postive
TRAJ3	AC008770.3	0.842224521	4.02E-145	postive
TRAJ5	AC008770.3	0.838549757	1.10E-142	postive
TRAJ6	AC008770.3	0.84191912	6.44E-145	postive
TRAJ8	AC008770.3	0.804175556	1.63E-122	postive
TRAJ10	AC008770.3	0.817397947	9.12E-130	postive
TRAJ12	AC008770.3	0.755376042	6.60E-100	postive
TRAJ13	AC008770.3	0.830411226	1.67E-137	postive
TRAJ14	AC008770.3	0.816728612	2.19E-129	postive
TRAJ16	AC008770.3	0.807223023	3.91E-124	postive
TRAJ17	AC008770.3	0.789645105	3.71E-115	postive
TRAJ18	AC008770.3	0.774443826	4.64E-108	postive
TRAJ21	AC008770.3	0.852728736	1.92E-152	postive
TRAJ31	AC008770.3	0.852770786	1.79E-152	postive
TRAJ37	AC008770.3	0.843779767	3.58E-146	postive
TRAJ38	AC008770.3	0.881361726	1.05E-175	postive
TRAJ39	AC008770.3	0.868572924	1.22E-164	postive
FABP7	AL022316.1	0.616181362	3.02E-57	postive
HCK	AL022316.1	0.630787138	1.05E-60	postive

CREB1	AC007566.1	0.630977119	9.42E-61	postive
CIITA	AC007566.1	0.50156792	1.98E-35	postive
RFXAP	AC007566.1	0.527940237	9.96E-40	postive
UBR1	AC007566.1	0.571427407	1.06E-47	postive
CYLD	AC007566.1	0.623855756	4.84E-59	postive
DDX17	AC007566.1	0.714042025	1.43E-84	postive
JAK2	AC007566.1	0.568143213	4.68E-47	postive
TXK	AC007566.1	0.52213033	9.50E-39	postive
NFAT5	AC007566.1	0.786345849	1.44E-113	postive
NFATC3	AC007566.1	0.535883417	4.25E-41	postive
PIK3R1	AC007566.1	0.522948965	6.93E-39	postive
LTB4R2	AC007566.1	0.524624852	3.63E-39	postive
GNRH1	AC007566.1	0.749350759	1.74E-97	postive
IL6ST	AC007566.1	0.507673337	2.16E-36	postive
ACVR2A	AC007566.1	0.536839632	2.89E-41	postive
ANGPTL1	AC007566.1	0.530646158	3.43E-40	postive
BMPR2	AC007566.1	0.533914211	9.35E-41	postive
CRLF3	AC007566.1	0.540388213	6.83E-42	postive
NR2C1	AC007566.1	0.570011319	2.02E-47	postive
NR2C2	AC007566.1	0.724183126	4.49E-88	postive
RORA	AC007566.1	0.706281368	5.42E-82	postive
SOS1	AC007566.1	0.53439046	7.73E-41	postive
BRAF	AC007566.1	0.655798554	4.43E-67	postive
CBLB	AC007566.1	0.574540288	2.57E-48	postive
TRAJ1	AC007566.1	0.673330729	6.36E-72	postive
TRAJ2	AC007566.1	0.667752863	2.40E-70	postive
TRAJ3	AC007566.1	0.686536213	8.49E-76	postive
TRAJ5	AC007566.1	0.635755335	6.33E-62	postive
TRAJ6	AC007566.1	0.696510411	7.29E-79	postive
TRAJ8	AC007566.1	0.621218306	2.03E-58	postive
TRAJ10	AC007566.1	0.676957302	5.75E-73	postive
TRAJ12	AC007566.1	0.647247828	7.82E-65	postive
TRAJ13	AC007566.1	0.638029743	1.72E-62	postive
TRAJ14	AC007566.1	0.640249894	4.77E-63	postive
TRAJ16	AC007566.1	0.683075595	9.21E-75	postive
TRAJ17	AC007566.1	0.609468418	1.02E-55	postive
TRAJ18	AC007566.1	0.665536352	9.94E-70	postive
TRAJ21	AC007566.1	0.65533661	5.89E-67	postive
TRAJ31	AC007566.1	0.677902806	3.06E-73	postive
TRAJ37	AC007566.1	0.690718063	4.56E-77	postive
TRAJ38	AC007566.1	0.722706685	1.49E-87	postive
TRAJ39	AC007566.1	0.666231352	6.37E-70	postive
GNRH1	AP000866.5	0.502197504	1.58E-35	postive
IRF9	AL365330.1	0.548120287	2.78E-43	postive
EPOR	AL365330.1	0.506619939	3.18E-36	postive
GIPR	AL365330.1	0.500348598	3.07E-35	postive
ICAM1	AC011511.5	0.625712988	1.75E-59	postive
CREB1	AC008906.1	0.774539375	4.20E-108	postive
RFXAP	AC008906.1	0.514829365	1.52E-37	postive
UBR1	AC008906.1	0.659150832	5.57E-68	postive
ZC3HAV1	AC008906.1	0.586565397	9.18E-51	postive
IL15	AC008906.1	0.653038328	2.40E-66	postive
CYLD	AC008906.1	0.687835154	3.44E-76	postive
EIF2AK2	AC008906.1	0.579501203	2.59E-49	postive
MAPK8	AC008906.1	0.513525965	2.48E-37	postive
TLR1	AC008906.1	0.509872078	9.63E-37	postive
LMBR1	AC008906.1	0.548607643	2.27E-43	postive
IREB2	AC008906.1	0.585034941	1.91E-50	postive
DDX17	AC008906.1	0.652249026	3.87E-66	postive
PIK3CG	AC008906.1	0.594768033	1.72E-52	postive
JAK2	AC008906.1	0.704206787	2.56E-81	postive
TXK	AC008906.1	0.60949902	1.01E-55	postive
NFAT5	AC008906.1	0.851113332	2.79E-151	postive
MALT1	AC008906.1	0.550708948	9.34E-44	postive
PIK3R1	AC008906.1	0.665985932	7.46E-70	postive

PIK3CA	AC008906.1	0.674425181	3.09E-72	postive
PIK3CB	AC008906.1	0.544728958	1.14E-42	postive
AKT3	AC008906.1	0.552342925	4.67E-44	postive
RASGRP3	AC008906.1	0.575713442	1.50E-48	postive
IGHD6-6	AC008906.1	0.523042203	6.69E-39	postive
PLXNC1	AC008906.1	0.570377199	1.71E-47	postive
GNRH1	AC008906.1	0.756670577	1.95E-100	postive
IL6ST	AC008906.1	0.623494663	5.89E-59	postive
IL7	AC008906.1	0.517283125	6.04E-38	postive
RABEP1	AC008906.1	0.565156721	1.78E-46	postive
ACVR2A	AC008906.1	0.61553961	4.24E-57	postive
ANGPTL1	AC008906.1	0.762977755	4.59E-103	postive
BMPR1A	AC008906.1	0.502316217	1.51E-35	postive
BMPR2	AC008906.1	0.689556638	1.03E-76	postive
CRLF3	AC008906.1	0.659820422	3.67E-68	postive
LIFR	AC008906.1	0.505342504	5.06E-36	postive
NR1D2	AC008906.1	0.50033524	3.08E-35	postive
NR2C2	AC008906.1	0.730467261	2.53E-90	postive
RORA	AC008906.1	0.81930595	7.33E-131	postive
SOS1	AC008906.1	0.687106917	5.71E-76	postive
SOS2	AC008906.1	0.581452539	1.04E-49	postive
BRAF	AC008906.1	0.81988672	3.38E-131	postive
PTPRC	AC008906.1	0.520360077	1.87E-38	postive
ITK	AC008906.1	0.586116927	1.14E-50	postive
CD28	AC008906.1	0.549263002	1.72E-43	postive
CBL	AC008906.1	0.641599873	2.18E-63	postive
CBLB	AC008906.1	0.732933361	3.18E-91	postive
RASGRP1	AC008906.1	0.56869394	3.66E-47	postive
PDK1	AC008906.1	0.575106034	1.98E-48	postive
TRAJ1	AC008906.1	0.808360872	9.52E-125	postive
TRAJ2	AC008906.1	0.801717105	3.17E-121	postive
TRAJ3	AC008906.1	0.867406599	1.09E-163	postive
TRAJ5	AC008906.1	0.825631021	1.38E-134	postive
TRAJ6	AC008906.1	0.848852582	1.12E-149	postive
TRAJ8	AC008906.1	0.82058302	1.33E-131	postive
TRAJ10	AC008906.1	0.818573681	1.94E-130	postive
TRAJ12	AC008906.1	0.780817295	5.76E-111	postive
TRAJ13	AC008906.1	0.83227634	1.15E-138	postive
TRAJ14	AC008906.1	0.842025323	5.46E-145	postive
TRAJ16	AC008906.1	0.830855597	8.84E-138	postive
TRAJ17	AC008906.1	0.803569209	3.41E-122	postive
TRAJ18	AC008906.1	0.798492588	1.45E-119	postive
TRAJ21	AC008906.1	0.862226539	1.42E-159	postive
TRAJ31	AC008906.1	0.850853105	4.28E-151	postive
TRAJ37	AC008906.1	0.834347176	5.64E-140	postive
TRAJ38	AC008906.1	0.89396993	6.08E-188	postive
TRAJ39	AC008906.1	0.867535116	8.57E-164	postive
PSMD2	AC099850.4	0.560580598	1.34E-45	postive
PSMD11	AC099850.4	0.535625313	4.71E-41	postive
PSME3	AC099850.4	0.533214889	1.24E-40	postive
ELAVL1	AC099850.4	0.504855412	6.04E-36	postive
BIRC5	AC099850.4	0.732890198	3.30E-91	postive
PAK2	AC099850.4	0.553396084	2.98E-44	postive
HSPA2	ERVH48-1	0.559653063	2.00E-45	postive
TGFBR3	ERVH48-1	0.564676562	2.20E-46	postive
RFXAP	AC005253.1	0.512494752	3.64E-37	postive
NFKBIZ	AC005253.1	0.542961808	2.38E-42	postive
DDX17	AC005253.1	0.623952322	4.59E-59	postive
IRF9	AC005253.1	0.563671438	3.43E-46	postive
NFAT5	AC005253.1	0.502999257	1.18E-35	postive
LTB4R2	AC005253.1	0.588029894	4.55E-51	postive
GNRH1	AC005253.1	0.653509813	1.80E-66	postive
NR2C1	AC005253.1	0.545489607	8.34E-43	postive
NR2C2	AC005253.1	0.668132138	1.88E-70	postive
BRAF	AC005253.1	0.519165459	2.95E-38	postive

TRAJ1	AC005253.1	0.502500862	1.42E-35	postive
TRAJ3	AC005253.1	0.51210093	4.22E-37	postive
CREB1	AC005479.2	0.575492146	1.66E-48	postive
CYLD	AC005479.2	0.546488064	5.50E-43	postive
DDX17	AC005479.2	0.570721453	1.46E-47	postive
NFAT5	AC005479.2	0.707817594	1.70E-82	postive
PIK3R1	AC005479.2	0.588434787	3.74E-51	postive
GNRH1	AC005479.2	0.556913069	6.58E-45	postive
IL6ST	AC005479.2	0.661141667	1.60E-68	postive
ANGPTL1	AC005479.2	0.568717809	3.62E-47	postive
BMPR2	AC005479.2	0.558908846	2.77E-45	postive
NR2C2	AC005479.2	0.534474575	7.48E-41	postive
RORA	AC005479.2	0.780210287	1.10E-110	postive
BRAF	AC005479.2	0.654907475	7.66E-67	postive
ITK	AC005479.2	0.509961919	9.32E-37	postive
CBLB	AC005479.2	0.503480198	9.95E-36	postive
TRAJ1	AC005479.2	0.577750865	5.84E-49	postive
TRAJ2	AC005479.2	0.590060069	1.71E-51	postive
TRAJ3	AC005479.2	0.600994262	7.74E-54	postive
TRAJ5	AC005479.2	0.700911813	2.94E-80	postive
TRAJ6	AC005479.2	0.614751973	6.43E-57	postive
TRAJ8	AC005479.2	0.620588532	2.85E-58	postive
TRAJ10	AC005479.2	0.62303524	7.57E-59	postive
TRAJ12	AC005479.2	0.579531655	2.55E-49	postive
TRAJ13	AC005479.2	0.677624465	3.68E-73	postive
TRAJ14	AC005479.2	0.624967111	2.64E-59	postive
TRAJ16	AC005479.2	0.636593805	3.92E-62	postive
TRAJ17	AC005479.2	0.61434141	7.99E-57	postive
TRAJ18	AC005479.2	0.63229976	4.48E-61	postive
TRAJ21	AC005479.2	0.689252132	1.28E-76	postive
TRAJ31	AC005479.2	0.708914569	7.39E-83	postive
TRAJ37	AC005479.2	0.714014916	1.46E-84	postive
TRAJ38	AC005479.2	0.687200016	5.36E-76	postive
TRAJ39	AC005479.2	0.700481082	4.04E-80	postive
CREB1	AC025287.3	0.67417498	3.65E-72	postive
CIITA	AC025287.3	0.506152188	3.77E-36	postive
RFXAP	AC025287.3	0.528678295	7.46E-40	postive
UBR1	AC025287.3	0.567954217	5.10E-47	postive
IL15	AC025287.3	0.528913436	6.80E-40	postive
CYLD	AC025287.3	0.656188875	3.49E-67	postive
DDX17	AC025287.3	0.669887157	6.04E-71	postive
JAK2	AC025287.3	0.570918076	1.34E-47	postive
TXK	AC025287.3	0.515886105	1.02E-37	postive
NFAT5	AC025287.3	0.851434293	1.64E-151	postive
NFATC3	AC025287.3	0.600115958	1.20E-53	postive
MALT1	AC025287.3	0.510129847	8.76E-37	postive
PIK3R1	AC025287.3	0.540972686	5.38E-42	postive
PIK3CA	AC025287.3	0.574109105	3.13E-48	postive
LTB4R2	AC025287.3	0.563721754	3.36E-46	postive
GNRH1	AC025287.3	0.764861123	7.25E-104	postive
IL6ST	AC025287.3	0.514129461	1.98E-37	postive
RABEP1	AC025287.3	0.532813756	1.45E-40	postive
ACVR2A	AC025287.3	0.561945951	7.35E-46	postive
ANGPTL1	AC025287.3	0.636945651	3.20E-62	postive
BMPR2	AC025287.3	0.569218052	2.89E-47	postive
CRLF3	AC025287.3	0.565212973	1.73E-46	postive
NR2C1	AC025287.3	0.523783817	5.02E-39	postive
NR2C2	AC025287.3	0.704669042	1.82E-81	postive
RORA	AC025287.3	0.748629889	3.36E-97	postive
SOS1	AC025287.3	0.581636833	9.51E-50	postive
SOS2	AC025287.3	0.538307098	1.59E-41	postive
BRAF	AC025287.3	0.656914991	2.23E-67	postive
CBL	AC025287.3	0.601277854	6.71E-54	postive
CBLB	AC025287.3	0.584966083	1.97E-50	postive
TRAJ1	AC025287.3	0.735147759	4.84E-92	postive

TRAJ2	AC025287.3	0.696711394	6.30E-79	postive
TRAJ3	AC025287.3	0.784098728	1.68E-112	postive
TRAJ5	AC025287.3	0.69845892	1.77E-79	postive
TRAJ6	AC025287.3	0.772509061	3.39E-107	postive
TRAJ8	AC025287.3	0.698500296	1.72E-79	postive
TRAJ10	AC025287.3	0.779343217	2.76E-110	postive
TRAJ12	AC025287.3	0.647516372	6.66E-65	postive
TRAJ13	AC025287.3	0.732311168	5.37E-91	postive
TRAJ14	AC025287.3	0.706404367	4.94E-82	postive
TRAJ16	AC025287.3	0.744602782	1.26E-95	postive
TRAJ17	AC025287.3	0.722798269	1.38E-87	postive
TRAJ18	AC025287.3	0.718008374	6.36E-86	postive
TRAJ21	AC025287.3	0.754580782	1.39E-99	postive
TRAJ31	AC025287.3	0.733687443	1.68E-91	postive
TRAJ37	AC025287.3	0.764723387	8.31E-104	postive
TRAJ38	AC025287.3	0.768788121	1.47E-105	postive
TRAJ39	AC025287.3	0.762043397	1.14E-102	postive
CREB1	AL157394.1	0.701843356	1.48E-80	postive
UBR1	AL157394.1	0.588398107	3.81E-51	postive
IL15	AL157394.1	0.679711987	9.05E-74	postive
CYLD	AL157394.1	0.67165085	1.91E-71	postive
DDX17	AL157394.1	0.548157481	2.74E-43	postive
PIK3CG	AL157394.1	0.540429778	6.71E-42	postive
JAK2	AL157394.1	0.671157517	2.64E-71	postive
TXK	AL157394.1	0.573585465	3.98E-48	postive
NFAT5	AL157394.1	0.832767873	5.63E-139	postive
NFATC3	AL157394.1	0.531638582	2.32E-40	postive
MALT1	AL157394.1	0.511889507	4.56E-37	postive
PIK3R1	AL157394.1	0.627650886	6.01E-60	postive
PIK3CA	AL157394.1	0.60215656	4.31E-54	postive
RASGRP3	AL157394.1	0.537449848	2.26E-41	postive
IGHD6-6	AL157394.1	0.507989798	1.93E-36	postive
PLXNC1	AL157394.1	0.53653123	3.27E-41	postive
GNRH1	AL157394.1	0.706769886	3.75E-82	postive
IL6ST	AL157394.1	0.592412395	5.44E-52	postive
IL7	AL157394.1	0.557267258	5.65E-45	postive
ACVR2A	AL157394.1	0.549100866	1.84E-43	postive
ANGPTL1	AL157394.1	0.693539958	6.15E-78	postive
BMPR2	AL157394.1	0.655389538	5.70E-67	postive
CRLF3	AL157394.1	0.602906003	2.95E-54	postive
IL18R1	AL157394.1	0.51007829	8.92E-37	postive
NR2C2	AL157394.1	0.604021196	1.67E-54	postive
RORA	AL157394.1	0.788702365	1.06E-114	postive
SOS1	AL157394.1	0.537874353	1.90E-41	postive
BRAF	AL157394.1	0.683795228	5.63E-75	postive
FAS	AL157394.1	0.55503623	1.48E-44	postive
PTPRC	AL157394.1	0.500147982	3.30E-35	postive
ITK	AL157394.1	0.540506552	6.51E-42	postive
CD28	AL157394.1	0.536984943	2.72E-41	postive
CBL	AL157394.1	0.5511312	7.82E-44	postive
CBLB	AL157394.1	0.636323701	4.57E-62	postive
RASGRP1	AL157394.1	0.546282886	6.00E-43	postive
TRAJ1	AL157394.1	0.717729458	7.93E-86	postive
TRAJ2	AL157394.1	0.728325505	1.50E-89	postive
TRAJ3	AL157394.1	0.806266811	1.27E-123	postive
TRAJ5	AL157394.1	0.72804928	1.89E-89	postive
TRAJ6	AL157394.1	0.797897225	2.92E-119	postive
TRAJ8	AL157394.1	0.692289566	1.50E-77	postive
TRAJ10	AL157394.1	0.803712199	2.87E-122	postive
TRAJ12	AL157394.1	0.73368468	1.68E-91	postive
TRAJ13	AL157394.1	0.729982163	3.79E-90	postive
TRAJ14	AL157394.1	0.738140116	3.69E-93	postive
TRAJ16	AL157394.1	0.757220688	1.16E-100	postive
TRAJ17	AL157394.1	0.725761668	1.24E-88	postive
TRAJ18	AL157394.1	0.804872824	7.00E-123	postive

TRAJ21	AL157394.1	0.78654105	1.16E-113	postive
TRAJ31	AL157394.1	0.787758692	3.03E-114	postive
TRAJ37	AL157394.1	0.801748577	3.05E-121	postive
TRAJ38	AL157394.1	0.818311772	2.74E-130	postive
TRAJ39	AL157394.1	0.788944252	8.12E-115	postive
CREB1	AL157402.2	0.731870451	7.79E-91	postive
UBR1	AL157402.2	0.556916636	6.57E-45	postive
ZC3HAV1	AL157402.2	0.552262739	4.83E-44	postive
IL15	AL157402.2	0.612283017	2.36E-56	postive
CYLD	AL157402.2	0.596528527	7.19E-53	postive
EIF2AK2	AL157402.2	0.550168513	1.17E-43	postive
MAPK8	AL157402.2	0.520857993	1.55E-38	postive
LMBR1	AL157402.2	0.56929929	2.78E-47	postive
LIMS1	AL157402.2	0.50705986	2.71E-36	postive
IREB2	AL157402.2	0.544855295	1.09E-42	postive
DDX17	AL157402.2	0.523799392	4.99E-39	postive
PIK3CG	AL157402.2	0.545551505	8.13E-43	postive
JAK2	AL157402.2	0.625768564	1.70E-59	postive
TXK	AL157402.2	0.538977909	1.21E-41	postive
NFAT5	AL157402.2	0.785536261	3.51E-113	postive
MALT1	AL157402.2	0.531673118	2.28E-40	postive
PIK3R1	AL157402.2	0.639999582	5.52E-63	postive
PIK3CA	AL157402.2	0.693474083	6.45E-78	postive
PIK3CB	AL157402.2	0.52841499	8.27E-40	postive
AKT3	AL157402.2	0.543651471	1.79E-42	postive
RASGRP3	AL157402.2	0.526507603	1.74E-39	postive
PLXNC1	AL157402.2	0.50752986	2.28E-36	postive
GNRH1	AL157402.2	0.5943396	2.12E-52	postive
IL6ST	AL157402.2	0.635716704	6.47E-62	postive
ACVR2A	AL157402.2	0.566823366	8.46E-47	postive
ANGPTL1	AL157402.2	0.808882206	4.97E-125	postive
BMPR2	AL157402.2	0.672195897	1.34E-71	postive
CRLF3	AL157402.2	0.623166713	7.05E-59	postive
NR2C2	AL157402.2	0.597930171	3.59E-53	postive
RORA	AL157402.2	0.817799248	5.38E-130	postive
SOS1	AL157402.2	0.655043505	7.05E-67	postive
SOS2	AL157402.2	0.577186956	7.58E-49	postive
BRAF	AL157402.2	0.790930017	8.77E-116	postive
ITK	AL157402.2	0.589340199	2.42E-51	postive
CD28	AL157402.2	0.530020684	4.39E-40	postive
CBL	AL157402.2	0.604635299	1.23E-54	postive
CBLB	AL157402.2	0.710075379	3.05E-83	postive
RASGRP1	AL157402.2	0.514147548	1.96E-37	postive
PDK1	AL157402.2	0.571172222	1.19E-47	postive
TRAJ1	AL157402.2	0.758581172	3.18E-101	postive
TRAJ2	AL157402.2	0.734701393	7.08E-92	postive
TRAJ3	AL157402.2	0.803544615	3.51E-122	postive
TRAJ5	AL157402.2	0.88567185	1.00E-179	postive
TRAJ6	AL157402.2	0.812169427	7.89E-127	postive
TRAJ8	AL157402.2	0.831062526	6.57E-138	postive
TRAJ10	AL157402.2	0.810457323	6.90E-126	postive
TRAJ12	AL157402.2	0.739798944	8.74E-94	postive
TRAJ13	AL157402.2	0.87851393	3.91E-173	postive
TRAJ14	AL157402.2	0.854223581	1.56E-153	postive
TRAJ16	AL157402.2	0.832436699	9.09E-139	postive
TRAJ17	AL157402.2	0.837359358	6.54E-142	postive
TRAJ18	AL157402.2	0.780374629	9.23E-111	postive
TRAJ21	AL157402.2	0.901047261	1.65E-195	postive
TRAJ31	AL157402.2	0.886382549	2.10E-180	postive
TRAJ37	AL157402.2	0.841300343	1.67E-144	postive
TRAJ38	AL157402.2	0.889192926	3.93E-183	postive
TRAJ39	AL157402.2	0.892577169	1.62E-186	postive
PSMD3	AC073508.3	0.705513982	9.64E-82	postive
CREB1	AC139887.2	0.589809013	1.93E-51	postive
RFXAP	AC139887.2	0.512400116	3.77E-37	postive

UBR1	AC139887.2	0.596590749	6.98E-53	postive
ZC3HAV1L	AC139887.2	0.578838688	3.52E-49	postive
MAPK8	AC139887.2	0.513984956	2.09E-37	postive
IREB2	AC139887.2	0.556039808	9.60E-45	postive
DDX17	AC139887.2	0.611888369	2.90E-56	postive
NFAT5	AC139887.2	0.680103035	6.95E-74	postive
GNRH1	AC139887.2	0.678188399	2.52E-73	postive
RABEP1	AC139887.2	0.517903321	4.77E-38	postive
ACVR2A	AC139887.2	0.528979822	6.62E-40	postive
ANGPTL1	AC139887.2	0.508981122	1.34E-36	postive
NR2C2	AC139887.2	0.694812125	2.48E-78	postive
RORA	AC139887.2	0.57133252	1.11E-47	postive
SOS1	AC139887.2	0.585191163	1.77E-50	postive
SOS2	AC139887.2	0.50260475	1.36E-35	postive
BRAF	AC139887.2	0.636071592	5.28E-62	postive
TRAJ1	AC139887.2	0.609565799	9.71E-56	postive
TRAJ2	AC139887.2	0.587311611	6.42E-51	postive
TRAJ3	AC139887.2	0.663097766	4.68E-69	postive
TRAJ5	AC139887.2	0.563495765	3.71E-46	postive
TRAJ6	AC139887.2	0.641695994	2.06E-63	postive
TRAJ8	AC139887.2	0.616042802	3.25E-57	postive
TRAJ10	AC139887.2	0.604620126	1.23E-54	postive
TRAJ12	AC139887.2	0.54310432	2.24E-42	postive
TRAJ13	AC139887.2	0.5911813	9.92E-52	postive
TRAJ14	AC139887.2	0.601688354	5.46E-54	postive
TRAJ16	AC139887.2	0.596308204	8.02E-53	postive
TRAJ17	AC139887.2	0.574862569	2.22E-48	postive
TRAJ18	AC139887.2	0.527441054	1.21E-39	postive
TRAJ21	AC139887.2	0.605516436	7.81E-55	postive
TRAJ31	AC139887.2	0.579618299	2.45E-49	postive
TRAJ37	AC139887.2	0.60306682	2.72E-54	postive
TRAJ38	AC139887.2	0.633985236	1.73E-61	postive
TRAJ39	AC139887.2	0.62724773	7.51E-60	postive
CREB1	AL359697.1	0.712807671	3.72E-84	postive
UBR1	AL359697.1	0.554275175	2.05E-44	postive
ZC3HAV1	AL359697.1	0.518495346	3.81E-38	postive
EIF2AK2	AL359697.1	0.535764702	4.45E-41	postive
MAPK8	AL359697.1	0.543636826	1.80E-42	postive
LMBR1	AL359697.1	0.521784455	1.09E-38	postive
IREB2	AL359697.1	0.549633686	1.47E-43	postive
DDX17	AL359697.1	0.519457457	2.64E-38	postive
JAK2	AL359697.1	0.557064672	6.17E-45	postive
TXK	AL359697.1	0.501208595	2.25E-35	postive
NFAT5	AL359697.1	0.726683377	5.82E-89	postive
PIK3R1	AL359697.1	0.519792449	2.33E-38	postive
PIK3CA	AL359697.1	0.63702874	3.05E-62	postive
GNRH1	AL359697.1	0.70080451	3.19E-80	postive
IL6ST	AL359697.1	0.538167683	1.69E-41	postive
ACVR2A	AL359697.1	0.630591552	1.17E-60	postive
ANGPTL1	AL359697.1	0.702021554	1.30E-80	postive
BMPR1A	AL359697.1	0.546183795	6.25E-43	postive
BMPR2	AL359697.1	0.678695224	1.80E-73	postive
CRLF3	AL359697.1	0.609600147	9.54E-56	postive
NR2C2	AL359697.1	0.633524869	2.25E-61	postive
RORA	AL359697.1	0.721973978	2.69E-87	postive
SOS1	AL359697.1	0.659100923	5.74E-68	postive
SOS2	AL359697.1	0.519696623	2.41E-38	postive
BRAF	AL359697.1	0.73544929	3.74E-92	postive
CBL	AL359697.1	0.507259187	2.52E-36	postive
CBLB	AL359697.1	0.661290672	1.46E-68	postive
PDK1	AL359697.1	0.519141019	2.98E-38	postive
TRAJ1	AL359697.1	0.682991899	9.75E-75	postive
TRAJ2	AL359697.1	0.604002881	1.69E-54	postive
TRAJ3	AL359697.1	0.660118402	3.04E-68	postive
TRAJ5	AL359697.1	0.76522496	5.07E-104	postive

TRAJ6	AL359697.1	0.716281621	2.48E-85	postive
TRAJ8	AL359697.1	0.726562859	6.43E-89	postive
TRAJ10	AL359697.1	0.697213263	4.38E-79	postive
TRAJ12	AL359697.1	0.656736526	2.49E-67	postive
TRAJ13	AL359697.1	0.744734054	1.12E-95	postive
TRAJ14	AL359697.1	0.710778647	1.78E-83	postive
TRAJ16	AL359697.1	0.736475097	1.55E-92	postive
TRAJ17	AL359697.1	0.707544599	2.09E-82	postive
TRAJ18	AL359697.1	0.610558625	5.80E-56	postive
TRAJ21	AL359697.1	0.739163428	1.52E-93	postive
TRAJ31	AL359697.1	0.797735215	3.53E-119	postive
TRAJ37	AL359697.1	0.711778543	8.24E-84	postive
TRAJ38	AL359697.1	0.785611412	3.23E-113	postive
TRAJ39	AL359697.1	0.753786301	2.92E-99	postive
CREB1	AC009318.3	0.69175287	2.19E-77	postive
UBR1	AC009318.3	0.557249064	5.69E-45	postive
ZC3HAV1	AC009318.3	0.551245753	7.44E-44	postive
IL15	AC009318.3	0.523624649	5.34E-39	postive
CYLD	AC009318.3	0.632446156	4.13E-61	postive
EIF2AK2	AC009318.3	0.572706968	5.94E-48	postive
IREB2	AC009318.3	0.537640392	2.09E-41	postive
DDX17	AC009318.3	0.537548916	2.17E-41	postive
JAK2	AC009318.3	0.625453672	2.02E-59	postive
TXK	AC009318.3	0.513760664	2.27E-37	postive
NFAT5	AC009318.3	0.732732432	3.77E-91	postive
PIK3R1	AC009318.3	0.588351874	3.90E-51	postive
PIK3CA	AC009318.3	0.660732328	2.07E-68	postive
PIK3CB	AC009318.3	0.521011675	1.46E-38	postive
RASGRP3	AC009318.3	0.520194082	2.00E-38	postive
PLXNC1	AC009318.3	0.507847216	2.03E-36	postive
GNRH1	AC009318.3	0.583352903	4.23E-50	postive
IL6ST	AC009318.3	0.563146466	4.33E-46	postive
ACVR2A	AC009318.3	0.570634683	1.52E-47	postive
ANGPTL1	AC009318.3	0.631186981	8.38E-61	postive
BMPR2	AC009318.3	0.655937975	4.07E-67	postive
CRLF3	AC009318.3	0.512507997	3.62E-37	postive
NR1D2	AC009318.3	0.514211498	1.92E-37	postive
NR2C2	AC009318.3	0.619415819	5.36E-58	postive
RORA	AC009318.3	0.74800478	5.92E-97	postive
SOS1	AC009318.3	0.640746689	3.58E-63	postive
SOS2	AC009318.3	0.504228564	7.59E-36	postive
BRAF	AC009318.3	0.685441515	1.81E-75	postive
ITK	AC009318.3	0.503900229	8.54E-36	postive
CBL	AC009318.3	0.536461206	3.36E-41	postive
CBLB	AC009318.3	0.645908802	1.73E-64	postive
TRAJ1	AC009318.3	0.69593247	1.11E-78	postive
TRAJ2	AC009318.3	0.674991573	2.13E-72	postive
TRAJ3	AC009318.3	0.711865821	7.71E-84	postive
TRAJ5	AC009318.3	0.741982776	1.29E-94	postive
TRAJ6	AC009318.3	0.721908268	2.83E-87	postive
TRAJ8	AC009318.3	0.696497125	7.36E-79	postive
TRAJ10	AC009318.3	0.702552208	8.77E-81	postive
TRAJ12	AC009318.3	0.715276271	5.46E-85	postive
TRAJ13	AC009318.3	0.71250887	4.69E-84	postive
TRAJ14	AC009318.3	0.722282206	2.09E-87	postive
TRAJ16	AC009318.3	0.743890155	2.38E-95	postive
TRAJ17	AC009318.3	0.712462961	4.86E-84	postive
TRAJ18	AC009318.3	0.726094614	9.44E-89	postive
TRAJ21	AC009318.3	0.746801826	1.76E-96	postive
TRAJ31	AC009318.3	0.770449306	2.75E-106	postive
TRAJ37	AC009318.3	0.731970001	7.16E-91	postive
TRAJ38	AC009318.3	0.788715048	1.05E-114	postive
TRAJ39	AC009318.3	0.743799804	2.58E-95	postive
CREB1	LINC01355	0.741677645	1.68E-94	postive
RFXAP	LINC01355	0.569731353	2.29E-47	postive

UBR1	LINC01355	0.584242053	2.78E-50	postive
ZC3HAV1	LINC01355	0.536305732	3.58E-41	postive
ZC3HAV1L	LINC01355	0.52004413	2.11E-38	postive
IL15	LINC01355	0.550740809	9.22E-44	postive
CYLD	LINC01355	0.583120625	4.72E-50	postive
EIF2AK2	LINC01355	0.536374532	3.48E-41	postive
MAPK8	LINC01355	0.575850154	1.41E-48	postive
NFKBIZ	LINC01355	0.54559082	8.00E-43	postive
LMBR1	LINC01355	0.50741112	2.38E-36	postive
IREB2	LINC01355	0.571586562	9.89E-48	postive
DDX17	LINC01355	0.69538133	1.64E-78	postive
JAK2	LINC01355	0.59113914	1.01E-51	postive
TXK	LINC01355	0.557490336	5.13E-45	postive
NFAT5	LINC01355	0.817209746	1.17E-129	postive
MALT1	LINC01355	0.514940696	1.46E-37	postive
PIK3R1	LINC01355	0.565382738	1.61E-46	postive
PIK3CA	LINC01355	0.622701899	9.08E-59	postive
LTB4R2	LINC01355	0.56754658	6.12E-47	postive
GNRH1	LINC01355	0.8097054	1.78E-125	postive
IL6ST	LINC01355	0.553145072	3.32E-44	postive
RABEP1	LINC01355	0.528089292	9.39E-40	postive
ACVR2A	LINC01355	0.654400041	1.04E-66	postive
ANGPTL1	LINC01355	0.706898888	3.40E-82	postive
BMPR1A	LINC01355	0.530607284	3.48E-40	postive
BMPR2	LINC01355	0.649485145	2.05E-65	postive
CRLF3	LINC01355	0.606588756	4.51E-55	postive
NR2C1	LINC01355	0.606511999	4.69E-55	postive
NR2C2	LINC01355	0.76747883	5.43E-105	postive
RORA	LINC01355	0.799254608	5.92E-120	postive
SOS1	LINC01355	0.684022688	4.81E-75	postive
SOS2	LINC01355	0.573988275	3.31E-48	postive
BRAF	LINC01355	0.779706681	1.88E-110	postive
CBL	LINC01355	0.573499218	4.14E-48	postive
CBLB	LINC01355	0.716498828	2.09E-85	postive
PDK1	LINC01355	0.567260442	6.96E-47	postive
TRAJ1	LINC01355	0.713694405	1.87E-84	postive
TRAJ2	LINC01355	0.718252249	5.25E-86	postive
TRAJ3	LINC01355	0.749851127	1.10E-97	postive
TRAJ5	LINC01355	0.751450002	2.54E-98	postive
TRAJ6	LINC01355	0.747797238	7.15E-97	postive
TRAJ8	LINC01355	0.716573105	1.97E-85	postive
TRAJ10	LINC01355	0.735154577	4.81E-92	postive
TRAJ12	LINC01355	0.688509259	2.15E-76	postive
TRAJ13	LINC01355	0.737436187	6.79E-93	postive
TRAJ14	LINC01355	0.724771169	2.78E-88	postive
TRAJ16	LINC01355	0.740477523	4.83E-94	postive
TRAJ17	LINC01355	0.699382038	9.04E-80	postive
TRAJ18	LINC01355	0.682477166	1.39E-74	postive
TRAJ21	LINC01355	0.751033948	3.73E-98	postive
TRAJ31	LINC01355	0.787234645	5.42E-114	postive
TRAJ37	LINC01355	0.770901764	1.74E-106	postive
TRAJ38	LINC01355	0.816118093	4.87E-129	postive
TRAJ39	LINC01355	0.763628738	2.43E-103	postive
CREB1	AC004223.4	0.748783698	2.92E-97	postive
UBR1	AC004223.4	0.602153048	4.32E-54	postive
ZC3HAV1	AC004223.4	0.573646848	3.87E-48	postive
ZC3HAV1L	AC004223.4	0.520710482	1.64E-38	postive
IL15	AC004223.4	0.550333728	1.10E-43	postive
CYLD	AC004223.4	0.582175397	7.38E-50	postive
EIF2AK2	AC004223.4	0.563210947	4.21E-46	postive
MAPK8	AC004223.4	0.557970451	4.17E-45	postive
TLR1	AC004223.4	0.50785907	2.02E-36	postive
LMBR1	AC004223.4	0.562383841	6.06E-46	postive
IREB2	AC004223.4	0.57864926	3.85E-49	postive
DDX17	AC004223.4	0.603862376	1.82E-54	postive

PIK3CG	AC004223.4	0.50723619	2.54E-36	postive
JAK2	AC004223.4	0.633752095	1.97E-61	postive
TXK	AC004223.4	0.548767925	2.12E-43	postive
NFAT5	AC004223.4	0.804478202	1.13E-122	postive
MALT1	AC004223.4	0.506752181	3.03E-36	postive
PIK3R1	AC004223.4	0.554289552	2.04E-44	postive
PIK3CA	AC004223.4	0.675209944	1.84E-72	postive
PIK3CB	AC004223.4	0.505117257	5.50E-36	postive
AKT3	AC004223.4	0.555219036	1.37E-44	postive
RASGRP3	AC004223.4	0.505174967	5.38E-36	postive
GNRH1	AC004223.4	0.738533674	2.63E-93	postive
IL6ST	AC004223.4	0.542940568	2.40E-42	postive
RABEP1	AC004223.4	0.55778762	4.51E-45	postive
ACVR2A	AC004223.4	0.576718731	9.42E-49	postive
ANGPTL1	AC004223.4	0.754803392	1.13E-99	postive
BMPR1A	AC004223.4	0.500054885	3.41E-35	postive
BMPR2	AC004223.4	0.662559305	6.57E-69	postive
CRLF3	AC004223.4	0.673434652	5.94E-72	postive
NR2C1	AC004223.4	0.504205795	7.65E-36	postive
NR2C2	AC004223.4	0.690264766	6.27E-77	postive
RORA	AC004223.4	0.755287209	7.17E-100	postive
SOS1	AC004223.4	0.688653394	1.94E-76	postive
SOS2	AC004223.4	0.565177847	1.76E-46	postive
BRAF	AC004223.4	0.793262862	6.22E-117	postive
CBL	AC004223.4	0.629031954	2.79E-60	postive
CBLB	AC004223.4	0.760636358	4.43E-102	postive
PDK1	AC004223.4	0.565459812	1.55E-46	postive
TRAJ1	AC004223.4	0.722759367	1.43E-87	postive
TRAJ2	AC004223.4	0.726055097	9.75E-89	postive
TRAJ3	AC004223.4	0.757581486	8.22E-101	postive
TRAJ5	AC004223.4	0.753029023	5.90E-99	postive
TRAJ6	AC004223.4	0.778598411	6.07E-110	postive
TRAJ8	AC004223.4	0.745897479	3.96E-96	postive
TRAJ10	AC004223.4	0.739643467	1.00E-93	postive
TRAJ12	AC004223.4	0.674543127	2.86E-72	postive
TRAJ13	AC004223.4	0.768350152	2.27E-105	postive
TRAJ14	AC004223.4	0.760510945	5.00E-102	postive
TRAJ16	AC004223.4	0.768941829	1.26E-105	postive
TRAJ17	AC004223.4	0.707191832	2.73E-82	postive
TRAJ18	AC004223.4	0.677133305	5.11E-73	postive
TRAJ21	AC004223.4	0.780102003	1.23E-110	postive
TRAJ31	AC004223.4	0.786664616	1.02E-113	postive
TRAJ37	AC004223.4	0.751757295	1.91E-98	postive
TRAJ38	AC004223.4	0.837449237	5.72E-142	postive
TRAJ39	AC004223.4	0.782060277	1.52E-111	postive
CREB1	AC127024.5	0.661423441	1.34E-68	postive
RFXAP	AC127024.5	0.533949927	9.22E-41	postive
UBR1	AC127024.5	0.538484846	1.48E-41	postive
MAPK8	AC127024.5	0.560098746	1.65E-45	postive
NFKBIZ	AC127024.5	0.527732628	1.08E-39	postive
IREB2	AC127024.5	0.503526954	9.78E-36	postive
DDX17	AC127024.5	0.647513802	6.67E-65	postive
JAK2	AC127024.5	0.511708623	4.88E-37	postive
NFAT5	AC127024.5	0.723124496	1.06E-87	postive
PIK3CA	AC127024.5	0.542585235	2.77E-42	postive
AKT3	AC127024.5	0.505999377	3.99E-36	postive
GNRH1	AC127024.5	0.788880303	8.72E-115	postive
IL6ST	AC127024.5	0.517943209	4.70E-38	postive
RABEP1	AC127024.5	0.508516683	1.59E-36	postive
ACVR2A	AC127024.5	0.535644051	4.67E-41	postive
ANGPTL1	AC127024.5	0.609469142	1.02E-55	postive
BMPR2	AC127024.5	0.562833616	4.97E-46	postive
CRLF3	AC127024.5	0.670843435	3.24E-71	postive
NR2C1	AC127024.5	0.571554162	1.00E-47	postive
NR2C2	AC127024.5	0.719973188	1.34E-86	postive

RORA	AC127024.5	0.67485897	2.32E-72	postive
SOS1	AC127024.5	0.615176601	5.14E-57	postive
BRAF	AC127024.5	0.735986827	2.36E-92	postive
CBL	AC127024.5	0.525751622	2.34E-39	postive
CBLB	AC127024.5	0.647484444	6.79E-65	postive
PDK1	AC127024.5	0.509831488	9.78E-37	postive
TRAJ1	AC127024.5	0.647501998	6.72E-65	postive
TRAJ2	AC127024.5	0.657970999	1.16E-67	postive
TRAJ3	AC127024.5	0.682964236	9.94E-75	postive
TRAJ5	AC127024.5	0.655686303	4.75E-67	postive
TRAJ6	AC127024.5	0.679729011	8.95E-74	postive
TRAJ8	AC127024.5	0.661273783	1.48E-68	postive
TRAJ10	AC127024.5	0.642237972	1.50E-63	postive
TRAJ12	AC127024.5	0.571626302	9.72E-48	postive
TRAJ13	AC127024.5	0.655834784	4.33E-67	postive
TRAJ14	AC127024.5	0.647903201	5.29E-65	postive
TRAJ16	AC127024.5	0.670408081	4.31E-71	postive
TRAJ17	AC127024.5	0.607286204	3.15E-55	postive
TRAJ18	AC127024.5	0.574484836	2.63E-48	postive
TRAJ21	AC127024.5	0.671185088	2.60E-71	postive
TRAJ31	AC127024.5	0.680858639	4.17E-74	postive
TRAJ37	AC127024.5	0.67548763	1.53E-72	postive
TRAJ38	AC127024.5	0.719269891	2.34E-86	postive
TRAJ39	AC127024.5	0.707526355	2.12E-82	postive
CREB1	AC006059.1	0.82068399	1.16E-131	postive
RFXAP	AC006059.1	0.522134356	9.49E-39	postive
UBR1	AC006059.1	0.691916883	1.95E-77	postive
ZC3HAV1	AC006059.1	0.563658768	3.45E-46	postive
ZC3HAV1L	AC006059.1	0.548286365	2.59E-43	postive
IL15	AC006059.1	0.610195046	7.01E-56	postive
CYLD	AC006059.1	0.622570735	9.75E-59	postive
EIF2AK2	AC006059.1	0.543396349	1.99E-42	postive
MAPK8	AC006059.1	0.578892607	3.43E-49	postive
TLR1	AC006059.1	0.528052532	9.53E-40	postive
LMBR1	AC006059.1	0.613568914	1.20E-56	postive
LIMS1	AC006059.1	0.544231133	1.41E-42	postive
IREB2	AC006059.1	0.643814905	5.95E-64	postive
DDX17	AC006059.1	0.610355483	6.45E-56	postive
PIK3CG	AC006059.1	0.56482635	2.06E-46	postive
JAK2	AC006059.1	0.683934329	5.11E-75	postive
TXK	AC006059.1	0.610326029	6.54E-56	postive
PPP3CB	AC006059.1	0.514890646	1.49E-37	postive
NFAT5	AC006059.1	0.86902757	5.17E-165	postive
MALT1	AC006059.1	0.547288659	3.94E-43	postive
PIK3R1	AC006059.1	0.6533017	2.04E-66	postive
PIK3CA	AC006059.1	0.701700702	1.65E-80	postive
PIK3CB	AC006059.1	0.531368076	2.58E-40	postive
AKT3	AC006059.1	0.563696294	3.40E-46	postive
RASGRP3	AC006059.1	0.544126124	1.47E-42	postive
IGHD4-4	AC006059.1	0.502965943	1.20E-35	postive
PLXNC1	AC006059.1	0.522371826	8.66E-39	postive
GNRH1	AC006059.1	0.786070489	1.95E-113	postive
IL6ST	AC006059.1	0.631422319	7.34E-61	postive
RABEP1	AC006059.1	0.597043747	5.57E-53	postive
ACVR2A	AC006059.1	0.654136831	1.23E-66	postive
ANGPTL1	AC006059.1	0.788673099	1.10E-114	postive
BMPR1A	AC006059.1	0.584220223	2.81E-50	postive
BMPR2	AC006059.1	0.760597627	4.60E-102	postive
CRLF3	AC006059.1	0.688555614	2.08E-76	postive
NR1D2	AC006059.1	0.544924277	1.05E-42	postive
NR2C2	AC006059.1	0.714042324	1.43E-84	postive
NR3C1	AC006059.1	0.502680216	1.33E-35	postive
RORA	AC006059.1	0.840830227	3.43E-144	postive
SOS1	AC006059.1	0.696507346	7.31E-79	postive
SOS2	AC006059.1	0.588309097	3.98E-51	postive

BRAF	AC006059.1	0.812104465	8.57E-127	postive
TEC	AC006059.1	0.509656497	1.04E-36	postive
CBL	AC006059.1	0.623521512	5.81E-59	postive
CBLB	AC006059.1	0.754950069	9.83E-100	postive
RASGRP1	AC006059.1	0.513179411	2.82E-37	postive
PDK1	AC006059.1	0.622225802	1.18E-58	postive
TRAJ1	AC006059.1	0.725861345	1.14E-88	postive
TRAJ2	AC006059.1	0.749645856	1.33E-97	postive
TRAJ3	AC006059.1	0.78715142	5.94E-114	postive
TRAJ5	AC006059.1	0.768369467	2.23E-105	postive
TRAJ6	AC006059.1	0.801984055	2.30E-121	postive
TRAJ8	AC006059.1	0.746485907	2.33E-96	postive
TRAJ10	AC006059.1	0.776074685	8.55E-109	postive
TRAJ12	AC006059.1	0.709128281	6.28E-83	postive
TRAJ13	AC006059.1	0.770315648	3.15E-106	postive
TRAJ14	AC006059.1	0.755016289	9.24E-100	postive
TRAJ16	AC006059.1	0.768203819	2.63E-105	postive
TRAJ17	AC006059.1	0.7163271	2.40E-85	postive
TRAJ18	AC006059.1	0.712533192	4.60E-84	postive
TRAJ21	AC006059.1	0.791701062	3.67E-116	postive
TRAJ31	AC006059.1	0.821394506	4.48E-132	postive
TRAJ37	AC006059.1	0.802245879	1.68E-121	postive
TRAJ38	AC006059.1	0.866462829	6.31E-163	postive
TRAJ39	AC006059.1	0.807698521	2.17E-124	postive
SCG2	DPP10-AS1	0.572899437	5.44E-48	postive
DDX17	AGBL5-IT1	0.519780232	2.34E-38	postive
GNRH1	AGBL5-IT1	0.627824616	5.46E-60	postive
NR2C2	AGBL5-IT1	0.584181539	2.86E-50	postive
IFI30	U62317.2	0.549473081	1.57E-43	postive
IRF3	U62317.2	0.55029792	1.11E-43	postive
TYK2	U62317.2	0.504254434	7.52E-36	postive
IRF9	U62317.2	0.525806138	2.29E-39	postive
TYMP	U62317.2	0.612398829	2.22E-56	postive
TNFRSF14	U62317.2	0.578706003	3.75E-49	postive
CREB1	AC083843.3	0.672683921	9.73E-72	postive
RFXAP	AC083843.3	0.507245164	2.53E-36	postive
UBR1	AC083843.3	0.584868811	2.06E-50	postive
ZC3HAV1	AC083843.3	0.547045451	4.36E-43	postive
CYLD	AC083843.3	0.505135683	5.46E-36	postive
EIF2AK2	AC083843.3	0.550500294	1.02E-43	postive
MAPK8	AC083843.3	0.52399917	4.62E-39	postive
IREB2	AC083843.3	0.543351811	2.02E-42	postive
DDX17	AC083843.3	0.661766994	1.08E-68	postive
PTK2	AC083843.3	0.514089181	2.01E-37	postive
TXK	AC083843.3	0.582326061	6.87E-50	postive
NFAT5	AC083843.3	0.695878506	1.15E-78	postive
PIK3R1	AC083843.3	0.613907108	1.00E-56	postive
PIK3CA	AC083843.3	0.557742731	4.60E-45	postive
AKT3	AC083843.3	0.534328531	7.93E-41	postive
IGHD6-6	AC083843.3	0.520541709	1.75E-38	postive
GNRH1	AC083843.3	0.722159351	2.31E-87	postive
IL6ST	AC083843.3	0.63779903	1.96E-62	postive
RABEP1	AC083843.3	0.510165406	8.64E-37	postive
ACVR2A	AC083843.3	0.569847706	2.17E-47	postive
ANGPTL1	AC083843.3	0.672818714	8.91E-72	postive
BMPR1A	AC083843.3	0.505935108	4.08E-36	postive
BMPR2	AC083843.3	0.650744566	9.62E-66	postive
CRLF3	AC083843.3	0.590234244	1.57E-51	postive
NR2C1	AC083843.3	0.51834519	4.04E-38	postive
NR2C2	AC083843.3	0.715211848	5.74E-85	postive
RORA	AC083843.3	0.730177014	3.22E-90	postive
SOS1	AC083843.3	0.609031242	1.28E-55	postive
SOS2	AC083843.3	0.516658629	7.64E-38	postive
BRAF	AC083843.3	0.818429908	2.34E-130	postive
CBL	AC083843.3	0.533206746	1.24E-40	postive

CBLB	AC083843.3	0.618040217	1.12E-57	postive
RASGRP1	AC083843.3	0.50669963	3.09E-36	postive
TRAJ1	AC083843.3	0.608583942	1.61E-55	postive
TRAJ2	AC083843.3	0.628390954	3.99E-60	postive
TRAJ3	AC083843.3	0.666952745	4.01E-70	postive
TRAJ5	AC083843.3	0.689687255	9.41E-77	postive
TRAJ6	AC083843.3	0.631229545	8.18E-61	postive
TRAJ8	AC083843.3	0.689532266	1.05E-76	postive
TRAJ10	AC083843.3	0.565243737	1.71E-46	postive
TRAJ12	AC083843.3	0.565054291	1.86E-46	postive
TRAJ13	AC083843.3	0.678730365	1.75E-73	postive
TRAJ14	AC083843.3	0.654628413	9.09E-67	postive
TRAJ16	AC083843.3	0.628995286	2.85E-60	postive
TRAJ17	AC083843.3	0.606254978	5.35E-55	postive
TRAJ18	AC083843.3	0.54492351	1.06E-42	postive
TRAJ21	AC083843.3	0.690614191	4.90E-77	postive
TRAJ31	AC083843.3	0.679078808	1.39E-73	postive
TRAJ37	AC083843.3	0.658111268	1.06E-67	postive
TRAJ38	AC083843.3	0.699451077	8.60E-80	postive
TRAJ39	AC083843.3	0.720426909	9.29E-87	postive
CIITA	LINC00893	0.50217331	1.59E-35	postive
DDX17	LINC00893	0.666287038	6.15E-70	postive
IRF9	LINC00893	0.528838282	7.00E-40	postive
NFAT5	LINC00893	0.517603257	5.35E-38	postive
LTB4R2	LINC00893	0.544658442	1.18E-42	postive
GNRH1	LINC00893	0.656646272	2.63E-67	postive
GIPR	LINC00893	0.544204625	1.42E-42	postive
IL11RA	LINC00893	0.514420027	1.77E-37	postive
NR2C2	LINC00893	0.590154169	1.63E-51	postive
RORA	LINC00893	0.508687671	1.49E-36	postive
CREB1	BET1-AS1	0.64181088	1.92E-63	postive
UBR1	BET1-AS1	0.558968281	2.70E-45	postive
ZC3HAV1L	BET1-AS1	0.518161905	4.33E-38	postive
IL15	BET1-AS1	0.512607443	3.49E-37	postive
IREB2	BET1-AS1	0.530601277	3.49E-40	postive
DDX17	BET1-AS1	0.576790479	9.11E-49	postive
TXK	BET1-AS1	0.512799786	3.25E-37	postive
NFAT5	BET1-AS1	0.634152394	1.57E-61	postive
PIK3R1	BET1-AS1	0.572026512	8.10E-48	postive
PIK3CA	BET1-AS1	0.512413067	3.75E-37	postive
IGHD4-4	BET1-AS1	0.577881837	5.49E-49	postive
GNRH1	BET1-AS1	0.674304086	3.35E-72	postive
IL6ST	BET1-AS1	0.585583018	1.47E-50	postive
ACVR2A	BET1-AS1	0.557588322	4.92E-45	postive
ANGPTL1	BET1-AS1	0.615932544	3.44E-57	postive
BMPR2	BET1-AS1	0.598384584	2.86E-53	postive
CRLF3	BET1-AS1	0.507425087	2.37E-36	postive
NR2C2	BET1-AS1	0.68835851	2.39E-76	postive
RORA	BET1-AS1	0.681877911	2.09E-74	postive
SOS1	BET1-AS1	0.570042741	1.99E-47	postive
SOS2	BET1-AS1	0.510781611	6.88E-37	postive
BRAF	BET1-AS1	0.743355456	3.83E-95	postive
TEC	BET1-AS1	0.502574	1.38E-35	postive
CBLB	BET1-AS1	0.543638229	1.80E-42	postive
RASGRP1	BET1-AS1	0.54004175	7.87E-42	postive
TRAJ1	BET1-AS1	0.595819518	1.02E-52	postive
TRAJ2	BET1-AS1	0.502817222	1.26E-35	postive
TRAJ3	BET1-AS1	0.636347056	4.51E-62	postive
TRAJ5	BET1-AS1	0.691053971	3.59E-77	postive
TRAJ6	BET1-AS1	0.602126326	4.38E-54	postive
TRAJ8	BET1-AS1	0.614312881	8.11E-57	postive
TRAJ10	BET1-AS1	0.597767249	3.89E-53	postive
TRAJ12	BET1-AS1	0.602040235	4.57E-54	postive
TRAJ13	BET1-AS1	0.614308206	8.13E-57	postive
TRAJ14	BET1-AS1	0.609496437	1.01E-55	postive

TRAJ16	BET1-AS1	0.574705925	2.38E-48	postive
TRAJ17	BET1-AS1	0.621819602	1.47E-58	postive
TRAJ18	BET1-AS1	0.551561513	6.51E-44	postive
TRAJ21	BET1-AS1	0.645511099	2.19E-64	postive
TRAJ31	BET1-AS1	0.679609777	9.70E-74	postive
TRAJ37	BET1-AS1	0.640271782	4.71E-63	postive
TRAJ38	BET1-AS1	0.659176755	5.48E-68	postive
TRAJ39	BET1-AS1	0.681313043	3.06E-74	postive
CREB1	AC009041.3	0.673914067	4.33E-72	postive
UBR1	AC009041.3	0.540688356	6.04E-42	postive
IFNAR1	AC009041.3	0.503949207	8.39E-36	postive
CYLD	AC009041.3	0.587668502	5.41E-51	postive
NFAT5	AC009041.3	0.698340654	1.93E-79	postive
NFATC3	AC009041.3	0.527033224	1.42E-39	postive
PIK3R1	AC009041.3	0.591911847	6.95E-52	postive
PIK3CA	AC009041.3	0.600032234	1.26E-53	postive
GNRH1	AC009041.3	0.54588725	7.07E-43	postive
IL6ST	AC009041.3	0.567860522	5.32E-47	postive
ACVR2A	AC009041.3	0.561971752	7.26E-46	postive
ANGPTL1	AC009041.3	0.54717925	4.12E-43	postive
BMPR2	AC009041.3	0.622025579	1.31E-58	postive
NR1D2	AC009041.3	0.520689341	1.65E-38	postive
NR2C2	AC009041.3	0.545125023	9.71E-43	postive
RORA	AC009041.3	0.692924893	9.54E-78	postive
SOS1	AC009041.3	0.530408551	3.77E-40	postive
SOS2	AC009041.3	0.565994429	1.22E-46	postive
BRAF	AC009041.3	0.676191057	9.58E-73	postive
CBLB	AC009041.3	0.559091489	2.56E-45	postive
TRAJ1	AC009041.3	0.590208402	1.59E-51	postive
TRAJ2	AC009041.3	0.632013224	5.27E-61	postive
TRAJ3	AC009041.3	0.653949268	1.38E-66	postive
TRAJ5	AC009041.3	0.617070281	1.88E-57	postive
TRAJ6	AC009041.3	0.63628331	4.68E-62	postive
TRAJ8	AC009041.3	0.643814748	5.96E-64	postive
TRAJ10	AC009041.3	0.617578813	1.43E-57	postive
TRAJ12	AC009041.3	0.576122224	1.24E-48	postive
TRAJ13	AC009041.3	0.635689277	6.57E-62	postive
TRAJ14	AC009041.3	0.627024352	8.50E-60	postive
TRAJ16	AC009041.3	0.633373016	2.45E-61	postive
TRAJ17	AC009041.3	0.609823388	8.50E-56	postive
TRAJ18	AC009041.3	0.576914186	8.60E-49	postive
TRAJ21	AC009041.3	0.642380571	1.38E-63	postive
TRAJ31	AC009041.3	0.661616953	1.19E-68	postive
TRAJ37	AC009041.3	0.655555789	5.15E-67	postive
TRAJ38	AC009041.3	0.688306989	2.48E-76	postive
TRAJ39	AC009041.3	0.671455633	2.17E-71	postive
GNRH2	LINC01607	0.534828655	6.49E-41	postive
CREB1	AC127024.4	0.72854074	1.26E-89	postive
RFXAP	AC127024.4	0.555803997	1.06E-44	postive
UBR1	AC127024.4	0.59132077	9.27E-52	postive
ZC3HAV1	AC127024.4	0.555213933	1.37E-44	postive
ZC3HAV1L	AC127024.4	0.566011659	1.22E-46	postive
IL15	AC127024.4	0.520714789	1.64E-38	postive
CYLD	AC127024.4	0.530656324	3.42E-40	postive
EIF2AK2	AC127024.4	0.536448099	3.38E-41	postive
MAPK8	AC127024.4	0.561135574	1.05E-45	postive
NFKBIZ	AC127024.4	0.54083604	5.69E-42	postive
LMBR1	AC127024.4	0.523454732	5.70E-39	postive
IREB2	AC127024.4	0.572672745	6.03E-48	postive
DDX17	AC127024.4	0.656563749	2.77E-67	postive
JAK2	AC127024.4	0.592630438	4.89E-52	postive
TXK	AC127024.4	0.567720933	5.66E-47	postive
NFAT5	AC127024.4	0.755006004	9.33E-100	postive
MALT1	AC127024.4	0.53018793	4.11E-40	postive
PIK3R1	AC127024.4	0.561841766	7.69E-46	postive

PIK3CA	AC127024.4	0.632644615	3.69E-61	postive
AKT3	AC127024.4	0.544296873	1.37E-42	postive
IGHD6-6	AC127024.4	0.516768276	7.33E-38	postive
LTB4R2	AC127024.4	0.552254489	4.85E-44	postive
GNRH1	AC127024.4	0.806669243	7.74E-124	postive
IL6ST	AC127024.4	0.560977937	1.12E-45	postive
RABEP1	AC127024.4	0.557131414	5.99E-45	postive
ACVR2A	AC127024.4	0.585706145	1.38E-50	postive
ANGPTL1	AC127024.4	0.708584153	9.50E-83	postive
BMPR1A	AC127024.4	0.508141422	1.82E-36	postive
BMPR2	AC127024.4	0.63312455	2.82E-61	postive
CRLF3	AC127024.4	0.700861264	3.06E-80	postive
NR2C1	AC127024.4	0.580206759	1.86E-49	postive
NR2C2	AC127024.4	0.772846417	2.40E-107	postive
RORA	AC127024.4	0.732580952	4.28E-91	postive
SOS1	AC127024.4	0.669492069	7.80E-71	postive
SOS2	AC127024.4	0.566047743	1.20E-46	postive
BRAF	AC127024.4	0.797722709	3.59E-119	postive
TEC	AC127024.4	0.517549626	5.46E-38	postive
CBL	AC127024.4	0.603335083	2.37E-54	postive
CBLB	AC127024.4	0.714512917	9.91E-85	postive
RASGRP1	AC127024.4	0.508455046	1.62E-36	postive
PDK1	AC127024.4	0.596217935	8.39E-53	postive
TRAJ1	AC127024.4	0.686708835	7.53E-76	postive
TRAJ2	AC127024.4	0.679593792	9.80E-74	postive
TRAJ3	AC127024.4	0.727079123	4.20E-89	postive
TRAJ5	AC127024.4	0.705074347	1.34E-81	postive
TRAJ6	AC127024.4	0.707956453	1.53E-82	postive
TRAJ8	AC127024.4	0.700782864	3.24E-80	postive
TRAJ10	AC127024.4	0.672148021	1.38E-71	postive
TRAJ12	AC127024.4	0.584295002	2.71E-50	postive
TRAJ13	AC127024.4	0.687056001	5.92E-76	postive
TRAJ14	AC127024.4	0.683053711	9.35E-75	postive
TRAJ16	AC127024.4	0.712036611	6.76E-84	postive
TRAJ17	AC127024.4	0.659424588	4.70E-68	postive
TRAJ18	AC127024.4	0.600383417	1.05E-53	postive
TRAJ21	AC127024.4	0.70948091	4.80E-83	postive
TRAJ31	AC127024.4	0.711964882	7.14E-84	postive
TRAJ37	AC127024.4	0.687898776	3.29E-76	postive
TRAJ38	AC127024.4	0.763569367	2.57E-103	postive
TRAJ39	AC127024.4	0.750135012	8.50E-98	postive
TMSB15A	LINC02550	0.555939352	1.00E-44	postive
MSTN	LINC02550	0.691432704	2.75E-77	postive
CREB1	AC058791.1	0.787705516	3.22E-114	postive
UBR1	AC058791.1	0.640573118	3.96E-63	postive
ZC3HAV1	AC058791.1	0.565656012	1.42E-46	postive
IL15	AC058791.1	0.604212231	1.52E-54	postive
CYLD	AC058791.1	0.612125496	2.56E-56	postive
EIF2AK2	AC058791.1	0.554095883	2.21E-44	postive
MAPK8	AC058791.1	0.587984918	4.65E-51	postive
LMBR1	AC058791.1	0.59302037	4.05E-52	postive
LIMS1	AC058791.1	0.510469776	7.72E-37	postive
IREB2	AC058791.1	0.611854346	2.95E-56	postive
DDX17	AC058791.1	0.583851775	3.34E-50	postive
PIK3CG	AC058791.1	0.532900694	1.40E-40	postive
JAK2	AC058791.1	0.633350421	2.48E-61	postive
TXK	AC058791.1	0.604310949	1.45E-54	postive
NFAT5	AC058791.1	0.839232293	3.91E-143	postive
MALT1	AC058791.1	0.543459136	1.93E-42	postive
PIK3R1	AC058791.1	0.675978027	1.10E-72	postive
PIK3CA	AC058791.1	0.706855293	3.52E-82	postive
PIK3CB	AC058791.1	0.54217889	3.28E-42	postive
AKT3	AC058791.1	0.537838501	1.93E-41	postive
RASGRP3	AC058791.1	0.528562403	7.80E-40	postive
GNRH1	AC058791.1	0.706596696	4.27E-82	postive

IL6ST	AC058791.1	0.666921961	4.09E-70	postive
RABEP1	AC058791.1	0.549770608	1.39E-43	postive
ACVR2A	AC058791.1	0.639189355	8.81E-63	postive
ANGPTL1	AC058791.1	0.814354307	4.80E-128	postive
BMPR1A	AC058791.1	0.562409611	5.99E-46	postive
BMPR2	AC058791.1	0.752351956	1.11E-98	postive
CRLF3	AC058791.1	0.628896005	3.01E-60	postive
LIFR	AC058791.1	0.503913864	8.50E-36	postive
NR1D2	AC058791.1	0.5252354	2.86E-39	postive
NR2C2	AC058791.1	0.662963912	5.09E-69	postive
RORA	AC058791.1	0.878189528	7.60E-173	postive
SOS1	AC058791.1	0.682491748	1.37E-74	postive
SOS2	AC058791.1	0.58468167	2.25E-50	postive
BRAF	AC058791.1	0.828414912	2.83E-136	postive
ITK	AC058791.1	0.55181407	5.85E-44	postive
CD28	AC058791.1	0.504733346	6.32E-36	postive
CBL	AC058791.1	0.587903354	4.84E-51	postive
CBLB	AC058791.1	0.742533155	7.92E-95	postive
RASGRP1	AC058791.1	0.508446337	1.63E-36	postive
PDK1	AC058791.1	0.598456062	2.76E-53	postive
TRAJ1	AC058791.1	0.712507896	4.70E-84	postive
TRAJ2	AC058791.1	0.729453797	5.89E-90	postive
TRAJ3	AC058791.1	0.786089526	1.91E-113	postive
TRAJ5	AC058791.1	0.823336093	3.22E-133	postive
TRAJ6	AC058791.1	0.790900484	9.07E-116	postive
TRAJ8	AC058791.1	0.772274138	4.30E-107	postive
TRAJ10	AC058791.1	0.779044508	3.79E-110	postive
TRAJ12	AC058791.1	0.731919161	7.48E-91	postive
TRAJ13	AC058791.1	0.814257747	5.43E-128	postive
TRAJ14	AC058791.1	0.789055778	7.17E-115	postive
TRAJ16	AC058791.1	0.783789467	2.35E-112	postive
TRAJ17	AC058791.1	0.769526796	6.98E-106	postive
TRAJ18	AC058791.1	0.749768613	1.19E-97	postive
TRAJ21	AC058791.1	0.843012914	1.18E-145	postive
TRAJ31	AC058791.1	0.864868737	1.19E-161	postive
TRAJ37	AC058791.1	0.830491655	1.49E-137	postive
TRAJ38	AC058791.1	0.879636245	3.86E-174	postive
TRAJ39	AC058791.1	0.845050503	4.88E-147	postive
CREB1	AP005131.7	0.781781853	2.05E-111	postive
RFXAP	AP005131.7	0.517547428	5.46E-38	postive
UBR1	AP005131.7	0.629361814	2.32E-60	postive
ZC3HAV1	AP005131.7	0.550941892	8.47E-44	postive
IL15	AP005131.7	0.573659509	3.85E-48	postive
CYLD	AP005131.7	0.654793422	8.21E-67	postive
EIF2AK2	AP005131.7	0.533559109	1.08E-40	postive
MAPK8	AP005131.7	0.558364318	3.51E-45	postive
LMBR1	AP005131.7	0.561441394	9.17E-46	postive
LIMS1	AP005131.7	0.536807007	2.93E-41	postive
IREB2	AP005131.7	0.590294958	1.52E-51	postive
DDX17	AP005131.7	0.601175951	7.07E-54	postive
PIK3CG	AP005131.7	0.56736651	6.63E-47	postive
JAK2	AP005131.7	0.641334684	2.54E-63	postive
TXK	AP005131.7	0.616864881	2.10E-57	postive
NFAT5	AP005131.7	0.868285421	2.10E-164	postive
NFATC3	AP005131.7	0.505669631	4.50E-36	postive
MALT1	AP005131.7	0.563579405	3.58E-46	postive
PIK3R1	AP005131.7	0.703247024	5.24E-81	postive
PIK3CA	AP005131.7	0.717411453	1.02E-85	postive
PIK3CB	AP005131.7	0.517159491	6.33E-38	postive
AKT3	AP005131.7	0.522779514	7.40E-39	postive
RASGRP3	AP005131.7	0.531440488	2.51E-40	postive
IGHD6-6	AP005131.7	0.548063771	2.85E-43	postive
GNRH1	AP005131.7	0.701414785	2.03E-80	postive
IL6ST	AP005131.7	0.70294234	6.57E-81	postive
RABEP1	AP005131.7	0.533660582	1.04E-40	postive

ACVR2A	AP005131.7	0.641222145	2.71E-63	postive
ANGPTL1	AP005131.7	0.774133804	6.39E-108	postive
BMPR1A	AP005131.7	0.545588482	8.01E-43	postive
BMPR2	AP005131.7	0.768398392	2.17E-105	postive
CRLF3	AP005131.7	0.62195876	1.36E-58	postive
NR1D2	AP005131.7	0.540641908	6.16E-42	postive
NR2C2	AP005131.7	0.655663854	4.81E-67	postive
RORA	AP005131.7	0.926653034	1.25E-228	postive
SOS1	AP005131.7	0.657896312	1.21E-67	postive
SOS2	AP005131.7	0.564587	2.29E-46	postive
BRAF	AP005131.7	0.790493391	1.43E-115	postive
ITK	AP005131.7	0.566439275	1.00E-46	postive
CD28	AP005131.7	0.549797713	1.37E-43	postive
CBL	AP005131.7	0.567866141	5.30E-47	postive
CBLB	AP005131.7	0.728693566	1.11E-89	postive
RASGRP1	AP005131.7	0.512099239	4.22E-37	postive
PDK1	AP005131.7	0.582430659	6.54E-50	postive
TRAJ1	AP005131.7	0.696590634	6.88E-79	postive
TRAJ2	AP005131.7	0.742906795	5.69E-95	postive
TRAJ3	AP005131.7	0.779900062	1.53E-110	postive
TRAJ5	AP005131.7	0.794342059	1.81E-117	postive
TRAJ6	AP005131.7	0.77590535	1.02E-108	postive
TRAJ8	AP005131.7	0.748621834	3.38E-97	postive
TRAJ10	AP005131.7	0.775936522	9.87E-109	postive
TRAJ12	AP005131.7	0.69789895	2.67E-79	postive
TRAJ13	AP005131.7	0.783704189	2.58E-112	postive
TRAJ14	AP005131.7	0.749789865	1.17E-97	postive
TRAJ16	AP005131.7	0.797172333	6.82E-119	postive
TRAJ17	AP005131.7	0.757832032	6.48E-101	postive
TRAJ18	AP005131.7	0.763021479	4.39E-103	postive
TRAJ21	AP005131.7	0.819596614	4.98E-131	postive
TRAJ31	AP005131.7	0.849119542	7.26E-150	postive
TRAJ37	AP005131.7	0.833496028	1.95E-139	postive
TRAJ38	AP005131.7	0.866081534	1.28E-162	postive
TRAJ39	AP005131.7	0.843242425	8.28E-146	postive
B2M	PCED1B-AS1	0.570213251	1.84E-47	postive
CD1D	PCED1B-AS1	0.646679953	1.10E-64	postive
CD4	PCED1B-AS1	0.604042143	1.66E-54	postive
CD8A	PCED1B-AS1	0.624438916	3.52E-59	postive
CD8B	PCED1B-AS1	0.553487983	2.87E-44	postive
CTSS	PCED1B-AS1	0.54300477	2.33E-42	postive
FCER1G	PCED1B-AS1	0.55771595	4.65E-45	postive
HLA-DMB	PCED1B-AS1	0.61688696	2.07E-57	postive
HLA-DOB	PCED1B-AS1	0.622318278	1.12E-58	postive
HLA-DPB1	PCED1B-AS1	0.509234935	1.22E-36	postive
HLA-DQA1	PCED1B-AS1	0.532986911	1.35E-40	postive
HLA-DRA	PCED1B-AS1	0.578858892	3.49E-49	postive
IFNG	PCED1B-AS1	0.512365853	3.82E-37	postive
LTA	PCED1B-AS1	0.700479147	4.05E-80	postive
CIITA	PCED1B-AS1	0.528521231	7.93E-40	postive
CXCL13	PCED1B-AS1	0.556261793	8.72E-45	postive
PTGDS	PCED1B-AS1	0.50201852	1.68E-35	postive
APOBEC3G	PCED1B-AS1	0.664914422	1.48E-69	postive
RBP5	PCED1B-AS1	0.60314708	2.61E-54	postive
CYBB	PCED1B-AS1	0.539789468	8.72E-42	postive
IL15	PCED1B-AS1	0.512496115	3.64E-37	postive
TLR7	PCED1B-AS1	0.524710989	3.51E-39	postive
CCL5	PCED1B-AS1	0.567646823	5.85E-47	postive
TLR8	PCED1B-AS1	0.528710305	7.36E-40	postive
CCL4	PCED1B-AS1	0.568265556	4.43E-47	postive
IL7R	PCED1B-AS1	0.523459553	5.69E-39	postive
CD40LG	PCED1B-AS1	0.593342087	3.46E-52	postive
PIK3CG	PCED1B-AS1	0.526867571	1.52E-39	postive
PDCD1	PCED1B-AS1	0.570660708	1.51E-47	postive
FASLG	PCED1B-AS1	0.569862385	2.16E-47	postive

CCL19	PCED1B-AS1	0.594925444	1.59E-52	postive
CCR7	PCED1B-AS1	0.592819741	4.46E-52	postive
CCR5	PCED1B-AS1	0.705807203	7.74E-82	postive
CCL4L2	PCED1B-AS1	0.510214405	8.49E-37	postive
XCL2	PCED1B-AS1	0.545579678	8.03E-43	postive
CXCR4	PCED1B-AS1	0.585447905	1.57E-50	postive
CXCR6	PCED1B-AS1	0.687027019	6.04E-76	postive
CCR4	PCED1B-AS1	0.529672259	5.04E-40	postive
IL10	PCED1B-AS1	0.536807478	2.92E-41	postive
CD86	PCED1B-AS1	0.614042903	9.35E-57	postive
CD79A	PCED1B-AS1	0.667964941	2.09E-70	postive
CD79B	PCED1B-AS1	0.675733856	1.30E-72	postive
BTK	PCED1B-AS1	0.798942402	8.56E-120	postive
VAV1	PCED1B-AS1	0.502427926	1.45E-35	postive
RAC2	PCED1B-AS1	0.648407247	3.92E-65	postive
CD19	PCED1B-AS1	0.65750571	1.55E-67	postive
PIK3R5	PCED1B-AS1	0.555753463	1.09E-44	postive
PIK3CD	PCED1B-AS1	0.514838993	1.52E-37	postive
INPP5D	PCED1B-AS1	0.553353605	3.04E-44	postive
CD22	PCED1B-AS1	0.520124492	2.05E-38	postive
CD72	PCED1B-AS1	0.681527612	2.65E-74	postive
PTPN6	PCED1B-AS1	0.508466505	1.62E-36	postive
FCGR2B	PCED1B-AS1	0.619404345	5.39E-58	postive
RASGRP3	PCED1B-AS1	0.565142584	1.79E-46	postive
PLCG2	PCED1B-AS1	0.629172348	2.58E-60	postive
PRKCB	PCED1B-AS1	0.550416893	1.06E-43	postive
IGHG1	PCED1B-AS1	0.582883767	5.28E-50	postive
IGHV3-15	PCED1B-AS1	0.504813882	6.14E-36	postive
IGHV5-51	PCED1B-AS1	0.534925962	6.24E-41	postive
IGKC	PCED1B-AS1	0.665375092	1.10E-69	postive
IGKJ5	PCED1B-AS1	0.651465899	6.22E-66	postive
IGKV1-5	PCED1B-AS1	0.540454192	6.65E-42	postive
IGKV3-11	PCED1B-AS1	0.58277407	5.56E-50	postive
IGKV3-15	PCED1B-AS1	0.557649628	4.79E-45	postive
IGKV3-20	PCED1B-AS1	0.605039099	9.97E-55	postive
IGKV3-7	PCED1B-AS1	0.535923862	4.18E-41	postive
IGKV3D-11	PCED1B-AS1	0.543162781	2.19E-42	postive
IGKV3D-20	PCED1B-AS1	0.502225119	1.56E-35	postive
IGKV4-1	PCED1B-AS1	0.529130199	6.24E-40	postive
IGLC2	PCED1B-AS1	0.59812968	3.25E-53	postive
IGLC3	PCED1B-AS1	0.589358719	2.40E-51	postive
IGLJ1	PCED1B-AS1	0.522691738	7.66E-39	postive
IGLV1-44	PCED1B-AS1	0.509589242	1.07E-36	postive
IGLV2-14	PCED1B-AS1	0.5174237	5.72E-38	postive
IGLV3-21	PCED1B-AS1	0.518413515	3.93E-38	postive
IGLV6-57	PCED1B-AS1	0.519367303	2.74E-38	postive
SEMA4D	PCED1B-AS1	0.536459876	3.37E-41	postive
CXCR3	PCED1B-AS1	0.501588459	1.97E-35	postive
PLXNC1	PCED1B-AS1	0.58586806	1.28E-50	postive
ADA2	PCED1B-AS1	0.509373172	1.16E-36	postive
EBI3	PCED1B-AS1	0.532603549	1.58E-40	postive
FLT3LG	PCED1B-AS1	0.589462655	2.28E-51	postive
GMFG	PCED1B-AS1	0.758221411	4.48E-101	postive
IL16	PCED1B-AS1	0.770415522	2.85E-106	postive
LTB	PCED1B-AS1	0.540653316	6.13E-42	postive
PNOC	PCED1B-AS1	0.601565873	5.81E-54	postive
TNFSF13B	PCED1B-AS1	0.668199241	1.80E-70	postive
TNFSF8	PCED1B-AS1	0.578916653	3.40E-49	postive
CSF2RB	PCED1B-AS1	0.64021936	4.86E-63	postive
IL10RA	PCED1B-AS1	0.758127512	4.89E-101	postive
IL12RB1	PCED1B-AS1	0.701330908	2.16E-80	postive
IL2RB	PCED1B-AS1	0.603438703	2.25E-54	postive
IL18RAP	PCED1B-AS1	0.622240439	1.17E-58	postive
IL21R	PCED1B-AS1	0.681041513	3.68E-74	postive
IL2RG	PCED1B-AS1	0.628312038	4.16E-60	postive

TNFRSF13C	PCED1B-AS1	0.54579265	7.35E-43	postive
TNFRSF17	PCED1B-AS1	0.684515144	3.43E-75	postive
ITGAL	PCED1B-AS1	0.705968232	6.86E-82	postive
TYROBP	PCED1B-AS1	0.553084859	3.41E-44	postive
LCK	PCED1B-AS1	0.641147149	2.83E-63	postive
NCR3	PCED1B-AS1	0.683862388	5.37E-75	postive
CD247	PCED1B-AS1	0.768287536	2.42E-105	postive
ZAP70	PCED1B-AS1	0.680576963	5.04E-74	postive
LCP2	PCED1B-AS1	0.696925498	5.40E-79	postive
HCST	PCED1B-AS1	0.648557537	3.58E-65	postive
CD48	PCED1B-AS1	0.698602385	1.60E-79	postive
CD244	PCED1B-AS1	0.59455363	1.91E-52	postive
SH2D1A	PCED1B-AS1	0.815855683	6.86E-129	postive
CD3D	PCED1B-AS1	0.754123308	2.13E-99	postive
CD3E	PCED1B-AS1	0.749741925	1.22E-97	postive
CD3G	PCED1B-AS1	0.724416922	3.72E-88	postive
PTPRC	PCED1B-AS1	0.693847323	4.94E-78	postive
ITK	PCED1B-AS1	0.644752857	3.43E-64	postive
CD28	PCED1B-AS1	0.643174575	8.67E-64	postive
ICOS	PCED1B-AS1	0.68866649	1.93E-76	postive
CTLA4	PCED1B-AS1	0.590582815	1.33E-51	postive
TRAC	PCED1B-AS1	0.763439284	2.92E-103	postive
TRAV3	PCED1B-AS1	0.514504909	1.72E-37	postive
TRAV4	PCED1B-AS1	0.705656772	8.66E-82	postive
TRAV8-2	PCED1B-AS1	0.658495898	8.37E-68	postive
TRAV8-3	PCED1B-AS1	0.605468789	8.01E-55	postive
TRAV8-4	PCED1B-AS1	0.616402271	2.68E-57	postive
TRAV8-6	PCED1B-AS1	0.617352701	1.62E-57	postive
TRAV9-2	PCED1B-AS1	0.643099909	9.06E-64	postive
TRAV12-2	PCED1B-AS1	0.617336762	1.63E-57	postive
TRAV12-3	PCED1B-AS1	0.568141221	4.69E-47	postive
TRAV13-2	PCED1B-AS1	0.564962456	1.94E-46	postive
TRAV14DV4	PCED1B-AS1	0.513083377	2.92E-37	postive
TRAV16	PCED1B-AS1	0.63804368	1.71E-62	postive
TRAV17	PCED1B-AS1	0.626619376	1.06E-59	postive
TRAV21	PCED1B-AS1	0.560221871	1.56E-45	postive
TRAV29DV5	PCED1B-AS1	0.619941567	4.04E-58	postive
TRAV39	PCED1B-AS1	0.504066243	8.05E-36	postive
TRAV41	PCED1B-AS1	0.5282362	8.87E-40	postive
TRBC1	PCED1B-AS1	0.773709511	9.89E-108	postive
TRBC2	PCED1B-AS1	0.753535105	3.69E-99	postive
TRBJ2-2	PCED1B-AS1	0.541871325	3.72E-42	postive
TRBJ2-3	PCED1B-AS1	0.585365925	1.63E-50	postive
TRBJ2-7	PCED1B-AS1	0.607467785	2.87E-55	postive
TRBV2	PCED1B-AS1	0.691404008	2.81E-77	postive
TRBV3-1	PCED1B-AS1	0.616887053	2.07E-57	postive
TRBV5-1	PCED1B-AS1	0.70856382	9.64E-83	postive
TRBV5-4	PCED1B-AS1	0.633272024	2.59E-61	postive
TRBV6-1	PCED1B-AS1	0.617024906	1.93E-57	postive
TRBV6-5	PCED1B-AS1	0.654854203	7.91E-67	postive
TRBV6-6	PCED1B-AS1	0.52420771	4.26E-39	postive
TRBV7-9	PCED1B-AS1	0.649556861	1.97E-65	postive
TRBV9	PCED1B-AS1	0.570811718	1.41E-47	postive
TRBV11-2	PCED1B-AS1	0.583763507	3.49E-50	postive
TRBV12-4	PCED1B-AS1	0.583965214	3.17E-50	postive
TRBV18	PCED1B-AS1	0.699311963	9.52E-80	postive
TRBV19	PCED1B-AS1	0.59916967	1.93E-53	postive
TRBV20-1	PCED1B-AS1	0.69758755	3.34E-79	postive
TRBV28	PCED1B-AS1	0.767430755	5.70E-105	postive
CALCA	AL160271.2	0.615852699	3.59E-57	postive
NPY	AL160271.2	0.600309089	1.09E-53	postive
CREB1	AC007938.3	0.60566888	7.23E-55	postive
MAPK8	AC007938.3	0.519012803	3.13E-38	postive
DDX17	AC007938.3	0.545449989	8.48E-43	postive
NFAT5	AC007938.3	0.657664978	1.40E-67	postive

GNRH1	AC007938.3	0.692309099	1.48E-77	postive
ACVR2A	AC007938.3	0.555240604	1.35E-44	postive
ANGPTL1	AC007938.3	0.548717061	2.16E-43	postive
BMPR2	AC007938.3	0.534562015	7.22E-41	postive
NR2C1	AC007938.3	0.554123649	2.19E-44	postive
NR2C2	AC007938.3	0.646827716	1.00E-64	postive
RORA	AC007938.3	0.605898787	6.43E-55	postive
SOS1	AC007938.3	0.604682917	1.20E-54	postive
BRAF	AC007938.3	0.700666353	3.53E-80	postive
CBLB	AC007938.3	0.567737829	5.62E-47	postive
TRAJ1	AC007938.3	0.630410727	1.29E-60	postive
TRAJ2	AC007938.3	0.574386655	2.76E-48	postive
TRAJ3	AC007938.3	0.650744329	9.62E-66	postive
TRAJ5	AC007938.3	0.641986717	1.74E-63	postive
TRAJ6	AC007938.3	0.661410345	1.36E-68	postive
TRAJ8	AC007938.3	0.670008824	5.58E-71	postive
TRAJ10	AC007938.3	0.614597158	6.98E-57	postive
TRAJ12	AC007938.3	0.650450737	1.15E-65	postive
TRAJ13	AC007938.3	0.658642271	7.64E-68	postive
TRAJ14	AC007938.3	0.656663903	2.60E-67	postive
TRAJ16	AC007938.3	0.653351329	1.98E-66	postive
TRAJ17	AC007938.3	0.638611798	1.23E-62	postive
TRAJ18	AC007938.3	0.57041189	1.68E-47	postive
TRAJ21	AC007938.3	0.664204137	2.32E-69	postive
TRAJ31	AC007938.3	0.680478782	5.39E-74	postive
TRAJ37	AC007938.3	0.655632712	4.91E-67	postive
TRAJ38	AC007938.3	0.688385638	2.34E-76	postive
TRAJ39	AC007938.3	0.659486268	4.52E-68	postive
CREB1	AL683813.2	0.532580947	1.59E-40	postive
RFXAP	AL683813.2	0.521878158	1.05E-38	postive
DDX17	AL683813.2	0.62503516	2.54E-59	postive
NFAT5	AL683813.2	0.608261027	1.91E-55	postive
PIK3R1	AL683813.2	0.500433632	2.98E-35	postive
LTB4R2	AL683813.2	0.572765321	5.79E-48	postive
GNRH1	AL683813.2	0.645794878	1.85E-64	postive
IL6ST	AL683813.2	0.518484073	3.83E-38	postive
ACVR2A	AL683813.2	0.506201357	3.70E-36	postive
ANGPTL1	AL683813.2	0.511333625	5.61E-37	postive
NR2C2	AL683813.2	0.664785516	1.60E-69	postive
RORA	AL683813.2	0.628692946	3.37E-60	postive
BRAF	AL683813.2	0.622374259	1.08E-58	postive
TRAJ2	AL683813.2	0.517909942	4.76E-38	postive
TRAJ3	AL683813.2	0.552379649	4.60E-44	postive
TRAJ5	AL683813.2	0.510880137	6.63E-37	postive
TRAJ6	AL683813.2	0.509144779	1.26E-36	postive
TRAJ10	AL683813.2	0.517433544	5.70E-38	postive
TRAJ16	AL683813.2	0.50354824	9.70E-36	postive
TRAJ21	AL683813.2	0.515603649	1.14E-37	postive
TRAJ31	AL683813.2	0.541367402	4.57E-42	postive
TRAJ37	AL683813.2	0.550354138	1.09E-43	postive
TRAJ38	AL683813.2	0.559502243	2.14E-45	postive
TRAJ39	AL683813.2	0.530073376	4.30E-40	postive
CREB1	AC024060.2	0.511514199	5.24E-37	postive
RFXAP	AC024060.2	0.520733678	1.62E-38	postive
DDX17	AC024060.2	0.70134374	2.14E-80	postive
NFAT5	AC024060.2	0.624130194	4.17E-59	postive
GNRH1	AC024060.2	0.799279631	5.75E-120	postive
ACVR2A	AC024060.2	0.521508259	1.21E-38	postive
ANGPTL1	AC024060.2	0.529395202	5.62E-40	postive
NR2C1	AC024060.2	0.593711341	2.88E-52	postive
NR2C2	AC024060.2	0.712757555	3.87E-84	postive
RORA	AC024060.2	0.647366326	7.29E-65	postive
BRAF	AC024060.2	0.59124021	9.64E-52	postive
CBLB	AC024060.2	0.517465374	5.63E-38	postive
TRAJ1	AC024060.2	0.560855958	1.19E-45	postive

TRAJ2	AC024060.2	0.520124924	2.05E-38	postive
TRAJ3	AC024060.2	0.56429864	2.60E-46	postive
TRAJ5	AC024060.2	0.59237966	5.53E-52	postive
TRAJ6	AC024060.2	0.556982642	6.39E-45	postive
TRAJ8	AC024060.2	0.538424594	1.52E-41	postive
TRAJ10	AC024060.2	0.542811548	2.53E-42	postive
TRAJ12	AC024060.2	0.509353277	1.17E-36	postive
TRAJ13	AC024060.2	0.558370903	3.50E-45	postive
TRAJ14	AC024060.2	0.532470408	1.66E-40	postive
TRAJ16	AC024060.2	0.564943701	1.95E-46	postive
TRAJ17	AC024060.2	0.532661357	1.54E-40	postive
TRAJ18	AC024060.2	0.510265807	8.33E-37	postive
TRAJ21	AC024060.2	0.572376677	6.91E-48	postive
TRAJ31	AC024060.2	0.602148134	4.33E-54	postive
TRAJ37	AC024060.2	0.588710119	3.28E-51	postive
TRAJ38	AC024060.2	0.600666671	9.13E-54	postive
TRAJ39	AC024060.2	0.596280445	8.13E-53	postive
CREB1	AC007991.2	0.539767454	8.80E-42	postive
IL15	AC007991.2	0.643555584	6.93E-64	postive
EIF2AK2	AC007991.2	0.518369339	4.00E-38	postive
JAK2	AC007991.2	0.605628766	7.38E-55	postive
NFAT5	AC007991.2	0.510960973	6.44E-37	postive
PIK3CA	AC007991.2	0.566027628	1.21E-46	postive
ANGPTL1	AC007991.2	0.565820736	1.32E-46	postive
RORA	AC007991.2	0.507104758	2.66E-36	postive
SOS1	AC007991.2	0.534147131	8.52E-41	postive
BRAF	AC007991.2	0.565967552	1.24E-46	postive
CBL	AC007991.2	0.526033653	2.10E-39	postive
CBLB	AC007991.2	0.572219337	7.42E-48	postive
TRAJ1	AC007991.2	0.603406221	2.29E-54	postive
TRAJ2	AC007991.2	0.519540469	2.56E-38	postive
TRAJ3	AC007991.2	0.628131552	4.60E-60	postive
TRAJ5	AC007991.2	0.60726045	3.20E-55	postive
TRAJ6	AC007991.2	0.600566282	9.60E-54	postive
TRAJ8	AC007991.2	0.552007709	5.39E-44	postive
TRAJ10	AC007991.2	0.582391279	6.67E-50	postive
TRAJ12	AC007991.2	0.608838593	1.42E-55	postive
TRAJ13	AC007991.2	0.555361718	1.28E-44	postive
TRAJ14	AC007991.2	0.623836563	4.89E-59	postive
TRAJ16	AC007991.2	0.587688741	5.36E-51	postive
TRAJ17	AC007991.2	0.594821546	1.67E-52	postive
TRAJ18	AC007991.2	0.619883129	4.17E-58	postive
TRAJ21	AC007991.2	0.61660355	2.41E-57	postive
TRAJ31	AC007991.2	0.583652552	3.67E-50	postive
TRAJ37	AC007991.2	0.525199651	2.90E-39	postive
TRAJ38	AC007991.2	0.637220259	2.74E-62	postive
TRAJ39	AC007991.2	0.605453259	8.07E-55	postive
IL1R2	LINC01843	0.544791311	1.11E-42	postive
CREB1	AC004067.1	0.546417241	5.67E-43	postive
CYLD	AC004067.1	0.536997225	2.71E-41	postive
DDX17	AC004067.1	0.551305541	7.26E-44	postive
JAK2	AC004067.1	0.508696237	1.49E-36	postive
NFAT5	AC004067.1	0.606289974	5.26E-55	postive
GNRH1	AC004067.1	0.636133611	5.10E-62	postive
ANGPTL1	AC004067.1	0.503409086	1.02E-35	postive
BMPR2	AC004067.1	0.501280269	2.20E-35	postive
NR2C2	AC004067.1	0.60663432	4.41E-55	postive
RORA	AC004067.1	0.601055439	7.51E-54	postive
SOS1	AC004067.1	0.50594088	4.07E-36	postive
BRAF	AC004067.1	0.603960464	1.73E-54	postive
CBLB	AC004067.1	0.591171066	9.97E-52	postive
TRAJ1	AC004067.1	0.548853692	2.04E-43	postive
TRAJ2	AC004067.1	0.550450499	1.04E-43	postive
TRAJ3	AC004067.1	0.583148361	4.66E-50	postive
TRAJ5	AC004067.1	0.538470074	1.49E-41	postive

TRAJ6	AC004067.1	0.546789662	4.85E-43	postive
TRAJ8	AC004067.1	0.511498955	5.27E-37	postive
TRAJ10	AC004067.1	0.529422585	5.56E-40	postive
TRAJ12	AC004067.1	0.530618199	3.47E-40	postive
TRAJ13	AC004067.1	0.525712168	2.38E-39	postive
TRAJ14	AC004067.1	0.529455441	5.49E-40	postive
TRAJ16	AC004067.1	0.55603624	9.61E-45	postive
TRAJ17	AC004067.1	0.512994282	3.02E-37	postive
TRAJ18	AC004067.1	0.537887694	1.89E-41	postive
TRAJ21	AC004067.1	0.544244736	1.40E-42	postive
TRAJ31	AC004067.1	0.584086562	2.99E-50	postive
TRAJ37	AC004067.1	0.551859637	5.74E-44	postive
TRAJ38	AC004067.1	0.604718519	1.17E-54	postive
TRAJ39	AC004067.1	0.559920504	1.78E-45	postive
DDX17	AP001107.4	0.629997165	1.63E-60	postive
IRF9	AP001107.4	0.503240177	1.08E-35	postive
NFAT5	AP001107.4	0.531715615	2.25E-40	postive
LTB4R2	AP001107.4	0.584612514	2.33E-50	postive
GNRH1	AP001107.4	0.674677703	2.62E-72	postive
NR2C1	AP001107.4	0.569392262	2.67E-47	postive
NR2C2	AP001107.4	0.65539284	5.69E-67	postive
BRAF	AP001107.4	0.515808881	1.05E-37	postive
TRAJ3	AP001107.4	0.511346061	5.58E-37	postive
FGR	AC009974.1	0.516855702	7.09E-38	postive
CREB1	AC010186.3	0.754807734	1.12E-99	postive
UBR1	AC010186.3	0.572242377	7.34E-48	postive
ZC3HAV1	AC010186.3	0.552457246	4.45E-44	postive
IL15	AC010186.3	0.605405578	8.27E-55	postive
CYLD	AC010186.3	0.628627785	3.50E-60	postive
EIF2AK2	AC010186.3	0.547950548	2.99E-43	postive
MAPK8	AC010186.3	0.521629119	1.15E-38	postive
TLR1	AC010186.3	0.54965954	1.46E-43	postive
LMBR1	AC010186.3	0.6092735	1.13E-55	postive
LIMS1	AC010186.3	0.559280102	2.36E-45	postive
IREB2	AC010186.3	0.557446073	5.23E-45	postive
DDX17	AC010186.3	0.530755594	3.29E-40	postive
PIK3CG	AC010186.3	0.585581867	1.47E-50	postive
JAK2	AC010186.3	0.671846001	1.69E-71	postive
TXK	AC010186.3	0.548590958	2.28E-43	postive
NFAT5	AC010186.3	0.821286971	5.18E-132	postive
MALT1	AC010186.3	0.542773159	2.57E-42	postive
PIK3R1	AC010186.3	0.633875179	1.84E-61	postive
PIK3CA	AC010186.3	0.713185899	2.78E-84	postive
PIK3CB	AC010186.3	0.503193239	1.10E-35	postive
AKT3	AC010186.3	0.507501754	2.30E-36	postive
RASGRP3	AC010186.3	0.544721199	1.15E-42	postive
PLXNC1	AC010186.3	0.520285493	1.93E-38	postive
GNRH1	AC010186.3	0.629752752	1.87E-60	postive
IL6ST	AC010186.3	0.636320741	4.58E-62	postive
ACVR2A	AC010186.3	0.557068683	6.16E-45	postive
ANGPTL1	AC010186.3	0.76879495	1.46E-105	postive
BMPR2	AC010186.3	0.678291891	2.35E-73	postive
CRLF3	AC010186.3	0.685988073	1.24E-75	postive
IL18R1	AC010186.3	0.520082727	2.08E-38	postive
NR2C2	AC010186.3	0.616255471	2.90E-57	postive
RORA	AC010186.3	0.842482069	2.70E-145	postive
SOS1	AC010186.3	0.642620417	1.20E-63	postive
SOS2	AC010186.3	0.571570186	9.97E-48	postive
BRAF	AC010186.3	0.778485115	6.84E-110	postive
PTPRC	AC010186.3	0.505963748	4.04E-36	postive
ITK	AC010186.3	0.576144839	1.23E-48	postive
CD28	AC010186.3	0.548249499	2.63E-43	postive
CBL	AC010186.3	0.601449803	6.16E-54	postive
CBLB	AC010186.3	0.745667434	4.87E-96	postive
RASGRP1	AC010186.3	0.503916603	8.49E-36	postive

PDK1	AC010186.3	0.616292475	2.84E-57	postive
TRAJ1	AC010186.3	0.722734673	1.45E-87	postive
TRAJ2	AC010186.3	0.742370787	9.15E-95	postive
TRAJ3	AC010186.3	0.769565965	6.71E-106	postive
TRAJ5	AC010186.3	0.825629989	1.38E-134	postive
TRAJ6	AC010186.3	0.780377545	9.20E-111	postive
TRAJ8	AC010186.3	0.752201215	1.27E-98	postive
TRAJ10	AC010186.3	0.780918864	5.17E-111	postive
TRAJ12	AC010186.3	0.676554272	7.52E-73	postive
TRAJ13	AC010186.3	0.80198436	2.30E-121	postive
TRAJ14	AC010186.3	0.775696361	1.27E-108	postive
TRAJ16	AC010186.3	0.799059981	7.45E-120	postive
TRAJ17	AC010186.3	0.753415442	4.12E-99	postive
TRAJ18	AC010186.3	0.7431243	4.70E-95	postive
TRAJ21	AC010186.3	0.823810601	1.69E-133	postive
TRAJ31	AC010186.3	0.838414017	1.34E-142	postive
TRAJ37	AC010186.3	0.811178816	2.77E-126	postive
TRAJ38	AC010186.3	0.865145999	7.16E-162	postive
TRAJ39	AC010186.3	0.841195597	1.96E-144	postive
PSMD4	ZNF687-AS1	0.589823637	1.92E-51	postive
CREB1	AC004832.5	0.803879718	2.34E-122	postive
RFXAP	AC004832.5	0.520464477	1.80E-38	postive
UBR1	AC004832.5	0.679087027	1.38E-73	postive
ZC3HAV1	AC004832.5	0.574510782	2.60E-48	postive
IL15	AC004832.5	0.587738795	5.23E-51	postive
CYLD	AC004832.5	0.614973381	5.72E-57	postive
EIF2AK2	AC004832.5	0.560531336	1.37E-45	postive
MAPK8	AC004832.5	0.573846173	3.53E-48	postive
LMBR1	AC004832.5	0.58440078	2.58E-50	postive
LIMS1	AC004832.5	0.532215256	1.84E-40	postive
IREB2	AC004832.5	0.626865996	9.27E-60	postive
DDX17	AC004832.5	0.633632563	2.11E-61	postive
PIK3CG	AC004832.5	0.535297129	5.37E-41	postive
JAK2	AC004832.5	0.654893407	7.72E-67	postive
TXK	AC004832.5	0.640735361	3.60E-63	postive
NFAT5	AC004832.5	0.857096871	1.17E-155	postive
MALT1	AC004832.5	0.553239068	3.19E-44	postive
PIK3R1	AC004832.5	0.648273949	4.24E-65	postive
PIK3CA	AC004832.5	0.721253733	4.79E-87	postive
PIK3CB	AC004832.5	0.550637914	9.63E-44	postive
AKT3	AC004832.5	0.552779711	3.88E-44	postive
RASGRP3	AC004832.5	0.52787248	1.02E-39	postive
IGHD6-6	AC004832.5	0.549738325	1.41E-43	postive
GNRH1	AC004832.5	0.798417974	1.59E-119	postive
IL6ST	AC004832.5	0.629394801	2.28E-60	postive
IL7	AC004832.5	0.52184118	1.06E-38	postive
RABEP1	AC004832.5	0.575091403	1.99E-48	postive
ACVR2A	AC004832.5	0.660728746	2.08E-68	postive
ANGPTL1	AC004832.5	0.782998322	5.54E-112	postive
BMPR1A	AC004832.5	0.567033497	7.70E-47	postive
BMPR2	AC004832.5	0.769312683	8.66E-106	postive
CRLF3	AC004832.5	0.655378257	5.74E-67	postive
NR1D2	AC004832.5	0.517672769	5.21E-38	postive
NR2C2	AC004832.5	0.711410038	1.09E-83	postive
RORA	AC004832.5	0.848848706	1.13E-149	postive
SOS1	AC004832.5	0.68884508	1.70E-76	postive
SOS2	AC004832.5	0.581760439	8.97E-50	postive
BRAF	AC004832.5	0.792011567	2.58E-116	postive
TEC	AC004832.5	0.50040648	3.00E-35	postive
CBL	AC004832.5	0.606262402	5.33E-55	postive
CBLB	AC004832.5	0.730700015	2.08E-90	postive
RASGRP1	AC004832.5	0.507494404	2.31E-36	postive
PDK1	AC004832.5	0.611410329	3.72E-56	postive
TRAJ1	AC004832.5	0.715336124	5.21E-85	postive
TRAJ2	AC004832.5	0.716069621	2.93E-85	postive

TRAJ3	AC004832.5	0.7787074	5.41E-110	postive
TRAJ5	AC004832.5	0.766878177	9.87E-105	postive
TRAJ6	AC004832.5	0.783289873	4.04E-112	postive
TRAJ8	AC004832.5	0.749027006	2.34E-97	postive
TRAJ10	AC004832.5	0.763990581	1.70E-103	postive
TRAJ12	AC004832.5	0.685797165	1.42E-75	postive
TRAJ13	AC004832.5	0.764016894	1.66E-103	postive
TRAJ14	AC004832.5	0.738574524	2.54E-93	postive
TRAJ16	AC004832.5	0.787915361	2.55E-114	postive
TRAJ17	AC004832.5	0.741547356	1.89E-94	postive
TRAJ18	AC004832.5	0.71862802	3.89E-86	postive
TRAJ21	AC004832.5	0.797154234	6.97E-119	postive
TRAJ31	AC004832.5	0.815323982	1.37E-128	postive
TRAJ37	AC004832.5	0.788676574	1.09E-114	postive
TRAJ38	AC004832.5	0.846383585	5.90E-148	postive
TRAJ39	AC004832.5	0.819358526	6.83E-131	postive
CREB1	GMDS-DT	0.817757384	5.69E-130	postive
RFXAP	GMDS-DT	0.557086031	6.11E-45	postive
UBR1	GMDS-DT	0.727198372	3.81E-89	postive
ZC3HAV1	GMDS-DT	0.582513835	6.29E-50	postive
ZC3HAV1L	GMDS-DT	0.519029098	3.11E-38	postive
IL15	GMDS-DT	0.550952762	8.43E-44	postive
CYLD	GMDS-DT	0.648036279	4.89E-65	postive
EIF2AK2	GMDS-DT	0.570768529	1.43E-47	postive
MAPK8	GMDS-DT	0.582544224	6.20E-50	postive
LMBR1	GMDS-DT	0.571670874	9.52E-48	postive
IREB2	GMDS-DT	0.645260382	2.54E-64	postive
DDX17	GMDS-DT	0.654620234	9.13E-67	postive
PIK3CG	GMDS-DT	0.56455223	2.32E-46	postive
JAK2	GMDS-DT	0.654252161	1.14E-66	postive
TXK	GMDS-DT	0.617991263	1.15E-57	postive
NFAT5	GMDS-DT	0.860117515	6.05E-158	postive
NFATC3	GMDS-DT	0.522859371	7.18E-39	postive
MALT1	GMDS-DT	0.536422059	3.42E-41	postive
PIK3R1	GMDS-DT	0.660890448	1.88E-68	postive
PIK3CA	GMDS-DT	0.696086908	9.90E-79	postive
PIK3CB	GMDS-DT	0.549541404	1.53E-43	postive
AKT3	GMDS-DT	0.504668611	6.47E-36	postive
RASGRP3	GMDS-DT	0.546918647	4.60E-43	postive
IGHD4-4	GMDS-DT	0.50297578	1.19E-35	postive
IGHD6-6	GMDS-DT	0.539363645	1.04E-41	postive
PLXNC1	GMDS-DT	0.512810099	3.24E-37	postive
ROBO2	GMDS-DT	0.507555354	2.26E-36	postive
GNRH1	GMDS-DT	0.771615659	8.42E-107	postive
IL6ST	GMDS-DT	0.64562681	2.05E-64	postive
IL7	GMDS-DT	0.521706065	1.12E-38	postive
RABEP1	GMDS-DT	0.587372326	6.24E-51	postive
ACVR2A	GMDS-DT	0.712352914	5.29E-84	postive
ANGPTL1	GMDS-DT	0.749998025	9.64E-98	postive
BMPR1A	GMDS-DT	0.61479947	6.27E-57	postive
BMPR2	GMDS-DT	0.73909938	1.61E-93	postive
CRLF3	GMDS-DT	0.645336548	2.43E-64	postive
NR1D2	GMDS-DT	0.565597497	1.46E-46	postive
NR2C2	GMDS-DT	0.762332629	8.59E-103	postive
NR3C1	GMDS-DT	0.522513865	8.20E-39	postive
NR3C2	GMDS-DT	0.501334412	2.15E-35	postive
RORA	GMDS-DT	0.843058965	1.10E-145	postive
SOS1	GMDS-DT	0.693919446	4.69E-78	postive
SOS2	GMDS-DT	0.611713506	3.18E-56	postive
BRAF	GMDS-DT	0.817704825	6.09E-130	postive
TEC	GMDS-DT	0.530223903	4.06E-40	postive
CBL	GMDS-DT	0.551840357	5.78E-44	postive
CBLB	GMDS-DT	0.693495553	6.35E-78	postive
RASGRP1	GMDS-DT	0.529630413	5.13E-40	postive
PDK1	GMDS-DT	0.591789177	7.38E-52	postive

TRAJ1	GMDS-DT	0.756842222	1.66E-100	postive
TRAJ2	GMDS-DT	0.726987917	4.53E-89	postive
TRAJ3	GMDS-DT	0.800237903	1.85E-120	postive
TRAJ5	GMDS-DT	0.798239749	1.96E-119	postive
TRAJ6	GMDS-DT	0.810481339	6.69E-126	postive
TRAJ8	GMDS-DT	0.777237771	2.54E-109	postive
TRAJ10	GMDS-DT	0.791111927	7.14E-116	postive
TRAJ12	GMDS-DT	0.757409896	9.68E-101	postive
TRAJ13	GMDS-DT	0.782477237	9.71E-112	postive
TRAJ14	GMDS-DT	0.77748788	1.95E-109	postive
TRAJ16	GMDS-DT	0.802065126	2.09E-121	postive
TRAJ17	GMDS-DT	0.774239291	5.73E-108	postive
TRAJ18	GMDS-DT	0.750116031	8.65E-98	postive
TRAJ21	GMDS-DT	0.814555829	3.70E-128	postive
TRAJ31	GMDS-DT	0.837010451	1.10E-141	postive
TRAJ37	GMDS-DT	0.806085615	1.59E-123	postive
TRAJ38	GMDS-DT	0.87050513	3.10E-166	postive
TRAJ39	GMDS-DT	0.838240044	1.75E-142	postive
DDX17	CTBP1-AS	0.649635221	1.88E-65	postive
IRF9	CTBP1-AS	0.595289026	1.33E-52	postive
NFAT5	CTBP1-AS	0.508999962	1.33E-36	postive
LTB4R2	CTBP1-AS	0.618472291	8.89E-58	postive
GNRH1	CTBP1-AS	0.673544631	5.53E-72	postive
NR2C2	CTBP1-AS	0.679271408	1.22E-73	postive
BRAF	CTBP1-AS	0.52903415	6.48E-40	postive
TRAJ1	CTBP1-AS	0.508047797	1.88E-36	postive
TRAJ3	CTBP1-AS	0.534058596	8.83E-41	postive
CREB1	AL109614.1	0.802504874	1.23E-121	postive
RFXAP	AL109614.1	0.568995685	3.19E-47	postive
UBR1	AL109614.1	0.699544108	8.03E-80	postive
ZC3HAV1	AL109614.1	0.601913359	4.87E-54	postive
ZC3HAV1L	AL109614.1	0.52658628	1.69E-39	postive
IL15	AL109614.1	0.553066553	3.43E-44	postive
CYLD	AL109614.1	0.634270766	1.47E-61	postive
EIF2AK2	AL109614.1	0.533253394	1.22E-40	postive
MAPK8	AL109614.1	0.587917248	4.80E-51	postive
LMBR1	AL109614.1	0.578404253	4.31E-49	postive
IREB2	AL109614.1	0.654256929	1.14E-66	postive
DDX17	AL109614.1	0.653445558	1.87E-66	postive
PIK3CG	AL109614.1	0.51910522	3.02E-38	postive
JAK2	AL109614.1	0.595840925	1.01E-52	postive
TXK	AL109614.1	0.635107862	9.15E-62	postive
NFAT5	AL109614.1	0.888802509	9.49E-183	postive
NFATC3	AL109614.1	0.557803373	4.48E-45	postive
MALT1	AL109614.1	0.545782756	7.38E-43	postive
PIK3R1	AL109614.1	0.664590247	1.82E-69	postive
PIK3CA	AL109614.1	0.685764958	1.45E-75	postive
PIK3CB	AL109614.1	0.527239576	1.31E-39	postive
AKT3	AL109614.1	0.576178274	1.21E-48	postive
GSK3B	AL109614.1	0.506169763	3.75E-36	postive
IGHD4-4	AL109614.1	0.561937068	7.37E-46	postive
IGHD6-6	AL109614.1	0.578131917	4.89E-49	postive
GNRH1	AL109614.1	0.778946886	4.20E-110	postive
IL6ST	AL109614.1	0.657146046	1.93E-67	postive
RABEP1	AL109614.1	0.596466954	7.42E-53	postive
ACVR2A	AL109614.1	0.700990684	2.78E-80	postive
ANGPTL1	AL109614.1	0.733151526	2.64E-91	postive
BMPR1A	AL109614.1	0.611681067	3.23E-56	postive
BMPR2	AL109614.1	0.755598036	5.35E-100	postive
CRLF3	AL109614.1	0.625292057	2.21E-59	postive
NR1D2	AL109614.1	0.56446797	2.41E-46	postive
NR2C1	AL109614.1	0.509839407	9.75E-37	postive
NR2C2	AL109614.1	0.735605904	3.27E-92	postive
RORA	AL109614.1	0.866099711	1.24E-162	postive
SOS1	AL109614.1	0.685406641	1.86E-75	postive

SOS2	AL109614.1	0.595343419	1.29E-52	postive
BRAF	AL109614.1	0.827119195	1.74E-135	postive
ITK	AL109614.1	0.508942965	1.36E-36	postive
CBL	AL109614.1	0.611309452	3.92E-56	postive
CBLB	AL109614.1	0.663896438	2.82E-69	postive
PDK1	AL109614.1	0.560047476	1.69E-45	postive
TRAJ1	AL109614.1	0.735897987	2.55E-92	postive
TRAJ2	AL109614.1	0.767052077	8.31E-105	postive
TRAJ3	AL109614.1	0.827689374	7.84E-136	postive
TRAJ5	AL109614.1	0.769398051	7.95E-106	postive
TRAJ6	AL109614.1	0.808043481	1.41E-124	postive
TRAJ8	AL109614.1	0.803838948	2.46E-122	postive
TRAJ10	AL109614.1	0.78459623	9.79E-113	postive
TRAJ12	AL109614.1	0.714194925	1.27E-84	postive
TRAJ13	AL109614.1	0.804191976	1.60E-122	postive
TRAJ14	AL109614.1	0.771867113	6.52E-107	postive
TRAJ16	AL109614.1	0.783604457	2.87E-112	postive
TRAJ17	AL109614.1	0.769025219	1.16E-105	postive
TRAJ18	AL109614.1	0.730388235	2.70E-90	postive
TRAJ21	AL109614.1	0.821381095	4.56E-132	postive
TRAJ31	AL109614.1	0.836544855	2.20E-141	postive
TRAJ37	AL109614.1	0.841941777	6.22E-145	postive
TRAJ38	AL109614.1	0.851208884	2.38E-151	postive
TRAJ39	AL109614.1	0.84499233	5.34E-147	postive
IRF9	AP001160.1	0.579886587	2.16E-49	postive
GNRH1	AP001160.1	0.524385057	3.98E-39	postive
EPOR	LMNTD2-AS1	0.511586937	5.10E-37	postive
GIPR	LMNTD2-AS1	0.509416397	1.14E-36	postive
CREB1	AC084824.6	0.589315459	2.45E-51	postive
DDX17	AC084824.6	0.602613848	3.42E-54	postive
JAK2	AC084824.6	0.513035672	2.98E-37	postive
NFAT5	AC084824.6	0.672554923	1.06E-71	postive
GNRH1	AC084824.6	0.721659422	3.46E-87	postive
ACVR2A	AC084824.6	0.550876448	8.71E-44	postive
ANGPTL1	AC084824.6	0.603810215	1.86E-54	postive
BMPR2	AC084824.6	0.526769179	1.57E-39	postive
NR2C1	AC084824.6	0.561218386	1.01E-45	postive
NR2C2	AC084824.6	0.598150565	3.21E-53	postive
RORA	AC084824.6	0.665017647	1.38E-69	postive
SOS1	AC084824.6	0.550678729	9.47E-44	postive
BRAF	AC084824.6	0.652170352	4.06E-66	postive
CBLB	AC084824.6	0.575551216	1.61E-48	postive
TRAJ1	AC084824.6	0.621447095	1.79E-58	postive
TRAJ2	AC084824.6	0.602086383	4.46E-54	postive
TRAJ3	AC084824.6	0.651348547	6.68E-66	postive
TRAJ5	AC084824.6	0.64619904	1.46E-64	postive
TRAJ6	AC084824.6	0.649721277	1.78E-65	postive
TRAJ8	AC084824.6	0.635825339	6.08E-62	postive
TRAJ10	AC084824.6	0.62926234	2.46E-60	postive
TRAJ12	AC084824.6	0.587860124	4.94E-51	postive
TRAJ13	AC084824.6	0.65937775	4.83E-68	postive
TRAJ14	AC084824.6	0.637988572	1.76E-62	postive
TRAJ16	AC084824.6	0.651366743	6.61E-66	postive
TRAJ17	AC084824.6	0.625548736	1.92E-59	postive
TRAJ18	AC084824.6	0.599094416	2.01E-53	postive
TRAJ21	AC084824.6	0.670876549	3.17E-71	postive
TRAJ31	AC084824.6	0.680920511	4.00E-74	postive
TRAJ37	AC084824.6	0.657370602	1.68E-67	postive
TRAJ38	AC084824.6	0.689723694	9.18E-77	postive
TRAJ39	AC084824.6	0.660688802	2.13E-68	postive
OSGIN1	AL391427.1	0.566659627	9.10E-47	postive
CREB1	AC107068.1	0.666673353	4.80E-70	postive
UBR1	AC107068.1	0.643295608	8.08E-64	postive
ZC3HAV1	AC107068.1	0.5533945	2.98E-44	postive
ZC3HAV1L	AC107068.1	0.577597893	6.27E-49	postive

MAPK8	AC107068.1	0.636491092	4.16E-62	postive
SP1	AC107068.1	0.500910533	2.51E-35	postive
IREB2	AC107068.1	0.652082187	4.28E-66	postive
DDX17	AC107068.1	0.603240292	2.49E-54	postive
TXK	AC107068.1	0.526346862	1.86E-39	postive
NFAT5	AC107068.1	0.693078221	8.55E-78	postive
MALT1	AC107068.1	0.504470412	6.95E-36	postive
PIK3CA	AC107068.1	0.537815901	1.95E-41	postive
GNRH1	AC107068.1	0.702397422	9.84E-81	postive
IL6ST	AC107068.1	0.502060862	1.66E-35	postive
RABEP1	AC107068.1	0.563815105	3.22E-46	postive
ACVR2A	AC107068.1	0.602803148	3.11E-54	postive
ACVR2B	AC107068.1	0.59815507	3.21E-53	postive
ANGPTL1	AC107068.1	0.620166243	3.58E-58	postive
BMPR1A	AC107068.1	0.592241724	5.92E-52	postive
BMPR2	AC107068.1	0.56477512	2.11E-46	postive
CRLF3	AC107068.1	0.51798955	4.62E-38	postive
NR2C1	AC107068.1	0.564566547	2.31E-46	postive
NR2C2	AC107068.1	0.670833221	3.26E-71	postive
RORA	AC107068.1	0.664677656	1.72E-69	postive
SOS1	AC107068.1	0.650454772	1.15E-65	postive
BRAF	AC107068.1	0.683531398	6.74E-75	postive
TEC	AC107068.1	0.581170714	1.18E-49	postive
CBL	AC107068.1	0.544791887	1.11E-42	postive
CBLB	AC107068.1	0.632908209	3.18E-61	postive
PDK1	AC107068.1	0.544067177	1.50E-42	postive
TRAJ1	AC107068.1	0.574475273	2.65E-48	postive
TRAJ2	AC107068.1	0.562092169	6.89E-46	postive
TRAJ3	AC107068.1	0.612969337	1.64E-56	postive
TRAJ5	AC107068.1	0.619437868	5.30E-58	postive
TRAJ6	AC107068.1	0.629111312	2.67E-60	postive
TRAJ8	AC107068.1	0.587319347	6.40E-51	postive
TRAJ10	AC107068.1	0.603172114	2.58E-54	postive
TRAJ12	AC107068.1	0.555928805	1.01E-44	postive
TRAJ13	AC107068.1	0.598258033	3.05E-53	postive
TRAJ14	AC107068.1	0.594866579	1.63E-52	postive
TRAJ16	AC107068.1	0.593930339	2.59E-52	postive
TRAJ17	AC107068.1	0.562982319	4.65E-46	postive
TRAJ18	AC107068.1	0.532403353	1.71E-40	postive
TRAJ21	AC107068.1	0.622278981	1.14E-58	postive
TRAJ31	AC107068.1	0.639229495	8.61E-63	postive
TRAJ37	AC107068.1	0.627476697	6.62E-60	postive
TRAJ38	AC107068.1	0.667211797	3.40E-70	postive
TRAJ39	AC107068.1	0.645254669	2.55E-64	postive
CIITA	AC083862.1	0.528643492	7.56E-40	postive
IL15	AC083862.1	0.537649142	2.08E-41	postive
CYLD	AC083862.1	0.519801749	2.32E-38	postive
EIF2AK2	AC083862.1	0.527335817	1.26E-39	postive
JAK2	AC083862.1	0.601631839	5.62E-54	postive
RASGRP3	AC083862.1	0.591688839	7.75E-52	postive
PLXNC1	AC083862.1	0.507890666	2.00E-36	postive
IL18RAP	AC083862.1	0.530481554	3.66E-40	postive
PTPRC	AC083862.1	0.504291983	7.41E-36	postive
ITK	AC083862.1	0.570799292	1.41E-47	postive
CBL	AC083862.1	0.521339853	1.29E-38	postive
CBLB	AC083862.1	0.532557377	1.61E-40	postive
TRAJ1	AC083862.1	0.554157448	2.15E-44	postive
TRAJ3	AC083862.1	0.535886372	4.24E-41	postive
TRAJ5	AC083862.1	0.568469167	4.05E-47	postive
TRAJ6	AC083862.1	0.503106237	1.14E-35	postive
TRAJ10	AC083862.1	0.503333996	1.05E-35	postive
TRAJ16	AC083862.1	0.503851795	8.70E-36	postive
TRAJ17	AC083862.1	0.502312413	1.52E-35	postive
TRAJ18	AC083862.1	0.525889676	2.22E-39	postive
TRAJ21	AC083862.1	0.512707302	3.36E-37	postive

TRAJ31	AC083862.1	0.531599365	2.35E-40	postive
TRAJ38	AC083862.1	0.530985325	3.00E-40	postive
TRAJ39	AC083862.1	0.507084094	2.68E-36	postive
CREB1	AC012557.1	0.799617379	3.86E-120	postive
RFXAP	AC012557.1	0.533620136	1.05E-40	postive
PSMD6	AC012557.1	0.544524276	1.25E-42	postive
UBR1	AC012557.1	0.647975943	5.07E-65	postive
ZC3HAV1	AC012557.1	0.574279621	2.89E-48	postive
IL15	AC012557.1	0.566408964	1.02E-46	postive
CYLD	AC012557.1	0.635308547	8.16E-62	postive
EIF2AK2	AC012557.1	0.58079914	1.41E-49	postive
MAPK8	AC012557.1	0.545119804	9.73E-43	postive
LMBR1	AC012557.1	0.570754996	1.44E-47	postive
IREB2	AC012557.1	0.60355033	2.13E-54	postive
DDX17	AC012557.1	0.634511977	1.28E-61	postive
PIK3CG	AC012557.1	0.528092854	9.38E-40	postive
JAK2	AC012557.1	0.661221205	1.53E-68	postive
TXK	AC012557.1	0.568317929	4.33E-47	postive
NFAT5	AC012557.1	0.867840535	4.84E-164	postive
NFATC3	AC012557.1	0.515070248	1.39E-37	postive
MALT1	AC012557.1	0.534569869	7.20E-41	postive
PIK3R1	AC012557.1	0.630974931	9.43E-61	postive
PIK3CA	AC012557.1	0.705172355	1.25E-81	postive
PIK3CB	AC012557.1	0.549388707	1.63E-43	postive
AKT3	AC012557.1	0.527753687	1.07E-39	postive
RASGRP3	AC012557.1	0.560403464	1.44E-45	postive
PLXNC1	AC012557.1	0.555565992	1.18E-44	postive
GNRH1	AC012557.1	0.763371401	3.12E-103	postive
IL6ST	AC012557.1	0.609739296	8.88E-56	postive
IL7	AC012557.1	0.504211312	7.63E-36	postive
RABEP1	AC012557.1	0.579224658	2.94E-49	postive
ACVR2A	AC012557.1	0.680143844	6.76E-74	postive
ANGPTL1	AC012557.1	0.749390113	1.68E-97	postive
BMPR1A	AC012557.1	0.551174906	7.67E-44	postive
BMPR2	AC012557.1	0.724197848	4.44E-88	postive
CRLF3	AC012557.1	0.670450335	4.19E-71	postive
NR1D2	AC012557.1	0.521484111	1.22E-38	postive
NR2C2	AC012557.1	0.745840732	4.17E-96	postive
RORA	AC012557.1	0.815584818	9.75E-129	postive
SOS1	AC012557.1	0.73015515	3.28E-90	postive
SOS2	AC012557.1	0.615335137	4.73E-57	postive
BRAF	AC012557.1	0.813384339	1.67E-127	postive
CBL	AC012557.1	0.61402476	9.44E-57	postive
CBLB	AC012557.1	0.707089193	2.95E-82	postive
RASGRP1	AC012557.1	0.543038063	2.30E-42	postive
PDK1	AC012557.1	0.551224929	7.51E-44	postive
TRAJ1	AC012557.1	0.86020159	5.22E-158	postive
TRAJ2	AC012557.1	0.764146409	1.46E-103	postive
TRAJ3	AC012557.1	0.843869004	3.12E-146	postive
TRAJ5	AC012557.1	0.900291692	1.13E-194	postive
TRAJ6	AC012557.1	0.884730494	7.80E-179	postive
TRAJ8	AC012557.1	0.889865923	8.51E-184	postive
TRAJ10	AC012557.1	0.854671566	7.34E-154	postive
TRAJ12	AC012557.1	0.842860289	1.50E-145	postive
TRAJ13	AC012557.1	0.896685905	8.81E-191	postive
TRAJ14	AC012557.1	0.88758145	1.47E-181	postive
TRAJ16	AC012557.1	0.893551513	1.64E-187	postive
TRAJ17	AC012557.1	0.867780771	5.41E-164	postive
TRAJ18	AC012557.1	0.80692052	5.68E-124	postive
TRAJ21	AC012557.1	0.907678881	3.87E-203	postive
TRAJ31	AC012557.1	0.920562344	9.22E-220	postive
TRAJ37	AC012557.1	0.880545744	5.81E-175	postive
TRAJ38	AC012557.1	0.929832426	1.43E-233	postive
TRAJ39	AC012557.1	0.915315708	1.13E-212	postive
CREB1	AC100830.2	0.782703498	7.61E-112	postive

RFXAP	AC100830.2	0.557564347	4.97E-45	postive
UBR1	AC100830.2	0.669849452	6.19E-71	postive
ZC3HAV1	AC100830.2	0.561653776	8.35E-46	postive
ZC3HAV1L	AC100830.2	0.558354538	3.53E-45	postive
IL15	AC100830.2	0.594202165	2.27E-52	postive
CYLD	AC100830.2	0.597190362	5.18E-53	postive
EIF2AK2	AC100830.2	0.507659933	2.17E-36	postive
MAPK8	AC100830.2	0.589291419	2.48E-51	postive
LMBR1	AC100830.2	0.568228425	4.51E-47	postive
IREB2	AC100830.2	0.653532154	1.77E-66	postive
DDX17	AC100830.2	0.653389739	1.94E-66	postive
PIK3CG	AC100830.2	0.512081468	4.25E-37	postive
JAK2	AC100830.2	0.604161334	1.56E-54	postive
TXK	AC100830.2	0.590168064	1.62E-51	postive
NFAT5	AC100830.2	0.877702232	2.06E-172	postive
NFATC3	AC100830.2	0.535868106	4.27E-41	postive
MALT1	AC100830.2	0.542359989	3.04E-42	postive
PIK3R1	AC100830.2	0.642235099	1.50E-63	postive
PIK3CA	AC100830.2	0.651271707	7.00E-66	postive
AKT3	AC100830.2	0.547886001	3.07E-43	postive
RASGRP3	AC100830.2	0.51044625	7.79E-37	postive
IGHD4-4	AC100830.2	0.596294831	8.08E-53	postive
IGHD6-6	AC100830.2	0.545159852	9.57E-43	postive
LTB4R2	AC100830.2	0.504449034	7.00E-36	postive
GNRH1	AC100830.2	0.799388152	5.06E-120	postive
IL6ST	AC100830.2	0.630943491	9.60E-61	postive
RABEP1	AC100830.2	0.602915212	2.94E-54	postive
ACVR2A	AC100830.2	0.669320145	8.72E-71	postive
ANGPTL1	AC100830.2	0.743852038	2.46E-95	postive
BMPR1A	AC100830.2	0.586216506	1.08E-50	postive
BMPR2	AC100830.2	0.722297763	2.07E-87	postive
CRLF3	AC100830.2	0.631315779	7.79E-61	postive
NR2C1	AC100830.2	0.511762783	4.78E-37	postive
NR2C2	AC100830.2	0.735726843	2.95E-92	postive
NR3C1	AC100830.2	0.50659932	3.20E-36	postive
RORA	AC100830.2	0.849764899	2.54E-150	postive
SOS1	AC100830.2	0.680078076	7.07E-74	postive
SOS2	AC100830.2	0.599523142	1.62E-53	postive
BRAF	AC100830.2	0.797331948	5.66E-119	postive
TEC	AC100830.2	0.501370146	2.13E-35	postive
CBL	AC100830.2	0.61150305	3.54E-56	postive
CBLB	AC100830.2	0.673996972	4.10E-72	postive
RASGRP1	AC100830.2	0.516475811	8.19E-38	postive
PDK1	AC100830.2	0.5933856	3.38E-52	postive
TRAJ1	AC100830.2	0.732847035	3.42E-91	postive
TRAJ2	AC100830.2	0.749112017	2.17E-97	postive
TRAJ3	AC100830.2	0.817032971	1.47E-129	postive
TRAJ5	AC100830.2	0.77354914	1.17E-107	postive
TRAJ6	AC100830.2	0.802513199	1.22E-121	postive
TRAJ8	AC100830.2	0.768063654	3.03E-105	postive
TRAJ10	AC100830.2	0.794775998	1.10E-117	postive
TRAJ12	AC100830.2	0.69813423	2.25E-79	postive
TRAJ13	AC100830.2	0.778940437	4.23E-110	postive
TRAJ14	AC100830.2	0.75260673	8.73E-99	postive
TRAJ16	AC100830.2	0.763604459	2.49E-103	postive
TRAJ17	AC100830.2	0.744930958	9.41E-96	postive
TRAJ18	AC100830.2	0.719117629	2.64E-86	postive
TRAJ21	AC100830.2	0.800535585	1.30E-120	postive
TRAJ31	AC100830.2	0.824472532	6.81E-134	postive
TRAJ37	AC100830.2	0.839454513	2.79E-143	postive
TRAJ38	AC100830.2	0.839827814	1.58E-143	postive
TRAJ39	AC100830.2	0.829549317	5.69E-137	postive
CREB1	INE1	0.58018815	1.88E-49	postive
CYLD	INE1	0.522345157	8.75E-39	postive
NFKBIZ	INE1	0.523925164	4.76E-39	postive

DDX17	INE1	0.669297188	8.85E-71	postive
TXK	INE1	0.508511161	1.59E-36	postive
NFAT5	INE1	0.711016414	1.48E-83	postive
LTB4R2	INE1	0.575787413	1.45E-48	postive
GNRH1	INE1	0.753272654	4.71E-99	postive
ANGPTL1	INE1	0.559591901	2.06E-45	postive
NR2C1	INE1	0.577806941	5.69E-49	postive
NR2C2	INE1	0.737776618	5.06E-93	postive
RORA	INE1	0.658799834	6.93E-68	postive
SOS1	INE1	0.589973268	1.78E-51	postive
BRAF	INE1	0.64204717	1.68E-63	postive
CBL	INE1	0.517246314	6.12E-38	postive
CBLB	INE1	0.645753567	1.90E-64	postive
TRAJ1	INE1	0.624984082	2.61E-59	postive
TRAJ2	INE1	0.565334856	1.64E-46	postive
TRAJ3	INE1	0.637427006	2.43E-62	postive
TRAJ5	INE1	0.613194784	1.46E-56	postive
TRAJ6	INE1	0.623583497	5.62E-59	postive
TRAJ8	INE1	0.554994082	1.50E-44	postive
TRAJ10	INE1	0.608655662	1.56E-55	postive
TRAJ12	INE1	0.587374185	6.23E-51	postive
TRAJ13	INE1	0.592302242	5.75E-52	postive
TRAJ14	INE1	0.584588732	2.36E-50	postive
TRAJ16	INE1	0.614460302	7.50E-57	postive
TRAJ17	INE1	0.568585472	3.84E-47	postive
TRAJ18	INE1	0.603432319	2.26E-54	postive
TRAJ21	INE1	0.616162139	3.05E-57	postive
TRAJ31	INE1	0.623853701	4.85E-59	postive
TRAJ37	INE1	0.623937875	4.63E-59	postive
TRAJ38	INE1	0.657498057	1.55E-67	postive
TRAJ39	INE1	0.615078675	5.41E-57	postive
CREB1	PAXIP1-AS2	0.52703622	1.42E-39	postive
CIITA	PAXIP1-AS2	0.559427354	2.21E-45	postive
CYLD	PAXIP1-AS2	0.622935026	8.00E-59	postive
TRIM22	PAXIP1-AS2	0.504507704	6.86E-36	postive
NFAT5	PAXIP1-AS2	0.544604091	1.20E-42	postive
NFATC3	PAXIP1-AS2	0.539979393	8.07E-42	postive
PIK3R1	PAXIP1-AS2	0.562830217	4.98E-46	postive
NR3C2	PAXIP1-AS2	0.512657434	3.43E-37	postive
RORA	PAXIP1-AS2	0.539213698	1.10E-41	postive
CD1D	LINC00996	0.524523839	3.77E-39	postive
CD4	LINC00996	0.58224106	7.15E-50	postive
HLA-DMB	LINC00996	0.500888462	2.53E-35	postive
HLA-DQA1	LINC00996	0.50555406	4.69E-36	postive
LTA	LINC00996	0.618847349	7.27E-58	postive
CIITA	LINC00996	0.643517735	7.09E-64	postive
IFI30	LINC00996	0.502277517	1.53E-35	postive
APOBEC3G	LINC00996	0.503558696	9.67E-36	postive
RBP5	LINC00996	0.524820643	3.36E-39	postive
CYBB	LINC00996	0.562956607	4.71E-46	postive
TLR7	LINC00996	0.573848088	3.53E-48	postive
TLR8	LINC00996	0.542978297	2.36E-42	postive
IL7R	LINC00996	0.503527587	9.78E-36	postive
CD40LG	LINC00996	0.581206299	1.16E-49	postive
TRIM22	LINC00996	0.554892853	1.57E-44	postive
PIK3CG	LINC00996	0.51317417	2.83E-37	postive
FASLG	LINC00996	0.508081608	1.86E-36	postive
CCR7	LINC00996	0.529741402	4.91E-40	postive
CCR8	LINC00996	0.510874044	6.65E-37	postive
CCR5	LINC00996	0.644287513	4.51E-64	postive
CXCR6	LINC00996	0.564074678	2.87E-46	postive
CCR4	LINC00996	0.534499886	7.40E-41	postive
CD86	LINC00996	0.513500072	2.50E-37	postive
BTK	LINC00996	0.669304383	8.81E-71	postive
NFATC2	LINC00996	0.509804621	9.87E-37	postive

PIK3R5	LINC00996	0.585008458	1.93E-50	postive
PIK3CD	LINC00996	0.600460487	1.01E-53	postive
INPP5D	LINC00996	0.5856331	1.43E-50	postive
CD22	LINC00996	0.512525757	3.60E-37	postive
CD72	LINC00996	0.558392064	3.47E-45	postive
PLCG2	LINC00996	0.554501301	1.86E-44	postive
PRKCB	LINC00996	0.592903137	4.28E-52	postive
SEMA4D	LINC00996	0.526160498	2.00E-39	postive
CMKLR1	LINC00996	0.586964771	7.59E-51	postive
PLXNC1	LINC00996	0.539483299	9.88E-42	postive
FLT3LG	LINC00996	0.514269032	1.88E-37	postive
IL16	LINC00996	0.658826196	6.82E-68	postive
TNFSF13B	LINC00996	0.522696653	7.64E-39	postive
CSF2RB	LINC00996	0.658427914	8.73E-68	postive
IL10RA	LINC00996	0.691122138	3.43E-77	postive
IL12RB1	LINC00996	0.662976196	5.05E-69	postive
IL2RB	LINC00996	0.593982396	2.52E-52	postive
IL18RAP	LINC00996	0.57987627	2.17E-49	postive
IL21R	LINC00996	0.660327816	2.67E-68	postive
PTGER4	LINC00996	0.542453706	2.93E-42	postive
ITGAL	LINC00996	0.718484287	4.36E-86	postive
LCK	LINC00996	0.57134724	1.10E-47	postive
NCR3	LINC00996	0.528408116	8.29E-40	postive
CD247	LINC00996	0.666702199	4.71E-70	postive
ZAP70	LINC00996	0.680929536	3.97E-74	postive
LCP2	LINC00996	0.661388643	1.37E-68	postive
CD244	LINC00996	0.523525138	5.55E-39	postive
SH2D1A	LINC00996	0.635484766	7.38E-62	postive
CD3D	LINC00996	0.504532941	6.79E-36	postive
CD3E	LINC00996	0.599965066	1.30E-53	postive
CD3G	LINC00996	0.61277849	1.82E-56	postive
PTPRC	LINC00996	0.671298197	2.41E-71	postive
ITK	LINC00996	0.599174079	1.93E-53	postive
GRAP2	LINC00996	0.561383666	9.40E-46	postive
CD28	LINC00996	0.59173302	7.58E-52	postive
ICOS	LINC00996	0.684496618	3.47E-75	postive
CTLA4	LINC00996	0.58344805	4.05E-50	postive
TRAC	LINC00996	0.610704972	5.37E-56	postive
TRAV4	LINC00996	0.561637275	8.41E-46	postive
TRAV8-2	LINC00996	0.599791057	1.42E-53	postive
TRAV8-3	LINC00996	0.625227943	2.28E-59	postive
TRAV8-4	LINC00996	0.57633358	1.13E-48	postive
TRAV8-6	LINC00996	0.588452913	3.71E-51	postive
TRAV9-2	LINC00996	0.578652535	3.84E-49	postive
TRAV13-2	LINC00996	0.506552381	3.26E-36	postive
TRAV17	LINC00996	0.522856504	7.19E-39	postive
TRAV21	LINC00996	0.527612739	1.13E-39	postive
TRAV29DV5	LINC00996	0.509701945	1.03E-36	postive
TRBC1	LINC00996	0.680328442	5.97E-74	postive
TRBC2	LINC00996	0.545555003	8.12E-43	postive
TRBJ2-2	LINC00996	0.505474683	4.83E-36	postive
TRBJ2-3	LINC00996	0.500689993	2.71E-35	postive
TRBJ2-7	LINC00996	0.60942116	1.05E-55	postive
TRBV2	LINC00996	0.55824796	3.69E-45	postive
TRBV5-1	LINC00996	0.526449622	1.78E-39	postive
TRBV5-4	LINC00996	0.546900845	4.63E-43	postive
TRBV6-5	LINC00996	0.506144366	3.78E-36	postive
TRBV6-6	LINC00996	0.548905171	2.00E-43	postive
TRBV18	LINC00996	0.559137467	2.51E-45	postive
TRBV19	LINC00996	0.540722462	5.96E-42	postive
TRBV20-1	LINC00996	0.593266252	3.59E-52	postive
TRBV28	LINC00996	0.567679574	5.77E-47	postive
CREB1	MACC1-AS1	0.753015597	5.97E-99	postive
RFXAP	MACC1-AS1	0.513016199	3.00E-37	postive
UBR1	MACC1-AS1	0.619066802	6.47E-58	postive

ZC3HAV1	MACC1-AS1	0.513230463	2.77E-37	postive
IL15	MACC1-AS1	0.565967766	1.24E-46	postive
CYLD	MACC1-AS1	0.599275416	1.83E-53	postive
MAPK8	MACC1-AS1	0.520654378	1.67E-38	postive
LMBR1	MACC1-AS1	0.541293458	4.72E-42	postive
IREB2	MACC1-AS1	0.595783648	1.04E-52	postive
DDX17	MACC1-AS1	0.525566491	2.52E-39	postive
JAK2	MACC1-AS1	0.585921495	1.25E-50	postive
NFAT5	MACC1-AS1	0.811542863	1.75E-126	postive
NFATC3	MACC1-AS1	0.506085084	3.86E-36	postive
PIK3R1	MACC1-AS1	0.607585228	2.70E-55	postive
PIK3CA	MACC1-AS1	0.648213655	4.40E-65	postive
RASGRP3	MACC1-AS1	0.511669678	4.95E-37	postive
PLXNC1	MACC1-AS1	0.510853651	6.70E-37	postive
GNRH1	MACC1-AS1	0.619346191	5.57E-58	postive
IL6ST	MACC1-AS1	0.603008575	2.80E-54	postive
RABEP1	MACC1-AS1	0.514099097	2.00E-37	postive
ACVR2A	MACC1-AS1	0.656537673	2.81E-67	postive
ANGPTL1	MACC1-AS1	0.670918313	3.09E-71	postive
BMPR1A	MACC1-AS1	0.569197848	2.91E-47	postive
BMPR2	MACC1-AS1	0.703774271	3.54E-81	postive
CRLF3	MACC1-AS1	0.584723846	2.21E-50	postive
NR1D2	MACC1-AS1	0.534867275	6.39E-41	postive
NR2C2	MACC1-AS1	0.638960054	1.01E-62	postive
RORA	MACC1-AS1	0.789804636	3.11E-115	postive
SOS1	MACC1-AS1	0.656413707	3.04E-67	postive
SOS2	MACC1-AS1	0.567385623	6.58E-47	postive
BRAF	MACC1-AS1	0.747425995	1.00E-96	postive
CBLB	MACC1-AS1	0.618716877	7.80E-58	postive
PDK1	MACC1-AS1	0.500814131	2.60E-35	postive
TRAJ1	MACC1-AS1	0.75116564	3.30E-98	postive
TRAJ2	MACC1-AS1	0.778723752	5.32E-110	postive
TRAJ3	MACC1-AS1	0.812194832	7.64E-127	postive
TRAJ5	MACC1-AS1	0.776786335	4.07E-109	postive
TRAJ6	MACC1-AS1	0.816056202	5.28E-129	postive
TRAJ8	MACC1-AS1	0.825785235	1.11E-134	postive
TRAJ10	MACC1-AS1	0.806720128	7.27E-124	postive
TRAJ12	MACC1-AS1	0.785300265	4.54E-113	postive
TRAJ13	MACC1-AS1	0.79702359	8.11E-119	postive
TRAJ14	MACC1-AS1	0.807954281	1.58E-124	postive
TRAJ16	MACC1-AS1	0.829413128	6.90E-137	postive
TRAJ17	MACC1-AS1	0.804580027	1.00E-122	postive
TRAJ18	MACC1-AS1	0.760319358	6.01E-102	postive
TRAJ21	MACC1-AS1	0.814897834	2.38E-128	postive
TRAJ31	MACC1-AS1	0.857962693	2.62E-156	postive
TRAJ37	MACC1-AS1	0.841232209	1.85E-144	postive
TRAJ38	MACC1-AS1	0.882118776	2.12E-176	postive
TRAJ39	MACC1-AS1	0.835731483	7.35E-141	postive
BPIFB1	AL163051.1	0.524936081	3.21E-39	postive
CREB1	TMEM161B-AS1	0.675656007	1.37E-72	postive
RFXAP	TMEM161B-AS1	0.542351366	3.05E-42	postive
UBR1	TMEM161B-AS1	0.607452684	2.89E-55	postive
IL15	TMEM161B-AS1	0.560869396	1.18E-45	postive
CYLD	TMEM161B-AS1	0.591489602	8.54E-52	postive
IREB2	TMEM161B-AS1	0.529598997	5.19E-40	postive
DDX17	TMEM161B-AS1	0.678401238	2.19E-73	postive
JAK2	TMEM161B-AS1	0.576429913	1.08E-48	postive
TXK	TMEM161B-AS1	0.530653934	3.42E-40	postive
NFAT5	TMEM161B-AS1	0.77767596	1.60E-109	postive
PIK3R1	TMEM161B-AS1	0.569801707	2.22E-47	postive
PIK3CA	TMEM161B-AS1	0.531256417	2.70E-40	postive
GNRH1	TMEM161B-AS1	0.755589833	5.40E-100	postive
IL6ST	TMEM161B-AS1	0.545102582	9.80E-43	postive
ACVR2A	TMEM161B-AS1	0.567198813	7.15E-47	postive
ANGPTL1	TMEM161B-AS1	0.653972235	1.36E-66	postive

BMPR2	TMEM161B-AS1	0.547248557	4.01E-43	postive
CRLF3	TMEM161B-AS1	0.565054572	1.86E-46	postive
NR2C2	TMEM161B-AS1	0.712589038	4.41E-84	postive
RORA	TMEM161B-AS1	0.736117481	2.11E-92	postive
SOS1	TMEM161B-AS1	0.577000309	8.27E-49	postive
BRAF	TMEM161B-AS1	0.71109186	1.40E-83	postive
CBLB	TMEM161B-AS1	0.613904596	1.01E-56	postive
PDK1	TMEM161B-AS1	0.536997926	2.71E-41	postive
TRAJ1	TMEM161B-AS1	0.707587073	2.02E-82	postive
TRAJ2	TMEM161B-AS1	0.708507806	1.01E-82	postive
TRAJ3	TMEM161B-AS1	0.757729466	7.15E-101	postive
TRAJ5	TMEM161B-AS1	0.706966374	3.23E-82	postive
TRAJ6	TMEM161B-AS1	0.75073604	4.90E-98	postive
TRAJ8	TMEM161B-AS1	0.689722578	9.18E-77	postive
TRAJ10	TMEM161B-AS1	0.728906847	9.27E-90	postive
TRAJ12	TMEM161B-AS1	0.715311069	5.31E-85	postive
TRAJ13	TMEM161B-AS1	0.699083835	1.12E-79	postive
TRAJ14	TMEM161B-AS1	0.727948467	2.05E-89	postive
TRAJ16	TMEM161B-AS1	0.711582534	9.59E-84	postive
TRAJ17	TMEM161B-AS1	0.676542166	7.59E-73	postive
TRAJ18	TMEM161B-AS1	0.706111549	6.16E-82	postive
TRAJ21	TMEM161B-AS1	0.737121497	8.90E-93	postive
TRAJ31	TMEM161B-AS1	0.738342392	3.10E-93	postive
TRAJ37	TMEM161B-AS1	0.744673455	1.18E-95	postive
TRAJ38	TMEM161B-AS1	0.793593564	4.26E-117	postive
TRAJ39	TMEM161B-AS1	0.750456558	6.33E-98	postive
GNRH1	KRT7-AS	0.549161121	1.80E-43	postive
NR2C2	KRT7-AS	0.530558701	3.55E-40	postive
DDX17	AL021707.4	0.733618384	1.78E-91	postive
GNRH1	AL021707.4	0.558142794	3.87E-45	postive
NR2C2	AL021707.4	0.545560613	8.10E-43	postive
BRAF	AL021707.4	0.510903077	6.58E-37	postive
DDX17	AC145423.3	0.612378107	2.24E-56	postive
NFAT5	AC145423.3	0.635677184	6.61E-62	postive
LTB4R2	AC145423.3	0.544066533	1.51E-42	postive
GNRH1	AC145423.3	0.734234119	1.05E-91	postive
NR2C1	AC145423.3	0.550864943	8.75E-44	postive
NR2C2	AC145423.3	0.65590262	4.16E-67	postive
RORA	AC145423.3	0.542310473	3.11E-42	postive
BRAF	AC145423.3	0.559708182	1.96E-45	postive
TRAJ1	AC145423.3	0.56347939	3.74E-46	postive
TRAJ2	AC145423.3	0.516104065	9.42E-38	postive
TRAJ3	AC145423.3	0.60818027	1.99E-55	postive
TRAJ5	AC145423.3	0.530409324	3.77E-40	postive
TRAJ6	AC145423.3	0.579596657	2.47E-49	postive
TRAJ8	AC145423.3	0.538326047	1.58E-41	postive
TRAJ10	AC145423.3	0.567425614	6.46E-47	postive
TRAJ12	AC145423.3	0.521566963	1.18E-38	postive
TRAJ13	AC145423.3	0.534329459	7.92E-41	postive
TRAJ14	AC145423.3	0.539170003	1.12E-41	postive
TRAJ16	AC145423.3	0.540772758	5.84E-42	postive
TRAJ17	AC145423.3	0.527316781	1.27E-39	postive
TRAJ18	AC145423.3	0.526700201	1.62E-39	postive
TRAJ21	AC145423.3	0.566170654	1.13E-46	postive
TRAJ31	AC145423.3	0.552573011	4.24E-44	postive
TRAJ37	AC145423.3	0.583286019	4.37E-50	postive
TRAJ38	AC145423.3	0.568126201	4.72E-47	postive
TRAJ39	AC145423.3	0.580179802	1.88E-49	postive
BMPR1B	LINC00578	0.513402543	2.60E-37	postive
CREB1	AC133644.1	0.583160452	4.64E-50	postive
IL15	AC133644.1	0.637987798	1.76E-62	postive
EIF2AK2	AC133644.1	0.53886238	1.27E-41	postive
LIMS1	AC133644.1	0.511637375	5.01E-37	postive
PIK3CG	AC133644.1	0.514744911	1.57E-37	postive
JAK2	AC133644.1	0.617565355	1.44E-57	postive

NFAT5	AC133644.1	0.588482916	3.66E-51	postive
PIK3CA	AC133644.1	0.58423559	2.79E-50	postive
RASGRP3	AC133644.1	0.515010257	1.42E-37	postive
GNRH1	AC133644.1	0.525872517	2.23E-39	postive
ANGPTL1	AC133644.1	0.679195926	1.28E-73	postive
BMPR2	AC133644.1	0.516317396	8.69E-38	postive
CRLF3	AC133644.1	0.611950157	2.81E-56	postive
NR2C2	AC133644.1	0.510684875	7.13E-37	postive
RORA	AC133644.1	0.610576585	5.75E-56	postive
SOS1	AC133644.1	0.545269401	9.14E-43	postive
BRAF	AC133644.1	0.602963636	2.86E-54	postive
PTPRC	AC133644.1	0.507236253	2.54E-36	postive
ITK	AC133644.1	0.569851386	2.17E-47	postive
CBLB	AC133644.1	0.642255823	1.48E-63	postive
PDK1	AC133644.1	0.571091101	1.24E-47	postive
TRAJ1	AC133644.1	0.617910696	1.20E-57	postive
TRAJ2	AC133644.1	0.561479139	9.02E-46	postive
TRAJ3	AC133644.1	0.63462281	1.21E-61	postive
TRAJ5	AC133644.1	0.689911422	8.04E-77	postive
TRAJ6	AC133644.1	0.635595589	6.93E-62	postive
TRAJ8	AC133644.1	0.611880249	2.91E-56	postive
TRAJ10	AC133644.1	0.637899486	1.85E-62	postive
TRAJ12	AC133644.1	0.588783527	3.17E-51	postive
TRAJ13	AC133644.1	0.63342091	2.38E-61	postive
TRAJ14	AC133644.1	0.647492717	6.76E-65	postive
TRAJ16	AC133644.1	0.663127966	4.59E-69	postive
TRAJ17	AC133644.1	0.654171475	1.20E-66	postive
TRAJ18	AC133644.1	0.624682427	3.08E-59	postive
TRAJ21	AC133644.1	0.667297893	3.22E-70	postive
TRAJ31	AC133644.1	0.682228034	1.64E-74	postive
TRAJ37	AC133644.1	0.608386811	1.79E-55	postive
TRAJ38	AC133644.1	0.710047534	3.11E-83	postive
TRAJ39	AC133644.1	0.67378088	4.73E-72	postive
NFKBIZ	AC008735.4	0.506844796	2.93E-36	postive
DDX17	AC008735.4	0.652741867	2.87E-66	postive
NFAT5	AC008735.4	0.605903108	6.41E-55	postive
LTB4R2	AC008735.4	0.576561963	1.01E-48	postive
GNRH1	AC008735.4	0.713133746	2.89E-84	postive
NR2C1	AC008735.4	0.560561196	1.35E-45	postive
NR2C2	AC008735.4	0.708597511	9.40E-83	postive
RORA	AC008735.4	0.572517105	6.48E-48	postive
SOS1	AC008735.4	0.542506245	2.87E-42	postive
BRAF	AC008735.4	0.601495848	6.01E-54	postive
CBL	AC008735.4	0.510520662	7.58E-37	postive
CBLB	AC008735.4	0.564129302	2.80E-46	postive
TRAJ1	AC008735.4	0.565255895	1.70E-46	postive
TRAJ2	AC008735.4	0.545653673	7.79E-43	postive
TRAJ3	AC008735.4	0.607612729	2.67E-55	postive
TRAJ5	AC008735.4	0.534822955	6.50E-41	postive
TRAJ6	AC008735.4	0.550690286	9.42E-44	postive
TRAJ8	AC008735.4	0.53109697	2.87E-40	postive
TRAJ10	AC008735.4	0.515915153	1.01E-37	postive
TRAJ12	AC008735.4	0.526094592	2.05E-39	postive
TRAJ13	AC008735.4	0.530371231	3.83E-40	postive
TRAJ14	AC008735.4	0.538812112	1.30E-41	postive
TRAJ16	AC008735.4	0.536970528	2.74E-41	postive
TRAJ17	AC008735.4	0.505853186	4.20E-36	postive
TRAJ18	AC008735.4	0.515683993	1.10E-37	postive
TRAJ21	AC008735.4	0.544955861	1.04E-42	postive
TRAJ31	AC008735.4	0.541683123	4.02E-42	postive
TRAJ37	AC008735.4	0.5546384	1.75E-44	postive
TRAJ38	AC008735.4	0.583725021	3.55E-50	postive
TRAJ39	AC008735.4	0.551114782	7.87E-44	postive
CREB1	ANKRD10-IT1	0.734923589	5.86E-92	postive
RFXAP	ANKRD10-IT1	0.56368564	3.41E-46	postive

UBR1	ANKRD10-IT1	0.600287831	1.10E-53	postive
ZC3HAV1	ANKRD10-IT1	0.545255615	9.19E-43	postive
IL15	ANKRD10-IT1	0.542349125	3.06E-42	postive
CYLD	ANKRD10-IT1	0.562073893	6.94E-46	postive
EIF2AK2	ANKRD10-IT1	0.516157652	9.23E-38	postive
MAPK8	ANKRD10-IT1	0.53164065	2.31E-40	postive
NFKBIZ	ANKRD10-IT1	0.5111526	6.00E-37	postive
IREB2	ANKRD10-IT1	0.572067639	7.95E-48	postive
DDX17	ANKRD10-IT1	0.698991838	1.20E-79	postive
JAK2	ANKRD10-IT1	0.610979444	4.66E-56	postive
TXK	ANKRD10-IT1	0.601113793	7.29E-54	postive
NFAT5	ANKRD10-IT1	0.759078373	1.98E-101	postive
MALT1	ANKRD10-IT1	0.523206912	6.28E-39	postive
PIK3R1	ANKRD10-IT1	0.568459917	4.06E-47	postive
PIK3CA	ANKRD10-IT1	0.662495542	6.84E-69	postive
PIK3CB	ANKRD10-IT1	0.50234817	1.50E-35	postive
RASGRP3	ANKRD10-IT1	0.509284769	1.20E-36	postive
LTB4R2	ANKRD10-IT1	0.518123751	4.39E-38	postive
GNRH1	ANKRD10-IT1	0.797455982	4.90E-119	postive
IL6ST	ANKRD10-IT1	0.538508772	1.47E-41	postive
RABEP1	ANKRD10-IT1	0.537726306	2.02E-41	postive
ACVR2A	ANKRD10-IT1	0.641008266	3.07E-63	postive
ANGPTL1	ANKRD10-IT1	0.694287426	3.61E-78	postive
BMPR1A	ANKRD10-IT1	0.507102871	2.66E-36	postive
BMPR2	ANKRD10-IT1	0.692220874	1.57E-77	postive
CRLF3	ANKRD10-IT1	0.568493717	4.00E-47	postive
NR2C1	ANKRD10-IT1	0.562028603	7.08E-46	postive
NR2C2	ANKRD10-IT1	0.754800889	1.13E-99	postive
RORA	ANKRD10-IT1	0.75277935	7.44E-99	postive
SOS1	ANKRD10-IT1	0.67874755	1.73E-73	postive
SOS2	ANKRD10-IT1	0.597139039	5.32E-53	postive
BRAF	ANKRD10-IT1	0.717098119	1.31E-85	postive
CBL	ANKRD10-IT1	0.589756132	1.98E-51	postive
CBLB	ANKRD10-IT1	0.725205088	1.95E-88	postive
PDK1	ANKRD10-IT1	0.557688661	4.71E-45	postive
TRAJ1	ANKRD10-IT1	0.670041436	5.46E-71	postive
TRAJ2	ANKRD10-IT1	0.618734237	7.73E-58	postive
TRAJ3	ANKRD10-IT1	0.704964397	1.46E-81	postive
TRAJ5	ANKRD10-IT1	0.680538931	5.18E-74	postive
TRAJ6	ANKRD10-IT1	0.691394172	2.83E-77	postive
TRAJ8	ANKRD10-IT1	0.644430915	4.14E-64	postive
TRAJ10	ANKRD10-IT1	0.677837596	3.19E-73	postive
TRAJ12	ANKRD10-IT1	0.634566318	1.24E-61	postive
TRAJ13	ANKRD10-IT1	0.65321766	2.15E-66	postive
TRAJ14	ANKRD10-IT1	0.648127669	4.63E-65	postive
TRAJ16	ANKRD10-IT1	0.69922596	1.01E-79	postive
TRAJ17	ANKRD10-IT1	0.66520541	1.23E-69	postive
TRAJ18	ANKRD10-IT1	0.650257091	1.29E-65	postive
TRAJ21	ANKRD10-IT1	0.68896339	1.56E-76	postive
TRAJ31	ANKRD10-IT1	0.710972366	1.53E-83	postive
TRAJ37	ANKRD10-IT1	0.675250796	1.79E-72	postive
TRAJ38	ANKRD10-IT1	0.74904357	2.30E-97	postive
TRAJ39	ANKRD10-IT1	0.703132072	5.71E-81	postive
CREB1	AL031667.3	0.546719098	5.00E-43	postive
NFAT5	AL031667.3	0.592999153	4.09E-52	postive
GNRH1	AL031667.3	0.506435849	3.40E-36	postive
ANGPTL1	AL031667.3	0.515527472	1.17E-37	postive
CRLF3	AL031667.3	0.505415512	4.93E-36	postive
RORA	AL031667.3	0.574059249	3.20E-48	postive
BRAF	AL031667.3	0.588830609	3.09E-51	postive
CBLB	AL031667.3	0.515893239	1.02E-37	postive
TRAJ2	AL031667.3	0.536084458	3.92E-41	postive
TRAJ3	AL031667.3	0.539335591	1.05E-41	postive
TRAJ5	AL031667.3	0.541062806	5.18E-42	postive
TRAJ6	AL031667.3	0.54933804	1.67E-43	postive

TRAJ8	AL031667.3	0.551386252	7.01E-44	postive
TRAJ10	AL031667.3	0.521878784	1.05E-38	postive
TRAJ13	AL031667.3	0.554817408	1.62E-44	postive
TRAJ14	AL031667.3	0.529079369	6.37E-40	postive
TRAJ16	AL031667.3	0.535252303	5.47E-41	postive
TRAJ17	AL031667.3	0.502694706	1.32E-35	postive
TRAJ21	AL031667.3	0.560057771	1.68E-45	postive
TRAJ31	AL031667.3	0.568761788	3.55E-47	postive
TRAJ37	AL031667.3	0.572037254	8.06E-48	postive
TRAJ38	AL031667.3	0.584694535	2.24E-50	postive
TRAJ39	AL031667.3	0.581186528	1.17E-49	postive
GIPR	AC108134.1	0.529652745	5.08E-40	postive
CREB1	AC011477.2	0.546949752	4.54E-43	postive
RFXAP	AC011477.2	0.534192876	8.37E-41	postive
NR2C2	AC011477.2	0.577948481	5.33E-49	postive
BRAF	AC011477.2	0.549393596	1.63E-43	postive
CIITA	AL031600.1	0.507510707	2.29E-36	postive
LMBR1L	AL031600.1	0.517099173	6.47E-38	postive
TYK2	AL031600.1	0.621112643	2.15E-58	postive
IRF9	AL031600.1	0.60351818	2.16E-54	postive
LTB4R2	AL031600.1	0.561811411	7.79E-46	postive
TNFRSF10A	AC100861.1	0.681986404	1.94E-74	postive
TNFRSF10B	AC100861.1	0.517822563	4.92E-38	postive
CREB1	CD44-AS1	0.740968711	3.14E-94	postive
UBR1	CD44-AS1	0.574754692	2.33E-48	postive
ZC3HAV1	CD44-AS1	0.544136525	1.46E-42	postive
IL15	CD44-AS1	0.660876597	1.89E-68	postive
CYLD	CD44-AS1	0.618466145	8.92E-58	postive
EIF2AK2	CD44-AS1	0.52540897	2.68E-39	postive
NFKBIZ	CD44-AS1	0.502764828	1.29E-35	postive
LMBR1	CD44-AS1	0.539078498	1.17E-41	postive
LIMS1	CD44-AS1	0.501933796	1.74E-35	postive
IREB2	CD44-AS1	0.527481424	1.19E-39	postive
DDX17	CD44-AS1	0.60337311	2.33E-54	postive
PIK3CG	CD44-AS1	0.520918884	1.51E-38	postive
JAK2	CD44-AS1	0.643980616	5.40E-64	postive
TXK	CD44-AS1	0.569394357	2.67E-47	postive
NFAT5	CD44-AS1	0.837536553	5.02E-142	postive
MALT1	CD44-AS1	0.543832385	1.66E-42	postive
PIK3R1	CD44-AS1	0.608604449	1.60E-55	postive
PIK3CA	CD44-AS1	0.68489211	2.65E-75	postive
PIK3CB	CD44-AS1	0.53680134	2.93E-41	postive
RASGRP3	CD44-AS1	0.532467478	1.67E-40	postive
IGHD6-6	CD44-AS1	0.539193038	1.11E-41	postive
PLXNC1	CD44-AS1	0.509707416	1.02E-36	postive
GNRH1	CD44-AS1	0.725114444	2.10E-88	postive
IL6ST	CD44-AS1	0.591209729	9.78E-52	postive
IL7	CD44-AS1	0.541198145	4.90E-42	postive
RABEP1	CD44-AS1	0.518162662	4.33E-38	postive
ACVR2A	CD44-AS1	0.591543158	8.32E-52	postive
ANGPTL1	CD44-AS1	0.749160511	2.07E-97	postive
BMPR2	CD44-AS1	0.67072262	3.51E-71	postive
CRLF3	CD44-AS1	0.60703707	3.58E-55	postive
IL18R1	CD44-AS1	0.533662607	1.03E-40	postive
NR2C2	CD44-AS1	0.68890291	1.63E-76	postive
RORA	CD44-AS1	0.810358459	7.81E-126	postive
SOS1	CD44-AS1	0.645130286	2.74E-64	postive
SOS2	CD44-AS1	0.580223524	1.85E-49	postive
BRAF	CD44-AS1	0.746827182	1.72E-96	postive
ITK	CD44-AS1	0.537551673	2.16E-41	postive
CBL	CD44-AS1	0.623965311	4.56E-59	postive
CBLB	CD44-AS1	0.677790463	3.29E-73	postive
RASGRP1	CD44-AS1	0.543832447	1.66E-42	postive
PDK1	CD44-AS1	0.568849598	3.41E-47	postive
TRAJ1	CD44-AS1	0.798656152	1.20E-119	postive

TRAJ2	CD44-AS1	0.705838394	7.56E-82	postive
TRAJ3	CD44-AS1	0.840405474	6.57E-144	postive
TRAJ5	CD44-AS1	0.854180636	1.68E-153	postive
TRAJ6	CD44-AS1	0.841225722	1.87E-144	postive
TRAJ8	CD44-AS1	0.793351806	5.62E-117	postive
TRAJ10	CD44-AS1	0.85759948	4.91E-156	postive
TRAJ12	CD44-AS1	0.772525194	3.33E-107	postive
TRAJ13	CD44-AS1	0.824280702	8.86E-134	postive
TRAJ14	CD44-AS1	0.818651037	1.75E-130	postive
TRAJ16	CD44-AS1	0.850802943	4.64E-151	postive
TRAJ17	CD44-AS1	0.853179222	9.03E-153	postive
TRAJ18	CD44-AS1	0.828572243	2.27E-136	postive
TRAJ21	CD44-AS1	0.874188606	2.38E-169	postive
TRAJ31	CD44-AS1	0.86325358	2.24E-160	postive
TRAJ37	CD44-AS1	0.842102196	4.85E-145	postive
TRAJ38	CD44-AS1	0.870530892	2.95E-166	postive
TRAJ39	CD44-AS1	0.884611666	1.01E-178	postive
RFXAP	ASH1L-AS1	0.502601325	1.37E-35	postive
GNRH1	ASH1L-AS1	0.518023377	4.56E-38	postive
CREB1	AC022306.2	0.558388055	3.48E-45	postive
UBR1	AC022306.2	0.58527593	1.70E-50	postive
IREB2	AC022306.2	0.501178056	2.28E-35	postive
DDX17	AC022306.2	0.623761033	5.10E-59	postive
NFAT5	AC022306.2	0.715721308	3.85E-85	postive
GNRH1	AC022306.2	0.737710416	5.36E-93	postive
IL6ST	AC022306.2	0.502731405	1.30E-35	postive
ACVR2A	AC022306.2	0.602963766	2.86E-54	postive
ANGPTL1	AC022306.2	0.524753267	3.45E-39	postive
BMPR1A	AC022306.2	0.532141141	1.90E-40	postive
BMPR2	AC022306.2	0.548979302	1.94E-43	postive
NR2C1	AC022306.2	0.511965761	4.43E-37	postive
NR2C2	AC022306.2	0.64109168	2.93E-63	postive
RORA	AC022306.2	0.658376253	9.01E-68	postive
SOS1	AC022306.2	0.512294728	3.92E-37	postive
BRAF	AC022306.2	0.611135676	4.29E-56	postive
TRAJ1	AC022306.2	0.600773108	8.65E-54	postive
TRAJ2	AC022306.2	0.602445273	3.72E-54	postive
TRAJ3	AC022306.2	0.62754157	6.38E-60	postive
TRAJ5	AC022306.2	0.586490767	9.52E-51	postive
TRAJ6	AC022306.2	0.650114758	1.41E-65	postive
TRAJ8	AC022306.2	0.630124843	1.52E-60	postive
TRAJ10	AC022306.2	0.616864128	2.10E-57	postive
TRAJ12	AC022306.2	0.61083032	5.03E-56	postive
TRAJ13	AC022306.2	0.620958378	2.34E-58	postive
TRAJ14	AC022306.2	0.613288444	1.39E-56	postive
TRAJ16	AC022306.2	0.622416024	1.06E-58	postive
TRAJ17	AC022306.2	0.585372907	1.62E-50	postive
TRAJ18	AC022306.2	0.575430128	1.71E-48	postive
TRAJ21	AC022306.2	0.631176102	8.43E-61	postive
TRAJ31	AC022306.2	0.663037019	4.86E-69	postive
TRAJ37	AC022306.2	0.67184965	1.68E-71	postive
TRAJ38	AC022306.2	0.672801036	9.01E-72	postive
TRAJ39	AC022306.2	0.645408456	2.33E-64	postive
CREB1	AL157392.3	0.758201076	4.56E-101	postive
RFXAP	AL157392.3	0.581331364	1.10E-49	postive
UBR1	AL157392.3	0.610077505	7.45E-56	postive
ZC3HAV1	AL157392.3	0.598569791	2.61E-53	postive
IL15	AL157392.3	0.530378628	3.81E-40	postive
CYLD	AL157392.3	0.614553285	7.14E-57	postive
EIF2AK2	AL157392.3	0.549900324	1.32E-43	postive
MAPK8	AL157392.3	0.578729127	3.71E-49	postive
NFKBIZ	AL157392.3	0.512381681	3.80E-37	postive
SP1	AL157392.3	0.50123691	2.23E-35	postive
IREB2	AL157392.3	0.582726345	5.69E-50	postive
DDX17	AL157392.3	0.738105174	3.81E-93	postive

JAK2	AL157392.3	0.603155058	2.60E-54	postive
TXK	AL157392.3	0.591070677	1.05E-51	postive
NFAT5	AL157392.3	0.820311494	1.92E-131	postive
MALT1	AL157392.3	0.52788227	1.02E-39	postive
PIK3R1	AL157392.3	0.628621814	3.51E-60	postive
PIK3CA	AL157392.3	0.660571693	2.29E-68	postive
PIK3CB	AL157392.3	0.5170595	6.57E-38	postive
RASGRP3	AL157392.3	0.547234855	4.03E-43	postive
IGHD6-6	AL157392.3	0.51306275	2.95E-37	postive
LTB4R2	AL157392.3	0.575132792	1.96E-48	postive
GNRH1	AL157392.3	0.815557274	1.01E-128	postive
IL6ST	AL157392.3	0.626067708	1.44E-59	postive
RABEP1	AL157392.3	0.574609091	2.49E-48	postive
ACVR2A	AL157392.3	0.671029673	2.87E-71	postive
ANGPTL1	AL157392.3	0.733450661	2.05E-91	postive
BMPR1A	AL157392.3	0.526872528	1.51E-39	postive
BMPR2	AL157392.3	0.704189354	2.60E-81	postive
CRLF3	AL157392.3	0.608192143	1.98E-55	postive
NR1D2	AL157392.3	0.514440373	1.76E-37	postive
NR2C1	AL157392.3	0.565617348	1.45E-46	postive
NR2C2	AL157392.3	0.827087065	1.82E-135	postive
RORA	AL157392.3	0.825057329	3.04E-134	postive
SOS1	AL157392.3	0.71904807	2.79E-86	postive
SOS2	AL157392.3	0.599978526	1.29E-53	postive
BRAF	AL157392.3	0.823326501	3.27E-133	postive
ITK	AL157392.3	0.521725627	1.11E-38	postive
CBL	AL157392.3	0.605649631	7.30E-55	postive
CBLB	AL157392.3	0.714109355	1.36E-84	postive
RASGRP1	AL157392.3	0.513057325	2.95E-37	postive
PDK1	AL157392.3	0.520955185	1.49E-38	postive
TRAJ1	AL157392.3	0.750037958	9.29E-98	postive
TRAJ2	AL157392.3	0.703702429	3.73E-81	postive
TRAJ3	AL157392.3	0.777505517	1.92E-109	postive
TRAJ5	AL157392.3	0.800993358	7.52E-121	postive
TRAJ6	AL157392.3	0.768331051	2.32E-105	postive
TRAJ8	AL157392.3	0.769800561	5.30E-106	postive
TRAJ10	AL157392.3	0.748577197	3.52E-97	postive
TRAJ12	AL157392.3	0.72117839	5.09E-87	postive
TRAJ13	AL157392.3	0.777022817	3.18E-109	postive
TRAJ14	AL157392.3	0.760400697	5.56E-102	postive
TRAJ16	AL157392.3	0.781621405	2.44E-111	postive
TRAJ17	AL157392.3	0.765061607	5.95E-104	postive
TRAJ18	AL157392.3	0.718735905	3.57E-86	postive
TRAJ21	AL157392.3	0.796357813	1.76E-118	postive
TRAJ31	AL157392.3	0.820487795	1.51E-131	postive
TRAJ37	AL157392.3	0.787117096	6.17E-114	postive
TRAJ38	AL157392.3	0.832458537	8.81E-139	postive
TRAJ39	AL157392.3	0.812531555	4.97E-127	postive
CREB1	AC105389.2	0.685307544	1.99E-75	postive
UBR1	AC105389.2	0.641156728	2.82E-63	postive
ZC3HAV1	AC105389.2	0.51277422	3.28E-37	postive
ZC3HAV1L	AC105389.2	0.560831918	1.20E-45	postive
MAPK8	AC105389.2	0.506116431	3.82E-36	postive
LMBR1	AC105389.2	0.522297053	8.91E-39	postive
IREB2	AC105389.2	0.607991447	2.19E-55	postive
DDX17	AC105389.2	0.527504544	1.18E-39	postive
NFAT5	AC105389.2	0.699436326	8.69E-80	postive
PIK3R1	AC105389.2	0.510202816	8.52E-37	postive
PIK3CA	AC105389.2	0.530353244	3.85E-40	postive
AKT3	AC105389.2	0.55789215	4.31E-45	postive
GNRH1	AC105389.2	0.648227505	4.36E-65	postive
RABEP1	AC105389.2	0.530988712	3.00E-40	postive
ACVR2A	AC105389.2	0.602192494	4.23E-54	postive
ANGPTL1	AC105389.2	0.628508732	3.73E-60	postive
BMPR1A	AC105389.2	0.558908992	2.77E-45	postive

BMPR2	AC105389.2	0.59813581	3.24E-53	postive
CRLF3	AC105389.2	0.587664529	5.42E-51	postive
LIFR	AC105389.2	0.500941219	2.48E-35	postive
NR2C2	AC105389.2	0.642593075	1.22E-63	postive
NR3C1	AC105389.2	0.514564281	1.68E-37	postive
RORA	AC105389.2	0.606309193	5.21E-55	postive
SOS1	AC105389.2	0.650139001	1.39E-65	postive
SOS2	AC105389.2	0.538853416	1.28E-41	postive
BRAF	AC105389.2	0.755446193	6.17E-100	postive
CBL	AC105389.2	0.52850255	7.99E-40	postive
CBLB	AC105389.2	0.564408869	2.48E-46	postive
TRAJ1	AC105389.2	0.712465082	4.85E-84	postive
TRAJ2	AC105389.2	0.696659053	6.55E-79	postive
TRAJ3	AC105389.2	0.740617969	4.27E-94	postive
TRAJ5	AC105389.2	0.70748173	2.19E-82	postive
TRAJ6	AC105389.2	0.746472084	2.36E-96	postive
TRAJ8	AC105389.2	0.797603924	4.12E-119	postive
TRAJ10	AC105389.2	0.68692642	6.48E-76	postive
TRAJ12	AC105389.2	0.682449649	1.41E-74	postive
TRAJ13	AC105389.2	0.75791151	6.01E-101	postive
TRAJ14	AC105389.2	0.76440782	1.13E-103	postive
TRAJ16	AC105389.2	0.696034578	1.03E-78	postive
TRAJ17	AC105389.2	0.691168714	3.31E-77	postive
TRAJ18	AC105389.2	0.581272051	1.13E-49	postive
TRAJ21	AC105389.2	0.745005247	8.81E-96	postive
TRAJ31	AC105389.2	0.73439428	9.20E-92	postive
TRAJ37	AC105389.2	0.71593437	3.26E-85	postive
TRAJ38	AC105389.2	0.769124822	1.05E-105	postive
TRAJ39	AC105389.2	0.750110145	8.70E-98	postive
IL11RA	AC110285.2	0.509415047	1.14E-36	postive
CREB1	AC073534.2	0.598045973	3.39E-53	postive
NFAT5	AC073534.2	0.632043728	5.18E-61	postive
IGHD4-4	AC073534.2	0.52295392	6.92E-39	postive
GNRH1	AC073534.2	0.610794613	5.13E-56	postive
ACVR2A	AC073534.2	0.594675647	1.80E-52	postive
ANGPTL1	AC073534.2	0.561306616	9.73E-46	postive
BMPR2	AC073534.2	0.545739136	7.52E-43	postive
NR2C2	AC073534.2	0.608036318	2.14E-55	postive
RORA	AC073534.2	0.601054341	7.51E-54	postive
SOS1	AC073534.2	0.574179083	3.03E-48	postive
SOS2	AC073534.2	0.501707868	1.88E-35	postive
BRAF	AC073534.2	0.667856652	2.25E-70	postive
TRAJ1	AC073534.2	0.710232657	2.70E-83	postive
TRAJ2	AC073534.2	0.582338328	6.83E-50	postive
TRAJ3	AC073534.2	0.704500519	2.06E-81	postive
TRAJ5	AC073534.2	0.756055325	3.48E-100	postive
TRAJ6	AC073534.2	0.714740136	8.30E-85	postive
TRAJ8	AC073534.2	0.793054439	7.89E-117	postive
TRAJ10	AC073534.2	0.706926919	3.33E-82	postive
TRAJ12	AC073534.2	0.702225953	1.12E-80	postive
TRAJ13	AC073534.2	0.748411787	4.09E-97	postive
TRAJ14	AC073534.2	0.745205448	7.37E-96	postive
TRAJ16	AC073534.2	0.723746623	6.41E-88	postive
TRAJ17	AC073534.2	0.771927545	6.13E-107	postive
TRAJ18	AC073534.2	0.612128918	2.56E-56	postive
TRAJ21	AC073534.2	0.744882415	9.83E-96	postive
TRAJ31	AC073534.2	0.781087866	4.31E-111	postive
TRAJ37	AC073534.2	0.731925126	7.44E-91	postive
TRAJ38	AC073534.2	0.737707557	5.37E-93	postive
TRAJ39	AC073534.2	0.778226472	8.98E-110	postive
LMBR1L	AC027796.4	0.507805824	2.06E-36	postive
ZC3HAV1L	AC131009.3	0.52462936	3.62E-39	postive
GNRH1	AC022784.5	0.517553481	5.45E-38	postive
CREB1	AC010245.2	0.568351899	4.26E-47	postive
CIITA	AC010245.2	0.514016979	2.06E-37	postive

CYLD	AC010245.2	0.596336451	7.91E-53	postive
DDX17	AC010245.2	0.658221919	9.92E-68	postive
JAK2	AC010245.2	0.503335237	1.05E-35	postive
NFAT5	AC010245.2	0.731595284	9.82E-91	postive
NFATC3	AC010245.2	0.500835496	2.58E-35	postive
PIK3R1	AC010245.2	0.56038204	1.46E-45	postive
LTB4R2	AC010245.2	0.525805066	2.29E-39	postive
GNRH1	AC010245.2	0.753921864	2.57E-99	postive
IL6ST	AC010245.2	0.585425605	1.58E-50	postive
ACVR2A	AC010245.2	0.515386181	1.23E-37	postive
ANGPTL1	AC010245.2	0.50955107	1.08E-36	postive
BMPR2	AC010245.2	0.523873526	4.85E-39	postive
NR2C2	AC010245.2	0.646207283	1.45E-64	postive
RORA	AC010245.2	0.720480827	8.90E-87	postive
BRAF	AC010245.2	0.600978335	7.81E-54	postive
CBLB	AC010245.2	0.514480818	1.73E-37	postive
TRAJ1	AC010245.2	0.607085626	3.50E-55	postive
TRAJ2	AC010245.2	0.613690334	1.13E-56	postive
TRAJ3	AC010245.2	0.645391496	2.35E-64	postive
TRAJ5	AC010245.2	0.61342899	1.29E-56	postive
TRAJ6	AC010245.2	0.626513974	1.13E-59	postive
TRAJ8	AC010245.2	0.569569934	2.46E-47	postive
TRAJ10	AC010245.2	0.632231012	4.66E-61	postive
TRAJ12	AC010245.2	0.575758883	1.47E-48	postive
TRAJ13	AC010245.2	0.592017616	6.60E-52	postive
TRAJ14	AC010245.2	0.576670851	9.63E-49	postive
TRAJ16	AC010245.2	0.605667249	7.23E-55	postive
TRAJ17	AC010245.2	0.570557773	1.58E-47	postive
TRAJ18	AC010245.2	0.605438257	8.13E-55	postive
TRAJ21	AC010245.2	0.613060252	1.57E-56	postive
TRAJ31	AC010245.2	0.652009354	4.48E-66	postive
TRAJ37	AC010245.2	0.68023517	6.36E-74	postive
TRAJ38	AC010245.2	0.658174892	1.02E-67	postive
TRAJ39	AC010245.2	0.641673571	2.09E-63	postive
CREB1	AC005261.1	0.61425334	8.37E-57	postive
UBR1	AC005261.1	0.506649168	3.14E-36	postive
ZC3HAV1	AC005261.1	0.545462916	8.43E-43	postive
IREB2	AC005261.1	0.514918717	1.47E-37	postive
DDX17	AC005261.1	0.597608789	4.21E-53	postive
TXK	AC005261.1	0.518282694	4.13E-38	postive
NFAT5	AC005261.1	0.641087893	2.93E-63	postive
PIK3R1	AC005261.1	0.544712905	1.15E-42	postive
PIK3CA	AC005261.1	0.512766569	3.29E-37	postive
GNRH1	AC005261.1	0.723962291	5.38E-88	postive
IL6ST	AC005261.1	0.535388842	5.18E-41	postive
ACVR2A	AC005261.1	0.593845189	2.70E-52	postive
ANGPTL1	AC005261.1	0.57156681	9.98E-48	postive
BMPR1A	AC005261.1	0.500951931	2.47E-35	postive
BMPR2	AC005261.1	0.62742176	6.82E-60	postive
NR2C1	AC005261.1	0.513685026	2.34E-37	postive
NR2C2	AC005261.1	0.672837602	8.80E-72	postive
RORA	AC005261.1	0.698450309	1.78E-79	postive
SOS1	AC005261.1	0.598031468	3.41E-53	postive
BRAF	AC005261.1	0.696160752	9.38E-79	postive
CBLB	AC005261.1	0.601831276	5.08E-54	postive
TRAJ1	AC005261.1	0.527766961	1.07E-39	postive
TRAJ2	AC005261.1	0.554588086	1.79E-44	postive
TRAJ3	AC005261.1	0.577314369	7.15E-49	postive
TRAJ5	AC005261.1	0.586526004	9.36E-51	postive
TRAJ6	AC005261.1	0.54735759	3.83E-43	postive
TRAJ8	AC005261.1	0.554069082	2.24E-44	postive
TRAJ10	AC005261.1	0.524732796	3.48E-39	postive
TRAJ12	AC005261.1	0.502661164	1.34E-35	postive
TRAJ13	AC005261.1	0.555735391	1.09E-44	postive
TRAJ14	AC005261.1	0.535743684	4.49E-41	postive

TRAJ16	AC005261.1	0.555857551	1.04E-44	postive
TRAJ17	AC005261.1	0.531519658	2.43E-40	postive
TRAJ21	AC005261.1	0.57320989	4.72E-48	postive
TRAJ31	AC005261.1	0.622967677	7.85E-59	postive
TRAJ37	AC005261.1	0.582269472	7.06E-50	postive
TRAJ38	AC005261.1	0.625841617	1.63E-59	postive
TRAJ39	AC005261.1	0.60434017	1.42E-54	postive
CRIM1	CRIM1-DT	0.635865845	5.94E-62	postive
TGFBR3	CRIM1-DT	0.506150788	3.77E-36	postive
HLA-DQA1	HLA-DQB1-AS1	0.518730915	3.49E-38	postive
HLA-DQB1	HLA-DQB1-AS1	0.666228688	6.39E-70	postive
HLA-DRB1	HLA-DQB1-AS1	0.505827736	4.24E-36	postive
HLA-DRB5	HLA-DQB1-AS1	0.613674547	1.14E-56	postive
CIITA	HLA-DQB1-AS1	0.658961889	6.26E-68	postive
CYLD	HLA-DQB1-AS1	0.514241556	1.90E-37	postive
TRIM22	HLA-DQB1-AS1	0.503492463	9.90E-36	postive
NFAT5	AC024560.3	0.511630134	5.02E-37	postive
LTB4R2	AC024560.3	0.509633338	1.05E-36	postive
GNRH1	AC024560.3	0.578727487	3.71E-49	postive
NR2C2	AC024560.3	0.61476246	6.40E-57	postive
BRAF	AC024560.3	0.508964363	1.35E-36	postive
TRAJ1	AC024560.3	0.506080044	3.87E-36	postive
TRAJ5	AC024560.3	0.520157008	2.02E-38	postive
TRAJ31	AC024560.3	0.503989886	8.27E-36	postive
TRAJ39	AC024560.3	0.513197557	2.80E-37	postive
CREB1	AC010168.2	0.669457778	7.98E-71	postive
UBR1	AC010168.2	0.606349179	5.10E-55	postive
CYLD	AC010168.2	0.51780451	4.95E-38	postive
TCF7L2	AC010168.2	0.502840325	1.25E-35	postive
SP1	AC010168.2	0.525835083	2.27E-39	postive
IREB2	AC010168.2	0.564792214	2.09E-46	postive
DDX17	AC010168.2	0.617124695	1.83E-57	postive
JAK2	AC010168.2	0.506791391	2.99E-36	postive
TXK	AC010168.2	0.570987624	1.30E-47	postive
NFAT5	AC010168.2	0.716433564	2.20E-85	postive
PIK3CA	AC010168.2	0.570638205	1.52E-47	postive
ROBO2	AC010168.2	0.645957679	1.68E-64	postive
GNRH1	AC010168.2	0.779981148	1.40E-110	postive
ACVR2A	AC010168.2	0.624432984	3.53E-59	postive
ANGPTL1	AC010168.2	0.573552401	4.04E-48	postive
BMPR1A	AC010168.2	0.529605838	5.17E-40	postive
BMPR2	AC010168.2	0.663044328	4.84E-69	postive
NR1D2	AC010168.2	0.511515249	5.24E-37	postive
NR2C1	AC010168.2	0.530317309	3.91E-40	postive
NR2C2	AC010168.2	0.645863977	1.78E-64	postive
RORA	AC010168.2	0.676730857	6.69E-73	postive
SOS1	AC010168.2	0.610723769	5.32E-56	postive
BRAF	AC010168.2	0.634516515	1.28E-61	postive
CBLB	AC010168.2	0.62229779	1.13E-58	postive
TRAJ1	AC010168.2	0.569493261	2.55E-47	postive
TRAJ2	AC010168.2	0.541201705	4.90E-42	postive
TRAJ3	AC010168.2	0.583274657	4.39E-50	postive
TRAJ5	AC010168.2	0.565496859	1.53E-46	postive
TRAJ6	AC010168.2	0.604779327	1.14E-54	postive
TRAJ8	AC010168.2	0.564242391	2.67E-46	postive
TRAJ10	AC010168.2	0.558950788	2.72E-45	postive
TRAJ12	AC010168.2	0.575806641	1.43E-48	postive
TRAJ13	AC010168.2	0.580624531	1.53E-49	postive
TRAJ14	AC010168.2	0.542636656	2.72E-42	postive
TRAJ16	AC010168.2	0.589550513	2.19E-51	postive
TRAJ17	AC010168.2	0.537029634	2.67E-41	postive
TRAJ18	AC010168.2	0.534914387	6.27E-41	postive
TRAJ21	AC010168.2	0.586257473	1.06E-50	postive
TRAJ31	AC010168.2	0.632064281	5.12E-61	postive
TRAJ37	AC010168.2	0.598821366	2.30E-53	postive

TRAJ38	AC010168.2	0.650126621	1.40E-65	postive
TRAJ39	AC010168.2	0.578751864	3.67E-49	postive
IL3RA	AC147067.2	0.515174096	1.34E-37	postive
TIE1	AC147067.2	0.503075567	1.15E-35	postive
CREB1	ATP1B3-AS1	0.651696715	5.41E-66	postive
IL15	ATP1B3-AS1	0.628243341	4.33E-60	postive
NFKBIZ	ATP1B3-AS1	0.545391982	8.69E-43	postive
LMBR1	ATP1B3-AS1	0.52212034	9.54E-39	postive
IREB2	ATP1B3-AS1	0.511714296	4.87E-37	postive
JAK2	ATP1B3-AS1	0.561772325	7.93E-46	postive
TXK	ATP1B3-AS1	0.509100548	1.28E-36	postive
NFAT5	ATP1B3-AS1	0.610694482	5.40E-56	postive
MALT1	ATP1B3-AS1	0.515734221	1.08E-37	postive
PIK3R1	ATP1B3-AS1	0.55208295	5.22E-44	postive
PIK3CA	ATP1B3-AS1	0.655816346	4.38E-67	postive
PIK3CB	ATP1B3-AS1	0.521460111	1.23E-38	postive
GNRH1	ATP1B3-AS1	0.581535254	9.97E-50	postive
IL6ST	ATP1B3-AS1	0.536902042	2.82E-41	postive
ANGPTL1	ATP1B3-AS1	0.742793484	6.30E-95	postive
BMPR2	ATP1B3-AS1	0.626695601	1.02E-59	postive
CRLF3	ATP1B3-AS1	0.517340879	5.91E-38	postive
IL18R1	ATP1B3-AS1	0.567368369	6.63E-47	postive
NR2C2	ATP1B3-AS1	0.555894843	1.02E-44	postive
RORA	ATP1B3-AS1	0.693387171	6.86E-78	postive
SOS1	ATP1B3-AS1	0.562783302	5.08E-46	postive
BRAF	ATP1B3-AS1	0.638484299	1.32E-62	postive
ITK	ATP1B3-AS1	0.514275407	1.87E-37	postive
CBL	ATP1B3-AS1	0.566586819	9.40E-47	postive
CBLB	ATP1B3-AS1	0.647853152	5.45E-65	postive
PDK1	ATP1B3-AS1	0.608404446	1.77E-55	postive
TRAJ3	ATP1B3-AS1	0.584865471	2.07E-50	postive
TRAJ5	ATP1B3-AS1	0.58112266	1.21E-49	postive
TRAJ6	ATP1B3-AS1	0.543777024	1.70E-42	postive
TRAJ10	ATP1B3-AS1	0.587512521	5.83E-51	postive
TRAJ13	ATP1B3-AS1	0.525116079	3.00E-39	postive
TRAJ14	ATP1B3-AS1	0.510765747	6.92E-37	postive
TRAJ16	ATP1B3-AS1	0.562625846	5.45E-46	postive
TRAJ17	ATP1B3-AS1	0.570407695	1.69E-47	postive
TRAJ18	ATP1B3-AS1	0.527571537	1.15E-39	postive
TRAJ21	ATP1B3-AS1	0.578464784	4.19E-49	postive
TRAJ31	ATP1B3-AS1	0.601366473	6.42E-54	postive
TRAJ37	ATP1B3-AS1	0.549416384	1.61E-43	postive
TRAJ38	ATP1B3-AS1	0.634691524	1.16E-61	postive
TRAJ39	ATP1B3-AS1	0.596634129	6.83E-53	postive
DDX17	AL590652.1	0.503491098	9.91E-36	postive
GNRH1	AL590652.1	0.545007581	1.02E-42	postive
NR2C2	AL590652.1	0.568139854	4.69E-47	postive
DDX17	SH3BP5-AS1	0.716457202	2.16E-85	postive
NFAT5	SH3BP5-AS1	0.583088618	4.80E-50	postive
LTB4R2	SH3BP5-AS1	0.600214784	1.15E-53	postive
GNRH1	SH3BP5-AS1	0.753489562	3.85E-99	postive
NR2C1	SH3BP5-AS1	0.575564645	1.60E-48	postive
NR2C2	SH3BP5-AS1	0.773234814	1.61E-107	postive
RORA	SH3BP5-AS1	0.577728234	5.90E-49	postive
BRAF	SH3BP5-AS1	0.553074622	3.42E-44	postive
TRAJ2	SH3BP5-AS1	0.513453012	2.55E-37	postive
TRAJ3	SH3BP5-AS1	0.547328891	3.87E-43	postive
TRAJ38	SH3BP5-AS1	0.509568595	1.08E-36	postive
CBLB	SRD5A3-AS1	0.5087567	1.45E-36	postive
CREB1	AC048341.1	0.538908704	1.25E-41	postive
UBR1	AC048341.1	0.558673859	3.07E-45	postive
DDX17	AC048341.1	0.620810396	2.53E-58	postive
TXK	AC048341.1	0.582333983	6.85E-50	postive
NFAT5	AC048341.1	0.637948217	1.80E-62	postive
IGHD4-4	AC048341.1	0.500511791	2.89E-35	postive

IGHD6-6	AC048341.1	0.537785862	1.97E-41	postive
GNRH1	AC048341.1	0.772871149	2.34E-107	postive
BMPR2	AC048341.1	0.524033762	4.56E-39	postive
NR2C2	AC048341.1	0.685661821	1.56E-75	postive
RORA	AC048341.1	0.633826644	1.89E-61	postive
BRAF	AC048341.1	0.602062213	4.52E-54	postive
TEC	AC048341.1	0.631880057	5.68E-61	postive
CBLB	AC048341.1	0.524663037	3.57E-39	postive
TRAJ1	AC048341.1	0.540422246	6.74E-42	postive
TRAJ3	AC048341.1	0.560594753	1.33E-45	postive
TRAJ5	AC048341.1	0.541123292	5.06E-42	postive
TRAJ6	AC048341.1	0.528768243	7.20E-40	postive
TRAJ12	AC048341.1	0.501196369	2.26E-35	postive
TRAJ21	AC048341.1	0.520613464	1.70E-38	postive
TRAJ31	AC048341.1	0.522597412	7.94E-39	postive
TRAJ37	AC048341.1	0.542272339	3.15E-42	postive
TRAJ38	AC048341.1	0.54767805	3.35E-43	postive
TRAJ39	AC048341.1	0.588610415	3.44E-51	postive
CREB1	AL157932.1	0.600660842	9.16E-54	postive
RFXAP	AL157932.1	0.500390171	3.02E-35	postive
UBR1	AL157932.1	0.504834967	6.09E-36	postive
IL15	AL157932.1	0.501346847	2.14E-35	postive
TXK	AL157932.1	0.552192696	4.98E-44	postive
NFAT5	AL157932.1	0.587440253	6.04E-51	postive
PIK3R1	AL157932.1	0.519297736	2.81E-38	postive
PIK3CA	AL157932.1	0.631609673	6.61E-61	postive
IGHD6-6	AL157932.1	0.521590838	1.17E-38	postive
LTB4R2	AL157932.1	0.523892414	4.82E-39	postive
GNRH1	AL157932.1	0.609194136	1.18E-55	postive
ANGPTL1	AL157932.1	0.599163853	1.94E-53	postive
BMPR2	AL157932.1	0.591070595	1.05E-51	postive
NR2C2	AL157932.1	0.621884423	1.41E-58	postive
RORA	AL157932.1	0.587472406	5.95E-51	postive
SOS1	AL157932.1	0.542526889	2.84E-42	postive
SOS2	AL157932.1	0.505239349	5.26E-36	postive
BRAF	AL157932.1	0.624377365	3.64E-59	postive
CBL	AL157932.1	0.557252281	5.69E-45	postive
CBLB	AL157932.1	0.569102777	3.04E-47	postive
TRAJ1	AL157932.1	0.514955562	1.45E-37	postive
TRAJ3	AL157932.1	0.602176684	4.27E-54	postive
TRAJ5	AL157932.1	0.548359765	2.51E-43	postive
TRAJ6	AL157932.1	0.540968321	5.39E-42	postive
TRAJ8	AL157932.1	0.537955291	1.84E-41	postive
TRAJ10	AL157932.1	0.535099639	5.82E-41	postive
TRAJ13	AL157932.1	0.502145125	1.61E-35	postive
TRAJ14	AL157932.1	0.517003539	6.71E-38	postive
TRAJ16	AL157932.1	0.559215372	2.43E-45	postive
TRAJ17	AL157932.1	0.555576472	1.17E-44	postive
TRAJ18	AL157932.1	0.507404264	2.39E-36	postive
TRAJ21	AL157932.1	0.563430136	3.82E-46	postive
TRAJ31	AL157932.1	0.546354273	5.82E-43	postive
TRAJ37	AL157932.1	0.525456425	2.63E-39	postive
TRAJ38	AL157932.1	0.583140821	4.68E-50	postive
TRAJ39	AL157932.1	0.614399273	7.75E-57	postive
CREB1	AC242426.2	0.708409777	1.08E-82	postive
RFXAP	AC242426.2	0.500588249	2.81E-35	postive
UBR1	AC242426.2	0.59741168	4.64E-53	postive
ZC3HAV1	AC242426.2	0.504936288	5.87E-36	postive
CYLD	AC242426.2	0.551530704	6.60E-44	postive
EIF2AK2	AC242426.2	0.562369273	6.10E-46	postive
IREB2	AC242426.2	0.527453689	1.21E-39	postive
DDX17	AC242426.2	0.594292272	2.17E-52	postive
JAK2	AC242426.2	0.551024377	8.18E-44	postive
NFAT5	AC242426.2	0.731055195	1.54E-90	postive
PIK3R1	AC242426.2	0.557364012	5.42E-45	postive

PIK3CA	AC242426.2	0.615488824	4.36E-57	postive
PIK3CB	AC242426.2	0.503924528	8.47E-36	postive
GNRH1	AC242426.2	0.63655744	4.00E-62	postive
IL6ST	AC242426.2	0.536346876	3.52E-41	postive
ACVR2A	AC242426.2	0.622869621	8.28E-59	postive
ANGPTL1	AC242426.2	0.599483778	1.65E-53	postive
BMPR2	AC242426.2	0.624446614	3.50E-59	postive
CRLF3	AC242426.2	0.555946681	9.99E-45	postive
NR2C2	AC242426.2	0.717979072	6.51E-86	postive
RORA	AC242426.2	0.682360443	1.50E-74	postive
SOS1	AC242426.2	0.678410767	2.17E-73	postive
SOS2	AC242426.2	0.577442327	6.74E-49	postive
BRAF	AC242426.2	0.734741252	6.85E-92	postive
CBLB	AC242426.2	0.581349748	1.09E-49	postive
RASGRP1	AC242426.2	0.512096871	4.22E-37	postive
TRAJ1	AC242426.2	0.767196292	7.20E-105	postive
TRAJ2	AC242426.2	0.642223542	1.51E-63	postive
TRAJ3	AC242426.2	0.733002535	3.00E-91	postive
TRAJ5	AC242426.2	0.796647823	1.26E-118	postive
TRAJ6	AC242426.2	0.769552146	6.80E-106	postive
TRAJ8	AC242426.2	0.780724793	6.35E-111	postive
TRAJ10	AC242426.2	0.742121784	1.14E-94	postive
TRAJ12	AC242426.2	0.797303341	5.85E-119	postive
TRAJ13	AC242426.2	0.774009213	7.26E-108	postive
TRAJ14	AC242426.2	0.792591159	1.34E-116	postive
TRAJ16	AC242426.2	0.780462359	8.41E-111	postive
TRAJ17	AC242426.2	0.776515724	5.40E-109	postive
TRAJ18	AC242426.2	0.730277263	2.96E-90	postive
TRAJ21	AC242426.2	0.79217494	2.15E-116	postive
TRAJ31	AC242426.2	0.80026492	1.79E-120	postive
TRAJ37	AC242426.2	0.756428238	2.45E-100	postive
TRAJ38	AC242426.2	0.809334201	2.83E-125	postive
TRAJ39	AC242426.2	0.796289258	1.91E-118	postive
DDX17	THUMPD3-AS1	0.621212055	2.04E-58	postive
NFAT5	THUMPD3-AS1	0.519345791	2.76E-38	postive
GNRH1	THUMPD3-AS1	0.677492361	4.02E-73	postive
NR2C1	THUMPD3-AS1	0.544443556	1.29E-42	postive
NR2C2	THUMPD3-AS1	0.730307571	2.89E-90	postive
BRAF	THUMPD3-AS1	0.519965428	2.18E-38	postive
TRAJ3	THUMPD3-AS1	0.507561873	2.25E-36	postive
TRAJ38	THUMPD3-AS1	0.519247899	2.86E-38	postive
CREB1	AL078581.2	0.616720927	2.26E-57	postive
UBR1	AL078581.2	0.515238463	1.30E-37	postive
CYLD	AL078581.2	0.51708194	6.51E-38	postive
LIMS1	AL078581.2	0.512730657	3.34E-37	postive
IREB2	AL078581.2	0.512173316	4.10E-37	postive
PIK3CG	AL078581.2	0.50306252	1.16E-35	postive
JAK2	AL078581.2	0.534406747	7.68E-41	postive
NFAT5	AL078581.2	0.575832736	1.42E-48	postive
PIK3R1	AL078581.2	0.528241696	8.85E-40	postive
PIK3CA	AL078581.2	0.599521969	1.62E-53	postive
IL6ST	AL078581.2	0.570307034	1.77E-47	postive
ACVR2A	AL078581.2	0.518365171	4.01E-38	postive
ANGPTL1	AL078581.2	0.500039505	3.43E-35	postive
BMPR1A	AL078581.2	0.521027589	1.45E-38	postive
BMPR2	AL078581.2	0.635070953	9.34E-62	postive
CRLF3	AL078581.2	0.515480663	1.19E-37	postive
NR1D2	AL078581.2	0.547432012	3.71E-43	postive
RORA	AL078581.2	0.622914604	8.08E-59	postive
BRAF	AL078581.2	0.506007515	3.97E-36	postive
TRAJ5	AL078581.2	0.507330041	2.45E-36	postive
TRAJ31	AL078581.2	0.53508908	5.84E-41	postive
TRAJ37	AL078581.2	0.501165264	2.29E-35	postive
TRAJ38	AL078581.2	0.536328811	3.55E-41	postive
LTB4R2	AC104463.2	0.50743687	2.36E-36	postive

GNRH1	AC104463.2	0.603264969	2.46E-54	postive
CREB1	NARF-IT1	0.632309665	4.46E-61	postive
ZC3HAV1L	NARF-IT1	0.520231739	1.97E-38	postive
IL15	NARF-IT1	0.506161965	3.76E-36	postive
MAPK8	NARF-IT1	0.539336141	1.05E-41	postive
NFKBIZ	NARF-IT1	0.629669025	1.96E-60	postive
DDX17	NARF-IT1	0.645557517	2.13E-64	postive
JAK2	NARF-IT1	0.522971361	6.87E-39	postive
NFAT5	NARF-IT1	0.653532596	1.77E-66	postive
PIK3CA	NARF-IT1	0.537418072	2.29E-41	postive
LTB4R2	NARF-IT1	0.569112919	3.03E-47	postive
GNRH1	NARF-IT1	0.746217172	2.97E-96	postive
ACVR2A	NARF-IT1	0.513566014	2.44E-37	postive
ANGPTL1	NARF-IT1	0.654006477	1.33E-66	postive
BMPR2	NARF-IT1	0.531095557	2.87E-40	postive
CRLF3	NARF-IT1	0.542351675	3.05E-42	postive
NR2C1	NARF-IT1	0.573714396	3.75E-48	postive
NR2C2	NARF-IT1	0.731385635	1.17E-90	postive
RORA	NARF-IT1	0.646376692	1.31E-64	postive
SOS1	NARF-IT1	0.610479597	6.04E-56	postive
SOS2	NARF-IT1	0.520978947	1.48E-38	postive
BRAF	NARF-IT1	0.676137582	9.93E-73	postive
CBL	NARF-IT1	0.570655513	1.51E-47	postive
CBLB	NARF-IT1	0.668325914	1.66E-70	postive
PDK1	NARF-IT1	0.552636386	4.12E-44	postive
TRAJ1	NARF-IT1	0.616241544	2.92E-57	postive
TRAJ2	NARF-IT1	0.543212654	2.14E-42	postive
TRAJ3	NARF-IT1	0.630604307	1.16E-60	postive
TRAJ5	NARF-IT1	0.647277098	7.68E-65	postive
TRAJ6	NARF-IT1	0.625576429	1.89E-59	postive
TRAJ8	NARF-IT1	0.574523696	2.59E-48	postive
TRAJ10	NARF-IT1	0.62094993	2.35E-58	postive
TRAJ12	NARF-IT1	0.543553008	1.86E-42	postive
TRAJ13	NARF-IT1	0.601508817	5.98E-54	postive
TRAJ14	NARF-IT1	0.601126947	7.24E-54	postive
TRAJ16	NARF-IT1	0.630482234	1.24E-60	postive
TRAJ17	NARF-IT1	0.604989	1.02E-54	postive
TRAJ18	NARF-IT1	0.562109142	6.84E-46	postive
TRAJ21	NARF-IT1	0.631330534	7.73E-61	postive
TRAJ31	NARF-IT1	0.636355878	4.49E-62	postive
TRAJ37	NARF-IT1	0.599373114	1.75E-53	postive
TRAJ38	NARF-IT1	0.669659058	7.00E-71	postive
TRAJ39	NARF-IT1	0.637282881	2.64E-62	postive
CREB1	AC025917.1	0.804579707	1.00E-122	postive
RFXAP	AC025917.1	0.545312009	8.98E-43	postive
UBR1	AC025917.1	0.687006009	6.13E-76	postive
ZC3HAV1	AC025917.1	0.583802046	3.42E-50	postive
ZC3HAV1L	AC025917.1	0.512580851	3.53E-37	postive
IL15	AC025917.1	0.576909345	8.62E-49	postive
CYLD	AC025917.1	0.65406855	1.28E-66	postive
EIF2AK2	AC025917.1	0.57283342	5.61E-48	postive
MAPK8	AC025917.1	0.568159388	4.65E-47	postive
LMBR1	AC025917.1	0.586216222	1.09E-50	postive
LIMS1	AC025917.1	0.520465538	1.80E-38	postive
IREB2	AC025917.1	0.636745548	3.59E-62	postive
DDX17	AC025917.1	0.672396859	1.17E-71	postive
PIK3CG	AC025917.1	0.572619199	6.18E-48	postive
JAK2	AC025917.1	0.669188671	9.50E-71	postive
TXK	AC025917.1	0.637770954	2.00E-62	postive
NFAT5	AC025917.1	0.871103931	9.80E-167	postive
MALT1	AC025917.1	0.560293037	1.52E-45	postive
PIK3R1	AC025917.1	0.700533709	3.89E-80	postive
PIK3CA	AC025917.1	0.69618973	9.19E-79	postive
PIK3CB	AC025917.1	0.514302324	1.85E-37	postive
AKT3	AC025917.1	0.533163057	1.26E-40	postive

RASGRP3	AC025917.1	0.564451459	2.43E-46	postive
IGHD4-4	AC025917.1	0.505814436	4.26E-36	postive
IGHD6-6	AC025917.1	0.53610008	3.89E-41	postive
PLXNC1	AC025917.1	0.520731793	1.62E-38	postive
GNRH1	AC025917.1	0.76320177	3.69E-103	postive
IL6ST	AC025917.1	0.688811178	1.74E-76	postive
RABEP1	AC025917.1	0.58710016	7.11E-51	postive
ACVR2A	AC025917.1	0.649962842	1.54E-65	postive
ANGPTL1	AC025917.1	0.778468006	6.96E-110	postive
BMPR1A	AC025917.1	0.552510238	4.35E-44	postive
BMPR2	AC025917.1	0.758015421	5.45E-101	postive
CRLF3	AC025917.1	0.661463366	1.31E-68	postive
LIFR	AC025917.1	0.535778402	4.43E-41	postive
NR1D2	AC025917.1	0.551359612	7.09E-44	postive
NR2C1	AC025917.1	0.500826983	2.58E-35	postive
NR2C2	AC025917.1	0.745969283	3.71E-96	postive
RORA	AC025917.1	0.890709032	1.23E-184	postive
SOS1	AC025917.1	0.712664482	4.16E-84	postive
SOS2	AC025917.1	0.605340127	8.55E-55	postive
BRAF	AC025917.1	0.850268422	1.12E-150	postive
ITK	AC025917.1	0.557217693	5.77E-45	postive
TEC	AC025917.1	0.518392849	3.96E-38	postive
CD28	AC025917.1	0.528264117	8.77E-40	postive
CBL	AC025917.1	0.620337979	3.26E-58	postive
CBLB	AC025917.1	0.797546788	4.41E-119	postive
RASGRP1	AC025917.1	0.540521053	6.47E-42	postive
PDK1	AC025917.1	0.599359309	1.76E-53	postive
TRAJ1	AC025917.1	0.731324456	1.23E-90	postive
TRAJ2	AC025917.1	0.787233069	5.43E-114	postive
TRAJ3	AC025917.1	0.799759648	3.26E-120	postive
TRAJ5	AC025917.1	0.792053598	2.46E-116	postive
TRAJ6	AC025917.1	0.788097016	2.08E-114	postive
TRAJ8	AC025917.1	0.755317834	6.97E-100	postive
TRAJ10	AC025917.1	0.75449255	1.51E-99	postive
TRAJ12	AC025917.1	0.720981652	5.96E-87	postive
TRAJ13	AC025917.1	0.776236882	7.22E-109	postive
TRAJ14	AC025917.1	0.771525794	9.23E-107	postive
TRAJ16	AC025917.1	0.773439711	1.30E-107	postive
TRAJ17	AC025917.1	0.716394525	2.27E-85	postive
TRAJ18	AC025917.1	0.740855186	3.47E-94	postive
TRAJ21	AC025917.1	0.807478048	2.85E-124	postive
TRAJ31	AC025917.1	0.826962686	2.17E-135	postive
TRAJ37	AC025917.1	0.819829653	3.65E-131	postive
TRAJ38	AC025917.1	0.875742303	1.08E-170	postive
TRAJ39	AC025917.1	0.82946174	6.44E-137	postive
NPY	LINC02315	0.675641981	1.38E-72	postive
CD79A	FAM30A	0.6742258	3.53E-72	postive
CD79B	FAM30A	0.560426887	1.43E-45	postive
CD19	FAM30A	0.669555823	7.49E-71	postive
PLCG2	FAM30A	0.650680932	1.00E-65	postive
IGHA1	FAM30A	0.561891906	7.52E-46	postive
IGHD1-1	FAM30A	0.529240172	5.98E-40	postive
IGHD2-2	FAM30A	0.615707811	3.88E-57	postive
IGHD6-25	FAM30A	0.61548123	4.37E-57	postive
IGHD6-6	FAM30A	0.5302708	3.98E-40	postive
IGHG1	FAM30A	0.654751082	8.43E-67	postive
IGHG3	FAM30A	0.508609215	1.53E-36	postive
IGHJ2	FAM30A	0.553233666	3.20E-44	postive
IGHJ3	FAM30A	0.588206193	4.18E-51	postive
IGHV3-11	FAM30A	0.563204934	4.22E-46	postive
IGHV3-15	FAM30A	0.544248132	1.40E-42	postive
IGHV3-21	FAM30A	0.506513692	3.30E-36	postive
IGHV3-30	FAM30A	0.564648719	2.23E-46	postive
IGHV3-33	FAM30A	0.530722914	3.33E-40	postive
IGHV3-38	FAM30A	0.531988708	2.02E-40	postive

IGHV3-48	FAM30A	0.521246607	1.33E-38	postive
IGHV3-74	FAM30A	0.553595204	2.74E-44	postive
IGHV4-28	FAM30A	0.640013273	5.47E-63	postive
IGHV4-39	FAM30A	0.540901778	5.54E-42	postive
IGHV4-59	FAM30A	0.53127884	2.67E-40	postive
IGHV4-61	FAM30A	0.528297186	8.66E-40	postive
IGHV5-51	FAM30A	0.592743397	4.63E-52	postive
IGKC	FAM30A	0.673420782	6.00E-72	postive
IGKJ5	FAM30A	0.80902559	4.16E-125	postive
IGKV1-39	FAM30A	0.522276943	8.98E-39	postive
IGKV1-5	FAM30A	0.561900082	7.50E-46	postive
IGKV1-6	FAM30A	0.52861166	7.65E-40	postive
IGKV3-11	FAM30A	0.601204518	6.97E-54	postive
IGKV3-15	FAM30A	0.577325764	7.11E-49	postive
IGKV3-20	FAM30A	0.671375525	2.29E-71	postive
IGKV3-7	FAM30A	0.537371253	2.33E-41	postive
IGKV3D-11	FAM30A	0.555815448	1.06E-44	postive
IGKV4-1	FAM30A	0.528736098	7.29E-40	postive
IGLC2	FAM30A	0.638021382	1.73E-62	postive
IGLC3	FAM30A	0.573810869	3.59E-48	postive
IGLJ1	FAM30A	0.769121551	1.05E-105	postive
IGLJ3	FAM30A	0.528653812	7.53E-40	postive
IGLV1-40	FAM30A	0.506350081	3.51E-36	postive
IGLV1-44	FAM30A	0.505956661	4.05E-36	postive
IGLV1-47	FAM30A	0.550748859	9.19E-44	postive
IGLV2-11	FAM30A	0.539986873	8.05E-42	postive
IGLV2-14	FAM30A	0.551015628	8.21E-44	postive
IGLV2-8	FAM30A	0.531962972	2.04E-40	postive
IGLV3-1	FAM30A	0.549866246	1.33E-43	postive
IGLV3-21	FAM30A	0.533478181	1.11E-40	postive
IGLV3-25	FAM30A	0.528291204	8.68E-40	postive
IGLV6-57	FAM30A	0.51074353	6.98E-37	postive
SEMA4D	FAM30A	0.544471776	1.27E-42	postive
IL16	FAM30A	0.62674427	9.92E-60	postive
PNOC	FAM30A	0.677006311	5.57E-73	postive
IL10RA	FAM30A	0.542235317	3.20E-42	postive
TNFRSF13C	FAM30A	0.606669305	4.33E-55	postive
TNFRSF17	FAM30A	0.66018499	2.92E-68	postive
ITGAL	FAM30A	0.523681024	5.23E-39	postive
CD247	FAM30A	0.513482796	2.52E-37	postive
ZAP70	FAM30A	0.569628647	2.40E-47	postive
SH2D1A	FAM30A	0.519042898	3.10E-38	postive
ITK	FAM30A	0.528766298	7.20E-40	postive
PDK1	FAM30A	0.505070239	5.59E-36	postive
TRBC1	FAM30A	0.572092857	7.86E-48	postive
TRBJ2-7	FAM30A	0.523292793	6.07E-39	postive
CREB1	AL596325.2	0.687147421	5.56E-76	postive
UBR1	AL596325.2	0.590664157	1.27E-51	postive
ZC3HAV1	AL596325.2	0.519794304	2.32E-38	postive
ZC3HAV1L	AL596325.2	0.516361991	8.55E-38	postive
IL15	AL596325.2	0.512752216	3.31E-37	postive
MAPK8	AL596325.2	0.533521363	1.09E-40	postive
LMBR1	AL596325.2	0.501763658	1.85E-35	postive
IREB2	AL596325.2	0.562923874	4.78E-46	postive
DDX17	AL596325.2	0.57631775	1.13E-48	postive
JAK2	AL596325.2	0.5094276	1.13E-36	postive
TXK	AL596325.2	0.531745962	2.22E-40	postive
NFAT5	AL596325.2	0.705203361	1.22E-81	postive
PIK3R1	AL596325.2	0.524169596	4.33E-39	postive
PIK3CA	AL596325.2	0.598067959	3.35E-53	postive
AKT3	AL596325.2	0.564703563	2.17E-46	postive
GNRH1	AL596325.2	0.717318087	1.10E-85	postive
IL6ST	AL596325.2	0.54364357	1.79E-42	postive
RABEP1	AL596325.2	0.515712003	1.09E-37	postive
ACVR2A	AL596325.2	0.545125586	9.70E-43	postive

ANGPTL1	AL596325.2	0.721194966	5.02E-87	postive
BMPR2	AL596325.2	0.639847923	6.02E-63	postive
CRLF3	AL596325.2	0.583243416	4.46E-50	postive
NR2C2	AL596325.2	0.639042879	9.59E-63	postive
RORA	AL596325.2	0.690313193	6.06E-77	postive
SOS1	AL596325.2	0.604409347	1.37E-54	postive
SOS2	AL596325.2	0.513893311	2.16E-37	postive
BRAF	AL596325.2	0.734364331	9.44E-92	postive
CBL	AL596325.2	0.58066496	1.50E-49	postive
CBLB	AL596325.2	0.659364669	4.87E-68	postive
PDK1	AL596325.2	0.545946569	6.90E-43	postive
TRAJ1	AL596325.2	0.564809941	2.07E-46	postive
TRAJ2	AL596325.2	0.616989595	1.96E-57	postive
TRAJ3	AL596325.2	0.667984144	2.07E-70	postive
TRAJ5	AL596325.2	0.60267952	3.31E-54	postive
TRAJ6	AL596325.2	0.634190662	1.54E-61	postive
TRAJ8	AL596325.2	0.637496586	2.34E-62	postive
TRAJ10	AL596325.2	0.605469069	8.01E-55	postive
TRAJ13	AL596325.2	0.645704282	1.95E-64	postive
TRAJ14	AL596325.2	0.606137032	5.69E-55	postive
TRAJ16	AL596325.2	0.612462301	2.15E-56	postive
TRAJ17	AL596325.2	0.592097659	6.35E-52	postive
TRAJ18	AL596325.2	0.514584932	1.67E-37	postive
TRAJ21	AL596325.2	0.652628583	3.08E-66	postive
TRAJ31	AL596325.2	0.651127938	7.63E-66	postive
TRAJ37	AL596325.2	0.627333365	7.16E-60	postive
TRAJ38	AL596325.2	0.68632834	9.81E-76	postive
TRAJ39	AL596325.2	0.659398051	4.77E-68	postive
CREB1	SMC5-AS1	0.7732536	1.58E-107	postive
RFXAP	SMC5-AS1	0.548328631	2.55E-43	postive
UBR1	SMC5-AS1	0.733940227	1.35E-91	postive
ZC3HAV1	SMC5-AS1	0.54424964	1.40E-42	postive
IL15	SMC5-AS1	0.505206398	5.32E-36	postive
CYLD	SMC5-AS1	0.61611108	3.13E-57	postive
EIF2AK2	SMC5-AS1	0.533010664	1.34E-40	postive
MAPK8	SMC5-AS1	0.542325722	3.09E-42	postive
LMBR1	SMC5-AS1	0.51333805	2.66E-37	postive
IREB2	SMC5-AS1	0.641537619	2.26E-63	postive
DDX17	SMC5-AS1	0.661260363	1.49E-68	postive
PIK3CG	SMC5-AS1	0.504113463	7.91E-36	postive
JAK2	SMC5-AS1	0.597067624	5.51E-53	postive
TXK	SMC5-AS1	0.621500476	1.74E-58	postive
NFAT5	SMC5-AS1	0.84220209	4.16E-145	postive
NFATC3	SMC5-AS1	0.532305733	1.78E-40	postive
PIK3R1	SMC5-AS1	0.653415424	1.91E-66	postive
PIK3CA	SMC5-AS1	0.632798292	3.38E-61	postive
IGHD6-6	SMC5-AS1	0.520107812	2.06E-38	postive
ROBO2	SMC5-AS1	0.55519389	1.38E-44	postive
GNRH1	SMC5-AS1	0.78008997	1.25E-110	postive
IL6ST	SMC5-AS1	0.647695274	5.99E-65	postive
RABEP1	SMC5-AS1	0.536256448	3.65E-41	postive
ACVR2A	SMC5-AS1	0.661165342	1.58E-68	postive
ANGPTL1	SMC5-AS1	0.676126746	1.00E-72	postive
BMPR1A	SMC5-AS1	0.615884571	3.53E-57	postive
BMPR2	SMC5-AS1	0.736096209	2.15E-92	postive
CRLF3	SMC5-AS1	0.612468661	2.14E-56	postive
NR1D2	SMC5-AS1	0.576172552	1.21E-48	postive
NR2C2	SMC5-AS1	0.743244098	4.22E-95	postive
NR3C1	SMC5-AS1	0.515228374	1.31E-37	postive
NR3C2	SMC5-AS1	0.549732536	1.41E-43	postive
RORA	SMC5-AS1	0.827834255	6.40E-136	postive
SOS1	SMC5-AS1	0.630141306	1.50E-60	postive
SOS2	SMC5-AS1	0.537712708	2.03E-41	postive
BRAF	SMC5-AS1	0.795454685	5.01E-118	postive
TEC	SMC5-AS1	0.548174477	2.72E-43	postive

CBL	SMC5-AS1	0.505378599	5.00E-36	postive
CBLB	SMC5-AS1	0.653021206	2.42E-66	postive
RASGRP1	SMC5-AS1	0.522273967	8.99E-39	postive
PDK1	SMC5-AS1	0.507102713	2.66E-36	postive
TRAJ1	SMC5-AS1	0.668101515	1.92E-70	postive
TRAJ2	SMC5-AS1	0.72194187	2.76E-87	postive
TRAJ3	SMC5-AS1	0.746703481	1.92E-96	postive
TRAJ5	SMC5-AS1	0.693054696	8.70E-78	postive
TRAJ6	SMC5-AS1	0.740460255	4.90E-94	postive
TRAJ8	SMC5-AS1	0.698699702	1.49E-79	postive
TRAJ10	SMC5-AS1	0.708989399	6.98E-83	postive
TRAJ12	SMC5-AS1	0.694079971	4.18E-78	postive
TRAJ13	SMC5-AS1	0.705012741	1.40E-81	postive
TRAJ14	SMC5-AS1	0.69490871	2.31E-78	postive
TRAJ16	SMC5-AS1	0.710176904	2.82E-83	postive
TRAJ17	SMC5-AS1	0.666417747	5.66E-70	postive
TRAJ18	SMC5-AS1	0.684124521	4.49E-75	postive
TRAJ21	SMC5-AS1	0.737436153	6.79E-93	postive
TRAJ31	SMC5-AS1	0.759837729	9.55E-102	postive
TRAJ37	SMC5-AS1	0.761637307	1.69E-102	postive
TRAJ38	SMC5-AS1	0.804749193	8.13E-123	postive
TRAJ39	SMC5-AS1	0.76275089	5.72E-103	postive
CREB1	AC087276.2	0.811030396	3.35E-126	postive
RFXAP	AC087276.2	0.529749874	4.89E-40	postive
UBR1	AC087276.2	0.688593679	2.03E-76	postive
ZC3HAV1	AC087276.2	0.582550846	6.18E-50	postive
ZC3HAV1L	AC087276.2	0.528743639	7.27E-40	postive
IL15	AC087276.2	0.584592869	2.35E-50	postive
CYLD	AC087276.2	0.625248166	2.26E-59	postive
EIF2AK2	AC087276.2	0.568588972	3.83E-47	postive
MAPK8	AC087276.2	0.600722681	8.88E-54	postive
LMBR1	AC087276.2	0.596507552	7.27E-53	postive
LIMS1	AC087276.2	0.520340956	1.89E-38	postive
IREB2	AC087276.2	0.641281979	2.62E-63	postive
DDX17	AC087276.2	0.646417651	1.28E-64	postive
PIK3CG	AC087276.2	0.549267784	1.72E-43	postive
JAK2	AC087276.2	0.67125566	2.48E-71	postive
TXK	AC087276.2	0.637410663	2.45E-62	postive
PPP3CB	AC087276.2	0.503126924	1.13E-35	postive
NFAT5	AC087276.2	0.870598836	2.59E-166	postive
MALT1	AC087276.2	0.558595676	3.18E-45	postive
PIK3R1	AC087276.2	0.657744408	1.33E-67	postive
PIK3CA	AC087276.2	0.705824218	7.64E-82	postive
PIK3CB	AC087276.2	0.542321031	3.09E-42	postive
AKT3	AC087276.2	0.564411223	2.47E-46	postive
RASGRP3	AC087276.2	0.551658341	6.25E-44	postive
IGHD6-6	AC087276.2	0.508400758	1.66E-36	postive
PLXNC1	AC087276.2	0.503657634	9.33E-36	postive
GNRH1	AC087276.2	0.809451278	2.44E-125	postive
IL6ST	AC087276.2	0.634240893	1.50E-61	postive
RABEP1	AC087276.2	0.597573447	4.28E-53	postive
ACVR2A	AC087276.2	0.669404008	8.26E-71	postive
ANGPTL1	AC087276.2	0.793387451	5.39E-117	postive
BMPR1A	AC087276.2	0.580143323	1.92E-49	postive
BMPR2	AC087276.2	0.755385679	6.54E-100	postive
CRLF3	AC087276.2	0.679690643	9.18E-74	postive
NR1D2	AC087276.2	0.534423598	7.63E-41	postive
NR2C1	AC087276.2	0.529114488	6.28E-40	postive
NR2C2	AC087276.2	0.735307737	4.22E-92	postive
RORA	AC087276.2	0.859741502	1.17E-157	postive
SOS1	AC087276.2	0.717899372	6.94E-86	postive
SOS2	AC087276.2	0.595623888	1.13E-52	postive
BRAF	AC087276.2	0.832268692	1.16E-138	postive
ITK	AC087276.2	0.523135312	6.45E-39	postive
TEC	AC087276.2	0.515568093	1.15E-37	postive

CBL	AC087276.2	0.624602003	3.22E-59	postive
CBLB	AC087276.2	0.765084684	5.82E-104	postive
RASGRP1	AC087276.2	0.519125271	3.00E-38	postive
PDK1	AC087276.2	0.623202257	6.91E-59	postive
TRAJ1	AC087276.2	0.755808173	4.39E-100	postive
TRAJ2	AC087276.2	0.744642099	1.22E-95	postive
TRAJ3	AC087276.2	0.791423098	5.03E-116	postive
TRAJ5	AC087276.2	0.812244876	7.17E-127	postive
TRAJ6	AC087276.2	0.811261752	2.50E-126	postive
TRAJ8	AC087276.2	0.776261037	7.04E-109	postive
TRAJ10	AC087276.2	0.779732917	1.83E-110	postive
TRAJ12	AC087276.2	0.731377445	1.18E-90	postive
TRAJ13	AC087276.2	0.802171449	1.84E-121	postive
TRAJ14	AC087276.2	0.784604428	9.70E-113	postive
TRAJ16	AC087276.2	0.794187565	2.16E-117	postive
TRAJ17	AC087276.2	0.748635171	3.34E-97	postive
TRAJ18	AC087276.2	0.730415204	2.64E-90	postive
TRAJ21	AC087276.2	0.822562598	9.23E-133	postive
TRAJ31	AC087276.2	0.846607475	4.13E-148	postive
TRAJ37	AC087276.2	0.820156958	2.36E-131	postive
TRAJ38	AC087276.2	0.879766328	2.95E-174	postive
TRAJ39	AC087276.2	0.839910126	1.40E-143	postive
IGHV1-24	AC133528.1	0.563569916	3.59E-46	postive
IGKV2-24	AC133528.1	0.637909644	1.84E-62	postive
IGKV2D-24	AC133528.1	0.506400206	3.44E-36	postive
CREB1	AL138921.2	0.631135786	8.62E-61	postive
UBR1	AL138921.2	0.578842992	3.51E-49	postive
CYLD	AL138921.2	0.526228904	1.94E-39	postive
DDX17	AL138921.2	0.661547958	1.24E-68	postive
JAK2	AL138921.2	0.501146946	2.30E-35	postive
NFAT5	AL138921.2	0.681528251	2.65E-74	postive
PIK3R1	AL138921.2	0.511764871	4.78E-37	postive
GNRH1	AL138921.2	0.705765992	7.98E-82	postive
RABEP1	AL138921.2	0.511892135	4.56E-37	postive
ACVR2A	AL138921.2	0.51921578	2.90E-38	postive
ANGPTL1	AL138921.2	0.570975225	1.31E-47	postive
BMPR1A	AL138921.2	0.506424172	3.41E-36	postive
BMPR2	AL138921.2	0.550685591	9.44E-44	postive
CRLF3	AL138921.2	0.51827585	4.14E-38	postive
NR2C1	AL138921.2	0.524613308	3.64E-39	postive
NR2C2	AL138921.2	0.683815982	5.55E-75	postive
RORA	AL138921.2	0.646285029	1.39E-64	postive
SOS1	AL138921.2	0.552961778	3.59E-44	postive
BRAF	AL138921.2	0.658503004	8.33E-68	postive
CBLB	AL138921.2	0.568858403	3.40E-47	postive
TRAJ1	AL138921.2	0.550020274	1.25E-43	postive
TRAJ2	AL138921.2	0.60302268	2.78E-54	postive
TRAJ3	AL138921.2	0.581782098	8.88E-50	postive
TRAJ5	AL138921.2	0.523085181	6.58E-39	postive
TRAJ6	AL138921.2	0.584649602	2.29E-50	postive
TRAJ8	AL138921.2	0.532074213	1.95E-40	postive
TRAJ10	AL138921.2	0.536554962	3.24E-41	postive
TRAJ13	AL138921.2	0.544267734	1.38E-42	postive
TRAJ14	AL138921.2	0.531879853	2.10E-40	postive
TRAJ16	AL138921.2	0.542137272	3.33E-42	postive
TRAJ21	AL138921.2	0.5494938	1.56E-43	postive
TRAJ31	AL138921.2	0.564429146	2.46E-46	postive
TRAJ37	AL138921.2	0.573498669	4.14E-48	postive
TRAJ38	AL138921.2	0.616342815	2.77E-57	postive
TRAJ39	AL138921.2	0.565331414	1.64E-46	postive
NFKBIZ	IGBP1-AS1	0.504303621	7.38E-36	postive
DDX17	IGBP1-AS1	0.579273602	2.88E-49	postive
NFAT5	IGBP1-AS1	0.516920284	6.92E-38	postive
LTB4R2	IGBP1-AS1	0.586532982	9.33E-51	postive
GNRH1	IGBP1-AS1	0.61337298	1.33E-56	postive

NR2C2	IGBP1-AS1	0.625113952	2.43E-59	postive
BRAF	IGBP1-AS1	0.532906985	1.40E-40	postive
CBLB	IGBP1-AS1	0.515741749	1.08E-37	postive
TRAJ1	IGBP1-AS1	0.609604948	9.52E-56	postive
TRAJ3	IGBP1-AS1	0.567211995	7.11E-47	postive
TRAJ5	IGBP1-AS1	0.605650381	7.30E-55	postive
TRAJ6	IGBP1-AS1	0.553718069	2.60E-44	postive
TRAJ8	IGBP1-AS1	0.572685027	6.00E-48	postive
TRAJ10	IGBP1-AS1	0.532363132	1.74E-40	postive
TRAJ12	IGBP1-AS1	0.591372619	9.04E-52	postive
TRAJ13	IGBP1-AS1	0.571563749	1.00E-47	postive
TRAJ14	IGBP1-AS1	0.593963127	2.55E-52	postive
TRAJ16	IGBP1-AS1	0.5984984	2.70E-53	postive
TRAJ17	IGBP1-AS1	0.591119135	1.02E-51	postive
TRAJ18	IGBP1-AS1	0.547863906	3.10E-43	postive
TRAJ21	IGBP1-AS1	0.587544514	5.74E-51	postive
TRAJ31	IGBP1-AS1	0.587370055	6.25E-51	postive
TRAJ37	IGBP1-AS1	0.539114673	1.15E-41	postive
TRAJ38	IGBP1-AS1	0.586458539	9.66E-51	postive
TRAJ39	IGBP1-AS1	0.572263636	7.27E-48	postive
CD4	LINC01857	0.517900768	4.78E-38	postive
HLA-DMB	LINC01857	0.508700529	1.48E-36	postive
LTA	LINC01857	0.663114339	4.63E-69	postive
CXCL13	LINC01857	0.735578227	3.35E-92	postive
PTGDS	LINC01857	0.638284931	1.48E-62	postive
RBP5	LINC01857	0.668145976	1.86E-70	postive
CD40LG	LINC01857	0.605231444	9.04E-55	postive
CCL19	LINC01857	0.732477619	4.67E-91	postive
CCR7	LINC01857	0.714725777	8.39E-85	postive
CCR5	LINC01857	0.501343211	2.15E-35	postive
CXCR4	LINC01857	0.646726024	1.07E-64	postive
CD79A	LINC01857	0.553353605	3.04E-44	postive
CD79B	LINC01857	0.721780605	3.14E-87	postive
BTK	LINC01857	0.664223413	2.29E-69	postive
RAC2	LINC01857	0.552689588	4.03E-44	postive
CD19	LINC01857	0.688012796	3.04E-76	postive
CR2	LINC01857	0.634799435	1.09E-61	postive
CD22	LINC01857	0.726161281	8.94E-89	postive
CD72	LINC01857	0.58255511	6.17E-50	postive
PLCG2	LINC01857	0.505339491	5.07E-36	postive
PRKCB	LINC01857	0.559108493	2.54E-45	postive
GMFG	LINC01857	0.61112415	4.32E-56	postive
IL16	LINC01857	0.698297871	1.99E-79	postive
LTB	LINC01857	0.589771654	1.96E-51	postive
TNFSF13B	LINC01857	0.534518319	7.35E-41	postive
TNFSF8	LINC01857	0.600859793	8.28E-54	postive
CSF2RB	LINC01857	0.513145619	2.86E-37	postive
IL10RA	LINC01857	0.563958363	3.02E-46	postive
IL2RG	LINC01857	0.557296277	5.58E-45	postive
TNFRSF13C	LINC01857	0.636732028	3.62E-62	postive
ITGAL	LINC01857	0.574104281	3.14E-48	postive
LCK	LINC01857	0.553898842	2.41E-44	postive
NCR3	LINC01857	0.806871514	6.03E-124	postive
CD247	LINC01857	0.609591484	9.58E-56	postive
HCST	LINC01857	0.56594737	1.25E-46	postive
CD48	LINC01857	0.751965776	1.58E-98	postive
SH2D1A	LINC01857	0.612233013	2.42E-56	postive
CD3D	LINC01857	0.607628024	2.64E-55	postive
CD3E	LINC01857	0.655468485	5.43E-67	postive
CD3G	LINC01857	0.568167771	4.63E-47	postive
PTPRC	LINC01857	0.521927524	1.03E-38	postive
TRAC	LINC01857	0.638058614	1.69E-62	postive
TRAV4	LINC01857	0.560335125	1.49E-45	postive
TRAV8-2	LINC01857	0.551866952	5.72E-44	postive
TRAV8-4	LINC01857	0.525829821	2.27E-39	postive

TRAV8-6	LINC01857	0.546343022	5.85E-43	postive
TRAV9-2	LINC01857	0.543740001	1.72E-42	postive
TRAV16	LINC01857	0.557522026	5.06E-45	postive
TRAV29DV5	LINC01857	0.519980014	2.17E-38	postive
TRBC1	LINC01857	0.562502128	5.75E-46	postive
TRBC2	LINC01857	0.63551387	7.26E-62	postive
TRBJ2-3	LINC01857	0.516324756	8.67E-38	postive
TRBV2	LINC01857	0.607069346	3.53E-55	postive
TRBV3-1	LINC01857	0.507248383	2.53E-36	postive
TRBV5-1	LINC01857	0.70265164	8.15E-81	postive
TRBV5-4	LINC01857	0.549222475	1.75E-43	postive
TRBV6-1	LINC01857	0.527573265	1.15E-39	postive
TRBV6-5	LINC01857	0.520005964	2.14E-38	postive
TRBV10-3	LINC01857	0.513807542	2.23E-37	postive
TRBV11-2	LINC01857	0.553211189	3.23E-44	postive
TRBV18	LINC01857	0.62396295	4.57E-59	postive
TRBV20-1	LINC01857	0.662373065	7.39E-69	postive
TRBV28	LINC01857	0.64316393	8.73E-64	postive
CREB1	AC007319.1	0.608266586	1.90E-55	postive
UBR1	AC007319.1	0.575157341	1.93E-48	postive
CYLD	AC007319.1	0.516435668	8.31E-38	postive
DDX17	AC007319.1	0.500070403	3.39E-35	postive
JAK2	AC007319.1	0.519664263	2.44E-38	postive
TXK	AC007319.1	0.538881592	1.26E-41	postive
NFAT5	AC007319.1	0.770621827	2.31E-106	postive
NFATC3	AC007319.1	0.516542982	7.98E-38	postive
PIK3CA	AC007319.1	0.522267616	9.01E-39	postive
ROBO2	AC007319.1	0.508091252	1.85E-36	postive
GNRH1	AC007319.1	0.708320076	1.16E-82	postive
IL6ST	AC007319.1	0.501061812	2.38E-35	postive
ANGPTL1	AC007319.1	0.501023414	2.41E-35	postive
BMPR2	AC007319.1	0.573571269	4.00E-48	postive
CRLF3	AC007319.1	0.568083158	4.81E-47	postive
NR2C2	AC007319.1	0.570054896	1.98E-47	postive
RORA	AC007319.1	0.699207251	1.03E-79	postive
BRAF	AC007319.1	0.572433544	6.73E-48	postive
TEC	AC007319.1	0.540413755	6.76E-42	postive
CBLB	AC007319.1	0.535755859	4.47E-41	postive
TRAJ1	AC007319.1	0.594116683	2.36E-52	postive
TRAJ2	AC007319.1	0.563849282	3.17E-46	postive
TRAJ3	AC007319.1	0.613372914	1.33E-56	postive
TRAJ5	AC007319.1	0.599787223	1.42E-53	postive
TRAJ6	AC007319.1	0.64522535	2.59E-64	postive
TRAJ8	AC007319.1	0.550319421	1.10E-43	postive
TRAJ10	AC007319.1	0.628649758	3.45E-60	postive
TRAJ12	AC007319.1	0.553407481	2.97E-44	postive
TRAJ13	AC007319.1	0.587836159	4.99E-51	postive
TRAJ14	AC007319.1	0.546272638	6.02E-43	postive
TRAJ16	AC007319.1	0.611086965	4.40E-56	postive
TRAJ17	AC007319.1	0.546380165	5.76E-43	postive
TRAJ18	AC007319.1	0.595436068	1.23E-52	postive
TRAJ21	AC007319.1	0.61548988	4.35E-57	postive
TRAJ31	AC007319.1	0.644930774	3.09E-64	postive
TRAJ37	AC007319.1	0.67744732	4.15E-73	postive
TRAJ38	AC007319.1	0.651029697	8.10E-66	postive
TRAJ39	AC007319.1	0.653487681	1.82E-66	postive
CREB1	AP000873.2	0.685322897	1.97E-75	postive
RFXAP	AP000873.2	0.517755271	5.05E-38	postive
UBR1	AP000873.2	0.635743621	6.37E-62	postive
ZC3HAV1	AP000873.2	0.537228598	2.47E-41	postive
CYLD	AP000873.2	0.60471786	1.17E-54	postive
EIF2AK2	AP000873.2	0.517337158	5.91E-38	postive
MAPK8	AP000873.2	0.5258068	2.29E-39	postive
EED	AP000873.2	0.52235693	8.71E-39	postive
IREB2	AP000873.2	0.562352645	6.14E-46	postive

DDX17	AP000873.2	0.663783249	3.03E-69	postive
JAK2	AP000873.2	0.586972098	7.56E-51	postive
TXK	AP000873.2	0.556898402	6.63E-45	postive
NFAT5	AP000873.2	0.830269126	2.04E-137	postive
NFATC3	AP000873.2	0.522581137	7.99E-39	postive
PIK3R1	AP000873.2	0.554699236	1.71E-44	postive
PIK3CA	AP000873.2	0.563894813	3.11E-46	postive
GNRH1	AP000873.2	0.748690569	3.18E-97	postive
IL6ST	AP000873.2	0.530425304	3.74E-40	postive
RABEP1	AP000873.2	0.53220551	1.85E-40	postive
ACVR2A	AP000873.2	0.559768224	1.91E-45	postive
ANGPTL1	AP000873.2	0.635842046	6.02E-62	postive
BMPR2	AP000873.2	0.588843619	3.08E-51	postive
CRLF3	AP000873.2	0.592677738	4.78E-52	postive
NR2C1	AP000873.2	0.552178064	5.01E-44	postive
NR2C2	AP000873.2	0.728293746	1.54E-89	postive
RORA	AP000873.2	0.783611887	2.85E-112	postive
SOS1	AP000873.2	0.637293629	2.62E-62	postive
SOS2	AP000873.2	0.531857321	2.12E-40	postive
BRAF	AP000873.2	0.74969358	1.27E-97	postive
CBL	AP000873.2	0.567831424	5.39E-47	postive
CBLB	AP000873.2	0.690151603	6.79E-77	postive
PDK1	AP000873.2	0.564714329	2.16E-46	postive
TRAJ1	AP000873.2	0.697483367	3.60E-79	postive
TRAJ2	AP000873.2	0.7254126	1.65E-88	postive
TRAJ3	AP000873.2	0.734467705	8.65E-92	postive
TRAJ5	AP000873.2	0.709107497	6.38E-83	postive
TRAJ6	AP000873.2	0.747556827	8.88E-97	postive
TRAJ8	AP000873.2	0.665401034	1.08E-69	postive
TRAJ10	AP000873.2	0.710770262	1.79E-83	postive
TRAJ12	AP000873.2	0.704833038	1.61E-81	postive
TRAJ13	AP000873.2	0.708109436	1.36E-82	postive
TRAJ14	AP000873.2	0.71105353	1.44E-83	postive
TRAJ16	AP000873.2	0.705487231	9.84E-82	postive
TRAJ17	AP000873.2	0.636887375	3.31E-62	postive
TRAJ18	AP000873.2	0.712985206	3.25E-84	postive
TRAJ21	AP000873.2	0.738429108	2.88E-93	postive
TRAJ31	AP000873.2	0.740634125	4.21E-94	postive
TRAJ37	AP000873.2	0.758781453	2.62E-101	postive
TRAJ38	AP000873.2	0.79901572	7.85E-120	postive
TRAJ39	AP000873.2	0.738255664	3.34E-93	postive
CREB1	AC015871.3	0.595978608	9.44E-53	postive
RFXAP	AC015871.3	0.558788817	2.92E-45	postive
UBR1	AC015871.3	0.538397678	1.54E-41	postive
NFKBIZ	AC015871.3	0.550087903	1.21E-43	postive
IREB2	AC015871.3	0.532132459	1.90E-40	postive
DDX17	AC015871.3	0.657054041	2.04E-67	postive
NFAT5	AC015871.3	0.651373284	6.58E-66	postive
PIK3R1	AC015871.3	0.50830765	1.71E-36	postive
LTB4R2	AC015871.3	0.58525744	1.71E-50	postive
GNRH1	AC015871.3	0.721601256	3.62E-87	postive
IL6ST	AC015871.3	0.521744146	1.10E-38	postive
ACVR2A	AC015871.3	0.525126313	2.99E-39	postive
ANGPTL1	AC015871.3	0.5990082	2.10E-53	postive
BMPR2	AC015871.3	0.558506717	3.30E-45	postive
NR2C2	AC015871.3	0.716725698	1.75E-85	postive
RORA	AC015871.3	0.675888705	1.17E-72	postive
SOS1	AC015871.3	0.562262651	6.39E-46	postive
SOS2	AC015871.3	0.502008585	1.69E-35	postive
BRAF	AC015871.3	0.656121133	3.63E-67	postive
CBL	AC015871.3	0.504808604	6.15E-36	postive
CBLB	AC015871.3	0.564832675	2.05E-46	postive
RASGRP1	AC015871.3	0.512010831	4.36E-37	postive
TRAJ1	AC015871.3	0.560578184	1.34E-45	postive
TRAJ2	AC015871.3	0.565183109	1.76E-46	postive

TRAJ3	AC015871.3	0.647397822	7.15E-65	postive
TRAJ5	AC015871.3	0.585294563	1.68E-50	postive
TRAJ6	AC015871.3	0.581671547	9.35E-50	postive
TRAJ8	AC015871.3	0.560447015	1.42E-45	postive
TRAJ10	AC015871.3	0.587737895	5.24E-51	postive
TRAJ12	AC015871.3	0.500574771	2.83E-35	postive
TRAJ13	AC015871.3	0.559974434	1.74E-45	postive
TRAJ14	AC015871.3	0.552671099	4.06E-44	postive
TRAJ16	AC015871.3	0.564975743	1.93E-46	postive
TRAJ17	AC015871.3	0.568849899	3.41E-47	postive
TRAJ18	AC015871.3	0.52959763	5.19E-40	postive
TRAJ21	AC015871.3	0.588916813	2.97E-51	postive
TRAJ31	AC015871.3	0.604891218	1.08E-54	postive
TRAJ37	AC015871.3	0.596518738	7.23E-53	postive
TRAJ38	AC015871.3	0.627972391	5.03E-60	postive
TRAJ39	AC015871.3	0.612748522	1.85E-56	postive
DDX17	AC232271.1	0.62733228	7.17E-60	postive
IRF9	AC232271.1	0.509279335	1.20E-36	postive
NFAT5	AC232271.1	0.541334494	4.64E-42	postive
LTB4R2	AC232271.1	0.540075342	7.76E-42	postive
GNRH1	AC232271.1	0.641166082	2.80E-63	postive
NR2C2	AC232271.1	0.610842034	5.00E-56	postive
TRAJ1	AC232271.1	0.513304344	2.69E-37	postive
TRAJ2	AC232271.1	0.50776705	2.09E-36	postive
TRAJ3	AC232271.1	0.544794507	1.11E-42	postive
MICA	AL645933.3	0.555632758	1.14E-44	postive
CREB1	AC084824.5	0.716696414	1.79E-85	postive
RFXAP	AC084824.5	0.505334237	5.08E-36	postive
UBR1	AC084824.5	0.586899257	7.83E-51	postive
ZC3HAV1	AC084824.5	0.508863891	1.40E-36	postive
ZC3HAV1L	AC084824.5	0.504568786	6.71E-36	postive
IL15	AC084824.5	0.515560683	1.16E-37	postive
CYLD	AC084824.5	0.523546763	5.51E-39	postive
MAPK8	AC084824.5	0.580523136	1.60E-49	postive
IREB2	AC084824.5	0.590143192	1.64E-51	postive
DDX17	AC084824.5	0.610027774	7.64E-56	postive
JAK2	AC084824.5	0.587145113	6.96E-51	postive
TXK	AC084824.5	0.513846878	2.20E-37	postive
NFAT5	AC084824.5	0.754353689	1.72E-99	postive
PIK3CA	AC084824.5	0.619131328	6.25E-58	postive
GNRH1	AC084824.5	0.754328951	1.76E-99	postive
RABEP1	AC084824.5	0.516908066	6.96E-38	postive
ACVR2A	AC084824.5	0.607345426	3.06E-55	postive
ANGPTL1	AC084824.5	0.673443732	5.91E-72	postive
BMPR1A	AC084824.5	0.525477603	2.60E-39	postive
BMPR2	AC084824.5	0.633134403	2.80E-61	postive
CRLF3	AC084824.5	0.583655998	3.67E-50	postive
NR2C1	AC084824.5	0.579574241	2.50E-49	postive
NR2C2	AC084824.5	0.66195836	9.60E-69	postive
RORA	AC084824.5	0.715273845	5.47E-85	postive
SOS1	AC084824.5	0.650423178	1.17E-65	postive
SOS2	AC084824.5	0.522519834	8.18E-39	postive
BRAF	AC084824.5	0.683706897	5.98E-75	postive
CBL	AC084824.5	0.54923177	1.74E-43	postive
CBLB	AC084824.5	0.639077289	9.40E-63	postive
PDK1	AC084824.5	0.581371447	1.08E-49	postive
TRAJ1	AC084824.5	0.640983906	3.12E-63	postive
TRAJ2	AC084824.5	0.619109709	6.32E-58	postive
TRAJ3	AC084824.5	0.681800162	2.20E-74	postive
TRAJ5	AC084824.5	0.660613343	2.23E-68	postive
TRAJ6	AC084824.5	0.697470306	3.64E-79	postive
TRAJ8	AC084824.5	0.649410552	2.15E-65	postive
TRAJ10	AC084824.5	0.685420882	1.84E-75	postive
TRAJ12	AC084824.5	0.586544922	9.27E-51	postive
TRAJ13	AC084824.5	0.664401534	2.05E-69	postive

TRAJ14	AC084824.5	0.642090885	1.63E-63	postive
TRAJ16	AC084824.5	0.690339206	5.95E-77	postive
TRAJ17	AC084824.5	0.651567378	5.85E-66	postive
TRAJ18	AC084824.5	0.619181111	6.08E-58	postive
TRAJ21	AC084824.5	0.686226911	1.05E-75	postive
TRAJ31	AC084824.5	0.705411767	1.04E-81	postive
TRAJ37	AC084824.5	0.682255731	1.61E-74	postive
TRAJ38	AC084824.5	0.731100263	1.49E-90	postive
TRAJ39	AC084824.5	0.694730916	2.62E-78	postive
CREB1	AF117829.1	0.739696304	9.56E-94	postive
UBR1	AF117829.1	0.59088016	1.15E-51	postive
ZC3HAV1	AF117829.1	0.570178471	1.87E-47	postive
ZC3HAV1L	AF117829.1	0.522363299	8.69E-39	postive
IL15	AF117829.1	0.6848917	2.65E-75	postive
CYLD	AF117829.1	0.587584186	5.64E-51	postive
EIF2AK2	AF117829.1	0.558512383	3.29E-45	postive
MAPK8	AF117829.1	0.540942192	5.45E-42	postive
TLR1	AF117829.1	0.531453197	2.49E-40	postive
NFKBIZ	AF117829.1	0.514634972	1.64E-37	postive
LMBR1	AF117829.1	0.571968047	8.32E-48	postive
IREB2	AF117829.1	0.549164349	1.79E-43	postive
DDX17	AF117829.1	0.576646126	9.74E-49	postive
PIK3CG	AF117829.1	0.540912165	5.51E-42	postive
JAK2	AF117829.1	0.661016677	1.74E-68	postive
PTK2	AF117829.1	0.509350403	1.17E-36	postive
TXK	AF117829.1	0.547245282	4.01E-43	postive
NFAT5	AF117829.1	0.789145519	6.49E-115	postive
MALT1	AF117829.1	0.54457931	1.22E-42	postive
PIK3R1	AF117829.1	0.565366955	1.62E-46	postive
PIK3CA	AF117829.1	0.68509572	2.30E-75	postive
PIK3CB	AF117829.1	0.568439764	4.10E-47	postive
AKT3	AF117829.1	0.511963817	4.44E-37	postive
RASGRP3	AF117829.1	0.528429857	8.22E-40	postive
PLXNC1	AF117829.1	0.510322888	8.15E-37	postive
GNRH1	AF117829.1	0.713291913	2.56E-84	postive
IL6ST	AF117829.1	0.524238509	4.21E-39	postive
IL7	AF117829.1	0.530438933	3.72E-40	postive
RABEP1	AF117829.1	0.510686213	7.13E-37	postive
ACVR2A	AF117829.1	0.556096285	9.37E-45	postive
ANGPTL1	AF117829.1	0.779586687	2.13E-110	postive
BMPR2	AF117829.1	0.617356078	1.61E-57	postive
CRLF3	AF117829.1	0.66536354	1.11E-69	postive
IL18R1	AF117829.1	0.531280893	2.67E-40	postive
NR2C2	AF117829.1	0.678446966	2.12E-73	postive
RORA	AF117829.1	0.734669263	7.28E-92	postive
SOS1	AF117829.1	0.65630699	3.24E-67	postive
SOS2	AF117829.1	0.567760271	5.56E-47	postive
BRAF	AF117829.1	0.762315123	8.74E-103	postive
ITK	AF117829.1	0.535841814	4.32E-41	postive
CBL	AF117829.1	0.633719603	2.01E-61	postive
CBLB	AF117829.1	0.712676148	4.12E-84	postive
RASGRP1	AF117829.1	0.519094583	3.04E-38	postive
PDK1	AF117829.1	0.636856959	3.37E-62	postive
TRAJ1	AF117829.1	0.771495712	9.52E-107	postive
TRAJ2	AF117829.1	0.715378963	5.04E-85	postive
TRAJ3	AF117829.1	0.82192369	2.19E-132	postive
TRAJ5	AF117829.1	0.81198575	9.97E-127	postive
TRAJ6	AF117829.1	0.823919222	1.45E-133	postive
TRAJ8	AF117829.1	0.773673127	1.03E-107	postive
TRAJ10	AF117829.1	0.819356572	6.85E-131	postive
TRAJ12	AF117829.1	0.741560152	1.87E-94	postive
TRAJ13	AF117829.1	0.804871261	7.01E-123	postive
TRAJ14	AF117829.1	0.813778287	1.01E-127	postive
TRAJ16	AF117829.1	0.80518545	4.78E-123	postive
TRAJ17	AF117829.1	0.799083752	7.24E-120	postive

TRAJ18	AF117829.1	0.775008343	2.59E-108	postive
TRAJ21	AF117829.1	0.843894639	2.99E-146	postive
TRAJ31	AF117829.1	0.820450956	1.59E-131	postive
TRAJ37	AF117829.1	0.784045239	1.78E-112	postive
TRAJ38	AF117829.1	0.859648047	1.38E-157	postive
TRAJ39	AF117829.1	0.83804389	2.34E-142	postive
CREB1	AL512791.1	0.628104483	4.67E-60	postive
CIITA	AL512791.1	0.520350667	1.88E-38	postive
RFXAP	AL512791.1	0.536804759	2.93E-41	postive
UBR1	AL512791.1	0.509306072	1.19E-36	postive
ZC3HAV1	AL512791.1	0.522374159	8.65E-39	postive
IL15	AL512791.1	0.5248166	3.37E-39	postive
CYLD	AL512791.1	0.591503434	8.48E-52	postive
NFKBIZ	AL512791.1	0.545218127	9.34E-43	postive
DDX17	AL512791.1	0.707957556	1.53E-82	postive
JAK2	AL512791.1	0.580553156	1.58E-49	postive
TXK	AL512791.1	0.575411386	1.72E-48	postive
NFAT5	AL512791.1	0.709490186	4.76E-83	postive
MALT1	AL512791.1	0.528167565	9.11E-40	postive
PIK3R1	AL512791.1	0.580028537	2.02E-49	postive
PIK3CA	AL512791.1	0.567118959	7.41E-47	postive
IGHD6-6	AL512791.1	0.512545049	3.57E-37	postive
LTB4R2	AL512791.1	0.572826401	5.63E-48	postive
GNRH1	AL512791.1	0.739746208	9.15E-94	postive
IL6ST	AL512791.1	0.519333354	2.77E-38	postive
RABEP1	AL512791.1	0.526085825	2.06E-39	postive
ACVR2A	AL512791.1	0.501232482	2.23E-35	postive
ANGPTL1	AL512791.1	0.639984499	5.56E-63	postive
BMPR2	AL512791.1	0.551317704	7.22E-44	postive
CRLF3	AL512791.1	0.520911122	1.52E-38	postive
NR2C1	AL512791.1	0.551548013	6.55E-44	postive
NR2C2	AL512791.1	0.708257519	1.22E-82	postive
RORA	AL512791.1	0.733165131	2.61E-91	postive
SOS1	AL512791.1	0.55798336	4.14E-45	postive
SOS2	AL512791.1	0.532641019	1.55E-40	postive
BRAF	AL512791.1	0.665912886	7.81E-70	postive
ITK	AL512791.1	0.572153827	7.64E-48	postive
CBL	AL512791.1	0.605315465	8.66E-55	postive
CBLB	AL512791.1	0.643042312	9.37E-64	postive
PDK1	AL512791.1	0.51904424	3.09E-38	postive
TRAJ1	AL512791.1	0.6177801	1.29E-57	postive
TRAJ2	AL512791.1	0.592014032	6.61E-52	postive
TRAJ3	AL512791.1	0.669263592	9.05E-71	postive
TRAJ5	AL512791.1	0.638634273	1.21E-62	postive
TRAJ6	AL512791.1	0.620705921	2.68E-58	postive
TRAJ8	AL512791.1	0.552981716	3.56E-44	postive
TRAJ10	AL512791.1	0.616061829	3.21E-57	postive
TRAJ12	AL512791.1	0.542843007	2.49E-42	postive
TRAJ13	AL512791.1	0.594990841	1.54E-52	postive
TRAJ14	AL512791.1	0.583637454	3.70E-50	postive
TRAJ16	AL512791.1	0.608952378	1.33E-55	postive
TRAJ17	AL512791.1	0.588104566	4.39E-51	postive
TRAJ18	AL512791.1	0.62307099	7.42E-59	postive
TRAJ21	AL512791.1	0.637837065	1.92E-62	postive
TRAJ31	AL512791.1	0.618680337	7.95E-58	postive
TRAJ37	AL512791.1	0.622319101	1.12E-58	postive
TRAJ38	AL512791.1	0.654391257	1.05E-66	postive
TRAJ39	AL512791.1	0.648939204	2.85E-65	postive
TYK2	AL139287.1	0.522804823	7.33E-39	postive
IRF9	AL139287.1	0.63711605	2.91E-62	postive
LTB4R2	AL139287.1	0.522305926	8.88E-39	postive
NR2C2	AL139287.1	0.511352763	5.57E-37	postive
PLCG1	AL139287.1	0.528919216	6.78E-40	postive
PGLYRP4	AC110741.1	0.571089941	1.24E-47	postive
IFNG	LINC01943	0.565120005	1.81E-46	postive

CXCL10	LINC01943	0.527157704	1.35E-39	postive
APOBEC3G	LINC01943	0.5474288	3.72E-43	postive
IL15	LINC01943	0.58418797	2.85E-50	postive
CCL4	LINC01943	0.517668047	5.22E-38	postive
CXCR6	LINC01943	0.604221819	1.51E-54	postive
IL10	LINC01943	0.526406695	1.81E-39	postive
CD86	LINC01943	0.508266108	1.74E-36	postive
FCGR2B	LINC01943	0.523142351	6.44E-39	postive
GMFG	LINC01943	0.582176811	7.37E-50	postive
TNFSF13B	LINC01943	0.636180963	4.96E-62	postive
IL18RAP	LINC01943	0.547711004	3.30E-43	postive
LCP2	LINC01943	0.524353488	4.03E-39	postive
CD48	LINC01943	0.584750624	2.18E-50	postive
SH2D1A	LINC01943	0.645614861	2.06E-64	postive
CD3D	LINC01943	0.589698212	2.04E-51	postive
PTPRC	LINC01943	0.50522411	5.29E-36	postive
ICOS	LINC01943	0.597437463	4.58E-53	postive
TRAV16	LINC01943	0.541692529	4.00E-42	postive
DDX17	AC135050.6	0.509079864	1.29E-36	postive
CREB1	MIR181A2HG	0.653814537	1.49E-66	postive
RFXAP	MIR181A2HG	0.528597293	7.70E-40	postive
IL15	MIR181A2HG	0.522011839	9.95E-39	postive
NFAT5	MIR181A2HG	0.711481149	1.04E-83	postive
PIK3R1	MIR181A2HG	0.515494356	1.19E-37	postive
PIK3CA	MIR181A2HG	0.539956406	8.15E-42	postive
GNRH1	MIR181A2HG	0.552010895	5.38E-44	postive
IL6ST	MIR181A2HG	0.532648173	1.55E-40	postive
ACVR2A	MIR181A2HG	0.55967131	1.99E-45	postive
ANGPTL1	MIR181A2HG	0.649675069	1.83E-65	postive
BMPR1A	MIR181A2HG	0.501376335	2.12E-35	postive
BMPR2	MIR181A2HG	0.558364615	3.51E-45	postive
CRLF3	MIR181A2HG	0.578676873	3.80E-49	postive
NR2C2	MIR181A2HG	0.532513184	1.64E-40	postive
RORA	MIR181A2HG	0.707351255	2.42E-82	postive
SOS1	MIR181A2HG	0.528791342	7.13E-40	postive
BRAF	MIR181A2HG	0.700774728	3.26E-80	postive
CBLB	MIR181A2HG	0.583355054	4.23E-50	postive
TRAJ1	MIR181A2HG	0.686423035	9.19E-76	postive
TRAJ2	MIR181A2HG	0.707453066	2.24E-82	postive
TRAJ3	MIR181A2HG	0.737477945	6.55E-93	postive
TRAJ5	MIR181A2HG	0.744225238	1.77E-95	postive
TRAJ6	MIR181A2HG	0.739471992	1.16E-93	postive
TRAJ8	MIR181A2HG	0.761820429	1.41E-102	postive
TRAJ10	MIR181A2HG	0.726282682	8.09E-89	postive
TRAJ12	MIR181A2HG	0.684744009	2.93E-75	postive
TRAJ13	MIR181A2HG	0.769496143	7.20E-106	postive
TRAJ14	MIR181A2HG	0.768265739	2.48E-105	postive
TRAJ16	MIR181A2HG	0.739768805	8.97E-94	postive
TRAJ17	MIR181A2HG	0.728245005	1.61E-89	postive
TRAJ18	MIR181A2HG	0.68322224	8.33E-75	postive
TRAJ21	MIR181A2HG	0.782714475	7.52E-112	postive
TRAJ31	MIR181A2HG	0.778332053	8.03E-110	postive
TRAJ37	MIR181A2HG	0.770220934	3.47E-106	postive
TRAJ38	MIR181A2HG	0.80008221	2.22E-120	postive
TRAJ39	MIR181A2HG	0.783566929	2.99E-112	postive
NFAT5	COLCA1	0.550563536	9.94E-44	postive
NFATC3	COLCA1	0.709954946	3.34E-83	postive
SYTL1	AC107464.2	0.517101312	6.47E-38	postive
GIPR	AC107464.2	0.522446418	8.41E-39	postive
CREB1	EML4-AS1	0.657008384	2.10E-67	postive
CIITA	EML4-AS1	0.529223088	6.02E-40	postive
TLR4	EML4-AS1	0.674881867	2.29E-72	postive
CYBB	EML4-AS1	0.611841747	2.97E-56	postive
IL15	EML4-AS1	0.639164622	8.94E-63	postive
TLR7	EML4-AS1	0.529810558	4.77E-40	postive

CYLD	EML4-AS1	0.641074839	2.96E-63	postive
TLR8	EML4-AS1	0.543489777	1.91E-42	postive
TLR1	EML4-AS1	0.64581335	1.83E-64	postive
LIMS1	EML4-AS1	0.509397982	1.15E-36	postive
IL7R	EML4-AS1	0.539179556	1.12E-41	postive
PIK3CG	EML4-AS1	0.683196872	8.48E-75	postive
JAK2	EML4-AS1	0.692472382	1.32E-77	postive
NFAT5	EML4-AS1	0.632074419	5.09E-61	postive
PIK3R1	EML4-AS1	0.56341601	3.84E-46	postive
PIK3CA	EML4-AS1	0.590656033	1.28E-51	postive
RASGRP3	EML4-AS1	0.628253819	4.30E-60	postive
PLXNC1	EML4-AS1	0.680029385	7.31E-74	postive
GNRH1	EML4-AS1	0.545586265	8.01E-43	postive
IL7	EML4-AS1	0.604904144	1.07E-54	postive
TNFSF13B	EML4-AS1	0.510183753	8.58E-37	postive
TNFSF8	EML4-AS1	0.538105577	1.73E-41	postive
ANGPTL1	EML4-AS1	0.631872116	5.70E-61	postive
BMPR2	EML4-AS1	0.571645014	9.63E-48	postive
CRLF3	EML4-AS1	0.672808331	8.97E-72	postive
IL18R1	EML4-AS1	0.503644983	9.37E-36	postive
IL18RAP	EML4-AS1	0.518571586	3.70E-38	postive
NR2C2	EML4-AS1	0.529922229	4.57E-40	postive
RORA	EML4-AS1	0.621769825	1.51E-58	postive
LCP2	EML4-AS1	0.581742046	9.05E-50	postive
SOS1	EML4-AS1	0.514495748	1.72E-37	postive
SOS2	EML4-AS1	0.528994317	6.58E-40	postive
BRAF	EML4-AS1	0.615465174	4.41E-57	postive
PTPRC	EML4-AS1	0.69933829	9.34E-80	postive
ITK	EML4-AS1	0.60143977	6.19E-54	postive
CD28	EML4-AS1	0.623837908	4.89E-59	postive
ICOS	EML4-AS1	0.626178544	1.35E-59	postive
CBL	EML4-AS1	0.570521239	1.60E-47	postive
CBLB	EML4-AS1	0.508425814	1.64E-36	postive
RASGRP1	EML4-AS1	0.550492569	1.02E-43	postive
PDK1	EML4-AS1	0.524361128	4.02E-39	postive
TRAJ1	EML4-AS1	0.720844118	6.65E-87	postive
TRAJ2	EML4-AS1	0.627852326	5.37E-60	postive
TRAJ3	EML4-AS1	0.717424907	1.01E-85	postive
TRAJ5	EML4-AS1	0.742620813	7.33E-95	postive
TRAJ6	EML4-AS1	0.721106166	5.39E-87	postive
TRAJ8	EML4-AS1	0.718023789	6.29E-86	postive
TRAJ10	EML4-AS1	0.705116393	1.30E-81	postive
TRAJ12	EML4-AS1	0.648325772	4.11E-65	postive
TRAJ13	EML4-AS1	0.723214075	9.87E-88	postive
TRAJ14	EML4-AS1	0.718045149	6.18E-86	postive
TRAJ16	EML4-AS1	0.715904776	3.34E-85	postive
TRAJ17	EML4-AS1	0.735921409	2.50E-92	postive
TRAJ18	EML4-AS1	0.668662764	1.33E-70	postive
TRAJ21	EML4-AS1	0.735373448	3.99E-92	postive
TRAJ31	EML4-AS1	0.711113785	1.37E-83	postive
TRAJ37	EML4-AS1	0.665161932	1.26E-69	postive
TRAJ38	EML4-AS1	0.7289735	8.77E-90	postive
TRAJ39	EML4-AS1	0.74661247	2.08E-96	postive
CREB1	AC078883.1	0.594394294	2.06E-52	postive
PIK3CG	AC078883.1	0.514040155	2.05E-37	postive
JAK2	AC078883.1	0.568050073	4.88E-47	postive
TXK	AC078883.1	0.555780767	1.07E-44	postive
NFAT5	AC078883.1	0.531163367	2.80E-40	postive
PIK3CA	AC078883.1	0.531245462	2.71E-40	postive
RASGRP3	AC078883.1	0.52446415	3.86E-39	postive
IGKJ5	AC078883.1	0.549795783	1.37E-43	postive
ROBO2	AC078883.1	0.582359421	6.77E-50	postive
GNRH1	AC078883.1	0.597409769	4.65E-53	postive
ACVR2A	AC078883.1	0.557988663	4.13E-45	postive
ANGPTL1	AC078883.1	0.52315158	6.41E-39	postive

BMPR2	AC078883.1	0.625919753	1.56E-59	postive
CRLF3	AC078883.1	0.511155328	5.99E-37	postive
RORA	AC078883.1	0.590143965	1.64E-51	postive
SOS1	AC078883.1	0.50388258	8.60E-36	postive
CBLB	AC078883.1	0.53323824	1.23E-40	postive
PDK1	AC078883.1	0.651111721	7.71E-66	postive
CREB1	ITCH-IT1	0.786637852	1.05E-113	postive
UBR1	ITCH-IT1	0.624659062	3.12E-59	postive
ZC3HAV1	ITCH-IT1	0.553318731	3.08E-44	postive
IL15	ITCH-IT1	0.605815427	6.71E-55	postive
CYLD	ITCH-IT1	0.603632973	2.04E-54	postive
EIF2AK2	ITCH-IT1	0.539396031	1.02E-41	postive
MAPK8	ITCH-IT1	0.572249808	7.32E-48	postive
TLR1	ITCH-IT1	0.516122518	9.36E-38	postive
LMBR1	ITCH-IT1	0.595883564	9.90E-53	postive
LIMS1	ITCH-IT1	0.546333051	5.87E-43	postive
IREB2	ITCH-IT1	0.596342689	7.89E-53	postive
DDX17	ITCH-IT1	0.574232607	2.96E-48	postive
PIK3CG	ITCH-IT1	0.544676084	1.17E-42	postive
JAK2	ITCH-IT1	0.664335987	2.13E-69	postive
TXK	ITCH-IT1	0.600549813	9.68E-54	postive
NFAT5	ITCH-IT1	0.841253067	1.79E-144	postive
MALT1	ITCH-IT1	0.555332435	1.30E-44	postive
PIK3R1	ITCH-IT1	0.639863522	5.97E-63	postive
PIK3CA	ITCH-IT1	0.722376704	1.94E-87	postive
PIK3CB	ITCH-IT1	0.543330815	2.04E-42	postive
AKT3	ITCH-IT1	0.535130918	5.74E-41	postive
RASGRP3	ITCH-IT1	0.530825804	3.20E-40	postive
GNRH1	ITCH-IT1	0.731172488	1.40E-90	postive
IL6ST	ITCH-IT1	0.639410055	7.76E-63	postive
RABEP1	ITCH-IT1	0.547631073	3.41E-43	postive
ACVR2A	ITCH-IT1	0.61275871	1.84E-56	postive
ANGPTL1	ITCH-IT1	0.818959712	1.16E-130	postive
BMPR1A	ITCH-IT1	0.525365983	2.72E-39	postive
BMPR2	ITCH-IT1	0.736494528	1.53E-92	postive
CRLF3	ITCH-IT1	0.653735331	1.57E-66	postive
IL18R1	ITCH-IT1	0.516766988	7.34E-38	postive
NR2C2	ITCH-IT1	0.652950065	2.53E-66	postive
RORA	ITCH-IT1	0.861674259	3.83E-159	postive
SOS1	ITCH-IT1	0.667940117	2.13E-70	postive
SOS2	ITCH-IT1	0.575756094	1.47E-48	postive
BRAF	ITCH-IT1	0.77895953	4.14E-110	postive
ITK	ITCH-IT1	0.535218758	5.55E-41	postive
CBL	ITCH-IT1	0.612477113	2.13E-56	postive
CBLB	ITCH-IT1	0.757274296	1.10E-100	postive
PDK1	ITCH-IT1	0.634840255	1.07E-61	postive
TRAJ1	ITCH-IT1	0.694570237	2.94E-78	postive
TRAJ2	ITCH-IT1	0.681098656	3.54E-74	postive
TRAJ3	ITCH-IT1	0.741817232	1.49E-94	postive
TRAJ5	ITCH-IT1	0.804678358	8.87E-123	postive
TRAJ6	ITCH-IT1	0.768698565	1.60E-105	postive
TRAJ8	ITCH-IT1	0.714736164	8.32E-85	postive
TRAJ10	ITCH-IT1	0.7746645	3.69E-108	postive
TRAJ12	ITCH-IT1	0.662313001	7.68E-69	postive
TRAJ13	ITCH-IT1	0.77994288	1.46E-110	postive
TRAJ14	ITCH-IT1	0.735352056	4.07E-92	postive
TRAJ16	ITCH-IT1	0.764985366	6.42E-104	postive
TRAJ17	ITCH-IT1	0.733464838	2.03E-91	postive
TRAJ18	ITCH-IT1	0.716510869	2.07E-85	postive
TRAJ21	ITCH-IT1	0.805516123	3.19E-123	postive
TRAJ31	ITCH-IT1	0.83222007	1.24E-138	postive
TRAJ37	ITCH-IT1	0.792613666	1.30E-116	postive
TRAJ38	ITCH-IT1	0.846063279	9.82E-148	postive
TRAJ39	ITCH-IT1	0.812649546	4.28E-127	postive
IL15	AC084117.1	0.549409842	1.62E-43	postive

NFKBIZ	AC084117.1	0.533637197	1.04E-40	postive
NFAT5	AC084117.1	0.53199329	2.01E-40	postive
GNRH1	AC084117.1	0.544281606	1.38E-42	postive
ANGPTL1	AC084117.1	0.615744168	3.80E-57	postive
IL18R1	AC084117.1	0.518651901	3.59E-38	postive
RORA	AC084117.1	0.573625989	3.90E-48	postive
BRAF	AC084117.1	0.516394495	8.44E-38	postive
CBLB	AC084117.1	0.546321453	5.90E-43	postive
PDK1	AC084117.1	0.530945113	3.05E-40	postive
TRAJ3	AC084117.1	0.501919341	1.75E-35	postive
TRAJ5	AC084117.1	0.527231243	1.31E-39	postive
TRAJ10	AC084117.1	0.526966804	1.46E-39	postive
TRAJ21	AC084117.1	0.522811483	7.31E-39	postive
TRAJ31	AC084117.1	0.515963591	9.93E-38	postive
TRAJ38	AC084117.1	0.539310941	1.06E-41	postive
TRAJ39	AC084117.1	0.533389522	1.15E-40	postive
CREB1	AL035409.1	0.573078538	5.01E-48	postive
PDGFRA	AL035409.1	0.531546257	2.40E-40	postive
JAK2	AL035409.1	0.584765391	2.17E-50	postive
PIK3CA	AL035409.1	0.544306681	1.36E-42	postive
RASGRP3	AL035409.1	0.512282691	3.94E-37	postive
PLXNC1	AL035409.1	0.546231902	6.12E-43	postive
INHBA	AL035409.1	0.509182355	1.24E-36	postive
ANGPTL1	AL035409.1	0.534216202	8.29E-41	postive
BMPR2	AL035409.1	0.542678931	2.67E-42	postive
RORA	AL035409.1	0.503748406	9.03E-36	postive
BRAF	AL035409.1	0.551164076	7.71E-44	postive
CBLB	AL035409.1	0.604809293	1.12E-54	postive
TRAJ38	AL035409.1	0.519494321	2.61E-38	postive
TYK2	AL135999.1	0.540407638	6.78E-42	postive
DDX17	AL135999.1	0.506878259	2.89E-36	postive
IRF9	AL135999.1	0.741505425	1.96E-94	postive
LTB4R2	AL135999.1	0.553198753	3.24E-44	postive
GIPR	AL135999.1	0.500089569	3.36E-35	postive
PTK2	UBR5-AS1	0.537384586	2.32E-41	postive
BRAF	UBR5-AS1	0.521653194	1.14E-38	postive
TPT1	GAS5	0.595835073	1.01E-52	postive
CREB1	AC097376.3	0.803125369	5.83E-122	postive
RFXAP	AC097376.3	0.590241074	1.57E-51	postive
UBR1	AC097376.3	0.691646917	2.36E-77	postive
ZC3HAV1	AC097376.3	0.582283104	7.01E-50	postive
ZC3HAV1L	AC097376.3	0.528858214	6.95E-40	postive
IL15	AC097376.3	0.529141583	6.21E-40	postive
CYLD	AC097376.3	0.605274079	8.84E-55	postive
EIF2AK2	AC097376.3	0.572016044	8.14E-48	postive
MAPK8	AC097376.3	0.597370643	4.74E-53	postive
LMBR1	AC097376.3	0.561486257	8.99E-46	postive
SP1	AC097376.3	0.507118822	2.65E-36	postive
IREB2	AC097376.3	0.650896686	8.78E-66	postive
DDX17	AC097376.3	0.663150527	4.53E-69	postive
JAK2	AC097376.3	0.603149859	2.61E-54	postive
TXK	AC097376.3	0.585523194	1.51E-50	postive
NFAT5	AC097376.3	0.845152117	4.15E-147	postive
NFATC3	AC097376.3	0.518469872	3.85E-38	postive
MALT1	AC097376.3	0.52200238	9.98E-39	postive
PIK3R1	AC097376.3	0.645681666	1.98E-64	postive
PIK3CA	AC097376.3	0.674840491	2.35E-72	postive
PIK3CB	AC097376.3	0.528761144	7.22E-40	postive
AKT3	AC097376.3	0.538901581	1.25E-41	postive
GSK3B	AC097376.3	0.501270443	2.20E-35	postive
ROBO2	AC097376.3	0.504526144	6.81E-36	postive
GNRH1	AC097376.3	0.773874423	8.34E-108	postive
IL6ST	AC097376.3	0.614023799	9.45E-57	postive
RABEP1	AC097376.3	0.595856701	1.00E-52	postive
ACVR2A	AC097376.3	0.694327426	3.50E-78	postive

ANGPTL1	AC097376.3	0.725337451	1.75E-88	postive
BMPR1A	AC097376.3	0.61260938	1.99E-56	postive
BMPR2	AC097376.3	0.747303609	1.12E-96	postive
CRLF3	AC097376.3	0.623806771	4.97E-59	postive
LIFR	AC097376.3	0.512959358	3.06E-37	postive
NR1D2	AC097376.3	0.568499572	3.99E-47	postive
NR2C1	AC097376.3	0.506861677	2.91E-36	postive
NR2C2	AC097376.3	0.751295309	2.93E-98	postive
NR3C2	AC097376.3	0.501716018	1.88E-35	postive
RORA	AC097376.3	0.819597403	4.97E-131	postive
SOS1	AC097376.3	0.741550373	1.88E-94	postive
SOS2	AC097376.3	0.620969938	2.32E-58	postive
BRAF	AC097376.3	0.83407911	8.34E-140	postive
TEC	AC097376.3	0.506634635	3.16E-36	postive
CBL	AC097376.3	0.570955289	1.32E-47	postive
CBLB	AC097376.3	0.718689496	3.71E-86	postive
PDK1	AC097376.3	0.570010425	2.02E-47	postive
TRAJ1	AC097376.3	0.74595962	3.75E-96	postive
TRAJ2	AC097376.3	0.734581267	7.85E-92	postive
TRAJ3	AC097376.3	0.783179299	4.55E-112	postive
TRAJ5	AC097376.3	0.775675255	1.30E-108	postive
TRAJ6	AC097376.3	0.793670575	3.90E-117	postive
TRAJ8	AC097376.3	0.778379949	7.64E-110	postive
TRAJ10	AC097376.3	0.754315721	1.78E-99	postive
TRAJ12	AC097376.3	0.750974134	3.94E-98	postive
TRAJ13	AC097376.3	0.778784535	4.98E-110	postive
TRAJ14	AC097376.3	0.775460391	1.62E-108	postive
TRAJ16	AC097376.3	0.77574458	1.21E-108	postive
TRAJ17	AC097376.3	0.736088627	2.16E-92	postive
TRAJ18	AC097376.3	0.714111798	1.35E-84	postive
TRAJ21	AC097376.3	0.79619148	2.14E-118	postive
TRAJ31	AC097376.3	0.821286679	5.18E-132	postive
TRAJ37	AC097376.3	0.800454907	1.43E-120	postive
TRAJ38	AC097376.3	0.855408795	2.10E-154	postive
TRAJ39	AC097376.3	0.812584213	4.65E-127	postive
GNRH1	AC019131.2	0.589961719	1.79E-51	postive
RORA	AC019131.2	0.535007605	6.04E-41	postive
BRAF	AC019131.2	0.510030866	9.08E-37	postive
CD1D	AC243960.1	0.550131081	1.19E-43	postive
HLA-DOB	AC243960.1	0.529932387	4.55E-40	postive
LTA	AC243960.1	0.605531921	7.75E-55	postive
CIITA	AC243960.1	0.508779393	1.44E-36	postive
CXCL13	AC243960.1	0.558135224	3.88E-45	postive
APOBEC3G	AC243960.1	0.543324111	2.05E-42	postive
RBP5	AC243960.1	0.608638608	1.57E-55	postive
IL7R	AC243960.1	0.500721791	2.68E-35	postive
CD40LG	AC243960.1	0.625191355	2.33E-59	postive
TRIM22	AC243960.1	0.525059877	3.06E-39	postive
PIK3CG	AC243960.1	0.512066124	4.27E-37	postive
CCL19	AC243960.1	0.546870746	4.69E-43	postive
CCR7	AC243960.1	0.572373701	6.92E-48	postive
CCR5	AC243960.1	0.507924653	1.97E-36	postive
CXCR4	AC243960.1	0.513640367	2.38E-37	postive
CXCR6	AC243960.1	0.593933263	2.59E-52	postive
CCR4	AC243960.1	0.600314789	1.09E-53	postive
CD79B	AC243960.1	0.626509111	1.13E-59	postive
BTK	AC243960.1	0.617425661	1.56E-57	postive
PPP3CC	AC243960.1	0.53831238	1.59E-41	postive
CD19	AC243960.1	0.647353756	7.34E-65	postive
CD22	AC243960.1	0.555124334	1.42E-44	postive
CD72	AC243960.1	0.534694563	6.84E-41	postive
RASGRP3	AC243960.1	0.528292224	8.68E-40	postive
PLCG2	AC243960.1	0.577230061	7.43E-49	postive
PRKCB	AC243960.1	0.546864837	4.70E-43	postive
IGKJ5	AC243960.1	0.550593713	9.81E-44	postive

PLXNC1	AC243960.1	0.506437123	3.40E-36	postive
GMFG	AC243960.1	0.544921771	1.06E-42	postive
IL16	AC243960.1	0.689996667	7.57E-77	postive
TNFSF13B	AC243960.1	0.550421634	1.06E-43	postive
TNFSF8	AC243960.1	0.540188007	7.41E-42	postive
CSF2RB	AC243960.1	0.523828504	4.94E-39	postive
IL10RA	AC243960.1	0.567602328	5.97E-47	postive
IL12RB1	AC243960.1	0.509606284	1.06E-36	postive
IL18RAP	AC243960.1	0.58637385	1.01E-50	postive
IL21R	AC243960.1	0.513119105	2.89E-37	postive
TNFRSF13C	AC243960.1	0.595562022	1.16E-52	postive
TNFRSF17	AC243960.1	0.581280452	1.12E-49	postive
ITGAL	AC243960.1	0.567671171	5.79E-47	postive
LCK	AC243960.1	0.515347014	1.25E-37	postive
NCR3	AC243960.1	0.626651955	1.04E-59	postive
CD247	AC243960.1	0.637860981	1.89E-62	postive
ZAP70	AC243960.1	0.613442909	1.28E-56	postive
CD48	AC243960.1	0.620827406	2.51E-58	postive
SH2D1A	AC243960.1	0.716998729	1.41E-85	postive
CD3D	AC243960.1	0.610941614	4.75E-56	postive
CD3E	AC243960.1	0.596223276	8.37E-53	postive
CD3G	AC243960.1	0.619628849	4.78E-58	postive
PTPRC	AC243960.1	0.634400416	1.37E-61	postive
ITK	AC243960.1	0.659889618	3.51E-68	postive
CD28	AC243960.1	0.608269561	1.90E-55	postive
ICOS	AC243960.1	0.647080302	8.64E-65	postive
CTLA4	AC243960.1	0.555768686	1.08E-44	postive
TRAC	AC243960.1	0.610631907	5.58E-56	postive
TRAV4	AC243960.1	0.591654623	7.88E-52	postive
TRAV8-2	AC243960.1	0.568020162	4.95E-47	postive
TRAV8-3	AC243960.1	0.555487927	1.22E-44	postive
TRAV8-6	AC243960.1	0.530532928	3.59E-40	postive
TRAV9-2	AC243960.1	0.560150069	1.61E-45	postive
TRAV16	AC243960.1	0.551161431	7.72E-44	postive
TRAV17	AC243960.1	0.513273687	2.72E-37	postive
TRAV29DV5	AC243960.1	0.500039012	3.43E-35	postive
TRBC1	AC243960.1	0.662051126	9.06E-69	postive
TRBC2	AC243960.1	0.587234683	6.66E-51	postive
TRBJ2-7	AC243960.1	0.5542382	2.08E-44	postive
TRBV2	AC243960.1	0.559134471	2.51E-45	postive
TRBV3-1	AC243960.1	0.506546775	3.26E-36	postive
TRBV5-1	AC243960.1	0.609634735	9.37E-56	postive
TRBV5-4	AC243960.1	0.527725798	1.08E-39	postive
TRBV6-1	AC243960.1	0.522260035	9.04E-39	postive
TRBV6-5	AC243960.1	0.546991244	4.46E-43	postive
TRBV7-9	AC243960.1	0.527229382	1.32E-39	postive
TRBV18	AC243960.1	0.545239041	9.26E-43	postive
TRBV19	AC243960.1	0.504522172	6.82E-36	postive
TRBV20-1	AC243960.1	0.570970463	1.31E-47	postive
TRBV28	AC243960.1	0.631984488	5.35E-61	postive
TYK2	LINC01786	0.507243441	2.53E-36	postive
SYTL1	LINC01786	0.516210777	9.05E-38	postive
IRF9	LINC01786	0.568501908	3.99E-47	postive
TNFRSF14	LINC01786	0.504005954	8.22E-36	postive
CREB1	AC080013.4	0.513777344	2.26E-37	postive
IL15	AC080013.4	0.523925921	4.75E-39	postive
FGF2	AC080013.4	0.591565068	8.23E-52	postive
CCL26	AC080013.4	0.512943218	3.08E-37	postive
JAK2	AC080013.4	0.525829677	2.27E-39	postive
NFAT5	AC080013.4	0.567869125	5.30E-47	postive
PIK3CA	AC080013.4	0.532331631	1.76E-40	postive
ANGPTL1	AC080013.4	0.575972561	1.33E-48	postive
RORA	AC080013.4	0.563089824	4.44E-46	postive
BRAF	AC080013.4	0.50194517	1.73E-35	postive
CBLB	AC080013.4	0.519849219	2.28E-38	postive

TRAJ1	AC080013.4	0.516841972	7.13E-38	postive
TRAJ2	AC080013.4	0.500405237	3.01E-35	postive
TRAJ3	AC080013.4	0.526236785	1.94E-39	postive
TRAJ5	AC080013.4	0.546876882	4.68E-43	postive
TRAJ6	AC080013.4	0.5404163	6.75E-42	postive
TRAJ8	AC080013.4	0.504315215	7.35E-36	postive
TRAJ10	AC080013.4	0.522471287	8.33E-39	postive
TRAJ13	AC080013.4	0.524257197	4.18E-39	postive
TRAJ14	AC080013.4	0.50437445	7.20E-36	postive
TRAJ16	AC080013.4	0.535440572	5.07E-41	postive
TRAJ17	AC080013.4	0.500382315	3.03E-35	postive
TRAJ21	AC080013.4	0.531588349	2.36E-40	postive
TRAJ31	AC080013.4	0.577753921	5.83E-49	postive
TRAJ37	AC080013.4	0.53104172	2.93E-40	postive
TRAJ38	AC080013.4	0.572971509	5.27E-48	postive
TRAJ39	AC080013.4	0.555137926	1.41E-44	postive
CREB1	AP001458.1	0.673359207	6.24E-72	postive
RFXAP	AP001458.1	0.508729179	1.47E-36	postive
UBR1	AP001458.1	0.520831202	1.56E-38	postive
ZC3HAV1	AP001458.1	0.505195699	5.34E-36	postive
IL15	AP001458.1	0.548822456	2.07E-43	postive
CYLD	AP001458.1	0.542561879	2.80E-42	postive
NFKBIZ	AP001458.1	0.506492606	3.33E-36	postive
DDX17	AP001458.1	0.628750962	3.26E-60	postive
JAK2	AP001458.1	0.575625501	1.56E-48	postive
TXK	AP001458.1	0.543130592	2.22E-42	postive
NFAT5	AP001458.1	0.710725026	1.85E-83	postive
PIK3R1	AP001458.1	0.537168707	2.53E-41	postive
PIK3CA	AP001458.1	0.564415479	2.47E-46	postive
RASGRP3	AP001458.1	0.5148901	1.49E-37	postive
LTB4R2	AP001458.1	0.529244566	5.97E-40	postive
GNRH1	AP001458.1	0.783083329	5.05E-112	postive
IL6ST	AP001458.1	0.527707926	1.09E-39	postive
RABEP1	AP001458.1	0.514878982	1.49E-37	postive
ACVR2A	AP001458.1	0.556040414	9.60E-45	postive
ANGPTL1	AP001458.1	0.682843208	1.08E-74	postive
BMPR2	AP001458.1	0.606846343	3.95E-55	postive
CRLF3	AP001458.1	0.600457542	1.01E-53	postive
NR2C1	AP001458.1	0.502086735	1.64E-35	postive
NR2C2	AP001458.1	0.716630272	1.89E-85	postive
RORA	AP001458.1	0.71669561	1.79E-85	postive
SOS1	AP001458.1	0.594373856	2.08E-52	postive
SOS2	AP001458.1	0.511360707	5.55E-37	postive
BRAF	AP001458.1	0.719218936	2.44E-86	postive
ITK	AP001458.1	0.503806681	8.84E-36	postive
CBL	AP001458.1	0.568604957	3.81E-47	postive
CBLB	AP001458.1	0.640748589	3.57E-63	postive
PDK1	AP001458.1	0.524199414	4.28E-39	postive
TRAJ1	AP001458.1	0.646021556	1.62E-64	postive
TRAJ2	AP001458.1	0.58128902	1.12E-49	postive
TRAJ3	AP001458.1	0.678968422	1.49E-73	postive
TRAJ5	AP001458.1	0.693574695	6.00E-78	postive
TRAJ6	AP001458.1	0.66093004	1.83E-68	postive
TRAJ8	AP001458.1	0.648826406	3.05E-65	postive
TRAJ10	AP001458.1	0.642624779	1.20E-63	postive
TRAJ12	AP001458.1	0.567624661	5.91E-47	postive
TRAJ13	AP001458.1	0.66356213	3.49E-69	postive
TRAJ14	AP001458.1	0.632337837	4.39E-61	postive
TRAJ16	AP001458.1	0.655117226	6.73E-67	postive
TRAJ17	AP001458.1	0.638499373	1.31E-62	postive
TRAJ18	AP001458.1	0.579015027	3.24E-49	postive
TRAJ21	AP001458.1	0.671508392	2.10E-71	postive
TRAJ31	AP001458.1	0.694787983	2.52E-78	postive
TRAJ37	AP001458.1	0.664718653	1.67E-69	postive
TRAJ38	AP001458.1	0.705737422	8.15E-82	postive

TRAJ39	AP001458.1	0.698237466	2.08E-79	postive
LMBR1L	AC011472.1	0.52478135	3.41E-39	postive
TYK2	AC011472.1	0.553827405	2.48E-44	postive
IRF9	AC011472.1	0.537689124	2.05E-41	postive
GNRH1	AC011472.1	0.529499719	5.40E-40	postive
EPOR	AC011472.1	0.616185755	3.01E-57	postive
GIPR	AC011472.1	0.515202998	1.32E-37	postive
ZC3HAV1L	AC093484.4	0.519793391	2.33E-38	postive
MAPK8	AC093484.4	0.507655388	2.18E-36	postive
DDX17	AC093484.4	0.519245922	2.87E-38	postive
GNRH1	AC093484.4	0.61424173	8.42E-57	postive
CRLF3	AC093484.4	0.511977166	4.42E-37	postive
NR2C1	AC093484.4	0.500306705	3.11E-35	postive
NR2C2	AC093484.4	0.530780309	3.25E-40	postive
BRAF	AC093484.4	0.548505459	2.37E-43	postive
TRAJ38	AC093484.4	0.506352615	3.50E-36	postive
CREB1	AC005021.1	0.691039293	3.63E-77	postive
UBR1	AC005021.1	0.603820716	1.85E-54	postive
ZC3HAV1	AC005021.1	0.521296322	1.31E-38	postive
ZC3HAV1L	AC005021.1	0.521078664	1.42E-38	postive
IL15	AC005021.1	0.595833979	1.01E-52	postive
CYLD	AC005021.1	0.550246658	1.14E-43	postive
EIF2AK2	AC005021.1	0.509261252	1.21E-36	postive
NFKBIZ	AC005021.1	0.511456303	5.36E-37	postive
LMBR1	AC005021.1	0.518615658	3.64E-38	postive
IREB2	AC005021.1	0.544166585	1.44E-42	postive
DDX17	AC005021.1	0.576307133	1.14E-48	postive
JAK2	AC005021.1	0.606849971	3.95E-55	postive
NFAT5	AC005021.1	0.72928789	6.76E-90	postive
PIK3R1	AC005021.1	0.565078665	1.84E-46	postive
PIK3CA	AC005021.1	0.574062033	3.20E-48	postive
GNRH1	AC005021.1	0.632391757	4.26E-61	postive
IL6ST	AC005021.1	0.519675846	2.43E-38	postive
ACVR2A	AC005021.1	0.517742938	5.07E-38	postive
ANGPTL1	AC005021.1	0.681339946	3.01E-74	postive
BMPR2	AC005021.1	0.5886853	3.32E-51	postive
CRLF3	AC005021.1	0.573996558	3.30E-48	postive
LIFR	AC005021.1	0.542043926	3.47E-42	postive
NR2C2	AC005021.1	0.684263644	4.08E-75	postive
RORA	AC005021.1	0.666083401	7.01E-70	postive
SOS1	AC005021.1	0.627518268	6.47E-60	postive
SOS2	AC005021.1	0.531851118	2.13E-40	postive
BRAF	AC005021.1	0.770307314	3.18E-106	postive
CBL	AC005021.1	0.569496325	2.55E-47	postive
CBLB	AC005021.1	0.710776713	1.78E-83	postive
RASGRP1	AC005021.1	0.528724682	7.32E-40	postive
PDK1	AC005021.1	0.501419021	2.09E-35	postive
TRAJ1	AC005021.1	0.694349872	3.45E-78	postive
TRAJ2	AC005021.1	0.739893168	8.05E-94	postive
TRAJ3	AC005021.1	0.780454839	8.47E-111	postive
TRAJ5	AC005021.1	0.687988483	3.09E-76	postive
TRAJ6	AC005021.1	0.755208359	7.72E-100	postive
TRAJ8	AC005021.1	0.686623163	8.00E-76	postive
TRAJ10	AC005021.1	0.726771286	5.42E-89	postive
TRAJ12	AC005021.1	0.751866635	1.73E-98	postive
TRAJ13	AC005021.1	0.69044222	5.53E-77	postive
TRAJ14	AC005021.1	0.760840402	3.64E-102	postive
TRAJ16	AC005021.1	0.697992957	2.49E-79	postive
TRAJ17	AC005021.1	0.664129869	2.43E-69	postive
TRAJ18	AC005021.1	0.730888161	1.78E-90	postive
TRAJ21	AC005021.1	0.741675018	1.69E-94	postive
TRAJ31	AC005021.1	0.728007244	1.95E-89	postive
TRAJ37	AC005021.1	0.717310305	1.10E-85	postive
TRAJ38	AC005021.1	0.811644294	1.54E-126	postive
TRAJ39	AC005021.1	0.720605352	8.06E-87	postive

RFXAP	AC012360.3	0.505784535	4.31E-36	postive
GNRH1	AC012360.3	0.530848722	3.17E-40	postive
NR2C1	AC012360.3	0.510968611	6.42E-37	postive
NR2C2	AC012360.3	0.528875766	6.90E-40	postive
FGFR1	AC087623.1	0.839087771	4.86E-143	postive
IRF9	AL031186.1	0.528308872	8.62E-40	postive
KCNH2	AC106897.2	0.557329932	5.50E-45	postive
ZC3HAV1L	AC005586.1	0.517441691	5.68E-38	postive
CHGA	AC005586.1	0.56217654	6.64E-46	postive
GRP	AC005586.1	0.562252482	6.42E-46	postive
TYK2	AP006621.4	0.502254334	1.55E-35	postive
IRF9	AP006621.4	0.50596136	4.04E-36	postive
LTB4R2	AP006621.4	0.503854797	8.69E-36	postive
CREB1	CREB3L2-AS1	0.765116015	5.64E-104	postive
RFXAP	CREB3L2-AS1	0.505810833	4.27E-36	postive
UBR1	CREB3L2-AS1	0.650617941	1.04E-65	postive
ZC3HAV1	CREB3L2-AS1	0.565803329	1.33E-46	postive
ZC3HAV1L	CREB3L2-AS1	0.554046573	2.26E-44	postive
IL15	CREB3L2-AS1	0.612485742	2.12E-56	postive
CYLD	CREB3L2-AS1	0.549612375	1.48E-43	postive
EIF2AK2	CREB3L2-AS1	0.559996342	1.73E-45	postive
MAPK8	CREB3L2-AS1	0.573256941	4.62E-48	postive
LMBR1	CREB3L2-AS1	0.600713556	8.92E-54	postive
LIMS1	CREB3L2-AS1	0.513150501	2.85E-37	postive
IREB2	CREB3L2-AS1	0.638772088	1.12E-62	postive
DDX17	CREB3L2-AS1	0.56829929	4.37E-47	postive
PIK3CG	CREB3L2-AS1	0.54849544	2.38E-43	postive
JAK2	CREB3L2-AS1	0.612571887	2.03E-56	postive
TXK	CREB3L2-AS1	0.607941537	2.25E-55	postive
NFAT5	CREB3L2-AS1	0.753418377	4.11E-99	postive
MALT1	CREB3L2-AS1	0.536490101	3.32E-41	postive
PIK3R1	CREB3L2-AS1	0.672645948	9.98E-72	postive
PIK3CA	CREB3L2-AS1	0.670924903	3.08E-71	postive
PIK3CB	CREB3L2-AS1	0.532312135	1.77E-40	postive
AKT3	CREB3L2-AS1	0.56549714	1.53E-46	postive
RASGRP3	CREB3L2-AS1	0.533078209	1.31E-40	postive
IGHD4-4	CREB3L2-AS1	0.561304652	9.73E-46	postive
IGHD6-6	CREB3L2-AS1	0.521735441	1.11E-38	postive
PLXNC1	CREB3L2-AS1	0.50166452	1.91E-35	postive
GNRH1	CREB3L2-AS1	0.696514	7.27E-79	postive
IL6ST	CREB3L2-AS1	0.644060417	5.15E-64	postive
RABEP1	CREB3L2-AS1	0.554097493	2.21E-44	postive
ACVR2A	CREB3L2-AS1	0.614708771	6.58E-57	postive
ANGPTL1	CREB3L2-AS1	0.800067612	2.26E-120	postive
BMPR1A	CREB3L2-AS1	0.553046471	3.46E-44	postive
BMPR2	CREB3L2-AS1	0.733746333	1.60E-91	postive
CRLF3	CREB3L2-AS1	0.639803974	6.18E-63	postive
LIFR	CREB3L2-AS1	0.512779952	3.27E-37	postive
NR1D2	CREB3L2-AS1	0.503911493	8.51E-36	postive
NR2C2	CREB3L2-AS1	0.670183096	4.98E-71	postive
NR3C1	CREB3L2-AS1	0.501020306	2.41E-35	postive
RORA	CREB3L2-AS1	0.804977655	6.16E-123	postive
SOS1	CREB3L2-AS1	0.675075302	2.01E-72	postive
SOS2	CREB3L2-AS1	0.572948882	5.32E-48	postive
BRAF	CREB3L2-AS1	0.847022389	2.13E-148	postive
ITK	CREB3L2-AS1	0.529148852	6.20E-40	postive
TEC	CREB3L2-AS1	0.50662302	3.18E-36	postive
CBL	CREB3L2-AS1	0.593958667	2.55E-52	postive
CBLB	CREB3L2-AS1	0.748181547	5.05E-97	postive
RASGRP1	CREB3L2-AS1	0.521852953	1.06E-38	postive
PDK1	CREB3L2-AS1	0.623235688	6.79E-59	postive
TRAJ1	CREB3L2-AS1	0.647818121	5.57E-65	postive
TRAJ2	CREB3L2-AS1	0.708899529	7.47E-83	postive
TRAJ3	CREB3L2-AS1	0.752623565	8.59E-99	postive
TRAJ5	CREB3L2-AS1	0.739089724	1.62E-93	postive

TRAJ6	CREB3L2-AS1	0.715002624	6.76E-85	postive
TRAJ8	CREB3L2-AS1	0.716735702	1.74E-85	postive
TRAJ10	CREB3L2-AS1	0.678214761	2.48E-73	postive
TRAJ12	CREB3L2-AS1	0.652192329	4.01E-66	postive
TRAJ13	CREB3L2-AS1	0.718079575	6.01E-86	postive
TRAJ14	CREB3L2-AS1	0.726137076	9.12E-89	postive
TRAJ16	CREB3L2-AS1	0.686179738	1.09E-75	postive
TRAJ17	CREB3L2-AS1	0.670515103	4.02E-71	postive
TRAJ18	CREB3L2-AS1	0.644099804	5.04E-64	postive
TRAJ21	CREB3L2-AS1	0.753686424	3.20E-99	postive
TRAJ31	CREB3L2-AS1	0.765276939	4.82E-104	postive
TRAJ37	CREB3L2-AS1	0.73453851	8.14E-92	postive
TRAJ38	CREB3L2-AS1	0.810410005	7.32E-126	postive
TRAJ39	CREB3L2-AS1	0.778519431	6.59E-110	postive
PDGFRA	BX322234.1	0.555842905	1.04E-44	postive
FGF7	BX322234.1	0.518201688	4.26E-38	postive
INHBA	BX322234.1	0.611666662	3.25E-56	postive
CREB1	MIATNB	0.651720378	5.33E-66	postive
UBR1	MIATNB	0.587419832	6.10E-51	postive
IL15	MIATNB	0.561479158	9.02E-46	postive
CYLD	MIATNB	0.627376494	6.99E-60	postive
IREB2	MIATNB	0.515045373	1.40E-37	postive
DDX17	MIATNB	0.629629888	2.00E-60	postive
PIK3CG	MIATNB	0.587810068	5.06E-51	postive
JAK2	MIATNB	0.650616659	1.04E-65	postive
TXK	MIATNB	0.606241735	5.39E-55	postive
NFAT5	MIATNB	0.705827014	7.62E-82	postive
MALT1	MIATNB	0.510649438	7.23E-37	postive
PIK3R1	MIATNB	0.608349784	1.82E-55	postive
PIK3CA	MIATNB	0.587969828	4.68E-51	postive
RASGRP3	MIATNB	0.580678302	1.49E-49	postive
IGHD6-6	MIATNB	0.544334935	1.35E-42	postive
PLXNC1	MIATNB	0.543722672	1.73E-42	postive
GNRH1	MIATNB	0.706969566	3.23E-82	postive
IL6ST	MIATNB	0.543573904	1.84E-42	postive
IL7	MIATNB	0.519041806	3.10E-38	postive
ACVR2A	MIATNB	0.583909192	3.25E-50	postive
ANGPTL1	MIATNB	0.662005022	9.32E-69	postive
BMPR2	MIATNB	0.626856153	9.32E-60	postive
CRLF3	MIATNB	0.542708565	2.64E-42	postive
NR2C2	MIATNB	0.658808439	6.89E-68	postive
RORA	MIATNB	0.751347842	2.79E-98	postive
SOS1	MIATNB	0.548676278	2.20E-43	postive
BRAF	MIATNB	0.651198411	7.32E-66	postive
PTPRC	MIATNB	0.500373958	3.04E-35	postive
ITK	MIATNB	0.586101291	1.15E-50	postive
CD28	MIATNB	0.518894678	3.28E-38	postive
CBLB	MIATNB	0.641547197	2.24E-63	postive
RASGRP1	MIATNB	0.529195792	6.08E-40	postive
PDK1	MIATNB	0.5512671	7.38E-44	postive
TRAJ1	MIATNB	0.665942354	7.67E-70	postive
TRAJ2	MIATNB	0.619305503	5.69E-58	postive
TRAJ3	MIATNB	0.693837328	4.98E-78	postive
TRAJ5	MIATNB	0.685859729	1.36E-75	postive
TRAJ6	MIATNB	0.674410123	3.12E-72	postive
TRAJ8	MIATNB	0.626363042	1.22E-59	postive
TRAJ10	MIATNB	0.663259966	4.22E-69	postive
TRAJ12	MIATNB	0.653253244	2.10E-66	postive
TRAJ13	MIATNB	0.634964571	9.92E-62	postive
TRAJ14	MIATNB	0.647112854	8.47E-65	postive
TRAJ16	MIATNB	0.684197128	4.27E-75	postive
TRAJ17	MIATNB	0.658753706	7.13E-68	postive
TRAJ18	MIATNB	0.677092613	5.25E-73	postive
TRAJ21	MIATNB	0.679189136	1.29E-73	postive
TRAJ31	MIATNB	0.700158189	5.12E-80	postive

TRAJ37	MIATNB	0.662683775	6.08E-69	postive
TRAJ38	MIATNB	0.738225748	3.43E-93	postive
TRAJ39	MIATNB	0.702175195	1.16E-80	postive
CREB1	AL731566.2	0.708382179	1.11E-82	postive
RFXAP	AL731566.2	0.576045189	1.29E-48	postive
UBR1	AL731566.2	0.685859953	1.36E-75	postive
ZC3HAV1	AL731566.2	0.519787011	2.33E-38	postive
ZC3HAV1L	AL731566.2	0.510816106	6.79E-37	postive
CYLD	AL731566.2	0.535196359	5.60E-41	postive
EIF2AK2	AL731566.2	0.511042473	6.25E-37	postive
MAPK8	AL731566.2	0.619039278	6.56E-58	postive
IREB2	AL731566.2	0.630538608	1.20E-60	postive
DDX17	AL731566.2	0.720333165	1.00E-86	postive
JAK2	AL731566.2	0.541011402	5.29E-42	postive
TXK	AL731566.2	0.613600202	1.18E-56	postive
NFAT5	AL731566.2	0.788336101	1.60E-114	postive
NFATC3	AL731566.2	0.501983946	1.71E-35	postive
MALT1	AL731566.2	0.518816018	3.37E-38	postive
PIK3R1	AL731566.2	0.547869927	3.09E-43	postive
PIK3CA	AL731566.2	0.56614298	1.15E-46	postive
IGHD6-6	AL731566.2	0.562240865	6.45E-46	postive
GNRH1	AL731566.2	0.82165421	3.16E-132	postive
IL6ST	AL731566.2	0.555602942	1.16E-44	postive
RABEP1	AL731566.2	0.576895271	8.68E-49	postive
ACVR2A	AL731566.2	0.638018896	1.73E-62	postive
ANGPTL1	AL731566.2	0.63369287	2.04E-61	postive
BMPR1A	AL731566.2	0.607302573	3.13E-55	postive
BMPR2	AL731566.2	0.647362094	7.30E-65	postive
CRLF3	AL731566.2	0.593578878	3.08E-52	postive
NR2C1	AL731566.2	0.638599982	1.24E-62	postive
NR2C2	AL731566.2	0.744851092	1.01E-95	postive
RORA	AL731566.2	0.778111144	1.01E-109	postive
SOS1	AL731566.2	0.621117158	2.14E-58	postive
SOS2	AL731566.2	0.509224382	1.22E-36	postive
BRAF	AL731566.2	0.723904022	5.64E-88	postive
TEC	AL731566.2	0.564249487	2.66E-46	postive
CBL	AL731566.2	0.520234805	1.97E-38	postive
CBLB	AL731566.2	0.609827266	8.48E-56	postive
PDK1	AL731566.2	0.590828189	1.18E-51	postive
TRAJ1	AL731566.2	0.636220969	4.85E-62	postive
TRAJ2	AL731566.2	0.643344975	7.85E-64	postive
TRAJ3	AL731566.2	0.6861985	1.07E-75	postive
TRAJ5	AL731566.2	0.64911399	2.56E-65	postive
TRAJ6	AL731566.2	0.674139721	3.73E-72	postive
TRAJ8	AL731566.2	0.624566162	3.28E-59	postive
TRAJ10	AL731566.2	0.636551752	4.01E-62	postive
TRAJ12	AL731566.2	0.603399368	2.30E-54	postive
TRAJ13	AL731566.2	0.632682225	3.61E-61	postive
TRAJ14	AL731566.2	0.616577018	2.44E-57	postive
TRAJ16	AL731566.2	0.642300786	1.45E-63	postive
TRAJ17	AL731566.2	0.594898021	1.61E-52	postive
TRAJ18	AL731566.2	0.615611568	4.08E-57	postive
TRAJ21	AL731566.2	0.66762629	2.60E-70	postive
TRAJ31	AL731566.2	0.669588976	7.33E-71	postive
TRAJ37	AL731566.2	0.685368941	1.90E-75	postive
TRAJ38	AL731566.2	0.708409897	1.08E-82	postive
TRAJ39	AL731566.2	0.699037667	1.16E-79	postive
CREB1	AC016590.2	0.72076567	7.09E-87	postive
RFXAP	AC016590.2	0.531821542	2.15E-40	postive
UBR1	AC016590.2	0.668332658	1.65E-70	postive
ZC3HAV1	AC016590.2	0.55689244	6.64E-45	postive
ZC3HAV1L	AC016590.2	0.549250005	1.73E-43	postive
IL15	AC016590.2	0.523212415	6.26E-39	postive
CYLD	AC016590.2	0.519624018	2.48E-38	postive
EIF2AK2	AC016590.2	0.5392224	1.10E-41	postive

MAPK8	AC016590.2	0.538984439	1.21E-41	postive
LMBR1	AC016590.2	0.531484442	2.46E-40	postive
IREB2	AC016590.2	0.610267605	6.75E-56	postive
DDX17	AC016590.2	0.626632696	1.05E-59	postive
JAK2	AC016590.2	0.567127075	7.38E-47	postive
TXK	AC016590.2	0.641705596	2.05E-63	postive
NFAT5	AC016590.2	0.764399774	1.14E-103	postive
MALT1	AC016590.2	0.520214585	1.98E-38	postive
PIK3R1	AC016590.2	0.604497299	1.31E-54	postive
PIK3CA	AC016590.2	0.63740919	2.46E-62	postive
PIK3CB	AC016590.2	0.502064395	1.66E-35	postive
IGHD4-4	AC016590.2	0.581484515	1.02E-49	postive
IGHD6-6	AC016590.2	0.61978903	4.39E-58	postive
GNRH1	AC016590.2	0.791343634	5.50E-116	postive
IL6ST	AC016590.2	0.574986146	2.09E-48	postive
RABEP1	AC016590.2	0.568248142	4.47E-47	postive
ACVR2A	AC016590.2	0.605463041	8.03E-55	postive
ANGPTL1	AC016590.2	0.685405039	1.86E-75	postive
BMPR1A	AC016590.2	0.532560527	1.61E-40	postive
BMPR2	AC016590.2	0.681395918	2.89E-74	postive
CRLF3	AC016590.2	0.59684209	6.16E-53	postive
NR2C2	AC016590.2	0.727902191	2.13E-89	postive
RORA	AC016590.2	0.757143622	1.25E-100	postive
SOS1	AC016590.2	0.62898733	2.86E-60	postive
SOS2	AC016590.2	0.540932861	5.47E-42	postive
BRAF	AC016590.2	0.755222741	7.62E-100	postive
TEC	AC016590.2	0.63799086	1.76E-62	postive
CBL	AC016590.2	0.572991864	5.22E-48	postive
CBLB	AC016590.2	0.639179616	8.86E-63	postive
RASGRP1	AC016590.2	0.50443682	7.04E-36	postive
PDK1	AC016590.2	0.592284489	5.80E-52	postive
TRAJ1	AC016590.2	0.621625075	1.63E-58	postive
TRAJ2	AC016590.2	0.611157512	4.24E-56	postive
TRAJ3	AC016590.2	0.713006441	3.19E-84	postive
TRAJ5	AC016590.2	0.643445563	7.40E-64	postive
TRAJ6	AC016590.2	0.670853286	3.22E-71	postive
TRAJ8	AC016590.2	0.622301657	1.13E-58	postive
TRAJ10	AC016590.2	0.642324186	1.43E-63	postive
TRAJ12	AC016590.2	0.583193919	4.56E-50	postive
TRAJ13	AC016590.2	0.607716106	2.53E-55	postive
TRAJ14	AC016590.2	0.608934605	1.35E-55	postive
TRAJ16	AC016590.2	0.636449853	4.25E-62	postive
TRAJ17	AC016590.2	0.614896978	5.96E-57	postive
TRAJ18	AC016590.2	0.612669959	1.92E-56	postive
TRAJ21	AC016590.2	0.666936351	4.06E-70	postive
TRAJ31	AC016590.2	0.66465299	1.75E-69	postive
TRAJ37	AC016590.2	0.669516002	7.68E-71	postive
TRAJ38	AC016590.2	0.703936087	3.14E-81	postive
TRAJ39	AC016590.2	0.723119374	1.07E-87	postive
CD19	LINC01480	0.537848061	1.92E-41	postive
IGKJ5	LINC01480	0.530032017	4.37E-40	postive
IL16	LINC01480	0.510169001	8.63E-37	postive
ZAP70	LINC01480	0.510451699	7.77E-37	postive
CREB1	AGAP1-IT1	0.598642853	2.51E-53	postive
UBR1	AGAP1-IT1	0.587454258	6.00E-51	postive
MAPK8	AGAP1-IT1	0.520228901	1.97E-38	postive
IREB2	AGAP1-IT1	0.525764064	2.33E-39	postive
DDX17	AGAP1-IT1	0.527549238	1.16E-39	postive
TXK	AGAP1-IT1	0.596870581	6.07E-53	postive
NFAT5	AGAP1-IT1	0.64171667	2.03E-63	postive
PIK3R1	AGAP1-IT1	0.520030469	2.12E-38	postive
PIK3CA	AGAP1-IT1	0.520119169	2.05E-38	postive
GNRH1	AGAP1-IT1	0.681241903	3.21E-74	postive
ACVR2A	AGAP1-IT1	0.508659529	1.51E-36	postive
ANGPTL1	AGAP1-IT1	0.609809286	8.56E-56	postive

BMPR2	AGAP1-IT1	0.600892471	8.15E-54	postive
NR2C2	AGAP1-IT1	0.598971492	2.13E-53	postive
RORA	AGAP1-IT1	0.661289204	1.46E-68	postive
SOS1	AGAP1-IT1	0.515352076	1.25E-37	postive
BRAF	AGAP1-IT1	0.665198804	1.23E-69	postive
TEC	AGAP1-IT1	0.568898823	3.33E-47	postive
CBLB	AGAP1-IT1	0.601502255	6.00E-54	postive
PDK1	AGAP1-IT1	0.520032555	2.12E-38	postive
TRAJ1	AGAP1-IT1	0.520248955	1.95E-38	postive
TRAJ2	AGAP1-IT1	0.517530097	5.50E-38	postive
TRAJ3	AGAP1-IT1	0.598334754	2.93E-53	postive
TRAJ5	AGAP1-IT1	0.554534136	1.83E-44	postive
TRAJ6	AGAP1-IT1	0.570110086	1.93E-47	postive
TRAJ8	AGAP1-IT1	0.516778426	7.30E-38	postive
TRAJ10	AGAP1-IT1	0.528564087	7.80E-40	postive
TRAJ12	AGAP1-IT1	0.554578292	1.80E-44	postive
TRAJ13	AGAP1-IT1	0.514938235	1.46E-37	postive
TRAJ14	AGAP1-IT1	0.537161688	2.53E-41	postive
TRAJ16	AGAP1-IT1	0.529590985	5.21E-40	postive
TRAJ17	AGAP1-IT1	0.509234243	1.22E-36	postive
TRAJ18	AGAP1-IT1	0.547581483	3.49E-43	postive
TRAJ21	AGAP1-IT1	0.579217143	2.95E-49	postive
TRAJ31	AGAP1-IT1	0.580948093	1.31E-49	postive
TRAJ37	AGAP1-IT1	0.573538545	4.06E-48	postive
TRAJ38	AGAP1-IT1	0.624816895	2.86E-59	postive
TRAJ39	AGAP1-IT1	0.61627955	2.86E-57	postive
NCK1	NCK1-DT	0.650198875	1.34E-65	postive
CREB1	OIP5-AS1	0.658354782	9.14E-68	postive
UBR1	OIP5-AS1	0.763204989	3.67E-103	postive
ZC3HAV1	OIP5-AS1	0.556641796	7.40E-45	postive
MAPK8	OIP5-AS1	0.502734087	1.30E-35	postive
SP1	OIP5-AS1	0.555287411	1.33E-44	postive
IREB2	OIP5-AS1	0.739975347	7.49E-94	postive
DDX17	OIP5-AS1	0.555802364	1.06E-44	postive
NFAT5	OIP5-AS1	0.640226015	4.84E-63	postive
NFATC3	OIP5-AS1	0.503858282	8.68E-36	postive
PIK3R1	OIP5-AS1	0.580242204	1.83E-49	postive
PIK3CA	OIP5-AS1	0.572609222	6.21E-48	postive
GNRH1	OIP5-AS1	0.5566592	7.35E-45	postive
IL6ST	OIP5-AS1	0.597420404	4.62E-53	postive
RABEP1	OIP5-AS1	0.518504001	3.80E-38	postive
ACVR2A	OIP5-AS1	0.585180517	1.78E-50	postive
ANGPTL1	OIP5-AS1	0.556909493	6.60E-45	postive
BMPR1A	OIP5-AS1	0.611437833	3.67E-56	postive
BMPR2	OIP5-AS1	0.651804742	5.07E-66	postive
NR2C2	OIP5-AS1	0.626275549	1.28E-59	postive
NR3C1	OIP5-AS1	0.57513429	1.96E-48	postive
PPARA	OIP5-AS1	0.50742531	2.37E-36	postive
RORA	OIP5-AS1	0.67979823	8.54E-74	postive
SOS1	OIP5-AS1	0.620541632	2.92E-58	postive
BRAF	OIP5-AS1	0.619882251	4.17E-58	postive
CBLB	OIP5-AS1	0.530405353	3.77E-40	postive
TRAJ1	OIP5-AS1	0.510164053	8.65E-37	postive
TRAJ2	OIP5-AS1	0.531560435	2.39E-40	postive
TRAJ3	OIP5-AS1	0.548415023	2.46E-43	postive
TRAJ5	OIP5-AS1	0.561007487	1.11E-45	postive
TRAJ6	OIP5-AS1	0.546446438	5.60E-43	postive
TRAJ8	OIP5-AS1	0.534789477	6.59E-41	postive
TRAJ10	OIP5-AS1	0.51936277	2.74E-38	postive
TRAJ12	OIP5-AS1	0.505074711	5.58E-36	postive
TRAJ13	OIP5-AS1	0.541761664	3.89E-42	postive
TRAJ14	OIP5-AS1	0.525326455	2.76E-39	postive
TRAJ16	OIP5-AS1	0.520487904	1.78E-38	postive
TRAJ17	OIP5-AS1	0.507905227	1.99E-36	postive
TRAJ18	OIP5-AS1	0.502249489	1.55E-35	postive

TRAJ21	OIP5-AS1	0.558308446	3.60E-45	postive
TRAJ31	OIP5-AS1	0.583898185	3.27E-50	postive
TRAJ37	OIP5-AS1	0.568078131	4.82E-47	postive
TRAJ38	OIP5-AS1	0.597654792	4.11E-53	postive
TRAJ39	OIP5-AS1	0.587325173	6.38E-51	postive
CIITA	AC108134.3	0.529649925	5.09E-40	postive
IFI30	AC108134.3	0.578870083	3.47E-49	postive
SLC11A1	AC108134.3	0.555390483	1.27E-44	postive
FGR	AC108134.3	0.641034823	3.02E-63	postive
BTK	AC108134.3	0.54394927	1.58E-42	postive
PIK3R5	AC108134.3	0.596092663	8.93E-53	postive
LILRB3	AC108134.3	0.556387484	8.26E-45	postive
IL10RA	AC108134.3	0.513533731	2.47E-37	postive
IL3RA	AC108134.3	0.508090276	1.86E-36	postive
ITGAL	AC108134.3	0.540155823	7.51E-42	postive
ZAP70	AC108134.3	0.555421548	1.25E-44	postive
CREB1	AC012181.2	0.596487157	7.34E-53	postive
UBR1	AC012181.2	0.515518505	1.17E-37	postive
CYLD	AC012181.2	0.609796525	8.62E-56	postive
DDX17	AC012181.2	0.620173231	3.57E-58	postive
PIK3CG	AC012181.2	0.62807189	4.76E-60	postive
JAK2	AC012181.2	0.598034441	3.41E-53	postive
TXK	AC012181.2	0.570795287	1.42E-47	postive
PPP3CC	AC012181.2	0.502527725	1.40E-35	postive
NFAT5	AC012181.2	0.579971304	2.08E-49	postive
PIK3R1	AC012181.2	0.596763134	6.40E-53	postive
PIK3CA	AC012181.2	0.525367649	2.72E-39	postive
RASGRP3	AC012181.2	0.588287285	4.02E-51	postive
IGHD6-6	AC012181.2	0.535744014	4.49E-41	postive
IGKJ5	AC012181.2	0.56897356	3.22E-47	postive
PLXNC1	AC012181.2	0.538396209	1.54E-41	postive
GNRH1	AC012181.2	0.613767515	1.08E-56	postive
IL6ST	AC012181.2	0.560948334	1.14E-45	postive
ANGPTL1	AC012181.2	0.608293087	1.88E-55	postive
BMPR2	AC012181.2	0.542904941	2.43E-42	postive
CRLF3	AC012181.2	0.562056636	7.00E-46	postive
NR2C2	AC012181.2	0.627892279	5.26E-60	postive
RORA	AC012181.2	0.689449078	1.11E-76	postive
BRAF	AC012181.2	0.66371904	3.16E-69	postive
PTPRC	AC012181.2	0.548018092	2.90E-43	postive
ITK	AC012181.2	0.647544283	6.55E-65	postive
CD28	AC012181.2	0.514182181	1.94E-37	postive
CBL	AC012181.2	0.503081134	1.15E-35	postive
CBLB	AC012181.2	0.59589471	9.84E-53	postive
PDK1	AC012181.2	0.603609985	2.06E-54	postive
TRAJ1	AC012181.2	0.512019946	4.35E-37	postive
TRAJ2	AC012181.2	0.58145094	1.04E-49	postive
TRAJ3	AC012181.2	0.56558919	1.47E-46	postive
TRAJ5	AC012181.2	0.563580272	3.57E-46	postive
TRAJ6	AC012181.2	0.505807779	4.27E-36	postive
TRAJ13	AC012181.2	0.504236065	7.57E-36	postive
TRAJ14	AC012181.2	0.502912477	1.22E-35	postive
TRAJ16	AC012181.2	0.502547359	1.39E-35	postive
TRAJ21	AC012181.2	0.535398223	5.16E-41	postive
TRAJ31	AC012181.2	0.535018047	6.01E-41	postive
TRAJ37	AC012181.2	0.505092944	5.54E-36	postive
TRAJ38	AC012181.2	0.59478234	1.70E-52	postive
TRAJ39	AC012181.2	0.551489696	6.71E-44	postive
CREB1	AC110792.3	0.799189161	6.40E-120	postive
RFXAP	AC110792.3	0.504467039	6.96E-36	postive
UBR1	AC110792.3	0.651064864	7.93E-66	postive
ZC3HAV1	AC110792.3	0.541865621	3.73E-42	postive
IL15	AC110792.3	0.564974474	1.93E-46	postive
CYLD	AC110792.3	0.649846499	1.65E-65	postive
EIF2AK2	AC110792.3	0.537152482	2.54E-41	postive

MAPK8	AC110792.3	0.55950552	2.14E-45	postive
LMBR1	AC110792.3	0.544229028	1.41E-42	postive
LIMS1	AC110792.3	0.506489011	3.33E-36	postive
IREB2	AC110792.3	0.601261711	6.77E-54	postive
DDX17	AC110792.3	0.631923347	5.54E-61	postive
PIK3CG	AC110792.3	0.524451982	3.88E-39	postive
JAK2	AC110792.3	0.674109305	3.81E-72	postive
TXK	AC110792.3	0.604432515	1.36E-54	postive
NFAT5	AC110792.3	0.864556729	2.10E-161	postive
MALT1	AC110792.3	0.527591357	1.14E-39	postive
PIK3R1	AC110792.3	0.646773017	1.04E-64	postive
PIK3CA	AC110792.3	0.696952588	5.29E-79	postive
PIK3CB	AC110792.3	0.522904818	7.05E-39	postive
RASGRP3	AC110792.3	0.541746747	3.92E-42	postive
PLXNC1	AC110792.3	0.50046889	2.94E-35	postive
ROBO2	AC110792.3	0.547316287	3.89E-43	postive
GNRH1	AC110792.3	0.764372527	1.17E-103	postive
IL6ST	AC110792.3	0.641473788	2.34E-63	postive
RABEP1	AC110792.3	0.558425424	3.42E-45	postive
ACVR2A	AC110792.3	0.671153152	2.65E-71	postive
ANGPTL1	AC110792.3	0.759897428	9.02E-102	postive
BMPR1A	AC110792.3	0.573531529	4.08E-48	postive
BMPR2	AC110792.3	0.760476207	5.17E-102	postive
CRLF3	AC110792.3	0.624539346	3.33E-59	postive
NR1D2	AC110792.3	0.568723386	3.61E-47	postive
NR2C2	AC110792.3	0.704259827	2.46E-81	postive
RORA	AC110792.3	0.871953807	1.89E-167	postive
SOS1	AC110792.3	0.68101067	3.76E-74	postive
SOS2	AC110792.3	0.569298193	2.79E-47	postive
BRAF	AC110792.3	0.768076505	2.99E-105	postive
CBL	AC110792.3	0.565698826	1.40E-46	postive
CBLB	AC110792.3	0.750035594	9.32E-98	postive
PDK1	AC110792.3	0.562627172	5.44E-46	postive
TRAJ1	AC110792.3	0.718446766	4.50E-86	postive
TRAJ2	AC110792.3	0.713747066	1.80E-84	postive
TRAJ3	AC110792.3	0.744770987	1.09E-95	postive
TRAJ5	AC110792.3	0.776705925	4.42E-109	postive
TRAJ6	AC110792.3	0.7837969	2.33E-112	postive
TRAJ8	AC110792.3	0.717020521	1.39E-85	postive
TRAJ10	AC110792.3	0.774840091	3.08E-108	postive
TRAJ12	AC110792.3	0.735388605	3.94E-92	postive
TRAJ13	AC110792.3	0.763163153	3.83E-103	postive
TRAJ14	AC110792.3	0.742453259	8.50E-95	postive
TRAJ16	AC110792.3	0.784600657	9.74E-113	postive
TRAJ17	AC110792.3	0.727914229	2.11E-89	postive
TRAJ18	AC110792.3	0.744774311	1.08E-95	postive
TRAJ21	AC110792.3	0.786225391	1.65E-113	postive
TRAJ31	AC110792.3	0.83975079	1.78E-143	postive
TRAJ37	AC110792.3	0.800316724	1.68E-120	postive
TRAJ38	AC110792.3	0.866790565	3.43E-163	postive
TRAJ39	AC110792.3	0.788555317	1.25E-114	postive
CREB1	AL158166.1	0.567470034	6.33E-47	postive
NFAT5	AL158166.1	0.567921736	5.17E-47	postive
GNRH1	AL158166.1	0.540589605	6.29E-42	postive
ANGPTL1	AL158166.1	0.504337569	7.29E-36	postive
BMPR2	AL158166.1	0.551528651	6.60E-44	postive
NR2C2	AL158166.1	0.539794018	8.70E-42	postive
RORA	AL158166.1	0.554495638	1.86E-44	postive
SOS1	AL158166.1	0.501328844	2.16E-35	postive
BRAF	AL158166.1	0.587609272	5.57E-51	postive
TRAJ2	AL158166.1	0.502230767	1.56E-35	postive
TRAJ3	AL158166.1	0.575546611	1.62E-48	postive
TRAJ5	AL158166.1	0.564717926	2.16E-46	postive
TRAJ6	AL158166.1	0.541841238	3.77E-42	postive
TRAJ8	AL158166.1	0.535411277	5.13E-41	postive

TRAJ10	AL158166.1	0.53992175	8.26E-42	postive
TRAJ12	AL158166.1	0.506726671	3.06E-36	postive
TRAJ13	AL158166.1	0.536777183	2.96E-41	postive
TRAJ14	AL158166.1	0.516704167	7.51E-38	postive
TRAJ16	AL158166.1	0.512943357	3.08E-37	postive
TRAJ17	AL158166.1	0.533549308	1.08E-40	postive
TRAJ21	AL158166.1	0.555382292	1.27E-44	postive
TRAJ31	AL158166.1	0.583396807	4.15E-50	postive
TRAJ37	AL158166.1	0.570580893	1.56E-47	postive
TRAJ38	AL158166.1	0.584548519	2.40E-50	postive
TRAJ39	AL158166.1	0.571251064	1.15E-47	postive
PSMD3	LINC02014	0.504089301	7.98E-36	postive
CREB1	AC022150.4	0.770155849	3.70E-106	postive
RFXAP	AC022150.4	0.505881558	4.16E-36	postive
UBR1	AC022150.4	0.695233776	1.83E-78	postive
ZC3HAV1	AC022150.4	0.636590564	3.93E-62	postive
ZC3HAV1L	AC022150.4	0.619455483	5.25E-58	postive
IL15	AC022150.4	0.507866742	2.01E-36	postive
CYLD	AC022150.4	0.531868404	2.11E-40	postive
EIF2AK2	AC022150.4	0.58825762	4.08E-51	postive
MAPK8	AC022150.4	0.576774903	9.18E-49	postive
LMBR1	AC022150.4	0.605915615	6.37E-55	postive
LIMS1	AC022150.4	0.561729133	8.08E-46	postive
IREB2	AC022150.4	0.670041384	5.46E-71	postive
DDX17	AC022150.4	0.578365291	4.39E-49	postive
PIK3CG	AC022150.4	0.553336242	3.06E-44	postive
JAK2	AC022150.4	0.631067417	8.96E-61	postive
TXK	AC022150.4	0.657788502	1.30E-67	postive
NFAT5	AC022150.4	0.726509567	6.71E-89	postive
MALT1	AC022150.4	0.54059862	6.27E-42	postive
PIK3R1	AC022150.4	0.608398859	1.78E-55	postive
PIK3CA	AC022150.4	0.71762091	8.64E-86	postive
PIK3CB	AC022150.4	0.594403296	2.05E-52	postive
GSK3B	AC022150.4	0.510678428	7.15E-37	postive
RASGRP3	AC022150.4	0.505736948	4.39E-36	postive
IGHD4-4	AC022150.4	0.520874966	1.54E-38	postive
IGHD6-6	AC022150.4	0.543044255	2.30E-42	postive
GNRH1	AC022150.4	0.741489784	1.99E-94	postive
IL6ST	AC022150.4	0.605922278	6.35E-55	postive
RABEP1	AC022150.4	0.594543702	1.92E-52	postive
ACVR2A	AC022150.4	0.63421641	1.52E-61	postive
ACVR2B	AC022150.4	0.565754208	1.36E-46	postive
ANGPTL1	AC022150.4	0.67261498	1.02E-71	postive
BMPR1A	AC022150.4	0.561561006	8.70E-46	postive
BMPR2	AC022150.4	0.718536952	4.19E-86	postive
CRLF3	AC022150.4	0.650918167	8.66E-66	postive
NR2C2	AC022150.4	0.728504278	1.29E-89	postive
RORA	AC022150.4	0.739060243	1.66E-93	postive
SOS1	AC022150.4	0.67341905	6.00E-72	postive
SOS2	AC022150.4	0.563688017	3.41E-46	postive
BRAF	AC022150.4	0.76539558	4.28E-104	postive
TEC	AC022150.4	0.678465656	2.09E-73	postive
CBL	AC022150.4	0.665066475	1.34E-69	postive
CBLB	AC022150.4	0.687485276	4.39E-76	postive
RASGRP1	AC022150.4	0.509251372	1.21E-36	postive
PDK1	AC022150.4	0.622604754	9.57E-59	postive
TRAJ1	AC022150.4	0.602397588	3.82E-54	postive
TRAJ2	AC022150.4	0.560669235	1.29E-45	postive
TRAJ3	AC022150.4	0.649073463	2.63E-65	postive
TRAJ5	AC022150.4	0.653938404	1.39E-66	postive
TRAJ6	AC022150.4	0.632609968	3.76E-61	postive
TRAJ8	AC022150.4	0.592414644	5.44E-52	postive
TRAJ10	AC022150.4	0.603825614	1.85E-54	postive
TRAJ12	AC022150.4	0.545391394	8.69E-43	postive
TRAJ13	AC022150.4	0.589549399	2.19E-51	postive

TRAJ14	AC022150.4	0.577246218	7.38E-49	postive
TRAJ16	AC022150.4	0.618030556	1.13E-57	postive
TRAJ17	AC022150.4	0.589518801	2.22E-51	postive
TRAJ18	AC022150.4	0.55888349	2.80E-45	postive
TRAJ21	AC022150.4	0.631625033	6.55E-61	postive
TRAJ31	AC022150.4	0.647104086	8.52E-65	postive
TRAJ37	AC022150.4	0.616726926	2.26E-57	postive
TRAJ38	AC022150.4	0.686564801	8.33E-76	postive
TRAJ39	AC022150.4	0.693173592	7.99E-78	postive
TMSB10	CYTOR	0.664885651	1.50E-69	postive
TMSB10	MIR4435-2HG	0.529340322	5.75E-40	postive
CREB1	ZNF460-AS1	0.546126704	6.40E-43	postive
UBR1	ZNF460-AS1	0.506268315	3.61E-36	postive
DDX17	ZNF460-AS1	0.650671251	1.01E-65	postive
TXK	ZNF460-AS1	0.505415708	4.93E-36	postive
NFAT5	ZNF460-AS1	0.640880713	3.31E-63	postive
PIK3R1	ZNF460-AS1	0.50206212	1.66E-35	postive
GNRH1	ZNF460-AS1	0.729385071	6.23E-90	postive
ACVR2A	ZNF460-AS1	0.525805202	2.29E-39	postive
ANGPTL1	ZNF460-AS1	0.514036774	2.05E-37	postive
NR2C1	ZNF460-AS1	0.50113454	2.31E-35	postive
NR2C2	ZNF460-AS1	0.68725095	5.17E-76	postive
RORA	ZNF460-AS1	0.64744054	6.97E-65	postive
BRAF	ZNF460-AS1	0.639247554	8.52E-63	postive
TRAJ1	ZNF460-AS1	0.577549729	6.41E-49	postive
TRAJ2	ZNF460-AS1	0.548507703	2.36E-43	postive
TRAJ3	ZNF460-AS1	0.626511341	1.13E-59	postive
TRAJ5	ZNF460-AS1	0.594391487	2.06E-52	postive
TRAJ6	ZNF460-AS1	0.587675896	5.39E-51	postive
TRAJ8	ZNF460-AS1	0.565247177	1.71E-46	postive
TRAJ10	ZNF460-AS1	0.575412197	1.72E-48	postive
TRAJ12	ZNF460-AS1	0.565492482	1.53E-46	postive
TRAJ13	ZNF460-AS1	0.56183932	7.70E-46	postive
TRAJ14	ZNF460-AS1	0.562366445	6.11E-46	postive
TRAJ16	ZNF460-AS1	0.572546421	6.39E-48	postive
TRAJ17	ZNF460-AS1	0.570311217	1.76E-47	postive
TRAJ18	ZNF460-AS1	0.553375775	3.01E-44	postive
TRAJ21	ZNF460-AS1	0.590189588	1.60E-51	postive
TRAJ31	ZNF460-AS1	0.606345431	5.11E-55	postive
TRAJ37	ZNF460-AS1	0.60205169	4.54E-54	postive
TRAJ38	ZNF460-AS1	0.623564656	5.67E-59	postive
TRAJ39	ZNF460-AS1	0.625093855	2.46E-59	postive
CREB1	AC025171.3	0.62959882	2.04E-60	postive
UBR1	AC025171.3	0.518201034	4.26E-38	postive
IL15	AC025171.3	0.529714292	4.96E-40	postive
DDX17	AC025171.3	0.510904638	6.57E-37	postive
JAK2	AC025171.3	0.503027274	1.17E-35	postive
TXK	AC025171.3	0.527765755	1.07E-39	postive
NFAT5	AC025171.3	0.631046881	9.06E-61	postive
PIK3R1	AC025171.3	0.627177256	7.81E-60	postive
PIK3CA	AC025171.3	0.569961288	2.07E-47	postive
IGHD4-4	AC025171.3	0.587786439	5.11E-51	postive
IGHD6-6	AC025171.3	0.506386421	3.46E-36	postive
GNRH1	AC025171.3	0.590365474	1.47E-51	postive
IL6ST	AC025171.3	0.665682305	9.05E-70	postive
ACVR2A	AC025171.3	0.524802501	3.39E-39	postive
ANGPTL1	AC025171.3	0.668362712	1.62E-70	postive
BMPR2	AC025171.3	0.617116959	1.83E-57	postive
CRLF3	AC025171.3	0.513839925	2.20E-37	postive
NR2C2	AC025171.3	0.608935506	1.35E-55	postive
RORA	AC025171.3	0.755975062	3.76E-100	postive
SOS1	AC025171.3	0.51939273	2.71E-38	postive
BRAF	AC025171.3	0.712639915	4.24E-84	postive
ITK	AC025171.3	0.511330763	5.61E-37	postive
CBLB	AC025171.3	0.590623616	1.30E-51	postive

PDK1	AC025171.3	0.544333593	1.35E-42	postive
TRAJ2	AC025171.3	0.507379453	2.41E-36	postive
TRAJ3	AC025171.3	0.571990515	8.23E-48	postive
TRAJ5	AC025171.3	0.60363906	2.03E-54	postive
TRAJ6	AC025171.3	0.512863286	3.17E-37	postive
TRAJ8	AC025171.3	0.504604335	6.62E-36	postive
TRAJ10	AC025171.3	0.536718735	3.03E-41	postive
TRAJ13	AC025171.3	0.526101023	2.04E-39	postive
TRAJ16	AC025171.3	0.5050542	5.62E-36	postive
TRAJ17	AC025171.3	0.521863194	1.05E-38	postive
TRAJ21	AC025171.3	0.567377763	6.60E-47	postive
TRAJ31	AC025171.3	0.611212191	4.13E-56	postive
TRAJ37	AC025171.3	0.593414259	3.34E-52	postive
TRAJ38	AC025171.3	0.614493242	7.37E-57	postive
TRAJ39	AC025171.3	0.621412357	1.83E-58	postive
CREB1	AC018926.3	0.786290569	1.53E-113	postive
RFXAP	AC018926.3	0.551110868	7.88E-44	postive
UBR1	AC018926.3	0.691776509	2.16E-77	postive
ZC3HAV1	AC018926.3	0.555690216	1.12E-44	postive
IL15	AC018926.3	0.541797534	3.83E-42	postive
CYLD	AC018926.3	0.66489259	1.50E-69	postive
EIF2AK2	AC018926.3	0.537135463	2.56E-41	postive
MAPK8	AC018926.3	0.554982691	1.51E-44	postive
LMBR1	AC018926.3	0.537651772	2.08E-41	postive
IREB2	AC018926.3	0.623406886	6.18E-59	postive
DDX17	AC018926.3	0.67094635	3.03E-71	postive
PIK3CG	AC018926.3	0.527014009	1.43E-39	postive
JAK2	AC018926.3	0.643659605	6.52E-64	postive
TXK	AC018926.3	0.601915483	4.87E-54	postive
NFAT5	AC018926.3	0.927877641	1.66E-230	postive
NFATC3	AC018926.3	0.604246184	1.49E-54	postive
MALT1	AC018926.3	0.515565425	1.15E-37	postive
PIK3R1	AC018926.3	0.648009192	4.97E-65	postive
PIK3CA	AC018926.3	0.647943438	5.16E-65	postive
RASGRP3	AC018926.3	0.54280644	2.53E-42	postive
IGHD4-4	AC018926.3	0.510910181	6.56E-37	postive
IGHD6-6	AC018926.3	0.546819435	4.79E-43	postive
PLXNC1	AC018926.3	0.515272072	1.29E-37	postive
ROBO2	AC018926.3	0.526665663	1.64E-39	postive
GNRH1	AC018926.3	0.788515835	1.31E-114	postive
IL6ST	AC018926.3	0.634847312	1.06E-61	postive
RABEP1	AC018926.3	0.587897131	4.85E-51	postive
ACVR2A	AC018926.3	0.688933926	1.60E-76	postive
ANGPTL1	AC018926.3	0.695220107	1.85E-78	postive
BMPR1A	AC018926.3	0.578637218	3.87E-49	postive
BMPR2	AC018926.3	0.723000178	1.17E-87	postive
CRLF3	AC018926.3	0.6486095	3.47E-65	postive
NR1D2	AC018926.3	0.546083298	6.52E-43	postive
NR2C1	AC018926.3	0.515494771	1.19E-37	postive
NR2C2	AC018926.3	0.729704259	4.78E-90	postive
RORA	AC018926.3	0.868671502	1.01E-164	postive
SOS1	AC018926.3	0.682761592	1.14E-74	postive
SOS2	AC018926.3	0.587937144	4.76E-51	postive
BRAF	AC018926.3	0.800638359	1.15E-120	postive
TEC	AC018926.3	0.503366584	1.04E-35	postive
CBL	AC018926.3	0.569231582	2.87E-47	postive
CBLB	AC018926.3	0.684296737	3.99E-75	postive
RASGRP1	AC018926.3	0.523916271	4.77E-39	postive
PDK1	AC018926.3	0.544202999	1.42E-42	postive
TRAJ1	AC018926.3	0.820715622	1.12E-131	postive
TRAJ2	AC018926.3	0.813847029	9.22E-128	postive
TRAJ3	AC018926.3	0.857086318	1.19E-155	postive
TRAJ5	AC018926.3	0.825714894	1.23E-134	postive
TRAJ6	AC018926.3	0.878223494	7.09E-173	postive
TRAJ8	AC018926.3	0.834553666	4.16E-140	postive

TRAJ10	AC018926.3	0.847937794	4.90E-149	postive
TRAJ12	AC018926.3	0.818774518	1.48E-130	postive
TRAJ13	AC018926.3	0.841627829	1.01E-144	postive
TRAJ14	AC018926.3	0.834844044	2.72E-140	postive
TRAJ16	AC018926.3	0.851146034	2.64E-151	postive
TRAJ17	AC018926.3	0.799727044	3.39E-120	postive
TRAJ18	AC018926.3	0.819520506	5.51E-131	postive
TRAJ21	AC018926.3	0.865763179	2.30E-162	postive
TRAJ31	AC018926.3	0.888300072	2.94E-182	postive
TRAJ37	AC018926.3	0.901421641	6.32E-196	postive
TRAJ38	AC018926.3	0.908924041	1.23E-204	postive
TRAJ39	AC018926.3	0.885438053	1.67E-179	postive
CXCL2	AC020916.1	0.519405522	2.70E-38	postive
FOS	AC020916.1	0.634646004	1.19E-61	postive
HBEGF	AC020916.1	0.561658653	8.33E-46	postive
NR4A1	AC020916.1	0.549578378	1.51E-43	postive
CREB1	AC006270.1	0.713390938	2.37E-84	postive
UBR1	AC006270.1	0.549349472	1.66E-43	postive
ZC3HAV1	AC006270.1	0.506298561	3.57E-36	postive
ZC3HAV1L	AC006270.1	0.528110178	9.32E-40	postive
IL15	AC006270.1	0.626038101	1.46E-59	postive
MAPK8	AC006270.1	0.521068004	1.43E-38	postive
NFKBIZ	AC006270.1	0.540710312	5.99E-42	postive
LMBR1	AC006270.1	0.567950607	5.11E-47	postive
LIMS1	AC006270.1	0.502676695	1.33E-35	postive
IREB2	AC006270.1	0.559584572	2.07E-45	postive
PIK3CG	AC006270.1	0.505363083	5.03E-36	postive
JAK2	AC006270.1	0.608728448	1.50E-55	postive
TXK	AC006270.1	0.519462474	2.64E-38	postive
NFAT5	AC006270.1	0.689598854	1.00E-76	postive
MALT1	AC006270.1	0.515827855	1.05E-37	postive
PIK3R1	AC006270.1	0.582957217	5.10E-50	postive
PIK3CA	AC006270.1	0.665921281	7.77E-70	postive
PIK3CB	AC006270.1	0.523307238	6.04E-39	postive
RASGRP3	AC006270.1	0.52519115	2.91E-39	postive
FGF7	AC006270.1	0.513777568	2.26E-37	postive
GNRH1	AC006270.1	0.640895617	3.28E-63	postive
IL6ST	AC006270.1	0.579791372	2.26E-49	postive
ACVR2A	AC006270.1	0.562245407	6.44E-46	postive
ANGPTL1	AC006270.1	0.775161021	2.21E-108	postive
BMPR2	AC006270.1	0.652133142	4.15E-66	postive
CRLF3	AC006270.1	0.591804464	7.32E-52	postive
NR2C2	AC006270.1	0.617384828	1.59E-57	postive
RORA	AC006270.1	0.751650321	2.11E-98	postive
SOS1	AC006270.1	0.614911882	5.91E-57	postive
SOS2	AC006270.1	0.5508526	8.79E-44	postive
BRAF	AC006270.1	0.721401665	4.25E-87	postive
CBL	AC006270.1	0.547660074	3.37E-43	postive
CBLB	AC006270.1	0.691886919	1.99E-77	postive
PDK1	AC006270.1	0.626591425	1.08E-59	postive
TRAJ1	AC006270.1	0.621733534	1.54E-58	postive
TRAJ2	AC006270.1	0.558131955	3.88E-45	postive
TRAJ3	AC006270.1	0.654386539	1.05E-66	postive
TRAJ5	AC006270.1	0.762281434	9.03E-103	postive
TRAJ6	AC006270.1	0.668382038	1.60E-70	postive
TRAJ8	AC006270.1	0.635426229	7.63E-62	postive
TRAJ10	AC006270.1	0.697805224	2.85E-79	postive
TRAJ12	AC006270.1	0.586244549	1.07E-50	postive
TRAJ13	AC006270.1	0.680574097	5.05E-74	postive
TRAJ14	AC006270.1	0.661398492	1.37E-68	postive
TRAJ16	AC006270.1	0.668043253	1.99E-70	postive
TRAJ17	AC006270.1	0.679876052	8.10E-74	postive
TRAJ18	AC006270.1	0.610250163	6.81E-56	postive
TRAJ21	AC006270.1	0.708571852	9.58E-83	postive
TRAJ31	AC006270.1	0.754111267	2.15E-99	postive

TRAJ37	AC006270.1	0.68654944	8.42E-76	postive
TRAJ38	AC006270.1	0.756365765	2.60E-100	postive
TRAJ39	AC006270.1	0.737365507	7.22E-93	postive
CREB1	AC007546.1	0.747896576	6.53E-97	postive
RFXAP	AC007546.1	0.511706004	4.88E-37	postive
UBR1	AC007546.1	0.575142057	1.95E-48	postive
ZC3HAV1	AC007546.1	0.54327921	2.08E-42	postive
IL15	AC007546.1	0.553904996	2.40E-44	postive
CYLD	AC007546.1	0.631224952	8.20E-61	postive
EIF2AK2	AC007546.1	0.530490336	3.65E-40	postive
MAPK8	AC007546.1	0.54764424	3.39E-43	postive
NFKBIZ	AC007546.1	0.519089157	3.04E-38	postive
LMBR1	AC007546.1	0.540815422	5.74E-42	postive
IREB2	AC007546.1	0.544559211	1.23E-42	postive
DDX17	AC007546.1	0.645446834	2.28E-64	postive
PIK3CG	AC007546.1	0.5259636	2.16E-39	postive
JAK2	AC007546.1	0.625361647	2.12E-59	postive
TXK	AC007546.1	0.586293248	1.05E-50	postive
NFAT5	AC007546.1	0.851749683	9.74E-152	postive
MALT1	AC007546.1	0.559770292	1.90E-45	postive
PIK3R1	AC007546.1	0.639067843	9.45E-63	postive
PIK3CA	AC007546.1	0.675795692	1.25E-72	postive
AKT3	AC007546.1	0.501895299	1.76E-35	postive
RASGRP3	AC007546.1	0.540406623	6.78E-42	postive
GNRH1	AC007546.1	0.749499754	1.52E-97	postive
IL6ST	AC007546.1	0.666977262	3.95E-70	postive
RABEP1	AC007546.1	0.523278426	6.11E-39	postive
ACVR2A	AC007546.1	0.613356186	1.34E-56	postive
ANGPTL1	AC007546.1	0.748953482	2.50E-97	postive
BMPR2	AC007546.1	0.704158529	2.66E-81	postive
CRLF3	AC007546.1	0.641597379	2.18E-63	postive
NR2C1	AC007546.1	0.550117895	1.20E-43	postive
NR2C2	AC007546.1	0.69769169	3.10E-79	postive
RORA	AC007546.1	0.885533869	1.35E-179	postive
SOS1	AC007546.1	0.642701223	1.14E-63	postive
SOS2	AC007546.1	0.572991799	5.22E-48	postive
BRAF	AC007546.1	0.806377962	1.11E-123	postive
ITK	AC007546.1	0.578313766	4.50E-49	postive
CD28	AC007546.1	0.535089004	5.84E-41	postive
CBL	AC007546.1	0.58713788	6.98E-51	postive
CBLB	AC007546.1	0.713827083	1.69E-84	postive
RASGRP1	AC007546.1	0.505371056	5.01E-36	postive
PDK1	AC007546.1	0.562589742	5.53E-46	postive
TRAJ1	AC007546.1	0.731698363	9.00E-91	postive
TRAJ2	AC007546.1	0.719288871	2.30E-86	postive
TRAJ3	AC007546.1	0.759902862	8.97E-102	postive
TRAJ5	AC007546.1	0.830971284	7.49E-138	postive
TRAJ6	AC007546.1	0.770740888	2.05E-106	postive
TRAJ8	AC007546.1	0.748553347	3.60E-97	postive
TRAJ10	AC007546.1	0.770601766	2.36E-106	postive
TRAJ12	AC007546.1	0.685818024	1.40E-75	postive
TRAJ13	AC007546.1	0.801216196	5.77E-121	postive
TRAJ14	AC007546.1	0.755183527	7.90E-100	postive
TRAJ16	AC007546.1	0.783483992	3.27E-112	postive
TRAJ17	AC007546.1	0.751241002	3.08E-98	postive
TRAJ18	AC007546.1	0.72987688	4.14E-90	postive
TRAJ21	AC007546.1	0.820208809	2.20E-131	postive
TRAJ31	AC007546.1	0.84228392	3.66E-145	postive
TRAJ37	AC007546.1	0.820666944	1.19E-131	postive
TRAJ38	AC007546.1	0.845532089	2.28E-147	postive
TRAJ39	AC007546.1	0.834851976	2.69E-140	postive
CREB1	AP000766.1	0.806772179	6.82E-124	postive
RFXAP	AP000766.1	0.56141689	9.27E-46	postive
UBR1	AP000766.1	0.682459957	1.40E-74	postive
ZC3HAV1	AP000766.1	0.584961732	1.97E-50	postive

IL15	AP000766.1	0.53643025	3.41E-41	postive
CYLD	AP000766.1	0.681957256	1.98E-74	postive
EIF2AK2	AP000766.1	0.562493308	5.77E-46	postive
MAPK8	AP000766.1	0.567224552	7.07E-47	postive
LMBR1	AP000766.1	0.555657558	1.13E-44	postive
LIMS1	AP000766.1	0.502048292	1.67E-35	postive
IREB2	AP000766.1	0.625552736	1.91E-59	postive
DDX17	AP000766.1	0.692059522	1.76E-77	postive
PIK3CG	AP000766.1	0.542632539	2.72E-42	postive
JAK2	AP000766.1	0.653693429	1.61E-66	postive
TXK	AP000766.1	0.632877973	3.24E-61	postive
NFAT5	AP000766.1	0.913136235	7.30E-210	postive
NFATC3	AP000766.1	0.593926186	2.60E-52	postive
MALT1	AP000766.1	0.540306701	7.06E-42	postive
PIK3R1	AP000766.1	0.693132074	8.23E-78	postive
PIK3CA	AP000766.1	0.669331547	8.66E-71	postive
PIK3CB	AP000766.1	0.514876299	1.50E-37	postive
AKT3	AP000766.1	0.522948167	6.94E-39	postive
RASGRP3	AP000766.1	0.532273511	1.80E-40	postive
IGHD6-6	AP000766.1	0.524941317	3.21E-39	postive
PLXNC1	AP000766.1	0.513052806	2.96E-37	postive
ROBO2	AP000766.1	0.506327637	3.54E-36	postive
GNRH1	AP000766.1	0.778870572	4.55E-110	postive
IL6ST	AP000766.1	0.681940883	2.00E-74	postive
RABEP1	AP000766.1	0.614864676	6.06E-57	postive
ACVR2A	AP000766.1	0.670328168	4.54E-71	postive
ANGPTL1	AP000766.1	0.744287153	1.67E-95	postive
BMPR1A	AP000766.1	0.570355785	1.73E-47	postive
BMPR2	AP000766.1	0.756174309	3.11E-100	postive
CRLF3	AP000766.1	0.642008628	1.72E-63	postive
LIFR	AP000766.1	0.537260484	2.44E-41	postive
NR1D2	AP000766.1	0.576027558	1.30E-48	postive
NR2C1	AP000766.1	0.537549594	2.17E-41	postive
NR2C2	AP000766.1	0.745737396	4.57E-96	postive
RORA	AP000766.1	0.887452087	1.96E-181	postive
SOS1	AP000766.1	0.712109835	6.39E-84	postive
SOS2	AP000766.1	0.604440727	1.35E-54	postive
BRAF	AP000766.1	0.832591348	7.27E-139	postive
ITK	AP000766.1	0.538004393	1.80E-41	postive
TEC	AP000766.1	0.512004116	4.37E-37	postive
CD28	AP000766.1	0.52067269	1.66E-38	postive
CBL	AP000766.1	0.659040088	5.97E-68	postive
CBLB	AP000766.1	0.749156247	2.08E-97	postive
RASGRP1	AP000766.1	0.532602985	1.58E-40	postive
PDK1	AP000766.1	0.539419151	1.01E-41	postive
TRAJ1	AP000766.1	0.77056918	2.44E-106	postive
TRAJ2	AP000766.1	0.784552325	1.03E-112	postive
TRAJ3	AP000766.1	0.820340803	1.84E-131	postive
TRAJ5	AP000766.1	0.811783611	1.29E-126	postive
TRAJ6	AP000766.1	0.831230848	5.16E-138	postive
TRAJ8	AP000766.1	0.776275943	6.93E-109	postive
TRAJ10	AP000766.1	0.799902895	2.75E-120	postive
TRAJ12	AP000766.1	0.775757929	1.19E-108	postive
TRAJ13	AP000766.1	0.811373551	2.17E-126	postive
TRAJ14	AP000766.1	0.799896438	2.77E-120	postive
TRAJ16	AP000766.1	0.802095922	2.01E-121	postive
TRAJ17	AP000766.1	0.749477991	1.55E-97	postive
TRAJ18	AP000766.1	0.796334631	1.81E-118	postive
TRAJ21	AP000766.1	0.84160853	1.04E-144	postive
TRAJ31	AP000766.1	0.851640447	1.17E-151	postive
TRAJ37	AP000766.1	0.860188261	5.34E-158	postive
TRAJ38	AP000766.1	0.881877703	3.53E-176	postive
TRAJ39	AP000766.1	0.848424621	2.24E-149	postive
CREB1	AC108449.2	0.816692296	2.30E-129	postive
RFXAP	AC108449.2	0.57750809	6.53E-49	postive

UBR1	AC108449.2	0.716838539	1.60E-85	postive
ZC3HAV1	AC108449.2	0.607502821	2.82E-55	postive
ZC3HAV1L	AC108449.2	0.579319894	2.81E-49	postive
IL15	AC108449.2	0.545777334	7.40E-43	postive
CYLD	AC108449.2	0.604083638	1.62E-54	postive
EIF2AK2	AC108449.2	0.561848111	7.67E-46	postive
MAPK8	AC108449.2	0.622058944	1.29E-58	postive
LMBR1	AC108449.2	0.581116689	1.21E-49	postive
SP1	AC108449.2	0.543789197	1.69E-42	postive
IREB2	AC108449.2	0.702413983	9.72E-81	postive
DDX17	AC108449.2	0.672108343	1.42E-71	postive
PIK3CG	AC108449.2	0.545996583	6.75E-43	postive
JAK2	AC108449.2	0.608704066	1.52E-55	postive
TXK	AC108449.2	0.628846934	3.09E-60	postive
PPP3CB	AC108449.2	0.533977698	9.12E-41	postive
NFAT5	AC108449.2	0.826493363	4.17E-135	postive
NFATC3	AC108449.2	0.508715707	1.47E-36	postive
MALT1	AC108449.2	0.551510688	6.65E-44	postive
PIK3R1	AC108449.2	0.683781479	5.68E-75	postive
PIK3CA	AC108449.2	0.69926816	9.83E-80	postive
PIK3CB	AC108449.2	0.538745888	1.33E-41	postive
AKT3	AC108449.2	0.584596478	2.35E-50	postive
GSK3B	AC108449.2	0.527654368	1.11E-39	postive
RASGRP3	AC108449.2	0.537343862	2.35E-41	postive
IGHD4-4	AC108449.2	0.587727193	5.26E-51	postive
IGHD6-6	AC108449.2	0.571486957	1.04E-47	postive
LTB4R2	AC108449.2	0.500823196	2.59E-35	postive
GNRH1	AC108449.2	0.807875796	1.74E-124	postive
IL6ST	AC108449.2	0.667489773	2.84E-70	postive
RABEP1	AC108449.2	0.645458867	2.26E-64	postive
ACVR2A	AC108449.2	0.697984139	2.51E-79	postive
ACVR2B	AC108449.2	0.518117328	4.40E-38	postive
ANGPTL1	AC108449.2	0.754554732	1.42E-99	postive
BMPR1A	AC108449.2	0.611409155	3.72E-56	postive
BMPR2	AC108449.2	0.789066142	7.09E-115	postive
CRLF3	AC108449.2	0.626585566	1.08E-59	postive
NR1D2	AC108449.2	0.564788044	2.09E-46	postive
NR2C1	AC108449.2	0.514246124	1.89E-37	postive
NR2C2	AC108449.2	0.785923571	2.29E-113	postive
NR3C1	AC108449.2	0.518196685	4.27E-38	postive
RORA	AC108449.2	0.833343075	2.44E-139	postive
SOS1	AC108449.2	0.751974647	1.57E-98	postive
SOS2	AC108449.2	0.62668864	1.02E-59	postive
BRAF	AC108449.2	0.822422933	1.12E-132	postive
TEC	AC108449.2	0.53023909	4.03E-40	postive
CBL	AC108449.2	0.647229255	7.91E-65	postive
CBLB	AC108449.2	0.709845683	3.63E-83	postive
RASGRP1	AC108449.2	0.525454408	2.63E-39	postive
PDK1	AC108449.2	0.597490752	4.46E-53	postive
TRAJ1	AC108449.2	0.686335045	9.76E-76	postive
TRAJ2	AC108449.2	0.689139708	1.38E-76	postive
TRAJ3	AC108449.2	0.76734354	6.21E-105	postive
TRAJ5	AC108449.2	0.733132208	2.69E-91	postive
TRAJ6	AC108449.2	0.735738637	2.92E-92	postive
TRAJ8	AC108449.2	0.72294264	1.23E-87	postive
TRAJ10	AC108449.2	0.705459174	1.00E-81	postive
TRAJ12	AC108449.2	0.654005532	1.33E-66	postive
TRAJ13	AC108449.2	0.713361644	2.42E-84	postive
TRAJ14	AC108449.2	0.697033536	4.99E-79	postive
TRAJ16	AC108449.2	0.715782896	3.67E-85	postive
TRAJ17	AC108449.2	0.695108194	2.00E-78	postive
TRAJ18	AC108449.2	0.654735597	8.51E-67	postive
TRAJ21	AC108449.2	0.738684001	2.31E-93	postive
TRAJ31	AC108449.2	0.763068916	4.20E-103	postive
TRAJ37	AC108449.2	0.755322802	6.93E-100	postive

TRAJ38	AC108449.2	0.791215776	6.35E-116	postive
TRAJ39	AC108449.2	0.777711427	1.54E-109	postive
CSPG5	SNHG19	0.515627545	1.13E-37	postive
IL34	SNHG19	0.511952223	4.46E-37	postive
BMPR1B	SNHG19	0.597812755	3.80E-53	postive
CREB1	MUC20-OT1	0.543779806	1.69E-42	postive
RFXAP	MUC20-OT1	0.510599267	7.36E-37	postive
ZC3HAV1L	MUC20-OT1	0.51209157	4.23E-37	postive
NFKBIZ	MUC20-OT1	0.5151221	1.36E-37	postive
DDX17	MUC20-OT1	0.632011794	5.27E-61	postive
NFAT5	MUC20-OT1	0.619443514	5.28E-58	postive
PIK3CA	MUC20-OT1	0.544520363	1.25E-42	postive
LTB4R2	MUC20-OT1	0.618861566	7.22E-58	postive
GNRH1	MUC20-OT1	0.738624086	2.43E-93	postive
ANGPTL1	MUC20-OT1	0.563148175	4.33E-46	postive
NR2C1	MUC20-OT1	0.593275798	3.57E-52	postive
NR2C2	MUC20-OT1	0.702826253	7.16E-81	postive
RORA	MUC20-OT1	0.594271282	2.19E-52	postive
SOS1	MUC20-OT1	0.55011612	1.20E-43	postive
BRAF	MUC20-OT1	0.626199734	1.34E-59	postive
CBL	MUC20-OT1	0.544264913	1.39E-42	postive
CBLB	MUC20-OT1	0.568580433	3.85E-47	postive
PDK1	MUC20-OT1	0.515457592	1.20E-37	postive
TRAJ1	MUC20-OT1	0.560036457	1.70E-45	postive
TRAJ2	MUC20-OT1	0.520087494	2.08E-38	postive
TRAJ3	MUC20-OT1	0.585149669	1.80E-50	postive
TRAJ5	MUC20-OT1	0.564672854	2.20E-46	postive
TRAJ6	MUC20-OT1	0.558519907	3.28E-45	postive
TRAJ8	MUC20-OT1	0.514885492	1.49E-37	postive
TRAJ10	MUC20-OT1	0.546471888	5.54E-43	postive
TRAJ13	MUC20-OT1	0.523614389	5.36E-39	postive
TRAJ14	MUC20-OT1	0.522622532	7.86E-39	postive
TRAJ16	MUC20-OT1	0.533938211	9.27E-41	postive
TRAJ17	MUC20-OT1	0.522113936	9.56E-39	postive
TRAJ18	MUC20-OT1	0.507549575	2.26E-36	postive
TRAJ21	MUC20-OT1	0.557747367	4.59E-45	postive
TRAJ31	MUC20-OT1	0.560365507	1.47E-45	postive
TRAJ37	MUC20-OT1	0.556287665	8.63E-45	postive
TRAJ38	MUC20-OT1	0.582873857	5.31E-50	postive
TRAJ39	MUC20-OT1	0.580031972	2.02E-49	postive
CREB1	AP003170.3	0.627191465	7.75E-60	postive
RFXAP	AP003170.3	0.514511959	1.71E-37	postive
UBR1	AP003170.3	0.549089599	1.85E-43	postive
DDX17	AP003170.3	0.65575837	4.54E-67	postive
JAK2	AP003170.3	0.502136965	1.61E-35	postive
TXK	AP003170.3	0.536858123	2.87E-41	postive
NFAT5	AP003170.3	0.716463712	2.15E-85	postive
MALT1	AP003170.3	0.507067379	2.70E-36	postive
PIK3R1	AP003170.3	0.537700514	2.04E-41	postive
PIK3CA	AP003170.3	0.518973429	3.18E-38	postive
GNRH1	AP003170.3	0.720347718	9.90E-87	postive
RABEP1	AP003170.3	0.512922635	3.11E-37	postive
ACVR2A	AP003170.3	0.522567235	8.03E-39	postive
ANGPTL1	AP003170.3	0.641373561	2.48E-63	postive
BMPR2	AP003170.3	0.557098028	6.08E-45	postive
CRLF3	AP003170.3	0.522949803	6.93E-39	postive
NR2C1	AP003170.3	0.542335719	3.07E-42	postive
NR2C2	AP003170.3	0.68059192	4.99E-74	postive
RORA	AP003170.3	0.700333201	4.51E-80	postive
SOS1	AP003170.3	0.602894278	2.97E-54	postive
BRAF	AP003170.3	0.70022871	4.86E-80	postive
CBL	AP003170.3	0.561325205	9.65E-46	postive
CBLB	AP003170.3	0.683563318	6.60E-75	postive
PDK1	AP003170.3	0.510279878	8.28E-37	postive
TRAJ1	AP003170.3	0.580225881	1.84E-49	postive

TRAJ2	AP003170.3	0.620020245	3.87E-58	postive
TRAJ3	AP003170.3	0.634528343	1.27E-61	postive
TRAJ5	AP003170.3	0.601303668	6.63E-54	postive
TRAJ6	AP003170.3	0.616168678	3.04E-57	postive
TRAJ8	AP003170.3	0.550003879	1.26E-43	postive
TRAJ10	AP003170.3	0.574948096	2.13E-48	postive
TRAJ12	AP003170.3	0.564771696	2.11E-46	postive
TRAJ13	AP003170.3	0.585054433	1.89E-50	postive
TRAJ14	AP003170.3	0.595438432	1.23E-52	postive
TRAJ16	AP003170.3	0.581939976	8.24E-50	postive
TRAJ17	AP003170.3	0.514189842	1.93E-37	postive
TRAJ18	AP003170.3	0.598042012	3.39E-53	postive
TRAJ21	AP003170.3	0.6282641	4.28E-60	postive
TRAJ31	AP003170.3	0.610370964	6.39E-56	postive
TRAJ37	AP003170.3	0.62947482	2.18E-60	postive
TRAJ38	AP003170.3	0.67385029	4.52E-72	postive
TRAJ39	AP003170.3	0.630629839	1.14E-60	postive
SOS1	AC005034.4	0.573078146	5.02E-48	postive
BRAF	AC005034.4	0.546621255	5.21E-43	postive
CREB1	AC073046.1	0.605615437	7.43E-55	postive
UBR1	AC073046.1	0.603284761	2.43E-54	postive
ZC3HAV1	AC073046.1	0.571268915	1.14E-47	postive
ZC3HAV1L	AC073046.1	0.637153032	2.84E-62	postive
EIF2AK2	AC073046.1	0.526927474	1.48E-39	postive
IREB2	AC073046.1	0.658668688	7.52E-68	postive
PIK3CG	AC073046.1	0.501333821	2.15E-35	postive
NFAT5	AC073046.1	0.524502591	3.80E-39	postive
PIK3CA	AC073046.1	0.611594099	3.38E-56	postive
PIK3CB	AC073046.1	0.505627471	4.56E-36	postive
GSK3B	AC073046.1	0.597346647	4.79E-53	postive
RABEP1	AC073046.1	0.534563756	7.21E-41	postive
BMPR2	AC073046.1	0.500671098	2.73E-35	postive
NR2C2	AC073046.1	0.552814411	3.82E-44	postive
PTPN11	AC073046.1	0.518123671	4.39E-38	postive
SOS1	AC073046.1	0.605834096	6.64E-55	postive
SOS2	AC073046.1	0.501797333	1.82E-35	postive
BRAF	AC073046.1	0.561986011	7.22E-46	postive
CBL	AC073046.1	0.603164847	2.59E-54	postive
TRAJ3	AC073046.1	0.539976287	8.08E-42	postive
TRAJ6	AC073046.1	0.504998585	5.74E-36	postive
OSGIN1	AP003119.1	0.579818689	2.23E-49	postive
NR0B1	AP003119.1	0.522408803	8.54E-39	postive
CREB1	AC012181.1	0.679572685	9.94E-74	postive
RFXAP	AC012181.1	0.517933069	4.72E-38	postive
UBR1	AC012181.1	0.557162565	5.91E-45	postive
ZC3HAV1	AC012181.1	0.518444676	3.89E-38	postive
IL15	AC012181.1	0.593509812	3.18E-52	postive
CYLD	AC012181.1	0.600307102	1.09E-53	postive
NFKBIZ	AC012181.1	0.521348275	1.28E-38	postive
IREB2	AC012181.1	0.525649077	2.44E-39	postive
DDX17	AC012181.1	0.658028047	1.12E-67	postive
PIK3CG	AC012181.1	0.619530403	5.04E-58	postive
JAK2	AC012181.1	0.647504614	6.71E-65	postive
TXK	AC012181.1	0.617524765	1.48E-57	postive
NFAT5	AC012181.1	0.662606125	6.38E-69	postive
MALT1	AC012181.1	0.528164416	9.12E-40	postive
PIK3R1	AC012181.1	0.619080703	6.42E-58	postive
PIK3CA	AC012181.1	0.619775139	4.42E-58	postive
RASGRP3	AC012181.1	0.615927791	3.45E-57	postive
IGHD6-6	AC012181.1	0.588671722	3.34E-51	postive
IGKJ5	AC012181.1	0.518900011	3.27E-38	postive
LTB4R2	AC012181.1	0.502978529	1.19E-35	postive
PLXNC1	AC012181.1	0.547852348	3.11E-43	postive
GNRH1	AC012181.1	0.722247237	2.15E-87	postive
IL6ST	AC012181.1	0.577131184	7.78E-49	postive

RABEP1	AC012181.1	0.521070663	1.43E-38	postive
ACVR2A	AC012181.1	0.546809427	4.81E-43	postive
ANGPTL1	AC012181.1	0.715456956	4.74E-85	postive
BMPR2	AC012181.1	0.622060324	1.29E-58	postive
CRLF3	AC012181.1	0.629452024	2.21E-60	postive
NR2C1	AC012181.1	0.508872543	1.39E-36	postive
NR2C2	AC012181.1	0.702561471	8.71E-81	postive
RORA	AC012181.1	0.736010462	2.31E-92	postive
SOS1	AC012181.1	0.586698942	8.61E-51	postive
SOS2	AC012181.1	0.544953126	1.04E-42	postive
BRAF	AC012181.1	0.728958534	8.88E-90	postive
PTPRC	AC012181.1	0.533928587	9.30E-41	postive
ITK	AC012181.1	0.618105623	1.08E-57	postive
CD28	AC012181.1	0.503182455	1.11E-35	postive
CBL	AC012181.1	0.587816261	5.04E-51	postive
CBLB	AC012181.1	0.702510857	9.04E-81	postive
RASGRP1	AC012181.1	0.517133696	6.39E-38	postive
PDK1	AC012181.1	0.684125466	4.48E-75	postive
TRAJ1	AC012181.1	0.599673739	1.50E-53	postive
TRAJ2	AC012181.1	0.627432749	6.78E-60	postive
TRAJ3	AC012181.1	0.671038145	2.86E-71	postive
TRAJ5	AC012181.1	0.636938045	3.22E-62	postive
TRAJ6	AC012181.1	0.612767924	1.83E-56	postive
TRAJ8	AC012181.1	0.572201524	7.48E-48	postive
TRAJ10	AC012181.1	0.577446996	6.72E-49	postive
TRAJ12	AC012181.1	0.53251015	1.64E-40	postive
TRAJ13	AC012181.1	0.574443076	2.69E-48	postive
TRAJ14	AC012181.1	0.595169625	1.41E-52	postive
TRAJ16	AC012181.1	0.604885125	1.08E-54	postive
TRAJ17	AC012181.1	0.57015392	1.89E-47	postive
TRAJ18	AC012181.1	0.583023173	4.95E-50	postive
TRAJ21	AC012181.1	0.626708416	1.01E-59	postive
TRAJ31	AC012181.1	0.619259239	5.83E-58	postive
TRAJ37	AC012181.1	0.585535725	1.50E-50	postive
TRAJ38	AC012181.1	0.690892824	4.03E-77	postive
TRAJ39	AC012181.1	0.652219435	3.94E-66	postive
DDX17	AC008982.2	0.523741068	5.11E-39	postive
GNRH1	AC008982.2	0.58405415	3.04E-50	postive
NR2C1	AC008982.2	0.536510786	3.30E-41	postive
NR2C2	AC008982.2	0.643602422	6.75E-64	postive
BRAF	AC008982.2	0.500654087	2.75E-35	postive
DEFA5	AL031058.1	0.72404225	5.04E-88	postive
FGF19	AL031058.1	0.559111061	2.54E-45	postive
SHC3	AL353150.1	0.67425709	3.45E-72	postive
CREB1	RHOA-IT1	0.789854532	2.94E-115	postive
UBR1	RHOA-IT1	0.678213738	2.48E-73	postive
ZC3HAV1	RHOA-IT1	0.549757795	1.40E-43	postive
ZC3HAV1L	RHOA-IT1	0.538278328	1.61E-41	postive
IL15	RHOA-IT1	0.561964736	7.29E-46	postive
CYLD	RHOA-IT1	0.601560135	5.82E-54	postive
EIF2AK2	RHOA-IT1	0.529744405	4.90E-40	postive
MAPK8	RHOA-IT1	0.54222887	3.21E-42	postive
TLR1	RHOA-IT1	0.508318839	1.71E-36	postive
NFKBIZ	RHOA-IT1	0.542566672	2.79E-42	postive
LMBR1	RHOA-IT1	0.556532561	7.76E-45	postive
SP1	RHOA-IT1	0.51461755	1.65E-37	postive
IREB2	RHOA-IT1	0.614333305	8.02E-57	postive
DDX17	RHOA-IT1	0.653610934	1.69E-66	postive
PIK3CG	RHOA-IT1	0.534000907	9.04E-41	postive
JAK2	RHOA-IT1	0.668086076	1.94E-70	postive
TXK	RHOA-IT1	0.607430664	2.93E-55	postive
NFAT5	RHOA-IT1	0.796025229	2.59E-118	postive
MALT1	RHOA-IT1	0.528784581	7.15E-40	postive
PIK3R1	RHOA-IT1	0.603335668	2.37E-54	postive
PIK3CA	RHOA-IT1	0.680974441	3.85E-74	postive

PIK3CB	RHOA-IT1	0.545844359	7.20E-43	postive
RASGRP3	RHOA-IT1	0.536763417	2.98E-41	postive
PLXNC1	RHOA-IT1	0.504267441	7.48E-36	postive
ROBO2	RHOA-IT1	0.519557128	2.54E-38	postive
GNRH1	RHOA-IT1	0.79617707	2.17E-118	postive
IL6ST	RHOA-IT1	0.568098733	4.78E-47	postive
RABEP1	RHOA-IT1	0.546917952	4.60E-43	postive
ACVR2A	RHOA-IT1	0.642315054	1.43E-63	postive
ANGPTL1	RHOA-IT1	0.737077597	9.25E-93	postive
BMPR1A	RHOA-IT1	0.567566099	6.07E-47	postive
BMPR2	RHOA-IT1	0.729566162	5.36E-90	postive
CRLF3	RHOA-IT1	0.633684239	2.05E-61	postive
NR1D2	RHOA-IT1	0.553155471	3.31E-44	postive
NR2C1	RHOA-IT1	0.533869244	9.52E-41	postive
NR2C2	RHOA-IT1	0.757888675	6.14E-101	postive
RORA	RHOA-IT1	0.770245427	3.38E-106	postive
SOS1	RHOA-IT1	0.687239806	5.21E-76	postive
SOS2	RHOA-IT1	0.565774753	1.35E-46	postive
BRAF	RHOA-IT1	0.757839726	6.44E-101	postive
CBL	RHOA-IT1	0.60486872	1.09E-54	postive
CBLB	RHOA-IT1	0.749192034	2.01E-97	postive
PDK1	RHOA-IT1	0.593864732	2.67E-52	postive
TRAJ1	RHOA-IT1	0.664035365	2.58E-69	postive
TRAJ2	RHOA-IT1	0.681377745	2.93E-74	postive
TRAJ3	RHOA-IT1	0.714624805	9.08E-85	postive
TRAJ5	RHOA-IT1	0.680778277	4.40E-74	postive
TRAJ6	RHOA-IT1	0.718558326	4.12E-86	postive
TRAJ8	RHOA-IT1	0.645853412	1.79E-64	postive
TRAJ10	RHOA-IT1	0.701201827	2.38E-80	postive
TRAJ12	RHOA-IT1	0.643563865	6.90E-64	postive
TRAJ13	RHOA-IT1	0.670665379	3.64E-71	postive
TRAJ14	RHOA-IT1	0.66284858	5.48E-69	postive
TRAJ16	RHOA-IT1	0.704435869	2.16E-81	postive
TRAJ17	RHOA-IT1	0.641442206	2.39E-63	postive
TRAJ18	RHOA-IT1	0.657115521	1.97E-67	postive
TRAJ21	RHOA-IT1	0.698568187	1.64E-79	postive
TRAJ31	RHOA-IT1	0.725129044	2.08E-88	postive
TRAJ37	RHOA-IT1	0.687627431	3.98E-76	postive
TRAJ38	RHOA-IT1	0.784069448	1.74E-112	postive
TRAJ39	RHOA-IT1	0.695965858	1.08E-78	postive
PSME3	AC005288.1	0.524167965	4.33E-39	postive
BECN1	AC005288.1	0.501969665	1.71E-35	postive
CREB1	AC002553.1	0.6278437	5.40E-60	postive
RFXAP	AC002553.1	0.520235279	1.96E-38	postive
UBR1	AC002553.1	0.548174556	2.72E-43	postive
MAPK8	AC002553.1	0.503571379	9.62E-36	postive
IREB2	AC002553.1	0.521697187	1.12E-38	postive
DDX17	AC002553.1	0.684630588	3.17E-75	postive
TXK	AC002553.1	0.533175644	1.26E-40	postive
NFAT5	AC002553.1	0.711657191	9.05E-84	postive
PIK3CA	AC002553.1	0.516713179	7.49E-38	postive
IGHD6-6	AC002553.1	0.505718973	4.42E-36	postive
LTB4R2	AC002553.1	0.540853632	5.65E-42	postive
GNRH1	AC002553.1	0.841580863	1.08E-144	postive
RABEP1	AC002553.1	0.540260197	7.20E-42	postive
ACVR2A	AC002553.1	0.620627858	2.79E-58	postive
ANGPTL1	AC002553.1	0.626073167	1.44E-59	postive
BMPR2	AC002553.1	0.578951089	3.34E-49	postive
CRLF3	AC002553.1	0.545651941	7.80E-43	postive
NR2C1	AC002553.1	0.577121217	7.82E-49	postive
NR2C2	AC002553.1	0.759499305	1.32E-101	postive
RORA	AC002553.1	0.678329053	2.30E-73	postive
SOS1	AC002553.1	0.620714227	2.66E-58	postive
BRAF	AC002553.1	0.724129654	4.69E-88	postive
CBL	AC002553.1	0.512043937	4.31E-37	postive

CBLB	AC002553.1	0.580445496	1.66E-49	postive
TRAJ1	AC002553.1	0.682710586	1.18E-74	postive
TRAJ2	AC002553.1	0.590470926	1.40E-51	postive
TRAJ3	AC002553.1	0.702190026	1.15E-80	postive
TRAJ5	AC002553.1	0.696688428	6.41E-79	postive
TRAJ6	AC002553.1	0.679766518	8.73E-74	postive
TRAJ8	AC002553.1	0.698110595	2.29E-79	postive
TRAJ10	AC002553.1	0.652310263	3.73E-66	postive
TRAJ12	AC002553.1	0.623674329	5.34E-59	postive
TRAJ13	AC002553.1	0.68048191	5.38E-74	postive
TRAJ14	AC002553.1	0.667805048	2.32E-70	postive
TRAJ16	AC002553.1	0.676778096	6.48E-73	postive
TRAJ17	AC002553.1	0.677633377	3.66E-73	postive
TRAJ18	AC002553.1	0.595427982	1.24E-52	postive
TRAJ21	AC002553.1	0.698526444	1.69E-79	postive
TRAJ31	AC002553.1	0.703437756	4.55E-81	postive
TRAJ37	AC002553.1	0.685103275	2.29E-75	postive
TRAJ38	AC002553.1	0.707727929	1.82E-82	postive
TRAJ39	AC002553.1	0.72623223	8.43E-89	postive
CREB1	AC130895.1	0.783227807	4.32E-112	postive
UBR1	AC130895.1	0.644047988	5.19E-64	postive
ZC3HAV1	AC130895.1	0.565407323	1.59E-46	postive
ZC3HAV1L	AC130895.1	0.542888065	2.45E-42	postive
IL15	AC130895.1	0.560142028	1.62E-45	postive
CYLD	AC130895.1	0.55134331	7.14E-44	postive
EIF2AK2	AC130895.1	0.543651119	1.79E-42	postive
MAPK8	AC130895.1	0.571074002	1.25E-47	postive
LMBR1	AC130895.1	0.607548653	2.75E-55	postive
LIMS1	AC130895.1	0.502150861	1.61E-35	postive
IREB2	AC130895.1	0.63425686	1.48E-61	postive
DDX17	AC130895.1	0.55680684	6.89E-45	postive
PIK3CG	AC130895.1	0.518667317	3.57E-38	postive
JAK2	AC130895.1	0.602457316	3.70E-54	postive
TXK	AC130895.1	0.556876783	6.69E-45	postive
PPP3CB	AC130895.1	0.517520827	5.52E-38	postive
NFAT5	AC130895.1	0.793396414	5.34E-117	postive
MALT1	AC130895.1	0.522150133	9.43E-39	postive
PIK3R1	AC130895.1	0.619596984	4.86E-58	postive
PIK3CA	AC130895.1	0.6780765	2.72E-73	postive
PIK3CB	AC130895.1	0.521357218	1.28E-38	postive
AKT3	AC130895.1	0.612286761	2.35E-56	postive
RASGRP3	AC130895.1	0.515059776	1.40E-37	postive
IGHD4-4	AC130895.1	0.524732575	3.48E-39	postive
PLXNC1	AC130895.1	0.505763592	4.34E-36	postive
GMFB	AC130895.1	0.501662588	1.91E-35	postive
GNRH1	AC130895.1	0.720306967	1.02E-86	postive
IL6ST	AC130895.1	0.610396586	6.31E-56	postive
RABEP1	AC130895.1	0.565662925	1.42E-46	postive
ACVR2A	AC130895.1	0.651911782	4.75E-66	postive
ANGPTL1	AC130895.1	0.77823556	8.89E-110	postive
BMPR1A	AC130895.1	0.573926062	3.40E-48	postive
BMPR2	AC130895.1	0.735317586	4.19E-92	postive
CRLF3	AC130895.1	0.680633988	4.85E-74	postive
NR2C2	AC130895.1	0.663529874	3.56E-69	postive
RORA	AC130895.1	0.777853489	1.33E-109	postive
SOS1	AC130895.1	0.698012485	2.45E-79	postive
SOS2	AC130895.1	0.587376209	6.23E-51	postive
BRAF	AC130895.1	0.829031882	1.18E-136	postive
CBL	AC130895.1	0.595749252	1.06E-52	postive
CBLB	AC130895.1	0.693418989	6.71E-78	postive
PDK1	AC130895.1	0.583764448	3.48E-50	postive
TRAJ1	AC130895.1	0.734189692	1.10E-91	postive
TRAJ2	AC130895.1	0.732480316	4.66E-91	postive
TRAJ3	AC130895.1	0.78078375	5.97E-111	postive
TRAJ5	AC130895.1	0.800405027	1.52E-120	postive

TRAJ6	AC130895.1	0.786583522	1.11E-113	postive
TRAJ8	AC130895.1	0.829760892	4.21E-137	postive
TRAJ10	AC130895.1	0.743347426	3.85E-95	postive
TRAJ12	AC130895.1	0.68299914	9.70E-75	postive
TRAJ13	AC130895.1	0.82139532	4.47E-132	postive
TRAJ14	AC130895.1	0.793235405	6.42E-117	postive
TRAJ16	AC130895.1	0.778082143	1.05E-109	postive
TRAJ17	AC130895.1	0.756317828	2.72E-100	postive
TRAJ18	AC130895.1	0.649246337	2.37E-65	postive
TRAJ21	AC130895.1	0.816496066	2.98E-129	postive
TRAJ31	AC130895.1	0.832619304	6.98E-139	postive
TRAJ37	AC130895.1	0.791839979	3.14E-116	postive
TRAJ38	AC130895.1	0.849997769	1.74E-150	postive
TRAJ39	AC130895.1	0.83676047	1.60E-141	postive
UCN	AC073389.3	0.61292474	1.68E-56	postive
DDX17	AC025178.2	0.591495721	8.51E-52	postive
NFAT5	AC025178.2	0.560296609	1.51E-45	postive
LTB4R2	AC025178.2	0.503752958	9.01E-36	postive
GNRH1	AC025178.2	0.677951852	2.96E-73	postive
ACVR2A	AC025178.2	0.508366549	1.68E-36	postive
ANGPTL1	AC025178.2	0.50891665	1.37E-36	postive
NR2C1	AC025178.2	0.563443208	3.80E-46	postive
NR2C2	AC025178.2	0.67926245	1.23E-73	postive
RORA	AC025178.2	0.568861322	3.39E-47	postive
SOS1	AC025178.2	0.530223947	4.05E-40	postive
BRAF	AC025178.2	0.658001789	1.14E-67	postive
CBLB	AC025178.2	0.55694277	6.50E-45	postive
TRAJ1	AC025178.2	0.57664704	9.73E-49	postive
TRAJ3	AC025178.2	0.581558774	9.86E-50	postive
TRAJ5	AC025178.2	0.622871192	8.28E-59	postive
TRAJ6	AC025178.2	0.572202254	7.48E-48	postive
TRAJ8	AC025178.2	0.5994794	1.66E-53	postive
TRAJ10	AC025178.2	0.553233249	3.20E-44	postive
TRAJ12	AC025178.2	0.590868774	1.15E-51	postive
TRAJ13	AC025178.2	0.594565463	1.90E-52	postive
TRAJ14	AC025178.2	0.604633986	1.23E-54	postive
TRAJ16	AC025178.2	0.576952224	8.45E-49	postive
TRAJ17	AC025178.2	0.600893356	8.15E-54	postive
TRAJ18	AC025178.2	0.53073022	3.32E-40	postive
TRAJ21	AC025178.2	0.614096863	9.09E-57	postive
TRAJ31	AC025178.2	0.6218314	1.46E-58	postive
TRAJ37	AC025178.2	0.584408568	2.57E-50	postive
TRAJ38	AC025178.2	0.619916846	4.09E-58	postive
TRAJ39	AC025178.2	0.620259457	3.40E-58	postive
CREB1	AL137782.1	0.708346054	1.14E-82	postive
UBR1	AL137782.1	0.664800509	1.59E-69	postive
ZC3HAV1	AL137782.1	0.502888298	1.23E-35	postive
CYLD	AL137782.1	0.587948624	4.73E-51	postive
EIF2AK2	AL137782.1	0.528723154	7.32E-40	postive
IREB2	AL137782.1	0.558681427	3.06E-45	postive
DDX17	AL137782.1	0.578064616	5.05E-49	postive
JAK2	AL137782.1	0.617288015	1.67E-57	postive
TXK	AL137782.1	0.619889226	4.16E-58	postive
NFAT5	AL137782.1	0.772538144	3.29E-107	postive
PIK3R1	AL137782.1	0.573483235	4.17E-48	postive
PIK3CA	AL137782.1	0.608668017	1.55E-55	postive
PIK3CB	AL137782.1	0.505913619	4.11E-36	postive
ROBO2	AL137782.1	0.608186369	1.98E-55	postive
GNRH1	AL137782.1	0.714804257	7.89E-85	postive
IL6ST	AL137782.1	0.508342295	1.69E-36	postive
RABEP1	AL137782.1	0.507227768	2.55E-36	postive
ACVR2A	AL137782.1	0.568607566	3.80E-47	postive
ANGPTL1	AL137782.1	0.628999401	2.84E-60	postive
BMPR1A	AL137782.1	0.517337129	5.91E-38	postive
BMPR2	AL137782.1	0.67528651	1.75E-72	postive

CRLF3	AL137782.1	0.536584694	3.20E-41	postive
NR1D2	AL137782.1	0.555378486	1.28E-44	postive
NR2C2	AL137782.1	0.649038572	2.68E-65	postive
NR3C2	AL137782.1	0.512436759	3.72E-37	postive
RORA	AL137782.1	0.728534273	1.26E-89	postive
SOS1	AL137782.1	0.603321715	2.39E-54	postive
BRAF	AL137782.1	0.687973422	3.13E-76	postive
TEC	AL137782.1	0.52735219	1.25E-39	postive
CBL	AL137782.1	0.525352392	2.73E-39	postive
CBLB	AL137782.1	0.688253415	2.57E-76	postive
TRAJ1	AL137782.1	0.618545681	8.55E-58	postive
TRAJ2	AL137782.1	0.615356945	4.67E-57	postive
TRAJ3	AL137782.1	0.660373454	2.60E-68	postive
TRAJ5	AL137782.1	0.623033983	7.58E-59	postive
TRAJ6	AL137782.1	0.689122996	1.40E-76	postive
TRAJ8	AL137782.1	0.567239491	7.02E-47	postive
TRAJ10	AL137782.1	0.647368917	7.27E-65	postive
TRAJ12	AL137782.1	0.724791894	2.74E-88	postive
TRAJ13	AL137782.1	0.613389089	1.32E-56	postive
TRAJ14	AL137782.1	0.636039202	5.38E-62	postive
TRAJ16	AL137782.1	0.635265571	8.36E-62	postive
TRAJ17	AL137782.1	0.577723754	5.91E-49	postive
TRAJ18	AL137782.1	0.69525968	1.80E-78	postive
TRAJ21	AL137782.1	0.666021487	7.29E-70	postive
TRAJ31	AL137782.1	0.685696057	1.52E-75	postive
TRAJ37	AL137782.1	0.672027272	1.50E-71	postive
TRAJ38	AL137782.1	0.744038185	2.09E-95	postive
TRAJ39	AL137782.1	0.645732247	1.92E-64	postive
CREB1	AL157392.4	0.616384997	2.71E-57	postive
IL15	AL157392.4	0.501457094	2.06E-35	postive
CYLD	AL157392.4	0.565592326	1.46E-46	postive
DDX17	AL157392.4	0.666569449	5.13E-70	postive
JAK2	AL157392.4	0.547955289	2.98E-43	postive
TXK	AL157392.4	0.529610732	5.17E-40	postive
NFAT5	AL157392.4	0.769414526	7.82E-106	postive
PIK3R1	AL157392.4	0.541459244	4.41E-42	postive
PIK3CA	AL157392.4	0.538296276	1.60E-41	postive
GNRH1	AL157392.4	0.742068325	1.19E-94	postive
IL6ST	AL157392.4	0.572065633	7.96E-48	postive
ACVR2A	AL157392.4	0.543156189	2.19E-42	postive
ANGPTL1	AL157392.4	0.677139971	5.09E-73	postive
BMPR2	AL157392.4	0.571235264	1.16E-47	postive
CRLF3	AL157392.4	0.525522301	2.56E-39	postive
NR2C2	AL157392.4	0.6533099	2.03E-66	postive
RORA	AL157392.4	0.794155842	2.24E-117	postive
SOS1	AL157392.4	0.524812631	3.37E-39	postive
BRAF	AL157392.4	0.674804349	2.41E-72	postive
ITK	AL157392.4	0.506811823	2.96E-36	postive
CBLB	AL157392.4	0.628449791	3.86E-60	postive
TRAJ1	AL157392.4	0.645891223	1.75E-64	postive
TRAJ2	AL157392.4	0.676339027	8.68E-73	postive
TRAJ3	AL157392.4	0.702406647	9.77E-81	postive
TRAJ5	AL157392.4	0.698398263	1.85E-79	postive
TRAJ6	AL157392.4	0.691683773	2.30E-77	postive
TRAJ8	AL157392.4	0.655349235	5.84E-67	postive
TRAJ10	AL157392.4	0.699368733	9.13E-80	postive
TRAJ12	AL157392.4	0.582777332	5.56E-50	postive
TRAJ13	AL157392.4	0.691176659	3.30E-77	postive
TRAJ14	AL157392.4	0.663557003	3.50E-69	postive
TRAJ16	AL157392.4	0.71606833	2.93E-85	postive
TRAJ17	AL157392.4	0.664744668	1.65E-69	postive
TRAJ18	AL157392.4	0.667325543	3.16E-70	postive
TRAJ21	AL157392.4	0.716817807	1.63E-85	postive
TRAJ31	AL157392.4	0.738026099	4.08E-93	postive
TRAJ37	AL157392.4	0.732180988	6.00E-91	postive

TRAJ38	AL157392.4	0.759332287	1.55E-101	postive
TRAJ39	AL157392.4	0.73744862	6.72E-93	postive
CAMP	LINC01063	0.513652517	2.36E-37	postive
NPY	AC010654.1	0.583623041	3.72E-50	postive
CREB1	SP2-AS1	0.633874056	1.84E-61	postive
UBR1	SP2-AS1	0.508785877	1.44E-36	postive
LMBR1	SP2-AS1	0.580797702	1.41E-49	postive
NFAT5	SP2-AS1	0.649841234	1.66E-65	postive
PIK3CA	SP2-AS1	0.559383507	2.25E-45	postive
GNRH1	SP2-AS1	0.550033397	1.24E-43	postive
ANGPTL1	SP2-AS1	0.553397724	2.98E-44	postive
BMPR2	SP2-AS1	0.551754607	6.00E-44	postive
CRLF3	SP2-AS1	0.612861041	1.74E-56	postive
NR2C2	SP2-AS1	0.574579624	2.52E-48	postive
RORA	SP2-AS1	0.558415193	3.44E-45	postive
SOS1	SP2-AS1	0.566845403	8.38E-47	postive
BRAF	SP2-AS1	0.700312199	4.58E-80	postive
CBL	SP2-AS1	0.507763033	2.09E-36	postive
CBLB	SP2-AS1	0.521515841	1.20E-38	postive
TRAJ1	SP2-AS1	0.572297629	7.16E-48	postive
TRAJ2	SP2-AS1	0.593607679	3.03E-52	postive
TRAJ3	SP2-AS1	0.638197453	1.56E-62	postive
TRAJ5	SP2-AS1	0.605838177	6.63E-55	postive
TRAJ6	SP2-AS1	0.632444221	4.13E-61	postive
TRAJ8	SP2-AS1	0.637454367	2.39E-62	postive
TRAJ10	SP2-AS1	0.595156563	1.42E-52	postive
TRAJ12	SP2-AS1	0.559787529	1.89E-45	postive
TRAJ13	SP2-AS1	0.621460724	1.78E-58	postive
TRAJ14	SP2-AS1	0.619431759	5.32E-58	postive
TRAJ16	SP2-AS1	0.618495056	8.78E-58	postive
TRAJ17	SP2-AS1	0.585656923	1.42E-50	postive
TRAJ18	SP2-AS1	0.534028746	8.94E-41	postive
TRAJ21	SP2-AS1	0.63279595	3.39E-61	postive
TRAJ31	SP2-AS1	0.63412916	1.59E-61	postive
TRAJ37	SP2-AS1	0.617834822	1.25E-57	postive
TRAJ38	SP2-AS1	0.664314373	2.16E-69	postive
TRAJ39	SP2-AS1	0.651713883	5.36E-66	postive
TNFRSF14	AC016773.2	0.639394887	7.83E-63	postive
CREB1	AC090617.5	0.563114877	4.39E-46	postive
JAK2	AC090617.5	0.510704856	7.08E-37	postive
NFAT5	AC090617.5	0.573003219	5.19E-48	postive
PIK3CA	AC090617.5	0.543589695	1.83E-42	postive
GNRH1	AC090617.5	0.567376037	6.61E-47	postive
RABEP1	AC090617.5	0.502053745	1.66E-35	postive
ACVR2A	AC090617.5	0.500055612	3.41E-35	postive
ANGPTL1	AC090617.5	0.607731096	2.51E-55	postive
BMPR2	AC090617.5	0.510351485	8.07E-37	postive
CRLF3	AC090617.5	0.508337791	1.69E-36	postive
NR2C2	AC090617.5	0.550598394	9.79E-44	postive
RORA	AC090617.5	0.621868384	1.43E-58	postive
SOS1	AC090617.5	0.500560781	2.84E-35	postive
BRAF	AC090617.5	0.550954811	8.42E-44	postive
CBLB	AC090617.5	0.560626906	1.31E-45	postive
PDK1	AC090617.5	0.508273061	1.74E-36	postive
TRAJ1	AC090617.5	0.50117731	2.28E-35	postive
TRAJ3	AC090617.5	0.52048311	1.79E-38	postive
TRAJ5	AC090617.5	0.563374957	3.91E-46	postive
TRAJ6	AC090617.5	0.532056791	1.96E-40	postive
TRAJ8	AC090617.5	0.509055905	1.30E-36	postive
TRAJ10	AC090617.5	0.546298928	5.96E-43	postive
TRAJ13	AC090617.5	0.511182183	5.93E-37	postive
TRAJ14	AC090617.5	0.510909536	6.56E-37	postive
TRAJ16	AC090617.5	0.584421619	2.55E-50	postive
TRAJ17	AC090617.5	0.544174229	1.44E-42	postive
TRAJ21	AC090617.5	0.550009268	1.26E-43	postive

TRAJ31	AC090617.5	0.561301555	9.75E-46	postive
TRAJ37	AC090617.5	0.522403769	8.55E-39	postive
TRAJ38	AC090617.5	0.604350477	1.42E-54	postive
TRAJ39	AC090617.5	0.587118338	7.05E-51	postive
GNRH1	AC010326.3	0.534113551	8.64E-41	postive
CREB1	ALG13-AS1	0.794956076	8.91E-118	postive
RFXAP	ALG13-AS1	0.522848117	7.21E-39	postive
UBR1	ALG13-AS1	0.658193903	1.01E-67	postive
ZC3HAV1	ALG13-AS1	0.554471141	1.88E-44	postive
ZC3HAV1L	ALG13-AS1	0.505279518	5.18E-36	postive
IL15	ALG13-AS1	0.588595853	3.47E-51	postive
CYLD	ALG13-AS1	0.624146927	4.13E-59	postive
EIF2AK2	ALG13-AS1	0.556685517	7.27E-45	postive
MAPK8	ALG13-AS1	0.553908791	2.40E-44	postive
NFKBIZ	ALG13-AS1	0.523237732	6.20E-39	postive
LMBR1	ALG13-AS1	0.562876743	4.88E-46	postive
IREB2	ALG13-AS1	0.621670284	1.59E-58	postive
DDX17	ALG13-AS1	0.646054932	1.59E-64	postive
PIK3CG	ALG13-AS1	0.523503617	5.60E-39	postive
JAK2	ALG13-AS1	0.664703544	1.69E-69	postive
TXK	ALG13-AS1	0.604065659	1.64E-54	postive
NFAT5	ALG13-AS1	0.852473265	2.93E-152	postive
MALT1	ALG13-AS1	0.537174339	2.52E-41	postive
PIK3R1	ALG13-AS1	0.646260327	1.41E-64	postive
PIK3CA	ALG13-AS1	0.692270743	1.52E-77	postive
PIK3CB	ALG13-AS1	0.536194031	3.75E-41	postive
RASGRP3	ALG13-AS1	0.559653935	2.00E-45	postive
LTB4R2	ALG13-AS1	0.525064474	3.06E-39	postive
PLXNC1	ALG13-AS1	0.517788899	4.98E-38	postive
GNRH1	ALG13-AS1	0.785716074	2.88E-113	postive
IL6ST	ALG13-AS1	0.623424616	6.12E-59	postive
RABEP1	ALG13-AS1	0.565971916	1.24E-46	postive
ACVR2A	ALG13-AS1	0.667568634	2.70E-70	postive
ANGPTL1	ALG13-AS1	0.739652583	9.93E-94	postive
BMPR1A	ALG13-AS1	0.551121058	7.85E-44	postive
BMPR2	ALG13-AS1	0.741817124	1.49E-94	postive
CRLF3	ALG13-AS1	0.625124043	2.42E-59	postive
NR1D2	ALG13-AS1	0.545955753	6.87E-43	postive
NR2C1	ALG13-AS1	0.508629471	1.52E-36	postive
NR2C2	ALG13-AS1	0.748980454	2.44E-97	postive
RORA	ALG13-AS1	0.849556046	3.57E-150	postive
SOS1	ALG13-AS1	0.698270096	2.04E-79	postive
SOS2	ALG13-AS1	0.595991761	9.38E-53	postive
BRAF	ALG13-AS1	0.78228647	1.19E-111	postive
TEC	ALG13-AS1	0.516549805	7.96E-38	postive
CBL	ALG13-AS1	0.582190757	7.33E-50	postive
CBLB	ALG13-AS1	0.747188655	1.24E-96	postive
RASGRP1	ALG13-AS1	0.520719112	1.63E-38	postive
PDK1	ALG13-AS1	0.57477008	2.31E-48	postive
TRAJ1	ALG13-AS1	0.738251948	3.35E-93	postive
TRAJ2	ALG13-AS1	0.707383674	2.36E-82	postive
TRAJ3	ALG13-AS1	0.767045054	8.36E-105	postive
TRAJ5	ALG13-AS1	0.785291445	4.58E-113	postive
TRAJ6	ALG13-AS1	0.784499833	1.09E-112	postive
TRAJ8	ALG13-AS1	0.720194653	1.12E-86	postive
TRAJ10	ALG13-AS1	0.776252471	7.10E-109	postive
TRAJ12	ALG13-AS1	0.742695198	6.87E-95	postive
TRAJ13	ALG13-AS1	0.744352214	1.58E-95	postive
TRAJ14	ALG13-AS1	0.740623928	4.25E-94	postive
TRAJ16	ALG13-AS1	0.775074766	2.41E-108	postive
TRAJ17	ALG13-AS1	0.732277657	5.53E-91	postive
TRAJ18	ALG13-AS1	0.744684993	1.17E-95	postive
TRAJ21	ALG13-AS1	0.77825077	8.75E-110	postive
TRAJ31	ALG13-AS1	0.826709545	3.08E-135	postive
TRAJ37	ALG13-AS1	0.798014982	2.55E-119	postive

TRAJ38	ALG13-AS1	0.855360631	2.28E-154	postive
TRAJ39	ALG13-AS1	0.79731064	5.80E-119	postive
NFYC	AL050341.2	0.529993761	4.44E-40	postive
NR2C2	AL035587.1	0.541386591	4.54E-42	postive
DDX17	MED8-AS1	0.564317364	2.58E-46	postive
NFAT5	MED8-AS1	0.523663794	5.26E-39	postive
GNRH1	MED8-AS1	0.630710761	1.09E-60	postive
NR2C1	MED8-AS1	0.584944838	1.99E-50	postive
NR2C2	MED8-AS1	0.596533299	7.18E-53	postive
RORA	MED8-AS1	0.505674968	4.49E-36	postive
BRAF	MED8-AS1	0.571515464	1.02E-47	postive
CBLB	MED8-AS1	0.563665885	3.44E-46	postive
TRAJ3	MED8-AS1	0.524606678	3.65E-39	postive
TRAJ6	MED8-AS1	0.500370462	3.04E-35	postive
TRAJ14	MED8-AS1	0.503524479	9.79E-36	postive
TRAJ21	MED8-AS1	0.507390484	2.40E-36	postive
TRAJ38	MED8-AS1	0.524224268	4.24E-39	postive
CREB1	AC092953.2	0.625480339	1.99E-59	postive
SP1	AC092953.2	0.521882778	1.05E-38	postive
DDX17	AC092953.2	0.535028965	5.98E-41	postive
TXK	AC092953.2	0.518574675	3.70E-38	postive
NFAT5	AC092953.2	0.584056286	3.03E-50	postive
PIK3CA	AC092953.2	0.631216943	8.24E-61	postive
GNRH1	AC092953.2	0.648248562	4.30E-65	postive
ACVR2A	AC092953.2	0.583815126	3.40E-50	postive
ANGPTL1	AC092953.2	0.556498975	7.87E-45	postive
BMPR2	AC092953.2	0.627093217	8.18E-60	postive
NR2C2	AC092953.2	0.597183366	5.20E-53	postive
RORA	AC092953.2	0.618129912	1.07E-57	postive
SOS1	AC092953.2	0.598608348	2.56E-53	postive
BRAF	AC092953.2	0.563305486	4.04E-46	postive
CBLB	AC092953.2	0.635850362	5.99E-62	postive
TRAJ5	AC092953.2	0.508749022	1.46E-36	postive
TRAJ16	AC092953.2	0.504434329	7.04E-36	postive
TRAJ31	AC092953.2	0.538363854	1.56E-41	postive
TRAJ38	AC092953.2	0.560706627	1.27E-45	postive
GCG	LINC00239	0.600515284	9.85E-54	postive
CREB1	LINC00641	0.693390365	6.85E-78	postive
RFXAP	LINC00641	0.568274849	4.41E-47	postive
UBR1	LINC00641	0.53411184	8.64E-41	postive
ZC3HAV1	LINC00641	0.547775462	3.21E-43	postive
ZC3HAV1L	LINC00641	0.513435106	2.56E-37	postive
IL15	LINC00641	0.514810271	1.53E-37	postive
CYLD	LINC00641	0.521515733	1.20E-38	postive
MAPK8	LINC00641	0.525784991	2.31E-39	postive
IREB2	LINC00641	0.557940214	4.22E-45	postive
DDX17	LINC00641	0.638583378	1.25E-62	postive
JAK2	LINC00641	0.512012308	4.36E-37	postive
TXK	LINC00641	0.544003926	1.54E-42	postive
NFAT5	LINC00641	0.710877866	1.65E-83	postive
PIK3R1	LINC00641	0.562722473	5.22E-46	postive
PIK3CA	LINC00641	0.588233146	4.13E-51	postive
AKT3	LINC00641	0.532703439	1.52E-40	postive
LTB4R2	LINC00641	0.579289814	2.85E-49	postive
GNRH1	LINC00641	0.692865785	9.95E-78	postive
IL6ST	LINC00641	0.539231778	1.09E-41	postive
RABEP1	LINC00641	0.533710263	1.01E-40	postive
ACVR2A	LINC00641	0.609629718	9.40E-56	postive
ANGPTL1	LINC00641	0.662625191	6.31E-69	postive
BMPR1A	LINC00641	0.500473569	2.93E-35	postive
BMPR2	LINC00641	0.602907165	2.95E-54	postive
CRLF3	LINC00641	0.573031732	5.12E-48	postive
NR2C2	LINC00641	0.745499952	5.66E-96	postive
RORA	LINC00641	0.690943632	3.89E-77	postive
SOS1	LINC00641	0.681641858	2.45E-74	postive

SOS2	LINC00641	0.598158986	3.20E-53	postive
BRAF	LINC00641	0.762338822	8.54E-103	postive
CBL	LINC00641	0.607076185	3.51E-55	postive
CBLB	LINC00641	0.626317612	1.25E-59	postive
PDK1	LINC00641	0.504641173	6.53E-36	postive
TRAJ1	LINC00641	0.740951134	3.19E-94	postive
TRAJ2	LINC00641	0.687591213	4.08E-76	postive
TRAJ3	LINC00641	0.768685005	1.63E-105	postive
TRAJ5	LINC00641	0.765413179	4.21E-104	postive
TRAJ6	LINC00641	0.739737364	9.22E-94	postive
TRAJ8	LINC00641	0.783913942	2.05E-112	postive
TRAJ10	LINC00641	0.702831754	7.13E-81	postive
TRAJ12	LINC00641	0.685753819	1.46E-75	postive
TRAJ13	LINC00641	0.760141793	7.13E-102	postive
TRAJ14	LINC00641	0.76524522	4.97E-104	postive
TRAJ16	LINC00641	0.742850299	5.99E-95	postive
TRAJ17	LINC00641	0.744900362	9.68E-96	postive
TRAJ18	LINC00641	0.646517793	1.21E-64	postive
TRAJ21	LINC00641	0.766948953	9.20E-105	postive
TRAJ31	LINC00641	0.755301906	7.07E-100	postive
TRAJ37	LINC00641	0.734119101	1.16E-91	postive
TRAJ38	LINC00641	0.774419362	4.76E-108	postive
TRAJ39	LINC00641	0.783204199	4.43E-112	postive
CREB1	AC018638.7	0.657967929	1.16E-67	postive
RFXAP	AC018638.7	0.535345072	5.27E-41	postive
UBR1	AC018638.7	0.533904357	9.39E-41	postive
ZC3HAV1	AC018638.7	0.509807477	9.86E-37	postive
CYLD	AC018638.7	0.598822389	2.30E-53	postive
DDX17	AC018638.7	0.679796834	8.55E-74	postive
JAK2	AC018638.7	0.544158451	1.45E-42	postive
TXK	AC018638.7	0.523643607	5.30E-39	postive
NFAT5	AC018638.7	0.840202378	8.95E-144	postive
NFATC3	AC018638.7	0.509342865	1.17E-36	postive
PIK3R1	AC018638.7	0.556462767	8.00E-45	postive
PIK3CA	AC018638.7	0.55112882	7.82E-44	postive
IGHD6-6	AC018638.7	0.507584122	2.23E-36	postive
GNRH1	AC018638.7	0.783022276	5.40E-112	postive
IL6ST	AC018638.7	0.559038481	2.62E-45	postive
ACVR2A	AC018638.7	0.601269708	6.74E-54	postive
ANGPTL1	AC018638.7	0.627134894	7.99E-60	postive
BMPR2	AC018638.7	0.607273935	3.17E-55	postive
CRLF3	AC018638.7	0.537442005	2.26E-41	postive
NR2C1	AC018638.7	0.564666237	2.21E-46	postive
NR2C2	AC018638.7	0.68608934	1.16E-75	postive
RORA	AC018638.7	0.806722904	7.24E-124	postive
SOS1	AC018638.7	0.581944013	8.23E-50	postive
SOS2	AC018638.7	0.519215264	2.90E-38	postive
BRAF	AC018638.7	0.723322262	9.04E-88	postive
CBL	AC018638.7	0.536009867	4.03E-41	postive
CBLB	AC018638.7	0.583663354	3.65E-50	postive
TRAJ1	AC018638.7	0.736792451	1.18E-92	postive
TRAJ2	AC018638.7	0.71183525	7.89E-84	postive
TRAJ3	AC018638.7	0.769269379	9.04E-106	postive
TRAJ5	AC018638.7	0.759353379	1.52E-101	postive
TRAJ6	AC018638.7	0.77802265	1.11E-109	postive
TRAJ8	AC018638.7	0.749509275	1.51E-97	postive
TRAJ10	AC018638.7	0.765022489	6.19E-104	postive
TRAJ12	AC018638.7	0.693442136	6.60E-78	postive
TRAJ13	AC018638.7	0.778550008	6.38E-110	postive
TRAJ14	AC018638.7	0.738907848	1.90E-93	postive
TRAJ16	AC018638.7	0.780089324	1.25E-110	postive
TRAJ17	AC018638.7	0.745153675	7.72E-96	postive
TRAJ18	AC018638.7	0.736882118	1.09E-92	postive
TRAJ21	AC018638.7	0.79498348	8.64E-118	postive
TRAJ31	AC018638.7	0.805128661	5.12E-123	postive

TRAJ37	AC018638.7	0.816465962	3.09E-129	postive
TRAJ38	AC018638.7	0.798655742	1.20E-119	postive
TRAJ39	AC018638.7	0.799407014	4.95E-120	postive
ACVR2B	AC012073.1	0.514264009	1.88E-37	postive
NR6A1	AC012073.1	0.526050083	2.08E-39	postive
DEFB1	ITGB1-DT	0.613082359	1.55E-56	postive
BPIFB1	ACBD3-AS1	0.510526648	7.56E-37	postive
DES	ACBD3-AS1	0.524299938	4.11E-39	postive
NFAT5	ACBD3-AS1	0.513266675	2.73E-37	postive
RORA	ACBD3-AS1	0.517401061	5.77E-38	postive
TRAJ38	ACBD3-AS1	0.518898039	3.27E-38	postive
CREB1	NUTM2A-AS1	0.629393612	2.28E-60	postive
UBR1	NUTM2A-AS1	0.549184795	1.78E-43	postive
MAPK8	NUTM2A-AS1	0.634962357	9.94E-62	postive
IREB2	NUTM2A-AS1	0.60144241	6.18E-54	postive
PPP3CB	NUTM2A-AS1	0.609690007	9.11E-56	postive
NFAT5	NUTM2A-AS1	0.636784293	3.51E-62	postive
CHUK	NUTM2A-AS1	0.563763847	3.30E-46	postive
PIK3CA	NUTM2A-AS1	0.524237412	4.21E-39	postive
GNRH1	NUTM2A-AS1	0.507021471	2.74E-36	postive
ACVR2A	NUTM2A-AS1	0.525805553	2.29E-39	postive
ANGPTL1	NUTM2A-AS1	0.570844093	1.39E-47	postive
BMPR1A	NUTM2A-AS1	0.641794282	1.94E-63	postive
BMPR2	NUTM2A-AS1	0.572248496	7.32E-48	postive
CRLF3	NUTM2A-AS1	0.517590677	5.37E-38	postive
RORA	NUTM2A-AS1	0.596702181	6.60E-53	postive
SOS1	NUTM2A-AS1	0.592009576	6.63E-52	postive
BRAF	NUTM2A-AS1	0.582206079	7.27E-50	postive
TRAJ1	NUTM2A-AS1	0.539516509	9.75E-42	postive
TRAJ2	NUTM2A-AS1	0.576104945	1.25E-48	postive
TRAJ3	NUTM2A-AS1	0.597033515	5.60E-53	postive
TRAJ5	NUTM2A-AS1	0.571083472	1.24E-47	postive
TRAJ6	NUTM2A-AS1	0.607277572	3.17E-55	postive
TRAJ8	NUTM2A-AS1	0.595776981	1.04E-52	postive
TRAJ10	NUTM2A-AS1	0.593013911	4.06E-52	postive
TRAJ12	NUTM2A-AS1	0.537948464	1.84E-41	postive
TRAJ13	NUTM2A-AS1	0.609559702	9.74E-56	postive
TRAJ14	NUTM2A-AS1	0.586680388	8.69E-51	postive
TRAJ16	NUTM2A-AS1	0.567303143	6.83E-47	postive
TRAJ17	NUTM2A-AS1	0.555025085	1.48E-44	postive
TRAJ18	NUTM2A-AS1	0.52243097	8.46E-39	postive
TRAJ21	NUTM2A-AS1	0.607957445	2.23E-55	postive
TRAJ31	NUTM2A-AS1	0.62233121	1.11E-58	postive
TRAJ37	NUTM2A-AS1	0.614723457	6.53E-57	postive
TRAJ38	NUTM2A-AS1	0.651935247	4.68E-66	postive
TRAJ39	NUTM2A-AS1	0.602790505	3.13E-54	postive
CREB1	AC138956.2	0.619394433	5.42E-58	postive
RFXAP	AC138956.2	0.525095119	3.02E-39	postive
UBR1	AC138956.2	0.523286009	6.09E-39	postive
CYLD	AC138956.2	0.535798913	4.39E-41	postive
NFKBIZ	AC138956.2	0.538922571	1.24E-41	postive
DDX17	AC138956.2	0.701222135	2.34E-80	postive
JAK2	AC138956.2	0.512862538	3.18E-37	postive
TXK	AC138956.2	0.509531273	1.09E-36	postive
NFAT5	AC138956.2	0.725728935	1.27E-88	postive
PIK3CA	AC138956.2	0.506375411	3.48E-36	postive
LTB4R2	AC138956.2	0.604425961	1.36E-54	postive
GNRH1	AC138956.2	0.773922366	7.94E-108	postive
IL6ST	AC138956.2	0.51161253	5.06E-37	postive
ACVR2A	AC138956.2	0.50198642	1.70E-35	postive
ANGPTL1	AC138956.2	0.571784873	9.04E-48	postive
BMPR2	AC138956.2	0.510941168	6.49E-37	postive
NR2C1	AC138956.2	0.553383158	3.00E-44	postive
NR2C2	AC138956.2	0.758149798	4.79E-101	postive
RORA	AC138956.2	0.682837661	1.08E-74	postive

SOS1	AC138956.2	0.57226036	7.28E-48	postive
BRAF	AC138956.2	0.645505558	2.20E-64	postive
CBL	AC138956.2	0.533285033	1.20E-40	postive
CBLB	AC138956.2	0.569247922	2.85E-47	postive
TRAJ1	AC138956.2	0.641083639	2.94E-63	postive
TRAJ2	AC138956.2	0.588807019	3.13E-51	postive
TRAJ3	AC138956.2	0.675649097	1.37E-72	postive
TRAJ5	AC138956.2	0.621352924	1.89E-58	postive
TRAJ6	AC138956.2	0.645145207	2.72E-64	postive
TRAJ8	AC138956.2	0.583730009	3.54E-50	postive
TRAJ10	AC138956.2	0.644567857	3.82E-64	postive
TRAJ12	AC138956.2	0.549843015	1.35E-43	postive
TRAJ13	AC138956.2	0.59450324	1.95E-52	postive
TRAJ14	AC138956.2	0.586318108	1.03E-50	postive
TRAJ16	AC138956.2	0.634493551	1.30E-61	postive
TRAJ17	AC138956.2	0.60420966	1.52E-54	postive
TRAJ18	AC138956.2	0.597131982	5.33E-53	postive
TRAJ21	AC138956.2	0.624868703	2.78E-59	postive
TRAJ31	AC138956.2	0.624374039	3.65E-59	postive
TRAJ37	AC138956.2	0.642331716	1.42E-63	postive
TRAJ38	AC138956.2	0.654537817	9.60E-67	postive
TRAJ39	AC138956.2	0.649960613	1.54E-65	postive
CREB1	AC007552.2	0.671227428	2.52E-71	postive
UBR1	AC007552.2	0.58614804	1.12E-50	postive
IL15	AC007552.2	0.528924741	6.77E-40	postive
CYLD	AC007552.2	0.525748519	2.34E-39	postive
IREB2	AC007552.2	0.556239486	8.81E-45	postive
DDX17	AC007552.2	0.529262504	5.92E-40	postive
NFAT5	AC007552.2	0.752649455	8.39E-99	postive
NFATC3	AC007552.2	0.508720371	1.47E-36	postive
PIK3R1	AC007552.2	0.598956479	2.15E-53	postive
PIK3CA	AC007552.2	0.551525367	6.61E-44	postive
IGHD4-4	AC007552.2	0.589143046	2.66E-51	postive
GNRH1	AC007552.2	0.631515256	6.97E-61	postive
IL6ST	AC007552.2	0.595699833	1.08E-52	postive
ACVR2A	AC007552.2	0.600594212	9.47E-54	postive
ANGPTL1	AC007552.2	0.602588304	3.46E-54	postive
BMPR1A	AC007552.2	0.525278504	2.81E-39	postive
BMPR2	AC007552.2	0.626811173	9.56E-60	postive
CRLF3	AC007552.2	0.508335493	1.70E-36	postive
NR2C2	AC007552.2	0.648335235	4.09E-65	postive
RORA	AC007552.2	0.754501628	1.50E-99	postive
SOS1	AC007552.2	0.588270731	4.05E-51	postive
SOS2	AC007552.2	0.52351544	5.57E-39	postive
BRAF	AC007552.2	0.722442298	1.84E-87	postive
CBLB	AC007552.2	0.520798985	1.58E-38	postive
TRAJ1	AC007552.2	0.681392395	2.90E-74	postive
TRAJ2	AC007552.2	0.647853325	5.45E-65	postive
TRAJ3	AC007552.2	0.748358473	4.30E-97	postive
TRAJ5	AC007552.2	0.751158654	3.32E-98	postive
TRAJ6	AC007552.2	0.727904345	2.13E-89	postive
TRAJ8	AC007552.2	0.724402488	3.76E-88	postive
TRAJ10	AC007552.2	0.733975411	1.31E-91	postive
TRAJ12	AC007552.2	0.722921731	1.25E-87	postive
TRAJ13	AC007552.2	0.722021184	2.58E-87	postive
TRAJ14	AC007552.2	0.718504215	4.30E-86	postive
TRAJ16	AC007552.2	0.692164062	1.64E-77	postive
TRAJ17	AC007552.2	0.729567836	5.35E-90	postive
TRAJ18	AC007552.2	0.695440684	1.58E-78	postive
TRAJ21	AC007552.2	0.753132474	5.36E-99	postive
TRAJ31	AC007552.2	0.785459375	3.81E-113	postive
TRAJ37	AC007552.2	0.791221133	6.31E-116	postive
TRAJ38	AC007552.2	0.767882526	3.63E-105	postive
TRAJ39	AC007552.2	0.784801024	7.83E-113	postive
CREB1	AC016727.1	0.541955413	3.59E-42	postive

DDX17	AC016727.1	0.554564125	1.81E-44	postive
NFAT5	AC016727.1	0.589952116	1.80E-51	postive
GNRH1	AC016727.1	0.617449495	1.54E-57	postive
ANGPTL1	AC016727.1	0.521884498	1.04E-38	postive
NR2C2	AC016727.1	0.59539887	1.26E-52	postive
RORA	AC016727.1	0.572927712	5.37E-48	postive
SOS1	AC016727.1	0.517754126	5.05E-38	postive
BRAF	AC016727.1	0.58820547	4.18E-51	postive
CBLB	AC016727.1	0.503679791	9.25E-36	postive
TRAJ5	AC016727.1	0.518525837	3.77E-38	postive
TRAJ6	AC016727.1	0.511470571	5.33E-37	postive
TRAJ13	AC016727.1	0.509535746	1.09E-36	postive
TRAJ21	AC016727.1	0.520544194	1.75E-38	postive
TRAJ31	AC016727.1	0.529313901	5.81E-40	postive
TRAJ37	AC016727.1	0.520359951	1.87E-38	postive
TRAJ38	AC016727.1	0.570256546	1.81E-47	postive
TRAJ39	AC016727.1	0.51997611	2.17E-38	postive
LMBR1L	AC114730.3	0.519178874	2.94E-38	postive
IRF3	AC114730.3	0.512377482	3.80E-37	postive
TYK2	AC114730.3	0.582170307	7.40E-50	postive
IRF9	AC114730.3	0.598222406	3.10E-53	postive
EPOR	AC114730.3	0.613364436	1.34E-56	postive
GIPR	AC114730.3	0.528075419	9.45E-40	postive
IL11RA	AC114730.3	0.511432717	5.40E-37	postive
NR3C2	KIAA1671-AS1	0.536956353	2.75E-41	postive
CTF1	CD2BP2-DT	0.521039455	1.44E-38	postive
CREB1	AC139887.1	0.607420715	2.94E-55	postive
UBR1	AC139887.1	0.576858695	8.83E-49	postive
DDX17	AC139887.1	0.62777943	5.60E-60	postive
NFAT5	AC139887.1	0.755569312	5.50E-100	postive
NFATC3	AC139887.1	0.518071639	4.48E-38	postive
IGHD4-4	AC139887.1	0.52285819	7.18E-39	postive
GNRH1	AC139887.1	0.697962952	2.54E-79	postive
ACVR2A	AC139887.1	0.570583263	1.56E-47	postive
ANGPTL1	AC139887.1	0.517880419	4.81E-38	postive
BMPR2	AC139887.1	0.50577687	4.32E-36	postive
NR2C2	AC139887.1	0.69084099	4.18E-77	postive
RORA	AC139887.1	0.647774858	5.71E-65	postive
SOS1	AC139887.1	0.555817964	1.06E-44	postive
SOS2	AC139887.1	0.516965772	6.81E-38	postive
BRAF	AC139887.1	0.641824499	1.91E-63	postive
TRAJ1	AC139887.1	0.693332433	7.13E-78	postive
TRAJ2	AC139887.1	0.651369045	6.60E-66	postive
TRAJ3	AC139887.1	0.753764596	2.98E-99	postive
TRAJ5	AC139887.1	0.647987106	5.03E-65	postive
TRAJ6	AC139887.1	0.730368148	2.75E-90	postive
TRAJ8	AC139887.1	0.717876212	7.06E-86	postive
TRAJ10	AC139887.1	0.715968851	3.17E-85	postive
TRAJ12	AC139887.1	0.67214183	1.39E-71	postive
TRAJ13	AC139887.1	0.684476963	3.52E-75	postive
TRAJ14	AC139887.1	0.69122192	3.19E-77	postive
TRAJ16	AC139887.1	0.694225696	3.77E-78	postive
TRAJ17	AC139887.1	0.700015647	5.69E-80	postive
TRAJ18	AC139887.1	0.654950795	7.46E-67	postive
TRAJ21	AC139887.1	0.703519051	4.28E-81	postive
TRAJ31	AC139887.1	0.700095308	5.36E-80	postive
TRAJ37	AC139887.1	0.73309166	2.78E-91	postive
TRAJ38	AC139887.1	0.718876241	3.20E-86	postive
TRAJ39	AC139887.1	0.728232824	1.62E-89	postive
CREB1	AC087286.2	0.776467436	5.68E-109	postive
UBR1	AC087286.2	0.671653506	1.91E-71	postive
ZC3HAV1	AC087286.2	0.549964877	1.28E-43	postive
ZC3HAV1L	AC087286.2	0.524755875	3.45E-39	postive
IL15	AC087286.2	0.545838096	7.22E-43	postive
CYLD	AC087286.2	0.547350569	3.84E-43	postive

EIF2AK2	AC087286.2	0.554890358	1.57E-44	postive
MAPK8	AC087286.2	0.570513347	1.61E-47	postive
PDGFRA	AC087286.2	0.505128925	5.47E-36	postive
LMBR1	AC087286.2	0.587395545	6.17E-51	postive
LIMS1	AC087286.2	0.549607233	1.49E-43	postive
IREB2	AC087286.2	0.636303349	4.63E-62	postive
DDX17	AC087286.2	0.579230811	2.93E-49	postive
PIK3CG	AC087286.2	0.533219605	1.23E-40	postive
JAK2	AC087286.2	0.639300399	8.27E-63	postive
TXK	AC087286.2	0.649980752	1.52E-65	postive
NFAT5	AC087286.2	0.746220658	2.96E-96	postive
MALT1	AC087286.2	0.530089081	4.28E-40	postive
PIK3R1	AC087286.2	0.640825667	3.42E-63	postive
PIK3CA	AC087286.2	0.702069734	1.25E-80	postive
PIK3CB	AC087286.2	0.550903641	8.61E-44	postive
AKT3	AC087286.2	0.514688208	1.60E-37	postive
RASGRP3	AC087286.2	0.521691368	1.12E-38	postive
ROBO2	AC087286.2	0.51551612	1.18E-37	postive
GNRH1	AC087286.2	0.764471239	1.06E-103	postive
IL6ST	AC087286.2	0.602304104	4.00E-54	postive
RABEP1	AC087286.2	0.566928449	8.07E-47	postive
ACVR2A	AC087286.2	0.618806722	7.43E-58	postive
ANGPTL1	AC087286.2	0.770880576	1.78E-106	postive
BMPR1A	AC087286.2	0.563694697	3.40E-46	postive
BMPR2	AC087286.2	0.773023737	2.00E-107	postive
CRLF3	AC087286.2	0.622668514	9.24E-59	postive
NR1D2	AC087286.2	0.528380189	8.38E-40	postive
NR2C2	AC087286.2	0.680162227	6.68E-74	postive
RORA	AC087286.2	0.789414207	4.81E-115	postive
SOS1	AC087286.2	0.665968272	7.54E-70	postive
SOS2	AC087286.2	0.5413964	4.52E-42	postive
BRAF	AC087286.2	0.77256216	3.21E-107	postive
TEC	AC087286.2	0.55102611	8.17E-44	postive
CBL	AC087286.2	0.581868356	8.53E-50	postive
CBLB	AC087286.2	0.762610537	6.56E-103	postive
PDK1	AC087286.2	0.623367078	6.32E-59	postive
TRAJ1	AC087286.2	0.571233932	1.16E-47	postive
TRAJ2	AC087286.2	0.581133265	1.20E-49	postive
TRAJ3	AC087286.2	0.63623068	4.82E-62	postive
TRAJ5	AC087286.2	0.649006108	2.74E-65	postive
TRAJ6	AC087286.2	0.636253011	4.76E-62	postive
TRAJ8	AC087286.2	0.5772238	7.45E-49	postive
TRAJ10	AC087286.2	0.602647894	3.36E-54	postive
TRAJ12	AC087286.2	0.569829954	2.19E-47	postive
TRAJ13	AC087286.2	0.604052796	1.65E-54	postive
TRAJ14	AC087286.2	0.588928125	2.95E-51	postive
TRAJ16	AC087286.2	0.61582961	3.64E-57	postive
TRAJ17	AC087286.2	0.569053802	3.11E-47	postive
TRAJ18	AC087286.2	0.571999069	8.20E-48	postive
TRAJ21	AC087286.2	0.642348081	1.41E-63	postive
TRAJ31	AC087286.2	0.681026427	3.72E-74	postive
TRAJ37	AC087286.2	0.630075202	1.56E-60	postive
TRAJ38	AC087286.2	0.72829923	1.53E-89	postive
TRAJ39	AC087286.2	0.66823651	1.76E-70	postive
LTA	AC004687.1	0.514179476	1.94E-37	postive
CIITA	AC004687.1	0.598546763	2.64E-53	postive
IFI30	AC004687.1	0.598116802	3.27E-53	postive
PIK3R5	AC004687.1	0.529352578	5.72E-40	postive
PIK3CD	AC004687.1	0.548389323	2.48E-43	postive
PTPN6	AC004687.1	0.507071178	2.70E-36	postive
FLT3LG	AC004687.1	0.52264817	7.79E-39	postive
IL16	AC004687.1	0.511072812	6.18E-37	postive
IL10RA	AC004687.1	0.529308097	5.82E-40	postive
IL12RB1	AC004687.1	0.541192176	4.92E-42	postive
TNFRSF14	AC004687.1	0.534855389	6.42E-41	postive

ITGAL	AC004687.1	0.614589758	7.01E-57	postive
CD247	AC004687.1	0.534723502	6.77E-41	postive
ZAP70	AC004687.1	0.694256049	3.69E-78	postive
TRBC1	AC004687.1	0.532739277	1.50E-40	postive
TRBJ2-7	AC004687.1	0.565859194	1.30E-46	postive
AKT1	AL928654.1	0.506042273	3.92E-36	postive
CREB1	AC090948.2	0.805486858	3.30E-123	postive
RFXAP	AC090948.2	0.546089978	6.50E-43	postive
UBR1	AC090948.2	0.670242772	4.79E-71	postive
ZC3HAV1	AC090948.2	0.611288874	3.96E-56	postive
ZC3HAV1L	AC090948.2	0.517839746	4.89E-38	postive
IL15	AC090948.2	0.674073525	3.90E-72	postive
CYLD	AC090948.2	0.693530934	6.19E-78	postive
EIF2AK2	AC090948.2	0.587450515	6.01E-51	postive
MAPK8	AC090948.2	0.562491143	5.78E-46	postive
TLR1	AC090948.2	0.526290483	1.90E-39	postive
LMBR1	AC090948.2	0.580960646	1.31E-49	postive
LIMS1	AC090948.2	0.536335434	3.54E-41	postive
IREB2	AC090948.2	0.632982899	3.05E-61	postive
DDX17	AC090948.2	0.635403461	7.73E-62	postive
PIK3CG	AC090948.2	0.631987679	5.34E-61	postive
JAK2	AC090948.2	0.721128639	5.30E-87	postive
TXK	AC090948.2	0.623468034	5.98E-59	postive
NFAT5	AC090948.2	0.86525873	5.82E-162	postive
NFATC3	AC090948.2	0.512873139	3.16E-37	postive
MALT1	AC090948.2	0.572896572	5.45E-48	postive
PIK3R1	AC090948.2	0.690031641	7.39E-77	postive
PIK3CA	AC090948.2	0.725963109	1.05E-88	postive
PIK3CB	AC090948.2	0.575666586	1.53E-48	postive
AKT3	AC090948.2	0.557817029	4.45E-45	postive
RASGRP3	AC090948.2	0.611782143	3.06E-56	postive
IGHD6-6	AC090948.2	0.556492331	7.90E-45	postive
PLXNC1	AC090948.2	0.599184572	1.92E-53	postive
GNRH1	AC090948.2	0.72491992	2.47E-88	postive
IL6ST	AC090948.2	0.64552563	2.17E-64	postive
IL7	AC090948.2	0.513388132	2.61E-37	postive
RABEP1	AC090948.2	0.593454406	3.27E-52	postive
ACVR2A	AC090948.2	0.637428124	2.43E-62	postive
ANGPTL1	AC090948.2	0.797600821	4.14E-119	postive
BMPR1A	AC090948.2	0.534389851	7.73E-41	postive
BMPR2	AC090948.2	0.731623107	9.59E-91	postive
CRLF3	AC090948.2	0.670184182	4.98E-71	postive
LIFR	AC090948.2	0.519965091	2.18E-38	postive
NR1D2	AC090948.2	0.533749906	9.99E-41	postive
NR2C2	AC090948.2	0.752112529	1.38E-98	postive
NR3C1	AC090948.2	0.513432876	2.57E-37	postive
RORA	AC090948.2	0.8534698	5.55E-153	postive
SOS1	AC090948.2	0.729713803	4.74E-90	postive
SOS2	AC090948.2	0.620413346	3.13E-58	postive
BRAF	AC090948.2	0.828857826	1.51E-136	postive
PTPRC	AC090948.2	0.550318536	1.10E-43	postive
ITK	AC090948.2	0.620002417	3.91E-58	postive
TEC	AC090948.2	0.506213527	3.69E-36	postive
CD28	AC090948.2	0.578509783	4.10E-49	postive
CBL	AC090948.2	0.660724291	2.08E-68	postive
CBLB	AC090948.2	0.758196959	4.58E-101	postive
RASGRP1	AC090948.2	0.577144529	7.73E-49	postive
PDK1	AC090948.2	0.629008363	2.83E-60	postive
TRAJ1	AC090948.2	0.819581081	5.08E-131	postive
TRAJ2	AC090948.2	0.806510331	9.41E-124	postive
TRAJ3	AC090948.2	0.884054606	3.37E-178	postive
TRAJ5	AC090948.2	0.8442252	1.78E-146	postive
TRAJ6	AC090948.2	0.864912805	1.10E-161	postive
TRAJ8	AC090948.2	0.817862972	4.95E-130	postive
TRAJ10	AC090948.2	0.842632498	2.14E-145	postive

TRAJ12	AC090948.2	0.806426164	1.04E-123	postive
TRAJ13	AC090948.2	0.828978768	1.28E-136	postive
TRAJ14	AC090948.2	0.84801473	4.33E-149	postive
TRAJ16	AC090948.2	0.84912529	7.19E-150	postive
TRAJ17	AC090948.2	0.82319675	3.90E-133	postive
TRAJ18	AC090948.2	0.834163543	7.37E-140	postive
TRAJ21	AC090948.2	0.872338538	8.97E-168	postive
TRAJ31	AC090948.2	0.870194539	5.61E-166	postive
TRAJ37	AC090948.2	0.850841537	4.36E-151	postive
TRAJ38	AC090948.2	0.916831301	1.13E-214	postive
TRAJ39	AC090948.2	0.881165529	1.59E-175	postive
NOX4	LINC02544	0.508087689	1.86E-36	postive
CLEC11A	LINC02544	0.564208337	2.71E-46	postive
CREB1	MALAT1	0.760295714	6.15E-102	postive
RFXAP	MALAT1	0.511525948	5.22E-37	postive
UBR1	MALAT1	0.658058167	1.10E-67	postive
ZC3HAV1	MALAT1	0.52764533	1.12E-39	postive
IL15	MALAT1	0.630097726	1.54E-60	postive
CYLD	MALAT1	0.646069089	1.57E-64	postive
MAPK8	MALAT1	0.542903857	2.43E-42	postive
LMBR1	MALAT1	0.545256372	9.19E-43	postive
IREB2	MALAT1	0.589452316	2.29E-51	postive
DDX17	MALAT1	0.583882841	3.29E-50	postive
PIK3CG	MALAT1	0.531551209	2.40E-40	postive
JAK2	MALAT1	0.642972675	9.76E-64	postive
TXK	MALAT1	0.566699756	8.94E-47	postive
NFAT5	MALAT1	0.896789813	6.83E-191	postive
NFATC3	MALAT1	0.573607933	3.94E-48	postive
MALT1	MALAT1	0.52144967	1.23E-38	postive
PIK3R1	MALAT1	0.636860073	3.36E-62	postive
PIK3CA	MALAT1	0.637935403	1.82E-62	postive
RASGRP3	MALAT1	0.505851289	4.21E-36	postive
GNRH1	MALAT1	0.707051697	3.03E-82	postive
IL6ST	MALAT1	0.610699281	5.39E-56	postive
RABEP1	MALAT1	0.551073677	8.01E-44	postive
ACVR2A	MALAT1	0.606026213	6.02E-55	postive
ANGPTL1	MALAT1	0.718071354	6.05E-86	postive
BMPR1A	MALAT1	0.547223277	4.05E-43	postive
BMPR2	MALAT1	0.679802846	8.51E-74	postive
CRLF3	MALAT1	0.60471586	1.18E-54	postive
LIFR	MALAT1	0.5009356	2.49E-35	postive
NR1D2	MALAT1	0.527448224	1.21E-39	postive
NR2C2	MALAT1	0.661893521	1.00E-68	postive
RORA	MALAT1	0.845455031	2.57E-147	postive
SOS1	MALAT1	0.631224527	8.20E-61	postive
SOS2	MALAT1	0.554301611	2.02E-44	postive
BRAF	MALAT1	0.765693367	3.19E-104	postive
CBL	MALAT1	0.57064502	1.52E-47	postive
CBLB	MALAT1	0.692522216	1.27E-77	postive
PDK1	MALAT1	0.563588218	3.56E-46	postive
TRAJ1	MALAT1	0.757083149	1.32E-100	postive
TRAJ2	MALAT1	0.808262859	1.08E-124	postive
TRAJ3	MALAT1	0.854394503	1.17E-153	postive
TRAJ5	MALAT1	0.773974785	7.52E-108	postive
TRAJ6	MALAT1	0.855663369	1.36E-154	postive
TRAJ8	MALAT1	0.762400273	8.04E-103	postive
TRAJ10	MALAT1	0.85120322	2.40E-151	postive
TRAJ12	MALAT1	0.809338594	2.81E-125	postive
TRAJ13	MALAT1	0.792855134	9.90E-117	postive
TRAJ14	MALAT1	0.811761806	1.32E-126	postive
TRAJ16	MALAT1	0.801097274	6.65E-121	postive
TRAJ17	MALAT1	0.765955136	2.47E-104	postive
TRAJ18	MALAT1	0.840413795	6.48E-144	postive
TRAJ21	MALAT1	0.841958558	6.06E-145	postive
TRAJ31	MALAT1	0.852633587	2.25E-152	postive

TRAJ37	MALAT1	0.87937696	6.60E-174	postive
TRAJ38	MALAT1	0.899202506	1.75E-193	postive
TRAJ39	MALAT1	0.843757407	3.71E-146	postive
CREB1	AL132989.1	0.661975712	9.50E-69	postive
RFXAP	AL132989.1	0.605963543	6.22E-55	postive
UBR1	AL132989.1	0.544980103	1.03E-42	postive
ZC3HAV1	AL132989.1	0.524694761	3.53E-39	postive
IL15	AL132989.1	0.500413967	3.00E-35	postive
CYLD	AL132989.1	0.569428016	2.63E-47	postive
MAPK8	AL132989.1	0.504570525	6.70E-36	postive
NFKBIZ	AL132989.1	0.501487611	2.04E-35	postive
DDX17	AL132989.1	0.743870018	2.42E-95	postive
JAK2	AL132989.1	0.538774444	1.32E-41	postive
TXK	AL132989.1	0.530573909	3.53E-40	postive
NFAT5	AL132989.1	0.732452525	4.77E-91	postive
MALT1	AL132989.1	0.503698255	9.19E-36	postive
PIK3R1	AL132989.1	0.550138003	1.19E-43	postive
PIK3CA	AL132989.1	0.544104335	1.48E-42	postive
IGHD6-6	AL132989.1	0.509859749	9.67E-37	postive
LTB4R2	AL132989.1	0.618970305	6.81E-58	postive
GNRH1	AL132989.1	0.788956231	8.02E-115	postive
IL6ST	AL132989.1	0.549515727	1.55E-43	postive
RABEP1	AL132989.1	0.539633424	9.29E-42	postive
ACVR2A	AL132989.1	0.583855851	3.34E-50	postive
ANGPTL1	AL132989.1	0.622567122	9.77E-59	postive
BMPR2	AL132989.1	0.565676513	1.41E-46	postive
CRLF3	AL132989.1	0.592354513	5.60E-52	postive
NR2C1	AL132989.1	0.624838139	2.83E-59	postive
NR2C2	AL132989.1	0.788119068	2.03E-114	postive
RORA	AL132989.1	0.712845486	3.62E-84	postive
SOS1	AL132989.1	0.61638586	2.71E-57	postive
SOS2	AL132989.1	0.576400972	1.09E-48	postive
BRAF	AL132989.1	0.752933286	6.45E-99	postive
ITK	AL132989.1	0.528056441	9.52E-40	postive
CBL	AL132989.1	0.564221172	2.69E-46	postive
CBLB	AL132989.1	0.592557197	5.07E-52	postive
TRAJ1	AL132989.1	0.688649298	1.95E-76	postive
TRAJ2	AL132989.1	0.697933933	2.60E-79	postive
TRAJ3	AL132989.1	0.741111578	2.77E-94	postive
TRAJ5	AL132989.1	0.676124395	1.00E-72	postive
TRAJ6	AL132989.1	0.693193512	7.88E-78	postive
TRAJ8	AL132989.1	0.687794413	3.54E-76	postive
TRAJ10	AL132989.1	0.656369639	3.12E-67	postive
TRAJ12	AL132989.1	0.614977849	5.71E-57	postive
TRAJ13	AL132989.1	0.674272599	3.42E-72	postive
TRAJ14	AL132989.1	0.676292723	8.96E-73	postive
TRAJ16	AL132989.1	0.674583488	2.78E-72	postive
TRAJ17	AL132989.1	0.649474394	2.07E-65	postive
TRAJ18	AL132989.1	0.62492936	2.69E-59	postive
TRAJ21	AL132989.1	0.692974513	9.21E-78	postive
TRAJ31	AL132989.1	0.679314542	1.18E-73	postive
TRAJ37	AL132989.1	0.683371023	7.52E-75	postive
TRAJ38	AL132989.1	0.722199605	2.24E-87	postive
TRAJ39	AL132989.1	0.708625721	9.20E-83	postive
CREB1	AC108727.1	0.760250375	6.43E-102	postive
UBR1	AC108727.1	0.590568895	1.34E-51	postive
ZC3HAV1	AC108727.1	0.557331407	5.50E-45	postive
ZC3HAV1L	AC108727.1	0.510808378	6.81E-37	postive
IL15	AC108727.1	0.635925732	5.74E-62	postive
CYLD	AC108727.1	0.590052889	1.71E-51	postive
EIF2AK2	AC108727.1	0.558732597	2.99E-45	postive
MAPK8	AC108727.1	0.553711034	2.61E-44	postive
NFKBIZ	AC108727.1	0.522130418	9.50E-39	postive
LMBR1	AC108727.1	0.587632775	5.51E-51	postive
LIMS1	AC108727.1	0.532695963	1.52E-40	postive

IREB2	AC108727.1	0.576111558	1.25E-48	postive
DDX17	AC108727.1	0.581792077	8.84E-50	postive
PIK3CG	AC108727.1	0.539034984	1.19E-41	postive
JAK2	AC108727.1	0.6583251	9.31E-68	postive
TXK	AC108727.1	0.592204785	6.02E-52	postive
NFAT5	AC108727.1	0.79320513	6.64E-117	postive
MALT1	AC108727.1	0.553545674	2.80E-44	postive
PIK3R1	AC108727.1	0.634471516	1.31E-61	postive
PIK3CA	AC108727.1	0.720512967	8.67E-87	postive
PIK3CB	AC108727.1	0.565667032	1.42E-46	postive
AKT3	AC108727.1	0.513852645	2.19E-37	postive
RASGRP3	AC108727.1	0.534684575	6.87E-41	postive
GNRH1	AC108727.1	0.696559963	7.03E-79	postive
IL6ST	AC108727.1	0.611246492	4.05E-56	postive
RABEP1	AC108727.1	0.534738895	6.72E-41	postive
ACVR2A	AC108727.1	0.574331361	2.83E-48	postive
ANGPTL1	AC108727.1	0.826394147	4.78E-135	postive
BMPR2	AC108727.1	0.696712675	6.30E-79	postive
CRLF3	AC108727.1	0.628232192	4.35E-60	postive
IL18R1	AC108727.1	0.522865482	7.16E-39	postive
NR2C2	AC108727.1	0.667082331	3.69E-70	postive
RORA	AC108727.1	0.822231905	1.45E-132	postive
SOS1	AC108727.1	0.681373243	2.94E-74	postive
SOS2	AC108727.1	0.579632187	2.43E-49	postive
BRAF	AC108727.1	0.793767192	3.49E-117	postive
ITK	AC108727.1	0.555853746	1.04E-44	postive
CBL	AC108727.1	0.643911035	5.63E-64	postive
CBLB	AC108727.1	0.787276002	5.18E-114	postive
RASGRP1	AC108727.1	0.513072999	2.94E-37	postive
PDK1	AC108727.1	0.639536598	7.21E-63	postive
TRAJ1	AC108727.1	0.700736598	3.35E-80	postive
TRAJ2	AC108727.1	0.676087473	1.03E-72	postive
TRAJ3	AC108727.1	0.753654622	3.30E-99	postive
TRAJ5	AC108727.1	0.806588969	8.54E-124	postive
TRAJ6	AC108727.1	0.757306805	1.07E-100	postive
TRAJ8	AC108727.1	0.702827062	7.16E-81	postive
TRAJ10	AC108727.1	0.760431336	5.40E-102	postive
TRAJ12	AC108727.1	0.686221223	1.06E-75	postive
TRAJ13	AC108727.1	0.762212754	9.65E-103	postive
TRAJ14	AC108727.1	0.753052249	5.77E-99	postive
TRAJ16	AC108727.1	0.753153134	5.26E-99	postive
TRAJ17	AC108727.1	0.735855398	2.64E-92	postive
TRAJ18	AC108727.1	0.73317316	2.59E-91	postive
TRAJ21	AC108727.1	0.805279583	4.26E-123	postive
TRAJ31	AC108727.1	0.810923604	3.83E-126	postive
TRAJ37	AC108727.1	0.766338261	1.69E-104	postive
TRAJ38	AC108727.1	0.841164252	2.06E-144	postive
TRAJ39	AC108727.1	0.805700543	2.54E-123	postive
GAL	PCAT19	0.559347683	2.29E-45	postive
CREB1	AC005070.3	0.825740993	1.18E-134	postive
RFXAP	AC005070.3	0.50919861	1.23E-36	postive
UBR1	AC005070.3	0.682204217	1.67E-74	postive
ZC3HAV1	AC005070.3	0.587645345	5.47E-51	postive
ZC3HAV1L	AC005070.3	0.524633697	3.61E-39	postive
IL15	AC005070.3	0.57777573	5.77E-49	postive
CYLD	AC005070.3	0.643882994	5.72E-64	postive
EIF2AK2	AC005070.3	0.555813889	1.06E-44	postive
MAPK8	AC005070.3	0.587549738	5.73E-51	postive
TLR1	AC005070.3	0.520849385	1.55E-38	postive
LMBR1	AC005070.3	0.616569074	2.46E-57	postive
LIMS1	AC005070.3	0.529985942	4.45E-40	postive
IREB2	AC005070.3	0.633408293	2.40E-61	postive
DDX17	AC005070.3	0.621771212	1.50E-58	postive
PIK3CG	AC005070.3	0.565391024	1.60E-46	postive
JAK2	AC005070.3	0.681458416	2.77E-74	postive

TXK	AC005070.3	0.600987171	7.77E-54	postive
PPP3CB	AC005070.3	0.515034953	1.41E-37	postive
NFAT5	AC005070.3	0.901559951	4.43E-196	postive
NFATC3	AC005070.3	0.512082394	4.25E-37	postive
MALT1	AC005070.3	0.558615172	3.15E-45	postive
PIK3R1	AC005070.3	0.663404452	3.85E-69	postive
PIK3CA	AC005070.3	0.711763567	8.34E-84	postive
PIK3CB	AC005070.3	0.532509486	1.64E-40	postive
AKT3	AC005070.3	0.574995845	2.08E-48	postive
RASGRP3	AC005070.3	0.540788102	5.80E-42	postive
PLXNC1	AC005070.3	0.514693603	1.60E-37	postive
GNRH1	AC005070.3	0.76917686	9.93E-106	postive
IL6ST	AC005070.3	0.660265729	2.78E-68	postive
RABEP1	AC005070.3	0.593403559	3.35E-52	postive
ACVR2A	AC005070.3	0.663389278	3.89E-69	postive
ANGPTL1	AC005070.3	0.794017383	2.62E-117	postive
BMPR1A	AC005070.3	0.585536768	1.50E-50	postive
BMPR2	AC005070.3	0.757494701	8.93E-101	postive
CRLF3	AC005070.3	0.696511943	7.28E-79	postive
LIFR	AC005070.3	0.505629561	4.56E-36	postive
NR1D2	AC005070.3	0.539936777	8.21E-42	postive
NR2C2	AC005070.3	0.702011142	1.31E-80	postive
NR3C1	AC005070.3	0.504404194	7.12E-36	postive
RORA	AC005070.3	0.879512659	4.99E-174	postive
SOS1	AC005070.3	0.702230445	1.11E-80	postive
SOS2	AC005070.3	0.6022598	4.09E-54	postive
BRAF	AC005070.3	0.835078515	1.93E-140	postive
ITK	AC005070.3	0.521042682	1.44E-38	postive
CD28	AC005070.3	0.506386878	3.46E-36	postive
CBL	AC005070.3	0.620637314	2.78E-58	postive
CBLB	AC005070.3	0.749253978	1.90E-97	postive
RASGRP1	AC005070.3	0.513838609	2.21E-37	postive
PDK1	AC005070.3	0.614391588	7.78E-57	postive
TRAJ1	AC005070.3	0.763517734	2.71E-103	postive
TRAJ2	AC005070.3	0.783675361	2.66E-112	postive
TRAJ3	AC005070.3	0.804636717	9.33E-123	postive
TRAJ5	AC005070.3	0.829546728	5.71E-137	postive
TRAJ6	AC005070.3	0.835464581	1.09E-140	postive
TRAJ8	AC005070.3	0.79942851	4.82E-120	postive
TRAJ10	AC005070.3	0.816471344	3.07E-129	postive
TRAJ12	AC005070.3	0.725534489	1.49E-88	postive
TRAJ13	AC005070.3	0.839192612	4.15E-143	postive
TRAJ14	AC005070.3	0.802169725	1.84E-121	postive
TRAJ16	AC005070.3	0.818332623	2.66E-130	postive
TRAJ17	AC005070.3	0.764189871	1.40E-103	postive
TRAJ18	AC005070.3	0.749244193	1.92E-97	postive
TRAJ21	AC005070.3	0.849351929	4.98E-150	postive
TRAJ31	AC005070.3	0.876021593	6.17E-171	postive
TRAJ37	AC005070.3	0.855311223	2.48E-154	postive
TRAJ38	AC005070.3	0.901181386	1.17E-195	postive
TRAJ39	AC005070.3	0.859653524	1.37E-157	postive
NFAT5	AC002563.1	0.522335516	8.78E-39	postive
GNRH1	AC002563.1	0.500692358	2.71E-35	postive
NR2C2	AC002563.1	0.501499494	2.03E-35	postive
RORA	AC002563.1	0.529140899	6.22E-40	postive
CREB1	AL355488.1	0.670997189	2.93E-71	postive
RFXAP	AL355488.1	0.574767576	2.31E-48	postive
UBR1	AL355488.1	0.563912258	3.09E-46	postive
ZC3HAV1L	AL355488.1	0.5381676	1.69E-41	postive
MAPK8	AL355488.1	0.562056725	7.00E-46	postive
IREB2	AL355488.1	0.570111433	1.93E-47	postive
DDX17	AL355488.1	0.670291894	4.64E-71	postive
TXK	AL355488.1	0.516759121	7.36E-38	postive
NFAT5	AL355488.1	0.699388419	9.00E-80	postive
PIK3CA	AL355488.1	0.521872971	1.05E-38	postive

AKT3	AL355488.1	0.521715611	1.11E-38	postive
LTB4R2	AL355488.1	0.522294049	8.92E-39	postive
GNRH1	AL355488.1	0.829477167	6.30E-137	postive
RABEP1	AL355488.1	0.500496095	2.91E-35	postive
ACVR2A	AL355488.1	0.588688615	3.31E-51	postive
ANGPTL1	AL355488.1	0.63001086	1.62E-60	postive
BMPR1A	AL355488.1	0.521350363	1.28E-38	postive
BMPR2	AL355488.1	0.596329718	7.94E-53	postive
CRLF3	AL355488.1	0.58274561	5.64E-50	postive
NR2C1	AL355488.1	0.625439256	2.03E-59	postive
NR2C2	AL355488.1	0.737232502	8.09E-93	postive
RORA	AL355488.1	0.659726679	3.89E-68	postive
SOS1	AL355488.1	0.643349962	7.82E-64	postive
BRAF	AL355488.1	0.741363054	2.22E-94	postive
CBL	AL355488.1	0.537623967	2.10E-41	postive
CBLB	AL355488.1	0.630079075	1.56E-60	postive
PDK1	AL355488.1	0.523638761	5.31E-39	postive
TRAJ1	AL355488.1	0.592839821	4.42E-52	postive
TRAJ2	AL355488.1	0.637751003	2.02E-62	postive
TRAJ3	AL355488.1	0.65171081	5.37E-66	postive
TRAJ5	AL355488.1	0.583963257	3.17E-50	postive
TRAJ6	AL355488.1	0.624086764	4.27E-59	postive
TRAJ8	AL355488.1	0.628584203	3.58E-60	postive
TRAJ10	AL355488.1	0.569325517	2.75E-47	postive
TRAJ12	AL355488.1	0.518843153	3.34E-38	postive
TRAJ13	AL355488.1	0.60610677	5.78E-55	postive
TRAJ14	AL355488.1	0.593800085	2.76E-52	postive
TRAJ16	AL355488.1	0.600633342	9.28E-54	postive
TRAJ17	AL355488.1	0.543278898	2.08E-42	postive
TRAJ21	AL355488.1	0.608974166	1.32E-55	postive
TRAJ31	AL355488.1	0.622268066	1.15E-58	postive
TRAJ37	AL355488.1	0.620292375	3.34E-58	postive
TRAJ38	AL355488.1	0.668685977	1.31E-70	postive
TRAJ39	AL355488.1	0.64020917	4.89E-63	postive
CREB1	AL162724.2	0.755518743	5.77E-100	postive
UBR1	AL162724.2	0.617976168	1.16E-57	postive
ZC3HAV1	AL162724.2	0.538762416	1.32E-41	postive
ZC3HAV1L	AL162724.2	0.5207774	1.60E-38	postive
IL15	AL162724.2	0.632145679	4.89E-61	postive
CYLD	AL162724.2	0.551077417	8.00E-44	postive
EIF2AK2	AL162724.2	0.500709343	2.70E-35	postive
MAPK8	AL162724.2	0.540514599	6.49E-42	postive
NFKBIZ	AL162724.2	0.506717909	3.07E-36	postive
LMBR1	AL162724.2	0.56113358	1.05E-45	postive
IREB2	AL162724.2	0.591324485	9.25E-52	postive
DDX17	AL162724.2	0.543339704	2.03E-42	postive
PIK3CG	AL162724.2	0.506672736	3.12E-36	postive
JAK2	AL162724.2	0.607141662	3.40E-55	postive
TXK	AL162724.2	0.561347206	9.55E-46	postive
NFAT5	AL162724.2	0.750481155	6.19E-98	postive
MALT1	AL162724.2	0.516050822	9.61E-38	postive
PIK3R1	AL162724.2	0.595196054	1.39E-52	postive
PIK3CA	AL162724.2	0.69092405	3.94E-77	postive
PIK3CB	AL162724.2	0.54904945	1.88E-43	postive
RASGRP3	AL162724.2	0.514627472	1.64E-37	postive
IGHD4-4	AL162724.2	0.502755915	1.29E-35	postive
GNRH1	AL162724.2	0.720545799	8.45E-87	postive
IL6ST	AL162724.2	0.568023718	4.94E-47	postive
IL7	AL162724.2	0.505132021	5.47E-36	postive
RABEP1	AL162724.2	0.520899304	1.52E-38	postive
ACVR2A	AL162724.2	0.639162744	8.95E-63	postive
ANGPTL1	AL162724.2	0.75482991	1.10E-99	postive
BMPR1A	AL162724.2	0.54317261	2.18E-42	postive
BMPR2	AL162724.2	0.701678364	1.67E-80	postive
CRLF3	AL162724.2	0.583470874	4.00E-50	postive

NR2C2	AL162724.2	0.680753069	4.48E-74	postive
RORA	AL162724.2	0.765499568	3.87E-104	postive
SOS1	AL162724.2	0.632755716	3.47E-61	postive
SOS2	AL162724.2	0.568588726	3.83E-47	postive
BRAF	AL162724.2	0.73603119	2.27E-92	postive
CBL	AL162724.2	0.557315335	5.53E-45	postive
CBLB	AL162724.2	0.656189608	3.48E-67	postive
PDK1	AL162724.2	0.597460576	4.53E-53	postive
TRAJ1	AL162724.2	0.66750876	2.81E-70	postive
TRAJ2	AL162724.2	0.600949942	7.92E-54	postive
TRAJ3	AL162724.2	0.725365425	1.71E-88	postive
TRAJ5	AL162724.2	0.742086087	1.18E-94	postive
TRAJ6	AL162724.2	0.723827986	6.00E-88	postive
TRAJ8	AL162724.2	0.690833122	4.20E-77	postive
TRAJ10	AL162724.2	0.742330646	9.48E-95	postive
TRAJ12	AL162724.2	0.656492794	2.89E-67	postive
TRAJ13	AL162724.2	0.707859417	1.65E-82	postive
TRAJ14	AL162724.2	0.690783101	4.35E-77	postive
TRAJ16	AL162724.2	0.720099111	1.21E-86	postive
TRAJ17	AL162724.2	0.739637341	1.01E-93	postive
TRAJ18	AL162724.2	0.664025856	2.60E-69	postive
TRAJ21	AL162724.2	0.737164422	8.58E-93	postive
TRAJ31	AL162724.2	0.778538037	6.47E-110	postive
TRAJ37	AL162724.2	0.721264045	4.75E-87	postive
TRAJ38	AL162724.2	0.785504475	3.63E-113	postive
TRAJ39	AL162724.2	0.762154305	1.02E-102	postive
BPIFB1	AL357093.2	0.507977466	1.93E-36	postive
DES	AL357093.2	0.650795793	9.33E-66	postive
CREB1	AP005899.1	0.685556692	1.67E-75	postive
UBR1	AP005899.1	0.582104726	7.63E-50	postive
ZC3HAV1	AP005899.1	0.532634554	1.56E-40	postive
ZC3HAV1L	AP005899.1	0.565237297	1.72E-46	postive
IL15	AP005899.1	0.514230614	1.90E-37	postive
EIF2AK2	AP005899.1	0.542728131	2.62E-42	postive
MAPK8	AP005899.1	0.617739542	1.32E-57	postive
NFKBIZ	AP005899.1	0.507651629	2.18E-36	postive
IREB2	AP005899.1	0.574919235	2.16E-48	postive
DDX17	AP005899.1	0.5703271	1.75E-47	postive
JAK2	AP005899.1	0.552263574	4.83E-44	postive
TXK	AP005899.1	0.505271124	5.20E-36	postive
NFAT5	AP005899.1	0.719515456	1.92E-86	postive
MALT1	AP005899.1	0.522856836	7.18E-39	postive
PIK3CA	AP005899.1	0.618717879	7.80E-58	postive
LTB4R2	AP005899.1	0.509109901	1.28E-36	postive
GNRH1	AP005899.1	0.723486185	7.91E-88	postive
RABEP1	AP005899.1	0.501414405	2.09E-35	postive
ACVR2A	AP005899.1	0.567554818	6.10E-47	postive
ANGPTL1	AP005899.1	0.643892379	5.69E-64	postive
BMPR1A	AP005899.1	0.529986578	4.45E-40	postive
BMPR2	AP005899.1	0.575486336	1.66E-48	postive
CRLF3	AP005899.1	0.599594328	1.56E-53	postive
NR2C1	AP005899.1	0.559651922	2.01E-45	postive
NR2C2	AP005899.1	0.726693724	5.77E-89	postive
RORA	AP005899.1	0.673617716	5.27E-72	postive
SOS1	AP005899.1	0.663539661	3.54E-69	postive
SOS2	AP005899.1	0.530774862	3.26E-40	postive
BRAF	AP005899.1	0.730634555	2.20E-90	postive
CBL	AP005899.1	0.52954569	5.30E-40	postive
CBLB	AP005899.1	0.637737583	2.03E-62	postive
PDK1	AP005899.1	0.578555022	4.02E-49	postive
TRAJ1	AP005899.1	0.680369573	5.80E-74	postive
TRAJ2	AP005899.1	0.618901509	7.07E-58	postive
TRAJ3	AP005899.1	0.694149941	3.98E-78	postive
TRAJ5	AP005899.1	0.698805534	1.38E-79	postive
TRAJ6	AP005899.1	0.705511463	9.66E-82	postive

TRAJ8	AP005899.1	0.681486473	2.72E-74	postive
TRAJ10	AP005899.1	0.697904898	2.65E-79	postive
TRAJ12	AP005899.1	0.630538898	1.20E-60	postive
TRAJ13	AP005899.1	0.67389676	4.38E-72	postive
TRAJ14	AP005899.1	0.679971843	7.60E-74	postive
TRAJ16	AP005899.1	0.713007643	3.19E-84	postive
TRAJ17	AP005899.1	0.687606715	4.04E-76	postive
TRAJ18	AP005899.1	0.626817171	9.53E-60	postive
TRAJ21	AP005899.1	0.704920446	1.50E-81	postive
TRAJ31	AP005899.1	0.714559759	9.55E-85	postive
TRAJ37	AP005899.1	0.673778833	4.74E-72	postive
TRAJ38	AP005899.1	0.752089475	1.41E-98	postive
TRAJ39	AP005899.1	0.725770508	1.23E-88	postive
A2M	TBX5-AS1	0.760362816	5.77E-102	postive
ELN	TBX5-AS1	0.626311017	1.26E-59	postive
PDGFRA	TBX5-AS1	0.517636269	5.28E-38	postive
PDGFRB	TBX5-AS1	0.600662527	9.15E-54	postive
SLIT2	TBX5-AS1	0.674537381	2.87E-72	postive
ROBO2	TBX5-AS1	0.566275626	1.08E-46	postive
VEGFD	TBX5-AS1	0.541573741	4.20E-42	postive
GDF10	TBX5-AS1	0.551461839	6.79E-44	postive
LTBP2	TBX5-AS1	0.806005885	1.75E-123	postive
ANGPT1	TBX5-AS1	0.648442544	3.83E-65	postive
CALCRL	TBX5-AS1	0.507382949	2.40E-36	postive
S1PR1	TBX5-AS1	0.537564468	2.15E-41	postive
TGFBR2	TBX5-AS1	0.541554006	4.24E-42	postive
FAM3D	LINC01559	0.546452802	5.59E-43	postive
HNF4A	LINC01559	0.532135318	1.90E-40	postive
IL1R2	LINC01559	0.632887423	3.22E-61	postive
IL22RA1	LINC01559	0.748229743	4.83E-97	postive
CREB1	AL031666.1	0.646253406	1.41E-64	postive
UBR1	AL031666.1	0.578389652	4.34E-49	postive
DDX17	AL031666.1	0.541401229	4.51E-42	postive
JAK2	AL031666.1	0.5009244	2.50E-35	postive
NFAT5	AL031666.1	0.73382396	1.49E-91	postive
PIK3R1	AL031666.1	0.504209588	7.64E-36	postive
GNRH1	AL031666.1	0.707183108	2.75E-82	postive
ACVR2A	AL031666.1	0.55062908	9.67E-44	postive
ANGPTL1	AL031666.1	0.65141662	6.41E-66	postive
BMPR2	AL031666.1	0.571417534	1.07E-47	postive
CRLF3	AL031666.1	0.541988956	3.54E-42	postive
NR2C2	AL031666.1	0.585706072	1.38E-50	postive
RORA	AL031666.1	0.6716084	1.97E-71	postive
SOS1	AL031666.1	0.535221635	5.54E-41	postive
BRAF	AL031666.1	0.695977806	1.07E-78	postive
CBLB	AL031666.1	0.56527572	1.69E-46	postive
TRAJ1	AL031666.1	0.645809122	1.84E-64	postive
TRAJ2	AL031666.1	0.656581018	2.74E-67	postive
TRAJ3	AL031666.1	0.68378689	5.66E-75	postive
TRAJ5	AL031666.1	0.645886204	1.75E-64	postive
TRAJ6	AL031666.1	0.708431643	1.07E-82	postive
TRAJ8	AL031666.1	0.696280331	8.61E-79	postive
TRAJ10	AL031666.1	0.656376044	3.11E-67	postive
TRAJ12	AL031666.1	0.621345239	1.89E-58	postive
TRAJ13	AL031666.1	0.698076511	2.34E-79	postive
TRAJ14	AL031666.1	0.685307002	1.99E-75	postive
TRAJ16	AL031666.1	0.665415399	1.07E-69	postive
TRAJ17	AL031666.1	0.611826326	2.99E-56	postive
TRAJ18	AL031666.1	0.575461336	1.68E-48	postive
TRAJ21	AL031666.1	0.696602648	6.82E-79	postive
TRAJ31	AL031666.1	0.705818861	7.67E-82	postive
TRAJ37	AL031666.1	0.700798469	3.20E-80	postive
TRAJ38	AL031666.1	0.741610107	1.79E-94	postive
TRAJ39	AL031666.1	0.703878676	3.27E-81	postive
TRAJ38	AC103591.3	0.525900588	2.21E-39	postive

DDX17	AC006042.1	0.562681984	5.31E-46	postive
LTB4R2	AC006042.1	0.572622221	6.18E-48	postive
GNRH1	AC006042.1	0.602180988	4.26E-54	postive
NR2C1	AC006042.1	0.517511549	5.54E-38	postive
NR2C2	AC006042.1	0.596422204	7.58E-53	postive
BRAF	AC006042.1	0.502322456	1.51E-35	postive
PTK2	OTUD6B-AS1	0.524213379	4.25E-39	postive
CREB1	AC024075.3	0.764098737	1.53E-103	postive
RFXAP	AC024075.3	0.572539802	6.41E-48	postive
UBR1	AC024075.3	0.655287676	6.07E-67	postive
ZC3HAV1	AC024075.3	0.635596819	6.92E-62	postive
IFNAR1	AC024075.3	0.504559812	6.73E-36	postive
CYLD	AC024075.3	0.650333813	1.23E-65	postive
EIF2AK2	AC024075.3	0.548840319	2.06E-43	postive
MAPK8	AC024075.3	0.512458673	3.69E-37	postive
SP1	AC024075.3	0.508067587	1.87E-36	postive
IREB2	AC024075.3	0.610048561	7.56E-56	postive
DDX17	AC024075.3	0.674561717	2.82E-72	postive
PIK3CG	AC024075.3	0.567003239	7.81E-47	postive
JAK2	AC024075.3	0.590968354	1.10E-51	postive
TXK	AC024075.3	0.569692882	2.33E-47	postive
NFAT5	AC024075.3	0.761339291	2.25E-102	postive
NFATC3	AC024075.3	0.582601069	6.04E-50	postive
MALT1	AC024075.3	0.502033205	1.68E-35	postive
PIK3R1	AC024075.3	0.695514763	1.49E-78	postive
PIK3CA	AC024075.3	0.637922801	1.83E-62	postive
GSK3B	AC024075.3	0.501884257	1.77E-35	postive
RASGRP3	AC024075.3	0.509692728	1.03E-36	postive
PLXNC1	AC024075.3	0.537792253	1.96E-41	postive
GNRH1	AC024075.3	0.662410643	7.22E-69	postive
IL6ST	AC024075.3	0.682391608	1.47E-74	postive
RABEP1	AC024075.3	0.59971662	1.47E-53	postive
ACVR2A	AC024075.3	0.669393787	8.32E-71	postive
ANGPTL1	AC024075.3	0.661966198	9.55E-69	postive
BMPR1A	AC024075.3	0.570629043	1.53E-47	postive
BMPR2	AC024075.3	0.727142343	3.99E-89	postive
CRLF3	AC024075.3	0.561637731	8.41E-46	postive
LIFR	AC024075.3	0.526135741	2.02E-39	postive
NR1D2	AC024075.3	0.603778183	1.90E-54	postive
NR2C1	AC024075.3	0.513484003	2.52E-37	postive
NR2C2	AC024075.3	0.768018457	3.17E-105	postive
NR3C2	AC024075.3	0.553395333	2.98E-44	postive
RORA	AC024075.3	0.793200058	6.68E-117	postive
SOS1	AC024075.3	0.675495803	1.52E-72	postive
SOS2	AC024075.3	0.615398199	4.57E-57	postive
BRAF	AC024075.3	0.787877042	2.66E-114	postive
ITK	AC024075.3	0.510991568	6.37E-37	postive
CD28	AC024075.3	0.513616822	2.40E-37	postive
CBL	AC024075.3	0.599982513	1.29E-53	postive
CBLB	AC024075.3	0.633679467	2.06E-61	postive
RASGRP1	AC024075.3	0.561499864	8.94E-46	postive
TRAJ1	AC024075.3	0.663444818	3.76E-69	postive
TRAJ2	AC024075.3	0.657059122	2.04E-67	postive
TRAJ3	AC024075.3	0.716743713	1.73E-85	postive
TRAJ5	AC024075.3	0.70972191	3.99E-83	postive
TRAJ6	AC024075.3	0.688303724	2.48E-76	postive
TRAJ8	AC024075.3	0.680604723	4.95E-74	postive
TRAJ10	AC024075.3	0.664650246	1.75E-69	postive
TRAJ12	AC024075.3	0.662561618	6.56E-69	postive
TRAJ13	AC024075.3	0.687456542	4.48E-76	postive
TRAJ14	AC024075.3	0.679550341	1.01E-73	postive
TRAJ16	AC024075.3	0.672739385	9.38E-72	postive
TRAJ17	AC024075.3	0.670933821	3.06E-71	postive
TRAJ18	AC024075.3	0.660152969	2.98E-68	postive
TRAJ21	AC024075.3	0.710429104	2.32E-83	postive

TRAJ31	AC024075.3	0.721345243	4.45E-87	postive
TRAJ37	AC024075.3	0.701254424	2.29E-80	postive
TRAJ38	AC024075.3	0.736756733	1.22E-92	postive
TRAJ39	AC024075.3	0.722179799	2.27E-87	postive
AGRP	AC013457.1	0.549002947	1.92E-43	postive
CREB1	AP003392.1	0.524794725	3.40E-39	postive
UBR1	AP003392.1	0.519682404	2.43E-38	postive
ZC3HAV1	AP003392.1	0.513653502	2.36E-37	postive
ZC3HAV1L	AP003392.1	0.512205884	4.06E-37	postive
DDX17	AP003392.1	0.636369813	4.45E-62	postive
NFAT5	AP003392.1	0.533013836	1.34E-40	postive
LTB4R2	AP003392.1	0.594859616	1.64E-52	postive
GNRH1	AP003392.1	0.655614238	4.96E-67	postive
NR2C1	AP003392.1	0.593504087	3.19E-52	postive
NR2C2	AP003392.1	0.736260458	1.87E-92	postive
RORA	AP003392.1	0.501594462	1.96E-35	postive
SOS1	AP003392.1	0.505708189	4.43E-36	postive
BRAF	AP003392.1	0.633370451	2.45E-61	postive
TEC	AP003392.1	0.538437907	1.51E-41	postive
CBL	AP003392.1	0.585064872	1.88E-50	postive
TRAJ2	AP003392.1	0.500007787	3.46E-35	postive
TRAJ3	AP003392.1	0.520898995	1.52E-38	postive
TYK2	AC007292.1	0.63918302	8.85E-63	postive
IRF9	AC007292.1	0.508478555	1.61E-36	postive
EPOR	AC007292.1	0.500100113	3.35E-35	postive
CREB1	ADNP-AS1	0.748132048	5.28E-97	postive
RFXAP	ADNP-AS1	0.559560682	2.09E-45	postive
UBR1	ADNP-AS1	0.581600329	9.67E-50	postive
ZC3HAV1	ADNP-AS1	0.506417144	3.42E-36	postive
CYLD	ADNP-AS1	0.607578431	2.71E-55	postive
EIF2AK2	ADNP-AS1	0.511768703	4.77E-37	postive
MAPK8	ADNP-AS1	0.536379766	3.48E-41	postive
LMBR1	ADNP-AS1	0.513362261	2.64E-37	postive
IREB2	ADNP-AS1	0.552304075	4.75E-44	postive
DDX17	ADNP-AS1	0.66459128	1.81E-69	postive
JAK2	ADNP-AS1	0.601296549	6.65E-54	postive
TXK	ADNP-AS1	0.557394219	5.35E-45	postive
NFAT5	ADNP-AS1	0.840321678	7.46E-144	postive
MALT1	ADNP-AS1	0.515116775	1.37E-37	postive
PIK3R1	ADNP-AS1	0.589678035	2.06E-51	postive
PIK3CA	ADNP-AS1	0.649925596	1.58E-65	postive
AKT3	ADNP-AS1	0.500084953	3.37E-35	postive
GNRH1	ADNP-AS1	0.762873534	5.08E-103	postive
IL6ST	ADNP-AS1	0.595240103	1.36E-52	postive
RABEP1	ADNP-AS1	0.525385616	2.70E-39	postive
ACVR2A	ADNP-AS1	0.651326664	6.77E-66	postive
ANGPTL1	ADNP-AS1	0.726508656	6.72E-89	postive
BMPR1A	ADNP-AS1	0.509423689	1.14E-36	postive
BMPR2	ADNP-AS1	0.670853673	3.22E-71	postive
CRLF3	ADNP-AS1	0.618530142	8.62E-58	postive
NR2C1	ADNP-AS1	0.542489674	2.88E-42	postive
NR2C2	ADNP-AS1	0.691579891	2.48E-77	postive
RORA	ADNP-AS1	0.827107464	1.77E-135	postive
SOS1	ADNP-AS1	0.664310092	2.17E-69	postive
SOS2	ADNP-AS1	0.595852398	1.01E-52	postive
BRAF	ADNP-AS1	0.767600252	4.81E-105	postive
CBL	ADNP-AS1	0.577860389	5.55E-49	postive
CBLB	ADNP-AS1	0.705236739	1.19E-81	postive
PDK1	ADNP-AS1	0.542517228	2.85E-42	postive
TRAJ1	ADNP-AS1	0.739412145	1.22E-93	postive
TRAJ2	ADNP-AS1	0.702848738	7.04E-81	postive
TRAJ3	ADNP-AS1	0.740364149	5.33E-94	postive
TRAJ5	ADNP-AS1	0.810220459	9.29E-126	postive
TRAJ6	ADNP-AS1	0.775657813	1.32E-108	postive
TRAJ8	ADNP-AS1	0.764000644	1.69E-103	postive

TRAJ10	ADNP-AS1	0.756295536	2.78E-100	postive
TRAJ12	ADNP-AS1	0.681514146	2.67E-74	postive
TRAJ13	ADNP-AS1	0.81060491	5.73E-126	postive
TRAJ14	ADNP-AS1	0.76310457	4.05E-103	postive
TRAJ16	ADNP-AS1	0.782113378	1.44E-111	postive
TRAJ17	ADNP-AS1	0.736179529	2.00E-92	postive
TRAJ18	ADNP-AS1	0.699632476	7.53E-80	postive
TRAJ21	ADNP-AS1	0.809468939	2.39E-125	postive
TRAJ31	ADNP-AS1	0.822575552	9.07E-133	postive
TRAJ37	ADNP-AS1	0.804357811	1.31E-122	postive
TRAJ38	ADNP-AS1	0.832176278	1.32E-138	postive
TRAJ39	ADNP-AS1	0.815776753	7.60E-129	postive
IRF9	ZNF436-AS1	0.547006858	4.43E-43	postive
NR2C2	HMGN3-AS1	0.521988428	1.00E-38	postive
BRAF	HMGN3-AS1	0.541367995	4.57E-42	postive
TRAJ5	HMGN3-AS1	0.574007507	3.28E-48	postive
TRAJ8	HMGN3-AS1	0.533282892	1.20E-40	postive
TRAJ13	HMGN3-AS1	0.506169902	3.75E-36	postive
TRAJ14	HMGN3-AS1	0.521627125	1.15E-38	postive
TRAJ17	HMGN3-AS1	0.552210812	4.94E-44	postive
TRAJ21	HMGN3-AS1	0.517648395	5.26E-38	postive
TRAJ31	HMGN3-AS1	0.544778375	1.12E-42	postive
TRAJ38	HMGN3-AS1	0.526703482	1.62E-39	postive
TRAJ39	HMGN3-AS1	0.53714818	2.55E-41	postive
UCN	AC005332.5	0.500378944	3.03E-35	postive
PTX3	AC079949.1	0.626689781	1.02E-59	postive
HGF	AC079949.1	0.624706465	3.04E-59	postive
SEMA6B	AC079949.1	0.605632357	7.36E-55	postive
SEMA7A	AC079949.1	0.722069909	2.49E-87	postive
IL24	AC079949.1	0.55226216	4.84E-44	postive
IL13RA2	AC079949.1	0.576044134	1.29E-48	postive
TIE1	AC079949.1	0.569066854	3.09E-47	postive
TNFRSF8	AC079949.1	0.916309292	5.58E-214	postive
GNRH1	FOXO6-AS1	0.521636201	1.15E-38	postive
PRDX1	AC145207.8	0.500635576	2.77E-35	postive
OSGIN1	AC145207.8	0.525276931	2.82E-39	postive
CREB1	AC114760.2	0.790199616	1.99E-115	postive
RFXAP	AC114760.2	0.508824562	1.42E-36	postive
UBR1	AC114760.2	0.635001696	9.72E-62	postive
ZC3HAV1	AC114760.2	0.572708566	5.94E-48	postive
IL15	AC114760.2	0.615197218	5.08E-57	postive
CYLD	AC114760.2	0.651011824	8.19E-66	postive
EIF2AK2	AC114760.2	0.567505346	6.23E-47	postive
MAPK8	AC114760.2	0.564038045	2.92E-46	postive
TLR1	AC114760.2	0.504821836	6.12E-36	postive
LMBR1	AC114760.2	0.5918062	7.32E-52	postive
LIMS1	AC114760.2	0.553978249	2.33E-44	postive
IREB2	AC114760.2	0.602656192	3.35E-54	postive
DDX17	AC114760.2	0.58965586	2.08E-51	postive
PIK3CG	AC114760.2	0.596493297	7.32E-53	postive
JAK2	AC114760.2	0.675900766	1.16E-72	postive
TXK	AC114760.2	0.60796386	2.22E-55	postive
NFAT5	AC114760.2	0.846628838	3.99E-148	postive
MALT1	AC114760.2	0.567453242	6.38E-47	postive
PIK3R1	AC114760.2	0.691675903	2.32E-77	postive
PIK3CA	AC114760.2	0.726880511	4.95E-89	postive
PIK3CB	AC114760.2	0.532336936	1.75E-40	postive
AKT3	AC114760.2	0.555273302	1.33E-44	postive
RASGRP3	AC114760.2	0.560774101	1.23E-45	postive
IGHD6-6	AC114760.2	0.532711938	1.51E-40	postive
PLXNC1	AC114760.2	0.525154861	2.95E-39	postive
GNRH1	AC114760.2	0.670732241	3.49E-71	postive
IL6ST	AC114760.2	0.689183892	1.34E-76	postive
RABEP1	AC114760.2	0.544750763	1.13E-42	postive
ACVR2A	AC114760.2	0.609508499	1.00E-55	postive

ANGPTL1	AC114760.2	0.816059654	5.26E-129	postive
BMPR1A	AC114760.2	0.520573421	1.73E-38	postive
BMPR2	AC114760.2	0.746496944	2.31E-96	postive
CRLF3	AC114760.2	0.656882458	2.27E-67	postive
IL18R1	AC114760.2	0.507318678	2.46E-36	postive
LIFR	AC114760.2	0.505642045	4.54E-36	postive
NR1D2	AC114760.2	0.510107966	8.83E-37	postive
NR2C2	AC114760.2	0.661450778	1.32E-68	postive
RORA	AC114760.2	0.887097052	4.32E-181	postive
SOS1	AC114760.2	0.682744599	1.15E-74	postive
SOS2	AC114760.2	0.589480734	2.26E-51	postive
BRAF	AC114760.2	0.824306021	8.55E-134	postive
ITK	AC114760.2	0.596328061	7.94E-53	postive
CD28	AC114760.2	0.549256207	1.73E-43	postive
CBL	AC114760.2	0.614270413	8.29E-57	postive
CBLB	AC114760.2	0.770402012	2.89E-106	postive
RASGRP1	AC114760.2	0.527815592	1.05E-39	postive
PDK1	AC114760.2	0.615720277	3.85E-57	postive
TRAJ1	AC114760.2	0.728701277	1.10E-89	postive
TRAJ2	AC114760.2	0.784148186	1.59E-112	postive
TRAJ3	AC114760.2	0.810985863	3.54E-126	postive
TRAJ5	AC114760.2	0.822130817	1.66E-132	postive
TRAJ6	AC114760.2	0.801542959	3.90E-121	postive
TRAJ8	AC114760.2	0.781352682	3.25E-111	postive
TRAJ10	AC114760.2	0.789514546	4.30E-115	postive
TRAJ12	AC114760.2	0.713162184	2.83E-84	postive
TRAJ13	AC114760.2	0.81263641	4.35E-127	postive
TRAJ14	AC114760.2	0.800495443	1.36E-120	postive
TRAJ16	AC114760.2	0.817681637	6.28E-130	postive
TRAJ17	AC114760.2	0.774666113	3.69E-108	postive
TRAJ18	AC114760.2	0.774793638	3.23E-108	postive
TRAJ21	AC114760.2	0.850125777	1.41E-150	postive
TRAJ31	AC114760.2	0.854489473	9.99E-154	postive
TRAJ37	AC114760.2	0.830626478	1.23E-137	postive
TRAJ38	AC114760.2	0.894593091	1.38E-188	postive
TRAJ39	AC114760.2	0.862880557	4.40E-160	postive
GMFG	AC025048.4	0.54253166	2.84E-42	postive
TYROBP	AC025048.4	0.550341855	1.09E-43	postive
LIF	AC004264.1	0.792284683	1.89E-116	postive
LTB4R	AL136295.7	0.539562402	9.57E-42	postive
DDX17	AL136295.7	0.506186889	3.72E-36	postive
IRF9	AL136295.7	0.596504621	7.28E-53	postive
LTB4R2	AL136295.7	0.54947458	1.57E-43	postive
GNRH1	AL136295.7	0.536561887	3.23E-41	postive
CREB1	KDM4A-AS1	0.626419414	1.19E-59	postive
RFXAP	KDM4A-AS1	0.569517153	2.52E-47	postive
UBR1	KDM4A-AS1	0.524613225	3.64E-39	postive
ZC3HAV1	KDM4A-AS1	0.502695943	1.32E-35	postive
IREB2	KDM4A-AS1	0.514080129	2.01E-37	postive
DDX17	KDM4A-AS1	0.64666406	1.11E-64	postive
NFAT5	KDM4A-AS1	0.763017552	4.41E-103	postive
PIK3CA	KDM4A-AS1	0.513951154	2.11E-37	postive
LTB4R2	KDM4A-AS1	0.561277497	9.85E-46	postive
GNRH1	KDM4A-AS1	0.759124959	1.89E-101	postive
ACVR2A	KDM4A-AS1	0.544958901	1.04E-42	postive
ANGPTL1	KDM4A-AS1	0.585533838	1.50E-50	postive
BMPR2	KDM4A-AS1	0.513680125	2.34E-37	postive
CRLF3	KDM4A-AS1	0.548600309	2.27E-43	postive
NR2C1	KDM4A-AS1	0.580279647	1.80E-49	postive
NR2C2	KDM4A-AS1	0.701346004	2.14E-80	postive
RORA	KDM4A-AS1	0.683729733	5.88E-75	postive
SOS1	KDM4A-AS1	0.601388167	6.35E-54	postive
BRAF	KDM4A-AS1	0.720033791	1.27E-86	postive
CBL	KDM4A-AS1	0.523134594	6.45E-39	postive
CBLB	KDM4A-AS1	0.582718507	5.71E-50	postive

TRAJ1	KDM4A-AS1	0.676885443	6.03E-73	postive
TRAJ2	KDM4A-AS1	0.713868081	1.64E-84	postive
TRAJ3	KDM4A-AS1	0.736564416	1.44E-92	postive
TRAJ5	KDM4A-AS1	0.647501379	6.72E-65	postive
TRAJ6	KDM4A-AS1	0.720136152	1.17E-86	postive
TRAJ8	KDM4A-AS1	0.698420271	1.82E-79	postive
TRAJ10	KDM4A-AS1	0.694122208	4.06E-78	postive
TRAJ12	KDM4A-AS1	0.65552942	5.23E-67	postive
TRAJ13	KDM4A-AS1	0.683463428	7.06E-75	postive
TRAJ14	KDM4A-AS1	0.693375524	6.92E-78	postive
TRAJ16	KDM4A-AS1	0.702855035	7.01E-81	postive
TRAJ17	KDM4A-AS1	0.657469992	1.58E-67	postive
TRAJ18	KDM4A-AS1	0.652554777	3.22E-66	postive
TRAJ21	KDM4A-AS1	0.708951976	7.18E-83	postive
TRAJ31	KDM4A-AS1	0.707950542	1.54E-82	postive
TRAJ37	KDM4A-AS1	0.72943607	5.97E-90	postive
TRAJ38	KDM4A-AS1	0.748270965	4.65E-97	postive
TRAJ39	KDM4A-AS1	0.722675857	1.52E-87	postive
LTB4R	LINC00174	0.559714897	1.95E-45	postive
LTB4R2	LINC00174	0.554968208	1.52E-44	postive
CREB1	AL359076.1	0.755303308	7.06E-100	postive
RFXAP	AL359076.1	0.532944247	1.38E-40	postive
UBR1	AL359076.1	0.621452983	1.79E-58	postive
ZC3HAV1	AL359076.1	0.572570953	6.32E-48	postive
IL15	AL359076.1	0.615019151	5.59E-57	postive
CYLD	AL359076.1	0.670923786	3.08E-71	postive
EIF2AK2	AL359076.1	0.54685645	4.72E-43	postive
MAPK8	AL359076.1	0.505393786	4.97E-36	postive
LMBR1	AL359076.1	0.536904494	2.81E-41	postive
LIMS1	AL359076.1	0.502872635	1.24E-35	postive
IREB2	AL359076.1	0.575749117	1.47E-48	postive
DDX17	AL359076.1	0.627125695	8.03E-60	postive
PIK3CG	AL359076.1	0.578181676	4.78E-49	postive
JAK2	AL359076.1	0.679640578	9.50E-74	postive
TXK	AL359076.1	0.627807527	5.51E-60	postive
NFAT5	AL359076.1	0.825446953	1.78E-134	postive
MALT1	AL359076.1	0.571007204	1.29E-47	postive
PIK3R1	AL359076.1	0.714104262	1.36E-84	postive
PIK3CA	AL359076.1	0.698308315	1.98E-79	postive
PIK3CB	AL359076.1	0.532526845	1.63E-40	postive
AKT3	AL359076.1	0.533643447	1.04E-40	postive
RASGRP3	AL359076.1	0.553427203	2.94E-44	postive
IGHD6-6	AL359076.1	0.532137257	1.90E-40	postive
PLXNC1	AL359076.1	0.508792323	1.43E-36	postive
GNRH1	AL359076.1	0.729332953	6.51E-90	postive
IL6ST	AL359076.1	0.67364475	5.17E-72	postive
RABEP1	AL359076.1	0.55944515	2.19E-45	postive
ACVR2A	AL359076.1	0.601883825	4.95E-54	postive
ANGPTL1	AL359076.1	0.764766829	7.96E-104	postive
BMPR1A	AL359076.1	0.505726703	4.40E-36	postive
BMPR2	AL359076.1	0.729126654	7.73E-90	postive
CRLF3	AL359076.1	0.633927567	1.79E-61	postive
IL18R1	AL359076.1	0.505988918	4.00E-36	postive
NR1D2	AL359076.1	0.514559917	1.68E-37	postive
NR2C2	AL359076.1	0.673308067	6.46E-72	postive
RORA	AL359076.1	0.878930104	1.66E-173	postive
SOS1	AL359076.1	0.659339799	4.95E-68	postive
SOS2	AL359076.1	0.563797251	3.25E-46	postive
BRAF	AL359076.1	0.796032062	2.57E-118	postive
ITK	AL359076.1	0.61704182	1.91E-57	postive
CD28	AL359076.1	0.559356762	2.28E-45	postive
CBL	AL359076.1	0.620874619	2.44E-58	postive
CBLB	AL359076.1	0.734170951	1.11E-91	postive
RASGRP1	AL359076.1	0.560772835	1.23E-45	postive
PDK1	AL359076.1	0.589493461	2.25E-51	postive

TRAJ1	AL359076.1	0.695698117	1.31E-78	postive
TRAJ2	AL359076.1	0.734379558	9.32E-92	postive
TRAJ3	AL359076.1	0.779743501	1.81E-110	postive
TRAJ5	AL359076.1	0.779570655	2.17E-110	postive
TRAJ6	AL359076.1	0.753518411	3.74E-99	postive
TRAJ8	AL359076.1	0.714521596	9.84E-85	postive
TRAJ10	AL359076.1	0.744350373	1.58E-95	postive
TRAJ12	AL359076.1	0.686765223	7.25E-76	postive
TRAJ13	AL359076.1	0.753479509	3.88E-99	postive
TRAJ14	AL359076.1	0.734970073	5.63E-92	postive
TRAJ16	AL359076.1	0.744437737	1.46E-95	postive
TRAJ17	AL359076.1	0.725170395	2.01E-88	postive
TRAJ18	AL359076.1	0.73800407	4.16E-93	postive
TRAJ21	AL359076.1	0.790896419	9.11E-116	postive
TRAJ31	AL359076.1	0.812924724	3.01E-127	postive
TRAJ37	AL359076.1	0.793384543	5.41E-117	postive
TRAJ38	AL359076.1	0.832876746	4.81E-139	postive
TRAJ39	AL359076.1	0.811405016	2.08E-126	postive
IRF9	AC003102.1	0.525711762	2.38E-39	postive
NR2C2	AL132657.1	0.604593411	1.25E-54	postive
RORA	AL132657.1	0.516993958	6.73E-38	postive
BRAF	AL132657.1	0.522827961	7.26E-39	postive
CREB1	AC021078.1	0.750330995	7.11E-98	postive
RFXAP	AC021078.1	0.52803558	9.59E-40	postive
UBR1	AC021078.1	0.62653773	1.11E-59	postive
ZC3HAV1	AC021078.1	0.539592417	9.45E-42	postive
ZC3HAV1L	AC021078.1	0.526009632	2.12E-39	postive
IL15	AC021078.1	0.588794597	3.15E-51	postive
CYLD	AC021078.1	0.603347018	2.36E-54	postive
EIF2AK2	AC021078.1	0.5241706	4.33E-39	postive
MAPK8	AC021078.1	0.534073836	8.78E-41	postive
NFKBIZ	AC021078.1	0.593546878	3.13E-52	postive
LMBR1	AC021078.1	0.520726863	1.63E-38	postive
IREB2	AC021078.1	0.570226574	1.83E-47	postive
DDX17	AC021078.1	0.700143902	5.18E-80	postive
PIK3CG	AC021078.1	0.501830112	1.80E-35	postive
JAK2	AC021078.1	0.629372705	2.31E-60	postive
TXK	AC021078.1	0.58637121	1.01E-50	postive
NFAT5	AC021078.1	0.818655036	1.74E-130	postive
MALT1	AC021078.1	0.532024124	1.99E-40	postive
PIK3R1	AC021078.1	0.635464376	7.47E-62	postive
PIK3CA	AC021078.1	0.644290232	4.50E-64	postive
PIK3CB	AC021078.1	0.510082374	8.91E-37	postive
RASGRP3	AC021078.1	0.507014392	2.75E-36	postive
LTB4R2	AC021078.1	0.557608393	4.87E-45	postive
GNRH1	AC021078.1	0.800327166	1.66E-120	postive
IL6ST	AC021078.1	0.656037985	3.83E-67	postive
RABEP1	AC021078.1	0.523951416	4.71E-39	postive
ACVR2A	AC021078.1	0.606825899	3.99E-55	postive
ANGPTL1	AC021078.1	0.742275802	9.95E-95	postive
BMPR2	AC021078.1	0.674718073	2.55E-72	postive
CRLF3	AC021078.1	0.610565558	5.78E-56	postive
NR2C1	AC021078.1	0.533120472	1.28E-40	postive
NR2C2	AC021078.1	0.762566365	6.84E-103	postive
RORA	AC021078.1	0.830465893	1.54E-137	postive
SOS1	AC021078.1	0.65733755	1.72E-67	postive
SOS2	AC021078.1	0.572761915	5.79E-48	postive
BRAF	AC021078.1	0.7964075	1.66E-118	postive
ITK	AC021078.1	0.526264436	1.92E-39	postive
TEC	AC021078.1	0.503504578	9.86E-36	postive
CBL	AC021078.1	0.578958095	3.33E-49	postive
CBLB	AC021078.1	0.732587921	4.25E-91	postive
RASGRP1	AC021078.1	0.523787282	5.02E-39	postive
PDK1	AC021078.1	0.571722863	9.30E-48	postive
TRAJ1	AC021078.1	0.688537874	2.11E-76	postive

TRAJ2	AC021078.1	0.698722194	1.46E-79	postive
TRAJ3	AC021078.1	0.75242261	1.04E-98	postive
TRAJ5	AC021078.1	0.754002988	2.38E-99	postive
TRAJ6	AC021078.1	0.735226116	4.53E-92	postive
TRAJ8	AC021078.1	0.693408861	6.76E-78	postive
TRAJ10	AC021078.1	0.731186397	1.38E-90	postive
TRAJ12	AC021078.1	0.645787378	1.86E-64	postive
TRAJ13	AC021078.1	0.723094337	1.09E-87	postive
TRAJ14	AC021078.1	0.707989357	1.49E-82	postive
TRAJ16	AC021078.1	0.726297673	7.99E-89	postive
TRAJ17	AC021078.1	0.691110396	3.45E-77	postive
TRAJ18	AC021078.1	0.679930552	7.81E-74	postive
TRAJ21	AC021078.1	0.752634339	8.51E-99	postive
TRAJ31	AC021078.1	0.773761404	9.37E-108	postive
TRAJ37	AC021078.1	0.757598627	8.09E-101	postive
TRAJ38	AC021078.1	0.808766626	5.75E-125	postive
TRAJ39	AC021078.1	0.775586889	1.42E-108	postive
CREB1	AC092794.1	0.712401921	5.10E-84	postive
UBR1	AC092794.1	0.587826774	5.02E-51	postive
CYLD	AC092794.1	0.574412157	2.72E-48	postive
MAPK8	AC092794.1	0.529124481	6.26E-40	postive
IREB2	AC092794.1	0.549153491	1.80E-43	postive
DDX17	AC092794.1	0.593184819	3.73E-52	postive
JAK2	AC092794.1	0.588390832	3.82E-51	postive
TXK	AC092794.1	0.513536726	2.47E-37	postive
NFAT5	AC092794.1	0.773638823	1.06E-107	postive
KRAS	AC092794.1	0.507136675	2.63E-36	postive
PIK3R1	AC092794.1	0.535154645	5.69E-41	postive
PIK3CA	AC092794.1	0.596887962	6.02E-53	postive
ROBO2	AC092794.1	0.51948231	2.62E-38	postive
GNRH1	AC092794.1	0.735394111	3.92E-92	postive
IL6ST	AC092794.1	0.523383888	5.86E-39	postive
ACVR2A	AC092794.1	0.603790418	1.88E-54	postive
ANGPTL1	AC092794.1	0.649845942	1.65E-65	postive
BMPR1A	AC092794.1	0.517162058	6.32E-38	postive
BMPR2	AC092794.1	0.663217314	4.34E-69	postive
CRLF3	AC092794.1	0.543297048	2.07E-42	postive
NR2C1	AC092794.1	0.524779502	3.42E-39	postive
NR2C2	AC092794.1	0.642153319	1.58E-63	postive
RORA	AC092794.1	0.748570264	3.54E-97	postive
SOS1	AC092794.1	0.623548644	5.72E-59	postive
SOS2	AC092794.1	0.502760996	1.29E-35	postive
BRAF	AC092794.1	0.685950627	1.27E-75	postive
CBL	AC092794.1	0.501062203	2.38E-35	postive
CBLB	AC092794.1	0.610448612	6.14E-56	postive
TRAJ1	AC092794.1	0.640135663	5.10E-63	postive
TRAJ2	AC092794.1	0.629532275	2.11E-60	postive
TRAJ3	AC092794.1	0.683954858	5.04E-75	postive
TRAJ5	AC092794.1	0.661788828	1.07E-68	postive
TRAJ6	AC092794.1	0.704157509	2.66E-81	postive
TRAJ8	AC092794.1	0.657305	1.75E-67	postive
TRAJ10	AC092794.1	0.689036109	1.49E-76	postive
TRAJ12	AC092794.1	0.648669017	3.35E-65	postive
TRAJ13	AC092794.1	0.680142093	6.77E-74	postive
TRAJ14	AC092794.1	0.655252116	6.20E-67	postive
TRAJ16	AC092794.1	0.690261388	6.29E-77	postive
TRAJ17	AC092794.1	0.66301062	4.94E-69	postive
TRAJ18	AC092794.1	0.651442938	6.31E-66	postive
TRAJ21	AC092794.1	0.699973554	5.87E-80	postive
TRAJ31	AC092794.1	0.728495818	1.30E-89	postive
TRAJ37	AC092794.1	0.711224426	1.26E-83	postive
TRAJ38	AC092794.1	0.747887187	6.59E-97	postive
TRAJ39	AC092794.1	0.691854861	2.04E-77	postive
CREB1	AC004918.5	0.774701174	3.55E-108	postive
UBR1	AC004918.5	0.654456923	1.01E-66	postive

ZC3HAV1	AC004918.5	0.586209756	1.09E-50	postive
ZC3HAV1L	AC004918.5	0.573398499	4.33E-48	postive
IL15	AC004918.5	0.509655917	1.04E-36	postive
CYLD	AC004918.5	0.605062675	9.85E-55	postive
EIF2AK2	AC004918.5	0.53098204	3.00E-40	postive
MAPK8	AC004918.5	0.523590504	5.41E-39	postive
TLR1	AC004918.5	0.565992746	1.23E-46	postive
NFKBIZ	AC004918.5	0.513140931	2.86E-37	postive
LMBR1	AC004918.5	0.648235935	4.34E-65	postive
LIMS1	AC004918.5	0.511822797	4.68E-37	postive
IREB2	AC004918.5	0.592484853	5.26E-52	postive
DDX17	AC004918.5	0.624789684	2.90E-59	postive
PIK3CG	AC004918.5	0.539706942	9.02E-42	postive
JAK2	AC004918.5	0.672047976	1.48E-71	postive
TXK	AC004918.5	0.601961534	4.76E-54	postive
NFAT5	AC004918.5	0.80166162	3.39E-121	postive
MALT1	AC004918.5	0.520903413	1.52E-38	postive
PIK3R1	AC004918.5	0.579876975	2.17E-49	postive
PIK3CA	AC004918.5	0.705766584	7.98E-82	postive
PIK3CB	AC004918.5	0.533648539	1.04E-40	postive
GSK3B	AC004918.5	0.519510553	2.59E-38	postive
RASGRP3	AC004918.5	0.520280973	1.93E-38	postive
GNRH1	AC004918.5	0.759890258	9.08E-102	postive
IL6ST	AC004918.5	0.527808086	1.05E-39	postive
RABEP1	AC004918.5	0.580825968	1.39E-49	postive
ACVR2A	AC004918.5	0.572060849	7.97E-48	postive
ANGPTL1	AC004918.5	0.695797187	1.22E-78	postive
BMPR2	AC004918.5	0.70280607	7.27E-81	postive
CRLF3	AC004918.5	0.616689429	2.30E-57	postive
NR1D2	AC004918.5	0.515701522	1.10E-37	postive
NR2C1	AC004918.5	0.501283267	2.19E-35	postive
NR2C2	AC004918.5	0.703988861	3.02E-81	postive
RORA	AC004918.5	0.780817992	5.75E-111	postive
SOS1	AC004918.5	0.676417124	8.24E-73	postive
SOS2	AC004918.5	0.563494261	3.71E-46	postive
BRAF	AC004918.5	0.747681263	7.94E-97	postive
TEC	AC004918.5	0.535429784	5.10E-41	postive
CBL	AC004918.5	0.625061901	2.50E-59	postive
CBLB	AC004918.5	0.739841108	8.42E-94	postive
PDK1	AC004918.5	0.606012332	6.06E-55	postive
TRAJ1	AC004918.5	0.64349889	7.17E-64	postive
TRAJ2	AC004918.5	0.617849631	1.24E-57	postive
TRAJ3	AC004918.5	0.667661421	2.55E-70	postive
TRAJ5	AC004918.5	0.671505829	2.10E-71	postive
TRAJ6	AC004918.5	0.690204342	6.54E-77	postive
TRAJ8	AC004918.5	0.598446955	2.77E-53	postive
TRAJ10	AC004918.5	0.673621818	5.25E-72	postive
TRAJ12	AC004918.5	0.594998847	1.53E-52	postive
TRAJ13	AC004918.5	0.641481557	2.33E-63	postive
TRAJ14	AC004918.5	0.617366331	1.61E-57	postive
TRAJ16	AC004918.5	0.680861576	4.16E-74	postive
TRAJ17	AC004918.5	0.615366724	4.65E-57	postive
TRAJ18	AC004918.5	0.63506164	9.39E-62	postive
TRAJ21	AC004918.5	0.670517801	4.01E-71	postive
TRAJ31	AC004918.5	0.699842831	6.46E-80	postive
TRAJ37	AC004918.5	0.674642335	2.68E-72	postive
TRAJ38	AC004918.5	0.749130788	2.13E-97	postive
TRAJ39	AC004918.5	0.687458584	4.47E-76	postive
CREB1	AC087222.1	0.605396074	8.31E-55	postive
UBR1	AC087222.1	0.500075012	3.38E-35	postive
DDX17	AC087222.1	0.569787834	2.23E-47	postive
NFAT5	AC087222.1	0.633990384	1.73E-61	postive
PIK3CA	AC087222.1	0.51780881	4.95E-38	postive
GNRH1	AC087222.1	0.695089311	2.03E-78	postive
ANGPTL1	AC087222.1	0.587841614	4.98E-51	postive

BMPR2	AC087222.1	0.549228948	1.75E-43	postive
CRLF3	AC087222.1	0.512462828	3.69E-37	postive
NR2C1	AC087222.1	0.511084065	6.15E-37	postive
NR2C2	AC087222.1	0.662099936	8.78E-69	postive
RORA	AC087222.1	0.578822556	3.55E-49	postive
SOS1	AC087222.1	0.575488565	1.66E-48	postive
BRAF	AC087222.1	0.626419627	1.19E-59	postive
CBL	AC087222.1	0.520479786	1.79E-38	postive
CBLB	AC087222.1	0.592245134	5.91E-52	postive
TRAJ1	AC087222.1	0.631614917	6.59E-61	postive
TRAJ2	AC087222.1	0.509957148	9.33E-37	postive
TRAJ3	AC087222.1	0.612487682	2.12E-56	postive
TRAJ5	AC087222.1	0.641754866	1.99E-63	postive
TRAJ6	AC087222.1	0.636547909	4.02E-62	postive
TRAJ8	AC087222.1	0.610432814	6.19E-56	postive
TRAJ10	AC087222.1	0.605721383	7.04E-55	postive
TRAJ12	AC087222.1	0.604409374	1.37E-54	postive
TRAJ13	AC087222.1	0.625805998	1.66E-59	postive
TRAJ14	AC087222.1	0.616927597	2.03E-57	postive
TRAJ16	AC087222.1	0.633068997	2.91E-61	postive
TRAJ17	AC087222.1	0.615954687	3.40E-57	postive
TRAJ18	AC087222.1	0.552230869	4.90E-44	postive
TRAJ21	AC087222.1	0.634621413	1.21E-61	postive
TRAJ31	AC087222.1	0.646695021	1.09E-64	postive
TRAJ37	AC087222.1	0.595438289	1.23E-52	postive
TRAJ38	AC087222.1	0.66518706	1.24E-69	postive
TRAJ39	AC087222.1	0.62187969	1.42E-58	postive
CREB1	AC002128.2	0.713876482	1.63E-84	postive
UBR1	AC002128.2	0.641881012	1.85E-63	postive
ZC3HAV1	AC002128.2	0.541357442	4.59E-42	postive
ZC3HAV1L	AC002128.2	0.519363773	2.74E-38	postive
CYLD	AC002128.2	0.524267968	4.17E-39	postive
EIF2AK2	AC002128.2	0.508293192	1.72E-36	postive
MAPK8	AC002128.2	0.529217744	6.03E-40	postive
NFKBIZ	AC002128.2	0.521419713	1.25E-38	postive
LMBR1	AC002128.2	0.513720213	2.31E-37	postive
IREB2	AC002128.2	0.56083281	1.20E-45	postive
DDX17	AC002128.2	0.645953068	1.69E-64	postive
JAK2	AC002128.2	0.591510518	8.45E-52	postive
TXK	AC002128.2	0.632583745	3.82E-61	postive
NFAT5	AC002128.2	0.739945145	7.69E-94	postive
MALT1	AC002128.2	0.519920971	2.22E-38	postive
PIK3R1	AC002128.2	0.527615886	1.13E-39	postive
PIK3CA	AC002128.2	0.645425859	2.30E-64	postive
PIK3CB	AC002128.2	0.51597944	9.87E-38	postive
ROBO2	AC002128.2	0.505443733	4.88E-36	postive
GNRH1	AC002128.2	0.816502592	2.95E-129	postive
IL6ST	AC002128.2	0.52461671	3.64E-39	postive
RABEP1	AC002128.2	0.547542502	3.54E-43	postive
ACVR2A	AC002128.2	0.597624455	4.18E-53	postive
ANGPTL1	AC002128.2	0.64290397	1.02E-63	postive
BMPR1A	AC002128.2	0.504547985	6.76E-36	postive
BMPR2	AC002128.2	0.659295547	5.09E-68	postive
CRLF3	AC002128.2	0.582921542	5.19E-50	postive
NR2C1	AC002128.2	0.561774388	7.92E-46	postive
NR2C2	AC002128.2	0.738416551	2.91E-93	postive
RORA	AC002128.2	0.730063376	3.54E-90	postive
SOS1	AC002128.2	0.612071569	2.63E-56	postive
SOS2	AC002128.2	0.511086093	6.15E-37	postive
BRAF	AC002128.2	0.689323035	1.22E-76	postive
TEC	AC002128.2	0.551841845	5.78E-44	postive
CBL	AC002128.2	0.562322815	6.22E-46	postive
CBLB	AC002128.2	0.67042678	4.25E-71	postive
PDK1	AC002128.2	0.570820867	1.40E-47	postive
TRAJ1	AC002128.2	0.587243056	6.64E-51	postive

TRAJ2	AC002128.2	0.524061161	4.51E-39	postive
TRAJ3	AC002128.2	0.602992859	2.82E-54	postive
TRAJ5	AC002128.2	0.610752493	5.24E-56	postive
TRAJ6	AC002128.2	0.617356666	1.61E-57	postive
TRAJ8	AC002128.2	0.540320755	7.02E-42	postive
TRAJ10	AC002128.2	0.601778431	5.22E-54	postive
TRAJ12	AC002128.2	0.528052039	9.53E-40	postive
TRAJ13	AC002128.2	0.573195122	4.75E-48	postive
TRAJ14	AC002128.2	0.540355977	6.92E-42	postive
TRAJ16	AC002128.2	0.599772879	1.43E-53	postive
TRAJ17	AC002128.2	0.554391098	1.95E-44	postive
TRAJ18	AC002128.2	0.55674838	7.07E-45	postive
TRAJ21	AC002128.2	0.603805437	1.87E-54	postive
TRAJ31	AC002128.2	0.634251372	1.49E-61	postive
TRAJ37	AC002128.2	0.605018553	1.01E-54	postive
TRAJ38	AC002128.2	0.660067961	3.14E-68	postive
TRAJ39	AC002128.2	0.632607822	3.77E-61	postive
KRAS	AC092747.4	0.503189906	1.10E-35	postive
CREB1	AC021851.1	0.600905935	8.09E-54	postive
ZC3HAV1L	AC021851.1	0.540362466	6.90E-42	postive
MAPK8	AC021851.1	0.530288426	3.95E-40	postive
IREB2	AC021851.1	0.530940385	3.05E-40	postive
DDX17	AC021851.1	0.524615343	3.64E-39	postive
NFAT5	AC021851.1	0.595891316	9.86E-53	postive
AKT3	AC021851.1	0.503142542	1.12E-35	postive
IGHD4-4	AC021851.1	0.526494502	1.75E-39	postive
GNRH1	AC021851.1	0.657237529	1.83E-67	postive
ACVR2A	AC021851.1	0.542528899	2.84E-42	postive
ANGPTL1	AC021851.1	0.591711205	7.66E-52	postive
BMPR1A	AC021851.1	0.505060586	5.61E-36	postive
BMPR2	AC021851.1	0.500672724	2.73E-35	postive
CRLF3	AC021851.1	0.564683157	2.19E-46	postive
NR2C1	AC021851.1	0.510588115	7.39E-37	postive
NR2C2	AC021851.1	0.630084338	1.55E-60	postive
RORA	AC021851.1	0.530180881	4.12E-40	postive
SOS1	AC021851.1	0.570731023	1.46E-47	postive
BRAF	AC021851.1	0.706385661	5.01E-82	postive
CBLB	AC021851.1	0.517109226	6.45E-38	postive
PDK1	AC021851.1	0.506629157	3.17E-36	postive
TRAJ1	AC021851.1	0.577779639	5.76E-49	postive
TRAJ2	AC021851.1	0.596490324	7.33E-53	postive
TRAJ3	AC021851.1	0.658315154	9.36E-68	postive
TRAJ5	AC021851.1	0.579173328	3.01E-49	postive
TRAJ6	AC021851.1	0.619814052	4.33E-58	postive
TRAJ8	AC021851.1	0.665060049	1.35E-69	postive
TRAJ10	AC021851.1	0.576221678	1.18E-48	postive
TRAJ12	AC021851.1	0.532001474	2.01E-40	postive
TRAJ13	AC021851.1	0.607895686	2.30E-55	postive
TRAJ14	AC021851.1	0.624333688	3.73E-59	postive
TRAJ16	AC021851.1	0.585272666	1.70E-50	postive
TRAJ17	AC021851.1	0.581751923	9.01E-50	postive
TRAJ21	AC021851.1	0.612787993	1.81E-56	postive
TRAJ31	AC021851.1	0.60858633	1.61E-55	postive
TRAJ37	AC021851.1	0.604278311	1.47E-54	postive
TRAJ38	AC021851.1	0.645527576	2.17E-64	postive
TRAJ39	AC021851.1	0.641392562	2.46E-63	postive
WNT5A	WNT5A-AS1	0.735731636	2.94E-92	postive
GNRH1	AC006001.2	0.538025875	1.79E-41	postive
NR2C2	AC006001.2	0.520846928	1.55E-38	postive
BRAF	AC006001.2	0.598152994	3.21E-53	postive
TRAJ3	AC006001.2	0.52235068	8.73E-39	postive
TRAJ8	AC006001.2	0.510882786	6.63E-37	postive
TRAJ39	AC006001.2	0.520203982	1.99E-38	postive
LMBR1L	SNHG12	0.516489792	8.15E-38	postive
IRF9	SNHG12	0.54981314	1.36E-43	postive

PSMD3	TMEM99	0.527621906	1.13E-39	postive
CREB1	AC016831.4	0.787261926	5.26E-114	postive
UBR1	AC016831.4	0.637345133	2.55E-62	postive
ZC3HAV1	AC016831.4	0.567930197	5.15E-47	postive
IL15	AC016831.4	0.599253507	1.85E-53	postive
CYLD	AC016831.4	0.612595951	2.00E-56	postive
EIF2AK2	AC016831.4	0.562330095	6.20E-46	postive
MAPK8	AC016831.4	0.582493488	6.35E-50	postive
LMBR1	AC016831.4	0.595339411	1.29E-52	postive
LIMS1	AC016831.4	0.513228888	2.77E-37	postive
IREB2	AC016831.4	0.615818341	3.66E-57	postive
DDX17	AC016831.4	0.583225041	4.50E-50	postive
PIK3CG	AC016831.4	0.534335309	7.90E-41	postive
JAK2	AC016831.4	0.626333902	1.24E-59	postive
TXK	AC016831.4	0.608315172	1.86E-55	postive
NFAT5	AC016831.4	0.835109164	1.84E-140	postive
MALT1	AC016831.4	0.546692171	5.05E-43	postive
PIK3R1	AC016831.4	0.688591335	2.03E-76	postive
PIK3CA	AC016831.4	0.707906877	1.59E-82	postive
PIK3CB	AC016831.4	0.543611664	1.82E-42	postive
AKT3	AC016831.4	0.542347695	3.06E-42	postive
RASGRP3	AC016831.4	0.530615589	3.47E-40	postive
IGHD6-6	AC016831.4	0.500852176	2.56E-35	postive
GNRH1	AC016831.4	0.695939414	1.10E-78	postive
IL6ST	AC016831.4	0.686262927	1.03E-75	postive
RABEP1	AC016831.4	0.543299017	2.07E-42	postive
ACVR2A	AC016831.4	0.646217167	1.44E-64	postive
ANGPTL1	AC016831.4	0.81631978	3.75E-129	postive
BMPR1A	AC016831.4	0.556013177	9.71E-45	postive
BMPR2	AC016831.4	0.762386431	8.15E-103	postive
CRLF3	AC016831.4	0.63007324	1.56E-60	postive
NR1D2	AC016831.4	0.52932805	5.77E-40	postive
NR2C2	AC016831.4	0.658226381	9.89E-68	postive
RORA	AC016831.4	0.88836923	2.52E-182	postive
SOS1	AC016831.4	0.684787837	2.84E-75	postive
SOS2	AC016831.4	0.585244778	1.72E-50	postive
BRAF	AC016831.4	0.834437224	4.94E-140	postive
ITK	AC016831.4	0.568799912	3.49E-47	postive
CD28	AC016831.4	0.52110221	1.41E-38	postive
CBL	AC016831.4	0.588223988	4.14E-51	postive
CBLB	AC016831.4	0.737689584	5.45E-93	postive
RASGRP1	AC016831.4	0.51735959	5.86E-38	postive
PDK1	AC016831.4	0.59080468	1.19E-51	postive
TRAJ1	AC016831.4	0.710590817	2.05E-83	postive
TRAJ2	AC016831.4	0.72919163	7.32E-90	postive
TRAJ3	AC016831.4	0.782103262	1.45E-111	postive
TRAJ5	AC016831.4	0.836975008	1.16E-141	postive
TRAJ6	AC016831.4	0.783761002	2.43E-112	postive
TRAJ8	AC016831.4	0.782598868	8.52E-112	postive
TRAJ10	AC016831.4	0.772011354	5.63E-107	postive
TRAJ12	AC016831.4	0.726620789	6.13E-89	postive
TRAJ13	AC016831.4	0.825167827	2.61E-134	postive
TRAJ14	AC016831.4	0.791567403	4.27E-116	postive
TRAJ16	AC016831.4	0.782065533	1.51E-111	postive
TRAJ17	AC016831.4	0.773766217	9.33E-108	postive
TRAJ18	AC016831.4	0.743021548	5.14E-95	postive
TRAJ21	AC016831.4	0.848896929	1.04E-149	postive
TRAJ31	AC016831.4	0.873112409	1.98E-168	postive
TRAJ37	AC016831.4	0.837244875	7.76E-142	postive
TRAJ38	AC016831.4	0.877542034	2.85E-172	postive
TRAJ39	AC016831.4	0.852877007	1.50E-152	postive
PRKCQ	PRKCQ-AS1	0.79002314	2.43E-115	postive
CREB1	AC090579.1	0.728065598	1.86E-89	postive
RFXAP	AC090579.1	0.518813545	3.38E-38	postive
UBR1	AC090579.1	0.640981268	3.12E-63	postive

ZC3HAV1	AC090579.1	0.536774971	2.96E-41	postive
CYLD	AC090579.1	0.540574036	6.33E-42	postive
EIF2AK2	AC090579.1	0.552448743	4.47E-44	postive
MAPK8	AC090579.1	0.508724241	1.47E-36	postive
LMBR1	AC090579.1	0.551453295	6.82E-44	postive
IREB2	AC090579.1	0.576555731	1.02E-48	postive
DDX17	AC090579.1	0.599109741	1.99E-53	postive
PIK3CG	AC090579.1	0.514085651	2.01E-37	postive
JAK2	AC090579.1	0.59089599	1.14E-51	postive
TXK	AC090579.1	0.607720705	2.52E-55	postive
NFAT5	AC090579.1	0.750702223	5.06E-98	postive
PIK3R1	AC090579.1	0.653231407	2.13E-66	postive
PIK3CA	AC090579.1	0.623312793	6.51E-59	postive
IGHD4-4	AC090579.1	0.509113847	1.27E-36	postive
IGHD6-6	AC090579.1	0.512291302	3.93E-37	postive
GNRH1	AC090579.1	0.729874862	4.14E-90	postive
IL6ST	AC090579.1	0.606120373	5.74E-55	postive
RABEP1	AC090579.1	0.526170878	1.99E-39	postive
ACVR2A	AC090579.1	0.603929554	1.75E-54	postive
ANGPTL1	AC090579.1	0.69065067	4.78E-77	postive
BMPR1A	AC090579.1	0.513904513	2.15E-37	postive
BMPR2	AC090579.1	0.678544769	1.99E-73	postive
CRLF3	AC090579.1	0.609359549	1.08E-55	postive
NR1D2	AC090579.1	0.50410172	7.94E-36	postive
NR2C2	AC090579.1	0.708157388	1.31E-82	postive
NR3C2	AC090579.1	0.50462888	6.56E-36	postive
RORA	AC090579.1	0.78069196	6.58E-111	postive
SOS1	AC090579.1	0.633308778	2.54E-61	postive
SOS2	AC090579.1	0.542047504	3.46E-42	postive
BRAF	AC090579.1	0.790197073	2.00E-115	postive
TEC	AC090579.1	0.598122651	3.26E-53	postive
CBL	AC090579.1	0.525340442	2.75E-39	postive
CBLB	AC090579.1	0.705434977	1.02E-81	postive
RASGRP1	AC090579.1	0.537098322	2.60E-41	postive
PDK1	AC090579.1	0.56409223	2.85E-46	postive
TRAJ1	AC090579.1	0.642929517	1.00E-63	postive
TRAJ2	AC090579.1	0.61414741	8.85E-57	postive
TRAJ3	AC090579.1	0.683324059	7.77E-75	postive
TRAJ5	AC090579.1	0.714681473	8.69E-85	postive
TRAJ6	AC090579.1	0.677435686	4.18E-73	postive
TRAJ8	AC090579.1	0.642657828	1.17E-63	postive
TRAJ10	AC090579.1	0.63890582	1.04E-62	postive
TRAJ12	AC090579.1	0.646156489	1.50E-64	postive
TRAJ13	AC090579.1	0.650066448	1.45E-65	postive
TRAJ14	AC090579.1	0.662178894	8.36E-69	postive
TRAJ16	AC090579.1	0.655825825	4.36E-67	postive
TRAJ17	AC090579.1	0.619878586	4.18E-58	postive
TRAJ18	AC090579.1	0.632613206	3.76E-61	postive
TRAJ21	AC090579.1	0.697363536	3.93E-79	postive
TRAJ31	AC090579.1	0.711838651	7.87E-84	postive
TRAJ37	AC090579.1	0.691248101	3.13E-77	postive
TRAJ38	AC090579.1	0.752086236	1.41E-98	postive
TRAJ39	AC090579.1	0.744849025	1.01E-95	postive
CREB1	AC026355.2	0.638801866	1.10E-62	postive
UBR1	AC026355.2	0.520844749	1.56E-38	postive
IL15	AC026355.2	0.546685585	5.07E-43	postive
IREB2	AC026355.2	0.501145909	2.31E-35	postive
JAK2	AC026355.2	0.503702131	9.18E-36	postive
NFAT5	AC026355.2	0.692934586	9.47E-78	postive
PIK3R1	AC026355.2	0.535891232	4.23E-41	postive
PIK3CA	AC026355.2	0.562778648	5.09E-46	postive
GNRH1	AC026355.2	0.572454331	6.67E-48	postive
IL6ST	AC026355.2	0.557959969	4.19E-45	postive
ACVR2A	AC026355.2	0.529036596	6.48E-40	postive
ANGPTL1	AC026355.2	0.662199573	8.25E-69	postive

BMPR2	AC026355.2	0.574677051	2.41E-48	postive
CRLF3	AC026355.2	0.537937922	1.85E-41	postive
NR2C2	AC026355.2	0.564588647	2.29E-46	postive
RORA	AC026355.2	0.720033385	1.27E-86	postive
SOS1	AC026355.2	0.554478327	1.88E-44	postive
SOS2	AC026355.2	0.501690974	1.90E-35	postive
BRAF	AC026355.2	0.680602348	4.96E-74	postive
CBLB	AC026355.2	0.584877897	2.05E-50	postive
PDK1	AC026355.2	0.505770373	4.33E-36	postive
TRAJ1	AC026355.2	0.611531699	3.49E-56	postive
TRAJ2	AC026355.2	0.610997698	4.61E-56	postive
TRAJ3	AC026355.2	0.662674034	6.12E-69	postive
TRAJ5	AC026355.2	0.727575901	2.79E-89	postive
TRAJ6	AC026355.2	0.67148398	2.14E-71	postive
TRAJ8	AC026355.2	0.663783255	3.03E-69	postive
TRAJ10	AC026355.2	0.697467902	3.65E-79	postive
TRAJ12	AC026355.2	0.607410809	2.96E-55	postive
TRAJ13	AC026355.2	0.697585189	3.35E-79	postive
TRAJ14	AC026355.2	0.672843059	8.77E-72	postive
TRAJ16	AC026355.2	0.651772137	5.17E-66	postive
TRAJ17	AC026355.2	0.663989705	2.66E-69	postive
TRAJ18	AC026355.2	0.61205125	2.66E-56	postive
TRAJ21	AC026355.2	0.704921661	1.50E-81	postive
TRAJ31	AC026355.2	0.752653641	8.36E-99	postive
TRAJ37	AC026355.2	0.727131432	4.03E-89	postive
TRAJ38	AC026355.2	0.745296548	6.79E-96	postive
TRAJ39	AC026355.2	0.724438098	3.65E-88	postive
CREB1	AP000786.1	0.653963034	1.36E-66	postive
UBR1	AP000786.1	0.556007891	9.73E-45	postive
ZC3HAV1	AP000786.1	0.525084668	3.03E-39	postive
CYLD	AP000786.1	0.525420332	2.66E-39	postive
EIF2AK2	AP000786.1	0.530877175	3.13E-40	postive
MAPK8	AP000786.1	0.508671435	1.50E-36	postive
LMBR1	AP000786.1	0.503490224	9.91E-36	postive
IREB2	AP000786.1	0.528722039	7.33E-40	postive
DDX17	AP000786.1	0.576656273	9.69E-49	postive
TXK	AP000786.1	0.576209519	1.19E-48	postive
NFAT5	AP000786.1	0.700461018	4.10E-80	postive
PIK3R1	AP000786.1	0.600775457	8.64E-54	postive
PIK3CA	AP000786.1	0.59330515	3.52E-52	postive
AKT3	AP000786.1	0.519984532	2.16E-38	postive
GNRH1	AP000786.1	0.620072829	3.76E-58	postive
IL6ST	AP000786.1	0.620091475	3.73E-58	postive
ACVR2A	AP000786.1	0.538798845	1.31E-41	postive
ANGPTL1	AP000786.1	0.691653462	2.35E-77	postive
BMPR2	AP000786.1	0.660407833	2.54E-68	postive
CRLF3	AP000786.1	0.56763096	5.89E-47	postive
LIFR	AP000786.1	0.502565319	1.38E-35	postive
NR2C2	AP000786.1	0.591944438	6.84E-52	postive
RORA	AP000786.1	0.778227738	8.97E-110	postive
SOS1	AP000786.1	0.613347953	1.35E-56	postive
BRAF	AP000786.1	0.758838675	2.49E-101	postive
ITK	AP000786.1	0.525080486	3.04E-39	postive
CBL	AP000786.1	0.570698743	1.48E-47	postive
CBLB	AP000786.1	0.648743136	3.20E-65	postive
TRAJ1	AP000786.1	0.536939901	2.77E-41	postive
TRAJ2	AP000786.1	0.608967266	1.32E-55	postive
TRAJ3	AP000786.1	0.605095913	9.69E-55	postive
TRAJ5	AP000786.1	0.660831755	1.95E-68	postive
TRAJ6	AP000786.1	0.584798185	2.13E-50	postive
TRAJ8	AP000786.1	0.610502036	5.97E-56	postive
TRAJ10	AP000786.1	0.535040766	5.96E-41	postive
TRAJ12	AP000786.1	0.516191002	9.12E-38	postive
TRAJ13	AP000786.1	0.667240462	3.34E-70	postive
TRAJ14	AP000786.1	0.607147397	3.39E-55	postive

TRAJ16	AP000786.1	0.573690117	3.79E-48	postive
TRAJ17	AP000786.1	0.543357979	2.02E-42	postive
TRAJ18	AP000786.1	0.535202158	5.58E-41	postive
TRAJ21	AP000786.1	0.667802206	2.33E-70	postive
TRAJ31	AP000786.1	0.670080081	5.33E-71	postive
TRAJ37	AP000786.1	0.653904841	1.41E-66	postive
TRAJ38	AP000786.1	0.679626662	9.59E-74	postive
TRAJ39	AP000786.1	0.663304609	4.11E-69	postive
CREB1	MBNL1-AS1	0.647748364	5.80E-65	postive
UBR1	MBNL1-AS1	0.507558887	2.25E-36	postive
CYLD	MBNL1-AS1	0.585889066	1.27E-50	postive
DDX17	MBNL1-AS1	0.541701172	3.99E-42	postive
JAK2	MBNL1-AS1	0.527416402	1.22E-39	postive
NFAT5	MBNL1-AS1	0.731482909	1.08E-90	postive
NFATC3	MBNL1-AS1	0.510198242	8.54E-37	postive
MALT1	MBNL1-AS1	0.500967196	2.46E-35	postive
PIK3R1	MBNL1-AS1	0.656102717	3.68E-67	postive
PIK3CA	MBNL1-AS1	0.619120686	6.28E-58	postive
GNRH1	MBNL1-AS1	0.550302967	1.11E-43	postive
IL6ST	MBNL1-AS1	0.624653483	3.13E-59	postive
ACVR2A	MBNL1-AS1	0.536348374	3.52E-41	postive
ANGPTL1	MBNL1-AS1	0.638063685	1.69E-62	postive
BMPR2	MBNL1-AS1	0.654593958	9.28E-67	postive
NR2C2	MBNL1-AS1	0.565236299	1.72E-46	postive
RORA	MBNL1-AS1	0.750781076	4.70E-98	postive
SOS1	MBNL1-AS1	0.597076638	5.48E-53	postive
SOS2	MBNL1-AS1	0.501419984	2.09E-35	postive
BRAF	MBNL1-AS1	0.673594973	5.35E-72	postive
CBLB	MBNL1-AS1	0.602356273	3.90E-54	postive
TRAJ1	MBNL1-AS1	0.636307844	4.61E-62	postive
TRAJ2	MBNL1-AS1	0.622912832	8.09E-59	postive
TRAJ3	MBNL1-AS1	0.659343831	4.94E-68	postive
TRAJ5	MBNL1-AS1	0.714998625	6.78E-85	postive
TRAJ6	MBNL1-AS1	0.676932668	5.85E-73	postive
TRAJ8	MBNL1-AS1	0.687795423	3.54E-76	postive
TRAJ10	MBNL1-AS1	0.661427308	1.34E-68	postive
TRAJ12	MBNL1-AS1	0.635197657	8.69E-62	postive
TRAJ13	MBNL1-AS1	0.720408186	9.43E-87	postive
TRAJ14	MBNL1-AS1	0.687164843	5.49E-76	postive
TRAJ16	MBNL1-AS1	0.692864933	9.95E-78	postive
TRAJ17	MBNL1-AS1	0.674382951	3.18E-72	postive
TRAJ18	MBNL1-AS1	0.662694784	6.04E-69	postive
TRAJ21	MBNL1-AS1	0.732059286	6.64E-91	postive
TRAJ31	MBNL1-AS1	0.734076579	1.21E-91	postive
TRAJ37	MBNL1-AS1	0.722361707	1.96E-87	postive
TRAJ38	MBNL1-AS1	0.730869304	1.80E-90	postive
TRAJ39	MBNL1-AS1	0.739808855	8.66E-94	postive
CREB1	AC068790.5	0.783963812	1.95E-112	postive
RFXAP	AC068790.5	0.558009712	4.10E-45	postive
UBR1	AC068790.5	0.664404817	2.04E-69	postive
ZC3HAV1	AC068790.5	0.584238557	2.78E-50	postive
ZC3HAV1L	AC068790.5	0.50430027	7.39E-36	postive
IL15	AC068790.5	0.568024707	4.94E-47	postive
CYLD	AC068790.5	0.629035695	2.79E-60	postive
EIF2AK2	AC068790.5	0.54963843	1.47E-43	postive
MAPK8	AC068790.5	0.568093594	4.79E-47	postive
LMBR1	AC068790.5	0.567809012	5.44E-47	postive
IREB2	AC068790.5	0.61187684	2.92E-56	postive
DDX17	AC068790.5	0.644506575	3.96E-64	postive
PIK3CG	AC068790.5	0.513135775	2.87E-37	postive
JAK2	AC068790.5	0.619370519	5.49E-58	postive
TXK	AC068790.5	0.573721613	3.74E-48	postive
NFAT5	AC068790.5	0.897054524	3.58E-191	postive
NFATC3	AC068790.5	0.553545002	2.80E-44	postive
MALT1	AC068790.5	0.53732348	2.37E-41	postive

PIK3R1	AC068790.5	0.631387671	7.49E-61	postive
PIK3CA	AC068790.5	0.675565529	1.45E-72	postive
PIK3CB	AC068790.5	0.528465773	8.10E-40	postive
AKT3	AC068790.5	0.546128462	6.39E-43	postive
RASGRP3	AC068790.5	0.513049	2.96E-37	postive
IGHD4-4	AC068790.5	0.520743601	1.62E-38	postive
IGHD6-6	AC068790.5	0.527976815	9.82E-40	postive
GNRH1	AC068790.5	0.781494946	2.79E-111	postive
IL6ST	AC068790.5	0.626372534	1.22E-59	postive
RABEP1	AC068790.5	0.573602913	3.95E-48	postive
ACVR2A	AC068790.5	0.66292079	5.23E-69	postive
ANGPTL1	AC068790.5	0.740381897	5.25E-94	postive
BMPR1A	AC068790.5	0.565380572	1.61E-46	postive
BMPR2	AC068790.5	0.711341671	1.15E-83	postive
CRLF3	AC068790.5	0.661980919	9.47E-69	postive
NR1D2	AC068790.5	0.517723301	5.11E-38	postive
NR2C1	AC068790.5	0.519651416	2.46E-38	postive
NR2C2	AC068790.5	0.740819223	3.58E-94	postive
RORA	AC068790.5	0.848765162	1.29E-149	postive
SOS1	AC068790.5	0.69441391	3.29E-78	postive
SOS2	AC068790.5	0.606172508	5.59E-55	postive
BRAF	AC068790.5	0.834013024	9.19E-140	postive
CBL	AC068790.5	0.603821575	1.85E-54	postive
CBLB	AC068790.5	0.663933498	2.76E-69	postive
RASGRP1	AC068790.5	0.541004711	5.31E-42	postive
PDK1	AC068790.5	0.560811752	1.21E-45	postive
TRAJ1	AC068790.5	0.833262426	2.74E-139	postive
TRAJ2	AC068790.5	0.777566519	1.80E-109	postive
TRAJ3	AC068790.5	0.864694426	1.64E-161	postive
TRAJ5	AC068790.5	0.874601738	1.05E-169	postive
TRAJ6	AC068790.5	0.881668139	5.50E-176	postive
TRAJ8	AC068790.5	0.874768531	7.54E-170	postive
TRAJ10	AC068790.5	0.861321525	7.17E-159	postive
TRAJ12	AC068790.5	0.818287421	2.83E-130	postive
TRAJ13	AC068790.5	0.884499	1.29E-178	postive
TRAJ14	AC068790.5	0.867157384	1.73E-163	postive
TRAJ16	AC068790.5	0.866122659	1.18E-162	postive
TRAJ17	AC068790.5	0.856976881	1.44E-155	postive
TRAJ18	AC068790.5	0.806220256	1.34E-123	postive
TRAJ21	AC068790.5	0.902326992	6.13E-197	postive
TRAJ31	AC068790.5	0.911753934	4.05E-208	postive
TRAJ37	AC068790.5	0.902588664	3.11E-197	postive
TRAJ38	AC068790.5	0.917458509	1.64E-215	postive
TRAJ39	AC068790.5	0.920208838	2.87E-219	postive
LMBR1L	LINC01089	0.547064412	4.33E-43	postive
IRF9	LINC01089	0.59753553	4.37E-53	postive
TNFRSF14	LINC01089	0.508502258	1.60E-36	postive
TNFRSF25	LINC01089	0.519323555	2.78E-38	postive
CREB1	AL606834.2	0.682505231	1.36E-74	postive
ZC3HAV1	AL606834.2	0.53016756	4.15E-40	postive
IL15	AL606834.2	0.513997106	2.08E-37	postive
CYLD	AL606834.2	0.654629118	9.08E-67	postive
DDX17	AL606834.2	0.573724775	3.73E-48	postive
PIK3CG	AL606834.2	0.515219253	1.31E-37	postive
JAK2	AL606834.2	0.606930581	3.79E-55	postive
TXK	AL606834.2	0.538610959	1.41E-41	postive
NFAT5	AL606834.2	0.741478017	2.01E-94	postive
NFATC2	AL606834.2	0.557497353	5.11E-45	postive
MALT1	AL606834.2	0.505486495	4.80E-36	postive
PIK3R1	AL606834.2	0.644628764	3.69E-64	postive
PIK3CA	AL606834.2	0.627275177	7.40E-60	postive
RASGRP3	AL606834.2	0.542324371	3.09E-42	postive
PLXNC1	AL606834.2	0.517655767	5.24E-38	postive
GNRH1	AL606834.2	0.594713386	1.76E-52	postive
IL6ST	AL606834.2	0.587232561	6.67E-51	postive

RABEP1	AL606834.2	0.51047719	7.70E-37	postive
ACVR2A	AL606834.2	0.549386661	1.63E-43	postive
ANGPTL1	AL606834.2	0.670537781	3.96E-71	postive
BMPR2	AL606834.2	0.663293867	4.13E-69	postive
CRLF3	AL606834.2	0.543995403	1.55E-42	postive
NR2C2	AL606834.2	0.569892771	2.13E-47	postive
RORA	AL606834.2	0.783115251	4.88E-112	postive
SOS1	AL606834.2	0.623443054	6.06E-59	postive
SOS2	AL606834.2	0.604560243	1.27E-54	postive
BRAF	AL606834.2	0.701308694	2.20E-80	postive
PTPRC	AL606834.2	0.51110491	6.10E-37	postive
ITK	AL606834.2	0.637515376	2.31E-62	postive
CD28	AL606834.2	0.583225867	4.49E-50	postive
CBL	AL606834.2	0.580664641	1.50E-49	postive
CBLB	AL606834.2	0.674228712	3.52E-72	postive
TRAJ1	AL606834.2	0.682832211	1.09E-74	postive
TRAJ2	AL606834.2	0.689253544	1.28E-76	postive
TRAJ3	AL606834.2	0.701270845	2.26E-80	postive
TRAJ5	AL606834.2	0.756377071	2.57E-100	postive
TRAJ6	AL606834.2	0.69585037	1.17E-78	postive
TRAJ8	AL606834.2	0.689351841	1.19E-76	postive
TRAJ10	AL606834.2	0.674312129	3.33E-72	postive
TRAJ12	AL606834.2	0.658985922	6.17E-68	postive
TRAJ13	AL606834.2	0.739959501	7.60E-94	postive
TRAJ14	AL606834.2	0.707853248	1.65E-82	postive
TRAJ16	AL606834.2	0.715469081	4.69E-85	postive
TRAJ17	AL606834.2	0.683446763	7.14E-75	postive
TRAJ18	AL606834.2	0.691182195	3.28E-77	postive
TRAJ21	AL606834.2	0.746220521	2.96E-96	postive
TRAJ31	AL606834.2	0.769195509	9.74E-106	postive
TRAJ37	AL606834.2	0.740622612	4.25E-94	postive
TRAJ38	AL606834.2	0.765485302	3.92E-104	postive
TRAJ39	AL606834.2	0.746427946	2.46E-96	postive
CREB1	BTBD9-AS1	0.773555015	1.16E-107	postive
RFXAP	BTBD9-AS1	0.512232383	4.02E-37	postive
UBR1	BTBD9-AS1	0.640562304	3.98E-63	postive
ZC3HAV1	BTBD9-AS1	0.555736294	1.09E-44	postive
IL15	BTBD9-AS1	0.542110126	3.37E-42	postive
CYLD	BTBD9-AS1	0.619593761	4.87E-58	postive
EIF2AK2	BTBD9-AS1	0.544034638	1.53E-42	postive
MAPK8	BTBD9-AS1	0.55723102	5.74E-45	postive
LMBR1	BTBD9-AS1	0.568131994	4.71E-47	postive
IREB2	BTBD9-AS1	0.605097137	9.68E-55	postive
DDX17	BTBD9-AS1	0.588449262	3.72E-51	postive
PIK3CG	BTBD9-AS1	0.507591173	2.23E-36	postive
JAK2	BTBD9-AS1	0.594960956	1.56E-52	postive
TXK	BTBD9-AS1	0.576554541	1.02E-48	postive
NFAT5	BTBD9-AS1	0.878659313	2.90E-173	postive
NFATC3	BTBD9-AS1	0.546712294	5.01E-43	postive
MALT1	BTBD9-AS1	0.526671358	1.64E-39	postive
PIK3R1	BTBD9-AS1	0.663915525	2.79E-69	postive
PIK3CA	BTBD9-AS1	0.672299397	1.25E-71	postive
PIK3CB	BTBD9-AS1	0.504195616	7.68E-36	postive
AKT3	BTBD9-AS1	0.507406716	2.38E-36	postive
RASGRP3	BTBD9-AS1	0.51666463	7.63E-38	postive
IGHD4-4	BTBD9-AS1	0.537942742	1.85E-41	postive
IGHD6-6	BTBD9-AS1	0.541938507	3.62E-42	postive
GNRH1	BTBD9-AS1	0.710976426	1.53E-83	postive
IL6ST	BTBD9-AS1	0.676129079	9.99E-73	postive
RABEP1	BTBD9-AS1	0.543571189	1.85E-42	postive
ACVR2A	BTBD9-AS1	0.67824637	2.43E-73	postive
ANGPTL1	BTBD9-AS1	0.725933945	1.08E-88	postive
BMPR1A	BTBD9-AS1	0.567189483	7.18E-47	postive
BMPR2	BTBD9-AS1	0.738739715	2.20E-93	postive
CRLF3	BTBD9-AS1	0.624791617	2.90E-59	postive

NR1D2	BTBD9-AS1	0.523242702	6.19E-39	postive
NR2C2	BTBD9-AS1	0.678382033	2.22E-73	postive
RORA	BTBD9-AS1	0.88473487	7.73E-179	postive
SOS1	BTBD9-AS1	0.670973033	2.98E-71	postive
SOS2	BTBD9-AS1	0.581235779	1.15E-49	postive
BRAF	BTBD9-AS1	0.803461598	3.88E-122	postive
ITK	BTBD9-AS1	0.506539037	3.27E-36	postive
CD28	BTBD9-AS1	0.512248784	3.99E-37	postive
CBL	BTBD9-AS1	0.541699348	3.99E-42	postive
CBLB	BTBD9-AS1	0.640606194	3.88E-63	postive
RASGRP1	BTBD9-AS1	0.514907316	1.48E-37	postive
PDK1	BTBD9-AS1	0.538399326	1.54E-41	postive
TRAJ1	BTBD9-AS1	0.782695844	7.67E-112	postive
TRAJ2	BTBD9-AS1	0.748891678	2.65E-97	postive
TRAJ3	BTBD9-AS1	0.818227473	3.06E-130	postive
TRAJ5	BTBD9-AS1	0.872776476	3.82E-168	postive
TRAJ6	BTBD9-AS1	0.835525055	9.97E-141	postive
TRAJ8	BTBD9-AS1	0.851608386	1.23E-151	postive
TRAJ10	BTBD9-AS1	0.827134487	1.70E-135	postive
TRAJ12	BTBD9-AS1	0.778361993	7.78E-110	postive
TRAJ13	BTBD9-AS1	0.866075774	1.29E-162	postive
TRAJ14	BTBD9-AS1	0.826560898	3.79E-135	postive
TRAJ16	BTBD9-AS1	0.841495198	1.24E-144	postive
TRAJ17	BTBD9-AS1	0.834479763	4.64E-140	postive
TRAJ18	BTBD9-AS1	0.779950001	1.45E-110	postive
TRAJ21	BTBD9-AS1	0.879023211	1.37E-173	postive
TRAJ31	BTBD9-AS1	0.914151934	3.66E-211	postive
TRAJ37	BTBD9-AS1	0.901950251	1.62E-196	postive
TRAJ38	BTBD9-AS1	0.892397235	2.47E-186	postive
TRAJ39	BTBD9-AS1	0.90965479	1.59E-205	postive
CREB1	AC018690.1	0.63999911	5.52E-63	postive
RFXAP	AC018690.1	0.511704867	4.89E-37	postive
UBR1	AC018690.1	0.558567618	3.22E-45	postive
ZC3HAV1	AC018690.1	0.529154004	6.18E-40	postive
ZC3HAV1L	AC018690.1	0.5056368	4.55E-36	postive
CYLD	AC018690.1	0.511877807	4.58E-37	postive
EIF2AK2	AC018690.1	0.51202004	4.35E-37	postive
IREB2	AC018690.1	0.536676006	3.08E-41	postive
DDX17	AC018690.1	0.665527467	1.00E-69	postive
JAK2	AC018690.1	0.51543175	1.21E-37	postive
TXK	AC018690.1	0.51554868	1.16E-37	postive
NFAT5	AC018690.1	0.740230505	5.99E-94	postive
PIK3CA	AC018690.1	0.548275051	2.61E-43	postive
GNRH1	AC018690.1	0.783327937	3.88E-112	postive
RABEP1	AC018690.1	0.500485382	2.92E-35	postive
ACVR2A	AC018690.1	0.57755596	6.39E-49	postive
ANGPTL1	AC018690.1	0.593394618	3.37E-52	postive
BMPR2	AC018690.1	0.551620968	6.35E-44	postive
NR2C1	AC018690.1	0.567433512	6.44E-47	postive
NR2C2	AC018690.1	0.754106483	2.16E-99	postive
RORA	AC018690.1	0.659985852	3.31E-68	postive
SOS1	AC018690.1	0.633631324	2.11E-61	postive
BRAF	AC018690.1	0.679936849	7.78E-74	postive
CBL	AC018690.1	0.531420344	2.53E-40	postive
CBLB	AC018690.1	0.615452713	4.44E-57	postive
TRAJ1	AC018690.1	0.665310074	1.15E-69	postive
TRAJ2	AC018690.1	0.638002887	1.75E-62	postive
TRAJ3	AC018690.1	0.689676609	9.49E-77	postive
TRAJ5	AC018690.1	0.636549643	4.02E-62	postive
TRAJ6	AC018690.1	0.689527111	1.05E-76	postive
TRAJ8	AC018690.1	0.627821035	5.47E-60	postive
TRAJ10	AC018690.1	0.673291837	6.53E-72	postive
TRAJ12	AC018690.1	0.647403421	7.13E-65	postive
TRAJ13	AC018690.1	0.632903577	3.19E-61	postive
TRAJ14	AC018690.1	0.647395939	7.16E-65	postive

TRAJ16	AC018690.1	0.677773385	3.33E-73	postive
TRAJ17	AC018690.1	0.631391206	7.47E-61	postive
TRAJ18	AC018690.1	0.6506401	1.02E-65	postive
TRAJ21	AC018690.1	0.666974069	3.96E-70	postive
TRAJ31	AC018690.1	0.67215807	1.37E-71	postive
TRAJ37	AC018690.1	0.666529353	5.27E-70	postive
TRAJ38	AC018690.1	0.712190681	6.00E-84	postive
TRAJ39	AC018690.1	0.676428312	8.18E-73	postive
CREB1	KIF26B-AS1	0.687323086	4.92E-76	postive
UBR1	KIF26B-AS1	0.574719371	2.37E-48	postive
ZC3HAV1	KIF26B-AS1	0.505453451	4.86E-36	postive
ZC3HAV1L	KIF26B-AS1	0.538171737	1.68E-41	postive
IL15	KIF26B-AS1	0.582714884	5.72E-50	postive
MAPK8	KIF26B-AS1	0.509031366	1.31E-36	postive
LMBR1	KIF26B-AS1	0.53236969	1.73E-40	postive
IREB2	KIF26B-AS1	0.573167145	4.82E-48	postive
DDX17	KIF26B-AS1	0.517381433	5.82E-38	postive
PIK3CG	KIF26B-AS1	0.509248181	1.21E-36	postive
JAK2	KIF26B-AS1	0.568265608	4.43E-47	postive
TXK	KIF26B-AS1	0.53489872	6.31E-41	postive
NFAT5	KIF26B-AS1	0.651979815	4.56E-66	postive
MALT1	KIF26B-AS1	0.508586662	1.55E-36	postive
PIK3R1	KIF26B-AS1	0.595426835	1.24E-52	postive
PIK3CA	KIF26B-AS1	0.630744371	1.07E-60	postive
RASGRP3	KIF26B-AS1	0.506933563	2.83E-36	postive
IGHD4-4	KIF26B-AS1	0.57226512	7.27E-48	postive
IGHD6-6	KIF26B-AS1	0.501193752	2.27E-35	postive
LTB4R2	KIF26B-AS1	0.503131549	1.13E-35	postive
FGF7	KIF26B-AS1	0.505001556	5.73E-36	postive
GNRH1	KIF26B-AS1	0.648302142	4.17E-65	postive
IL6ST	KIF26B-AS1	0.577794633	5.72E-49	postive
RABEP1	KIF26B-AS1	0.513585888	2.42E-37	postive
ACVR2A	KIF26B-AS1	0.579235945	2.93E-49	postive
ANGPTL1	KIF26B-AS1	0.726739073	5.56E-89	postive
BMPR2	KIF26B-AS1	0.655975796	3.97E-67	postive
CRLF3	KIF26B-AS1	0.58413875	2.92E-50	postive
NR2C2	KIF26B-AS1	0.645056039	2.87E-64	postive
RORA	KIF26B-AS1	0.72140725	4.24E-87	postive
SOS1	KIF26B-AS1	0.601959573	4.76E-54	postive
SOS2	KIF26B-AS1	0.54053122	6.44E-42	postive
BRAF	KIF26B-AS1	0.727812343	2.30E-89	postive
TEC	KIF26B-AS1	0.514905637	1.48E-37	postive
CBL	KIF26B-AS1	0.526417362	1.81E-39	postive
CBLB	KIF26B-AS1	0.6721673	1.37E-71	postive
PDK1	KIF26B-AS1	0.612522353	2.08E-56	postive
TRAJ1	KIF26B-AS1	0.565983955	1.23E-46	postive
TRAJ2	KIF26B-AS1	0.575307743	1.81E-48	postive
TRAJ3	KIF26B-AS1	0.651880221	4.84E-66	postive
TRAJ5	KIF26B-AS1	0.651395117	6.50E-66	postive
TRAJ6	KIF26B-AS1	0.612118133	2.57E-56	postive
TRAJ8	KIF26B-AS1	0.602602649	3.44E-54	postive
TRAJ10	KIF26B-AS1	0.612688321	1.91E-56	postive
TRAJ12	KIF26B-AS1	0.530031657	4.37E-40	postive
TRAJ13	KIF26B-AS1	0.582405566	6.62E-50	postive
TRAJ14	KIF26B-AS1	0.599270702	1.84E-53	postive
TRAJ16	KIF26B-AS1	0.616535019	2.50E-57	postive
TRAJ17	KIF26B-AS1	0.606800996	4.05E-55	postive
TRAJ18	KIF26B-AS1	0.545386829	8.71E-43	postive
TRAJ21	KIF26B-AS1	0.63055197	1.20E-60	postive
TRAJ31	KIF26B-AS1	0.655293347	6.04E-67	postive
TRAJ37	KIF26B-AS1	0.614705136	6.59E-57	postive
TRAJ38	KIF26B-AS1	0.700292823	4.64E-80	postive
TRAJ39	KIF26B-AS1	0.68953592	1.05E-76	postive
DDX17	AL136295.6	0.511911482	4.52E-37	postive
NFAT5	AL136295.6	0.53361052	1.06E-40	postive

LTB4R2	AL136295.6	0.506196881	3.71E-36	postive
GNRH1	AL136295.6	0.604439917	1.35E-54	postive
NR2C2	AL136295.6	0.526354134	1.85E-39	postive
RORA	AL136295.6	0.511080361	6.16E-37	postive
BRAF	AL136295.6	0.566303066	1.07E-46	postive
TRAJ2	AL136295.6	0.502758328	1.29E-35	postive
TRAJ3	AL136295.6	0.525987402	2.14E-39	postive
TRAJ5	AL136295.6	0.502594501	1.37E-35	postive
TRAJ8	AL136295.6	0.514640907	1.63E-37	postive
TRAJ13	AL136295.6	0.515177269	1.34E-37	postive
TRAJ21	AL136295.6	0.520424622	1.83E-38	postive
TRAJ31	AL136295.6	0.512611621	3.49E-37	postive
TRAJ37	AL136295.6	0.519551389	2.55E-38	postive
TRAJ38	AL136295.6	0.518247756	4.19E-38	postive
TRAJ39	AL136295.6	0.537036124	2.67E-41	postive
CREB1	AL360219.2	0.658683802	7.45E-68	postive
UBR1	AL360219.2	0.502591318	1.37E-35	postive
CYLD	AL360219.2	0.537579507	2.14E-41	postive
NFKBIZ	AL360219.2	0.512852866	3.19E-37	postive
DDX17	AL360219.2	0.518131558	4.38E-38	postive
JAK2	AL360219.2	0.598869484	2.25E-53	postive
NFAT5	AL360219.2	0.741961922	1.31E-94	postive
PIK3R1	AL360219.2	0.543721275	1.74E-42	postive
PIK3CA	AL360219.2	0.597479836	4.49E-53	postive
GNRH1	AL360219.2	0.637248257	2.69E-62	postive
IL6ST	AL360219.2	0.550655921	9.56E-44	postive
ACVR2A	AL360219.2	0.518817184	3.37E-38	postive
ANGPTL1	AL360219.2	0.675827115	1.22E-72	postive
BMPR2	AL360219.2	0.620016583	3.88E-58	postive
CRLF3	AL360219.2	0.559058721	2.60E-45	postive
NR2C2	AL360219.2	0.594261356	2.20E-52	postive
RORA	AL360219.2	0.755434615	6.24E-100	postive
SOS1	AL360219.2	0.571661643	9.56E-48	postive
BRAF	AL360219.2	0.671744576	1.80E-71	postive
CBLB	AL360219.2	0.719060512	2.76E-86	postive
TRAJ1	AL360219.2	0.612782586	1.81E-56	postive
TRAJ2	AL360219.2	0.609728296	8.93E-56	postive
TRAJ3	AL360219.2	0.609955322	7.94E-56	postive
TRAJ5	AL360219.2	0.706271334	5.46E-82	postive
TRAJ6	AL360219.2	0.655912895	4.13E-67	postive
TRAJ8	AL360219.2	0.595487947	1.20E-52	postive
TRAJ10	AL360219.2	0.664728849	1.66E-69	postive
TRAJ12	AL360219.2	0.574097552	3.15E-48	postive
TRAJ13	AL360219.2	0.65865709	7.57E-68	postive
TRAJ14	AL360219.2	0.633349091	2.48E-61	postive
TRAJ16	AL360219.2	0.673420426	6.00E-72	postive
TRAJ17	AL360219.2	0.597405586	4.66E-53	postive
TRAJ18	AL360219.2	0.616687341	2.31E-57	postive
TRAJ21	AL360219.2	0.672643941	9.99E-72	postive
TRAJ31	AL360219.2	0.720265233	1.06E-86	postive
TRAJ37	AL360219.2	0.687943551	3.19E-76	postive
TRAJ38	AL360219.2	0.748241821	4.78E-97	postive
TRAJ39	AL360219.2	0.697257265	4.25E-79	postive
DDX17	AC004918.1	0.514125517	1.98E-37	postive
GNRH1	AC004918.1	0.544271267	1.38E-42	postive
CDK4	GIHCG	0.630066445	1.57E-60	postive
CREB1	AL049552.1	0.753277706	4.68E-99	postive
RFXAP	AL049552.1	0.542382395	3.02E-42	postive
UBR1	AL049552.1	0.653173944	2.21E-66	postive
ZC3HAV1	AL049552.1	0.55075374	9.17E-44	postive
ZC3HAV1L	AL049552.1	0.506138249	3.79E-36	postive
IL15	AL049552.1	0.509482284	1.11E-36	postive
CYLD	AL049552.1	0.579599967	2.47E-49	postive
EIF2AK2	AL049552.1	0.544621329	1.20E-42	postive
MAPK8	AL049552.1	0.580582891	1.56E-49	postive

LMBR1	AL049552.1	0.520430572	1.82E-38	postive
IREB2	AL049552.1	0.604152505	1.57E-54	postive
DDX17	AL049552.1	0.692129647	1.68E-77	postive
PIK3CG	AL049552.1	0.500954394	2.47E-35	postive
JAK2	AL049552.1	0.609710925	9.01E-56	postive
TXK	AL049552.1	0.62316558	7.05E-59	postive
NFAT5	AL049552.1	0.809900161	1.39E-125	postive
MALT1	AL049552.1	0.543997838	1.55E-42	postive
PIK3R1	AL049552.1	0.632347202	4.36E-61	postive
PIK3CA	AL049552.1	0.653769875	1.54E-66	postive
AKT3	AL049552.1	0.50030089	3.12E-35	postive
RASGRP3	AL049552.1	0.526223403	1.95E-39	postive
IGHD6-6	AL049552.1	0.504090935	7.97E-36	postive
LTB4R2	AL049552.1	0.521770344	1.09E-38	postive
GNRH1	AL049552.1	0.799248805	5.96E-120	postive
IL6ST	AL049552.1	0.648511773	3.68E-65	postive
RABEP1	AL049552.1	0.571271231	1.14E-47	postive
ACVR2A	AL049552.1	0.628929677	2.96E-60	postive
ANGPTL1	AL049552.1	0.758134999	4.86E-101	postive
BMPR1A	AL049552.1	0.53534727	5.27E-41	postive
BMPR2	AL049552.1	0.717153457	1.25E-85	postive
CRLF3	AL049552.1	0.625182888	2.34E-59	postive
NR2C1	AL049552.1	0.545368128	8.77E-43	postive
NR2C2	AL049552.1	0.764118516	1.50E-103	postive
RORA	AL049552.1	0.839480209	2.68E-143	postive
SOS1	AL049552.1	0.672706283	9.59E-72	postive
SOS2	AL049552.1	0.542592669	2.77E-42	postive
BRAF	AL049552.1	0.812991259	2.76E-127	postive
TEC	AL049552.1	0.515687579	1.10E-37	postive
CBL	AL049552.1	0.562445044	5.90E-46	postive
CBLB	AL049552.1	0.739631973	1.01E-93	postive
PDK1	AL049552.1	0.560857355	1.18E-45	postive
TRAJ1	AL049552.1	0.650978271	8.36E-66	postive
TRAJ2	AL049552.1	0.702671866	8.03E-81	postive
TRAJ3	AL049552.1	0.707866981	1.64E-82	postive
TRAJ5	AL049552.1	0.711193954	1.29E-83	postive
TRAJ6	AL049552.1	0.70036342	4.41E-80	postive
TRAJ8	AL049552.1	0.676707655	6.79E-73	postive
TRAJ10	AL049552.1	0.669480122	7.87E-71	postive
TRAJ12	AL049552.1	0.614163062	8.78E-57	postive
TRAJ13	AL049552.1	0.688694515	1.89E-76	postive
TRAJ14	AL049552.1	0.672884169	8.53E-72	postive
TRAJ16	AL049552.1	0.711530395	9.98E-84	postive
TRAJ17	AL049552.1	0.64247075	1.31E-63	postive
TRAJ18	AL049552.1	0.63636903	4.46E-62	postive
TRAJ21	AL049552.1	0.716446811	2.18E-85	postive
TRAJ31	AL049552.1	0.743547939	3.23E-95	postive
TRAJ37	AL049552.1	0.721874513	2.91E-87	postive
TRAJ38	AL049552.1	0.793349479	5.63E-117	postive
TRAJ39	AL049552.1	0.744874315	9.90E-96	postive
CREB1	WAKMAR2	0.508718257	1.47E-36	postive
TLR4	WAKMAR2	0.545458932	8.45E-43	postive
IL15	WAKMAR2	0.634584806	1.23E-61	postive
TNFAIP3	WAKMAR2	0.682580972	1.29E-74	postive
CYLD	WAKMAR2	0.535346407	5.27E-41	postive
IL7R	WAKMAR2	0.624951479	2.66E-59	postive
PIK3CG	WAKMAR2	0.537849689	1.92E-41	postive
JAK2	WAKMAR2	0.5547158	1.70E-44	postive
NFAT5	WAKMAR2	0.524627453	3.62E-39	postive
PIK3CA	WAKMAR2	0.559840045	1.85E-45	postive
FPR2	WAKMAR2	0.610254813	6.79E-56	postive
ANGPTL1	WAKMAR2	0.609200813	1.17E-55	postive
IL18R1	WAKMAR2	0.707716915	1.83E-82	postive
IL18RAP	WAKMAR2	0.580904233	1.34E-49	postive
RORA	WAKMAR2	0.587616043	5.55E-51	postive

LCP2	WAKMAR2	0.529138404	6.22E-40	postive
PTPRC	WAKMAR2	0.556711214	7.18E-45	postive
ITK	WAKMAR2	0.637468188	2.37E-62	postive
CD28	WAKMAR2	0.507355435	2.43E-36	postive
CBL	WAKMAR2	0.524011553	4.60E-39	postive
CBLB	WAKMAR2	0.509958452	9.33E-37	postive
PDK1	WAKMAR2	0.507797694	2.07E-36	postive
TRAJ3	WAKMAR2	0.598837114	2.28E-53	postive
TRAJ5	WAKMAR2	0.532323316	1.76E-40	postive
TRAJ6	WAKMAR2	0.520979638	1.48E-38	postive
TRAJ10	WAKMAR2	0.552991707	3.54E-44	postive
TRAJ13	WAKMAR2	0.504972877	5.79E-36	postive
TRAJ16	WAKMAR2	0.539135095	1.14E-41	postive
TRAJ17	WAKMAR2	0.562406166	6.00E-46	postive
TRAJ18	WAKMAR2	0.558408432	3.45E-45	postive
TRAJ21	WAKMAR2	0.557581546	4.93E-45	postive
TRAJ31	WAKMAR2	0.522313348	8.86E-39	postive
TRAJ38	WAKMAR2	0.553971202	2.33E-44	postive
TRAJ39	WAKMAR2	0.559237783	2.40E-45	postive
NR2C2	AC009237.15	0.552854141	3.76E-44	postive
BRAF	AC009237.15	0.528978164	6.63E-40	postive
TRAJ1	AC009237.15	0.525231773	2.87E-39	postive
TRAJ5	AC009237.15	0.582269691	7.06E-50	postive
TRAJ8	AC009237.15	0.522610004	7.90E-39	postive
TRAJ12	AC009237.15	0.547419096	3.73E-43	postive
TRAJ13	AC009237.15	0.504528262	6.81E-36	postive
TRAJ14	AC009237.15	0.530169462	4.14E-40	postive
TRAJ16	AC009237.15	0.509693186	1.03E-36	postive
TRAJ17	AC009237.15	0.562637302	5.42E-46	postive
TRAJ21	AC009237.15	0.527568733	1.15E-39	postive
TRAJ31	AC009237.15	0.527968744	9.85E-40	postive
TRAJ39	AC009237.15	0.540403291	6.79E-42	postive
GIPR	AL591895.1	0.503630486	9.42E-36	postive
CREB1	AC079684.2	0.620708303	2.67E-58	postive
ZC3HAV1L	AC079684.2	0.56895893	3.25E-47	postive
IL15	AC079684.2	0.523608242	5.38E-39	postive
EIF2AK2	AC079684.2	0.513018955	3.00E-37	postive
MAPK8	AC079684.2	0.586974917	7.55E-51	postive
NFKBIZ	AC079684.2	0.512437053	3.72E-37	postive
LMBR1	AC079684.2	0.515214873	1.32E-37	postive
EED	AC079684.2	0.523018662	6.75E-39	postive
IREB2	AC079684.2	0.555321855	1.31E-44	postive
DDX17	AC079684.2	0.520348132	1.88E-38	postive
JAK2	AC079684.2	0.512857447	3.18E-37	postive
NFAT5	AC079684.2	0.661770641	1.08E-68	postive
MALT1	AC079684.2	0.516608089	7.79E-38	postive
PIK3CA	AC079684.2	0.575799332	1.44E-48	postive
AKT3	AC079684.2	0.508525254	1.58E-36	postive
GNRH1	AC079684.2	0.667642522	2.58E-70	postive
ANGPTL1	AC079684.2	0.664017645	2.61E-69	postive
BMPR2	AC079684.2	0.53061039	3.48E-40	postive
CRLF3	AC079684.2	0.596353123	7.85E-53	postive
NR2C1	AC079684.2	0.607163484	3.36E-55	postive
NR2C2	AC079684.2	0.599156624	1.95E-53	postive
RORA	AC079684.2	0.651387489	6.53E-66	postive
SOS1	AC079684.2	0.609270403	1.13E-55	postive
BRAF	AC079684.2	0.694181725	3.89E-78	postive
CBL	AC079684.2	0.579389189	2.72E-49	postive
CBLB	AC079684.2	0.672335769	1.22E-71	postive
PDK1	AC079684.2	0.644062698	5.15E-64	postive
TRAJ1	AC079684.2	0.593021386	4.04E-52	postive
TRAJ2	AC079684.2	0.589391077	2.36E-51	postive
TRAJ3	AC079684.2	0.624130394	4.17E-59	postive
TRAJ5	AC079684.2	0.645881601	1.76E-64	postive
TRAJ6	AC079684.2	0.634538978	1.26E-61	postive

TRAJ8	AC079684.2	0.590565702	1.34E-51	postive
TRAJ10	AC079684.2	0.608730628	1.50E-55	postive
TRAJ12	AC079684.2	0.534443027	7.57E-41	postive
TRAJ13	AC079684.2	0.625364508	2.12E-59	postive
TRAJ14	AC079684.2	0.613628736	1.16E-56	postive
TRAJ16	AC079684.2	0.614054342	9.29E-57	postive
TRAJ17	AC079684.2	0.574818544	2.26E-48	postive
TRAJ18	AC079684.2	0.546701979	5.03E-43	postive
TRAJ21	AC079684.2	0.644815967	3.30E-64	postive
TRAJ31	AC079684.2	0.651276959	6.98E-66	postive
TRAJ37	AC079684.2	0.626027595	1.47E-59	postive
TRAJ38	AC079684.2	0.685747042	1.47E-75	postive
TRAJ39	AC079684.2	0.643262615	8.23E-64	postive
CREB1	A2M-AS1	0.688150609	2.76E-76	postive
RFXAP	A2M-AS1	0.54242428	2.96E-42	postive
UBR1	A2M-AS1	0.557176522	5.88E-45	postive
IL15	A2M-AS1	0.534008695	9.01E-41	postive
CYLD	A2M-AS1	0.620300024	3.33E-58	postive
EIF2AK2	A2M-AS1	0.518448489	3.88E-38	postive
LIMS1	A2M-AS1	0.543380942	2.00E-42	postive
IREB2	A2M-AS1	0.589226497	2.56E-51	postive
DDX17	A2M-AS1	0.579633185	2.43E-49	postive
PIK3CG	A2M-AS1	0.558628794	3.13E-45	postive
JAK2	A2M-AS1	0.604730022	1.17E-54	postive
TXK	A2M-AS1	0.536432741	3.40E-41	postive
NFAT5	A2M-AS1	0.749064681	2.26E-97	postive
MALT1	A2M-AS1	0.5177107	5.13E-38	postive
PIK3R1	A2M-AS1	0.677126032	5.14E-73	postive
PIK3CA	A2M-AS1	0.634168233	1.56E-61	postive
RASGRP3	A2M-AS1	0.548709703	2.17E-43	postive
IGHD6-6	A2M-AS1	0.506924983	2.84E-36	postive
PLXNC1	A2M-AS1	0.565936358	1.26E-46	postive
GNRH1	A2M-AS1	0.59530655	1.32E-52	postive
IL6ST	A2M-AS1	0.594877131	1.63E-52	postive
ACVR2A	A2M-AS1	0.615616826	4.07E-57	postive
ANGPTL1	A2M-AS1	0.71827714	5.14E-86	postive
BMPR1A	A2M-AS1	0.505665294	4.50E-36	postive
BMPR2	A2M-AS1	0.681990128	1.93E-74	postive
CRLF3	A2M-AS1	0.55745603	5.21E-45	postive
NR2C2	A2M-AS1	0.605801538	6.75E-55	postive
RORA	A2M-AS1	0.794365029	1.76E-117	postive
SOS1	A2M-AS1	0.675471616	1.55E-72	postive
SOS2	A2M-AS1	0.55813256	3.88E-45	postive
BRAF	A2M-AS1	0.687297332	5.01E-76	postive
ITK	A2M-AS1	0.547192807	4.10E-43	postive
CD28	A2M-AS1	0.555697719	1.11E-44	postive
CBL	A2M-AS1	0.523193256	6.31E-39	postive
CBLB	A2M-AS1	0.655135117	6.66E-67	postive
PDK1	A2M-AS1	0.52586863	2.24E-39	postive
TRAJ1	A2M-AS1	0.693496344	6.35E-78	postive
TRAJ2	A2M-AS1	0.688534358	2.11E-76	postive
TRAJ3	A2M-AS1	0.745432637	6.01E-96	postive
TRAJ5	A2M-AS1	0.759314692	1.58E-101	postive
TRAJ6	A2M-AS1	0.734728169	6.93E-92	postive
TRAJ8	A2M-AS1	0.725324288	1.77E-88	postive
TRAJ10	A2M-AS1	0.71095147	1.56E-83	postive
TRAJ12	A2M-AS1	0.733169348	2.60E-91	postive
TRAJ13	A2M-AS1	0.739459032	1.18E-93	postive
TRAJ14	A2M-AS1	0.753748929	3.02E-99	postive
TRAJ16	A2M-AS1	0.738304836	3.20E-93	postive
TRAJ17	A2M-AS1	0.720130652	1.18E-86	postive
TRAJ18	A2M-AS1	0.734402445	9.14E-92	postive
TRAJ21	A2M-AS1	0.773889161	8.22E-108	postive
TRAJ31	A2M-AS1	0.776569687	5.10E-109	postive
TRAJ37	A2M-AS1	0.758341667	3.99E-101	postive

TRAJ38	A2M-AS1	0.803751041	2.74E-122	postive
TRAJ39	A2M-AS1	0.784143456	1.60E-112	postive
IRF9	AL136295.2	0.652659099	3.02E-66	postive
CREB1	AC015813.1	0.54832551	2.55E-43	postive
NFKBIZ	AC015813.1	0.532152564	1.89E-40	postive
DDX17	AC015813.1	0.566267241	1.08E-46	postive
NFAT5	AC015813.1	0.532699129	1.52E-40	postive
LTB4R2	AC015813.1	0.647601539	6.33E-65	postive
GNRH1	AC015813.1	0.711371765	1.13E-83	postive
ANGPTL1	AC015813.1	0.566457231	9.96E-47	postive
CRLF3	AC015813.1	0.503878522	8.61E-36	postive
NR2C1	AC015813.1	0.507801266	2.06E-36	postive
NR2C2	AC015813.1	0.704725003	1.74E-81	postive
RORA	AC015813.1	0.55024046	1.14E-43	postive
SOS1	AC015813.1	0.517079498	6.52E-38	postive
BRAF	AC015813.1	0.635232615	8.52E-62	postive
CBLB	AC015813.1	0.571012584	1.28E-47	postive
TRAJ1	AC015813.1	0.509281868	1.20E-36	postive
TRAJ3	AC015813.1	0.562451907	5.88E-46	postive
TRAJ5	AC015813.1	0.548465374	2.41E-43	postive
TRAJ6	AC015813.1	0.504638811	6.54E-36	postive
TRAJ8	AC015813.1	0.503696605	9.20E-36	postive
TRAJ16	AC015813.1	0.508820239	1.42E-36	postive
TRAJ17	AC015813.1	0.504149063	7.81E-36	postive
TRAJ21	AC015813.1	0.516648143	7.67E-38	postive
TRAJ31	AC015813.1	0.534313977	7.97E-41	postive
TRAJ37	AC015813.1	0.500104725	3.35E-35	postive
TRAJ38	AC015813.1	0.552682604	4.04E-44	postive
TRAJ39	AC015813.1	0.562573146	5.57E-46	postive
ROBO2	ARHGEF2-AS1	0.521917004	1.03E-38	postive
MSR1	PARAL1	0.51416502	1.95E-37	postive
CCL23	PARAL1	0.608943393	1.34E-55	postive
AGRP	PARAL1	0.625035468	2.54E-59	postive
RETN	PARAL1	0.678154964	2.58E-73	postive
DUOX2	AC010547.2	0.603787988	1.89E-54	postive
ACKR4	AC010547.2	0.607004294	3.64E-55	postive
FAM3D	AC010547.2	0.524781438	3.41E-39	postive
MIA	AC010547.2	0.50198153	1.71E-35	postive
IL1R2	AC010547.2	0.557526359	5.05E-45	postive
CREB1	AC066613.1	0.712529364	4.62E-84	postive
RFXAP	AC066613.1	0.503774741	8.94E-36	postive
UBR1	AC066613.1	0.603261068	2.46E-54	postive
ZC3HAV1	AC066613.1	0.537319234	2.38E-41	postive
ZC3HAV1L	AC066613.1	0.559638125	2.02E-45	postive
IL15	AC066613.1	0.601637018	5.60E-54	postive
CYLD	AC066613.1	0.581046831	1.25E-49	postive
EIF2AK2	AC066613.1	0.502535514	1.40E-35	postive
MAPK8	AC066613.1	0.507872781	2.01E-36	postive
TLR1	AC066613.1	0.545984068	6.79E-43	postive
NFKBIZ	AC066613.1	0.580540875	1.59E-49	postive
LMBR1	AC066613.1	0.508141178	1.82E-36	postive
IREB2	AC066613.1	0.576971233	8.38E-49	postive
DDX17	AC066613.1	0.627396805	6.92E-60	postive
PIK3CG	AC066613.1	0.533405252	1.15E-40	postive
JAK2	AC066613.1	0.666733411	4.62E-70	postive
TXK	AC066613.1	0.523680567	5.23E-39	postive
NFAT5	AC066613.1	0.802207581	1.76E-121	postive
MALT1	AC066613.1	0.509855082	9.69E-37	postive
PIK3R1	AC066613.1	0.52702124	1.43E-39	postive
PIK3CA	AC066613.1	0.632514654	3.97E-61	postive
AKT3	AC066613.1	0.504503326	6.87E-36	postive
RASGRP3	AC066613.1	0.543670773	1.77E-42	postive
LTB4R2	AC066613.1	0.519513357	2.59E-38	postive
GNRH1	AC066613.1	0.762428802	7.82E-103	postive
IL6ST	AC066613.1	0.507570383	2.25E-36	postive

RABEP1	AC066613.1	0.560558743	1.35E-45	postive
ACVR2A	AC066613.1	0.549705627	1.43E-43	postive
ANGPTL1	AC066613.1	0.703271674	5.14E-81	postive
BMPR2	AC066613.1	0.591867393	7.10E-52	postive
CRLF3	AC066613.1	0.659242548	5.26E-68	postive
NR2C1	AC066613.1	0.534971896	6.12E-41	postive
NR2C2	AC066613.1	0.71530534	5.34E-85	postive
RORA	AC066613.1	0.728250535	1.60E-89	postive
SOS1	AC066613.1	0.65373552	1.57E-66	postive
SOS2	AC066613.1	0.567079417	7.54E-47	postive
BRAF	AC066613.1	0.717511995	9.42E-86	postive
TEC	AC066613.1	0.505798548	4.29E-36	postive
CBL	AC066613.1	0.636680073	3.73E-62	postive
CBLB	AC066613.1	0.698765459	1.42E-79	postive
PDK1	AC066613.1	0.597927348	3.59E-53	postive
TRAJ1	AC066613.1	0.749428459	1.62E-97	postive
TRAJ2	AC066613.1	0.713557276	2.08E-84	postive
TRAJ3	AC066613.1	0.769424442	7.74E-106	postive
TRAJ5	AC066613.1	0.746025367	3.53E-96	postive
TRAJ6	AC066613.1	0.781338346	3.30E-111	postive
TRAJ8	AC066613.1	0.710529163	2.15E-83	postive
TRAJ10	AC066613.1	0.773896956	8.15E-108	postive
TRAJ12	AC066613.1	0.649390637	2.17E-65	postive
TRAJ13	AC066613.1	0.73305801	2.86E-91	postive
TRAJ14	AC066613.1	0.733667774	1.71E-91	postive
TRAJ16	AC066613.1	0.755506562	5.83E-100	postive
TRAJ17	AC066613.1	0.706669895	4.04E-82	postive
TRAJ18	AC066613.1	0.691389585	2.84E-77	postive
TRAJ21	AC066613.1	0.755060833	8.86E-100	postive
TRAJ31	AC066613.1	0.752204241	1.27E-98	postive
TRAJ37	AC066613.1	0.74500264	8.83E-96	postive
TRAJ38	AC066613.1	0.804101046	1.79E-122	postive
TRAJ39	AC066613.1	0.77104118	1.51E-106	postive
CREB1	MAGI2-AS3	0.594374994	2.08E-52	postive
UBR1	MAGI2-AS3	0.501409447	2.10E-35	postive
CYLD	MAGI2-AS3	0.555597023	1.16E-44	postive
PDGFRA	MAGI2-AS3	0.550811194	8.95E-44	postive
LIMS1	MAGI2-AS3	0.511498824	5.27E-37	postive
PIK3CG	MAGI2-AS3	0.529197951	6.08E-40	postive
JAK2	MAGI2-AS3	0.593241574	3.63E-52	postive
TXK	MAGI2-AS3	0.504099249	7.95E-36	postive
NFAT5	MAGI2-AS3	0.628063892	4.78E-60	postive
PIK3R1	MAGI2-AS3	0.660601881	2.25E-68	postive
PIK3CA	MAGI2-AS3	0.552608591	4.17E-44	postive
RASGRP3	MAGI2-AS3	0.512744403	3.32E-37	postive
SLIT2	MAGI2-AS3	0.508129201	1.83E-36	postive
PLXNC1	MAGI2-AS3	0.540576134	6.32E-42	postive
FGF7	MAGI2-AS3	0.546800519	4.83E-43	postive
GNRH1	MAGI2-AS3	0.550569938	9.91E-44	postive
IL6ST	MAGI2-AS3	0.599171849	1.93E-53	postive
ACVR2A	MAGI2-AS3	0.524605746	3.65E-39	postive
ANGPTL1	MAGI2-AS3	0.641470879	2.35E-63	postive
BMPR2	MAGI2-AS3	0.655257711	6.18E-67	postive
NR2C2	MAGI2-AS3	0.504172916	7.74E-36	postive
RORA	MAGI2-AS3	0.697684201	3.12E-79	postive
BRAF	MAGI2-AS3	0.53575004	4.48E-41	postive
CBLB	MAGI2-AS3	0.59394906	2.57E-52	postive
TRAJ1	MAGI2-AS3	0.504030426	8.15E-36	postive
TRAJ3	MAGI2-AS3	0.501936359	1.74E-35	postive
TRAJ5	MAGI2-AS3	0.565437721	1.57E-46	postive
TRAJ6	MAGI2-AS3	0.527913593	1.01E-39	postive
TRAJ10	MAGI2-AS3	0.522476786	8.32E-39	postive
TRAJ13	MAGI2-AS3	0.519395405	2.71E-38	postive
TRAJ16	MAGI2-AS3	0.542357519	3.05E-42	postive
TRAJ18	MAGI2-AS3	0.527238625	1.31E-39	postive

TRAJ21	MAGI2-AS3	0.544484282	1.27E-42	postive
TRAJ31	MAGI2-AS3	0.574142719	3.08E-48	postive
TRAJ37	MAGI2-AS3	0.55107692	8.00E-44	postive
TRAJ38	MAGI2-AS3	0.588166502	4.26E-51	postive
TRAJ39	MAGI2-AS3	0.570250571	1.81E-47	postive
CREB1	AC027097.1	0.61843357	9.08E-58	postive
RFXAP	AC027097.1	0.512646359	3.44E-37	postive
MAPK8	AC027097.1	0.53997417	8.09E-42	postive
DDX17	AC027097.1	0.551294445	7.29E-44	postive
NFAT5	AC027097.1	0.643136196	8.87E-64	postive
GNRH1	AC027097.1	0.630792796	1.04E-60	postive
IL6ST	AC027097.1	0.502617718	1.36E-35	postive
ACVR2A	AC027097.1	0.525072449	3.05E-39	postive
ANGPTL1	AC027097.1	0.577351702	7.03E-49	postive
BMPR1A	AC027097.1	0.501186725	2.27E-35	postive
BMPR2	AC027097.1	0.511191621	5.91E-37	postive
CRLF3	AC027097.1	0.577303798	7.18E-49	postive
NR2C2	AC027097.1	0.614026425	9.43E-57	postive
RORA	AC027097.1	0.632580114	3.83E-61	postive
SOS1	AC027097.1	0.576515993	1.03E-48	postive
BRAF	AC027097.1	0.613153611	1.49E-56	postive
CBLB	AC027097.1	0.504460324	6.98E-36	postive
PDK1	AC027097.1	0.513598542	2.41E-37	postive
TRAJ1	AC027097.1	0.603280759	2.44E-54	postive
TRAJ2	AC027097.1	0.612393235	2.22E-56	postive
TRAJ3	AC027097.1	0.638252956	1.51E-62	postive
TRAJ5	AC027097.1	0.63223969	4.64E-61	postive
TRAJ6	AC027097.1	0.646442039	1.26E-64	postive
TRAJ8	AC027097.1	0.627957385	5.07E-60	postive
TRAJ10	AC027097.1	0.629100682	2.69E-60	postive
TRAJ12	AC027097.1	0.585700586	1.39E-50	postive
TRAJ13	AC027097.1	0.624247106	3.91E-59	postive
TRAJ14	AC027097.1	0.625139916	2.40E-59	postive
TRAJ16	AC027097.1	0.638787122	1.11E-62	postive
TRAJ17	AC027097.1	0.612785947	1.81E-56	postive
TRAJ18	AC027097.1	0.583126248	4.71E-50	postive
TRAJ21	AC027097.1	0.642738229	1.12E-63	postive
TRAJ31	AC027097.1	0.65410013	1.26E-66	postive
TRAJ37	AC027097.1	0.645847745	1.80E-64	postive
TRAJ38	AC027097.1	0.682553181	1.32E-74	postive
TRAJ39	AC027097.1	0.662152331	8.50E-69	postive
CREB1	RFX3-AS1	0.640326897	4.56E-63	postive
UBR1	RFX3-AS1	0.542231962	3.21E-42	postive
ZC3HAV1	RFX3-AS1	0.535046235	5.94E-41	postive
ZC3HAV1L	RFX3-AS1	0.502311307	1.52E-35	postive
IL15	RFX3-AS1	0.572448039	6.69E-48	postive
CYLD	RFX3-AS1	0.512005248	4.37E-37	postive
EIF2AK2	RFX3-AS1	0.555101373	1.44E-44	postive
IREB2	RFX3-AS1	0.519578554	2.52E-38	postive
DDX17	RFX3-AS1	0.553683824	2.64E-44	postive
JAK2	RFX3-AS1	0.569658638	2.37E-47	postive
TXK	RFX3-AS1	0.510243807	8.39E-37	postive
NFAT5	RFX3-AS1	0.593093224	3.90E-52	postive
PIK3R1	RFX3-AS1	0.585592597	1.46E-50	postive
PIK3CA	RFX3-AS1	0.57421764	2.98E-48	postive
PIK3CB	RFX3-AS1	0.513074538	2.93E-37	postive
RASGRP3	RFX3-AS1	0.527909177	1.01E-39	postive
LTB4R2	RFX3-AS1	0.543102988	2.24E-42	postive
PLXNC1	RFX3-AS1	0.539038479	1.18E-41	postive
GNRH1	RFX3-AS1	0.574471667	2.65E-48	postive
IL6ST	RFX3-AS1	0.519512709	2.59E-38	postive
ACVR2A	RFX3-AS1	0.523787476	5.02E-39	postive
ANGPTL1	RFX3-AS1	0.639281991	8.35E-63	postive
BMPR2	RFX3-AS1	0.570884999	1.36E-47	postive
CRLF3	RFX3-AS1	0.516363163	8.54E-38	postive

NR2C2	RFX3-AS1	0.710941938	1.57E-83	postive
RORA	RFX3-AS1	0.630455012	1.26E-60	postive
SOS1	RFX3-AS1	0.609078609	1.25E-55	postive
SOS2	RFX3-AS1	0.546654136	5.14E-43	postive
BRAF	RFX3-AS1	0.734191048	1.09E-91	postive
CBL	RFX3-AS1	0.511683442	4.92E-37	postive
CBLB	RFX3-AS1	0.620299197	3.33E-58	postive
RASGRP1	RFX3-AS1	0.54050533	6.51E-42	postive
TRAJ1	RFX3-AS1	0.647713936	5.92E-65	postive
TRAJ2	RFX3-AS1	0.586879714	7.90E-51	postive
TRAJ3	RFX3-AS1	0.696495357	7.37E-79	postive
TRAJ5	RFX3-AS1	0.68294764	1.01E-74	postive
TRAJ6	RFX3-AS1	0.638607315	1.23E-62	postive
TRAJ8	RFX3-AS1	0.641943621	1.78E-63	postive
TRAJ10	RFX3-AS1	0.61401052	9.51E-57	postive
TRAJ12	RFX3-AS1	0.674554011	2.84E-72	postive
TRAJ13	RFX3-AS1	0.61218091	2.49E-56	postive
TRAJ14	RFX3-AS1	0.67438644	3.17E-72	postive
TRAJ16	RFX3-AS1	0.638991517	9.88E-63	postive
TRAJ17	RFX3-AS1	0.656196101	3.47E-67	postive
TRAJ18	RFX3-AS1	0.62799413	4.97E-60	postive
TRAJ21	RFX3-AS1	0.665048402	1.36E-69	postive
TRAJ31	RFX3-AS1	0.665064672	1.34E-69	postive
TRAJ37	RFX3-AS1	0.611269096	4.00E-56	postive
TRAJ38	RFX3-AS1	0.704825635	1.61E-81	postive
TRAJ39	RFX3-AS1	0.688450449	2.24E-76	postive
IRF9	AC073842.2	0.564621442	2.25E-46	postive
CREB1	SNHG26	0.586753364	8.39E-51	postive
IL15	SNHG26	0.527979364	9.81E-40	postive
EIF2AK2	SNHG26	0.516187603	9.13E-38	postive
LMBR1	SNHG26	0.500849912	2.56E-35	postive
JAK2	SNHG26	0.513282127	2.72E-37	postive
NFAT5	SNHG26	0.532193947	1.86E-40	postive
PIK3CA	SNHG26	0.55137674	7.04E-44	postive
PLXNC1	SNHG26	0.508659826	1.51E-36	postive
ANGPTL1	SNHG26	0.624763612	2.95E-59	postive
BMPR2	SNHG26	0.51502283	1.42E-37	postive
CRLF3	SNHG26	0.564832156	2.05E-46	postive
NR2C2	SNHG26	0.55767581	4.73E-45	postive
RORA	SNHG26	0.546952338	4.53E-43	postive
SOS1	SNHG26	0.590684054	1.26E-51	postive
BRAF	SNHG26	0.701968985	1.35E-80	postive
CBLB	SNHG26	0.56621236	1.11E-46	postive
PDK1	SNHG26	0.50930786	1.19E-36	postive
TRAJ1	SNHG26	0.674583424	2.78E-72	postive
TRAJ2	SNHG26	0.608004752	2.18E-55	postive
TRAJ3	SNHG26	0.670772945	3.40E-71	postive
TRAJ5	SNHG26	0.733737264	1.61E-91	postive
TRAJ6	SNHG26	0.663250116	4.25E-69	postive
TRAJ8	SNHG26	0.744343282	1.59E-95	postive
TRAJ10	SNHG26	0.625058563	2.51E-59	postive
TRAJ12	SNHG26	0.663719175	3.16E-69	postive
TRAJ13	SNHG26	0.700444999	4.15E-80	postive
TRAJ14	SNHG26	0.74209179	1.17E-94	postive
TRAJ16	SNHG26	0.702692397	7.91E-81	postive
TRAJ17	SNHG26	0.712152195	6.18E-84	postive
TRAJ18	SNHG26	0.595027211	1.51E-52	postive
TRAJ21	SNHG26	0.715626001	4.15E-85	postive
TRAJ31	SNHG26	0.705918944	7.12E-82	postive
TRAJ37	SNHG26	0.624868293	2.78E-59	postive
TRAJ38	SNHG26	0.732556546	4.37E-91	postive
TRAJ39	SNHG26	0.723295984	9.23E-88	postive
NR2C2	AC093297.2	0.51528111	1.28E-37	postive
BRAF	AC093297.2	0.544211389	1.42E-42	postive
PTGS2	PACERR	0.500105015	3.35E-35	postive

CREB1	AC010542.6	0.506674698	3.12E-36	postive
CYLD	AC010542.6	0.512551043	3.57E-37	postive
NFKBIZ	AC010542.6	0.602877727	2.99E-54	postive
DDX17	AC010542.6	0.59661261	6.90E-53	postive
JAK2	AC010542.6	0.520381226	1.86E-38	postive
NFAT5	AC010542.6	0.636019026	5.44E-62	postive
LTB4R2	AC010542.6	0.517231469	6.16E-38	postive
GNRH1	AC010542.6	0.75076988	4.75E-98	postive
ANGPTL1	AC010542.6	0.507595672	2.22E-36	postive
NR2C1	AC010542.6	0.5195467	2.56E-38	postive
NR2C2	AC010542.6	0.585774035	1.34E-50	postive
RORA	AC010542.6	0.596582661	7.00E-53	postive
BRAF	AC010542.6	0.522703363	7.62E-39	postive
CBLB	AC010542.6	0.582378867	6.70E-50	postive
TRAJ1	AC010542.6	0.56892749	3.29E-47	postive
TRAJ2	AC010542.6	0.535280935	5.41E-41	postive
TRAJ3	AC010542.6	0.564824326	2.06E-46	postive
TRAJ5	AC010542.6	0.556217341	8.89E-45	postive
TRAJ6	AC010542.6	0.561523895	8.84E-46	postive
TRAJ10	AC010542.6	0.558428813	3.42E-45	postive
TRAJ13	AC010542.6	0.534593485	7.13E-41	postive
TRAJ14	AC010542.6	0.511318477	5.64E-37	postive
TRAJ16	AC010542.6	0.556293683	8.60E-45	postive
TRAJ18	AC010542.6	0.50562703	4.57E-36	postive
TRAJ21	AC010542.6	0.54031407	7.04E-42	postive
TRAJ31	AC010542.6	0.575642821	1.55E-48	postive
TRAJ37	AC010542.6	0.55771211	4.66E-45	postive
TRAJ38	AC010542.6	0.595051524	1.49E-52	postive
TRAJ39	AC010542.6	0.552363833	4.63E-44	postive
CREB1	AL109761.1	0.536623999	3.15E-41	postive
JAK2	AL109761.1	0.510629034	7.28E-37	postive
NFAT5	AL109761.1	0.578936048	3.37E-49	postive
PIK3CA	AL109761.1	0.519264937	2.84E-38	postive
ANGPTL1	AL109761.1	0.549700776	1.43E-43	postive
CRLF3	AL109761.1	0.516637291	7.70E-38	postive
RORA	AL109761.1	0.546756461	4.92E-43	postive
CBLB	AL109761.1	0.608500809	1.69E-55	postive
PDK1	AL109761.1	0.512688807	3.39E-37	postive
TRAJ5	AL109761.1	0.541414592	4.49E-42	postive
TRAJ6	AL109761.1	0.528735403	7.29E-40	postive
TRAJ10	AL109761.1	0.533124737	1.28E-40	postive
TRAJ16	AL109761.1	0.555728383	1.10E-44	postive
TRAJ21	AL109761.1	0.512063757	4.28E-37	postive
TRAJ31	AL109761.1	0.541106752	5.09E-42	postive
TRAJ37	AL109761.1	0.506801691	2.97E-36	postive
TRAJ38	AL109761.1	0.601319721	6.57E-54	postive
TRAJ39	AL109761.1	0.536561685	3.23E-41	postive
B2M	AL590764.1	0.563955341	3.03E-46	postive
HLA-DMB	AL590764.1	0.53605531	3.96E-41	postive
HLA-DRA	AL590764.1	0.500196862	3.24E-35	postive
ACKR4	AL590764.1	0.533911174	9.37E-41	postive
BTK	AL590764.1	0.55808297	3.97E-45	postive
GMFG	AL590764.1	0.624125634	4.18E-59	postive
TNFSF13B	AL590764.1	0.500583907	2.82E-35	postive
CD48	AL590764.1	0.639813088	6.15E-63	postive
SH2D1A	AL590764.1	0.538241271	1.64E-41	postive
CD3D	AL590764.1	0.54505769	9.98E-43	postive
TRAV16	AL590764.1	0.505602699	4.61E-36	postive
TRBV5-1	AL590764.1	0.503158338	1.12E-35	postive
IRF9	AC233728.1	0.519906834	2.23E-38	postive
CREB1	AC097641.2	0.603025982	2.78E-54	postive
UBR1	AC097641.2	0.520449539	1.81E-38	postive
ZC3HAV1	AC097641.2	0.538495375	1.48E-41	postive
ZC3HAV1L	AC097641.2	0.568084467	4.81E-47	postive
DDX17	AC097641.2	0.609337789	1.09E-55	postive

JAK2	AC097641.2	0.502211304	1.57E-35	postive
TXK	AC097641.2	0.500865891	2.55E-35	postive
NFAT5	AC097641.2	0.645130396	2.74E-64	postive
PIK3CA	AC097641.2	0.529430535	5.55E-40	postive
AKT3	AC097641.2	0.503643238	9.38E-36	postive
LTB4R2	AC097641.2	0.51405418	2.03E-37	postive
GNRH1	AC097641.2	0.79786968	3.02E-119	postive
ACVR2A	AC097641.2	0.506420306	3.42E-36	postive
ANGPTL1	AC097641.2	0.564941399	1.96E-46	postive
BMPR2	AC097641.2	0.514440971	1.76E-37	postive
CRLF3	AC097641.2	0.565948373	1.25E-46	postive
NR2C1	AC097641.2	0.584609294	2.33E-50	postive
NR2C2	AC097641.2	0.733269179	2.39E-91	postive
RORA	AC097641.2	0.588606987	3.45E-51	postive
SOS1	AC097641.2	0.576022591	1.30E-48	postive
BRAF	AC097641.2	0.668295227	1.69E-70	postive
CBL	AC097641.2	0.565883449	1.29E-46	postive
CBLB	AC097641.2	0.587816345	5.04E-51	postive
PDK1	AC097641.2	0.538997378	1.20E-41	postive
TRAJ1	AC097641.2	0.616988234	1.96E-57	postive
TRAJ2	AC097641.2	0.557763594	4.56E-45	postive
TRAJ3	AC097641.2	0.638816236	1.09E-62	postive
TRAJ5	AC097641.2	0.600032934	1.25E-53	postive
TRAJ6	AC097641.2	0.627381138	6.98E-60	postive
TRAJ8	AC097641.2	0.599225888	1.88E-53	postive
TRAJ10	AC097641.2	0.585176657	1.78E-50	postive
TRAJ12	AC097641.2	0.523964473	4.68E-39	postive
TRAJ13	AC097641.2	0.584317737	2.68E-50	postive
TRAJ14	AC097641.2	0.581495757	1.02E-49	postive
TRAJ16	AC097641.2	0.611898359	2.88E-56	postive
TRAJ17	AC097641.2	0.577800614	5.71E-49	postive
TRAJ18	AC097641.2	0.51753842	5.48E-38	postive
TRAJ21	AC097641.2	0.603922613	1.76E-54	postive
TRAJ31	AC097641.2	0.59681848	6.23E-53	postive
TRAJ37	AC097641.2	0.569045988	3.12E-47	postive
TRAJ38	AC097641.2	0.635096627	9.21E-62	postive
TRAJ39	AC097641.2	0.631061759	8.99E-61	postive
CREB1	AC011472.4	0.690398586	5.71E-77	postive
UBR1	AC011472.4	0.571803679	8.96E-48	postive
CYLD	AC011472.4	0.514606714	1.65E-37	postive
MAPK8	AC011472.4	0.508925898	1.36E-36	postive
IREB2	AC011472.4	0.532478836	1.66E-40	postive
DDX17	AC011472.4	0.605329119	8.60E-55	postive
TXK	AC011472.4	0.538782941	1.31E-41	postive
NFAT5	AC011472.4	0.746735132	1.87E-96	postive
PIK3R1	AC011472.4	0.566471425	9.90E-47	postive
PIK3CA	AC011472.4	0.567658629	5.82E-47	postive
ROBO2	AC011472.4	0.619052177	6.52E-58	postive
GNRH1	AC011472.4	0.745221209	7.26E-96	postive
IL6ST	AC011472.4	0.593848497	2.70E-52	postive
ACVR2A	AC011472.4	0.63772819	2.05E-62	postive
ANGPTL1	AC011472.4	0.674092054	3.85E-72	postive
BMPR1A	AC011472.4	0.558825787	2.87E-45	postive
BMPR2	AC011472.4	0.719261716	2.35E-86	postive
CRLF3	AC011472.4	0.506661578	3.13E-36	postive
NR1D2	AC011472.4	0.542017631	3.50E-42	postive
NR2C2	AC011472.4	0.637614919	2.18E-62	postive
RORA	AC011472.4	0.772206849	4.61E-107	postive
SOS1	AC011472.4	0.584303953	2.70E-50	postive
BRAF	AC011472.4	0.7143154	1.16E-84	postive
CBLB	AC011472.4	0.610738477	5.28E-56	postive
TRAJ1	AC011472.4	0.560144255	1.62E-45	postive
TRAJ2	AC011472.4	0.590333352	1.50E-51	postive
TRAJ3	AC011472.4	0.618905874	7.05E-58	postive
TRAJ5	AC011472.4	0.628829209	3.13E-60	postive

TRAJ6	AC011472.4	0.630702543	1.10E-60	postive
TRAJ8	AC011472.4	0.633203308	2.69E-61	postive
TRAJ10	AC011472.4	0.603301357	2.41E-54	postive
TRAJ12	AC011472.4	0.593950648	2.56E-52	postive
TRAJ13	AC011472.4	0.653426601	1.89E-66	postive
TRAJ14	AC011472.4	0.601282219	6.70E-54	postive
TRAJ16	AC011472.4	0.637787279	1.98E-62	postive
TRAJ17	AC011472.4	0.602361501	3.89E-54	postive
TRAJ18	AC011472.4	0.56882368	3.45E-47	postive
TRAJ21	AC011472.4	0.658483533	8.43E-68	postive
TRAJ31	AC011472.4	0.715607777	4.21E-85	postive
TRAJ37	AC011472.4	0.675369591	1.65E-72	postive
TRAJ38	AC011472.4	0.712779534	3.81E-84	postive
TRAJ39	AC011472.4	0.652972975	2.49E-66	postive
TYK2	AL928654.2	0.510828837	6.76E-37	postive
DDX17	AL928654.2	0.508391086	1.66E-36	postive
IRF9	AL928654.2	0.711213256	1.27E-83	postive
NFATC4	AL928654.2	0.517131146	6.39E-38	postive
LTB4R2	AL928654.2	0.507020778	2.75E-36	postive
GNRH1	AL928654.2	0.520994159	1.47E-38	postive
DDX17	AL049840.6	0.554651814	1.74E-44	postive
LTB4R2	AL049840.6	0.522569166	8.03E-39	postive
GNRH1	AL049840.6	0.674447373	3.05E-72	postive
NR2C2	AL049840.6	0.530374774	3.82E-40	postive
RORA	AL049840.6	0.50357796	9.60E-36	postive
BRAF	AL049840.6	0.528705134	7.38E-40	postive
TRAJ3	AL049840.6	0.527697339	1.10E-39	postive
CREB1	AL132989.2	0.81559126	9.67E-129	postive
RFXAP	AL132989.2	0.539315771	1.06E-41	postive
UBR1	AL132989.2	0.689988182	7.62E-77	postive
ZC3HAV1	AL132989.2	0.594028871	2.47E-52	postive
ZC3HAV1L	AL132989.2	0.508846	1.41E-36	postive
IL15	AL132989.2	0.588309291	3.98E-51	postive
CYLD	AL132989.2	0.653212558	2.16E-66	postive
EIF2AK2	AL132989.2	0.571056149	1.26E-47	postive
MAPK8	AL132989.2	0.577864728	5.54E-49	postive
LMBR1	AL132989.2	0.598569059	2.61E-53	postive
LIMS1	AL132989.2	0.510230241	8.44E-37	postive
IREB2	AL132989.2	0.635707904	6.50E-62	postive
DDX17	AL132989.2	0.623950003	4.60E-59	postive
PIK3CG	AL132989.2	0.567759868	5.56E-47	postive
JAK2	AL132989.2	0.663380581	3.91E-69	postive
TXK	AL132989.2	0.594453546	2.00E-52	postive
PPP3CB	AL132989.2	0.515676339	1.11E-37	postive
NFAT5	AL132989.2	0.909112646	7.28E-205	postive
NFATC3	AL132989.2	0.545901335	7.03E-43	postive
MALT1	AL132989.2	0.552066504	5.25E-44	postive
PIK3R1	AL132989.2	0.664955982	1.44E-69	postive
PIK3CA	AL132989.2	0.699235824	1.01E-79	postive
PIK3CB	AL132989.2	0.531215329	2.74E-40	postive
AKT3	AL132989.2	0.596423903	7.58E-53	postive
RASGRP3	AL132989.2	0.533267373	1.21E-40	postive
IGHD6-6	AL132989.2	0.539573913	9.52E-42	postive
PLXNC1	AL132989.2	0.53684592	2.88E-41	postive
GNRH1	AL132989.2	0.751968606	1.58E-98	postive
IL6ST	AL132989.2	0.649254314	2.36E-65	postive
RABEP1	AL132989.2	0.593455071	3.27E-52	postive
ACVR2A	AL132989.2	0.668846529	1.19E-70	postive
ANGPTL1	AL132989.2	0.771884318	6.41E-107	postive
BMPR1A	AL132989.2	0.585758358	1.35E-50	postive
BMPR2	AL132989.2	0.742363355	9.21E-95	postive
CRLF3	AL132989.2	0.696298655	8.49E-79	postive
LIFR	AL132989.2	0.538491433	1.48E-41	postive
NR1D2	AL132989.2	0.536427212	3.41E-41	postive
NR2C2	AL132989.2	0.704072919	2.83E-81	postive

NR3C1	AL132989.2	0.514133912	1.97E-37	postive
RORA	AL132989.2	0.855292946	2.56E-154	postive
SOS1	AL132989.2	0.707948356	1.54E-82	postive
SOS2	AL132989.2	0.613490558	1.25E-56	postive
BRAF	AL132989.2	0.850391478	9.12E-151	postive
ITK	AL132989.2	0.525175231	2.93E-39	postive
CD28	AL132989.2	0.511063192	6.20E-37	postive
CBL	AL132989.2	0.623388102	6.25E-59	postive
CBLB	AL132989.2	0.713605048	2.01E-84	postive
RASGRP1	AL132989.2	0.531911385	2.08E-40	postive
PDK1	AL132989.2	0.584907693	2.02E-50	postive
TRAJ1	AL132989.2	0.82011788	2.48E-131	postive
TRAJ2	AL132989.2	0.843468314	5.83E-146	postive
TRAJ3	AL132989.2	0.882776839	5.23E-177	postive
TRAJ5	AL132989.2	0.84805522	4.06E-149	postive
TRAJ6	AL132989.2	0.890831204	9.32E-185	postive
TRAJ8	AL132989.2	0.879056548	1.28E-173	postive
TRAJ10	AL132989.2	0.852540935	2.62E-152	postive
TRAJ12	AL132989.2	0.798194619	2.06E-119	postive
TRAJ13	AL132989.2	0.886251461	2.80E-180	postive
TRAJ14	AL132989.2	0.874254997	2.09E-169	postive
TRAJ16	AL132989.2	0.865309166	5.30E-162	postive
TRAJ17	AL132989.2	0.823659625	2.07E-133	postive
TRAJ18	AL132989.2	0.804384628	1.27E-122	postive
TRAJ21	AL132989.2	0.903370244	4.05E-198	postive
TRAJ31	AL132989.2	0.901326992	8.05E-196	postive
TRAJ37	AL132989.2	0.897608833	9.16E-192	postive
TRAJ38	AL132989.2	0.932724301	2.86E-238	postive
TRAJ39	AL132989.2	0.911785685	3.70E-208	postive
CREB1	AC090517.2	0.631527212	6.92E-61	postive
RFXAP	AC090517.2	0.51373563	2.29E-37	postive
UBR1	AC090517.2	0.573577187	3.99E-48	postive
MAPK8	AC090517.2	0.569491318	2.55E-47	postive
IREB2	AC090517.2	0.608377609	1.80E-55	postive
DDX17	AC090517.2	0.523482321	5.64E-39	postive
NFAT5	AC090517.2	0.705410836	1.04E-81	postive
PIK3CA	AC090517.2	0.504650001	6.51E-36	postive
GNRH1	AC090517.2	0.696779158	6.00E-79	postive
ACVR2A	AC090517.2	0.559699492	1.96E-45	postive
ANGPTL1	AC090517.2	0.608166126	2.00E-55	postive
BMPR1A	AC090517.2	0.526633162	1.66E-39	postive
BMPR2	AC090517.2	0.573030702	5.13E-48	postive
CRLF3	AC090517.2	0.567669571	5.79E-47	postive
NR2C1	AC090517.2	0.549028959	1.90E-43	postive
NR2C2	AC090517.2	0.580010315	2.04E-49	postive
RORA	AC090517.2	0.646182897	1.47E-64	postive
SOS1	AC090517.2	0.601199275	6.98E-54	postive
BRAF	AC090517.2	0.65821166	9.98E-68	postive
CBLB	AC090517.2	0.538402908	1.53E-41	postive
PDK1	AC090517.2	0.553067293	3.43E-44	postive
TRAJ1	AC090517.2	0.596141366	8.71E-53	postive
TRAJ2	AC090517.2	0.615529187	4.26E-57	postive
TRAJ3	AC090517.2	0.639053574	9.53E-63	postive
TRAJ5	AC090517.2	0.606860873	3.92E-55	postive
TRAJ6	AC090517.2	0.668097511	1.92E-70	postive
TRAJ8	AC090517.2	0.634110833	1.61E-61	postive
TRAJ10	AC090517.2	0.624736904	2.99E-59	postive
TRAJ12	AC090517.2	0.574584046	2.52E-48	postive
TRAJ13	AC090517.2	0.634604587	1.22E-61	postive
TRAJ14	AC090517.2	0.617506023	1.49E-57	postive
TRAJ16	AC090517.2	0.619550282	4.99E-58	postive
TRAJ17	AC090517.2	0.575498332	1.65E-48	postive
TRAJ18	AC090517.2	0.538293235	1.60E-41	postive
TRAJ21	AC090517.2	0.638485609	1.32E-62	postive
TRAJ31	AC090517.2	0.665617645	9.44E-70	postive

TRAJ37	AC090517.2	0.661005176	1.75E-68	postive
TRAJ38	AC090517.2	0.687868829	3.36E-76	postive
TRAJ39	AC090517.2	0.651358878	6.64E-66	postive
SLC10A2	LINC01671	0.544213191	1.42E-42	postive
LEFTY2	LINC01671	0.55385794	2.45E-44	postive
CREB1	RNF213-AS1	0.697306767	4.10E-79	postive
UBR1	RNF213-AS1	0.580812675	1.40E-49	postive
ZC3HAV1	RNF213-AS1	0.553323897	3.08E-44	postive
ZC3HAV1L	RNF213-AS1	0.50836169	1.68E-36	postive
IL15	RNF213-AS1	0.617516136	1.48E-57	postive
CYLD	RNF213-AS1	0.581944346	8.23E-50	postive
EIF2AK2	RNF213-AS1	0.57324434	4.65E-48	postive
NFKBIZ	RNF213-AS1	0.516789437	7.27E-38	postive
LMBR1	RNF213-AS1	0.503836249	8.74E-36	postive
IREB2	RNF213-AS1	0.531995014	2.01E-40	postive
DDX17	RNF213-AS1	0.582456518	6.46E-50	postive
PIK3CG	RNF213-AS1	0.547492887	3.62E-43	postive
JAK2	RNF213-AS1	0.667717889	2.45E-70	postive
TXK	RNF213-AS1	0.525119769	2.99E-39	postive
NFAT5	RNF213-AS1	0.708248204	1.23E-82	postive
PIK3R1	RNF213-AS1	0.576458386	1.06E-48	postive
PIK3CA	RNF213-AS1	0.630452231	1.26E-60	postive
PIK3CB	RNF213-AS1	0.531424086	2.52E-40	postive
RASGRP3	RNF213-AS1	0.549181597	1.78E-43	postive
PLXNC1	RNF213-AS1	0.526391048	1.83E-39	postive
GNRH1	RNF213-AS1	0.644723001	3.49E-64	postive
RABEP1	RNF213-AS1	0.521107117	1.41E-38	postive
ACVR2A	RNF213-AS1	0.53223718	1.83E-40	postive
ANGPTL1	RNF213-AS1	0.734596369	7.75E-92	postive
BMPR2	RNF213-AS1	0.577696594	5.99E-49	postive
CRLF3	RNF213-AS1	0.578784317	3.61E-49	postive
LIFR	RNF213-AS1	0.533105504	1.29E-40	postive
NR2C2	RNF213-AS1	0.695331874	1.70E-78	postive
RORA	RNF213-AS1	0.672917729	8.35E-72	postive
SOS1	RNF213-AS1	0.654839645	7.98E-67	postive
SOS2	RNF213-AS1	0.556904985	6.61E-45	postive
BRAF	RNF213-AS1	0.752960839	6.29E-99	postive
ITK	RNF213-AS1	0.512503967	3.63E-37	postive
CBL	RNF213-AS1	0.61644086	2.63E-57	postive
CBLB	RNF213-AS1	0.716526831	2.05E-85	postive
RASGRP1	RNF213-AS1	0.50929576	1.19E-36	postive
PDK1	RNF213-AS1	0.533893476	9.43E-41	postive
TRAJ1	RNF213-AS1	0.750768872	4.76E-98	postive
TRAJ2	RNF213-AS1	0.677370307	4.36E-73	postive
TRAJ3	RNF213-AS1	0.771984944	5.78E-107	postive
TRAJ5	RNF213-AS1	0.774490563	4.42E-108	postive
TRAJ6	RNF213-AS1	0.774964697	2.71E-108	postive
TRAJ8	RNF213-AS1	0.719008684	2.88E-86	postive
TRAJ10	RNF213-AS1	0.753942252	2.52E-99	postive
TRAJ12	RNF213-AS1	0.772321914	4.10E-107	postive
TRAJ13	RNF213-AS1	0.743949857	2.26E-95	postive
TRAJ14	RNF213-AS1	0.800124155	2.12E-120	postive
TRAJ16	RNF213-AS1	0.750079951	8.94E-98	postive
TRAJ17	RNF213-AS1	0.74536067	6.41E-96	postive
TRAJ18	RNF213-AS1	0.761624236	1.71E-102	postive
TRAJ21	RNF213-AS1	0.791372564	5.32E-116	postive
TRAJ31	RNF213-AS1	0.760437241	5.37E-102	postive
TRAJ37	RNF213-AS1	0.711000109	1.50E-83	postive
TRAJ38	RNF213-AS1	0.823601845	2.24E-133	postive
TRAJ39	RNF213-AS1	0.768185685	2.68E-105	postive
CREB1	AL139011.1	0.61521025	5.05E-57	postive
RFXAP	AL139011.1	0.514355992	1.82E-37	postive
UBR1	AL139011.1	0.535879997	4.25E-41	postive
CYLD	AL139011.1	0.554273641	2.05E-44	postive
DDX17	AL139011.1	0.683401239	7.37E-75	postive

JAK2	AL139011.1	0.505943531	4.07E-36	postive
TXK	AL139011.1	0.507027398	2.74E-36	postive
NFAT5	AL139011.1	0.755447131	6.17E-100	postive
NFATC3	AL139011.1	0.52267155	7.72E-39	postive
PIK3R1	AL139011.1	0.51810308	4.42E-38	postive
GNRH1	AL139011.1	0.691843632	2.06E-77	postive
IL6ST	AL139011.1	0.524075082	4.49E-39	postive
RABEP1	AL139011.1	0.507005176	2.76E-36	postive
ACVR2A	AL139011.1	0.544102748	1.48E-42	postive
ANGPTL1	AL139011.1	0.56882803	3.44E-47	postive
BMPR2	AL139011.1	0.513621465	2.39E-37	postive
CRLF3	AL139011.1	0.501585818	1.97E-35	postive
NR2C2	AL139011.1	0.720133475	1.17E-86	postive
RORA	AL139011.1	0.723857492	5.86E-88	postive
SOS1	AL139011.1	0.58133643	1.09E-49	postive
BRAF	AL139011.1	0.677131112	5.12E-73	postive
CBLB	AL139011.1	0.584414367	2.56E-50	postive
TRAJ1	AL139011.1	0.640388594	4.40E-63	postive
TRAJ2	AL139011.1	0.599403337	1.72E-53	postive
TRAJ3	AL139011.1	0.653652125	1.65E-66	postive
TRAJ5	AL139011.1	0.656029027	3.85E-67	postive
TRAJ6	AL139011.1	0.668885882	1.16E-70	postive
TRAJ8	AL139011.1	0.614771108	6.37E-57	postive
TRAJ10	AL139011.1	0.642314982	1.43E-63	postive
TRAJ12	AL139011.1	0.619379293	5.47E-58	postive
TRAJ13	AL139011.1	0.641381197	2.47E-63	postive
TRAJ14	AL139011.1	0.631456492	7.20E-61	postive
TRAJ16	AL139011.1	0.645228971	2.59E-64	postive
TRAJ17	AL139011.1	0.600501518	9.92E-54	postive
TRAJ18	AL139011.1	0.645053654	2.87E-64	postive
TRAJ21	AL139011.1	0.665340563	1.13E-69	postive
TRAJ31	AL139011.1	0.663321593	4.06E-69	postive
TRAJ37	AL139011.1	0.683343545	7.67E-75	postive
TRAJ38	AL139011.1	0.697048122	4.94E-79	postive
TRAJ39	AL139011.1	0.682159415	1.72E-74	postive
LMBR1L	AL022328.3	0.527958269	9.89E-40	postive
TYK2	AL022328.3	0.54990632	1.31E-43	postive
DDX17	AL022328.3	0.520386748	1.85E-38	postive
IRF9	AL022328.3	0.623549303	5.72E-59	postive
LTB4R2	AL022328.3	0.563464029	3.76E-46	postive
GNRH1	AL022328.3	0.533387845	1.15E-40	postive
CREB1	AC098484.1	0.588490269	3.65E-51	postive
UBR1	AC098484.1	0.552059618	5.27E-44	postive
IREB2	AC098484.1	0.534022246	8.96E-41	postive
NFAT5	AC098484.1	0.540854239	5.64E-42	postive
RABEP1	AC098484.1	0.519550686	2.55E-38	postive
ACVR2A	AC098484.1	0.515351556	1.25E-37	postive
BMPR1A	AC098484.1	0.515166305	1.34E-37	postive
NR2C2	AC098484.1	0.527741747	1.08E-39	postive
RORA	AC098484.1	0.516255403	8.90E-38	postive
SOS1	AC098484.1	0.508376692	1.67E-36	postive
BRAF	AC098484.1	0.580681077	1.49E-49	postive
CREB1	AP002907.1	0.735074867	5.15E-92	postive
UBR1	AP002907.1	0.569463231	2.59E-47	postive
ZC3HAV1	AP002907.1	0.553668473	2.65E-44	postive
ZC3HAV1L	AP002907.1	0.529814194	4.77E-40	postive
IL15	AP002907.1	0.551698459	6.14E-44	postive
CYLD	AP002907.1	0.540517531	6.48E-42	postive
EIF2AK2	AP002907.1	0.530246892	4.02E-40	postive
MAPK8	AP002907.1	0.572399924	6.83E-48	postive
LMBR1	AP002907.1	0.568885545	3.35E-47	postive
IREB2	AP002907.1	0.565420346	1.58E-46	postive
DDX17	AP002907.1	0.611735671	3.14E-56	postive
JAK2	AP002907.1	0.577746229	5.85E-49	postive
PTK2	AP002907.1	0.522670394	7.72E-39	postive

TXK	AP002907.1	0.547588352	3.48E-43	postive
NFAT5	AP002907.1	0.759618634	1.18E-101	postive
MALT1	AP002907.1	0.533127284	1.28E-40	postive
PIK3R1	AP002907.1	0.607046453	3.57E-55	postive
PIK3CA	AP002907.1	0.646741824	1.06E-64	postive
PIK3CB	AP002907.1	0.504533611	6.79E-36	postive
AKT3	AP002907.1	0.597329148	4.84E-53	postive
GNRH1	AP002907.1	0.709850433	3.62E-83	postive
IL6ST	AP002907.1	0.61563354	4.03E-57	postive
RABEP1	AP002907.1	0.529784635	4.82E-40	postive
ACVR2A	AP002907.1	0.595453786	1.22E-52	postive
ANGPTL1	AP002907.1	0.78819856	1.86E-114	postive
BMPR1A	AP002907.1	0.502748899	1.30E-35	postive
BMPR2	AP002907.1	0.673579884	5.40E-72	postive
CRLF3	AP002907.1	0.657224137	1.84E-67	postive
NR2C2	AP002907.1	0.67136717	2.30E-71	postive
RORA	AP002907.1	0.77642682	5.92E-109	postive
SOS1	AP002907.1	0.671602997	1.98E-71	postive
SOS2	AP002907.1	0.575242083	1.86E-48	postive
BRAF	AP002907.1	0.825727738	1.21E-134	postive
ITK	AP002907.1	0.559605796	2.05E-45	postive
CBL	AP002907.1	0.614152113	8.83E-57	postive
CBLB	AP002907.1	0.718059263	6.11E-86	postive
PDK1	AP002907.1	0.587477291	5.93E-51	postive
TRAJ1	AP002907.1	0.691994695	1.85E-77	postive
TRAJ2	AP002907.1	0.70591782	7.12E-82	postive
TRAJ3	AP002907.1	0.747610565	8.46E-97	postive
TRAJ5	AP002907.1	0.794696457	1.20E-117	postive
TRAJ6	AP002907.1	0.736638959	1.35E-92	postive
TRAJ8	AP002907.1	0.772127056	5.00E-107	postive
TRAJ10	AP002907.1	0.704781891	1.67E-81	postive
TRAJ12	AP002907.1	0.623814996	4.95E-59	postive
TRAJ13	AP002907.1	0.80152261	4.00E-121	postive
TRAJ14	AP002907.1	0.761579417	1.78E-102	postive
TRAJ16	AP002907.1	0.732317109	5.35E-91	postive
TRAJ17	AP002907.1	0.715526708	4.49E-85	postive
TRAJ18	AP002907.1	0.637024889	3.06E-62	postive
TRAJ21	AP002907.1	0.802041942	2.15E-121	postive
TRAJ31	AP002907.1	0.794013381	2.63E-117	postive
TRAJ37	AP002907.1	0.756738153	1.83E-100	postive
TRAJ38	AP002907.1	0.809633727	1.94E-125	postive
TRAJ39	AP002907.1	0.802698564	9.75E-122	postive
CREB1	AL158166.2	0.771050786	1.50E-106	postive
RFXAP	AL158166.2	0.50038852	3.02E-35	postive
UBR1	AL158166.2	0.641561422	2.23E-63	postive
ZC3HAV1	AL158166.2	0.536186508	3.76E-41	postive
ZC3HAV1L	AL158166.2	0.543272804	2.09E-42	postive
IL15	AL158166.2	0.619233095	5.91E-58	postive
CYLD	AL158166.2	0.557751339	4.58E-45	postive
MAPK8	AL158166.2	0.579810103	2.24E-49	postive
LMBR1	AL158166.2	0.592262532	5.86E-52	postive
IREB2	AL158166.2	0.61901032	6.66E-58	postive
DDX17	AL158166.2	0.559246849	2.39E-45	postive
PIK3CG	AL158166.2	0.502273765	1.54E-35	postive
JAK2	AL158166.2	0.596274145	8.16E-53	postive
TXK	AL158166.2	0.55189899	5.64E-44	postive
PPP3CB	AL158166.2	0.507031554	2.73E-36	postive
NFAT5	AL158166.2	0.819302812	7.36E-131	postive
MALT1	AL158166.2	0.535969155	4.10E-41	postive
PIK3R1	AL158166.2	0.615444053	4.46E-57	postive
PIK3CA	AL158166.2	0.65298104	2.48E-66	postive
AKT3	AL158166.2	0.513051342	2.96E-37	postive
RASGRP3	AL158166.2	0.510528772	7.56E-37	postive
IGHD4-4	AL158166.2	0.569650462	2.38E-47	postive
IGHD6-6	AL158166.2	0.501838141	1.80E-35	postive

GNRH1	AL158166.2	0.72775748	2.40E-89	postive
IL6ST	AL158166.2	0.626981832	8.70E-60	postive
RABEP1	AL158166.2	0.550367243	1.08E-43	postive
ACVR2A	AL158166.2	0.62108846	2.18E-58	postive
ANGPTL1	AL158166.2	0.738132044	3.72E-93	postive
BMPR1A	AL158166.2	0.573756819	3.68E-48	postive
BMPR2	AL158166.2	0.724962454	2.38E-88	postive
CRLF3	AL158166.2	0.620400327	3.16E-58	postive
NR2C2	AL158166.2	0.677818674	3.23E-73	postive
NR3C1	AL158166.2	0.522784007	7.39E-39	postive
RORA	AL158166.2	0.820622391	1.26E-131	postive
SOS1	AL158166.2	0.649037715	2.68E-65	postive
SOS2	AL158166.2	0.559276426	2.36E-45	postive
BRAF	AL158166.2	0.775104543	2.34E-108	postive
CBL	AL158166.2	0.569233744	2.87E-47	postive
CBLB	AL158166.2	0.667249172	3.32E-70	postive
PDK1	AL158166.2	0.598863557	2.25E-53	postive
TRAJ1	AL158166.2	0.667648423	2.57E-70	postive
TRAJ2	AL158166.2	0.693019883	8.91E-78	postive
TRAJ3	AL158166.2	0.760211459	6.67E-102	postive
TRAJ5	AL158166.2	0.746847208	1.69E-96	postive
TRAJ6	AL158166.2	0.748513514	3.73E-97	postive
TRAJ8	AL158166.2	0.709124004	6.30E-83	postive
TRAJ10	AL158166.2	0.75513146	8.30E-100	postive
TRAJ12	AL158166.2	0.661598715	1.20E-68	postive
TRAJ13	AL158166.2	0.727819184	2.28E-89	postive
TRAJ14	AL158166.2	0.705903183	7.20E-82	postive
TRAJ16	AL158166.2	0.716363109	2.33E-85	postive
TRAJ17	AL158166.2	0.706629068	4.17E-82	postive
TRAJ18	AL158166.2	0.678092772	2.69E-73	postive
TRAJ21	AL158166.2	0.754166517	2.05E-99	postive
TRAJ31	AL158166.2	0.797217406	6.47E-119	postive
TRAJ37	AL158166.2	0.790557915	1.33E-115	postive
TRAJ38	AL158166.2	0.813062558	2.52E-127	postive
TRAJ39	AL158166.2	0.785988317	2.14E-113	postive
GNRH1	AL391834.2	0.540180781	7.43E-42	postive
CREB1	AC011442.1	0.607979745	2.21E-55	postive
UBR1	AC011442.1	0.51892135	3.24E-38	postive
DDX17	AC011442.1	0.507689853	2.15E-36	postive
NFAT5	AC011442.1	0.653521662	1.79E-66	postive
PIK3R1	AC011442.1	0.501102045	2.34E-35	postive
PIK3CA	AC011442.1	0.559176792	2.47E-45	postive
GNRH1	AC011442.1	0.584183643	2.86E-50	postive
ACVR2A	AC011442.1	0.538082738	1.75E-41	postive
ANGPTL1	AC011442.1	0.567928979	5.16E-47	postive
BMPR2	AC011442.1	0.550366871	1.08E-43	postive
NR2C2	AC011442.1	0.609000655	1.30E-55	postive
RORA	AC011442.1	0.607119709	3.43E-55	postive
SOS1	AC011442.1	0.508750693	1.46E-36	postive
SOS2	AC011442.1	0.503917403	8.49E-36	postive
BRAF	AC011442.1	0.635265743	8.36E-62	postive
CBL	AC011442.1	0.509809404	9.86E-37	postive
CBLB	AC011442.1	0.541477187	4.37E-42	postive
TRAJ1	AC011442.1	0.598547967	2.64E-53	postive
TRAJ2	AC011442.1	0.535907709	4.20E-41	postive
TRAJ3	AC011442.1	0.653619758	1.68E-66	postive
TRAJ5	AC011442.1	0.603062727	2.72E-54	postive
TRAJ6	AC011442.1	0.63734546	2.55E-62	postive
TRAJ8	AC011442.1	0.602301879	4.00E-54	postive
TRAJ10	AC011442.1	0.636867652	3.35E-62	postive
TRAJ12	AC011442.1	0.591275221	9.47E-52	postive
TRAJ13	AC011442.1	0.600635913	9.27E-54	postive
TRAJ14	AC011442.1	0.61179029	3.05E-56	postive
TRAJ16	AC011442.1	0.623271727	6.66E-59	postive
TRAJ17	AC011442.1	0.633995472	1.72E-61	postive

TRAJ18	AC011442.1	0.613177095	1.47E-56	postive
TRAJ21	AC011442.1	0.647109262	8.49E-65	postive
TRAJ31	AC011442.1	0.631088773	8.85E-61	postive
TRAJ37	AC011442.1	0.606669305	4.33E-55	postive
TRAJ38	AC011442.1	0.646922958	9.49E-65	postive
TRAJ39	AC011442.1	0.655699227	4.71E-67	postive
CREB1	AC096586.2	0.787886667	2.63E-114	postive
RFXAP	AC096586.2	0.562158506	6.69E-46	postive
UBR1	AC096586.2	0.664206711	2.32E-69	postive
ZC3HAV1	AC096586.2	0.569211449	2.90E-47	postive
ZC3HAV1L	AC096586.2	0.541588502	4.18E-42	postive
IL15	AC096586.2	0.609445149	1.03E-55	postive
CYLD	AC096586.2	0.614896911	5.96E-57	postive
EIF2AK2	AC096586.2	0.513800689	2.24E-37	postive
MAPK8	AC096586.2	0.579629325	2.44E-49	postive
LMBR1	AC096586.2	0.550082918	1.22E-43	postive
IREB2	AC096586.2	0.619808695	4.34E-58	postive
DDX17	AC096586.2	0.636918181	3.25E-62	postive
PIK3CG	AC096586.2	0.508697717	1.48E-36	postive
JAK2	AC096586.2	0.62485511	2.80E-59	postive
TXK	AC096586.2	0.539787599	8.73E-42	postive
NFAT5	AC096586.2	0.866513639	5.74E-163	postive
NFATC3	AC096586.2	0.508849852	1.40E-36	postive
MALT1	AC096586.2	0.538940978	1.23E-41	postive
PIK3R1	AC096586.2	0.591590849	8.13E-52	postive
PIK3CA	AC096586.2	0.649655586	1.85E-65	postive
AKT3	AC096586.2	0.544396523	1.31E-42	postive
RASGRP3	AC096586.2	0.510958429	6.44E-37	postive
IGHD4-4	AC096586.2	0.515472384	1.20E-37	postive
LTB4R2	AC096586.2	0.536662445	3.10E-41	postive
GNRH1	AC096586.2	0.771664638	8.01E-107	postive
IL6ST	AC096586.2	0.587673983	5.40E-51	postive
RABEP1	AC096586.2	0.584767437	2.16E-50	postive
ACVR2A	AC096586.2	0.658047886	1.11E-67	postive
ANGPTL1	AC096586.2	0.730577096	2.31E-90	postive
BMPR1A	AC096586.2	0.581146314	1.20E-49	postive
BMPR2	AC096586.2	0.685331079	1.96E-75	postive
CRLF3	AC096586.2	0.645828057	1.82E-64	postive
NR1D2	AC096586.2	0.512088809	4.24E-37	postive
NR2C1	AC096586.2	0.528316667	8.59E-40	postive
NR2C2	AC096586.2	0.738491963	2.72E-93	postive
RORA	AC096586.2	0.79722128	6.44E-119	postive
SOS1	AC096586.2	0.674158133	3.69E-72	postive
SOS2	AC096586.2	0.605686884	7.16E-55	postive
BRAF	AC096586.2	0.78956687	4.05E-115	postive
CBL	AC096586.2	0.595185122	1.40E-52	postive
CBLB	AC096586.2	0.678590255	1.93E-73	postive
PDK1	AC096586.2	0.563320099	4.01E-46	postive
TRAJ1	AC096586.2	0.759976626	8.36E-102	postive
TRAJ2	AC096586.2	0.7895552	4.11E-115	postive
TRAJ3	AC096586.2	0.83115012	5.80E-138	postive
TRAJ5	AC096586.2	0.761355484	2.21E-102	postive
TRAJ6	AC096586.2	0.835370553	1.25E-140	postive
TRAJ8	AC096586.2	0.794187587	2.16E-117	postive
TRAJ10	AC096586.2	0.820539554	1.41E-131	postive
TRAJ12	AC096586.2	0.729672238	4.91E-90	postive
TRAJ13	AC096586.2	0.793030717	8.10E-117	postive
TRAJ14	AC096586.2	0.790405235	1.58E-115	postive
TRAJ16	AC096586.2	0.799417439	4.89E-120	postive
TRAJ17	AC096586.2	0.763380423	3.10E-103	postive
TRAJ18	AC096586.2	0.733605236	1.80E-91	postive
TRAJ21	AC096586.2	0.806828203	6.36E-124	postive
TRAJ31	AC096586.2	0.827532272	9.77E-136	postive
TRAJ37	AC096586.2	0.830452244	1.57E-137	postive
TRAJ38	AC096586.2	0.867183778	1.65E-163	postive

TRAJ39	AC096586.2	0.820055776	2.70E-131	postive
CREB1	AC138956.1	0.668625277	1.37E-70	postive
RFXAP	AC138956.1	0.527645994	1.12E-39	postive
UBR1	AC138956.1	0.522768399	7.43E-39	postive
IL15	AC138956.1	0.510477481	7.70E-37	postive
CYLD	AC138956.1	0.590958686	1.11E-51	postive
NFKBIZ	AC138956.1	0.520992594	1.47E-38	postive
DDX17	AC138956.1	0.702244691	1.10E-80	postive
JAK2	AC138956.1	0.541740553	3.93E-42	postive
TXK	AC138956.1	0.518503737	3.80E-38	postive
NFAT5	AC138956.1	0.76693092	9.37E-105	postive
PIK3R1	AC138956.1	0.574220195	2.97E-48	postive
PIK3CA	AC138956.1	0.58620594	1.09E-50	postive
LTB4R2	AC138956.1	0.566195828	1.12E-46	postive
GNRH1	AC138956.1	0.746426264	2.46E-96	postive
IL6ST	AC138956.1	0.601219841	6.91E-54	postive
ACVR2A	AC138956.1	0.578032615	5.12E-49	postive
ANGPTL1	AC138956.1	0.667657519	2.55E-70	postive
BMPR2	AC138956.1	0.598172573	3.18E-53	postive
CRLF3	AC138956.1	0.51593906	1.00E-37	postive
NR2C1	AC138956.1	0.533893552	9.43E-41	postive
NR2C2	AC138956.1	0.724564244	3.30E-88	postive
RORA	AC138956.1	0.79462275	1.31E-117	postive
SOS1	AC138956.1	0.622488611	1.02E-58	postive
SOS2	AC138956.1	0.543892281	1.62E-42	postive
BRAF	AC138956.1	0.721651254	3.48E-87	postive
ITK	AC138956.1	0.532058567	1.96E-40	postive
CBL	AC138956.1	0.554866658	1.59E-44	postive
CBLB	AC138956.1	0.623377668	6.28E-59	postive
TRAJ1	AC138956.1	0.722037909	2.55E-87	postive
TRAJ2	AC138956.1	0.665965113	7.56E-70	postive
TRAJ3	AC138956.1	0.725461112	1.59E-88	postive
TRAJ5	AC138956.1	0.770924558	1.70E-106	postive
TRAJ6	AC138956.1	0.711909579	7.45E-84	postive
TRAJ8	AC138956.1	0.712598471	4.38E-84	postive
TRAJ10	AC138956.1	0.720803519	6.87E-87	postive
TRAJ12	AC138956.1	0.664135556	2.42E-69	postive
TRAJ13	AC138956.1	0.739502796	1.13E-93	postive
TRAJ14	AC138956.1	0.713756187	1.78E-84	postive
TRAJ16	AC138956.1	0.744645979	1.21E-95	postive
TRAJ17	AC138956.1	0.734827211	6.36E-92	postive
TRAJ18	AC138956.1	0.698653414	1.54E-79	postive
TRAJ21	AC138956.1	0.762466263	7.54E-103	postive
TRAJ31	AC138956.1	0.772347397	3.99E-107	postive
TRAJ37	AC138956.1	0.753268166	4.73E-99	postive
TRAJ38	AC138956.1	0.771055389	1.49E-106	postive
TRAJ39	AC138956.1	0.77301372	2.02E-107	postive
CREB1	AC007406.5	0.502578478	1.38E-35	postive
DDX17	AC007406.5	0.520974468	1.48E-38	postive
NR2C2	AC007406.5	0.523188585	6.32E-39	postive
RORA	AC007406.5	0.534499838	7.40E-41	postive
SOS1	AC007406.5	0.521395458	1.26E-38	postive
CREB1	AC010201.2	0.715787785	3.66E-85	postive
RFXAP	AC010201.2	0.561680813	8.25E-46	postive
UBR1	AC010201.2	0.621263638	1.98E-58	postive
ZC3HAV1	AC010201.2	0.521072961	1.43E-38	postive
CYLD	AC010201.2	0.601365334	6.42E-54	postive
MAPK8	AC010201.2	0.541549726	4.24E-42	postive
IREB2	AC010201.2	0.536962482	2.75E-41	postive
DDX17	AC010201.2	0.718425995	4.57E-86	postive
JAK2	AC010201.2	0.54718452	4.11E-43	postive
TXK	AC010201.2	0.54116641	4.97E-42	postive
NFAT5	AC010201.2	0.873234427	1.56E-168	postive
NFATC3	AC010201.2	0.579705329	2.35E-49	postive
PIK3R1	AC010201.2	0.566020748	1.21E-46	postive

PIK3CA	AC010201.2	0.545889509	7.06E-43	postive
ROBO2	AC010201.2	0.504208495	7.64E-36	postive
GNRH1	AC010201.2	0.832100048	1.48E-138	postive
IL6ST	AC010201.2	0.566340479	1.05E-46	postive
RABEP1	AC010201.2	0.533557978	1.08E-40	postive
ACVR2A	AC010201.2	0.651004956	8.22E-66	postive
ANGPTL1	AC010201.2	0.601187346	7.03E-54	postive
BMPR1A	AC010201.2	0.534920142	6.25E-41	postive
BMPR2	AC010201.2	0.639135129	9.09E-63	postive
CRLF3	AC010201.2	0.570932688	1.33E-47	postive
NR1D2	AC010201.2	0.507643225	2.19E-36	postive
NR2C1	AC010201.2	0.601399674	6.31E-54	postive
NR2C2	AC010201.2	0.772367817	3.91E-107	postive
RORA	AC010201.2	0.804585561	9.93E-123	postive
SOS1	AC010201.2	0.638744181	1.14E-62	postive
SOS2	AC010201.2	0.547810668	3.17E-43	postive
BRAF	AC010201.2	0.753994285	2.40E-99	postive
CBL	AC010201.2	0.509967904	9.30E-37	postive
CBLB	AC010201.2	0.592675744	4.79E-52	postive
TRAJ1	AC010201.2	0.770329603	3.10E-106	postive
TRAJ2	AC010201.2	0.728924295	9.14E-90	postive
TRAJ3	AC010201.2	0.791602238	4.10E-116	postive
TRAJ5	AC010201.2	0.767515237	5.24E-105	postive
TRAJ6	AC010201.2	0.803088118	6.10E-122	postive
TRAJ8	AC010201.2	0.765772021	2.95E-104	postive
TRAJ10	AC010201.2	0.78134512	3.27E-111	postive
TRAJ12	AC010201.2	0.762182294	9.94E-103	postive
TRAJ13	AC010201.2	0.779531871	2.26E-110	postive
TRAJ14	AC010201.2	0.760687151	4.22E-102	postive
TRAJ16	AC010201.2	0.778925587	4.29E-110	postive
TRAJ17	AC010201.2	0.744643339	1.22E-95	postive
TRAJ18	AC010201.2	0.74800317	5.93E-97	postive
TRAJ21	AC010201.2	0.794993495	8.54E-118	postive
TRAJ31	AC010201.2	0.812826265	3.41E-127	postive
TRAJ37	AC010201.2	0.835692222	7.79E-141	postive
TRAJ38	AC010201.2	0.823526694	2.49E-133	postive
TRAJ39	AC010201.2	0.813927202	8.32E-128	postive
CREB1	AP000692.1	0.718261897	5.21E-86	postive
RFXAP	AP000692.1	0.541756265	3.90E-42	postive
UBR1	AP000692.1	0.598062476	3.36E-53	postive
IL15	AP000692.1	0.535959601	4.12E-41	postive
CYLD	AP000692.1	0.625027506	2.55E-59	postive
NFKBIZ	AP000692.1	0.579174155	3.01E-49	postive
IREB2	AP000692.1	0.526038296	2.09E-39	postive
DDX17	AP000692.1	0.688976224	1.55E-76	postive
PIK3CG	AP000692.1	0.52064122	1.68E-38	postive
JAK2	AP000692.1	0.648179816	4.49E-65	postive
TXK	AP000692.1	0.569112769	3.03E-47	postive
NFAT5	AP000692.1	0.821032269	7.29E-132	postive
MALT1	AP000692.1	0.525581131	2.50E-39	postive
PIK3R1	AP000692.1	0.562856888	4.92E-46	postive
PIK3CA	AP000692.1	0.639304955	8.24E-63	postive
RASGRP3	AP000692.1	0.540559598	6.37E-42	postive
LTB4R2	AP000692.1	0.558502771	3.31E-45	postive
GNRH1	AP000692.1	0.785262838	4.73E-113	postive
IL6ST	AP000692.1	0.529564813	5.26E-40	postive
RABEP1	AP000692.1	0.53542313	5.11E-41	postive
ACVR2A	AP000692.1	0.600525188	9.80E-54	postive
ANGPTL1	AP000692.1	0.668465055	1.52E-70	postive
BMPR2	AP000692.1	0.644581829	3.79E-64	postive
CRLF3	AP000692.1	0.586959613	7.60E-51	postive
NR2C1	AP000692.1	0.567395049	6.55E-47	postive
NR2C2	AP000692.1	0.754000159	2.39E-99	postive
RORA	AP000692.1	0.774049839	6.96E-108	postive
SOS1	AP000692.1	0.644044829	5.20E-64	postive

SOS2	AP000692.1	0.542256116	3.18E-42	postive
BRAF	AP000692.1	0.685717144	1.50E-75	postive
CBL	AP000692.1	0.596640809	6.81E-53	postive
CBLB	AP000692.1	0.690616658	4.89E-77	postive
PDK1	AP000692.1	0.547327735	3.88E-43	postive
TRAJ1	AP000692.1	0.706497826	4.60E-82	postive
TRAJ2	AP000692.1	0.667521743	2.79E-70	postive
TRAJ3	AP000692.1	0.73328762	2.35E-91	postive
TRAJ5	AP000692.1	0.694442998	3.23E-78	postive
TRAJ6	AP000692.1	0.733725131	1.63E-91	postive
TRAJ8	AP000692.1	0.638215031	1.55E-62	postive
TRAJ10	AP000692.1	0.740305603	5.61E-94	postive
TRAJ12	AP000692.1	0.654337437	1.09E-66	postive
TRAJ13	AP000692.1	0.66654297	5.22E-70	postive
TRAJ14	AP000692.1	0.660088138	3.10E-68	postive
TRAJ16	AP000692.1	0.734728722	6.92E-92	postive
TRAJ17	AP000692.1	0.677051109	5.40E-73	postive
TRAJ18	AP000692.1	0.712430821	4.98E-84	postive
TRAJ21	AP000692.1	0.705010568	1.41E-81	postive
TRAJ31	AP000692.1	0.723040978	1.13E-87	postive
TRAJ37	AP000692.1	0.71826255	5.20E-86	postive
TRAJ38	AP000692.1	0.772392252	3.82E-107	postive
TRAJ39	AP000692.1	0.722959225	1.21E-87	postive
TYK2	LINC00265	0.549022751	1.90E-43	postive
LTB4R	LINC00265	0.528114873	9.30E-40	postive
IRF9	LINC00265	0.509527333	1.09E-36	postive
LTB4R2	LINC00265	0.512918683	3.11E-37	postive
B2M	USP30-AS1	0.652517976	3.29E-66	postive
CD4	USP30-AS1	0.514487166	1.73E-37	postive
CD8A	USP30-AS1	0.627696658	5.86E-60	postive
CD8B	USP30-AS1	0.556093402	9.38E-45	postive
CD74	USP30-AS1	0.510400968	7.92E-37	postive
CTSS	USP30-AS1	0.555780672	1.07E-44	postive
FCER1G	USP30-AS1	0.578535079	4.06E-49	postive
HLA-B	USP30-AS1	0.592412656	5.44E-52	postive
HLA-C	USP30-AS1	0.567054366	7.63E-47	postive
HLA-DMB	USP30-AS1	0.595633248	1.12E-52	postive
HLA-DOB	USP30-AS1	0.506820479	2.95E-36	postive
HLA-DPB1	USP30-AS1	0.501719086	1.88E-35	postive
HLA-DQA1	USP30-AS1	0.543781072	1.69E-42	postive
HLA-DRA	USP30-AS1	0.552196181	4.97E-44	postive
HLA-E	USP30-AS1	0.588289154	4.02E-51	postive
HLA-F	USP30-AS1	0.626524119	1.12E-59	postive
IFNG	USP30-AS1	0.564343573	2.55E-46	postive
LTA	USP30-AS1	0.595636588	1.12E-52	postive
PSMB8	USP30-AS1	0.590160753	1.63E-51	postive
PSME2	USP30-AS1	0.556136551	9.21E-45	postive
TAP1	USP30-AS1	0.594548409	1.91E-52	postive
TAP2	USP30-AS1	0.574113389	3.12E-48	postive
CXCL10	USP30-AS1	0.573419828	4.29E-48	postive
CXCL9	USP30-AS1	0.623568973	5.66E-59	postive
APOBEC3G	USP30-AS1	0.692170218	1.63E-77	postive
STAT1	USP30-AS1	0.547186146	4.11E-43	postive
IRF1	USP30-AS1	0.74262875	7.28E-95	postive
CD40	USP30-AS1	0.588493273	3.64E-51	postive
CCL5	USP30-AS1	0.695160782	1.93E-78	postive
CCL4	USP30-AS1	0.668801988	1.22E-70	postive
PDCD1	USP30-AS1	0.613434411	1.29E-56	postive
FASLG	USP30-AS1	0.635768525	6.28E-62	postive
GBP2	USP30-AS1	0.559116693	2.53E-45	postive
CCL3	USP30-AS1	0.526820859	1.54E-39	postive
CCR5	USP30-AS1	0.625896926	1.58E-59	postive
CCL4L2	USP30-AS1	0.59787326	3.69E-53	postive
XCL2	USP30-AS1	0.593810558	2.75E-52	postive
CXCR6	USP30-AS1	0.6308498	1.01E-60	postive

CD86	USP30-AS1	0.523124883	6.48E-39	postive
BTK	USP30-AS1	0.581558735	9.86E-50	postive
RAC2	USP30-AS1	0.60683912	3.97E-55	postive
CD72	USP30-AS1	0.687843368	3.42E-76	postive
PTPN6	USP30-AS1	0.524516458	3.78E-39	postive
TYMP	USP30-AS1	0.508406537	1.65E-36	postive
CMKLR1	USP30-AS1	0.512693017	3.38E-37	postive
CXCR3	USP30-AS1	0.51842468	3.92E-38	postive
EBI3	USP30-AS1	0.560582154	1.34E-45	postive
FLT3LG	USP30-AS1	0.635366548	7.89E-62	postive
GMFG	USP30-AS1	0.651903983	4.77E-66	postive
IL16	USP30-AS1	0.522313453	8.86E-39	postive
TNFSF13B	USP30-AS1	0.614851733	6.10E-57	postive
CSF2RB	USP30-AS1	0.537276111	2.42E-41	postive
IL10RA	USP30-AS1	0.586256025	1.06E-50	postive
IL12RB1	USP30-AS1	0.694406211	3.31E-78	postive
IL15RA	USP30-AS1	0.642462462	1.32E-63	postive
IL2RB	USP30-AS1	0.637586135	2.22E-62	postive
IL18RAP	USP30-AS1	0.5796089	2.46E-49	postive
IL21R	USP30-AS1	0.602448393	3.72E-54	postive
IL2RG	USP30-AS1	0.600957791	7.89E-54	postive
NR1H3	USP30-AS1	0.527457125	1.20E-39	postive
ITGAL	USP30-AS1	0.639692618	6.59E-63	postive
TYROBP	USP30-AS1	0.554811635	1.63E-44	postive
LCK	USP30-AS1	0.632054569	5.15E-61	postive
NCR3	USP30-AS1	0.560565934	1.35E-45	postive
CD247	USP30-AS1	0.762610736	6.55E-103	postive
ZAP70	USP30-AS1	0.628031711	4.87E-60	postive
LCP2	USP30-AS1	0.567518971	6.20E-47	postive
HCST	USP30-AS1	0.687730399	3.70E-76	postive
CD48	USP30-AS1	0.590041119	1.72E-51	postive
CD244	USP30-AS1	0.61087624	4.91E-56	postive
SH2D1A	USP30-AS1	0.626991617	8.65E-60	postive
GZMB	USP30-AS1	0.594783205	1.70E-52	postive
PRF1	USP30-AS1	0.58648467	9.54E-51	postive
CD3D	USP30-AS1	0.727338217	3.40E-89	postive
CD3E	USP30-AS1	0.704019523	2.95E-81	postive
CD3G	USP30-AS1	0.57480107	2.28E-48	postive
ICOS	USP30-AS1	0.533467387	1.12E-40	postive
TRAC	USP30-AS1	0.695141134	1.95E-78	postive
TRAV4	USP30-AS1	0.585803955	1.32E-50	postive
TRAV8-2	USP30-AS1	0.584959584	1.98E-50	postive
TRAV8-3	USP30-AS1	0.516264352	8.87E-38	postive
TRAV8-4	USP30-AS1	0.548591637	2.28E-43	postive
TRAV8-6	USP30-AS1	0.53127366	2.68E-40	postive
TRAV9-2	USP30-AS1	0.503038286	1.17E-35	postive
TRAV12-2	USP30-AS1	0.527973099	9.83E-40	postive
TRAV16	USP30-AS1	0.589363424	2.39E-51	postive
TRAV21	USP30-AS1	0.516396788	8.44E-38	postive
TRAV29DV5	USP30-AS1	0.513029552	2.98E-37	postive
TRBC1	USP30-AS1	0.652967972	2.50E-66	postive
TRBC2	USP30-AS1	0.710846792	1.69E-83	postive
TRBJ2-2	USP30-AS1	0.517685418	5.18E-38	postive
TRBJ2-3	USP30-AS1	0.575741624	1.48E-48	postive
TRBJ2-7	USP30-AS1	0.516827324	7.17E-38	postive
TRBV2	USP30-AS1	0.543499549	1.90E-42	postive
TRBV3-1	USP30-AS1	0.516450658	8.27E-38	postive
TRBV5-1	USP30-AS1	0.585299165	1.68E-50	postive
TRBV5-4	USP30-AS1	0.515443278	1.21E-37	postive
TRBV6-5	USP30-AS1	0.58046649	1.65E-49	postive
TRBV7-9	USP30-AS1	0.587767278	5.16E-51	postive
TRBV9	USP30-AS1	0.530587287	3.51E-40	postive
TRBV10-3	USP30-AS1	0.501120105	2.33E-35	postive
TRBV11-2	USP30-AS1	0.520192294	2.00E-38	postive
TRBV18	USP30-AS1	0.560945505	1.14E-45	postive

TRBV20-1	USP30-AS1	0.560290775	1.52E-45	postive
TRBV28	USP30-AS1	0.702890157	6.83E-81	postive
SCTR	AC013275.1	0.564071736	2.88E-46	postive
RASGRP1	AC013275.1	0.529529575	5.33E-40	postive
DDX17	AP000254.2	0.574421594	2.71E-48	postive
GNRH1	AP000254.2	0.616631959	2.37E-57	postive
NR2C2	AP000254.2	0.540261764	7.19E-42	postive
RORA	AP000254.2	0.513983592	2.09E-37	postive
CBL	CCDC84-DT	0.51056551	7.45E-37	postive
CREB1	PSMA3-AS1	0.62616426	1.37E-59	postive
RFXAP	PSMA3-AS1	0.628320886	4.14E-60	postive
UBR1	PSMA3-AS1	0.521163838	1.38E-38	postive
CYLD	PSMA3-AS1	0.54316385	2.19E-42	postive
DDX17	PSMA3-AS1	0.763717112	2.23E-103	postive
JAK2	PSMA3-AS1	0.506198121	3.71E-36	postive
TXK	PSMA3-AS1	0.503243829	1.08E-35	postive
NFAT5	PSMA3-AS1	0.710525132	2.16E-83	postive
PIK3R1	PSMA3-AS1	0.538606733	1.41E-41	postive
LTB4R2	PSMA3-AS1	0.563421986	3.83E-46	postive
GNRH1	PSMA3-AS1	0.804372672	1.29E-122	postive
IL6ST	PSMA3-AS1	0.551242232	7.46E-44	postive
RABEP1	PSMA3-AS1	0.532154193	1.89E-40	postive
ACVR2A	PSMA3-AS1	0.579298999	2.84E-49	postive
ANGPTL1	PSMA3-AS1	0.578675888	3.80E-49	postive
BMPR2	PSMA3-AS1	0.535890899	4.23E-41	postive
CRLF3	PSMA3-AS1	0.528058666	9.51E-40	postive
NR2C1	PSMA3-AS1	0.634417195	1.35E-61	postive
NR2C2	PSMA3-AS1	0.761967478	1.22E-102	postive
RORA	PSMA3-AS1	0.70672159	3.89E-82	postive
SOS1	PSMA3-AS1	0.563489841	3.72E-46	postive
SOS2	PSMA3-AS1	0.545558792	8.10E-43	postive
BRAF	PSMA3-AS1	0.701899953	1.42E-80	postive
CBLB	PSMA3-AS1	0.552625641	4.14E-44	postive
TRAJ1	PSMA3-AS1	0.647463001	6.88E-65	postive
TRAJ2	PSMA3-AS1	0.656337228	3.18E-67	postive
TRAJ3	PSMA3-AS1	0.687671684	3.86E-76	postive
TRAJ5	PSMA3-AS1	0.626014164	1.48E-59	postive
TRAJ6	PSMA3-AS1	0.649749773	1.75E-65	postive
TRAJ8	PSMA3-AS1	0.63012787	1.52E-60	postive
TRAJ10	PSMA3-AS1	0.619482084	5.17E-58	postive
TRAJ12	PSMA3-AS1	0.588971057	2.89E-51	postive
TRAJ13	PSMA3-AS1	0.623348178	6.38E-59	postive
TRAJ14	PSMA3-AS1	0.624952711	2.66E-59	postive
TRAJ16	PSMA3-AS1	0.638281807	1.49E-62	postive
TRAJ17	PSMA3-AS1	0.599599561	1.56E-53	postive
TRAJ18	PSMA3-AS1	0.59966796	1.51E-53	postive
TRAJ21	PSMA3-AS1	0.64353672	7.01E-64	postive
TRAJ31	PSMA3-AS1	0.639501763	7.36E-63	postive
TRAJ37	PSMA3-AS1	0.656086787	3.71E-67	postive
TRAJ38	PSMA3-AS1	0.680310503	6.04E-74	postive
TRAJ39	PSMA3-AS1	0.663206886	4.37E-69	postive
CREB1	AC092279.1	0.61600481	3.31E-57	postive
UBR1	AC092279.1	0.547394616	3.77E-43	postive
ZC3HAV1L	AC092279.1	0.525779952	2.32E-39	postive
IREB2	AC092279.1	0.528308444	8.62E-40	postive
NFAT5	AC092279.1	0.582703199	5.75E-50	postive
AKT3	AC092279.1	0.538535729	1.45E-41	postive
IGHD4-4	AC092279.1	0.504633511	6.55E-36	postive
GNRH1	AC092279.1	0.610442442	6.16E-56	postive
ACVR2A	AC092279.1	0.560975485	1.12E-45	postive
ANGPTL1	AC092279.1	0.553210425	3.23E-44	postive
BMPR1A	AC092279.1	0.512574852	3.53E-37	postive
BMPR2	AC092279.1	0.535622757	4.71E-41	postive
CRLF3	AC092279.1	0.539940211	8.20E-42	postive
NR2C2	AC092279.1	0.596075599	9.00E-53	postive

RORA	AC092279.1	0.526978025	1.45E-39	postive
SOS1	AC092279.1	0.586925793	7.73E-51	postive
SOS2	AC092279.1	0.522082562	9.68E-39	postive
BRAF	AC092279.1	0.685727042	1.49E-75	postive
TRAJ1	AC092279.1	0.608543842	1.65E-55	postive
TRAJ2	AC092279.1	0.592038816	6.53E-52	postive
TRAJ3	AC092279.1	0.660131211	3.02E-68	postive
TRAJ5	AC092279.1	0.618095404	1.09E-57	postive
TRAJ6	AC092279.1	0.641356789	2.51E-63	postive
TRAJ8	AC092279.1	0.71249043	4.76E-84	postive
TRAJ10	AC092279.1	0.59516589	1.41E-52	postive
TRAJ12	AC092279.1	0.555083423	1.45E-44	postive
TRAJ13	AC092279.1	0.653460623	1.85E-66	postive
TRAJ14	AC092279.1	0.659880012	3.53E-68	postive
TRAJ16	AC092279.1	0.606976503	3.70E-55	postive
TRAJ17	AC092279.1	0.624795652	2.89E-59	postive
TRAJ21	AC092279.1	0.654405014	1.04E-66	postive
TRAJ31	AC092279.1	0.621958644	1.36E-58	postive
TRAJ37	AC092279.1	0.610198683	6.99E-56	postive
TRAJ38	AC092279.1	0.646691098	1.09E-64	postive
TRAJ39	AC092279.1	0.679890304	8.03E-74	postive
SLPI	AC129507.2	0.563654756	3.46E-46	postive
DDX17	AC011462.4	0.521303681	1.31E-38	postive
IRF9	AC011462.4	0.535059678	5.91E-41	postive
GNRH1	AC011462.4	0.597826615	3.78E-53	postive
CREB1	AC078846.1	0.674272111	3.42E-72	postive
RFXAP	AC078846.1	0.540461191	6.63E-42	postive
UBR1	AC078846.1	0.574723516	2.36E-48	postive
ZC3HAV1	AC078846.1	0.548062506	2.85E-43	postive
ZC3HAV1L	AC078846.1	0.594714129	1.76E-52	postive
EIF2AK2	AC078846.1	0.501433524	2.08E-35	postive
MAPK8	AC078846.1	0.524754912	3.45E-39	postive
LMBR1	AC078846.1	0.546642849	5.16E-43	postive
IREB2	AC078846.1	0.55553766	1.19E-44	postive
DDX17	AC078846.1	0.584959639	1.98E-50	postive
JAK2	AC078846.1	0.501530452	2.01E-35	postive
NFAT5	AC078846.1	0.735994657	2.35E-92	postive
PIK3CA	AC078846.1	0.542620653	2.73E-42	postive
AKT3	AC078846.1	0.566802044	8.54E-47	postive
IGHD6-6	AC078846.1	0.518875882	3.30E-38	postive
GNRH1	AC078846.1	0.719816797	1.51E-86	postive
RABEP1	AC078846.1	0.51492393	1.47E-37	postive
ACVR2A	AC078846.1	0.561574694	8.65E-46	postive
ANGPTL1	AC078846.1	0.61103547	4.52E-56	postive
BMPR2	AC078846.1	0.558725508	3.00E-45	postive
CRLF3	AC078846.1	0.626761081	9.82E-60	postive
NR2C2	AC078846.1	0.689250825	1.28E-76	postive
RORA	AC078846.1	0.653674903	1.63E-66	postive
SOS1	AC078846.1	0.649919029	1.58E-65	postive
SOS2	AC078846.1	0.529240016	5.98E-40	postive
BRAF	AC078846.1	0.784657784	9.15E-113	postive
CBL	AC078846.1	0.576534353	1.03E-48	postive
CBLB	AC078846.1	0.584900119	2.03E-50	postive
PDK1	AC078846.1	0.554994838	1.50E-44	postive
TRAJ1	AC078846.1	0.746830797	1.71E-96	postive
TRAJ2	AC078846.1	0.704253505	2.48E-81	postive
TRAJ3	AC078846.1	0.77419919	5.97E-108	postive
TRAJ5	AC078846.1	0.732364473	5.14E-91	postive
TRAJ6	AC078846.1	0.759210777	1.74E-101	postive
TRAJ8	AC078846.1	0.792007978	2.59E-116	postive
TRAJ10	AC078846.1	0.694659443	2.76E-78	postive
TRAJ12	AC078846.1	0.671788724	1.75E-71	postive
TRAJ13	AC078846.1	0.757604186	8.05E-101	postive
TRAJ14	AC078846.1	0.760849932	3.61E-102	postive
TRAJ16	AC078846.1	0.732109188	6.37E-91	postive

TRAJ17	AC078846.1	0.706637946	4.14E-82	postive
TRAJ18	AC078846.1	0.623544643	5.74E-59	postive
TRAJ21	AC078846.1	0.763684099	2.30E-103	postive
TRAJ31	AC078846.1	0.737864082	4.69E-93	postive
TRAJ37	AC078846.1	0.734557732	8.01E-92	postive
TRAJ38	AC078846.1	0.770205017	3.52E-106	postive
TRAJ39	AC078846.1	0.789722945	3.40E-115	postive
CIITA	AL135818.1	0.519794919	2.32E-38	postive
CREB1	AC063965.2	0.771701012	7.72E-107	postive
UBR1	AC063965.2	0.63486286	1.05E-61	postive
ZC3HAV1	AC063965.2	0.540706764	6.00E-42	postive
IL15	AC063965.2	0.610257477	6.78E-56	postive
CYLD	AC063965.2	0.600021418	1.26E-53	postive
EIF2AK2	AC063965.2	0.51011474	8.80E-37	postive
MAPK8	AC063965.2	0.570083089	1.95E-47	postive
TLR1	AC063965.2	0.517077142	6.53E-38	postive
LMBR1	AC063965.2	0.575321369	1.79E-48	postive
LIMS1	AC063965.2	0.53826167	1.62E-41	postive
IREB2	AC063965.2	0.591669689	7.82E-52	postive
DDX17	AC063965.2	0.580663276	1.50E-49	postive
PIK3CG	AC063965.2	0.542678847	2.67E-42	postive
JAK2	AC063965.2	0.653236525	2.13E-66	postive
TXK	AC063965.2	0.598433185	2.79E-53	postive
NFAT5	AC063965.2	0.837346124	6.67E-142	postive
MALT1	AC063965.2	0.547048907	4.35E-43	postive
PIK3R1	AC063965.2	0.61906399	6.48E-58	postive
PIK3CA	AC063965.2	0.693565498	6.04E-78	postive
PIK3CB	AC063965.2	0.515630428	1.13E-37	postive
AKT3	AC063965.2	0.558190369	3.79E-45	postive
RASGRP3	AC063965.2	0.503548799	9.70E-36	postive
GNRH1	AC063965.2	0.739502713	1.13E-93	postive
IL6ST	AC063965.2	0.606122693	5.73E-55	postive
RABEP1	AC063965.2	0.55324494	3.18E-44	postive
ACVR2A	AC063965.2	0.576196794	1.20E-48	postive
ANGPTL1	AC063965.2	0.795974249	2.75E-118	postive
BMPR1A	AC063965.2	0.526863069	1.52E-39	postive
BMPR2	AC063965.2	0.716114257	2.83E-85	postive
CRLF3	AC063965.2	0.652042665	4.39E-66	postive
IL18R1	AC063965.2	0.533184928	1.25E-40	postive
NR2C2	AC063965.2	0.647150661	8.28E-65	postive
RORA	AC063965.2	0.822602918	8.74E-133	postive
SOS1	AC063965.2	0.644911153	3.12E-64	postive
SOS2	AC063965.2	0.550315549	1.10E-43	postive
BRAF	AC063965.2	0.767691314	4.39E-105	postive
ITK	AC063965.2	0.511285905	5.71E-37	postive
CBL	AC063965.2	0.620710167	2.67E-58	postive
CBLB	AC063965.2	0.756331071	2.68E-100	postive
PDK1	AC063965.2	0.625573463	1.89E-59	postive
TRAJ1	AC063965.2	0.662925	5.22E-69	postive
TRAJ2	AC063965.2	0.721079137	5.51E-87	postive
TRAJ3	AC063965.2	0.760182815	6.86E-102	postive
TRAJ5	AC063965.2	0.713135237	2.89E-84	postive
TRAJ6	AC063965.2	0.758306998	4.13E-101	postive
TRAJ8	AC063965.2	0.685132286	2.24E-75	postive
TRAJ10	AC063965.2	0.746399121	2.52E-96	postive
TRAJ12	AC063965.2	0.630610658	1.16E-60	postive
TRAJ13	AC063965.2	0.725768585	1.23E-88	postive
TRAJ14	AC063965.2	0.707108785	2.90E-82	postive
TRAJ16	AC063965.2	0.739874771	8.18E-94	postive
TRAJ17	AC063965.2	0.681899461	2.06E-74	postive
TRAJ18	AC063965.2	0.70061141	3.67E-80	postive
TRAJ21	AC063965.2	0.764856685	7.29E-104	postive
TRAJ31	AC063965.2	0.767902516	3.56E-105	postive
TRAJ37	AC063965.2	0.75618121	3.09E-100	postive
TRAJ38	AC063965.2	0.824478706	6.75E-134	postive

TRAJ39	AC063965.2	0.771113888	1.40E-106	postive
CREB1	AC004908.2	0.695230557	1.83E-78	postive
RFXAP	AC004908.2	0.588438408	3.74E-51	postive
UBR1	AC004908.2	0.579100221	3.12E-49	postive
ZC3HAV1L	AC004908.2	0.514178552	1.94E-37	postive
CYLD	AC004908.2	0.525914347	2.20E-39	postive
MAPK8	AC004908.2	0.548750545	2.13E-43	postive
IREB2	AC004908.2	0.558566876	3.22E-45	postive
DDX17	AC004908.2	0.671371155	2.30E-71	postive
JAK2	AC004908.2	0.538405823	1.53E-41	postive
TXK	AC004908.2	0.546069933	6.55E-43	postive
NFAT5	AC004908.2	0.755506486	5.83E-100	postive
PIK3R1	AC004908.2	0.579173871	3.01E-49	postive
PIK3CA	AC004908.2	0.556636763	7.42E-45	postive
IGHD6-6	AC004908.2	0.508528385	1.58E-36	postive
GNRH1	AC004908.2	0.815213609	1.58E-128	postive
IL6ST	AC004908.2	0.588107927	4.38E-51	postive
RABEP1	AC004908.2	0.522758979	7.46E-39	postive
ACVR2A	AC004908.2	0.602926239	2.92E-54	postive
ANGPTL1	AC004908.2	0.637070675	2.98E-62	postive
BMPR2	AC004908.2	0.646262105	1.40E-64	postive
CRLF3	AC004908.2	0.569603197	2.43E-47	postive
NR2C1	AC004908.2	0.589540827	2.20E-51	postive
NR2C2	AC004908.2	0.720928474	6.22E-87	postive
RORA	AC004908.2	0.792317185	1.83E-116	postive
SOS1	AC004908.2	0.629941721	1.68E-60	postive
BRAF	AC004908.2	0.738876816	1.95E-93	postive
CBL	AC004908.2	0.52424185	4.21E-39	postive
CBLB	AC004908.2	0.637828743	1.93E-62	postive
PDK1	AC004908.2	0.554019381	2.28E-44	postive
TRAJ1	AC004908.2	0.611129733	4.31E-56	postive
TRAJ2	AC004908.2	0.621433642	1.81E-58	postive
TRAJ3	AC004908.2	0.668658724	1.34E-70	postive
TRAJ5	AC004908.2	0.680859901	4.16E-74	postive
TRAJ6	AC004908.2	0.653130485	2.27E-66	postive
TRAJ8	AC004908.2	0.627923154	5.17E-60	postive
TRAJ10	AC004908.2	0.627041197	8.42E-60	postive
TRAJ12	AC004908.2	0.579890334	2.16E-49	postive
TRAJ13	AC004908.2	0.644195776	4.76E-64	postive
TRAJ14	AC004908.2	0.614835913	6.15E-57	postive
TRAJ16	AC004908.2	0.644035489	5.23E-64	postive
TRAJ17	AC004908.2	0.600965053	7.86E-54	postive
TRAJ18	AC004908.2	0.592056325	6.48E-52	postive
TRAJ21	AC004908.2	0.662237507	8.05E-69	postive
TRAJ31	AC004908.2	0.692749515	1.08E-77	postive
TRAJ37	AC004908.2	0.696776519	6.01E-79	postive
TRAJ38	AC004908.2	0.714365307	1.11E-84	postive
TRAJ39	AC004908.2	0.696739636	6.18E-79	postive
CREB1	AC007216.3	0.790908738	8.98E-116	postive
RFXAP	AC007216.3	0.530607466	3.48E-40	postive
UBR1	AC007216.3	0.654627835	9.09E-67	postive
ZC3HAV1	AC007216.3	0.574050743	3.21E-48	postive
ZC3HAV1L	AC007216.3	0.516257675	8.89E-38	postive
IL15	AC007216.3	0.587698569	5.34E-51	postive
CYLD	AC007216.3	0.605618281	7.42E-55	postive
EIF2AK2	AC007216.3	0.544360566	1.33E-42	postive
MAPK8	AC007216.3	0.588666898	3.35E-51	postive
NFKBIZ	AC007216.3	0.504741935	6.30E-36	postive
LMBR1	AC007216.3	0.584022643	3.08E-50	postive
LIMS1	AC007216.3	0.517327376	5.94E-38	postive
IREB2	AC007216.3	0.617703366	1.34E-57	postive
DDX17	AC007216.3	0.636730822	3.62E-62	postive
PIK3CG	AC007216.3	0.557251921	5.69E-45	postive
JAK2	AC007216.3	0.637462407	2.38E-62	postive
TXK	AC007216.3	0.611943429	2.82E-56	postive

NFAT5	AC007216.3	0.833256767	2.77E-139	postive
MALT1	AC007216.3	0.576668309	9.64E-49	postive
PIK3R1	AC007216.3	0.683705548	5.98E-75	postive
PIK3CA	AC007216.3	0.694910202	2.31E-78	postive
PIK3CB	AC007216.3	0.526718473	1.61E-39	postive
AKT3	AC007216.3	0.544751026	1.13E-42	postive
RASGRP3	AC007216.3	0.538334999	1.58E-41	postive
IGHD4-4	AC007216.3	0.512294981	3.92E-37	postive
IGHD6-6	AC007216.3	0.535707837	4.56E-41	postive
GNRH1	AC007216.3	0.757101793	1.30E-100	postive
IL6ST	AC007216.3	0.70643853	4.81E-82	postive
RABEP1	AC007216.3	0.561419429	9.26E-46	postive
ACVR2A	AC007216.3	0.655602502	5.00E-67	postive
ANGPTL1	AC007216.3	0.801769548	2.98E-121	postive
BMPR1A	AC007216.3	0.569225925	2.88E-47	postive
BMPR2	AC007216.3	0.749337596	1.76E-97	postive
CRLF3	AC007216.3	0.653500189	1.81E-66	postive
NR1D2	AC007216.3	0.515263985	1.29E-37	postive
NR2C1	AC007216.3	0.508318644	1.71E-36	postive
NR2C2	AC007216.3	0.720896349	6.38E-87	postive
NR3C1	AC007216.3	0.501499787	2.03E-35	postive
RORA	AC007216.3	0.885096677	3.52E-179	postive
SOS1	AC007216.3	0.66940187	8.27E-71	postive
SOS2	AC007216.3	0.593022006	4.04E-52	postive
BRAF	AC007216.3	0.841939871	6.23E-145	postive
ITK	AC007216.3	0.55394886	2.35E-44	postive
CBL	AC007216.3	0.586837136	8.06E-51	postive
CBLB	AC007216.3	0.743641771	2.97E-95	postive
RASGRP1	AC007216.3	0.534683678	6.87E-41	postive
PDK1	AC007216.3	0.616966164	1.99E-57	postive
TRAJ1	AC007216.3	0.683744997	5.82E-75	postive
TRAJ2	AC007216.3	0.722125645	2.38E-87	postive
TRAJ3	AC007216.3	0.760622624	4.49E-102	postive
TRAJ5	AC007216.3	0.790984158	8.25E-116	postive
TRAJ6	AC007216.3	0.751286084	2.96E-98	postive
TRAJ8	AC007216.3	0.731791646	8.32E-91	postive
TRAJ10	AC007216.3	0.742200131	1.06E-94	postive
TRAJ12	AC007216.3	0.648084995	4.75E-65	postive
TRAJ13	AC007216.3	0.75970118	1.09E-101	postive
TRAJ14	AC007216.3	0.730156369	3.28E-90	postive
TRAJ16	AC007216.3	0.748218762	4.88E-97	postive
TRAJ17	AC007216.3	0.716977788	1.44E-85	postive
TRAJ18	AC007216.3	0.687716881	3.74E-76	postive
TRAJ21	AC007216.3	0.787579896	3.70E-114	postive
TRAJ31	AC007216.3	0.818186887	3.23E-130	postive
TRAJ37	AC007216.3	0.786060712	1.97E-113	postive
TRAJ38	AC007216.3	0.837664742	4.14E-142	postive
TRAJ39	AC007216.3	0.816182492	4.48E-129	postive
CREB1	AC000123.1	0.734617808	7.61E-92	postive
UBR1	AC000123.1	0.581795156	8.83E-50	postive
ZC3HAV1	AC000123.1	0.564909149	1.98E-46	postive
ZC3HAV1L	AC000123.1	0.548767388	2.12E-43	postive
IL15	AC000123.1	0.556204624	8.94E-45	postive
CYLD	AC000123.1	0.551703432	6.13E-44	postive
EIF2AK2	AC000123.1	0.500420559	2.99E-35	postive
MAPK8	AC000123.1	0.527854305	1.03E-39	postive
NFKBIZ	AC000123.1	0.528543883	7.86E-40	postive
LMBR1	AC000123.1	0.580224291	1.84E-49	postive
IREB2	AC000123.1	0.556816003	6.87E-45	postive
DDX17	AC000123.1	0.58526087	1.71E-50	postive
JAK2	AC000123.1	0.584550327	2.40E-50	postive
NFAT5	AC000123.1	0.767921382	3.49E-105	postive
PIK3R1	AC000123.1	0.555704214	1.11E-44	postive
PIK3CA	AC000123.1	0.632638514	3.70E-61	postive
PIK3CB	AC000123.1	0.517231173	6.16E-38	postive

RASGRP3	AC000123.1	0.522556153	8.07E-39	postive
GNRH1	AC000123.1	0.733329455	2.27E-91	postive
IL6ST	AC000123.1	0.520268747	1.94E-38	postive
RABEP1	AC000123.1	0.537405032	2.30E-41	postive
ACVR2A	AC000123.1	0.623234068	6.79E-59	postive
ANGPTL1	AC000123.1	0.691603338	2.44E-77	postive
BMPR1A	AC000123.1	0.525506121	2.58E-39	postive
BMPR2	AC000123.1	0.672002209	1.52E-71	postive
CRLF3	AC000123.1	0.546790484	4.85E-43	postive
NR2C2	AC000123.1	0.696514388	7.27E-79	postive
RORA	AC000123.1	0.744756183	1.10E-95	postive
SOS1	AC000123.1	0.688066264	2.93E-76	postive
SOS2	AC000123.1	0.56406258	2.89E-46	postive
BRAF	AC000123.1	0.771633564	8.27E-107	postive
CBL	AC000123.1	0.578317643	4.49E-49	postive
CBLB	AC000123.1	0.666574773	5.12E-70	postive
PDK1	AC000123.1	0.517357531	5.87E-38	postive
TRAJ1	AC000123.1	0.700695084	3.45E-80	postive
TRAJ2	AC000123.1	0.635021375	9.61E-62	postive
TRAJ3	AC000123.1	0.710715639	1.87E-83	postive
TRAJ5	AC000123.1	0.767042403	8.39E-105	postive
TRAJ6	AC000123.1	0.732562529	4.35E-91	postive
TRAJ8	AC000123.1	0.706686984	3.99E-82	postive
TRAJ10	AC000123.1	0.731636463	9.48E-91	postive
TRAJ12	AC000123.1	0.699881077	6.28E-80	postive
TRAJ13	AC000123.1	0.739637554	1.01E-93	postive
TRAJ14	AC000123.1	0.714764871	8.14E-85	postive
TRAJ16	AC000123.1	0.717187057	1.22E-85	postive
TRAJ17	AC000123.1	0.714737715	8.31E-85	postive
TRAJ18	AC000123.1	0.656162953	3.54E-67	postive
TRAJ21	AC000123.1	0.741827153	1.48E-94	postive
TRAJ31	AC000123.1	0.799685846	3.56E-120	postive
TRAJ37	AC000123.1	0.750280163	7.45E-98	postive
TRAJ38	AC000123.1	0.793360117	5.56E-117	postive
TRAJ39	AC000123.1	0.740039427	7.08E-94	postive
IL1B	MIR3945HG	0.523632358	5.33E-39	postive
MARCO	MIR3945HG	0.550859993	8.77E-44	postive
AQP9	MIR3945HG	0.536725748	3.02E-41	postive
FPR2	MIR3945HG	0.654975496	7.35E-67	postive
LTB4R2	AC020594.1	0.500518804	2.89E-35	postive
CREB1	AC005838.3	0.767510725	5.26E-105	postive
RFXAP	AC005838.3	0.518944707	3.21E-38	postive
UBR1	AC005838.3	0.68830882	2.47E-76	postive
ZC3HAV1	AC005838.3	0.561908563	7.47E-46	postive
ZC3HAV1L	AC005838.3	0.508112897	1.84E-36	postive
IL15	AC005838.3	0.571941481	8.42E-48	postive
CYLD	AC005838.3	0.607258559	3.20E-55	postive
EIF2AK2	AC005838.3	0.539565712	9.55E-42	postive
MAPK8	AC005838.3	0.555992728	9.79E-45	postive
LMBR1	AC005838.3	0.557865583	4.36E-45	postive
IREB2	AC005838.3	0.610427526	6.21E-56	postive
DDX17	AC005838.3	0.630022181	1.61E-60	postive
PIK3CG	AC005838.3	0.533981047	9.11E-41	postive
JAK2	AC005838.3	0.641720054	2.03E-63	postive
TXK	AC005838.3	0.629241959	2.48E-60	postive
NFAT5	AC005838.3	0.865158039	7.00E-162	postive
NFATC3	AC005838.3	0.519397475	2.70E-38	postive
MALT1	AC005838.3	0.529162788	6.16E-40	postive
PIK3R1	AC005838.3	0.62608084	1.43E-59	postive
PIK3CA	AC005838.3	0.661469547	1.31E-68	postive
PIK3CB	AC005838.3	0.523823181	4.95E-39	postive
AKT3	AC005838.3	0.526994242	1.44E-39	postive
RASGRP3	AC005838.3	0.502705937	1.32E-35	postive
IGHD6-6	AC005838.3	0.555585128	1.17E-44	postive
GNRH1	AC005838.3	0.799490768	4.48E-120	postive

IL6ST	AC005838.3	0.582156388	7.45E-50	postive
RABEP1	AC005838.3	0.598584811	2.59E-53	postive
ACVR2A	AC005838.3	0.614448719	7.55E-57	postive
ANGPTL1	AC005838.3	0.730032408	3.63E-90	postive
BMPR1A	AC005838.3	0.549442123	1.60E-43	postive
BMPR2	AC005838.3	0.700229659	4.86E-80	postive
CRLF3	AC005838.3	0.646132238	1.52E-64	postive
NR2C2	AC005838.3	0.712863518	3.57E-84	postive
RORA	AC005838.3	0.800737664	1.02E-120	postive
SOS1	AC005838.3	0.663530826	3.56E-69	postive
SOS2	AC005838.3	0.558587307	3.19E-45	postive
BRAF	AC005838.3	0.785140235	5.41E-113	postive
TEC	AC005838.3	0.588959055	2.91E-51	postive
CBL	AC005838.3	0.613824012	1.05E-56	postive
CBLB	AC005838.3	0.708969661	7.08E-83	postive
RASGRP1	AC005838.3	0.520685117	1.65E-38	postive
PDK1	AC005838.3	0.593665231	2.95E-52	postive
TRAJ1	AC005838.3	0.743560607	3.19E-95	postive
TRAJ2	AC005838.3	0.731622313	9.60E-91	postive
TRAJ3	AC005838.3	0.811570459	1.69E-126	postive
TRAJ5	AC005838.3	0.743652631	2.94E-95	postive
TRAJ6	AC005838.3	0.812118345	8.42E-127	postive
TRAJ8	AC005838.3	0.73507423	5.15E-92	postive
TRAJ10	AC005838.3	0.775279992	1.95E-108	postive
TRAJ12	AC005838.3	0.730847248	1.84E-90	postive
TRAJ13	AC005838.3	0.747695678	7.84E-97	postive
TRAJ14	AC005838.3	0.757673703	7.53E-101	postive
TRAJ16	AC005838.3	0.763164375	3.82E-103	postive
TRAJ17	AC005838.3	0.719070992	2.74E-86	postive
TRAJ18	AC005838.3	0.753467347	3.93E-99	postive
TRAJ21	AC005838.3	0.796812667	1.04E-118	postive
TRAJ31	AC005838.3	0.787406981	4.48E-114	postive
TRAJ37	AC005838.3	0.794594789	1.35E-117	postive
TRAJ38	AC005838.3	0.83747882	5.47E-142	postive
TRAJ39	AC005838.3	0.817103267	1.34E-129	postive
CREB1	AC112496.1	0.505710393	4.43E-36	postive
NFKBIZ	AC112496.1	0.531187914	2.77E-40	postive
JAK2	AC112496.1	0.523957487	4.70E-39	postive
NFAT5	AC112496.1	0.558899592	2.78E-45	postive
GNRH1	AC112496.1	0.527042918	1.42E-39	postive
ANGPTL1	AC112496.1	0.570184826	1.87E-47	postive
RORA	AC112496.1	0.535808446	4.38E-41	postive
CBLB	AC112496.1	0.565039447	1.87E-46	postive
PDK1	AC112496.1	0.537325168	2.37E-41	postive
TRAJ10	AC112496.1	0.541092947	5.12E-42	postive
TRAJ38	AC112496.1	0.528191168	9.03E-40	postive
CREB1	AC087286.4	0.77082323	1.88E-106	postive
UBR1	AC087286.4	0.665248252	1.19E-69	postive
ZC3HAV1	AC087286.4	0.538196318	1.67E-41	postive
IL15	AC087286.4	0.541112831	5.08E-42	postive
CYLD	AC087286.4	0.558029141	4.06E-45	postive
EIF2AK2	AC087286.4	0.54493295	1.05E-42	postive
MAPK8	AC087286.4	0.571857205	8.75E-48	postive
LMBR1	AC087286.4	0.57575959	1.47E-48	postive
LIMS1	AC087286.4	0.521052673	1.44E-38	postive
IREB2	AC087286.4	0.629873354	1.75E-60	postive
DDX17	AC087286.4	0.576639856	9.77E-49	postive
PIK3CG	AC087286.4	0.511421417	5.43E-37	postive
JAK2	AC087286.4	0.619993032	3.93E-58	postive
TXK	AC087286.4	0.648508952	3.68E-65	postive
NFAT5	AC087286.4	0.747891338	6.56E-97	postive
MALT1	AC087286.4	0.519441019	2.66E-38	postive
PIK3R1	AC087286.4	0.652118244	4.19E-66	postive
PIK3CA	AC087286.4	0.6878407	3.43E-76	postive
PIK3CB	AC087286.4	0.540089824	7.72E-42	postive

AKT3	AC087286.4	0.509599083	1.07E-36	postive
ROBO2	AC087286.4	0.53165284	2.30E-40	postive
GNRH1	AC087286.4	0.730642182	2.18E-90	postive
IL6ST	AC087286.4	0.620442827	3.08E-58	postive
RABEP1	AC087286.4	0.549523418	1.54E-43	postive
ACVR2A	AC087286.4	0.615958865	3.40E-57	postive
ANGPTL1	AC087286.4	0.77670995	4.41E-109	postive
BMPR1A	AC087286.4	0.569514986	2.53E-47	postive
BMPR2	AC087286.4	0.779215401	3.16E-110	postive
CRLF3	AC087286.4	0.602199633	4.22E-54	postive
NR1D2	AC087286.4	0.560578645	1.34E-45	postive
NR2C2	AC087286.4	0.649993741	1.51E-65	postive
RORA	AC087286.4	0.80683305	6.32E-124	postive
SOS1	AC087286.4	0.654796019	8.20E-67	postive
SOS2	AC087286.4	0.527754742	1.07E-39	postive
BRAF	AC087286.4	0.781298554	3.44E-111	postive
CBL	AC087286.4	0.563808319	3.23E-46	postive
CBLB	AC087286.4	0.768742661	1.54E-105	postive
PDK1	AC087286.4	0.590498925	1.38E-51	postive
TRAJ1	AC087286.4	0.546376317	5.77E-43	postive
TRAJ2	AC087286.4	0.616533861	2.50E-57	postive
TRAJ3	AC087286.4	0.637592239	2.21E-62	postive
TRAJ5	AC087286.4	0.630631741	1.14E-60	postive
TRAJ6	AC087286.4	0.629740564	1.88E-60	postive
TRAJ8	AC087286.4	0.579829802	2.22E-49	postive
TRAJ10	AC087286.4	0.592021017	6.59E-52	postive
TRAJ12	AC087286.4	0.578165001	4.82E-49	postive
TRAJ13	AC087286.4	0.613400159	1.31E-56	postive
TRAJ14	AC087286.4	0.595905961	9.79E-53	postive
TRAJ16	AC087286.4	0.610890413	4.88E-56	postive
TRAJ17	AC087286.4	0.558898575	2.78E-45	postive
TRAJ18	AC087286.4	0.580995985	1.28E-49	postive
TRAJ21	AC087286.4	0.64824856	4.30E-65	postive
TRAJ31	AC087286.4	0.689173428	1.35E-76	postive
TRAJ37	AC087286.4	0.642529938	1.26E-63	postive
TRAJ38	AC087286.4	0.739762697	9.02E-94	postive
TRAJ39	AC087286.4	0.653438801	1.88E-66	postive
CREB1	AC124312.2	0.703573125	4.11E-81	postive
RFXAP	AC124312.2	0.532965528	1.37E-40	postive
UBR1	AC124312.2	0.672989224	7.96E-72	postive
ZC3HAV1	AC124312.2	0.512159355	4.13E-37	postive
CYLD	AC124312.2	0.645160427	2.70E-64	postive
MAPK8	AC124312.2	0.507509832	2.30E-36	postive
IREB2	AC124312.2	0.574288503	2.88E-48	postive
DDX17	AC124312.2	0.6455508	2.14E-64	postive
JAK2	AC124312.2	0.553127206	3.35E-44	postive
TXK	AC124312.2	0.586006174	1.20E-50	postive
NFAT5	AC124312.2	0.878770039	2.31E-173	postive
NFATC3	AC124312.2	0.650788143	9.37E-66	postive
MALT1	AC124312.2	0.510188924	8.57E-37	postive
PIK3R1	AC124312.2	0.636957201	3.18E-62	postive
PIK3CA	AC124312.2	0.579976791	2.07E-49	postive
IGHD6-6	AC124312.2	0.573152129	4.85E-48	postive
ROBO2	AC124312.2	0.50340344	1.02E-35	postive
GNRH1	AC124312.2	0.696370759	8.06E-79	postive
IL6ST	AC124312.2	0.652006353	4.49E-66	postive
RABEP1	AC124312.2	0.559473334	2.17E-45	postive
ACVR2A	AC124312.2	0.589609565	2.12E-51	postive
ANGPTL1	AC124312.2	0.599618245	1.54E-53	postive
BMPR1A	AC124312.2	0.526673498	1.63E-39	postive
BMPR2	AC124312.2	0.661728186	1.11E-68	postive
CRLF3	AC124312.2	0.520998911	1.47E-38	postive
NR1D2	AC124312.2	0.508177656	1.80E-36	postive
NR2C2	AC124312.2	0.679917694	7.88E-74	postive
NR3C2	AC124312.2	0.501108552	2.34E-35	postive

RORA	AC124312.2	0.852374915	3.45E-152	postive
SOS1	AC124312.2	0.580596989	1.55E-49	postive
SOS2	AC124312.2	0.50310928	1.14E-35	postive
BRAF	AC124312.2	0.68542622	1.83E-75	postive
TEC	AC124312.2	0.530775451	3.26E-40	postive
CBL	AC124312.2	0.514729777	1.58E-37	postive
CBLB	AC124312.2	0.575266289	1.84E-48	postive
TRAJ1	AC124312.2	0.66883149	1.20E-70	postive
TRAJ2	AC124312.2	0.718312929	5.00E-86	postive
TRAJ3	AC124312.2	0.760804839	3.77E-102	postive
TRAJ5	AC124312.2	0.669158303	9.69E-71	postive
TRAJ6	AC124312.2	0.747227504	1.20E-96	postive
TRAJ8	AC124312.2	0.660854457	1.92E-68	postive
TRAJ10	AC124312.2	0.746718656	1.89E-96	postive
TRAJ12	AC124312.2	0.675721356	1.31E-72	postive
TRAJ13	AC124312.2	0.677272551	4.66E-73	postive
TRAJ14	AC124312.2	0.667076626	3.71E-70	postive
TRAJ16	AC124312.2	0.726605377	6.21E-89	postive
TRAJ17	AC124312.2	0.681009739	3.76E-74	postive
TRAJ18	AC124312.2	0.760288468	6.19E-102	postive
TRAJ21	AC124312.2	0.73425531	1.04E-91	postive
TRAJ31	AC124312.2	0.739101601	1.60E-93	postive
TRAJ37	AC124312.2	0.795836151	3.22E-118	postive
TRAJ38	AC124312.2	0.770530983	2.53E-106	postive
TRAJ39	AC124312.2	0.768763697	1.50E-105	postive
CREB1	AC005674.2	0.569120884	3.02E-47	postive
CIITA	AC005674.2	0.516883672	7.02E-38	postive
RFXAP	AC005674.2	0.523505993	5.59E-39	postive
CYLD	AC005674.2	0.586104926	1.14E-50	postive
DDX17	AC005674.2	0.709971975	3.30E-83	postive
JAK2	AC005674.2	0.579485638	2.60E-49	postive
TXK	AC005674.2	0.545330289	8.91E-43	postive
NFAT5	AC005674.2	0.662100788	8.78E-69	postive
PIK3R1	AC005674.2	0.525482191	2.60E-39	postive
RASGRP3	AC005674.2	0.511324039	5.63E-37	postive
LTB4R2	AC005674.2	0.522430381	8.47E-39	postive
GNRH1	AC005674.2	0.75389057	2.65E-99	postive
ACVR2A	AC005674.2	0.552067127	5.25E-44	postive
ANGPTL1	AC005674.2	0.50937737	1.16E-36	postive
BMPR2	AC005674.2	0.525143896	2.97E-39	postive
CRLF3	AC005674.2	0.51782812	4.91E-38	postive
NR2C1	AC005674.2	0.609270492	1.13E-55	postive
NR2C2	AC005674.2	0.687108691	5.71E-76	postive
RORA	AC005674.2	0.671629181	1.94E-71	postive
SOS1	AC005674.2	0.52108532	1.42E-38	postive
BRAF	AC005674.2	0.56866925	3.70E-47	postive
CBLB	AC005674.2	0.550628616	9.67E-44	postive
TRAJ1	AC005674.2	0.635936415	5.71E-62	postive
TRAJ2	AC005674.2	0.584789935	2.14E-50	postive
TRAJ3	AC005674.2	0.630531937	1.21E-60	postive
TRAJ5	AC005674.2	0.600485862	1.00E-53	postive
TRAJ6	AC005674.2	0.604209096	1.52E-54	postive
TRAJ8	AC005674.2	0.563383795	3.90E-46	postive
TRAJ10	AC005674.2	0.58001018	2.04E-49	postive
TRAJ12	AC005674.2	0.558597619	3.17E-45	postive
TRAJ13	AC005674.2	0.568801621	3.48E-47	postive
TRAJ14	AC005674.2	0.563972238	3.01E-46	postive
TRAJ16	AC005674.2	0.609362863	1.08E-55	postive
TRAJ17	AC005674.2	0.56996709	2.06E-47	postive
TRAJ18	AC005674.2	0.585342532	1.65E-50	postive
TRAJ21	AC005674.2	0.589020679	2.82E-51	postive
TRAJ31	AC005674.2	0.598358344	2.90E-53	postive
TRAJ37	AC005674.2	0.597441215	4.57E-53	postive
TRAJ38	AC005674.2	0.624549444	3.31E-59	postive
TRAJ39	AC005674.2	0.617928171	1.19E-57	postive

CREB1	GABPB1-AS1	0.512924904	3.10E-37	postive
DDX17	GABPB1-AS1	0.633192438	2.71E-61	postive
NFAT5	GABPB1-AS1	0.572682271	6.01E-48	postive
LTB4R2	GABPB1-AS1	0.524564496	3.71E-39	postive
GNRH1	GABPB1-AS1	0.639830801	6.08E-63	postive
ANGPTL1	GABPB1-AS1	0.583712522	3.57E-50	postive
NR2C2	GABPB1-AS1	0.701320348	2.18E-80	postive
RORA	GABPB1-AS1	0.612089413	2.61E-56	postive
SOS1	GABPB1-AS1	0.518011544	4.58E-38	postive
BRAF	GABPB1-AS1	0.629715469	1.91E-60	postive
CBLB	GABPB1-AS1	0.52042074	1.83E-38	postive
TRAJ3	GABPB1-AS1	0.525854432	2.25E-39	postive
TRAJ31	GABPB1-AS1	0.501755643	1.85E-35	postive
TRAJ38	GABPB1-AS1	0.523582393	5.43E-39	postive
TRAJ39	GABPB1-AS1	0.509347263	1.17E-36	postive
CREB1	AL355075.2	0.696730741	6.22E-79	postive
RFXAP	AL355075.2	0.566021088	1.21E-46	postive
UBR1	AL355075.2	0.573840211	3.54E-48	postive
CYLD	AL355075.2	0.601498529	6.01E-54	postive
MAPK8	AL355075.2	0.53175445	2.21E-40	postive
IREB2	AL355075.2	0.506592433	3.21E-36	postive
DDX17	AL355075.2	0.696922662	5.41E-79	postive
JAK2	AL355075.2	0.555796001	1.07E-44	postive
TXK	AL355075.2	0.507166427	2.60E-36	postive
NFAT5	AL355075.2	0.868141179	2.75E-164	postive
NFATC3	AL355075.2	0.552461252	4.44E-44	postive
PIK3R1	AL355075.2	0.541061898	5.18E-42	postive
PIK3CA	AL355075.2	0.54185691	3.74E-42	postive
AKT3	AL355075.2	0.520433454	1.82E-38	postive
IGHD6-6	AL355075.2	0.502272467	1.54E-35	postive
LTB4R2	AL355075.2	0.506690838	3.10E-36	postive
GNRH1	AL355075.2	0.775938941	9.85E-109	postive
IL6ST	AL355075.2	0.560702309	1.27E-45	postive
RABEP1	AL355075.2	0.553704614	2.61E-44	postive
ACVR2A	AL355075.2	0.563211644	4.21E-46	postive
ANGPTL1	AL355075.2	0.623430185	6.11E-59	postive
BMPR2	AL355075.2	0.569088912	3.06E-47	postive
CRLF3	AL355075.2	0.640717024	3.64E-63	postive
NR2C1	AL355075.2	0.60997559	7.85E-56	postive
NR2C2	AL355075.2	0.700206378	4.94E-80	postive
RORA	AL355075.2	0.787378419	4.62E-114	postive
SOS1	AL355075.2	0.593163185	3.77E-52	postive
SOS2	AL355075.2	0.542953504	2.38E-42	postive
BRAF	AL355075.2	0.746473399	2.36E-96	postive
CBL	AL355075.2	0.575367266	1.76E-48	postive
CBLB	AL355075.2	0.615160997	5.18E-57	postive
PDK1	AL355075.2	0.514046135	2.04E-37	postive
TRAJ1	AL355075.2	0.725636965	1.37E-88	postive
TRAJ2	AL355075.2	0.774171545	6.14E-108	postive
TRAJ3	AL355075.2	0.768748381	1.53E-105	postive
TRAJ5	AL355075.2	0.711361617	1.14E-83	postive
TRAJ6	AL355075.2	0.770969035	1.62E-106	postive
TRAJ8	AL355075.2	0.733948772	1.34E-91	postive
TRAJ10	AL355075.2	0.746000166	3.61E-96	postive
TRAJ12	AL355075.2	0.625759933	1.71E-59	postive
TRAJ13	AL355075.2	0.757389989	9.86E-101	postive
TRAJ14	AL355075.2	0.717916117	6.84E-86	postive
TRAJ16	AL355075.2	0.746235504	2.92E-96	postive
TRAJ17	AL355075.2	0.67114232	2.67E-71	postive
TRAJ18	AL355075.2	0.681337335	3.01E-74	postive
TRAJ21	AL355075.2	0.758580224	3.18E-101	postive
TRAJ31	AL355075.2	0.753942411	2.52E-99	postive
TRAJ37	AL355075.2	0.799985946	2.49E-120	postive
TRAJ38	AL355075.2	0.78806297	2.17E-114	postive
TRAJ39	AL355075.2	0.775383353	1.75E-108	postive

CREB1	AC080162.1	0.794335947	1.82E-117	postive
RFXAP	AC080162.1	0.503008225	1.18E-35	postive
UBR1	AC080162.1	0.637127542	2.89E-62	postive
ZC3HAV1	AC080162.1	0.57779677	5.72E-49	postive
ZC3HAV1L	AC080162.1	0.542267161	3.16E-42	postive
IL15	AC080162.1	0.641378166	2.48E-63	postive
CYLD	AC080162.1	0.602300337	4.01E-54	postive
EIF2AK2	AC080162.1	0.572731515	5.87E-48	postive
MAPK8	AC080162.1	0.568412431	4.15E-47	postive
TLR1	AC080162.1	0.539647352	9.24E-42	postive
NFKBIZ	AC080162.1	0.530741432	3.30E-40	postive
LMBR1	AC080162.1	0.607626712	2.65E-55	postive
LIMS1	AC080162.1	0.551928415	5.57E-44	postive
IREB2	AC080162.1	0.619844885	4.26E-58	postive
DDX17	AC080162.1	0.588580906	3.49E-51	postive
PIK3CG	AC080162.1	0.577887206	5.48E-49	postive
JAK2	AC080162.1	0.689770048	8.88E-77	postive
TXK	AC080162.1	0.597568118	4.30E-53	postive
NFAT5	AC080162.1	0.801674311	3.34E-121	postive
MALT1	AC080162.1	0.563937633	3.05E-46	postive
PIK3R1	AC080162.1	0.653767919	1.54E-66	postive
PIK3CA	AC080162.1	0.731451394	1.11E-90	postive
PIK3CB	AC080162.1	0.561469383	9.06E-46	postive
AKT3	AC080162.1	0.540490327	6.55E-42	postive
RASGRP3	AC080162.1	0.56465302	2.22E-46	postive
PLXNC1	AC080162.1	0.523531293	5.54E-39	postive
GNRH1	AC080162.1	0.70562832	8.85E-82	postive
IL6ST	AC080162.1	0.625181129	2.34E-59	postive
RABEP1	AC080162.1	0.561001959	1.11E-45	postive
ACVR2A	AC080162.1	0.60905706	1.26E-55	postive
ANGPTL1	AC080162.1	0.834485971	4.60E-140	postive
BMPR1A	AC080162.1	0.525122547	2.99E-39	postive
BMPR2	AC080162.1	0.727980929	2.00E-89	postive
CRLF3	AC080162.1	0.660717723	2.09E-68	postive
IL18R1	AC080162.1	0.505752711	4.36E-36	postive
NR2C2	AC080162.1	0.691752033	2.19E-77	postive
RORA	AC080162.1	0.824783539	4.44E-134	postive
SOS1	AC080162.1	0.705152414	1.26E-81	postive
SOS2	AC080162.1	0.600882113	8.19E-54	postive
BRAF	AC080162.1	0.812820144	3.44E-127	postive
ITK	AC080162.1	0.552249562	4.86E-44	postive
CBL	AC080162.1	0.638135195	1.62E-62	postive
CBLB	AC080162.1	0.801145034	6.28E-121	postive
RASGRP1	AC080162.1	0.519627161	2.48E-38	postive
PDK1	AC080162.1	0.645402831	2.34E-64	postive
TRAJ1	AC080162.1	0.700615621	3.66E-80	postive
TRAJ2	AC080162.1	0.718040619	6.20E-86	postive
TRAJ3	AC080162.1	0.76735437	6.15E-105	postive
TRAJ5	AC080162.1	0.786603034	1.09E-113	postive
TRAJ6	AC080162.1	0.767806786	3.92E-105	postive
TRAJ8	AC080162.1	0.720872254	6.51E-87	postive
TRAJ10	AC080162.1	0.760846311	3.62E-102	postive
TRAJ12	AC080162.1	0.688601894	2.01E-76	postive
TRAJ13	AC080162.1	0.755408382	6.40E-100	postive
TRAJ14	AC080162.1	0.757080801	1.32E-100	postive
TRAJ16	AC080162.1	0.764988984	6.40E-104	postive
TRAJ17	AC080162.1	0.727160545	3.93E-89	postive
TRAJ18	AC080162.1	0.722170076	2.29E-87	postive
TRAJ21	AC080162.1	0.794719848	1.17E-117	postive
TRAJ31	AC080162.1	0.808828657	5.32E-125	postive
TRAJ37	AC080162.1	0.761858588	1.36E-102	postive
TRAJ38	AC080162.1	0.859492942	1.82E-157	postive
TRAJ39	AC080162.1	0.798863259	9.40E-120	postive
LMBR1L	AC104564.3	0.514751478	1.57E-37	postive
TYK2	AC104564.3	0.542846418	2.49E-42	postive

IRF9	AC104564.3	0.621147191	2.11E-58	postive
TPT1	ZFAS1	0.580440658	1.67E-49	postive
GAL	ZFAS1	0.568375641	4.22E-47	postive
FABP7	AC063948.1	0.777564797	1.80E-109	postive
HCK	AC063948.1	0.677112652	5.18E-73	postive
SEMA3D	AC063948.1	0.536315819	3.57E-41	postive
CREB1	AC067852.3	0.722411301	1.89E-87	postive
RFXAP	AC067852.3	0.544321037	1.35E-42	postive
UBR1	AC067852.3	0.608144768	2.03E-55	postive
ZC3HAV1	AC067852.3	0.557980507	4.15E-45	postive
ZC3HAV1L	AC067852.3	0.561430471	9.21E-46	postive
IL15	AC067852.3	0.50435838	7.24E-36	postive
CYLD	AC067852.3	0.562929097	4.77E-46	postive
EIF2AK2	AC067852.3	0.520791739	1.59E-38	postive
MAPK8	AC067852.3	0.535735161	4.51E-41	postive
NFKBIZ	AC067852.3	0.571223659	1.17E-47	postive
LMBR1	AC067852.3	0.52084756	1.55E-38	postive
IREB2	AC067852.3	0.558710339	3.02E-45	postive
DDX17	AC067852.3	0.676836449	6.23E-73	postive
JAK2	AC067852.3	0.604182134	1.54E-54	postive
TXK	AC067852.3	0.570481171	1.63E-47	postive
NFAT5	AC067852.3	0.775230967	2.05E-108	postive
MALT1	AC067852.3	0.512776746	3.28E-37	postive
PIK3R1	AC067852.3	0.564653914	2.22E-46	postive
PIK3CA	AC067852.3	0.614911577	5.91E-57	postive
RASGRP3	AC067852.3	0.511608047	5.06E-37	postive
LTB4R2	AC067852.3	0.60242903	3.76E-54	postive
GNRH1	AC067852.3	0.782958214	5.78E-112	postive
IL6ST	AC067852.3	0.560835011	1.20E-45	postive
RABEP1	AC067852.3	0.567330259	6.74E-47	postive
ACVR2A	AC067852.3	0.560388221	1.45E-45	postive
ANGPTL1	AC067852.3	0.690312099	6.07E-77	postive
BMPR2	AC067852.3	0.637055167	3.01E-62	postive
CRLF3	AC067852.3	0.632862275	3.26E-61	postive
NR2C1	AC067852.3	0.56013624	1.62E-45	postive
NR2C2	AC067852.3	0.790995626	8.14E-116	postive
RORA	AC067852.3	0.747779215	7.26E-97	postive
SOS1	AC067852.3	0.65149845	6.10E-66	postive
SOS2	AC067852.3	0.553321082	3.08E-44	postive
BRAF	AC067852.3	0.781472524	2.86E-111	postive
TEC	AC067852.3	0.553008932	3.52E-44	postive
CBL	AC067852.3	0.625376393	2.11E-59	postive
CBLB	AC067852.3	0.715954766	3.21E-85	postive
RASGRP1	AC067852.3	0.504565117	6.72E-36	postive
PDK1	AC067852.3	0.541424818	4.47E-42	postive
TRAJ1	AC067852.3	0.662577895	6.50E-69	postive
TRAJ2	AC067852.3	0.650334208	1.23E-65	postive
TRAJ3	AC067852.3	0.700918179	2.93E-80	postive
TRAJ5	AC067852.3	0.67748385	4.05E-73	postive
TRAJ6	AC067852.3	0.694168487	3.93E-78	postive
TRAJ8	AC067852.3	0.62067482	2.72E-58	postive
TRAJ10	AC067852.3	0.672162604	1.37E-71	postive
TRAJ12	AC067852.3	0.61221378	2.44E-56	postive
TRAJ13	AC067852.3	0.639651703	6.75E-63	postive
TRAJ14	AC067852.3	0.649115293	2.56E-65	postive
TRAJ16	AC067852.3	0.673407737	6.05E-72	postive
TRAJ17	AC067852.3	0.614300664	8.16E-57	postive
TRAJ18	AC067852.3	0.637506299	2.32E-62	postive
TRAJ21	AC067852.3	0.675286206	1.75E-72	postive
TRAJ31	AC067852.3	0.687891154	3.31E-76	postive
TRAJ37	AC067852.3	0.675652581	1.37E-72	postive
TRAJ38	AC067852.3	0.745833915	4.19E-96	postive
TRAJ39	AC067852.3	0.697121807	4.68E-79	postive
CREB1	DLEU1	0.733608418	1.79E-91	postive
RFXAP	DLEU1	0.516719477	7.47E-38	postive

UBR1	DLEU1	0.627983383	5.00E-60	postive
IL15	DLEU1	0.526947739	1.47E-39	postive
CYLD	DLEU1	0.592180195	6.10E-52	postive
MAPK8	DLEU1	0.520251992	1.95E-38	postive
LMBR1	DLEU1	0.508954536	1.35E-36	postive
IREB2	DLEU1	0.570242988	1.82E-47	postive
DDX17	DLEU1	0.535308023	5.35E-41	postive
JAK2	DLEU1	0.558319442	3.58E-45	postive
NFAT5	DLEU1	0.908431199	4.85E-204	postive
NFATC3	DLEU1	0.612194784	2.47E-56	postive
PIK3R1	DLEU1	0.531164276	2.80E-40	postive
PIK3CA	DLEU1	0.601117473	7.28E-54	postive
AKT3	DLEU1	0.523295359	6.07E-39	postive
GNRH1	DLEU1	0.666891176	4.18E-70	postive
IL6ST	DLEU1	0.552265206	4.83E-44	postive
RABEP1	DLEU1	0.527510525	1.18E-39	postive
ACVR2A	DLEU1	0.630772201	1.06E-60	postive
ANGPTL1	DLEU1	0.646824843	1.01E-64	postive
BMPR1A	DLEU1	0.569191231	2.92E-47	postive
BMPR2	DLEU1	0.627539173	6.39E-60	postive
CRLF3	DLEU1	0.644029796	5.25E-64	postive
NR2C2	DLEU1	0.603471971	2.21E-54	postive
RORA	DLEU1	0.766658642	1.23E-104	postive
SOS1	DLEU1	0.615572713	4.17E-57	postive
SOS2	DLEU1	0.53902483	1.19E-41	postive
BRAF	DLEU1	0.711865292	7.71E-84	postive
CBL	DLEU1	0.520959273	1.49E-38	postive
CBLB	DLEU1	0.531985959	2.02E-40	postive
PDK1	DLEU1	0.506803987	2.97E-36	postive
TRAJ1	DLEU1	0.85089982	3.96E-151	postive
TRAJ2	DLEU1	0.83582518	6.40E-141	postive
TRAJ3	DLEU1	0.888410318	2.30E-182	postive
TRAJ5	DLEU1	0.831312898	4.59E-138	postive
TRAJ6	DLEU1	0.929458255	5.62E-233	postive
TRAJ8	DLEU1	0.917209424	3.54E-215	postive
TRAJ10	DLEU1	0.918436469	7.84E-217	postive
TRAJ12	DLEU1	0.822928747	5.61E-133	postive
TRAJ13	DLEU1	0.914482608	1.37E-211	postive
TRAJ14	DLEU1	0.894647403	1.21E-188	postive
TRAJ16	DLEU1	0.907322309	1.03E-202	postive
TRAJ17	DLEU1	0.878108479	8.98E-173	postive
TRAJ18	DLEU1	0.833473983	2.02E-139	postive
TRAJ21	DLEU1	0.919108539	9.48E-218	postive
TRAJ31	DLEU1	0.909906661	7.84E-206	postive
TRAJ37	DLEU1	0.939513537	3.53E-250	postive
TRAJ38	DLEU1	0.921255092	9.83E-221	postive
TRAJ39	DLEU1	0.925450239	8.07E-227	postive
CIITA	HCG27	0.508952515	1.35E-36	postive
DDX17	HCG27	0.575572772	1.60E-48	postive
IRF9	HCG27	0.579297237	2.84E-49	postive
LTB4R2	HCG27	0.591741091	7.55E-52	postive
GNRH1	HCG27	0.650306582	1.25E-65	postive
NR2C2	HCG27	0.559088477	2.56E-45	postive
TYK2	AL021707.3	0.549558918	1.52E-43	postive
DDX17	AL021707.3	0.578035095	5.12E-49	postive
IRF9	AL021707.3	0.545905485	7.02E-43	postive
CREB1	STARD4-AS1	0.678889487	1.58E-73	postive
RFXAP	STARD4-AS1	0.531807196	2.17E-40	postive
UBR1	STARD4-AS1	0.51620856	9.06E-38	postive
CYLD	STARD4-AS1	0.548143592	2.75E-43	postive
IREB2	STARD4-AS1	0.508990833	1.33E-36	postive
DDX17	STARD4-AS1	0.593182261	3.74E-52	postive
JAK2	STARD4-AS1	0.521270264	1.32E-38	postive
NFAT5	STARD4-AS1	0.766023782	2.30E-104	postive
NFATC3	STARD4-AS1	0.562020212	7.11E-46	postive

PIK3R1	STARD4-AS1	0.557553091	4.99E-45	postive
PIK3CA	STARD4-AS1	0.570681113	1.49E-47	postive
GNRH1	STARD4-AS1	0.661130779	1.62E-68	postive
ACVR2A	STARD4-AS1	0.581646029	9.47E-50	postive
ANGPTL1	STARD4-AS1	0.565578172	1.47E-46	postive
BMPR1A	STARD4-AS1	0.515579862	1.15E-37	postive
BMPR2	STARD4-AS1	0.579408106	2.70E-49	postive
CRLF3	STARD4-AS1	0.516474203	8.19E-38	postive
NR2C2	STARD4-AS1	0.618056125	1.11E-57	postive
RORA	STARD4-AS1	0.724417821	3.71E-88	postive
SOS1	STARD4-AS1	0.650042892	1.47E-65	postive
BRAF	STARD4-AS1	0.596856228	6.12E-53	postive
CBLB	STARD4-AS1	0.582719495	5.71E-50	postive
TRAJ1	STARD4-AS1	0.650626426	1.03E-65	postive
TRAJ2	STARD4-AS1	0.648349569	4.05E-65	postive
TRAJ3	STARD4-AS1	0.66916597	9.64E-71	postive
TRAJ5	STARD4-AS1	0.643336492	7.89E-64	postive
TRAJ6	STARD4-AS1	0.695011285	2.15E-78	postive
TRAJ8	STARD4-AS1	0.629915278	1.71E-60	postive
TRAJ10	STARD4-AS1	0.685703684	1.51E-75	postive
TRAJ12	STARD4-AS1	0.631297438	7.87E-61	postive
TRAJ13	STARD4-AS1	0.653994499	1.34E-66	postive
TRAJ14	STARD4-AS1	0.643201599	8.53E-64	postive
TRAJ16	STARD4-AS1	0.681451368	2.79E-74	postive
TRAJ17	STARD4-AS1	0.624919818	2.70E-59	postive
TRAJ18	STARD4-AS1	0.657205098	1.86E-67	postive
TRAJ21	STARD4-AS1	0.673824888	4.59E-72	postive
TRAJ31	STARD4-AS1	0.688979125	1.55E-76	postive
TRAJ37	STARD4-AS1	0.712096714	6.45E-84	postive
TRAJ38	STARD4-AS1	0.723959432	5.39E-88	postive
TRAJ39	STARD4-AS1	0.688695116	1.89E-76	postive
CIITA	AL731567.1	0.601471391	6.09E-54	postive
IFI30	AL731567.1	0.515837642	1.04E-37	postive
NOD2	AL731567.1	0.547185985	4.11E-43	postive
IL15	AL731567.1	0.513380944	2.62E-37	postive
NFKBIZ	AL731567.1	0.554727953	1.69E-44	postive
SLC11A1	AL731567.1	0.579551714	2.53E-49	postive
FGR	AL731567.1	0.506509551	3.31E-36	postive
PIK3R5	AL731567.1	0.537445623	2.26E-41	postive
GNRH1	AL731567.1	0.549957553	1.28E-43	postive
SH3BP2	AL731567.1	0.582473236	6.41E-50	postive
CREB1	AL391834.1	0.722569308	1.66E-87	postive
RFXAP	AL391834.1	0.518834084	3.35E-38	postive
UBR1	AL391834.1	0.574719576	2.37E-48	postive
ZC3HAV1	AL391834.1	0.505007191	5.72E-36	postive
IL15	AL391834.1	0.52151274	1.20E-38	postive
CYLD	AL391834.1	0.57447541	2.65E-48	postive
EIF2AK2	AL391834.1	0.507251398	2.52E-36	postive
MAPK8	AL391834.1	0.506799933	2.98E-36	postive
NFKBIZ	AL391834.1	0.544175401	1.44E-42	postive
LMBR1	AL391834.1	0.508814409	1.42E-36	postive
IREB2	AL391834.1	0.532536013	1.62E-40	postive
DDX17	AL391834.1	0.648636239	3.41E-65	postive
PIK3CG	AL391834.1	0.507138735	2.63E-36	postive
JAK2	AL391834.1	0.658898099	6.52E-68	postive
TXK	AL391834.1	0.574631772	2.46E-48	postive
NFAT5	AL391834.1	0.772117169	5.05E-107	postive
MALT1	AL391834.1	0.519905016	2.23E-38	postive
PIK3R1	AL391834.1	0.611326891	3.89E-56	postive
PIK3CA	AL391834.1	0.638990683	9.89E-63	postive
RASGRP3	AL391834.1	0.525178106	2.93E-39	postive
GNRH1	AL391834.1	0.747952509	6.21E-97	postive
IL6ST	AL391834.1	0.632649068	3.68E-61	postive
RABEP1	AL391834.1	0.559465902	2.17E-45	postive
ACVR2A	AL391834.1	0.558973009	2.70E-45	postive

ANGPTL1	AL391834.1	0.708277152	1.20E-82	postive
BMPR2	AL391834.1	0.659410652	4.74E-68	postive
CRLF3	AL391834.1	0.634605223	1.22E-61	postive
NR2C1	AL391834.1	0.543305033	2.06E-42	postive
NR2C2	AL391834.1	0.700323202	4.54E-80	postive
RORA	AL391834.1	0.81190387	1.11E-126	postive
SOS1	AL391834.1	0.609419595	1.05E-55	postive
SOS2	AL391834.1	0.539097703	1.16E-41	postive
BRAF	AL391834.1	0.725201327	1.96E-88	postive
ITK	AL391834.1	0.523051724	6.66E-39	postive
TEC	AL391834.1	0.507510151	2.30E-36	postive
CBL	AL391834.1	0.58094869	1.31E-49	postive
CBLB	AL391834.1	0.699052385	1.15E-79	postive
RASGRP1	AL391834.1	0.5198023	2.32E-38	postive
PDK1	AL391834.1	0.568487894	4.01E-47	postive
TRAJ1	AL391834.1	0.639517	7.29E-63	postive
TRAJ2	AL391834.1	0.62840063	3.97E-60	postive
TRAJ3	AL391834.1	0.638899018	1.04E-62	postive
TRAJ5	AL391834.1	0.733390429	2.16E-91	postive
TRAJ6	AL391834.1	0.670463471	4.15E-71	postive
TRAJ8	AL391834.1	0.602083954	4.47E-54	postive
TRAJ10	AL391834.1	0.670399156	4.33E-71	postive
TRAJ12	AL391834.1	0.583826893	3.38E-50	postive
TRAJ13	AL391834.1	0.666295959	6.12E-70	postive
TRAJ14	AL391834.1	0.636605241	3.89E-62	postive
TRAJ16	AL391834.1	0.666401248	5.72E-70	postive
TRAJ17	AL391834.1	0.610100614	7.36E-56	postive
TRAJ18	AL391834.1	0.639206857	8.73E-63	postive
TRAJ21	AL391834.1	0.688663158	1.93E-76	postive
TRAJ31	AL391834.1	0.7269394	4.72E-89	postive
TRAJ37	AL391834.1	0.702518614	8.99E-81	postive
TRAJ38	AL391834.1	0.748349464	4.33E-97	postive
TRAJ39	AL391834.1	0.71091085	1.61E-83	postive
CREB1	AC093495.1	0.544257402	1.39E-42	postive
UBR1	AC093495.1	0.514606573	1.65E-37	postive
CYLD	AC093495.1	0.532200711	1.85E-40	postive
DDX17	AC093495.1	0.726886084	4.93E-89	postive
TXK	AC093495.1	0.541275697	4.75E-42	postive
NFAT5	AC093495.1	0.650387292	1.19E-65	postive
PIK3R1	AC093495.1	0.549569472	1.51E-43	postive
LTB4R2	AC093495.1	0.578188152	4.77E-49	postive
GNRH1	AC093495.1	0.758314333	4.10E-101	postive
IL6ST	AC093495.1	0.513526297	2.48E-37	postive
ACVR2A	AC093495.1	0.531639352	2.32E-40	postive
ANGPTL1	AC093495.1	0.543181463	2.17E-42	postive
BMPR2	AC093495.1	0.511596859	5.09E-37	postive
NR2C1	AC093495.1	0.531424305	2.52E-40	postive
NR2C2	AC093495.1	0.796077755	2.44E-118	postive
NR3C2	AC093495.1	0.510007088	9.16E-37	postive
RORA	AC093495.1	0.660655374	2.18E-68	postive
BRAF	AC093495.1	0.600476491	1.00E-53	postive
CBLB	AC093495.1	0.539022725	1.19E-41	postive
TRAJ1	AC093495.1	0.538733513	1.34E-41	postive
TRAJ3	AC093495.1	0.560988211	1.12E-45	postive
TRAJ5	AC093495.1	0.537388558	2.31E-41	postive
TRAJ6	AC093495.1	0.525764635	2.33E-39	postive
TRAJ10	AC093495.1	0.506782596	3.00E-36	postive
TRAJ16	AC093495.1	0.514571783	1.68E-37	postive
TRAJ18	AC093495.1	0.50778218	2.08E-36	postive
TRAJ21	AC093495.1	0.51778845	4.98E-38	postive
TRAJ31	AC093495.1	0.523076615	6.60E-39	postive
TRAJ37	AC093495.1	0.530928042	3.07E-40	postive
TRAJ38	AC093495.1	0.55344092	2.93E-44	postive
TRAJ39	AC093495.1	0.553103813	3.38E-44	postive
CREB1	DLEU2	0.779401354	2.60E-110	postive

RFXAP	DLEU2	0.561663306	8.32E-46	postive
PSMD6	DLEU2	0.543391019	1.99E-42	postive
UBR1	DLEU2	0.611637374	3.30E-56	postive
ZC3HAV1	DLEU2	0.575788341	1.45E-48	postive
ZC3HAV1L	DLEU2	0.579232135	2.93E-49	postive
IL15	DLEU2	0.596382623	7.73E-53	postive
CYLD	DLEU2	0.550957485	8.41E-44	postive
EIF2AK2	DLEU2	0.582496855	6.34E-50	postive
MAPK8	DLEU2	0.56285788	4.92E-46	postive
LMBR1	DLEU2	0.597903335	3.64E-53	postive
EED	DLEU2	0.502945937	1.21E-35	postive
IREB2	DLEU2	0.624617315	3.19E-59	postive
DDX17	DLEU2	0.540258009	7.20E-42	postive
JAK2	DLEU2	0.602495917	3.63E-54	postive
TXK	DLEU2	0.527709548	1.09E-39	postive
NFAT5	DLEU2	0.798527683	1.39E-119	postive
MALT1	DLEU2	0.52153562	1.19E-38	postive
PIK3R1	DLEU2	0.552837504	3.78E-44	postive
PIK3CA	DLEU2	0.67018779	4.97E-71	postive
PIK3CB	DLEU2	0.536719715	3.03E-41	postive
AKT3	DLEU2	0.563087178	4.44E-46	postive
PLXNC1	DLEU2	0.516633889	7.71E-38	postive
GNRH1	DLEU2	0.691898098	1.98E-77	postive
IL6ST	DLEU2	0.541526997	4.28E-42	postive
RABEP1	DLEU2	0.553746253	2.57E-44	postive
ACVR2A	DLEU2	0.629053746	2.76E-60	postive
ANGPTL1	DLEU2	0.710859174	1.67E-83	postive
BMPR1A	DLEU2	0.570377793	1.71E-47	postive
BMPR2	DLEU2	0.673573058	5.42E-72	postive
CRLF3	DLEU2	0.694443761	3.22E-78	postive
NR2C2	DLEU2	0.677904812	3.05E-73	postive
RORA	DLEU2	0.732675727	3.95E-91	postive
SOS1	DLEU2	0.682436762	1.42E-74	postive
SOS2	DLEU2	0.577140005	7.75E-49	postive
BRAF	DLEU2	0.80458962	9.88E-123	postive
CBL	DLEU2	0.623425978	6.12E-59	postive
CBLB	DLEU2	0.649119257	2.56E-65	postive
PDK1	DLEU2	0.601641929	5.59E-54	postive
TRAJ1	DLEU2	0.78901199	7.53E-115	postive
TRAJ2	DLEU2	0.766105615	2.12E-104	postive
TRAJ3	DLEU2	0.822738465	7.27E-133	postive
TRAJ5	DLEU2	0.811189726	2.74E-126	postive
TRAJ6	DLEU2	0.835726698	7.40E-141	postive
TRAJ8	DLEU2	0.833666261	1.52E-139	postive
TRAJ10	DLEU2	0.810311084	8.29E-126	postive
TRAJ12	DLEU2	0.76613399	2.07E-104	postive
TRAJ13	DLEU2	0.821054008	7.08E-132	postive
TRAJ14	DLEU2	0.825236249	2.38E-134	postive
TRAJ16	DLEU2	0.817957812	4.37E-130	postive
TRAJ17	DLEU2	0.799501721	4.42E-120	postive
TRAJ18	DLEU2	0.737647491	5.66E-93	postive
TRAJ21	DLEU2	0.837688152	4.00E-142	postive
TRAJ31	DLEU2	0.843059204	1.10E-145	postive
TRAJ37	DLEU2	0.816745013	2.15E-129	postive
TRAJ38	DLEU2	0.869559789	1.88E-165	postive
TRAJ39	DLEU2	0.8471276	1.80E-148	postive
FABP7	AL139393.3	0.773170958	1.72E-107	postive
HCK	AL139393.3	0.56285056	4.93E-46	postive
SEMA3D	AL139393.3	0.570412974	1.68E-47	postive
CREB1	AC073896.3	0.532365505	1.73E-40	postive
IREB2	AC073896.3	0.525782767	2.31E-39	postive
DDX17	AC073896.3	0.555917553	1.01E-44	postive
NFAT5	AC073896.3	0.53498392	6.09E-41	postive
GNRH1	AC073896.3	0.61047189	6.07E-56	postive
NR2C1	AC073896.3	0.504757222	6.26E-36	postive

NR2C2	AC073896.3	0.617215122	1.74E-57	postive
RORA	AC073896.3	0.525160658	2.95E-39	postive
SOS1	AC073896.3	0.505762035	4.35E-36	postive
BRAF	AC073896.3	0.528358549	8.45E-40	postive
TRAJ3	AC073896.3	0.50056706	2.84E-35	postive
CREB1	AC009120.3	0.689493483	1.08E-76	postive
RFXAP	AC009120.3	0.521969963	1.01E-38	postive
UBR1	AC009120.3	0.629549208	2.09E-60	postive
CYLD	AC009120.3	0.689922858	7.98E-77	postive
IREB2	AC009120.3	0.504595516	6.64E-36	postive
DDX17	AC009120.3	0.705210227	1.21E-81	postive
JAK2	AC009120.3	0.594342382	2.12E-52	postive
TXK	AC009120.3	0.557501937	5.10E-45	postive
NFAT5	AC009120.3	0.907210464	1.40E-202	postive
NFATC3	AC009120.3	0.708898316	7.48E-83	postive
PIK3R1	AC009120.3	0.579274748	2.87E-49	postive
PIK3CA	AC009120.3	0.548156926	2.74E-43	postive
ROBO2	AC009120.3	0.54151122	4.31E-42	postive
GNRH1	AC009120.3	0.771000342	1.57E-106	postive
IL6ST	AC009120.3	0.570093435	1.95E-47	postive
RABEP1	AC009120.3	0.539083159	1.16E-41	postive
ACVR2A	AC009120.3	0.580821966	1.39E-49	postive
ANGPTL1	AC009120.3	0.56719838	7.15E-47	postive
BMPR2	AC009120.3	0.598801702	2.32E-53	postive
CRLF3	AC009120.3	0.5337995	9.79E-41	postive
NR1D2	AC009120.3	0.511783549	4.74E-37	postive
NR2C1	AC009120.3	0.534360209	7.83E-41	postive
NR2C2	AC009120.3	0.740128964	6.55E-94	postive
NR3C2	AC009120.3	0.516714778	7.48E-38	postive
RORA	AC009120.3	0.811763342	1.32E-126	postive
SOS1	AC009120.3	0.573064947	5.05E-48	postive
SOS2	AC009120.3	0.503607161	9.50E-36	postive
BRAF	AC009120.3	0.663069372	4.76E-69	postive
CBL	AC009120.3	0.532631913	1.56E-40	postive
CBLB	AC009120.3	0.58900327	2.85E-51	postive
TRAJ1	AC009120.3	0.749459689	1.58E-97	postive
TRAJ2	AC009120.3	0.710929333	1.58E-83	postive
TRAJ3	AC009120.3	0.763808329	2.04E-103	postive
TRAJ5	AC009120.3	0.723417139	8.37E-88	postive
TRAJ6	AC009120.3	0.790170996	2.06E-115	postive
TRAJ8	AC009120.3	0.678230415	2.45E-73	postive
TRAJ10	AC009120.3	0.777078469	3.00E-109	postive
TRAJ12	AC009120.3	0.740292274	5.68E-94	postive
TRAJ13	AC009120.3	0.725632057	1.38E-88	postive
TRAJ14	AC009120.3	0.71361448	1.99E-84	postive
TRAJ16	AC009120.3	0.748836087	2.78E-97	postive
TRAJ17	AC009120.3	0.688835033	1.71E-76	postive
TRAJ18	AC009120.3	0.782426058	1.03E-111	postive
TRAJ21	AC009120.3	0.7581374	4.85E-101	postive
TRAJ31	AC009120.3	0.761925601	1.27E-102	postive
TRAJ37	AC009120.3	0.810871968	4.09E-126	postive
TRAJ38	AC009120.3	0.791103252	7.21E-116	postive
TRAJ39	AC009120.3	0.771903098	6.29E-107	postive
DEFB1	EPB41L4A-AS1	0.500663511	2.74E-35	postive
CREB1	AC005104.1	0.663151412	4.52E-69	postive
UBR1	AC005104.1	0.538528753	1.46E-41	postive
ZC3HAV1L	AC005104.1	0.558361215	3.52E-45	postive
MAPK8	AC005104.1	0.523376989	5.88E-39	postive
NFKBIZ	AC005104.1	0.593738263	2.85E-52	postive
IREB2	AC005104.1	0.531518272	2.43E-40	postive
DDX17	AC005104.1	0.656652193	2.62E-67	postive
JAK2	AC005104.1	0.524581353	3.69E-39	postive
TXK	AC005104.1	0.519837109	2.29E-38	postive
NFAT5	AC005104.1	0.688807203	1.75E-76	postive
PIK3CA	AC005104.1	0.575561796	1.61E-48	postive

LTB4R2	AC005104.1	0.573881435	3.47E-48	postive
GNRH1	AC005104.1	0.786846801	8.31E-114	postive
ACVR2A	AC005104.1	0.535630456	4.70E-41	postive
ANGPTL1	AC005104.1	0.684046976	4.73E-75	postive
BMPR2	AC005104.1	0.598483889	2.72E-53	postive
CRLF3	AC005104.1	0.592270938	5.83E-52	postive
NR2C1	AC005104.1	0.57698043	8.34E-49	postive
NR2C2	AC005104.1	0.704942261	1.48E-81	postive
RORA	AC005104.1	0.70649169	4.62E-82	postive
SOS1	AC005104.1	0.615475199	4.39E-57	postive
BRAF	AC005104.1	0.72544786	1.60E-88	postive
CBL	AC005104.1	0.572367739	6.93E-48	postive
CBLB	AC005104.1	0.707792588	1.73E-82	postive
PDK1	AC005104.1	0.587841269	4.98E-51	postive
TRAJ1	AC005104.1	0.568179031	4.61E-47	postive
TRAJ2	AC005104.1	0.575367386	1.76E-48	postive
TRAJ3	AC005104.1	0.611166579	4.22E-56	postive
TRAJ5	AC005104.1	0.621585402	1.66E-58	postive
TRAJ6	AC005104.1	0.589183142	2.61E-51	postive
TRAJ8	AC005104.1	0.560757337	1.24E-45	postive
TRAJ10	AC005104.1	0.574968772	2.11E-48	postive
TRAJ13	AC005104.1	0.585323471	1.66E-50	postive
TRAJ14	AC005104.1	0.558354512	3.53E-45	postive
TRAJ16	AC005104.1	0.591755505	7.50E-52	postive
TRAJ17	AC005104.1	0.543300067	2.07E-42	postive
TRAJ21	AC005104.1	0.601937328	4.81E-54	postive
TRAJ31	AC005104.1	0.619189099	6.05E-58	postive
TRAJ37	AC005104.1	0.597257186	5.01E-53	postive
TRAJ38	AC005104.1	0.661591249	1.21E-68	postive
TRAJ39	AC005104.1	0.626927318	8.96E-60	postive
CREB1	AC037198.2	0.651274056	6.99E-66	postive
UBR1	AC037198.2	0.539563453	9.56E-42	postive
TLR4	AC037198.2	0.503060894	1.16E-35	postive
IL15	AC037198.2	0.570475264	1.64E-47	postive
CYLD	AC037198.2	0.550710934	9.34E-44	postive
TLR1	AC037198.2	0.55662312	7.46E-45	postive
NFKBIZ	AC037198.2	0.549371612	1.64E-43	postive
LIMS1	AC037198.2	0.523298341	6.06E-39	postive
PIK3CG	AC037198.2	0.551818338	5.84E-44	postive
JAK2	AC037198.2	0.657917708	1.20E-67	postive
TXK	AC037198.2	0.507184329	2.59E-36	postive
NFAT5	AC037198.2	0.698609482	1.59E-79	postive
MALT1	AC037198.2	0.505488819	4.80E-36	postive
PIK3R1	AC037198.2	0.505442649	4.88E-36	postive
PIK3CA	AC037198.2	0.641097828	2.92E-63	postive
RASGRP3	AC037198.2	0.51992012	2.22E-38	postive
GNRH1	AC037198.2	0.595691115	1.09E-52	postive
IL7	AC037198.2	0.524289898	4.13E-39	postive
ANGPTL1	AC037198.2	0.666236458	6.35E-70	postive
BMPR2	AC037198.2	0.553348244	3.04E-44	postive
CRLF3	AC037198.2	0.565729989	1.38E-46	postive
IL18R1	AC037198.2	0.539400635	1.02E-41	postive
NR2C2	AC037198.2	0.592056915	6.48E-52	postive
RORA	AC037198.2	0.681289956	3.11E-74	postive
SOS1	AC037198.2	0.539426295	1.01E-41	postive
BRAF	AC037198.2	0.579937249	2.11E-49	postive
TEC	AC037198.2	0.506369788	3.48E-36	postive
CBL	AC037198.2	0.541501516	4.33E-42	postive
CBLB	AC037198.2	0.636683289	3.72E-62	postive
PDK1	AC037198.2	0.585172649	1.78E-50	postive
TRAJ1	AC037198.2	0.627679209	5.91E-60	postive
TRAJ2	AC037198.2	0.55593015	1.01E-44	postive
TRAJ3	AC037198.2	0.646518132	1.21E-64	postive
TRAJ5	AC037198.2	0.659651958	4.07E-68	postive
TRAJ6	AC037198.2	0.672389414	1.18E-71	postive

TRAJ8	AC037198.2	0.559272474	2.37E-45	postive
TRAJ10	AC037198.2	0.711966061	7.13E-84	postive
TRAJ12	AC037198.2	0.548366701	2.51E-43	postive
TRAJ13	AC037198.2	0.597009168	5.67E-53	postive
TRAJ14	AC037198.2	0.607332783	3.08E-55	postive
TRAJ16	AC037198.2	0.698732349	1.45E-79	postive
TRAJ17	AC037198.2	0.651560919	5.88E-66	postive
TRAJ18	AC037198.2	0.658663488	7.54E-68	postive
TRAJ21	AC037198.2	0.65727502	1.78E-67	postive
TRAJ31	AC037198.2	0.652348786	3.65E-66	postive
TRAJ37	AC037198.2	0.621427204	1.81E-58	postive
TRAJ38	AC037198.2	0.712810577	3.72E-84	postive
TRAJ39	AC037198.2	0.686653556	7.83E-76	postive
CREB1	HCG18	0.791881656	2.99E-116	postive
RFXAP	HCG18	0.571952327	8.38E-48	postive
UBR1	HCG18	0.66249193	6.86E-69	postive
ZC3HAV1	HCG18	0.576089931	1.26E-48	postive
ZC3HAV1L	HCG18	0.565723627	1.38E-46	postive
IL15	HCG18	0.508593985	1.54E-36	postive
CYLD	HCG18	0.540922519	5.49E-42	postive
MAPK14	HCG18	0.501433701	2.08E-35	postive
EIF2AK2	HCG18	0.566371151	1.04E-46	postive
MAPK8	HCG18	0.625605864	1.86E-59	postive
LMBR1	HCG18	0.576066686	1.27E-48	postive
IREB2	HCG18	0.663853951	2.90E-69	postive
DDX17	HCG18	0.595990446	9.39E-53	postive
JAK2	HCG18	0.563979875	3.00E-46	postive
TXK	HCG18	0.541372538	4.56E-42	postive
NFAT5	HCG18	0.784819307	7.67E-113	postive
MALT1	HCG18	0.541105768	5.09E-42	postive
PIK3R1	HCG18	0.612229273	2.42E-56	postive
PIK3CA	HCG18	0.691123538	3.42E-77	postive
PIK3CB	HCG18	0.54551653	8.25E-43	postive
IGHD4-4	HCG18	0.514159046	1.96E-37	postive
GNRH1	HCG18	0.682624154	1.25E-74	postive
IL6ST	HCG18	0.615926585	3.45E-57	postive
RABEP1	HCG18	0.575884757	1.38E-48	postive
ACVR2A	HCG18	0.67486335	2.31E-72	postive
ACVR2B	HCG18	0.543035348	2.30E-42	postive
ANGPTL1	HCG18	0.70046023	4.10E-80	postive
BMPR1A	HCG18	0.608350652	1.82E-55	postive
BMPR2	HCG18	0.713570923	2.06E-84	postive
CRLF3	HCG18	0.604020284	1.68E-54	postive
NR1D2	HCG18	0.516187248	9.13E-38	postive
NR2C2	HCG18	0.725641509	1.37E-88	postive
RORA	HCG18	0.769492717	7.22E-106	postive
SOS1	HCG18	0.741566857	1.86E-94	postive
SOS2	HCG18	0.616948461	2.01E-57	postive
BRAF	HCG18	0.792210479	2.06E-116	postive
CBL	HCG18	0.571596861	9.85E-48	postive
CBLB	HCG18	0.656787015	2.41E-67	postive
PDK1	HCG18	0.561257514	9.94E-46	postive
TRAJ1	HCG18	0.706548106	4.43E-82	postive
TRAJ2	HCG18	0.648403409	3.92E-65	postive
TRAJ3	HCG18	0.743312231	3.98E-95	postive
TRAJ5	HCG18	0.769271385	9.03E-106	postive
TRAJ6	HCG18	0.750878584	4.30E-98	postive
TRAJ8	HCG18	0.753454307	3.97E-99	postive
TRAJ10	HCG18	0.733587204	1.83E-91	postive
TRAJ12	HCG18	0.70914588	6.19E-83	postive
TRAJ13	HCG18	0.745259194	7.02E-96	postive
TRAJ14	HCG18	0.735190042	4.67E-92	postive
TRAJ16	HCG18	0.751443827	2.56E-98	postive
TRAJ17	HCG18	0.747047447	1.41E-96	postive
TRAJ18	HCG18	0.681251703	3.19E-74	postive

TRAJ21	HCG18	0.768333951	2.31E-105	postive
TRAJ31	HCG18	0.797789816	3.31E-119	postive
TRAJ37	HCG18	0.764739604	8.17E-104	postive
TRAJ38	HCG18	0.806366186	1.12E-123	postive
TRAJ39	HCG18	0.79565271	3.99E-118	postive
CREB1	AC009318.2	0.64211053	1.62E-63	postive
UBR1	AC009318.2	0.51410191	2.00E-37	postive
ZC3HAV1	AC009318.2	0.533827382	9.69E-41	postive
CYLD	AC009318.2	0.536353095	3.51E-41	postive
EIF2AK2	AC009318.2	0.534523142	7.33E-41	postive
IREB2	AC009318.2	0.533085767	1.30E-40	postive
DDX17	AC009318.2	0.507948728	1.95E-36	postive
JAK2	AC009318.2	0.545390608	8.69E-43	postive
TXK	AC009318.2	0.502774972	1.28E-35	postive
NFAT5	AC009318.2	0.677719883	3.45E-73	postive
PIK3R1	AC009318.2	0.544220769	1.41E-42	postive
PIK3CA	AC009318.2	0.604093704	1.61E-54	postive
RASGRP3	AC009318.2	0.507735107	2.11E-36	postive
GNRH1	AC009318.2	0.597087965	5.45E-53	postive
IL6ST	AC009318.2	0.561057047	1.09E-45	postive
ACVR2A	AC009318.2	0.563548724	3.62E-46	postive
ANGPTL1	AC009318.2	0.637038415	3.04E-62	postive
BMPR2	AC009318.2	0.614776413	6.35E-57	postive
CRLF3	AC009318.2	0.526907522	1.49E-39	postive
NR2C2	AC009318.2	0.587968612	4.69E-51	postive
RORA	AC009318.2	0.721240223	4.84E-87	postive
SOS1	AC009318.2	0.601743419	5.31E-54	postive
BRAF	AC009318.2	0.686977907	6.25E-76	postive
CBLB	AC009318.2	0.583365816	4.21E-50	postive
TRAJ1	AC009318.2	0.65557674	5.08E-67	postive
TRAJ2	AC009318.2	0.585283104	1.69E-50	postive
TRAJ3	AC009318.2	0.658766079	7.08E-68	postive
TRAJ5	AC009318.2	0.756803331	1.72E-100	postive
TRAJ6	AC009318.2	0.666573465	5.12E-70	postive
TRAJ8	AC009318.2	0.697764988	2.94E-79	postive
TRAJ10	AC009318.2	0.649439663	2.11E-65	postive
TRAJ12	AC009318.2	0.612121946	2.56E-56	postive
TRAJ13	AC009318.2	0.709168683	6.09E-83	postive
TRAJ14	AC009318.2	0.681848467	2.13E-74	postive
TRAJ16	AC009318.2	0.689282772	1.25E-76	postive
TRAJ17	AC009318.2	0.691454605	2.71E-77	postive
TRAJ18	AC009318.2	0.616664833	2.33E-57	postive
TRAJ21	AC009318.2	0.723007986	1.17E-87	postive
TRAJ31	AC009318.2	0.750592822	5.59E-98	postive
TRAJ37	AC009318.2	0.703251884	5.22E-81	postive
TRAJ38	AC009318.2	0.722497759	1.76E-87	postive
TRAJ39	AC009318.2	0.752600237	8.78E-99	postive
TYK2	AC011498.6	0.62387026	4.80E-59	postive
LTB4R2	AC011498.6	0.544597204	1.21E-42	postive
JAK1	PITPNA-AS1	-0.503530341	9.77E-36	negative
IRF9	TMEM147-AS1	0.507003976	2.76E-36	postive
IRF9	LINC01176	0.526078425	2.06E-39	postive
CMTM6	FGD5-AS1	0.519291551	2.82E-38	postive
RAF1	FGD5-AS1	0.507428021	2.37E-36	postive
CREB1	AC019080.5	0.742523792	7.99E-95	postive
UBR1	AC019080.5	0.613916346	1.00E-56	postive
ZC3HAV1	AC019080.5	0.569958105	2.07E-47	postive
ZC3HAV1L	AC019080.5	0.540367153	6.89E-42	postive
IL15	AC019080.5	0.583266986	4.41E-50	postive
CYLD	AC019080.5	0.53227501	1.80E-40	postive
EIF2AK2	AC019080.5	0.589135286	2.67E-51	postive
MAPK8	AC019080.5	0.536019583	4.02E-41	postive
LMBR1	AC019080.5	0.593884891	2.65E-52	postive
IREB2	AC019080.5	0.607810386	2.41E-55	postive
DDX17	AC019080.5	0.567255401	6.97E-47	postive

JAK2	AC019080.5	0.5683955	4.18E-47	postive
TXK	AC019080.5	0.54266359	2.69E-42	postive
NFAT5	AC019080.5	0.766827352	1.04E-104	postive
PIK3R1	AC019080.5	0.595141656	1.43E-52	postive
PIK3CA	AC019080.5	0.639476679	7.47E-63	postive
PIK3CB	AC019080.5	0.521708595	1.12E-38	postive
AKT3	AC019080.5	0.522637827	7.82E-39	postive
RASGRP3	AC019080.5	0.503757719	9.00E-36	postive
IGHD4-4	AC019080.5	0.525752005	2.34E-39	postive
IGHD6-6	AC019080.5	0.528538402	7.88E-40	postive
PLXNC1	AC019080.5	0.500098341	3.35E-35	postive
GNRH1	AC019080.5	0.704828566	1.61E-81	postive
IL6ST	AC019080.5	0.588272399	4.05E-51	postive
RABEP1	AC019080.5	0.519588977	2.51E-38	postive
ACVR2A	AC019080.5	0.603799586	1.87E-54	postive
ANGPTL1	AC019080.5	0.713429224	2.30E-84	postive
BMPR1A	AC019080.5	0.531094851	2.87E-40	postive
BMPR2	AC019080.5	0.693118356	8.31E-78	postive
CRLF3	AC019080.5	0.652417071	3.50E-66	postive
NR2C2	AC019080.5	0.694526705	3.04E-78	postive
NR3C1	AC019080.5	0.503912092	8.51E-36	postive
RORA	AC019080.5	0.743959584	2.24E-95	postive
SOS1	AC019080.5	0.680109872	6.92E-74	postive
SOS2	AC019080.5	0.560546196	1.36E-45	postive
BRAF	AC019080.5	0.826916499	2.31E-135	postive
TEC	AC019080.5	0.505741155	4.38E-36	postive
CBL	AC019080.5	0.585802184	1.32E-50	postive
CBLB	AC019080.5	0.669848849	6.19E-71	postive
RASGRP1	AC019080.5	0.505167441	5.40E-36	postive
PDK1	AC019080.5	0.548537199	2.33E-43	postive
TRAJ1	AC019080.5	0.719457011	2.01E-86	postive
TRAJ2	AC019080.5	0.721103016	5.41E-87	postive
TRAJ3	AC019080.5	0.788316479	1.63E-114	postive
TRAJ5	AC019080.5	0.774219992	5.84E-108	postive
TRAJ6	AC019080.5	0.766922815	9.45E-105	postive
TRAJ8	AC019080.5	0.794506624	1.50E-117	postive
TRAJ10	AC019080.5	0.72275905	1.43E-87	postive
TRAJ12	AC019080.5	0.707413975	2.31E-82	postive
TRAJ13	AC019080.5	0.770217559	3.48E-106	postive
TRAJ14	AC019080.5	0.776388494	6.16E-109	postive
TRAJ16	AC019080.5	0.760000116	8.17E-102	postive
TRAJ17	AC019080.5	0.741382891	2.18E-94	postive
TRAJ18	AC019080.5	0.677899861	3.06E-73	postive
TRAJ21	AC019080.5	0.794938678	9.10E-118	postive
TRAJ31	AC019080.5	0.80247136	1.28E-121	postive
TRAJ37	AC019080.5	0.774207976	5.92E-108	postive
TRAJ38	AC019080.5	0.826927143	2.28E-135	postive
TRAJ39	AC019080.5	0.821585584	3.46E-132	postive
PTGFR	AC243967.2	0.60352618	2.15E-54	postive
BMPR1B	AL096865.1	0.519113516	3.01E-38	postive
IL15	GK-IT1	0.5248992	3.26E-39	postive
FPR2	GK-IT1	0.595081542	1.47E-52	postive
ANGPTL1	GK-IT1	0.553927005	2.38E-44	postive
IL18R1	GK-IT1	0.545251459	9.21E-43	postive
TRAJ10	GK-IT1	0.546474061	5.54E-43	postive
CREB1	AC011939.2	0.798638427	1.22E-119	postive
UBR1	AC011939.2	0.663242625	4.27E-69	postive
ZC3HAV1	AC011939.2	0.563367331	3.93E-46	postive
IL15	AC011939.2	0.568137922	4.69E-47	postive
CYLD	AC011939.2	0.603095193	2.68E-54	postive
EIF2AK2	AC011939.2	0.554880987	1.58E-44	postive
MAPK8	AC011939.2	0.592851818	4.39E-52	postive
LMBR1	AC011939.2	0.598894005	2.22E-53	postive
LIMS1	AC011939.2	0.528472893	8.08E-40	postive
IREB2	AC011939.2	0.630995965	9.32E-61	postive

DDX17	AC011939.2	0.598639419	2.52E-53	postive
PIK3CG	AC011939.2	0.521679645	1.13E-38	postive
JAK2	AC011939.2	0.628882518	3.03E-60	postive
TXK	AC011939.2	0.636037388	5.39E-62	postive
PPP3CB	AC011939.2	0.501867661	1.78E-35	postive
NFAT5	AC011939.2	0.84557757	2.12E-147	postive
MALT1	AC011939.2	0.546831498	4.77E-43	postive
PIK3R1	AC011939.2	0.671410988	2.24E-71	postive
PIK3CA	AC011939.2	0.709662803	4.18E-83	postive
PIK3CB	AC011939.2	0.539899375	8.34E-42	postive
AKT3	AC011939.2	0.553552833	2.79E-44	postive
RASGRP3	AC011939.2	0.513936775	2.13E-37	postive
IGHD6-6	AC011939.2	0.504517284	6.83E-36	postive
GNRH1	AC011939.2	0.7577367	7.10E-101	postive
IL6ST	AC011939.2	0.668036367	2.00E-70	postive
RABEP1	AC011939.2	0.56254444	5.65E-46	postive
ACVR2A	AC011939.2	0.65338359	1.94E-66	postive
ANGPTL1	AC011939.2	0.804131385	1.72E-122	postive
BMPR1A	AC011939.2	0.574919838	2.16E-48	postive
BMPR2	AC011939.2	0.786145186	1.80E-113	postive
CRLF3	AC011939.2	0.647276323	7.69E-65	postive
NR1D2	AC011939.2	0.537715285	2.03E-41	postive
NR2C2	AC011939.2	0.666420098	5.65E-70	postive
RORA	AC011939.2	0.88065063	4.67E-175	postive
SOS1	AC011939.2	0.682450671	1.41E-74	postive
SOS2	AC011939.2	0.570919919	1.34E-47	postive
BRAF	AC011939.2	0.816319245	3.75E-129	postive
ITK	AC011939.2	0.531938462	2.06E-40	postive
CBL	AC011939.2	0.595387263	1.26E-52	postive
CBLB	AC011939.2	0.744758692	1.10E-95	postive
RASGRP1	AC011939.2	0.504209765	7.64E-36	postive
PDK1	AC011939.2	0.60174994	5.29E-54	postive
TRAJ1	AC011939.2	0.674894027	2.27E-72	postive
TRAJ2	AC011939.2	0.69712806	4.66E-79	postive
TRAJ3	AC011939.2	0.741089523	2.83E-94	postive
TRAJ5	AC011939.2	0.782298672	1.18E-111	postive
TRAJ6	AC011939.2	0.752489226	9.73E-99	postive
TRAJ8	AC011939.2	0.731202555	1.37E-90	postive
TRAJ10	AC011939.2	0.732192652	5.94E-91	postive
TRAJ12	AC011939.2	0.669430735	8.12E-71	postive
TRAJ13	AC011939.2	0.777625665	1.69E-109	postive
TRAJ14	AC011939.2	0.727481237	3.02E-89	postive
TRAJ16	AC011939.2	0.743480588	3.42E-95	postive
TRAJ17	AC011939.2	0.712464152	4.86E-84	postive
TRAJ18	AC011939.2	0.689546994	1.04E-76	postive
TRAJ21	AC011939.2	0.796902256	9.35E-119	postive
TRAJ31	AC011939.2	0.828100379	4.40E-136	postive
TRAJ37	AC011939.2	0.796177235	2.17E-118	postive
TRAJ38	AC011939.2	0.838245954	1.73E-142	postive
TRAJ39	AC011939.2	0.804499847	1.10E-122	postive
CAMP	LINC01842	0.568060323	4.86E-47	postive
NR2C2	AC005332.3	0.610528547	5.89E-56	postive
BRAF	AC005332.3	0.649208726	2.42E-65	postive
TRAJ1	AC005332.3	0.569835244	2.19E-47	postive
TRAJ3	AC005332.3	0.542163328	3.30E-42	postive
TRAJ5	AC005332.3	0.629036168	2.79E-60	postive
TRAJ6	AC005332.3	0.548943825	1.97E-43	postive
TRAJ8	AC005332.3	0.588620488	3.42E-51	postive
TRAJ10	AC005332.3	0.544939311	1.05E-42	postive
TRAJ12	AC005332.3	0.577844562	5.59E-49	postive
TRAJ13	AC005332.3	0.582783137	5.54E-50	postive
TRAJ14	AC005332.3	0.606197735	5.51E-55	postive
TRAJ16	AC005332.3	0.579255961	2.90E-49	postive
TRAJ17	AC005332.3	0.605005171	1.01E-54	postive
TRAJ18	AC005332.3	0.522031313	9.87E-39	postive

TRAJ21	AC005332.3	0.59165332	7.88E-52	postive
TRAJ31	AC005332.3	0.607247006	3.22E-55	postive
TRAJ37	AC005332.3	0.538663553	1.38E-41	postive
TRAJ38	AC005332.3	0.583657595	3.66E-50	postive
TRAJ39	AC005332.3	0.588714635	3.27E-51	postive
GNRH1	HMGA1P4	0.508837632	1.41E-36	postive
NFKBIZ	AC025857.2	0.583461216	4.02E-50	postive
JAK2	AC025857.2	0.534003737	9.03E-41	postive
NFAT5	AC025857.2	0.504047945	8.10E-36	postive
GNRH1	AC025857.2	0.532242812	1.82E-40	postive
ANGPTL1	AC025857.2	0.544165738	1.44E-42	postive
CBLB	AC025857.2	0.566551033	9.55E-47	postive
TRAJ5	AC025857.2	0.525305639	2.78E-39	postive
TRAJ10	AC025857.2	0.510114119	8.81E-37	postive
TRAJ31	AC025857.2	0.505636963	4.55E-36	postive
TRAJ38	AC025857.2	0.545956509	6.87E-43	postive
CREB1	AP001160.4	0.687039011	5.99E-76	postive
RFXAP	AP001160.4	0.513064831	2.94E-37	postive
UBR1	AP001160.4	0.558493009	3.32E-45	postive
IL15	AP001160.4	0.524331452	4.06E-39	postive
CYLD	AP001160.4	0.555603839	1.16E-44	postive
MAPK8	AP001160.4	0.522719628	7.57E-39	postive
NFKBIZ	AP001160.4	0.503850997	8.70E-36	postive
IREB2	AP001160.4	0.511235244	5.82E-37	postive
DDX17	AP001160.4	0.649709924	1.79E-65	postive
JAK2	AP001160.4	0.578150562	4.85E-49	postive
TXK	AP001160.4	0.555043075	1.47E-44	postive
NFAT5	AP001160.4	0.834241865	6.58E-140	postive
PIK3R1	AP001160.4	0.551300408	7.27E-44	postive
PIK3CA	AP001160.4	0.578183235	4.78E-49	postive
GNRH1	AP001160.4	0.809639669	1.93E-125	postive
IL6ST	AP001160.4	0.571538399	1.01E-47	postive
ACVR2A	AP001160.4	0.589655337	2.08E-51	postive
ANGPTL1	AP001160.4	0.702454242	9.43E-81	postive
BMPR2	AP001160.4	0.625263485	2.24E-59	postive
CRLF3	AP001160.4	0.607930554	2.26E-55	postive
NR2C1	AP001160.4	0.562348556	6.15E-46	postive
NR2C2	AP001160.4	0.685269492	2.04E-75	postive
RORA	AP001160.4	0.819803038	3.78E-131	postive
SOS1	AP001160.4	0.600414294	1.04E-53	postive
SOS2	AP001160.4	0.513339941	2.66E-37	postive
BRAF	AP001160.4	0.759479144	1.35E-101	postive
CBL	AP001160.4	0.543487009	1.91E-42	postive
CBLB	AP001160.4	0.714079751	1.39E-84	postive
PDK1	AP001160.4	0.541421045	4.47E-42	postive
TRAJ1	AP001160.4	0.703847955	3.35E-81	postive
TRAJ2	AP001160.4	0.72170139	3.34E-87	postive
TRAJ3	AP001160.4	0.747258826	1.16E-96	postive
TRAJ5	AP001160.4	0.755310324	7.01E-100	postive
TRAJ6	AP001160.4	0.760229506	6.56E-102	postive
TRAJ8	AP001160.4	0.718800625	3.40E-86	postive
TRAJ10	AP001160.4	0.7441484	1.89E-95	postive
TRAJ12	AP001160.4	0.681357923	2.97E-74	postive
TRAJ13	AP001160.4	0.761327361	2.28E-102	postive
TRAJ14	AP001160.4	0.735044092	5.29E-92	postive
TRAJ16	AP001160.4	0.756261486	2.87E-100	postive
TRAJ17	AP001160.4	0.699245284	9.99E-80	postive
TRAJ18	AP001160.4	0.703279717	5.11E-81	postive
TRAJ21	AP001160.4	0.779609348	2.08E-110	postive
TRAJ31	AP001160.4	0.804970784	6.21E-123	postive
TRAJ37	AP001160.4	0.798922808	8.76E-120	postive
TRAJ38	AP001160.4	0.827136381	1.70E-135	postive
TRAJ39	AP001160.4	0.785414456	4.01E-113	postive
CREB1	MIR4453HG	0.523643689	5.30E-39	postive
RFXAP	MIR4453HG	0.609989003	7.80E-56	postive

DDX17	MIR4453HG	0.554352185	1.98E-44	postive
NFAT5	MIR4453HG	0.544142809	1.46E-42	postive
GNRH1	MIR4453HG	0.580427376	1.68E-49	postive
NR2C2	MIR4453HG	0.572159032	7.63E-48	postive
RORA	MIR4453HG	0.569599632	2.43E-47	postive
SOS1	MIR4453HG	0.545098279	9.81E-43	postive
BRAF	MIR4453HG	0.572592041	6.26E-48	postive
TRAJ1	MIR4453HG	0.511065289	6.19E-37	postive
TRAJ3	MIR4453HG	0.525021131	3.11E-39	postive
TRAJ5	MIR4453HG	0.520416688	1.83E-38	postive
TRAJ6	MIR4453HG	0.507498968	2.30E-36	postive
TRAJ16	MIR4453HG	0.50233175	1.51E-35	postive
TRAJ21	MIR4453HG	0.508237884	1.76E-36	postive
TRAJ31	MIR4453HG	0.508175949	1.80E-36	postive
TRAJ37	MIR4453HG	0.509780207	9.96E-37	postive
TRAJ38	MIR4453HG	0.53759076	2.13E-41	postive
TRAJ39	MIR4453HG	0.520631879	1.69E-38	postive
CREB1	MIR222HG	0.587289031	6.49E-51	postive
IL15	MIR222HG	0.599606403	1.55E-53	postive
CYLD	MIR222HG	0.532730514	1.50E-40	postive
JAK2	MIR222HG	0.608670243	1.54E-55	postive
NFAT5	MIR222HG	0.621761916	1.51E-58	postive
PIK3R1	MIR222HG	0.52998207	4.46E-40	postive
PIK3CA	MIR222HG	0.520644333	1.68E-38	postive
GNRH1	MIR222HG	0.526713301	1.61E-39	postive
ANGPTL1	MIR222HG	0.642878807	1.03E-63	postive
BMPR2	MIR222HG	0.512599359	3.50E-37	postive
NR2C2	MIR222HG	0.526263497	1.92E-39	postive
RORA	MIR222HG	0.629502212	2.15E-60	postive
SOS1	MIR222HG	0.509706127	1.02E-36	postive
BRAF	MIR222HG	0.645160885	2.69E-64	postive
ITK	MIR222HG	0.511693399	4.91E-37	postive
CBL	MIR222HG	0.531897224	2.09E-40	postive
CBLB	MIR222HG	0.720446123	9.15E-87	postive
TRAJ1	MIR222HG	0.543151888	2.20E-42	postive
TRAJ2	MIR222HG	0.615622384	4.06E-57	postive
TRAJ3	MIR222HG	0.635168061	8.84E-62	postive
TRAJ5	MIR222HG	0.581081297	1.23E-49	postive
TRAJ6	MIR222HG	0.612328782	2.30E-56	postive
TRAJ10	MIR222HG	0.592026498	6.57E-52	postive
TRAJ12	MIR222HG	0.645329086	2.44E-64	postive
TRAJ13	MIR222HG	0.557902976	4.29E-45	postive
TRAJ14	MIR222HG	0.625164375	2.37E-59	postive
TRAJ16	MIR222HG	0.565510527	1.52E-46	postive
TRAJ17	MIR222HG	0.517986147	4.62E-38	postive
TRAJ18	MIR222HG	0.66766998	2.53E-70	postive
TRAJ21	MIR222HG	0.626150749	1.38E-59	postive
TRAJ31	MIR222HG	0.609736984	8.89E-56	postive
TRAJ37	MIR222HG	0.592505279	5.20E-52	postive
TRAJ38	MIR222HG	0.698620505	1.58E-79	postive
TRAJ39	MIR222HG	0.582114427	7.59E-50	postive
CIITA	AL023653.1	0.567629695	5.90E-47	postive
IL15	AL023653.1	0.535149399	5.70E-41	postive
CYLD	AL023653.1	0.52310954	6.52E-39	postive
DDX17	AL023653.1	0.572360882	6.96E-48	postive
JAK2	AL023653.1	0.503527317	9.78E-36	postive
NFAT5	AL023653.1	0.509061967	1.30E-36	postive
RASGRP3	AL023653.1	0.552059522	5.27E-44	postive
IGHD4-4	AL023653.1	0.516645536	7.68E-38	postive
IGHD6-6	AL023653.1	0.500839257	2.57E-35	postive
GNRH1	AL023653.1	0.635834021	6.05E-62	postive
NR2C2	AL023653.1	0.610150349	7.17E-56	postive
RORA	AL023653.1	0.565131377	1.80E-46	postive
BRAF	AL023653.1	0.563678997	3.42E-46	postive
ITK	AL023653.1	0.581650179	9.45E-50	postive

CD28	AL023653.1	0.528855378	6.95E-40	postive
TRAJ1	AL023653.1	0.573235488	4.67E-48	postive
TRAJ2	AL023653.1	0.517193622	6.24E-38	postive
TRAJ3	AL023653.1	0.621631132	1.62E-58	postive
TRAJ5	AL023653.1	0.556261023	8.73E-45	postive
TRAJ6	AL023653.1	0.531575608	2.37E-40	postive
TRAJ8	AL023653.1	0.509797452	9.90E-37	postive
TRAJ10	AL023653.1	0.50343844	1.01E-35	postive
TRAJ12	AL023653.1	0.518408575	3.94E-38	postive
TRAJ14	AL023653.1	0.527091655	1.39E-39	postive
TRAJ16	AL023653.1	0.509312163	1.18E-36	postive
TRAJ17	AL023653.1	0.519182043	2.94E-38	postive
TRAJ18	AL023653.1	0.51593284	1.00E-37	postive
TRAJ21	AL023653.1	0.526471413	1.77E-39	postive
TRAJ31	AL023653.1	0.527747055	1.07E-39	postive
TRAJ37	AL023653.1	0.516068889	9.55E-38	postive
TRAJ38	AL023653.1	0.549367804	1.65E-43	postive
TRAJ39	AL023653.1	0.558334456	3.56E-45	postive
CREB1	AC124016.3	0.505159966	5.41E-36	postive
DDX17	AC124016.3	0.536734058	3.01E-41	postive
NFAT5	AC124016.3	0.589550694	2.19E-51	postive
GNRH1	AC124016.3	0.633603476	2.15E-61	postive
ACVR2A	AC124016.3	0.526578402	1.70E-39	postive
NR2C2	AC124016.3	0.579944244	2.10E-49	postive
RORA	AC124016.3	0.599175229	1.93E-53	postive
BRAF	AC124016.3	0.527919928	1.00E-39	postive
TRAJ1	AC124016.3	0.5116473	4.99E-37	postive
TRAJ3	AC124016.3	0.513730587	2.30E-37	postive
TRAJ5	AC124016.3	0.544079402	1.50E-42	postive
TRAJ6	AC124016.3	0.544700139	1.16E-42	postive
TRAJ8	AC124016.3	0.522884037	7.11E-39	postive
TRAJ10	AC124016.3	0.533539346	1.09E-40	postive
TRAJ13	AC124016.3	0.526896849	1.50E-39	postive
TRAJ14	AC124016.3	0.517703041	5.15E-38	postive
TRAJ16	AC124016.3	0.547264469	3.98E-43	postive
TRAJ17	AC124016.3	0.510361869	8.04E-37	postive
TRAJ21	AC124016.3	0.537213758	2.48E-41	postive
TRAJ31	AC124016.3	0.573713621	3.75E-48	postive
TRAJ37	AC124016.3	0.56630235	1.07E-46	postive
TRAJ38	AC124016.3	0.573854073	3.52E-48	postive
TRAJ39	AC124016.3	0.564411073	2.47E-46	postive
KRAS	AC022364.1	0.656339842	3.18E-67	postive
NR2C2	THAP9-AS1	0.529270333	5.91E-40	postive
CREB1	BCL2L1-AS1	0.791101832	7.22E-116	postive
RFXAP	BCL2L1-AS1	0.535169242	5.66E-41	postive
UBR1	BCL2L1-AS1	0.683792586	5.64E-75	postive
ZC3HAV1	BCL2L1-AS1	0.56829066	4.38E-47	postive
IL15	BCL2L1-AS1	0.595397658	1.26E-52	postive
CYLD	BCL2L1-AS1	0.648924175	2.87E-65	postive
EIF2AK2	BCL2L1-AS1	0.549254697	1.73E-43	postive
MAPK8	BCL2L1-AS1	0.56018807	1.59E-45	postive
LMBR1	BCL2L1-AS1	0.567174953	7.23E-47	postive
LIMS1	BCL2L1-AS1	0.521707669	1.12E-38	postive
IREB2	BCL2L1-AS1	0.614635214	6.84E-57	postive
DDX17	BCL2L1-AS1	0.627039578	8.42E-60	postive
PIK3CG	BCL2L1-AS1	0.555768018	1.08E-44	postive
JAK2	BCL2L1-AS1	0.659333192	4.97E-68	postive
TXK	BCL2L1-AS1	0.628477614	3.80E-60	postive
NFAT5	BCL2L1-AS1	0.902588501	3.11E-197	postive
NFATC3	BCL2L1-AS1	0.551833521	5.80E-44	postive
MALT1	BCL2L1-AS1	0.549507357	1.55E-43	postive
PIK3R1	BCL2L1-AS1	0.647846394	5.47E-65	postive
PIK3CA	BCL2L1-AS1	0.700444336	4.15E-80	postive
PIK3CB	BCL2L1-AS1	0.528949024	6.70E-40	postive
AKT3	BCL2L1-AS1	0.522677883	7.70E-39	postive

RASGRP3	BCL2L1-AS1	0.528490612	8.03E-40	postive
IGHD4-4	BCL2L1-AS1	0.500829045	2.58E-35	postive
IGHD6-6	BCL2L1-AS1	0.591350975	9.13E-52	postive
PLXNC1	BCL2L1-AS1	0.503018201	1.18E-35	postive
GNRH1	BCL2L1-AS1	0.784264693	1.40E-112	postive
IL6ST	BCL2L1-AS1	0.623367074	6.32E-59	postive
IL7	BCL2L1-AS1	0.52258682	7.97E-39	postive
RABEP1	BCL2L1-AS1	0.57581461	1.43E-48	postive
ACVR2A	BCL2L1-AS1	0.64722706	7.92E-65	postive
ANGPTL1	BCL2L1-AS1	0.741839073	1.46E-94	postive
BMPR1A	BCL2L1-AS1	0.561778735	7.91E-46	postive
BMPR2	BCL2L1-AS1	0.735978497	2.38E-92	postive
CRLF3	BCL2L1-AS1	0.654911197	7.64E-67	postive
NR1D2	BCL2L1-AS1	0.51699864	6.72E-38	postive
NR2C2	BCL2L1-AS1	0.715513925	4.53E-85	postive
RORA	BCL2L1-AS1	0.854371664	1.22E-153	postive
SOS1	BCL2L1-AS1	0.669601175	7.27E-71	postive
SOS2	BCL2L1-AS1	0.576090923	1.26E-48	postive
BRAF	BCL2L1-AS1	0.782889876	6.22E-112	postive
TEC	BCL2L1-AS1	0.540873792	5.60E-42	postive
CBL	BCL2L1-AS1	0.602553448	3.53E-54	postive
CBLB	BCL2L1-AS1	0.702473331	9.30E-81	postive
RASGRP1	BCL2L1-AS1	0.524922145	3.23E-39	postive
PDK1	BCL2L1-AS1	0.595866244	9.98E-53	postive
TRAJ1	BCL2L1-AS1	0.775390134	1.74E-108	postive
TRAJ2	BCL2L1-AS1	0.767773705	4.05E-105	postive
TRAJ3	BCL2L1-AS1	0.849671449	2.96E-150	postive
TRAJ5	BCL2L1-AS1	0.786666666	1.01E-113	postive
TRAJ6	BCL2L1-AS1	0.847782939	6.29E-149	postive
TRAJ8	BCL2L1-AS1	0.786106354	1.88E-113	postive
TRAJ10	BCL2L1-AS1	0.827828344	6.45E-136	postive
TRAJ12	BCL2L1-AS1	0.764176335	1.42E-103	postive
TRAJ13	BCL2L1-AS1	0.791509887	4.56E-116	postive
TRAJ14	BCL2L1-AS1	0.789702489	3.48E-115	postive
TRAJ16	BCL2L1-AS1	0.832579948	7.39E-139	postive
TRAJ17	BCL2L1-AS1	0.787860908	2.71E-114	postive
TRAJ18	BCL2L1-AS1	0.804636722	9.33E-123	postive
TRAJ21	BCL2L1-AS1	0.840297706	7.74E-144	postive
TRAJ31	BCL2L1-AS1	0.842929907	1.35E-145	postive
TRAJ37	BCL2L1-AS1	0.847180513	1.65E-148	postive
TRAJ38	BCL2L1-AS1	0.881887669	3.46E-176	postive
TRAJ39	BCL2L1-AS1	0.868974415	5.71E-165	postive
CREB1	AC007878.1	0.817658526	6.48E-130	postive
RFXAP	AC007878.1	0.512387885	3.79E-37	postive
UBR1	AC007878.1	0.666831404	4.34E-70	postive
ZC3HAV1	AC007878.1	0.584085088	2.99E-50	postive
ZC3HAV1L	AC007878.1	0.505582435	4.64E-36	postive
IL15	AC007878.1	0.605518196	7.81E-55	postive
CYLD	AC007878.1	0.636899174	3.29E-62	postive
EIF2AK2	AC007878.1	0.583286688	4.37E-50	postive
MAPK8	AC007878.1	0.582912682	5.21E-50	postive
TLR1	AC007878.1	0.503300018	1.06E-35	postive
LMBR1	AC007878.1	0.613578972	1.19E-56	postive
LIMS1	AC007878.1	0.542578909	2.78E-42	postive
IREB2	AC007878.1	0.634684856	1.16E-61	postive
DDX17	AC007878.1	0.609407277	1.05E-55	postive
PIK3CG	AC007878.1	0.559964969	1.75E-45	postive
JAK2	AC007878.1	0.679811062	8.47E-74	postive
TXK	AC007878.1	0.610923759	4.79E-56	postive
PPP3CB	AC007878.1	0.503464631	1.00E-35	postive
NFAT5	AC007878.1	0.874486586	1.32E-169	postive
MALT1	AC007878.1	0.557645637	4.80E-45	postive
PIK3R1	AC007878.1	0.670777023	3.39E-71	postive
PIK3CA	AC007878.1	0.730336339	2.82E-90	postive
PIK3CB	AC007878.1	0.553430876	2.94E-44	postive

AKT3	AC007878.1	0.553602389	2.73E-44	postive
RASGRP3	AC007878.1	0.561094462	1.07E-45	postive
PLXNC1	AC007878.1	0.530323158	3.90E-40	postive
GNRH1	AC007878.1	0.737952199	4.35E-93	postive
IL6ST	AC007878.1	0.65646898	2.93E-67	postive
RABEP1	AC007878.1	0.575815941	1.43E-48	postive
ACVR2A	AC007878.1	0.661095203	1.65E-68	postive
ANGPTL1	AC007878.1	0.81313691	2.29E-127	postive
BMPR1A	AC007878.1	0.561436678	9.19E-46	postive
BMPR2	AC007878.1	0.764523906	1.01E-103	postive
CRLF3	AC007878.1	0.676858335	6.14E-73	postive
LIFR	AC007878.1	0.500999473	2.43E-35	postive
NR1D2	AC007878.1	0.532437252	1.69E-40	postive
NR2C2	AC007878.1	0.702694185	7.90E-81	postive
NR3C1	AC007878.1	0.501064116	2.37E-35	postive
RORA	AC007878.1	0.874221307	2.23E-169	postive
SOS1	AC007878.1	0.723271895	9.41E-88	postive
SOS2	AC007878.1	0.607504767	2.82E-55	postive
BRAF	AC007878.1	0.83720379	8.25E-142	postive
ITK	AC007878.1	0.536527658	3.27E-41	postive
CD28	AC007878.1	0.515762598	1.07E-37	postive
CBL	AC007878.1	0.621208706	2.04E-58	postive
CBLB	AC007878.1	0.773283721	1.53E-107	postive
RASGRP1	AC007878.1	0.52852162	7.93E-40	postive
PDK1	AC007878.1	0.609606446	9.51E-56	postive
TRAJ1	AC007878.1	0.783911322	2.06E-112	postive
TRAJ2	AC007878.1	0.770447228	2.76E-106	postive
TRAJ3	AC007878.1	0.820162629	2.34E-131	postive
TRAJ5	AC007878.1	0.863171556	2.60E-160	postive
TRAJ6	AC007878.1	0.843392231	6.56E-146	postive
TRAJ8	AC007878.1	0.823207745	3.84E-133	postive
TRAJ10	AC007878.1	0.821267382	5.31E-132	postive
TRAJ12	AC007878.1	0.778916282	4.34E-110	postive
TRAJ13	AC007878.1	0.853351038	6.78E-153	postive
TRAJ14	AC007878.1	0.837144458	9.01E-142	postive
TRAJ16	AC007878.1	0.842712592	1.89E-145	postive
TRAJ17	AC007878.1	0.806079012	1.60E-123	postive
TRAJ18	AC007878.1	0.782188391	1.33E-111	postive
TRAJ21	AC007878.1	0.875529786	1.65E-170	postive
TRAJ31	AC007878.1	0.896313389	2.18E-190	postive
TRAJ37	AC007878.1	0.861761932	3.27E-159	postive
TRAJ38	AC007878.1	0.921591451	3.29E-221	postive
TRAJ39	AC007878.1	0.884591398	1.06E-178	postive
CREB1	MIR155HG	0.536829302	2.90E-41	postive
IFNG	MIR155HG	0.64103239	3.03E-63	postive
CIITA	MIR155HG	0.547954291	2.98E-43	postive
TLR4	MIR155HG	0.583102056	4.77E-50	postive
APOBEC3G	MIR155HG	0.518838327	3.35E-38	postive
CYBB	MIR155HG	0.550733221	9.25E-44	postive
IL15	MIR155HG	0.754631215	1.33E-99	postive
CYLD	MIR155HG	0.608482564	1.70E-55	postive
TLR1	MIR155HG	0.562928259	4.77E-46	postive
IL7R	MIR155HG	0.563387598	3.89E-46	postive
TRIM22	MIR155HG	0.544142591	1.46E-42	postive
TANK	MIR155HG	0.505696892	4.45E-36	postive
PIK3CG	MIR155HG	0.624020778	4.42E-59	postive
CCR5	MIR155HG	0.548823947	2.07E-43	postive
CXCR6	MIR155HG	0.632012249	5.27E-61	postive
JAK2	MIR155HG	0.710585217	2.06E-83	postive
BTK	MIR155HG	0.5459796	6.80E-43	postive
PPP3CC	MIR155HG	0.513932319	2.13E-37	postive
NFAT5	MIR155HG	0.561687808	8.23E-46	postive
NFATC2	MIR155HG	0.52491689	3.24E-39	postive
CD72	MIR155HG	0.503475466	9.96E-36	postive
FCGR2B	MIR155HG	0.500103948	3.35E-35	postive

RASGRP3	MIR155HG	0.633919749	1.80E-61	postive
PRKCB	MIR155HG	0.538869337	1.27E-41	postive
IGKJ5	MIR155HG	0.500180673	3.26E-35	postive
PLXNC1	MIR155HG	0.644628285	3.69E-64	postive
GNRH1	MIR155HG	0.51989775	2.23E-38	postive
IL7	MIR155HG	0.548566474	2.31E-43	postive
TNFSF13B	MIR155HG	0.625746822	1.72E-59	postive
TNFSF8	MIR155HG	0.596323916	7.96E-53	postive
ANGPTL1	MIR155HG	0.561842979	7.69E-46	postive
CRLF3	MIR155HG	0.657246569	1.82E-67	postive
IL10RA	MIR155HG	0.559835365	1.85E-45	postive
IL12RB1	MIR155HG	0.528464868	8.11E-40	postive
IL18R1	MIR155HG	0.514103866	2.00E-37	postive
IL18RAP	MIR155HG	0.699003823	1.19E-79	postive
RORA	MIR155HG	0.60126143	6.77E-54	postive
CD247	MIR155HG	0.535486	4.98E-41	postive
LCP2	MIR155HG	0.65320611	2.16E-66	postive
SH2D1A	MIR155HG	0.715069047	6.42E-85	postive
CD3D	MIR155HG	0.531474489	2.47E-40	postive
CD3G	MIR155HG	0.630495805	1.23E-60	postive
PTPRC	MIR155HG	0.732968194	3.09E-91	postive
ITK	MIR155HG	0.747288596	1.13E-96	postive
CD28	MIR155HG	0.681280959	3.13E-74	postive
ICOS	MIR155HG	0.752741194	7.71E-99	postive
CTLA4	MIR155HG	0.620183676	3.55E-58	postive
CBLB	MIR155HG	0.525197807	2.90E-39	postive
PDK1	MIR155HG	0.596447569	7.49E-53	postive
TRAJ1	MIR155HG	0.632602471	3.78E-61	postive
TRAJ2	MIR155HG	0.61231687	2.32E-56	postive
TRAJ3	MIR155HG	0.655170122	6.52E-67	postive
TRAJ5	MIR155HG	0.651653611	5.55E-66	postive
TRAJ6	MIR155HG	0.620711031	2.67E-58	postive
TRAJ8	MIR155HG	0.562618052	5.47E-46	postive
TRAJ10	MIR155HG	0.615225237	5.01E-57	postive
TRAJ12	MIR155HG	0.540452026	6.65E-42	postive
TRAJ13	MIR155HG	0.599998911	1.28E-53	postive
TRAJ14	MIR155HG	0.596482343	7.36E-53	postive
TRAJ16	MIR155HG	0.620520037	2.96E-58	postive
TRAJ17	MIR155HG	0.595179286	1.40E-52	postive
TRAJ18	MIR155HG	0.620742229	2.62E-58	postive
TRAJ21	MIR155HG	0.61963894	4.75E-58	postive
TRAJ31	MIR155HG	0.621005888	2.28E-58	postive
TRAJ37	MIR155HG	0.594209179	2.26E-52	postive
TRAJ38	MIR155HG	0.647427728	7.02E-65	postive
TRAJ39	MIR155HG	0.625433484	2.04E-59	postive
TRAV4	MIR155HG	0.521404023	1.26E-38	postive
TRBC1	MIR155HG	0.6051983	9.19E-55	postive
TRBV28	MIR155HG	0.50253758	1.40E-35	postive
CREB1	ERVK13-1	0.723306137	9.16E-88	postive
RFXAP	ERVK13-1	0.551230109	7.49E-44	postive
UBR1	ERVK13-1	0.647711462	5.93E-65	postive
ZC3HAV1	ERVK13-1	0.518156878	4.33E-38	postive
CYLD	ERVK13-1	0.663305409	4.10E-69	postive
IREB2	ERVK13-1	0.542053586	3.45E-42	postive
DDX17	ERVK13-1	0.753532817	3.69E-99	postive
PIK3CG	ERVK13-1	0.500618547	2.78E-35	postive
JAK2	ERVK13-1	0.600300519	1.10E-53	postive
TXK	ERVK13-1	0.608240107	1.93E-55	postive
NFAT5	ERVK13-1	0.859217928	2.94E-157	postive
NFATC3	ERVK13-1	0.558609417	3.16E-45	postive
PIK3R1	ERVK13-1	0.643408588	7.56E-64	postive
PIK3CA	ERVK13-1	0.599075789	2.03E-53	postive
RASGRP3	ERVK13-1	0.500074142	3.38E-35	postive
IGHD6-6	ERVK13-1	0.534749198	6.70E-41	postive
LTB4R2	ERVK13-1	0.551669033	6.22E-44	postive

ROBO2	ERVK13-1	0.520180215	2.01E-38	postive
GNRH1	ERVK13-1	0.816683961	2.33E-129	postive
IL6ST	ERVK13-1	0.643572345	6.87E-64	postive
RABEP1	ERVK13-1	0.538305724	1.59E-41	postive
ACVR2A	ERVK13-1	0.635545323	7.13E-62	postive
ANGPTL1	ERVK13-1	0.655996601	3.92E-67	postive
BMPR2	ERVK13-1	0.675333306	1.69E-72	postive
CRLF3	ERVK13-1	0.556735449	7.11E-45	postive
NR1D2	ERVK13-1	0.527493154	1.19E-39	postive
NR2C1	ERVK13-1	0.564374042	2.52E-46	postive
NR2C2	ERVK13-1	0.779022448	3.88E-110	postive
NR3C2	ERVK13-1	0.516710308	7.50E-38	postive
RORA	ERVK13-1	0.859244855	2.80E-157	postive
SOS1	ERVK13-1	0.614661174	6.75E-57	postive
SOS2	ERVK13-1	0.552022085	5.35E-44	postive
BRAF	ERVK13-1	0.761736536	1.53E-102	postive
TEC	ERVK13-1	0.511744632	4.81E-37	postive
CBL	ERVK13-1	0.565203256	1.74E-46	postive
CBLB	ERVK13-1	0.674207433	3.57E-72	postive
TRAJ1	ERVK13-1	0.701034185	2.69E-80	postive
TRAJ2	ERVK13-1	0.693809403	5.08E-78	postive
TRAJ3	ERVK13-1	0.742698134	6.85E-95	postive
TRAJ5	ERVK13-1	0.727421516	3.17E-89	postive
TRAJ6	ERVK13-1	0.7295328	5.51E-90	postive
TRAJ8	ERVK13-1	0.676776962	6.49E-73	postive
TRAJ10	ERVK13-1	0.714548212	9.64E-85	postive
TRAJ12	ERVK13-1	0.672643151	1.00E-71	postive
TRAJ13	ERVK13-1	0.703125814	5.73E-81	postive
TRAJ14	ERVK13-1	0.681740671	2.29E-74	postive
TRAJ16	ERVK13-1	0.724162326	4.57E-88	postive
TRAJ17	ERVK13-1	0.674572139	2.81E-72	postive
TRAJ18	ERVK13-1	0.710471777	2.25E-83	postive
TRAJ21	ERVK13-1	0.735124943	4.94E-92	postive
TRAJ31	ERVK13-1	0.753737679	3.05E-99	postive
TRAJ37	ERVK13-1	0.763627828	2.43E-103	postive
TRAJ38	ERVK13-1	0.783090691	5.01E-112	postive
TRAJ39	ERVK13-1	0.761525666	1.88E-102	postive
DDX17	DGUOK-AS1	0.512607571	3.49E-37	postive
NFAT5	DGUOK-AS1	0.51096327	6.43E-37	postive
GNRH1	DGUOK-AS1	0.622758407	8.80E-59	postive
NR2C2	DGUOK-AS1	0.527065047	1.40E-39	postive
BRAF	DGUOK-AS1	0.551677334	6.20E-44	postive
TRAJ8	DGUOK-AS1	0.502077722	1.65E-35	postive
TRAJ21	DGUOK-AS1	0.500227833	3.20E-35	postive
TRAJ31	DGUOK-AS1	0.502212384	1.57E-35	postive
TRAJ38	DGUOK-AS1	0.527558352	1.16E-39	postive
KIR2DL1	LINC02036	0.622800676	8.60E-59	postive
CIITA	AC116914.2	0.503043784	1.16E-35	postive
DDX17	AC116914.2	0.553849959	2.46E-44	postive
IRF9	AC116914.2	0.572933227	5.36E-48	postive
GNRH1	AC116914.2	0.586775245	8.31E-51	postive
IL18RAP	AC116914.2	0.523997783	4.62E-39	postive
NR2C2	AC116914.2	0.510149909	8.69E-37	postive
ZAP70	AC116914.2	0.51186181	4.61E-37	postive
CREB1	AC005540.1	0.63062487	1.15E-60	postive
UBR1	AC005540.1	0.578344852	4.43E-49	postive
ZC3HAV1	AC005540.1	0.501592981	1.96E-35	postive
ZC3HAV1L	AC005540.1	0.564828665	2.06E-46	postive
IL15	AC005540.1	0.595290516	1.33E-52	postive
LMBR1	AC005540.1	0.518868784	3.31E-38	postive
IREB2	AC005540.1	0.557555909	4.99E-45	postive
JAK2	AC005540.1	0.503970248	8.33E-36	postive
NFAT5	AC005540.1	0.591052897	1.06E-51	postive
PIK3R1	AC005540.1	0.527896197	1.01E-39	postive
PIK3CA	AC005540.1	0.518464202	3.86E-38	postive

IGHD4-4	AC005540.1	0.558678283	3.06E-45	postive
GNRH1	AC005540.1	0.578792772	3.60E-49	postive
ACVR2A	AC005540.1	0.509159025	1.25E-36	postive
ANGPTL1	AC005540.1	0.636913226	3.26E-62	postive
BMPR2	AC005540.1	0.546896451	4.64E-43	postive
CRLF3	AC005540.1	0.53816856	1.69E-41	postive
NR2C2	AC005540.1	0.591691242	7.74E-52	postive
RORA	AC005540.1	0.559902945	1.80E-45	postive
SOS1	AC005540.1	0.559489544	2.15E-45	postive
SOS2	AC005540.1	0.505944385	4.07E-36	postive
BRAF	AC005540.1	0.723805557	6.11E-88	postive
CBL	AC005540.1	0.528965643	6.66E-40	postive
CBLB	AC005540.1	0.543388622	1.99E-42	postive
PDK1	AC005540.1	0.510564038	7.46E-37	postive
TRAJ1	AC005540.1	0.61417595	8.72E-57	postive
TRAJ2	AC005540.1	0.612209565	2.45E-56	postive
TRAJ3	AC005540.1	0.718760399	3.51E-86	postive
TRAJ5	AC005540.1	0.63844372	1.36E-62	postive
TRAJ6	AC005540.1	0.664170488	2.37E-69	postive
TRAJ8	AC005540.1	0.670791131	3.36E-71	postive
TRAJ10	AC005540.1	0.632920084	3.16E-61	postive
TRAJ12	AC005540.1	0.648967212	2.80E-65	postive
TRAJ13	AC005540.1	0.63656726	3.98E-62	postive
TRAJ14	AC005540.1	0.686933401	6.45E-76	postive
TRAJ16	AC005540.1	0.587989144	4.64E-51	postive
TRAJ17	AC005540.1	0.635759508	6.31E-62	postive
TRAJ18	AC005540.1	0.583740734	3.52E-50	postive
TRAJ21	AC005540.1	0.678760549	1.72E-73	postive
TRAJ31	AC005540.1	0.651709065	5.37E-66	postive
TRAJ37	AC005540.1	0.630572981	1.18E-60	postive
TRAJ38	AC005540.1	0.694105698	4.11E-78	postive
TRAJ39	AC005540.1	0.683153024	8.74E-75	postive
CREB1	AC125257.1	0.500614853	2.79E-35	postive
MAPK8	AC125257.1	0.521306	1.30E-38	postive
BRAF	AC125257.1	0.593693139	2.91E-52	postive
DDX17	UBE2Q1-AS1	0.595074073	1.48E-52	postive
NFAT5	UBE2Q1-AS1	0.560494993	1.39E-45	postive
LTB4R2	UBE2Q1-AS1	0.51129014	5.70E-37	postive
GNRH1	UBE2Q1-AS1	0.65247557	3.38E-66	postive
NR2C1	UBE2Q1-AS1	0.539744764	8.88E-42	postive
NR2C2	UBE2Q1-AS1	0.666558053	5.17E-70	postive
BRAF	UBE2Q1-AS1	0.56245094	5.88E-46	postive
TRAJ1	UBE2Q1-AS1	0.559788945	1.89E-45	postive
TRAJ2	UBE2Q1-AS1	0.507214222	2.56E-36	postive
TRAJ3	UBE2Q1-AS1	0.607652352	2.61E-55	postive
TRAJ6	UBE2Q1-AS1	0.565958556	1.24E-46	postive
TRAJ8	UBE2Q1-AS1	0.539668667	9.16E-42	postive
TRAJ10	UBE2Q1-AS1	0.555023357	1.49E-44	postive
TRAJ13	UBE2Q1-AS1	0.519593247	2.51E-38	postive
TRAJ14	UBE2Q1-AS1	0.530542502	3.58E-40	postive
TRAJ16	UBE2Q1-AS1	0.554095042	2.21E-44	postive
TRAJ17	UBE2Q1-AS1	0.548004305	2.92E-43	postive
TRAJ18	UBE2Q1-AS1	0.507627225	2.20E-36	postive
TRAJ21	UBE2Q1-AS1	0.542118163	3.36E-42	postive
TRAJ31	UBE2Q1-AS1	0.507409438	2.38E-36	postive
TRAJ37	UBE2Q1-AS1	0.524583651	3.69E-39	postive
TRAJ38	UBE2Q1-AS1	0.547222465	4.05E-43	postive
TRAJ39	UBE2Q1-AS1	0.542384363	3.01E-42	postive
DDX17	AL133410.1	0.607692653	2.56E-55	postive
NFAT5	AL133410.1	0.52078364	1.59E-38	postive
GNRH1	AL133410.1	0.660947476	1.81E-68	postive
NR2C2	AL133410.1	0.602170181	4.28E-54	postive
CD79A	LINC00582	0.640342473	4.52E-63	postive
CD79B	LINC00582	0.545305854	9.00E-43	postive
IGHA1	LINC00582	0.522098252	9.62E-39	postive

IGHD6-25	LINC00582	0.515018643	1.42E-37	postive
IGHG1	LINC00582	0.719320092	2.25E-86	postive
IGHG2	LINC00582	0.585266404	1.71E-50	postive
IGHG3	LINC00582	0.644390796	4.24E-64	postive
IGHJ2	LINC00582	0.52051784	1.76E-38	postive
IGHJ3	LINC00582	0.581809548	8.77E-50	postive
IGHV1-18	LINC00582	0.556839041	6.80E-45	postive
IGHV1-2	LINC00582	0.504510388	6.85E-36	postive
IGHV1-3	LINC00582	0.500008563	3.46E-35	postive
IGHV1-46	LINC00582	0.537812325	1.95E-41	postive
IGHV3-11	LINC00582	0.53162135	2.33E-40	postive
IGHV3-15	LINC00582	0.660980062	1.78E-68	postive
IGHV3-21	LINC00582	0.666180135	6.59E-70	postive
IGHV3-23	LINC00582	0.729580838	5.29E-90	postive
IGHV3-30	LINC00582	0.634681914	1.17E-61	postive
IGHV3-33	LINC00582	0.55177316	5.95E-44	postive
IGHV3-38	LINC00582	0.553021427	3.50E-44	postive
IGHV3-48	LINC00582	0.558670809	3.07E-45	postive
IGHV3-74	LINC00582	0.591042444	1.06E-51	postive
IGHV4-28	LINC00582	0.548160978	2.73E-43	postive
IGHV4-34	LINC00582	0.57035014	1.73E-47	postive
IGHV4-39	LINC00582	0.500424949	2.98E-35	postive
IGHV4-59	LINC00582	0.536241895	3.67E-41	postive
IGHV5-51	LINC00582	0.652645995	3.04E-66	postive
IGKC	LINC00582	0.848603435	1.68E-149	postive
IGKJ5	LINC00582	0.730088792	3.47E-90	postive
IGKV1-12	LINC00582	0.631030977	9.14E-61	postive
IGKV1-13	LINC00582	0.605001326	1.02E-54	postive
IGKV1-33	LINC00582	0.569932708	2.09E-47	postive
IGKV1-39	LINC00582	0.552810938	3.83E-44	postive
IGKV1-5	LINC00582	0.711554923	9.79E-84	postive
IGKV1-6	LINC00582	0.54572909	7.55E-43	postive
IGKV1-9	LINC00582	0.666229469	6.38E-70	postive
IGKV1D-33	LINC00582	0.555135562	1.42E-44	postive
IGKV1D-39	LINC00582	0.582473647	6.41E-50	postive
IGKV2-30	LINC00582	0.58299769	5.01E-50	postive
IGKV3-11	LINC00582	0.67514683	1.92E-72	postive
IGKV3-15	LINC00582	0.6942114	3.81E-78	postive
IGKV3-20	LINC00582	0.72877853	1.03E-89	postive
IGKV3-7	LINC00582	0.620463687	3.05E-58	postive
IGKV3D-11	LINC00582	0.592829795	4.44E-52	postive
IGKV3D-20	LINC00582	0.56346662	3.76E-46	postive
IGKV4-1	LINC00582	0.721802523	3.08E-87	postive
IGKV6-21	LINC00582	0.502698943	1.32E-35	postive
IGLC2	LINC00582	0.734832003	6.34E-92	postive
IGLC3	LINC00582	0.746828304	1.72E-96	postive
IGLJ1	LINC00582	0.546128252	6.39E-43	postive
IGLJ3	LINC00582	0.521824723	1.07E-38	postive
IGLV1-40	LINC00582	0.572906759	5.42E-48	postive
IGLV1-44	LINC00582	0.709595752	4.39E-83	postive
IGLV1-47	LINC00582	0.616755989	2.22E-57	postive
IGLV2-11	LINC00582	0.593355832	3.43E-52	postive
IGLV2-14	LINC00582	0.611076388	4.43E-56	postive
IGLV2-8	LINC00582	0.588866736	3.04E-51	postive
IGLV3-1	LINC00582	0.557207034	5.80E-45	postive
IGLV3-10	LINC00582	0.62966235	1.97E-60	postive
IGLV3-21	LINC00582	0.594494625	1.96E-52	postive
IGLV3-25	LINC00582	0.665090922	1.32E-69	postive
IGLV3-9	LINC00582	0.564704579	2.17E-46	postive
IGLV6-57	LINC00582	0.742382546	9.05E-95	postive
IGLV8-61	LINC00582	0.543424086	1.96E-42	postive
PNOC	LINC00582	0.68696551	6.30E-76	postive
TNFSF13B	LINC00582	0.50435235	7.25E-36	postive
TNFRSF17	LINC00582	0.885174841	2.97E-179	postive
CD48	LINC00582	0.589997538	1.76E-51	postive

SH2D1A	LINC00582	0.558080846	3.97E-45	postive
CD3D	LINC00582	0.51700567	6.70E-38	postive
TRAV16	LINC00582	0.503914426	8.50E-36	postive
TRBV5-1	LINC00582	0.509775138	9.98E-37	postive
CREB1	AL022067.1	0.715784208	3.67E-85	postive
UBR1	AL022067.1	0.565634157	1.44E-46	postive
ZC3HAV1	AL022067.1	0.532856713	1.43E-40	postive
IL15	AL022067.1	0.625241989	2.27E-59	postive
CYLD	AL022067.1	0.582810657	5.47E-50	postive
EIF2AK2	AL022067.1	0.539045169	1.18E-41	postive
MAPK8	AL022067.1	0.533402442	1.15E-40	postive
LMBR1	AL022067.1	0.547394609	3.77E-43	postive
LIMS1	AL022067.1	0.518937527	3.22E-38	postive
IREB2	AL022067.1	0.571838393	8.82E-48	postive
DDX17	AL022067.1	0.536761528	2.98E-41	postive
PIK3CG	AL022067.1	0.575068671	2.02E-48	postive
JAK2	AL022067.1	0.619352665	5.55E-58	postive
TXK	AL022067.1	0.589813985	1.92E-51	postive
NFAT5	AL022067.1	0.700540767	3.87E-80	postive
MALT1	AL022067.1	0.539628723	9.31E-42	postive
PIK3R1	AL022067.1	0.673019657	7.81E-72	postive
PIK3CA	AL022067.1	0.679340012	1.16E-73	postive
PIK3CB	AL022067.1	0.512725285	3.34E-37	postive
AKT3	AL022067.1	0.536977713	2.73E-41	postive
RASGRP3	AL022067.1	0.575299575	1.81E-48	postive
IGHD6-6	AL022067.1	0.556171196	9.07E-45	postive
PLXNC1	AL022067.1	0.533197817	1.25E-40	postive
GNRH1	AL022067.1	0.59808774	3.32E-53	postive
IL6ST	AL022067.1	0.649505196	2.03E-65	postive
ACVR2A	AL022067.1	0.574323843	2.84E-48	postive
ANGPTL1	AL022067.1	0.782525351	9.22E-112	postive
BMPR2	AL022067.1	0.711378323	1.12E-83	postive
CRLF3	AL022067.1	0.595039809	1.50E-52	postive
NR2C2	AL022067.1	0.620707286	2.67E-58	postive
RORA	AL022067.1	0.813948114	8.10E-128	postive
SOS1	AL022067.1	0.647020797	8.95E-65	postive
SOS2	AL022067.1	0.543041071	2.30E-42	postive
BRAF	AL022067.1	0.791082645	7.38E-116	postive
ITK	AL022067.1	0.631870978	5.71E-61	postive
CD28	AL022067.1	0.541482808	4.36E-42	postive
CBL	AL022067.1	0.564340654	2.55E-46	postive
CBLB	AL022067.1	0.728445544	1.36E-89	postive
RASGRP1	AL022067.1	0.510922812	6.53E-37	postive
PDK1	AL022067.1	0.625514929	1.95E-59	postive
TRAJ1	AL022067.1	0.630265487	1.40E-60	postive
TRAJ2	AL022067.1	0.694072898	4.20E-78	postive
TRAJ3	AL022067.1	0.732187239	5.96E-91	postive
TRAJ5	AL022067.1	0.742044048	1.22E-94	postive
TRAJ6	AL022067.1	0.67966263	9.36E-74	postive
TRAJ8	AL022067.1	0.703847322	3.35E-81	postive
TRAJ10	AL022067.1	0.659046644	5.94E-68	postive
TRAJ12	AL022067.1	0.635683723	6.59E-62	postive
TRAJ13	AL022067.1	0.703436111	4.55E-81	postive
TRAJ14	AL022067.1	0.710698172	1.89E-83	postive
TRAJ16	AL022067.1	0.712921818	3.41E-84	postive
TRAJ17	AL022067.1	0.70144324	1.99E-80	postive
TRAJ18	AL022067.1	0.666683987	4.77E-70	postive
TRAJ21	AL022067.1	0.747614218	8.44E-97	postive
TRAJ31	AL022067.1	0.760412697	5.50E-102	postive
TRAJ37	AL022067.1	0.712472862	4.82E-84	postive
TRAJ38	AL022067.1	0.798365283	1.69E-119	postive
TRAJ39	AL022067.1	0.768357547	2.26E-105	postive
GIPR	AC234582.1	0.58790121	4.84E-51	postive
CREB1	AC025171.4	0.537625681	2.10E-41	postive
RFXAP	AC025171.4	0.526317844	1.88E-39	postive

DDX17	AC025171.4	0.634607891	1.22E-61	postive
TXK	AC025171.4	0.505386161	4.98E-36	postive
NFAT5	AC025171.4	0.635164545	8.86E-62	postive
PIK3R1	AC025171.4	0.544064542	1.51E-42	postive
IGHD4-4	AC025171.4	0.501629217	1.94E-35	postive
IGHD6-6	AC025171.4	0.504478473	6.93E-36	postive
LTB4R2	AC025171.4	0.522665719	7.73E-39	postive
GNRH1	AC025171.4	0.687961008	3.15E-76	postive
IL6ST	AC025171.4	0.610209273	6.95E-56	postive
ACVR2A	AC025171.4	0.513206744	2.79E-37	postive
ANGPTL1	AC025171.4	0.535729968	4.52E-41	postive
BMPR2	AC025171.4	0.540901789	5.54E-42	postive
NR2C2	AC025171.4	0.697054195	4.92E-79	postive
RORA	AC025171.4	0.682701552	1.19E-74	postive
BRAF	AC025171.4	0.668273381	1.72E-70	postive
CBLB	AC025171.4	0.514531644	1.70E-37	postive
TRAJ1	AC025171.4	0.507365836	2.42E-36	postive
TRAJ2	AC025171.4	0.525084506	3.03E-39	postive
TRAJ3	AC025171.4	0.606118472	5.74E-55	postive
TRAJ5	AC025171.4	0.572184398	7.54E-48	postive
TRAJ6	AC025171.4	0.531430303	2.52E-40	postive
TRAJ8	AC025171.4	0.535777277	4.43E-41	postive
TRAJ10	AC025171.4	0.528329316	8.55E-40	postive
TRAJ12	AC025171.4	0.509138718	1.26E-36	postive
TRAJ13	AC025171.4	0.522645082	7.79E-39	postive
TRAJ14	AC025171.4	0.518614616	3.64E-38	postive
TRAJ16	AC025171.4	0.543160562	2.19E-42	postive
TRAJ17	AC025171.4	0.53900386	1.20E-41	postive
TRAJ18	AC025171.4	0.536680149	3.08E-41	postive
TRAJ21	AC025171.4	0.568380979	4.21E-47	postive
TRAJ31	AC025171.4	0.590115597	1.66E-51	postive
TRAJ37	AC025171.4	0.591468286	8.63E-52	postive
TRAJ38	AC025171.4	0.598078567	3.33E-53	postive
TRAJ39	AC025171.4	0.614816976	6.22E-57	postive
RFXAP	AL603839.3	0.534735329	6.73E-41	postive
NFAT5	AL603839.3	0.562343535	6.17E-46	postive
GNRH1	AL603839.3	0.555041269	1.47E-44	postive
NR2C2	AL603839.3	0.537276673	2.42E-41	postive
BRAF	AL603839.3	0.568901285	3.33E-47	postive
TRAJ2	AL603839.3	0.52720579	1.33E-39	postive
TRAJ3	AL603839.3	0.537172239	2.52E-41	postive
TRAJ6	AL603839.3	0.516805846	7.23E-38	postive
TRAJ8	AL603839.3	0.507439612	2.36E-36	postive
TRAJ37	AL603839.3	0.536404198	3.44E-41	postive
TRAJ38	AL603839.3	0.532354497	1.74E-40	postive
TRAJ39	AL603839.3	0.523932855	4.74E-39	postive
DDX17	MIR3936HG	0.565047704	1.87E-46	postive
LTB4R2	MIR3936HG	0.550033296	1.24E-43	postive
GNRH1	MIR3936HG	0.566185566	1.12E-46	postive
NR2C2	MIR3936HG	0.571386165	1.08E-47	postive
DDX17	AL031775.1	0.514798481	1.54E-37	postive
NFAT5	AL031775.1	0.510822637	6.78E-37	postive
GNRH1	AL031775.1	0.575413781	1.72E-48	postive
NR2C2	AL031775.1	0.506808331	2.97E-36	postive
RORA	AL031775.1	0.535985837	4.07E-41	postive
BRAF	AL031775.1	0.533224265	1.23E-40	postive
TRAJ1	AL031775.1	0.505595374	4.62E-36	postive
TRAJ5	AL031775.1	0.546411836	5.68E-43	postive
TRAJ8	AL031775.1	0.527264122	1.30E-39	postive
TRAJ13	AL031775.1	0.528414295	8.27E-40	postive
TRAJ14	AL031775.1	0.51558994	1.14E-37	postive
TRAJ16	AL031775.1	0.509099938	1.28E-36	postive
TRAJ17	AL031775.1	0.505827163	4.24E-36	postive
TRAJ21	AL031775.1	0.533206886	1.24E-40	postive
TRAJ31	AL031775.1	0.572573698	6.31E-48	postive

TRAJ37	AL031775.1	0.529378905	5.66E-40	postive
TRAJ38	AL031775.1	0.527043796	1.41E-39	postive
TRAJ39	AL031775.1	0.553601817	2.73E-44	postive
MAPK8	AC004241.3	0.53307597	1.31E-40	postive
EED	AC004241.3	0.502414845	1.46E-35	postive
NFAT5	AC004241.3	0.523313125	6.02E-39	postive
AKT3	AC004241.3	0.508560499	1.56E-36	postive
ANGPTL1	AC004241.3	0.517602203	5.35E-38	postive
BRAF	AC004241.3	0.532560624	1.61E-40	postive
PDK1	AC004241.3	0.528070534	9.46E-40	postive
TRAJ1	AC004241.3	0.534890449	6.33E-41	postive
TRAJ2	AC004241.3	0.548197891	2.69E-43	postive
TRAJ3	AC004241.3	0.558415288	3.44E-45	postive
TRAJ5	AC004241.3	0.542952744	2.38E-42	postive
TRAJ6	AC004241.3	0.596682853	6.66E-53	postive
TRAJ8	AC004241.3	0.607729883	2.51E-55	postive
TRAJ10	AC004241.3	0.539605638	9.40E-42	postive
TRAJ12	AC004241.3	0.508014098	1.91E-36	postive
TRAJ13	AC004241.3	0.591242801	9.63E-52	postive
TRAJ14	AC004241.3	0.592233106	5.94E-52	postive
TRAJ16	AC004241.3	0.575862532	1.40E-48	postive
TRAJ17	AC004241.3	0.54511053	9.76E-43	postive
TRAJ21	AC004241.3	0.589928909	1.82E-51	postive
TRAJ31	AC004241.3	0.581343486	1.09E-49	postive
TRAJ37	AC004241.3	0.557253788	5.68E-45	postive
TRAJ38	AC004241.3	0.610525568	5.90E-56	postive
TRAJ39	AC004241.3	0.587953511	4.72E-51	postive
CREB1	C1RL-AS1	0.583820557	3.39E-50	postive
UBR1	C1RL-AS1	0.558958933	2.71E-45	postive
ZC3HAV1L	C1RL-AS1	0.523311055	6.03E-39	postive
IL15	C1RL-AS1	0.50496567	5.81E-36	postive
CYLD	C1RL-AS1	0.544345395	1.34E-42	postive
NFKBIZ	C1RL-AS1	0.51238517	3.79E-37	postive
SP1	C1RL-AS1	0.501384189	2.12E-35	postive
IREB2	C1RL-AS1	0.52032361	1.90E-38	postive
DDX17	C1RL-AS1	0.681957325	1.98E-74	postive
JAK2	C1RL-AS1	0.525912136	2.20E-39	postive
NFAT5	C1RL-AS1	0.682034719	1.87E-74	postive
PIK3CA	C1RL-AS1	0.529742631	4.90E-40	postive
LTB4R2	C1RL-AS1	0.560490574	1.39E-45	postive
GNRH1	C1RL-AS1	0.730289826	2.93E-90	postive
ANGPTL1	C1RL-AS1	0.559343431	2.29E-45	postive
BMPR2	C1RL-AS1	0.506950667	2.82E-36	postive
NR2C1	C1RL-AS1	0.53996897	8.11E-42	postive
NR2C2	C1RL-AS1	0.713079187	3.02E-84	postive
RORA	C1RL-AS1	0.643538191	7.01E-64	postive
SOS1	C1RL-AS1	0.572138536	7.70E-48	postive
BRAF	C1RL-AS1	0.579231145	2.93E-49	postive
CBL	C1RL-AS1	0.542966545	2.37E-42	postive
CBLB	C1RL-AS1	0.55133786	7.16E-44	postive
TRAJ1	C1RL-AS1	0.602963902	2.86E-54	postive
TRAJ2	C1RL-AS1	0.575124743	1.96E-48	postive
TRAJ3	C1RL-AS1	0.656038009	3.83E-67	postive
TRAJ5	C1RL-AS1	0.554252997	2.07E-44	postive
TRAJ6	C1RL-AS1	0.601665728	5.52E-54	postive
TRAJ8	C1RL-AS1	0.5389343	1.24E-41	postive
TRAJ10	C1RL-AS1	0.595044777	1.50E-52	postive
TRAJ12	C1RL-AS1	0.509092289	1.28E-36	postive
TRAJ13	C1RL-AS1	0.536897433	2.82E-41	postive
TRAJ14	C1RL-AS1	0.533251484	1.22E-40	postive
TRAJ16	C1RL-AS1	0.585089685	1.86E-50	postive
TRAJ17	C1RL-AS1	0.561523965	8.84E-46	postive
TRAJ18	C1RL-AS1	0.562913802	4.80E-46	postive
TRAJ21	C1RL-AS1	0.568570732	3.87E-47	postive
TRAJ31	C1RL-AS1	0.567687405	5.75E-47	postive

TRAJ37	C1RL-AS1	0.573819803	3.57E-48	postive
TRAJ38	C1RL-AS1	0.614610674	6.93E-57	postive
TRAJ39	C1RL-AS1	0.593241221	3.63E-52	postive
IGHA1	AC012236.1	0.531756037	2.21E-40	postive
IGHG1	AC012236.1	0.549609181	1.49E-43	postive
IGHG3	AC012236.1	0.534551218	7.25E-41	postive
IGHV1-2	AC012236.1	0.533443983	1.13E-40	postive
IGHV3-11	AC012236.1	0.501673251	1.91E-35	postive
IGHV3-48	AC012236.1	0.558087417	3.96E-45	postive
IGKC	AC012236.1	0.601975261	4.72E-54	postive
IGKJ5	AC012236.1	0.538134786	1.71E-41	postive
IGLC2	AC012236.1	0.590361101	1.48E-51	postive
IGLC3	AC012236.1	0.588325618	3.95E-51	postive
IGLJ1	AC012236.1	0.50172032	1.88E-35	postive
IGLV1-40	AC012236.1	0.539764318	8.81E-42	postive
IGLV6-57	AC012236.1	0.513498562	2.50E-37	postive
PNOC	AC012236.1	0.502432349	1.45E-35	postive
TNFRSF17	AC012236.1	0.529747037	4.89E-40	postive
IRF9	AL109811.2	0.503930558	8.45E-36	postive
IL11RA	AL109811.2	0.578012195	5.17E-49	postive
AGER	FENDRR	0.77706297	3.05E-109	postive
SEMA3G	FENDRR	0.682977696	9.85E-75	postive
EDNRB	FENDRR	0.754985212	9.52E-100	postive
VEGFD	FENDRR	0.732343	5.23E-91	postive
GDF10	FENDRR	0.545725919	7.56E-43	postive
ACVRL1	FENDRR	0.604100374	1.61E-54	postive
CALCRL	FENDRR	0.627193327	7.74E-60	postive
S1PR1	FENDRR	0.64768775	6.02E-65	postive
TEK	FENDRR	0.729058991	8.17E-90	postive
VIPR1	FENDRR	0.705235233	1.19E-81	postive
RFXAP	AL513534.3	0.50159319	1.96E-35	postive
MAPK8	AL513534.3	0.526539224	1.72E-39	postive
IREB2	AL513534.3	0.502171123	1.59E-35	postive
TXK	AL513534.3	0.523392217	5.84E-39	postive
ACVR2B	AL513534.3	0.531434735	2.51E-40	postive
BMPR1A	AL513534.3	0.534375217	7.78E-41	postive
SOS1	AL513534.3	0.518389809	3.97E-38	postive
BRAF	AL513534.3	0.508894905	1.38E-36	postive
SEMA3G	AC026369.2	0.556357244	8.37E-45	postive
EDNRB	AC026369.2	0.509970607	9.29E-37	postive
ACVRL1	AC026369.2	0.510409029	7.90E-37	postive
EREG	LINC01214	0.530083359	4.29E-40	postive
FABP7	LINC02617	0.717708389	8.07E-86	postive
HCK	LINC02617	0.534044616	8.88E-41	postive
GNRH1	NAALADL2-AS2	0.530391017	3.80E-40	postive
TEC	NAALADL2-AS2	0.640608493	3.87E-63	postive
CREB1	LINC01004	0.525681689	2.41E-39	postive
RFXAP	LINC01004	0.524109914	4.43E-39	postive
DDX17	LINC01004	0.633308237	2.54E-61	postive
NFAT5	LINC01004	0.654255938	1.14E-66	postive
GNRH1	LINC01004	0.710828007	1.71E-83	postive
ACVR2A	LINC01004	0.521405961	1.26E-38	postive
NR2C1	LINC01004	0.571971169	8.31E-48	postive
NR2C2	LINC01004	0.603777825	1.90E-54	postive
RORA	LINC01004	0.606516871	4.68E-55	postive
BRAF	LINC01004	0.640705436	3.66E-63	postive
TRAJ1	LINC01004	0.593061125	3.97E-52	postive
TRAJ2	LINC01004	0.617409454	1.57E-57	postive
TRAJ3	LINC01004	0.635868903	5.93E-62	postive
TRAJ5	LINC01004	0.554434907	1.91E-44	postive
TRAJ6	LINC01004	0.612525309	2.08E-56	postive
TRAJ8	LINC01004	0.629518116	2.13E-60	postive
TRAJ10	LINC01004	0.575332608	1.78E-48	postive
TRAJ12	LINC01004	0.536316774	3.57E-41	postive
TRAJ13	LINC01004	0.604601132	1.25E-54	postive

TRAJ14	LINC01004	0.590999093	1.08E-51	postive
TRAJ16	LINC01004	0.586730327	8.49E-51	postive
TRAJ17	LINC01004	0.556872973	6.70E-45	postive
TRAJ21	LINC01004	0.599111369	1.99E-53	postive
TRAJ31	LINC01004	0.598588627	2.58E-53	postive
TRAJ37	LINC01004	0.624885161	2.76E-59	postive
TRAJ38	LINC01004	0.625240771	2.27E-59	postive
TRAJ39	LINC01004	0.611594007	3.38E-56	postive
CD1D	AC145098.1	0.520503043	1.77E-38	postive
CD4	AC145098.1	0.639201917	8.75E-63	postive
FCER1G	AC145098.1	0.529237887	5.98E-40	postive
LTA	AC145098.1	0.525334359	2.75E-39	postive
CIITA	AC145098.1	0.545443427	8.50E-43	postive
IFI30	AC145098.1	0.525399525	2.69E-39	postive
TLR4	AC145098.1	0.53624621	3.67E-41	postive
NOD2	AC145098.1	0.513118637	2.89E-37	postive
CYBB	AC145098.1	0.586252723	1.07E-50	postive
TLR7	AC145098.1	0.542040338	3.47E-42	postive
TLR8	AC145098.1	0.534527154	7.32E-41	postive
CCR5	AC145098.1	0.62719732	7.72E-60	postive
CCR1	AC145098.1	0.515210659	1.32E-37	postive
FGR	AC145098.1	0.597965511	3.52E-53	postive
CD86	AC145098.1	0.568526929	3.94E-47	postive
BTK	AC145098.1	0.684409927	3.69E-75	postive
VAV1	AC145098.1	0.510087689	8.89E-37	postive
PIK3R5	AC145098.1	0.693625266	5.79E-78	postive
PIK3CD	AC145098.1	0.570016382	2.01E-47	postive
INPP5D	AC145098.1	0.595381435	1.27E-52	postive
CD72	AC145098.1	0.568347491	4.27E-47	postive
PTPN6	AC145098.1	0.52339481	5.84E-39	postive
LILRB3	AC145098.1	0.606003507	6.09E-55	postive
RASGRP3	AC145098.1	0.547340628	3.85E-43	postive
PLCG2	AC145098.1	0.608010092	2.17E-55	postive
PRKCB	AC145098.1	0.540218677	7.32E-42	postive
SEMA4D	AC145098.1	0.609764318	8.76E-56	postive
CMKLR1	AC145098.1	0.635970491	5.60E-62	postive
PLXNC1	AC145098.1	0.517517734	5.52E-38	postive
PTAFR	AC145098.1	0.506910795	2.86E-36	postive
ADA2	AC145098.1	0.579162446	3.03E-49	postive
IL16	AC145098.1	0.629886629	1.73E-60	postive
C3AR1	AC145098.1	0.557435957	5.25E-45	postive
CSF1R	AC145098.1	0.541635577	4.10E-42	postive
CSF2RB	AC145098.1	0.739299069	1.35E-93	postive
IL10RA	AC145098.1	0.740052594	7.00E-94	postive
IL12RB1	AC145098.1	0.675813562	1.23E-72	postive
IL2RB	AC145098.1	0.531903331	2.08E-40	postive
IL17RA	AC145098.1	0.516394654	8.44E-38	postive
IL21R	AC145098.1	0.645003677	2.96E-64	postive
ITGAL	AC145098.1	0.672647295	9.97E-72	postive
ITGB2	AC145098.1	0.550981264	8.33E-44	postive
FCGR3A	AC145098.1	0.545525753	8.22E-43	postive
CD247	AC145098.1	0.54733702	3.86E-43	postive
ZAP70	AC145098.1	0.549880094	1.33E-43	postive
LCP2	AC145098.1	0.691879682	2.00E-77	postive
CD3E	AC145098.1	0.51119151	5.91E-37	postive
PTPRC	AC145098.1	0.604651932	1.21E-54	postive
CD28	AC145098.1	0.511458743	5.35E-37	postive
TRAC	AC145098.1	0.502987828	1.19E-35	postive
TRAV8-4	AC145098.1	0.505456965	4.86E-36	postive
TRBC1	AC145098.1	0.58534006	1.65E-50	postive
TRBJ2-7	AC145098.1	0.541186298	4.93E-42	postive
TRBV2	AC145098.1	0.501972831	1.71E-35	postive
CREB1	AL137779.2	0.723321123	9.05E-88	postive
UBR1	AL137779.2	0.579308643	2.83E-49	postive
ZC3HAV1	AL137779.2	0.521490946	1.21E-38	postive

IL15	AL137779.2	0.639374275	7.92E-63	postive
CYLD	AL137779.2	0.560110796	1.64E-45	postive
EIF2AK2	AL137779.2	0.523737019	5.11E-39	postive
LMBR1	AL137779.2	0.557391705	5.35E-45	postive
IREB2	AL137779.2	0.553279353	3.13E-44	postive
DDX17	AL137779.2	0.532413052	1.70E-40	postive
PIK3CG	AL137779.2	0.524697671	3.53E-39	postive
JAK2	AL137779.2	0.593753071	2.83E-52	postive
TXK	AL137779.2	0.560174804	1.60E-45	postive
NFAT5	AL137779.2	0.72280041	1.38E-87	postive
MALT1	AL137779.2	0.501125739	2.32E-35	postive
PIK3R1	AL137779.2	0.628612768	3.52E-60	postive
PIK3CA	AL137779.2	0.652740258	2.87E-66	postive
PIK3CB	AL137779.2	0.532431904	1.69E-40	postive
RASGRP3	AL137779.2	0.510005773	9.17E-37	postive
IGHD4-4	AL137779.2	0.544256462	1.39E-42	postive
PLXNC1	AL137779.2	0.50544006	4.89E-36	postive
GNRH1	AL137779.2	0.661317765	1.44E-68	postive
IL6ST	AL137779.2	0.563695472	3.40E-46	postive
IL7	AL137779.2	0.509145511	1.26E-36	postive
RABEP1	AL137779.2	0.505836721	4.23E-36	postive
ACVR2A	AL137779.2	0.603427067	2.26E-54	postive
ANGPTL1	AL137779.2	0.767990028	3.26E-105	postive
BMPR2	AL137779.2	0.670309847	4.59E-71	postive
CRLF3	AL137779.2	0.576926521	8.55E-49	postive
NR2C2	AL137779.2	0.636526942	4.07E-62	postive
RORA	AL137779.2	0.747484233	9.49E-97	postive
SOS1	AL137779.2	0.620192135	3.53E-58	postive
SOS2	AL137779.2	0.586661246	8.77E-51	postive
BRAF	AL137779.2	0.73391581	1.38E-91	postive
ITK	AL137779.2	0.519615927	2.49E-38	postive
CBL	AL137779.2	0.567263288	6.95E-47	postive
CBLB	AL137779.2	0.64492146	3.10E-64	postive
PDK1	AL137779.2	0.58881315	3.12E-51	postive
TRAJ1	AL137779.2	0.667443126	2.93E-70	postive
TRAJ2	AL137779.2	0.600476566	1.00E-53	postive
TRAJ3	AL137779.2	0.737405744	6.97E-93	postive
TRAJ5	AL137779.2	0.751369074	2.74E-98	postive
TRAJ6	AL137779.2	0.712675594	4.12E-84	postive
TRAJ8	AL137779.2	0.689639727	9.73E-77	postive
TRAJ10	AL137779.2	0.722543703	1.70E-87	postive
TRAJ12	AL137779.2	0.665426374	1.07E-69	postive
TRAJ13	AL137779.2	0.705369264	1.07E-81	postive
TRAJ14	AL137779.2	0.701853393	1.47E-80	postive
TRAJ16	AL137779.2	0.696186678	9.21E-79	postive
TRAJ17	AL137779.2	0.732466768	4.71E-91	postive
TRAJ18	AL137779.2	0.679020185	1.44E-73	postive
TRAJ21	AL137779.2	0.749044023	2.30E-97	postive
TRAJ31	AL137779.2	0.756809113	1.71E-100	postive
TRAJ37	AL137779.2	0.718285736	5.11E-86	postive
TRAJ38	AL137779.2	0.765232428	5.03E-104	postive
TRAJ39	AL137779.2	0.769126702	1.04E-105	postive
CREB1	LINC00894	0.672546155	1.07E-71	postive
RFXAP	LINC00894	0.524231767	4.22E-39	postive
UBR1	LINC00894	0.585894965	1.27E-50	postive
IL15	LINC00894	0.519772909	2.34E-38	postive
CYLD	LINC00894	0.589748769	1.99E-51	postive
EIF2AK2	LINC00894	0.500954236	2.47E-35	postive
NFKBIZ	LINC00894	0.501255846	2.22E-35	postive
IREB2	LINC00894	0.509417946	1.14E-36	postive
DDX17	LINC00894	0.689839552	8.46E-77	postive
JAK2	LINC00894	0.579692653	2.36E-49	postive
NFAT5	LINC00894	0.799191542	6.38E-120	postive
PIK3R1	LINC00894	0.505333624	5.08E-36	postive
PIK3CA	LINC00894	0.557865293	4.36E-45	postive

LTB4R2	LINC00894	0.559172694	2.47E-45	postive
GNRH1	LINC00894	0.775161952	2.21E-108	postive
ACVR2A	LINC00894	0.598302048	2.98E-53	postive
ANGPTL1	LINC00894	0.602706536	3.26E-54	postive
BMPR2	LINC00894	0.562178353	6.63E-46	postive
CRLF3	LINC00894	0.563114506	4.39E-46	postive
NR2C1	LINC00894	0.535901212	4.21E-41	postive
NR2C2	LINC00894	0.763891444	1.88E-103	postive
RORA	LINC00894	0.718303381	5.04E-86	postive
SOS1	LINC00894	0.631881282	5.67E-61	postive
SOS2	LINC00894	0.524877745	3.29E-39	postive
BRAF	LINC00894	0.701917874	1.40E-80	postive
CBL	LINC00894	0.516392848	8.45E-38	postive
CBLB	LINC00894	0.598436888	2.79E-53	postive
TRAJ1	LINC00894	0.796817129	1.03E-118	postive
TRAJ2	LINC00894	0.703605105	4.01E-81	postive
TRAJ3	LINC00894	0.783345447	3.80E-112	postive
TRAJ5	LINC00894	0.781402425	3.08E-111	postive
TRAJ6	LINC00894	0.802884678	7.79E-122	postive
TRAJ8	LINC00894	0.773482085	1.25E-107	postive
TRAJ10	LINC00894	0.785013381	6.21E-113	postive
TRAJ12	LINC00894	0.780459961	8.43E-111	postive
TRAJ13	LINC00894	0.766804461	1.06E-104	postive
TRAJ14	LINC00894	0.780598798	7.27E-111	postive
TRAJ16	LINC00894	0.797803485	3.26E-119	postive
TRAJ17	LINC00894	0.765859219	2.71E-104	postive
TRAJ18	LINC00894	0.74789705	6.53E-97	postive
TRAJ21	LINC00894	0.787160261	5.88E-114	postive
TRAJ31	LINC00894	0.800958907	7.84E-121	postive
TRAJ37	LINC00894	0.791267008	6.00E-116	postive
TRAJ38	LINC00894	0.825434999	1.81E-134	postive
TRAJ39	LINC00894	0.795641787	4.04E-118	postive
PLCG1	ZNF528-AS1	0.503819188	8.80E-36	postive
GAL	UBL7-AS1	0.858703688	7.22E-157	postive
CREB1	AL157838.1	0.611312183	3.92E-56	postive
ZC3HAV1L	AL157838.1	0.534028842	8.94E-41	postive
NFKBIZ	AL157838.1	0.56382348	3.21E-46	postive
LMBR1	AL157838.1	0.509989879	9.22E-37	postive
NFAT5	AL157838.1	0.588727311	3.25E-51	postive
PIK3CA	AL157838.1	0.551077978	7.99E-44	postive
GNRH1	AL157838.1	0.611417239	3.71E-56	postive
ANGPTL1	AL157838.1	0.605344851	8.53E-55	postive
BMPR2	AL157838.1	0.517204293	6.22E-38	postive
NR2C2	AL157838.1	0.591757	7.49E-52	postive
RORA	AL157838.1	0.564300313	2.60E-46	postive
SOS1	AL157838.1	0.571181395	1.19E-47	postive
BRAF	AL157838.1	0.605329528	8.60E-55	postive
CBL	AL157838.1	0.510672591	7.16E-37	postive
CBLB	AL157838.1	0.552873413	3.73E-44	postive
TRAJ1	AL157838.1	0.546289595	5.98E-43	postive
TRAJ3	AL157838.1	0.571016475	1.28E-47	postive
TRAJ5	AL157838.1	0.61178517	3.06E-56	postive
TRAJ6	AL157838.1	0.566967296	7.93E-47	postive
TRAJ8	AL157838.1	0.535148828	5.70E-41	postive
TRAJ10	AL157838.1	0.587179132	6.84E-51	postive
TRAJ13	AL157838.1	0.56126979	9.88E-46	postive
TRAJ14	AL157838.1	0.551126442	7.83E-44	postive
TRAJ16	AL157838.1	0.565946461	1.25E-46	postive
TRAJ17	AL157838.1	0.575078702	2.01E-48	postive
TRAJ18	AL157838.1	0.51574533	1.08E-37	postive
TRAJ21	AL157838.1	0.587296365	6.47E-51	postive
TRAJ31	AL157838.1	0.595245092	1.36E-52	postive
TRAJ37	AL157838.1	0.549914591	1.31E-43	postive
TRAJ38	AL157838.1	0.610721522	5.33E-56	postive
TRAJ39	AL157838.1	0.598788755	2.34E-53	postive

CREB1	AL049840.2	0.796703088	1.18E-118	postive
RFXAP	AL049840.2	0.553643124	2.68E-44	postive
UBR1	AL049840.2	0.670435596	4.23E-71	postive
ZC3HAV1	AL049840.2	0.605875338	6.50E-55	postive
ZC3HAV1L	AL049840.2	0.560303109	1.51E-45	postive
IL15	AL049840.2	0.596458051	7.45E-53	postive
CYLD	AL049840.2	0.632364019	4.32E-61	postive
EIF2AK2	AL049840.2	0.54756688	3.51E-43	postive
MAPK8	AL049840.2	0.576512605	1.04E-48	postive
LMBR1	AL049840.2	0.585372758	1.62E-50	postive
SP1	AL049840.2	0.500145425	3.30E-35	postive
IREB2	AL049840.2	0.617433228	1.55E-57	postive
DDX17	AL049840.2	0.702191548	1.15E-80	postive
PIK3CG	AL049840.2	0.545410067	8.62E-43	postive
JAK2	AL049840.2	0.649696811	1.81E-65	postive
TXK	AL049840.2	0.63449833	1.29E-61	postive
NFAT5	AL049840.2	0.860154032	5.68E-158	postive
MALT1	AL049840.2	0.5685188	3.96E-47	postive
PIK3R1	AL049840.2	0.647225692	7.92E-65	postive
PIK3CA	AL049840.2	0.691965715	1.89E-77	postive
PIK3CB	AL049840.2	0.540041088	7.87E-42	postive
AKT3	AL049840.2	0.566852386	8.35E-47	postive
GSK3B	AL049840.2	0.505994575	3.99E-36	postive
RASGRP3	AL049840.2	0.532934463	1.38E-40	postive
IGHD4-4	AL049840.2	0.527661325	1.11E-39	postive
IGHD6-6	AL049840.2	0.56068913	1.27E-45	postive
LTB4R2	AL049840.2	0.53017209	4.14E-40	postive
GNRH1	AL049840.2	0.83508769	1.90E-140	postive
IL6ST	AL049840.2	0.604816367	1.12E-54	postive
RABEP1	AL049840.2	0.605718513	7.05E-55	postive
ACVR2A	AL049840.2	0.661926987	9.79E-69	postive
ANGPTL1	AL049840.2	0.784696056	8.78E-113	postive
BMPR1A	AL049840.2	0.543474788	1.92E-42	postive
BMPR2	AL049840.2	0.730240892	3.05E-90	postive
CRLF3	AL049840.2	0.639879089	5.91E-63	postive
NR1D2	AL049840.2	0.507389115	2.40E-36	postive
NR2C1	AL049840.2	0.566944329	8.01E-47	postive
NR2C2	AL049840.2	0.768133247	2.83E-105	postive
RORA	AL049840.2	0.837788344	3.44E-142	postive
SOS1	AL049840.2	0.702609205	8.41E-81	postive
SOS2	AL049840.2	0.617938549	1.18E-57	postive
BRAF	AL049840.2	0.815880246	6.64E-129	postive
ITK	AL049840.2	0.54488233	1.07E-42	postive
TEC	AL049840.2	0.50985279	9.70E-37	postive
CBL	AL049840.2	0.667488157	2.85E-70	postive
CBLB	AL049840.2	0.717534535	9.25E-86	postive
RASGRP1	AL049840.2	0.519314885	2.79E-38	postive
PDK1	AL049840.2	0.612643073	1.95E-56	postive
TRAJ1	AL049840.2	0.743772045	2.64E-95	postive
TRAJ2	AL049840.2	0.729760371	4.56E-90	postive
TRAJ3	AL049840.2	0.811279165	2.44E-126	postive
TRAJ5	AL049840.2	0.773074504	1.90E-107	postive
TRAJ6	AL049840.2	0.789275241	5.61E-115	postive
TRAJ8	AL049840.2	0.749279481	1.86E-97	postive
TRAJ10	AL049840.2	0.77194461	6.02E-107	postive
TRAJ12	AL049840.2	0.692183072	1.62E-77	postive
TRAJ13	AL049840.2	0.771458497	9.88E-107	postive
TRAJ14	AL049840.2	0.748426212	4.04E-97	postive
TRAJ16	AL049840.2	0.765095161	5.76E-104	postive
TRAJ17	AL049840.2	0.746446043	2.42E-96	postive
TRAJ18	AL049840.2	0.724942861	2.42E-88	postive
TRAJ21	AL049840.2	0.80172455	3.14E-121	postive
TRAJ31	AL049840.2	0.801599712	3.65E-121	postive
TRAJ37	AL049840.2	0.790480899	1.45E-115	postive
TRAJ38	AL049840.2	0.831530837	3.36E-138	postive

TRAJ39	AL049840.2	0.813305384	1.85E-127	postive
CYLD	AL121584.1	0.513796498	2.24E-37	postive
NFAT5	AL121584.1	0.774783455	3.26E-108	postive
NFATC3	AL121584.1	0.71527356	5.47E-85	postive
GNRH1	AL121584.1	0.527145339	1.36E-39	postive
RORA	AL121584.1	0.584431859	2.54E-50	postive
TRAJ1	AL121584.1	0.646139957	1.51E-64	postive
TRAJ2	AL121584.1	0.606700834	4.26E-55	postive
TRAJ3	AL121584.1	0.635830559	6.06E-62	postive
TRAJ5	AL121584.1	0.543861972	1.64E-42	postive
TRAJ6	AL121584.1	0.705427314	1.03E-81	postive
TRAJ8	AL121584.1	0.590689398	1.26E-51	postive
TRAJ10	AL121584.1	0.708237822	1.24E-82	postive
TRAJ12	AL121584.1	0.660442809	2.49E-68	postive
TRAJ13	AL121584.1	0.610020239	7.67E-56	postive
TRAJ14	AL121584.1	0.592643273	4.86E-52	postive
TRAJ16	AL121584.1	0.656487495	2.90E-67	postive
TRAJ17	AL121584.1	0.594987853	1.54E-52	postive
TRAJ18	AL121584.1	0.698090356	2.32E-79	postive
TRAJ21	AL121584.1	0.628658317	3.44E-60	postive
TRAJ31	AL121584.1	0.635071548	9.34E-62	postive
TRAJ37	AL121584.1	0.747202739	1.22E-96	postive
TRAJ38	AL121584.1	0.631935926	5.50E-61	postive
TRAJ39	AL121584.1	0.652263465	3.84E-66	postive
CREB1	WARS2-AS1	0.57604826	1.28E-48	postive
UBR1	WARS2-AS1	0.549334635	1.67E-43	postive
ZC3HAV1	WARS2-AS1	0.50362222	9.45E-36	postive
EIF2AK2	WARS2-AS1	0.507647093	2.18E-36	postive
DDX17	WARS2-AS1	0.551503813	6.67E-44	postive
NFAT5	WARS2-AS1	0.580773439	1.43E-49	postive
PIK3CA	WARS2-AS1	0.511661429	4.96E-37	postive
LTB4R2	WARS2-AS1	0.509983784	9.24E-37	postive
GNRH1	WARS2-AS1	0.603954677	1.73E-54	postive
ANGPTL1	WARS2-AS1	0.549246031	1.73E-43	postive
BMPR2	WARS2-AS1	0.522039918	9.84E-39	postive
NR2C2	WARS2-AS1	0.645179002	2.67E-64	postive
RORA	WARS2-AS1	0.579596671	2.47E-49	postive
SOS1	WARS2-AS1	0.546588839	5.28E-43	postive
BRAF	WARS2-AS1	0.66968523	6.89E-71	postive
CBLB	WARS2-AS1	0.56725373	6.98E-47	postive
TRAJ2	WARS2-AS1	0.518361681	4.01E-38	postive
TRAJ3	WARS2-AS1	0.522832467	7.25E-39	postive
TRAJ16	WARS2-AS1	0.503146182	1.12E-35	postive
TRAJ31	WARS2-AS1	0.507145989	2.62E-36	postive
TRAJ38	WARS2-AS1	0.567486392	6.29E-47	postive
TRAJ39	WARS2-AS1	0.523623378	5.34E-39	postive
CREB1	AC113139.1	0.533876752	9.50E-41	postive
DDX17	AC113139.1	0.612432046	2.18E-56	postive
NFAT5	AC113139.1	0.640549784	4.01E-63	postive
GNRH1	AC113139.1	0.686820009	6.98E-76	postive
NR2C1	AC113139.1	0.601773095	5.23E-54	postive
NR2C2	AC113139.1	0.595922437	9.71E-53	postive
RORA	AC113139.1	0.574206123	2.99E-48	postive
SOS1	AC113139.1	0.510691181	7.11E-37	postive
BRAF	AC113139.1	0.563028265	4.56E-46	postive
CBLB	AC113139.1	0.508813014	1.42E-36	postive
TRAJ1	AC113139.1	0.576764743	9.22E-49	postive
TRAJ2	AC113139.1	0.600274038	1.11E-53	postive
TRAJ3	AC113139.1	0.599163113	1.94E-53	postive
TRAJ5	AC113139.1	0.533573672	1.07E-40	postive
TRAJ6	AC113139.1	0.606963386	3.72E-55	postive
TRAJ8	AC113139.1	0.571126034	1.22E-47	postive
TRAJ10	AC113139.1	0.571818364	8.90E-48	postive
TRAJ12	AC113139.1	0.553474111	2.88E-44	postive
TRAJ13	AC113139.1	0.570321059	1.76E-47	postive

TRAJ14	AC113139.1	0.570682585	1.49E-47	postive
TRAJ16	AC113139.1	0.585919968	1.25E-50	postive
TRAJ17	AC113139.1	0.528997273	6.58E-40	postive
TRAJ18	AC113139.1	0.553485991	2.87E-44	postive
TRAJ21	AC113139.1	0.581481573	1.02E-49	postive
TRAJ31	AC113139.1	0.578312514	4.50E-49	postive
TRAJ37	AC113139.1	0.600419016	1.03E-53	postive
TRAJ38	AC113139.1	0.614897032	5.96E-57	postive
TRAJ39	AC113139.1	0.573990638	3.30E-48	postive
UMODL1	LINC01419	0.514926211	1.47E-37	postive
BPIFB1	LINC01765	0.551018433	8.20E-44	postive
DES	LINC01765	0.670579305	3.85E-71	postive
CYLD	AC005479.1	0.51136404	5.54E-37	postive
NFAT5	AC005479.1	0.566461768	9.94E-47	postive
PIK3R1	AC005479.1	0.515302017	1.27E-37	postive
IL6ST	AC005479.1	0.580655919	1.51E-49	postive
RORA	AC005479.1	0.610438118	6.17E-56	postive
BRAF	AC005479.1	0.549099102	1.84E-43	postive
TRAJ5	AC005479.1	0.537358599	2.34E-41	postive
TRAJ10	AC005479.1	0.503798337	8.87E-36	postive
TRAJ13	AC005479.1	0.512400403	3.77E-37	postive
TRAJ18	AC005479.1	0.513709236	2.31E-37	postive
TRAJ21	AC005479.1	0.529050603	6.44E-40	postive
TRAJ31	AC005479.1	0.530045554	4.35E-40	postive
TRAJ37	AC005479.1	0.528761902	7.21E-40	postive
TRAJ38	AC005479.1	0.538315593	1.59E-41	postive
TRAJ39	AC005479.1	0.538108533	1.73E-41	postive
OSGIN1	AC090772.3	0.524024366	4.58E-39	postive
CREB1	AC011477.1	0.755770528	4.55E-100	postive
RFXAP	AC011477.1	0.59149331	8.52E-52	postive
UBR1	AC011477.1	0.635395332	7.77E-62	postive
ZC3HAV1	AC011477.1	0.559299886	2.34E-45	postive
IL15	AC011477.1	0.511924313	4.50E-37	postive
CYLD	AC011477.1	0.588309828	3.98E-51	postive
EIF2AK2	AC011477.1	0.513527383	2.48E-37	postive
MAPK8	AC011477.1	0.504203868	7.65E-36	postive
LMBR1	AC011477.1	0.503950999	8.39E-36	postive
IREB2	AC011477.1	0.588250638	4.09E-51	postive
DDX17	AC011477.1	0.663389281	3.89E-69	postive
JAK2	AC011477.1	0.581020464	1.27E-49	postive
TXK	AC011477.1	0.559043234	2.61E-45	postive
NFAT5	AC011477.1	0.798772174	1.05E-119	postive
MALT1	AC011477.1	0.516117085	9.38E-38	postive
PIK3R1	AC011477.1	0.623924482	4.66E-59	postive
PIK3CA	AC011477.1	0.605210807	9.14E-55	postive
AKT3	AC011477.1	0.513110765	2.89E-37	postive
IGHD4-4	AC011477.1	0.514105058	2.00E-37	postive
IGHD6-6	AC011477.1	0.521053136	1.44E-38	postive
PLXNC1	AC011477.1	0.521098586	1.41E-38	postive
GNRH1	AC011477.1	0.772157101	4.85E-107	postive
IL6ST	AC011477.1	0.616110318	3.13E-57	postive
RABEP1	AC011477.1	0.553603209	2.73E-44	postive
ACVR2A	AC011477.1	0.684490869	3.49E-75	postive
ANGPTL1	AC011477.1	0.676154577	9.82E-73	postive
BMPR1A	AC011477.1	0.577788508	5.74E-49	postive
BMPR2	AC011477.1	0.684354864	3.83E-75	postive
CRLF3	AC011477.1	0.636807173	3.47E-62	postive
NR1D2	AC011477.1	0.515565482	1.15E-37	postive
NR2C1	AC011477.1	0.537567719	2.15E-41	postive
NR2C2	AC011477.1	0.755813953	4.37E-100	postive
RORA	AC011477.1	0.754376575	1.68E-99	postive
SOS1	AC011477.1	0.654960947	7.41E-67	postive
SOS2	AC011477.1	0.596796147	6.30E-53	postive
BRAF	AC011477.1	0.796441646	1.60E-118	postive
CBL	AC011477.1	0.550818477	8.92E-44	postive

CBLB	AC011477.1	0.6185854	8.37E-58	postive
RASGRP1	AC011477.1	0.551253398	7.42E-44	postive
TRAJ1	AC011477.1	0.73368769	1.68E-91	postive
TRAJ2	AC011477.1	0.736595172	1.40E-92	postive
TRAJ3	AC011477.1	0.780410219	8.89E-111	postive
TRAJ5	AC011477.1	0.730527608	2.40E-90	postive
TRAJ6	AC011477.1	0.764308942	1.25E-103	postive
TRAJ8	AC011477.1	0.778890775	4.46E-110	postive
TRAJ10	AC011477.1	0.73047332	2.51E-90	postive
TRAJ12	AC011477.1	0.667222445	3.38E-70	postive
TRAJ13	AC011477.1	0.759059273	2.01E-101	postive
TRAJ14	AC011477.1	0.740553028	4.52E-94	postive
TRAJ16	AC011477.1	0.747093669	1.35E-96	postive
TRAJ17	AC011477.1	0.723017745	1.16E-87	postive
TRAJ18	AC011477.1	0.658283232	9.55E-68	postive
TRAJ21	AC011477.1	0.765546609	3.69E-104	postive
TRAJ31	AC011477.1	0.771823104	6.82E-107	postive
TRAJ37	AC011477.1	0.759346784	1.53E-101	postive
TRAJ38	AC011477.1	0.790030399	2.41E-115	postive
TRAJ39	AC011477.1	0.790889928	9.17E-116	postive
FABP7	AC022075.1	0.678831107	1.64E-73	postive
HCK	AC022075.1	0.515537048	1.17E-37	postive
SEMA3D	AC022075.1	0.51968062	2.43E-38	postive
CD4	TRG-AS1	0.505936053	4.08E-36	postive
CD8A	TRG-AS1	0.704726317	1.74E-81	postive
CD8B	TRG-AS1	0.558518481	3.28E-45	postive
CTSS	TRG-AS1	0.504848695	6.06E-36	postive
IFNG	TRG-AS1	0.714583012	9.38E-85	postive
LTA	TRG-AS1	0.585964209	1.22E-50	postive
CIITA	TRG-AS1	0.634048299	1.67E-61	postive
CXCL9	TRG-AS1	0.601709957	5.40E-54	postive
TLR4	TRG-AS1	0.523287156	6.09E-39	postive
APOBEC3G	TRG-AS1	0.602182129	4.25E-54	postive
RBP5	TRG-AS1	0.501508133	2.02E-35	postive
CYBB	TRG-AS1	0.567349273	6.69E-47	postive
STAT1	TRG-AS1	0.522811029	7.31E-39	postive
IRF1	TRG-AS1	0.571339175	1.11E-47	postive
IL15	TRG-AS1	0.624886295	2.75E-59	postive
TLR7	TRG-AS1	0.560349647	1.48E-45	postive
CCL5	TRG-AS1	0.650985063	8.32E-66	postive
CYLD	TRG-AS1	0.541805487	3.82E-42	postive
TLR8	TRG-AS1	0.577391157	6.90E-49	postive
CCL4	TRG-AS1	0.647334757	7.42E-65	postive
IL7R	TRG-AS1	0.556056623	9.53E-45	postive
CD40LG	TRG-AS1	0.596537793	7.16E-53	postive
TRIM22	TRG-AS1	0.536098776	3.89E-41	postive
PIK3CG	TRG-AS1	0.585486575	1.54E-50	postive
PDCD1	TRG-AS1	0.599166833	1.94E-53	postive
FASLG	TRG-AS1	0.729219813	7.15E-90	postive
CCR5	TRG-AS1	0.763824308	2.01E-103	postive
CCL4L2	TRG-AS1	0.510416506	7.88E-37	postive
XCL2	TRG-AS1	0.53319642	1.25E-40	postive
CXCR4	TRG-AS1	0.527485897	1.19E-39	postive
CXCR6	TRG-AS1	0.784312855	1.33E-112	postive
CCR4	TRG-AS1	0.509840838	9.74E-37	postive
JAK2	TRG-AS1	0.605672717	7.21E-55	postive
CD86	TRG-AS1	0.520513929	1.77E-38	postive
BTK	TRG-AS1	0.653186509	2.19E-66	postive
RAC2	TRG-AS1	0.504147026	7.81E-36	postive
PPP3CC	TRG-AS1	0.5223998	8.57E-39	postive
NFATC2	TRG-AS1	0.572739338	5.85E-48	postive
CD19	TRG-AS1	0.501558353	1.99E-35	postive
PIK3R5	TRG-AS1	0.56369075	3.40E-46	postive
PIK3CD	TRG-AS1	0.554794525	1.64E-44	postive
INPP5D	TRG-AS1	0.559462263	2.18E-45	postive

CD72	TRG-AS1	0.649288214	2.31E-65	postive
RASGRP3	TRG-AS1	0.652103517	4.23E-66	postive
PLCG2	TRG-AS1	0.566853368	8.35E-47	postive
PRKCB	TRG-AS1	0.612999328	1.62E-56	postive
IGKJ5	TRG-AS1	0.579511765	2.57E-49	postive
SEMA4D	TRG-AS1	0.609412764	1.05E-55	postive
CMKLR1	TRG-AS1	0.576144329	1.23E-48	postive
PLXNC1	TRG-AS1	0.644175842	4.82E-64	postive
ADA2	TRG-AS1	0.516640581	7.70E-38	postive
FLT3LG	TRG-AS1	0.527679809	1.10E-39	postive
GMFG	TRG-AS1	0.526147142	2.01E-39	postive
IL16	TRG-AS1	0.690925484	3.94E-77	postive
TNFSF13B	TRG-AS1	0.673398344	6.08E-72	postive
TNFSF8	TRG-AS1	0.549239354	1.74E-43	postive
CSF2RB	TRG-AS1	0.639146733	9.03E-63	postive
IL10RA	TRG-AS1	0.745476138	5.78E-96	postive
IL12RB1	TRG-AS1	0.687333964	4.88E-76	postive
IL2RB	TRG-AS1	0.687032287	6.02E-76	postive
IL18RAP	TRG-AS1	0.736491452	1.53E-92	postive
IL21R	TRG-AS1	0.636654256	3.79E-62	postive
IL2RG	TRG-AS1	0.569852669	2.17E-47	postive
ITGAL	TRG-AS1	0.737550241	6.15E-93	postive
LCK	TRG-AS1	0.638193753	1.56E-62	postive
NCR3	TRG-AS1	0.580725878	1.46E-49	postive
CD247	TRG-AS1	0.800959593	7.83E-121	postive
ZAP70	TRG-AS1	0.725021894	2.27E-88	postive
LCP2	TRG-AS1	0.718644481	3.84E-86	postive
HCST	TRG-AS1	0.50806626	1.87E-36	postive
CD48	TRG-AS1	0.548660556	2.22E-43	postive
CD244	TRG-AS1	0.713842119	1.67E-84	postive
SH2D1A	TRG-AS1	0.803304879	4.69E-122	postive
GZMB	TRG-AS1	0.533042591	1.32E-40	postive
PRF1	TRG-AS1	0.522836553	7.24E-39	postive
CD3D	TRG-AS1	0.675895244	1.17E-72	postive
CD3E	TRG-AS1	0.737787188	5.01E-93	postive
CD3G	TRG-AS1	0.778082124	1.05E-109	postive
PTPRC	TRG-AS1	0.757460379	9.23E-101	postive
ITK	TRG-AS1	0.761977056	1.21E-102	postive
GRAP2	TRG-AS1	0.581450646	1.04E-49	postive
CD28	TRG-AS1	0.639491792	7.40E-63	postive
ICOS	TRG-AS1	0.714918118	7.22E-85	postive
CTLA4	TRG-AS1	0.634095054	1.63E-61	postive
TRAC	TRG-AS1	0.720496608	8.79E-87	postive
TRAV3	TRG-AS1	0.525629094	2.46E-39	postive
TRAV4	TRG-AS1	0.645312171	2.46E-64	postive
TRAV8-2	TRG-AS1	0.642600377	1.21E-63	postive
TRAV8-3	TRG-AS1	0.667012687	3.86E-70	postive
TRAV8-4	TRG-AS1	0.597507066	4.43E-53	postive
TRAV8-6	TRG-AS1	0.610748332	5.25E-56	postive
TRAV9-2	TRG-AS1	0.591522625	8.40E-52	postive
TRAV12-2	TRG-AS1	0.569352556	2.72E-47	postive
TRAV12-3	TRG-AS1	0.529662722	5.06E-40	postive
TRAV13-1	TRG-AS1	0.503879101	8.61E-36	postive
TRAV13-2	TRG-AS1	0.586034416	1.18E-50	postive
TRAV14DV4	TRG-AS1	0.565368252	1.62E-46	postive
TRAV16	TRG-AS1	0.608710647	1.51E-55	postive
TRAV17	TRG-AS1	0.619441957	5.29E-58	postive
TRAV21	TRG-AS1	0.60488061	1.08E-54	postive
TRAV29DV5	TRG-AS1	0.620699591	2.69E-58	postive
TRAV41	TRG-AS1	0.571681927	9.47E-48	postive
TRBC1	TRG-AS1	0.799865173	2.88E-120	postive
TRBC2	TRG-AS1	0.68265009	1.23E-74	postive
TRBJ2-2	TRG-AS1	0.546094511	6.48E-43	postive
TRBJ2-3	TRG-AS1	0.600344532	1.07E-53	postive
TRBJ2-7	TRG-AS1	0.62791098	5.20E-60	postive

TRBV2	TRG-AS1	0.629108564	2.68E-60	postive
TRBV3-1	TRG-AS1	0.565290061	1.68E-46	postive
TRBV5-1	TRG-AS1	0.625043927	2.53E-59	postive
TRBV5-4	TRG-AS1	0.595478924	1.21E-52	postive
TRBV6-1	TRG-AS1	0.556070074	9.47E-45	postive
TRBV6-5	TRG-AS1	0.599583565	1.57E-53	postive
TRBV6-6	TRG-AS1	0.506544706	3.27E-36	postive
TRBV7-9	TRG-AS1	0.655914345	4.13E-67	postive
TRBV9	TRG-AS1	0.5355557	4.84E-41	postive
TRBV10-3	TRG-AS1	0.521754559	1.10E-38	postive
TRBV11-2	TRG-AS1	0.518510798	3.79E-38	postive
TRBV12-4	TRG-AS1	0.541227884	4.84E-42	postive
TRBV18	TRG-AS1	0.619140495	6.21E-58	postive
TRBV19	TRG-AS1	0.555670527	1.13E-44	postive
TRBV20-1	TRG-AS1	0.584976019	1.96E-50	postive
TRBV28	TRG-AS1	0.716558957	2.00E-85	postive
TRDV1	TRG-AS1	0.513932986	2.13E-37	postive
IRF9	AL139349.1	0.533800996	9.79E-41	postive
GIPR	AL139349.1	0.50583703	4.23E-36	postive
CREB1	AC011468.1	0.6022595	4.09E-54	postive
UBR1	AC011468.1	0.506791507	2.99E-36	postive
DDX17	AC011468.1	0.624557254	3.30E-59	postive
TXK	AC011468.1	0.55941949	2.22E-45	postive
NFAT5	AC011468.1	0.652351912	3.64E-66	postive
GNRH1	AC011468.1	0.834992505	2.19E-140	postive
IL6ST	AC011468.1	0.52129143	1.31E-38	postive
ACVR2A	AC011468.1	0.56773158	5.63E-47	postive
ANGPTL1	AC011468.1	0.579225367	2.94E-49	postive
BMPR2	AC011468.1	0.575170378	1.92E-48	postive
CRLF3	AC011468.1	0.515736892	1.08E-37	postive
NR2C1	AC011468.1	0.586138166	1.13E-50	postive
NR2C2	AC011468.1	0.696853784	5.69E-79	postive
RORA	AC011468.1	0.668724182	1.28E-70	postive
SOS1	AC011468.1	0.523866595	4.86E-39	postive
BRAF	AC011468.1	0.668058016	1.97E-70	postive
TEC	AC011468.1	0.501637251	1.93E-35	postive
CBLB	AC011468.1	0.606645333	4.38E-55	postive
TRAJ1	AC011468.1	0.563937328	3.05E-46	postive
TRAJ2	AC011468.1	0.525591461	2.49E-39	postive
TRAJ3	AC011468.1	0.603980021	1.71E-54	postive
TRAJ5	AC011468.1	0.601171698	7.08E-54	postive
TRAJ6	AC011468.1	0.577584134	6.31E-49	postive
TRAJ8	AC011468.1	0.554811657	1.63E-44	postive
TRAJ10	AC011468.1	0.554213029	2.10E-44	postive
TRAJ13	AC011468.1	0.554053308	2.25E-44	postive
TRAJ14	AC011468.1	0.535316689	5.33E-41	postive
TRAJ16	AC011468.1	0.566813531	8.50E-47	postive
TRAJ17	AC011468.1	0.54023588	7.27E-42	postive
TRAJ18	AC011468.1	0.504658843	6.49E-36	postive
TRAJ21	AC011468.1	0.586240521	1.07E-50	postive
TRAJ31	AC011468.1	0.610894297	4.87E-56	postive
TRAJ37	AC011468.1	0.588670087	3.34E-51	postive
TRAJ38	AC011468.1	0.619198258	6.03E-58	postive
TRAJ39	AC011468.1	0.630231827	1.43E-60	postive
LMBR1L	PTOV1-AS2	0.559418615	2.22E-45	postive
IRF3	PTOV1-AS2	0.693050768	8.72E-78	postive
TYK2	PTOV1-AS2	0.676671495	6.96E-73	postive
IRF9	PTOV1-AS2	0.654629502	9.08E-67	postive
PLXNB1	PTOV1-AS2	0.581417495	1.05E-49	postive
RABEP2	PTOV1-AS2	0.507897841	1.99E-36	postive
EPOR	PTOV1-AS2	0.558121809	3.90E-45	postive
GIPR	PTOV1-AS2	0.579740938	2.31E-49	postive
TNFRSF14	PTOV1-AS2	0.678992895	1.47E-73	postive
TNFRSF25	PTOV1-AS2	0.552425294	4.51E-44	postive
ZAP70	PTOV1-AS2	0.500257308	3.17E-35	postive

CREB1	AC005046.2	0.598218525	3.11E-53	postive
IL15	AC005046.2	0.561874913	7.58E-46	postive
NFKBIZ	AC005046.2	0.509813322	9.84E-37	postive
LIMS1	AC005046.2	0.509965395	9.30E-37	postive
DDX17	AC005046.2	0.515910874	1.01E-37	postive
JAK2	AC005046.2	0.602636924	3.38E-54	postive
TXK	AC005046.2	0.566590475	9.39E-47	postive
NFAT5	AC005046.2	0.614688956	6.65E-57	postive
MALT1	AC005046.2	0.501407708	2.10E-35	postive
PIK3R1	AC005046.2	0.53419722	8.35E-41	postive
PIK3CA	AC005046.2	0.595393375	1.26E-52	postive
GNRH1	AC005046.2	0.673439686	5.92E-72	postive
ANGPTL1	AC005046.2	0.704853049	1.58E-81	postive
BMPR2	AC005046.2	0.576004529	1.31E-48	postive
IL18R1	AC005046.2	0.545627678	7.88E-43	postive
NR2C2	AC005046.2	0.574669281	2.42E-48	postive
RORA	AC005046.2	0.664812817	1.58E-69	postive
SOS1	AC005046.2	0.543320303	2.05E-42	postive
BRAF	AC005046.2	0.581299811	1.11E-49	postive
CBL	AC005046.2	0.553691311	2.63E-44	postive
CBLB	AC005046.2	0.688861793	1.68E-76	postive
PDK1	AC005046.2	0.55564272	1.14E-44	postive
TRAJ3	AC005046.2	0.520306873	1.91E-38	postive
TRAJ5	AC005046.2	0.539828756	8.58E-42	postive
TRAJ6	AC005046.2	0.511205284	5.88E-37	postive
TRAJ10	AC005046.2	0.521655283	1.14E-38	postive
TRAJ16	AC005046.2	0.517060452	6.57E-38	postive
TRAJ21	AC005046.2	0.519427673	2.67E-38	postive
TRAJ31	AC005046.2	0.540401454	6.79E-42	postive
TRAJ38	AC005046.2	0.59099138	1.09E-51	postive
TRAJ39	AC005046.2	0.540790954	5.79E-42	postive
CREB1	AC099343.3	0.757680863	7.48E-101	postive
RFXAP	AC099343.3	0.533532217	1.09E-40	postive
UBR1	AC099343.3	0.606298436	5.24E-55	postive
ZC3HAV1	AC099343.3	0.561906077	7.48E-46	postive
ZC3HAV1L	AC099343.3	0.507873499	2.01E-36	postive
IL15	AC099343.3	0.665415718	1.07E-69	postive
CYLD	AC099343.3	0.624626133	3.18E-59	postive
EIF2AK2	AC099343.3	0.523571033	5.45E-39	postive
MAPK8	AC099343.3	0.537291924	2.40E-41	postive
NFKBIZ	AC099343.3	0.518938364	3.22E-38	postive
LMBR1	AC099343.3	0.539866947	8.45E-42	postive
IREB2	AC099343.3	0.580389585	1.71E-49	postive
DDX17	AC099343.3	0.631380933	7.51E-61	postive
PIK3CG	AC099343.3	0.531495364	2.45E-40	postive
JAK2	AC099343.3	0.660865174	1.91E-68	postive
TXK	AC099343.3	0.591456407	8.68E-52	postive
NFAT5	AC099343.3	0.812032635	9.39E-127	postive
MALT1	AC099343.3	0.531335837	2.61E-40	postive
PIK3R1	AC099343.3	0.604078515	1.63E-54	postive
PIK3CA	AC099343.3	0.668870459	1.17E-70	postive
PIK3CB	AC099343.3	0.526371981	1.84E-39	postive
AKT3	AC099343.3	0.526259281	1.92E-39	postive
RASGRP3	AC099343.3	0.577942391	5.34E-49	postive
IGHD4-4	AC099343.3	0.516369235	8.52E-38	postive
IGHD6-6	AC099343.3	0.525184941	2.92E-39	postive
LTB4R2	AC099343.3	0.521864916	1.05E-38	postive
PLXNC1	AC099343.3	0.527855369	1.03E-39	postive
GNRH1	AC099343.3	0.798480352	1.47E-119	postive
IL6ST	AC099343.3	0.547685222	3.34E-43	postive
IL7	AC099343.3	0.504688246	6.42E-36	postive
RABEP1	AC099343.3	0.557573202	4.95E-45	postive
ACVR2A	AC099343.3	0.638596446	1.24E-62	postive
ANGPTL1	AC099343.3	0.761339632	2.25E-102	postive
BMPR2	AC099343.3	0.691608314	2.43E-77	postive

CRLF3	AC099343.3	0.632908115	3.18E-61	postive
NR2C2	AC099343.3	0.717498111	9.52E-86	postive
RORA	AC099343.3	0.799051452	7.53E-120	postive
SOS1	AC099343.3	0.677962807	2.94E-73	postive
SOS2	AC099343.3	0.572069265	7.94E-48	postive
BRAF	AC099343.3	0.764839499	7.41E-104	postive
ITK	AC099343.3	0.549239048	1.74E-43	postive
CD28	AC099343.3	0.503427329	1.01E-35	postive
CBL	AC099343.3	0.619219472	5.96E-58	postive
CBLB	AC099343.3	0.71628916	2.47E-85	postive
RASGRP1	AC099343.3	0.518961586	3.19E-38	postive
PDK1	AC099343.3	0.622719955	8.99E-59	postive
TRAJ1	AC099343.3	0.764961783	6.57E-104	postive
TRAJ2	AC099343.3	0.71002238	3.17E-83	postive
TRAJ3	AC099343.3	0.814058531	7.03E-128	postive
TRAJ5	AC099343.3	0.796666801	1.23E-118	postive
TRAJ6	AC099343.3	0.797572677	4.27E-119	postive
TRAJ8	AC099343.3	0.764665353	8.79E-104	postive
TRAJ10	AC099343.3	0.78679908	8.76E-114	postive
TRAJ12	AC099343.3	0.731975973	7.13E-91	postive
TRAJ13	AC099343.3	0.781231707	3.70E-111	postive
TRAJ14	AC099343.3	0.769856034	5.01E-106	postive
TRAJ16	AC099343.3	0.78701914	6.87E-114	postive
TRAJ17	AC099343.3	0.777024165	3.17E-109	postive
TRAJ18	AC099343.3	0.733172082	2.60E-91	postive
TRAJ21	AC099343.3	0.804426335	1.21E-122	postive
TRAJ31	AC099343.3	0.830771793	9.97E-138	postive
TRAJ37	AC099343.3	0.793723009	3.67E-117	postive
TRAJ38	AC099343.3	0.848114454	3.69E-149	postive
TRAJ39	AC099343.3	0.813812675	9.64E-128	postive
CD1D	LINC00426	0.500318632	3.10E-35	postive
CD8A	LINC00426	0.501352431	2.14E-35	postive
IFNG	LINC00426	0.546816229	4.80E-43	postive
LTA	LINC00426	0.571270641	1.14E-47	postive
CIITA	LINC00426	0.684159017	4.38E-75	postive
RBP5	LINC00426	0.610820289	5.06E-56	postive
IL15	LINC00426	0.577242216	7.39E-49	postive
CYLD	LINC00426	0.69311127	8.35E-78	postive
IL7R	LINC00426	0.635641191	6.75E-62	postive
CD40LG	LINC00426	0.656042127	3.82E-67	postive
TRIM22	LINC00426	0.595624521	1.12E-52	postive
PIK3CG	LINC00426	0.663518781	3.58E-69	postive
CCR7	LINC00426	0.58308416	4.81E-50	postive
CCR8	LINC00426	0.524398057	3.96E-39	postive
CCR5	LINC00426	0.620036519	3.84E-58	postive
CXCR4	LINC00426	0.54292419	2.41E-42	postive
CXCR6	LINC00426	0.633328706	2.51E-61	postive
CCR4	LINC00426	0.662356752	7.47E-69	postive
JAK2	LINC00426	0.632071149	5.10E-61	postive
TXK	LINC00426	0.501547277	2.00E-35	postive
BTK	LINC00426	0.573804369	3.60E-48	postive
PPP3CC	LINC00426	0.577278566	7.27E-49	postive
NFATC2	LINC00426	0.660520734	2.37E-68	postive
CD19	LINC00426	0.589926954	1.82E-51	postive
PIK3R5	LINC00426	0.548488444	2.38E-43	postive
PIK3R1	LINC00426	0.510293372	8.24E-37	postive
INPP5D	LINC00426	0.601027792	7.61E-54	postive
CD22	LINC00426	0.529500882	5.39E-40	postive
RASGRP3	LINC00426	0.657478173	1.57E-67	postive
PLCG2	LINC00426	0.663925893	2.77E-69	postive
PRKCB	LINC00426	0.691547206	2.54E-77	postive
IGHD6-6	LINC00426	0.522089575	9.65E-39	postive
IGKJ5	LINC00426	0.602358215	3.89E-54	postive
SEMA4D	LINC00426	0.646347958	1.33E-64	postive
PLXNC1	LINC00426	0.603694378	1.98E-54	postive

IL16	LINC00426	0.702041514	1.28E-80	postive
TNFSF13B	LINC00426	0.500980533	2.45E-35	postive
TNFSF8	LINC00426	0.660532977	2.35E-68	postive
CRLF3	LINC00426	0.500665789	2.74E-35	postive
CSF2RB	LINC00426	0.59332587	3.48E-52	postive
IL10RA	LINC00426	0.670535586	3.96E-71	postive
IL12RB1	LINC00426	0.597530995	4.38E-53	postive
IL2RB	LINC00426	0.529301336	5.83E-40	postive
IL18RAP	LINC00426	0.665333526	1.13E-69	postive
IL21R	LINC00426	0.570431076	1.67E-47	postive
RORA	LINC00426	0.56364635	3.47E-46	postive
TNFRSF13C	LINC00426	0.603234101	2.50E-54	postive
ITGAL	LINC00426	0.65988731	3.52E-68	postive
LCK	LINC00426	0.530248989	4.02E-40	postive
NCR3	LINC00426	0.544253186	1.39E-42	postive
CD247	LINC00426	0.670793459	3.35E-71	postive
ZAP70	LINC00426	0.687287689	5.04E-76	postive
LCP2	LINC00426	0.613520473	1.23E-56	postive
SH2D1A	LINC00426	0.730336416	2.82E-90	postive
CD3D	LINC00426	0.514644683	1.63E-37	postive
CD3E	LINC00426	0.627690391	5.88E-60	postive
CD3G	LINC00426	0.690992227	3.75E-77	postive
PTPRC	LINC00426	0.7572603	1.12E-100	postive
ITK	LINC00426	0.863852978	7.58E-161	postive
GRAP2	LINC00426	0.548316603	2.56E-43	postive
CD28	LINC00426	0.766513089	1.42E-104	postive
ICOS	LINC00426	0.729319323	6.58E-90	postive
CTLA4	LINC00426	0.66063612	2.20E-68	postive
TRAC	LINC00426	0.607712326	2.53E-55	postive
TRAJ1	LINC00426	0.560742029	1.25E-45	postive
TRAJ2	LINC00426	0.588150965	4.29E-51	postive
TRAJ3	LINC00426	0.607906458	2.29E-55	postive
TRAJ5	LINC00426	0.503069615	1.15E-35	postive
TRAJ18	LINC00426	0.540767929	5.85E-42	postive
TRAJ38	LINC00426	0.521410126	1.25E-38	postive
TRAV4	LINC00426	0.608161176	2.01E-55	postive
TRAV8-2	LINC00426	0.576163682	1.22E-48	postive
TRAV8-3	LINC00426	0.614985638	5.69E-57	postive
TRAV8-4	LINC00426	0.546425725	5.65E-43	postive
TRAV8-6	LINC00426	0.531583966	2.37E-40	postive
TRAV9-2	LINC00426	0.571339158	1.11E-47	postive
TRAV17	LINC00426	0.552054819	5.28E-44	postive
TRAV29DV5	LINC00426	0.525694378	2.39E-39	postive
TRBC1	LINC00426	0.740553972	4.52E-94	postive
TRBC2	LINC00426	0.564948953	1.95E-46	postive
TRBJ2-2	LINC00426	0.509917427	9.47E-37	postive
TRBJ2-7	LINC00426	0.635304624	8.18E-62	postive
TRBV2	LINC00426	0.540793569	5.79E-42	postive
TRBV3-1	LINC00426	0.520222606	1.97E-38	postive
TRBV5-1	LINC00426	0.550058437	1.23E-43	postive
TRBV5-4	LINC00426	0.537230676	2.47E-41	postive
TRBV6-5	LINC00426	0.508547786	1.57E-36	postive
TRBV7-9	LINC00426	0.531970395	2.03E-40	postive
TRBV18	LINC00426	0.558328366	3.57E-45	postive
TRBV20-1	LINC00426	0.549357958	1.65E-43	postive
TRBV28	LINC00426	0.62236628	1.09E-58	postive
CREB1	MIR29B2CHG	0.759722158	1.07E-101	postive
RFXAP	MIR29B2CHG	0.50420688	7.65E-36	postive
UBR1	MIR29B2CHG	0.678926257	1.54E-73	postive
ZC3HAV1	MIR29B2CHG	0.556875432	6.69E-45	postive
IL15	MIR29B2CHG	0.54057774	6.32E-42	postive
CYLD	MIR29B2CHG	0.684868842	2.69E-75	postive
EIF2AK2	MIR29B2CHG	0.522625206	7.85E-39	postive
LMBR1	MIR29B2CHG	0.506392449	3.45E-36	postive
IREB2	MIR29B2CHG	0.561442668	9.16E-46	postive

DDX17	MIR29B2CHG	0.65754212	1.51E-67	postive
PIK3CG	MIR29B2CHG	0.511927568	4.50E-37	postive
JAK2	MIR29B2CHG	0.634666983	1.18E-61	postive
TXK	MIR29B2CHG	0.603280764	2.44E-54	postive
NFAT5	MIR29B2CHG	0.900895542	2.43E-195	postive
NFATC3	MIR29B2CHG	0.618010243	1.14E-57	postive
PIK3R1	MIR29B2CHG	0.63320346	2.69E-61	postive
PIK3CA	MIR29B2CHG	0.645460616	2.26E-64	postive
PIK3CB	MIR29B2CHG	0.527863376	1.03E-39	postive
RASGRP3	MIR29B2CHG	0.500693114	2.71E-35	postive
IGHD6-6	MIR29B2CHG	0.52278335	7.39E-39	postive
ROBO2	MIR29B2CHG	0.55741023	5.31E-45	postive
GNRH1	MIR29B2CHG	0.780715497	6.42E-111	postive
IL6ST	MIR29B2CHG	0.613114969	1.52E-56	postive
IL7	MIR29B2CHG	0.525089909	3.03E-39	postive
RABEP1	MIR29B2CHG	0.55501713	1.49E-44	postive
ACVR2A	MIR29B2CHG	0.643607172	6.73E-64	postive
ANGPTL1	MIR29B2CHG	0.668454976	1.53E-70	postive
BMPR1A	MIR29B2CHG	0.513530972	2.47E-37	postive
BMPR2	MIR29B2CHG	0.684984418	2.48E-75	postive
CRLF3	MIR29B2CHG	0.584792349	2.14E-50	postive
NR1D2	MIR29B2CHG	0.542684498	2.66E-42	postive
NR2C2	MIR29B2CHG	0.741455288	2.05E-94	postive
NR3C2	MIR29B2CHG	0.528908471	6.81E-40	postive
RORA	MIR29B2CHG	0.832370621	1.00E-138	postive
SOS1	MIR29B2CHG	0.635332712	8.05E-62	postive
SOS2	MIR29B2CHG	0.557901853	4.29E-45	postive
BRAF	MIR29B2CHG	0.741651164	1.72E-94	postive
TEC	MIR29B2CHG	0.521428396	1.24E-38	postive
CBL	MIR29B2CHG	0.567407842	6.51E-47	postive
CBLB	MIR29B2CHG	0.632403108	4.23E-61	postive
RASGRP1	MIR29B2CHG	0.526567181	1.70E-39	postive
TRAJ1	MIR29B2CHG	0.816800493	2.00E-129	postive
TRAJ2	MIR29B2CHG	0.727175453	3.88E-89	postive
TRAJ3	MIR29B2CHG	0.827097232	1.80E-135	postive
TRAJ5	MIR29B2CHG	0.818897628	1.26E-130	postive
TRAJ6	MIR29B2CHG	0.85819808	1.74E-156	postive
TRAJ8	MIR29B2CHG	0.788959683	7.98E-115	postive
TRAJ10	MIR29B2CHG	0.842995903	1.22E-145	postive
TRAJ12	MIR29B2CHG	0.836347711	2.95E-141	postive
TRAJ13	MIR29B2CHG	0.816283877	3.92E-129	postive
TRAJ14	MIR29B2CHG	0.811963112	1.03E-126	postive
TRAJ16	MIR29B2CHG	0.839772923	1.72E-143	postive
TRAJ17	MIR29B2CHG	0.81581964	7.19E-129	postive
TRAJ18	MIR29B2CHG	0.839982545	1.25E-143	postive
TRAJ21	MIR29B2CHG	0.85415631	1.75E-153	postive
TRAJ31	MIR29B2CHG	0.862772353	5.35E-160	postive
TRAJ37	MIR29B2CHG	0.861176775	9.28E-159	postive
TRAJ38	MIR29B2CHG	0.877445164	3.47E-172	postive
TRAJ39	MIR29B2CHG	0.865747905	2.37E-162	postive
RABEP2	ASMTL-AS1	0.501358919	2.14E-35	postive
CREB1	AC084871.2	0.596534831	7.17E-53	postive
TLR4	AC084871.2	0.504286892	7.43E-36	postive
IL15	AC084871.2	0.629236941	2.49E-60	postive
TLR1	AC084871.2	0.513842968	2.20E-37	postive
NFKBIZ	AC084871.2	0.504848822	6.06E-36	postive
PIK3CG	AC084871.2	0.512370325	3.81E-37	postive
JAK2	AC084871.2	0.593998564	2.50E-52	postive
NFAT5	AC084871.2	0.591543507	8.32E-52	postive
PIK3CA	AC084871.2	0.609982426	7.82E-56	postive
FPR2	AC084871.2	0.515681566	1.10E-37	postive
GNRH1	AC084871.2	0.523978377	4.66E-39	postive
ANGPTL1	AC084871.2	0.719020398	2.85E-86	postive
BMPR2	AC084871.2	0.509880847	9.60E-37	postive
CRLF3	AC084871.2	0.514245664	1.89E-37	postive

IL18R1	AC084871.2	0.577763111	5.81E-49	postive
NR2C2	AC084871.2	0.51153622	5.20E-37	postive
RORA	AC084871.2	0.601178181	7.06E-54	postive
SOS1	AC084871.2	0.503986628	8.28E-36	postive
BRAF	AC084871.2	0.595119959	1.44E-52	postive
CBL	AC084871.2	0.566817264	8.48E-47	postive
CBLB	AC084871.2	0.671386833	2.27E-71	postive
PDK1	AC084871.2	0.588742391	3.23E-51	postive
TRAJ1	AC084871.2	0.527559932	1.16E-39	postive
TRAJ2	AC084871.2	0.523172423	6.36E-39	postive
TRAJ3	AC084871.2	0.621374624	1.86E-58	postive
TRAJ5	AC084871.2	0.589239053	2.54E-51	postive
TRAJ6	AC084871.2	0.599969554	1.30E-53	postive
TRAJ10	AC084871.2	0.624426367	3.54E-59	postive
TRAJ13	AC084871.2	0.549467594	1.58E-43	postive
TRAJ14	AC084871.2	0.579776259	2.27E-49	postive
TRAJ16	AC084871.2	0.589931939	1.82E-51	postive
TRAJ17	AC084871.2	0.578750108	3.67E-49	postive
TRAJ18	AC084871.2	0.604884767	1.08E-54	postive
TRAJ21	AC084871.2	0.619936463	4.05E-58	postive
TRAJ31	AC084871.2	0.585872473	1.28E-50	postive
TRAJ37	AC084871.2	0.545176003	9.50E-43	postive
TRAJ38	AC084871.2	0.657757844	1.32E-67	postive
TRAJ39	AC084871.2	0.617026404	1.92E-57	postive
CCRL2	CCR5AS	0.652169583	4.06E-66	postive
CREB1	AC138393.3	0.765338743	4.53E-104	postive
UBR1	AC138393.3	0.646796545	1.02E-64	postive
ZC3HAV1	AC138393.3	0.564870081	2.02E-46	postive
ZC3HAV1L	AC138393.3	0.571952918	8.38E-48	postive
IL15	AC138393.3	0.588290725	4.01E-51	postive
CYLD	AC138393.3	0.576002534	1.31E-48	postive
EIF2AK2	AC138393.3	0.523225886	6.23E-39	postive
MAPK8	AC138393.3	0.547986463	2.94E-43	postive
TLR1	AC138393.3	0.545983123	6.79E-43	postive
LMBR1	AC138393.3	0.587244467	6.63E-51	postive
IREB2	AC138393.3	0.600200746	1.15E-53	postive
DDX17	AC138393.3	0.583456307	4.03E-50	postive
PIK3CG	AC138393.3	0.563398897	3.87E-46	postive
JAK2	AC138393.3	0.641289532	2.61E-63	postive
TXK	AC138393.3	0.556319459	8.51E-45	postive
NFAT5	AC138393.3	0.801049168	7.04E-121	postive
MALT1	AC138393.3	0.540460099	6.63E-42	postive
PIK3R1	AC138393.3	0.59948817	1.65E-53	postive
PIK3CA	AC138393.3	0.665908617	7.84E-70	postive
PIK3CB	AC138393.3	0.509089008	1.29E-36	postive
AKT3	AC138393.3	0.607573716	2.72E-55	postive
RASGRP3	AC138393.3	0.504160612	7.78E-36	postive
GMFB	AC138393.3	0.509296348	1.19E-36	postive
GNRH1	AC138393.3	0.722894394	1.28E-87	postive
IL6ST	AC138393.3	0.594457332	2.00E-52	postive
RABEP1	AC138393.3	0.557283143	5.61E-45	postive
ACVR2A	AC138393.3	0.583933456	3.22E-50	postive
ANGPTL1	AC138393.3	0.770510153	2.59E-106	postive
BMPR1A	AC138393.3	0.532645762	1.55E-40	postive
BMPR2	AC138393.3	0.666938366	4.05E-70	postive
CRLF3	AC138393.3	0.698904538	1.28E-79	postive
NR2C2	AC138393.3	0.670936023	3.05E-71	postive
RORA	AC138393.3	0.756850985	1.64E-100	postive
SOS1	AC138393.3	0.648132037	4.61E-65	postive
SOS2	AC138393.3	0.574733262	2.35E-48	postive
BRAF	AC138393.3	0.803267526	4.91E-122	postive
ITK	AC138393.3	0.506465963	3.36E-36	postive
CBL	AC138393.3	0.628812804	3.15E-60	postive
CBLB	AC138393.3	0.708548729	9.75E-83	postive
RASGRP1	AC138393.3	0.501799804	1.82E-35	postive

PDK1	AC138393.3	0.615041142	5.52E-57	postive
TRAJ1	AC138393.3	0.707608527	1.99E-82	postive
TRAJ2	AC138393.3	0.750181291	8.15E-98	postive
TRAJ3	AC138393.3	0.784630188	9.43E-113	postive
TRAJ5	AC138393.3	0.744472075	1.42E-95	postive
TRAJ6	AC138393.3	0.773619585	1.08E-107	postive
TRAJ8	AC138393.3	0.762025603	1.16E-102	postive
TRAJ10	AC138393.3	0.746454648	2.40E-96	postive
TRAJ12	AC138393.3	0.627761743	5.65E-60	postive
TRAJ13	AC138393.3	0.771032797	1.52E-106	postive
TRAJ14	AC138393.3	0.754122531	2.13E-99	postive
TRAJ16	AC138393.3	0.748179859	5.05E-97	postive
TRAJ17	AC138393.3	0.70810908	1.36E-82	postive
TRAJ18	AC138393.3	0.654916373	7.62E-67	postive
TRAJ21	AC138393.3	0.785083991	5.75E-113	postive
TRAJ31	AC138393.3	0.769690777	5.92E-106	postive
TRAJ37	AC138393.3	0.750851954	4.41E-98	postive
TRAJ38	AC138393.3	0.826549729	3.85E-135	postive
TRAJ39	AC138393.3	0.796998589	8.35E-119	postive
CREB1	AC018682.1	0.657868506	1.24E-67	postive
IL15	AC018682.1	0.603027212	2.77E-54	postive
TLR1	AC018682.1	0.530626845	3.46E-40	postive
NFKBIZ	AC018682.1	0.554098924	2.21E-44	postive
LMBR1	AC018682.1	0.521905469	1.04E-38	postive
LIMS1	AC018682.1	0.518240488	4.20E-38	postive
PIK3CG	AC018682.1	0.511921836	4.51E-37	postive
JAK2	AC018682.1	0.60823599	1.93E-55	postive
NFAT5	AC018682.1	0.672199246	1.34E-71	postive
MALT1	AC018682.1	0.505044924	5.64E-36	postive
PIK3R1	AC018682.1	0.515857275	1.03E-37	postive
PIK3CA	AC018682.1	0.645266729	2.53E-64	postive
GNRH1	AC018682.1	0.611340053	3.86E-56	postive
IL6ST	AC018682.1	0.510743896	6.98E-37	postive
ANGPTL1	AC018682.1	0.75650328	2.28E-100	postive
BMPR2	AC018682.1	0.578188695	4.76E-49	postive
CRLF3	AC018682.1	0.584070839	3.01E-50	postive
IL18R1	AC018682.1	0.552451983	4.46E-44	postive
NR2C2	AC018682.1	0.558514447	3.29E-45	postive
RORA	AC018682.1	0.695646268	1.36E-78	postive
SOS1	AC018682.1	0.564374119	2.52E-46	postive
SOS2	AC018682.1	0.503630647	9.42E-36	postive
BRAF	AC018682.1	0.640095272	5.22E-63	postive
CBL	AC018682.1	0.585970188	1.22E-50	postive
CBLB	AC018682.1	0.704427632	2.17E-81	postive
PDK1	AC018682.1	0.62656071	1.10E-59	postive
TRAJ1	AC018682.1	0.572759367	5.80E-48	postive
TRAJ2	AC018682.1	0.551215463	7.54E-44	postive
TRAJ3	AC018682.1	0.627492987	6.56E-60	postive
TRAJ5	AC018682.1	0.66996891	5.73E-71	postive
TRAJ6	AC018682.1	0.632398477	4.24E-61	postive
TRAJ8	AC018682.1	0.557939652	4.22E-45	postive
TRAJ10	AC018682.1	0.661232979	1.51E-68	postive
TRAJ13	AC018682.1	0.619682339	4.65E-58	postive
TRAJ14	AC018682.1	0.600248246	1.13E-53	postive
TRAJ16	AC018682.1	0.641278184	2.63E-63	postive
TRAJ17	AC018682.1	0.611296183	3.95E-56	postive
TRAJ18	AC018682.1	0.586643336	8.85E-51	postive
TRAJ21	AC018682.1	0.658198993	1.01E-67	postive
TRAJ31	AC018682.1	0.660263896	2.78E-68	postive
TRAJ37	AC018682.1	0.617675118	1.36E-57	postive
TRAJ38	AC018682.1	0.702437485	9.55E-81	postive
TRAJ39	AC018682.1	0.673582668	5.39E-72	postive
CREB1	N4BP2L2-IT2	0.793341927	5.68E-117	postive
RFXAP	N4BP2L2-IT2	0.551692374	6.16E-44	postive
UBR1	N4BP2L2-IT2	0.657466977	1.58E-67	postive

ZC3HAV1	N4BP2L2-IT2	0.579066369	3.17E-49	postive
IL15	N4BP2L2-IT2	0.585977099	1.22E-50	postive
CYLD	N4BP2L2-IT2	0.635143774	8.96E-62	postive
EIF2AK2	N4BP2L2-IT2	0.577603699	6.25E-49	postive
MAPK8	N4BP2L2-IT2	0.579299358	2.84E-49	postive
LMBR1	N4BP2L2-IT2	0.558932261	2.74E-45	postive
IREB2	N4BP2L2-IT2	0.624673707	3.09E-59	postive
DDX17	N4BP2L2-IT2	0.662063346	8.99E-69	postive
PIK3CG	N4BP2L2-IT2	0.527003476	1.44E-39	postive
JAK2	N4BP2L2-IT2	0.635948036	5.67E-62	postive
TXK	N4BP2L2-IT2	0.638914456	1.03E-62	postive
NFAT5	N4BP2L2-IT2	0.873002251	2.46E-168	postive
NFATC3	N4BP2L2-IT2	0.500792002	2.62E-35	postive
MALT1	N4BP2L2-IT2	0.551145599	7.77E-44	postive
PIK3R1	N4BP2L2-IT2	0.683510867	6.84E-75	postive
PIK3CA	N4BP2L2-IT2	0.699965263	5.90E-80	postive
PIK3CB	N4BP2L2-IT2	0.537777427	1.98E-41	postive
AKT3	N4BP2L2-IT2	0.520949272	1.50E-38	postive
RASGRP3	N4BP2L2-IT2	0.555022615	1.49E-44	postive
IGHD4-4	N4BP2L2-IT2	0.526804017	1.55E-39	postive
IGHD6-6	N4BP2L2-IT2	0.566556958	9.53E-47	postive
PLXNC1	N4BP2L2-IT2	0.511321025	5.63E-37	postive
GNRH1	N4BP2L2-IT2	0.787947244	2.46E-114	postive
IL6ST	N4BP2L2-IT2	0.677082852	5.29E-73	postive
RABEP1	N4BP2L2-IT2	0.566092779	1.17E-46	postive
ACVR2A	N4BP2L2-IT2	0.691567798	2.50E-77	postive
ANGPTL1	N4BP2L2-IT2	0.776673429	4.58E-109	postive
BMPR1A	N4BP2L2-IT2	0.565260346	1.70E-46	postive
BMPR2	N4BP2L2-IT2	0.762561306	6.88E-103	postive
CRLF3	N4BP2L2-IT2	0.642539997	1.26E-63	postive
NR1D2	N4BP2L2-IT2	0.524229564	4.23E-39	postive
NR2C2	N4BP2L2-IT2	0.744436055	1.46E-95	postive
RORA	N4BP2L2-IT2	0.892168961	4.20E-186	postive
SOS1	N4BP2L2-IT2	0.706018625	6.60E-82	postive
SOS2	N4BP2L2-IT2	0.597326165	4.84E-53	postive
BRAF	N4BP2L2-IT2	0.831552602	3.25E-138	postive
ITK	N4BP2L2-IT2	0.552638165	4.12E-44	postive
CD28	N4BP2L2-IT2	0.515432319	1.21E-37	postive
CBL	N4BP2L2-IT2	0.593070985	3.95E-52	postive
CBLB	N4BP2L2-IT2	0.70745526	2.23E-82	postive
RASGRP1	N4BP2L2-IT2	0.548124051	2.78E-43	postive
PDK1	N4BP2L2-IT2	0.578808146	3.57E-49	postive
TRAJ1	N4BP2L2-IT2	0.780536002	7.77E-111	postive
TRAJ2	N4BP2L2-IT2	0.743121728	4.71E-95	postive
TRAJ3	N4BP2L2-IT2	0.823547485	2.42E-133	postive
TRAJ5	N4BP2L2-IT2	0.864330241	3.18E-161	postive
TRAJ6	N4BP2L2-IT2	0.823708309	1.94E-133	postive
TRAJ8	N4BP2L2-IT2	0.820675736	1.18E-131	postive
TRAJ10	N4BP2L2-IT2	0.808919993	4.74E-125	postive
TRAJ12	N4BP2L2-IT2	0.772207424	4.61E-107	postive
TRAJ13	N4BP2L2-IT2	0.840771553	3.75E-144	postive
TRAJ14	N4BP2L2-IT2	0.813207375	2.10E-127	postive
TRAJ16	N4BP2L2-IT2	0.828366173	3.03E-136	postive
TRAJ17	N4BP2L2-IT2	0.817930673	4.53E-130	postive
TRAJ18	N4BP2L2-IT2	0.780660636	6.81E-111	postive
TRAJ21	N4BP2L2-IT2	0.868903409	6.53E-165	postive
TRAJ31	N4BP2L2-IT2	0.89145694	2.20E-185	postive
TRAJ37	N4BP2L2-IT2	0.867151096	1.75E-163	postive
TRAJ38	N4BP2L2-IT2	0.889460118	2.14E-183	postive
TRAJ39	N4BP2L2-IT2	0.891101825	5.00E-185	postive
LTA	AC012645.3	0.53366287	1.03E-40	postive
CIITA	AC012645.3	0.52089752	1.53E-38	postive
TYK2	AC012645.3	0.556847355	6.77E-45	postive
IRF9	AC012645.3	0.522409653	8.53E-39	postive
PDCD1	AC012645.3	0.540597235	6.27E-42	postive

CCR7	AC012645.3	0.502054009	1.66E-35	postive
PTK2B	AC012645.3	0.517930366	4.72E-38	postive
CD19	AC012645.3	0.587019344	7.39E-51	postive
PIK3CD	AC012645.3	0.626924402	8.98E-60	postive
CD22	AC012645.3	0.513588362	2.42E-37	postive
PTPN6	AC012645.3	0.512785696	3.27E-37	postive
PLCG2	AC012645.3	0.51470522	1.59E-37	postive
IGLJ1	AC012645.3	0.534279805	8.08E-41	postive
SEMA4D	AC012645.3	0.543769472	1.70E-42	postive
FLT3LG	AC012645.3	0.550737443	9.23E-44	postive
IL16	AC012645.3	0.57398221	3.32E-48	postive
IL10RA	AC012645.3	0.544017369	1.54E-42	postive
IL12RB1	AC012645.3	0.561381345	9.41E-46	postive
IL21R	AC012645.3	0.513066995	2.94E-37	postive
TNFRSF13C	AC012645.3	0.52549268	2.59E-39	postive
TNFRSF14	AC012645.3	0.503158237	1.12E-35	postive
ITGAL	AC012645.3	0.655941169	4.06E-67	postive
CD247	AC012645.3	0.563380694	3.90E-46	postive
ZAP70	AC012645.3	0.775569857	1.44E-108	postive
CD3E	AC012645.3	0.543488967	1.91E-42	postive
TRAC	AC012645.3	0.516628965	7.73E-38	postive
TRBC1	AC012645.3	0.620054928	3.80E-58	postive
TRBJ2-2	AC012645.3	0.540903377	5.53E-42	postive
TRBJ2-7	AC012645.3	0.671884981	1.64E-71	postive
TRBV28	AC012645.3	0.540007017	7.98E-42	postive
CRIM1	AC245041.2	0.635431787	7.61E-62	postive
TGFBR3	AC245041.2	0.628699253	3.36E-60	postive
CREB1	AC004908.3	0.554005766	2.30E-44	postive
DDX17	AC004908.3	0.539997095	8.01E-42	postive
NFAT5	AC004908.3	0.624432837	3.53E-59	postive
PIK3R1	AC004908.3	0.501049794	2.39E-35	postive
IGHD4-4	AC004908.3	0.538901569	1.25E-41	postive
GNRH1	AC004908.3	0.653048672	2.38E-66	postive
IL6ST	AC004908.3	0.504626456	6.57E-36	postive
ACVR2A	AC004908.3	0.557399644	5.34E-45	postive
ANGPTL1	AC004908.3	0.510488289	7.67E-37	postive
BMPR2	AC004908.3	0.539928398	8.24E-42	postive
NR2C2	AC004908.3	0.582528959	6.25E-50	postive
RORA	AC004908.3	0.6507872	9.38E-66	postive
BRAF	AC004908.3	0.589813419	1.93E-51	postive
TRAJ2	AC004908.3	0.563415607	3.84E-46	postive
TRAJ3	AC004908.3	0.601441788	6.18E-54	postive
TRAJ5	AC004908.3	0.529356459	5.71E-40	postive
TRAJ6	AC004908.3	0.554905039	1.56E-44	postive
TRAJ8	AC004908.3	0.552224183	4.91E-44	postive
TRAJ10	AC004908.3	0.563034436	4.55E-46	postive
TRAJ12	AC004908.3	0.527027882	1.42E-39	postive
TRAJ13	AC004908.3	0.532816298	1.45E-40	postive
TRAJ14	AC004908.3	0.522505418	8.23E-39	postive
TRAJ16	AC004908.3	0.517678864	5.20E-38	postive
TRAJ17	AC004908.3	0.537050219	2.65E-41	postive
TRAJ18	AC004908.3	0.511800093	4.72E-37	postive
TRAJ21	AC004908.3	0.547770539	3.22E-43	postive
TRAJ31	AC004908.3	0.602161037	4.30E-54	postive
TRAJ37	AC004908.3	0.623374628	6.29E-59	postive
TRAJ38	AC004908.3	0.597629465	4.17E-53	postive
TRAJ39	AC004908.3	0.574046247	3.22E-48	postive
CREB1	MCCC1-AS1	0.583681807	3.62E-50	postive
RFXAP	MCCC1-AS1	0.532097376	1.93E-40	postive
UBR1	MCCC1-AS1	0.50678442	2.99E-36	postive
CYLD	MCCC1-AS1	0.528515857	7.95E-40	postive
DDX17	MCCC1-AS1	0.667066583	3.73E-70	postive
NFAT5	MCCC1-AS1	0.728054997	1.88E-89	postive
LTB4R2	MCCC1-AS1	0.644704364	3.53E-64	postive
GNRH1	MCCC1-AS1	0.740881728	3.39E-94	postive

ACVR2A	MCCC1-AS1	0.533696565	1.02E-40	postive
ANGPTL1	MCCC1-AS1	0.552361589	4.64E-44	postive
BMPR2	MCCC1-AS1	0.504284012	7.44E-36	postive
NR2C1	MCCC1-AS1	0.518585238	3.68E-38	postive
NR2C2	MCCC1-AS1	0.682710494	1.18E-74	postive
RORA	MCCC1-AS1	0.669820386	6.31E-71	postive
SOS1	MCCC1-AS1	0.514609877	1.65E-37	postive
BRAF	MCCC1-AS1	0.634111869	1.61E-61	postive
CBLB	MCCC1-AS1	0.547343604	3.85E-43	postive
TRAJ1	MCCC1-AS1	0.608408722	1.77E-55	postive
TRAJ2	MCCC1-AS1	0.63953951	7.20E-63	postive
TRAJ3	MCCC1-AS1	0.663462346	3.72E-69	postive
TRAJ5	MCCC1-AS1	0.587497841	5.87E-51	postive
TRAJ6	MCCC1-AS1	0.645637727	2.03E-64	postive
TRAJ8	MCCC1-AS1	0.606947057	3.75E-55	postive
TRAJ10	MCCC1-AS1	0.6414275	2.41E-63	postive
TRAJ12	MCCC1-AS1	0.57275776	5.81E-48	postive
TRAJ13	MCCC1-AS1	0.60112708	7.24E-54	postive
TRAJ14	MCCC1-AS1	0.60223758	4.14E-54	postive
TRAJ16	MCCC1-AS1	0.641141955	2.84E-63	postive
TRAJ17	MCCC1-AS1	0.595597549	1.14E-52	postive
TRAJ18	MCCC1-AS1	0.604467548	1.33E-54	postive
TRAJ21	MCCC1-AS1	0.627901487	5.23E-60	postive
TRAJ31	MCCC1-AS1	0.635455868	7.50E-62	postive
TRAJ37	MCCC1-AS1	0.660475201	2.44E-68	postive
TRAJ38	MCCC1-AS1	0.678906061	1.56E-73	postive
TRAJ39	MCCC1-AS1	0.64728938	7.63E-65	postive
CREB1	NFYC-AS1	0.584328226	2.67E-50	postive
RFXAP	NFYC-AS1	0.602378544	3.85E-54	postive
UBR1	NFYC-AS1	0.535291512	5.39E-41	postive
ZC3HAV1L	NFYC-AS1	0.511275163	5.73E-37	postive
DDX17	NFYC-AS1	0.66730549	3.20E-70	postive
NFAT5	NFYC-AS1	0.688264051	2.55E-76	postive
LTB4R2	NFYC-AS1	0.523212796	6.26E-39	postive
GNRH1	NFYC-AS1	0.773249715	1.59E-107	postive
RABEP1	NFYC-AS1	0.505521409	4.74E-36	postive
ACVR2A	NFYC-AS1	0.541846111	3.76E-42	postive
CRLF3	NFYC-AS1	0.529033537	6.48E-40	postive
NR2C1	NFYC-AS1	0.59053162	1.36E-51	postive
NR2C2	NFYC-AS1	0.718703058	3.67E-86	postive
RORA	NFYC-AS1	0.615001617	5.64E-57	postive
SOS1	NFYC-AS1	0.555812118	1.06E-44	postive
BRAF	NFYC-AS1	0.660143742	3.00E-68	postive
CBLB	NFYC-AS1	0.513408029	2.59E-37	postive
TRAJ1	NFYC-AS1	0.618301242	9.74E-58	postive
TRAJ2	NFYC-AS1	0.642073206	1.65E-63	postive
TRAJ3	NFYC-AS1	0.6600038	3.27E-68	postive
TRAJ5	NFYC-AS1	0.550865408	8.75E-44	postive
TRAJ6	NFYC-AS1	0.629821154	1.80E-60	postive
TRAJ8	NFYC-AS1	0.629883435	1.74E-60	postive
TRAJ10	NFYC-AS1	0.570145043	1.90E-47	postive
TRAJ12	NFYC-AS1	0.523520183	5.56E-39	postive
TRAJ13	NFYC-AS1	0.585000578	1.94E-50	postive
TRAJ14	NFYC-AS1	0.587050044	7.28E-51	postive
TRAJ16	NFYC-AS1	0.592928611	4.23E-52	postive
TRAJ17	NFYC-AS1	0.538137658	1.71E-41	postive
TRAJ18	NFYC-AS1	0.502325944	1.51E-35	postive
TRAJ21	NFYC-AS1	0.593282048	3.56E-52	postive
TRAJ31	NFYC-AS1	0.570624255	1.53E-47	postive
TRAJ37	NFYC-AS1	0.61233302	2.30E-56	postive
TRAJ38	NFYC-AS1	0.627383484	6.97E-60	postive
TRAJ39	NFYC-AS1	0.631810291	5.90E-61	postive
CREB1	AL021707.7	0.621392751	1.85E-58	postive
UBR1	AL021707.7	0.50707602	2.69E-36	postive
ZC3HAV1	AL021707.7	0.506816217	2.96E-36	postive

IL15	AL021707.7	0.532582845	1.59E-40	postive
CYLD	AL021707.7	0.564172029	2.75E-46	postive
NFKBIZ	AL021707.7	0.578273962	4.58E-49	postive
DDX17	AL021707.7	0.730539657	2.38E-90	postive
JAK2	AL021707.7	0.573722665	3.74E-48	postive
TXK	AL021707.7	0.528705012	7.38E-40	postive
NFAT5	AL021707.7	0.692098816	1.72E-77	postive
PIK3R1	AL021707.7	0.525718714	2.37E-39	postive
PIK3CA	AL021707.7	0.57960503	2.46E-49	postive
RASGRP3	AL021707.7	0.551204535	7.58E-44	postive
LTB4R2	AL021707.7	0.557209208	5.79E-45	postive
GNRH1	AL021707.7	0.736617577	1.37E-92	postive
ACVR2A	AL021707.7	0.563790537	3.26E-46	postive
ANGPTL1	AL021707.7	0.6361013	5.19E-62	postive
BMPR2	AL021707.7	0.585038045	1.90E-50	postive
CRLF3	AL021707.7	0.512304208	3.91E-37	postive
NR2C1	AL021707.7	0.539611215	9.38E-42	postive
NR2C2	AL021707.7	0.72281893	1.36E-87	postive
RORA	AL021707.7	0.694266554	3.66E-78	postive
SOS1	AL021707.7	0.591317299	9.28E-52	postive
SOS2	AL021707.7	0.518744366	3.47E-38	postive
BRAF	AL021707.7	0.662922436	5.23E-69	postive
ITK	AL021707.7	0.520914715	1.52E-38	postive
CBL	AL021707.7	0.550952221	8.43E-44	postive
CBLB	AL021707.7	0.649467141	2.07E-65	postive
TRAJ1	AL021707.7	0.66498976	1.41E-69	postive
TRAJ2	AL021707.7	0.596959991	5.81E-53	postive
TRAJ3	AL021707.7	0.688730181	1.84E-76	postive
TRAJ5	AL021707.7	0.679171135	1.30E-73	postive
TRAJ6	AL021707.7	0.667394076	3.02E-70	postive
TRAJ8	AL021707.7	0.621583811	1.66E-58	postive
TRAJ10	AL021707.7	0.656057199	3.78E-67	postive
TRAJ12	AL021707.7	0.651410544	6.43E-66	postive
TRAJ13	AL021707.7	0.644717086	3.50E-64	postive
TRAJ14	AL021707.7	0.644075465	5.11E-64	postive
TRAJ16	AL021707.7	0.675095024	1.98E-72	postive
TRAJ17	AL021707.7	0.660725175	2.08E-68	postive
TRAJ18	AL021707.7	0.656311519	3.23E-67	postive
TRAJ21	AL021707.7	0.678115477	2.65E-73	postive
TRAJ31	AL021707.7	0.692449722	1.34E-77	postive
TRAJ37	AL021707.7	0.649511795	2.02E-65	postive
TRAJ38	AL021707.7	0.710192729	2.79E-83	postive
TRAJ39	AL021707.7	0.672760224	9.26E-72	postive
CXCL13	LINC01215	0.503897057	8.55E-36	postive
RBP5	LINC01215	0.531312712	2.64E-40	postive
TNFAIP3	LINC01215	0.527323822	1.27E-39	postive
IL7R	LINC01215	0.564197747	2.72E-46	postive
CCR7	LINC01215	0.537911687	1.87E-41	postive
CD19	LINC01215	0.510993307	6.36E-37	postive
CD22	LINC01215	0.514551438	1.69E-37	postive
LTB	LINC01215	0.509993872	9.21E-37	postive
TNFSF8	LINC01215	0.520732264	1.62E-38	postive
TNFRSF13C	LINC01215	0.568037974	4.91E-47	postive
NCR3	LINC01215	0.509501761	1.10E-36	postive
PTPRC	LINC01215	0.506849485	2.92E-36	postive
ITK	LINC01215	0.533481608	1.11E-40	postive
LTA	LINC00926	0.540588226	6.29E-42	postive
PTGDS	LINC00926	0.601534378	5.90E-54	postive
RBP5	LINC00926	0.60207698	4.49E-54	postive
CD40LG	LINC00926	0.559904244	1.80E-45	postive
CCL19	LINC00926	0.507627778	2.20E-36	postive
CCR7	LINC00926	0.585696525	1.39E-50	postive
CXCR4	LINC00926	0.53951697	9.75E-42	postive
PTK2B	LINC00926	0.506622978	3.18E-36	postive
CD79B	LINC00926	0.582108001	7.62E-50	postive

CD19	LINC00926	0.79842829	1.57E-119	postive
CR2	LINC00926	0.522861525	7.17E-39	postive
CD22	LINC00926	0.764141214	1.47E-103	postive
PLCG2	LINC00926	0.574855882	2.22E-48	postive
IL16	LINC00926	0.593108911	3.87E-52	postive
IL24	LINC00926	0.510478614	7.70E-37	postive
LTB	LINC00926	0.526459863	1.78E-39	postive
TNFRSF13C	LINC00926	0.795215733	6.61E-118	postive
ITGAL	LINC00926	0.507702513	2.14E-36	postive
NCR3	LINC00926	0.607743541	2.49E-55	postive
ZAP70	LINC00926	0.609278536	1.13E-55	postive
ITK	LINC00926	0.514696194	1.60E-37	postive
TRBC1	LINC00926	0.505352232	5.05E-36	postive
TRBJ2-7	LINC00926	0.550567754	9.92E-44	postive
CREB1	AL133243.2	0.806296849	1.22E-123	postive
RFXAP	AL133243.2	0.510764317	6.92E-37	postive
UBR1	AL133243.2	0.638655099	1.20E-62	postive
ZC3HAV1	AL133243.2	0.59804474	3.39E-53	postive
ZC3HAV1L	AL133243.2	0.552416766	4.53E-44	postive
IL15	AL133243.2	0.607880874	2.32E-55	postive
CYLD	AL133243.2	0.593091521	3.91E-52	postive
EIF2AK2	AL133243.2	0.613316209	1.37E-56	postive
MAPK8	AL133243.2	0.584245052	2.77E-50	postive
TLR1	AL133243.2	0.532527906	1.63E-40	postive
LMBR1	AL133243.2	0.62783515	5.43E-60	postive
LIMS1	AL133243.2	0.563945592	3.04E-46	postive
IREB2	AL133243.2	0.642047428	1.68E-63	postive
DDX17	AL133243.2	0.577368098	6.97E-49	postive
PIK3CG	AL133243.2	0.554301762	2.02E-44	postive
JAK2	AL133243.2	0.671435706	2.20E-71	postive
TXK	AL133243.2	0.586162806	1.11E-50	postive
NFAT5	AL133243.2	0.812084964	8.78E-127	postive
MALT1	AL133243.2	0.552217992	4.93E-44	postive
PIK3R1	AL133243.2	0.634758708	1.12E-61	postive
PIK3CA	AL133243.2	0.7452242	7.24E-96	postive
PIK3CB	AL133243.2	0.573264803	4.61E-48	postive
AKT3	AL133243.2	0.585990002	1.21E-50	postive
GSK3B	AL133243.2	0.52020697	1.99E-38	postive
RASGRP3	AL133243.2	0.54446844	1.27E-42	postive
PLXNC1	AL133243.2	0.533376959	1.16E-40	postive
GNRH1	AL133243.2	0.713818152	1.70E-84	postive
IL6ST	AL133243.2	0.614450884	7.54E-57	postive
RABEP1	AL133243.2	0.567549178	6.11E-47	postive
ACVR2A	AL133243.2	0.627803081	5.52E-60	postive
ANGPTL1	AL133243.2	0.812288108	6.78E-127	postive
BMPR1A	AL133243.2	0.554785397	1.65E-44	postive
BMPR2	AL133243.2	0.735242038	4.47E-92	postive
CRLF3	AL133243.2	0.704775753	1.68E-81	postive
LIFR	AL133243.2	0.510959205	6.44E-37	postive
NR2C2	AL133243.2	0.683451601	7.12E-75	postive
RORA	AL133243.2	0.806284795	1.24E-123	postive
SOS1	AL133243.2	0.742595811	7.50E-95	postive
SOS2	AL133243.2	0.612635619	1.96E-56	postive
BRAF	AL133243.2	0.841800553	7.73E-145	postive
ITK	AL133243.2	0.524098425	4.45E-39	postive
CBL	AL133243.2	0.654919583	7.60E-67	postive
CBLB	AL133243.2	0.790640771	1.21E-115	postive
RASGRP1	AL133243.2	0.514723157	1.58E-37	postive
PDK1	AL133243.2	0.635681924	6.60E-62	postive
TRAJ1	AL133243.2	0.746572972	2.16E-96	postive
TRAJ2	AL133243.2	0.74006821	6.91E-94	postive
TRAJ3	AL133243.2	0.789234706	5.87E-115	postive
TRAJ5	AL133243.2	0.82170442	2.95E-132	postive
TRAJ6	AL133243.2	0.802375769	1.44E-121	postive
TRAJ8	AL133243.2	0.789187095	6.20E-115	postive

TRAJ10	AL133243.2	0.773303162	1.50E-107	postive
TRAJ12	AL133243.2	0.716450194	2.17E-85	postive
TRAJ13	AL133243.2	0.808990113	4.35E-125	postive
TRAJ14	AL133243.2	0.803530023	3.57E-122	postive
TRAJ16	AL133243.2	0.800947413	7.95E-121	postive
TRAJ17	AL133243.2	0.763265139	3.46E-103	postive
TRAJ18	AL133243.2	0.722256329	2.14E-87	postive
TRAJ21	AL133243.2	0.832824737	5.18E-139	postive
TRAJ31	AL133243.2	0.841280391	1.72E-144	postive
TRAJ37	AL133243.2	0.791844	3.12E-116	postive
TRAJ38	AL133243.2	0.879549854	4.62E-174	postive
TRAJ39	AL133243.2	0.839800184	1.65E-143	postive
KCNH2	MCF2L-AS1	0.546704045	5.03E-43	postive
LMBR1L	AC006435.2	0.544907271	1.06E-42	postive
DDX17	AC006435.2	0.546951986	4.53E-43	postive
IRF9	AC006435.2	0.59062826	1.30E-51	postive
LTB4R2	AC006435.2	0.545232097	9.28E-43	postive
GNRH1	AC006435.2	0.545185067	9.47E-43	postive
GIPR	AC006435.2	0.547832094	3.14E-43	postive
NR2C2	AC006435.2	0.557937859	4.23E-45	postive
TNFRSF25	H1FX-AS1	0.502483773	1.43E-35	postive
DDX17	AC026367.3	0.516412021	8.39E-38	postive
GNRH1	AC026367.3	0.598339685	2.93E-53	postive
NR2C2	AC026367.3	0.533075534	1.31E-40	postive
CREB1	AC007014.2	0.722865737	1.31E-87	postive
UBR1	AC007014.2	0.552840046	3.78E-44	postive
ZC3HAV1	AC007014.2	0.542981741	2.36E-42	postive
IL15	AC007014.2	0.561490103	8.97E-46	postive
CYLD	AC007014.2	0.569629762	2.40E-47	postive
EIF2AK2	AC007014.2	0.52504945	3.08E-39	postive
MAPK8	AC007014.2	0.52635325	1.85E-39	postive
LMBR1	AC007014.2	0.562132278	6.77E-46	postive
IREB2	AC007014.2	0.524238207	4.21E-39	postive
DDX17	AC007014.2	0.554153794	2.16E-44	postive
JAK2	AC007014.2	0.577864564	5.54E-49	postive
TXK	AC007014.2	0.544732131	1.14E-42	postive
NFAT5	AC007014.2	0.800116908	2.13E-120	postive
MALT1	AC007014.2	0.515093867	1.38E-37	postive
PIK3R1	AC007014.2	0.605873611	6.51E-55	postive
PIK3CA	AC007014.2	0.665345629	1.12E-69	postive
AKT3	AC007014.2	0.524539137	3.75E-39	postive
RASGRP3	AC007014.2	0.506945312	2.82E-36	postive
GNRH1	AC007014.2	0.682289666	1.58E-74	postive
IL6ST	AC007014.2	0.634703998	1.15E-61	postive
ACVR2A	AC007014.2	0.58023589	1.83E-49	postive
ANGPTL1	AC007014.2	0.765142833	5.50E-104	postive
BMPR2	AC007014.2	0.69992172	6.09E-80	postive
CRLF3	AC007014.2	0.626677552	1.03E-59	postive
NR2C2	AC007014.2	0.614160451	8.79E-57	postive
RORA	AC007014.2	0.8470282	2.11E-148	postive
SOS1	AC007014.2	0.636588021	3.93E-62	postive
SOS2	AC007014.2	0.543202716	2.15E-42	postive
BRAF	AC007014.2	0.795010927	8.37E-118	postive
ITK	AC007014.2	0.539569571	9.54E-42	postive
CBL	AC007014.2	0.577941063	5.35E-49	postive
CBLB	AC007014.2	0.712393355	5.13E-84	postive
PDK1	AC007014.2	0.563750058	3.32E-46	postive
TRAJ1	AC007014.2	0.695236103	1.83E-78	postive
TRAJ2	AC007014.2	0.711708566	8.70E-84	postive
TRAJ3	AC007014.2	0.741340943	2.27E-94	postive
TRAJ5	AC007014.2	0.820922509	8.45E-132	postive
TRAJ6	AC007014.2	0.749547675	1.46E-97	postive
TRAJ8	AC007014.2	0.762851518	5.19E-103	postive
TRAJ10	AC007014.2	0.738848805	2.00E-93	postive
TRAJ12	AC007014.2	0.656460433	2.95E-67	postive

TRAJ13	AC007014.2	0.802371582	1.45E-121	postive
TRAJ14	AC007014.2	0.759241184	1.69E-101	postive
TRAJ16	AC007014.2	0.77039953	2.89E-106	postive
TRAJ17	AC007014.2	0.742885013	5.81E-95	postive
TRAJ18	AC007014.2	0.688058479	2.95E-76	postive
TRAJ21	AC007014.2	0.814622224	3.40E-128	postive
TRAJ31	AC007014.2	0.843618759	4.61E-146	postive
TRAJ37	AC007014.2	0.805008763	5.93E-123	postive
TRAJ38	AC007014.2	0.840472355	5.93E-144	postive
TRAJ39	AC007014.2	0.834360386	5.53E-140	postive
HTR3A	SLCO4A1-AS1	0.542850847	2.49E-42	postive
CREB1	AP000866.6	0.754918123	1.01E-99	postive
RFXAP	AP000866.6	0.539729099	8.94E-42	postive
UBR1	AP000866.6	0.647313698	7.52E-65	postive
ZC3HAV1	AP000866.6	0.517363988	5.85E-38	postive
CYLD	AP000866.6	0.619987442	3.94E-58	postive
MAPK8	AP000866.6	0.547174437	4.13E-43	postive
LMBR1	AP000866.6	0.521216521	1.35E-38	postive
IREB2	AP000866.6	0.580520752	1.61E-49	postive
DDX17	AP000866.6	0.630908779	9.79E-61	postive
JAK2	AP000866.6	0.575451735	1.69E-48	postive
TXK	AP000866.6	0.531047017	2.93E-40	postive
NFAT5	AP000866.6	0.904592884	1.61E-199	postive
NFATC3	AP000866.6	0.569282615	2.81E-47	postive
PIK3R1	AP000866.6	0.584192354	2.84E-50	postive
PIK3CA	AP000866.6	0.59188427	7.04E-52	postive
AKT3	AP000866.6	0.584616493	2.33E-50	postive
ROBO2	AP000866.6	0.500581974	2.82E-35	postive
GNRH1	AP000866.6	0.759187307	1.78E-101	postive
IL6ST	AP000866.6	0.591467299	8.63E-52	postive
RABEP1	AP000866.6	0.568649071	3.73E-47	postive
ACVR2A	AP000866.6	0.643016095	9.52E-64	postive
ANGPTL1	AP000866.6	0.670013045	5.57E-71	postive
BMPR1A	AP000866.6	0.577661276	6.09E-49	postive
BMPR2	AP000866.6	0.689497108	1.08E-76	postive
CRLF3	AP000866.6	0.652009235	4.48E-66	postive
LIFR	AP000866.6	0.511011736	6.32E-37	postive
NR1D2	AP000866.6	0.529149743	6.19E-40	postive
NR2C1	AP000866.6	0.534211255	8.31E-41	postive
NR2C2	AP000866.6	0.654364306	1.07E-66	postive
RORA	AP000866.6	0.803594115	3.31E-122	postive
SOS1	AP000866.6	0.645403549	2.33E-64	postive
SOS2	AP000866.6	0.548963231	1.95E-43	postive
BRAF	AP000866.6	0.786077783	1.94E-113	postive
CBL	AP000866.6	0.566641488	9.17E-47	postive
CBLB	AP000866.6	0.629269229	2.45E-60	postive
TRAJ1	AP000866.6	0.772779848	2.57E-107	postive
TRAJ2	AP000866.6	0.849187092	6.51E-150	postive
TRAJ3	AP000866.6	0.838529321	1.13E-142	postive
TRAJ5	AP000866.6	0.747950268	6.22E-97	postive
TRAJ6	AP000866.6	0.854621168	8.00E-154	postive
TRAJ8	AP000866.6	0.847252004	1.47E-148	postive
TRAJ10	AP000866.6	0.80114944	6.25E-121	postive
TRAJ12	AP000866.6	0.748054665	5.66E-97	postive
TRAJ13	AP000866.6	0.843480916	5.71E-146	postive
TRAJ14	AP000866.6	0.817156436	1.25E-129	postive
TRAJ16	AP000866.6	0.817060193	1.42E-129	postive
TRAJ17	AP000866.6	0.748424593	4.05E-97	postive
TRAJ18	AP000866.6	0.739133904	1.56E-93	postive
TRAJ21	AP000866.6	0.837066331	1.01E-141	postive
TRAJ31	AP000866.6	0.843597579	4.76E-146	postive
TRAJ37	AP000866.6	0.872491067	6.66E-168	postive
TRAJ38	AP000866.6	0.878253463	6.67E-173	postive
TRAJ39	AP000866.6	0.836574999	2.10E-141	postive
CREB1	AC006213.4	0.502052041	1.66E-35	postive

BRAF	AC006213.4	0.52010843	2.06E-38	postive
TNFRSF10A	TNFRSF10A-AS1	0.828068739	4.60E-136	postive
TNFRSF10B	TNFRSF10A-AS1	0.675402917	1.62E-72	postive
CREB1	AC053527.2	0.597808321	3.81E-53	postive
UBR1	AC053527.2	0.5162757	8.83E-38	postive
IL15	AC053527.2	0.55002534	1.25E-43	postive
IREB2	AC053527.2	0.522993439	6.82E-39	postive
NFAT5	AC053527.2	0.584234251	2.79E-50	postive
PIK3R1	AC053527.2	0.537483011	2.23E-41	postive
PIK3CA	AC053527.2	0.505569765	4.66E-36	postive
IGHD4-4	AC053527.2	0.595994793	9.37E-53	postive
GNRH1	AC053527.2	0.563242118	4.15E-46	postive
ACVR2A	AC053527.2	0.543209631	2.14E-42	postive
ANGPTL1	AC053527.2	0.597336346	4.82E-53	postive
BMPR2	AC053527.2	0.559696257	1.97E-45	postive
NR2C2	AC053527.2	0.599207157	1.90E-53	postive
RORA	AC053527.2	0.634891906	1.03E-61	postive
SOS1	AC053527.2	0.532196332	1.86E-40	postive
BRAF	AC053527.2	0.66360545	3.39E-69	postive
CBLB	AC053527.2	0.516539998	7.99E-38	postive
TRAJ1	AC053527.2	0.534039681	8.90E-41	postive
TRAJ2	AC053527.2	0.531339451	2.61E-40	postive
TRAJ3	AC053527.2	0.635737434	6.39E-62	postive
TRAJ5	AC053527.2	0.598059246	3.36E-53	postive
TRAJ6	AC053527.2	0.577308608	7.17E-49	postive
TRAJ8	AC053527.2	0.572385065	6.88E-48	postive
TRAJ10	AC053527.2	0.581158123	1.19E-49	postive
TRAJ12	AC053527.2	0.590752388	1.22E-51	postive
TRAJ13	AC053527.2	0.544576623	1.22E-42	postive
TRAJ14	AC053527.2	0.579630664	2.43E-49	postive
TRAJ16	AC053527.2	0.537587171	2.13E-41	postive
TRAJ17	AC053527.2	0.585319276	1.66E-50	postive
TRAJ18	AC053527.2	0.548357465	2.52E-43	postive
TRAJ21	AC053527.2	0.591625696	7.99E-52	postive
TRAJ31	AC053527.2	0.625196711	2.32E-59	postive
TRAJ37	AC053527.2	0.607720347	2.52E-55	postive
TRAJ38	AC053527.2	0.641868921	1.86E-63	postive
TRAJ39	AC053527.2	0.630400814	1.30E-60	postive
BMPR2	HCG11	0.518248729	4.19E-38	postive
SOS1	HCG11	0.517618406	5.32E-38	postive
C8G	SAPCD1-AS1	0.571344554	1.10E-47	postive
APOM	SAPCD1-AS1	0.515516223	1.18E-37	postive
VTN	SAPCD1-AS1	0.553418003	2.95E-44	postive
ALB	SAPCD1-AS1	0.563597171	3.55E-46	postive
AGTR1	SAPCD1-AS1	0.520049776	2.11E-38	postive
TRBV4-1	SAPCD1-AS1	0.50356016	9.66E-36	postive
CREB1	AC018926.2	0.61735284	1.62E-57	postive
UBR1	AC018926.2	0.568916745	3.31E-47	postive
IL15	AC018926.2	0.544906362	1.06E-42	postive
CYLD	AC018926.2	0.548679595	2.20E-43	postive
DDX17	AC018926.2	0.57330986	4.51E-48	postive
JAK2	AC018926.2	0.547281582	3.95E-43	postive
TXK	AC018926.2	0.549040255	1.89E-43	postive
NFAT5	AC018926.2	0.756489402	2.31E-100	postive
PIK3R1	AC018926.2	0.528959954	6.67E-40	postive
GNRH1	AC018926.2	0.712230118	5.82E-84	postive
IL6ST	AC018926.2	0.51310183	2.90E-37	postive
IL7	AC018926.2	0.512692568	3.38E-37	postive
ACVR2A	AC018926.2	0.506948076	2.82E-36	postive
ANGPTL1	AC018926.2	0.590141151	1.64E-51	postive
BMPR2	AC018926.2	0.573915194	3.42E-48	postive
CRLF3	AC018926.2	0.532970182	1.36E-40	postive
NR2C2	AC018926.2	0.589799338	1.94E-51	postive
RORA	AC018926.2	0.737440986	6.76E-93	postive
BRAF	AC018926.2	0.643307947	8.02E-64	postive

CBLB	AC018926.2	0.54752836	3.56E-43	postive
TRAJ1	AC018926.2	0.647846726	5.47E-65	postive
TRAJ2	AC018926.2	0.618239727	1.01E-57	postive
TRAJ3	AC018926.2	0.697305531	4.10E-79	postive
TRAJ5	AC018926.2	0.673666791	5.10E-72	postive
TRAJ6	AC018926.2	0.706600494	4.26E-82	postive
TRAJ8	AC018926.2	0.633446757	2.35E-61	postive
TRAJ10	AC018926.2	0.70072168	3.39E-80	postive
TRAJ12	AC018926.2	0.689999898	7.56E-77	postive
TRAJ13	AC018926.2	0.661374997	1.39E-68	postive
TRAJ14	AC018926.2	0.66313358	4.57E-69	postive
TRAJ16	AC018926.2	0.682843772	1.08E-74	postive
TRAJ17	AC018926.2	0.657406383	1.64E-67	postive
TRAJ18	AC018926.2	0.698671363	1.52E-79	postive
TRAJ21	AC018926.2	0.710802866	1.75E-83	postive
TRAJ31	AC018926.2	0.72135492	4.42E-87	postive
TRAJ37	AC018926.2	0.730883121	1.78E-90	postive
TRAJ38	AC018926.2	0.740974909	3.12E-94	postive
TRAJ39	AC018926.2	0.720440091	9.19E-87	postive
GNRH1	AC005840.4	0.563914822	3.08E-46	postive
CREB1	RAP2C-AS1	0.732208326	5.86E-91	postive
RFXAP	RAP2C-AS1	0.521542963	1.19E-38	postive
UBR1	RAP2C-AS1	0.620810183	2.53E-58	postive
ZC3HAV1	RAP2C-AS1	0.507119825	2.65E-36	postive
IFNAR1	RAP2C-AS1	0.534318483	7.96E-41	postive
CYLD	RAP2C-AS1	0.657552397	1.50E-67	postive
EIF2AK2	RAP2C-AS1	0.51155886	5.16E-37	postive
MAPK8	RAP2C-AS1	0.510211709	8.50E-37	postive
LMBR1	RAP2C-AS1	0.541661884	4.05E-42	postive
IREB2	RAP2C-AS1	0.557940967	4.22E-45	postive
DDX17	RAP2C-AS1	0.591960271	6.79E-52	postive
PIK3CG	RAP2C-AS1	0.547562527	3.51E-43	postive
JAK2	RAP2C-AS1	0.572258256	7.29E-48	postive
TXK	RAP2C-AS1	0.507540142	2.27E-36	postive
NFAT5	RAP2C-AS1	0.827201525	1.55E-135	postive
NFATC3	RAP2C-AS1	0.574710124	2.38E-48	postive
MALT1	RAP2C-AS1	0.50025632	3.17E-35	postive
PIK3R1	RAP2C-AS1	0.690029021	7.40E-77	postive
PIK3CA	RAP2C-AS1	0.616851557	2.11E-57	postive
AKT3	RAP2C-AS1	0.524405091	3.95E-39	postive
PLXNC1	RAP2C-AS1	0.533840034	9.64E-41	postive
GNRH1	RAP2C-AS1	0.629272684	2.44E-60	postive
IL6ST	RAP2C-AS1	0.636237252	4.80E-62	postive
RABEP1	RAP2C-AS1	0.546242204	6.10E-43	postive
ACVR2A	RAP2C-AS1	0.583436036	4.07E-50	postive
ANGPTL1	RAP2C-AS1	0.654882339	7.78E-67	postive
BMPR1A	RAP2C-AS1	0.540645078	6.15E-42	postive
BMPR2	RAP2C-AS1	0.691259897	3.11E-77	postive
CRLF3	RAP2C-AS1	0.572041028	8.05E-48	postive
NR1D2	RAP2C-AS1	0.579014182	3.25E-49	postive
NR2C2	RAP2C-AS1	0.655290481	6.06E-67	postive
NR3C1	RAP2C-AS1	0.50874095	1.46E-36	postive
RORA	RAP2C-AS1	0.812671687	4.16E-127	postive
SOS1	RAP2C-AS1	0.65843872	8.67E-68	postive
SOS2	RAP2C-AS1	0.609024768	1.29E-55	postive
BRAF	RAP2C-AS1	0.759497548	1.32E-101	postive
ITK	RAP2C-AS1	0.500875102	2.54E-35	postive
CD28	RAP2C-AS1	0.523417076	5.79E-39	postive
CBL	RAP2C-AS1	0.538864249	1.27E-41	postive
CBLB	RAP2C-AS1	0.622148554	1.23E-58	postive
TRAJ1	RAP2C-AS1	0.69540942	1.61E-78	postive
TRAJ2	RAP2C-AS1	0.734486403	8.51E-92	postive
TRAJ3	RAP2C-AS1	0.756132494	3.24E-100	postive
TRAJ5	RAP2C-AS1	0.715793674	3.64E-85	postive
TRAJ6	RAP2C-AS1	0.755176676	7.95E-100	postive

TRAJ8	RAP2C-AS1	0.72880531	1.01E-89	postive
TRAJ10	RAP2C-AS1	0.718984891	2.93E-86	postive
TRAJ12	RAP2C-AS1	0.688473826	2.20E-76	postive
TRAJ13	RAP2C-AS1	0.745229072	7.21E-96	postive
TRAJ14	RAP2C-AS1	0.730948668	1.69E-90	postive
TRAJ16	RAP2C-AS1	0.735855687	2.64E-92	postive
TRAJ17	RAP2C-AS1	0.682328026	1.53E-74	postive
TRAJ18	RAP2C-AS1	0.705558445	9.33E-82	postive
TRAJ21	RAP2C-AS1	0.760532325	4.90E-102	postive
TRAJ31	RAP2C-AS1	0.762799983	5.45E-103	postive
TRAJ37	RAP2C-AS1	0.781210169	3.78E-111	postive
TRAJ38	RAP2C-AS1	0.795881559	3.06E-118	postive
TRAJ39	RAP2C-AS1	0.771854397	6.61E-107	postive
CREB1	AC020915.3	0.536378423	3.48E-41	postive
NFAT5	AC020915.3	0.566854684	8.34E-47	postive
GNRH1	AC020915.3	0.659794442	3.73E-68	postive
ANGPTL1	AC020915.3	0.500814062	2.60E-35	postive
NR2C2	AC020915.3	0.622917724	8.07E-59	postive
RORA	AC020915.3	0.536317396	3.56E-41	postive
SOS1	AC020915.3	0.535473045	5.01E-41	postive
BRAF	AC020915.3	0.603268057	2.46E-54	postive
CBLB	AC020915.3	0.515160694	1.34E-37	postive
TRAJ1	AC020915.3	0.524332578	4.06E-39	postive
TRAJ3	AC020915.3	0.564516389	2.36E-46	postive
TRAJ5	AC020915.3	0.555228101	1.36E-44	postive
TRAJ6	AC020915.3	0.540493814	6.54E-42	postive
TRAJ8	AC020915.3	0.555416956	1.25E-44	postive
TRAJ10	AC020915.3	0.513662781	2.36E-37	postive
TRAJ13	AC020915.3	0.538253287	1.63E-41	postive
TRAJ14	AC020915.3	0.53204618	1.97E-40	postive
TRAJ16	AC020915.3	0.535714532	4.54E-41	postive
TRAJ17	AC020915.3	0.525525965	2.56E-39	postive
TRAJ21	AC020915.3	0.557920104	4.26E-45	postive
TRAJ31	AC020915.3	0.554879571	1.58E-44	postive
TRAJ37	AC020915.3	0.545844079	7.20E-43	postive
TRAJ38	AC020915.3	0.573680855	3.81E-48	postive
TRAJ39	AC020915.3	0.587963679	4.70E-51	postive
CD4	AC138207.5	0.528265766	8.77E-40	postive
FCER1G	AC138207.5	0.530884757	3.12E-40	postive
CYBB	AC138207.5	0.553411214	2.96E-44	postive
TLR7	AC138207.5	0.590067913	1.70E-51	postive
CCR5	AC138207.5	0.525923796	2.19E-39	postive
CD86	AC138207.5	0.561313316	9.70E-46	postive
BTK	AC138207.5	0.538868455	1.27E-41	postive
RASGRP3	AC138207.5	0.51614133	9.29E-38	postive
CMKLR1	AC138207.5	0.506632967	3.16E-36	postive
PLXNC1	AC138207.5	0.544961898	1.04E-42	postive
CSF1R	AC138207.5	0.533171306	1.26E-40	postive
IL10RA	AC138207.5	0.523508164	5.59E-39	postive
LCP2	AC138207.5	0.572346318	7.00E-48	postive
CREB1	AC087284.1	0.782737055	7.34E-112	postive
RFXAP	AC087284.1	0.511868387	4.60E-37	postive
UBR1	AC087284.1	0.638602264	1.24E-62	postive
ZC3HAV1	AC087284.1	0.547866826	3.09E-43	postive
ZC3HAV1L	AC087284.1	0.513466682	2.53E-37	postive
IL15	AC087284.1	0.64132164	2.56E-63	postive
CYLD	AC087284.1	0.589437189	2.31E-51	postive
EIF2AK2	AC087284.1	0.511875094	4.59E-37	postive
MAPK8	AC087284.1	0.561364948	9.48E-46	postive
LMBR1	AC087284.1	0.575986777	1.32E-48	postive
IREB2	AC087284.1	0.610193282	7.01E-56	postive
DDX17	AC087284.1	0.569327968	2.75E-47	postive
PIK3CG	AC087284.1	0.507980839	1.93E-36	postive
JAK2	AC087284.1	0.631415536	7.37E-61	postive
TXK	AC087284.1	0.539820377	8.61E-42	postive

NFAT5	AC087284.1	0.86536183	4.81E-162	postive
NFATC3	AC087284.1	0.503700739	9.18E-36	postive
MALT1	AC087284.1	0.522206298	9.23E-39	postive
PIK3R1	AC087284.1	0.597737599	3.95E-53	postive
PIK3CA	AC087284.1	0.677103656	5.22E-73	postive
PIK3CB	AC087284.1	0.525514586	2.57E-39	postive
AKT3	AC087284.1	0.527271368	1.29E-39	postive
RASGRP3	AC087284.1	0.522691205	7.66E-39	postive
IGHD4-4	AC087284.1	0.517212299	6.20E-38	postive
PLXNC1	AC087284.1	0.501002301	2.43E-35	postive
GNRH1	AC087284.1	0.741369223	2.21E-94	postive
IL6ST	AC087284.1	0.578913642	3.40E-49	postive
IL7	AC087284.1	0.5024321	1.45E-35	postive
RABEP1	AC087284.1	0.5560764	9.45E-45	postive
ACVR2A	AC087284.1	0.653125202	2.27E-66	postive
ANGPTL1	AC087284.1	0.755514105	5.79E-100	postive
BMPR1A	AC087284.1	0.563823756	3.21E-46	postive
BMPR2	AC087284.1	0.699961064	5.92E-80	postive
CRLF3	AC087284.1	0.64066732	3.74E-63	postive
NR2C2	AC087284.1	0.687744429	3.67E-76	postive
NR3C1	AC087284.1	0.504285932	7.43E-36	postive
RORA	AC087284.1	0.801769691	2.98E-121	postive
SOS1	AC087284.1	0.681153425	3.41E-74	postive
SOS2	AC087284.1	0.594087824	2.40E-52	postive
BRAF	AC087284.1	0.771288579	1.17E-106	postive
CBL	AC087284.1	0.593542669	3.13E-52	postive
CBLB	AC087284.1	0.674044768	3.97E-72	postive
RASGRP1	AC087284.1	0.509841755	9.74E-37	postive
PDK1	AC087284.1	0.589210494	2.58E-51	postive
TRAJ1	AC087284.1	0.809957518	1.29E-125	postive
TRAJ2	AC087284.1	0.751829794	1.79E-98	postive
TRAJ3	AC087284.1	0.854949065	4.59E-154	postive
TRAJ5	AC087284.1	0.841745483	8.41E-145	postive
TRAJ6	AC087284.1	0.877029027	8.08E-172	postive
TRAJ8	AC087284.1	0.839215908	4.00E-143	postive
TRAJ10	AC087284.1	0.877384518	3.93E-172	postive
TRAJ12	AC087284.1	0.80335799	4.40E-122	postive
TRAJ13	AC087284.1	0.848040854	4.15E-149	postive
TRAJ14	AC087284.1	0.844905432	6.13E-147	postive
TRAJ16	AC087284.1	0.854113126	1.88E-153	postive
TRAJ17	AC087284.1	0.846356784	6.16E-148	postive
TRAJ18	AC087284.1	0.79999743	2.46E-120	postive
TRAJ21	AC087284.1	0.873158347	1.81E-168	postive
TRAJ31	AC087284.1	0.895955971	5.19E-190	postive
TRAJ37	AC087284.1	0.875596859	1.45E-170	postive
TRAJ38	AC087284.1	0.907060809	2.10E-202	postive
TRAJ39	AC087284.1	0.887002854	5.32E-181	postive
CTF1	AC106782.5	0.506533558	3.28E-36	postive
RORA	AL359962.1	0.505547262	4.70E-36	postive
TRAJ38	AL359962.1	0.517606273	5.34E-38	postive
CREB1	AC016831.1	0.716861883	1.57E-85	postive
UBR1	AC016831.1	0.594287947	2.17E-52	postive
ZC3HAV1	AC016831.1	0.564551013	2.33E-46	postive
IL15	AC016831.1	0.586577303	9.13E-51	postive
CYLD	AC016831.1	0.567142233	7.33E-47	postive
EIF2AK2	AC016831.1	0.573426092	4.28E-48	postive
LMBR1	AC016831.1	0.56462665	2.25E-46	postive
IREB2	AC016831.1	0.537263465	2.43E-41	postive
DDX17	AC016831.1	0.539183453	1.12E-41	postive
PIK3CG	AC016831.1	0.51505659	1.40E-37	postive
JAK2	AC016831.1	0.624541986	3.33E-59	postive
TXK	AC016831.1	0.565682133	1.41E-46	postive
NFAT5	AC016831.1	0.719336334	2.22E-86	postive
PIK3R1	AC016831.1	0.599436344	1.69E-53	postive
PIK3CA	AC016831.1	0.644635122	3.67E-64	postive

PIK3CB	AC016831.1	0.538714043	1.35E-41	postive
RASGRP3	AC016831.1	0.525042274	3.08E-39	postive
PLXNC1	AC016831.1	0.508234474	1.76E-36	postive
GNRH1	AC016831.1	0.666597178	5.04E-70	postive
IL6ST	AC016831.1	0.559220012	2.42E-45	postive
ACVR2A	AC016831.1	0.546397375	5.72E-43	postive
ANGPTL1	AC016831.1	0.715265104	5.51E-85	postive
BMPR2	AC016831.1	0.671927437	1.60E-71	postive
CRLF3	AC016831.1	0.595148652	1.42E-52	postive
NR2C2	AC016831.1	0.668814193	1.21E-70	postive
RORA	AC016831.1	0.739046502	1.68E-93	postive
SOS1	AC016831.1	0.625026399	2.55E-59	postive
SOS2	AC016831.1	0.530655121	3.42E-40	postive
BRAF	AC016831.1	0.756411576	2.49E-100	postive
ITK	AC016831.1	0.502516214	1.41E-35	postive
TEC	AC016831.1	0.5072562	2.52E-36	postive
CBL	AC016831.1	0.57052663	1.60E-47	postive
CBLB	AC016831.1	0.690132283	6.89E-77	postive
RASGRP1	AC016831.1	0.520690536	1.65E-38	postive
PDK1	AC016831.1	0.549997648	1.26E-43	postive
TRAJ1	AC016831.1	0.668420661	1.56E-70	postive
TRAJ2	AC016831.1	0.641805284	1.93E-63	postive
TRAJ3	AC016831.1	0.724699954	2.95E-88	postive
TRAJ5	AC016831.1	0.74972678	1.24E-97	postive
TRAJ6	AC016831.1	0.720421463	9.33E-87	postive
TRAJ8	AC016831.1	0.68616999	1.09E-75	postive
TRAJ10	AC016831.1	0.69483298	2.44E-78	postive
TRAJ12	AC016831.1	0.687920549	3.24E-76	postive
TRAJ13	AC016831.1	0.697597084	3.32E-79	postive
TRAJ14	AC016831.1	0.711841544	7.85E-84	postive
TRAJ16	AC016831.1	0.715777612	3.69E-85	postive
TRAJ17	AC016831.1	0.69826319	2.05E-79	postive
TRAJ18	AC016831.1	0.690558131	5.10E-77	postive
TRAJ21	AC016831.1	0.746234044	2.93E-96	postive
TRAJ31	AC016831.1	0.75206428	1.44E-98	postive
TRAJ37	AC016831.1	0.702740188	7.63E-81	postive
TRAJ38	AC016831.1	0.784397459	1.22E-112	postive
TRAJ39	AC016831.1	0.758929442	2.28E-101	postive
DDX17	AL390728.6	0.564754628	2.13E-46	postive
GNRH1	AL390728.6	0.629605727	2.03E-60	postive
NR2C1	AL390728.6	0.582320158	6.89E-50	postive
NR2C2	AL390728.6	0.521327911	1.29E-38	postive
CREB1	AC002553.2	0.789732208	3.37E-115	postive
RFXAP	AC002553.2	0.540978931	5.36E-42	postive
PSMD6	AC002553.2	0.509296962	1.19E-36	postive
UBR1	AC002553.2	0.647102037	8.53E-65	postive
ZC3HAV1	AC002553.2	0.576123002	1.24E-48	postive
IL15	AC002553.2	0.559656833	2.00E-45	postive
CYLD	AC002553.2	0.608042075	2.14E-55	postive
EIF2AK2	AC002553.2	0.578060265	5.06E-49	postive
MAPK8	AC002553.2	0.579772037	2.28E-49	postive
LMBR1	AC002553.2	0.560489607	1.39E-45	postive
IREB2	AC002553.2	0.614966764	5.74E-57	postive
DDX17	AC002553.2	0.671424104	2.22E-71	postive
JAK2	AC002553.2	0.639375582	7.92E-63	postive
TXK	AC002553.2	0.607955749	2.23E-55	postive
NFAT5	AC002553.2	0.856691736	2.35E-155	postive
MALT1	AC002553.2	0.538612608	1.41E-41	postive
PIK3R1	AC002553.2	0.645262035	2.54E-64	postive
PIK3CA	AC002553.2	0.681011198	3.76E-74	postive
PIK3CB	AC002553.2	0.532509034	1.64E-40	postive
AKT3	AC002553.2	0.517030618	6.64E-38	postive
RASGRP3	AC002553.2	0.530108994	4.24E-40	postive
GNRH1	AC002553.2	0.80205161	2.12E-121	postive
IL6ST	AC002553.2	0.630727583	1.08E-60	postive

RABEP1	AC002553.2	0.588713614	3.27E-51	postive
ACVR2A	AC002553.2	0.684232133	4.17E-75	postive
ANGPTL1	AC002553.2	0.770178028	3.62E-106	postive
BMPR1A	AC002553.2	0.564594572	2.28E-46	postive
BMPR2	AC002553.2	0.736042694	2.25E-92	postive
CRLF3	AC002553.2	0.653036246	2.40E-66	postive
NR1D2	AC002553.2	0.520248833	1.95E-38	postive
NR2C1	AC002553.2	0.542386608	3.01E-42	postive
NR2C2	AC002553.2	0.744306625	1.64E-95	postive
RORA	AC002553.2	0.846472386	5.12E-148	postive
SOS1	AC002553.2	0.716108498	2.84E-85	postive
SOS2	AC002553.2	0.594287857	2.17E-52	postive
BRAF	AC002553.2	0.838200176	1.85E-142	postive
ITK	AC002553.2	0.519415708	2.69E-38	postive
CBL	AC002553.2	0.590926609	1.12E-51	postive
CBLB	AC002553.2	0.739976133	7.49E-94	postive
RASGRP1	AC002553.2	0.522827804	7.27E-39	postive
PDK1	AC002553.2	0.562912015	4.80E-46	postive
TRAJ1	AC002553.2	0.782542726	9.05E-112	postive
TRAJ2	AC002553.2	0.734847215	6.26E-92	postive
TRAJ3	AC002553.2	0.795283318	6.11E-118	postive
TRAJ5	AC002553.2	0.851330983	1.95E-151	postive
TRAJ6	AC002553.2	0.823261187	3.57E-133	postive
TRAJ8	AC002553.2	0.81073712	4.85E-126	postive
TRAJ10	AC002553.2	0.793981056	2.73E-117	postive
TRAJ12	AC002553.2	0.790169861	2.06E-115	postive
TRAJ13	AC002553.2	0.841784332	7.92E-145	postive
TRAJ14	AC002553.2	0.82098751	7.74E-132	postive
TRAJ16	AC002553.2	0.817418986	8.88E-130	postive
TRAJ17	AC002553.2	0.78985093	2.95E-115	postive
TRAJ18	AC002553.2	0.766416038	1.56E-104	postive
TRAJ21	AC002553.2	0.859322902	2.45E-157	postive
TRAJ31	AC002553.2	0.88278573	5.13E-177	postive
TRAJ37	AC002553.2	0.843939594	2.79E-146	postive
TRAJ38	AC002553.2	0.892550062	1.73E-186	postive
TRAJ39	AC002553.2	0.855547508	1.66E-154	postive
CREB1	AC096921.2	0.660524273	2.36E-68	postive
UBR1	AC096921.2	0.60880643	1.44E-55	postive
CYLD	AC096921.2	0.586932254	7.70E-51	postive
TCF7L2	AC096921.2	0.503296492	1.06E-35	postive
IREB2	AC096921.2	0.511018361	6.30E-37	postive
DDX17	AC096921.2	0.584508364	2.45E-50	postive
JAK2	AC096921.2	0.570627164	1.53E-47	postive
TXK	AC096921.2	0.626733063	9.98E-60	postive
NFAT5	AC096921.2	0.687205104	5.34E-76	postive
PIK3R1	AC096921.2	0.646314363	1.36E-64	postive
PIK3CA	AC096921.2	0.599477575	1.66E-53	postive
RASGRP3	AC096921.2	0.508059694	1.88E-36	postive
ROBO2	AC096921.2	0.607100286	3.47E-55	postive
GNRH1	AC096921.2	0.668886983	1.15E-70	postive
IL6ST	AC096921.2	0.593798574	2.76E-52	postive
RABEP1	AC096921.2	0.532049041	1.97E-40	postive
ACVR2A	AC096921.2	0.593962081	2.55E-52	postive
ANGPTL1	AC096921.2	0.63452317	1.28E-61	postive
BMPR2	AC096921.2	0.75124175	3.08E-98	postive
NR1D2	AC096921.2	0.58922994	2.55E-51	postive
NR2C2	AC096921.2	0.637636462	2.16E-62	postive
NR3C2	AC096921.2	0.545767313	7.43E-43	postive
RORA	AC096921.2	0.736653367	1.33E-92	postive
TGFBR2	AC096921.2	0.56656418	9.50E-47	postive
SOS1	AC096921.2	0.589905401	1.84E-51	postive
BRAF	AC096921.2	0.688045469	2.97E-76	postive
ITK	AC096921.2	0.506112516	3.83E-36	postive
CBLB	AC096921.2	0.651049641	8.00E-66	postive
TRAJ1	AC096921.2	0.529188341	6.10E-40	postive

TRAJ2	AC096921.2	0.568730985	3.60E-47	postive
TRAJ3	AC096921.2	0.571835338	8.84E-48	postive
TRAJ5	AC096921.2	0.576091761	1.26E-48	postive
TRAJ6	AC096921.2	0.56757077	6.05E-47	postive
TRAJ8	AC096921.2	0.546035267	6.65E-43	postive
TRAJ10	AC096921.2	0.523416733	5.79E-39	postive
TRAJ12	AC096921.2	0.569378382	2.69E-47	postive
TRAJ13	AC096921.2	0.568320128	4.33E-47	postive
TRAJ14	AC096921.2	0.546070995	6.55E-43	postive
TRAJ16	AC096921.2	0.561089322	1.07E-45	postive
TRAJ17	AC096921.2	0.519523094	2.58E-38	postive
TRAJ18	AC096921.2	0.55350765	2.84E-44	postive
TRAJ21	AC096921.2	0.585289934	1.69E-50	postive
TRAJ31	AC096921.2	0.626446251	1.17E-59	postive
TRAJ37	AC096921.2	0.598452	2.77E-53	postive
TRAJ38	AC096921.2	0.646316974	1.36E-64	postive
TRAJ39	AC096921.2	0.581412878	1.06E-49	postive
CREB1	AL354989.1	0.723494841	7.86E-88	postive
RFXAP	AL354989.1	0.500860045	2.55E-35	postive
PSMD6	AL354989.1	0.528369015	8.42E-40	postive
UBR1	AL354989.1	0.578947288	3.35E-49	postive
ZC3HAV1	AL354989.1	0.581966431	8.14E-50	postive
ZC3HAV1L	AL354989.1	0.51641981	8.36E-38	postive
IL15	AL354989.1	0.578625515	3.89E-49	postive
CYLD	AL354989.1	0.577829022	5.63E-49	postive
EIF2AK2	AL354989.1	0.602012404	4.63E-54	postive
NFKBIZ	AL354989.1	0.531598697	2.35E-40	postive
LMBR1	AL354989.1	0.53042674	3.74E-40	postive
IREB2	AL354989.1	0.545765052	7.44E-43	postive
DDX17	AL354989.1	0.613826807	1.05E-56	postive
JAK2	AL354989.1	0.610442088	6.16E-56	postive
TXK	AL354989.1	0.515638479	1.12E-37	postive
NFAT5	AL354989.1	0.733576706	1.84E-91	postive
PIK3R1	AL354989.1	0.566603812	9.33E-47	postive
PIK3CA	AL354989.1	0.640128678	5.12E-63	postive
PIK3CB	AL354989.1	0.596800136	6.29E-53	postive
RASGRP3	AL354989.1	0.516846175	7.12E-38	postive
LTB4R2	AL354989.1	0.505319263	5.11E-36	postive
PLXNC1	AL354989.1	0.555593005	1.16E-44	postive
GNRH1	AL354989.1	0.677576875	3.80E-73	postive
IL6ST	AL354989.1	0.534998174	6.06E-41	postive
ACVR2A	AL354989.1	0.55469669	1.71E-44	postive
ANGPTL1	AL354989.1	0.678847584	1.62E-73	postive
BMPR2	AL354989.1	0.617100408	1.85E-57	postive
CRLF3	AL354989.1	0.608058015	2.12E-55	postive
LIFR	AL354989.1	0.515286605	1.28E-37	postive
NR2C2	AL354989.1	0.731996977	7.00E-91	postive
RORA	AL354989.1	0.695671873	1.33E-78	postive
SOS1	AL354989.1	0.671714535	1.84E-71	postive
SOS2	AL354989.1	0.580313149	1.77E-49	postive
BRAF	AL354989.1	0.794364131	1.76E-117	postive
ITK	AL354989.1	0.504548489	6.76E-36	postive
CBL	AL354989.1	0.620279774	3.37E-58	postive
CBLB	AL354989.1	0.658378142	9.00E-68	postive
RASGRP1	AL354989.1	0.553460981	2.90E-44	postive
PDK1	AL354989.1	0.524059614	4.51E-39	postive
TRAJ1	AL354989.1	0.755157463	8.10E-100	postive
TRAJ2	AL354989.1	0.687316319	4.94E-76	postive
TRAJ3	AL354989.1	0.783667377	2.68E-112	postive
TRAJ5	AL354989.1	0.780640311	6.95E-111	postive
TRAJ6	AL354989.1	0.779471946	2.41E-110	postive
TRAJ8	AL354989.1	0.746554483	2.20E-96	postive
TRAJ10	AL354989.1	0.760968284	3.22E-102	postive
TRAJ12	AL354989.1	0.767221712	7.02E-105	postive
TRAJ13	AL354989.1	0.754809462	1.12E-99	postive

TRAJ14	AL354989.1	0.786735095	9.40E-114	postive
TRAJ16	AL354989.1	0.759765684	1.02E-101	postive
TRAJ17	AL354989.1	0.757897954	6.09E-101	postive
TRAJ18	AL354989.1	0.75566308	5.04E-100	postive
TRAJ21	AL354989.1	0.798298305	1.83E-119	postive
TRAJ31	AL354989.1	0.788583326	1.21E-114	postive
TRAJ37	AL354989.1	0.742379793	9.07E-95	postive
TRAJ38	AL354989.1	0.815454892	1.15E-128	postive
TRAJ39	AL354989.1	0.778317625	8.16E-110	postive
CREB1	EGOT	0.578204166	4.73E-49	postive
NFAT5	EGOT	0.627208679	7.67E-60	postive
GNRH1	EGOT	0.576873082	8.77E-49	postive
IL6ST	EGOT	0.523376277	5.88E-39	postive
ANGPTL1	EGOT	0.535867346	4.27E-41	postive
BMPR2	EGOT	0.548979675	1.94E-43	postive
CRLF3	EGOT	0.541851271	3.75E-42	postive
NR2C2	EGOT	0.502637146	1.35E-35	postive
RORA	EGOT	0.63662685	3.85E-62	postive
BRAF	EGOT	0.606206959	5.49E-55	postive
CBLB	EGOT	0.504006539	8.22E-36	postive
TRAJ1	EGOT	0.535287049	5.40E-41	postive
TRAJ2	EGOT	0.563121115	4.38E-46	postive
TRAJ3	EGOT	0.593597949	3.05E-52	postive
TRAJ5	EGOT	0.579766407	2.28E-49	postive
TRAJ6	EGOT	0.579486253	2.60E-49	postive
TRAJ8	EGOT	0.574033815	3.24E-48	postive
TRAJ10	EGOT	0.552428195	4.51E-44	postive
TRAJ12	EGOT	0.507063079	2.70E-36	postive
TRAJ13	EGOT	0.593977869	2.53E-52	postive
TRAJ14	EGOT	0.564570099	2.31E-46	postive
TRAJ16	EGOT	0.568620866	3.78E-47	postive
TRAJ17	EGOT	0.53940656	1.02E-41	postive
TRAJ18	EGOT	0.506787401	2.99E-36	postive
TRAJ21	EGOT	0.596125155	8.78E-53	postive
TRAJ31	EGOT	0.608711449	1.51E-55	postive
TRAJ37	EGOT	0.598791111	2.34E-53	postive
TRAJ38	EGOT	0.628514228	3.72E-60	postive
TRAJ39	EGOT	0.624727081	3.01E-59	postive
CREB1	AL513008.1	0.691335767	2.95E-77	postive
UBR1	AL513008.1	0.644095934	5.05E-64	postive
ZC3HAV1	AL513008.1	0.522052624	9.79E-39	postive
IL15	AL513008.1	0.549110363	1.83E-43	postive
CYLD	AL513008.1	0.550459917	1.04E-43	postive
EIF2AK2	AL513008.1	0.516949664	6.85E-38	postive
MAPK8	AL513008.1	0.516032485	9.68E-38	postive
LMBR1	AL513008.1	0.500240906	3.19E-35	postive
IREB2	AL513008.1	0.568396084	4.18E-47	postive
DDX17	AL513008.1	0.602027665	4.60E-54	postive
PIK3CG	AL513008.1	0.511200292	5.89E-37	postive
JAK2	AL513008.1	0.582354528	6.78E-50	postive
TXK	AL513008.1	0.643567309	6.89E-64	postive
NFAT5	AL513008.1	0.752119992	1.37E-98	postive
MALT1	AL513008.1	0.504697834	6.40E-36	postive
PIK3R1	AL513008.1	0.642647021	1.18E-63	postive
PIK3CA	AL513008.1	0.603799004	1.88E-54	postive
IGHD6-6	AL513008.1	0.567902858	5.22E-47	postive
GNRH1	AL513008.1	0.731607029	9.72E-91	postive
IL6ST	AL513008.1	0.588885982	3.01E-51	postive
RABEP1	AL513008.1	0.530896417	3.11E-40	postive
ACVR2A	AL513008.1	0.551203329	7.58E-44	postive
ANGPTL1	AL513008.1	0.702456824	9.41E-81	postive
BMPR1A	AL513008.1	0.50215582	1.60E-35	postive
BMPR2	AL513008.1	0.659968417	3.34E-68	postive
CRLF3	AL513008.1	0.554144605	2.17E-44	postive
NR2C2	AL513008.1	0.673706936	4.97E-72	postive

RORA	AL513008.1	0.766782382	1.09E-104	postive
SOS1	AL513008.1	0.582624681	5.97E-50	postive
SOS2	AL513008.1	0.502283417	1.53E-35	postive
BRAF	AL513008.1	0.746042257	3.48E-96	postive
TEC	AL513008.1	0.590291685	1.53E-51	postive
CBL	AL513008.1	0.553158983	3.30E-44	postive
CBLB	AL513008.1	0.675491677	1.53E-72	postive
RASGRP1	AL513008.1	0.508293294	1.72E-36	postive
PDK1	AL513008.1	0.566502178	9.76E-47	postive
TRAJ1	AL513008.1	0.583797659	3.43E-50	postive
TRAJ2	AL513008.1	0.618138374	1.06E-57	postive
TRAJ3	AL513008.1	0.706046225	6.47E-82	postive
TRAJ5	AL513008.1	0.62539642	2.08E-59	postive
TRAJ6	AL513008.1	0.661440215	1.33E-68	postive
TRAJ8	AL513008.1	0.574657331	2.43E-48	postive
TRAJ10	AL513008.1	0.631048739	9.05E-61	postive
TRAJ12	AL513008.1	0.613367214	1.33E-56	postive
TRAJ13	AL513008.1	0.59326055	3.60E-52	postive
TRAJ14	AL513008.1	0.616169231	3.04E-57	postive
TRAJ16	AL513008.1	0.602562699	3.51E-54	postive
TRAJ17	AL513008.1	0.580993949	1.29E-49	postive
TRAJ18	AL513008.1	0.658576996	7.96E-68	postive
TRAJ21	AL513008.1	0.668723295	1.28E-70	postive
TRAJ31	AL513008.1	0.657477111	1.57E-67	postive
TRAJ37	AL513008.1	0.663557344	3.50E-69	postive
TRAJ38	AL513008.1	0.71105059	1.44E-83	postive
TRAJ39	AL513008.1	0.694732683	2.62E-78	postive
CREB1	AL662844.3	0.660706387	2.11E-68	postive
RFXAP	AL662844.3	0.512403174	3.77E-37	postive
UBR1	AL662844.3	0.555353385	1.29E-44	postive
ZC3HAV1	AL662844.3	0.50509419	5.54E-36	postive
ZC3HAV1L	AL662844.3	0.513216433	2.78E-37	postive
IL15	AL662844.3	0.51023375	8.43E-37	postive
CYLD	AL662844.3	0.561580707	8.62E-46	postive
NFKBIZ	AL662844.3	0.534995748	6.07E-41	postive
IREB2	AL662844.3	0.505967343	4.03E-36	postive
DDX17	AL662844.3	0.683857304	5.39E-75	postive
JAK2	AL662844.3	0.577585191	6.31E-49	postive
TXK	AL662844.3	0.590604468	1.31E-51	postive
NFAT5	AL662844.3	0.72189652	2.86E-87	postive
PIK3R1	AL662844.3	0.546494997	5.49E-43	postive
PIK3CA	AL662844.3	0.580870121	1.36E-49	postive
RASGRP3	AL662844.3	0.507125176	2.64E-36	postive
IGHD4-4	AL662844.3	0.522170413	9.36E-39	postive
IGHD6-6	AL662844.3	0.506758512	3.02E-36	postive
LTB4R2	AL662844.3	0.570192298	1.86E-47	postive
GNRH1	AL662844.3	0.821867894	2.36E-132	postive
IL6ST	AL662844.3	0.546998707	4.45E-43	postive
RABEP1	AL662844.3	0.523673789	5.24E-39	postive
ACVR2A	AL662844.3	0.542725644	2.62E-42	postive
ANGPTL1	AL662844.3	0.626731961	9.98E-60	postive
BMPR2	AL662844.3	0.59365143	2.97E-52	postive
CRLF3	AL662844.3	0.605208922	9.14E-55	postive
NR2C1	AL662844.3	0.543906341	1.61E-42	postive
NR2C2	AL662844.3	0.746782808	1.79E-96	postive
RORA	AL662844.3	0.732790237	3.59E-91	postive
SOS1	AL662844.3	0.593279835	3.56E-52	postive
SOS2	AL662844.3	0.533694735	1.02E-40	postive
BRAF	AL662844.3	0.702244832	1.10E-80	postive
TEC	AL662844.3	0.549319167	1.68E-43	postive
CBL	AL662844.3	0.568264415	4.44E-47	postive
CBLB	AL662844.3	0.651970157	4.59E-66	postive
PDK1	AL662844.3	0.576473856	1.05E-48	postive
TRAJ1	AL662844.3	0.640037291	5.40E-63	postive
TRAJ2	AL662844.3	0.533236773	1.23E-40	postive

TRAJ3	AL662844.3	0.631315339	7.80E-61	postive
TRAJ5	AL662844.3	0.686462091	8.94E-76	postive
TRAJ6	AL662844.3	0.622017684	1.32E-58	postive
TRAJ8	AL662844.3	0.564734636	2.14E-46	postive
TRAJ10	AL662844.3	0.60518438	9.26E-55	postive
TRAJ12	AL662844.3	0.532655166	1.55E-40	postive
TRAJ13	AL662844.3	0.601043811	7.55E-54	postive
TRAJ14	AL662844.3	0.568776641	3.52E-47	postive
TRAJ16	AL662844.3	0.598382608	2.86E-53	postive
TRAJ17	AL662844.3	0.569111524	3.03E-47	postive
TRAJ18	AL662844.3	0.562470199	5.83E-46	postive
TRAJ21	AL662844.3	0.623114119	7.25E-59	postive
TRAJ31	AL662844.3	0.644578762	3.80E-64	postive
TRAJ37	AL662844.3	0.634787889	1.10E-61	postive
TRAJ38	AL662844.3	0.65779428	1.29E-67	postive
TRAJ39	AL662844.3	0.666304929	6.08E-70	postive
DDX17	AC022400.1	0.512374926	3.81E-37	postive
GNRH1	AC022400.1	0.505264382	5.21E-36	postive
NR2C2	AC022400.1	0.577210258	7.50E-49	postive
LMBR1L	AC084018.1	0.522026242	9.89E-39	postive
IRF9	AC084018.1	0.610107204	7.33E-56	postive
CREB1	AC015849.3	0.709157869	6.14E-83	postive
RFXAP	AC015849.3	0.531361648	2.58E-40	postive
UBR1	AC015849.3	0.586687282	8.66E-51	postive
ZC3HAV1	AC015849.3	0.577083204	7.96E-49	postive
ZC3HAV1L	AC015849.3	0.566956721	7.97E-47	postive
IL15	AC015849.3	0.508276744	1.73E-36	postive
CYLD	AC015849.3	0.541114133	5.07E-42	postive
EIF2AK2	AC015849.3	0.529601	5.18E-40	postive
MAPK8	AC015849.3	0.531092442	2.88E-40	postive
NFKBIZ	AC015849.3	0.534080011	8.75E-41	postive
LMBR1	AC015849.3	0.507710045	2.13E-36	postive
IREB2	AC015849.3	0.564567504	2.31E-46	postive
DDX17	AC015849.3	0.682405366	1.46E-74	postive
JAK2	AC015849.3	0.536626281	3.15E-41	postive
TXK	AC015849.3	0.541559179	4.23E-42	postive
NFAT5	AC015849.3	0.728080445	1.84E-89	postive
PIK3R1	AC015849.3	0.554299821	2.03E-44	postive
PIK3CA	AC015849.3	0.602096849	4.44E-54	postive
AKT3	AC015849.3	0.523023158	6.74E-39	postive
LTB4R2	AC015849.3	0.629398611	2.28E-60	postive
GNRH1	AC015849.3	0.769766375	5.48E-106	postive
IL6ST	AC015849.3	0.55484036	1.61E-44	postive
RABEP1	AC015849.3	0.532156011	1.89E-40	postive
ACVR2A	AC015849.3	0.563174913	4.28E-46	postive
ANGPTL1	AC015849.3	0.68442782	3.64E-75	postive
BMPR2	AC015849.3	0.624817867	2.86E-59	postive
CRLF3	AC015849.3	0.629104265	2.68E-60	postive
NR2C1	AC015849.3	0.545480319	8.37E-43	postive
NR2C2	AC015849.3	0.782882674	6.27E-112	postive
RORA	AC015849.3	0.70161929	1.75E-80	postive
SOS1	AC015849.3	0.681508869	2.68E-74	postive
SOS2	AC015849.3	0.548274336	2.61E-43	postive
BRAF	AC015849.3	0.79789293	2.94E-119	postive
ITK	AC015849.3	0.505003914	5.73E-36	postive
CBL	AC015849.3	0.62595599	1.53E-59	postive
CBLB	AC015849.3	0.668440565	1.54E-70	postive
RASGRP1	AC015849.3	0.500420844	2.99E-35	postive
PDK1	AC015849.3	0.529125324	6.25E-40	postive
TRAJ1	AC015849.3	0.657097376	1.99E-67	postive
TRAJ2	AC015849.3	0.675258394	1.78E-72	postive
TRAJ3	AC015849.3	0.725297212	1.81E-88	postive
TRAJ5	AC015849.3	0.669630889	7.13E-71	postive
TRAJ6	AC015849.3	0.669828698	6.27E-71	postive
TRAJ8	AC015849.3	0.685775257	1.44E-75	postive

TRAJ10	AC015849.3	0.633602022	2.15E-61	postive
TRAJ12	AC015849.3	0.555387775	1.27E-44	postive
TRAJ13	AC015849.3	0.669907255	5.96E-71	postive
TRAJ14	AC015849.3	0.65885149	6.71E-68	postive
TRAJ16	AC015849.3	0.664894403	1.50E-69	postive
TRAJ17	AC015849.3	0.633012127	3.00E-61	postive
TRAJ18	AC015849.3	0.565002632	1.90E-46	postive
TRAJ21	AC015849.3	0.678792743	1.68E-73	postive
TRAJ31	AC015849.3	0.676743603	6.63E-73	postive
TRAJ37	AC015849.3	0.663135694	4.57E-69	postive
TRAJ38	AC015849.3	0.718589613	4.01E-86	postive
TRAJ39	AC015849.3	0.704594644	1.92E-81	postive
DDX17	AC024361.1	0.524616976	3.64E-39	postive
GNRH1	AC024361.1	0.515664813	1.11E-37	postive
NR2C1	AC024361.1	0.507404896	2.39E-36	postive
NR2C2	AC024361.1	0.569421217	2.64E-47	postive
BRAF	AC024361.1	0.518235943	4.21E-38	postive
CREB1	NDUFV2-AS1	0.702736854	7.65E-81	postive
RFXAP	NDUFV2-AS1	0.527978198	9.81E-40	postive
UBR1	NDUFV2-AS1	0.575366036	1.76E-48	postive
ZC3HAV1	NDUFV2-AS1	0.504661151	6.49E-36	postive
IL15	NDUFV2-AS1	0.507703195	2.14E-36	postive
CYLD	NDUFV2-AS1	0.538525466	1.46E-41	postive
MAPK8	NDUFV2-AS1	0.555838321	1.05E-44	postive
IREB2	NDUFV2-AS1	0.539711237	9.00E-42	postive
DDX17	NDUFV2-AS1	0.663949573	2.73E-69	postive
JAK2	NDUFV2-AS1	0.556281344	8.65E-45	postive
TXK	NDUFV2-AS1	0.554295817	2.03E-44	postive
NFAT5	NDUFV2-AS1	0.791755417	3.45E-116	postive
MALT1	NDUFV2-AS1	0.503006124	1.18E-35	postive
PIK3R1	NDUFV2-AS1	0.555632888	1.14E-44	postive
PIK3CA	NDUFV2-AS1	0.598494165	2.71E-53	postive
AKT3	NDUFV2-AS1	0.502895107	1.23E-35	postive
RASGRP3	NDUFV2-AS1	0.502277715	1.53E-35	postive
GNRH1	NDUFV2-AS1	0.799550329	4.18E-120	postive
IL6ST	NDUFV2-AS1	0.543985106	1.56E-42	postive
RABEP1	NDUFV2-AS1	0.510017923	9.13E-37	postive
ACVR2A	NDUFV2-AS1	0.632733974	3.51E-61	postive
ANGPTL1	NDUFV2-AS1	0.709957019	3.34E-83	postive
BMPR1A	NDUFV2-AS1	0.515127267	1.36E-37	postive
BMPR2	NDUFV2-AS1	0.629224259	2.51E-60	postive
CRLF3	NDUFV2-AS1	0.601885419	4.94E-54	postive
NR2C1	NDUFV2-AS1	0.532885992	1.41E-40	postive
NR2C2	NDUFV2-AS1	0.733431217	2.09E-91	postive
RORA	NDUFV2-AS1	0.778329672	8.05E-110	postive
SOS1	NDUFV2-AS1	0.664856201	1.53E-69	postive
SOS2	NDUFV2-AS1	0.544715041	1.15E-42	postive
BRAF	NDUFV2-AS1	0.756570661	2.14E-100	postive
CBL	NDUFV2-AS1	0.544158814	1.45E-42	postive
CBLB	NDUFV2-AS1	0.6886944	1.89E-76	postive
PDK1	NDUFV2-AS1	0.553675207	2.65E-44	postive
TRAJ1	NDUFV2-AS1	0.749077805	2.23E-97	postive
TRAJ2	NDUFV2-AS1	0.677519961	3.95E-73	postive
TRAJ3	NDUFV2-AS1	0.753990654	2.41E-99	postive
TRAJ5	NDUFV2-AS1	0.79490565	9.45E-118	postive
TRAJ6	NDUFV2-AS1	0.771042421	1.51E-106	postive
TRAJ8	NDUFV2-AS1	0.770072422	4.03E-106	postive
TRAJ10	NDUFV2-AS1	0.745469726	5.81E-96	postive
TRAJ12	NDUFV2-AS1	0.707307571	2.50E-82	postive
TRAJ13	NDUFV2-AS1	0.791560662	4.30E-116	postive
TRAJ14	NDUFV2-AS1	0.765811394	2.84E-104	postive
TRAJ16	NDUFV2-AS1	0.776557156	5.17E-109	postive
TRAJ17	NDUFV2-AS1	0.749978516	9.81E-98	postive
TRAJ18	NDUFV2-AS1	0.688031802	3.00E-76	postive
TRAJ21	NDUFV2-AS1	0.802380277	1.43E-121	postive

TRAJ31	NDUFV2-AS1	0.810495232	6.58E-126	postive
TRAJ37	NDUFV2-AS1	0.780975743	4.86E-111	postive
TRAJ38	NDUFV2-AS1	0.821914187	2.22E-132	postive
TRAJ39	NDUFV2-AS1	0.810268481	8.75E-126	postive
GAL	AL022313.2	0.898138245	2.48E-192	postive
CYLD	AC016957.2	0.560995205	1.11E-45	postive
DDX17	AC016957.2	0.528926909	6.76E-40	postive
JAK2	AC016957.2	0.561058544	1.08E-45	postive
TXK	AC016957.2	0.542284831	3.14E-42	postive
NFAT5	AC016957.2	0.557013229	6.31E-45	postive
NFATC2	AC016957.2	0.502567577	1.38E-35	postive
PIK3R1	AC016957.2	0.527390627	1.24E-39	postive
RASGRP3	AC016957.2	0.53654255	3.25E-41	postive
GNRH1	AC016957.2	0.600746325	8.77E-54	postive
ACVR2A	AC016957.2	0.516757079	7.36E-38	postive
ANGPTL1	AC016957.2	0.588956085	2.91E-51	postive
NR2C2	AC016957.2	0.520786995	1.59E-38	postive
RORA	AC016957.2	0.646612962	1.14E-64	postive
BRAF	AC016957.2	0.541168838	4.96E-42	postive
ITK	AC016957.2	0.608223814	1.94E-55	postive
CD28	AC016957.2	0.524144095	4.37E-39	postive
CBLB	AC016957.2	0.582336177	6.84E-50	postive
TRAJ1	AC016957.2	0.580928048	1.33E-49	postive
TRAJ2	AC016957.2	0.540755993	5.88E-42	postive
TRAJ3	AC016957.2	0.553175051	3.28E-44	postive
TRAJ5	AC016957.2	0.613448192	1.28E-56	postive
TRAJ6	AC016957.2	0.549471766	1.58E-43	postive
TRAJ8	AC016957.2	0.53048863	3.65E-40	postive
TRAJ10	AC016957.2	0.522178902	9.33E-39	postive
TRAJ12	AC016957.2	0.567492729	6.27E-47	postive
TRAJ13	AC016957.2	0.57509958	1.99E-48	postive
TRAJ14	AC016957.2	0.565737711	1.37E-46	postive
TRAJ16	AC016957.2	0.560940254	1.14E-45	postive
TRAJ17	AC016957.2	0.543130219	2.22E-42	postive
TRAJ18	AC016957.2	0.582799631	5.50E-50	postive
TRAJ21	AC016957.2	0.589416158	2.33E-51	postive
TRAJ31	AC016957.2	0.628069256	4.77E-60	postive
TRAJ37	AC016957.2	0.568363709	4.24E-47	postive
TRAJ38	AC016957.2	0.615557732	4.20E-57	postive
TRAJ39	AC016957.2	0.56744935	6.39E-47	postive
CIITA	AC005332.4	0.514078504	2.02E-37	postive
NFATC2	AC005332.4	0.535996105	4.06E-41	postive
RASGRP3	AC005332.4	0.511323582	5.63E-37	postive
IL18RAP	AC005332.4	0.500009882	3.46E-35	postive
ZAP70	AC005332.4	0.518044712	4.52E-38	postive
PTPRC	AC005332.4	0.512408526	3.76E-37	postive
ITK	AC005332.4	0.601885718	4.94E-54	postive
CD28	AC005332.4	0.513132287	2.87E-37	postive
TRBC1	AC005332.4	0.507451281	2.35E-36	postive
GNRH1	AL513218.1	0.566378791	1.03E-46	postive
CREB1	AC008969.1	0.564427811	2.46E-46	postive
RFXAP	AC008969.1	0.607718466	2.52E-55	postive
DDX17	AC008969.1	0.593916228	2.61E-52	postive
NFAT5	AC008969.1	0.626028056	1.47E-59	postive
LTB4R2	AC008969.1	0.54629645	5.96E-43	postive
GNRH1	AC008969.1	0.661515889	1.27E-68	postive
ANGPTL1	AC008969.1	0.515958892	9.95E-38	postive
NR2C1	AC008969.1	0.514218307	1.91E-37	postive
NR2C2	AC008969.1	0.694117552	4.07E-78	postive
RORA	AC008969.1	0.578543445	4.04E-49	postive
SOS1	AC008969.1	0.526608976	1.68E-39	postive
BRAF	AC008969.1	0.652251976	3.87E-66	postive
CBL	AC008969.1	0.533319908	1.19E-40	postive
CBLB	AC008969.1	0.513141968	2.86E-37	postive
TRAJ1	AC008969.1	0.533235596	1.23E-40	postive

TRAJ2	AC008969.1	0.556854249	6.75E-45	postive
TRAJ3	AC008969.1	0.603846539	1.83E-54	postive
TRAJ5	AC008969.1	0.52072037	1.63E-38	postive
TRAJ6	AC008969.1	0.549279799	1.71E-43	postive
TRAJ8	AC008969.1	0.540612647	6.23E-42	postive
TRAJ10	AC008969.1	0.522390152	8.60E-39	postive
TRAJ13	AC008969.1	0.529846619	4.71E-40	postive
TRAJ14	AC008969.1	0.51872666	3.49E-38	postive
TRAJ16	AC008969.1	0.531768564	2.20E-40	postive
TRAJ17	AC008969.1	0.510130456	8.75E-37	postive
TRAJ21	AC008969.1	0.546210283	6.18E-43	postive
TRAJ31	AC008969.1	0.52428616	4.14E-39	postive
TRAJ37	AC008969.1	0.5458763	7.10E-43	postive
TRAJ38	AC008969.1	0.565750031	1.37E-46	postive
TRAJ39	AC008969.1	0.566417039	1.01E-46	postive
CREB1	AC026368.1	0.517191314	6.25E-38	postive
UBR1	AC026368.1	0.559775966	1.90E-45	postive
SP1	AC026368.1	0.525632249	2.45E-39	postive
IREB2	AC026368.1	0.544718207	1.15E-42	postive
NFAT5	AC026368.1	0.565440263	1.57E-46	postive
GNRH1	AC026368.1	0.590599266	1.32E-51	postive
ACVR2A	AC026368.1	0.501125215	2.32E-35	postive
ANGPTL1	AC026368.1	0.521903797	1.04E-38	postive
BMPR2	AC026368.1	0.51624882	8.92E-38	postive
NR2C2	AC026368.1	0.52340422	5.82E-39	postive
RORA	AC026368.1	0.558007654	4.10E-45	postive
SOS1	AC026368.1	0.530358094	3.85E-40	postive
BRAF	AC026368.1	0.573815163	3.58E-48	postive
CBLB	AC026368.1	0.514570941	1.68E-37	postive
TRAJ3	AC026368.1	0.507897474	1.99E-36	postive
TRAJ5	AC026368.1	0.501525683	2.01E-35	postive
TRAJ6	AC026368.1	0.510433391	7.83E-37	postive
TRAJ8	AC026368.1	0.508072765	1.87E-36	postive
TRAJ12	AC026368.1	0.506412312	3.43E-36	postive
TRAJ13	AC026368.1	0.519224481	2.89E-38	postive
TRAJ14	AC026368.1	0.50460518	6.62E-36	postive
TRAJ21	AC026368.1	0.523661773	5.27E-39	postive
TRAJ31	AC026368.1	0.55377366	2.54E-44	postive
TRAJ37	AC026368.1	0.517910496	4.76E-38	postive
TRAJ38	AC026368.1	0.564093959	2.85E-46	postive
TRAJ39	AC026368.1	0.513537703	2.47E-37	postive
CREB1	NORAD	0.60457758	1.26E-54	postive
UBR1	NORAD	0.554124835	2.18E-44	postive
IREB2	NORAD	0.52744559	1.21E-39	postive
NFAT5	NORAD	0.520344499	1.88E-38	postive
PIK3R1	NORAD	0.522555075	8.07E-39	postive
PIK3CA	NORAD	0.53185134	2.13E-40	postive
IL6ST	NORAD	0.50810897	1.84E-36	postive
ACVR2A	NORAD	0.516104881	9.42E-38	postive
BMPR1A	NORAD	0.546509106	5.46E-43	postive
BMPR2	NORAD	0.621808108	1.47E-58	postive
NR1D2	NORAD	0.566240632	1.10E-46	postive
NR2C2	NORAD	0.506337889	3.52E-36	postive
RORA	NORAD	0.526453177	1.78E-39	postive
SOS1	NORAD	0.594897835	1.61E-52	postive
BRAF	NORAD	0.553719946	2.60E-44	postive
CREB1	AF131215.5	0.634020869	1.70E-61	postive
UBR1	AF131215.5	0.59052792	1.36E-51	postive
CYLD	AF131215.5	0.666763781	4.53E-70	postive
DDX17	AF131215.5	0.551309148	7.25E-44	postive
JAK2	AF131215.5	0.585442539	1.57E-50	postive
TXK	AF131215.5	0.569422923	2.63E-47	postive
NFAT5	AF131215.5	0.723389111	8.56E-88	postive
NFATC3	AF131215.5	0.602596402	3.45E-54	postive
PIK3R1	AF131215.5	0.562467243	5.84E-46	postive

PIK3CA	AF131215.5	0.510848559	6.71E-37	postive
ROBO2	AF131215.5	0.689004741	1.52E-76	postive
GNRH1	AF131215.5	0.646207986	1.45E-64	postive
IL33	AF131215.5	0.582321776	6.89E-50	postive
IL6ST	AF131215.5	0.500560531	2.84E-35	postive
RABEP1	AF131215.5	0.514965478	1.45E-37	postive
ACVR2A	AF131215.5	0.609378495	1.07E-55	postive
BMPR2	AF131215.5	0.633398601	2.41E-61	postive
NR1D2	AF131215.5	0.569908497	2.12E-47	postive
NR2C2	AF131215.5	0.57994155	2.11E-49	postive
NR3C2	AF131215.5	0.580411534	1.69E-49	postive
RORA	AF131215.5	0.694603296	2.88E-78	postive
SOS1	AF131215.5	0.511973171	4.42E-37	postive
BRAF	AF131215.5	0.514808857	1.53E-37	postive
TRAJ1	AF131215.5	0.603002638	2.81E-54	postive
TRAJ2	AF131215.5	0.572313017	7.11E-48	postive
TRAJ3	AF131215.5	0.578147251	4.86E-49	postive
TRAJ5	AF131215.5	0.560316009	1.50E-45	postive
TRAJ6	AF131215.5	0.623440145	6.07E-59	postive
TRAJ8	AF131215.5	0.548848314	2.05E-43	postive
TRAJ10	AF131215.5	0.595665755	1.10E-52	postive
TRAJ12	AF131215.5	0.593442752	3.29E-52	postive
TRAJ13	AF131215.5	0.581898258	8.41E-50	postive
TRAJ14	AF131215.5	0.540836686	5.69E-42	postive
TRAJ16	AF131215.5	0.604882573	1.08E-54	postive
TRAJ17	AF131215.5	0.550688727	9.43E-44	postive
TRAJ18	AF131215.5	0.595921824	9.71E-53	postive
TRAJ21	AF131215.5	0.574434238	2.70E-48	postive
TRAJ31	AF131215.5	0.621263455	1.98E-58	postive
TRAJ37	AF131215.5	0.624169674	4.08E-59	postive
TRAJ38	AF131215.5	0.631636461	6.51E-61	postive
TRAJ39	AF131215.5	0.573617839	3.92E-48	postive
CREB1	AC024075.1	0.71827029	5.17E-86	postive
RFXAP	AC024075.1	0.573740451	3.71E-48	postive
UBR1	AC024075.1	0.632805642	3.37E-61	postive
ZC3HAV1	AC024075.1	0.58529711	1.68E-50	postive
CYLD	AC024075.1	0.639987314	5.56E-63	postive
IREB2	AC024075.1	0.590806287	1.19E-51	postive
DDX17	AC024075.1	0.674539821	2.87E-72	postive
PIK3CG	AC024075.1	0.552978643	3.56E-44	postive
JAK2	AC024075.1	0.564721945	2.16E-46	postive
TXK	AC024075.1	0.524741116	3.47E-39	postive
NFAT5	AC024075.1	0.719971176	1.34E-86	postive
NFATC3	AC024075.1	0.627516493	6.47E-60	postive
PIK3R1	AC024075.1	0.656397976	3.06E-67	postive
PIK3CA	AC024075.1	0.568072485	4.83E-47	postive
SLIT2	AC024075.1	0.504009563	8.21E-36	postive
GNRH1	AC024075.1	0.614728769	6.51E-57	postive
IL6ST	AC024075.1	0.63729824	2.62E-62	postive
RABEP1	AC024075.1	0.608374983	1.80E-55	postive
ACVR2A	AC024075.1	0.607234497	3.24E-55	postive
ANGPTL1	AC024075.1	0.561504387	8.92E-46	postive
BMPR1A	AC024075.1	0.526071398	2.07E-39	postive
BMPR2	AC024075.1	0.648066648	4.80E-65	postive
CRLF3	AC024075.1	0.511976998	4.42E-37	postive
LIFR	AC024075.1	0.509813149	9.84E-37	postive
NR1D2	AC024075.1	0.612148441	2.53E-56	postive
NR2C1	AC024075.1	0.520488767	1.78E-38	postive
NR2C2	AC024075.1	0.769092452	1.08E-105	postive
NR3C2	AC024075.1	0.570311328	1.76E-47	postive
RORA	AC024075.1	0.741777715	1.54E-94	postive
SOS1	AC024075.1	0.63712449	2.89E-62	postive
SOS2	AC024075.1	0.574422135	2.71E-48	postive
BRAF	AC024075.1	0.696093735	9.85E-79	postive
CBL	AC024075.1	0.590419661	1.44E-51	postive

CBLB	AC024075.1	0.58845717	3.70E-51	postive
RASGRP1	AC024075.1	0.511949876	4.46E-37	postive
TRAJ1	AC024075.1	0.575000355	2.08E-48	postive
TRAJ2	AC024075.1	0.595861205	1.00E-52	postive
TRAJ3	AC024075.1	0.613538844	1.22E-56	postive
TRAJ5	AC024075.1	0.582635676	5.94E-50	postive
TRAJ6	AC024075.1	0.594999037	1.53E-52	postive
TRAJ8	AC024075.1	0.54345563	1.94E-42	postive
TRAJ10	AC024075.1	0.576877999	8.75E-49	postive
TRAJ12	AC024075.1	0.542720104	2.62E-42	postive
TRAJ13	AC024075.1	0.560034948	1.70E-45	postive
TRAJ14	AC024075.1	0.547674584	3.35E-43	postive
TRAJ16	AC024075.1	0.569962104	2.06E-47	postive
TRAJ17	AC024075.1	0.53740705	2.30E-41	postive
TRAJ18	AC024075.1	0.576575617	1.01E-48	postive
TRAJ21	AC024075.1	0.577174392	7.63E-49	postive
TRAJ31	AC024075.1	0.587183105	6.83E-51	postive
TRAJ37	AC024075.1	0.602918455	2.93E-54	postive
TRAJ38	AC024075.1	0.627103775	8.13E-60	postive
TRAJ39	AC024075.1	0.591088075	1.04E-51	postive
CREB1	AC027097.2	0.688266492	2.55E-76	postive
RFXAP	AC027097.2	0.529316595	5.80E-40	postive
IL15	AC027097.2	0.558103623	3.93E-45	postive
CYLD	AC027097.2	0.56703254	7.70E-47	postive
MAPK8	AC027097.2	0.502996771	1.18E-35	postive
LIMS1	AC027097.2	0.512439135	3.72E-37	postive
DDX17	AC027097.2	0.552712925	3.99E-44	postive
PIK3CG	AC027097.2	0.588528435	3.58E-51	postive
JAK2	AC027097.2	0.633100523	2.85E-61	postive
TXK	AC027097.2	0.566667192	9.07E-47	postive
NFAT5	AC027097.2	0.685734733	1.48E-75	postive
MALT1	AC027097.2	0.545192082	9.44E-43	postive
PIK3R1	AC027097.2	0.621922601	1.39E-58	postive
PIK3CA	AC027097.2	0.61646243	2.60E-57	postive
AKT3	AC027097.2	0.566779956	8.62E-47	postive
RASGRP3	AC027097.2	0.579801836	2.25E-49	postive
IGHD6-6	AC027097.2	0.511633277	5.02E-37	postive
PLXNC1	AC027097.2	0.552885688	3.71E-44	postive
GNRH1	AC027097.2	0.677845942	3.17E-73	postive
IL6ST	AC027097.2	0.574199521	3.00E-48	postive
RABEP1	AC027097.2	0.560480292	1.40E-45	postive
TNFSF8	AC027097.2	0.508817233	1.42E-36	postive
ACVR2A	AC027097.2	0.583431849	4.08E-50	postive
ANGPTL1	AC027097.2	0.737569089	6.05E-93	postive
BMPR2	AC027097.2	0.63623136	4.82E-62	postive
CRLF3	AC027097.2	0.677247241	4.74E-73	postive
NR2C2	AC027097.2	0.646381264	1.31E-64	postive
RORA	AC027097.2	0.727654052	2.62E-89	postive
SOS1	AC027097.2	0.6309024	9.83E-61	postive
SOS2	AC027097.2	0.526641294	1.66E-39	postive
BRAF	AC027097.2	0.707268664	2.57E-82	postive
PTPRC	AC027097.2	0.542076958	3.42E-42	postive
ITK	AC027097.2	0.60785663	2.35E-55	postive
CD28	AC027097.2	0.558515441	3.29E-45	postive
CBL	AC027097.2	0.589502218	2.24E-51	postive
CBLB	AC027097.2	0.602105614	4.42E-54	postive
PDK1	AC027097.2	0.572911274	5.41E-48	postive
TRAJ1	AC027097.2	0.638418483	1.37E-62	postive
TRAJ2	AC027097.2	0.639897378	5.85E-63	postive
TRAJ3	AC027097.2	0.684131533	4.47E-75	postive
TRAJ5	AC027097.2	0.7036175	3.98E-81	postive
TRAJ6	AC027097.2	0.655878247	4.22E-67	postive
TRAJ8	AC027097.2	0.653658006	1.64E-66	postive
TRAJ10	AC027097.2	0.63647931	4.18E-62	postive
TRAJ12	AC027097.2	0.575673547	1.53E-48	postive

TRAJ13	AC027097.2	0.659363831	4.88E-68	postive
TRAJ14	AC027097.2	0.638558916	1.27E-62	postive
TRAJ16	AC027097.2	0.655788236	4.46E-67	postive
TRAJ17	AC027097.2	0.636392165	4.40E-62	postive
TRAJ18	AC027097.2	0.606678982	4.31E-55	postive
TRAJ21	AC027097.2	0.679930166	7.81E-74	postive
TRAJ31	AC027097.2	0.689499309	1.07E-76	postive
TRAJ37	AC027097.2	0.66660469	5.02E-70	postive
TRAJ38	AC027097.2	0.711255306	1.23E-83	postive
TRAJ39	AC027097.2	0.712206181	5.93E-84	postive
KCNH2	MIAT	0.683045693	9.40E-75	postive
CHGA	MIAT	0.730399736	2.67E-90	postive
GRP	MIAT	0.738282377	3.27E-93	postive
CREB1	EP300-AS1	0.684415283	3.67E-75	postive
RFXAP	EP300-AS1	0.526383333	1.83E-39	postive
UBR1	EP300-AS1	0.596633434	6.83E-53	postive
CYLD	EP300-AS1	0.540746123	5.90E-42	postive
IREB2	EP300-AS1	0.508269876	1.74E-36	postive
DDX17	EP300-AS1	0.659487421	4.52E-68	postive
JAK2	EP300-AS1	0.538392531	1.54E-41	postive
TXK	EP300-AS1	0.580291581	1.79E-49	postive
NFAT5	EP300-AS1	0.735404372	3.89E-92	postive
PIK3R1	EP300-AS1	0.652249337	3.87E-66	postive
PIK3CA	EP300-AS1	0.573795926	3.61E-48	postive
GNRH1	EP300-AS1	0.656775529	2.43E-67	postive
IL6ST	EP300-AS1	0.61822751	1.01E-57	postive
ACVR2A	EP300-AS1	0.579356241	2.77E-49	postive
ANGPTL1	EP300-AS1	0.663902025	2.81E-69	postive
BMPR2	EP300-AS1	0.670224647	4.85E-71	postive
CRLF3	EP300-AS1	0.556850695	6.76E-45	postive
NR1D2	EP300-AS1	0.511858473	4.61E-37	postive
NR2C2	EP300-AS1	0.633785003	1.94E-61	postive
NR3C2	EP300-AS1	0.536895588	2.82E-41	postive
RORA	EP300-AS1	0.780724241	6.36E-111	postive
SOS1	EP300-AS1	0.555430528	1.25E-44	postive
SOS2	EP300-AS1	0.530295313	3.94E-40	postive
BRAF	EP300-AS1	0.731059463	1.54E-90	postive
CBLB	EP300-AS1	0.626622164	1.06E-59	postive
RASGRP1	EP300-AS1	0.511885368	4.57E-37	postive
TRAJ1	EP300-AS1	0.602734713	3.22E-54	postive
TRAJ2	EP300-AS1	0.617115231	1.84E-57	postive
TRAJ3	EP300-AS1	0.636883327	3.32E-62	postive
TRAJ5	EP300-AS1	0.656793172	2.40E-67	postive
TRAJ6	EP300-AS1	0.640574371	3.95E-63	postive
TRAJ8	EP300-AS1	0.639507876	7.33E-63	postive
TRAJ10	EP300-AS1	0.618159531	1.05E-57	postive
TRAJ12	EP300-AS1	0.577030002	8.15E-49	postive
TRAJ13	EP300-AS1	0.657233976	1.83E-67	postive
TRAJ14	EP300-AS1	0.628625275	3.50E-60	postive
TRAJ16	EP300-AS1	0.65008358	1.43E-65	postive
TRAJ17	EP300-AS1	0.605949799	6.26E-55	postive
TRAJ18	EP300-AS1	0.593239879	3.63E-52	postive
TRAJ21	EP300-AS1	0.673254185	6.69E-72	postive
TRAJ31	EP300-AS1	0.688142201	2.78E-76	postive
TRAJ37	EP300-AS1	0.67481609	2.39E-72	postive
TRAJ38	EP300-AS1	0.710421115	2.34E-83	postive
TRAJ39	EP300-AS1	0.692279645	1.51E-77	postive
CREB1	UGDH-AS1	0.596374522	7.76E-53	postive
UBR1	UGDH-AS1	0.562942566	4.74E-46	postive
DDX17	UGDH-AS1	0.546137359	6.37E-43	postive
NFAT5	UGDH-AS1	0.646935538	9.41E-65	postive
GNRH1	UGDH-AS1	0.548416239	2.46E-43	postive
IL6ST	UGDH-AS1	0.520831707	1.56E-38	postive
ACVR2A	UGDH-AS1	0.546872946	4.69E-43	postive
ANGPTL1	UGDH-AS1	0.533116965	1.29E-40	postive

BMPR2	UGDH-AS1	0.508147632	1.82E-36	postive
NR2C2	UGDH-AS1	0.55068725	9.43E-44	postive
RORA	UGDH-AS1	0.663227736	4.31E-69	postive
BRAF	UGDH-AS1	0.595773371	1.05E-52	postive
TRAJ2	UGDH-AS1	0.537085876	2.61E-41	postive
TRAJ3	UGDH-AS1	0.546526811	5.42E-43	postive
TRAJ5	UGDH-AS1	0.547299631	3.92E-43	postive
TRAJ6	UGDH-AS1	0.557917247	4.26E-45	postive
TRAJ8	UGDH-AS1	0.540920867	5.49E-42	postive
TRAJ10	UGDH-AS1	0.538043531	1.77E-41	postive
TRAJ12	UGDH-AS1	0.523884589	4.83E-39	postive
TRAJ13	UGDH-AS1	0.559877316	1.82E-45	postive
TRAJ14	UGDH-AS1	0.544242971	1.40E-42	postive
TRAJ16	UGDH-AS1	0.535688887	4.59E-41	postive
TRAJ17	UGDH-AS1	0.50797798	1.93E-36	postive
TRAJ18	UGDH-AS1	0.512296044	3.92E-37	postive
TRAJ21	UGDH-AS1	0.574997036	2.08E-48	postive
TRAJ31	UGDH-AS1	0.576601103	9.94E-49	postive
TRAJ37	UGDH-AS1	0.592216128	5.99E-52	postive
TRAJ38	UGDH-AS1	0.614032212	9.40E-57	postive
TRAJ39	UGDH-AS1	0.585703813	1.39E-50	postive
CREB1	AL031670.1	0.690458183	5.47E-77	postive
RFXAP	AL031670.1	0.555052304	1.47E-44	postive
UBR1	AL031670.1	0.526671474	1.64E-39	postive
ZC3HAV1	AL031670.1	0.53771702	2.02E-41	postive
IL15	AL031670.1	0.519769824	2.35E-38	postive
CYLD	AL031670.1	0.578684435	3.78E-49	postive
EIF2AK2	AL031670.1	0.543209223	2.14E-42	postive
MAPK8	AL031670.1	0.513889466	2.16E-37	postive
NFKBIZ	AL031670.1	0.526417159	1.81E-39	postive
IREB2	AL031670.1	0.514730648	1.58E-37	postive
DDX17	AL031670.1	0.692752779	1.08E-77	postive
JAK2	AL031670.1	0.58347515	3.99E-50	postive
TXK	AL031670.1	0.57799574	5.21E-49	postive
NFAT5	AL031670.1	0.746824155	1.72E-96	postive
MALT1	AL031670.1	0.540359528	6.91E-42	postive
PIK3R1	AL031670.1	0.585824343	1.31E-50	postive
PIK3CA	AL031670.1	0.640393435	4.39E-63	postive
RASGRP3	AL031670.1	0.509616617	1.06E-36	postive
LTB4R2	AL031670.1	0.553648331	2.68E-44	postive
GNRH1	AL031670.1	0.761064743	2.93E-102	postive
IL6ST	AL031670.1	0.545756888	7.46E-43	postive
RABEP1	AL031670.1	0.504979613	5.78E-36	postive
ACVR2A	AL031670.1	0.568974586	3.22E-47	postive
ANGPTL1	AL031670.1	0.71625766	2.53E-85	postive
BMPR2	AL031670.1	0.626555992	1.10E-59	postive
CRLF3	AL031670.1	0.567656724	5.83E-47	postive
NR2C1	AL031670.1	0.593932908	2.59E-52	postive
NR2C2	AL031670.1	0.721543084	3.80E-87	postive
RORA	AL031670.1	0.793525637	4.61E-117	postive
SOS1	AL031670.1	0.651493309	6.12E-66	postive
SOS2	AL031670.1	0.537525642	2.19E-41	postive
BRAF	AL031670.1	0.732757082	3.69E-91	postive
ITK	AL031670.1	0.583221117	4.50E-50	postive
CBL	AL031670.1	0.624767611	2.94E-59	postive
CBLB	AL031670.1	0.709546211	4.56E-83	postive
PDK1	AL031670.1	0.545651057	7.80E-43	postive
TRAJ1	AL031670.1	0.654602902	9.23E-67	postive
TRAJ2	AL031670.1	0.642604574	1.21E-63	postive
TRAJ3	AL031670.1	0.674991995	2.12E-72	postive
TRAJ5	AL031670.1	0.722076564	2.47E-87	postive
TRAJ6	AL031670.1	0.65799885	1.14E-67	postive
TRAJ8	AL031670.1	0.638401851	1.39E-62	postive
TRAJ10	AL031670.1	0.639711822	6.52E-63	postive
TRAJ12	AL031670.1	0.598908025	2.20E-53	postive

TRAJ13	AL031670.1	0.681301588	3.09E-74	postive
TRAJ14	AL031670.1	0.653900879	1.42E-66	postive
TRAJ16	AL031670.1	0.667721815	2.45E-70	postive
TRAJ17	AL031670.1	0.63937715	7.91E-63	postive
TRAJ18	AL031670.1	0.636140998	5.08E-62	postive
TRAJ21	AL031670.1	0.706361285	5.10E-82	postive
TRAJ31	AL031670.1	0.715823117	3.56E-85	postive
TRAJ37	AL031670.1	0.691499676	2.62E-77	postive
TRAJ38	AL031670.1	0.732599098	4.21E-91	postive
TRAJ39	AL031670.1	0.708585349	9.49E-83	postive
CREB1	AC008870.2	0.500302303	3.12E-35	postive
NFKBIZ	AC008870.2	0.54530956	8.99E-43	postive
DDX17	AC008870.2	0.678747658	1.73E-73	postive
NFAT5	AC008870.2	0.603924194	1.76E-54	postive
LTB4R2	AC008870.2	0.594534472	1.92E-52	postive
GNRH1	AC008870.2	0.733558251	1.87E-91	postive
ANGPTL1	AC008870.2	0.543264257	2.10E-42	postive
NR2C1	AC008870.2	0.555799455	1.06E-44	postive
NR2C2	AC008870.2	0.681104757	3.53E-74	postive
RORA	AC008870.2	0.63998323	5.57E-63	postive
BRAF	AC008870.2	0.618415001	9.17E-58	postive
CBLB	AC008870.2	0.586377062	1.00E-50	postive
TRAJ2	AC008870.2	0.519436644	2.66E-38	postive
TRAJ3	AC008870.2	0.543842246	1.65E-42	postive
TRAJ5	AC008870.2	0.536585609	3.20E-41	postive
TRAJ16	AC008870.2	0.515118213	1.37E-37	postive
TRAJ21	AC008870.2	0.521091402	1.42E-38	postive
TRAJ31	AC008870.2	0.517792519	4.98E-38	postive
TRAJ37	AC008870.2	0.515523902	1.17E-37	postive
TRAJ38	AC008870.2	0.559642557	2.01E-45	postive
TRAJ39	AC008870.2	0.552006118	5.39E-44	postive
BPIFB1	SRGAP3-AS2	0.598020935	3.43E-53	postive
DES	SRGAP3-AS2	0.689448256	1.11E-76	postive
DDX17	AC121761.2	0.51425883	1.88E-37	postive
NFAT5	AC121761.2	0.54965535	1.46E-43	postive
GNRH1	AC121761.2	0.638373399	1.41E-62	postive
NR2C1	AC121761.2	0.513234423	2.76E-37	postive
TRAJ2	AC121761.2	0.501201301	2.26E-35	postive
TRAJ37	AC121761.2	0.510575434	7.43E-37	postive
TRAJ38	AC121761.2	0.505257018	5.22E-36	postive
CREB1	AC124283.3	0.788451404	1.41E-114	postive
RFXAP	AC124283.3	0.511406051	5.46E-37	postive
UBR1	AC124283.3	0.634680381	1.17E-61	postive
ZC3HAV1	AC124283.3	0.570206086	1.85E-47	postive
ZC3HAV1L	AC124283.3	0.553243618	3.18E-44	postive
IL15	AC124283.3	0.600175258	1.17E-53	postive
CYLD	AC124283.3	0.573143361	4.87E-48	postive
EIF2AK2	AC124283.3	0.550828777	8.88E-44	postive
MAPK8	AC124283.3	0.575221238	1.88E-48	postive
TLR1	AC124283.3	0.506016257	3.96E-36	postive
LMBR1	AC124283.3	0.602123276	4.38E-54	postive
LIMS1	AC124283.3	0.524173106	4.32E-39	postive
IREB2	AC124283.3	0.617027419	1.92E-57	postive
DDX17	AC124283.3	0.604774537	1.14E-54	postive
PIK3CG	AC124283.3	0.533715806	1.01E-40	postive
JAK2	AC124283.3	0.633410162	2.40E-61	postive
TXK	AC124283.3	0.577948669	5.33E-49	postive
NFAT5	AC124283.3	0.815218056	1.57E-128	postive
MALT1	AC124283.3	0.548912222	1.99E-43	postive
PIK3R1	AC124283.3	0.611317049	3.91E-56	postive
PIK3CA	AC124283.3	0.707293332	2.53E-82	postive
PIK3CB	AC124283.3	0.537522371	2.19E-41	postive
AKT3	AC124283.3	0.588159745	4.27E-51	postive
RASGRP3	AC124283.3	0.53619015	3.75E-41	postive
IGHD6-6	AC124283.3	0.514150752	1.96E-37	postive

PLXNC1	AC124283.3	0.502501365	1.42E-35	postive
GNRH1	AC124283.3	0.750612062	5.49E-98	postive
IL6ST	AC124283.3	0.590737595	1.23E-51	postive
RABEP1	AC124283.3	0.568083555	4.81E-47	postive
ACVR2A	AC124283.3	0.619847956	4.25E-58	postive
ANGPTL1	AC124283.3	0.799318022	5.49E-120	postive
BMPR1A	AC124283.3	0.531945233	2.05E-40	postive
BMPR2	AC124283.3	0.723132204	1.05E-87	postive
CRLF3	AC124283.3	0.678272326	2.39E-73	postive
NR2C2	AC124283.3	0.707415802	2.30E-82	postive
RORA	AC124283.3	0.795211338	6.64E-118	postive
SOS1	AC124283.3	0.715089098	6.32E-85	postive
SOS2	AC124283.3	0.59802227	3.43E-53	postive
BRAF	AC124283.3	0.8143542	4.80E-128	postive
ITK	AC124283.3	0.508715715	1.47E-36	postive
CBL	AC124283.3	0.640749189	3.57E-63	postive
CBLB	AC124283.3	0.739209711	1.46E-93	postive
RASGRP1	AC124283.3	0.509643221	1.05E-36	postive
PDK1	AC124283.3	0.625448328	2.02E-59	postive
TRAJ1	AC124283.3	0.743397625	3.69E-95	postive
TRAJ2	AC124283.3	0.724126517	4.71E-88	postive
TRAJ3	AC124283.3	0.794929937	9.19E-118	postive
TRAJ5	AC124283.3	0.799237013	6.05E-120	postive
TRAJ6	AC124283.3	0.795953747	2.81E-118	postive
TRAJ8	AC124283.3	0.789937774	2.68E-115	postive
TRAJ10	AC124283.3	0.772486243	3.47E-107	postive
TRAJ12	AC124283.3	0.68372768	5.89E-75	postive
TRAJ13	AC124283.3	0.797013345	8.21E-119	postive
TRAJ14	AC124283.3	0.781672976	2.30E-111	postive
TRAJ16	AC124283.3	0.798570534	1.33E-119	postive
TRAJ17	AC124283.3	0.765990646	2.38E-104	postive
TRAJ18	AC124283.3	0.695462538	1.55E-78	postive
TRAJ21	AC124283.3	0.816672933	2.36E-129	postive
TRAJ31	AC124283.3	0.821546428	3.65E-132	postive
TRAJ37	AC124283.3	0.784924458	6.84E-113	postive
TRAJ38	AC124283.3	0.855828319	1.03E-154	postive
TRAJ39	AC124283.3	0.836940695	1.22E-141	postive
IRF3	AL513320.1	0.57563493	1.55E-48	postive
TYK2	AL513320.1	0.554465722	1.89E-44	postive
IRF9	AL513320.1	0.618455326	8.97E-58	postive
RABEP2	AL513320.1	0.503265457	1.07E-35	postive
TNFRSF14	AL513320.1	0.666031514	7.24E-70	postive
TNFRSF25	AL513320.1	0.528672583	7.47E-40	postive
CREB1	PAXBP1-AS1	0.725994374	1.02E-88	postive
RFXAP	PAXBP1-AS1	0.57793186	5.37E-49	postive
UBR1	PAXBP1-AS1	0.59755701	4.32E-53	postive
ZC3HAV1	PAXBP1-AS1	0.537633488	2.09E-41	postive
ZC3HAV1L	PAXBP1-AS1	0.565689822	1.40E-46	postive
IFNAR1	PAXBP1-AS1	0.520396209	1.85E-38	postive
CYLD	PAXBP1-AS1	0.575680696	1.52E-48	postive
EIF2AK2	PAXBP1-AS1	0.520870737	1.54E-38	postive
MAPK8	PAXBP1-AS1	0.544270173	1.38E-42	postive
LMBR1	PAXBP1-AS1	0.551559538	6.52E-44	postive
IREB2	PAXBP1-AS1	0.577235693	7.41E-49	postive
DDX17	PAXBP1-AS1	0.632751932	3.47E-61	postive
PIK3CG	PAXBP1-AS1	0.510602283	7.35E-37	postive
JAK2	PAXBP1-AS1	0.608525753	1.66E-55	postive
TXK	PAXBP1-AS1	0.533731442	1.01E-40	postive
NFAT5	PAXBP1-AS1	0.764945918	6.67E-104	postive
MALT1	PAXBP1-AS1	0.505462239	4.85E-36	postive
PIK3R1	PAXBP1-AS1	0.598362244	2.89E-53	postive
PIK3CA	PAXBP1-AS1	0.622987036	7.77E-59	postive
AKT3	PAXBP1-AS1	0.541288079	4.73E-42	postive
RASGRP3	PAXBP1-AS1	0.506584485	3.22E-36	postive
LTB4R2	PAXBP1-AS1	0.52825856	8.79E-40	postive

GNRH1	PAXBP1-AS1	0.71435685	1.12E-84	postive
IL6ST	PAXBP1-AS1	0.562273797	6.36E-46	postive
RABEP1	PAXBP1-AS1	0.561773037	7.93E-46	postive
ACVR2A	PAXBP1-AS1	0.564768588	2.11E-46	postive
ANGPTL1	PAXBP1-AS1	0.686069194	1.17E-75	postive
BMPR2	PAXBP1-AS1	0.653187309	2.19E-66	postive
CRLF3	PAXBP1-AS1	0.628997583	2.85E-60	postive
NR2C1	PAXBP1-AS1	0.538895752	1.25E-41	postive
NR2C2	PAXBP1-AS1	0.72559944	1.42E-88	postive
RORA	PAXBP1-AS1	0.767189665	7.24E-105	postive
SOS1	PAXBP1-AS1	0.666680764	4.78E-70	postive
SOS2	PAXBP1-AS1	0.578241052	4.65E-49	postive
BRAF	PAXBP1-AS1	0.775485102	1.58E-108	postive
CBL	PAXBP1-AS1	0.594742839	1.74E-52	postive
CBLB	PAXBP1-AS1	0.713986272	1.49E-84	postive
PDK1	PAXBP1-AS1	0.581940878	8.24E-50	postive
TRAJ1	PAXBP1-AS1	0.637296371	2.62E-62	postive
TRAJ2	PAXBP1-AS1	0.655257114	6.18E-67	postive
TRAJ3	PAXBP1-AS1	0.678329722	2.30E-73	postive
TRAJ5	PAXBP1-AS1	0.675895703	1.17E-72	postive
TRAJ6	PAXBP1-AS1	0.685305542	1.99E-75	postive
TRAJ8	PAXBP1-AS1	0.63075226	1.07E-60	postive
TRAJ10	PAXBP1-AS1	0.656570159	2.76E-67	postive
TRAJ12	PAXBP1-AS1	0.607125897	3.42E-55	postive
TRAJ13	PAXBP1-AS1	0.655673216	4.79E-67	postive
TRAJ14	PAXBP1-AS1	0.655490017	5.36E-67	postive
TRAJ16	PAXBP1-AS1	0.670069024	5.37E-71	postive
TRAJ17	PAXBP1-AS1	0.607617781	2.66E-55	postive
TRAJ18	PAXBP1-AS1	0.624344331	3.71E-59	postive
TRAJ21	PAXBP1-AS1	0.680430231	5.57E-74	postive
TRAJ31	PAXBP1-AS1	0.693072142	8.59E-78	postive
TRAJ37	PAXBP1-AS1	0.681571731	2.57E-74	postive
TRAJ38	PAXBP1-AS1	0.751171443	3.28E-98	postive
TRAJ39	PAXBP1-AS1	0.69359253	5.93E-78	postive
CREB1	AL137003.1	0.643014595	9.52E-64	postive
RFXAP	AL137003.1	0.524807843	3.38E-39	postive
UBR1	AL137003.1	0.502109149	1.63E-35	postive
CYLD	AL137003.1	0.537453713	2.25E-41	postive
NFKBIZ	AL137003.1	0.501754945	1.85E-35	postive
DDX17	AL137003.1	0.637251972	2.69E-62	postive
JAK2	AL137003.1	0.530691031	3.37E-40	postive
NFAT5	AL137003.1	0.622989878	7.76E-59	postive
PIK3R1	AL137003.1	0.604243723	1.50E-54	postive
PIK3CA	AL137003.1	0.54447256	1.27E-42	postive
PLXNC1	AL137003.1	0.511936492	4.48E-37	postive
GNRH1	AL137003.1	0.63369856	2.04E-61	postive
IL6ST	AL137003.1	0.601522346	5.93E-54	postive
ACVR2A	AL137003.1	0.546079691	6.53E-43	postive
ANGPTL1	AL137003.1	0.620218965	3.48E-58	postive
BMPR2	AL137003.1	0.612659333	1.94E-56	postive
CRLF3	AL137003.1	0.564995065	1.91E-46	postive
NR2C1	AL137003.1	0.514563756	1.68E-37	postive
NR2C2	AL137003.1	0.636803769	3.47E-62	postive
RORA	AL137003.1	0.703926602	3.16E-81	postive
SOS1	AL137003.1	0.577211119	7.50E-49	postive
SOS2	AL137003.1	0.553041562	3.47E-44	postive
BRAF	AL137003.1	0.700680435	3.49E-80	postive
ITK	AL137003.1	0.544394261	1.31E-42	postive
CBL	AL137003.1	0.514285037	1.87E-37	postive
CBLB	AL137003.1	0.633672984	2.06E-61	postive
RASGRP1	AL137003.1	0.536075665	3.93E-41	postive
TRAJ1	AL137003.1	0.574794883	2.29E-48	postive
TRAJ2	AL137003.1	0.558683108	3.06E-45	postive
TRAJ3	AL137003.1	0.597559418	4.31E-53	postive
TRAJ5	AL137003.1	0.660911233	1.85E-68	postive

TRAJ6	AL137003.1	0.566023742	1.21E-46	postive
TRAJ8	AL137003.1	0.589296434	2.47E-51	postive
TRAJ10	AL137003.1	0.55104444	8.11E-44	postive
TRAJ12	AL137003.1	0.523496809	5.61E-39	postive
TRAJ13	AL137003.1	0.608965872	1.33E-55	postive
TRAJ14	AL137003.1	0.586057086	1.17E-50	postive
TRAJ16	AL137003.1	0.589310373	2.45E-51	postive
TRAJ17	AL137003.1	0.572810073	5.67E-48	postive
TRAJ18	AL137003.1	0.534542015	7.28E-41	postive
TRAJ21	AL137003.1	0.621793547	1.49E-58	postive
TRAJ31	AL137003.1	0.63930428	8.25E-63	postive
TRAJ37	AL137003.1	0.60105356	7.52E-54	postive
TRAJ38	AL137003.1	0.648569486	3.55E-65	postive
TRAJ39	AL137003.1	0.633084321	2.88E-61	postive
CREB1	AC104695.4	0.704224852	2.53E-81	postive
UBR1	AC104695.4	0.558884881	2.80E-45	postive
ZC3HAV1	AC104695.4	0.501211583	2.25E-35	postive
IL15	AC104695.4	0.598742101	2.39E-53	postive
EIF2AK2	AC104695.4	0.52298076	6.85E-39	postive
MAPK8	AC104695.4	0.50906655	1.30E-36	postive
NFKBIZ	AC104695.4	0.537890943	1.89E-41	postive
LMBR1	AC104695.4	0.53889762	1.25E-41	postive
LIMS1	AC104695.4	0.512774102	3.28E-37	postive
IREB2	AC104695.4	0.554677005	1.72E-44	postive
DDX17	AC104695.4	0.52895116	6.70E-40	postive
JAK2	AC104695.4	0.601987074	4.69E-54	postive
TXK	AC104695.4	0.58111454	1.22E-49	postive
NFAT5	AC104695.4	0.684316323	3.93E-75	postive
MALT1	AC104695.4	0.508769105	1.45E-36	postive
PIK3R1	AC104695.4	0.571603635	9.82E-48	postive
PIK3CA	AC104695.4	0.688292496	2.50E-76	postive
PIK3CB	AC104695.4	0.547348118	3.84E-43	postive
RASGRP3	AC104695.4	0.52403556	4.56E-39	postive
GNRH1	AC104695.4	0.692896737	9.73E-78	postive
IL6ST	AC104695.4	0.561832591	7.72E-46	postive
ACVR2A	AC104695.4	0.554882899	1.58E-44	postive
ANGPTL1	AC104695.4	0.773149534	1.76E-107	postive
BMPR2	AC104695.4	0.691102022	3.47E-77	postive
CRLF3	AC104695.4	0.585688156	1.40E-50	postive
IL18R1	AC104695.4	0.505975461	4.02E-36	postive
NR2C2	AC104695.4	0.645152845	2.71E-64	postive
RORA	AC104695.4	0.742783516	6.35E-95	postive
SOS1	AC104695.4	0.638439674	1.36E-62	postive
SOS2	AC104695.4	0.525223818	2.87E-39	postive
BRAF	AC104695.4	0.709328032	5.39E-83	postive
CBL	AC104695.4	0.563248754	4.14E-46	postive
CBLB	AC104695.4	0.7092121	5.89E-83	postive
PDK1	AC104695.4	0.597497539	4.45E-53	postive
TRAJ1	AC104695.4	0.614846479	6.12E-57	postive
TRAJ2	AC104695.4	0.521019755	1.46E-38	postive
TRAJ3	AC104695.4	0.64000313	5.50E-63	postive
TRAJ5	AC104695.4	0.74150534	1.96E-94	postive
TRAJ6	AC104695.4	0.648355996	4.04E-65	postive
TRAJ8	AC104695.4	0.625231016	2.28E-59	postive
TRAJ10	AC104695.4	0.661473224	1.30E-68	postive
TRAJ12	AC104695.4	0.587367794	6.25E-51	postive
TRAJ13	AC104695.4	0.660263027	2.78E-68	postive
TRAJ14	AC104695.4	0.639958943	5.65E-63	postive
TRAJ16	AC104695.4	0.683659175	6.18E-75	postive
TRAJ17	AC104695.4	0.68363765	6.27E-75	postive
TRAJ18	AC104695.4	0.612566606	2.03E-56	postive
TRAJ21	AC104695.4	0.700768306	3.27E-80	postive
TRAJ31	AC104695.4	0.731239378	1.32E-90	postive
TRAJ37	AC104695.4	0.653067351	2.36E-66	postive
TRAJ38	AC104695.4	0.738940223	1.85E-93	postive

TRAJ39	AC104695.4	0.728391862	1.42E-89	postive
CREB1	AP001432.1	0.729798631	4.42E-90	postive
UBR1	AP001432.1	0.607720401	2.52E-55	postive
ZC3HAV1	AP001432.1	0.554676266	1.72E-44	postive
ZC3HAV1L	AP001432.1	0.608392534	1.78E-55	postive
IL15	AP001432.1	0.554456099	1.90E-44	postive
CYLD	AP001432.1	0.569867665	2.15E-47	postive
EIF2AK2	AP001432.1	0.530041097	4.36E-40	postive
MAPK8	AP001432.1	0.520099771	2.07E-38	postive
TLR1	AP001432.1	0.5248685	3.30E-39	postive
LMBR1	AP001432.1	0.583024994	4.94E-50	postive
IREB2	AP001432.1	0.585206051	1.76E-50	postive
DDX17	AP001432.1	0.571190726	1.18E-47	postive
PIK3CG	AP001432.1	0.529627823	5.13E-40	postive
JAK2	AP001432.1	0.645873738	1.77E-64	postive
TXK	AP001432.1	0.506751492	3.03E-36	postive
NFAT5	AP001432.1	0.773591505	1.12E-107	postive
PIK3R1	AP001432.1	0.520990942	1.47E-38	postive
PIK3CA	AP001432.1	0.646503723	1.22E-64	postive
PIK3CB	AP001432.1	0.534217079	8.29E-41	postive
RASGRP3	AP001432.1	0.509775746	9.98E-37	postive
PLXNC1	AP001432.1	0.516069048	9.55E-38	postive
GNRH1	AP001432.1	0.681772919	2.24E-74	postive
RABEP1	AP001432.1	0.569536633	2.50E-47	postive
ACVR2A	AP001432.1	0.564782399	2.10E-46	postive
ANGPTL1	AP001432.1	0.663676553	3.24E-69	postive
BMPR2	AP001432.1	0.610549121	5.83E-56	postive
CRLF3	AP001432.1	0.687321667	4.92E-76	postive
NR2C2	AP001432.1	0.71187212	7.67E-84	postive
RORA	AP001432.1	0.683666695	6.14E-75	postive
SOS1	AP001432.1	0.660899626	1.87E-68	postive
SOS2	AP001432.1	0.573018786	5.15E-48	postive
BRAF	AP001432.1	0.726842786	5.11E-89	postive
TEC	AP001432.1	0.532527523	1.63E-40	postive
CBL	AP001432.1	0.632090222	5.04E-61	postive
CBLB	AP001432.1	0.64940054	2.16E-65	postive
PDK1	AP001432.1	0.608905207	1.37E-55	postive
TRAJ1	AP001432.1	0.715101078	6.26E-85	postive
TRAJ2	AP001432.1	0.647988754	5.03E-65	postive
TRAJ3	AP001432.1	0.712436259	4.96E-84	postive
TRAJ5	AP001432.1	0.71196611	7.13E-84	postive
TRAJ6	AP001432.1	0.745467563	5.83E-96	postive
TRAJ8	AP001432.1	0.670909904	3.11E-71	postive
TRAJ10	AP001432.1	0.740178492	6.27E-94	postive
TRAJ12	AP001432.1	0.652560153	3.21E-66	postive
TRAJ13	AP001432.1	0.689428122	1.13E-76	postive
TRAJ14	AP001432.1	0.692652367	1.16E-77	postive
TRAJ16	AP001432.1	0.720499841	8.77E-87	postive
TRAJ17	AP001432.1	0.680224412	6.40E-74	postive
TRAJ18	AP001432.1	0.672365174	1.20E-71	postive
TRAJ21	AP001432.1	0.712906896	3.45E-84	postive
TRAJ31	AP001432.1	0.723855292	5.87E-88	postive
TRAJ37	AP001432.1	0.705158568	1.26E-81	postive
TRAJ38	AP001432.1	0.766305811	1.74E-104	postive
TRAJ39	AP001432.1	0.72953089	5.52E-90	postive
CREB1	CR936218.1	0.799268938	5.82E-120	postive
RFXAP	CR936218.1	0.566974816	7.90E-47	postive
UBR1	CR936218.1	0.669933422	5.86E-71	postive
ZC3HAV1	CR936218.1	0.589891348	1.85E-51	postive
ZC3HAV1L	CR936218.1	0.575568771	1.60E-48	postive
IL15	CR936218.1	0.574650185	2.44E-48	postive
CYLD	CR936218.1	0.587066912	7.22E-51	postive
EIF2AK2	CR936218.1	0.56739698	6.54E-47	postive
MAPK8	CR936218.1	0.595830357	1.02E-52	postive
LMBR1	CR936218.1	0.588308591	3.98E-51	postive

IREB2	CR936218.1	0.646934816	9.42E-65	postive
DDX17	CR936218.1	0.671811913	1.72E-71	postive
PIK3CG	CR936218.1	0.52166752	1.14E-38	postive
JAK2	CR936218.1	0.610987197	4.64E-56	postive
TXK	CR936218.1	0.612766976	1.83E-56	postive
PPP3CB	CR936218.1	0.504993273	5.75E-36	postive
NFAT5	CR936218.1	0.818223663	3.07E-130	postive
MALT1	CR936218.1	0.552920258	3.65E-44	postive
PIK3R1	CR936218.1	0.63891858	1.03E-62	postive
PIK3CA	CR936218.1	0.683588877	6.48E-75	postive
PIK3CB	CR936218.1	0.536407846	3.44E-41	postive
AKT3	CR936218.1	0.581725849	9.12E-50	postive
RASGRP3	CR936218.1	0.53569853	4.57E-41	postive
IGHD4-4	CR936218.1	0.54290687	2.43E-42	postive
IGHD6-6	CR936218.1	0.540778032	5.82E-42	postive
LTB4R2	CR936218.1	0.536178516	3.77E-41	postive
PLXNC1	CR936218.1	0.507570795	2.24E-36	postive
GNRH1	CR936218.1	0.812899356	3.11E-127	postive
IL6ST	CR936218.1	0.620999026	2.28E-58	postive
RABEP1	CR936218.1	0.596146353	8.69E-53	postive
ACVR2A	CR936218.1	0.675375295	1.65E-72	postive
ANGPTL1	CR936218.1	0.774100341	6.61E-108	postive
BMPR1A	CR936218.1	0.579903044	2.14E-49	postive
BMPR2	CR936218.1	0.750238306	7.74E-98	postive
CRLF3	CR936218.1	0.672825184	8.87E-72	postive
NR1D2	CR936218.1	0.507831664	2.04E-36	postive
NR2C1	CR936218.1	0.53625064	3.66E-41	postive
NR2C2	CR936218.1	0.771874623	6.47E-107	postive
RORA	CR936218.1	0.806696158	7.49E-124	postive
SOS1	CR936218.1	0.724239091	4.29E-88	postive
SOS2	CR936218.1	0.614970221	5.73E-57	postive
BRAF	CR936218.1	0.852068777	5.74E-152	postive
ITK	CR936218.1	0.514494832	1.73E-37	postive
CBL	CR936218.1	0.632517377	3.97E-61	postive
CBLB	CR936218.1	0.724338321	3.96E-88	postive
RASGRP1	CR936218.1	0.529922761	4.57E-40	postive
PDK1	CR936218.1	0.592048451	6.50E-52	postive
TRAJ1	CR936218.1	0.732602153	4.20E-91	postive
TRAJ2	CR936218.1	0.732250823	5.65E-91	postive
TRAJ3	CR936218.1	0.797215329	6.49E-119	postive
TRAJ5	CR936218.1	0.773729275	9.69E-108	postive
TRAJ6	CR936218.1	0.773407447	1.35E-107	postive
TRAJ8	CR936218.1	0.785241267	4.84E-113	postive
TRAJ10	CR936218.1	0.733511576	1.95E-91	postive
TRAJ12	CR936218.1	0.683745981	5.82E-75	postive
TRAJ13	CR936218.1	0.772965905	2.12E-107	postive
TRAJ14	CR936218.1	0.7589965	2.14E-101	postive
TRAJ16	CR936218.1	0.762869335	5.10E-103	postive
TRAJ17	CR936218.1	0.735059497	5.22E-92	postive
TRAJ18	CR936218.1	0.6682179	1.78E-70	postive
TRAJ21	CR936218.1	0.790277835	1.83E-115	postive
TRAJ31	CR936218.1	0.802359804	1.47E-121	postive
TRAJ37	CR936218.1	0.774489422	4.42E-108	postive
TRAJ38	CR936218.1	0.834176729	7.23E-140	postive
TRAJ39	CR936218.1	0.81433649	4.91E-128	postive
TMSB15A	AC109322.1	0.555815293	1.06E-44	postive
MSTN	AC109322.1	0.6241543	4.11E-59	postive
CREB1	GARS1-DT	0.654678781	8.81E-67	postive
RFXAP	GARS1-DT	0.522161235	9.39E-39	postive
UBR1	GARS1-DT	0.570592571	1.55E-47	postive
IL15	GARS1-DT	0.503721815	9.11E-36	postive
CYLD	GARS1-DT	0.640469959	4.20E-63	postive
DDX17	GARS1-DT	0.667599479	2.65E-70	postive
JAK2	GARS1-DT	0.560677909	1.28E-45	postive
NFAT5	GARS1-DT	0.863718617	9.67E-161	postive

NFATC3	GARS1-DT	0.60733273	3.08E-55	postive
PIK3R1	GARS1-DT	0.548897734	2.01E-43	postive
PIK3CA	GARS1-DT	0.504898317	5.95E-36	postive
LTB4R2	GARS1-DT	0.541369251	4.57E-42	postive
GNRH1	GARS1-DT	0.740176723	6.28E-94	postive
IL6ST	GARS1-DT	0.54454515	1.23E-42	postive
ACVR2A	GARS1-DT	0.56986055	2.16E-47	postive
ANGPTL1	GARS1-DT	0.549484491	1.57E-43	postive
BMPR2	GARS1-DT	0.551169829	7.69E-44	postive
CRLF3	GARS1-DT	0.537497083	2.21E-41	postive
NR2C1	GARS1-DT	0.526111432	2.04E-39	postive
NR2C2	GARS1-DT	0.738578857	2.53E-93	postive
RORA	GARS1-DT	0.766032363	2.28E-104	postive
SOS1	GARS1-DT	0.561626896	8.45E-46	postive
BRAF	GARS1-DT	0.696566605	7.00E-79	postive
CBLB	GARS1-DT	0.575827464	1.42E-48	postive
TRAJ1	GARS1-DT	0.767895989	3.58E-105	postive
TRAJ2	GARS1-DT	0.766622348	1.27E-104	postive
TRAJ3	GARS1-DT	0.80745204	2.94E-124	postive
TRAJ5	GARS1-DT	0.7358532	2.65E-92	postive
TRAJ6	GARS1-DT	0.816631777	2.49E-129	postive
TRAJ8	GARS1-DT	0.744149145	1.89E-95	postive
TRAJ10	GARS1-DT	0.811187839	2.74E-126	postive
TRAJ12	GARS1-DT	0.797403017	5.21E-119	postive
TRAJ13	GARS1-DT	0.758981147	2.17E-101	postive
TRAJ14	GARS1-DT	0.772762268	2.61E-107	postive
TRAJ16	GARS1-DT	0.790284108	1.81E-115	postive
TRAJ17	GARS1-DT	0.745975381	3.69E-96	postive
TRAJ18	GARS1-DT	0.807591374	2.48E-124	postive
TRAJ21	GARS1-DT	0.789847145	2.96E-115	postive
TRAJ31	GARS1-DT	0.799801262	3.10E-120	postive
TRAJ37	GARS1-DT	0.840312311	7.57E-144	postive
TRAJ38	GARS1-DT	0.83165556	2.80E-138	postive
TRAJ39	GARS1-DT	0.793503349	4.72E-117	postive
CREB1	AC245014.3	0.736049127	2.24E-92	postive
RFXAP	AC245014.3	0.527822936	1.04E-39	postive
UBR1	AC245014.3	0.661511824	1.27E-68	postive
ZC3HAV1	AC245014.3	0.570393274	1.70E-47	postive
IL15	AC245014.3	0.522879762	7.12E-39	postive
CYLD	AC245014.3	0.696136582	9.55E-79	postive
EIF2AK2	AC245014.3	0.553953733	2.35E-44	postive
LMBR1	AC245014.3	0.514888612	1.49E-37	postive
IREB2	AC245014.3	0.564189205	2.73E-46	postive
DDX17	AC245014.3	0.57467891	2.41E-48	postive
PIK3CG	AC245014.3	0.60600105	6.10E-55	postive
JAK2	AC245014.3	0.655681634	4.76E-67	postive
TXK	AC245014.3	0.595543445	1.17E-52	postive
NFAT5	AC245014.3	0.797745883	3.49E-119	postive
NFATC3	AC245014.3	0.580099473	1.96E-49	postive
MALT1	AC245014.3	0.530716244	3.34E-40	postive
PIK3R1	AC245014.3	0.659043844	5.95E-68	postive
PIK3CA	AC245014.3	0.666972369	3.96E-70	postive
PIK3CB	AC245014.3	0.510195362	8.55E-37	postive
RASGRP3	AC245014.3	0.515471949	1.20E-37	postive
IGHD6-6	AC245014.3	0.516147526	9.27E-38	postive
PLXNC1	AC245014.3	0.508819847	1.42E-36	postive
GNRH1	AC245014.3	0.620803526	2.54E-58	postive
IL6ST	AC245014.3	0.616987803	1.96E-57	postive
RABEP1	AC245014.3	0.514973127	1.44E-37	postive
ACVR2A	AC245014.3	0.587827534	5.01E-51	postive
ANGPTL1	AC245014.3	0.662020493	9.23E-69	postive
BMPR1A	AC245014.3	0.518994828	3.15E-38	postive
BMPR2	AC245014.3	0.656271377	3.31E-67	postive
CRLF3	AC245014.3	0.588113273	4.37E-51	postive
LIFR	AC245014.3	0.503359967	1.04E-35	postive

NR1D2	AC245014.3	0.539601106	9.42E-42	postive
NR2C2	AC245014.3	0.66684464	4.30E-70	postive
RORA	AC245014.3	0.817853427	5.01E-130	postive
SOS1	AC245014.3	0.610018048	7.68E-56	postive
SOS2	AC245014.3	0.540797134	5.78E-42	postive
BRAF	AC245014.3	0.741385433	2.18E-94	postive
ITK	AC245014.3	0.513652864	2.36E-37	postive
CBL	AC245014.3	0.520470839	1.80E-38	postive
CBLB	AC245014.3	0.650415743	1.17E-65	postive
PDK1	AC245014.3	0.536249746	3.66E-41	postive
TRAJ1	AC245014.3	0.706436369	4.82E-82	postive
TRAJ2	AC245014.3	0.720214854	1.10E-86	postive
TRAJ3	AC245014.3	0.756458689	2.38E-100	postive
TRAJ5	AC245014.3	0.743570671	3.16E-95	postive
TRAJ6	AC245014.3	0.753197516	5.05E-99	postive
TRAJ8	AC245014.3	0.705732501	8.18E-82	postive
TRAJ10	AC245014.3	0.73520678	4.60E-92	postive
TRAJ12	AC245014.3	0.74507854	8.25E-96	postive
TRAJ13	AC245014.3	0.719891818	1.42E-86	postive
TRAJ14	AC245014.3	0.744225112	1.77E-95	postive
TRAJ16	AC245014.3	0.765053598	6.00E-104	postive
TRAJ17	AC245014.3	0.731591043	9.85E-91	postive
TRAJ18	AC245014.3	0.768377676	2.21E-105	postive
TRAJ21	AC245014.3	0.777626046	1.69E-109	postive
TRAJ31	AC245014.3	0.766608682	1.29E-104	postive
TRAJ37	AC245014.3	0.754971712	9.64E-100	postive
TRAJ38	AC245014.3	0.832855231	4.96E-139	postive
TRAJ39	AC245014.3	0.797754222	3.46E-119	postive
CREB1	LINC01578	0.595062394	1.48E-52	postive
UBR1	LINC01578	0.534910971	6.28E-41	postive
IL15	LINC01578	0.512313485	3.90E-37	postive
DDX17	LINC01578	0.543395804	1.99E-42	postive
NFAT5	LINC01578	0.666999862	3.89E-70	postive
PIK3R1	LINC01578	0.525635959	2.45E-39	postive
GNRH1	LINC01578	0.656957805	2.17E-67	postive
IL6ST	LINC01578	0.563153342	4.32E-46	postive
ANGPTL1	LINC01578	0.607477868	2.86E-55	postive
BMPR2	LINC01578	0.544829232	1.10E-42	postive
NR2C2	LINC01578	0.618308457	9.71E-58	postive
RORA	LINC01578	0.664918268	1.47E-69	postive
BRAF	LINC01578	0.676043328	1.06E-72	postive
CBLB	LINC01578	0.551618146	6.36E-44	postive
TRAJ1	LINC01578	0.569244521	2.85E-47	postive
TRAJ2	LINC01578	0.563163051	4.30E-46	postive
TRAJ3	LINC01578	0.650360675	1.21E-65	postive
TRAJ5	LINC01578	0.627743577	5.71E-60	postive
TRAJ6	LINC01578	0.631606111	6.62E-61	postive
TRAJ8	LINC01578	0.580713409	1.47E-49	postive
TRAJ10	LINC01578	0.625454439	2.02E-59	postive
TRAJ12	LINC01578	0.626420702	1.19E-59	postive
TRAJ13	LINC01578	0.604035493	1.66E-54	postive
TRAJ14	LINC01578	0.625392611	2.09E-59	postive
TRAJ16	LINC01578	0.603113531	2.66E-54	postive
TRAJ17	LINC01578	0.600809777	8.50E-54	postive
TRAJ18	LINC01578	0.630793077	1.04E-60	postive
TRAJ21	LINC01578	0.655634792	4.90E-67	postive
TRAJ31	LINC01578	0.661977579	9.49E-69	postive
TRAJ37	LINC01578	0.652657944	3.02E-66	postive
TRAJ38	LINC01578	0.687365122	4.78E-76	postive
TRAJ39	LINC01578	0.657742643	1.34E-67	postive
TYK2	AC003070.1	0.514881676	1.49E-37	postive
IRF9	AC003070.1	0.544909687	1.06E-42	postive
LTB4R2	AC003070.1	0.50754229	2.27E-36	postive
GIPR	AC003070.1	0.532467381	1.67E-40	postive
DDX17	AC073957.3	0.526206309	1.96E-39	postive

LTB4R2	AC073957.3	0.606686949	4.29E-55	postive
GNRH1	AC073957.3	0.546448839	5.59E-43	postive
NR2C2	AC073957.3	0.615287617	4.85E-57	postive
BRAF	AC073957.3	0.531533183	2.41E-40	postive
FGF2	AP001528.1	0.626225892	1.32E-59	postive
NFAT5	AP001528.1	0.601008342	7.69E-54	postive
GNRH1	AP001528.1	0.50210882	1.63E-35	postive
ANGPTL1	AP001528.1	0.508390915	1.66E-36	postive
BMPR2	AP001528.1	0.503208472	1.10E-35	postive
RORA	AP001528.1	0.614059221	9.27E-57	postive
TRAJ5	AP001528.1	0.50009217	3.36E-35	postive
TRAJ21	AP001528.1	0.508732978	1.47E-36	postive
TRAJ31	AP001528.1	0.536015976	4.02E-41	postive
TRAJ37	AP001528.1	0.532305504	1.78E-40	postive
TRAJ38	AP001528.1	0.546258609	6.06E-43	postive
TRAJ39	AP001528.1	0.535982222	4.08E-41	postive
CD8A	PRKAR1B-AS1	0.511596262	5.09E-37	postive
FCER1G	PRKAR1B-AS1	0.636752913	3.58E-62	postive
CXCL10	PRKAR1B-AS1	0.502801118	1.27E-35	postive
APOBEC3G	PRKAR1B-AS1	0.568066635	4.85E-47	postive
CCL5	PRKAR1B-AS1	0.506429144	3.41E-36	postive
CCL4	PRKAR1B-AS1	0.590219002	1.58E-51	postive
CCL3	PRKAR1B-AS1	0.66920864	9.38E-71	postive
CCL4L2	PRKAR1B-AS1	0.554189931	2.12E-44	postive
CXCR6	PRKAR1B-AS1	0.50878594	1.44E-36	postive
CD86	PRKAR1B-AS1	0.54327442	2.09E-42	postive
BTK	PRKAR1B-AS1	0.541034855	5.24E-42	postive
RAC2	PRKAR1B-AS1	0.547961046	2.97E-43	postive
CD72	PRKAR1B-AS1	0.525921835	2.19E-39	postive
FCGR2B	PRKAR1B-AS1	0.503775335	8.94E-36	postive
GMFG	PRKAR1B-AS1	0.687158056	5.51E-76	postive
IL32	PRKAR1B-AS1	0.500285693	3.14E-35	postive
TNFSF13B	PRKAR1B-AS1	0.503747176	9.03E-36	postive
TNFRSF4	PRKAR1B-AS1	0.571925798	8.48E-48	postive
TYROBP	PRKAR1B-AS1	0.660567504	2.30E-68	postive
LCK	PRKAR1B-AS1	0.501683471	1.90E-35	postive
CD247	PRKAR1B-AS1	0.504197636	7.67E-36	postive
HCST	PRKAR1B-AS1	0.720210904	1.10E-86	postive
CD48	PRKAR1B-AS1	0.5305579	3.55E-40	postive
SH2D1A	PRKAR1B-AS1	0.553514144	2.84E-44	postive
CD3D	PRKAR1B-AS1	0.624132836	4.16E-59	postive
TRAC	PRKAR1B-AS1	0.51451647	1.71E-37	postive
TRAV13-1	PRKAR1B-AS1	0.581346278	1.09E-49	postive
TRBC2	PRKAR1B-AS1	0.580566614	1.57E-49	postive
CIITA	DBH-AS1	0.508375044	1.67E-36	postive
TAP2	DBH-AS1	0.501894292	1.76E-35	postive
PDCD1	DBH-AS1	0.628767713	3.23E-60	postive
FASLG	DBH-AS1	0.536190444	3.75E-41	postive
PIK3CD	DBH-AS1	0.506100064	3.84E-36	postive
SEMA4D	DBH-AS1	0.503545432	9.71E-36	postive
CXCR3	DBH-AS1	0.50333873	1.05E-35	postive
FLT3LG	DBH-AS1	0.518484427	3.83E-38	postive
IL12RB1	DBH-AS1	0.537194219	2.50E-41	postive
IL2RB	DBH-AS1	0.55924373	2.40E-45	postive
IL18RAP	DBH-AS1	0.547593188	3.47E-43	postive
IL21R	DBH-AS1	0.516093108	9.46E-38	postive
ITGAL	DBH-AS1	0.566808179	8.52E-47	postive
CD247	DBH-AS1	0.586066676	1.17E-50	postive
ZAP70	DBH-AS1	0.729792368	4.44E-90	postive
CD3E	DBH-AS1	0.532359154	1.74E-40	postive
TRAC	DBH-AS1	0.521362929	1.28E-38	postive
TRBC1	DBH-AS1	0.580069789	1.98E-49	postive
TRBC2	DBH-AS1	0.517156449	6.33E-38	postive
TRBJ2-2	DBH-AS1	0.519335426	2.77E-38	postive
TRBJ2-7	DBH-AS1	0.580450789	1.66E-49	postive

TRBV28	DBH-AS1	0.514090235	2.01E-37	postive
AGTR2	RBPMS-AS1	0.541651312	4.07E-42	postive
B2M	PSMB8-AS1	0.618481016	8.85E-58	postive
CD8A	PSMB8-AS1	0.552752888	3.92E-44	postive
CD8B	PSMB8-AS1	0.51596806	9.92E-38	postive
CTSS	PSMB8-AS1	0.505044151	5.64E-36	postive
HLA-A	PSMB8-AS1	0.598392118	2.85E-53	postive
HLA-B	PSMB8-AS1	0.683909821	5.20E-75	postive
HLA-C	PSMB8-AS1	0.661510862	1.27E-68	postive
HLA-DMA	PSMB8-AS1	0.501511482	2.02E-35	postive
HLA-DMB	PSMB8-AS1	0.590171247	1.62E-51	postive
HLA-DOB	PSMB8-AS1	0.6093546	1.08E-55	postive
HLA-DPB1	PSMB8-AS1	0.511911808	4.52E-37	postive
HLA-DRA	PSMB8-AS1	0.561459148	9.10E-46	postive
HLA-E	PSMB8-AS1	0.65426859	1.13E-66	postive
HLA-F	PSMB8-AS1	0.731459505	1.10E-90	postive
LTA	PSMB8-AS1	0.605427506	8.18E-55	postive
PSMB8	PSMB8-AS1	0.682071902	1.83E-74	postive
PSME2	PSMB8-AS1	0.513338381	2.66E-37	postive
TAP1	PSMB8-AS1	0.579664939	2.40E-49	postive
TAP2	PSMB8-AS1	0.519892813	2.24E-38	postive
CXCL10	PSMB8-AS1	0.502779704	1.28E-35	postive
APOBEC3G	PSMB8-AS1	0.696315484	8.39E-79	postive
IRF1	PSMB8-AS1	0.60854184	1.65E-55	postive
CD40	PSMB8-AS1	0.503488482	9.92E-36	postive
CCL5	PSMB8-AS1	0.567452796	6.38E-47	postive
CCL4	PSMB8-AS1	0.508159573	1.81E-36	postive
PDCD1	PSMB8-AS1	0.520525955	1.76E-38	postive
FASLG	PSMB8-AS1	0.52539774	2.69E-39	postive
CCL19	PSMB8-AS1	0.502699416	1.32E-35	postive
CCR5	PSMB8-AS1	0.536298061	3.59E-41	postive
CXCR6	PSMB8-AS1	0.572427104	6.75E-48	postive
CD79B	PSMB8-AS1	0.515831445	1.04E-37	postive
BTK	PSMB8-AS1	0.560438543	1.42E-45	postive
CD72	PSMB8-AS1	0.574020104	3.26E-48	postive
EBI3	PSMB8-AS1	0.517632796	5.29E-38	postive
FLT3LG	PSMB8-AS1	0.614773909	6.36E-57	postive
GMFG	PSMB8-AS1	0.611874961	2.92E-56	postive
IL16	PSMB8-AS1	0.571322755	1.12E-47	postive
TNFSF13B	PSMB8-AS1	0.553027973	3.49E-44	postive
IL10RA	PSMB8-AS1	0.533765607	9.93E-41	postive
IL12RB1	PSMB8-AS1	0.58616977	1.11E-50	postive
IL2RB	PSMB8-AS1	0.510720428	7.04E-37	postive
IL18RAP	PSMB8-AS1	0.542304039	3.11E-42	postive
IL21R	PSMB8-AS1	0.506898752	2.87E-36	postive
IL2RG	PSMB8-AS1	0.524624743	3.63E-39	postive
ITGAL	PSMB8-AS1	0.563815332	3.22E-46	postive
LCK	PSMB8-AS1	0.545694961	7.66E-43	postive
NCR3	PSMB8-AS1	0.560959955	1.13E-45	postive
CD247	PSMB8-AS1	0.666556518	5.18E-70	postive
ZAP70	PSMB8-AS1	0.599363925	1.75E-53	postive
HCST	PSMB8-AS1	0.559807732	1.87E-45	postive
CD48	PSMB8-AS1	0.591698937	7.71E-52	postive
SH2D1A	PSMB8-AS1	0.643207244	8.51E-64	postive
CD3D	PSMB8-AS1	0.689006619	1.52E-76	postive
CD3E	PSMB8-AS1	0.633010698	3.00E-61	postive
CD3G	PSMB8-AS1	0.545687353	7.68E-43	postive
ICOS	PSMB8-AS1	0.54488507	1.07E-42	postive
TRAC	PSMB8-AS1	0.656886434	2.27E-67	postive
TRAV4	PSMB8-AS1	0.596079763	8.98E-53	postive
TRAV8-2	PSMB8-AS1	0.546850337	4.73E-43	postive
TRAV8-6	PSMB8-AS1	0.528781691	7.16E-40	postive
TRAV9-2	PSMB8-AS1	0.536189798	3.75E-41	postive
TRAV16	PSMB8-AS1	0.575974481	1.33E-48	postive
TRBC1	PSMB8-AS1	0.620770976	2.58E-58	postive

TRBC2	PSMB8-AS1	0.667481332	2.86E-70	postive
TRBJ2-2	PSMB8-AS1	0.516227855	8.99E-38	postive
TRBJ2-3	PSMB8-AS1	0.511060354	6.21E-37	postive
TRBJ2-7	PSMB8-AS1	0.513170182	2.83E-37	postive
TRBV3-1	PSMB8-AS1	0.51029058	8.25E-37	postive
TRBV5-1	PSMB8-AS1	0.564702178	2.18E-46	postive
TRBV5-4	PSMB8-AS1	0.514451539	1.75E-37	postive
TRBV6-5	PSMB8-AS1	0.551757713	5.99E-44	postive
TRBV7-9	PSMB8-AS1	0.55863744	3.12E-45	postive
TRBV11-2	PSMB8-AS1	0.511827199	4.67E-37	postive
TRBV18	PSMB8-AS1	0.526318881	1.88E-39	postive
TRBV20-1	PSMB8-AS1	0.535585042	4.79E-41	postive
TRBV28	PSMB8-AS1	0.670106289	5.24E-71	postive
TXLNA	MRPL20-AS1	0.500392823	3.02E-35	postive
CREB1	AC087286.1	0.713455173	2.25E-84	postive
UBR1	AC087286.1	0.643449396	7.38E-64	postive
ZC3HAV1	AC087286.1	0.51276548	3.29E-37	postive
EIF2AK2	AC087286.1	0.544253721	1.39E-42	postive
MAPK8	AC087286.1	0.533327982	1.18E-40	postive
LMBR1	AC087286.1	0.529700195	4.99E-40	postive
IREB2	AC087286.1	0.600651902	9.20E-54	postive
DDX17	AC087286.1	0.585618324	1.44E-50	postive
JAK2	AC087286.1	0.565696756	1.40E-46	postive
TXK	AC087286.1	0.657674335	1.39E-67	postive
NFAT5	AC087286.1	0.696540755	7.13E-79	postive
PIK3R1	AC087286.1	0.608000599	2.18E-55	postive
PIK3CA	AC087286.1	0.637278971	2.65E-62	postive
PIK3CB	AC087286.1	0.509717709	1.02E-36	postive
IGHD6-6	AC087286.1	0.518116003	4.40E-38	postive
ROBO2	AC087286.1	0.550992184	8.29E-44	postive
GNRH1	AC087286.1	0.760409358	5.51E-102	postive
IL6ST	AC087286.1	0.564680871	2.20E-46	postive
RABEP1	AC087286.1	0.529403893	5.60E-40	postive
ACVR2A	AC087286.1	0.596054054	9.10E-53	postive
ANGPTL1	AC087286.1	0.690798518	4.31E-77	postive
BMPR1A	AC087286.1	0.528046783	9.55E-40	postive
BMPR2	AC087286.1	0.735705984	3.00E-92	postive
CRLF3	AC087286.1	0.570566591	1.57E-47	postive
NR1D2	AC087286.1	0.503783824	8.91E-36	postive
NR2C2	AC087286.1	0.672041835	1.48E-71	postive
RORA	AC087286.1	0.746521426	2.26E-96	postive
SOS1	AC087286.1	0.628860728	3.07E-60	postive
BRAF	AC087286.1	0.734561038	7.98E-92	postive
TEC	AC087286.1	0.579444922	2.65E-49	postive
CBL	AC087286.1	0.524952961	3.19E-39	postive
CBLB	AC087286.1	0.707589132	2.02E-82	postive
PDK1	AC087286.1	0.567058452	7.61E-47	postive
TRAJ1	AC087286.1	0.535243269	5.49E-41	postive
TRAJ2	AC087286.1	0.528328332	8.55E-40	postive
TRAJ3	AC087286.1	0.593769264	2.80E-52	postive
TRAJ5	AC087286.1	0.598257487	3.05E-53	postive
TRAJ6	AC087286.1	0.580707676	1.47E-49	postive
TRAJ8	AC087286.1	0.538239163	1.64E-41	postive
TRAJ10	AC087286.1	0.527471274	1.20E-39	postive
TRAJ12	AC087286.1	0.551993011	5.42E-44	postive
TRAJ13	AC087286.1	0.543041082	2.30E-42	postive
TRAJ14	AC087286.1	0.538476965	1.49E-41	postive
TRAJ16	AC087286.1	0.568160451	4.65E-47	postive
TRAJ17	AC087286.1	0.520295529	1.92E-38	postive
TRAJ18	AC087286.1	0.533602738	1.06E-40	postive
TRAJ21	AC087286.1	0.592781911	4.55E-52	postive
TRAJ31	AC087286.1	0.622205883	1.19E-58	postive
TRAJ37	AC087286.1	0.582993991	5.02E-50	postive
TRAJ38	AC087286.1	0.665724304	8.82E-70	postive
TRAJ39	AC087286.1	0.631081086	8.89E-61	postive

TYK2	ASB16-AS1	0.503344117	1.04E-35	postive
NFKBIZ	LUCAT1	0.509213504	1.23E-36	postive
NENF	ZSCAN16-AS1	0.512877214	3.16E-37	postive
CREB1	LINC01389	0.535962376	4.11E-41	postive
NFAT5	LINC01389	0.58364227	3.69E-50	postive
GNRH1	LINC01389	0.651285107	6.94E-66	postive
ACVR2A	LINC01389	0.526279434	1.91E-39	postive
BMPR2	LINC01389	0.529219921	6.02E-40	postive
NR2C2	LINC01389	0.536417644	3.42E-41	postive
RORA	LINC01389	0.546586077	5.28E-43	postive
SOS1	LINC01389	0.510667405	7.18E-37	postive
BRAF	LINC01389	0.596779676	6.35E-53	postive
CBLB	LINC01389	0.511816023	4.69E-37	postive
TRAJ1	LINC01389	0.51944536	2.66E-38	postive
TRAJ3	LINC01389	0.554261227	2.06E-44	postive
TRAJ5	LINC01389	0.538265277	1.62E-41	postive
TRAJ6	LINC01389	0.55169065	6.16E-44	postive
TRAJ8	LINC01389	0.552859138	3.75E-44	postive
TRAJ10	LINC01389	0.514601011	1.66E-37	postive
TRAJ12	LINC01389	0.50459724	6.64E-36	postive
TRAJ13	LINC01389	0.537399528	2.30E-41	postive
TRAJ14	LINC01389	0.524890822	3.27E-39	postive
TRAJ16	LINC01389	0.554397529	1.94E-44	postive
TRAJ17	LINC01389	0.525390838	2.69E-39	postive
TRAJ21	LINC01389	0.553972916	2.33E-44	postive
TRAJ31	LINC01389	0.577090983	7.93E-49	postive
TRAJ37	LINC01389	0.543881414	1.62E-42	postive
TRAJ38	LINC01389	0.589775056	1.96E-51	postive
TRAJ39	LINC01389	0.571410712	1.07E-47	postive
IL11RA	AC080038.2	0.542103175	3.38E-42	postive
CREB1	LINC00630	0.799190975	6.38E-120	postive
RFXAP	LINC00630	0.542249944	3.18E-42	postive
UBR1	LINC00630	0.683920357	5.16E-75	postive
ZC3HAV1	LINC00630	0.590201695	1.60E-51	postive
ZC3HAV1L	LINC00630	0.589134583	2.67E-51	postive
IL15	LINC00630	0.548759456	2.13E-43	postive
CYLD	LINC00630	0.639673462	6.66E-63	postive
EIF2AK2	LINC00630	0.59998068	1.29E-53	postive
MAPK8	LINC00630	0.586989724	7.50E-51	postive
TLR1	LINC00630	0.504721764	6.34E-36	postive
LMBR1	LINC00630	0.662299647	7.74E-69	postive
EED	LINC00630	0.520217948	1.98E-38	postive
LIMS1	LINC00630	0.501846693	1.79E-35	postive
IREB2	LINC00630	0.644165002	4.85E-64	postive
DDX17	LINC00630	0.548635535	2.24E-43	postive
PIK3CG	LINC00630	0.582953801	5.11E-50	postive
JAK2	LINC00630	0.64884288	3.02E-65	postive
TXK	LINC00630	0.52284698	7.21E-39	postive
NFAT5	LINC00630	0.840737338	3.95E-144	postive
NFATC3	LINC00630	0.582940702	5.14E-50	postive
MALT1	LINC00630	0.546165389	6.30E-43	postive
PIK3R1	LINC00630	0.618051534	1.11E-57	postive
PIK3CA	LINC00630	0.714605138	9.22E-85	postive
PIK3CB	LINC00630	0.529314739	5.80E-40	postive
AKT3	LINC00630	0.519615667	2.49E-38	postive
GSK3B	LINC00630	0.505062059	5.61E-36	postive
IGHD6-6	LINC00630	0.500000147	3.47E-35	postive
PLXNC1	LINC00630	0.518470992	3.85E-38	postive
GNRH1	LINC00630	0.656649463	2.62E-67	postive
IL6ST	LINC00630	0.595844114	1.01E-52	postive
RABEP1	LINC00630	0.575201589	1.90E-48	postive
ACVR2A	LINC00630	0.593624533	3.01E-52	postive
ANGPTL1	LINC00630	0.659441655	4.65E-68	postive
BMPR1A	LINC00630	0.585300462	1.68E-50	postive
BMPR2	LINC00630	0.687908646	3.27E-76	postive

CRLF3	LINC00630	0.677155462	5.04E-73	postive
NR1D2	LINC00630	0.570017862	2.01E-47	postive
NR2C2	LINC00630	0.677339722	4.45E-73	postive
RORA	LINC00630	0.79913297	6.84E-120	postive
SOS1	LINC00630	0.668151252	1.86E-70	postive
SOS2	LINC00630	0.610738637	5.28E-56	postive
BRAF	LINC00630	0.795435155	5.13E-118	postive
CBL	LINC00630	0.585565218	1.48E-50	postive
CBLB	LINC00630	0.655853706	4.28E-67	postive
PDK1	LINC00630	0.621647436	1.61E-58	postive
TRAJ1	LINC00630	0.713946377	1.54E-84	postive
TRAJ2	LINC00630	0.744653163	1.21E-95	postive
TRAJ3	LINC00630	0.774800232	3.21E-108	postive
TRAJ5	LINC00630	0.734033463	1.25E-91	postive
TRAJ6	LINC00630	0.783026458	5.37E-112	postive
TRAJ8	LINC00630	0.727768173	2.38E-89	postive
TRAJ10	LINC00630	0.764992523	6.37E-104	postive
TRAJ12	LINC00630	0.716755698	1.71E-85	postive
TRAJ13	LINC00630	0.736440592	1.60E-92	postive
TRAJ14	LINC00630	0.73961063	1.03E-93	postive
TRAJ16	LINC00630	0.758857749	2.44E-101	postive
TRAJ17	LINC00630	0.712992409	3.23E-84	postive
TRAJ18	LINC00630	0.727311034	3.47E-89	postive
TRAJ21	LINC00630	0.770426956	2.81E-106	postive
TRAJ31	LINC00630	0.776688127	4.51E-109	postive
TRAJ37	LINC00630	0.77742561	2.08E-109	postive
TRAJ38	LINC00630	0.835569874	9.33E-141	postive
TRAJ39	LINC00630	0.788931616	8.24E-115	postive
NFKBIZ	AC062037.3	0.505675584	4.49E-36	postive
DDX17	AC062037.3	0.571424821	1.06E-47	postive
NFAT5	AC062037.3	0.609558115	9.75E-56	postive
LTB4R2	AC062037.3	0.535158889	5.68E-41	postive
GNRH1	AC062037.3	0.674578768	2.79E-72	postive
ANGPTL1	AC062037.3	0.502906588	1.22E-35	postive
NR2C2	AC062037.3	0.594864766	1.64E-52	postive
RORA	AC062037.3	0.549647119	1.46E-43	postive
BRAF	AC062037.3	0.50258106	1.38E-35	postive
TRAJ1	AC062037.3	0.588801494	3.14E-51	postive
TRAJ2	AC062037.3	0.530739851	3.31E-40	postive
TRAJ3	AC062037.3	0.601989714	4.69E-54	postive
TRAJ5	AC062037.3	0.562206334	6.55E-46	postive
TRAJ6	AC062037.3	0.587462205	5.98E-51	postive
TRAJ8	AC062037.3	0.540015535	7.95E-42	postive
TRAJ10	AC062037.3	0.598611022	2.56E-53	postive
TRAJ12	AC062037.3	0.529977292	4.47E-40	postive
TRAJ13	AC062037.3	0.565443659	1.56E-46	postive
TRAJ14	AC062037.3	0.550805624	8.97E-44	postive
TRAJ16	AC062037.3	0.580492754	1.63E-49	postive
TRAJ17	AC062037.3	0.562701299	5.27E-46	postive
TRAJ18	AC062037.3	0.56190967	7.46E-46	postive
TRAJ21	AC062037.3	0.58151248	1.01E-49	postive
TRAJ31	AC062037.3	0.58634587	1.02E-50	postive
TRAJ37	AC062037.3	0.581158216	1.19E-49	postive
TRAJ38	AC062037.3	0.591872373	7.08E-52	postive
TRAJ39	AC062037.3	0.576486959	1.05E-48	postive
GNRH1	GPRC5D-AS1	0.537697027	2.04E-41	postive
DDX17	AC244197.2	0.525842372	2.26E-39	postive
IRF9	AC244197.2	0.528670757	7.48E-40	postive
LTB4R2	AC244197.2	0.504834536	6.09E-36	postive
GNRH1	AC244197.2	0.564687337	2.19E-46	postive
NR2C2	AC244197.2	0.543429548	1.96E-42	postive
TRAJ1	AC244197.2	0.511899175	4.55E-37	postive
TRAJ3	AC244197.2	0.517792517	4.98E-38	postive
TRAJ5	AC244197.2	0.500487017	2.92E-35	postive
TRAJ16	AC244197.2	0.512368503	3.82E-37	postive

TRAJ31	AC244197.2	0.506282915	3.59E-36	postive
TRAJ37	AC244197.2	0.503077065	1.15E-35	postive
TRAJ38	AC244197.2	0.504335143	7.30E-36	postive
CREB1	AC073651.1	0.761013485	3.08E-102	postive
UBR1	AC073651.1	0.655226223	6.30E-67	postive
ZC3HAV1	AC073651.1	0.52878164	7.16E-40	postive
ZC3HAV1L	AC073651.1	0.504463948	6.97E-36	postive
IL15	AC073651.1	0.611247452	4.05E-56	postive
CYLD	AC073651.1	0.565414579	1.59E-46	postive
EIF2AK2	AC073651.1	0.522518827	8.18E-39	postive
MAPK8	AC073651.1	0.566056937	1.19E-46	postive
LMBR1	AC073651.1	0.568183982	4.60E-47	postive
IREB2	AC073651.1	0.626864671	9.28E-60	postive
DDX17	AC073651.1	0.565753763	1.36E-46	postive
PIK3CG	AC073651.1	0.508695398	1.49E-36	postive
JAK2	AC073651.1	0.607276627	3.17E-55	postive
TXK	AC073651.1	0.605259837	8.91E-55	postive
NFAT5	AC073651.1	0.783203482	4.44E-112	postive
MALT1	AC073651.1	0.520440386	1.82E-38	postive
PIK3R1	AC073651.1	0.659440788	4.65E-68	postive
PIK3CA	AC073651.1	0.657818684	1.27E-67	postive
PIK3CB	AC073651.1	0.524320046	4.08E-39	postive
RASGRP3	AC073651.1	0.510422718	7.86E-37	postive
IGHD4-4	AC073651.1	0.537061206	2.64E-41	postive
GNRH1	AC073651.1	0.706888198	3.43E-82	postive
IL6ST	AC073651.1	0.632895193	3.20E-61	postive
RABEP1	AC073651.1	0.542393165	3.00E-42	postive
ACVR2A	AC073651.1	0.629168175	2.59E-60	postive
ANGPTL1	AC073651.1	0.751440087	2.56E-98	postive
BMPR1A	AC073651.1	0.570030381	2.00E-47	postive
BMPR2	AC073651.1	0.739569102	1.07E-93	postive
CRLF3	AC073651.1	0.582072207	7.75E-50	postive
NR1D2	AC073651.1	0.544021478	1.53E-42	postive
NR2C2	AC073651.1	0.662803064	5.64E-69	postive
RORA	AC073651.1	0.829036951	1.18E-136	postive
SOS1	AC073651.1	0.643868519	5.77E-64	postive
SOS2	AC073651.1	0.54912102	1.83E-43	postive
BRAF	AC073651.1	0.783270339	4.13E-112	postive
TEC	AC073651.1	0.502411697	1.46E-35	postive
CBL	AC073651.1	0.545525884	8.22E-43	postive
CBLB	AC073651.1	0.719101621	2.67E-86	postive
RASGRP1	AC073651.1	0.500099605	3.35E-35	postive
PDK1	AC073651.1	0.592373452	5.55E-52	postive
TRAJ1	AC073651.1	0.617372743	1.60E-57	postive
TRAJ2	AC073651.1	0.673114047	7.34E-72	postive
TRAJ3	AC073651.1	0.723065337	1.11E-87	postive
TRAJ5	AC073651.1	0.699856675	6.39E-80	postive
TRAJ6	AC073651.1	0.70376268	3.57E-81	postive
TRAJ8	AC073651.1	0.646615733	1.14E-64	postive
TRAJ10	AC073651.1	0.695063285	2.07E-78	postive
TRAJ12	AC073651.1	0.678026947	2.81E-73	postive
TRAJ13	AC073651.1	0.670532591	3.97E-71	postive
TRAJ14	AC073651.1	0.674897413	2.26E-72	postive
TRAJ16	AC073651.1	0.665063004	1.34E-69	postive
TRAJ17	AC073651.1	0.648801625	3.09E-65	postive
TRAJ18	AC073651.1	0.678755821	1.72E-73	postive
TRAJ21	AC073651.1	0.717372494	1.05E-85	postive
TRAJ31	AC073651.1	0.761339027	2.25E-102	postive
TRAJ37	AC073651.1	0.742268743	1.00E-94	postive
TRAJ38	AC073651.1	0.795921772	2.92E-118	postive
TRAJ39	AC073651.1	0.732358889	5.16E-91	postive
CREB1	AC120349.1	0.646139433	1.51E-64	postive
UBR1	AC120349.1	0.564121858	2.81E-46	postive
IL15	AC120349.1	0.53920138	1.11E-41	postive
IREB2	AC120349.1	0.532852659	1.43E-40	postive

DDX17	AC120349.1	0.564979203	1.92E-46	postive
JAK2	AC120349.1	0.527915207	1.01E-39	postive
TXK	AC120349.1	0.572388124	6.87E-48	postive
NFAT5	AC120349.1	0.686920425	6.51E-76	postive
MALT1	AC120349.1	0.524904679	3.25E-39	postive
PIK3R1	AC120349.1	0.563001	4.62E-46	postive
PIK3CA	AC120349.1	0.590311498	1.51E-51	postive
IGHD4-4	AC120349.1	0.525836833	2.27E-39	postive
IGHD6-6	AC120349.1	0.553081498	3.41E-44	postive
GNRH1	AC120349.1	0.736845933	1.13E-92	postive
IL6ST	AC120349.1	0.537977654	1.82E-41	postive
ACVR2A	AC120349.1	0.57804582	5.09E-49	postive
ANGPTL1	AC120349.1	0.670342717	4.49E-71	postive
BMPR2	AC120349.1	0.626047992	1.46E-59	postive
CRLF3	AC120349.1	0.515553314	1.16E-37	postive
NR2C2	AC120349.1	0.662158166	8.47E-69	postive
RORA	AC120349.1	0.724555687	3.32E-88	postive
SOS1	AC120349.1	0.55988788	1.81E-45	postive
BRAF	AC120349.1	0.687561695	4.16E-76	postive
TEC	AC120349.1	0.540986587	5.35E-42	postive
CBLB	AC120349.1	0.595212674	1.38E-52	postive
PDK1	AC120349.1	0.563870751	3.14E-46	postive
TRAJ1	AC120349.1	0.602084473	4.47E-54	postive
TRAJ2	AC120349.1	0.569252706	2.84E-47	postive
TRAJ3	AC120349.1	0.667932783	2.14E-70	postive
TRAJ5	AC120349.1	0.670531204	3.97E-71	postive
TRAJ6	AC120349.1	0.642196898	1.54E-63	postive
TRAJ8	AC120349.1	0.616778894	2.20E-57	postive
TRAJ10	AC120349.1	0.637823805	1.94E-62	postive
TRAJ12	AC120349.1	0.599011098	2.09E-53	postive
TRAJ13	AC120349.1	0.605210078	9.14E-55	postive
TRAJ14	AC120349.1	0.612408477	2.21E-56	postive
TRAJ16	AC120349.1	0.644651671	3.64E-64	postive
TRAJ17	AC120349.1	0.644956319	3.04E-64	postive
TRAJ18	AC120349.1	0.611302963	3.93E-56	postive
TRAJ21	AC120349.1	0.664695643	1.70E-69	postive
TRAJ31	AC120349.1	0.688173996	2.72E-76	postive
TRAJ37	AC120349.1	0.654117307	1.24E-66	postive
TRAJ38	AC120349.1	0.703664013	3.84E-81	postive
TRAJ39	AC120349.1	0.715518383	4.52E-85	postive
KCNH2	AC004540.2	0.518840281	3.34E-38	postive
PPIA	SNHG15	0.576637705	9.78E-49	postive
CREB1	LINC02352	0.620226048	3.47E-58	postive
RFXAP	LINC02352	0.50624369	3.65E-36	postive
UBR1	LINC02352	0.594669027	1.80E-52	postive
IREB2	LINC02352	0.538306875	1.59E-41	postive
DDX17	LINC02352	0.6452689	2.53E-64	postive
TXK	LINC02352	0.546826098	4.78E-43	postive
NFAT5	LINC02352	0.666458666	5.51E-70	postive
PIK3R1	LINC02352	0.500499393	2.91E-35	postive
IGHD4-4	LINC02352	0.537321584	2.38E-41	postive
IGHD6-6	LINC02352	0.541187328	4.92E-42	postive
GNRH1	LINC02352	0.793594849	4.25E-117	postive
ACVR2A	LINC02352	0.607703896	2.54E-55	postive
ANGPTL1	LINC02352	0.58126172	1.13E-49	postive
BMPR2	LINC02352	0.607752414	2.48E-55	postive
CRLF3	LINC02352	0.523500627	5.60E-39	postive
NR2C2	LINC02352	0.706877326	3.46E-82	postive
RORA	LINC02352	0.649942757	1.56E-65	postive
SOS1	LINC02352	0.577502238	6.55E-49	postive
BRAF	LINC02352	0.688341326	2.42E-76	postive
CBLB	LINC02352	0.530679212	3.39E-40	postive
TRAJ1	LINC02352	0.637391971	2.48E-62	postive
TRAJ2	LINC02352	0.583625155	3.72E-50	postive
TRAJ3	LINC02352	0.659935822	3.41E-68	postive

TRAJ5	LINC02352	0.62359289	5.59E-59	postive
TRAJ6	LINC02352	0.635188886	8.74E-62	postive
TRAJ8	LINC02352	0.677860036	3.14E-73	postive
TRAJ10	LINC02352	0.565352047	1.63E-46	postive
TRAJ12	LINC02352	0.573331324	4.47E-48	postive
TRAJ13	LINC02352	0.633069721	2.90E-61	postive
TRAJ14	LINC02352	0.614722273	6.53E-57	postive
TRAJ16	LINC02352	0.620186299	3.54E-58	postive
TRAJ17	LINC02352	0.599876631	1.36E-53	postive
TRAJ18	LINC02352	0.503694073	9.21E-36	postive
TRAJ21	LINC02352	0.635011985	9.66E-62	postive
TRAJ31	LINC02352	0.646916186	9.52E-65	postive
TRAJ37	LINC02352	0.624227348	3.95E-59	postive
TRAJ38	LINC02352	0.661006947	1.75E-68	postive
TRAJ39	LINC02352	0.669524332	7.64E-71	postive
GNRH1	LINC00115	0.517912481	4.76E-38	postive
CREB1	AL513550.1	0.70318505	5.49E-81	postive
RFXAP	AL513550.1	0.570903681	1.35E-47	postive
UBR1	AL513550.1	0.601253731	6.79E-54	postive
CYLD	AL513550.1	0.542713075	2.63E-42	postive
EIF2AK2	AL513550.1	0.527806593	1.05E-39	postive
MAPK8	AL513550.1	0.508192597	1.79E-36	postive
IREB2	AL513550.1	0.611892179	2.89E-56	postive
DDX17	AL513550.1	0.575127009	1.96E-48	postive
PIK3CG	AL513550.1	0.528666866	7.49E-40	postive
JAK2	AL513550.1	0.565658423	1.42E-46	postive
TXK	AL513550.1	0.527626831	1.13E-39	postive
NFAT5	AL513550.1	0.699953776	5.95E-80	postive
PIK3R1	AL513550.1	0.555847052	1.04E-44	postive
PIK3CA	AL513550.1	0.607146337	3.39E-55	postive
GNRH1	AL513550.1	0.6434684	7.30E-64	postive
IL6ST	AL513550.1	0.50971512	1.02E-36	postive
ACVR2A	AL513550.1	0.607239877	3.23E-55	postive
ANGPTL1	AL513550.1	0.650320935	1.24E-65	postive
BMPR1A	AL513550.1	0.516928476	6.90E-38	postive
BMPR2	AL513550.1	0.627250516	7.50E-60	postive
CRLF3	AL513550.1	0.617943909	1.18E-57	postive
NR2C2	AL513550.1	0.620099113	3.71E-58	postive
RORA	AL513550.1	0.709297802	5.52E-83	postive
SOS1	AL513550.1	0.599375093	1.74E-53	postive
SOS2	AL513550.1	0.522616687	7.88E-39	postive
BRAF	AL513550.1	0.693011907	8.96E-78	postive
CBL	AL513550.1	0.502855165	1.25E-35	postive
CBLB	AL513550.1	0.583469948	4.00E-50	postive
PDK1	AL513550.1	0.596714136	6.56E-53	postive
TRAJ1	AL513550.1	0.623034696	7.57E-59	postive
TRAJ2	AL513550.1	0.642933448	9.99E-64	postive
TRAJ3	AL513550.1	0.662293605	7.77E-69	postive
TRAJ5	AL513550.1	0.656342621	3.17E-67	postive
TRAJ6	AL513550.1	0.654206316	1.18E-66	postive
TRAJ8	AL513550.1	0.637643422	2.15E-62	postive
TRAJ10	AL513550.1	0.628402656	3.96E-60	postive
TRAJ12	AL513550.1	0.593078288	3.93E-52	postive
TRAJ13	AL513550.1	0.638794771	1.11E-62	postive
TRAJ14	AL513550.1	0.63284936	3.29E-61	postive
TRAJ16	AL513550.1	0.646068208	1.58E-64	postive
TRAJ17	AL513550.1	0.619866459	4.21E-58	postive
TRAJ18	AL513550.1	0.608115159	2.06E-55	postive
TRAJ21	AL513550.1	0.66782982	2.28E-70	postive
TRAJ31	AL513550.1	0.660822842	1.96E-68	postive
TRAJ37	AL513550.1	0.653515421	1.79E-66	postive
TRAJ38	AL513550.1	0.713602787	2.01E-84	postive
TRAJ39	AL513550.1	0.694908076	2.31E-78	postive
NFKBIZ	AC008760.1	0.500334875	3.08E-35	postive
GNRH1	AC008760.1	0.543116572	2.23E-42	postive

GIPR	AC008760.1	0.500089822	3.36E-35	postive
B2M	LINC01871	0.53967861	9.12E-42	postive
CD8A	LINC01871	0.748039761	5.74E-97	postive
CD8B	LINC01871	0.638323685	1.45E-62	postive
IFNG	LINC01871	0.750644072	5.33E-98	postive
PSMB8	LINC01871	0.528599206	7.69E-40	postive
PSME2	LINC01871	0.527989749	9.77E-40	postive
TAP1	LINC01871	0.587662641	5.43E-51	postive
TAP2	LINC01871	0.568054123	4.87E-47	postive
CXCL10	LINC01871	0.559291138	2.35E-45	postive
CXCL9	LINC01871	0.58615133	1.12E-50	postive
APOBEC3G	LINC01871	0.576517041	1.03E-48	postive
STAT1	LINC01871	0.550220493	1.15E-43	postive
IRF1	LINC01871	0.666008669	7.35E-70	postive
CCL5	LINC01871	0.752692251	8.06E-99	postive
CCL4	LINC01871	0.768748853	1.53E-105	postive
PDCD1	LINC01871	0.61746267	1.53E-57	postive
FASLG	LINC01871	0.723692863	6.69E-88	postive
GBP2	LINC01871	0.541077093	5.15E-42	postive
CCR5	LINC01871	0.552525214	4.32E-44	postive
CCL4L2	LINC01871	0.608114764	2.06E-55	postive
XCL2	LINC01871	0.650764839	9.50E-66	postive
CXCR6	LINC01871	0.763842714	1.97E-103	postive
CD72	LINC01871	0.605610571	7.45E-55	postive
TNFSF13B	LINC01871	0.578098155	4.97E-49	postive
IL12RB1	LINC01871	0.581386596	1.07E-49	postive
IL15RA	LINC01871	0.613645055	1.15E-56	postive
IL2RB	LINC01871	0.634571463	1.24E-61	postive
IL18RAP	LINC01871	0.676126126	1.00E-72	postive
IL2RG	LINC01871	0.501247497	2.22E-35	postive
LCK	LINC01871	0.574238861	2.95E-48	postive
CD247	LINC01871	0.733290149	2.35E-91	postive
ZAP70	LINC01871	0.524775197	3.42E-39	postive
LCP2	LINC01871	0.505262936	5.21E-36	postive
HCST	LINC01871	0.533447668	1.13E-40	postive
CD244	LINC01871	0.681293234	3.10E-74	postive
SH2D1A	LINC01871	0.625528454	1.94E-59	postive
GZMB	LINC01871	0.803211996	5.25E-122	postive
PRF1	LINC01871	0.60628903	5.26E-55	postive
CD3D	LINC01871	0.727129897	4.03E-89	postive
CD3E	LINC01871	0.655022281	7.14E-67	postive
CD3G	LINC01871	0.607206747	3.28E-55	postive
ICOS	LINC01871	0.503949989	8.39E-36	postive
CTLA4	LINC01871	0.501069933	2.37E-35	postive
TRAC	LINC01871	0.643436681	7.44E-64	postive
TRAV4	LINC01871	0.529646627	5.09E-40	postive
TRAV8-2	LINC01871	0.505190643	5.35E-36	postive
TRAV12-2	LINC01871	0.52150401	1.21E-38	postive
TRAV13-2	LINC01871	0.533935943	9.27E-41	postive
TRAV16	LINC01871	0.545711529	7.61E-43	postive
TRAV19	LINC01871	0.539160498	1.13E-41	postive
TRAV21	LINC01871	0.527558828	1.16E-39	postive
TRAV29DV5	LINC01871	0.553634727	2.69E-44	postive
TRBC1	LINC01871	0.587303147	6.45E-51	postive
TRBC2	LINC01871	0.692099579	1.71E-77	postive
TRBJ2-3	LINC01871	0.520210093	1.98E-38	postive
TRBV3-1	LINC01871	0.514562609	1.68E-37	postive
TRBV5-1	LINC01871	0.50779222	2.07E-36	postive
TRBV6-5	LINC01871	0.514179159	1.94E-37	postive
TRBV7-9	LINC01871	0.633088637	2.87E-61	postive
TRBV9	LINC01871	0.514681004	1.61E-37	postive
TRBV18	LINC01871	0.50956509	1.08E-36	postive
TRBV28	LINC01871	0.645021013	2.93E-64	postive
TRDV1	LINC01871	0.54358182	1.84E-42	postive
RARA	RARA-AS1	0.509187562	1.24E-36	postive

CREB1	AL021707.8	0.633477146	2.31E-61	postive
CIITA	AL021707.8	0.503791786	8.89E-36	postive
RFXAP	AL021707.8	0.503744009	9.04E-36	postive
UBR1	AL021707.8	0.504222822	7.60E-36	postive
IL15	AL021707.8	0.54599847	6.75E-43	postive
CYLD	AL021707.8	0.590914137	1.13E-51	postive
NFKBIZ	AL021707.8	0.577803186	5.70E-49	postive
DDX17	AL021707.8	0.788267991	1.72E-114	postive
JAK2	AL021707.8	0.560315959	1.50E-45	postive
TXK	AL021707.8	0.535860952	4.28E-41	postive
NFAT5	AL021707.8	0.72687815	4.96E-89	postive
PIK3R1	AL021707.8	0.520184476	2.00E-38	postive
PIK3CA	AL021707.8	0.549204012	1.76E-43	postive
RASGRP3	AL021707.8	0.523248043	6.18E-39	postive
LTB4R2	AL021707.8	0.568826911	3.44E-47	postive
GNRH1	AL021707.8	0.784648143	9.25E-113	postive
ACVR2A	AL021707.8	0.546392219	5.73E-43	postive
ANGPTL1	AL021707.8	0.619965293	3.99E-58	postive
BMPR2	AL021707.8	0.568704202	3.64E-47	postive
CRLF3	AL021707.8	0.531842607	2.14E-40	postive
NR2C1	AL021707.8	0.546762895	4.91E-43	postive
NR2C2	AL021707.8	0.71260337	4.36E-84	postive
RORA	AL021707.8	0.713752205	1.79E-84	postive
SOS1	AL021707.8	0.57645899	1.06E-48	postive
SOS2	AL021707.8	0.504449574	7.00E-36	postive
BRAF	AL021707.8	0.674485608	2.97E-72	postive
ITK	AL021707.8	0.531402827	2.54E-40	postive
CBL	AL021707.8	0.53575065	4.48E-41	postive
CBLB	AL021707.8	0.620430871	3.10E-58	postive
TRAJ1	AL021707.8	0.691124747	3.42E-77	postive
TRAJ2	AL021707.8	0.652980836	2.48E-66	postive
TRAJ3	AL021707.8	0.720607517	8.04E-87	postive
TRAJ5	AL021707.8	0.686401906	9.32E-76	postive
TRAJ6	AL021707.8	0.686684872	7.66E-76	postive
TRAJ8	AL021707.8	0.659828289	3.65E-68	postive
TRAJ10	AL021707.8	0.689491379	1.08E-76	postive
TRAJ12	AL021707.8	0.638682496	1.18E-62	postive
TRAJ13	AL021707.8	0.668877031	1.16E-70	postive
TRAJ14	AL021707.8	0.657474537	1.58E-67	postive
TRAJ16	AL021707.8	0.701908905	1.41E-80	postive
TRAJ17	AL021707.8	0.686354796	9.63E-76	postive
TRAJ18	AL021707.8	0.666143308	6.74E-70	postive
TRAJ21	AL021707.8	0.693309643	7.25E-78	postive
TRAJ31	AL021707.8	0.714037679	1.43E-84	postive
TRAJ37	AL021707.8	0.695290723	1.76E-78	postive
TRAJ38	AL021707.8	0.727501252	2.97E-89	postive
TRAJ39	AL021707.8	0.708368493	1.12E-82	postive
TGFB2	TGFB2-AS1	0.896169093	3.10E-190	postive
CREB1	AC074032.1	0.701955522	1.36E-80	postive
RFXAP	AC074032.1	0.518442317	3.89E-38	postive
UBR1	AC074032.1	0.561745745	8.02E-46	postive
ZC3HAV1	AC074032.1	0.507493396	2.31E-36	postive
IL15	AC074032.1	0.6034973	2.19E-54	postive
CYLD	AC074032.1	0.561467149	9.07E-46	postive
MAPK8	AC074032.1	0.511162409	5.97E-37	postive
IREB2	AC074032.1	0.543218523	2.14E-42	postive
DDX17	AC074032.1	0.56195534	7.32E-46	postive
JAK2	AC074032.1	0.591063576	1.05E-51	postive
TXK	AC074032.1	0.521179034	1.37E-38	postive
NFAT5	AC074032.1	0.793783111	3.43E-117	postive
PIK3R1	AC074032.1	0.559530651	2.11E-45	postive
PIK3CA	AC074032.1	0.628879217	3.04E-60	postive
GNRH1	AC074032.1	0.703529996	4.24E-81	postive
IL6ST	AC074032.1	0.555920692	1.01E-44	postive
RABEP1	AC074032.1	0.507312445	2.47E-36	postive

ACVR2A	AC074032.1	0.623090276	7.35E-59	postive
ANGPTL1	AC074032.1	0.749283098	1.85E-97	postive
BMPR1A	AC074032.1	0.50710073	2.67E-36	postive
BMPR2	AC074032.1	0.631727425	6.19E-61	postive
CRLF3	AC074032.1	0.591715422	7.65E-52	postive
NR2C2	AC074032.1	0.665670128	9.13E-70	postive
RORA	AC074032.1	0.755062431	8.85E-100	postive
SOS1	AC074032.1	0.631468936	7.15E-61	postive
SOS2	AC074032.1	0.56339863	3.87E-46	postive
BRAF	AC074032.1	0.731174206	1.40E-90	postive
CBL	AC074032.1	0.553033992	3.48E-44	postive
CBLB	AC074032.1	0.655837785	4.33E-67	postive
PDK1	AC074032.1	0.531998875	2.01E-40	postive
TRAJ1	AC074032.1	0.779837988	1.63E-110	postive
TRAJ2	AC074032.1	0.715618102	4.18E-85	postive
TRAJ3	AC074032.1	0.811446912	1.98E-126	postive
TRAJ5	AC074032.1	0.816198006	4.39E-129	postive
TRAJ6	AC074032.1	0.828143279	4.14E-136	postive
TRAJ8	AC074032.1	0.811984005	9.99E-127	postive
TRAJ10	AC074032.1	0.819889478	3.37E-131	postive
TRAJ12	AC074032.1	0.750408914	6.62E-98	postive
TRAJ13	AC074032.1	0.822098756	1.73E-132	postive
TRAJ14	AC074032.1	0.818418917	2.38E-130	postive
TRAJ16	AC074032.1	0.820936903	8.29E-132	postive
TRAJ17	AC074032.1	0.812325818	6.46E-127	postive
TRAJ18	AC074032.1	0.753655005	3.30E-99	postive
TRAJ21	AC074032.1	0.839264205	3.72E-143	postive
TRAJ31	AC074032.1	0.852172368	4.84E-152	postive
TRAJ37	AC074032.1	0.809322456	2.87E-125	postive
TRAJ38	AC074032.1	0.859775853	1.10E-157	postive
TRAJ39	AC074032.1	0.848049205	4.10E-149	postive
CREB1	LINC00852	0.684277388	4.04E-75	postive
RFXAP	LINC00852	0.526828291	1.54E-39	postive
UBR1	LINC00852	0.563559576	3.61E-46	postive
ZC3HAV1	LINC00852	0.57180233	8.97E-48	postive
IL15	LINC00852	0.576408474	1.09E-48	postive
CYLD	LINC00852	0.596589133	6.98E-53	postive
IREB2	LINC00852	0.522994572	6.81E-39	postive
DDX17	LINC00852	0.659099123	5.75E-68	postive
JAK2	LINC00852	0.595062039	1.48E-52	postive
TXK	LINC00852	0.580103601	1.95E-49	postive
NFAT5	LINC00852	0.772686361	2.82E-107	postive
PIK3R1	LINC00852	0.61230388	2.33E-56	postive
PIK3CA	LINC00852	0.578783755	3.61E-49	postive
RASGRP3	LINC00852	0.563396673	3.88E-46	postive
IGHD4-4	LINC00852	0.526850154	1.53E-39	postive
IGHD6-6	LINC00852	0.51296669	3.05E-37	postive
LTB4R2	LINC00852	0.55834569	3.54E-45	postive
PLXNC1	LINC00852	0.509636922	1.05E-36	postive
GNRH1	LINC00852	0.764552324	9.83E-104	postive
IL6ST	LINC00852	0.571233609	1.16E-47	postive
RABEP1	LINC00852	0.536467144	3.36E-41	postive
ACVR2A	LINC00852	0.600862977	8.27E-54	postive
ANGPTL1	LINC00852	0.6891476	1.37E-76	postive
BMPR2	LINC00852	0.62371991	5.21E-59	postive
CRLF3	LINC00852	0.589837226	1.90E-51	postive
NR2C2	LINC00852	0.7749979	2.62E-108	postive
RORA	LINC00852	0.774026101	7.14E-108	postive
SOS1	LINC00852	0.618447622	9.01E-58	postive
SOS2	LINC00852	0.552495944	4.38E-44	postive
BRAF	LINC00852	0.747892216	6.56E-97	postive
ITK	LINC00852	0.533505626	1.10E-40	postive
TEC	LINC00852	0.501768046	1.84E-35	postive
CD28	LINC00852	0.507569358	2.25E-36	postive
CBL	LINC00852	0.599826232	1.39E-53	postive

CBLB	LINC00852	0.65064403	1.02E-65	postive
RASGRP1	LINC00852	0.530753002	3.29E-40	postive
TRAJ1	LINC00852	0.746796981	1.76E-96	postive
TRAJ2	LINC00852	0.680146189	6.75E-74	postive
TRAJ3	LINC00852	0.76775578	4.12E-105	postive
TRAJ5	LINC00852	0.791366027	5.36E-116	postive
TRAJ6	LINC00852	0.749333356	1.77E-97	postive
TRAJ8	LINC00852	0.730564387	2.33E-90	postive
TRAJ10	LINC00852	0.736136274	2.08E-92	postive
TRAJ12	LINC00852	0.690782358	4.35E-77	postive
TRAJ13	LINC00852	0.74241153	8.82E-95	postive
TRAJ14	LINC00852	0.735733138	2.93E-92	postive
TRAJ16	LINC00852	0.738200978	3.50E-93	postive
TRAJ17	LINC00852	0.730268642	2.98E-90	postive
TRAJ18	LINC00852	0.701783813	1.55E-80	postive
TRAJ21	LINC00852	0.765576084	3.59E-104	postive
TRAJ31	LINC00852	0.779225521	3.13E-110	postive
TRAJ37	LINC00852	0.763236165	3.56E-103	postive
TRAJ38	LINC00852	0.782578134	8.71E-112	postive
TRAJ39	LINC00852	0.798345161	1.73E-119	postive
PIK3R1	AC104083.1	0.621604836	1.65E-58	postive
IL6ST	AC104083.1	0.546842659	4.75E-43	postive
BMPR2	AC104083.1	0.553307343	3.10E-44	postive
RORA	AC104083.1	0.524030322	4.57E-39	postive
DDX17	AC008764.2	0.586563741	9.19E-51	postive
IL11RA	AC008764.2	0.503958302	8.37E-36	postive
NR2C2	AC008764.2	0.612001775	2.73E-56	postive
GNRH1	AC145423.2	0.549054943	1.88E-43	postive
UBR1	TAPT1-AS1	0.510917588	6.54E-37	postive
DDX17	TAPT1-AS1	0.551741342	6.03E-44	postive
NFAT5	TAPT1-AS1	0.551414402	6.93E-44	postive
GNRH1	TAPT1-AS1	0.558049284	4.03E-45	postive
NR2C2	TAPT1-AS1	0.615000892	5.64E-57	postive
BRAF	TAPT1-AS1	0.619925047	4.08E-58	postive
TRAJ2	TAPT1-AS1	0.54503486	1.01E-42	postive
TRAJ3	TAPT1-AS1	0.561452174	9.12E-46	postive
TRAJ6	TAPT1-AS1	0.505876358	4.17E-36	postive
TRAJ8	TAPT1-AS1	0.531457694	2.49E-40	postive
TRAJ14	TAPT1-AS1	0.52867462	7.47E-40	postive
TRAJ21	TAPT1-AS1	0.521756892	1.10E-38	postive
TRAJ37	TAPT1-AS1	0.512049951	4.30E-37	postive
TRAJ38	TAPT1-AS1	0.550410923	1.06E-43	postive
TRAJ39	TAPT1-AS1	0.537594372	2.13E-41	postive
CREB1	AC012467.1	0.687761871	3.62E-76	postive
RFXAP	AC012467.1	0.502094756	1.64E-35	postive
UBR1	AC012467.1	0.642508357	1.28E-63	postive
ZC3HAV1	AC012467.1	0.510586087	7.40E-37	postive
EIF2AK2	AC012467.1	0.520701382	1.64E-38	postive
MAPK8	AC012467.1	0.540592571	6.28E-42	postive
IREB2	AC012467.1	0.589856744	1.89E-51	postive
DDX17	AC012467.1	0.641034477	3.03E-63	postive
TXK	AC012467.1	0.596651801	6.77E-53	postive
NFAT5	AC012467.1	0.713087666	3.00E-84	postive
PIK3R1	AC012467.1	0.58011747	1.94E-49	postive
PIK3CA	AC012467.1	0.564787618	2.09E-46	postive
IGHD6-6	AC012467.1	0.538438455	1.51E-41	postive
ROBO2	AC012467.1	0.556632286	7.43E-45	postive
GNRH1	AC012467.1	0.771525828	9.23E-107	postive
IL6ST	AC012467.1	0.591565064	8.23E-52	postive
RABEP1	AC012467.1	0.530012234	4.41E-40	postive
ACVR2A	AC012467.1	0.633493146	2.29E-61	postive
ANGPTL1	AC012467.1	0.5889038	2.99E-51	postive
BMPR1A	AC012467.1	0.549645258	1.46E-43	postive
BMPR2	AC012467.1	0.692035082	1.79E-77	postive
CRLF3	AC012467.1	0.541677189	4.03E-42	postive

NR1D2	AC012467.1	0.500106982	3.34E-35	postive
NR2C1	AC012467.1	0.516694766	7.54E-38	postive
NR2C2	AC012467.1	0.733701925	1.66E-91	postive
NR3C2	AC012467.1	0.515547744	1.16E-37	postive
RORA	AC012467.1	0.738707554	2.26E-93	postive
SOS1	AC012467.1	0.628939091	2.94E-60	postive
SOS2	AC012467.1	0.501646308	1.93E-35	postive
BRAF	AC012467.1	0.736445136	1.59E-92	postive
TEC	AC012467.1	0.551641502	6.29E-44	postive
CBLB	AC012467.1	0.606846443	3.95E-55	postive
TRAJ1	AC012467.1	0.591065926	1.05E-51	postive
TRAJ2	AC012467.1	0.553636682	2.69E-44	postive
TRAJ3	AC012467.1	0.614758656	6.41E-57	postive
TRAJ5	AC012467.1	0.631096315	8.81E-61	postive
TRAJ6	AC012467.1	0.610521836	5.91E-56	postive
TRAJ8	AC012467.1	0.617533368	1.47E-57	postive
TRAJ10	AC012467.1	0.553820959	2.49E-44	postive
TRAJ12	AC012467.1	0.582344464	6.81E-50	postive
TRAJ13	AC012467.1	0.592848293	4.40E-52	postive
TRAJ14	AC012467.1	0.577357977	7.01E-49	postive
TRAJ16	AC012467.1	0.610601791	5.67E-56	postive
TRAJ17	AC012467.1	0.570635015	1.52E-47	postive
TRAJ18	AC012467.1	0.541406636	4.50E-42	postive
TRAJ21	AC012467.1	0.62013263	3.65E-58	postive
TRAJ31	AC012467.1	0.650123192	1.40E-65	postive
TRAJ37	AC012467.1	0.634375005	1.39E-61	postive
TRAJ38	AC012467.1	0.66633889	5.95E-70	postive
TRAJ39	AC012467.1	0.674630353	2.70E-72	postive
MAVS	AL160006.1	0.511829514	4.66E-37	postive
CREB1	AC093388.1	0.711149632	1.34E-83	postive
UBR1	AC093388.1	0.588923915	2.96E-51	postive
ZC3HAV1	AC093388.1	0.540898433	5.54E-42	postive
IL15	AC093388.1	0.509513882	1.10E-36	postive
CYLD	AC093388.1	0.565290726	1.67E-46	postive
EIF2AK2	AC093388.1	0.568229232	4.51E-47	postive
MAPK8	AC093388.1	0.502118889	1.63E-35	postive
LMBR1	AC093388.1	0.508231864	1.76E-36	postive
LIMS1	AC093388.1	0.515512644	1.18E-37	postive
IREB2	AC093388.1	0.589089267	2.73E-51	postive
DDX17	AC093388.1	0.531430251	2.52E-40	postive
JAK2	AC093388.1	0.565437149	1.57E-46	postive
TXK	AC093388.1	0.549038545	1.89E-43	postive
NFAT5	AC093388.1	0.755803993	4.41E-100	postive
PIK3R1	AC093388.1	0.565192557	1.75E-46	postive
PIK3CA	AC093388.1	0.670173387	5.02E-71	postive
PIK3CB	AC093388.1	0.500700042	2.70E-35	postive
AKT3	AC093388.1	0.502580488	1.38E-35	postive
ROBO2	AC093388.1	0.508749691	1.46E-36	postive
GNRH1	AC093388.1	0.679480181	1.06E-73	postive
IL6ST	AC093388.1	0.513870649	2.18E-37	postive
RABEP1	AC093388.1	0.507232223	2.54E-36	postive
ACVR2A	AC093388.1	0.610150044	7.17E-56	postive
ANGPTL1	AC093388.1	0.676992891	5.62E-73	postive
BMPR1A	AC093388.1	0.534998711	6.06E-41	postive
BMPR2	AC093388.1	0.730243213	3.05E-90	postive
CRLF3	AC093388.1	0.591487626	8.54E-52	postive
NR1D2	AC093388.1	0.50292372	1.22E-35	postive
NR2C2	AC093388.1	0.607351703	3.05E-55	postive
RORA	AC093388.1	0.717937381	6.73E-86	postive
SOS1	AC093388.1	0.661638985	1.17E-68	postive
SOS2	AC093388.1	0.516939394	6.87E-38	postive
BRAF	AC093388.1	0.709957861	3.33E-83	postive
CBL	AC093388.1	0.539916621	8.28E-42	postive
CBLB	AC093388.1	0.641561672	2.23E-63	postive
PDK1	AC093388.1	0.501131318	2.32E-35	postive

TRAJ1	AC093388.1	0.680851328	4.19E-74	postive
TRAJ2	AC093388.1	0.663546569	3.52E-69	postive
TRAJ3	AC093388.1	0.720751165	7.17E-87	postive
TRAJ5	AC093388.1	0.704918962	1.51E-81	postive
TRAJ6	AC093388.1	0.731028727	1.58E-90	postive
TRAJ8	AC093388.1	0.732224997	5.78E-91	postive
TRAJ10	AC093388.1	0.684407303	3.70E-75	postive
TRAJ12	AC093388.1	0.710077719	3.04E-83	postive
TRAJ13	AC093388.1	0.720506317	8.72E-87	postive
TRAJ14	AC093388.1	0.715205666	5.77E-85	postive
TRAJ16	AC093388.1	0.745494901	5.68E-96	postive
TRAJ17	AC093388.1	0.707707081	1.85E-82	postive
TRAJ18	AC093388.1	0.677671391	3.57E-73	postive
TRAJ21	AC093388.1	0.741042058	2.95E-94	postive
TRAJ31	AC093388.1	0.767319656	6.36E-105	postive
TRAJ37	AC093388.1	0.719645708	1.73E-86	postive
TRAJ38	AC093388.1	0.785076596	5.80E-113	postive
TRAJ39	AC093388.1	0.748700356	3.15E-97	postive
RABEP2	ZNF213-AS1	0.523769476	5.05E-39	postive
CREB1	AC004884.2	0.714439907	1.05E-84	postive
UBR1	AC004884.2	0.585780765	1.34E-50	postive
ZC3HAV1	AC004884.2	0.534619025	7.05E-41	postive
ZC3HAV1L	AC004884.2	0.541707363	3.98E-42	postive
IL15	AC004884.2	0.541023265	5.27E-42	postive
MAPK8	AC004884.2	0.53872496	1.35E-41	postive
LMBR1	AC004884.2	0.575219216	1.88E-48	postive
IREB2	AC004884.2	0.582999576	5.00E-50	postive
DDX17	AC004884.2	0.506838397	2.93E-36	postive
JAK2	AC004884.2	0.536313392	3.57E-41	postive
NFAT5	AC004884.2	0.74059339	4.36E-94	postive
PIK3R1	AC004884.2	0.562300699	6.28E-46	postive
PIK3CA	AC004884.2	0.607169872	3.35E-55	postive
AKT3	AC004884.2	0.638097743	1.65E-62	postive
IGHD4-4	AC004884.2	0.530845993	3.17E-40	postive
GMFB	AC004884.2	0.523090648	6.56E-39	postive
GNRH1	AC004884.2	0.667590136	2.67E-70	postive
IL6ST	AC004884.2	0.562616967	5.47E-46	postive
RABEP1	AC004884.2	0.531093745	2.87E-40	postive
ACVR2A	AC004884.2	0.576080968	1.26E-48	postive
ANGPTL1	AC004884.2	0.742833642	6.08E-95	postive
BMPR1A	AC004884.2	0.52374922	5.09E-39	postive
BMPR2	AC004884.2	0.65025992	1.29E-65	postive
CRLF3	AC004884.2	0.659205795	5.38E-68	postive
NR2C2	AC004884.2	0.600551888	9.67E-54	postive
RORA	AC004884.2	0.7030731	5.96E-81	postive
SOS1	AC004884.2	0.636481183	4.18E-62	postive
SOS2	AC004884.2	0.547539533	3.55E-43	postive
BRAF	AC004884.2	0.796328229	1.82E-118	postive
CBL	AC004884.2	0.588224237	4.14E-51	postive
CBLB	AC004884.2	0.628973291	2.88E-60	postive
PDK1	AC004884.2	0.555397801	1.27E-44	postive
TRAJ1	AC004884.2	0.690761476	4.42E-77	postive
TRAJ2	AC004884.2	0.720689784	7.53E-87	postive
TRAJ3	AC004884.2	0.767213496	7.07E-105	postive
TRAJ5	AC004884.2	0.744145823	1.90E-95	postive
TRAJ6	AC004884.2	0.75153363	2.35E-98	postive
TRAJ8	AC004884.2	0.81001099	1.21E-125	postive
TRAJ10	AC004884.2	0.705945489	6.97E-82	postive
TRAJ12	AC004884.2	0.60829893	1.87E-55	postive
TRAJ13	AC004884.2	0.796107228	2.36E-118	postive
TRAJ14	AC004884.2	0.765065353	5.93E-104	postive
TRAJ16	AC004884.2	0.724054655	4.99E-88	postive
TRAJ17	AC004884.2	0.713129369	2.90E-84	postive
TRAJ18	AC004884.2	0.592396556	5.49E-52	postive
TRAJ21	AC004884.2	0.78474007	8.37E-113	postive

TRAJ31	AC004884.2	0.773621883	1.08E-107	postive
TRAJ37	AC004884.2	0.752900234	6.65E-99	postive
TRAJ38	AC004884.2	0.792312079	1.84E-116	postive
TRAJ39	AC004884.2	0.801018948	7.30E-121	postive
CXCL17	LINC02489	0.509438315	1.13E-36	postive
LTB4R	SLC9A3-AS1	0.530782625	3.25E-40	postive
NDP	LINC00922	0.789749999	3.30E-115	postive
CREB1	AP001486.2	0.66193375	9.75E-69	postive
RFXAP	AP001486.2	0.524611773	3.65E-39	postive
UBR1	AP001486.2	0.615037841	5.53E-57	postive
ZC3HAV1	AP001486.2	0.502520923	1.41E-35	postive
CYLD	AP001486.2	0.532375144	1.73E-40	postive
MAPK8	AP001486.2	0.519960248	2.18E-38	postive
IREB2	AP001486.2	0.561225789	1.01E-45	postive
DDX17	AP001486.2	0.69433656	3.48E-78	postive
TXK	AP001486.2	0.534383408	7.75E-41	postive
NFAT5	AP001486.2	0.718039192	6.21E-86	postive
NFATC3	AP001486.2	0.504521154	6.82E-36	postive
PIK3R1	AP001486.2	0.601216828	6.92E-54	postive
ROBO2	AP001486.2	0.536600397	3.18E-41	postive
GNRH1	AP001486.2	0.732157541	6.12E-91	postive
IL6ST	AP001486.2	0.625622031	1.84E-59	postive
RABEP1	AP001486.2	0.565633005	1.44E-46	postive
ACVR2A	AP001486.2	0.612162526	2.51E-56	postive
ANGPTL1	AP001486.2	0.598036383	3.40E-53	postive
BMPR1A	AP001486.2	0.552951451	3.61E-44	postive
BMPR2	AP001486.2	0.615754379	3.78E-57	postive
CRLF3	AP001486.2	0.503587931	9.57E-36	postive
NR1D2	AP001486.2	0.518606442	3.65E-38	postive
NR2C1	AP001486.2	0.537333341	2.36E-41	postive
NR2C2	AP001486.2	0.764540524	9.94E-104	postive
NR3C2	AP001486.2	0.544836001	1.09E-42	postive
RORA	AP001486.2	0.729748985	4.60E-90	postive
SOS1	AP001486.2	0.605676114	7.20E-55	postive
BRAF	AP001486.2	0.70874226	8.42E-83	postive
TEC	AP001486.2	0.513895744	2.16E-37	postive
CBLB	AP001486.2	0.577978564	5.25E-49	postive
TRAJ1	AP001486.2	0.589828034	1.91E-51	postive
TRAJ2	AP001486.2	0.550662653	9.53E-44	postive
TRAJ3	AP001486.2	0.604632542	1.23E-54	postive
TRAJ5	AP001486.2	0.634108026	1.61E-61	postive
TRAJ6	AP001486.2	0.618677388	7.97E-58	postive
TRAJ8	AP001486.2	0.581767969	8.94E-50	postive
TRAJ10	AP001486.2	0.579399154	2.71E-49	postive
TRAJ12	AP001486.2	0.60175971	5.27E-54	postive
TRAJ13	AP001486.2	0.597572209	4.29E-53	postive
TRAJ14	AP001486.2	0.596271604	8.17E-53	postive
TRAJ16	AP001486.2	0.599489577	1.65E-53	postive
TRAJ17	AP001486.2	0.561696454	8.20E-46	postive
TRAJ18	AP001486.2	0.576340235	1.12E-48	postive
TRAJ21	AP001486.2	0.623090375	7.35E-59	postive
TRAJ31	AP001486.2	0.637840992	1.92E-62	postive
TRAJ37	AP001486.2	0.63245381	4.11E-61	postive
TRAJ38	AP001486.2	0.663332583	4.03E-69	postive
TRAJ39	AP001486.2	0.644832372	3.27E-64	postive
CREB1	GAS8-AS1	0.562906399	4.81E-46	postive
UBR1	GAS8-AS1	0.53079898	3.23E-40	postive
ZC3HAV1L	GAS8-AS1	0.521313988	1.30E-38	postive
DDX17	GAS8-AS1	0.59906648	2.04E-53	postive
NFAT5	GAS8-AS1	0.628740162	3.28E-60	postive
GNRH1	GAS8-AS1	0.730813246	1.89E-90	postive
ANGPTL1	GAS8-AS1	0.556702648	7.21E-45	postive
CRLF3	GAS8-AS1	0.502097813	1.64E-35	postive
NR2C2	GAS8-AS1	0.664138177	2.42E-69	postive
RORA	GAS8-AS1	0.524464357	3.86E-39	postive

SOS1	GAS8-AS1	0.527946995	9.93E-40	postive
BRAF	GAS8-AS1	0.661008791	1.74E-68	postive
CBL	GAS8-AS1	0.507261865	2.51E-36	postive
CBLB	GAS8-AS1	0.552778772	3.88E-44	postive
TRAJ1	GAS8-AS1	0.558028146	4.06E-45	postive
TRAJ2	GAS8-AS1	0.59249632	5.23E-52	postive
TRAJ3	GAS8-AS1	0.639932109	5.74E-63	postive
TRAJ5	GAS8-AS1	0.503330692	1.05E-35	postive
TRAJ6	GAS8-AS1	0.59782338	3.78E-53	postive
TRAJ8	GAS8-AS1	0.584825611	2.11E-50	postive
TRAJ10	GAS8-AS1	0.530460162	3.69E-40	postive
TRAJ13	GAS8-AS1	0.553135808	3.33E-44	postive
TRAJ14	GAS8-AS1	0.569973544	2.05E-47	postive
TRAJ16	GAS8-AS1	0.534871041	6.38E-41	postive
TRAJ21	GAS8-AS1	0.567643817	5.86E-47	postive
TRAJ31	GAS8-AS1	0.528606249	7.67E-40	postive
TRAJ37	GAS8-AS1	0.543051606	2.29E-42	postive
TRAJ38	GAS8-AS1	0.596024434	9.23E-53	postive
TRAJ39	GAS8-AS1	0.580243924	1.83E-49	postive
DDX17	AL021707.2	0.590799432	1.19E-51	postive
GRAP2	AL021707.2	0.517844081	4.88E-38	postive
CREB1	ABALON	0.635418799	7.66E-62	postive
UBR1	ABALON	0.591726529	7.61E-52	postive
CYLD	ABALON	0.518603462	3.66E-38	postive
LMBR1	ABALON	0.558108014	3.93E-45	postive
LIMS1	ABALON	0.507999242	1.92E-36	postive
IREB2	ABALON	0.578659875	3.83E-49	postive
PIK3CG	ABALON	0.502284066	1.53E-35	postive
JAK2	ABALON	0.545411715	8.62E-43	postive
NFAT5	ABALON	0.707997246	1.48E-82	postive
MALT1	ABALON	0.51741423	5.74E-38	postive
PIK3CA	ABALON	0.626135223	1.39E-59	postive
GSK3B	ABALON	0.516141353	9.29E-38	postive
GNRH1	ABALON	0.519007808	3.14E-38	postive
ANGPTL1	ABALON	0.595447467	1.23E-52	postive
BMPR2	ABALON	0.573434503	4.26E-48	postive
CRLF3	ABALON	0.552255521	4.85E-44	postive
NR2C2	ABALON	0.512544823	3.57E-37	postive
RORA	ABALON	0.651295309	6.90E-66	postive
SOS1	ABALON	0.559918799	1.79E-45	postive
BRAF	ABALON	0.567570802	6.05E-47	postive
CBL	ABALON	0.564838333	2.05E-46	postive
CBLB	ABALON	0.517214058	6.20E-38	postive
PDK1	ABALON	0.501583438	1.97E-35	postive
TRAJ1	ABALON	0.584540278	2.41E-50	postive
TRAJ2	ABALON	0.571841548	8.81E-48	postive
TRAJ3	ABALON	0.625450134	2.02E-59	postive
TRAJ5	ABALON	0.601749174	5.29E-54	postive
TRAJ6	ABALON	0.646945215	9.36E-65	postive
TRAJ8	ABALON	0.590760753	1.22E-51	postive
TRAJ10	ABALON	0.6318774	5.69E-61	postive
TRAJ12	ABALON	0.550452478	1.04E-43	postive
TRAJ13	ABALON	0.614277135	8.26E-57	postive
TRAJ14	ABALON	0.586565627	9.18E-51	postive
TRAJ16	ABALON	0.623056859	7.48E-59	postive
TRAJ17	ABALON	0.597712258	4.00E-53	postive
TRAJ18	ABALON	0.604188789	1.54E-54	postive
TRAJ21	ABALON	0.642396945	1.37E-63	postive
TRAJ31	ABALON	0.628821682	3.14E-60	postive
TRAJ37	ABALON	0.643554682	6.94E-64	postive
TRAJ38	ABALON	0.65566425	4.81E-67	postive
TRAJ39	ABALON	0.664799773	1.59E-69	postive
SCTR	HAGLR	0.586115946	1.14E-50	postive
BPIFB1	AC108134.4	0.539679924	9.12E-42	postive
DES	AC108134.4	0.644515148	3.94E-64	postive

GIPR	AC108134.4	0.532647247	1.55E-40	postive
SYTL1	TNFRSF14-AS1	0.532187594	1.86E-40	postive
IRF9	TNFRSF14-AS1	0.535466301	5.02E-41	postive
GIPR	TNFRSF14-AS1	0.625017693	2.56E-59	postive
TNFRSF14	TNFRSF14-AS1	0.738093662	3.85E-93	postive
IRF3	AC069281.2	0.597223419	5.10E-53	postive
TYK2	AC069281.2	0.581236586	1.15E-49	postive
SYTL1	AC069281.2	0.532827359	1.44E-40	postive
IRF9	AC069281.2	0.520407645	1.84E-38	postive
RABEP2	AC069281.2	0.518129289	4.38E-38	postive
GIPR	AC069281.2	0.511160174	5.98E-37	postive
OGFR	AC069281.2	0.581452338	1.04E-49	postive
TNFRSF14	AC069281.2	0.568521076	3.95E-47	postive
TNFRSF25	AC069281.2	0.510130546	8.75E-37	postive
DDX17	KIF1C-AS1	0.53094521	3.05E-40	postive
IRF9	KIF1C-AS1	0.544568514	1.22E-42	postive
LTB4R2	KIF1C-AS1	0.505402677	4.95E-36	postive
GNRH1	KIF1C-AS1	0.609225636	1.16E-55	postive
NR2C2	KIF1C-AS1	0.549783392	1.38E-43	postive
CREB1	AC068792.1	0.548427918	2.44E-43	postive
PSMD6	AC068792.1	0.516965195	6.81E-38	postive
EIF2AK2	AC068792.1	0.548354855	2.52E-43	postive
NFAT5	AC068792.1	0.531803118	2.17E-40	postive
PIK3CA	AC068792.1	0.540685184	6.05E-42	postive
ACVR2A	AC068792.1	0.522896818	7.07E-39	postive
ANGPTL1	AC068792.1	0.561678011	8.26E-46	postive
BMPR2	AC068792.1	0.536923578	2.79E-41	postive
RORA	AC068792.1	0.56515771	1.78E-46	postive
SOS1	AC068792.1	0.581755403	8.99E-50	postive
BRAF	AC068792.1	0.590207863	1.59E-51	postive
TRAJ1	AC068792.1	0.588372715	3.86E-51	postive
TRAJ3	AC068792.1	0.528025595	9.63E-40	postive
TRAJ5	AC068792.1	0.694170028	3.92E-78	postive
TRAJ6	AC068792.1	0.566734483	8.80E-47	postive
TRAJ8	AC068792.1	0.634093907	1.63E-61	postive
TRAJ10	AC068792.1	0.547787232	3.20E-43	postive
TRAJ12	AC068792.1	0.553953145	2.35E-44	postive
TRAJ13	AC068792.1	0.649647145	1.86E-65	postive
TRAJ14	AC068792.1	0.603870712	1.81E-54	postive
TRAJ16	AC068792.1	0.61871493	7.81E-58	postive
TRAJ17	AC068792.1	0.638513127	1.30E-62	postive
TRAJ18	AC068792.1	0.518551445	3.73E-38	postive
TRAJ21	AC068792.1	0.644987043	2.99E-64	postive
TRAJ31	AC068792.1	0.678935793	1.53E-73	postive
TRAJ37	AC068792.1	0.578290154	4.55E-49	postive
TRAJ38	AC068792.1	0.615590349	4.13E-57	postive
TRAJ39	AC068792.1	0.645513704	2.19E-64	postive
CRABP1	AC046143.1	0.527340762	1.26E-39	postive
IL17RD	AC046143.1	0.517839741	4.89E-38	postive
DDX17	LENG8-AS1	0.507821278	2.05E-36	postive
IRF9	LENG8-AS1	0.524066591	4.50E-39	postive
GNRH1	LENG8-AS1	0.537473959	2.23E-41	postive
NR2C2	LENG8-AS1	0.564771621	2.11E-46	postive
DDX17	RUSC1-AS1	0.608738229	1.49E-55	postive
IRF9	RUSC1-AS1	0.517233767	6.15E-38	postive
LTB4R2	RUSC1-AS1	0.591798026	7.35E-52	postive
GNRH1	RUSC1-AS1	0.657793805	1.29E-67	postive
NR2C1	RUSC1-AS1	0.500833219	2.58E-35	postive
NR2C2	RUSC1-AS1	0.66558981	9.61E-70	postive
BRAF	RUSC1-AS1	0.579249988	2.91E-49	postive
TRAJ3	RUSC1-AS1	0.514858289	1.51E-37	postive
CREB1	NEAT1	0.746049074	3.46E-96	postive
UBR1	NEAT1	0.604442605	1.35E-54	postive
ZC3HAV1	NEAT1	0.527159798	1.35E-39	postive
IL15	NEAT1	0.585185569	1.77E-50	postive

CYLD	NEAT1	0.579700651	2.36E-49	postive
EIF2AK2	NEAT1	0.543100166	2.24E-42	postive
MAPK8	NEAT1	0.534003534	9.03E-41	postive
NFKBIZ	NEAT1	0.532051786	1.97E-40	postive
LMBR1	NEAT1	0.549353362	1.66E-43	postive
LIMS1	NEAT1	0.519289495	2.82E-38	postive
IREB2	NEAT1	0.569115364	3.03E-47	postive
DDX17	NEAT1	0.606407944	4.95E-55	postive
PIK3CG	NEAT1	0.52682758	1.54E-39	postive
JAK2	NEAT1	0.658905083	6.49E-68	postive
TXK	NEAT1	0.603077891	2.70E-54	postive
NFAT5	NEAT1	0.764529873	1.00E-103	postive
MALT1	NEAT1	0.528892993	6.85E-40	postive
PIK3R1	NEAT1	0.619892078	4.15E-58	postive
PIK3CA	NEAT1	0.686239088	1.04E-75	postive
PIK3CB	NEAT1	0.525221562	2.88E-39	postive
RASGRP3	NEAT1	0.546204619	6.19E-43	postive
GNRH1	NEAT1	0.721278836	4.70E-87	postive
IL6ST	NEAT1	0.603015399	2.79E-54	postive
RABEP1	NEAT1	0.522928289	6.99E-39	postive
ACVR2A	NEAT1	0.565185235	1.76E-46	postive
ANGPTL1	NEAT1	0.791160422	6.76E-116	postive
BMPR2	NEAT1	0.704209337	2.56E-81	postive
CRLF3	NEAT1	0.62070552	2.68E-58	postive
NR2C2	NEAT1	0.686679585	7.69E-76	postive
RORA	NEAT1	0.806199569	1.38E-123	postive
SOS1	NEAT1	0.666709559	4.69E-70	postive
SOS2	NEAT1	0.541141571	5.02E-42	postive
BRAF	NEAT1	0.772311677	4.14E-107	postive
ITK	NEAT1	0.521098308	1.41E-38	postive
CBL	NEAT1	0.596442833	7.51E-53	postive
CBLB	NEAT1	0.80559107	2.91E-123	postive
PDK1	NEAT1	0.60063688	9.27E-54	postive
TRAJ1	NEAT1	0.650043057	1.47E-65	postive
TRAJ2	NEAT1	0.660169635	2.95E-68	postive
TRAJ3	NEAT1	0.698284831	2.01E-79	postive
TRAJ5	NEAT1	0.735826502	2.71E-92	postive
TRAJ6	NEAT1	0.705713232	8.30E-82	postive
TRAJ8	NEAT1	0.650525748	1.10E-65	postive
TRAJ10	NEAT1	0.685504557	1.73E-75	postive
TRAJ12	NEAT1	0.648867913	2.97E-65	postive
TRAJ13	NEAT1	0.690516712	5.25E-77	postive
TRAJ14	NEAT1	0.694727288	2.63E-78	postive
TRAJ16	NEAT1	0.718526497	4.22E-86	postive
TRAJ17	NEAT1	0.659488345	4.51E-68	postive
TRAJ18	NEAT1	0.676581149	7.39E-73	postive
TRAJ21	NEAT1	0.733492848	1.98E-91	postive
TRAJ31	NEAT1	0.751580598	2.25E-98	postive
TRAJ37	NEAT1	0.703223717	5.33E-81	postive
TRAJ38	NEAT1	0.810867244	4.11E-126	postive
TRAJ39	NEAT1	0.741654125	1.72E-94	postive
PTK2	AP003352.1	0.553441705	2.92E-44	postive
CREB1	AC019117.3	0.500007519	3.46E-35	postive
JAK2	AC019117.3	0.530400617	3.78E-40	postive
NFAT5	AC019117.3	0.552885488	3.71E-44	postive
CBLB	AC019117.3	0.571244748	1.16E-47	postive
TRAJ1	AC019117.3	0.527024443	1.43E-39	postive
TRAJ5	AC019117.3	0.548645388	2.23E-43	postive
TRAJ6	AC019117.3	0.552007616	5.39E-44	postive
TRAJ10	AC019117.3	0.55290606	3.68E-44	postive
TRAJ13	AC019117.3	0.504281396	7.44E-36	postive
TRAJ14	AC019117.3	0.516951178	6.84E-38	postive
TRAJ16	AC019117.3	0.529277117	5.89E-40	postive
TRAJ21	AC019117.3	0.507976419	1.93E-36	postive
TRAJ31	AC019117.3	0.528290282	8.68E-40	postive

TRAJ37	AC019117.3	0.513131918	2.87E-37	postive
TRAJ38	AC019117.3	0.587157572	6.92E-51	postive
TRAJ39	AC019117.3	0.517610705	5.33E-38	postive
FGFR1	AP003119.3	0.614269632	8.30E-57	postive
CREB1	AC010761.3	0.778907774	4.38E-110	postive
RFXAP	AC010761.3	0.549705137	1.43E-43	postive
UBR1	AC010761.3	0.673373132	6.19E-72	postive
ZC3HAV1	AC010761.3	0.591933409	6.88E-52	postive
ZC3HAV1L	AC010761.3	0.529622494	5.14E-40	postive
IL15	AC010761.3	0.516596132	7.83E-38	postive
CYLD	AC010761.3	0.586503147	9.46E-51	postive
EIF2AK2	AC010761.3	0.569000026	3.19E-47	postive
MAPK8	AC010761.3	0.590113858	1.66E-51	postive
LMBR1	AC010761.3	0.563914283	3.08E-46	postive
LIMS1	AC010761.3	0.500701996	2.70E-35	postive
IREB2	AC010761.3	0.639391845	7.84E-63	postive
DDX17	AC010761.3	0.664978837	1.42E-69	postive
PIK3CG	AC010761.3	0.529687732	5.01E-40	postive
JAK2	AC010761.3	0.617215902	1.74E-57	postive
TXK	AC010761.3	0.642332799	1.42E-63	postive
NFAT5	AC010761.3	0.81086143	4.14E-126	postive
MALT1	AC010761.3	0.541058528	5.19E-42	postive
PIK3R1	AC010761.3	0.644294621	4.49E-64	postive
PIK3CA	AC010761.3	0.675139499	1.93E-72	postive
PIK3CB	AC010761.3	0.507931592	1.97E-36	postive
AKT3	AC010761.3	0.563332489	3.99E-46	postive
RASGRP3	AC010761.3	0.527429971	1.22E-39	postive
IGHD4-4	AC010761.3	0.520072431	2.09E-38	postive
IGHD6-6	AC010761.3	0.567969035	5.06E-47	postive
LTB4R2	AC010761.3	0.509568246	1.08E-36	postive
GNRH1	AC010761.3	0.805805687	2.24E-123	postive
IL6ST	AC010761.3	0.631138573	8.61E-61	postive
RABEP1	AC010761.3	0.587770642	5.15E-51	postive
ACVR2A	AC010761.3	0.641462608	2.36E-63	postive
ANGPTL1	AC010761.3	0.739915082	7.90E-94	postive
BMPR1A	AC010761.3	0.555817071	1.06E-44	postive
BMPR2	AC010761.3	0.750057882	9.13E-98	postive
CRLF3	AC010761.3	0.675186099	1.87E-72	postive
NR1D2	AC010761.3	0.511809201	4.70E-37	postive
NR2C1	AC010761.3	0.535924522	4.18E-41	postive
NR2C2	AC010761.3	0.761246221	2.46E-102	postive
RORA	AC010761.3	0.81647088	3.07E-129	postive
SOS1	AC010761.3	0.703391206	4.71E-81	postive
SOS2	AC010761.3	0.573803548	3.60E-48	postive
BRAF	AC010761.3	0.836372788	2.84E-141	postive
TEC	AC010761.3	0.537841059	1.93E-41	postive
CBL	AC010761.3	0.60495415	1.04E-54	postive
CBLB	AC010761.3	0.744601133	1.26E-95	postive
RASGRP1	AC010761.3	0.516329802	8.65E-38	postive
PDK1	AC010761.3	0.586563998	9.19E-51	postive
TRAJ1	AC010761.3	0.684570376	3.30E-75	postive
TRAJ2	AC010761.3	0.717769833	7.68E-86	postive
TRAJ3	AC010761.3	0.750613778	5.48E-98	postive
TRAJ5	AC010761.3	0.715963804	3.19E-85	postive
TRAJ6	AC010761.3	0.728765511	1.04E-89	postive
TRAJ8	AC010761.3	0.724257478	4.23E-88	postive
TRAJ10	AC010761.3	0.67089264	3.14E-71	postive
TRAJ12	AC010761.3	0.628962116	2.90E-60	postive
TRAJ13	AC010761.3	0.71009776	2.99E-83	postive
TRAJ14	AC010761.3	0.697781996	2.90E-79	postive
TRAJ16	AC010761.3	0.723788903	6.19E-88	postive
TRAJ17	AC010761.3	0.661988351	9.42E-69	postive
TRAJ18	AC010761.3	0.633242788	2.63E-61	postive
TRAJ21	AC010761.3	0.734673257	7.26E-92	postive
TRAJ31	AC010761.3	0.746813728	1.74E-96	postive

TRAJ37	AC010761.3	0.732184376	5.98E-91	postive
TRAJ38	AC010761.3	0.796614879	1.31E-118	postive
TRAJ39	AC010761.3	0.774066432	6.85E-108	postive
CREB1	AC008669.1	0.615142072	5.23E-57	postive
UBR1	AC008669.1	0.550003018	1.26E-43	postive
CYLD	AC008669.1	0.541710976	3.97E-42	postive
DDX17	AC008669.1	0.551933152	5.56E-44	postive
NFAT5	AC008669.1	0.67888265	1.58E-73	postive
NFATC3	AC008669.1	0.504893544	5.96E-36	postive
PIK3R1	AC008669.1	0.601379822	6.38E-54	postive
GNRH1	AC008669.1	0.554928492	1.55E-44	postive
IL6ST	AC008669.1	0.651510992	6.06E-66	postive
RABEP1	AC008669.1	0.509464204	1.12E-36	postive
ACVR2A	AC008669.1	0.594561801	1.90E-52	postive
ANGPTL1	AC008669.1	0.575179538	1.91E-48	postive
BMPR1A	AC008669.1	0.515925544	1.01E-37	postive
BMPR2	AC008669.1	0.573606404	3.94E-48	postive
NR1D2	AC008669.1	0.503241636	1.08E-35	postive
NR2C2	AC008669.1	0.602769978	3.16E-54	postive
NR3C2	AC008669.1	0.52720217	1.33E-39	postive
RORA	AC008669.1	0.715585997	4.28E-85	postive
BRAF	AC008669.1	0.580288464	1.79E-49	postive
TRAJ1	AC008669.1	0.569332958	2.74E-47	postive
TRAJ2	AC008669.1	0.549145522	1.81E-43	postive
TRAJ3	AC008669.1	0.586755746	8.38E-51	postive
TRAJ5	AC008669.1	0.623394137	6.23E-59	postive
TRAJ6	AC008669.1	0.618937589	6.93E-58	postive
TRAJ8	AC008669.1	0.582060126	7.79E-50	postive
TRAJ10	AC008669.1	0.6256459	1.82E-59	postive
TRAJ12	AC008669.1	0.598497274	2.70E-53	postive
TRAJ13	AC008669.1	0.605339059	8.56E-55	postive
TRAJ14	AC008669.1	0.598497969	2.70E-53	postive
TRAJ16	AC008669.1	0.617428523	1.55E-57	postive
TRAJ17	AC008669.1	0.595609707	1.13E-52	postive
TRAJ18	AC008669.1	0.613505479	1.24E-56	postive
TRAJ21	AC008669.1	0.62340089	6.20E-59	postive
TRAJ31	AC008669.1	0.658353425	9.14E-68	postive
TRAJ37	AC008669.1	0.653846317	1.47E-66	postive
TRAJ38	AC008669.1	0.662843496	5.49E-69	postive
TRAJ39	AC008669.1	0.633398692	2.41E-61	postive
CREB1	AC018809.2	0.645014058	2.94E-64	postive
RFXAP	AC018809.2	0.531821902	2.15E-40	postive
UBR1	AC018809.2	0.619112317	6.31E-58	postive
CYLD	AC018809.2	0.566454633	9.97E-47	postive
IREB2	AC018809.2	0.525646285	2.44E-39	postive
DDX17	AC018809.2	0.663983228	2.67E-69	postive
NFAT5	AC018809.2	0.776832781	3.88E-109	postive
NFATC3	AC018809.2	0.605079416	9.77E-55	postive
PIK3R1	AC018809.2	0.573920443	3.41E-48	postive
GNRH1	AC018809.2	0.731333891	1.22E-90	postive
IL6ST	AC018809.2	0.604449788	1.35E-54	postive
RABEP1	AC018809.2	0.539557457	9.59E-42	postive
ACVR2A	AC018809.2	0.59716427	5.25E-53	postive
ANGPTL1	AC018809.2	0.533068265	1.31E-40	postive
BMPR2	AC018809.2	0.594917313	1.59E-52	postive
NR1D2	AC018809.2	0.528177037	9.08E-40	postive
NR2C2	AC018809.2	0.781562866	2.59E-111	postive
NR3C2	AC018809.2	0.5629868	4.65E-46	postive
RORA	AC018809.2	0.721494904	3.95E-87	postive
SOS1	AC018809.2	0.565817443	1.32E-46	postive
BRAF	AC018809.2	0.680929645	3.97E-74	postive
RASGRP1	AC018809.2	0.501972335	1.71E-35	postive
TRAJ1	AC018809.2	0.664208467	2.31E-69	postive
TRAJ2	AC018809.2	0.630301147	1.38E-60	postive
TRAJ3	AC018809.2	0.699377318	9.08E-80	postive

TRAJ5	AC018809.2	0.665851862	8.13E-70	postive
TRAJ6	AC018809.2	0.688780297	1.78E-76	postive
TRAJ8	AC018809.2	0.666220273	6.42E-70	postive
TRAJ10	AC018809.2	0.668573263	1.41E-70	postive
TRAJ12	AC018809.2	0.651316599	6.81E-66	postive
TRAJ13	AC018809.2	0.660102079	3.08E-68	postive
TRAJ14	AC018809.2	0.649595098	1.92E-65	postive
TRAJ16	AC018809.2	0.66316307	4.49E-69	postive
TRAJ17	AC018809.2	0.637277221	2.65E-62	postive
TRAJ18	AC018809.2	0.644527308	3.92E-64	postive
TRAJ21	AC018809.2	0.677329085	4.49E-73	postive
TRAJ31	AC018809.2	0.697348003	3.98E-79	postive
TRAJ37	AC018809.2	0.722029482	2.57E-87	postive
TRAJ38	AC018809.2	0.698428753	1.81E-79	postive
TRAJ39	AC018809.2	0.715437533	4.81E-85	postive
IRF9	AC037459.2	0.503048101	1.16E-35	postive
PLXNB1	AC037459.2	0.537348784	2.35E-41	postive
CREB1	AC131971.1	0.765292006	4.75E-104	postive
UBR1	AC131971.1	0.614095266	9.10E-57	postive
ZC3HAV1	AC131971.1	0.57871933	3.72E-49	postive
IL15	AC131971.1	0.623151942	7.10E-59	postive
CYLD	AC131971.1	0.590404312	1.45E-51	postive
EIF2AK2	AC131971.1	0.566831682	8.43E-47	postive
MAPK8	AC131971.1	0.565181472	1.76E-46	postive
LMBR1	AC131971.1	0.596607377	6.92E-53	postive
LIMS1	AC131971.1	0.526957325	1.46E-39	postive
IREB2	AC131971.1	0.599044897	2.06E-53	postive
DDX17	AC131971.1	0.56257542	5.57E-46	postive
PIK3CG	AC131971.1	0.557409451	5.31E-45	postive
JAK2	AC131971.1	0.628339709	4.10E-60	postive
TXK	AC131971.1	0.609182878	1.18E-55	postive
NFAT5	AC131971.1	0.78153053	2.69E-111	postive
MALT1	AC131971.1	0.554621177	1.77E-44	postive
PIK3R1	AC131971.1	0.680739866	4.52E-74	postive
PIK3CA	AC131971.1	0.715594989	4.25E-85	postive
PIK3CB	AC131971.1	0.556818737	6.86E-45	postive
AKT3	AC131971.1	0.568043248	4.90E-47	postive
RASGRP3	AC131971.1	0.527711483	1.09E-39	postive
IGHD6-6	AC131971.1	0.514376426	1.80E-37	postive
GNRH1	AC131971.1	0.667884721	2.20E-70	postive
IL6ST	AC131971.1	0.667380206	3.05E-70	postive
RABEP1	AC131971.1	0.529061382	6.41E-40	postive
ACVR2A	AC131971.1	0.599718689	1.47E-53	postive
ANGPTL1	AC131971.1	0.838952243	5.97E-143	postive
BMPR1A	AC131971.1	0.514249065	1.89E-37	postive
BMPR2	AC131971.1	0.735967556	2.40E-92	postive
CRLF3	AC131971.1	0.632406469	4.22E-61	postive
IL18R1	AC131971.1	0.51633643	8.63E-38	postive
NR2C2	AC131971.1	0.640016605	5.46E-63	postive
RORA	AC131971.1	0.845112399	4.42E-147	postive
SOS1	AC131971.1	0.667661095	2.55E-70	postive
SOS2	AC131971.1	0.580192741	1.87E-49	postive
BRAF	AC131971.1	0.82945637	6.49E-137	postive
ITK	AC131971.1	0.591704867	7.69E-52	postive
CD28	AC131971.1	0.512823149	3.22E-37	postive
CBL	AC131971.1	0.618501721	8.75E-58	postive
CBLB	AC131971.1	0.746592792	2.12E-96	postive
RASGRP1	AC131971.1	0.525723014	2.37E-39	postive
PDK1	AC131971.1	0.613680722	1.13E-56	postive
TRAJ1	AC131971.1	0.677844179	3.18E-73	postive
TRAJ2	AC131971.1	0.701117663	2.53E-80	postive
TRAJ3	AC131971.1	0.768919132	1.29E-105	postive
TRAJ5	AC131971.1	0.805726208	2.47E-123	postive
TRAJ6	AC131971.1	0.74734854	1.07E-96	postive
TRAJ8	AC131971.1	0.751450926	2.54E-98	postive

TRAJ10	AC131971.1	0.734006198	1.28E-91	postive
TRAJ12	AC131971.1	0.668666849	1.33E-70	postive
TRAJ13	AC131971.1	0.790733522	1.09E-115	postive
TRAJ14	AC131971.1	0.76493312	6.76E-104	postive
TRAJ16	AC131971.1	0.747836438	6.90E-97	postive
TRAJ17	AC131971.1	0.748341569	4.36E-97	postive
TRAJ18	AC131971.1	0.707557017	2.07E-82	postive
TRAJ21	AC131971.1	0.823394867	2.97E-133	postive
TRAJ31	AC131971.1	0.8228074	6.62E-133	postive
TRAJ37	AC131971.1	0.778314491	8.18E-110	postive
TRAJ38	AC131971.1	0.840321642	7.46E-144	postive
TRAJ39	AC131971.1	0.82686141	2.50E-135	postive
CREB1	AP003486.1	0.586709144	8.57E-51	postive
CIITA	AP003486.1	0.529401631	5.61E-40	postive
UBR1	AP003486.1	0.531167042	2.79E-40	postive
ZC3HAV1	AP003486.1	0.572954803	5.31E-48	postive
CYLD	AP003486.1	0.622670275	9.23E-59	postive
DDX17	AP003486.1	0.663365697	3.95E-69	postive
JAK2	AP003486.1	0.527718048	1.09E-39	postive
TXK	AP003486.1	0.513472838	2.53E-37	postive
NFAT5	AP003486.1	0.678441628	2.13E-73	postive
NFATC3	AP003486.1	0.502295064	1.53E-35	postive
PIK3R1	AP003486.1	0.568344309	4.28E-47	postive
PIK3CA	AP003486.1	0.518190517	4.28E-38	postive
GNRH1	AP003486.1	0.652988105	2.47E-66	postive
IL6ST	AP003486.1	0.515729527	1.08E-37	postive
RABEP1	AP003486.1	0.500626388	2.78E-35	postive
ACVR2A	AP003486.1	0.526133623	2.02E-39	postive
ANGPTL1	AP003486.1	0.5405469	6.40E-42	postive
BMPR2	AP003486.1	0.556997299	6.35E-45	postive
NR2C2	AP003486.1	0.690281258	6.20E-77	postive
RORA	AP003486.1	0.669138709	9.81E-71	postive
SOS1	AP003486.1	0.552372924	4.61E-44	postive
SOS2	AP003486.1	0.502146921	1.61E-35	postive
BRAF	AP003486.1	0.635585649	6.97E-62	postive
CBL	AP003486.1	0.61724247	1.72E-57	postive
CBLB	AP003486.1	0.608854226	1.40E-55	postive
TRAJ1	AP003486.1	0.554329996	2.00E-44	postive
TRAJ2	AP003486.1	0.570921295	1.34E-47	postive
TRAJ3	AP003486.1	0.602774688	3.15E-54	postive
TRAJ5	AP003486.1	0.556972923	6.42E-45	postive
TRAJ6	AP003486.1	0.567289487	6.87E-47	postive
TRAJ8	AP003486.1	0.514347536	1.82E-37	postive
TRAJ10	AP003486.1	0.539667165	9.17E-42	postive
TRAJ12	AP003486.1	0.531261677	2.69E-40	postive
TRAJ13	AP003486.1	0.536920092	2.79E-41	postive
TRAJ14	AP003486.1	0.538968095	1.22E-41	postive
TRAJ16	AP003486.1	0.54945207	1.59E-43	postive
TRAJ17	AP003486.1	0.511360889	5.55E-37	postive
TRAJ18	AP003486.1	0.558134324	3.88E-45	postive
TRAJ21	AP003486.1	0.561877363	7.57E-46	postive
TRAJ31	AP003486.1	0.5683516	4.27E-47	postive
TRAJ37	AP003486.1	0.576706051	9.47E-49	postive
TRAJ38	AP003486.1	0.603707973	1.96E-54	postive
TRAJ39	AP003486.1	0.568981409	3.21E-47	postive
IRF3	AL390719.2	0.521394955	1.26E-38	postive
SYTL1	AL390719.2	0.573110006	4.94E-48	postive
IRF9	AL390719.2	0.51286098	3.18E-37	postive
PLXNB1	AL390719.2	0.561499951	8.94E-46	postive
RABEP2	AL390719.2	0.545547241	8.14E-43	postive
GIPR	AL390719.2	0.624956192	2.65E-59	postive
TNFRSF14	AL390719.2	0.540279824	7.14E-42	postive
CREB1	AC005034.6	0.687844945	3.42E-76	postive
UBR1	AC005034.6	0.574141582	3.08E-48	postive
ZC3HAV1	AC005034.6	0.528212945	8.95E-40	postive

CYLD	AC005034.6	0.523863436	4.87E-39	postive
EIF2AK2	AC005034.6	0.559093985	2.56E-45	postive
MAPK8	AC005034.6	0.521435512	1.24E-38	postive
IREB2	AC005034.6	0.590742506	1.23E-51	postive
DDX17	AC005034.6	0.574789895	2.29E-48	postive
JAK2	AC005034.6	0.504564187	6.72E-36	postive
NFAT5	AC005034.6	0.697359331	3.94E-79	postive
PIK3R1	AC005034.6	0.570315835	1.76E-47	postive
PIK3CA	AC005034.6	0.598030428	3.41E-53	postive
GNRH1	AC005034.6	0.624806405	2.88E-59	postive
IL6ST	AC005034.6	0.572106726	7.81E-48	postive
RABEP1	AC005034.6	0.544576836	1.22E-42	postive
ACVR2A	AC005034.6	0.594885096	1.62E-52	postive
ANGPTL1	AC005034.6	0.631405144	7.41E-61	postive
BMPR1A	AC005034.6	0.526937443	1.47E-39	postive
BMPR2	AC005034.6	0.678906473	1.56E-73	postive
NR1D2	AC005034.6	0.518603286	3.66E-38	postive
NR2C2	AC005034.6	0.671826691	1.71E-71	postive
RORA	AC005034.6	0.709563475	4.50E-83	postive
SOS1	AC005034.6	0.722499102	1.76E-87	postive
SOS2	AC005034.6	0.529622999	5.14E-40	postive
BRAF	AC005034.6	0.692308819	1.48E-77	postive
CBL	AC005034.6	0.552661496	4.08E-44	postive
CBLB	AC005034.6	0.641298977	2.59E-63	postive
TRAJ1	AC005034.6	0.575266191	1.84E-48	postive
TRAJ2	AC005034.6	0.605371073	8.42E-55	postive
TRAJ3	AC005034.6	0.628495323	3.76E-60	postive
TRAJ5	AC005034.6	0.614574638	7.06E-57	postive
TRAJ6	AC005034.6	0.61251922	2.08E-56	postive
TRAJ8	AC005034.6	0.603078262	2.70E-54	postive
TRAJ10	AC005034.6	0.574355909	2.80E-48	postive
TRAJ12	AC005034.6	0.574992216	2.09E-48	postive
TRAJ13	AC005034.6	0.599845545	1.38E-53	postive
TRAJ14	AC005034.6	0.598857077	2.26E-53	postive
TRAJ16	AC005034.6	0.605186457	9.25E-55	postive
TRAJ17	AC005034.6	0.562450393	5.88E-46	postive
TRAJ18	AC005034.6	0.55901755	2.64E-45	postive
TRAJ21	AC005034.6	0.616243282	2.92E-57	postive
TRAJ31	AC005034.6	0.649202521	2.43E-65	postive
TRAJ37	AC005034.6	0.634287125	1.46E-61	postive
TRAJ38	AC005034.6	0.674540947	2.86E-72	postive
TRAJ39	AC005034.6	0.63040491	1.30E-60	postive
DDX17	AP002840.2	0.513470001	2.53E-37	postive
CREB1	AC008115.3	0.760712208	4.12E-102	postive
UBR1	AC008115.3	0.588603362	3.45E-51	postive
ZC3HAV1	AC008115.3	0.58973568	2.00E-51	postive
ZC3HAV1L	AC008115.3	0.522115677	9.56E-39	postive
IL15	AC008115.3	0.658352771	9.15E-68	postive
CYLD	AC008115.3	0.665968392	7.54E-70	postive
EIF2AK2	AC008115.3	0.574320194	2.84E-48	postive
MAPK8	AC008115.3	0.528561778	7.80E-40	postive
TLR1	AC008115.3	0.509605674	1.06E-36	postive
NFKBIZ	AC008115.3	0.538677098	1.37E-41	postive
LMBR1	AC008115.3	0.585285834	1.69E-50	postive
LIMS1	AC008115.3	0.512685069	3.39E-37	postive
IREB2	AC008115.3	0.569536582	2.50E-47	postive
DDX17	AC008115.3	0.632776127	3.43E-61	postive
PIK3CG	AC008115.3	0.62654421	1.11E-59	postive
JAK2	AC008115.3	0.704453478	2.13E-81	postive
TXK	AC008115.3	0.609917802	8.09E-56	postive
NFAT5	AC008115.3	0.792969598	8.69E-117	postive
NFATC2	AC008115.3	0.510091307	8.88E-37	postive
MALT1	AC008115.3	0.575683989	1.52E-48	postive
PIK3R1	AC008115.3	0.655526809	5.24E-67	postive
PIK3CA	AC008115.3	0.705770705	7.95E-82	postive

PIK3CB	AC008115.3	0.5600758	1.67E-45	postive
AKT3	AC008115.3	0.504226618	7.59E-36	postive
RASGRP3	AC008115.3	0.615022921	5.57E-57	postive
IGHD6-6	AC008115.3	0.55227484	4.81E-44	postive
LTB4R2	AC008115.3	0.50915109	1.26E-36	postive
PLXNC1	AC008115.3	0.563953189	3.03E-46	postive
GNRH1	AC008115.3	0.703085312	5.91E-81	postive
IL6ST	AC008115.3	0.621703127	1.56E-58	postive
RABEP1	AC008115.3	0.534640698	6.99E-41	postive
ACVR2A	AC008115.3	0.557169242	5.89E-45	postive
ANGPTL1	AC008115.3	0.780777211	6.01E-111	postive
BMPR2	AC008115.3	0.672542983	1.07E-71	postive
CRLF3	AC008115.3	0.67252171	1.08E-71	postive
IL18R1	AC008115.3	0.570987066	1.30E-47	postive
NR2C2	AC008115.3	0.713379413	2.39E-84	postive
RORA	AC008115.3	0.835876356	5.93E-141	postive
SOS1	AC008115.3	0.665409534	1.08E-69	postive
SOS2	AC008115.3	0.585000928	1.94E-50	postive
BRAF	AC008115.3	0.788686222	1.08E-114	postive
PTPRC	AC008115.3	0.573100499	4.96E-48	postive
ITK	AC008115.3	0.675463413	1.55E-72	postive
CD28	AC008115.3	0.58040021	1.70E-49	postive
CBL	AC008115.3	0.65771381	1.36E-67	postive
CBLB	AC008115.3	0.730656291	2.16E-90	postive
RASGRP1	AC008115.3	0.573722739	3.74E-48	postive
PDK1	AC008115.3	0.67128387	2.43E-71	postive
TRAJ1	AC008115.3	0.731916245	7.49E-91	postive
TRAJ2	AC008115.3	0.695944331	1.10E-78	postive
TRAJ3	AC008115.3	0.781579808	2.55E-111	postive
TRAJ5	AC008115.3	0.818444994	2.29E-130	postive
TRAJ6	AC008115.3	0.751096366	3.52E-98	postive
TRAJ8	AC008115.3	0.704852764	1.58E-81	postive
TRAJ10	AC008115.3	0.755291822	7.14E-100	postive
TRAJ12	AC008115.3	0.674038717	3.99E-72	postive
TRAJ13	AC008115.3	0.756225773	2.96E-100	postive
TRAJ14	AC008115.3	0.737254325	7.94E-93	postive
TRAJ16	AC008115.3	0.755187245	7.87E-100	postive
TRAJ17	AC008115.3	0.751031407	3.74E-98	postive
TRAJ18	AC008115.3	0.747001888	1.47E-96	postive
TRAJ21	AC008115.3	0.798572374	1.32E-119	postive
TRAJ31	AC008115.3	0.797023449	8.12E-119	postive
TRAJ37	AC008115.3	0.764257983	1.31E-103	postive
TRAJ38	AC008115.3	0.822349529	1.23E-132	postive
TRAJ39	AC008115.3	0.810577938	5.93E-126	postive
CREB1	AC018752.1	0.814956958	2.20E-128	postive
RFXAP	AC018752.1	0.544711651	1.15E-42	postive
UBR1	AC018752.1	0.691613295	2.42E-77	postive
ZC3HAV1	AC018752.1	0.572122336	7.75E-48	postive
ZC3HAV1L	AC018752.1	0.521581412	1.17E-38	postive
IL15	AC018752.1	0.584338095	2.65E-50	postive
CYLD	AC018752.1	0.638662952	1.19E-62	postive
EIF2AK2	AC018752.1	0.527182378	1.34E-39	postive
MAPK8	AC018752.1	0.565011804	1.90E-46	postive
LMBR1	AC018752.1	0.577398148	6.88E-49	postive
IREB2	AC018752.1	0.656650117	2.62E-67	postive
DDX17	AC018752.1	0.603270899	2.45E-54	postive
PIK3CG	AC018752.1	0.552077152	5.23E-44	postive
JAK2	AC018752.1	0.617190808	1.76E-57	postive
TXK	AC018752.1	0.550801937	8.98E-44	postive
PPP3CB	AC018752.1	0.510217947	8.48E-37	postive
NFAT5	AC018752.1	0.869520451	2.03E-165	postive
NFATC3	AC018752.1	0.56390287	3.10E-46	postive
MALT1	AC018752.1	0.535362601	5.23E-41	postive
PIK3R1	AC018752.1	0.677893322	3.08E-73	postive
PIK3CA	AC018752.1	0.679057463	1.41E-73	postive

PIK3CB	AC018752.1	0.518027467	4.55E-38	postive
AKT3	AC018752.1	0.55481909	1.62E-44	postive
RASGRP3	AC018752.1	0.527807329	1.05E-39	postive
IGHD4-4	AC018752.1	0.556470525	7.97E-45	postive
IGHD6-6	AC018752.1	0.511516279	5.24E-37	postive
PLXNC1	AC018752.1	0.524531327	3.76E-39	postive
GNRH1	AC018752.1	0.712242413	5.76E-84	postive
IL6ST	AC018752.1	0.669562841	7.45E-71	postive
RABEP1	AC018752.1	0.607266898	3.18E-55	postive
ACVR2A	AC018752.1	0.68229186	1.57E-74	postive
ANGPTL1	AC018752.1	0.744384673	1.53E-95	postive
BMPR1A	AC018752.1	0.600039331	1.25E-53	postive
BMPR2	AC018752.1	0.75233094	1.13E-98	postive
CRLF3	AC018752.1	0.640884608	3.30E-63	postive
LIFR	AC018752.1	0.506822956	2.95E-36	postive
NR1D2	AC018752.1	0.563450282	3.79E-46	postive
NR2C2	AC018752.1	0.718901493	3.13E-86	postive
NR3C1	AC018752.1	0.543240017	2.12E-42	postive
RORA	AC018752.1	0.837715589	3.84E-142	postive
SOS1	AC018752.1	0.706654643	4.09E-82	postive
SOS2	AC018752.1	0.615399975	4.57E-57	postive
BRAF	AC018752.1	0.825465875	1.73E-134	postive
CD28	AC018752.1	0.515627912	1.13E-37	postive
CBL	AC018752.1	0.587817534	5.04E-51	postive
CBLB	AC018752.1	0.673212372	6.88E-72	postive
RASGRP1	AC018752.1	0.52613329	2.02E-39	postive
PDK1	AC018752.1	0.55025664	1.13E-43	postive
TRAJ1	AC018752.1	0.766681015	1.20E-104	postive
TRAJ2	AC018752.1	0.785585805	3.32E-113	postive
TRAJ3	AC018752.1	0.841925243	6.38E-145	postive
TRAJ5	AC018752.1	0.808532728	7.69E-125	postive
TRAJ6	AC018752.1	0.835664137	8.12E-141	postive
TRAJ8	AC018752.1	0.829545873	5.72E-137	postive
TRAJ10	AC018752.1	0.815975412	5.87E-129	postive
TRAJ12	AC018752.1	0.771995271	5.72E-107	postive
TRAJ13	AC018752.1	0.824496429	6.59E-134	postive
TRAJ14	AC018752.1	0.820947185	8.17E-132	postive
TRAJ16	AC018752.1	0.810467148	6.81E-126	postive
TRAJ17	AC018752.1	0.794871874	9.82E-118	postive
TRAJ18	AC018752.1	0.754464091	1.55E-99	postive
TRAJ21	AC018752.1	0.837546579	4.94E-142	postive
TRAJ31	AC018752.1	0.862499893	8.72E-160	postive
TRAJ37	AC018752.1	0.860383518	3.79E-158	postive
TRAJ38	AC018752.1	0.888514382	1.82E-182	postive
TRAJ39	AC018752.1	0.862564884	7.76E-160	postive
CREB1	AC116366.2	0.736872418	1.10E-92	postive
CIITA	AC116366.2	0.636919448	3.25E-62	postive
RFXAP	AC116366.2	0.544321574	1.35E-42	postive
UBR1	AC116366.2	0.597471719	4.51E-53	postive
ZC3HAV1	AC116366.2	0.574984206	2.09E-48	postive
IL15	AC116366.2	0.670671856	3.63E-71	postive
CYLD	AC116366.2	0.729906483	4.04E-90	postive
EIF2AK2	AC116366.2	0.543421445	1.96E-42	postive
NFKBIZ	AC116366.2	0.540066307	7.79E-42	postive
IREB2	AC116366.2	0.535519108	4.92E-41	postive
DDX17	AC116366.2	0.707122025	2.87E-82	postive
PIK3CG	AC116366.2	0.59640413	7.65E-53	postive
JAK2	AC116366.2	0.732957004	3.11E-91	postive
TXK	AC116366.2	0.59151393	8.44E-52	postive
NFAT5	AC116366.2	0.842096547	4.90E-145	postive
NFATC3	AC116366.2	0.54495018	1.04E-42	postive
MALT1	AC116366.2	0.525457516	2.63E-39	postive
PIK3R1	AC116366.2	0.633192133	2.71E-61	postive
PIK3CA	AC116366.2	0.636700119	3.69E-62	postive
RASGRP3	AC116366.2	0.641515371	2.29E-63	postive

IGHD6-6	AC116366.2	0.548400083	2.47E-43	postive
LTB4R2	AC116366.2	0.548151085	2.75E-43	postive
PLXNC1	AC116366.2	0.609064538	1.26E-55	postive
GNRH1	AC116366.2	0.768115135	2.88E-105	postive
IL6ST	AC116366.2	0.580836814	1.38E-49	postive
IL7	AC116366.2	0.529170498	6.14E-40	postive
RABEP1	AC116366.2	0.565688555	1.40E-46	postive
ACVR2A	AC116366.2	0.60343168	2.26E-54	postive
ANGPTL1	AC116366.2	0.686003808	1.23E-75	postive
BMPR2	AC116366.2	0.639323076	8.16E-63	postive
CRLF3	AC116366.2	0.625984831	1.51E-59	postive
NR2C1	AC116366.2	0.519523264	2.58E-38	postive
NR2C2	AC116366.2	0.76963229	6.28E-106	postive
RORA	AC116366.2	0.802922315	7.45E-122	postive
SOS1	AC116366.2	0.66790538	2.18E-70	postive
SOS2	AC116366.2	0.586612812	8.98E-51	postive
BRAF	AC116366.2	0.729130318	7.70E-90	postive
PTPRC	AC116366.2	0.571303991	1.12E-47	postive
ITK	AC116366.2	0.618621624	8.21E-58	postive
CD28	AC116366.2	0.594360447	2.10E-52	postive
ICOS	AC116366.2	0.502578091	1.38E-35	postive
CBL	AC116366.2	0.631922011	5.54E-61	postive
CBLB	AC116366.2	0.683582061	6.51E-75	postive
RASGRP1	AC116366.2	0.586121566	1.14E-50	postive
PDK1	AC116366.2	0.547879201	3.08E-43	postive
TRAJ1	AC116366.2	0.841752287	8.33E-145	postive
TRAJ2	AC116366.2	0.783747816	2.46E-112	postive
TRAJ3	AC116366.2	0.860468312	3.26E-158	postive
TRAJ5	AC116366.2	0.818978596	1.13E-130	postive
TRAJ6	AC116366.2	0.839104496	4.74E-143	postive
TRAJ8	AC116366.2	0.770610448	2.34E-106	postive
TRAJ10	AC116366.2	0.828403781	2.87E-136	postive
TRAJ12	AC116366.2	0.792142729	2.22E-116	postive
TRAJ13	AC116366.2	0.781511678	2.74E-111	postive
TRAJ14	AC116366.2	0.801941471	2.42E-121	postive
TRAJ16	AC116366.2	0.827027326	1.98E-135	postive
TRAJ17	AC116366.2	0.796083407	2.42E-118	postive
TRAJ18	AC116366.2	0.831531125	3.35E-138	postive
TRAJ21	AC116366.2	0.818479926	2.19E-130	postive
TRAJ31	AC116366.2	0.825515082	1.62E-134	postive
TRAJ37	AC116366.2	0.82304848	4.77E-133	postive
TRAJ38	AC116366.2	0.860329777	4.16E-158	postive
TRAJ39	AC116366.2	0.836033896	4.70E-141	postive
CREB1	LINC01290	0.614150913	8.83E-57	postive
UBR1	LINC01290	0.6043655	1.41E-54	postive
CYLD	LINC01290	0.53201811	1.99E-40	postive
DDX17	LINC01290	0.57111641	1.22E-47	postive
JAK2	LINC01290	0.513788825	2.25E-37	postive
TXK	LINC01290	0.5415704	4.21E-42	postive
NFAT5	LINC01290	0.684424836	3.65E-75	postive
PIK3R1	LINC01290	0.521716353	1.11E-38	postive
PIK3CA	LINC01290	0.500521813	2.88E-35	postive
ROBO2	LINC01290	0.654039219	1.30E-66	postive
GNRH1	LINC01290	0.638848829	1.07E-62	postive
ACVR2A	LINC01290	0.531455749	2.49E-40	postive
ANGPTL1	LINC01290	0.513993194	2.08E-37	postive
BMPR1A	LINC01290	0.50578307	4.31E-36	postive
BMPR2	LINC01290	0.617088942	1.86E-57	postive
NR1D2	LINC01290	0.572128572	7.73E-48	postive
NR2C2	LINC01290	0.606105159	5.78E-55	postive
NR3C2	LINC01290	0.544117144	1.47E-42	postive
RORA	LINC01290	0.644882098	3.18E-64	postive
SOS1	LINC01290	0.508862288	1.40E-36	postive
BRAF	LINC01290	0.601565793	5.81E-54	postive
CBLB	LINC01290	0.568970092	3.23E-47	postive

TRAJ1	LINC01290	0.515704644	1.10E-37	postive
TRAJ2	LINC01290	0.56514094	1.79E-46	postive
TRAJ3	LINC01290	0.538566851	1.43E-41	postive
TRAJ5	LINC01290	0.502565696	1.38E-35	postive
TRAJ6	LINC01290	0.586041411	1.18E-50	postive
TRAJ10	LINC01290	0.542929581	2.41E-42	postive
TRAJ12	LINC01290	0.628884998	3.03E-60	postive
TRAJ13	LINC01290	0.522539618	8.12E-39	postive
TRAJ14	LINC01290	0.539708208	9.01E-42	postive
TRAJ16	LINC01290	0.534508784	7.37E-41	postive
TRAJ18	LINC01290	0.594632177	1.83E-52	postive
TRAJ21	LINC01290	0.551173272	7.68E-44	postive
TRAJ31	LINC01290	0.591166026	9.99E-52	postive
TRAJ37	LINC01290	0.578355195	4.41E-49	postive
TRAJ38	LINC01290	0.640942209	3.19E-63	postive
TRAJ39	LINC01290	0.516166684	9.20E-38	postive
TMSB15A	AC027031.2	0.700173364	5.07E-80	postive
MSTN	AC027031.2	0.796762801	1.10E-118	postive
CREB1	FAM13A-AS1	0.734063772	1.22E-91	postive
RFXAP	FAM13A-AS1	0.540453437	6.65E-42	postive
UBR1	FAM13A-AS1	0.632668296	3.64E-61	postive
ZC3HAV1	FAM13A-AS1	0.52541722	2.67E-39	postive
IL15	FAM13A-AS1	0.541891834	3.69E-42	postive
CYLD	FAM13A-AS1	0.616637476	2.37E-57	postive
EIF2AK2	FAM13A-AS1	0.55802779	4.06E-45	postive
MAPK8	FAM13A-AS1	0.536285938	3.61E-41	postive
NFKBIZ	FAM13A-AS1	0.511752792	4.80E-37	postive
IREB2	FAM13A-AS1	0.582681394	5.81E-50	postive
DDX17	FAM13A-AS1	0.717053523	1.35E-85	postive
JAK2	FAM13A-AS1	0.627166424	7.85E-60	postive
TXK	FAM13A-AS1	0.6716985	1.86E-71	postive
NFAT5	FAM13A-AS1	0.836268195	3.32E-141	postive
MALT1	FAM13A-AS1	0.508487934	1.60E-36	postive
PIK3R1	FAM13A-AS1	0.628432446	3.90E-60	postive
PIK3CA	FAM13A-AS1	0.616452854	2.61E-57	postive
RASGRP3	FAM13A-AS1	0.510882829	6.63E-37	postive
LTB4R2	FAM13A-AS1	0.517451204	5.66E-38	postive
ROBO2	FAM13A-AS1	0.520644517	1.68E-38	postive
GNRH1	FAM13A-AS1	0.83044907	1.58E-137	postive
IL6ST	FAM13A-AS1	0.617572777	1.44E-57	postive
RABEP1	FAM13A-AS1	0.560164443	1.60E-45	postive
ACVR2A	FAM13A-AS1	0.630764819	1.06E-60	postive
ANGPTL1	FAM13A-AS1	0.690951028	3.87E-77	postive
BMPR1A	FAM13A-AS1	0.529271627	5.90E-40	postive
BMPR2	FAM13A-AS1	0.692286424	1.50E-77	postive
CRLF3	FAM13A-AS1	0.595766101	1.05E-52	postive
NR1D2	FAM13A-AS1	0.527311115	1.27E-39	postive
NR2C1	FAM13A-AS1	0.576262838	1.16E-48	postive
NR2C2	FAM13A-AS1	0.792326874	1.81E-116	postive
RORA	FAM13A-AS1	0.824038654	1.23E-133	postive
SOS1	FAM13A-AS1	0.658824515	6.82E-68	postive
SOS2	FAM13A-AS1	0.541018547	5.28E-42	postive
BRAF	FAM13A-AS1	0.784140113	1.61E-112	postive
ITK	FAM13A-AS1	0.504209076	7.64E-36	postive
TEC	FAM13A-AS1	0.502733105	1.30E-35	postive
CBL	FAM13A-AS1	0.55370573	2.61E-44	postive
CBLB	FAM13A-AS1	0.705360834	1.08E-81	postive
RASGRP1	FAM13A-AS1	0.500825597	2.59E-35	postive
PDK1	FAM13A-AS1	0.502504764	1.41E-35	postive
TRAJ1	FAM13A-AS1	0.69731675	4.07E-79	postive
TRAJ2	FAM13A-AS1	0.693846858	4.94E-78	postive
TRAJ3	FAM13A-AS1	0.73511604	4.97E-92	postive
TRAJ5	FAM13A-AS1	0.722466976	1.80E-87	postive
TRAJ6	FAM13A-AS1	0.734990977	5.53E-92	postive
TRAJ8	FAM13A-AS1	0.671961183	1.56E-71	postive

TRAJ10	FAM13A-AS1	0.720217734	1.10E-86	postive
TRAJ12	FAM13A-AS1	0.702463837	9.37E-81	postive
TRAJ13	FAM13A-AS1	0.700750876	3.32E-80	postive
TRAJ14	FAM13A-AS1	0.696877703	5.59E-79	postive
TRAJ16	FAM13A-AS1	0.724043259	5.04E-88	postive
TRAJ17	FAM13A-AS1	0.673054655	7.63E-72	postive
TRAJ18	FAM13A-AS1	0.714037833	1.43E-84	postive
TRAJ21	FAM13A-AS1	0.734882029	6.07E-92	postive
TRAJ31	FAM13A-AS1	0.764977175	6.47E-104	postive
TRAJ37	FAM13A-AS1	0.755756218	4.61E-100	postive
TRAJ38	FAM13A-AS1	0.802510006	1.22E-121	postive
TRAJ39	FAM13A-AS1	0.743196709	4.41E-95	postive
IGHV4-28	AC093001.1	0.50221595	1.57E-35	postive
CREB1	AC009032.1	0.773569288	1.14E-107	postive
RFXAP	AC009032.1	0.50667232	3.12E-36	postive
UBR1	AC009032.1	0.654635239	9.05E-67	postive
ZC3HAV1	AC009032.1	0.530699183	3.36E-40	postive
IL15	AC009032.1	0.519468001	2.63E-38	postive
CYLD	AC009032.1	0.663920106	2.78E-69	postive
MAPK8	AC009032.1	0.542978732	2.36E-42	postive
LMBR1	AC009032.1	0.546383398	5.75E-43	postive
IREB2	AC009032.1	0.579829398	2.22E-49	postive
DDX17	AC009032.1	0.592076055	6.42E-52	postive
PIK3CG	AC009032.1	0.513050373	2.96E-37	postive
JAK2	AC009032.1	0.618329433	9.60E-58	postive
TXK	AC009032.1	0.540006104	7.98E-42	postive
NFAT5	AC009032.1	0.959315841	6.52E-295	postive
NFATC3	AC009032.1	0.637228738	2.72E-62	postive
MALT1	AC009032.1	0.510391983	7.95E-37	postive
PIK3R1	AC009032.1	0.607906081	2.29E-55	postive
PIK3CA	AC009032.1	0.640594025	3.91E-63	postive
AKT3	AC009032.1	0.546824851	4.78E-43	postive
IGHD6-6	AC009032.1	0.519954453	2.19E-38	postive
GNRH1	AC009032.1	0.735151925	4.82E-92	postive
IL6ST	AC009032.1	0.618322654	9.63E-58	postive
RABEP1	AC009032.1	0.559942548	1.77E-45	postive
ACVR2A	AC009032.1	0.644086322	5.08E-64	postive
ANGPTL1	AC009032.1	0.683354762	7.61E-75	postive
BMPR1A	AC009032.1	0.569041692	3.13E-47	postive
BMPR2	AC009032.1	0.698315662	1.97E-79	postive
CRLF3	AC009032.1	0.655924978	4.10E-67	postive
NR1D2	AC009032.1	0.518523144	3.77E-38	postive
NR2C2	AC009032.1	0.643255601	8.27E-64	postive
RORA	AC009032.1	0.857178392	1.02E-155	postive
SOS1	AC009032.1	0.643255995	8.27E-64	postive
SOS2	AC009032.1	0.556162939	9.10E-45	postive
BRAF	AC009032.1	0.760677606	4.26E-102	postive
CBL	AC009032.1	0.559454819	2.19E-45	postive
CBLB	AC009032.1	0.625466762	2.00E-59	postive
PDK1	AC009032.1	0.51378228	2.25E-37	postive
TRAJ1	AC009032.1	0.826303673	5.42E-135	postive
TRAJ2	AC009032.1	0.849435762	4.34E-150	postive
TRAJ3	AC009032.1	0.872832852	3.42E-168	postive
TRAJ5	AC009032.1	0.825453328	1.76E-134	postive
TRAJ6	AC009032.1	0.910566179	1.21E-206	postive
TRAJ8	AC009032.1	0.877882088	1.43E-172	postive
TRAJ10	AC009032.1	0.887041771	4.88E-181	postive
TRAJ12	AC009032.1	0.796362524	1.75E-118	postive
TRAJ13	AC009032.1	0.895598842	1.23E-189	postive
TRAJ14	AC009032.1	0.858880864	5.30E-157	postive
TRAJ16	AC009032.1	0.888996071	6.13E-183	postive
TRAJ17	AC009032.1	0.828246128	3.59E-136	postive
TRAJ18	AC009032.1	0.828032798	4.84E-136	postive
TRAJ21	AC009032.1	0.900447264	7.59E-195	postive
TRAJ31	AC009032.1	0.910519339	1.39E-206	postive

TRAJ37	AC009032.1	0.941536706	5.37E-254	postive
TRAJ38	AC009032.1	0.924951748	4.45E-226	postive
TRAJ39	AC009032.1	0.911520463	7.94E-208	postive
CREB1	AL353804.2	0.72088993	6.41E-87	postive
UBR1	AL353804.2	0.601388102	6.35E-54	postive
CYLD	AL353804.2	0.640519171	4.08E-63	postive
IREB2	AL353804.2	0.525908008	2.20E-39	postive
DDX17	AL353804.2	0.558893332	2.79E-45	postive
JAK2	AL353804.2	0.635071549	9.34E-62	postive
NFAT5	AL353804.2	0.890117167	4.79E-184	postive
NFATC3	AL353804.2	0.58050159	1.62E-49	postive
PIK3R1	AL353804.2	0.552163031	5.04E-44	postive
PIK3CA	AL353804.2	0.603227922	2.51E-54	postive
GNRH1	AL353804.2	0.655153062	6.59E-67	postive
IL6ST	AL353804.2	0.562165432	6.67E-46	postive
RABEP1	AL353804.2	0.506658698	3.13E-36	postive
ACVR2A	AL353804.2	0.563990659	2.98E-46	postive
ANGPTL1	AL353804.2	0.610497401	5.99E-56	postive
BMPR1A	AL353804.2	0.501167222	2.29E-35	postive
BMPR2	AL353804.2	0.638242187	1.52E-62	postive
CRLF3	AL353804.2	0.589271293	2.50E-51	postive
NR1D2	AL353804.2	0.506114249	3.82E-36	postive
NR2C2	AL353804.2	0.60875853	1.48E-55	postive
RORA	AL353804.2	0.806857895	6.13E-124	postive
SOS1	AL353804.2	0.592383575	5.52E-52	postive
SOS2	AL353804.2	0.500784948	2.62E-35	postive
BRAF	AL353804.2	0.659688716	3.98E-68	postive
CBLB	AL353804.2	0.658998494	6.12E-68	postive
TRAJ1	AL353804.2	0.722846015	1.33E-87	postive
TRAJ2	AL353804.2	0.804740526	8.22E-123	postive
TRAJ3	AL353804.2	0.763530156	2.67E-103	postive
TRAJ5	AL353804.2	0.696822902	5.82E-79	postive
TRAJ6	AL353804.2	0.811811001	1.24E-126	postive
TRAJ8	AL353804.2	0.706583478	4.32E-82	postive
TRAJ10	AL353804.2	0.805677614	2.62E-123	postive
TRAJ12	AL353804.2	0.722332724	2.01E-87	postive
TRAJ13	AL353804.2	0.729109086	7.84E-90	postive
TRAJ14	AL353804.2	0.734338227	9.65E-92	postive
TRAJ16	AL353804.2	0.808112076	1.30E-124	postive
TRAJ17	AL353804.2	0.687357605	4.80E-76	postive
TRAJ18	AL353804.2	0.785263923	4.72E-113	postive
TRAJ21	AL353804.2	0.756479182	2.33E-100	postive
TRAJ31	AL353804.2	0.794862596	9.93E-118	postive
TRAJ37	AL353804.2	0.828338566	3.15E-136	postive
TRAJ38	AL353804.2	0.857432804	6.55E-156	postive
TRAJ39	AL353804.2	0.771427691	1.02E-106	postive
CREB1	AL354696.1	0.623401486	6.20E-59	postive
RFXAP	AL354696.1	0.545521101	8.23E-43	postive
DDX17	AL354696.1	0.530431973	3.73E-40	postive
NFAT5	AL354696.1	0.645812031	1.83E-64	postive
GNRH1	AL354696.1	0.682640954	1.24E-74	postive
ACVR2A	AL354696.1	0.568018103	4.95E-47	postive
ANGPTL1	AL354696.1	0.595501201	1.20E-52	postive
BMPR2	AL354696.1	0.554007876	2.30E-44	postive
CRLF3	AL354696.1	0.560165079	1.60E-45	postive
NR2C2	AL354696.1	0.553428385	2.94E-44	postive
RORA	AL354696.1	0.638712511	1.16E-62	postive
SOS1	AL354696.1	0.509060281	1.30E-36	postive
BRAF	AL354696.1	0.626811861	9.55E-60	postive
CBLB	AL354696.1	0.540367123	6.89E-42	postive
PDK1	AL354696.1	0.53420613	8.32E-41	postive
TRAJ1	AL354696.1	0.57715054	7.71E-49	postive
TRAJ2	AL354696.1	0.573594562	3.96E-48	postive
TRAJ3	AL354696.1	0.595815965	1.02E-52	postive
TRAJ5	AL354696.1	0.610667921	5.48E-56	postive

TRAJ6	AL354696.1	0.618882881	7.14E-58	postive
TRAJ8	AL354696.1	0.590930282	1.12E-51	postive
TRAJ10	AL354696.1	0.601520178	5.94E-54	postive
TRAJ12	AL354696.1	0.560826654	1.20E-45	postive
TRAJ13	AL354696.1	0.591241938	9.63E-52	postive
TRAJ14	AL354696.1	0.587251129	6.61E-51	postive
TRAJ16	AL354696.1	0.617730279	1.32E-57	postive
TRAJ17	AL354696.1	0.573016574	5.16E-48	postive
TRAJ18	AL354696.1	0.553586036	2.75E-44	postive
TRAJ21	AL354696.1	0.618993684	6.72E-58	postive
TRAJ31	AL354696.1	0.65588483	4.20E-67	postive
TRAJ37	AL354696.1	0.633637909	2.11E-61	postive
TRAJ38	AL354696.1	0.673059265	7.61E-72	postive
TRAJ39	AL354696.1	0.639206801	8.73E-63	postive
DDX17	AL121772.3	0.589975208	1.78E-51	postive
TXK	AL121772.3	0.547500052	3.61E-43	postive
NFAT5	AL121772.3	0.575878381	1.39E-48	postive
GNRH1	AL121772.3	0.70561962	8.91E-82	postive
NR2C2	AL121772.3	0.653834797	1.48E-66	postive
RORA	AL121772.3	0.586042885	1.18E-50	postive
BRAF	AL121772.3	0.553551664	2.79E-44	postive
TEC	AL121772.3	0.574641449	2.45E-48	postive
CBLB	AL121772.3	0.553574508	2.76E-44	postive
GIPR	Z84492.1	0.536882202	2.84E-41	postive
IL11RA	Z84492.1	0.551375816	7.05E-44	postive
GNRH1	AC011481.1	0.56562148	1.45E-46	postive
CREB1	AC138932.6	0.793705305	3.75E-117	postive
UBR1	AC138932.6	0.66426445	2.23E-69	postive
ZC3HAV1	AC138932.6	0.560991046	1.12E-45	postive
ZC3HAV1L	AC138932.6	0.501340525	2.15E-35	postive
IL15	AC138932.6	0.551118899	7.86E-44	postive
CYLD	AC138932.6	0.620364853	3.22E-58	postive
EIF2AK2	AC138932.6	0.569506785	2.54E-47	postive
MAPK8	AC138932.6	0.561591947	8.58E-46	postive
TLR1	AC138932.6	0.504357104	7.24E-36	postive
NFKBIZ	AC138932.6	0.531728115	2.24E-40	postive
LMBR1	AC138932.6	0.574605872	2.49E-48	postive
LIMS1	AC138932.6	0.538447761	1.51E-41	postive
IREB2	AC138932.6	0.618852688	7.25E-58	postive
DDX17	AC138932.6	0.640048688	5.36E-63	postive
PIK3CG	AC138932.6	0.565779759	1.35E-46	postive
JAK2	AC138932.6	0.681593292	2.53E-74	postive
TXK	AC138932.6	0.635754279	6.33E-62	postive
NFAT5	AC138932.6	0.804244788	1.50E-122	postive
MALT1	AC138932.6	0.546297076	5.96E-43	postive
PIK3R1	AC138932.6	0.657798943	1.29E-67	postive
PIK3CA	AC138932.6	0.717058003	1.35E-85	postive
PIK3CB	AC138932.6	0.548251494	2.63E-43	postive
RASGRP3	AC138932.6	0.563163847	4.30E-46	postive
PLXNC1	AC138932.6	0.510840647	6.73E-37	postive
GNRH1	AC138932.6	0.767887396	3.61E-105	postive
IL6ST	AC138932.6	0.645566288	2.12E-64	postive
RABEP1	AC138932.6	0.552726261	3.97E-44	postive
ACVR2A	AC138932.6	0.647468987	6.85E-65	postive
ANGPTL1	AC138932.6	0.772450465	3.59E-107	postive
BMPR1A	AC138932.6	0.547038735	4.37E-43	postive
BMPR2	AC138932.6	0.755712331	4.81E-100	postive
CRLF3	AC138932.6	0.638562371	1.27E-62	postive
NR1D2	AC138932.6	0.549648826	1.46E-43	postive
NR2C1	AC138932.6	0.513557389	2.45E-37	postive
NR2C2	AC138932.6	0.724378753	3.83E-88	postive
RORA	AC138932.6	0.850400962	8.98E-151	postive
SOS1	AC138932.6	0.687910527	3.27E-76	postive
SOS2	AC138932.6	0.574286424	2.89E-48	postive
BRAF	AC138932.6	0.796519633	1.46E-118	postive

ITK	AC138932.6	0.51545259	1.20E-37	postive
TEC	AC138932.6	0.51238906	3.79E-37	postive
CBL	AC138932.6	0.590294277	1.53E-51	postive
CBLB	AC138932.6	0.804589315	9.88E-123	postive
RASGRP1	AC138932.6	0.510048214	9.02E-37	postive
PDK1	AC138932.6	0.618619871	8.22E-58	postive
TRAJ1	AC138932.6	0.653858897	1.45E-66	postive
TRAJ2	AC138932.6	0.667174126	3.48E-70	postive
TRAJ3	AC138932.6	0.688130996	2.80E-76	postive
TRAJ5	AC138932.6	0.734782587	6.61E-92	postive
TRAJ6	AC138932.6	0.704654086	1.84E-81	postive
TRAJ8	AC138932.6	0.643531452	7.03E-64	postive
TRAJ10	AC138932.6	0.682759046	1.14E-74	postive
TRAJ12	AC138932.6	0.644551751	3.86E-64	postive
TRAJ13	AC138932.6	0.686044098	1.19E-75	postive
TRAJ14	AC138932.6	0.671114771	2.72E-71	postive
TRAJ16	AC138932.6	0.707756676	1.78E-82	postive
TRAJ17	AC138932.6	0.642606861	1.21E-63	postive
TRAJ18	AC138932.6	0.658769471	7.06E-68	postive
TRAJ21	AC138932.6	0.716714718	1.77E-85	postive
TRAJ31	AC138932.6	0.759475779	1.35E-101	postive
TRAJ37	AC138932.6	0.708083621	1.39E-82	postive
TRAJ38	AC138932.6	0.806970507	5.34E-124	postive
TRAJ39	AC138932.6	0.73238989	5.03E-91	postive
PDGFRA	AP001434.1	0.549331541	1.67E-43	postive
FGF7	AP001434.1	0.504263292	7.49E-36	postive
INHBA	AP001434.1	0.55688785	6.66E-45	postive
CREB1	AC090425.3	0.717775244	7.65E-86	postive
RFXAP	AC090425.3	0.556949744	6.48E-45	postive
UBR1	AC090425.3	0.56120597	1.02E-45	postive
ZC3HAV1	AC090425.3	0.548490872	2.38E-43	postive
ZC3HAV1L	AC090425.3	0.513345643	2.65E-37	postive
CYLD	AC090425.3	0.543736833	1.72E-42	postive
EIF2AK2	AC090425.3	0.53650009	3.31E-41	postive
MAPK8	AC090425.3	0.580553326	1.58E-49	postive
LMBR1	AC090425.3	0.502278973	1.53E-35	postive
IREB2	AC090425.3	0.572499339	6.53E-48	postive
DDX17	AC090425.3	0.62452299	3.36E-59	postive
JAK2	AC090425.3	0.57646658	1.06E-48	postive
TXK	AC090425.3	0.536558989	3.23E-41	postive
NFAT5	AC090425.3	0.778610957	5.99E-110	postive
MALT1	AC090425.3	0.519924972	2.21E-38	postive
PIK3R1	AC090425.3	0.528530636	7.90E-40	postive
PIK3CA	AC090425.3	0.707000303	3.15E-82	postive
PIK3CB	AC090425.3	0.530216335	4.07E-40	postive
GSK3B	AC090425.3	0.517082471	6.51E-38	postive
GNRH1	AC090425.3	0.777355859	2.24E-109	postive
IL6ST	AC090425.3	0.516547926	7.97E-38	postive
RABEP1	AC090425.3	0.542968709	2.37E-42	postive
ACVR2A	AC090425.3	0.607718639	2.52E-55	postive
ANGPTL1	AC090425.3	0.682772871	1.13E-74	postive
BMPR1A	AC090425.3	0.511050134	6.23E-37	postive
BMPR2	AC090425.3	0.62827526	4.25E-60	postive
CRLF3	AC090425.3	0.613898118	1.01E-56	postive
NR2C1	AC090425.3	0.569131213	3.00E-47	postive
NR2C2	AC090425.3	0.723139478	1.05E-87	postive
RORA	AC090425.3	0.75240365	1.05E-98	postive
SOS1	AC090425.3	0.675856159	1.20E-72	postive
SOS2	AC090425.3	0.542970653	2.37E-42	postive
BRAF	AC090425.3	0.72351523	7.73E-88	postive
CBL	AC090425.3	0.600572098	9.57E-54	postive
CBLB	AC090425.3	0.668207186	1.79E-70	postive
PDK1	AC090425.3	0.575929003	1.36E-48	postive
TRAJ1	AC090425.3	0.686539108	8.48E-76	postive
TRAJ2	AC090425.3	0.622890891	8.19E-59	postive

TRAJ3	AC090425.3	0.677417168	4.23E-73	postive
TRAJ5	AC090425.3	0.718630433	3.89E-86	postive
TRAJ6	AC090425.3	0.705576229	9.20E-82	postive
TRAJ8	AC090425.3	0.667334787	3.14E-70	postive
TRAJ10	AC090425.3	0.701167844	2.44E-80	postive
TRAJ12	AC090425.3	0.612085889	2.61E-56	postive
TRAJ13	AC090425.3	0.690941972	3.89E-77	postive
TRAJ14	AC090425.3	0.658450973	8.61E-68	postive
TRAJ16	AC090425.3	0.721558271	3.75E-87	postive
TRAJ17	AC090425.3	0.68220334	1.67E-74	postive
TRAJ18	AC090425.3	0.641136935	2.85E-63	postive
TRAJ21	AC090425.3	0.709477168	4.81E-83	postive
TRAJ31	AC090425.3	0.737435767	6.79E-93	postive
TRAJ37	AC090425.3	0.708922997	7.34E-83	postive
TRAJ38	AC090425.3	0.746762653	1.82E-96	postive
TRAJ39	AC090425.3	0.733323703	2.28E-91	postive
MAVS	MIR600HG	0.508682341	1.49E-36	postive
DDX17	MIR600HG	0.599731999	1.46E-53	postive
LTB4R2	MIR600HG	0.546266541	6.04E-43	postive
GNRH1	MIR600HG	0.560657052	1.29E-45	postive
IL11RA	MIR600HG	0.520473859	1.79E-38	postive
NR2C2	MIR600HG	0.623762883	5.09E-59	postive
TYK2	AL022328.1	0.538693511	1.36E-41	postive
DDX17	AL022328.1	0.501571255	1.98E-35	postive
IRF9	AL022328.1	0.561344712	9.57E-46	postive
LTB4R2	AL022328.1	0.529708909	4.97E-40	postive
GIPR	AL022328.1	0.519711744	2.40E-38	postive
IL11RA	AL022328.1	0.515404684	1.23E-37	postive
CREB1	ADAMTSL4-AS1	0.725620778	1.39E-88	postive
UBR1	ADAMTSL4-AS1	0.588391311	3.82E-51	postive
ZC3HAV1	ADAMTSL4-AS1	0.561334242	9.61E-46	postive
IL15	ADAMTSL4-AS1	0.617622795	1.40E-57	postive
CYLD	ADAMTSL4-AS1	0.592943711	4.20E-52	postive
EIF2AK2	ADAMTSL4-AS1	0.560443254	1.42E-45	postive
NFKBIZ	ADAMTSL4-AS1	0.502812131	1.27E-35	postive
LMBR1	ADAMTSL4-AS1	0.536790445	2.95E-41	postive
LIMS1	ADAMTSL4-AS1	0.513430003	2.57E-37	postive
IREB2	ADAMTSL4-AS1	0.542984467	2.35E-42	postive
DDX17	ADAMTSL4-AS1	0.553728484	2.59E-44	postive
PIK3CG	ADAMTSL4-AS1	0.555374059	1.28E-44	postive
JAK2	ADAMTSL4-AS1	0.645479802	2.23E-64	postive
TXK	ADAMTSL4-AS1	0.559985239	1.73E-45	postive
NFAT5	ADAMTSL4-AS1	0.763647695	2.38E-103	postive
MALT1	ADAMTSL4-AS1	0.536952851	2.76E-41	postive
PIK3R1	ADAMTSL4-AS1	0.600485396	1.00E-53	postive
PIK3CA	ADAMTSL4-AS1	0.713622182	1.98E-84	postive
PIK3CB	ADAMTSL4-AS1	0.557133516	5.99E-45	postive
RASGRP3	ADAMTSL4-AS1	0.545092533	9.84E-43	postive
IGHD6-6	ADAMTSL4-AS1	0.52097267	1.48E-38	postive
PLXNC1	ADAMTSL4-AS1	0.516792837	7.27E-38	postive
GNRH1	ADAMTSL4-AS1	0.652019847	4.45E-66	postive
IL6ST	ADAMTSL4-AS1	0.588054107	4.50E-51	postive
IL7	ADAMTSL4-AS1	0.563270774	4.10E-46	postive
ACVR2A	ADAMTSL4-AS1	0.583339707	4.26E-50	postive
ANGPTL1	ADAMTSL4-AS1	0.744753455	1.10E-95	postive
BMPR2	ADAMTSL4-AS1	0.657956483	1.17E-67	postive
CRLF3	ADAMTSL4-AS1	0.597613436	4.20E-53	postive
IL18R1	ADAMTSL4-AS1	0.517271752	6.06E-38	postive
NR2C2	ADAMTSL4-AS1	0.686805422	7.05E-76	postive
RORA	ADAMTSL4-AS1	0.772895239	2.28E-107	postive
SOS1	ADAMTSL4-AS1	0.643285899	8.12E-64	postive
SOS2	ADAMTSL4-AS1	0.580755644	1.44E-49	postive
BRAF	ADAMTSL4-AS1	0.722369416	1.95E-87	postive
ITK	ADAMTSL4-AS1	0.510860765	6.68E-37	postive
CBL	ADAMTSL4-AS1	0.578714516	3.73E-49	postive

CBLB	ADAMTSL4-AS1	0.665522695	1.00E-69	postive
RASGRP1	ADAMTSL4-AS1	0.543574835	1.84E-42	postive
PDK1	ADAMTSL4-AS1	0.575859008	1.40E-48	postive
TRAJ1	ADAMTSL4-AS1	0.769106157	1.07E-105	postive
TRAJ2	ADAMTSL4-AS1	0.638348842	1.43E-62	postive
TRAJ3	ADAMTSL4-AS1	0.783742597	2.47E-112	postive
TRAJ5	ADAMTSL4-AS1	0.846923483	2.49E-148	postive
TRAJ6	ADAMTSL4-AS1	0.793087342	7.60E-117	postive
TRAJ8	ADAMTSL4-AS1	0.775791707	1.15E-108	postive
TRAJ10	ADAMTSL4-AS1	0.812815599	3.46E-127	postive
TRAJ12	ADAMTSL4-AS1	0.730964823	1.67E-90	postive
TRAJ13	ADAMTSL4-AS1	0.788224002	1.81E-114	postive
TRAJ14	ADAMTSL4-AS1	0.788974862	7.85E-115	postive
TRAJ16	ADAMTSL4-AS1	0.839278002	3.65E-143	postive
TRAJ17	ADAMTSL4-AS1	0.85003702	1.63E-150	postive
TRAJ18	ADAMTSL4-AS1	0.776621202	4.83E-109	postive
TRAJ21	ADAMTSL4-AS1	0.838311352	1.57E-142	postive
TRAJ31	ADAMTSL4-AS1	0.832338355	1.05E-138	postive
TRAJ37	ADAMTSL4-AS1	0.773583774	1.13E-107	postive
TRAJ38	ADAMTSL4-AS1	0.841987969	5.79E-145	postive
TRAJ39	ADAMTSL4-AS1	0.865941201	1.66E-162	postive
DDX17	RAD51-AS1	0.691108237	3.46E-77	postive
IRF9	RAD51-AS1	0.534192087	8.37E-41	postive
LTB4R2	RAD51-AS1	0.503486276	9.92E-36	postive
GNRH1	RAD51-AS1	0.66704535	3.78E-70	postive
IL11RA	RAD51-AS1	0.51296567	3.06E-37	postive
NR2C1	RAD51-AS1	0.533540927	1.09E-40	postive
NR2C2	RAD51-AS1	0.638133278	1.62E-62	postive
NR2C2	AC018521.6	0.531577987	2.37E-40	postive
CREB1	AC087392.1	0.695307811	1.73E-78	postive
UBR1	AC087392.1	0.5986194	2.54E-53	postive
CYLD	AC087392.1	0.633518323	2.25E-61	postive
IREB2	AC087392.1	0.505857084	4.20E-36	postive
DDX17	AC087392.1	0.539408526	1.02E-41	postive
JAK2	AC087392.1	0.585428468	1.58E-50	postive
NFAT5	AC087392.1	0.894344442	2.49E-188	postive
NFATC3	AC087392.1	0.676790877	6.43E-73	postive
PIK3R1	AC087392.1	0.571503432	1.03E-47	postive
PIK3CA	AC087392.1	0.577903753	5.44E-49	postive
GHRL	AC087392.1	0.52203781	9.85E-39	postive
GNRH1	AC087392.1	0.677492023	4.02E-73	postive
IL6ST	AC087392.1	0.56807367	4.83E-47	postive
RABEP1	AC087392.1	0.556653149	7.37E-45	postive
ACVR2A	AC087392.1	0.588052344	4.50E-51	postive
ANGPTL1	AC087392.1	0.605594872	7.51E-55	postive
BMPR1A	AC087392.1	0.527036054	1.42E-39	postive
BMPR2	AC087392.1	0.609118449	1.22E-55	postive
CRLF3	AC087392.1	0.601772281	5.23E-54	postive
NR2C2	AC087392.1	0.599186831	1.92E-53	postive
RORA	AC087392.1	0.80209123	2.02E-121	postive
SOS1	AC087392.1	0.550323388	1.10E-43	postive
BRAF	AC087392.1	0.651399585	6.48E-66	postive
CBL	AC087392.1	0.510085844	8.90E-37	postive
CBLB	AC087392.1	0.541466796	4.39E-42	postive
TRAJ1	AC087392.1	0.74345025	3.52E-95	postive
TRAJ2	AC087392.1	0.74643239	2.45E-96	postive
TRAJ3	AC087392.1	0.784577184	9.99E-113	postive
TRAJ5	AC087392.1	0.736746039	1.23E-92	postive
TRAJ6	AC087392.1	0.819296223	7.42E-131	postive
TRAJ8	AC087392.1	0.750947523	4.04E-98	postive
TRAJ10	AC087392.1	0.822456934	1.07E-132	postive
TRAJ12	AC087392.1	0.708216216	1.26E-82	postive
TRAJ13	AC087392.1	0.775351905	1.81E-108	postive
TRAJ14	AC087392.1	0.746666453	1.98E-96	postive
TRAJ16	AC087392.1	0.799076399	7.31E-120	postive

TRAJ17	AC087392.1	0.743706826	2.80E-95	postive
TRAJ18	AC087392.1	0.760070374	7.64E-102	postive
TRAJ21	AC087392.1	0.794317487	1.86E-117	postive
TRAJ31	AC087392.1	0.804921681	6.59E-123	postive
TRAJ37	AC087392.1	0.847242611	1.50E-148	postive
TRAJ38	AC087392.1	0.825804267	1.08E-134	postive
TRAJ39	AC087392.1	0.819961158	3.06E-131	postive
BMPR1B	LINC00662	0.587641694	5.48E-51	postive
CREB1	LINC01376	0.619686321	4.64E-58	postive
UBR1	LINC01376	0.551351565	7.12E-44	postive
EIF2AK2	LINC01376	0.50832565	1.70E-36	postive
MAPK8	LINC01376	0.52131724	1.30E-38	postive
IREB2	LINC01376	0.531027983	2.95E-40	postive
NFAT5	LINC01376	0.624883882	2.76E-59	postive
PIK3CA	LINC01376	0.546420658	5.66E-43	postive
GNRH1	LINC01376	0.588694374	3.30E-51	postive
ACVR2A	LINC01376	0.54279949	2.54E-42	postive
ANGPTL1	LINC01376	0.53113395	2.83E-40	postive
BMPR1A	LINC01376	0.525252698	2.84E-39	postive
BMPR2	LINC01376	0.573324149	4.48E-48	postive
NR2C2	LINC01376	0.631062646	8.98E-61	postive
RORA	LINC01376	0.57933235	2.80E-49	postive
SOS1	LINC01376	0.648249458	4.30E-65	postive
BRAF	LINC01376	0.64894178	2.84E-65	postive
CBLB	LINC01376	0.521840895	1.06E-38	postive
TRAJ1	LINC01376	0.542394777	3.00E-42	postive
TRAJ3	LINC01376	0.557170655	5.89E-45	postive
TRAJ5	LINC01376	0.584032223	3.07E-50	postive
TRAJ6	LINC01376	0.571797391	8.99E-48	postive
TRAJ8	LINC01376	0.568870583	3.38E-47	postive
TRAJ10	LINC01376	0.55095131	8.43E-44	postive
TRAJ12	LINC01376	0.565835303	1.31E-46	postive
TRAJ13	LINC01376	0.57131134	1.12E-47	postive
TRAJ14	LINC01376	0.56232203	6.23E-46	postive
TRAJ16	LINC01376	0.583117118	4.73E-50	postive
TRAJ17	LINC01376	0.570779655	1.43E-47	postive
TRAJ18	LINC01376	0.514468677	1.74E-37	postive
TRAJ21	LINC01376	0.589931467	1.82E-51	postive
TRAJ31	LINC01376	0.618423285	9.13E-58	postive
TRAJ37	LINC01376	0.574648806	2.44E-48	postive
TRAJ38	LINC01376	0.629886732	1.73E-60	postive
TRAJ39	LINC01376	0.592467112	5.30E-52	postive
CREB1	AC007038.1	0.749234082	1.94E-97	postive
RFXAP	AC007038.1	0.582824299	5.43E-50	postive
UBR1	AC007038.1	0.618772632	7.57E-58	postive
ZC3HAV1	AC007038.1	0.542779375	2.56E-42	postive
ZC3HAV1L	AC007038.1	0.540094097	7.70E-42	postive
IL15	AC007038.1	0.565095434	1.83E-46	postive
CYLD	AC007038.1	0.548590649	2.28E-43	postive
EIF2AK2	AC007038.1	0.509249116	1.21E-36	postive
MAPK8	AC007038.1	0.573212033	4.72E-48	postive
NFKBIZ	AC007038.1	0.524911993	3.24E-39	postive
LMBR1	AC007038.1	0.521828066	1.07E-38	postive
IREB2	AC007038.1	0.598382743	2.86E-53	postive
DDX17	AC007038.1	0.680586142	5.01E-74	postive
JAK2	AC007038.1	0.589893104	1.85E-51	postive
TXK	AC007038.1	0.565642275	1.43E-46	postive
NFAT5	AC007038.1	0.813091685	2.43E-127	postive
MALT1	AC007038.1	0.521000445	1.47E-38	postive
PIK3R1	AC007038.1	0.567195442	7.16E-47	postive
PIK3CA	AC007038.1	0.610811616	5.08E-56	postive
AKT3	AC007038.1	0.537411749	2.29E-41	postive
RASGRP3	AC007038.1	0.505843909	4.22E-36	postive
IGHD4-4	AC007038.1	0.529560387	5.27E-40	postive
IGHD6-6	AC007038.1	0.514118545	1.99E-37	postive

LTB4R2	AC007038.1	0.527772147	1.06E-39	postive
GNRH1	AC007038.1	0.839747716	1.79E-143	postive
IL6ST	AC007038.1	0.575510876	1.64E-48	postive
RABEP1	AC007038.1	0.552569561	4.24E-44	postive
ACVR2A	AC007038.1	0.645785731	1.86E-64	postive
ANGPTL1	AC007038.1	0.723734978	6.47E-88	postive
BMPR1A	AC007038.1	0.547287505	3.94E-43	postive
BMPR2	AC007038.1	0.675999174	1.09E-72	postive
CRLF3	AC007038.1	0.663024689	4.90E-69	postive
NR2C1	AC007038.1	0.579589981	2.48E-49	postive
NR2C2	AC007038.1	0.746080978	3.36E-96	postive
RORA	AC007038.1	0.790336567	1.71E-115	postive
SOS1	AC007038.1	0.65744111	1.61E-67	postive
SOS2	AC007038.1	0.547763539	3.23E-43	postive
BRAF	AC007038.1	0.795893942	3.02E-118	postive
CBL	AC007038.1	0.574499507	2.62E-48	postive
CBLB	AC007038.1	0.704943284	1.48E-81	postive
PDK1	AC007038.1	0.605199899	9.19E-55	postive
TRAJ1	AC007038.1	0.698980082	1.21E-79	postive
TRAJ2	AC007038.1	0.73943745	1.20E-93	postive
TRAJ3	AC007038.1	0.761854634	1.37E-102	postive
TRAJ5	AC007038.1	0.723432012	8.27E-88	postive
TRAJ6	AC007038.1	0.749958168	1.00E-97	postive
TRAJ8	AC007038.1	0.728885448	9.44E-90	postive
TRAJ10	AC007038.1	0.722645259	1.56E-87	postive
TRAJ12	AC007038.1	0.64427755	4.54E-64	postive
TRAJ13	AC007038.1	0.723507836	7.78E-88	postive
TRAJ14	AC007038.1	0.714597868	9.27E-85	postive
TRAJ16	AC007038.1	0.735139386	4.88E-92	postive
TRAJ17	AC007038.1	0.680021272	7.35E-74	postive
TRAJ18	AC007038.1	0.646778517	1.03E-64	postive
TRAJ21	AC007038.1	0.741461673	2.04E-94	postive
TRAJ31	AC007038.1	0.76694248	9.26E-105	postive
TRAJ37	AC007038.1	0.760835289	3.66E-102	postive
TRAJ38	AC007038.1	0.809461075	2.41E-125	postive
TRAJ39	AC007038.1	0.775338746	1.84E-108	postive
CREB1	AC020978.3	0.633923705	1.79E-61	postive
UBR1	AC020978.3	0.546326739	5.89E-43	postive
CYLD	AC020978.3	0.62907424	2.73E-60	postive
DDX17	AC020978.3	0.543014939	2.32E-42	postive
JAK2	AC020978.3	0.517509451	5.54E-38	postive
NFAT5	AC020978.3	0.925239381	1.66E-226	postive
NFATC3	AC020978.3	0.719395595	2.12E-86	postive
GNRH1	AC020978.3	0.65359444	1.71E-66	postive
IL6ST	AC020978.3	0.51074913	6.96E-37	postive
ACVR2A	AC020978.3	0.55645769	8.02E-45	postive
BMPR2	AC020978.3	0.546654332	5.13E-43	postive
CRLF3	AC020978.3	0.518681258	3.55E-38	postive
NR2C2	AC020978.3	0.555358493	1.29E-44	postive
RORA	AC020978.3	0.768797981	1.45E-105	postive
SOS1	AC020978.3	0.506826633	2.95E-36	postive
BRAF	AC020978.3	0.585613022	1.45E-50	postive
TRAJ1	AC020978.3	0.79368141	3.85E-117	postive
TRAJ2	AC020978.3	0.800217462	1.89E-120	postive
TRAJ3	AC020978.3	0.816137753	4.75E-129	postive
TRAJ5	AC020978.3	0.737283793	7.74E-93	postive
TRAJ6	AC020978.3	0.872547641	5.97E-168	postive
TRAJ8	AC020978.3	0.796295341	1.89E-118	postive
TRAJ10	AC020978.3	0.874056784	3.09E-169	postive
TRAJ12	AC020978.3	0.790254177	1.88E-115	postive
TRAJ13	AC020978.3	0.819496626	5.69E-131	postive
TRAJ14	AC020978.3	0.790455025	1.50E-115	postive
TRAJ16	AC020978.3	0.84461339	9.70E-147	postive
TRAJ17	AC020978.3	0.777705101	1.55E-109	postive
TRAJ18	AC020978.3	0.842996037	1.22E-145	postive

TRAJ21	AC020978.3	0.829358713	7.45E-137	postive
TRAJ31	AC020978.3	0.844700795	8.46E-147	postive
TRAJ37	AC020978.3	0.922889714	4.60E-223	postive
TRAJ38	AC020978.3	0.841657061	9.64E-145	postive
TRAJ39	AC020978.3	0.837949002	2.70E-142	postive
CREB1	AC037487.2	0.739394076	1.24E-93	postive
UBR1	AC037487.2	0.563324566	4.00E-46	postive
ZC3HAV1	AC037487.2	0.515424692	1.22E-37	postive
IL15	AC037487.2	0.585745272	1.36E-50	postive
CYLD	AC037487.2	0.558578334	3.20E-45	postive
EIF2AK2	AC037487.2	0.511425386	5.42E-37	postive
MAPK8	AC037487.2	0.519202361	2.91E-38	postive
TLR1	AC037487.2	0.555342165	1.30E-44	postive
NFKBIZ	AC037487.2	0.531022216	2.96E-40	postive
LMBR1	AC037487.2	0.572864891	5.53E-48	postive
LIMS1	AC037487.2	0.547384803	3.78E-43	postive
IREB2	AC037487.2	0.546430676	5.64E-43	postive
DDX17	AC037487.2	0.515069943	1.39E-37	postive
PIK3CG	AC037487.2	0.537133379	2.56E-41	postive
JAK2	AC037487.2	0.670704424	3.55E-71	postive
TXK	AC037487.2	0.536362881	3.50E-41	postive
NFAT5	AC037487.2	0.764990741	6.38E-104	postive
MALT1	AC037487.2	0.524777224	3.42E-39	postive
PIK3R1	AC037487.2	0.570985522	1.30E-47	postive
PIK3CA	AC037487.2	0.697271046	4.20E-79	postive
PIK3CB	AC037487.2	0.515637683	1.12E-37	postive
RASGRP3	AC037487.2	0.532308792	1.77E-40	postive
GNRH1	AC037487.2	0.655201186	6.40E-67	postive
IL6ST	AC037487.2	0.567554141	6.10E-47	postive
RABEP1	AC037487.2	0.509629552	1.05E-36	postive
ACVR2A	AC037487.2	0.538836213	1.29E-41	postive
ANGPTL1	AC037487.2	0.784269181	1.40E-112	postive
BMPR2	AC037487.2	0.658798863	6.93E-68	postive
CRLF3	AC037487.2	0.641937814	1.79E-63	postive
NR2C2	AC037487.2	0.611426502	3.69E-56	postive
RORA	AC037487.2	0.771801133	6.97E-107	postive
SOS1	AC037487.2	0.640630546	3.83E-63	postive
SOS2	AC037487.2	0.549779096	1.38E-43	postive
BRAF	AC037487.2	0.716839352	1.60E-85	postive
CBL	AC037487.2	0.594599503	1.86E-52	postive
CBLB	AC037487.2	0.75510705	8.49E-100	postive
PDK1	AC037487.2	0.621087015	2.18E-58	postive
TRAJ1	AC037487.2	0.685102934	2.29E-75	postive
TRAJ2	AC037487.2	0.637038881	3.04E-62	postive
TRAJ3	AC037487.2	0.689083045	1.44E-76	postive
TRAJ5	AC037487.2	0.786354849	1.43E-113	postive
TRAJ6	AC037487.2	0.736925932	1.05E-92	postive
TRAJ8	AC037487.2	0.678849128	1.62E-73	postive
TRAJ10	AC037487.2	0.745285632	6.86E-96	postive
TRAJ12	AC037487.2	0.624744461	2.98E-59	postive
TRAJ13	AC037487.2	0.741848167	1.45E-94	postive
TRAJ14	AC037487.2	0.718086261	5.98E-86	postive
TRAJ16	AC037487.2	0.752562502	9.09E-99	postive
TRAJ17	AC037487.2	0.703871914	3.29E-81	postive
TRAJ18	AC037487.2	0.678015202	2.83E-73	postive
TRAJ21	AC037487.2	0.763946121	1.78E-103	postive
TRAJ31	AC037487.2	0.783917889	2.05E-112	postive
TRAJ37	AC037487.2	0.729511152	5.61E-90	postive
TRAJ38	AC037487.2	0.815941527	6.13E-129	postive
TRAJ39	AC037487.2	0.774078918	6.76E-108	postive
CREB1	AC026202.2	0.741792988	1.52E-94	postive
RFXAP	AC026202.2	0.51339201	2.61E-37	postive
UBR1	AC026202.2	0.623580142	5.63E-59	postive
IL15	AC026202.2	0.572219453	7.42E-48	postive
CYLD	AC026202.2	0.58235875	6.77E-50	postive

EIF2AK2	AC026202.2	0.502888682	1.23E-35	postive
MAPK8	AC026202.2	0.535675736	4.62E-41	postive
LMBR1	AC026202.2	0.504038348	8.13E-36	postive
IREB2	AC026202.2	0.587868022	4.92E-51	postive
DDX17	AC026202.2	0.597873242	3.69E-53	postive
PIK3CG	AC026202.2	0.556446888	8.05E-45	postive
JAK2	AC026202.2	0.63467358	1.17E-61	postive
TXK	AC026202.2	0.56972745	2.30E-47	postive
PPP3CB	AC026202.2	0.508485855	1.60E-36	postive
NFAT5	AC026202.2	0.754601857	1.36E-99	postive
PIK3R1	AC026202.2	0.59188152	7.05E-52	postive
PIK3CA	AC026202.2	0.626207473	1.33E-59	postive
RASGRP3	AC026202.2	0.563468154	3.76E-46	postive
IGHD6-6	AC026202.2	0.539463494	9.96E-42	postive
PLXNC1	AC026202.2	0.514412319	1.78E-37	postive
GNRH1	AC026202.2	0.706638958	4.14E-82	postive
IL6ST	AC026202.2	0.576025288	1.30E-48	postive
RABEP1	AC026202.2	0.520626798	1.69E-38	postive
ACVR2A	AC026202.2	0.61901103	6.66E-58	postive
ANGPTL1	AC026202.2	0.752290377	1.17E-98	postive
BMPR1A	AC026202.2	0.527169999	1.35E-39	postive
BMPR2	AC026202.2	0.682148023	1.74E-74	postive
CRLF3	AC026202.2	0.621218872	2.03E-58	postive
NR2C2	AC026202.2	0.685511737	1.73E-75	postive
RORA	AC026202.2	0.767773586	4.05E-105	postive
SOS1	AC026202.2	0.659267683	5.18E-68	postive
SOS2	AC026202.2	0.544756174	1.13E-42	postive
BRAF	AC026202.2	0.73397308	1.32E-91	postive
CBL	AC026202.2	0.53236494	1.74E-40	postive
CBLB	AC026202.2	0.707170338	2.77E-82	postive
PDK1	AC026202.2	0.659496015	4.49E-68	postive
TRAJ1	AC026202.2	0.689582453	1.01E-76	postive
TRAJ2	AC026202.2	0.681622013	2.48E-74	postive
TRAJ3	AC026202.2	0.734187434	1.10E-91	postive
TRAJ5	AC026202.2	0.730035402	3.62E-90	postive
TRAJ6	AC026202.2	0.73286708	3.36E-91	postive
TRAJ8	AC026202.2	0.703186058	5.48E-81	postive
TRAJ10	AC026202.2	0.695699315	1.31E-78	postive
TRAJ12	AC026202.2	0.680585604	5.02E-74	postive
TRAJ13	AC026202.2	0.703865504	3.31E-81	postive
TRAJ14	AC026202.2	0.713192551	2.76E-84	postive
TRAJ16	AC026202.2	0.730499957	2.46E-90	postive
TRAJ17	AC026202.2	0.683353251	7.62E-75	postive
TRAJ18	AC026202.2	0.668721804	1.28E-70	postive
TRAJ21	AC026202.2	0.73708958	9.15E-93	postive
TRAJ31	AC026202.2	0.753971817	2.45E-99	postive
TRAJ37	AC026202.2	0.717912363	6.87E-86	postive
TRAJ38	AC026202.2	0.803091236	6.07E-122	postive
TRAJ39	AC026202.2	0.756366427	2.60E-100	postive
CREB1	AP001178.2	0.74974813	1.21E-97	postive
RFXAP	AP001178.2	0.515976275	9.89E-38	postive
UBR1	AP001178.2	0.650638883	1.03E-65	postive
ZC3HAV1	AP001178.2	0.534796655	6.57E-41	postive
ZC3HAV1L	AP001178.2	0.561899522	7.50E-46	postive
IL15	AP001178.2	0.593163766	3.77E-52	postive
CYLD	AP001178.2	0.542498456	2.87E-42	postive
EIF2AK2	AP001178.2	0.513077459	2.93E-37	postive
MAPK8	AP001178.2	0.557482906	5.15E-45	postive
LMBR1	AP001178.2	0.559068756	2.59E-45	postive
IREB2	AP001178.2	0.62171334	1.55E-58	postive
DDX17	AP001178.2	0.619111905	6.31E-58	postive
PIK3CG	AP001178.2	0.510849096	6.71E-37	postive
JAK2	AP001178.2	0.617725735	1.33E-57	postive
TXK	AP001178.2	0.603639102	2.03E-54	postive
NFAT5	AP001178.2	0.799916144	2.71E-120	postive

MALT1	AP001178.2	0.53218058	1.87E-40	postive
PIK3R1	AP001178.2	0.611227671	4.09E-56	postive
PIK3CA	AP001178.2	0.635918628	5.76E-62	postive
AKT3	AP001178.2	0.525454389	2.63E-39	postive
RASGRP3	AP001178.2	0.514401272	1.79E-37	postive
IGHD4-4	AP001178.2	0.547857768	3.10E-43	postive
IGHD6-6	AP001178.2	0.510901146	6.58E-37	postive
GNRH1	AP001178.2	0.777817226	1.38E-109	postive
IL6ST	AP001178.2	0.589416055	2.33E-51	postive
RABEP1	AP001178.2	0.559322991	2.31E-45	postive
ACVR2A	AP001178.2	0.583464328	4.02E-50	postive
ANGPTL1	AP001178.2	0.748904013	2.62E-97	postive
BMPR1A	AP001178.2	0.522180044	9.32E-39	postive
BMPR2	AP001178.2	0.684895902	2.64E-75	postive
CRLF3	AP001178.2	0.632717248	3.54E-61	postive
NR2C2	AP001178.2	0.715477657	4.66E-85	postive
RORA	AP001178.2	0.787260918	5.26E-114	postive
SOS1	AP001178.2	0.654696009	8.72E-67	postive
SOS2	AP001178.2	0.55158037	6.46E-44	postive
BRAF	AP001178.2	0.779463573	2.43E-110	postive
TEC	AP001178.2	0.57309497	4.98E-48	postive
CBL	AP001178.2	0.600422339	1.03E-53	postive
CBLB	AP001178.2	0.747560487	8.86E-97	postive
RASGRP1	AP001178.2	0.506903054	2.87E-36	postive
PDK1	AP001178.2	0.620527461	2.95E-58	postive
TRAJ1	AP001178.2	0.657448193	1.60E-67	postive
TRAJ2	AP001178.2	0.687239931	5.21E-76	postive
TRAJ3	AP001178.2	0.739740187	9.20E-94	postive
TRAJ5	AP001178.2	0.693569791	6.02E-78	postive
TRAJ6	AP001178.2	0.722115857	2.40E-87	postive
TRAJ8	AP001178.2	0.652999697	2.45E-66	postive
TRAJ10	AP001178.2	0.699049756	1.15E-79	postive
TRAJ12	AP001178.2	0.622416998	1.06E-58	postive
TRAJ13	AP001178.2	0.66859979	1.39E-70	postive
TRAJ14	AP001178.2	0.677764006	3.35E-73	postive
TRAJ16	AP001178.2	0.674805528	2.40E-72	postive
TRAJ17	AP001178.2	0.628183578	4.47E-60	postive
TRAJ18	AP001178.2	0.648898959	2.92E-65	postive
TRAJ21	AP001178.2	0.712032845	6.78E-84	postive
TRAJ31	AP001178.2	0.723958451	5.40E-88	postive
TRAJ37	AP001178.2	0.725119271	2.10E-88	postive
TRAJ38	AP001178.2	0.782815619	6.74E-112	postive
TRAJ39	AP001178.2	0.745451801	5.91E-96	postive
CREB1	SCARNA9	0.764565216	9.70E-104	postive
UBR1	SCARNA9	0.714033095	1.44E-84	postive
ZC3HAV1	SCARNA9	0.584165874	2.88E-50	postive
IL15	SCARNA9	0.547818144	3.16E-43	postive
CYLD	SCARNA9	0.650018009	1.49E-65	postive
EIF2AK2	SCARNA9	0.577798917	5.71E-49	postive
MAPK8	SCARNA9	0.527219929	1.32E-39	postive
LMBR1	SCARNA9	0.555077963	1.45E-44	postive
LIMS1	SCARNA9	0.515566165	1.15E-37	postive
IREB2	SCARNA9	0.612048346	2.67E-56	postive
DDX17	SCARNA9	0.597216341	5.12E-53	postive
PIK3CG	SCARNA9	0.601304274	6.62E-54	postive
JAK2	SCARNA9	0.684837186	2.75E-75	postive
TXK	SCARNA9	0.635811966	6.12E-62	postive
NFAT5	SCARNA9	0.829743351	4.32E-137	postive
NFATC3	SCARNA9	0.523118202	6.50E-39	postive
MALT1	SCARNA9	0.532536569	1.62E-40	postive
PIK3R1	SCARNA9	0.658233319	9.85E-68	postive
PIK3CA	SCARNA9	0.675234232	1.81E-72	postive
PIK3CB	SCARNA9	0.521448823	1.23E-38	postive
RASGRP3	SCARNA9	0.525863784	2.24E-39	postive
IGHD6-6	SCARNA9	0.531506618	2.44E-40	postive

PLXNC1	SCARNA9	0.520507548	1.77E-38	postive
GNRH1	SCARNA9	0.706478863	4.67E-82	postive
IL6ST	SCARNA9	0.608955055	1.33E-55	postive
RABEP1	SCARNA9	0.561604961	8.53E-46	postive
ACVR2A	SCARNA9	0.603063062	2.72E-54	postive
ANGPTL1	SCARNA9	0.69936	9.19E-80	postive
BMPR1A	SCARNA9	0.562278726	6.35E-46	postive
BMPR2	SCARNA9	0.702890701	6.83E-81	postive
CRLF3	SCARNA9	0.637782487	1.98E-62	postive
LIFR	SCARNA9	0.529203286	6.06E-40	postive
NR1D2	SCARNA9	0.550357806	1.08E-43	postive
NR2C2	SCARNA9	0.690755454	4.44E-77	postive
RORA	SCARNA9	0.803655563	3.07E-122	postive
SOS1	SCARNA9	0.630152253	1.49E-60	postive
SOS2	SCARNA9	0.541036558	5.24E-42	postive
BRAF	SCARNA9	0.785834318	2.53E-113	postive
TEC	SCARNA9	0.568957303	3.25E-47	postive
CBL	SCARNA9	0.564943718	1.95E-46	postive
CBLB	SCARNA9	0.715257647	5.54E-85	postive
RASGRP1	SCARNA9	0.512850516	3.19E-37	postive
PDK1	SCARNA9	0.570707944	1.47E-47	postive
TRAJ1	SCARNA9	0.706892962	3.42E-82	postive
TRAJ2	SCARNA9	0.763307189	3.33E-103	postive
TRAJ3	SCARNA9	0.782281556	1.20E-111	postive
TRAJ5	SCARNA9	0.708124804	1.35E-82	postive
TRAJ6	SCARNA9	0.77745448	2.02E-109	postive
TRAJ8	SCARNA9	0.701084748	2.59E-80	postive
TRAJ10	SCARNA9	0.735669126	3.10E-92	postive
TRAJ12	SCARNA9	0.743392887	3.70E-95	postive
TRAJ13	SCARNA9	0.705517925	9.61E-82	postive
TRAJ14	SCARNA9	0.739869249	8.22E-94	postive
TRAJ16	SCARNA9	0.749356051	1.73E-97	postive
TRAJ17	SCARNA9	0.684589289	3.26E-75	postive
TRAJ18	SCARNA9	0.76021678	6.64E-102	postive
TRAJ21	SCARNA9	0.765775826	2.94E-104	postive
TRAJ31	SCARNA9	0.757909599	6.02E-101	postive
TRAJ37	SCARNA9	0.753843892	2.76E-99	postive
TRAJ38	SCARNA9	0.840997388	2.66E-144	postive
TRAJ39	SCARNA9	0.785189285	5.12E-113	postive
DDX17	AP001271.1	0.555515479	1.20E-44	postive
GNRH1	AP001271.1	0.574079364	3.17E-48	postive
NR2C1	AP001271.1	0.548596208	2.28E-43	postive
NR2C2	AP001271.1	0.588515872	3.60E-51	postive
BRAF	AP001271.1	0.501005205	2.42E-35	postive
ZC3HAV1L	SNHG4	0.54110963	5.08E-42	postive
DDX17	AL022322.1	0.530776086	3.26E-40	postive
TRAJ17	LINC01138	0.503360942	1.04E-35	postive
LCN2	AC109446.3	0.610089827	7.40E-56	postive
CREB1	AL606834.1	0.605612027	7.44E-55	postive
NFAT5	AL606834.1	0.626987332	8.67E-60	postive
PIK3CA	AL606834.1	0.587920398	4.80E-51	postive
GNRH1	AL606834.1	0.534026015	8.95E-41	postive
ANGPTL1	AL606834.1	0.590691628	1.26E-51	postive
BMPR2	AL606834.1	0.538536707	1.45E-41	postive
RORA	AL606834.1	0.598518221	2.68E-53	postive
SOS1	AL606834.1	0.546465276	5.56E-43	postive
SOS2	AL606834.1	0.565621723	1.45E-46	postive
BRAF	AL606834.1	0.604611845	1.24E-54	postive
CBLB	AL606834.1	0.557216802	5.77E-45	postive
TRAJ1	AL606834.1	0.514047784	2.04E-37	postive
TRAJ2	AL606834.1	0.559477657	2.16E-45	postive
TRAJ3	AL606834.1	0.584442559	2.53E-50	postive
TRAJ5	AL606834.1	0.557160164	5.92E-45	postive
TRAJ6	AL606834.1	0.588109805	4.38E-51	postive
TRAJ8	AL606834.1	0.559362892	2.27E-45	postive

TRAJ10	AL606834.1	0.566825771	8.45E-47	postive
TRAJ13	AL606834.1	0.590143581	1.64E-51	postive
TRAJ14	AL606834.1	0.568406409	4.16E-47	postive
TRAJ16	AL606834.1	0.569172406	2.95E-47	postive
TRAJ17	AL606834.1	0.54304132	2.30E-42	postive
TRAJ18	AL606834.1	0.51689406	6.99E-38	postive
TRAJ21	AL606834.1	0.602953369	2.88E-54	postive
TRAJ31	AL606834.1	0.608400089	1.78E-55	postive
TRAJ37	AL606834.1	0.594188783	2.28E-52	postive
TRAJ38	AL606834.1	0.626955232	8.83E-60	postive
TRAJ39	AL606834.1	0.602199628	4.22E-54	postive
CXCL13	AC018755.4	0.539354159	1.04E-41	postive
RBP5	AC018755.4	0.532817389	1.45E-40	postive
IL7R	AC018755.4	0.520838914	1.56E-38	postive
CD40LG	AC018755.4	0.524584279	3.68E-39	postive
CCR7	AC018755.4	0.53402022	8.97E-41	postive
CXCR4	AC018755.4	0.544713573	1.15E-42	postive
BTK	AC018755.4	0.59720945	5.13E-53	postive
CD22	AC018755.4	0.525589706	2.49E-39	postive
PRKCB	AC018755.4	0.558721583	3.01E-45	postive
IL16	AC018755.4	0.559375538	2.26E-45	postive
TNFSF8	AC018755.4	0.584474665	2.49E-50	postive
IL10RA	AC018755.4	0.500222303	3.21E-35	postive
TNFRSF13C	AC018755.4	0.527774421	1.06E-39	postive
NCR3	AC018755.4	0.547098198	4.27E-43	postive
PTPRC	AC018755.4	0.552355073	4.65E-44	postive
ITK	AC018755.4	0.578201277	4.74E-49	postive
CD28	AC018755.4	0.552597178	4.19E-44	postive
TYK2	AP006623.1	0.504786469	6.20E-36	postive
IRF9	AP006623.1	0.52447056	3.85E-39	postive
LTB4R2	AP006623.1	0.558038315	4.05E-45	postive
GIPR	AP006623.1	0.508655434	1.51E-36	postive
IL11RA	AP006623.1	0.525556891	2.53E-39	postive
CREB1	Z83843.1	0.802935595	7.33E-122	postive
RFXAP	Z83843.1	0.541357481	4.59E-42	postive
UBR1	Z83843.1	0.685577073	1.65E-75	postive
ZC3HAV1	Z83843.1	0.557932891	4.24E-45	postive
IL15	Z83843.1	0.566817434	8.48E-47	postive
CYLD	Z83843.1	0.671173253	2.62E-71	postive
EIF2AK2	Z83843.1	0.551996523	5.41E-44	postive
MAPK8	Z83843.1	0.56990485	2.12E-47	postive
LMBR1	Z83843.1	0.56041272	1.44E-45	postive
LIMS1	Z83843.1	0.505144569	5.44E-36	postive
IREB2	Z83843.1	0.617524771	1.48E-57	postive
DDX17	Z83843.1	0.658777004	7.03E-68	postive
PIK3CG	Z83843.1	0.537115144	2.58E-41	postive
JAK2	Z83843.1	0.663876153	2.86E-69	postive
TXK	Z83843.1	0.62179985	1.48E-58	postive
NFAT5	Z83843.1	0.935309977	1.19E-242	postive
NFATC3	Z83843.1	0.581624013	9.57E-50	postive
MALT1	Z83843.1	0.540312988	7.04E-42	postive
PIK3R1	Z83843.1	0.664181246	2.36E-69	postive
PIK3CA	Z83843.1	0.680436358	5.55E-74	postive
PIK3CB	Z83843.1	0.503593354	9.55E-36	postive
AKT3	Z83843.1	0.513735717	2.29E-37	postive
RASGRP3	Z83843.1	0.533818169	9.72E-41	postive
IGHD6-6	Z83843.1	0.540300755	7.08E-42	postive
PLXNC1	Z83843.1	0.505579457	4.65E-36	postive
ROBO2	Z83843.1	0.519658893	2.45E-38	postive
GNRH1	Z83843.1	0.788659066	1.12E-114	postive
IL6ST	Z83843.1	0.65152571	6.00E-66	postive
RABEP1	Z83843.1	0.583748941	3.51E-50	postive
ACVR2A	Z83843.1	0.665358527	1.11E-69	postive
ANGPTL1	Z83843.1	0.733541246	1.90E-91	postive
BMPR1A	Z83843.1	0.573810567	3.59E-48	postive

BMPR2	Z83843.1	0.749419377	1.64E-97	postive
CRLF3	Z83843.1	0.653329339	2.01E-66	postive
NR1D2	Z83843.1	0.552843566	3.78E-44	postive
NR2C1	Z83843.1	0.507882686	2.00E-36	postive
NR2C2	Z83843.1	0.719728321	1.62E-86	postive
RORA	Z83843.1	0.894189349	3.61E-188	postive
SOS1	Z83843.1	0.688126433	2.81E-76	postive
SOS2	Z83843.1	0.579899873	2.15E-49	postive
BRAF	Z83843.1	0.801378487	4.75E-121	postive
TEC	Z83843.1	0.513885007	2.17E-37	postive
CD28	Z83843.1	0.502102145	1.63E-35	postive
CBL	Z83843.1	0.588359904	3.88E-51	postive
CBLB	Z83843.1	0.724405843	3.75E-88	postive
RASGRP1	Z83843.1	0.513677771	2.34E-37	postive
PDK1	Z83843.1	0.567675784	5.78E-47	postive
TRAJ1	Z83843.1	0.789509034	4.32E-115	postive
TRAJ2	Z83843.1	0.810022202	1.19E-125	postive
TRAJ3	Z83843.1	0.840982869	2.72E-144	postive
TRAJ5	Z83843.1	0.813219226	2.06E-127	postive
TRAJ6	Z83843.1	0.860703903	2.15E-158	postive
TRAJ8	Z83843.1	0.799763134	3.25E-120	postive
TRAJ10	Z83843.1	0.835462617	1.09E-140	postive
TRAJ12	Z83843.1	0.794967844	8.79E-118	postive
TRAJ13	Z83843.1	0.82296626	5.33E-133	postive
TRAJ14	Z83843.1	0.812452714	5.50E-127	postive
TRAJ16	Z83843.1	0.841101179	2.26E-144	postive
TRAJ17	Z83843.1	0.777252294	2.50E-109	postive
TRAJ18	Z83843.1	0.818234585	3.03E-130	postive
TRAJ21	Z83843.1	0.855877792	9.46E-155	postive
TRAJ31	Z83843.1	0.87730215	4.64E-172	postive
TRAJ37	Z83843.1	0.88876066	1.04E-182	postive
TRAJ38	Z83843.1	0.910870604	5.10E-207	postive
TRAJ39	Z83843.1	0.872397477	8.00E-168	postive
TPT1	SNHG29	0.574240704	2.95E-48	postive
GAL	SNHG29	0.660200191	2.89E-68	postive
CREB1	USP46-AS1	0.525218531	2.88E-39	postive
UBR1	USP46-AS1	0.571540216	1.01E-47	postive
IREB2	USP46-AS1	0.539646277	9.25E-42	postive
NFAT5	USP46-AS1	0.537320932	2.38E-41	postive
PIK3CA	USP46-AS1	0.514259227	1.88E-37	postive
BMPR2	USP46-AS1	0.502140273	1.61E-35	postive
NR1D2	USP46-AS1	0.5153562	1.25E-37	postive
NR2C2	USP46-AS1	0.504184156	7.71E-36	postive
SOS1	USP46-AS1	0.556344732	8.42E-45	postive
DDX17	AL162586.1	0.539362026	1.04E-41	postive
LTB4R2	AL162586.1	0.569938128	2.09E-47	postive
GNRH1	AL162586.1	0.512293993	3.92E-37	postive
IL11RA	AL162586.1	0.547273207	3.97E-43	postive
NR2C2	AL162586.1	0.528802672	7.10E-40	postive
DDX17	AC048382.2	0.56013961	1.62E-45	postive
GNRH1	AC048382.2	0.622099795	1.26E-58	postive
NR2C1	AC048382.2	0.568346527	4.27E-47	postive
NR2C2	AC048382.2	0.612467057	2.14E-56	postive
BRAF	AC048382.2	0.508253702	1.75E-36	postive
GNRH1	AC010883.1	0.578911503	3.40E-49	postive
CREB1	C2orf27A	0.522349652	8.73E-39	postive
NOX4	C2orf27A	0.581956784	8.18E-50	postive
PDGFRA	C2orf27A	0.587589655	5.62E-51	postive
LIMS1	C2orf27A	0.608046372	2.13E-55	postive
TNFSF4	C2orf27A	0.503077772	1.15E-35	postive
PIK3CA	C2orf27A	0.526226633	1.95E-39	postive
INHBA	C2orf27A	0.58302655	4.94E-50	postive
BMPR2	C2orf27A	0.571948367	8.39E-48	postive
CREB1	FTX	0.808674446	6.44E-125	postive
RFXAP	FTX	0.537021194	2.68E-41	postive

UBR1	FTX	0.699067916	1.14E-79	postive
ZC3HAV1	FTX	0.572772712	5.77E-48	postive
IL15	FTX	0.572579507	6.30E-48	postive
CYLD	FTX	0.670778855	3.38E-71	postive
EIF2AK2	FTX	0.56188956	7.53E-46	postive
MAPK8	FTX	0.57047347	1.64E-47	postive
LMBR1	FTX	0.578012948	5.17E-49	postive
LIMS1	FTX	0.528456111	8.13E-40	postive
IREB2	FTX	0.627411169	6.86E-60	postive
DDX17	FTX	0.642389015	1.37E-63	postive
PIK3CG	FTX	0.566170851	1.13E-46	postive
JAK2	FTX	0.670017884	5.55E-71	postive
TXK	FTX	0.633630639	2.11E-61	postive
NFAT5	FTX	0.926457912	2.46E-228	postive
NFATC3	FTX	0.579058791	3.18E-49	postive
MALT1	FTX	0.550625179	9.68E-44	postive
PIK3R1	FTX	0.674635602	2.69E-72	postive
PIK3CA	FTX	0.698687406	1.50E-79	postive
PIK3CB	FTX	0.520196216	1.99E-38	postive
AKT3	FTX	0.532160415	1.88E-40	postive
RASGRP3	FTX	0.535706249	4.56E-41	postive
IGHD6-6	FTX	0.569779091	2.24E-47	postive
PLXNC1	FTX	0.516197983	9.09E-38	postive
ROBO2	FTX	0.502214693	1.57E-35	postive
GNRH1	FTX	0.780393402	9.05E-111	postive
IL6ST	FTX	0.66137904	1.38E-68	postive
IL7	FTX	0.505888767	4.15E-36	postive
RABEP1	FTX	0.586040507	1.18E-50	postive
ACVR2A	FTX	0.664614206	1.79E-69	postive
ANGPTL1	FTX	0.746582699	2.14E-96	postive
BMPR1A	FTX	0.578178563	4.79E-49	postive
BMPR2	FTX	0.756707266	1.88E-100	postive
CRLF3	FTX	0.665488915	1.02E-69	postive
NR1D2	FTX	0.54789294	3.06E-43	postive
NR2C2	FTX	0.715417457	4.89E-85	postive
NR3C1	FTX	0.504573983	6.69E-36	postive
RORA	FTX	0.894045803	5.07E-188	postive
SOS1	FTX	0.682498311	1.37E-74	postive
SOS2	FTX	0.586292902	1.05E-50	postive
BRAF	FTX	0.809718352	1.75E-125	postive
ITK	FTX	0.506310473	3.56E-36	postive
TEC	FTX	0.533850436	9.60E-41	postive
CD28	FTX	0.506307612	3.56E-36	postive
CBL	FTX	0.596861197	6.10E-53	postive
CBLB	FTX	0.717705084	8.09E-86	postive
RASGRP1	FTX	0.528712637	7.36E-40	postive
PDK1	FTX	0.58888134	3.02E-51	postive
TRAJ1	FTX	0.785349968	4.30E-113	postive
TRAJ2	FTX	0.795414376	5.25E-118	postive
TRAJ3	FTX	0.843712919	3.98E-146	postive
TRAJ5	FTX	0.818313864	2.73E-130	postive
TRAJ6	FTX	0.857373107	7.26E-156	postive
TRAJ8	FTX	0.802691351	9.84E-122	postive
TRAJ10	FTX	0.832174948	1.33E-138	postive
TRAJ12	FTX	0.776205724	7.46E-109	postive
TRAJ13	FTX	0.823719203	1.91E-133	postive
TRAJ14	FTX	0.809723503	1.74E-125	postive
TRAJ16	FTX	0.839065306	5.03E-143	postive
TRAJ17	FTX	0.783870084	2.15E-112	postive
TRAJ18	FTX	0.809650175	1.90E-125	postive
TRAJ21	FTX	0.860057596	6.73E-158	postive
TRAJ31	FTX	0.871520676	4.38E-167	postive
TRAJ37	FTX	0.878415049	4.79E-173	postive
TRAJ38	FTX	0.903702602	1.69E-198	postive
TRAJ39	FTX	0.884045231	3.44E-178	postive

CYLD	AC093726.1	0.530986934	3.00E-40	postive
DDX17	AC093726.1	0.50418025	7.72E-36	postive
NFAT5	AC093726.1	0.540091467	7.71E-42	postive
PIK3R1	AC093726.1	0.505450441	4.87E-36	postive
RORA	AC093726.1	0.539434781	1.01E-41	postive
CD8A	LINC02195	0.56494286	1.95E-46	postive
IFNG	LINC02195	0.698257224	2.05E-79	postive
TAP1	LINC02195	0.50953973	1.09E-36	postive
TAP2	LINC02195	0.514611573	1.65E-37	postive
IRF1	LINC02195	0.52571011	2.38E-39	postive
CCL5	LINC02195	0.621122966	2.14E-58	postive
CCL4	LINC02195	0.614770726	6.37E-57	postive
FASLG	LINC02195	0.562513894	5.72E-46	postive
XCL2	LINC02195	0.528535039	7.89E-40	postive
CXCR6	LINC02195	0.562264385	6.39E-46	postive
IL15RA	LINC02195	0.536673686	3.09E-41	postive
IL18RAP	LINC02195	0.523054493	6.66E-39	postive
CD247	LINC02195	0.518919241	3.24E-38	postive
GZMB	LINC02195	0.714531056	9.77E-85	postive
CD3D	LINC02195	0.532573213	1.60E-40	postive
IFNG	LINC02362	0.507417246	2.37E-36	postive
PIK3CG	LINC02362	0.555262102	1.34E-44	postive
CXCR6	LINC02362	0.550240019	1.14E-43	postive
CD79A	LINC02362	0.557152714	5.94E-45	postive
RASGRP3	LINC02362	0.562290926	6.31E-46	postive
PLCG2	LINC02362	0.526889819	1.50E-39	postive
IGHD2-2	LINC02362	0.579133291	3.07E-49	postive
IGHD3-9	LINC02362	0.507771691	2.09E-36	postive
IGHD6-25	LINC02362	0.560516933	1.37E-45	postive
IGHD6-6	LINC02362	0.515027657	1.41E-37	postive
IGHG1	LINC02362	0.645299917	2.48E-64	postive
IGHG3	LINC02362	0.520164193	2.02E-38	postive
IGHJ3	LINC02362	0.557921887	4.26E-45	postive
IGHV1-18	LINC02362	0.555083557	1.45E-44	postive
IGHV3-15	LINC02362	0.621099605	2.16E-58	postive
IGHV3-21	LINC02362	0.564860258	2.03E-46	postive
IGHV3-23	LINC02362	0.557719474	4.65E-45	postive
IGHV3-30	LINC02362	0.629307265	2.40E-60	postive
IGHV3-33	LINC02362	0.558432495	3.41E-45	postive
IGHV3-38	LINC02362	0.527823075	1.04E-39	postive
IGHV3-48	LINC02362	0.511725936	4.85E-37	postive
IGHV3-74	LINC02362	0.573468563	4.20E-48	postive
IGHV4-28	LINC02362	0.501646269	1.93E-35	postive
IGHV4-39	LINC02362	0.541726013	3.95E-42	postive
IGHV4-59	LINC02362	0.525345075	2.74E-39	postive
IGHV5-51	LINC02362	0.587172972	6.87E-51	postive
IGKC	LINC02362	0.691534521	2.56E-77	postive
IGKJ5	LINC02362	0.837825666	3.25E-142	postive
IGKV1-39	LINC02362	0.559773068	1.90E-45	postive
IGKV1-5	LINC02362	0.625564188	1.90E-59	postive
IGKV1-6	LINC02362	0.531274083	2.68E-40	postive
IGKV1-9	LINC02362	0.566888359	8.22E-47	postive
IGKV1D-39	LINC02362	0.50356344	9.65E-36	postive
IGKV3-11	LINC02362	0.572748147	5.83E-48	postive
IGKV3-15	LINC02362	0.583895679	3.27E-50	postive
IGKV3-20	LINC02362	0.624090412	4.26E-59	postive
IGKV3-7	LINC02362	0.517673556	5.21E-38	postive
IGKV3D-11	LINC02362	0.519481418	2.62E-38	postive
IGKV4-1	LINC02362	0.596611603	6.90E-53	postive
IGLC2	LINC02362	0.659031099	6.00E-68	postive
IGLC3	LINC02362	0.593990595	2.51E-52	postive
IGLJ1	LINC02362	0.633374527	2.44E-61	postive
IGLJ3	LINC02362	0.558468987	3.36E-45	postive
IGLV1-40	LINC02362	0.540512234	6.49E-42	postive
IGLV1-44	LINC02362	0.551148042	7.76E-44	postive

IGLV1-47	LINC02362	0.569094406	3.05E-47	postive
IGLV2-11	LINC02362	0.597833455	3.76E-53	postive
IGLV2-14	LINC02362	0.586869855	7.94E-51	postive
IGLV3-1	LINC02362	0.545347955	8.85E-43	postive
IGLV3-21	LINC02362	0.601738449	5.32E-54	postive
IGLV3-25	LINC02362	0.583624692	3.72E-50	postive
IGLV6-57	LINC02362	0.595830356	1.02E-52	postive
PLXNC1	LINC02362	0.560614092	1.32E-45	postive
PNOC	LINC02362	0.619685438	4.64E-58	postive
TNFSF13B	LINC02362	0.511628957	5.03E-37	postive
IL18RAP	LINC02362	0.551734166	6.05E-44	postive
TNFRSF17	LINC02362	0.731274631	1.28E-90	postive
SH2D1A	LINC02362	0.572011485	8.15E-48	postive
CD3G	LINC02362	0.545882953	7.08E-43	postive
PTPRC	LINC02362	0.541163723	4.97E-42	postive
ITK	LINC02362	0.558700515	3.03E-45	postive
PDK1	LINC02362	0.624869108	2.78E-59	postive
TRBC1	LINC02362	0.514132742	1.98E-37	postive
CREB1	AC016542.1	0.656779413	2.42E-67	postive
UBR1	AC016542.1	0.594967895	1.56E-52	postive
MAPK8	AC016542.1	0.539799909	8.68E-42	postive
IREB2	AC016542.1	0.567370874	6.62E-47	postive
TXK	AC016542.1	0.529549021	5.29E-40	postive
NFAT5	AC016542.1	0.624195782	4.02E-59	postive
PIK3CA	AC016542.1	0.530830127	3.19E-40	postive
ROBO2	AC016542.1	0.649823849	1.67E-65	postive
GNRH1	AC016542.1	0.700473715	4.06E-80	postive
ACVR2A	AC016542.1	0.596910236	5.95E-53	postive
ANGPTL1	AC016542.1	0.58903801	2.80E-51	postive
BMPR1A	AC016542.1	0.589677055	2.06E-51	postive
BMPR2	AC016542.1	0.701892184	1.43E-80	postive
CRLF3	AC016542.1	0.516285192	8.80E-38	postive
NR1D2	AC016542.1	0.517891683	4.79E-38	postive
NR2C2	AC016542.1	0.563313602	4.02E-46	postive
RORA	AC016542.1	0.628382103	4.01E-60	postive
SOS1	AC016542.1	0.57111718	1.22E-47	postive
BRAF	AC016542.1	0.681421563	2.84E-74	postive
CBLB	AC016542.1	0.562108178	6.84E-46	postive
TRAJ6	AC016542.1	0.501969196	1.71E-35	postive
TRAJ8	AC016542.1	0.519029275	3.11E-38	postive
TRAJ13	AC016542.1	0.512976626	3.04E-37	postive
TRAJ21	AC016542.1	0.504077721	8.01E-36	postive
TRAJ31	AC016542.1	0.557495747	5.12E-45	postive
TRAJ37	AC016542.1	0.513616468	2.40E-37	postive
TRAJ38	AC016542.1	0.571489164	1.03E-47	postive
TRAJ39	AC016542.1	0.502923208	1.22E-35	postive
CMTM8	AC097639.1	0.584590742	2.35E-50	postive
CREB1	SNHG14	0.603882285	1.80E-54	postive
UBR1	SNHG14	0.636007008	5.48E-62	postive
CYLD	SNHG14	0.52496487	3.18E-39	postive
IREB2	SNHG14	0.563177776	4.27E-46	postive
DDX17	SNHG14	0.608223501	1.95E-55	postive
NFAT5	SNHG14	0.757691291	7.41E-101	postive
NFATC3	SNHG14	0.560817904	1.20E-45	postive
MALT1	SNHG14	0.502125765	1.62E-35	postive
PIK3R1	SNHG14	0.531121311	2.84E-40	postive
GNRH1	SNHG14	0.638258402	1.51E-62	postive
IL6ST	SNHG14	0.568526934	3.94E-47	postive
RABEP1	SNHG14	0.527065832	1.40E-39	postive
ACVR2A	SNHG14	0.522977018	6.86E-39	postive
ANGPTL1	SNHG14	0.531016099	2.96E-40	postive
BMPR2	SNHG14	0.555423769	1.25E-44	postive
NR2C2	SNHG14	0.625217429	2.30E-59	postive
RORA	SNHG14	0.738472319	2.77E-93	postive
SOS1	SNHG14	0.542080539	3.41E-42	postive

BRAF	SNHG14	0.604645178	1.22E-54	postive
TRAJ1	SNHG14	0.570386703	1.70E-47	postive
TRAJ2	SNHG14	0.595475395	1.21E-52	postive
TRAJ3	SNHG14	0.6407268	3.62E-63	postive
TRAJ5	SNHG14	0.580193892	1.87E-49	postive
TRAJ6	SNHG14	0.638095086	1.66E-62	postive
TRAJ8	SNHG14	0.568124789	4.72E-47	postive
TRAJ10	SNHG14	0.631131415	8.64E-61	postive
TRAJ12	SNHG14	0.573592664	3.96E-48	postive
TRAJ13	SNHG14	0.585689375	1.40E-50	postive
TRAJ14	SNHG14	0.568965336	3.24E-47	postive
TRAJ16	SNHG14	0.61342608	1.29E-56	postive
TRAJ17	SNHG14	0.582237982	7.16E-50	postive
TRAJ18	SNHG14	0.622988432	7.77E-59	postive
TRAJ21	SNHG14	0.626541619	1.11E-59	postive
TRAJ31	SNHG14	0.633945835	1.77E-61	postive
TRAJ37	SNHG14	0.679803566	8.51E-74	postive
TRAJ38	SNHG14	0.652662721	3.01E-66	postive
TRAJ39	SNHG14	0.648638533	3.41E-65	postive
PTGFR	DPYD-AS1	0.64876565	3.16E-65	postive
TEC	DPYD-AS1	0.565153729	1.78E-46	postive
NFAT5	TMEM9B-AS1	0.540815177	5.74E-42	postive
NFATC3	TMEM9B-AS1	0.512333657	3.87E-37	postive
NR2C2	TMEM9B-AS1	0.542233179	3.21E-42	postive
DDX17	AC087752.3	0.566452041	9.98E-47	postive
IGHD6-6	AC087752.3	0.558338219	3.55E-45	postive
IGKJ5	AC087752.3	0.527472987	1.20E-39	postive
GNRH1	AC087752.3	0.562723681	5.22E-46	postive
NR2C2	AC087752.3	0.623372872	6.30E-59	postive
RORA	AC087752.3	0.56619234	1.12E-46	postive
BRAF	AC087752.3	0.565836159	1.31E-46	postive
TRAJ3	AC087752.3	0.509836634	9.76E-37	postive
TRAJ5	AC087752.3	0.536275178	3.63E-41	postive
TRAJ39	AC087752.3	0.525721339	2.37E-39	postive
BPIFB4	LINC02593	0.566799174	8.55E-47	postive
CREB1	AC013403.2	0.668733802	1.27E-70	postive
RFXAP	AC013403.2	0.5004954	2.91E-35	postive
UBR1	AC013403.2	0.536087726	3.91E-41	postive
ZC3HAV1	AC013403.2	0.567028768	7.72E-47	postive
CYLD	AC013403.2	0.567336126	6.73E-47	postive
EIF2AK2	AC013403.2	0.555058562	1.46E-44	postive
MAPK8	AC013403.2	0.500947015	2.48E-35	postive
IREB2	AC013403.2	0.508849867	1.40E-36	postive
DDX17	AC013403.2	0.646382401	1.31E-64	postive
JAK2	AC013403.2	0.526695074	1.62E-39	postive
TXK	AC013403.2	0.563462742	3.77E-46	postive
NFAT5	AC013403.2	0.7538998	2.62E-99	postive
PIK3R1	AC013403.2	0.599339757	1.78E-53	postive
PIK3CA	AC013403.2	0.589984205	1.77E-51	postive
IGHD6-6	AC013403.2	0.501279824	2.20E-35	postive
GNRH1	AC013403.2	0.723005847	1.17E-87	postive
IL6ST	AC013403.2	0.620633265	2.78E-58	postive
RABEP1	AC013403.2	0.505600493	4.61E-36	postive
ACVR2A	AC013403.2	0.616131214	3.10E-57	postive
ANGPTL1	AC013403.2	0.652682016	2.98E-66	postive
BMPR2	AC013403.2	0.637616138	2.18E-62	postive
CRLF3	AC013403.2	0.547750698	3.25E-43	postive
NR2C1	AC013403.2	0.504549189	6.75E-36	postive
NR2C2	AC013403.2	0.733773937	1.56E-91	postive
RORA	AC013403.2	0.786787088	8.88E-114	postive
SOS1	AC013403.2	0.653462264	1.85E-66	postive
SOS2	AC013403.2	0.53484484	6.44E-41	postive
BRAF	AC013403.2	0.757452398	9.30E-101	postive
CBL	AC013403.2	0.540857466	5.64E-42	postive
CBLB	AC013403.2	0.618939157	6.92E-58	postive

TRAJ1	AC013403.2	0.701400538	2.05E-80	postive
TRAJ2	AC013403.2	0.623549938	5.72E-59	postive
TRAJ3	AC013403.2	0.69167784	2.31E-77	postive
TRAJ5	AC013403.2	0.79825343	1.92E-119	postive
TRAJ6	AC013403.2	0.698578408	1.63E-79	postive
TRAJ8	AC013403.2	0.740486702	4.79E-94	postive
TRAJ10	AC013403.2	0.676962097	5.73E-73	postive
TRAJ12	AC013403.2	0.65451533	9.74E-67	postive
TRAJ13	AC013403.2	0.765681458	3.23E-104	postive
TRAJ14	AC013403.2	0.7117198	8.62E-84	postive
TRAJ16	AC013403.2	0.729999618	3.73E-90	postive
TRAJ17	AC013403.2	0.727793535	2.33E-89	postive
TRAJ18	AC013403.2	0.660211609	2.87E-68	postive
TRAJ21	AC013403.2	0.769969938	4.47E-106	postive
TRAJ31	AC013403.2	0.789571302	4.03E-115	postive
TRAJ37	AC013403.2	0.757586333	8.19E-101	postive
TRAJ38	AC013403.2	0.751265776	3.01E-98	postive
TRAJ39	AC013403.2	0.791292604	5.82E-116	postive
SEMA6D	MIR9-3HG	0.608201341	1.97E-55	postive
CSPG5	MIR9-3HG	0.691185806	3.27E-77	postive
IL34	MIR9-3HG	0.668865769	1.17E-70	postive
BMPR1B	MIR9-3HG	0.761954202	1.24E-102	postive
DDX17	AC012467.2	0.563760953	3.30E-46	postive
GNRH1	AC012467.2	0.580380317	1.71E-49	postive
ACVR2B	AC012467.2	0.508020772	1.90E-36	postive
NR2C2	AC012467.2	0.644259808	4.58E-64	postive
GCG	LINC01116	0.512062759	4.28E-37	postive
CREB1	AC002064.3	0.756147816	3.19E-100	postive
UBR1	AC002064.3	0.599114043	1.99E-53	postive
ZC3HAV1	AC002064.3	0.53431517	7.97E-41	postive
IL15	AC002064.3	0.617731547	1.32E-57	postive
CYLD	AC002064.3	0.605782728	6.82E-55	postive
EIF2AK2	AC002064.3	0.500426827	2.98E-35	postive
MAPK8	AC002064.3	0.525758207	2.34E-39	postive
TLR1	AC002064.3	0.535069275	5.89E-41	postive
LMBR1	AC002064.3	0.553928201	2.38E-44	postive
LIMS1	AC002064.3	0.521797448	1.08E-38	postive
IREB2	AC002064.3	0.553833768	2.47E-44	postive
DDX17	AC002064.3	0.563322952	4.01E-46	postive
PIK3CG	AC002064.3	0.530959937	3.03E-40	postive
JAK2	AC002064.3	0.651288803	6.93E-66	postive
TXK	AC002064.3	0.547658026	3.38E-43	postive
NFAT5	AC002064.3	0.839120394	4.63E-143	postive
MALT1	AC002064.3	0.535480313	4.99E-41	postive
PIK3R1	AC002064.3	0.575576345	1.60E-48	postive
PIK3CA	AC002064.3	0.713012509	3.18E-84	postive
PIK3CB	AC002064.3	0.532827402	1.44E-40	postive
AKT3	AC002064.3	0.522168923	9.36E-39	postive
RASGRP3	AC002064.3	0.510920178	6.54E-37	postive
IGHD6-6	AC002064.3	0.504348118	7.26E-36	postive
GNRH1	AC002064.3	0.71826604	5.19E-86	postive
IL6ST	AC002064.3	0.563978404	3.00E-46	postive
IL7	AC002064.3	0.550654298	9.56E-44	postive
RABEP1	AC002064.3	0.535320649	5.32E-41	postive
ACVR2A	AC002064.3	0.58803178	4.55E-51	postive
ANGPTL1	AC002064.3	0.767591459	4.85E-105	postive
BMPR2	AC002064.3	0.677516271	3.96E-73	postive
CRLF3	AC002064.3	0.636925415	3.24E-62	postive
IL18R1	AC002064.3	0.540356023	6.92E-42	postive
NR2C2	AC002064.3	0.657084608	2.01E-67	postive
RORA	AC002064.3	0.785047943	5.98E-113	postive
SOS1	AC002064.3	0.645975151	1.66E-64	postive
SOS2	AC002064.3	0.575110207	1.98E-48	postive
BRAF	AC002064.3	0.713816184	1.70E-84	postive
CBL	AC002064.3	0.613170791	1.48E-56	postive

CBLB	AC002064.3	0.685389021	1.88E-75	postive
PDK1	AC002064.3	0.598314986	2.96E-53	postive
TRAJ1	AC002064.3	0.76085928	3.57E-102	postive
TRAJ2	AC002064.3	0.693914149	4.71E-78	postive
TRAJ3	AC002064.3	0.80166794	3.36E-121	postive
TRAJ5	AC002064.3	0.794177534	2.18E-117	postive
TRAJ6	AC002064.3	0.823487705	2.62E-133	postive
TRAJ8	AC002064.3	0.769435478	7.65E-106	postive
TRAJ10	AC002064.3	0.836874876	1.35E-141	postive
TRAJ12	AC002064.3	0.69781416	2.84E-79	postive
TRAJ13	AC002064.3	0.795278066	6.15E-118	postive
TRAJ14	AC002064.3	0.77448076	4.46E-108	postive
TRAJ16	AC002064.3	0.840895641	3.10E-144	postive
TRAJ17	AC002064.3	0.813498467	1.44E-127	postive
TRAJ18	AC002064.3	0.768287432	2.42E-105	postive
TRAJ21	AC002064.3	0.831895864	1.98E-138	postive
TRAJ31	AC002064.3	0.827809238	6.63E-136	postive
TRAJ37	AC002064.3	0.798694701	1.15E-119	postive
TRAJ38	AC002064.3	0.852739053	1.88E-152	postive
TRAJ39	AC002064.3	0.843922617	2.87E-146	postive
CREB1	AL354733.3	0.643734402	6.24E-64	postive
RFXAP	AL354733.3	0.524072396	4.49E-39	postive
UBR1	AL354733.3	0.546516461	5.44E-43	postive
ZC3HAV1	AL354733.3	0.561376476	9.43E-46	postive
CYLD	AL354733.3	0.575075177	2.01E-48	postive
IREB2	AL354733.3	0.505954359	4.05E-36	postive
DDX17	AL354733.3	0.74435562	1.57E-95	postive
JAK2	AL354733.3	0.549862931	1.34E-43	postive
TXK	AL354733.3	0.588535869	3.57E-51	postive
NFAT5	AL354733.3	0.705124652	1.29E-81	postive
PIK3R1	AL354733.3	0.5425189	2.85E-42	postive
PIK3CA	AL354733.3	0.558904261	2.78E-45	postive
LTB4R2	AL354733.3	0.558769823	2.94E-45	postive
GNRH1	AL354733.3	0.744628494	1.23E-95	postive
IL6ST	AL354733.3	0.528743756	7.27E-40	postive
ACVR2A	AL354733.3	0.539572131	9.53E-42	postive
ANGPTL1	AL354733.3	0.611652146	3.28E-56	postive
BMPR2	AL354733.3	0.560786401	1.22E-45	postive
CRLF3	AL354733.3	0.518044055	4.52E-38	postive
NR2C1	AL354733.3	0.581329323	1.10E-49	postive
NR2C2	AL354733.3	0.780803823	5.84E-111	postive
RORA	AL354733.3	0.724243423	4.28E-88	postive
SOS1	AL354733.3	0.594205712	2.26E-52	postive
SOS2	AL354733.3	0.515882641	1.02E-37	postive
BRAF	AL354733.3	0.722312144	2.04E-87	postive
ITK	AL354733.3	0.533178516	1.25E-40	postive
CBL	AL354733.3	0.571676402	9.50E-48	postive
CBLB	AL354733.3	0.639634051	6.82E-63	postive
TRAJ1	AL354733.3	0.637941744	1.81E-62	postive
TRAJ2	AL354733.3	0.625401673	2.08E-59	postive
TRAJ3	AL354733.3	0.665715693	8.86E-70	postive
TRAJ5	AL354733.3	0.648039805	4.88E-65	postive
TRAJ6	AL354733.3	0.634325731	1.43E-61	postive
TRAJ8	AL354733.3	0.594772684	1.71E-52	postive
TRAJ10	AL354733.3	0.605422242	8.20E-55	postive
TRAJ12	AL354733.3	0.568845305	3.42E-47	postive
TRAJ13	AL354733.3	0.620355998	3.23E-58	postive
TRAJ14	AL354733.3	0.608199952	1.97E-55	postive
TRAJ16	AL354733.3	0.62419413	4.02E-59	postive
TRAJ17	AL354733.3	0.5913674	9.06E-52	postive
TRAJ18	AL354733.3	0.611406053	3.73E-56	postive
TRAJ21	AL354733.3	0.646309861	1.37E-64	postive
TRAJ31	AL354733.3	0.636867624	3.35E-62	postive
TRAJ37	AL354733.3	0.630118505	1.52E-60	postive
TRAJ38	AL354733.3	0.672900519	8.44E-72	postive

TRAJ39	AL354733.3	0.650487577	1.12E-65	postive
IGHD4-4	AC244517.7	0.549119017	1.83E-43	postive
UBR1	AL606489.1	0.504488234	6.91E-36	postive
SP1	AL606489.1	0.542687225	2.66E-42	postive
TXK	AL606489.1	0.504373371	7.20E-36	postive
PIK3CA	AL606489.1	0.512829976	3.21E-37	postive
PIK3CB	AL606489.1	0.514309614	1.85E-37	postive
UBR1	DGCR11	0.512135357	4.16E-37	postive
NFAT5	DGCR11	0.536727599	3.02E-41	postive
GNRH1	DGCR11	0.505178308	5.37E-36	postive
RORA	DGCR11	0.519715274	2.40E-38	postive
BRAF	DGCR11	0.526877881	1.51E-39	postive
TRAJ3	DGCR11	0.515268861	1.29E-37	postive
TRAJ6	DGCR11	0.505309477	5.12E-36	postive
TRAJ8	DGCR11	0.502880916	1.23E-35	postive
TRAJ10	DGCR11	0.511934962	4.49E-37	postive
TRAJ16	DGCR11	0.502096168	1.64E-35	postive
TRAJ21	DGCR11	0.522184603	9.31E-39	postive
TRAJ31	DGCR11	0.537763234	1.99E-41	postive
TRAJ37	DGCR11	0.521538751	1.19E-38	postive
TRAJ38	DGCR11	0.54855679	2.32E-43	postive
TRAJ39	DGCR11	0.536987686	2.72E-41	postive
DDX17	CAPN10-DT	0.522014221	9.94E-39	postive
IRF9	CAPN10-DT	0.505094244	5.54E-36	postive
LTB4R2	CAPN10-DT	0.50182677	1.81E-35	postive
GNRH1	CAPN10-DT	0.582236634	7.17E-50	postive
NR2C2	CAPN10-DT	0.517553093	5.45E-38	postive
CREB1	AC124319.1	0.571337465	1.11E-47	postive
IL15	AC124319.1	0.556320259	8.51E-45	postive
EIF2AK2	AC124319.1	0.54887821	2.02E-43	postive
JAK2	AC124319.1	0.541721372	3.96E-42	postive
NFAT5	AC124319.1	0.626418711	1.19E-59	postive
PIK3CA	AC124319.1	0.512064277	4.27E-37	postive
GNRH1	AC124319.1	0.561557201	8.71E-46	postive
ANGPTL1	AC124319.1	0.616587548	2.43E-57	postive
CRLF3	AC124319.1	0.502110837	1.63E-35	postive
NR2C2	AC124319.1	0.600141414	1.19E-53	postive
RORA	AC124319.1	0.555489824	1.22E-44	postive
SOS1	AC124319.1	0.586223133	1.08E-50	postive
BRAF	AC124319.1	0.665244585	1.20E-69	postive
CBL	AC124319.1	0.535495294	4.96E-41	postive
CBLB	AC124319.1	0.64640871	1.29E-64	postive
TRAJ1	AC124319.1	0.701909689	1.41E-80	postive
TRAJ2	AC124319.1	0.611198923	4.15E-56	postive
TRAJ3	AC124319.1	0.712499691	4.73E-84	postive
TRAJ5	AC124319.1	0.727828138	2.27E-89	postive
TRAJ6	AC124319.1	0.722568188	1.66E-87	postive
TRAJ8	AC124319.1	0.672592657	1.03E-71	postive
TRAJ10	AC124319.1	0.681385742	2.91E-74	postive
TRAJ12	AC124319.1	0.763138369	3.92E-103	postive
TRAJ13	AC124319.1	0.702140127	1.19E-80	postive
TRAJ14	AC124319.1	0.758828191	2.51E-101	postive
TRAJ16	AC124319.1	0.692599884	1.20E-77	postive
TRAJ17	AC124319.1	0.683991999	4.92E-75	postive
TRAJ18	AC124319.1	0.715570379	4.34E-85	postive
TRAJ21	AC124319.1	0.745890418	3.99E-96	postive
TRAJ31	AC124319.1	0.71744013	9.97E-86	postive
TRAJ37	AC124319.1	0.668970359	1.09E-70	postive
TRAJ38	AC124319.1	0.754011345	2.37E-99	postive
TRAJ39	AC124319.1	0.702994193	6.32E-81	postive
DDX17	AC130456.3	0.503605227	9.51E-36	postive
NFAT5	AC130456.3	0.534763025	6.66E-41	postive
GNRH1	AC130456.3	0.520778153	1.60E-38	postive
NR2C2	AC130456.3	0.514827806	1.52E-37	postive
RORA	AC130456.3	0.58013977	1.92E-49	postive

CBLB	AC130456.3	0.56027211	1.53E-45	postive
DDX17	HM13-IT1	0.59609476	8.92E-53	postive
NFAT5	HM13-IT1	0.501129316	2.32E-35	postive
LTB4R2	HM13-IT1	0.524099041	4.45E-39	postive
GNRH1	HM13-IT1	0.654820287	8.08E-67	postive
ANGPTL1	HM13-IT1	0.506898713	2.87E-36	postive
NR2C1	HM13-IT1	0.508997199	1.33E-36	postive
NR2C2	HM13-IT1	0.618284565	9.83E-58	postive
BRAF	HM13-IT1	0.51564062	1.12E-37	postive
CREB1	AL359921.1	0.608568651	1.63E-55	postive
RFXAP	AL359921.1	0.505946513	4.06E-36	postive
UBR1	AL359921.1	0.511475396	5.32E-37	postive
ZC3HAV1	AL359921.1	0.503300336	1.06E-35	postive
CYLD	AL359921.1	0.506419645	3.42E-36	postive
DDX17	AL359921.1	0.670274351	4.70E-71	postive
NFAT5	AL359921.1	0.725386308	1.69E-88	postive
PIK3CA	AL359921.1	0.542697893	2.65E-42	postive
IGHD6-6	AL359921.1	0.504774825	6.22E-36	postive
LTB4R2	AL359921.1	0.522063765	9.75E-39	postive
GNRH1	AL359921.1	0.74889003	2.65E-97	postive
ACVR2A	AL359921.1	0.515015556	1.42E-37	postive
ANGPTL1	AL359921.1	0.609844316	8.41E-56	postive
BMPR2	AL359921.1	0.524470674	3.85E-39	postive
NR2C1	AL359921.1	0.52669423	1.62E-39	postive
NR2C2	AL359921.1	0.717977115	6.52E-86	postive
RORA	AL359921.1	0.672998857	7.91E-72	postive
SOS1	AL359921.1	0.575174548	1.92E-48	postive
SOS2	AL359921.1	0.514289936	1.86E-37	postive
BRAF	AL359921.1	0.675026435	2.08E-72	postive
CBL	AL359921.1	0.562226171	6.49E-46	postive
CBLB	AL359921.1	0.547755015	3.24E-43	postive
TRAJ1	AL359921.1	0.643780404	6.08E-64	postive
TRAJ2	AL359921.1	0.613986535	9.63E-57	postive
TRAJ3	AL359921.1	0.716048568	2.98E-85	postive
TRAJ5	AL359921.1	0.644771665	3.39E-64	postive
TRAJ6	AL359921.1	0.671605321	1.97E-71	postive
TRAJ8	AL359921.1	0.645452579	2.27E-64	postive
TRAJ10	AL359921.1	0.670020665	5.54E-71	postive
TRAJ12	AL359921.1	0.587103554	7.10E-51	postive
TRAJ13	AL359921.1	0.653870306	1.44E-66	postive
TRAJ14	AL359921.1	0.640742354	3.59E-63	postive
TRAJ16	AL359921.1	0.667879077	2.21E-70	postive
TRAJ17	AL359921.1	0.663912433	2.79E-69	postive
TRAJ18	AL359921.1	0.637137671	2.87E-62	postive
TRAJ21	AL359921.1	0.688546712	2.09E-76	postive
TRAJ31	AL359921.1	0.663339876	4.01E-69	postive
TRAJ37	AL359921.1	0.664348225	2.12E-69	postive
TRAJ38	AL359921.1	0.684312444	3.94E-75	postive
TRAJ39	AL359921.1	0.698643209	1.55E-79	postive
CREB1	AL513327.1	0.672889235	8.50E-72	postive
UBR1	AL513327.1	0.519737597	2.38E-38	postive
IL15	AL513327.1	0.554311567	2.02E-44	postive
CYLD	AL513327.1	0.50977246	9.99E-37	postive
NFKBIZ	AL513327.1	0.581876786	8.49E-50	postive
LMBR1	AL513327.1	0.502399545	1.47E-35	postive
DDX17	AL513327.1	0.535601514	4.76E-41	postive
JAK2	AL513327.1	0.616938324	2.02E-57	postive
TXK	AL513327.1	0.521032271	1.45E-38	postive
NFAT5	AL513327.1	0.71981808	1.51E-86	postive
PIK3R1	AL513327.1	0.511655422	4.98E-37	postive
PIK3CA	AL513327.1	0.622032308	1.31E-58	postive
GNRH1	AL513327.1	0.693423886	6.68E-78	postive
IL6ST	AL513327.1	0.505475846	4.82E-36	postive
ANGPTL1	AL513327.1	0.710680173	1.92E-83	postive
BMPR2	AL513327.1	0.592100915	6.34E-52	postive

CRLF3	AL513327.1	0.564373736	2.52E-46	postive
NR2C2	AL513327.1	0.619798846	4.36E-58	postive
RORA	AL513327.1	0.723564942	7.42E-88	postive
SOS1	AL513327.1	0.569787711	2.23E-47	postive
BRAF	AL513327.1	0.637503404	2.33E-62	postive
CBL	AL513327.1	0.5593916	2.25E-45	postive
CBLB	AL513327.1	0.726407369	7.30E-89	postive
PDK1	AL513327.1	0.591096829	1.03E-51	postive
TRAJ1	AL513327.1	0.56530389	1.67E-46	postive
TRAJ2	AL513327.1	0.542616686	2.74E-42	postive
TRAJ3	AL513327.1	0.592656283	4.83E-52	postive
TRAJ5	AL513327.1	0.642001163	1.72E-63	postive
TRAJ6	AL513327.1	0.623830047	4.91E-59	postive
TRAJ8	AL513327.1	0.50874383	1.46E-36	postive
TRAJ10	AL513327.1	0.647898767	5.30E-65	postive
TRAJ12	AL513327.1	0.502251986	1.55E-35	postive
TRAJ13	AL513327.1	0.584059939	3.03E-50	postive
TRAJ14	AL513327.1	0.562770532	5.11E-46	postive
TRAJ16	AL513327.1	0.616105956	3.14E-57	postive
TRAJ17	AL513327.1	0.556917264	6.57E-45	postive
TRAJ18	AL513327.1	0.581835525	8.66E-50	postive
TRAJ21	AL513327.1	0.618148709	1.06E-57	postive
TRAJ31	AL513327.1	0.648611962	3.46E-65	postive
TRAJ37	AL513327.1	0.617633673	1.39E-57	postive
TRAJ38	AL513327.1	0.6950249	2.13E-78	postive
TRAJ39	AL513327.1	0.633289553	2.56E-61	postive
DES	ANKRD44-AS1	0.53276593	1.48E-40	postive
GHRL	ANKRD44-AS1	0.592366329	5.57E-52	postive
CREB1	LIMS1-AS1	0.783035486	5.32E-112	postive
RFXAP	LIMS1-AS1	0.503714362	9.14E-36	postive
UBR1	LIMS1-AS1	0.631497073	7.04E-61	postive
ZC3HAV1	LIMS1-AS1	0.558005432	4.10E-45	postive
ZC3HAV1L	LIMS1-AS1	0.506478349	3.35E-36	postive
IL15	LIMS1-AS1	0.612377572	2.24E-56	postive
CYLD	LIMS1-AS1	0.626439714	1.17E-59	postive
EIF2AK2	LIMS1-AS1	0.549234302	1.74E-43	postive
MAPK8	LIMS1-AS1	0.560714226	1.26E-45	postive
TLR1	LIMS1-AS1	0.532275355	1.80E-40	postive
NFKBIZ	LIMS1-AS1	0.536656035	3.11E-41	postive
LMBR1	LIMS1-AS1	0.584136829	2.92E-50	postive
LIMS1	LIMS1-AS1	0.59494529	1.57E-52	postive
IREB2	LIMS1-AS1	0.59512594	1.44E-52	postive
DDX17	LIMS1-AS1	0.619940499	4.04E-58	postive
PIK3CG	LIMS1-AS1	0.587051836	7.28E-51	postive
JAK2	LIMS1-AS1	0.691101469	3.48E-77	postive
TXK	LIMS1-AS1	0.62747932	6.61E-60	postive
NFAT5	LIMS1-AS1	0.834263077	6.37E-140	postive
MALT1	LIMS1-AS1	0.572842074	5.59E-48	postive
PIK3R1	LIMS1-AS1	0.646985138	9.14E-65	postive
PIK3CA	LIMS1-AS1	0.728497211	1.30E-89	postive
PIK3CB	LIMS1-AS1	0.532913424	1.39E-40	postive
AKT3	LIMS1-AS1	0.528031267	9.61E-40	postive
RASGRP3	LIMS1-AS1	0.568271087	4.42E-47	postive
IGHD6-6	LIMS1-AS1	0.533295491	1.20E-40	postive
PLXNC1	LIMS1-AS1	0.506928815	2.84E-36	postive
FGF7	LIMS1-AS1	0.504534924	6.79E-36	postive
GNRH1	LIMS1-AS1	0.745422722	6.06E-96	postive
IL6ST	LIMS1-AS1	0.63486196	1.05E-61	postive
IL7	LIMS1-AS1	0.505213104	5.31E-36	postive
RABEP1	LIMS1-AS1	0.561207258	1.02E-45	postive
ACVR2A	LIMS1-AS1	0.595587705	1.15E-52	postive
ANGPTL1	LIMS1-AS1	0.798403662	1.61E-119	postive
BMPR1A	LIMS1-AS1	0.502436539	1.45E-35	postive
BMPR2	LIMS1-AS1	0.727710338	2.50E-89	postive
CRLF3	LIMS1-AS1	0.659667355	4.04E-68	postive

IL18R1	LIMS1-AS1	0.546363998	5.80E-43	postive
NR2C2	LIMS1-AS1	0.703083474	5.92E-81	postive
RORA	LIMS1-AS1	0.850829769	4.44E-151	postive
SOS1	LIMS1-AS1	0.679465183	1.07E-73	postive
SOS2	LIMS1-AS1	0.575368791	1.76E-48	postive
BRAF	LIMS1-AS1	0.775240676	2.03E-108	postive
ITK	LIMS1-AS1	0.548417537	2.45E-43	postive
TEC	LIMS1-AS1	0.53104075	2.94E-40	postive
CD28	LIMS1-AS1	0.504835344	6.09E-36	postive
CBL	LIMS1-AS1	0.63749491	2.34E-62	postive
CBLB	LIMS1-AS1	0.778281839	8.47E-110	postive
RASGRP1	LIMS1-AS1	0.527674848	1.11E-39	postive
PDK1	LIMS1-AS1	0.643787965	6.05E-64	postive
TRAJ1	LIMS1-AS1	0.701699502	1.65E-80	postive
TRAJ2	LIMS1-AS1	0.690997826	3.74E-77	postive
TRAJ3	LIMS1-AS1	0.757404539	9.73E-101	postive
TRAJ5	LIMS1-AS1	0.776865522	3.75E-109	postive
TRAJ6	LIMS1-AS1	0.757196338	1.18E-100	postive
TRAJ8	LIMS1-AS1	0.692532199	1.26E-77	postive
TRAJ10	LIMS1-AS1	0.757151098	1.24E-100	postive
TRAJ12	LIMS1-AS1	0.648953087	2.82E-65	postive
TRAJ13	LIMS1-AS1	0.731815708	8.16E-91	postive
TRAJ14	LIMS1-AS1	0.716170476	2.71E-85	postive
TRAJ16	LIMS1-AS1	0.765962374	2.45E-104	postive
TRAJ17	LIMS1-AS1	0.716995234	1.42E-85	postive
TRAJ18	LIMS1-AS1	0.720121192	1.19E-86	postive
TRAJ21	LIMS1-AS1	0.776349453	6.42E-109	postive
TRAJ31	LIMS1-AS1	0.786289216	1.54E-113	postive
TRAJ37	LIMS1-AS1	0.760021229	8.01E-102	postive
TRAJ38	LIMS1-AS1	0.830655744	1.18E-137	postive
TRAJ39	LIMS1-AS1	0.805605943	2.86E-123	postive
CREB1	AC234775.3	0.807603165	2.44E-124	postive
RFXAP	AC234775.3	0.527510929	1.18E-39	postive
UBR1	AC234775.3	0.709786701	3.80E-83	postive
ZC3HAV1	AC234775.3	0.607511521	2.81E-55	postive
IFNAR1	AC234775.3	0.513855133	2.19E-37	postive
IL15	AC234775.3	0.568489747	4.01E-47	postive
CYLD	AC234775.3	0.710101986	2.99E-83	postive
EIF2AK2	AC234775.3	0.582844706	5.38E-50	postive
MAPK8	AC234775.3	0.55173017	6.06E-44	postive
LMBR1	AC234775.3	0.586763718	8.35E-51	postive
LIMS1	AC234775.3	0.512742745	3.32E-37	postive
IREB2	AC234775.3	0.634328243	1.42E-61	postive
DDX17	AC234775.3	0.630742414	1.07E-60	postive
PIK3CG	AC234775.3	0.597570519	4.29E-53	postive
JAK2	AC234775.3	0.667391616	3.03E-70	postive
TXK	AC234775.3	0.622103949	1.26E-58	postive
NFAT5	AC234775.3	0.854471849	1.03E-153	postive
NFATC3	AC234775.3	0.562852223	4.93E-46	postive
MALT1	AC234775.3	0.568201118	4.56E-47	postive
PIK3R1	AC234775.3	0.726582118	6.33E-89	postive
PIK3CA	AC234775.3	0.735405439	3.88E-92	postive
PIK3CB	AC234775.3	0.583558607	3.84E-50	postive
AKT3	AC234775.3	0.507173674	2.60E-36	postive
RASGRP3	AC234775.3	0.566063659	1.19E-46	postive
PLXNC1	AC234775.3	0.545511476	8.27E-43	postive
GNRH1	AC234775.3	0.685365963	1.91E-75	postive
IL6ST	AC234775.3	0.715756955	3.75E-85	postive
RABEP1	AC234775.3	0.599594283	1.56E-53	postive
ACVR2A	AC234775.3	0.661752466	1.09E-68	postive
ANGPTL1	AC234775.3	0.770154906	3.70E-106	postive
BMPR1A	AC234775.3	0.566345732	1.05E-46	postive
BMPR2	AC234775.3	0.769479819	7.32E-106	postive
CRLF3	AC234775.3	0.593908756	2.62E-52	postive
LIFR	AC234775.3	0.533453657	1.12E-40	postive

NR1D2	AC234775.3	0.603452448	2.24E-54	postive
NR2C2	AC234775.3	0.742150673	1.11E-94	postive
NR3C1	AC234775.3	0.503291206	1.06E-35	postive
NR3C2	AC234775.3	0.506924418	2.84E-36	postive
RORA	AC234775.3	0.889219842	3.69E-183	postive
SOS1	AC234775.3	0.709721144	3.99E-83	postive
SOS2	AC234775.3	0.627838606	5.42E-60	postive
BRAF	AC234775.3	0.833477044	2.01E-139	postive
ITK	AC234775.3	0.557848237	4.39E-45	postive
CD28	AC234775.3	0.548032732	2.88E-43	postive
CBL	AC234775.3	0.614034687	9.39E-57	postive
CBLB	AC234775.3	0.732657566	4.01E-91	postive
RASGRP1	AC234775.3	0.546663239	5.12E-43	postive
PDK1	AC234775.3	0.562163964	6.67E-46	postive
TRAJ1	AC234775.3	0.740041688	7.07E-94	postive
TRAJ2	AC234775.3	0.701117234	2.53E-80	postive
TRAJ3	AC234775.3	0.784848588	7.43E-113	postive
TRAJ5	AC234775.3	0.827345759	1.27E-135	postive
TRAJ6	AC234775.3	0.792055381	2.46E-116	postive
TRAJ8	AC234775.3	0.751564258	2.29E-98	postive
TRAJ10	AC234775.3	0.780212956	1.10E-110	postive
TRAJ12	AC234775.3	0.777683523	1.59E-109	postive
TRAJ13	AC234775.3	0.792088852	2.37E-116	postive
TRAJ14	AC234775.3	0.78742158	4.41E-114	postive
TRAJ16	AC234775.3	0.78332216	3.90E-112	postive
TRAJ17	AC234775.3	0.782948877	5.84E-112	postive
TRAJ18	AC234775.3	0.776274986	6.94E-109	postive
TRAJ21	AC234775.3	0.829943523	3.25E-137	postive
TRAJ31	AC234775.3	0.842497688	2.63E-145	postive
TRAJ37	AC234775.3	0.812886019	3.16E-127	postive
TRAJ38	AC234775.3	0.872346763	8.83E-168	postive
TRAJ39	AC234775.3	0.837901909	2.90E-142	postive
CREB1	AC093799.1	0.747961133	6.16E-97	postive
UBR1	AC093799.1	0.621433744	1.81E-58	postive
ZC3HAV1	AC093799.1	0.507278948	2.50E-36	postive
IL15	AC093799.1	0.540146573	7.54E-42	postive
CYLD	AC093799.1	0.527288345	1.29E-39	postive
MAPK8	AC093799.1	0.522364131	8.69E-39	postive
LMBR1	AC093799.1	0.556254931	8.75E-45	postive
IREB2	AC093799.1	0.586683126	8.68E-51	postive
DDX17	AC093799.1	0.530157541	4.16E-40	postive
JAK2	AC093799.1	0.615333	4.73E-57	postive
TXK	AC093799.1	0.552718226	3.98E-44	postive
NFAT5	AC093799.1	0.731162718	1.41E-90	postive
PIK3R1	AC093799.1	0.549993863	1.26E-43	postive
PIK3CA	AC093799.1	0.654288017	1.12E-66	postive
PIK3CB	AC093799.1	0.51530801	1.27E-37	postive
ROBO2	AC093799.1	0.573885001	3.47E-48	postive
GNRH1	AC093799.1	0.729865556	4.18E-90	postive
IL6ST	AC093799.1	0.502621731	1.36E-35	postive
RABEP1	AC093799.1	0.534310694	7.98E-41	postive
ACVR2A	AC093799.1	0.617451575	1.53E-57	postive
ANGPTL1	AC093799.1	0.713285882	2.57E-84	postive
BMPR1A	AC093799.1	0.562638014	5.42E-46	postive
BMPR2	AC093799.1	0.72469262	2.97E-88	postive
CRLF3	AC093799.1	0.588053365	4.50E-51	postive
NR1D2	AC093799.1	0.533559552	1.08E-40	postive
NR2C2	AC093799.1	0.627844592	5.40E-60	postive
RORA	AC093799.1	0.701439916	2.00E-80	postive
SOS1	AC093799.1	0.641309948	2.58E-63	postive
SOS2	AC093799.1	0.523242734	6.19E-39	postive
BRAF	AC093799.1	0.710002561	3.22E-83	postive
CBL	AC093799.1	0.553855642	2.45E-44	postive
CBLB	AC093799.1	0.705921821	7.10E-82	postive
PDK1	AC093799.1	0.537744759	2.00E-41	postive

TRAJ1	AC093799.1	0.599026348	2.08E-53	postive
TRAJ2	AC093799.1	0.596029912	9.21E-53	postive
TRAJ3	AC093799.1	0.636556995	4.00E-62	postive
TRAJ5	AC093799.1	0.625213066	2.30E-59	postive
TRAJ6	AC093799.1	0.669963033	5.75E-71	postive
TRAJ8	AC093799.1	0.610683881	5.43E-56	postive
TRAJ10	AC093799.1	0.633997554	1.72E-61	postive
TRAJ12	AC093799.1	0.617959102	1.17E-57	postive
TRAJ13	AC093799.1	0.631297088	7.88E-61	postive
TRAJ14	AC093799.1	0.61665503	2.35E-57	postive
TRAJ16	AC093799.1	0.639885717	5.89E-63	postive
TRAJ17	AC093799.1	0.590061398	1.71E-51	postive
TRAJ18	AC093799.1	0.577130916	7.78E-49	postive
TRAJ21	AC093799.1	0.642064467	1.66E-63	postive
TRAJ31	AC093799.1	0.694339668	3.47E-78	postive
TRAJ37	AC093799.1	0.634979959	9.84E-62	postive
TRAJ38	AC093799.1	0.738144381	3.68E-93	postive
TRAJ39	AC093799.1	0.633872022	1.84E-61	postive
CREB1	AC026470.2	0.721174329	5.11E-87	postive
UBR1	AC026470.2	0.584867338	2.06E-50	postive
ZC3HAV1	AC026470.2	0.520320399	1.90E-38	postive
IL15	AC026470.2	0.599218506	1.89E-53	postive
CYLD	AC026470.2	0.592081852	6.40E-52	postive
MAPK8	AC026470.2	0.503076361	1.15E-35	postive
LMBR1	AC026470.2	0.523307941	6.04E-39	postive
IREB2	AC026470.2	0.528771718	7.19E-40	postive
DDX17	AC026470.2	0.52627106	1.91E-39	postive
PIK3CG	AC026470.2	0.501520576	2.02E-35	postive
JAK2	AC026470.2	0.605926365	6.34E-55	postive
TXK	AC026470.2	0.541226437	4.85E-42	postive
NFAT5	AC026470.2	0.801618629	3.57E-121	postive
MALT1	AC026470.2	0.509809502	9.86E-37	postive
PIK3R1	AC026470.2	0.551738332	6.04E-44	postive
PIK3CA	AC026470.2	0.683928119	5.14E-75	postive
PIK3CB	AC026470.2	0.52887524	6.90E-40	postive
AKT3	AC026470.2	0.52110797	1.41E-38	postive
IGHD6-6	AC026470.2	0.501612487	1.95E-35	postive
GNRH1	AC026470.2	0.678405185	2.18E-73	postive
IL6ST	AC026470.2	0.525164239	2.94E-39	postive
IL7	AC026470.2	0.529613067	5.16E-40	postive
RABEP1	AC026470.2	0.501341556	2.15E-35	postive
ACVR2A	AC026470.2	0.554725082	1.69E-44	postive
ANGPTL1	AC026470.2	0.734795135	6.54E-92	postive
BMPR2	AC026470.2	0.661246975	1.50E-68	postive
CRLF3	AC026470.2	0.596810466	6.26E-53	postive
IL18R1	AC026470.2	0.554047507	2.26E-44	postive
NR2C2	AC026470.2	0.597601771	4.22E-53	postive
RORA	AC026470.2	0.733574876	1.85E-91	postive
SOS1	AC026470.2	0.605206072	9.16E-55	postive
SOS2	AC026470.2	0.528652607	7.53E-40	postive
BRAF	AC026470.2	0.683791542	5.64E-75	postive
CBL	AC026470.2	0.597503753	4.43E-53	postive
CBLB	AC026470.2	0.6349966	9.74E-62	postive
PDK1	AC026470.2	0.549565036	1.51E-43	postive
TRAJ1	AC026470.2	0.711242628	1.25E-83	postive
TRAJ2	AC026470.2	0.677022065	5.51E-73	postive
TRAJ3	AC026470.2	0.792805631	1.05E-116	postive
TRAJ5	AC026470.2	0.721724788	3.28E-87	postive
TRAJ6	AC026470.2	0.794767453	1.11E-117	postive
TRAJ8	AC026470.2	0.742587575	7.55E-95	postive
TRAJ10	AC026470.2	0.792087684	2.37E-116	postive
TRAJ12	AC026470.2	0.683469025	7.04E-75	postive
TRAJ13	AC026470.2	0.762876981	5.06E-103	postive
TRAJ14	AC026470.2	0.746647435	2.02E-96	postive
TRAJ16	AC026470.2	0.80026431	1.79E-120	postive

TRAJ17	AC026470.2	0.781535919	2.67E-111	postive
TRAJ18	AC026470.2	0.751601639	2.21E-98	postive
TRAJ21	AC026470.2	0.805214141	4.61E-123	postive
TRAJ31	AC026470.2	0.780590531	7.33E-111	postive
TRAJ37	AC026470.2	0.757076424	1.33E-100	postive
TRAJ38	AC026470.2	0.811835313	1.21E-126	postive
TRAJ39	AC026470.2	0.795507454	4.72E-118	postive
LMBR1L	AL360181.2	0.500022058	3.45E-35	postive
TYK2	AL360181.2	0.518149584	4.35E-38	postive
CREB1	AL133371.2	0.561586939	8.60E-46	postive
CYLD	AL133371.2	0.584939842	1.99E-50	postive
IL7R	AL133371.2	0.545487963	8.35E-43	postive
PIK3CG	AL133371.2	0.590657566	1.28E-51	postive
JAK2	AL133371.2	0.587035646	7.33E-51	postive
NFAT5	AL133371.2	0.558185698	3.80E-45	postive
NFATC2	AL133371.2	0.600631126	9.29E-54	postive
PIK3R1	AL133371.2	0.66664028	4.91E-70	postive
PIK3CA	AL133371.2	0.502660554	1.34E-35	postive
PLXNC1	AL133371.2	0.531417833	2.53E-40	postive
IL6ST	AL133371.2	0.597153884	5.28E-53	postive
TNFSF8	AL133371.2	0.531949803	2.05E-40	postive
ANGPTL1	AL133371.2	0.654040225	1.30E-66	postive
BMPR2	AL133371.2	0.557480497	5.15E-45	postive
LIFR	AL133371.2	0.521096354	1.41E-38	postive
RORA	AL133371.2	0.68950435	1.07E-76	postive
BRAF	AL133371.2	0.639721502	6.48E-63	postive
PTPRC	AL133371.2	0.575159226	1.93E-48	postive
ITK	AL133371.2	0.685310066	1.98E-75	postive
CD28	AL133371.2	0.60876727	1.47E-55	postive
CBL	AL133371.2	0.506318868	3.55E-36	postive
CBLB	AL133371.2	0.631639802	6.50E-61	postive
TRAJ2	AL133371.2	0.561529772	8.82E-46	postive
TRAJ3	AL133371.2	0.537234098	2.46E-41	postive
TRAJ5	AL133371.2	0.611246996	4.05E-56	postive
TRAJ6	AL133371.2	0.514252739	1.89E-37	postive
TRAJ13	AL133371.2	0.584716129	2.22E-50	postive
TRAJ14	AL133371.2	0.564756139	2.12E-46	postive
TRAJ18	AL133371.2	0.557682257	4.72E-45	postive
TRAJ21	AL133371.2	0.602384576	3.84E-54	postive
TRAJ31	AL133371.2	0.584716104	2.22E-50	postive
TRAJ37	AL133371.2	0.565966365	1.24E-46	postive
TRAJ38	AL133371.2	0.610630832	5.59E-56	postive
TRAJ39	AL133371.2	0.569360841	2.71E-47	postive
CREB1	AC090739.1	0.763049295	4.28E-103	postive
RFXAP	AC090739.1	0.518357647	4.02E-38	postive
UBR1	AC090739.1	0.64526985	2.53E-64	postive
ZC3HAV1	AC090739.1	0.563469584	3.75E-46	postive
CYLD	AC090739.1	0.609197469	1.18E-55	postive
EIF2AK2	AC090739.1	0.54612754	6.40E-43	postive
MAPK8	AC090739.1	0.55802903	4.06E-45	postive
LMBR1	AC090739.1	0.559381386	2.26E-45	postive
IREB2	AC090739.1	0.598424554	2.80E-53	postive
DDX17	AC090739.1	0.63608538	5.24E-62	postive
PIK3CG	AC090739.1	0.514256022	1.89E-37	postive
JAK2	AC090739.1	0.585167979	1.79E-50	postive
TXK	AC090739.1	0.597571572	4.29E-53	postive
NFAT5	AC090739.1	0.877650609	2.29E-172	postive
NFATC3	AC090739.1	0.532955558	1.37E-40	postive
MALT1	AC090739.1	0.544944146	1.05E-42	postive
PIK3R1	AC090739.1	0.653329893	2.01E-66	postive
PIK3CA	AC090739.1	0.653867887	1.45E-66	postive
AKT3	AC090739.1	0.572337307	7.03E-48	postive
IGHD4-4	AC090739.1	0.51524899	1.30E-37	postive
IGHD6-6	AC090739.1	0.570821299	1.40E-47	postive
GNRH1	AC090739.1	0.758558137	3.25E-101	postive

IL6ST	AC090739.1	0.677061566	5.36E-73	postive
RABEP1	AC090739.1	0.558183151	3.80E-45	postive
ACVR2A	AC090739.1	0.634450823	1.33E-61	postive
ANGPTL1	AC090739.1	0.719972248	1.34E-86	postive
BMPR1A	AC090739.1	0.543773472	1.70E-42	postive
BMPR2	AC090739.1	0.723448815	8.16E-88	postive
CRLF3	AC090739.1	0.66854949	1.44E-70	postive
NR2C2	AC090739.1	0.686873069	6.72E-76	postive
RORA	AC090739.1	0.869853908	1.07E-165	postive
SOS1	AC090739.1	0.667256544	3.30E-70	postive
SOS2	AC090739.1	0.556232553	8.83E-45	postive
BRAF	AC090739.1	0.816652119	2.43E-129	postive
ITK	AC090739.1	0.509635623	1.05E-36	postive
CBL	AC090739.1	0.572903161	5.43E-48	postive
CBLB	AC090739.1	0.656980712	2.14E-67	postive
RASGRP1	AC090739.1	0.507688865	2.15E-36	postive
PDK1	AC090739.1	0.550096625	1.21E-43	postive
TRAJ1	AC090739.1	0.736282462	1.83E-92	postive
TRAJ2	AC090739.1	0.750186015	8.12E-98	postive
TRAJ3	AC090739.1	0.782197434	1.31E-111	postive
TRAJ5	AC090739.1	0.811944048	1.05E-126	postive
TRAJ6	AC090739.1	0.787013851	6.91E-114	postive
TRAJ8	AC090739.1	0.808000505	1.49E-124	postive
TRAJ10	AC090739.1	0.751940083	1.62E-98	postive
TRAJ12	AC090739.1	0.66281396	5.60E-69	postive
TRAJ13	AC090739.1	0.82553768	1.57E-134	postive
TRAJ14	AC090739.1	0.765939143	2.50E-104	postive
TRAJ16	AC090739.1	0.787130363	6.08E-114	postive
TRAJ17	AC090739.1	0.741911165	1.37E-94	postive
TRAJ18	AC090739.1	0.69647608	7.47E-79	postive
TRAJ21	AC090739.1	0.829090408	1.09E-136	postive
TRAJ31	AC090739.1	0.838973904	5.77E-143	postive
TRAJ37	AC090739.1	0.846766425	3.21E-148	postive
TRAJ38	AC090739.1	0.837046486	1.04E-141	postive
TRAJ39	AC090739.1	0.867657996	6.81E-164	postive
NR0B1	LINC00942	0.500593045	2.81E-35	postive
FAM3D	AL158206.1	0.509155408	1.25E-36	postive
IL22RA1	AL158206.1	0.637685404	2.10E-62	postive
GNRH1	AL445222.2	0.578802415	3.58E-49	postive
NR2C2	AL445222.2	0.545177233	9.50E-43	postive
BRAF	AL445222.2	0.582438388	6.52E-50	postive
CREB1	AF129075.2	0.789459024	4.57E-115	postive
RFXAP	AF129075.2	0.527066151	1.40E-39	postive
UBR1	AF129075.2	0.646507423	1.21E-64	postive
ZC3HAV1	AF129075.2	0.567300808	6.83E-47	postive
IL15	AF129075.2	0.531717916	2.24E-40	postive
CYLD	AF129075.2	0.630232339	1.43E-60	postive
EIF2AK2	AF129075.2	0.560929535	1.15E-45	postive
MAPK8	AF129075.2	0.535886748	4.24E-41	postive
NFKBIZ	AF129075.2	0.512360919	3.83E-37	postive
LMBR1	AF129075.2	0.553752819	2.56E-44	postive
IREB2	AF129075.2	0.5943243	2.13E-52	postive
DDX17	AF129075.2	0.67804068	2.79E-73	postive
PIK3CG	AF129075.2	0.526529602	1.73E-39	postive
JAK2	AF129075.2	0.67572915	1.30E-72	postive
TXK	AF129075.2	0.578141469	4.87E-49	postive
NFAT5	AF129075.2	0.843144562	9.65E-146	postive
MALT1	AF129075.2	0.538387857	1.54E-41	postive
PIK3R1	AF129075.2	0.625040448	2.53E-59	postive
PIK3CA	AF129075.2	0.673303467	6.48E-72	postive
PIK3CB	AF129075.2	0.519586298	2.52E-38	postive
RASGRP3	AF129075.2	0.547940443	3.00E-43	postive
PLXNC1	AF129075.2	0.523868002	4.86E-39	postive
GNRH1	AF129075.2	0.771427424	1.02E-106	postive
IL6ST	AF129075.2	0.62357089	5.65E-59	postive

RABEP1	AF129075.2	0.57192506	8.48E-48	postive
ACVR2A	AF129075.2	0.655937712	4.07E-67	postive
ANGPTL1	AF129075.2	0.746073881	3.38E-96	postive
BMPR1A	AF129075.2	0.540855903	5.64E-42	postive
BMPR2	AF129075.2	0.715557028	4.38E-85	postive
CRLF3	AF129075.2	0.672046708	1.48E-71	postive
NR1D2	AF129075.2	0.534280355	8.08E-41	postive
NR2C1	AF129075.2	0.534464006	7.51E-41	postive
NR2C2	AF129075.2	0.74454244	1.33E-95	postive
RORA	AF129075.2	0.830445271	1.59E-137	postive
SOS1	AF129075.2	0.700481308	4.04E-80	postive
SOS2	AF129075.2	0.594500053	1.96E-52	postive
BRAF	AF129075.2	0.79623456	2.03E-118	postive
CBL	AF129075.2	0.589158001	2.64E-51	postive
CBLB	AF129075.2	0.742767817	6.44E-95	postive
RASGRP1	AF129075.2	0.535716286	4.54E-41	postive
PDK1	AF129075.2	0.560543072	1.36E-45	postive
TRAJ1	AF129075.2	0.762663887	6.22E-103	postive
TRAJ2	AF129075.2	0.722088764	2.45E-87	postive
TRAJ3	AF129075.2	0.750339962	7.05E-98	postive
TRAJ5	AF129075.2	0.813465497	1.51E-127	postive
TRAJ6	AF129075.2	0.796143848	2.26E-118	postive
TRAJ8	AF129075.2	0.753988954	2.42E-99	postive
TRAJ10	AF129075.2	0.776722839	4.35E-109	postive
TRAJ12	AF129075.2	0.734239008	1.05E-91	postive
TRAJ13	AF129075.2	0.788623021	1.16E-114	postive
TRAJ14	AF129075.2	0.772888585	2.30E-107	postive
TRAJ16	AF129075.2	0.803092725	6.06E-122	postive
TRAJ17	AF129075.2	0.740538726	4.58E-94	postive
TRAJ18	AF129075.2	0.731956764	7.24E-91	postive
TRAJ21	AF129075.2	0.802647819	1.04E-121	postive
TRAJ31	AF129075.2	0.835630036	8.54E-141	postive
TRAJ37	AF129075.2	0.792360037	1.74E-116	postive
TRAJ38	AF129075.2	0.866280368	8.85E-163	postive
TRAJ39	AF129075.2	0.806234672	1.32E-123	postive
CREB1	AC090948.3	0.725841521	1.16E-88	postive
RFXAP	AC090948.3	0.548648366	2.23E-43	postive
UBR1	AC090948.3	0.600288332	1.10E-53	postive
ZC3HAV1	AC090948.3	0.563065556	4.49E-46	postive
IL15	AC090948.3	0.662779784	5.72E-69	postive
CYLD	AC090948.3	0.659824236	3.66E-68	postive
EIF2AK2	AC090948.3	0.517639689	5.27E-38	postive
MAPK8	AC090948.3	0.532914476	1.39E-40	postive
NFKBIZ	AC090948.3	0.510742639	6.98E-37	postive
IREB2	AC090948.3	0.560652451	1.30E-45	postive
DDX17	AC090948.3	0.672337355	1.22E-71	postive
PIK3CG	AC090948.3	0.563986581	2.99E-46	postive
JAK2	AC090948.3	0.662552935	6.60E-69	postive
TXK	AC090948.3	0.621108726	2.15E-58	postive
NFAT5	AC090948.3	0.823821594	1.66E-133	postive
MALT1	AC090948.3	0.536736261	3.01E-41	postive
PIK3R1	AC090948.3	0.634478273	1.31E-61	postive
PIK3CA	AC090948.3	0.648915895	2.89E-65	postive
AKT3	AC090948.3	0.524648041	3.59E-39	postive
RASGRP3	AC090948.3	0.60523435	9.03E-55	postive
IGHD4-4	AC090948.3	0.52350969	5.58E-39	postive
IGHD6-6	AC090948.3	0.578567686	4.00E-49	postive
LTB4R2	AC090948.3	0.545691134	7.67E-43	postive
PLXNC1	AC090948.3	0.536914647	2.80E-41	postive
GNRH1	AC090948.3	0.785264767	4.72E-113	postive
IL6ST	AC090948.3	0.602756616	3.18E-54	postive
RABEP1	AC090948.3	0.551688672	6.17E-44	postive
ACVR2A	AC090948.3	0.59330399	3.52E-52	postive
ANGPTL1	AC090948.3	0.74816299	5.13E-97	postive
BMPR2	AC090948.3	0.659251945	5.23E-68	postive

CRLF3	AC090948.3	0.639388356	7.86E-63	postive
IL18R1	AC090948.3	0.506257775	3.63E-36	postive
NR2C1	AC090948.3	0.523212461	6.26E-39	postive
NR2C2	AC090948.3	0.774197683	5.98E-108	postive
RORA	AC090948.3	0.828130468	4.22E-136	postive
SOS1	AC090948.3	0.659968055	3.34E-68	postive
SOS2	AC090948.3	0.553305125	3.10E-44	postive
BRAF	AC090948.3	0.788782575	9.73E-115	postive
PTPRC	AC090948.3	0.513901605	2.15E-37	postive
ITK	AC090948.3	0.645418491	2.31E-64	postive
CD28	AC090948.3	0.567119158	7.41E-47	postive
CBL	AC090948.3	0.626579817	1.09E-59	postive
CBLB	AC090948.3	0.739612027	1.03E-93	postive
RASGRP1	AC090948.3	0.544853747	1.09E-42	postive
PDK1	AC090948.3	0.604070075	1.63E-54	postive
TRAJ1	AC090948.3	0.765060266	5.96E-104	postive
TRAJ2	AC090948.3	0.780053105	1.30E-110	postive
TRAJ3	AC090948.3	0.842725895	1.85E-145	postive
TRAJ5	AC090948.3	0.776593843	4.97E-109	postive
TRAJ6	AC090948.3	0.791193041	6.52E-116	postive
TRAJ8	AC090948.3	0.749654749	1.32E-97	postive
TRAJ10	AC090948.3	0.77302575	2.00E-107	postive
TRAJ12	AC090948.3	0.715659902	4.04E-85	postive
TRAJ13	AC090948.3	0.759287151	1.62E-101	postive
TRAJ14	AC090948.3	0.771175654	1.32E-106	postive
TRAJ16	AC090948.3	0.781342422	3.28E-111	postive
TRAJ17	AC090948.3	0.749749439	1.21E-97	postive
TRAJ18	AC090948.3	0.761218376	2.53E-102	postive
TRAJ21	AC090948.3	0.798800747	1.01E-119	postive
TRAJ31	AC090948.3	0.801147352	6.26E-121	postive
TRAJ37	AC090948.3	0.797249989	6.23E-119	postive
TRAJ38	AC090948.3	0.84747156	1.04E-148	postive
TRAJ39	AC090948.3	0.821263151	5.34E-132	postive
CREB1	AL162724.1	0.756878622	1.60E-100	postive
UBR1	AL162724.1	0.633892185	1.82E-61	postive
ZC3HAV1	AL162724.1	0.549601865	1.49E-43	postive
ZC3HAV1L	AL162724.1	0.540975424	5.37E-42	postive
IL15	AL162724.1	0.648356256	4.04E-65	postive
CYLD	AL162724.1	0.549302997	1.69E-43	postive
MAPK8	AL162724.1	0.54200402	3.52E-42	postive
TLR1	AL162724.1	0.514348987	1.82E-37	postive
NFKBIZ	AL162724.1	0.501622538	1.94E-35	postive
LMBR1	AL162724.1	0.576459515	1.06E-48	postive
LIMS1	AL162724.1	0.5001038	3.35E-35	postive
IREB2	AL162724.1	0.603282821	2.44E-54	postive
DDX17	AL162724.1	0.531609244	2.34E-40	postive
PIK3CG	AL162724.1	0.528080307	9.43E-40	postive
JAK2	AL162724.1	0.62578011	1.69E-59	postive
TXK	AL162724.1	0.572172966	7.58E-48	postive
NFAT5	AL162724.1	0.759284883	1.62E-101	postive
MALT1	AL162724.1	0.530902511	3.10E-40	postive
PIK3R1	AL162724.1	0.58944681	2.30E-51	postive
PIK3CA	AL162724.1	0.698393029	1.86E-79	postive
PIK3CB	AL162724.1	0.558690543	3.05E-45	postive
RASGRP3	AL162724.1	0.510165405	8.64E-37	postive
IGHD4-4	AL162724.1	0.513660977	2.36E-37	postive
GNRH1	AL162724.1	0.714669639	8.77E-85	postive
IL6ST	AL162724.1	0.564719909	2.16E-46	postive
IL7	AL162724.1	0.520300049	1.92E-38	postive
RABEP1	AL162724.1	0.537053588	2.65E-41	postive
ACVR2A	AL162724.1	0.60520783	9.15E-55	postive
ANGPTL1	AL162724.1	0.761203982	2.56E-102	postive
BMPR1A	AL162724.1	0.539208644	1.10E-41	postive
BMPR2	AL162724.1	0.69587743	1.15E-78	postive
CRLF3	AL162724.1	0.606266885	5.32E-55	postive

IL18R1	AL162724.1	0.509313859	1.18E-36	postive
NR2C2	AL162724.1	0.66675245	4.57E-70	postive
RORA	AL162724.1	0.752708322	7.94E-99	postive
SOS1	AL162724.1	0.625963042	1.53E-59	postive
SOS2	AL162724.1	0.565906283	1.27E-46	postive
BRAF	AL162724.1	0.724734237	2.87E-88	postive
CBL	AL162724.1	0.585350976	1.64E-50	postive
CBLB	AL162724.1	0.668264007	1.73E-70	postive
RASGRP1	AL162724.1	0.504280077	7.45E-36	postive
PDK1	AL162724.1	0.623733857	5.17E-59	postive
TRAJ1	AL162724.1	0.656698083	2.55E-67	postive
TRAJ2	AL162724.1	0.609449839	1.03E-55	postive
TRAJ3	AL162724.1	0.736717479	1.26E-92	postive
TRAJ5	AL162724.1	0.712128879	6.29E-84	postive
TRAJ6	AL162724.1	0.726096379	9.43E-89	postive
TRAJ8	AL162724.1	0.66577992	8.51E-70	postive
TRAJ10	AL162724.1	0.745161683	7.66E-96	postive
TRAJ12	AL162724.1	0.628923542	2.97E-60	postive
TRAJ13	AL162724.1	0.682585269	1.29E-74	postive
TRAJ14	AL162724.1	0.672974556	8.04E-72	postive
TRAJ16	AL162724.1	0.709084057	6.49E-83	postive
TRAJ17	AL162724.1	0.715756491	3.75E-85	postive
TRAJ18	AL162724.1	0.664592276	1.81E-69	postive
TRAJ21	AL162724.1	0.723846177	5.91E-88	postive
TRAJ31	AL162724.1	0.750913721	4.16E-98	postive
TRAJ37	AL162724.1	0.707885577	1.61E-82	postive
TRAJ38	AL162724.1	0.773922369	7.94E-108	postive
TRAJ39	AL162724.1	0.751358169	2.77E-98	postive
CREB1	NPTN-IT1	0.800563548	1.26E-120	postive
RFXAP	NPTN-IT1	0.515753091	1.08E-37	postive
UBR1	NPTN-IT1	0.672412468	1.16E-71	postive
ZC3HAV1	NPTN-IT1	0.582016434	7.95E-50	postive
ZC3HAV1L	NPTN-IT1	0.545825441	7.25E-43	postive
IL15	NPTN-IT1	0.63216182	4.84E-61	postive
CYLD	NPTN-IT1	0.639296743	8.28E-63	postive
EIF2AK2	NPTN-IT1	0.580330538	1.75E-49	postive
MAPK8	NPTN-IT1	0.556963077	6.44E-45	postive
NFKBIZ	NPTN-IT1	0.522624433	7.86E-39	postive
LMBR1	NPTN-IT1	0.593177527	3.75E-52	postive
LIMS1	NPTN-IT1	0.525139992	2.97E-39	postive
IREB2	NPTN-IT1	0.63820584	1.55E-62	postive
DDX17	NPTN-IT1	0.657349255	1.70E-67	postive
PIK3CG	NPTN-IT1	0.562761632	5.13E-46	postive
JAK2	NPTN-IT1	0.701335714	2.16E-80	postive
TXK	NPTN-IT1	0.624312072	3.77E-59	postive
NFAT5	NPTN-IT1	0.857664582	4.39E-156	postive
MALT1	NPTN-IT1	0.557218762	5.77E-45	postive
PIK3R1	NPTN-IT1	0.659439693	4.65E-68	postive
PIK3CA	NPTN-IT1	0.711673385	8.94E-84	postive
PIK3CB	NPTN-IT1	0.557835928	4.42E-45	postive
AKT3	NPTN-IT1	0.506097967	3.85E-36	postive
RASGRP3	NPTN-IT1	0.582462652	6.44E-50	postive
PLXNC1	NPTN-IT1	0.534226978	8.25E-41	postive
GNRH1	NPTN-IT1	0.769278288	8.96E-106	postive
IL6ST	NPTN-IT1	0.61411056	9.02E-57	postive
RABEP1	NPTN-IT1	0.579429756	2.67E-49	postive
ACVR2A	NPTN-IT1	0.630868314	1.00E-60	postive
ANGPTL1	NPTN-IT1	0.790833188	9.78E-116	postive
BMPR1A	NPTN-IT1	0.520496006	1.78E-38	postive
BMPR2	NPTN-IT1	0.725385127	1.69E-88	postive
CRLF3	NPTN-IT1	0.646413941	1.28E-64	postive
LIFR	NPTN-IT1	0.500465786	2.94E-35	postive
NR1D2	NPTN-IT1	0.523542973	5.51E-39	postive
NR2C2	NPTN-IT1	0.750193628	8.06E-98	postive
RORA	NPTN-IT1	0.849434033	4.35E-150	postive

SOS1	NPTN-IT1	0.717651028	8.44E-86	postive
SOS2	NPTN-IT1	0.608623743	1.58E-55	postive
BRAF	NPTN-IT1	0.810051542	1.15E-125	postive
ITK	NPTN-IT1	0.533608032	1.06E-40	postive
TEC	NPTN-IT1	0.550174055	1.17E-43	postive
CD28	NPTN-IT1	0.500334056	3.08E-35	postive
CBL	NPTN-IT1	0.651018198	8.16E-66	postive
CBLB	NPTN-IT1	0.795682036	3.85E-118	postive
RASGRP1	NPTN-IT1	0.550401413	1.06E-43	postive
PDK1	NPTN-IT1	0.620856504	2.47E-58	postive
TRAJ1	NPTN-IT1	0.769207733	9.62E-106	postive
TRAJ2	NPTN-IT1	0.732228635	5.76E-91	postive
TRAJ3	NPTN-IT1	0.799404606	4.96E-120	postive
TRAJ5	NPTN-IT1	0.82411935	1.10E-133	postive
TRAJ6	NPTN-IT1	0.813929902	8.29E-128	postive
TRAJ8	NPTN-IT1	0.74249253	8.21E-95	postive
TRAJ10	NPTN-IT1	0.798673249	1.18E-119	postive
TRAJ12	NPTN-IT1	0.771374793	1.08E-106	postive
TRAJ13	NPTN-IT1	0.784886524	7.13E-113	postive
TRAJ14	NPTN-IT1	0.792872942	9.70E-117	postive
TRAJ16	NPTN-IT1	0.788907826	8.46E-115	postive
TRAJ17	NPTN-IT1	0.754338725	1.74E-99	postive
TRAJ18	NPTN-IT1	0.781113086	4.20E-111	postive
TRAJ21	NPTN-IT1	0.824833244	4.14E-134	postive
TRAJ31	NPTN-IT1	0.841416619	1.40E-144	postive
TRAJ37	NPTN-IT1	0.814913216	2.33E-128	postive
TRAJ38	NPTN-IT1	0.881490749	7.99E-176	postive
TRAJ39	NPTN-IT1	0.831563582	3.20E-138	postive
BIRC5	RNASEH1-AS1	0.527607504	1.13E-39	postive
CREB1	AC098851.1	0.736042086	2.25E-92	postive
RFXAP	AC098851.1	0.533017641	1.34E-40	postive
UBR1	AC098851.1	0.620867057	2.45E-58	postive
ZC3HAV1	AC098851.1	0.53067287	3.40E-40	postive
IL15	AC098851.1	0.526598904	1.68E-39	postive
CYLD	AC098851.1	0.620595527	2.84E-58	postive
EIF2AK2	AC098851.1	0.50958826	1.07E-36	postive
LMBR1	AC098851.1	0.522071576	9.72E-39	postive
IREB2	AC098851.1	0.561984179	7.22E-46	postive
DDX17	AC098851.1	0.670296754	4.63E-71	postive
PIK3CG	AC098851.1	0.506791493	2.99E-36	postive
JAK2	AC098851.1	0.613757087	1.09E-56	postive
TXK	AC098851.1	0.626794401	9.65E-60	postive
NFAT5	AC098851.1	0.832038406	1.62E-138	postive
MALT1	AC098851.1	0.518741001	3.47E-38	postive
PIK3R1	AC098851.1	0.654364901	1.07E-66	postive
PIK3CA	AC098851.1	0.656891663	2.26E-67	postive
RASGRP3	AC098851.1	0.506853543	2.92E-36	postive
IGHD4-4	AC098851.1	0.520987539	1.47E-38	postive
IGHD6-6	AC098851.1	0.565763929	1.36E-46	postive
LTB4R2	AC098851.1	0.539550864	9.61E-42	postive
GNRH1	AC098851.1	0.776148477	7.92E-109	postive
IL6ST	AC098851.1	0.630032591	1.60E-60	postive
RABEP1	AC098851.1	0.55700772	6.32E-45	postive
ACVR2A	AC098851.1	0.654345517	1.08E-66	postive
ANGPTL1	AC098851.1	0.709511632	4.69E-83	postive
BMPR1A	AC098851.1	0.503924097	8.47E-36	postive
BMPR2	AC098851.1	0.715451571	4.76E-85	postive
CRLF3	AC098851.1	0.587394934	6.17E-51	postive
NR2C2	AC098851.1	0.712765034	3.85E-84	postive
RORA	AC098851.1	0.856958111	1.49E-155	postive
SOS1	AC098851.1	0.634390255	1.38E-61	postive
SOS2	AC098851.1	0.571835217	8.84E-48	postive
BRAF	AC098851.1	0.741185065	2.60E-94	postive
ITK	AC098851.1	0.511984145	4.40E-37	postive
CBL	AC098851.1	0.557150161	5.94E-45	postive

CBLB	AC098851.1	0.685572443	1.66E-75	postive
PDK1	AC098851.1	0.526492132	1.75E-39	postive
TRAJ1	AC098851.1	0.675441212	1.58E-72	postive
TRAJ2	AC098851.1	0.673345037	6.30E-72	postive
TRAJ3	AC098851.1	0.730605088	2.25E-90	postive
TRAJ5	AC098851.1	0.727198345	3.81E-89	postive
TRAJ6	AC098851.1	0.714498224	1.00E-84	postive
TRAJ8	AC098851.1	0.689354061	1.19E-76	postive
TRAJ10	AC098851.1	0.712829954	3.66E-84	postive
TRAJ12	AC098851.1	0.635931354	5.72E-62	postive
TRAJ13	AC098851.1	0.697498092	3.57E-79	postive
TRAJ14	AC098851.1	0.663458064	3.73E-69	postive
TRAJ16	AC098851.1	0.730528611	2.40E-90	postive
TRAJ17	AC098851.1	0.694557906	2.97E-78	postive
TRAJ18	AC098851.1	0.677715024	3.47E-73	postive
TRAJ21	AC098851.1	0.730834623	1.86E-90	postive
TRAJ31	AC098851.1	0.763170354	3.80E-103	postive
TRAJ37	AC098851.1	0.75949691	1.32E-101	postive
TRAJ38	AC098851.1	0.777858349	1.32E-109	postive
TRAJ39	AC098851.1	0.772648528	2.94E-107	postive
NFAT5	LINC01184	0.525516016	2.57E-39	postive
RORA	LINC01184	0.526302649	1.89E-39	postive
BRAF	LINC01184	0.505402676	4.95E-36	postive
TRAJ38	LINC01184	0.517015135	6.68E-38	postive
TRAJ39	LINC01184	0.505664074	4.50E-36	postive
CREB1	AC009090.1	0.553109827	3.37E-44	postive
UBR1	AC009090.1	0.518771957	3.43E-38	postive
DDX17	AC009090.1	0.644008367	5.31E-64	postive
TXK	AC009090.1	0.543477572	1.92E-42	postive
NFAT5	AC009090.1	0.638654354	1.20E-62	postive
PIK3R1	AC009090.1	0.50181197	1.81E-35	postive
ROBO2	AC009090.1	0.511483883	5.30E-37	postive
GNRH1	AC009090.1	0.77713614	2.82E-109	postive
IL6ST	AC009090.1	0.524757053	3.45E-39	postive
ACVR2A	AC009090.1	0.507523718	2.28E-36	postive
ANGPTL1	AC009090.1	0.553265315	3.15E-44	postive
BMPR2	AC009090.1	0.535792405	4.40E-41	postive
NR2C2	AC009090.1	0.649778657	1.72E-65	postive
RORA	AC009090.1	0.637680787	2.10E-62	postive
BRAF	AC009090.1	0.612407119	2.21E-56	postive
CBLB	AC009090.1	0.539101532	1.15E-41	postive
TRAJ3	AC009090.1	0.500981932	2.44E-35	postive
TRAJ31	AC009090.1	0.519552665	2.55E-38	postive
TRAJ37	AC009090.1	0.501304586	2.18E-35	postive
TRAJ38	AC009090.1	0.534415565	7.65E-41	postive
IL1R2	C8orf31	0.512134955	4.16E-37	postive
CREB1	AC112722.1	0.521951863	1.02E-38	postive
UBR1	AC112722.1	0.521792526	1.08E-38	postive
DDX17	AC112722.1	0.619742397	4.50E-58	postive
TXK	AC112722.1	0.522434382	8.45E-39	postive
NFAT5	AC112722.1	0.573561081	4.02E-48	postive
PIK3R1	AC112722.1	0.575438531	1.70E-48	postive
LTB4R2	AC112722.1	0.53363302	1.05E-40	postive
GNRH1	AC112722.1	0.631249816	8.09E-61	postive
IL6ST	AC112722.1	0.552924882	3.65E-44	postive
ANGPTL1	AC112722.1	0.537472188	2.24E-41	postive
NR2C2	AC112722.1	0.635800341	6.17E-62	postive
RORA	AC112722.1	0.60959222	9.58E-56	postive
BRAF	AC112722.1	0.615055915	5.48E-57	postive
TRAJ2	AC112722.1	0.527959367	9.89E-40	postive
TRAJ3	AC112722.1	0.558622991	3.14E-45	postive
TRAJ6	AC112722.1	0.507687972	2.15E-36	postive
TRAJ12	AC112722.1	0.504290265	7.42E-36	postive
TRAJ14	AC112722.1	0.501205726	2.26E-35	postive
TRAJ18	AC112722.1	0.502239472	1.56E-35	postive

TRAJ21	AC112722.1	0.509289162	1.19E-36	postive
TRAJ31	AC112722.1	0.506244942	3.64E-36	postive
TRAJ37	AC112722.1	0.50986033	9.67E-37	postive
TRAJ38	AC112722.1	0.557236435	5.73E-45	postive
TRAJ39	AC112722.1	0.526403281	1.82E-39	postive
GCG	MIR1915HG	0.527057739	1.41E-39	postive
CREB1	AC027277.2	0.673349819	6.28E-72	postive
UBR1	AC027277.2	0.622101016	1.26E-58	postive
ZC3HAV1	AC027277.2	0.517771288	5.02E-38	postive
IL15	AC027277.2	0.517116044	6.43E-38	postive
CYLD	AC027277.2	0.506562977	3.25E-36	postive
EIF2AK2	AC027277.2	0.523342335	5.96E-39	postive
IREB2	AC027277.2	0.556502123	7.86E-45	postive
DDX17	AC027277.2	0.601367786	6.42E-54	postive
JAK2	AC027277.2	0.537106453	2.59E-41	postive
TXK	AC027277.2	0.628750167	3.27E-60	postive
NFAT5	AC027277.2	0.716306362	2.43E-85	postive
PIK3R1	AC027277.2	0.615183299	5.12E-57	postive
PIK3CA	AC027277.2	0.596517376	7.23E-53	postive
PIK3CB	AC027277.2	0.502620679	1.36E-35	postive
IGHD4-4	AC027277.2	0.509487491	1.11E-36	postive
IGHD6-6	AC027277.2	0.547464155	3.66E-43	postive
GNRH1	AC027277.2	0.753485425	3.86E-99	postive
IL6ST	AC027277.2	0.56608968	1.17E-46	postive
RABEP1	AC027277.2	0.51285393	3.19E-37	postive
ACVR2A	AC027277.2	0.577938059	5.35E-49	postive
ANGPTL1	AC027277.2	0.673468261	5.81E-72	postive
BMPR2	AC027277.2	0.674266792	3.43E-72	postive
CRLF3	AC027277.2	0.520123977	2.05E-38	postive
NR2C2	AC027277.2	0.702655511	8.13E-81	postive
RORA	AC027277.2	0.731529223	1.04E-90	postive
SOS1	AC027277.2	0.579395038	2.72E-49	postive
BRAF	AC027277.2	0.720992666	5.91E-87	postive
TEC	AC027277.2	0.602706366	3.26E-54	postive
CBL	AC027277.2	0.524800978	3.39E-39	postive
CBLB	AC027277.2	0.619296209	5.72E-58	postive
RASGRP1	AC027277.2	0.52055152	1.74E-38	postive
PDK1	AC027277.2	0.507120085	2.65E-36	postive
TRAJ1	AC027277.2	0.603014181	2.79E-54	postive
TRAJ2	AC027277.2	0.543587999	1.83E-42	postive
TRAJ3	AC027277.2	0.680288446	6.13E-74	postive
TRAJ5	AC027277.2	0.655942333	4.06E-67	postive
TRAJ6	AC027277.2	0.6489802	2.78E-65	postive
TRAJ8	AC027277.2	0.593661229	2.96E-52	postive
TRAJ10	AC027277.2	0.620415648	3.13E-58	postive
TRAJ12	AC027277.2	0.633712116	2.02E-61	postive
TRAJ13	AC027277.2	0.599722276	1.47E-53	postive
TRAJ14	AC027277.2	0.610213304	6.94E-56	postive
TRAJ16	AC027277.2	0.609624091	9.42E-56	postive
TRAJ17	AC027277.2	0.617840099	1.25E-57	postive
TRAJ18	AC027277.2	0.631166994	8.47E-61	postive
TRAJ21	AC027277.2	0.665861481	8.08E-70	postive
TRAJ31	AC027277.2	0.667446464	2.92E-70	postive
TRAJ37	AC027277.2	0.650039705	1.47E-65	postive
TRAJ38	AC027277.2	0.687192488	5.38E-76	postive
TRAJ39	AC027277.2	0.699642479	7.48E-80	postive
CREB1	AC002044.1	0.660367701	2.61E-68	postive
UBR1	AC002044.1	0.556177937	9.04E-45	postive
CYLD	AC002044.1	0.514194667	1.93E-37	postive
MAPK8	AC002044.1	0.522790213	7.37E-39	postive
IREB2	AC002044.1	0.515454952	1.20E-37	postive
DDX17	AC002044.1	0.548440972	2.43E-43	postive
TXK	AC002044.1	0.526964424	1.46E-39	postive
NFAT5	AC002044.1	0.778808381	4.86E-110	postive
PIK3R1	AC002044.1	0.568703939	3.64E-47	postive

PIK3CA	AC002044.1	0.530738526	3.31E-40	postive
AKT3	AC002044.1	0.5597054	1.96E-45	postive
GNRH1	AC002044.1	0.709381084	5.18E-83	postive
IL6ST	AC002044.1	0.582551942	6.18E-50	postive
ACVR2A	AC002044.1	0.587649465	5.46E-51	postive
ANGPTL1	AC002044.1	0.684282262	4.03E-75	postive
BMPR1A	AC002044.1	0.532225555	1.83E-40	postive
BMPR2	AC002044.1	0.647187825	8.10E-65	postive
CRLF3	AC002044.1	0.584427475	2.54E-50	postive
NR2C2	AC002044.1	0.570768732	1.43E-47	postive
RORA	AC002044.1	0.76246424	7.56E-103	postive
SOS1	AC002044.1	0.561397128	9.35E-46	postive
BRAF	AC002044.1	0.766202782	1.93E-104	postive
CBLB	AC002044.1	0.590603424	1.31E-51	postive
TRAJ1	AC002044.1	0.629645629	1.98E-60	postive
TRAJ2	AC002044.1	0.730182613	3.21E-90	postive
TRAJ3	AC002044.1	0.733918561	1.38E-91	postive
TRAJ5	AC002044.1	0.680646091	4.81E-74	postive
TRAJ6	AC002044.1	0.71743403	1.00E-85	postive
TRAJ8	AC002044.1	0.752587727	8.88E-99	postive
TRAJ10	AC002044.1	0.66361565	3.37E-69	postive
TRAJ12	AC002044.1	0.605426931	8.18E-55	postive
TRAJ13	AC002044.1	0.759269555	1.65E-101	postive
TRAJ14	AC002044.1	0.709918596	3.43E-83	postive
TRAJ16	AC002044.1	0.682441054	1.42E-74	postive
TRAJ17	AC002044.1	0.647666317	6.09E-65	postive
TRAJ18	AC002044.1	0.600730938	8.84E-54	postive
TRAJ21	AC002044.1	0.752716685	7.88E-99	postive
TRAJ31	AC002044.1	0.765648418	3.34E-104	postive
TRAJ37	AC002044.1	0.766142241	2.05E-104	postive
TRAJ38	AC002044.1	0.76780857	3.91E-105	postive
TRAJ39	AC002044.1	0.760351632	5.83E-102	postive
TRAJ38	AZIN1-AS1	0.500535767	2.87E-35	postive
PSMD3	AC067852.2	0.567395741	6.55E-47	postive
PSME3	AC067852.2	0.519508128	2.59E-38	postive
CREB1	AC139887.4	0.696954214	5.29E-79	postive
RFXAP	AC139887.4	0.513332008	2.67E-37	postive
UBR1	AC139887.4	0.60176572	5.25E-54	postive
CYLD	AC139887.4	0.610308596	6.60E-56	postive
IREB2	AC139887.4	0.524133208	4.39E-39	postive
DDX17	AC139887.4	0.648871108	2.97E-65	postive
JAK2	AC139887.4	0.521236414	1.34E-38	postive
NFAT5	AC139887.4	0.881711994	5.01E-176	postive
NFATC3	AC139887.4	0.607678739	2.58E-55	postive
PIK3R1	AC139887.4	0.550436972	1.05E-43	postive
PIK3CA	AC139887.4	0.547515269	3.58E-43	postive
GNRH1	AC139887.4	0.704472649	2.10E-81	postive
IL6ST	AC139887.4	0.572587493	6.27E-48	postive
RABEP1	AC139887.4	0.517383515	5.81E-38	postive
ACVR2A	AC139887.4	0.628065127	4.78E-60	postive
ANGPTL1	AC139887.4	0.605820525	6.69E-55	postive
BMPR1A	AC139887.4	0.501048015	2.39E-35	postive
BMPR2	AC139887.4	0.605679336	7.19E-55	postive
CRLF3	AC139887.4	0.523593181	5.41E-39	postive
NR2C2	AC139887.4	0.692918227	9.58E-78	postive
RORA	AC139887.4	0.807104928	4.52E-124	postive
SOS1	AC139887.4	0.622531335	9.96E-59	postive
SOS2	AC139887.4	0.557908244	4.28E-45	postive
BRAF	AC139887.4	0.717856765	7.17E-86	postive
CBLB	AC139887.4	0.555023793	1.49E-44	postive
TRAJ1	AC139887.4	0.805616529	2.82E-123	postive
TRAJ2	AC139887.4	0.76296075	4.66E-103	postive
TRAJ3	AC139887.4	0.829153211	9.97E-137	postive
TRAJ5	AC139887.4	0.816400969	3.37E-129	postive
TRAJ6	AC139887.4	0.848612557	1.65E-149	postive

TRAJ8	AC139887.4	0.83588614	5.85E-141	postive
TRAJ10	AC139887.4	0.845860243	1.36E-147	postive
TRAJ12	AC139887.4	0.794969857	8.77E-118	postive
TRAJ13	AC139887.4	0.845882207	1.31E-147	postive
TRAJ14	AC139887.4	0.826386569	4.83E-135	postive
TRAJ16	AC139887.4	0.846885808	2.65E-148	postive
TRAJ17	AC139887.4	0.823507343	2.55E-133	postive
TRAJ18	AC139887.4	0.803214194	5.24E-122	postive
TRAJ21	AC139887.4	0.854766392	6.25E-154	postive
TRAJ31	AC139887.4	0.871202201	8.11E-167	postive
TRAJ37	AC139887.4	0.894053463	4.98E-188	postive
TRAJ38	AC139887.4	0.865912623	1.75E-162	postive
TRAJ39	AC139887.4	0.869704204	1.43E-165	postive
CREB1	AC096741.1	0.763229219	3.59E-103	postive
UBR1	AC096741.1	0.663743513	3.11E-69	postive
ZC3HAV1	AC096741.1	0.524147736	4.36E-39	postive
IL15	AC096741.1	0.503440665	1.01E-35	postive
CYLD	AC096741.1	0.579295181	2.85E-49	postive
EIF2AK2	AC096741.1	0.555976914	9.86E-45	postive
MAPK8	AC096741.1	0.544771125	1.12E-42	postive
LMBR1	AC096741.1	0.549752826	1.40E-43	postive
SP1	AC096741.1	0.500088386	3.37E-35	postive
IREB2	AC096741.1	0.617358488	1.61E-57	postive
DDX17	AC096741.1	0.569312436	2.77E-47	postive
JAK2	AC096741.1	0.609249482	1.14E-55	postive
TXK	AC096741.1	0.604672901	1.20E-54	postive
NFAT5	AC096741.1	0.764161876	1.44E-103	postive
PIK3R1	AC096741.1	0.63244841	4.12E-61	postive
PIK3CA	AC096741.1	0.655466916	5.43E-67	postive
PIK3CB	AC096741.1	0.52568805	2.40E-39	postive
ROBO2	AC096741.1	0.605513887	7.82E-55	postive
GNRH1	AC096741.1	0.694616708	2.85E-78	postive
IL6ST	AC096741.1	0.584156695	2.89E-50	postive
RABEP1	AC096741.1	0.537122743	2.58E-41	postive
ACVR2A	AC096741.1	0.642442402	1.33E-63	postive
ANGPTL1	AC096741.1	0.710759821	1.80E-83	postive
BMPR1A	AC096741.1	0.580868859	1.36E-49	postive
BMPR2	AC096741.1	0.763886439	1.89E-103	postive
CRLF3	AC096741.1	0.574066249	3.19E-48	postive
LIFR	AC096741.1	0.504181306	7.72E-36	postive
NR1D2	AC096741.1	0.608134836	2.04E-55	postive
NR2C2	AC096741.1	0.641659221	2.10E-63	postive
RORA	AC096741.1	0.781672341	2.31E-111	postive
SOS1	AC096741.1	0.669931837	5.87E-71	postive
SOS2	AC096741.1	0.522939447	6.96E-39	postive
BRAF	AC096741.1	0.770754574	2.02E-106	postive
CBL	AC096741.1	0.525688071	2.40E-39	postive
CBLB	AC096741.1	0.73501799	5.41E-92	postive
PDK1	AC096741.1	0.50955374	1.08E-36	postive
TRAJ1	AC096741.1	0.622479554	1.02E-58	postive
TRAJ2	AC096741.1	0.666210661	6.46E-70	postive
TRAJ3	AC096741.1	0.674643059	2.68E-72	postive
TRAJ5	AC096741.1	0.670990746	2.95E-71	postive
TRAJ6	AC096741.1	0.694367467	3.41E-78	postive
TRAJ8	AC096741.1	0.65436591	1.07E-66	postive
TRAJ10	AC096741.1	0.63906217	9.49E-63	postive
TRAJ12	AC096741.1	0.719397335	2.11E-86	postive
TRAJ13	AC096741.1	0.676243612	9.25E-73	postive
TRAJ14	AC096741.1	0.680772307	4.42E-74	postive
TRAJ16	AC096741.1	0.670942434	3.04E-71	postive
TRAJ17	AC096741.1	0.61827598	9.88E-58	postive
TRAJ18	AC096741.1	0.659188333	5.44E-68	postive
TRAJ21	AC096741.1	0.70208179	1.24E-80	postive
TRAJ31	AC096741.1	0.748654689	3.28E-97	postive
TRAJ37	AC096741.1	0.703108225	5.81E-81	postive

TRAJ38	AC096741.1	0.79360788	4.19E-117	postive
TRAJ39	AC096741.1	0.685054645	2.37E-75	postive
CREB1	AC008537.2	0.573603011	3.95E-48	postive
UBR1	AC008537.2	0.570492389	1.62E-47	postive
IREB2	AC008537.2	0.518614852	3.64E-38	postive
DDX17	AC008537.2	0.602827746	3.07E-54	postive
TXK	AC008537.2	0.574571893	2.53E-48	postive
NFAT5	AC008537.2	0.608313964	1.86E-55	postive
PIK3R1	AC008537.2	0.577499111	6.56E-49	postive
IGHD4-4	AC008537.2	0.525204589	2.90E-39	postive
IGHD6-6	AC008537.2	0.517604961	5.34E-38	postive
GNRH1	AC008537.2	0.701378019	2.09E-80	postive
IL6ST	AC008537.2	0.543762134	1.71E-42	postive
ACVR2A	AC008537.2	0.527084663	1.39E-39	postive
ANGPTL1	AC008537.2	0.530839969	3.18E-40	postive
BMPR2	AC008537.2	0.62173479	1.53E-58	postive
NR2C2	AC008537.2	0.680329504	5.96E-74	postive
NR3C2	AC008537.2	0.516310452	8.72E-38	postive
RORA	AC008537.2	0.662329399	7.60E-69	postive
SOS1	AC008537.2	0.52765008	1.12E-39	postive
BRAF	AC008537.2	0.646622923	1.13E-64	postive
TEC	AC008537.2	0.551979476	5.45E-44	postive
CBLB	AC008537.2	0.557048594	6.21E-45	postive
TRAJ3	AC008537.2	0.535146502	5.71E-41	postive
TRAJ38	AC008537.2	0.518382321	3.98E-38	postive
TRAJ39	AC008537.2	0.521102872	1.41E-38	postive
RFXAP	AL139089.1	0.51254981	3.57E-37	postive
GNRH1	AL139089.1	0.551196739	7.60E-44	postive
TYK2	LINC02604	0.501371481	2.13E-35	postive
LTB4R	LINC02604	0.501253283	2.22E-35	postive
RABEP2	LINC02604	0.509341881	1.17E-36	postive
CREB1	AC022973.4	0.763251657	3.51E-103	postive
UBR1	AC022973.4	0.607518044	2.80E-55	postive
ZC3HAV1	AC022973.4	0.562335832	6.19E-46	postive
ZC3HAV1L	AC022973.4	0.526776603	1.57E-39	postive
IL15	AC022973.4	0.599058184	2.04E-53	postive
CYLD	AC022973.4	0.571714331	9.33E-48	postive
EIF2AK2	AC022973.4	0.530043318	4.35E-40	postive
MAPK8	AC022973.4	0.547044378	4.36E-43	postive
TLR1	AC022973.4	0.536400616	3.45E-41	postive
LMBR1	AC022973.4	0.596038077	9.17E-53	postive
LIMS1	AC022973.4	0.518075364	4.47E-38	postive
IREB2	AC022973.4	0.591023879	1.07E-51	postive
DDX17	AC022973.4	0.53762843	2.10E-41	postive
PIK3CG	AC022973.4	0.551680047	6.19E-44	postive
JAK2	AC022973.4	0.633722144	2.01E-61	postive
TXK	AC022973.4	0.531411834	2.53E-40	postive
NFAT5	AC022973.4	0.80087578	8.66E-121	postive
MALT1	AC022973.4	0.533372726	1.16E-40	postive
PIK3R1	AC022973.4	0.602323082	3.96E-54	postive
PIK3CA	AC022973.4	0.683650352	6.21E-75	postive
PIK3CB	AC022973.4	0.516845905	7.12E-38	postive
AKT3	AC022973.4	0.613920448	9.97E-57	postive
RASGRP3	AC022973.4	0.520134711	2.04E-38	postive
PLXNC1	AC022973.4	0.507277228	2.50E-36	postive
GMFB	AC022973.4	0.514758237	1.56E-37	postive
GNRH1	AC022973.4	0.677661507	3.59E-73	postive
IL6ST	AC022973.4	0.594957451	1.56E-52	postive
RABEP1	AC022973.4	0.546071841	6.55E-43	postive
ACVR2A	AC022973.4	0.589811567	1.93E-51	postive
ANGPTL1	AC022973.4	0.797468403	4.83E-119	postive
BMPR1A	AC022973.4	0.51289844	3.13E-37	postive
BMPR2	AC022973.4	0.678949498	1.51E-73	postive
CRLF3	AC022973.4	0.685390973	1.88E-75	postive
NR2C2	AC022973.4	0.634827318	1.07E-61	postive

RORA	AC022973.4	0.768018288	3.17E-105	postive
SOS1	AC022973.4	0.671676807	1.88E-71	postive
SOS2	AC022973.4	0.585129891	1.82E-50	postive
BRAF	AC022973.4	0.802414027	1.37E-121	postive
ITK	AC022973.4	0.51601881	9.73E-38	postive
CBL	AC022973.4	0.625906628	1.57E-59	postive
CBLB	AC022973.4	0.705546665	9.41E-82	postive
PDK1	AC022973.4	0.602526178	3.57E-54	postive
TRAJ1	AC022973.4	0.757911336	6.01E-101	postive
TRAJ2	AC022973.4	0.753344036	4.40E-99	postive
TRAJ3	AC022973.4	0.808769335	5.73E-125	postive
TRAJ5	AC022973.4	0.822989353	5.17E-133	postive
TRAJ6	AC022973.4	0.817615546	6.85E-130	postive
TRAJ8	AC022973.4	0.831447507	3.78E-138	postive
TRAJ10	AC022973.4	0.793017004	8.23E-117	postive
TRAJ12	AC022973.4	0.679659338	9.38E-74	postive
TRAJ13	AC022973.4	0.846048374	1.01E-147	postive
TRAJ14	AC022973.4	0.823993588	1.31E-133	postive
TRAJ16	AC022973.4	0.810829453	4.31E-126	postive
TRAJ17	AC022973.4	0.785795074	2.64E-113	postive
TRAJ18	AC022973.4	0.701206374	2.37E-80	postive
TRAJ21	AC022973.4	0.85124803	2.23E-151	postive
TRAJ31	AC022973.4	0.840169937	9.40E-144	postive
TRAJ37	AC022973.4	0.806739355	7.10E-124	postive
TRAJ38	AC022973.4	0.867893774	4.38E-164	postive
TRAJ39	AC022973.4	0.861863904	2.73E-159	postive
CREB1	AL121839.2	0.598296397	2.99E-53	postive
DDX17	AL121839.2	0.530276011	3.97E-40	postive
NFAT5	AL121839.2	0.571003794	1.29E-47	postive
GNRH1	AL121839.2	0.610393469	6.32E-56	postive
ACVR2A	AL121839.2	0.582075094	7.74E-50	postive
ANGPTL1	AL121839.2	0.505702514	4.44E-36	postive
BMPR2	AL121839.2	0.521397989	1.26E-38	postive
NR2C2	AL121839.2	0.610428505	6.21E-56	postive
RORA	AL121839.2	0.535325951	5.31E-41	postive
SOS1	AL121839.2	0.574681863	2.41E-48	postive
SOS2	AL121839.2	0.500944226	2.48E-35	postive
BRAF	AL121839.2	0.631987604	5.34E-61	postive
TRAJ1	AL121839.2	0.630748365	1.07E-60	postive
TRAJ2	AL121839.2	0.541292757	4.72E-42	postive
TRAJ3	AL121839.2	0.596533158	7.18E-53	postive
TRAJ5	AL121839.2	0.643587177	6.81E-64	postive
TRAJ6	AL121839.2	0.618858028	7.23E-58	postive
TRAJ8	AL121839.2	0.71214333	6.22E-84	postive
TRAJ10	AL121839.2	0.567558506	6.09E-47	postive
TRAJ12	AL121839.2	0.602416623	3.78E-54	postive
TRAJ13	AL121839.2	0.665648316	9.25E-70	postive
TRAJ14	AL121839.2	0.650059093	1.45E-65	postive
TRAJ16	AL121839.2	0.650444978	1.15E-65	postive
TRAJ17	AL121839.2	0.646267681	1.40E-64	postive
TRAJ21	AL121839.2	0.647600757	6.34E-65	postive
TRAJ31	AL121839.2	0.661063471	1.68E-68	postive
TRAJ37	AL121839.2	0.603533035	2.15E-54	postive
TRAJ38	AL121839.2	0.649232461	2.39E-65	postive
TRAJ39	AL121839.2	0.660763758	2.03E-68	postive
CREB1	AC090198.1	0.659169832	5.50E-68	postive
UBR1	AC090198.1	0.525813	2.29E-39	postive
ZC3HAV1	AC090198.1	0.537777994	1.98E-41	postive
CYLD	AC090198.1	0.52204054	9.84E-39	postive
MAPK8	AC090198.1	0.543240501	2.12E-42	postive
DDX17	AC090198.1	0.688313804	2.46E-76	postive
JAK2	AC090198.1	0.506417401	3.42E-36	postive
PTK2	AC090198.1	0.631850596	5.77E-61	postive
NFAT5	AC090198.1	0.702411842	9.73E-81	postive
PIK3R1	AC090198.1	0.576111667	1.25E-48	postive

PIK3CA	AC090198.1	0.542785811	2.55E-42	postive
GNRH1	AC090198.1	0.677230254	4.79E-73	postive
IL6ST	AC090198.1	0.610021771	7.67E-56	postive
ACVR2A	AC090198.1	0.574507623	2.61E-48	postive
ANGPTL1	AC090198.1	0.664628103	1.77E-69	postive
BMPR1A	AC090198.1	0.517493571	5.57E-38	postive
BMPR2	AC090198.1	0.578417975	4.28E-49	postive
CRLF3	AC090198.1	0.53584378	4.31E-41	postive
NR2C1	AC090198.1	0.507121666	2.65E-36	postive
NR2C2	AC090198.1	0.713612559	2.00E-84	postive
RORA	AC090198.1	0.720710997	7.40E-87	postive
SOS1	AC090198.1	0.611363544	3.81E-56	postive
SOS2	AC090198.1	0.522397095	8.58E-39	postive
BRAF	AC090198.1	0.731751719	8.61E-91	postive
CBLB	AC090198.1	0.610680224	5.44E-56	postive
TRAJ1	AC090198.1	0.628949247	2.92E-60	postive
TRAJ2	AC090198.1	0.610085764	7.42E-56	postive
TRAJ3	AC090198.1	0.648431372	3.86E-65	postive
TRAJ5	AC090198.1	0.708005484	1.47E-82	postive
TRAJ6	AC090198.1	0.654360049	1.07E-66	postive
TRAJ8	AC090198.1	0.657515786	1.54E-67	postive
TRAJ10	AC090198.1	0.627686744	5.89E-60	postive
TRAJ12	AC090198.1	0.580404322	1.70E-49	postive
TRAJ13	AC090198.1	0.686886723	6.66E-76	postive
TRAJ14	AC090198.1	0.657976627	1.16E-67	postive
TRAJ16	AC090198.1	0.651312693	6.83E-66	postive
TRAJ17	AC090198.1	0.633291787	2.56E-61	postive
TRAJ18	AC090198.1	0.59042258	1.43E-51	postive
TRAJ21	AC090198.1	0.692937319	9.45E-78	postive
TRAJ31	AC090198.1	0.7045018	2.06E-81	postive
TRAJ37	AC090198.1	0.671479719	2.14E-71	postive
TRAJ38	AC090198.1	0.708625645	9.20E-83	postive
TRAJ39	AC090198.1	0.697883945	2.69E-79	postive
DDX17	CCDC18-AS1	0.580111468	1.94E-49	postive
NFAT5	CCDC18-AS1	0.506184823	3.73E-36	postive
LTB4R2	CCDC18-AS1	0.549399705	1.62E-43	postive
GNRH1	CCDC18-AS1	0.599547538	1.60E-53	postive
NR2C2	CCDC18-AS1	0.693021157	8.91E-78	postive
RORA	CCDC18-AS1	0.512983884	3.04E-37	postive
BRAF	CCDC18-AS1	0.627413476	6.85E-60	postive
CBLB	CCDC18-AS1	0.519170535	2.95E-38	postive
BRAF	AC012368.1	0.518197539	4.27E-38	postive
RFXAP	AC010615.2	0.502790767	1.28E-35	postive
NR2C2	AC010615.2	0.514816146	1.53E-37	postive
BRAF	AC010615.2	0.579158384	3.03E-49	postive
GNRH1	AL136304.1	0.524217895	4.25E-39	postive
NR2C2	AL136304.1	0.505325843	5.09E-36	postive
CREB1	AC011676.1	0.549056901	1.88E-43	postive
UBR1	AC011676.1	0.519388468	2.71E-38	postive
NFAT5	AC011676.1	0.68053174	5.20E-74	postive
GNRH1	AC011676.1	0.542578777	2.78E-42	postive
CRLF3	AC011676.1	0.525818394	2.28E-39	postive
NR2C2	AC011676.1	0.537552915	2.16E-41	postive
RORA	AC011676.1	0.56979722	2.22E-47	postive
BRAF	AC011676.1	0.55141082	6.94E-44	postive
TRAJ1	AC011676.1	0.576194485	1.20E-48	postive
TRAJ2	AC011676.1	0.54209914	3.39E-42	postive
TRAJ3	AC011676.1	0.586323789	1.03E-50	postive
TRAJ5	AC011676.1	0.582820435	5.44E-50	postive
TRAJ6	AC011676.1	0.623777316	5.05E-59	postive
TRAJ8	AC011676.1	0.531318711	2.63E-40	postive
TRAJ10	AC011676.1	0.620434395	3.10E-58	postive
TRAJ12	AC011676.1	0.562772873	5.10E-46	postive
TRAJ13	AC011676.1	0.579690298	2.37E-49	postive
TRAJ14	AC011676.1	0.565499588	1.53E-46	postive

TRAJ16	AC011676.1	0.588912309	2.97E-51	postive
TRAJ17	AC011676.1	0.54726306	3.98E-43	postive
TRAJ18	AC011676.1	0.592202356	6.03E-52	postive
TRAJ21	AC011676.1	0.598452235	2.77E-53	postive
TRAJ31	AC011676.1	0.620459165	3.06E-58	postive
TRAJ37	AC011676.1	0.615685764	3.92E-57	postive
TRAJ38	AC011676.1	0.635899136	5.83E-62	postive
TRAJ39	AC011676.1	0.599649822	1.52E-53	postive
B2M	CARD8-AS1	0.663445994	3.75E-69	postive
CD1D	CARD8-AS1	0.531748312	2.22E-40	postive
CTSS	CARD8-AS1	0.583666687	3.65E-50	postive
FCER1G	CARD8-AS1	0.560332924	1.49E-45	postive
HLA-DMB	CARD8-AS1	0.729448972	5.91E-90	postive
HLA-DRA	CARD8-AS1	0.683005237	9.66E-75	postive
CXCL10	CARD8-AS1	0.548438781	2.43E-43	postive
TLR4	CARD8-AS1	0.584482278	2.48E-50	postive
APOBEC3G	CARD8-AS1	0.620980252	2.31E-58	postive
CYBB	CARD8-AS1	0.535809338	4.37E-41	postive
TLR8	CARD8-AS1	0.598448753	2.77E-53	postive
MSR1	CARD8-AS1	0.566225193	1.10E-46	postive
CCL3	CARD8-AS1	0.560863921	1.18E-45	postive
CXCR6	CARD8-AS1	0.582787581	5.53E-50	postive
IL10	CARD8-AS1	0.698092362	2.32E-79	postive
CD86	CARD8-AS1	0.674680275	2.61E-72	postive
BTK	CARD8-AS1	0.722465242	1.81E-87	postive
CD72	CARD8-AS1	0.571956458	8.36E-48	postive
FCGR2B	CARD8-AS1	0.615664986	3.97E-57	postive
IGKV1-12	CARD8-AS1	0.514771551	1.55E-37	postive
GMFG	CARD8-AS1	0.77487558	2.97E-108	postive
IL16	CARD8-AS1	0.523253908	6.16E-39	postive
TNFSF13B	CARD8-AS1	0.749627261	1.35E-97	postive
C3AR1	CARD8-AS1	0.501288693	2.19E-35	postive
IL10RA	CARD8-AS1	0.514968286	1.44E-37	postive
TNFRSF17	CARD8-AS1	0.61670121	2.29E-57	postive
TYROBP	CARD8-AS1	0.607303467	3.13E-55	postive
CD247	CARD8-AS1	0.500587426	2.82E-35	postive
LCP2	CARD8-AS1	0.509440189	1.13E-36	postive
HCST	CARD8-AS1	0.507916145	1.98E-36	postive
CD48	CARD8-AS1	0.807473174	2.87E-124	postive
CD244	CARD8-AS1	0.532185395	1.86E-40	postive
SH2D1A	CARD8-AS1	0.663523027	3.57E-69	postive
CD3D	CARD8-AS1	0.621349698	1.89E-58	postive
CD3G	CARD8-AS1	0.534761006	6.66E-41	postive
PTPRC	CARD8-AS1	0.57793609	5.36E-49	postive
ICOS	CARD8-AS1	0.571712089	9.34E-48	postive
TRAV4	CARD8-AS1	0.500081629	3.37E-35	postive
TRAV9-2	CARD8-AS1	0.513703901	2.32E-37	postive
TRAV16	CARD8-AS1	0.649945529	1.56E-65	postive
TRAV39	CARD8-AS1	0.51051157	7.60E-37	postive
TRBV2	CARD8-AS1	0.557816947	4.45E-45	postive
TRBV5-1	CARD8-AS1	0.591530531	8.37E-52	postive
TRBV6-5	CARD8-AS1	0.532881703	1.41E-40	postive
TRBV18	CARD8-AS1	0.510092699	8.88E-37	postive
TRBV20-1	CARD8-AS1	0.52032641	1.90E-38	postive
CREB1	AL139120.1	0.81037691	7.63E-126	postive
RFXAP	AL139120.1	0.524023777	4.58E-39	postive
UBR1	AL139120.1	0.670999459	2.93E-71	postive
ZC3HAV1	AL139120.1	0.582826571	5.43E-50	postive
ZC3HAV1L	AL139120.1	0.527051392	1.41E-39	postive
IL15	AL139120.1	0.617622992	1.40E-57	postive
CYLD	AL139120.1	0.607788312	2.43E-55	postive
EIF2AK2	AL139120.1	0.56811231	4.75E-47	postive
MAPK8	AL139120.1	0.581212418	1.16E-49	postive
TLR1	AL139120.1	0.500577588	2.83E-35	postive
LMBR1	AL139120.1	0.611753807	3.11E-56	postive

LIMS1	AL139120.1	0.530529044	3.59E-40	postive
IREB2	AL139120.1	0.643512907	7.11E-64	postive
DDX17	AL139120.1	0.590115582	1.66E-51	postive
PIK3CG	AL139120.1	0.550660542	9.54E-44	postive
JAK2	AL139120.1	0.649674033	1.83E-65	postive
TXK	AL139120.1	0.601420909	6.25E-54	postive
PPP3CB	AL139120.1	0.507014804	2.75E-36	postive
NFAT5	AL139120.1	0.845862576	1.35E-147	postive
MALT1	AL139120.1	0.552806014	3.84E-44	postive
PIK3R1	AL139120.1	0.655652881	4.85E-67	postive
PIK3CA	AL139120.1	0.726732197	5.59E-89	postive
PIK3CB	AL139120.1	0.560138789	1.62E-45	postive
AKT3	AL139120.1	0.568745896	3.57E-47	postive
RASGRP3	AL139120.1	0.548274803	2.61E-43	postive
IGHD4-4	AL139120.1	0.51933751	2.77E-38	postive
IGHD6-6	AL139120.1	0.535918461	4.19E-41	postive
PLXNC1	AL139120.1	0.526126263	2.02E-39	postive
GNRH1	AL139120.1	0.743074587	4.91E-95	postive
IL6ST	AL139120.1	0.638708165	1.16E-62	postive
IL7	AL139120.1	0.50946917	1.12E-36	postive
RABEP1	AL139120.1	0.575784203	1.45E-48	postive
ACVR2A	AL139120.1	0.669686252	6.88E-71	postive
ANGPTL1	AL139120.1	0.800311008	1.69E-120	postive
BMPR1A	AL139120.1	0.578356447	4.41E-49	postive
BMPR2	AL139120.1	0.762357256	8.39E-103	postive
CRLF3	AL139120.1	0.672974281	8.04E-72	postive
NR1D2	AL139120.1	0.514753848	1.57E-37	postive
NR2C2	AL139120.1	0.70396394	3.07E-81	postive
NR3C1	AL139120.1	0.513324021	2.67E-37	postive
RORA	AL139120.1	0.837233169	7.89E-142	postive
SOS1	AL139120.1	0.711213953	1.27E-83	postive
SOS2	AL139120.1	0.607357915	3.04E-55	postive
BRAF	AL139120.1	0.827697864	7.75E-136	postive
ITK	AL139120.1	0.508986752	1.33E-36	postive
CBL	AL139120.1	0.612192606	2.47E-56	postive
CBLB	AL139120.1	0.725332097	1.76E-88	postive
RASGRP1	AL139120.1	0.52908523	6.35E-40	postive
PDK1	AL139120.1	0.612255032	2.39E-56	postive
TRAJ1	AL139120.1	0.771061637	1.48E-106	postive
TRAJ2	AL139120.1	0.748401832	4.13E-97	postive
TRAJ3	AL139120.1	0.828562912	2.30E-136	postive
TRAJ5	AL139120.1	0.833332873	2.48E-139	postive
TRAJ6	AL139120.1	0.830694315	1.11E-137	postive
TRAJ8	AL139120.1	0.831359007	4.30E-138	postive
TRAJ10	AL139120.1	0.809608543	2.00E-125	postive
TRAJ12	AL139120.1	0.753407243	4.15E-99	postive
TRAJ13	AL139120.1	0.830932431	7.92E-138	postive
TRAJ14	AL139120.1	0.819336413	7.04E-131	postive
TRAJ16	AL139120.1	0.83378367	1.28E-139	postive
TRAJ17	AL139120.1	0.81554842	1.02E-128	postive
TRAJ18	AL139120.1	0.750303527	7.29E-98	postive
TRAJ21	AL139120.1	0.856431376	3.67E-155	postive
TRAJ31	AL139120.1	0.872516874	6.34E-168	postive
TRAJ37	AL139120.1	0.83569483	7.76E-141	postive
TRAJ38	AL139120.1	0.896121721	3.48E-190	postive
TRAJ39	AL139120.1	0.878873104	1.87E-173	postive
LMBR1L	AC132872.3	0.596852251	6.13E-53	postive
TYK2	AC132872.3	0.565972754	1.24E-46	postive
LTB4R	AC132872.3	0.504578254	6.68E-36	postive
IRF9	AC132872.3	0.51702808	6.65E-38	postive
CREB1	SOS1-IT1	0.810082726	1.11E-125	postive
RFXAP	SOS1-IT1	0.558995414	2.67E-45	postive
UBR1	SOS1-IT1	0.636263948	4.73E-62	postive
ZC3HAV1	SOS1-IT1	0.597244747	5.04E-53	postive
ZC3HAV1L	SOS1-IT1	0.566761688	8.69E-47	postive

IL15	SOS1-IT1	0.583254783	4.43E-50	postive
CYLD	SOS1-IT1	0.600947258	7.93E-54	postive
EIF2AK2	SOS1-IT1	0.618942806	6.91E-58	postive
MAPK8	SOS1-IT1	0.618661679	8.03E-58	postive
LMBR1	SOS1-IT1	0.618517258	8.68E-58	postive
LIMS1	SOS1-IT1	0.53316448	1.26E-40	postive
IREB2	SOS1-IT1	0.655313703	5.97E-67	postive
DDX17	SOS1-IT1	0.61268977	1.90E-56	postive
PIK3CG	SOS1-IT1	0.53884078	1.28E-41	postive
JAK2	SOS1-IT1	0.644168627	4.84E-64	postive
TXK	SOS1-IT1	0.584025896	3.08E-50	postive
PPP3CB	SOS1-IT1	0.50843987	1.63E-36	postive
NFAT5	SOS1-IT1	0.83233172	1.06E-138	postive
MALT1	SOS1-IT1	0.561961174	7.30E-46	postive
PIK3R1	SOS1-IT1	0.651140633	7.58E-66	postive
PIK3CA	SOS1-IT1	0.720366099	9.75E-87	postive
PIK3CB	SOS1-IT1	0.52225705	9.05E-39	postive
AKT3	SOS1-IT1	0.597914417	3.62E-53	postive
GSK3B	SOS1-IT1	0.527417369	1.22E-39	postive
RASGRP3	SOS1-IT1	0.550225325	1.15E-43	postive
IGHD6-6	SOS1-IT1	0.501510719	2.02E-35	postive
PLXNC1	SOS1-IT1	0.519142575	2.98E-38	postive
GNRH1	SOS1-IT1	0.728701288	1.10E-89	postive
IL6ST	SOS1-IT1	0.613366699	1.33E-56	postive
RABEP1	SOS1-IT1	0.619073034	6.44E-58	postive
ACVR2A	SOS1-IT1	0.668665689	1.33E-70	postive
ANGPTL1	SOS1-IT1	0.80066768	1.11E-120	postive
BMPR1A	SOS1-IT1	0.575126593	1.96E-48	postive
BMPR2	SOS1-IT1	0.749585836	1.41E-97	postive
CRLF3	SOS1-IT1	0.659372973	4.85E-68	postive
LIFR	SOS1-IT1	0.523105644	6.53E-39	postive
NR1D2	SOS1-IT1	0.514051422	2.04E-37	postive
NR2C2	SOS1-IT1	0.695873798	1.15E-78	postive
RORA	SOS1-IT1	0.829659687	4.86E-137	postive
SOS1	SOS1-IT1	0.808875663	5.01E-125	postive
SOS2	SOS1-IT1	0.640771392	3.53E-63	postive
BRAF	SOS1-IT1	0.833521923	1.88E-139	postive
ITK	SOS1-IT1	0.537684068	2.05E-41	postive
CBL	SOS1-IT1	0.667587232	2.67E-70	postive
CBLB	SOS1-IT1	0.746208604	3.00E-96	postive
RASGRP1	SOS1-IT1	0.502164173	1.60E-35	postive
PDK1	SOS1-IT1	0.635556097	7.09E-62	postive
TRAJ1	SOS1-IT1	0.76745432	5.57E-105	postive
TRAJ2	SOS1-IT1	0.745311537	6.70E-96	postive
TRAJ3	SOS1-IT1	0.802510409	1.22E-121	postive
TRAJ5	SOS1-IT1	0.844120445	2.10E-146	postive
TRAJ6	SOS1-IT1	0.81053881	6.22E-126	postive
TRAJ8	SOS1-IT1	0.808055418	1.39E-124	postive
TRAJ10	SOS1-IT1	0.784763613	8.15E-113	postive
TRAJ12	SOS1-IT1	0.748318208	4.46E-97	postive
TRAJ13	SOS1-IT1	0.831109172	6.15E-138	postive
TRAJ14	SOS1-IT1	0.814909737	2.34E-128	postive
TRAJ16	SOS1-IT1	0.805601394	2.87E-123	postive
TRAJ17	SOS1-IT1	0.784595137	9.80E-113	postive
TRAJ18	SOS1-IT1	0.742213915	1.05E-94	postive
TRAJ21	SOS1-IT1	0.849083745	7.69E-150	postive
TRAJ31	SOS1-IT1	0.86217549	1.56E-159	postive
TRAJ37	SOS1-IT1	0.828075481	4.56E-136	postive
TRAJ38	SOS1-IT1	0.878733453	2.49E-173	postive
TRAJ39	SOS1-IT1	0.854629656	7.88E-154	postive
CREB1	DHDDS-AS1	0.805782217	2.30E-123	postive
RFXAP	DHDDS-AS1	0.544262831	1.39E-42	postive
UBR1	DHDDS-AS1	0.674142708	3.73E-72	postive
ZC3HAV1	DHDDS-AS1	0.588689458	3.31E-51	postive
IL15	DHDDS-AS1	0.546116737	6.43E-43	postive

CYLD	DHDDS-AS1	0.666896616	4.16E-70	postive
EIF2AK2	DHDDS-AS1	0.573239819	4.66E-48	postive
MAPK8	DHDDS-AS1	0.57248482	6.57E-48	postive
LMBR1	DHDDS-AS1	0.581185241	1.18E-49	postive
IREB2	DHDDS-AS1	0.623244145	6.76E-59	postive
DDX17	DHDDS-AS1	0.660044306	3.19E-68	postive
PIK3CG	DHDDS-AS1	0.551715289	6.10E-44	postive
JAK2	DHDDS-AS1	0.647436558	6.99E-65	postive
TXK	DHDDS-AS1	0.61362468	1.17E-56	postive
NFAT5	DHDDS-AS1	0.901346141	7.66E-196	postive
NFATC3	DHDDS-AS1	0.536260576	3.65E-41	postive
MALT1	DHDDS-AS1	0.540016549	7.95E-42	postive
PIK3R1	DHDDS-AS1	0.673631387	5.22E-72	postive
PIK3CA	DHDDS-AS1	0.693333814	7.13E-78	postive
PIK3CB	DHDDS-AS1	0.511114892	6.08E-37	postive
AKT3	DHDDS-AS1	0.563593391	3.55E-46	postive
RASGRP3	DHDDS-AS1	0.534312634	7.98E-41	postive
IGHD6-6	DHDDS-AS1	0.529594569	5.20E-40	postive
PLXNC1	DHDDS-AS1	0.515101236	1.37E-37	postive
GNRH1	DHDDS-AS1	0.766239208	1.86E-104	postive
IL6ST	DHDDS-AS1	0.66879378	1.23E-70	postive
RABEP1	DHDDS-AS1	0.573228413	4.68E-48	postive
ACVR2A	DHDDS-AS1	0.66781236	2.31E-70	postive
ANGPTL1	DHDDS-AS1	0.753148802	5.28E-99	postive
BMPR1A	DHDDS-AS1	0.560471867	1.40E-45	postive
BMPR2	DHDDS-AS1	0.750041038	9.27E-98	postive
CRLF3	DHDDS-AS1	0.669064528	1.03E-70	postive
LIFR	DHDDS-AS1	0.512146714	4.15E-37	postive
NR1D2	DHDDS-AS1	0.559683381	1.98E-45	postive
NR2C1	DHDDS-AS1	0.515779923	1.06E-37	postive
NR2C2	DHDDS-AS1	0.729126123	7.73E-90	postive
RORA	DHDDS-AS1	0.889299761	3.08E-183	postive
SOS1	DHDDS-AS1	0.707034622	3.07E-82	postive
SOS2	DHDDS-AS1	0.593983737	2.52E-52	postive
BRAF	DHDDS-AS1	0.849635774	3.14E-150	postive
ITK	DHDDS-AS1	0.538921877	1.24E-41	postive
CD28	DHDDS-AS1	0.526374092	1.84E-39	postive
CBL	DHDDS-AS1	0.601605221	5.69E-54	postive
CBLB	DHDDS-AS1	0.737878848	4.63E-93	postive
RASGRP1	DHDDS-AS1	0.508700047	1.48E-36	postive
PDK1	DHDDS-AS1	0.559816814	1.87E-45	postive
TRAJ1	DHDDS-AS1	0.784048758	1.78E-112	postive
TRAJ2	DHDDS-AS1	0.817885211	4.81E-130	postive
TRAJ3	DHDDS-AS1	0.833618967	1.63E-139	postive
TRAJ5	DHDDS-AS1	0.832112093	1.45E-138	postive
TRAJ6	DHDDS-AS1	0.839959173	1.30E-143	postive
TRAJ8	DHDDS-AS1	0.837191181	8.40E-142	postive
TRAJ10	DHDDS-AS1	0.804719556	8.43E-123	postive
TRAJ12	DHDDS-AS1	0.763436463	2.93E-103	postive
TRAJ13	DHDDS-AS1	0.853323282	7.10E-153	postive
TRAJ14	DHDDS-AS1	0.827142259	1.69E-135	postive
TRAJ16	DHDDS-AS1	0.844671817	8.85E-147	postive
TRAJ17	DHDDS-AS1	0.789791057	3.15E-115	postive
TRAJ18	DHDDS-AS1	0.773398196	1.36E-107	postive
TRAJ21	DHDDS-AS1	0.863269478	2.18E-160	postive
TRAJ31	DHDDS-AS1	0.881891362	3.43E-176	postive
TRAJ37	DHDDS-AS1	0.874624495	1.00E-169	postive
TRAJ38	DHDDS-AS1	0.911155266	2.26E-207	postive
TRAJ39	DHDDS-AS1	0.879764467	2.96E-174	postive
F2RL1	LINC01133	0.516747866	7.39E-38	postive
ACKR4	LINC01133	0.54618087	6.26E-43	postive
FAM3D	LINC01133	0.704241335	2.50E-81	postive
MIA	LINC01133	0.552429379	4.50E-44	postive
IL1R2	LINC01133	0.620613796	2.81E-58	postive
CREB1	AC079907.1	0.551176059	7.67E-44	postive

RFXAP	AC079907.1	0.505500336	4.78E-36	postive
DDX17	AC079907.1	0.565998931	1.22E-46	postive
NFAT5	AC079907.1	0.635075114	9.32E-62	postive
GNRH1	AC079907.1	0.710885888	1.64E-83	postive
ANGPTL1	AC079907.1	0.580636446	1.52E-49	postive
CRLF3	AC079907.1	0.525452886	2.63E-39	postive
NR2C1	AC079907.1	0.658340775	9.22E-68	postive
NR2C2	AC079907.1	0.57857891	3.97E-49	postive
RORA	AC079907.1	0.588097801	4.40E-51	postive
SOS1	AC079907.1	0.521513748	1.20E-38	postive
BRAF	AC079907.1	0.612011081	2.72E-56	postive
CBLB	AC079907.1	0.580794632	1.41E-49	postive
PDK1	AC079907.1	0.507050286	2.72E-36	postive
TRAJ1	AC079907.1	0.533522787	1.09E-40	postive
TRAJ2	AC079907.1	0.601269175	6.74E-54	postive
TRAJ3	AC079907.1	0.583117845	4.73E-50	postive
TRAJ5	AC079907.1	0.519232228	2.88E-38	postive
TRAJ6	AC079907.1	0.576870154	8.78E-49	postive
TRAJ8	AC079907.1	0.538422954	1.52E-41	postive
TRAJ10	AC079907.1	0.540667334	6.09E-42	postive
TRAJ13	AC079907.1	0.535957635	4.12E-41	postive
TRAJ14	AC079907.1	0.544327138	1.35E-42	postive
TRAJ16	AC079907.1	0.561617185	8.49E-46	postive
TRAJ21	AC079907.1	0.548421538	2.45E-43	postive
TRAJ31	AC079907.1	0.561504673	8.92E-46	postive
TRAJ37	AC079907.1	0.56883368	3.43E-47	postive
TRAJ38	AC079907.1	0.625062082	2.50E-59	postive
TRAJ39	AC079907.1	0.556944553	6.50E-45	postive
CREB1	GEMIN7-AS1	0.509498626	1.11E-36	postive
RFXAP	GEMIN7-AS1	0.524134011	4.39E-39	postive
DDX17	GEMIN7-AS1	0.636791497	3.50E-62	postive
NFAT5	GEMIN7-AS1	0.600532187	9.77E-54	postive
LTB4R2	GEMIN7-AS1	0.540747187	5.90E-42	postive
GNRH1	GEMIN7-AS1	0.699637337	7.50E-80	postive
ACVR2A	GEMIN7-AS1	0.519542858	2.56E-38	postive
ANGPTL1	GEMIN7-AS1	0.556818276	6.86E-45	postive
NR2C1	GEMIN7-AS1	0.523280042	6.10E-39	postive
NR2C2	GEMIN7-AS1	0.643442701	7.41E-64	postive
RORA	GEMIN7-AS1	0.542523642	2.84E-42	postive
BRAF	GEMIN7-AS1	0.581691575	9.27E-50	postive
TRAJ1	GEMIN7-AS1	0.561386668	9.39E-46	postive
TRAJ2	GEMIN7-AS1	0.567782356	5.51E-47	postive
TRAJ3	GEMIN7-AS1	0.616766872	2.21E-57	postive
TRAJ5	GEMIN7-AS1	0.518415067	3.93E-38	postive
TRAJ6	GEMIN7-AS1	0.572178611	7.56E-48	postive
TRAJ8	GEMIN7-AS1	0.569806324	2.22E-47	postive
TRAJ10	GEMIN7-AS1	0.557283339	5.61E-45	postive
TRAJ13	GEMIN7-AS1	0.547189976	4.11E-43	postive
TRAJ14	GEMIN7-AS1	0.550633492	9.65E-44	postive
TRAJ16	GEMIN7-AS1	0.561413626	9.28E-46	postive
TRAJ17	GEMIN7-AS1	0.539054091	1.18E-41	postive
TRAJ21	GEMIN7-AS1	0.561675122	8.27E-46	postive
TRAJ31	GEMIN7-AS1	0.546160221	6.31E-43	postive
TRAJ37	GEMIN7-AS1	0.556658288	7.35E-45	postive
TRAJ38	GEMIN7-AS1	0.585540587	1.50E-50	postive
TRAJ39	GEMIN7-AS1	0.573133188	4.89E-48	postive
HLA-G	HLA-F-AS1	0.559432979	2.21E-45	postive
CREB1	DUBR	0.526286981	1.90E-39	postive
NFAT5	DUBR	0.552467742	4.43E-44	postive
AKT3	DUBR	0.54372908	1.73E-42	postive
BMPR2	DUBR	0.51063767	7.26E-37	postive
RORA	DUBR	0.507023887	2.74E-36	postive
TRAJ2	DUBR	0.525123575	2.99E-39	postive
TRAJ3	DUBR	0.519800799	2.32E-38	postive
TRAJ8	DUBR	0.513453859	2.55E-37	postive

TRAJ38	DUBR	0.510786224	6.87E-37	postive
TRAJ39	DUBR	0.514986512	1.43E-37	postive
CREB1	AC004492.1	0.807375279	3.24E-124	postive
UBR1	AC004492.1	0.669816871	6.32E-71	postive
ZC3HAV1	AC004492.1	0.595958477	9.54E-53	postive
ZC3HAV1L	AC004492.1	0.559593501	2.06E-45	postive
IL15	AC004492.1	0.58394522	3.20E-50	postive
CYLD	AC004492.1	0.622115526	1.25E-58	postive
EIF2AK2	AC004492.1	0.578640725	3.86E-49	postive
MAPK8	AC004492.1	0.569035582	3.14E-47	postive
TLR1	AC004492.1	0.512852536	3.19E-37	postive
NFKBIZ	AC004492.1	0.538292493	1.60E-41	postive
LMBR1	AC004492.1	0.610932921	4.77E-56	postive
LIMS1	AC004492.1	0.531718629	2.24E-40	postive
IREB2	AC004492.1	0.624122491	4.18E-59	postive
DDX17	AC004492.1	0.630612306	1.16E-60	postive
PIK3CG	AC004492.1	0.566672573	9.05E-47	postive
JAK2	AC004492.1	0.699683502	7.25E-80	postive
TXK	AC004492.1	0.612129238	2.55E-56	postive
NFAT5	AC004492.1	0.847320668	1.32E-148	postive
MALT1	AC004492.1	0.556615705	7.49E-45	postive
PIK3R1	AC004492.1	0.647056947	8.76E-65	postive
PIK3CA	AC004492.1	0.715208693	5.75E-85	postive
PIK3CB	AC004492.1	0.549462562	1.58E-43	postive
AKT3	AC004492.1	0.512800537	3.25E-37	postive
RASGRP3	AC004492.1	0.564651933	2.22E-46	postive
PLXNC1	AC004492.1	0.509299303	1.19E-36	postive
GNRH1	AC004492.1	0.757938288	5.86E-101	postive
IL6ST	AC004492.1	0.628619231	3.51E-60	postive
RABEP1	AC004492.1	0.58673282	8.48E-51	postive
ACVR2A	AC004492.1	0.627126052	8.03E-60	postive
ANGPTL1	AC004492.1	0.78408221	1.71E-112	postive
BMPR1A	AC004492.1	0.532324494	1.76E-40	postive
BMPR2	AC004492.1	0.725596861	1.42E-88	postive
CRLF3	AC004492.1	0.672426539	1.15E-71	postive
NR1D2	AC004492.1	0.526992057	1.44E-39	postive
NR2C1	AC004492.1	0.505983356	4.01E-36	postive
NR2C2	AC004492.1	0.732495822	4.60E-91	postive
RORA	AC004492.1	0.846431101	5.47E-148	postive
SOS1	AC004492.1	0.715937847	3.25E-85	postive
SOS2	AC004492.1	0.593396613	3.36E-52	postive
BRAF	AC004492.1	0.825597403	1.44E-134	postive
ITK	AC004492.1	0.516016023	9.74E-38	postive
TEC	AC004492.1	0.528966311	6.66E-40	postive
CBL	AC004492.1	0.633589335	2.16E-61	postive
CBLB	AC004492.1	0.803238769	5.08E-122	postive
RASGRP1	AC004492.1	0.523133071	6.46E-39	postive
PDK1	AC004492.1	0.631949783	5.46E-61	postive
TRAJ1	AC004492.1	0.731179694	1.39E-90	postive
TRAJ2	AC004492.1	0.71803029	6.25E-86	postive
TRAJ3	AC004492.1	0.759212372	1.74E-101	postive
TRAJ5	AC004492.1	0.801276984	5.36E-121	postive
TRAJ6	AC004492.1	0.782417479	1.04E-111	postive
TRAJ8	AC004492.1	0.716501425	2.09E-85	postive
TRAJ10	AC004492.1	0.764679671	8.67E-104	postive
TRAJ12	AC004492.1	0.70686771	3.48E-82	postive
TRAJ13	AC004492.1	0.762730199	5.84E-103	postive
TRAJ14	AC004492.1	0.755016583	9.24E-100	postive
TRAJ16	AC004492.1	0.765707814	3.15E-104	postive
TRAJ17	AC004492.1	0.712002867	6.93E-84	postive
TRAJ18	AC004492.1	0.72353682	7.60E-88	postive
TRAJ21	AC004492.1	0.789489089	4.42E-115	postive
TRAJ31	AC004492.1	0.819058627	1.02E-130	postive
TRAJ37	AC004492.1	0.786439539	1.30E-113	postive
TRAJ38	AC004492.1	0.861356575	6.74E-159	postive

TRAJ39	AC004492.1	0.803599743	3.29E-122	postive
CREB1	CFLAR-AS1	0.741110057	2.77E-94	postive
UBR1	CFLAR-AS1	0.635206936	8.65E-62	postive
ZC3HAV1	CFLAR-AS1	0.559070954	2.58E-45	postive
IL15	CFLAR-AS1	0.596607319	6.92E-53	postive
CYLD	CFLAR-AS1	0.596601609	6.94E-53	postive
EIF2AK2	CFLAR-AS1	0.574539358	2.57E-48	postive
MAPK8	CFLAR-AS1	0.525738406	2.35E-39	postive
LMBR1	CFLAR-AS1	0.558139025	3.87E-45	postive
LIMS1	CFLAR-AS1	0.548167763	2.73E-43	postive
IREB2	CFLAR-AS1	0.583041301	4.90E-50	postive
DDX17	CFLAR-AS1	0.591650543	7.89E-52	postive
PIK3CG	CFLAR-AS1	0.585845144	1.30E-50	postive
JAK2	CFLAR-AS1	0.669405989	8.25E-71	postive
TXK	CFLAR-AS1	0.649723516	1.78E-65	postive
NFAT5	CFLAR-AS1	0.758266492	4.29E-101	postive
MALT1	CFLAR-AS1	0.549858578	1.34E-43	postive
PIK3R1	CFLAR-AS1	0.669118056	9.94E-71	postive
PIK3CA	CFLAR-AS1	0.694006029	4.41E-78	postive
PIK3CB	CFLAR-AS1	0.535122124	5.77E-41	postive
RASGRP3	CFLAR-AS1	0.54562717	7.88E-43	postive
IGHD6-6	CFLAR-AS1	0.545102115	9.80E-43	postive
GNRH1	CFLAR-AS1	0.687209946	5.32E-76	postive
IL6ST	CFLAR-AS1	0.638928888	1.02E-62	postive
RABEP1	CFLAR-AS1	0.52802323	9.64E-40	postive
ACVR2A	CFLAR-AS1	0.544965632	1.04E-42	postive
ANGPTL1	CFLAR-AS1	0.775032272	2.52E-108	postive
BMPR2	CFLAR-AS1	0.707852455	1.65E-82	postive
CRLF3	CFLAR-AS1	0.617928916	1.19E-57	postive
IL18R1	CFLAR-AS1	0.521683795	1.13E-38	postive
NR2C2	CFLAR-AS1	0.677987287	2.89E-73	postive
RORA	CFLAR-AS1	0.816863321	1.84E-129	postive
SOS1	CFLAR-AS1	0.635919767	5.76E-62	postive
SOS2	CFLAR-AS1	0.540217805	7.32E-42	postive
BRAF	CFLAR-AS1	0.78332356	3.90E-112	postive
ITK	CFLAR-AS1	0.549097258	1.84E-43	postive
TEC	CFLAR-AS1	0.568549705	3.90E-47	postive
CBL	CFLAR-AS1	0.596024266	9.23E-53	postive
CBLB	CFLAR-AS1	0.783529349	3.12E-112	postive
RASGRP1	CFLAR-AS1	0.529295139	5.85E-40	postive
PDK1	CFLAR-AS1	0.626067052	1.44E-59	postive
TRAJ1	CFLAR-AS1	0.617324491	1.64E-57	postive
TRAJ2	CFLAR-AS1	0.673379558	6.16E-72	postive
TRAJ3	CFLAR-AS1	0.718433359	4.54E-86	postive
TRAJ5	CFLAR-AS1	0.692938398	9.45E-78	postive
TRAJ6	CFLAR-AS1	0.683823374	5.52E-75	postive
TRAJ8	CFLAR-AS1	0.61413375	8.91E-57	postive
TRAJ10	CFLAR-AS1	0.660384605	2.58E-68	postive
TRAJ12	CFLAR-AS1	0.621262891	1.98E-58	postive
TRAJ13	CFLAR-AS1	0.638847633	1.07E-62	postive
TRAJ14	CFLAR-AS1	0.661877097	1.01E-68	postive
TRAJ16	CFLAR-AS1	0.682588212	1.29E-74	postive
TRAJ17	CFLAR-AS1	0.627886891	5.27E-60	postive
TRAJ18	CFLAR-AS1	0.687743262	3.67E-76	postive
TRAJ21	CFLAR-AS1	0.711239248	1.25E-83	postive
TRAJ31	CFLAR-AS1	0.706520706	4.52E-82	postive
TRAJ37	CFLAR-AS1	0.681570397	2.57E-74	postive
TRAJ38	CFLAR-AS1	0.78004977	1.30E-110	postive
TRAJ39	CFLAR-AS1	0.736963184	1.02E-92	postive
CREB1	AL049840.7	0.822476452	1.04E-132	postive
RFXAP	AL049840.7	0.547157628	4.16E-43	postive
UBR1	AL049840.7	0.687630529	3.97E-76	postive
ZC3HAV1	AL049840.7	0.591900949	6.99E-52	postive
ZC3HAV1L	AL049840.7	0.509334989	1.17E-36	postive
IL15	AL049840.7	0.614920465	5.88E-57	postive

CYLD	AL049840.7	0.652518961	3.29E-66	postive
EIF2AK2	AL049840.7	0.561778975	7.91E-46	postive
MAPK8	AL049840.7	0.589049586	2.78E-51	postive
LMBR1	AL049840.7	0.598951895	2.16E-53	postive
LIMS1	AL049840.7	0.531012581	2.97E-40	postive
IREB2	AL049840.7	0.638634751	1.21E-62	postive
DDX17	AL049840.7	0.645285697	2.50E-64	postive
PIK3CG	AL049840.7	0.566927619	8.07E-47	postive
JAK2	AL049840.7	0.666441896	5.57E-70	postive
TXK	AL049840.7	0.634465671	1.32E-61	postive
PPP3CB	AL049840.7	0.517394274	5.79E-38	postive
NFAT5	AL049840.7	0.891362028	2.74E-185	postive
NFATC3	AL049840.7	0.505710766	4.43E-36	postive
MALT1	AL049840.7	0.567998469	5.00E-47	postive
PIK3R1	AL049840.7	0.680576783	5.05E-74	postive
PIK3CA	AL049840.7	0.722140243	2.35E-87	postive
PIK3CB	AL049840.7	0.543692634	1.76E-42	postive
AKT3	AL049840.7	0.594917133	1.59E-52	postive
RASGRP3	AL049840.7	0.543133174	2.21E-42	postive
IGHD4-4	AL049840.7	0.509126399	1.27E-36	postive
IGHD6-6	AL049840.7	0.565656346	1.42E-46	postive
PLXNC1	AL049840.7	0.518425068	3.92E-38	postive
GNRH1	AL049840.7	0.789220656	5.97E-115	postive
IL6ST	AL049840.7	0.65812817	1.05E-67	postive
IL7	AL049840.7	0.502476685	1.43E-35	postive
RABEP1	AL049840.7	0.599929879	1.32E-53	postive
ACVR2A	AL049840.7	0.667548704	2.74E-70	postive
ANGPTL1	AL049840.7	0.801800675	2.87E-121	postive
BMPR1A	AL049840.7	0.583523001	3.91E-50	postive
BMPR2	AL049840.7	0.776794409	4.03E-109	postive
CRLF3	AL049840.7	0.676833393	6.25E-73	postive
NR1D2	AL049840.7	0.53672078	3.03E-41	postive
NR2C2	AL049840.7	0.71905778	2.77E-86	postive
NR3C1	AL049840.7	0.511034354	6.27E-37	postive
RORA	AL049840.7	0.870540442	2.89E-166	postive
SOS1	AL049840.7	0.704871502	1.56E-81	postive
SOS2	AL049840.7	0.608337351	1.83E-55	postive
BRAF	AL049840.7	0.832357577	1.02E-138	postive
ITK	AL049840.7	0.546064195	6.57E-43	postive
CD28	AL049840.7	0.512345556	3.85E-37	postive
CBL	AL049840.7	0.642608795	1.21E-63	postive
CBLB	AL049840.7	0.735156853	4.80E-92	postive
RASGRP1	AL049840.7	0.533235781	1.23E-40	postive
PDK1	AL049840.7	0.609315752	1.11E-55	postive
TRAJ1	AL049840.7	0.761916384	1.29E-102	postive
TRAJ2	AL049840.7	0.785904166	2.34E-113	postive
TRAJ3	AL049840.7	0.844856378	6.62E-147	postive
TRAJ5	AL049840.7	0.799491738	4.47E-120	postive
TRAJ6	AL049840.7	0.834592963	3.93E-140	postive
TRAJ8	AL049840.7	0.808189322	1.18E-124	postive
TRAJ10	AL049840.7	0.807192212	4.06E-124	postive
TRAJ12	AL049840.7	0.732875503	3.34E-91	postive
TRAJ13	AL049840.7	0.82063273	1.25E-131	postive
TRAJ14	AL049840.7	0.799805785	3.09E-120	postive
TRAJ16	AL049840.7	0.82049936	1.49E-131	postive
TRAJ17	AL049840.7	0.784842325	7.48E-113	postive
TRAJ18	AL049840.7	0.764539526	9.95E-104	postive
TRAJ21	AL049840.7	0.849123645	7.21E-150	postive
TRAJ31	AL049840.7	0.853952727	2.47E-153	postive
TRAJ37	AL049840.7	0.842866429	1.49E-145	postive
TRAJ38	AL049840.7	0.888771534	1.02E-182	postive
TRAJ39	AL049840.7	0.863142839	2.74E-160	postive
CREB1	AC015911.3	0.707142577	2.83E-82	postive
IFNG	AC015911.3	0.544703214	1.16E-42	postive
CIITA	AC015911.3	0.591114918	1.02E-51	postive

UBR1	AC015911.3	0.54355855	1.86E-42	postive
ZC3HAV1	AC015911.3	0.578211957	4.71E-49	postive
IL15	AC015911.3	0.707063774	3.00E-82	postive
CYLD	AC015911.3	0.70015785	5.12E-80	postive
EIF2AK2	AC015911.3	0.559887799	1.81E-45	postive
TLR1	AC015911.3	0.500721535	2.68E-35	postive
IREB2	AC015911.3	0.523356737	5.92E-39	postive
IL7R	AC015911.3	0.501307961	2.17E-35	postive
DDX17	AC015911.3	0.59018396	1.61E-51	postive
PIK3CG	AC015911.3	0.622445997	1.04E-58	postive
JAK2	AC015911.3	0.723199198	9.99E-88	postive
TXK	AC015911.3	0.554541721	1.83E-44	postive
NFAT5	AC015911.3	0.737283211	7.75E-93	postive
NFATC2	AC015911.3	0.533447014	1.13E-40	postive
PIK3R1	AC015911.3	0.616594239	2.42E-57	postive
PIK3CA	AC015911.3	0.633244689	2.63E-61	postive
PIK3CB	AC015911.3	0.508281966	1.73E-36	postive
RASGRP3	AC015911.3	0.67880013	1.67E-73	postive
PRKCB	AC015911.3	0.500499555	2.91E-35	postive
IGHD6-6	AC015911.3	0.506032812	3.94E-36	postive
PLXNC1	AC015911.3	0.676391851	8.38E-73	postive
GNRH1	AC015911.3	0.635752958	6.33E-62	postive
IL6ST	AC015911.3	0.529190639	6.09E-40	postive
RABEP1	AC015911.3	0.520672887	1.66E-38	postive
ACVR2A	AC015911.3	0.559148006	2.50E-45	postive
ANGPTL1	AC015911.3	0.699283535	9.72E-80	postive
BMPR2	AC015911.3	0.628227611	4.36E-60	postive
CRLF3	AC015911.3	0.632968524	3.07E-61	postive
IL18RAP	AC015911.3	0.547425525	3.72E-43	postive
NR2C2	AC015911.3	0.675866454	1.19E-72	postive
RORA	AC015911.3	0.7270385	4.35E-89	postive
SOS1	AC015911.3	0.678744216	1.74E-73	postive
SOS2	AC015911.3	0.568856159	3.40E-47	postive
BRAF	AC015911.3	0.738290498	3.24E-93	postive
PTPRC	AC015911.3	0.635882437	5.88E-62	postive
ITK	AC015911.3	0.719795422	1.54E-86	postive
CD28	AC015911.3	0.651618919	5.67E-66	postive
ICOS	AC015911.3	0.594343474	2.11E-52	postive
CBL	AC015911.3	0.636235906	4.81E-62	postive
CBLB	AC015911.3	0.692785691	1.05E-77	postive
RASGRP1	AC015911.3	0.583615218	3.74E-50	postive
PDK1	AC015911.3	0.547373593	3.80E-43	postive
TRAJ1	AC015911.3	0.835529119	9.91E-141	postive
TRAJ2	AC015911.3	0.795368812	5.54E-118	postive
TRAJ3	AC015911.3	0.870861879	1.56E-166	postive
TRAJ5	AC015911.3	0.82468126	5.11E-134	postive
TRAJ6	AC015911.3	0.824388547	7.64E-134	postive
TRAJ8	AC015911.3	0.807562341	2.57E-124	postive
TRAJ10	AC015911.3	0.785881578	2.40E-113	postive
TRAJ12	AC015911.3	0.824928759	3.63E-134	postive
TRAJ13	AC015911.3	0.805228928	4.53E-123	postive
TRAJ14	AC015911.3	0.842867333	1.48E-145	postive
TRAJ16	AC015911.3	0.810385881	7.55E-126	postive
TRAJ17	AC015911.3	0.806483276	9.73E-124	postive
TRAJ18	AC015911.3	0.814823924	2.62E-128	postive
TRAJ21	AC015911.3	0.833241259	2.83E-139	postive
TRAJ31	AC015911.3	0.8235662	2.35E-133	postive
TRAJ37	AC015911.3	0.791448625	4.88E-116	postive
TRAJ38	AC015911.3	0.864310728	3.30E-161	postive
TRAJ39	AC015911.3	0.817453199	8.49E-130	postive
IL15	LINC02100	0.633647881	2.09E-61	postive
IL18R1	LINC02100	0.50216396	1.60E-35	postive
KCNH2	GAS6-DT	0.548260216	2.62E-43	postive
BPIFB1	ELN-AS1	0.584425007	2.55E-50	postive
DES	ELN-AS1	0.632016673	5.26E-61	postive

BPIFB1	AL645924.1	0.653486517	1.82E-66	postive
DES	AL645924.1	0.668274703	1.71E-70	postive
GIPR	AL645924.1	0.54255313	2.81E-42	postive
CREB1	AC253536.3	0.754041702	2.30E-99	postive
UBR1	AC253536.3	0.650671327	1.01E-65	postive
ZC3HAV1	AC253536.3	0.612097039	2.60E-56	postive
ZC3HAV1L	AC253536.3	0.548031421	2.89E-43	postive
IL15	AC253536.3	0.529324023	5.78E-40	postive
CYLD	AC253536.3	0.558890925	2.79E-45	postive
EIF2AK2	AC253536.3	0.593524259	3.16E-52	postive
MAPK8	AC253536.3	0.530280809	3.96E-40	postive
LMBR1	AC253536.3	0.580394464	1.70E-49	postive
IREB2	AC253536.3	0.613709576	1.11E-56	postive
DDX17	AC253536.3	0.626713113	1.01E-59	postive
PIK3CG	AC253536.3	0.510291657	8.25E-37	postive
JAK2	AC253536.3	0.60120585	6.96E-54	postive
TXK	AC253536.3	0.599311972	1.80E-53	postive
NFAT5	AC253536.3	0.764688964	8.59E-104	postive
MALT1	AC253536.3	0.514771539	1.55E-37	postive
PIK3R1	AC253536.3	0.606573255	4.55E-55	postive
PIK3CA	AC253536.3	0.698548522	1.66E-79	postive
PIK3CB	AC253536.3	0.577943372	5.34E-49	postive
AKT3	AC253536.3	0.523448947	5.72E-39	postive
RASGRP3	AC253536.3	0.525938975	2.18E-39	postive
IGHD6-6	AC253536.3	0.52858407	7.74E-40	postive
PLXNC1	AC253536.3	0.508467423	1.62E-36	postive
GNRH1	AC253536.3	0.741712273	1.63E-94	postive
IL6ST	AC253536.3	0.572872824	5.51E-48	postive
IL7	AC253536.3	0.511727941	4.84E-37	postive
RABEP1	AC253536.3	0.565386183	1.61E-46	postive
ACVR2A	AC253536.3	0.653160766	2.23E-66	postive
ANGPTL1	AC253536.3	0.733597109	1.81E-91	postive
BMPR1A	AC253536.3	0.512400596	3.77E-37	postive
BMPR2	AC253536.3	0.700578393	3.76E-80	postive
CRLF3	AC253536.3	0.621759203	1.51E-58	postive
NR2C2	AC253536.3	0.757179386	1.20E-100	postive
RORA	AC253536.3	0.744621745	1.24E-95	postive
SOS1	AC253536.3	0.718802919	3.39E-86	postive
SOS2	AC253536.3	0.608189662	1.98E-55	postive
BRAF	AC253536.3	0.796519982	1.46E-118	postive
TEC	AC253536.3	0.524148458	4.36E-39	postive
CBL	AC253536.3	0.627578584	6.25E-60	postive
CBLB	AC253536.3	0.661549359	1.24E-68	postive
RASGRP1	AC253536.3	0.53361237	1.06E-40	postive
PDK1	AC253536.3	0.547934025	3.01E-43	postive
TRAJ1	AC253536.3	0.781204988	3.80E-111	postive
TRAJ2	AC253536.3	0.623442547	6.06E-59	postive
TRAJ3	AC253536.3	0.775688237	1.28E-108	postive
TRAJ5	AC253536.3	0.840219431	8.72E-144	postive
TRAJ6	AC253536.3	0.779966518	1.42E-110	postive
TRAJ8	AC253536.3	0.812374067	6.08E-127	postive
TRAJ10	AC253536.3	0.749121309	2.15E-97	postive
TRAJ12	AC253536.3	0.732147822	6.17E-91	postive
TRAJ13	AC253536.3	0.805447313	3.47E-123	postive
TRAJ14	AC253536.3	0.787703479	3.23E-114	postive
TRAJ16	AC253536.3	0.79323094	6.45E-117	postive
TRAJ17	AC253536.3	0.812915801	3.04E-127	postive
TRAJ18	AC253536.3	0.693989476	4.46E-78	postive
TRAJ21	AC253536.3	0.824218783	9.64E-134	postive
TRAJ31	AC253536.3	0.815715987	8.23E-129	postive
TRAJ37	AC253536.3	0.757348483	1.03E-100	postive
TRAJ38	AC253536.3	0.811932144	1.07E-126	postive
TRAJ39	AC253536.3	0.849783696	2.46E-150	postive
CREB1	GAS5-AS1	0.766199049	1.94E-104	postive
RFXAP	GAS5-AS1	0.546302863	5.95E-43	postive

UBR1	GAS5-AS1	0.660472544	2.44E-68	postive
ZC3HAV1	GAS5-AS1	0.56662833	9.23E-47	postive
IL15	GAS5-AS1	0.531041528	2.93E-40	postive
CYLD	GAS5-AS1	0.589336599	2.42E-51	postive
EIF2AK2	GAS5-AS1	0.559462421	2.18E-45	postive
MAPK8	GAS5-AS1	0.529243781	5.97E-40	postive
LMBR1	GAS5-AS1	0.525758824	2.33E-39	postive
IREB2	GAS5-AS1	0.614962981	5.75E-57	postive
DDX17	GAS5-AS1	0.631375353	7.54E-61	postive
PIK3CG	GAS5-AS1	0.511113623	6.08E-37	postive
JAK2	GAS5-AS1	0.60268853	3.29E-54	postive
TXK	GAS5-AS1	0.608666791	1.55E-55	postive
NFAT5	GAS5-AS1	0.821122639	6.46E-132	postive
MALT1	GAS5-AS1	0.518511738	3.79E-38	postive
PIK3R1	GAS5-AS1	0.643651541	6.55E-64	postive
PIK3CA	GAS5-AS1	0.659978417	3.32E-68	postive
AKT3	GAS5-AS1	0.542085596	3.41E-42	postive
IGHD4-4	GAS5-AS1	0.502845921	1.25E-35	postive
IGHD6-6	GAS5-AS1	0.530513688	3.62E-40	postive
GNRH1	GAS5-AS1	0.771792654	7.03E-107	postive
IL6ST	GAS5-AS1	0.613704955	1.12E-56	postive
RABEP1	GAS5-AS1	0.56511396	1.81E-46	postive
ACVR2A	GAS5-AS1	0.677009848	5.55E-73	postive
ANGPTL1	GAS5-AS1	0.734910715	5.93E-92	postive
BMPR1A	GAS5-AS1	0.573697233	3.78E-48	postive
BMPR2	GAS5-AS1	0.711619584	9.32E-84	postive
CRLF3	GAS5-AS1	0.639523481	7.27E-63	postive
NR1D2	GAS5-AS1	0.508880172	1.39E-36	postive
NR2C2	GAS5-AS1	0.703547284	4.19E-81	postive
RORA	GAS5-AS1	0.820550833	1.39E-131	postive
SOS1	GAS5-AS1	0.661584768	1.21E-68	postive
SOS2	GAS5-AS1	0.607544478	2.76E-55	postive
BRAF	GAS5-AS1	0.799690443	3.54E-120	postive
CBL	GAS5-AS1	0.564431574	2.45E-46	postive
CBLB	GAS5-AS1	0.663492526	3.64E-69	postive
PDK1	GAS5-AS1	0.561660495	8.33E-46	postive
TRAJ1	GAS5-AS1	0.725870137	1.13E-88	postive
TRAJ2	GAS5-AS1	0.722903902	1.27E-87	postive
TRAJ3	GAS5-AS1	0.771453103	9.94E-107	postive
TRAJ5	GAS5-AS1	0.78571948	2.87E-113	postive
TRAJ6	GAS5-AS1	0.782184817	1.33E-111	postive
TRAJ8	GAS5-AS1	0.774481368	4.46E-108	postive
TRAJ10	GAS5-AS1	0.754921845	1.01E-99	postive
TRAJ12	GAS5-AS1	0.697106326	4.74E-79	postive
TRAJ13	GAS5-AS1	0.783388353	3.63E-112	postive
TRAJ14	GAS5-AS1	0.765860647	2.71E-104	postive
TRAJ16	GAS5-AS1	0.765554936	3.66E-104	postive
TRAJ17	GAS5-AS1	0.74083282	3.54E-94	postive
TRAJ18	GAS5-AS1	0.703511846	4.30E-81	postive
TRAJ21	GAS5-AS1	0.810520502	6.37E-126	postive
TRAJ31	GAS5-AS1	0.815592582	9.66E-129	postive
TRAJ37	GAS5-AS1	0.793626491	4.10E-117	postive
TRAJ38	GAS5-AS1	0.832890346	4.71E-139	postive
TRAJ39	GAS5-AS1	0.831260504	4.95E-138	postive
CREB1	AL450263.1	0.614877913	6.02E-57	postive
RFXAP	AL450263.1	0.523903405	4.80E-39	postive
UBR1	AL450263.1	0.597766699	3.89E-53	postive
CYLD	AL450263.1	0.508189125	1.79E-36	postive
IREB2	AL450263.1	0.527631659	1.12E-39	postive
DDX17	AL450263.1	0.702299434	1.06E-80	postive
TXK	AL450263.1	0.598121735	3.26E-53	postive
NFAT5	AL450263.1	0.661392941	1.37E-68	postive
PIK3R1	AL450263.1	0.533395312	1.15E-40	postive
PIK3CA	AL450263.1	0.508246654	1.75E-36	postive
LTB4R2	AL450263.1	0.500223095	3.21E-35	postive

ROBO2	AL450263.1	0.59226746	5.84E-52	postive
GNRH1	AL450263.1	0.765622532	3.42E-104	postive
IL6ST	AL450263.1	0.518400667	3.95E-38	postive
RABEP1	AL450263.1	0.50645405	3.38E-36	postive
ACVR2A	AL450263.1	0.601196324	6.99E-54	postive
BMPR2	AL450263.1	0.612243758	2.41E-56	postive
NR1D2	AL450263.1	0.518795853	3.40E-38	postive
NR2C1	AL450263.1	0.537650983	2.08E-41	postive
NR2C2	AL450263.1	0.765786363	2.91E-104	postive
NR3C2	AL450263.1	0.554547609	1.82E-44	postive
RORA	AL450263.1	0.662307647	7.71E-69	postive
SOS1	AL450263.1	0.5870334	7.34E-51	postive
BRAF	AL450263.1	0.67440126	3.14E-72	postive
CBLB	AL450263.1	0.552118823	5.14E-44	postive
TRAJ1	AL450263.1	0.545362818	8.79E-43	postive
TRAJ2	AL450263.1	0.502674988	1.33E-35	postive
TRAJ3	AL450263.1	0.562774811	5.10E-46	postive
TRAJ5	AL450263.1	0.524051128	4.53E-39	postive
TRAJ6	AL450263.1	0.533744179	1.00E-40	postive
TRAJ8	AL450263.1	0.521102186	1.41E-38	postive
TRAJ12	AL450263.1	0.528030329	9.61E-40	postive
TRAJ16	AL450263.1	0.52207371	9.71E-39	postive
TRAJ21	AL450263.1	0.520715477	1.64E-38	postive
TRAJ31	AL450263.1	0.543235291	2.12E-42	postive
TRAJ37	AL450263.1	0.545911428	7.00E-43	postive
TRAJ38	AL450263.1	0.565226974	1.72E-46	postive
TRAJ39	AL450263.1	0.557818488	4.45E-45	postive
CREB1	AC008124.1	0.703705931	3.72E-81	postive
RFXAP	AC008124.1	0.64117048	2.80E-63	postive
UBR1	AC008124.1	0.586854177	8.00E-51	postive
CYLD	AC008124.1	0.616824283	2.14E-57	postive
MAPK8	AC008124.1	0.536062367	3.95E-41	postive
SP1	AC008124.1	0.54118044	4.94E-42	postive
IREB2	AC008124.1	0.58509638	1.85E-50	postive
DDX17	AC008124.1	0.699117364	1.10E-79	postive
JAK2	AC008124.1	0.533939483	9.26E-41	postive
TXK	AC008124.1	0.534831923	6.48E-41	postive
NFAT5	AC008124.1	0.775546142	1.48E-108	postive
NFATC3	AC008124.1	0.567189266	7.18E-47	postive
PIK3R1	AC008124.1	0.586855776	7.99E-51	postive
PIK3CA	AC008124.1	0.576550314	1.02E-48	postive
ROBO2	AC008124.1	0.502347874	1.50E-35	postive
GNRH1	AC008124.1	0.738118448	3.76E-93	postive
IL6ST	AC008124.1	0.559649878	2.01E-45	postive
RABEP1	AC008124.1	0.558626823	3.13E-45	postive
ACVR2A	AC008124.1	0.679440948	1.09E-73	postive
ANGPTL1	AC008124.1	0.626135838	1.39E-59	postive
BMPR1A	AC008124.1	0.553437948	2.93E-44	postive
BMPR2	AC008124.1	0.662203923	8.23E-69	postive
CRLF3	AC008124.1	0.512180091	4.09E-37	postive
NR2C1	AC008124.1	0.599166417	1.94E-53	postive
NR2C2	AC008124.1	0.694387082	3.36E-78	postive
RORA	AC008124.1	0.777446918	2.04E-109	postive
SOS1	AC008124.1	0.643186499	8.61E-64	postive
SOS2	AC008124.1	0.54486344	1.08E-42	postive
BRAF	AC008124.1	0.665661649	9.17E-70	postive
CBL	AC008124.1	0.512270751	3.96E-37	postive
CBLB	AC008124.1	0.588963162	2.90E-51	postive
TRAJ1	AC008124.1	0.662932608	5.19E-69	postive
TRAJ2	AC008124.1	0.631873696	5.70E-61	postive
TRAJ3	AC008124.1	0.682135867	1.75E-74	postive
TRAJ5	AC008124.1	0.669674207	6.94E-71	postive
TRAJ6	AC008124.1	0.686645335	7.87E-76	postive
TRAJ8	AC008124.1	0.650905838	8.73E-66	postive
TRAJ10	AC008124.1	0.674814902	2.39E-72	postive

TRAJ12	AC008124.1	0.632248865	4.61E-61	postive
TRAJ13	AC008124.1	0.677185722	4.94E-73	postive
TRAJ14	AC008124.1	0.647599882	6.34E-65	postive
TRAJ16	AC008124.1	0.673036705	7.72E-72	postive
TRAJ17	AC008124.1	0.652388451	3.56E-66	postive
TRAJ18	AC008124.1	0.65047927	1.13E-65	postive
TRAJ21	AC008124.1	0.689766529	8.90E-77	postive
TRAJ31	AC008124.1	0.703869454	3.30E-81	postive
TRAJ37	AC008124.1	0.708238618	1.23E-82	postive
TRAJ38	AC008124.1	0.720125434	1.18E-86	postive
TRAJ39	AC008124.1	0.697603177	3.30E-79	postive
DES	LINC02541	0.51175979	4.79E-37	postive
CREB1	AC129510.1	0.501816247	1.81E-35	postive
DDX17	AC129510.1	0.651780493	5.14E-66	postive
NFAT5	AC129510.1	0.581709002	9.19E-50	postive
LTB4R2	AC129510.1	0.595466404	1.22E-52	postive
GNRH1	AC129510.1	0.703384189	4.73E-81	postive
ANGPTL1	AC129510.1	0.508302257	1.72E-36	postive
NR2C1	AC129510.1	0.573283441	4.57E-48	postive
NR2C2	AC129510.1	0.71209332	6.47E-84	postive
RORA	AC129510.1	0.563124258	4.37E-46	postive
SOS1	AC129510.1	0.52222666	9.16E-39	postive
BRAF	AC129510.1	0.577191938	7.57E-49	postive
CBLB	AC129510.1	0.536891743	2.83E-41	postive
TRAJ1	AC129510.1	0.563797222	3.25E-46	postive
TRAJ3	AC129510.1	0.563818432	3.22E-46	postive
TRAJ5	AC129510.1	0.531705345	2.26E-40	postive
TRAJ6	AC129510.1	0.545286767	9.07E-43	postive
TRAJ8	AC129510.1	0.513025451	2.99E-37	postive
TRAJ10	AC129510.1	0.527491484	1.19E-39	postive
TRAJ13	AC129510.1	0.508039431	1.89E-36	postive
TRAJ14	AC129510.1	0.518319326	4.08E-38	postive
TRAJ16	AC129510.1	0.559440277	2.20E-45	postive
TRAJ17	AC129510.1	0.524239019	4.21E-39	postive
TRAJ18	AC129510.1	0.50147729	2.05E-35	postive
TRAJ21	AC129510.1	0.531358848	2.59E-40	postive
TRAJ31	AC129510.1	0.528615044	7.64E-40	postive
TRAJ37	AC129510.1	0.514430984	1.77E-37	postive
TRAJ38	AC129510.1	0.564849211	2.04E-46	postive
TRAJ39	AC129510.1	0.542057192	3.45E-42	postive
PTX3	LINC02154	0.639712899	6.51E-63	postive
HGF	LINC02154	0.65443392	1.02E-66	postive
SEMA6B	LINC02154	0.619444743	5.28E-58	postive
SEMA7A	LINC02154	0.732449115	4.78E-91	postive
IL24	LINC02154	0.609375215	1.07E-55	postive
IL13RA2	LINC02154	0.594466584	1.99E-52	postive
TIE1	LINC02154	0.607180039	3.33E-55	postive
TNFRSF8	LINC02154	0.940387089	8.23E-252	postive
CREB1	GK-AS1	0.506273946	3.61E-36	postive
NFKBIZ	GK-AS1	0.525870974	2.24E-39	postive
JAK2	GK-AS1	0.514751297	1.57E-37	postive
NFAT5	GK-AS1	0.560391341	1.45E-45	postive
GNRH1	GK-AS1	0.530143555	4.19E-40	postive
ANGPTL1	GK-AS1	0.592265343	5.85E-52	postive
IL18R1	GK-AS1	0.537464369	2.24E-41	postive
RORA	GK-AS1	0.567931666	5.15E-47	postive
CBLB	GK-AS1	0.56346545	3.76E-46	postive
PDK1	GK-AS1	0.534098742	8.69E-41	postive
TRAJ5	GK-AS1	0.504598112	6.64E-36	postive
TRAJ10	GK-AS1	0.534657091	6.95E-41	postive
TRAJ38	GK-AS1	0.531557424	2.39E-40	postive
TRAJ39	GK-AS1	0.503406624	1.02E-35	postive
CREB1	AL391001.1	0.632324744	4.42E-61	postive
UBR1	AL391001.1	0.579546743	2.53E-49	postive
IREB2	AL391001.1	0.54363678	1.80E-42	postive

NFAT5	AL391001.1	0.817773563	5.57E-130	postive
NFATC3	AL391001.1	0.56077076	1.23E-45	postive
GNRH1	AL391001.1	0.639809182	6.16E-63	postive
ACVR2A	AL391001.1	0.579000668	3.27E-49	postive
ANGPTL1	AL391001.1	0.502341231	1.50E-35	postive
BMPR1A	AL391001.1	0.556767632	7.01E-45	postive
BMPR2	AL391001.1	0.564582898	2.29E-46	postive
CRLF3	AL391001.1	0.526692716	1.62E-39	postive
NR2C2	AL391001.1	0.537224547	2.47E-41	postive
RORA	AL391001.1	0.664905558	1.49E-69	postive
SOS1	AL391001.1	0.540931512	5.47E-42	postive
BRAF	AL391001.1	0.595622748	1.13E-52	postive
TRAJ1	AL391001.1	0.680822308	4.27E-74	postive
TRAJ2	AL391001.1	0.746913012	1.59E-96	postive
TRAJ3	AL391001.1	0.741227979	2.50E-94	postive
TRAJ5	AL391001.1	0.614075072	9.19E-57	postive
TRAJ6	AL391001.1	0.767526107	5.18E-105	postive
TRAJ8	AL391001.1	0.752215748	1.25E-98	postive
TRAJ10	AL391001.1	0.741982215	1.29E-94	postive
TRAJ12	AL391001.1	0.673106499	7.37E-72	postive
TRAJ13	AL391001.1	0.720227036	1.09E-86	postive
TRAJ14	AL391001.1	0.702779	7.41E-81	postive
TRAJ16	AL391001.1	0.738199612	3.51E-93	postive
TRAJ17	AL391001.1	0.67247879	1.11E-71	postive
TRAJ18	AL391001.1	0.666155365	6.69E-70	postive
TRAJ21	AL391001.1	0.718524275	4.23E-86	postive
TRAJ31	AL391001.1	0.740798273	3.65E-94	postive
TRAJ37	AL391001.1	0.795565313	4.41E-118	postive
TRAJ38	AL391001.1	0.768010841	3.19E-105	postive
TRAJ39	AL391001.1	0.742129275	1.13E-94	postive
CYLD	AC093010.2	0.507166846	2.60E-36	postive
NFAT5	AC093010.2	0.511028684	6.28E-37	postive
PIK3R1	AC093010.2	0.507106127	2.66E-36	postive
BMPR2	AC093010.2	0.528245619	8.84E-40	postive
RORA	AC093010.2	0.565197955	1.75E-46	postive
NOX4	MSC-AS1	0.528090569	9.39E-40	postive
PDGFRA	MSC-AS1	0.541598905	4.16E-42	postive
LIMS1	MSC-AS1	0.582831825	5.41E-50	postive
VCAM1	MSC-AS1	0.5045127	6.84E-36	postive
TNFSF4	MSC-AS1	0.596266772	8.19E-53	postive
FGF7	MSC-AS1	0.501872308	1.78E-35	postive
GREM1	MSC-AS1	0.545372433	8.76E-43	postive
INHBA	MSC-AS1	0.556592517	7.56E-45	postive
CREB1	AC009948.3	0.527429307	1.22E-39	postive
NFAT5	AC009948.3	0.580858975	1.37E-49	postive
GNRH1	AC009948.3	0.553231734	3.20E-44	postive
CRLF3	AC009948.3	0.533676751	1.03E-40	postive
NR2C2	AC009948.3	0.506038039	3.93E-36	postive
BRAF	AC009948.3	0.567279249	6.90E-47	postive
TRAJ1	AC009948.3	0.515404328	1.23E-37	postive
TRAJ2	AC009948.3	0.500345873	3.07E-35	postive
TRAJ3	AC009948.3	0.543848395	1.65E-42	postive
TRAJ5	AC009948.3	0.505409366	4.94E-36	postive
TRAJ6	AC009948.3	0.548690248	2.19E-43	postive
TRAJ8	AC009948.3	0.511221276	5.85E-37	postive
TRAJ10	AC009948.3	0.519130749	2.99E-38	postive
TRAJ13	AC009948.3	0.511674409	4.94E-37	postive
TRAJ14	AC009948.3	0.513134837	2.87E-37	postive
TRAJ16	AC009948.3	0.519855112	2.27E-38	postive
TRAJ21	AC009948.3	0.528127396	9.26E-40	postive
TRAJ31	AC009948.3	0.542626196	2.73E-42	postive
TRAJ37	AC009948.3	0.546632827	5.18E-43	postive
TRAJ38	AC009948.3	0.558447529	3.39E-45	postive
TRAJ39	AC009948.3	0.554121807	2.19E-44	postive
GNRH1	AC011461.1	0.52064214	1.68E-38	postive

CREB1	SAMD12-AS1	0.626043294	1.46E-59	postive
UBR1	SAMD12-AS1	0.516875759	7.04E-38	postive
LMBR1	SAMD12-AS1	0.514968768	1.44E-37	postive
IREB2	SAMD12-AS1	0.500962536	2.46E-35	postive
PTK2	SAMD12-AS1	0.548288769	2.59E-43	postive
NFAT5	SAMD12-AS1	0.5076809	2.16E-36	postive
PIK3CA	SAMD12-AS1	0.531560271	2.39E-40	postive
ACVR2A	SAMD12-AS1	0.539575104	9.52E-42	postive
ANGPTL1	SAMD12-AS1	0.506762824	3.02E-36	postive
BMPR1A	SAMD12-AS1	0.500094635	3.36E-35	postive
NR2C2	SAMD12-AS1	0.512605988	3.49E-37	postive
RORA	SAMD12-AS1	0.505366136	5.02E-36	postive
BRAF	SAMD12-AS1	0.608256424	1.91E-55	postive
TRAJ1	SAMD12-AS1	0.535409817	5.14E-41	postive
TRAJ2	SAMD12-AS1	0.511686573	4.92E-37	postive
TRAJ3	SAMD12-AS1	0.543050968	2.29E-42	postive
TRAJ5	SAMD12-AS1	0.571883147	8.65E-48	postive
TRAJ6	SAMD12-AS1	0.562717662	5.23E-46	postive
TRAJ8	SAMD12-AS1	0.579588743	2.48E-49	postive
TRAJ10	SAMD12-AS1	0.553350568	3.04E-44	postive
TRAJ12	SAMD12-AS1	0.530948056	3.05E-40	postive
TRAJ13	SAMD12-AS1	0.56525698	1.70E-46	postive
TRAJ14	SAMD12-AS1	0.587852588	4.95E-51	postive
TRAJ16	SAMD12-AS1	0.56086656	1.18E-45	postive
TRAJ17	SAMD12-AS1	0.553635883	2.69E-44	postive
TRAJ21	SAMD12-AS1	0.575602235	1.58E-48	postive
TRAJ31	SAMD12-AS1	0.582838124	5.40E-50	postive
TRAJ37	SAMD12-AS1	0.523064838	6.63E-39	postive
TRAJ38	SAMD12-AS1	0.612231947	2.42E-56	postive
TRAJ39	SAMD12-AS1	0.588308697	3.98E-51	postive
CREB1	Z82243.1	0.728567519	1.23E-89	postive
RFXAP	Z82243.1	0.526937498	1.47E-39	postive
UBR1	Z82243.1	0.67567377	1.35E-72	postive
ZC3HAV1	Z82243.1	0.582557571	6.16E-50	postive
ZC3HAV1L	Z82243.1	0.553971137	2.33E-44	postive
IL15	Z82243.1	0.525503549	2.58E-39	postive
CYLD	Z82243.1	0.543683066	1.76E-42	postive
EIF2AK2	Z82243.1	0.553392768	2.99E-44	postive
MAPK8	Z82243.1	0.563299295	4.05E-46	postive
LMBR1	Z82243.1	0.5490967	1.85E-43	postive
IREB2	Z82243.1	0.634073086	1.65E-61	postive
DDX17	Z82243.1	0.664347053	2.12E-69	postive
PIK3CG	Z82243.1	0.50246484	1.43E-35	postive
JAK2	Z82243.1	0.606130915	5.71E-55	postive
TXK	Z82243.1	0.638796298	1.11E-62	postive
NFAT5	Z82243.1	0.779204691	3.20E-110	postive
MALT1	Z82243.1	0.532975673	1.36E-40	postive
PIK3R1	Z82243.1	0.599393621	1.73E-53	postive
PIK3CA	Z82243.1	0.672537216	1.07E-71	postive
PIK3CB	Z82243.1	0.509032347	1.31E-36	postive
AKT3	Z82243.1	0.519867502	2.26E-38	postive
RASGRP3	Z82243.1	0.501387746	2.11E-35	postive
IGHD4-4	Z82243.1	0.505780678	4.32E-36	postive
IGHD6-6	Z82243.1	0.55768414	4.72E-45	postive
LTB4R2	Z82243.1	0.507571715	2.24E-36	postive
GNRH1	Z82243.1	0.811049652	3.27E-126	postive
IL6ST	Z82243.1	0.567107529	7.45E-47	postive
RABEP1	Z82243.1	0.558141586	3.87E-45	postive
ACVR2A	Z82243.1	0.604324905	1.44E-54	postive
ANGPTL1	Z82243.1	0.715838035	3.52E-85	postive
BMPR1A	Z82243.1	0.529472187	5.45E-40	postive
BMPR2	Z82243.1	0.699258472	9.90E-80	postive
CRLF3	Z82243.1	0.636859735	3.37E-62	postive
NR2C1	Z82243.1	0.535971679	4.10E-41	postive
NR2C2	Z82243.1	0.742843372	6.02E-95	postive

RORA	Z82243.1	0.779521641	2.28E-110	postive
SOS1	Z82243.1	0.665058462	1.35E-69	postive
SOS2	Z82243.1	0.540775836	5.83E-42	postive
BRAF	Z82243.1	0.779171454	3.31E-110	postive
TEC	Z82243.1	0.563987978	2.98E-46	postive
CBL	Z82243.1	0.60186942	4.98E-54	postive
CBLB	Z82243.1	0.731966027	7.19E-91	postive
PDK1	Z82243.1	0.628537992	3.67E-60	postive
TRAJ1	Z82243.1	0.62747593	6.62E-60	postive
TRAJ2	Z82243.1	0.65050176	1.11E-65	postive
TRAJ3	Z82243.1	0.702766582	7.48E-81	postive
TRAJ5	Z82243.1	0.660673116	2.15E-68	postive
TRAJ6	Z82243.1	0.677372377	4.36E-73	postive
TRAJ8	Z82243.1	0.635843105	6.02E-62	postive
TRAJ10	Z82243.1	0.640568325	3.97E-63	postive
TRAJ12	Z82243.1	0.5787399	3.69E-49	postive
TRAJ13	Z82243.1	0.636215731	4.86E-62	postive
TRAJ14	Z82243.1	0.629553295	2.09E-60	postive
TRAJ16	Z82243.1	0.664852858	1.54E-69	postive
TRAJ17	Z82243.1	0.611686745	3.22E-56	postive
TRAJ18	Z82243.1	0.601069124	7.46E-54	postive
TRAJ21	Z82243.1	0.677941795	2.98E-73	postive
TRAJ31	Z82243.1	0.688688436	1.90E-76	postive
TRAJ37	Z82243.1	0.674401174	3.14E-72	postive
TRAJ38	Z82243.1	0.743777826	2.63E-95	postive
TRAJ39	Z82243.1	0.720376176	9.68E-87	postive
CREB1	AC005920.2	0.741118254	2.75E-94	postive
UBR1	AC005920.2	0.605090075	9.72E-55	postive
ZC3HAV1	AC005920.2	0.532038156	1.98E-40	postive
IL15	AC005920.2	0.633260901	2.61E-61	postive
CYLD	AC005920.2	0.593705842	2.89E-52	postive
EIF2AK2	AC005920.2	0.507462375	2.34E-36	postive
MAPK8	AC005920.2	0.54782013	3.15E-43	postive
LMBR1	AC005920.2	0.558072816	3.99E-45	postive
LIMS1	AC005920.2	0.523162863	6.38E-39	postive
IREB2	AC005920.2	0.561030803	1.10E-45	postive
DDX17	AC005920.2	0.560740763	1.25E-45	postive
PIK3CG	AC005920.2	0.538763816	1.32E-41	postive
JAK2	AC005920.2	0.637001039	3.10E-62	postive
TXK	AC005920.2	0.602079165	4.48E-54	postive
NFAT5	AC005920.2	0.820162127	2.34E-131	postive
MALT1	AC005920.2	0.546213343	6.17E-43	postive
PIK3R1	AC005920.2	0.633083832	2.88E-61	postive
PIK3CA	AC005920.2	0.68513088	2.24E-75	postive
PIK3CB	AC005920.2	0.527111321	1.38E-39	postive
AKT3	AC005920.2	0.515025657	1.41E-37	postive
IGHD6-6	AC005920.2	0.513565272	2.44E-37	postive
GNRH1	AC005920.2	0.703591946	4.05E-81	postive
IL6ST	AC005920.2	0.620894634	2.42E-58	postive
RABEP1	AC005920.2	0.5226997	7.63E-39	postive
ACVR2A	AC005920.2	0.553990894	2.31E-44	postive
ANGPTL1	AC005920.2	0.799434538	4.79E-120	postive
BMPR2	AC005920.2	0.690633359	4.84E-77	postive
CRLF3	AC005920.2	0.611112223	4.35E-56	postive
IL18R1	AC005920.2	0.559381787	2.26E-45	postive
NR2C2	AC005920.2	0.620679949	2.71E-58	postive
RORA	AC005920.2	0.834997038	2.17E-140	postive
SOS1	AC005920.2	0.610752802	5.24E-56	postive
SOS2	AC005920.2	0.543571654	1.85E-42	postive
BRAF	AC005920.2	0.74774949	7.46E-97	postive
ITK	AC005920.2	0.544741165	1.14E-42	postive
CBL	AC005920.2	0.608813714	1.43E-55	postive
CBLB	AC005920.2	0.725857987	1.15E-88	postive
PDK1	AC005920.2	0.623020258	7.63E-59	postive
TRAJ1	AC005920.2	0.652078785	4.29E-66	postive

TRAJ2	AC005920.2	0.678210031	2.49E-73	postive
TRAJ3	AC005920.2	0.757200332	1.18E-100	postive
TRAJ5	AC005920.2	0.739429998	1.21E-93	postive
TRAJ6	AC005920.2	0.748619232	3.39E-97	postive
TRAJ8	AC005920.2	0.666626022	4.95E-70	postive
TRAJ10	AC005920.2	0.759997832	8.19E-102	postive
TRAJ12	AC005920.2	0.636468895	4.21E-62	postive
TRAJ13	AC005920.2	0.72866577	1.13E-89	postive
TRAJ14	AC005920.2	0.705758377	8.03E-82	postive
TRAJ16	AC005920.2	0.727642065	2.64E-89	postive
TRAJ17	AC005920.2	0.704954788	1.47E-81	postive
TRAJ18	AC005920.2	0.734101735	1.18E-91	postive
TRAJ21	AC005920.2	0.782144713	1.39E-111	postive
TRAJ31	AC005920.2	0.779366127	2.69E-110	postive
TRAJ37	AC005920.2	0.76634953	1.67E-104	postive
TRAJ38	AC005920.2	0.809295956	2.97E-125	postive
TRAJ39	AC005920.2	0.78407021	1.73E-112	postive
CREB1	AL136320.1	0.762265196	9.17E-103	postive
UBR1	AL136320.1	0.630505056	1.23E-60	postive
ZC3HAV1	AL136320.1	0.555311928	1.31E-44	postive
IL15	AL136320.1	0.625638854	1.82E-59	postive
CYLD	AL136320.1	0.635160351	8.88E-62	postive
EIF2AK2	AL136320.1	0.561242989	1.00E-45	postive
MAPK8	AL136320.1	0.544733351	1.14E-42	postive
LMBR1	AL136320.1	0.57308771	4.99E-48	postive
LIMS1	AL136320.1	0.546615517	5.22E-43	postive
IREB2	AL136320.1	0.588051692	4.50E-51	postive
DDX17	AL136320.1	0.575339826	1.78E-48	postive
PIK3CG	AL136320.1	0.590288697	1.53E-51	postive
JAK2	AL136320.1	0.675849235	1.20E-72	postive
TXK	AL136320.1	0.614212093	8.55E-57	postive
NFAT5	AL136320.1	0.812640243	4.33E-127	postive
MALT1	AL136320.1	0.554419502	1.93E-44	postive
PIK3R1	AL136320.1	0.684964896	2.52E-75	postive
PIK3CA	AL136320.1	0.706172308	5.88E-82	postive
PIK3CB	AL136320.1	0.529789312	4.81E-40	postive
AKT3	AL136320.1	0.501599317	1.96E-35	postive
RASGRP3	AL136320.1	0.552327732	4.70E-44	postive
IGHD6-6	AL136320.1	0.514092161	2.01E-37	postive
PLXNC1	AL136320.1	0.506846859	2.93E-36	postive
GNRH1	AL136320.1	0.654843894	7.96E-67	postive
IL6ST	AL136320.1	0.662459438	7.00E-69	postive
RABEP1	AL136320.1	0.529461464	5.48E-40	postive
ACVR2A	AL136320.1	0.572311653	7.11E-48	postive
ANGPTL1	AL136320.1	0.800042965	2.33E-120	postive
BMPR2	AL136320.1	0.717039283	1.37E-85	postive
CRLF3	AL136320.1	0.620668044	2.73E-58	postive
IL18R1	AL136320.1	0.508162355	1.81E-36	postive
LIFR	AL136320.1	0.509971554	9.28E-37	postive
NR1D2	AL136320.1	0.507001773	2.76E-36	postive
NR2C2	AL136320.1	0.652711502	2.92E-66	postive
RORA	AL136320.1	0.86390768	6.86E-161	postive
SOS1	AL136320.1	0.652932043	2.56E-66	postive
SOS2	AL136320.1	0.560840298	1.19E-45	postive
BRAF	AL136320.1	0.800009339	2.42E-120	postive
ITK	AL136320.1	0.569930609	2.09E-47	postive
CD28	AL136320.1	0.519988517	2.16E-38	postive
CBL	AL136320.1	0.597237396	5.06E-53	postive
CBLB	AL136320.1	0.784621138	9.52E-113	postive
RASGRP1	AL136320.1	0.519634768	2.47E-38	postive
PDK1	AL136320.1	0.617038721	1.91E-57	postive
TRAJ1	AL136320.1	0.682213396	1.66E-74	postive
TRAJ2	AL136320.1	0.743220402	4.31E-95	postive
TRAJ3	AL136320.1	0.777040606	3.12E-109	postive
TRAJ5	AL136320.1	0.771182547	1.31E-106	postive

TRAJ6	AL136320.1	0.760368372	5.74E-102	postive
TRAJ8	AL136320.1	0.702210615	1.13E-80	postive
TRAJ10	AL136320.1	0.74801823	5.85E-97	postive
TRAJ12	AL136320.1	0.708626542	9.19E-83	postive
TRAJ13	AL136320.1	0.740099205	6.72E-94	postive
TRAJ14	AL136320.1	0.756171276	3.12E-100	postive
TRAJ16	AL136320.1	0.759645195	1.15E-101	postive
TRAJ17	AL136320.1	0.716678406	1.82E-85	postive
TRAJ18	AL136320.1	0.765821912	2.81E-104	postive
TRAJ21	AL136320.1	0.799513539	4.36E-120	postive
TRAJ31	AL136320.1	0.801515316	4.03E-121	postive
TRAJ37	AL136320.1	0.780753754	6.16E-111	postive
TRAJ38	AL136320.1	0.862146358	1.64E-159	postive
TRAJ39	AL136320.1	0.811546021	1.74E-126	postive
SEMA7A	AP000695.2	0.680107872	6.93E-74	postive
CREB1	LAMC1-AS1	0.73257752	4.29E-91	postive
RFXAP	LAMC1-AS1	0.555037528	1.48E-44	postive
UBR1	LAMC1-AS1	0.617510937	1.49E-57	postive
ZC3HAV1	LAMC1-AS1	0.559023212	2.64E-45	postive
ZC3HAV1L	LAMC1-AS1	0.57918258	3.00E-49	postive
IL15	LAMC1-AS1	0.540241099	7.25E-42	postive
CYLD	LAMC1-AS1	0.515977765	9.88E-38	postive
EIF2AK2	LAMC1-AS1	0.500178786	3.26E-35	postive
MAPK8	LAMC1-AS1	0.584243261	2.78E-50	postive
LMBR1	LAMC1-AS1	0.540297511	7.09E-42	postive
IREB2	LAMC1-AS1	0.597869022	3.70E-53	postive
DDX17	LAMC1-AS1	0.62203301	1.31E-58	postive
JAK2	LAMC1-AS1	0.53595675	4.12E-41	postive
TXK	LAMC1-AS1	0.519816234	2.31E-38	postive
NFAT5	LAMC1-AS1	0.767909805	3.53E-105	postive
MALT1	LAMC1-AS1	0.581276614	1.13E-49	postive
PIK3R1	LAMC1-AS1	0.559129113	2.52E-45	postive
PIK3CA	LAMC1-AS1	0.612948197	1.66E-56	postive
AKT3	LAMC1-AS1	0.594910843	1.60E-52	postive
LTB4R2	LAMC1-AS1	0.51307661	2.93E-37	postive
GNRH1	LAMC1-AS1	0.725233749	1.91E-88	postive
IL6ST	LAMC1-AS1	0.567573833	6.05E-47	postive
RABEP1	LAMC1-AS1	0.580670034	1.50E-49	postive
ACVR2A	LAMC1-AS1	0.591851253	7.16E-52	postive
ANGPTL1	LAMC1-AS1	0.756965736	1.47E-100	postive
BMPR1A	LAMC1-AS1	0.548985246	1.93E-43	postive
BMPR2	LAMC1-AS1	0.633915461	1.80E-61	postive
CRLF3	LAMC1-AS1	0.635689795	6.57E-62	postive
LIFR	LAMC1-AS1	0.517075252	6.53E-38	postive
NR2C1	LAMC1-AS1	0.518613898	3.64E-38	postive
NR2C2	LAMC1-AS1	0.701538236	1.86E-80	postive
RORA	LAMC1-AS1	0.732482664	4.65E-91	postive
SOS1	LAMC1-AS1	0.665789188	8.46E-70	postive
SOS2	LAMC1-AS1	0.567189654	7.18E-47	postive
BRAF	LAMC1-AS1	0.813477294	1.48E-127	postive
CBL	LAMC1-AS1	0.62183416	1.45E-58	postive
CBLB	LAMC1-AS1	0.676547799	7.56E-73	postive
PDK1	LAMC1-AS1	0.565474817	1.54E-46	postive
TRAJ1	LAMC1-AS1	0.669194111	9.47E-71	postive
TRAJ2	LAMC1-AS1	0.735895064	2.55E-92	postive
TRAJ3	LAMC1-AS1	0.770258628	3.34E-106	postive
TRAJ5	LAMC1-AS1	0.69519403	1.88E-78	postive
TRAJ6	LAMC1-AS1	0.731475774	1.09E-90	postive
TRAJ8	LAMC1-AS1	0.740005548	7.30E-94	postive
TRAJ10	LAMC1-AS1	0.698550752	1.66E-79	postive
TRAJ12	LAMC1-AS1	0.609224135	1.16E-55	postive
TRAJ13	LAMC1-AS1	0.727965151	2.02E-89	postive
TRAJ14	LAMC1-AS1	0.726743779	5.54E-89	postive
TRAJ16	LAMC1-AS1	0.704312237	2.37E-81	postive
TRAJ17	LAMC1-AS1	0.674644407	2.67E-72	postive

TRAJ18	LAMC1-AS1	0.61808488	1.09E-57	postive
TRAJ21	LAMC1-AS1	0.743551687	3.22E-95	postive
TRAJ31	LAMC1-AS1	0.725958513	1.06E-88	postive
TRAJ37	LAMC1-AS1	0.723992867	5.25E-88	postive
TRAJ38	LAMC1-AS1	0.783300003	4.00E-112	postive
TRAJ39	LAMC1-AS1	0.756739648	1.83E-100	postive
MSTN	HAGLROS	0.600453481	1.02E-53	postive
CD4	SMIM25	0.567742242	5.61E-47	postive
CTSB	SMIM25	0.523498155	5.61E-39	postive
FCER1G	SMIM25	0.651225749	7.20E-66	postive
GRN	SMIM25	0.508531727	1.58E-36	postive
MSR1	SMIM25	0.642892044	1.02E-63	postive
SLC11A1	SMIM25	0.720290923	1.04E-86	postive
MARCO	SMIM25	0.769767283	5.48E-106	postive
CD14	SMIM25	0.526340047	1.86E-39	postive
SLC29A3	SMIM25	0.544182109	1.43E-42	postive
AGER	SMIM25	0.505843356	4.22E-36	postive
CCL18	SMIM25	0.528307772	8.62E-40	postive
CCL23	SMIM25	0.686177072	1.09E-75	postive
CCR1	SMIM25	0.547227122	4.04E-43	postive
FGR	SMIM25	0.789408229	4.84E-115	postive
CD86	SMIM25	0.51871149	3.51E-38	postive
OLR1	SMIM25	0.506453307	3.38E-36	postive
BTK	SMIM25	0.592583833	5.01E-52	postive
PIK3R5	SMIM25	0.598682868	2.47E-53	postive
LILRB3	SMIM25	0.725692933	1.31E-88	postive
C5AR1	SMIM25	0.664472267	1.96E-69	postive
CCRL2	SMIM25	0.515078316	1.39E-37	postive
PTAFR	SMIM25	0.508110517	1.84E-36	postive
AGRP	SMIM25	0.57610065	1.25E-48	postive
GMFG	SMIM25	0.525436761	2.65E-39	postive
TNFSF12	SMIM25	0.535768903	4.45E-41	postive
ACVRL1	SMIM25	0.520318104	1.90E-38	postive
C3AR1	SMIM25	0.552920792	3.65E-44	postive
IL3RA	SMIM25	0.519663218	2.44E-38	postive
ITGB2	SMIM25	0.599102665	2.00E-53	postive
TYROBP	SMIM25	0.702100952	1.23E-80	postive
FCGR3A	SMIM25	0.514507499	1.72E-37	postive
CD8A	AC004585.1	0.514107026	1.99E-37	postive
LTA	AC004585.1	0.659565787	4.30E-68	postive
CXCL9	AC004585.1	0.527416615	1.22E-39	postive
APOBEC3G	AC004585.1	0.576529944	1.03E-48	postive
RBP5	AC004585.1	0.503842633	8.72E-36	postive
IRF1	AC004585.1	0.542364672	3.04E-42	postive
CCL5	AC004585.1	0.581374939	1.08E-49	postive
CCL4	AC004585.1	0.517239802	6.14E-38	postive
PDCD1	AC004585.1	0.55860115	3.17E-45	postive
FASLG	AC004585.1	0.533583643	1.07E-40	postive
CCL19	AC004585.1	0.582738613	5.66E-50	postive
CCR7	AC004585.1	0.551050805	8.09E-44	postive
CCR5	AC004585.1	0.573070516	5.03E-48	postive
CXCR6	AC004585.1	0.558527261	3.27E-45	postive
CD79B	AC004585.1	0.533484925	1.11E-40	postive
BTK	AC004585.1	0.505087723	5.55E-36	postive
RAC2	AC004585.1	0.503542487	9.73E-36	postive
CD19	AC004585.1	0.5692458	2.85E-47	postive
CD72	AC004585.1	0.624129019	4.17E-59	postive
GMFG	AC004585.1	0.515767793	1.07E-37	postive
IL16	AC004585.1	0.558853111	2.84E-45	postive
TNFSF13B	AC004585.1	0.539905278	8.32E-42	postive
IL10RA	AC004585.1	0.531761069	2.21E-40	postive
IL12RB1	AC004585.1	0.565995311	1.22E-46	postive
IL2RB	AC004585.1	0.608591856	1.61E-55	postive
IL18RAP	AC004585.1	0.523150421	6.42E-39	postive
IL21R	AC004585.1	0.600613527	9.38E-54	postive

IL2RG	AC004585.1	0.573865828	3.50E-48	postive
ITGAL	AC004585.1	0.576349256	1.12E-48	postive
LCK	AC004585.1	0.582737074	5.66E-50	postive
NCR3	AC004585.1	0.588498374	3.63E-51	postive
CD247	AC004585.1	0.697984173	2.51E-79	postive
ZAP70	AC004585.1	0.627702205	5.84E-60	postive
CD48	AC004585.1	0.609049614	1.27E-55	postive
SH2D1A	AC004585.1	0.680227277	6.39E-74	postive
CD3D	AC004585.1	0.672218677	1.32E-71	postive
CD3E	AC004585.1	0.649821405	1.68E-65	postive
CD3G	AC004585.1	0.572923478	5.38E-48	postive
ICOS	AC004585.1	0.554063742	2.24E-44	postive
TRAC	AC004585.1	0.672290354	1.26E-71	postive
TRAV3	AC004585.1	0.511610573	5.06E-37	postive
TRAV4	AC004585.1	0.636524471	4.08E-62	postive
TRAV8-2	AC004585.1	0.585984085	1.21E-50	postive
TRAV8-3	AC004585.1	0.528130514	9.24E-40	postive
TRAV8-4	AC004585.1	0.562108513	6.84E-46	postive
TRAV8-6	AC004585.1	0.550556303	9.97E-44	postive
TRAV9-2	AC004585.1	0.590306808	1.52E-51	postive
TRAV13-2	AC004585.1	0.528182713	9.06E-40	postive
TRAV16	AC004585.1	0.529364866	5.69E-40	postive
TRAV29DV5	AC004585.1	0.510107323	8.83E-37	postive
TRBC1	AC004585.1	0.663696735	3.20E-69	postive
TRBC2	AC004585.1	0.645465237	2.25E-64	postive
TRBJ2-2	AC004585.1	0.509345175	1.17E-36	postive
TRBJ2-3	AC004585.1	0.550774397	9.09E-44	postive
TRBJ2-7	AC004585.1	0.571143496	1.21E-47	postive
TRBV2	AC004585.1	0.569274104	2.82E-47	postive
TRBV5-1	AC004585.1	0.6440445	5.20E-64	postive
TRBV5-4	AC004585.1	0.544120286	1.47E-42	postive
TRBV6-1	AC004585.1	0.54592276	6.97E-43	postive
TRBV6-5	AC004585.1	0.582573259	6.12E-50	postive
TRBV7-9	AC004585.1	0.541341911	4.62E-42	postive
TRBV9	AC004585.1	0.517659449	5.23E-38	postive
TRBV18	AC004585.1	0.565884748	1.29E-46	postive
TRBV20-1	AC004585.1	0.597121642	5.36E-53	postive
TRBV28	AC004585.1	0.682894728	1.04E-74	postive
CREB1	AC037198.1	0.641918893	1.81E-63	postive
IL15	AC037198.1	0.549881397	1.33E-43	postive
TLR1	AC037198.1	0.530388087	3.80E-40	postive
NFKBIZ	AC037198.1	0.560384368	1.46E-45	postive
LIMS1	AC037198.1	0.51627247	8.84E-38	postive
JAK2	AC037198.1	0.622355349	1.10E-58	postive
NFAT5	AC037198.1	0.686193398	1.08E-75	postive
PIK3CA	AC037198.1	0.619508659	5.10E-58	postive
FGF7	AC037198.1	0.504078954	8.01E-36	postive
GNRH1	AC037198.1	0.590265538	1.55E-51	postive
ANGPTL1	AC037198.1	0.671844526	1.69E-71	postive
BMPR2	AC037198.1	0.549526749	1.54E-43	postive
CRLF3	AC037198.1	0.562506398	5.74E-46	postive
NR2C2	AC037198.1	0.556210481	8.92E-45	postive
RORA	AC037198.1	0.685569198	1.66E-75	postive
SOS1	AC037198.1	0.539966697	8.11E-42	postive
BRAF	AC037198.1	0.575929025	1.36E-48	postive
CBL	AC037198.1	0.518610381	3.65E-38	postive
CBLB	AC037198.1	0.670668504	3.63E-71	postive
PDK1	AC037198.1	0.594890759	1.62E-52	postive
TRAJ1	AC037198.1	0.593003815	4.08E-52	postive
TRAJ2	AC037198.1	0.529982526	4.46E-40	postive
TRAJ3	AC037198.1	0.592103607	6.33E-52	postive
TRAJ5	AC037198.1	0.667793891	2.34E-70	postive
TRAJ6	AC037198.1	0.638716101	1.16E-62	postive
TRAJ8	AC037198.1	0.531758674	2.21E-40	postive
TRAJ10	AC037198.1	0.679464176	1.07E-73	postive

TRAJ12	AC037198.1	0.509650238	1.05E-36	postive
TRAJ13	AC037198.1	0.589167891	2.63E-51	postive
TRAJ14	AC037198.1	0.581571559	9.80E-50	postive
TRAJ16	AC037198.1	0.660561349	2.31E-68	postive
TRAJ17	AC037198.1	0.601829291	5.08E-54	postive
TRAJ18	AC037198.1	0.602977543	2.84E-54	postive
TRAJ21	AC037198.1	0.628792331	3.19E-60	postive
TRAJ31	AC037198.1	0.658327054	9.29E-68	postive
TRAJ37	AC037198.1	0.623796263	5.00E-59	postive
TRAJ38	AC037198.1	0.701735122	1.61E-80	postive
TRAJ39	AC037198.1	0.666626653	4.95E-70	postive
CREB1	AL645568.1	0.668636834	1.36E-70	postive
RFXAP	AL645568.1	0.537079031	2.62E-41	postive
UBR1	AL645568.1	0.537638953	2.09E-41	postive
IL15	AL645568.1	0.629925777	1.70E-60	postive
CYLD	AL645568.1	0.553655717	2.67E-44	postive
IREB2	AL645568.1	0.507063589	2.70E-36	postive
DDX17	AL645568.1	0.607095489	3.48E-55	postive
PIK3CG	AL645568.1	0.515532132	1.17E-37	postive
JAK2	AL645568.1	0.607404187	2.97E-55	postive
TXK	AL645568.1	0.556531984	7.76E-45	postive
NFAT5	AL645568.1	0.741427923	2.10E-94	postive
MALT1	AL645568.1	0.510151185	8.69E-37	postive
PIK3R1	AL645568.1	0.573435113	4.26E-48	postive
PIK3CA	AL645568.1	0.622448976	1.04E-58	postive
RASGRP3	AL645568.1	0.512076823	4.25E-37	postive
IGHD6-6	AL645568.1	0.54306216	2.28E-42	postive
LTB4R2	AL645568.1	0.511827414	4.67E-37	postive
GNRH1	AL645568.1	0.710546599	2.12E-83	postive
IL6ST	AL645568.1	0.538161407	1.69E-41	postive
ACVR2A	AL645568.1	0.527060128	1.41E-39	postive
ANGPTL1	AL645568.1	0.722274541	2.11E-87	postive
BMPR2	AL645568.1	0.57965429	2.41E-49	postive
CRLF3	AL645568.1	0.617101737	1.85E-57	postive
IL18R1	AL645568.1	0.503792132	8.89E-36	postive
NR2C2	AL645568.1	0.670264647	4.73E-71	postive
RORA	AL645568.1	0.750535493	5.89E-98	postive
SOS1	AL645568.1	0.59874142	2.39E-53	postive
SOS2	AL645568.1	0.51930591	2.80E-38	postive
BRAF	AL645568.1	0.721551484	3.77E-87	postive
ITK	AL645568.1	0.559383299	2.25E-45	postive
CBL	AL645568.1	0.58291824	5.20E-50	postive
CBLB	AL645568.1	0.691370274	2.87E-77	postive
PDK1	AL645568.1	0.642229245	1.51E-63	postive
TRAJ1	AL645568.1	0.652447142	3.43E-66	postive
TRAJ2	AL645568.1	0.654232904	1.16E-66	postive
TRAJ3	AL645568.1	0.741309962	2.33E-94	postive
TRAJ5	AL645568.1	0.697458313	3.67E-79	postive
TRAJ6	AL645568.1	0.698818172	1.37E-79	postive
TRAJ8	AL645568.1	0.636139906	5.08E-62	postive
TRAJ10	AL645568.1	0.699960028	5.92E-80	postive
TRAJ12	AL645568.1	0.612023849	2.70E-56	postive
TRAJ13	AL645568.1	0.654871207	7.83E-67	postive
TRAJ14	AL645568.1	0.666107011	6.90E-70	postive
TRAJ16	AL645568.1	0.691804385	2.11E-77	postive
TRAJ17	AL645568.1	0.672001837	1.52E-71	postive
TRAJ18	AL645568.1	0.687238448	5.21E-76	postive
TRAJ21	AL645568.1	0.715896583	3.36E-85	postive
TRAJ31	AL645568.1	0.704983874	1.43E-81	postive
TRAJ37	AL645568.1	0.695914734	1.12E-78	postive
TRAJ38	AL645568.1	0.754534523	1.45E-99	postive
TRAJ39	AL645568.1	0.740479659	4.82E-94	postive
IRF9	AL031709.1	0.614484847	7.41E-57	postive
GIPR	AL031709.1	0.50722971	2.54E-36	postive
CREB1	AC055822.1	0.562869398	4.89E-46	postive

IL15	AC055822.1	0.501676439	1.91E-35	postive
DDX17	AC055822.1	0.612768677	1.83E-56	postive
NFAT5	AC055822.1	0.629145604	2.62E-60	postive
PIK3R1	AC055822.1	0.51175353	4.80E-37	postive
GNRH1	AC055822.1	0.681731414	2.30E-74	postive
ACVR2A	AC055822.1	0.565395737	1.60E-46	postive
ANGPTL1	AC055822.1	0.564356446	2.54E-46	postive
CRLF3	AC055822.1	0.533876579	9.50E-41	postive
NR2C1	AC055822.1	0.50695958	2.81E-36	postive
NR2C2	AC055822.1	0.627532598	6.41E-60	postive
RORA	AC055822.1	0.648208969	4.41E-65	postive
SOS1	AC055822.1	0.513188479	2.81E-37	postive
BRAF	AC055822.1	0.632084165	5.06E-61	postive
ITK	AC055822.1	0.503670477	9.29E-36	postive
CBLB	AC055822.1	0.541747789	3.91E-42	postive
TRAJ1	AC055822.1	0.657153295	1.92E-67	postive
TRAJ2	AC055822.1	0.590910669	1.13E-51	postive
TRAJ3	AC055822.1	0.652686053	2.97E-66	postive
TRAJ5	AC055822.1	0.69371285	5.44E-78	postive
TRAJ6	AC055822.1	0.643758086	6.16E-64	postive
TRAJ8	AC055822.1	0.672369312	1.20E-71	postive
TRAJ10	AC055822.1	0.620373563	3.20E-58	postive
TRAJ12	AC055822.1	0.603846641	1.83E-54	postive
TRAJ13	AC055822.1	0.674324516	3.30E-72	postive
TRAJ14	AC055822.1	0.658942298	6.34E-68	postive
TRAJ16	AC055822.1	0.667276511	3.26E-70	postive
TRAJ17	AC055822.1	0.662586456	6.46E-69	postive
TRAJ18	AC055822.1	0.602852567	3.03E-54	postive
TRAJ21	AC055822.1	0.682530801	1.34E-74	postive
TRAJ31	AC055822.1	0.691147657	3.36E-77	postive
TRAJ37	AC055822.1	0.660213087	2.87E-68	postive
TRAJ38	AC055822.1	0.682638044	1.24E-74	postive
TRAJ39	AC055822.1	0.700493006	4.01E-80	postive
CREB1	AC234772.2	0.600745424	8.77E-54	postive
IL15	AC234772.2	0.577373238	6.96E-49	postive
CYLD	AC234772.2	0.506938401	2.83E-36	postive
JAK2	AC234772.2	0.587316275	6.41E-51	postive
NFAT5	AC234772.2	0.626728762	1.00E-59	postive
PIK3R1	AC234772.2	0.529847418	4.70E-40	postive
PIK3CA	AC234772.2	0.537531869	2.18E-41	postive
ANGPTL1	AC234772.2	0.664191564	2.34E-69	postive
BMPR2	AC234772.2	0.535804521	4.38E-41	postive
LIFR	AC234772.2	0.514678858	1.61E-37	postive
NR2C2	AC234772.2	0.501871622	1.78E-35	postive
RORA	AC234772.2	0.634900366	1.03E-61	postive
SOS1	AC234772.2	0.537754021	1.99E-41	postive
BRAF	AC234772.2	0.639087796	9.35E-63	postive
CBL	AC234772.2	0.510109364	8.82E-37	postive
CBLB	AC234772.2	0.733834921	1.48E-91	postive
TRAJ1	AC234772.2	0.54633364	5.87E-43	postive
TRAJ2	AC234772.2	0.640184272	4.96E-63	postive
TRAJ3	AC234772.2	0.640438213	4.28E-63	postive
TRAJ5	AC234772.2	0.581985235	8.07E-50	postive
TRAJ6	AC234772.2	0.633786137	1.94E-61	postive
TRAJ8	AC234772.2	0.512871275	3.17E-37	postive
TRAJ10	AC234772.2	0.607895989	2.30E-55	postive
TRAJ12	AC234772.2	0.679400889	1.12E-73	postive
TRAJ13	AC234772.2	0.569411445	2.65E-47	postive
TRAJ14	AC234772.2	0.647802695	5.62E-65	postive
TRAJ16	AC234772.2	0.578258553	4.61E-49	postive
TRAJ17	AC234772.2	0.526880668	1.51E-39	postive
TRAJ18	AC234772.2	0.686924924	6.48E-76	postive
TRAJ21	AC234772.2	0.642047454	1.68E-63	postive
TRAJ31	AC234772.2	0.626363359	1.22E-59	postive
TRAJ37	AC234772.2	0.60964643	9.32E-56	postive

TRAJ38	AC234772.2	0.72686874	5.00E-89	postive
TRAJ39	AC234772.2	0.590062275	1.71E-51	postive
CD48	LINC00324	0.576134719	1.23E-48	postive
CREB1	AC005670.3	0.554292657	2.03E-44	postive
UBR1	AC005670.3	0.5089721	1.34E-36	postive
NFAT5	AC005670.3	0.524896485	3.26E-39	postive
PIK3CA	AC005670.3	0.513998877	2.08E-37	postive
NR2C2	AC005670.3	0.525118035	3.00E-39	postive
BRAF	AC005670.3	0.506153414	3.77E-36	postive
NR2C2	AP001469.3	0.500847556	2.57E-35	postive
BRAF	AP001469.3	0.538641618	1.39E-41	postive
BPIFB1	UCKL1-AS1	0.634750693	1.12E-61	postive
DES	UCKL1-AS1	0.63108688	8.86E-61	postive
GIPR	UCKL1-AS1	0.510014066	9.14E-37	postive
CREB1	AL136531.1	0.563420943	3.84E-46	postive
RFXAP	AL136531.1	0.534034893	8.91E-41	postive
DDX17	AL136531.1	0.574624287	2.47E-48	postive
NFAT5	AL136531.1	0.647596043	6.35E-65	postive
GNRH1	AL136531.1	0.687894134	3.30E-76	postive
ACVR2A	AL136531.1	0.528307781	8.62E-40	postive
ANGPTL1	AL136531.1	0.60265584	3.35E-54	postive
NR2C2	AL136531.1	0.667567465	2.70E-70	postive
RORA	AL136531.1	0.619581287	4.90E-58	postive
SOS1	AL136531.1	0.60760407	2.68E-55	postive
BRAF	AL136531.1	0.61179573	3.04E-56	postive
CBLB	AL136531.1	0.535626255	4.71E-41	postive
TRAJ1	AL136531.1	0.577124935	7.80E-49	postive
TRAJ2	AL136531.1	0.553799712	2.51E-44	postive
TRAJ3	AL136531.1	0.632018229	5.25E-61	postive
TRAJ5	AL136531.1	0.597697365	4.03E-53	postive
TRAJ6	AL136531.1	0.610292922	6.66E-56	postive
TRAJ8	AL136531.1	0.581665917	9.38E-50	postive
TRAJ10	AL136531.1	0.599460421	1.67E-53	postive
TRAJ12	AL136531.1	0.552957325	3.60E-44	postive
TRAJ13	AL136531.1	0.581973444	8.12E-50	postive
TRAJ14	AL136531.1	0.592084776	6.39E-52	postive
TRAJ16	AL136531.1	0.597139669	5.31E-53	postive
TRAJ17	AL136531.1	0.583657452	3.66E-50	postive
TRAJ18	AL136531.1	0.559880499	1.82E-45	postive
TRAJ21	AL136531.1	0.616143821	3.08E-57	postive
TRAJ31	AL136531.1	0.608008651	2.17E-55	postive
TRAJ37	AL136531.1	0.612841278	1.76E-56	postive
TRAJ38	AL136531.1	0.640240225	4.80E-63	postive
TRAJ39	AL136531.1	0.636084745	5.24E-62	postive
CREB1	AC245884.8	0.605116581	9.59E-55	postive
RFXAP	AC245884.8	0.524012159	4.60E-39	postive
IL15	AC245884.8	0.504431373	7.05E-36	postive
DDX17	AC245884.8	0.658347888	9.17E-68	postive
TXK	AC245884.8	0.525655414	2.43E-39	postive
NFAT5	AC245884.8	0.665496257	1.02E-69	postive
PIK3R1	AC245884.8	0.506347709	3.51E-36	postive
IGHD4-4	AC245884.8	0.553240428	3.19E-44	postive
IGHD6-6	AC245884.8	0.539294904	1.07E-41	postive
LTB4R2	AC245884.8	0.571576143	9.94E-48	postive
GNRH1	AC245884.8	0.805228815	4.53E-123	postive
ACVR2A	AC245884.8	0.594604722	1.86E-52	postive
ANGPTL1	AC245884.8	0.597122055	5.36E-53	postive
BMPR2	AC245884.8	0.559765711	1.91E-45	postive
CRLF3	AC245884.8	0.525415871	2.67E-39	postive
NR2C1	AC245884.8	0.528986979	6.60E-40	postive
NR2C2	AC245884.8	0.747442938	9.85E-97	postive
RORA	AC245884.8	0.6544468	1.02E-66	postive
SOS1	AC245884.8	0.580258736	1.81E-49	postive
BRAF	AC245884.8	0.699673995	7.30E-80	postive
CBL	AC245884.8	0.506423565	3.42E-36	postive

CBLB	AC245884.8	0.588079714	4.44E-51	postive
TRAJ1	AC245884.8	0.673646212	5.17E-72	postive
TRAJ2	AC245884.8	0.610661661	5.50E-56	postive
TRAJ3	AC245884.8	0.717560465	9.07E-86	postive
TRAJ5	AC245884.8	0.695681823	1.33E-78	postive
TRAJ6	AC245884.8	0.663303995	4.11E-69	postive
TRAJ8	AC245884.8	0.698801404	1.38E-79	postive
TRAJ10	AC245884.8	0.628577521	3.59E-60	postive
TRAJ12	AC245884.8	0.629239774	2.49E-60	postive
TRAJ13	AC245884.8	0.666146944	6.73E-70	postive
TRAJ14	AC245884.8	0.66882697	1.20E-70	postive
TRAJ16	AC245884.8	0.666588994	5.07E-70	postive
TRAJ17	AC245884.8	0.670802365	3.33E-71	postive
TRAJ18	AC245884.8	0.587457526	5.99E-51	postive
TRAJ21	AC245884.8	0.685508441	1.73E-75	postive
TRAJ31	AC245884.8	0.692258656	1.53E-77	postive
TRAJ37	AC245884.8	0.668603837	1.39E-70	postive
TRAJ38	AC245884.8	0.70033683	4.49E-80	postive
TRAJ39	AC245884.8	0.719994081	1.31E-86	postive
CREB1	HIF1A-AS3	0.599788318	1.42E-53	postive
IL15	HIF1A-AS3	0.581175443	1.18E-49	postive
TLR1	HIF1A-AS3	0.537488139	2.22E-41	postive
NFKBIZ	HIF1A-AS3	0.549882932	1.32E-43	postive
JAK2	HIF1A-AS3	0.57504922	2.03E-48	postive
NFAT5	HIF1A-AS3	0.600225345	1.14E-53	postive
PIK3CA	HIF1A-AS3	0.597995539	3.47E-53	postive
GNRH1	HIF1A-AS3	0.547745708	3.25E-43	postive
ANGPTL1	HIF1A-AS3	0.686143088	1.12E-75	postive
BMPR2	HIF1A-AS3	0.505798568	4.29E-36	postive
CRLF3	HIF1A-AS3	0.537698536	2.04E-41	postive
IL18R1	HIF1A-AS3	0.529606189	5.17E-40	postive
RORA	HIF1A-AS3	0.604084434	1.62E-54	postive
BRAF	HIF1A-AS3	0.548822541	2.07E-43	postive
CBL	HIF1A-AS3	0.532395993	1.71E-40	postive
CBLB	HIF1A-AS3	0.633646846	2.10E-61	postive
PDK1	HIF1A-AS3	0.615036528	5.53E-57	postive
TRAJ1	HIF1A-AS3	0.512335416	3.86E-37	postive
TRAJ3	HIF1A-AS3	0.552977772	3.57E-44	postive
TRAJ5	HIF1A-AS3	0.597836435	3.76E-53	postive
TRAJ6	HIF1A-AS3	0.572441496	6.71E-48	postive
TRAJ10	HIF1A-AS3	0.618720553	7.78E-58	postive
TRAJ13	HIF1A-AS3	0.539227063	1.10E-41	postive
TRAJ14	HIF1A-AS3	0.523655652	5.28E-39	postive
TRAJ16	HIF1A-AS3	0.573577084	3.99E-48	postive
TRAJ17	HIF1A-AS3	0.5472063	4.08E-43	postive
TRAJ18	HIF1A-AS3	0.521955938	1.02E-38	postive
TRAJ21	HIF1A-AS3	0.577599747	6.26E-49	postive
TRAJ31	HIF1A-AS3	0.58442661	2.54E-50	postive
TRAJ37	HIF1A-AS3	0.540579184	6.32E-42	postive
TRAJ38	HIF1A-AS3	0.62732777	7.18E-60	postive
TRAJ39	HIF1A-AS3	0.595810966	1.03E-52	postive
CREB1	LANCL1-AS1	0.509224314	1.22E-36	postive
DDX17	LANCL1-AS1	0.515281029	1.28E-37	postive
NFAT5	LANCL1-AS1	0.578091362	4.99E-49	postive
GNRH1	LANCL1-AS1	0.543406251	1.98E-42	postive
ANGPTL1	LANCL1-AS1	0.50851347	1.59E-36	postive
RORA	LANCL1-AS1	0.569865339	2.16E-47	postive
BRAF	LANCL1-AS1	0.505680658	4.48E-36	postive
TRAJ2	LANCL1-AS1	0.538394762	1.54E-41	postive
TRAJ3	LANCL1-AS1	0.528284816	8.70E-40	postive
TRAJ6	LANCL1-AS1	0.539147064	1.13E-41	postive
TRAJ8	LANCL1-AS1	0.511353101	5.57E-37	postive
TRAJ10	LANCL1-AS1	0.510096223	8.87E-37	postive
TRAJ13	LANCL1-AS1	0.514248218	1.89E-37	postive
TRAJ14	LANCL1-AS1	0.52044867	1.81E-38	postive

TRAJ16	LANCL1-AS1	0.509119123	1.27E-36	postive
TRAJ21	LANCL1-AS1	0.535093866	5.83E-41	postive
TRAJ31	LANCL1-AS1	0.523512048	5.58E-39	postive
TRAJ37	LANCL1-AS1	0.541288718	4.72E-42	postive
TRAJ38	LANCL1-AS1	0.569879768	2.14E-47	postive
TRAJ39	LANCL1-AS1	0.537771308	1.98E-41	postive
CREB1	AF178030.1	0.736622544	1.37E-92	postive
UBR1	AF178030.1	0.550402556	1.06E-43	postive
ZC3HAV1	AF178030.1	0.538460787	1.50E-41	postive
IL15	AF178030.1	0.611757298	3.10E-56	postive
CYLD	AF178030.1	0.528552404	7.83E-40	postive
EIF2AK2	AF178030.1	0.55295997	3.59E-44	postive
MAPK8	AF178030.1	0.506375239	3.48E-36	postive
TLR1	AF178030.1	0.514130211	1.98E-37	postive
NFKBIZ	AF178030.1	0.50340485	1.02E-35	postive
LMBR1	AF178030.1	0.574233444	2.96E-48	postive
LIMS1	AF178030.1	0.509423412	1.14E-36	postive
IREB2	AF178030.1	0.558851243	2.84E-45	postive
PIK3CG	AF178030.1	0.51111977	6.07E-37	postive
JAK2	AF178030.1	0.639474235	7.48E-63	postive
TXK	AF178030.1	0.506399309	3.45E-36	postive
NFAT5	AF178030.1	0.708872305	7.63E-83	postive
MALT1	AF178030.1	0.507756396	2.10E-36	postive
PIK3R1	AF178030.1	0.571308919	1.12E-47	postive
PIK3CA	AF178030.1	0.715403472	4.94E-85	postive
PIK3CB	AF178030.1	0.572485425	6.57E-48	postive
RASGRP3	AF178030.1	0.551146899	7.76E-44	postive
PLXNC1	AF178030.1	0.526884444	1.51E-39	postive
GNRH1	AF178030.1	0.619342046	5.58E-58	postive
IL6ST	AF178030.1	0.545940115	6.92E-43	postive
IL7	AF178030.1	0.52180218	1.08E-38	postive
ACVR2A	AF178030.1	0.600785827	8.60E-54	postive
ANGPTL1	AF178030.1	0.774589972	3.99E-108	postive
BMPR2	AF178030.1	0.667134913	3.57E-70	postive
CRLF3	AF178030.1	0.609638544	9.35E-56	postive
NR2C2	AF178030.1	0.638207753	1.55E-62	postive
RORA	AF178030.1	0.726069781	9.63E-89	postive
SOS1	AF178030.1	0.678228232	2.46E-73	postive
SOS2	AF178030.1	0.587565335	5.69E-51	postive
BRAF	AF178030.1	0.729300093	6.69E-90	postive
CBL	AF178030.1	0.574904692	2.17E-48	postive
CBLB	AF178030.1	0.69250375	1.29E-77	postive
RASGRP1	AF178030.1	0.508645219	1.51E-36	postive
PDK1	AF178030.1	0.585372207	1.62E-50	postive
TRAJ1	AF178030.1	0.753772803	2.95E-99	postive
TRAJ2	AF178030.1	0.594321856	2.14E-52	postive
TRAJ3	AF178030.1	0.723250446	9.58E-88	postive
TRAJ5	AF178030.1	0.868127224	2.82E-164	postive
TRAJ6	AF178030.1	0.769496878	7.19E-106	postive
TRAJ8	AF178030.1	0.772046988	5.43E-107	postive
TRAJ10	AF178030.1	0.781560519	2.60E-111	postive
TRAJ12	AF178030.1	0.735137579	4.88E-92	postive
TRAJ13	AF178030.1	0.802005361	2.24E-121	postive
TRAJ14	AF178030.1	0.792655604	1.24E-116	postive
TRAJ16	AF178030.1	0.804147204	1.69E-122	postive
TRAJ17	AF178030.1	0.822300629	1.32E-132	postive
TRAJ18	AF178030.1	0.715896093	3.36E-85	postive
TRAJ21	AF178030.1	0.822856215	6.19E-133	postive
TRAJ31	AF178030.1	0.847859275	5.56E-149	postive
TRAJ37	AF178030.1	0.750128392	8.56E-98	postive
TRAJ38	AF178030.1	0.839515363	2.54E-143	postive
TRAJ39	AF178030.1	0.827940572	5.51E-136	postive
NR2C2	AC006547.1	0.518843718	3.34E-38	postive
PROC	AC026689.1	0.51571832	1.09E-37	postive
TAC1	AC026689.1	0.528303942	8.64E-40	postive

CREB1	AC093110.1	0.54161926	4.13E-42	postive
DDX17	AC093110.1	0.604858976	1.09E-54	postive
AGER	AC093110.1	0.504169094	7.75E-36	postive
TXK	AC093110.1	0.552823907	3.81E-44	postive
NFAT5	AC093110.1	0.560239369	1.55E-45	postive
PIK3R1	AC093110.1	0.546611627	5.23E-43	postive
EDNRB	AC093110.1	0.542464654	2.91E-42	postive
GNRH1	AC093110.1	0.63977987	6.26E-63	postive
ANGPTL1	AC093110.1	0.569116643	3.02E-47	postive
BMPR2	AC093110.1	0.593464578	3.25E-52	postive
CALCRL	AC093110.1	0.514574384	1.67E-37	postive
NR2C1	AC093110.1	0.522676056	7.70E-39	postive
NR2C2	AC093110.1	0.58049773	1.62E-49	postive
RORA	AC093110.1	0.631009915	9.25E-61	postive
SOS1	AC093110.1	0.505781302	4.32E-36	postive
BRAF	AC093110.1	0.558370535	3.50E-45	postive
CBLB	AC093110.1	0.57626378	1.16E-48	postive
TRAJ38	AC093110.1	0.526757355	1.58E-39	postive
LTB4R2	AC137932.3	0.51159448	5.09E-37	postive
GNRH1	AC137932.3	0.559907348	1.79E-45	postive
NR2C2	AC137932.3	0.562107755	6.84E-46	postive
BRAF	AC137932.3	0.532763317	1.48E-40	postive
NFAT5	AC004982.1	0.619957462	4.01E-58	postive
NFATC3	AC004982.1	0.507387197	2.40E-36	postive
NR2C2	AC004982.1	0.511959709	4.44E-37	postive
RORA	AC004982.1	0.558865545	2.82E-45	postive
BRAF	AC004982.1	0.50580563	4.28E-36	postive
TRAJ2	AC004982.1	0.565296935	1.67E-46	postive
TRAJ3	AC004982.1	0.535689905	4.59E-41	postive
TRAJ6	AC004982.1	0.541703172	3.99E-42	postive
TRAJ10	AC004982.1	0.519029655	3.11E-38	postive
TRAJ12	AC004982.1	0.532658169	1.54E-40	postive
TRAJ16	AC004982.1	0.544219703	1.41E-42	postive
TRAJ18	AC004982.1	0.544498964	1.26E-42	postive
TRAJ31	AC004982.1	0.506685358	3.10E-36	postive
TRAJ37	AC004982.1	0.563539387	3.64E-46	postive
TRAJ38	AC004982.1	0.552312026	4.73E-44	postive
TRAJ39	AC004982.1	0.503475523	9.96E-36	postive
FABP7	AC007996.1	0.514182352	1.94E-37	postive
GNRH1	AL450384.2	0.516632736	7.72E-38	postive
NR2C1	AL450384.2	0.504031234	8.15E-36	postive
NR2C2	AL450384.2	0.51390234	2.15E-37	postive
CREB1	SCAT2	0.642561721	1.24E-63	postive
RFXAP	SCAT2	0.515656581	1.12E-37	postive
ZC3HAV1L	SCAT2	0.516267391	8.86E-38	postive
IL15	SCAT2	0.581411948	1.06E-49	postive
MAPK8	SCAT2	0.554242851	2.08E-44	postive
EED	SCAT2	0.541999808	3.53E-42	postive
IREB2	SCAT2	0.526173722	1.99E-39	postive
DDX17	SCAT2	0.571229001	1.16E-47	postive
JAK2	SCAT2	0.509572347	1.08E-36	postive
NFAT5	SCAT2	0.705892013	7.26E-82	postive
PIK3CA	SCAT2	0.576438298	1.07E-48	postive
AKT3	SCAT2	0.526216443	1.95E-39	postive
GNRH1	SCAT2	0.725887709	1.12E-88	postive
ACVR2A	SCAT2	0.537646201	2.08E-41	postive
ANGPTL1	SCAT2	0.665712659	8.88E-70	postive
BMPR2	SCAT2	0.534372711	7.79E-41	postive
CRLF3	SCAT2	0.592927081	4.23E-52	postive
NR2C1	SCAT2	0.586134485	1.13E-50	postive
NR2C2	SCAT2	0.648922541	2.88E-65	postive
RORA	SCAT2	0.658846231	6.73E-68	postive
SOS1	SCAT2	0.624707324	3.04E-59	postive
BRAF	SCAT2	0.715526863	4.49E-85	postive
CBL	SCAT2	0.542086529	3.41E-42	postive

CBLB	SCAT2	0.622925652	8.04E-59	postive
PDK1	SCAT2	0.580419423	1.68E-49	postive
TRAJ1	SCAT2	0.695619653	1.39E-78	postive
TRAJ2	SCAT2	0.672212566	1.33E-71	postive
TRAJ3	SCAT2	0.740576754	4.43E-94	postive
TRAJ5	SCAT2	0.711666306	8.99E-84	postive
TRAJ6	SCAT2	0.73761252	5.83E-93	postive
TRAJ8	SCAT2	0.737919398	4.47E-93	postive
TRAJ10	SCAT2	0.714492336	1.01E-84	postive
TRAJ12	SCAT2	0.659459602	4.59E-68	postive
TRAJ13	SCAT2	0.73375756	1.58E-91	postive
TRAJ14	SCAT2	0.73227361	5.55E-91	postive
TRAJ16	SCAT2	0.733005003	2.99E-91	postive
TRAJ17	SCAT2	0.719733169	1.62E-86	postive
TRAJ18	SCAT2	0.64259968	1.21E-63	postive
TRAJ21	SCAT2	0.747745948	7.49E-97	postive
TRAJ31	SCAT2	0.748474456	3.87E-97	postive
TRAJ37	SCAT2	0.724185043	4.49E-88	postive
TRAJ38	SCAT2	0.773842141	8.62E-108	postive
TRAJ39	SCAT2	0.748756319	2.99E-97	postive
PSMD3	IDH1-AS1	0.679629425	9.57E-74	postive
CREB1	OSMR-AS1	0.643827353	5.91E-64	postive
UBR1	OSMR-AS1	0.525616047	2.47E-39	postive
IL15	OSMR-AS1	0.608996673	1.30E-55	postive
CYLD	OSMR-AS1	0.502107305	1.63E-35	postive
JAK2	OSMR-AS1	0.542393995	3.00E-42	postive
NFAT5	OSMR-AS1	0.681759146	2.26E-74	postive
PIK3R1	OSMR-AS1	0.508070748	1.87E-36	postive
PIK3CA	OSMR-AS1	0.621486455	1.75E-58	postive
PIK3CB	OSMR-AS1	0.505359548	5.03E-36	postive
GNRH1	OSMR-AS1	0.593656034	2.96E-52	postive
ANGPTL1	OSMR-AS1	0.67279292	9.06E-72	postive
BMPR2	OSMR-AS1	0.594440346	2.02E-52	postive
CRLF3	OSMR-AS1	0.520683187	1.66E-38	postive
NR2C2	OSMR-AS1	0.592419816	5.42E-52	postive
RORA	OSMR-AS1	0.663805961	2.99E-69	postive
SOS1	OSMR-AS1	0.557738559	4.61E-45	postive
BRAF	OSMR-AS1	0.661834731	1.04E-68	postive
CBL	OSMR-AS1	0.524483661	3.83E-39	postive
CBLB	OSMR-AS1	0.617451488	1.53E-57	postive
PDK1	OSMR-AS1	0.522945091	6.94E-39	postive
TRAJ1	OSMR-AS1	0.652028469	4.43E-66	postive
TRAJ2	OSMR-AS1	0.592294087	5.77E-52	postive
TRAJ3	OSMR-AS1	0.729840181	4.27E-90	postive
TRAJ5	OSMR-AS1	0.697682714	3.12E-79	postive
TRAJ6	OSMR-AS1	0.711540066	9.90E-84	postive
TRAJ8	OSMR-AS1	0.669578939	7.38E-71	postive
TRAJ10	OSMR-AS1	0.724930301	2.45E-88	postive
TRAJ12	OSMR-AS1	0.68072127	4.57E-74	postive
TRAJ13	OSMR-AS1	0.676097271	1.02E-72	postive
TRAJ14	OSMR-AS1	0.699570258	7.88E-80	postive
TRAJ16	OSMR-AS1	0.722932855	1.24E-87	postive
TRAJ17	OSMR-AS1	0.734642661	7.45E-92	postive
TRAJ18	OSMR-AS1	0.717652607	8.43E-86	postive
TRAJ21	OSMR-AS1	0.737585811	5.97E-93	postive
TRAJ31	OSMR-AS1	0.728534031	1.26E-89	postive
TRAJ37	OSMR-AS1	0.688360042	2.39E-76	postive
TRAJ38	OSMR-AS1	0.757946208	5.82E-101	postive
TRAJ39	OSMR-AS1	0.741795167	1.52E-94	postive
CREB1	AP001381.1	0.800213443	1.90E-120	postive
UBR1	AP001381.1	0.64476291	3.41E-64	postive
ZC3HAV1	AP001381.1	0.568293907	4.38E-47	postive
IL15	AP001381.1	0.585509353	1.52E-50	postive
CYLD	AP001381.1	0.605435855	8.14E-55	postive
EIF2AK2	AP001381.1	0.571645016	9.63E-48	postive

MAPK8	AP001381.1	0.563771069	3.29E-46	postive
TLR1	AP001381.1	0.519350915	2.75E-38	postive
LMBR1	AP001381.1	0.588499655	3.63E-51	postive
LIMS1	AP001381.1	0.544921751	1.06E-42	postive
IREB2	AP001381.1	0.615928598	3.45E-57	postive
DDX17	AP001381.1	0.57958251	2.49E-49	postive
PIK3CG	AP001381.1	0.546200859	6.20E-43	postive
JAK2	AP001381.1	0.668101272	1.92E-70	postive
TXK	AP001381.1	0.596698299	6.61E-53	postive
NFAT5	AP001381.1	0.824164876	1.04E-133	postive
MALT1	AP001381.1	0.547000498	4.44E-43	postive
PIK3R1	AP001381.1	0.634466912	1.32E-61	postive
PIK3CA	AP001381.1	0.73479216	6.56E-92	postive
PIK3CB	AP001381.1	0.551268797	7.37E-44	postive
AKT3	AP001381.1	0.528785047	7.15E-40	postive
RASGRP3	AP001381.1	0.551437214	6.86E-44	postive
PLXNC1	AP001381.1	0.523380823	5.87E-39	postive
GNRH1	AP001381.1	0.725616889	1.40E-88	postive
IL6ST	AP001381.1	0.624737169	2.99E-59	postive
IL7	AP001381.1	0.51172978	4.84E-37	postive
RABEP1	AP001381.1	0.548177411	2.71E-43	postive
ACVR2A	AP001381.1	0.655915223	4.13E-67	postive
ANGPTL1	AP001381.1	0.789667013	3.62E-115	postive
BMPR1A	AP001381.1	0.55881939	2.88E-45	postive
BMPR2	AP001381.1	0.757303751	1.07E-100	postive
CRLF3	AP001381.1	0.661127244	1.62E-68	postive
NR1D2	AP001381.1	0.517554313	5.45E-38	postive
NR2C2	AP001381.1	0.68908227	1.44E-76	postive
RORA	AP001381.1	0.827450606	1.10E-135	postive
SOS1	AP001381.1	0.701806174	1.52E-80	postive
SOS2	AP001381.1	0.589096649	2.72E-51	postive
BRAF	AP001381.1	0.79436744	1.75E-117	postive
CBL	AP001381.1	0.583068454	4.84E-50	postive
CBLB	AP001381.1	0.752603181	8.76E-99	postive
RASGRP1	AP001381.1	0.507533498	2.28E-36	postive
PDK1	AP001381.1	0.595406846	1.25E-52	postive
TRAJ1	AP001381.1	0.745365862	6.38E-96	postive
TRAJ2	AP001381.1	0.725155645	2.04E-88	postive
TRAJ3	AP001381.1	0.776334784	6.52E-109	postive
TRAJ5	AP001381.1	0.80849638	8.05E-125	postive
TRAJ6	AP001381.1	0.801287697	5.30E-121	postive
TRAJ8	AP001381.1	0.783851095	2.20E-112	postive
TRAJ10	AP001381.1	0.782327004	1.14E-111	postive
TRAJ12	AP001381.1	0.72986381	4.18E-90	postive
TRAJ13	AP001381.1	0.793769418	3.48E-117	postive
TRAJ14	AP001381.1	0.783592484	2.91E-112	postive
TRAJ16	AP001381.1	0.826705008	3.10E-135	postive
TRAJ17	AP001381.1	0.778137819	9.86E-110	postive
TRAJ18	AP001381.1	0.735433093	3.79E-92	postive
TRAJ21	AP001381.1	0.819687264	4.41E-131	postive
TRAJ31	AP001381.1	0.851910899	7.46E-152	postive
TRAJ37	AP001381.1	0.793949244	2.84E-117	postive
TRAJ38	AP001381.1	0.882645962	6.91E-177	postive
TRAJ39	AP001381.1	0.837261535	7.57E-142	postive
DDX17	AC120053.1	0.577329049	7.10E-49	postive
NFAT5	AC120053.1	0.5416483	4.08E-42	postive
GNRH1	AC120053.1	0.649709069	1.79E-65	postive
NR2C2	AC120053.1	0.636732945	3.62E-62	postive
BRAF	AC120053.1	0.634611645	1.21E-61	postive
TRAJ1	AC120053.1	0.53993415	8.22E-42	postive
TRAJ2	AC120053.1	0.541944711	3.61E-42	postive
TRAJ3	AC120053.1	0.584138941	2.92E-50	postive
TRAJ5	AC120053.1	0.552507817	4.36E-44	postive
TRAJ6	AC120053.1	0.548760575	2.13E-43	postive
TRAJ8	AC120053.1	0.605467787	8.01E-55	postive

TRAJ10	AC120053.1	0.52165741	1.14E-38	postive
TRAJ13	AC120053.1	0.581157197	1.19E-49	postive
TRAJ14	AC120053.1	0.575076898	2.01E-48	postive
TRAJ16	AC120053.1	0.540690333	6.04E-42	postive
TRAJ17	AC120053.1	0.542678545	2.67E-42	postive
TRAJ21	AC120053.1	0.570890421	1.36E-47	postive
TRAJ31	AC120053.1	0.570283293	1.79E-47	postive
TRAJ37	AC120053.1	0.554801533	1.63E-44	postive
TRAJ38	AC120053.1	0.574231024	2.96E-48	postive
TRAJ39	AC120053.1	0.577138979	7.75E-49	postive
GNRH1	AC010761.1	0.532232163	1.83E-40	postive
B2M	HCP5	0.641838918	1.89E-63	postive
HLA-A	HCP5	0.736467465	1.56E-92	postive
HLA-B	HCP5	0.781773822	2.07E-111	postive
HLA-C	HCP5	0.706840412	3.56E-82	postive
HLA-DMB	HCP5	0.530511967	3.62E-40	postive
HLA-DRA	HCP5	0.518684997	3.55E-38	postive
HLA-E	HCP5	0.625339127	2.15E-59	postive
HLA-F	HCP5	0.751570632	2.27E-98	postive
HLA-H	HCP5	0.602425678	3.76E-54	postive
PSMB8	HCP5	0.728602703	1.19E-89	postive
PSME1	HCP5	0.506147137	3.78E-36	postive
PSME2	HCP5	0.517594964	5.36E-38	postive
TAP1	HCP5	0.714484974	1.01E-84	postive
TAP2	HCP5	0.534621235	7.05E-41	postive
CXCL10	HCP5	0.578968044	3.32E-49	postive
CXCL9	HCP5	0.547324866	3.88E-43	postive
APOBEC3G	HCP5	0.61055205	5.82E-56	postive
STAT1	HCP5	0.56283535	4.97E-46	postive
IRF1	HCP5	0.606318108	5.18E-55	postive
CD40	HCP5	0.506044326	3.92E-36	postive
FASLG	HCP5	0.512064975	4.27E-37	postive
GBP2	HCP5	0.523828659	4.94E-39	postive
TNFSF13B	HCP5	0.501166116	2.29E-35	postive
IL12RB1	HCP5	0.508508949	1.59E-36	postive
CREB1	AC022211.2	0.676697932	6.84E-73	postive
UBR1	AC022211.2	0.530807297	3.22E-40	postive
ZC3HAV1	AC022211.2	0.539978319	8.07E-42	postive
ZC3HAV1L	AC022211.2	0.518024029	4.56E-38	postive
EIF2AK2	AC022211.2	0.545408133	8.63E-43	postive
MAPK8	AC022211.2	0.521280914	1.32E-38	postive
LMBR1	AC022211.2	0.508095588	1.85E-36	postive
IREB2	AC022211.2	0.503824897	8.78E-36	postive
DDX17	AC022211.2	0.568137782	4.69E-47	postive
JAK2	AC022211.2	0.525821813	2.28E-39	postive
NFAT5	AC022211.2	0.618763839	7.61E-58	postive
PIK3CA	AC022211.2	0.602022664	4.61E-54	postive
PIK3CB	AC022211.2	0.52614969	2.01E-39	postive
GNRH1	AC022211.2	0.68571124	1.50E-75	postive
ACVR2A	AC022211.2	0.505929463	4.09E-36	postive
ANGPTL1	AC022211.2	0.66202598	9.20E-69	postive
BMPR2	AC022211.2	0.593550539	3.12E-52	postive
CRLF3	AC022211.2	0.570808241	1.41E-47	postive
NR2C2	AC022211.2	0.696088892	9.88E-79	postive
RORA	AC022211.2	0.595908846	9.78E-53	postive
SOS1	AC022211.2	0.663491505	3.65E-69	postive
SOS2	AC022211.2	0.517523024	5.51E-38	postive
BRAF	AC022211.2	0.716023783	3.04E-85	postive
CBL	AC022211.2	0.575901806	1.37E-48	postive
CBLB	AC022211.2	0.636036217	5.39E-62	postive
PDK1	AC022211.2	0.504486233	6.91E-36	postive
TRAJ1	AC022211.2	0.607061922	3.54E-55	postive
TRAJ2	AC022211.2	0.520179647	2.01E-38	postive
TRAJ3	AC022211.2	0.609471077	1.02E-55	postive
TRAJ5	AC022211.2	0.669421198	8.17E-71	postive

TRAJ6	AC022211.2	0.627691435	5.87E-60	postive
TRAJ8	AC022211.2	0.618307714	9.71E-58	postive
TRAJ10	AC022211.2	0.598882642	2.23E-53	postive
TRAJ12	AC022211.2	0.577128858	7.79E-49	postive
TRAJ13	AC022211.2	0.641526128	2.27E-63	postive
TRAJ14	AC022211.2	0.631874683	5.69E-61	postive
TRAJ16	AC022211.2	0.633592582	2.16E-61	postive
TRAJ17	AC022211.2	0.623418917	6.14E-59	postive
TRAJ18	AC022211.2	0.549642728	1.47E-43	postive
TRAJ21	AC022211.2	0.657846364	1.25E-67	postive
TRAJ31	AC022211.2	0.655839886	4.32E-67	postive
TRAJ37	AC022211.2	0.579736638	2.32E-49	postive
TRAJ38	AC022211.2	0.674855901	2.32E-72	postive
TRAJ39	AC022211.2	0.644428356	4.15E-64	postive
CREB1	AC006017.1	0.751363889	2.75E-98	postive
RFXAP	AC006017.1	0.528165563	9.12E-40	postive
UBR1	AC006017.1	0.605700912	7.11E-55	postive
ZC3HAV1	AC006017.1	0.579678399	2.38E-49	postive
ZC3HAV1L	AC006017.1	0.555239613	1.35E-44	postive
IL15	AC006017.1	0.5706173	1.54E-47	postive
CYLD	AC006017.1	0.565226221	1.72E-46	postive
EIF2AK2	AC006017.1	0.521318541	1.30E-38	postive
MAPK8	AC006017.1	0.548311858	2.57E-43	postive
NFKBIZ	AC006017.1	0.505198881	5.33E-36	postive
LMBR1	AC006017.1	0.585340378	1.65E-50	postive
IREB2	AC006017.1	0.566129355	1.15E-46	postive
DDX17	AC006017.1	0.644925288	3.10E-64	postive
JAK2	AC006017.1	0.598837142	2.28E-53	postive
TXK	AC006017.1	0.534094718	8.70E-41	postive
NFAT5	AC006017.1	0.801946648	2.41E-121	postive
MALT1	AC006017.1	0.51124855	5.79E-37	postive
PIK3R1	AC006017.1	0.583154344	4.65E-50	postive
PIK3CA	AC006017.1	0.625087164	2.47E-59	postive
PIK3CB	AC006017.1	0.506703693	3.08E-36	postive
AKT3	AC006017.1	0.514885746	1.49E-37	postive
RASGRP3	AC006017.1	0.518902527	3.27E-38	postive
LTB4R2	AC006017.1	0.519010706	3.13E-38	postive
GNRH1	AC006017.1	0.756218437	2.99E-100	postive
IL6ST	AC006017.1	0.576812619	9.02E-49	postive
RABEP1	AC006017.1	0.543645866	1.79E-42	postive
ACVR2A	AC006017.1	0.617033055	1.92E-57	postive
ANGPTL1	AC006017.1	0.727738248	2.44E-89	postive
BMPR1A	AC006017.1	0.512941753	3.08E-37	postive
BMPR2	AC006017.1	0.664835975	1.55E-69	postive
CRLF3	AC006017.1	0.640732037	3.61E-63	postive
LIFR	AC006017.1	0.508901352	1.38E-36	postive
NR2C1	AC006017.1	0.514305395	1.85E-37	postive
NR2C2	AC006017.1	0.742575992	7.63E-95	postive
RORA	AC006017.1	0.772530933	3.31E-107	postive
SOS1	AC006017.1	0.68675617	7.29E-76	postive
SOS2	AC006017.1	0.58805093	4.50E-51	postive
BRAF	AC006017.1	0.862570206	7.69E-160	postive
CBL	AC006017.1	0.599621589	1.54E-53	postive
CBLB	AC006017.1	0.694289763	3.60E-78	postive
RASGRP1	AC006017.1	0.511812802	4.69E-37	postive
PDK1	AC006017.1	0.556258857	8.73E-45	postive
TRAJ1	AC006017.1	0.765539266	3.72E-104	postive
TRAJ2	AC006017.1	0.744639338	1.22E-95	postive
TRAJ3	AC006017.1	0.811881437	1.14E-126	postive
TRAJ5	AC006017.1	0.800658798	1.12E-120	postive
TRAJ6	AC006017.1	0.804365105	1.30E-122	postive
TRAJ8	AC006017.1	0.812221952	7.38E-127	postive
TRAJ10	AC006017.1	0.7710779	1.45E-106	postive
TRAJ12	AC006017.1	0.73299481	3.02E-91	postive
TRAJ13	AC006017.1	0.808334027	9.85E-125	postive

TRAJ14	AC006017.1	0.806781007	6.74E-124	postive
TRAJ16	AC006017.1	0.796530374	1.44E-118	postive
TRAJ17	AC006017.1	0.771042282	1.51E-106	postive
TRAJ18	AC006017.1	0.708269309	1.21E-82	postive
TRAJ21	AC006017.1	0.821335874	4.85E-132	postive
TRAJ31	AC006017.1	0.831355103	4.32E-138	postive
TRAJ37	AC006017.1	0.803859546	2.40E-122	postive
TRAJ38	AC006017.1	0.857525448	5.58E-156	postive
TRAJ39	AC006017.1	0.829297784	8.13E-137	postive
DDX17	AC024075.2	0.53352182	1.09E-40	postive
GNRH1	AC024075.2	0.537352216	2.35E-41	postive
IL11RA	AC024075.2	0.546885153	4.66E-43	postive
NR2C2	AC024075.2	0.501590684	1.96E-35	postive
CCL13	LINC02345	0.510618714	7.31E-37	postive
MSR1	LINC02345	0.519031156	3.11E-38	postive
CREB1	LINC01534	0.626618825	1.06E-59	postive
RFXAP	LINC01534	0.574500702	2.62E-48	postive
UBR1	LINC01534	0.559298877	2.34E-45	postive
ZC3HAV1L	LINC01534	0.521605172	1.16E-38	postive
DDX17	LINC01534	0.603594965	2.08E-54	postive
TXK	LINC01534	0.506518812	3.30E-36	postive
NFAT5	LINC01534	0.686584938	8.21E-76	postive
PIK3R1	LINC01534	0.548180128	2.71E-43	postive
PIK3CA	LINC01534	0.522527379	8.16E-39	postive
GNRH1	LINC01534	0.723052577	1.12E-87	postive
IL6ST	LINC01534	0.526177549	1.98E-39	postive
RABEP1	LINC01534	0.505545772	4.70E-36	postive
ACVR2A	LINC01534	0.549882972	1.32E-43	postive
ANGPTL1	LINC01534	0.572107512	7.81E-48	postive
BMPR2	LINC01534	0.5607765	1.23E-45	postive
CRLF3	LINC01534	0.557268324	5.65E-45	postive
NR2C1	LINC01534	0.502160805	1.60E-35	postive
NR2C2	LINC01534	0.694472621	3.16E-78	postive
RORA	LINC01534	0.69359332	5.92E-78	postive
SOS1	LINC01534	0.561628515	8.45E-46	postive
SOS2	LINC01534	0.51055298	7.49E-37	postive
BRAF	LINC01534	0.7028076	7.26E-81	postive
CBLB	LINC01534	0.548387174	2.49E-43	postive
PDK1	LINC01534	0.541968153	3.57E-42	postive
TRAJ1	LINC01534	0.571879145	8.66E-48	postive
TRAJ2	LINC01534	0.560599446	1.33E-45	postive
TRAJ3	LINC01534	0.610766408	5.20E-56	postive
TRAJ5	LINC01534	0.620572067	2.88E-58	postive
TRAJ6	LINC01534	0.604061729	1.64E-54	postive
TRAJ8	LINC01534	0.581793226	8.83E-50	postive
TRAJ10	LINC01534	0.5897072	2.03E-51	postive
TRAJ12	LINC01534	0.529639495	5.11E-40	postive
TRAJ13	LINC01534	0.596267949	8.18E-53	postive
TRAJ14	LINC01534	0.566379453	1.03E-46	postive
TRAJ16	LINC01534	0.575312083	1.80E-48	postive
TRAJ17	LINC01534	0.558982713	2.68E-45	postive
TRAJ18	LINC01534	0.540413635	6.76E-42	postive
TRAJ21	LINC01534	0.617362956	1.61E-57	postive
TRAJ31	LINC01534	0.633264761	2.60E-61	postive
TRAJ37	LINC01534	0.630374693	1.32E-60	postive
TRAJ38	LINC01534	0.641334986	2.54E-63	postive
TRAJ39	LINC01534	0.644817248	3.30E-64	postive
DDX17	AC093726.2	0.583153679	4.65E-50	postive
NFAT5	AC093726.2	0.584444325	2.52E-50	postive
LTB4R2	AC093726.2	0.502029404	1.68E-35	postive
GNRH1	AC093726.2	0.65127163	7.00E-66	postive
NR2C2	AC093726.2	0.598458331	2.76E-53	postive
RORA	AC093726.2	0.546921109	4.59E-43	postive
BRAF	AC093726.2	0.559000027	2.66E-45	postive
TRAJ1	AC093726.2	0.511328504	5.62E-37	postive

TRAJ2	AC093726.2	0.516267953	8.86E-38	postive
TRAJ3	AC093726.2	0.552374154	4.61E-44	postive
TRAJ5	AC093726.2	0.504677452	6.45E-36	postive
TRAJ6	AC093726.2	0.520194192	2.00E-38	postive
TRAJ10	AC093726.2	0.519707755	2.40E-38	postive
TRAJ16	AC093726.2	0.512698441	3.38E-37	postive
TRAJ21	AC093726.2	0.501526616	2.01E-35	postive
TRAJ31	AC093726.2	0.53184323	2.14E-40	postive
TRAJ37	AC093726.2	0.530519194	3.61E-40	postive
TRAJ38	AC093726.2	0.54976677	1.39E-43	postive
TRAJ39	AC093726.2	0.516548775	7.97E-38	postive
CD8A	LINC02446	0.718452921	4.47E-86	postive
CD8B	LINC02446	0.610603346	5.67E-56	postive
IFNG	LINC02446	0.752839546	7.03E-99	postive
TAP1	LINC02446	0.534562612	7.22E-41	postive
TAP2	LINC02446	0.545814554	7.29E-43	postive
CXCL9	LINC02446	0.602820451	3.08E-54	postive
STAT1	LINC02446	0.568214079	4.54E-47	postive
IRF1	LINC02446	0.566452634	9.98E-47	postive
CCL5	LINC02446	0.743290649	4.05E-95	postive
CCL4	LINC02446	0.666897917	4.16E-70	postive
PDCD1	LINC02446	0.513440379	2.56E-37	postive
FASLG	LINC02446	0.717607175	8.74E-86	postive
GBP2	LINC02446	0.556182937	9.02E-45	postive
CCR5	LINC02446	0.535456494	5.04E-41	postive
CCL4L2	LINC02446	0.510990861	6.37E-37	postive
XCL2	LINC02446	0.521292188	1.31E-38	postive
CXCR6	LINC02446	0.691221174	3.19E-77	postive
CD72	LINC02446	0.520383866	1.86E-38	postive
IL12RB1	LINC02446	0.518285021	4.13E-38	postive
IL15RA	LINC02446	0.51010667	8.83E-37	postive
IL2RB	LINC02446	0.582651838	5.89E-50	postive
IL18RAP	LINC02446	0.558179137	3.81E-45	postive
CD247	LINC02446	0.608464692	1.72E-55	postive
CD244	LINC02446	0.663048588	4.83E-69	postive
SH2D1A	LINC02446	0.563034643	4.55E-46	postive
GZMB	LINC02446	0.766600661	1.30E-104	postive
PRF1	LINC02446	0.624625614	3.18E-59	postive
CD3D	LINC02446	0.544230596	1.41E-42	postive
CD3E	LINC02446	0.553518586	2.83E-44	postive
CD3G	LINC02446	0.539940269	8.20E-42	postive
TRAC	LINC02446	0.519499593	2.60E-38	postive
TRAV29DV5	LINC02446	0.512178115	4.10E-37	postive
TRBC1	LINC02446	0.521068839	1.43E-38	postive
TRBC2	LINC02446	0.552594227	4.20E-44	postive
TRBJ2-1	LINC02446	0.521481203	1.22E-38	postive
TRBV28	LINC02446	0.51488175	1.49E-37	postive
TRDC	LINC02446	0.515042311	1.40E-37	postive
TRDV1	LINC02446	0.651438172	6.33E-66	postive
CREB1	AC096992.2	0.651863271	4.89E-66	postive
RFXAP	AC096992.2	0.532413445	1.70E-40	postive
ZC3HAV1	AC096992.2	0.565721752	1.38E-46	postive
CYLD	AC096992.2	0.501075886	2.36E-35	postive
EIF2AK2	AC096992.2	0.545389511	8.70E-43	postive
IREB2	AC096992.2	0.512500941	3.63E-37	postive
DDX17	AC096992.2	0.628025802	4.88E-60	postive
JAK2	AC096992.2	0.512996286	3.02E-37	postive
TXK	AC096992.2	0.512225817	4.03E-37	postive
NFAT5	AC096992.2	0.659646815	4.09E-68	postive
PIK3R1	AC096992.2	0.530969973	3.02E-40	postive
PIK3CA	AC096992.2	0.586668245	8.74E-51	postive
PIK3CB	AC096992.2	0.526896728	1.50E-39	postive
LTB4R2	AC096992.2	0.523818347	4.96E-39	postive
GNRH1	AC096992.2	0.71021886	2.73E-83	postive
ACVR2A	AC096992.2	0.549106747	1.84E-43	postive

ANGPTL1	AC096992.2	0.652769432	2.82E-66	postive
BMPR2	AC096992.2	0.577823598	5.65E-49	postive
CRLF3	AC096992.2	0.574401897	2.74E-48	postive
NR2C2	AC096992.2	0.726372947	7.51E-89	postive
RORA	AC096992.2	0.651801252	5.08E-66	postive
SOS1	AC096992.2	0.684788719	2.84E-75	postive
SOS2	AC096992.2	0.532800854	1.46E-40	postive
BRAF	AC096992.2	0.759758916	1.03E-101	postive
CBL	AC096992.2	0.587905753	4.83E-51	postive
CBLB	AC096992.2	0.66363659	3.33E-69	postive
PDK1	AC096992.2	0.500014748	3.46E-35	postive
TRAJ1	AC096992.2	0.670853191	3.22E-71	postive
TRAJ2	AC096992.2	0.602391593	3.83E-54	postive
TRAJ3	AC096992.2	0.693412036	6.74E-78	postive
TRAJ5	AC096992.2	0.716730783	1.74E-85	postive
TRAJ6	AC096992.2	0.666357464	5.88E-70	postive
TRAJ8	AC096992.2	0.689950271	7.83E-77	postive
TRAJ10	AC096992.2	0.620871169	2.45E-58	postive
TRAJ12	AC096992.2	0.60575458	6.92E-55	postive
TRAJ13	AC096992.2	0.678111428	2.66E-73	postive
TRAJ14	AC096992.2	0.679886356	8.05E-74	postive
TRAJ16	AC096992.2	0.666969402	3.97E-70	postive
TRAJ17	AC096992.2	0.654586401	9.32E-67	postive
TRAJ18	AC096992.2	0.581543836	9.93E-50	postive
TRAJ21	AC096992.2	0.693947639	4.60E-78	postive
TRAJ31	AC096992.2	0.685131531	2.24E-75	postive
TRAJ37	AC096992.2	0.651409126	6.44E-66	postive
TRAJ38	AC096992.2	0.696418389	7.79E-79	postive
TRAJ39	AC096992.2	0.722742889	1.44E-87	postive
CREB1	AL592148.3	0.756990366	1.44E-100	postive
UBR1	AL592148.3	0.63468184	1.17E-61	postive
ZC3HAV1	AL592148.3	0.556132196	9.22E-45	postive
CYLD	AL592148.3	0.618255095	9.99E-58	postive
EIF2AK2	AL592148.3	0.515321327	1.26E-37	postive
MAPK8	AL592148.3	0.534642309	6.99E-41	postive
LMBR1	AL592148.3	0.503408913	1.02E-35	postive
IREB2	AL592148.3	0.573353295	4.42E-48	postive
DDX17	AL592148.3	0.634824425	1.07E-61	postive
JAK2	AL592148.3	0.602477002	3.67E-54	postive
TXK	AL592148.3	0.574697195	2.39E-48	postive
NFAT5	AL592148.3	0.827414176	1.15E-135	postive
NFATC3	AL592148.3	0.509295842	1.19E-36	postive
MALT1	AL592148.3	0.509253418	1.21E-36	postive
PIK3R1	AL592148.3	0.592710728	4.71E-52	postive
PIK3CA	AL592148.3	0.641661519	2.10E-63	postive
AKT3	AL592148.3	0.51606064	9.58E-38	postive
ROBO2	AL592148.3	0.595604338	1.14E-52	postive
GNRH1	AL592148.3	0.774276718	5.51E-108	postive
IL6ST	AL592148.3	0.582386824	6.68E-50	postive
RABEP1	AL592148.3	0.57103734	1.27E-47	postive
ACVR2A	AL592148.3	0.647551791	6.52E-65	postive
ANGPTL1	AL592148.3	0.687578969	4.11E-76	postive
BMPR1A	AL592148.3	0.556724548	7.14E-45	postive
BMPR2	AL592148.3	0.73491447	5.91E-92	postive
CRLF3	AL592148.3	0.574653092	2.44E-48	postive
NR1D2	AL592148.3	0.566277479	1.08E-46	postive
NR2C1	AL592148.3	0.511452639	5.37E-37	postive
NR2C2	AL592148.3	0.704422718	2.18E-81	postive
RORA	AL592148.3	0.80100477	7.42E-121	postive
SOS1	AL592148.3	0.660348754	2.64E-68	postive
SOS2	AL592148.3	0.542684433	2.66E-42	postive
BRAF	AL592148.3	0.751297118	2.93E-98	postive
CBL	AL592148.3	0.565615385	1.45E-46	postive
CBLB	AL592148.3	0.708587582	9.47E-83	postive
TRAJ1	AL592148.3	0.66984721	6.20E-71	postive

TRAJ2	AL592148.3	0.680837257	4.23E-74	postive
TRAJ3	AL592148.3	0.698896693	1.29E-79	postive
TRAJ5	AL592148.3	0.695860187	1.17E-78	postive
TRAJ6	AL592148.3	0.730629486	2.21E-90	postive
TRAJ8	AL592148.3	0.68263272	1.25E-74	postive
TRAJ10	AL592148.3	0.695859446	1.17E-78	postive
TRAJ12	AL592148.3	0.688517068	2.14E-76	postive
TRAJ13	AL592148.3	0.713224796	2.70E-84	postive
TRAJ14	AL592148.3	0.688860899	1.68E-76	postive
TRAJ16	AL592148.3	0.72242879	1.86E-87	postive
TRAJ17	AL592148.3	0.661293229	1.46E-68	postive
TRAJ18	AL592148.3	0.669635639	7.11E-71	postive
TRAJ21	AL592148.3	0.724890082	2.53E-88	postive
TRAJ31	AL592148.3	0.768654451	1.68E-105	postive
TRAJ37	AL592148.3	0.735022637	5.39E-92	postive
TRAJ38	AL592148.3	0.798958687	8.40E-120	postive
TRAJ39	AL592148.3	0.716967226	1.45E-85	postive
IGKV1D-42	AL049870.3	0.795111759	7.45E-118	postive
LBP	AC067930.3	0.549043342	1.89E-43	postive
RBP4	AC067930.3	0.515361035	1.25E-37	postive
C8G	AC067930.3	0.655833906	4.34E-67	postive
TFR2	AC067930.3	0.617746957	1.31E-57	postive
APOM	AC067930.3	0.599513688	1.63E-53	postive
APOH	AC067930.3	0.542210176	3.24E-42	postive
VTN	AC067930.3	0.682990086	9.77E-75	postive
ALB	AC067930.3	0.684525669	3.41E-75	postive
AGTR1	AC067930.3	0.640809506	3.45E-63	postive
TRBV4-1	AC067930.3	0.624900359	2.73E-59	postive
CREB1	AC091185.1	0.577338863	7.07E-49	postive
UBR1	AC091185.1	0.593968676	2.54E-52	postive
IREB2	AC091185.1	0.527620564	1.13E-39	postive
DDX17	AC091185.1	0.652371434	3.60E-66	postive
TXK	AC091185.1	0.595298813	1.32E-52	postive
NFAT5	AC091185.1	0.630642824	1.14E-60	postive
PIK3R1	AC091185.1	0.514779254	1.55E-37	postive
IGHD4-4	AC091185.1	0.511577339	5.12E-37	postive
IGHD6-6	AC091185.1	0.563541076	3.64E-46	postive
LTB4R2	AC091185.1	0.506615217	3.18E-36	postive
GNRH1	AC091185.1	0.872288569	9.89E-168	postive
ACVR2A	AC091185.1	0.527249006	1.31E-39	postive
ANGPTL1	AC091185.1	0.532893884	1.41E-40	postive
BMPR2	AC091185.1	0.561333875	9.61E-46	postive
NR2C1	AC091185.1	0.555775053	1.08E-44	postive
NR2C2	AC091185.1	0.737305646	7.60E-93	postive
RORA	AC091185.1	0.623782661	5.04E-59	postive
SOS1	AC091185.1	0.535877696	4.26E-41	postive
BRAF	AC091185.1	0.612172337	2.50E-56	postive
TEC	AC091185.1	0.588292776	4.01E-51	postive
CBLB	AC091185.1	0.524492886	3.82E-39	postive
TRAJ1	AC091185.1	0.501411278	2.10E-35	postive
TRAJ3	AC091185.1	0.569780635	2.24E-47	postive
TRAJ6	AC091185.1	0.508987963	1.33E-36	postive
TRAJ38	AC091185.1	0.524217844	4.25E-39	postive
TRAJ39	AC091185.1	0.545766981	7.43E-43	postive
NFAT5	LINC01655	0.540773785	5.83E-42	postive
NR2C2	LINC01655	0.573204343	4.73E-48	postive
RORA	LINC01655	0.534913991	6.27E-41	postive
BRAF	LINC01655	0.533512901	1.10E-40	postive
TRAJ1	LINC01655	0.5028604	1.24E-35	postive
TRAJ5	LINC01655	0.556475465	7.95E-45	postive
TRAJ6	LINC01655	0.509459021	1.12E-36	postive
TRAJ8	LINC01655	0.508450195	1.63E-36	postive
TRAJ10	LINC01655	0.511746829	4.81E-37	postive
TRAJ12	LINC01655	0.541893539	3.69E-42	postive
TRAJ13	LINC01655	0.517295154	6.01E-38	postive

TRAJ14	LINC01655	0.520423309	1.83E-38	postive
TRAJ16	LINC01655	0.513259589	2.74E-37	postive
TRAJ17	LINC01655	0.525140495	2.97E-39	postive
TRAJ18	LINC01655	0.522302084	8.90E-39	postive
TRAJ21	LINC01655	0.538459447	1.50E-41	postive
TRAJ31	LINC01655	0.572566408	6.33E-48	postive
TRAJ37	LINC01655	0.540234458	7.27E-42	postive
TRAJ38	LINC01655	0.554440919	1.91E-44	postive
TRAJ39	LINC01655	0.558344347	3.54E-45	postive
CREB1	MAL2-AS1	0.785992378	2.13E-113	postive
UBR1	MAL2-AS1	0.667920513	2.15E-70	postive
ZC3HAV1	MAL2-AS1	0.579910884	2.14E-49	postive
IL15	MAL2-AS1	0.531656925	2.30E-40	postive
CYLD	MAL2-AS1	0.584241527	2.78E-50	postive
EIF2AK2	MAL2-AS1	0.58110271	1.22E-49	postive
MAPK8	MAL2-AS1	0.56502103	1.89E-46	postive
LMBR1	MAL2-AS1	0.58628702	1.05E-50	postive
SP1	MAL2-AS1	0.503404057	1.02E-35	postive
IREB2	MAL2-AS1	0.613299537	1.38E-56	postive
DDX17	MAL2-AS1	0.604229962	1.51E-54	postive
PIK3CG	MAL2-AS1	0.502211866	1.57E-35	postive
JAK2	MAL2-AS1	0.627448633	6.72E-60	postive
TXK	MAL2-AS1	0.637671755	2.11E-62	postive
NFAT5	MAL2-AS1	0.793327667	5.77E-117	postive
MALT1	MAL2-AS1	0.523056325	6.65E-39	postive
PIK3R1	MAL2-AS1	0.635142774	8.97E-62	postive
PIK3CA	MAL2-AS1	0.704291947	2.41E-81	postive
PIK3CB	MAL2-AS1	0.567663457	5.81E-47	postive
AKT3	MAL2-AS1	0.53947573	9.91E-42	postive
ROBO2	MAL2-AS1	0.535658077	4.65E-41	postive
GNRH1	MAL2-AS1	0.756174149	3.11E-100	postive
IL6ST	MAL2-AS1	0.605478191	7.97E-55	postive
RABEP1	MAL2-AS1	0.554588344	1.79E-44	postive
ACVR2A	MAL2-AS1	0.642730258	1.12E-63	postive
ANGPTL1	MAL2-AS1	0.783443638	3.42E-112	postive
BMPR1A	MAL2-AS1	0.566091118	1.17E-46	postive
BMPR2	MAL2-AS1	0.76244211	7.72E-103	postive
CRLF3	MAL2-AS1	0.637501201	2.33E-62	postive
NR1D2	MAL2-AS1	0.551414488	6.93E-44	postive
NR2C2	MAL2-AS1	0.684965851	2.52E-75	postive
RORA	MAL2-AS1	0.793264058	6.21E-117	postive
SOS1	MAL2-AS1	0.686113865	1.14E-75	postive
SOS2	MAL2-AS1	0.559585479	2.06E-45	postive
BRAF	MAL2-AS1	0.807435355	3.00E-124	postive
CBL	MAL2-AS1	0.600176551	1.17E-53	postive
CBLB	MAL2-AS1	0.742118784	1.14E-94	postive
PDK1	MAL2-AS1	0.55836682	3.51E-45	postive
TRAJ1	MAL2-AS1	0.674348834	3.25E-72	postive
TRAJ2	MAL2-AS1	0.652926263	2.57E-66	postive
TRAJ3	MAL2-AS1	0.720945033	6.14E-87	postive
TRAJ5	MAL2-AS1	0.741049185	2.93E-94	postive
TRAJ6	MAL2-AS1	0.735626574	3.21E-92	postive
TRAJ8	MAL2-AS1	0.718626015	3.90E-86	postive
TRAJ10	MAL2-AS1	0.689851818	8.39E-77	postive
TRAJ12	MAL2-AS1	0.686652578	7.83E-76	postive
TRAJ13	MAL2-AS1	0.749383954	1.69E-97	postive
TRAJ14	MAL2-AS1	0.721222264	4.91E-87	postive
TRAJ16	MAL2-AS1	0.725043201	2.23E-88	postive
TRAJ17	MAL2-AS1	0.694435779	3.24E-78	postive
TRAJ18	MAL2-AS1	0.667382023	3.05E-70	postive
TRAJ21	MAL2-AS1	0.76944534	7.58E-106	postive
TRAJ31	MAL2-AS1	0.783714429	2.55E-112	postive
TRAJ37	MAL2-AS1	0.727461631	3.07E-89	postive
TRAJ38	MAL2-AS1	0.809374575	2.69E-125	postive
TRAJ39	MAL2-AS1	0.759385272	1.47E-101	postive

NPY	ODC1-DT	0.515083003	1.38E-37	postive
CREB1	AL442125.2	0.605970178	6.20E-55	postive
ZC3HAV1	AL442125.2	0.504888133	5.97E-36	postive
NFAT5	AL442125.2	0.609509574	1.00E-55	postive
PIK3CA	AL442125.2	0.590015837	1.75E-51	postive
GNRH1	AL442125.2	0.579021326	3.23E-49	postive
ANGPTL1	AL442125.2	0.584931526	2.00E-50	postive
BMPR2	AL442125.2	0.562996583	4.63E-46	postive
NR2C2	AL442125.2	0.524062408	4.51E-39	postive
RORA	AL442125.2	0.59641779	7.60E-53	postive
SOS1	AL442125.2	0.539819434	8.62E-42	postive
BRAF	AL442125.2	0.582993273	5.02E-50	postive
CBL	AL442125.2	0.523174694	6.36E-39	postive
CBLB	AL442125.2	0.566556437	9.53E-47	postive
TRAJ1	AL442125.2	0.529322322	5.79E-40	postive
TRAJ2	AL442125.2	0.506593218	3.21E-36	postive
TRAJ3	AL442125.2	0.56072976	1.25E-45	postive
TRAJ5	AL442125.2	0.551788225	5.91E-44	postive
TRAJ6	AL442125.2	0.587750468	5.20E-51	postive
TRAJ8	AL442125.2	0.532427193	1.69E-40	postive
TRAJ10	AL442125.2	0.554815101	1.62E-44	postive
TRAJ13	AL442125.2	0.553008271	3.52E-44	postive
TRAJ14	AL442125.2	0.53763831	2.09E-41	postive
TRAJ16	AL442125.2	0.578363863	4.39E-49	postive
TRAJ17	AL442125.2	0.5395534	9.60E-42	postive
TRAJ18	AL442125.2	0.532461766	1.67E-40	postive
TRAJ21	AL442125.2	0.575982152	1.32E-48	postive
TRAJ31	AL442125.2	0.578423303	4.27E-49	postive
TRAJ37	AL442125.2	0.550857622	8.78E-44	postive
TRAJ38	AL442125.2	0.615215622	5.03E-57	postive
TRAJ39	AL442125.2	0.568655428	3.72E-47	postive
CREB1	AP001033.4	0.788335137	1.60E-114	postive
UBR1	AP001033.4	0.645460187	2.26E-64	postive
ZC3HAV1	AP001033.4	0.561757262	7.98E-46	postive
ZC3HAV1L	AP001033.4	0.503323373	1.05E-35	postive
IL15	AP001033.4	0.547474022	3.65E-43	postive
CYLD	AP001033.4	0.575765805	1.46E-48	postive
EIF2AK2	AP001033.4	0.568297957	4.37E-47	postive
MAPK8	AP001033.4	0.595043444	1.50E-52	postive
LMBR1	AP001033.4	0.58605675	1.17E-50	postive
LIMS1	AP001033.4	0.505270671	5.20E-36	postive
IREB2	AP001033.4	0.63331539	2.53E-61	postive
DDX17	AP001033.4	0.599671169	1.50E-53	postive
JAK2	AP001033.4	0.616492823	2.56E-57	postive
TXK	AP001033.4	0.595000117	1.53E-52	postive
PPP3CB	AP001033.4	0.506502768	3.32E-36	postive
NFAT5	AP001033.4	0.829356617	7.48E-137	postive
MALT1	AP001033.4	0.519651568	2.45E-38	postive
PIK3R1	AP001033.4	0.614668575	6.72E-57	postive
PIK3CA	AP001033.4	0.693102553	8.40E-78	postive
PIK3CB	AP001033.4	0.51558054	1.15E-37	postive
AKT3	AP001033.4	0.578276904	4.57E-49	postive
RASGRP3	AP001033.4	0.502217947	1.57E-35	postive
GNRH1	AP001033.4	0.758256295	4.33E-101	postive
IL6ST	AP001033.4	0.613739611	1.10E-56	postive
RABEP1	AP001033.4	0.553695372	2.62E-44	postive
ACVR2A	AP001033.4	0.642266243	1.48E-63	postive
ANGPTL1	AP001033.4	0.776160643	7.82E-109	postive
BMPR1A	AP001033.4	0.568753916	3.56E-47	postive
BMPR2	AP001033.4	0.753518276	3.74E-99	postive
CRLF3	AP001033.4	0.665037015	1.37E-69	postive
NR1D2	AP001033.4	0.513518755	2.49E-37	postive
NR2C1	AP001033.4	0.5030565	1.16E-35	postive
NR2C2	AP001033.4	0.680277294	6.18E-74	postive
RORA	AP001033.4	0.823077961	4.58E-133	postive

SOS1	AP001033.4	0.712309235	5.47E-84	postive
SOS2	AP001033.4	0.564413335	2.47E-46	postive
BRAF	AP001033.4	0.819672621	4.50E-131	postive
ITK	AP001033.4	0.50996891	9.29E-37	postive
CBL	AP001033.4	0.595993403	9.38E-53	postive
CBLB	AP001033.4	0.746812201	1.74E-96	postive
PDK1	AP001033.4	0.593860607	2.68E-52	postive
TRAJ1	AP001033.4	0.707097891	2.93E-82	postive
TRAJ2	AP001033.4	0.715479055	4.66E-85	postive
TRAJ3	AP001033.4	0.749861354	1.09E-97	postive
TRAJ5	AP001033.4	0.78241735	1.04E-111	postive
TRAJ6	AP001033.4	0.766333225	1.70E-104	postive
TRAJ8	AP001033.4	0.764163382	1.44E-103	postive
TRAJ10	AP001033.4	0.731000961	1.62E-90	postive
TRAJ12	AP001033.4	0.691112036	3.45E-77	postive
TRAJ13	AP001033.4	0.792765821	1.10E-116	postive
TRAJ14	AP001033.4	0.755766338	4.57E-100	postive
TRAJ16	AP001033.4	0.763525822	2.69E-103	postive
TRAJ17	AP001033.4	0.721206588	4.98E-87	postive
TRAJ18	AP001033.4	0.677867334	3.13E-73	postive
TRAJ21	AP001033.4	0.800156102	2.04E-120	postive
TRAJ31	AP001033.4	0.827347252	1.27E-135	postive
TRAJ37	AP001033.4	0.790737893	1.09E-115	postive
TRAJ38	AP001033.4	0.845831001	1.42E-147	postive
TRAJ39	AP001033.4	0.805595976	2.89E-123	postive
CREB1	AC048344.4	0.739731832	9.27E-94	postive
PSMD6	AC048344.4	0.505238923	5.26E-36	postive
UBR1	AC048344.4	0.538662591	1.38E-41	postive
ZC3HAV1	AC048344.4	0.525726623	2.36E-39	postive
CYLD	AC048344.4	0.519077811	3.05E-38	postive
EIF2AK2	AC048344.4	0.582229711	7.19E-50	postive
MAPK8	AC048344.4	0.542583738	2.78E-42	postive
LMBR1	AC048344.4	0.586286265	1.05E-50	postive
LIMS1	AC048344.4	0.521065448	1.43E-38	postive
IREB2	AC048344.4	0.584219297	2.81E-50	postive
DDX17	AC048344.4	0.505888573	4.15E-36	postive
JAK2	AC048344.4	0.603899148	1.78E-54	postive
TXK	AC048344.4	0.537209115	2.49E-41	postive
NFAT5	AC048344.4	0.70420611	2.57E-81	postive
MALT1	AC048344.4	0.514335067	1.83E-37	postive
PIK3R1	AC048344.4	0.565204929	1.74E-46	postive
PIK3CA	AC048344.4	0.713504489	2.17E-84	postive
PIK3CB	AC048344.4	0.5355083	4.94E-41	postive
AKT3	AC048344.4	0.527105992	1.38E-39	postive
RASGRP3	AC048344.4	0.501317143	2.17E-35	postive
GNRH1	AC048344.4	0.647146356	8.31E-65	postive
IL6ST	AC048344.4	0.5526954	4.02E-44	postive
ACVR2A	AC048344.4	0.610720646	5.33E-56	postive
ANGPTL1	AC048344.4	0.735238127	4.48E-92	postive
BMPR2	AC048344.4	0.70037899	4.36E-80	postive
CRLF3	AC048344.4	0.652921796	2.57E-66	postive
NR2C2	AC048344.4	0.601318395	6.58E-54	postive
RORA	AC048344.4	0.747110978	1.33E-96	postive
SOS1	AC048344.4	0.681352829	2.98E-74	postive
SOS2	AC048344.4	0.529054111	6.43E-40	postive
BRAF	AC048344.4	0.746585851	2.13E-96	postive
CBL	AC048344.4	0.569986379	2.04E-47	postive
CBLB	AC048344.4	0.694085738	4.17E-78	postive
PDK1	AC048344.4	0.581175667	1.18E-49	postive
TRAJ1	AC048344.4	0.68527922	2.03E-75	postive
TRAJ2	AC048344.4	0.591512139	8.44E-52	postive
TRAJ3	AC048344.4	0.647532082	6.60E-65	postive
TRAJ5	AC048344.4	0.806030384	1.70E-123	postive
TRAJ6	AC048344.4	0.698008758	2.46E-79	postive
TRAJ8	AC048344.4	0.736274292	1.85E-92	postive

TRAJ10	AC048344.4	0.672302055	1.25E-71	postive
TRAJ12	AC048344.4	0.634680121	1.17E-61	postive
TRAJ13	AC048344.4	0.772386552	3.84E-107	postive
TRAJ14	AC048344.4	0.718807661	3.38E-86	postive
TRAJ16	AC048344.4	0.742379747	9.07E-95	postive
TRAJ17	AC048344.4	0.721868964	2.92E-87	postive
TRAJ18	AC048344.4	0.612049269	2.66E-56	postive
TRAJ21	AC048344.4	0.764844262	7.37E-104	postive
TRAJ31	AC048344.4	0.798045995	2.46E-119	postive
TRAJ37	AC048344.4	0.709171808	6.07E-83	postive
TRAJ38	AC048344.4	0.782736413	7.34E-112	postive
TRAJ39	AC048344.4	0.769982518	4.41E-106	postive
CREB1	AC026356.1	0.709377822	5.19E-83	postive
UBR1	AC026356.1	0.505148124	5.43E-36	postive
ZC3HAV1	AC026356.1	0.523419854	5.78E-39	postive
IL15	AC026356.1	0.535122492	5.76E-41	postive
CYLD	AC026356.1	0.523036448	6.70E-39	postive
EIF2AK2	AC026356.1	0.602708559	3.26E-54	postive
LMBR1	AC026356.1	0.6345785	1.24E-61	postive
LIMS1	AC026356.1	0.59419453	2.27E-52	postive
IREB2	AC026356.1	0.519247776	2.86E-38	postive
JAK2	AC026356.1	0.621232775	2.01E-58	postive
TXK	AC026356.1	0.536035785	3.99E-41	postive
NFAT5	AC026356.1	0.66874125	1.27E-70	postive
MALT1	AC026356.1	0.509062905	1.30E-36	postive
PIK3R1	AC026356.1	0.553679217	2.64E-44	postive
PIK3CA	AC026356.1	0.732016875	6.89E-91	postive
PIK3CB	AC026356.1	0.537267892	2.43E-41	postive
AKT3	AC026356.1	0.519816958	2.30E-38	postive
RASGRP3	AC026356.1	0.528853494	6.96E-40	postive
PLXNC1	AC026356.1	0.519873738	2.26E-38	postive
GNRH1	AC026356.1	0.596614767	6.89E-53	postive
IL6ST	AC026356.1	0.51834195	4.04E-38	postive
ACVR2A	AC026356.1	0.534720312	6.77E-41	postive
ANGPTL1	AC026356.1	0.721438479	4.13E-87	postive
BMPR2	AC026356.1	0.691535411	2.56E-77	postive
CRLF3	AC026356.1	0.608052434	2.12E-55	postive
NR2C2	AC026356.1	0.562747526	5.16E-46	postive
RORA	AC026356.1	0.695794348	1.22E-78	postive
SOS1	AC026356.1	0.676594585	7.33E-73	postive
SOS2	AC026356.1	0.524936849	3.21E-39	postive
BRAF	AC026356.1	0.706906718	3.38E-82	postive
ITK	AC026356.1	0.501958474	1.72E-35	postive
CBL	AC026356.1	0.621551649	1.69E-58	postive
CBLB	AC026356.1	0.716152888	2.75E-85	postive
PDK1	AC026356.1	0.559365902	2.27E-45	postive
TRAJ1	AC026356.1	0.648146665	4.57E-65	postive
TRAJ2	AC026356.1	0.56414592	2.78E-46	postive
TRAJ3	AC026356.1	0.638528359	1.29E-62	postive
TRAJ5	AC026356.1	0.760341707	5.89E-102	postive
TRAJ6	AC026356.1	0.658524111	8.22E-68	postive
TRAJ8	AC026356.1	0.68101987	3.74E-74	postive
TRAJ10	AC026356.1	0.635663485	6.67E-62	postive
TRAJ12	AC026356.1	0.585410976	1.59E-50	postive
TRAJ13	AC026356.1	0.706350955	5.14E-82	postive
TRAJ14	AC026356.1	0.674119078	3.78E-72	postive
TRAJ16	AC026356.1	0.708965879	7.10E-83	postive
TRAJ17	AC026356.1	0.67965977	9.38E-74	postive
TRAJ18	AC026356.1	0.594911821	1.60E-52	postive
TRAJ21	AC026356.1	0.719617195	1.77E-86	postive
TRAJ31	AC026356.1	0.735756497	2.88E-92	postive
TRAJ37	AC026356.1	0.657689523	1.38E-67	postive
TRAJ38	AC026356.1	0.737881731	4.62E-93	postive
TRAJ39	AC026356.1	0.737894513	4.57E-93	postive
A2M	AP001189.3	0.523925488	4.76E-39	postive

PDGFRB	AP001189.3	0.615952599	3.41E-57	postive
SEMA3G	AP001189.3	0.581969809	8.13E-50	postive
ACVRL1	AP001189.3	0.56870275	3.64E-47	postive
S1PR1	AP001189.3	0.638988608	9.90E-63	postive
TEK	AP001189.3	0.60776704	2.46E-55	postive
TIE1	AP001189.3	0.541399051	4.52E-42	postive
KRAS	AL049836.1	0.561986843	7.22E-46	postive
CREB1	AC002128.1	0.60276794	3.16E-54	postive
RFXAP	AC002128.1	0.526621961	1.67E-39	postive
UBR1	AC002128.1	0.521081948	1.42E-38	postive
CYLD	AC002128.1	0.509943229	9.38E-37	postive
NFKBIZ	AC002128.1	0.56149446	8.96E-46	postive
DDX17	AC002128.1	0.685753108	1.46E-75	postive
JAK2	AC002128.1	0.521435387	1.24E-38	postive
TXK	AC002128.1	0.538647568	1.39E-41	postive
NFAT5	AC002128.1	0.671517154	2.09E-71	postive
PIK3CA	AC002128.1	0.533765362	9.93E-41	postive
LTB4R2	AC002128.1	0.617784513	1.28E-57	postive
GNRH1	AC002128.1	0.794964314	8.83E-118	postive
ACVR2A	AC002128.1	0.553663485	2.66E-44	postive
ANGPTL1	AC002128.1	0.575082098	2.00E-48	postive
BMPR2	AC002128.1	0.532684575	1.53E-40	postive
CRLF3	AC002128.1	0.518284078	4.13E-38	postive
NR2C1	AC002128.1	0.582213155	7.25E-50	postive
NR2C2	AC002128.1	0.747749174	7.47E-97	postive
RORA	AC002128.1	0.667199633	3.43E-70	postive
SOS1	AC002128.1	0.544073191	1.50E-42	postive
BRAF	AC002128.1	0.656822124	2.36E-67	postive
CBL	AC002128.1	0.518412446	3.93E-38	postive
CBLB	AC002128.1	0.592063167	6.46E-52	postive
TRAJ1	AC002128.1	0.603764822	1.91E-54	postive
TRAJ2	AC002128.1	0.577393374	6.89E-49	postive
TRAJ3	AC002128.1	0.631709153	6.25E-61	postive
TRAJ5	AC002128.1	0.598543104	2.64E-53	postive
TRAJ6	AC002128.1	0.60463842	1.22E-54	postive
TRAJ8	AC002128.1	0.573714081	3.75E-48	postive
TRAJ10	AC002128.1	0.579662577	2.40E-49	postive
TRAJ12	AC002128.1	0.541912446	3.66E-42	postive
TRAJ13	AC002128.1	0.578551142	4.03E-49	postive
TRAJ14	AC002128.1	0.567854989	5.33E-47	postive
TRAJ16	AC002128.1	0.597912785	3.62E-53	postive
TRAJ17	AC002128.1	0.56992793	2.10E-47	postive
TRAJ18	AC002128.1	0.54519286	9.44E-43	postive
TRAJ21	AC002128.1	0.602596804	3.45E-54	postive
TRAJ31	AC002128.1	0.61343746	1.29E-56	postive
TRAJ37	AC002128.1	0.592075496	6.42E-52	postive
TRAJ38	AC002128.1	0.644365482	4.31E-64	postive
TRAJ39	AC002128.1	0.620819859	2.52E-58	postive
CREB1	AP001628.1	0.601479728	6.06E-54	postive
UBR1	AP001628.1	0.541924353	3.64E-42	postive
CYLD	AP001628.1	0.507227717	2.55E-36	postive
DDX17	AP001628.1	0.566704126	8.92E-47	postive
NFAT5	AP001628.1	0.82265901	8.10E-133	postive
NFATC3	AP001628.1	0.556820277	6.85E-45	postive
GNRH1	AP001628.1	0.706747331	3.81E-82	postive
ACVR2A	AP001628.1	0.505082025	5.57E-36	postive
ANGPTL1	AP001628.1	0.501151594	2.30E-35	postive
CRLF3	AP001628.1	0.554530419	1.84E-44	postive
NR2C2	AP001628.1	0.631974234	5.38E-61	postive
RORA	AP001628.1	0.688115426	2.83E-76	postive
SOS1	AP001628.1	0.502225841	1.56E-35	postive
BRAF	AP001628.1	0.613901105	1.01E-56	postive
TRAJ1	AP001628.1	0.69782031	2.82E-79	postive
TRAJ2	AP001628.1	0.729151873	7.57E-90	postive
TRAJ3	AP001628.1	0.730154971	3.28E-90	postive

TRAJ5	AP001628.1	0.63360231	2.15E-61	postive
TRAJ6	AP001628.1	0.757284524	1.09E-100	postive
TRAJ8	AP001628.1	0.687134271	5.61E-76	postive
TRAJ10	AP001628.1	0.746036077	3.50E-96	postive
TRAJ12	AP001628.1	0.629889987	1.73E-60	postive
TRAJ13	AP001628.1	0.682958356	9.98E-75	postive
TRAJ14	AP001628.1	0.671925263	1.60E-71	postive
TRAJ16	AP001628.1	0.713213484	2.72E-84	postive
TRAJ17	AP001628.1	0.637355444	2.53E-62	postive
TRAJ18	AP001628.1	0.653774452	1.53E-66	postive
TRAJ21	AP001628.1	0.685958177	1.27E-75	postive
TRAJ31	AP001628.1	0.711797645	8.12E-84	postive
TRAJ37	AP001628.1	0.778478875	6.88E-110	postive
TRAJ38	AP001628.1	0.748292446	4.56E-97	postive
TRAJ39	AP001628.1	0.723635609	7.01E-88	postive
CREB1	KLF7-IT1	0.776043548	8.83E-109	postive
UBR1	KLF7-IT1	0.649583249	1.94E-65	postive
ZC3HAV1	KLF7-IT1	0.557290288	5.59E-45	postive
ZC3HAV1L	KLF7-IT1	0.512351634	3.84E-37	postive
IL15	KLF7-IT1	0.655925583	4.10E-67	postive
CYLD	KLF7-IT1	0.599113298	1.99E-53	postive
EIF2AK2	KLF7-IT1	0.533155372	1.27E-40	postive
MAPK8	KLF7-IT1	0.54841301	2.46E-43	postive
TLR1	KLF7-IT1	0.520591864	1.71E-38	postive
NFKBIZ	KLF7-IT1	0.502829151	1.26E-35	postive
LMBR1	KLF7-IT1	0.575550853	1.61E-48	postive
LIMS1	KLF7-IT1	0.5324754	1.66E-40	postive
IREB2	KLF7-IT1	0.604079789	1.63E-54	postive
DDX17	KLF7-IT1	0.556353419	8.38E-45	postive
PIK3CG	KLF7-IT1	0.560632369	1.31E-45	postive
JAK2	KLF7-IT1	0.662534818	6.68E-69	postive
TXK	KLF7-IT1	0.593757148	2.82E-52	postive
NFAT5	KLF7-IT1	0.783438556	3.44E-112	postive
MALT1	KLF7-IT1	0.54544901	8.48E-43	postive
PIK3R1	KLF7-IT1	0.62814453	4.57E-60	postive
PIK3CA	KLF7-IT1	0.715641821	4.10E-85	postive
PIK3CB	KLF7-IT1	0.56288693	4.85E-46	postive
AKT3	KLF7-IT1	0.525242169	2.85E-39	postive
RASGRP3	KLF7-IT1	0.52548073	2.60E-39	postive
GNRH1	KLF7-IT1	0.697337593	4.01E-79	postive
IL6ST	KLF7-IT1	0.589520108	2.22E-51	postive
RABEP1	KLF7-IT1	0.541006734	5.30E-42	postive
ACVR2A	KLF7-IT1	0.579868569	2.18E-49	postive
ANGPTL1	KLF7-IT1	0.801193883	5.92E-121	postive
BMPR1A	KLF7-IT1	0.524080542	4.48E-39	postive
BMPR2	KLF7-IT1	0.726183243	8.78E-89	postive
CRLF3	KLF7-IT1	0.618545971	8.55E-58	postive
IL18R1	KLF7-IT1	0.553064797	3.44E-44	postive
NR2C2	KLF7-IT1	0.659976182	3.33E-68	postive
RORA	KLF7-IT1	0.785106622	5.61E-113	postive
SOS1	KLF7-IT1	0.65240848	3.52E-66	postive
SOS2	KLF7-IT1	0.558818751	2.88E-45	postive
BRAF	KLF7-IT1	0.761893784	1.31E-102	postive
ITK	KLF7-IT1	0.517666715	5.22E-38	postive
CBL	KLF7-IT1	0.622731069	8.93E-59	postive
CBLB	KLF7-IT1	0.755045824	8.99E-100	postive
RASGRP1	KLF7-IT1	0.510781752	6.88E-37	postive
PDK1	KLF7-IT1	0.622611955	9.53E-59	postive
TRAJ1	KLF7-IT1	0.652086408	4.27E-66	postive
TRAJ2	KLF7-IT1	0.691158165	3.34E-77	postive
TRAJ3	KLF7-IT1	0.762099082	1.08E-102	postive
TRAJ5	KLF7-IT1	0.700647994	3.58E-80	postive
TRAJ6	KLF7-IT1	0.742181628	1.08E-94	postive
TRAJ8	KLF7-IT1	0.667651235	2.56E-70	postive
TRAJ10	KLF7-IT1	0.734656261	7.36E-92	postive

TRAJ12	KLF7-IT1	0.663056873	4.80E-69	postive
TRAJ13	KLF7-IT1	0.69359106	5.93E-78	postive
TRAJ14	KLF7-IT1	0.705835112	7.58E-82	postive
TRAJ16	KLF7-IT1	0.723549192	7.52E-88	postive
TRAJ17	KLF7-IT1	0.694080193	4.18E-78	postive
TRAJ18	KLF7-IT1	0.709992305	3.25E-83	postive
TRAJ21	KLF7-IT1	0.748168911	5.10E-97	postive
TRAJ31	KLF7-IT1	0.750522491	5.96E-98	postive
TRAJ37	KLF7-IT1	0.715936617	3.25E-85	postive
TRAJ38	KLF7-IT1	0.816106762	4.95E-129	postive
TRAJ39	KLF7-IT1	0.748782042	2.92E-97	postive
NFAT5	AC124045.1	0.541776797	3.87E-42	postive
GNRH1	AC124045.1	0.556164061	9.10E-45	postive
NR2C2	AC124045.1	0.567017721	7.75E-47	postive
RORA	AC124045.1	0.548149747	2.75E-43	postive
TRAJ31	AC124045.1	0.506514437	3.30E-36	postive
TRAJ39	AC124045.1	0.505960827	4.04E-36	postive
CREB1	AC002550.2	0.692270223	1.52E-77	postive
UBR1	AC002550.2	0.54887221	2.03E-43	postive
ZC3HAV1	AC002550.2	0.511628241	5.03E-37	postive
CYLD	AC002550.2	0.566984724	7.87E-47	postive
EIF2AK2	AC002550.2	0.501456542	2.06E-35	postive
MAPK8	AC002550.2	0.505685679	4.47E-36	postive
IREB2	AC002550.2	0.526883303	1.51E-39	postive
DDX17	AC002550.2	0.622606704	9.56E-59	postive
JAK2	AC002550.2	0.59973698	1.46E-53	postive
TXK	AC002550.2	0.543861202	1.64E-42	postive
NFAT5	AC002550.2	0.794546942	1.43E-117	postive
MALT1	AC002550.2	0.536486838	3.33E-41	postive
PIK3R1	AC002550.2	0.580696404	1.48E-49	postive
PIK3CA	AC002550.2	0.608406067	1.77E-55	postive
GNRH1	AC002550.2	0.698676819	1.51E-79	postive
IL6ST	AC002550.2	0.603660193	2.01E-54	postive
RABEP1	AC002550.2	0.506160488	3.76E-36	postive
ACVR2A	AC002550.2	0.547786601	3.20E-43	postive
ANGPTL1	AC002550.2	0.718687528	3.71E-86	postive
BMPR2	AC002550.2	0.628418175	3.93E-60	postive
CRLF3	AC002550.2	0.58602873	1.19E-50	postive
NR2C2	AC002550.2	0.656843438	2.33E-67	postive
RORA	AC002550.2	0.819296605	7.42E-131	postive
SOS1	AC002550.2	0.63015191	1.50E-60	postive
SOS2	AC002550.2	0.515269329	1.29E-37	postive
BRAF	AC002550.2	0.739824351	8.55E-94	postive
CBL	AC002550.2	0.56373242	3.34E-46	postive
CBLB	AC002550.2	0.736396139	1.66E-92	postive
PDK1	AC002550.2	0.548511509	2.36E-43	postive
TRAJ1	AC002550.2	0.649803518	1.70E-65	postive
TRAJ2	AC002550.2	0.698266723	2.04E-79	postive
TRAJ3	AC002550.2	0.671817055	1.72E-71	postive
TRAJ5	AC002550.2	0.731925427	7.44E-91	postive
TRAJ6	AC002550.2	0.692539994	1.25E-77	postive
TRAJ8	AC002550.2	0.653502586	1.81E-66	postive
TRAJ10	AC002550.2	0.677579025	3.80E-73	postive
TRAJ12	AC002550.2	0.577672765	6.05E-49	postive
TRAJ13	AC002550.2	0.697190603	4.46E-79	postive
TRAJ14	AC002550.2	0.671564348	2.03E-71	postive
TRAJ16	AC002550.2	0.712278952	5.60E-84	postive
TRAJ17	AC002550.2	0.623446458	6.05E-59	postive
TRAJ18	AC002550.2	0.637368386	2.51E-62	postive
TRAJ21	AC002550.2	0.715096044	6.28E-85	postive
TRAJ31	AC002550.2	0.742862876	5.92E-95	postive
TRAJ37	AC002550.2	0.733438357	2.07E-91	postive
TRAJ38	AC002550.2	0.781593838	2.51E-111	postive
TRAJ39	AC002550.2	0.747354009	1.07E-96	postive
CREB1	AP001625.2	0.585203793	1.76E-50	postive

UBR1	AP001625.2	0.532851503	1.43E-40	postive
ZC3HAV1	AP001625.2	0.501937237	1.73E-35	postive
ZC3HAV1L	AP001625.2	0.509601418	1.06E-36	postive
MAPK8	AP001625.2	0.509799215	9.89E-37	postive
IREB2	AP001625.2	0.507237738	2.54E-36	postive
DDX17	AP001625.2	0.577184168	7.59E-49	postive
TXK	AP001625.2	0.541470527	4.39E-42	postive
NFAT5	AP001625.2	0.660386557	2.57E-68	postive
PIK3CA	AP001625.2	0.510173486	8.62E-37	postive
LTB4R2	AP001625.2	0.572746927	5.83E-48	postive
GNRH1	AP001625.2	0.711882461	7.61E-84	postive
ACVR2A	AP001625.2	0.518251658	4.18E-38	postive
ANGPTL1	AP001625.2	0.58553943	1.50E-50	postive
BMPR2	AP001625.2	0.530368354	3.83E-40	postive
CRLF3	AP001625.2	0.521867643	1.05E-38	postive
NR2C1	AP001625.2	0.534529019	7.31E-41	postive
NR2C2	AP001625.2	0.66504097	1.36E-69	postive
RORA	AP001625.2	0.638793065	1.11E-62	postive
SOS1	AP001625.2	0.557635102	4.82E-45	postive
BRAF	AP001625.2	0.694055298	4.26E-78	postive
CBL	AP001625.2	0.524727886	3.49E-39	postive
CBLB	AP001625.2	0.641965865	1.76E-63	postive
TRAJ1	AP001625.2	0.545849795	7.18E-43	postive
TRAJ2	AP001625.2	0.541661987	4.05E-42	postive
TRAJ3	AP001625.2	0.581627186	9.55E-50	postive
TRAJ5	AP001625.2	0.554693064	1.71E-44	postive
TRAJ6	AP001625.2	0.576674575	9.61E-49	postive
TRAJ8	AP001625.2	0.54053336	6.44E-42	postive
TRAJ10	AP001625.2	0.532527788	1.63E-40	postive
TRAJ12	AP001625.2	0.517088122	6.50E-38	postive
TRAJ13	AP001625.2	0.557544684	5.01E-45	postive
TRAJ14	AP001625.2	0.547915495	3.03E-43	postive
TRAJ16	AP001625.2	0.548410415	2.46E-43	postive
TRAJ17	AP001625.2	0.504982263	5.77E-36	postive
TRAJ18	AP001625.2	0.506285766	3.59E-36	postive
TRAJ21	AP001625.2	0.578345642	4.43E-49	postive
TRAJ31	AP001625.2	0.582528958	6.25E-50	postive
TRAJ37	AP001625.2	0.567671294	5.79E-47	postive
TRAJ38	AP001625.2	0.622453275	1.04E-58	postive
TRAJ39	AP001625.2	0.58033534	1.75E-49	postive
CHGB	H19	0.585496136	1.53E-50	postive
UCN	AC074117.1	0.53205	1.97E-40	postive
GNRH1	SNHG20	0.517090058	6.49E-38	postive
OSGIN1	AL512353.1	0.515617318	1.13E-37	postive
CREB1	AC016394.3	0.616154214	3.06E-57	postive
UBR1	AC016394.3	0.553855893	2.45E-44	postive
ZC3HAV1L	AC016394.3	0.572734189	5.87E-48	postive
MAPK8	AC016394.3	0.555797704	1.07E-44	postive
EED	AC016394.3	0.501206183	2.26E-35	postive
IREB2	AC016394.3	0.523051088	6.67E-39	postive
DDX17	AC016394.3	0.56572396	1.38E-46	postive
NFAT5	AC016394.3	0.708347584	1.14E-82	postive
AKT3	AC016394.3	0.522871107	7.14E-39	postive
GNRH1	AC016394.3	0.715979179	3.15E-85	postive
ANGPTL1	AC016394.3	0.570687494	1.49E-47	postive
BMPR2	AC016394.3	0.502488951	1.42E-35	postive
CRLF3	AC016394.3	0.580078561	1.97E-49	postive
NR2C1	AC016394.3	0.553615774	2.72E-44	postive
NR2C2	AC016394.3	0.627562546	6.31E-60	postive
RORA	AC016394.3	0.630356539	1.33E-60	postive
SOS1	AC016394.3	0.585531028	1.50E-50	postive
BRAF	AC016394.3	0.683391444	7.42E-75	postive
CBL	AC016394.3	0.508255511	1.75E-36	postive
CBLB	AC016394.3	0.551243534	7.45E-44	postive
PDK1	AC016394.3	0.553903288	2.40E-44	postive

TRAJ1	AC016394.3	0.620992612	2.29E-58	postive
TRAJ2	AC016394.3	0.663521739	3.58E-69	postive
TRAJ3	AC016394.3	0.662301286	7.74E-69	postive
TRAJ5	AC016394.3	0.61548889	4.36E-57	postive
TRAJ6	AC016394.3	0.675828793	1.22E-72	postive
TRAJ8	AC016394.3	0.644826373	3.28E-64	postive
TRAJ10	AC016394.3	0.635990585	5.53E-62	postive
TRAJ12	AC016394.3	0.571466208	1.04E-47	postive
TRAJ13	AC016394.3	0.642497677	1.29E-63	postive
TRAJ14	AC016394.3	0.633477885	2.31E-61	postive
TRAJ16	AC016394.3	0.639178949	8.87E-63	postive
TRAJ17	AC016394.3	0.583925949	3.23E-50	postive
TRAJ18	AC016394.3	0.562785654	5.08E-46	postive
TRAJ21	AC016394.3	0.657380834	1.67E-67	postive
TRAJ31	AC016394.3	0.65485096	7.93E-67	postive
TRAJ37	AC016394.3	0.665882201	7.97E-70	postive
TRAJ38	AC016394.3	0.698325208	1.96E-79	postive
TRAJ39	AC016394.3	0.671384817	2.28E-71	postive
CREB1	AL139041.1	0.53982896	8.58E-42	postive
DDX17	AL139041.1	0.638983544	9.93E-63	postive
NFAT5	AL139041.1	0.660203722	2.89E-68	postive
PIK3R1	AL139041.1	0.535490985	4.97E-41	postive
GNRH1	AL139041.1	0.719032112	2.83E-86	postive
IL6ST	AL139041.1	0.570156473	1.89E-47	postive
BMPR2	AL139041.1	0.504017925	8.19E-36	postive
NR2C2	AL139041.1	0.661234957	1.51E-68	postive
RORA	AL139041.1	0.678736152	1.75E-73	postive
BRAF	AL139041.1	0.56754393	6.13E-47	postive
TRAJ1	AL139041.1	0.54889218	2.01E-43	postive
TRAJ2	AL139041.1	0.536110453	3.87E-41	postive
TRAJ3	AL139041.1	0.596305208	8.04E-53	postive
TRAJ5	AL139041.1	0.563288609	4.07E-46	postive
TRAJ6	AL139041.1	0.564684621	2.19E-46	postive
TRAJ8	AL139041.1	0.519562193	2.54E-38	postive
TRAJ10	AL139041.1	0.571446054	1.05E-47	postive
TRAJ12	AL139041.1	0.51462646	1.64E-37	postive
TRAJ13	AL139041.1	0.527608025	1.13E-39	postive
TRAJ14	AL139041.1	0.513866717	2.18E-37	postive
TRAJ16	AL139041.1	0.541158509	4.98E-42	postive
TRAJ17	AL139041.1	0.525260758	2.83E-39	postive
TRAJ18	AL139041.1	0.554134533	2.18E-44	postive
TRAJ21	AL139041.1	0.561182221	1.03E-45	postive
TRAJ31	AL139041.1	0.584878179	2.05E-50	postive
TRAJ37	AL139041.1	0.610979103	4.66E-56	postive
TRAJ38	AL139041.1	0.59450684	1.95E-52	postive
TRAJ39	AL139041.1	0.600334461	1.08E-53	postive
CREB1	AC004253.1	0.549871148	1.33E-43	postive
NFKBIZ	AC004253.1	0.522980953	6.85E-39	postive
DDX17	AC004253.1	0.655611926	4.97E-67	postive
NFAT5	AC004253.1	0.652605311	3.12E-66	postive
LTB4R2	AC004253.1	0.621502411	1.74E-58	postive
GNRH1	AC004253.1	0.77239796	3.79E-107	postive
ANGPTL1	AC004253.1	0.57100022	1.29E-47	postive
CRLF3	AC004253.1	0.574353178	2.80E-48	postive
NR2C1	AC004253.1	0.576850634	8.86E-49	postive
NR2C2	AC004253.1	0.683591494	6.47E-75	postive
RORA	AC004253.1	0.626193085	1.34E-59	postive
BRAF	AC004253.1	0.674957044	2.17E-72	postive
CBLB	AC004253.1	0.595350468	1.29E-52	postive
TRAJ1	AC004253.1	0.576794951	9.09E-49	postive
TRAJ2	AC004253.1	0.634864828	1.05E-61	postive
TRAJ3	AC004253.1	0.628109551	4.66E-60	postive
TRAJ5	AC004253.1	0.562225556	6.50E-46	postive
TRAJ6	AC004253.1	0.588117878	4.36E-51	postive
TRAJ8	AC004253.1	0.571119097	1.22E-47	postive

TRAJ10	AC004253.1	0.556426452	8.12E-45	postive
TRAJ12	AC004253.1	0.501657932	1.92E-35	postive
TRAJ13	AC004253.1	0.574456561	2.67E-48	postive
TRAJ14	AC004253.1	0.568389736	4.19E-47	postive
TRAJ16	AC004253.1	0.574007949	3.28E-48	postive
TRAJ17	AC004253.1	0.52157714	1.18E-38	postive
TRAJ18	AC004253.1	0.515764198	1.07E-37	postive
TRAJ21	AC004253.1	0.586435176	9.77E-51	postive
TRAJ31	AC004253.1	0.591069923	1.05E-51	postive
TRAJ37	AC004253.1	0.594507867	1.95E-52	postive
TRAJ38	AC004253.1	0.64066229	3.76E-63	postive
TRAJ39	AC004253.1	0.595417642	1.25E-52	postive
CREB1	AC108010.1	0.761001001	3.12E-102	postive
CIITA	AC108010.1	0.509463828	1.12E-36	postive
RFXAP	AC108010.1	0.519528278	2.57E-38	postive
UBR1	AC108010.1	0.665581526	9.66E-70	postive
ZC3HAV1	AC108010.1	0.6560233	3.86E-67	postive
ZC3HAV1L	AC108010.1	0.650636043	1.03E-65	postive
IL15	AC108010.1	0.561994939	7.19E-46	postive
CYLD	AC108010.1	0.654937463	7.52E-67	postive
EIF2AK2	AC108010.1	0.578347998	4.42E-49	postive
MAPK8	AC108010.1	0.514203856	1.92E-37	postive
NFKBIZ	AC108010.1	0.533028392	1.33E-40	postive
LMBR1	AC108010.1	0.591176893	9.94E-52	postive
IREB2	AC108010.1	0.592228971	5.95E-52	postive
DDX17	AC108010.1	0.676992118	5.62E-73	postive
PIK3CG	AC108010.1	0.558162441	3.83E-45	postive
JAK2	AC108010.1	0.660384	2.58E-68	postive
TXK	AC108010.1	0.592299222	5.75E-52	postive
NFAT5	AC108010.1	0.818264027	2.92E-130	postive
NFATC3	AC108010.1	0.514222626	1.91E-37	postive
MALT1	AC108010.1	0.533456595	1.12E-40	postive
PIK3R1	AC108010.1	0.593403463	3.35E-52	postive
PIK3CA	AC108010.1	0.681700673	2.35E-74	postive
PIK3CB	AC108010.1	0.559356919	2.28E-45	postive
RASGRP3	AC108010.1	0.528820278	7.05E-40	postive
IGHD6-6	AC108010.1	0.53554847	4.86E-41	postive
LTB4R2	AC108010.1	0.572086271	7.88E-48	postive
GNRH1	AC108010.1	0.752887716	6.73E-99	postive
IL6ST	AC108010.1	0.551191909	7.62E-44	postive
RABEP1	AC108010.1	0.60409208	1.62E-54	postive
ACVR2A	AC108010.1	0.562461773	5.85E-46	postive
ANGPTL1	AC108010.1	0.66265896	6.17E-69	postive
BMPR2	AC108010.1	0.655797773	4.43E-67	postive
CRLF3	AC108010.1	0.607255305	3.20E-55	postive
NR1D2	AC108010.1	0.501753544	1.85E-35	postive
NR2C1	AC108010.1	0.525378593	2.71E-39	postive
NR2C2	AC108010.1	0.805721021	2.48E-123	postive
RORA	AC108010.1	0.762708607	5.96E-103	postive
SOS1	AC108010.1	0.700732201	3.36E-80	postive
SOS2	AC108010.1	0.590029498	1.73E-51	postive
BRAF	AC108010.1	0.800482248	1.38E-120	postive
ITK	AC108010.1	0.517280832	6.04E-38	postive
TEC	AC108010.1	0.558854289	2.84E-45	postive
CBL	AC108010.1	0.696866608	5.63E-79	postive
CBLB	AC108010.1	0.687176665	5.44E-76	postive
RASGRP1	AC108010.1	0.526228191	1.94E-39	postive
PDK1	AC108010.1	0.579913083	2.13E-49	postive
TRAJ1	AC108010.1	0.717620611	8.65E-86	postive
TRAJ2	AC108010.1	0.669538433	7.57E-71	postive
TRAJ3	AC108010.1	0.766396379	1.59E-104	postive
TRAJ5	AC108010.1	0.711956736	7.19E-84	postive
TRAJ6	AC108010.1	0.741686391	1.67E-94	postive
TRAJ8	AC108010.1	0.65528545	6.07E-67	postive
TRAJ10	AC108010.1	0.726932572	4.74E-89	postive

TRAJ12	AC108010.1	0.650318126	1.24E-65	postive
TRAJ13	AC108010.1	0.681135023	3.46E-74	postive
TRAJ14	AC108010.1	0.675429176	1.59E-72	postive
TRAJ16	AC108010.1	0.707932753	1.56E-82	postive
TRAJ17	AC108010.1	0.678964101	1.50E-73	postive
TRAJ18	AC108010.1	0.69755107	3.43E-79	postive
TRAJ21	AC108010.1	0.716565185	1.99E-85	postive
TRAJ31	AC108010.1	0.717182981	1.22E-85	postive
TRAJ37	AC108010.1	0.715379868	5.03E-85	postive
TRAJ38	AC108010.1	0.758994762	2.14E-101	postive
TRAJ39	AC108010.1	0.732832603	3.46E-91	postive
IRF3	AC008735.2	0.531276264	2.67E-40	postive
TYK2	AC008735.2	0.531704312	2.26E-40	postive
IRF9	AC008735.2	0.578404572	4.31E-49	postive
TNFRSF25	AC008735.2	0.513073147	2.94E-37	postive
CREB1	AC005632.3	0.716952959	1.46E-85	postive
UBR1	AC005632.3	0.571169102	1.20E-47	postive
ZC3HAV1	AC005632.3	0.500393783	3.02E-35	postive
IL15	AC005632.3	0.621826348	1.46E-58	postive
CYLD	AC005632.3	0.607559668	2.74E-55	postive
TLR1	AC005632.3	0.537562383	2.16E-41	postive
NFKBIZ	AC005632.3	0.523388862	5.85E-39	postive
LMBR1	AC005632.3	0.512829392	3.22E-37	postive
LIMS1	AC005632.3	0.52019906	1.99E-38	postive
IREB2	AC005632.3	0.532056556	1.96E-40	postive
DDX17	AC005632.3	0.567875296	5.28E-47	postive
PIK3CG	AC005632.3	0.562808578	5.03E-46	postive
JAK2	AC005632.3	0.6731915	6.97E-72	postive
TXK	AC005632.3	0.573825518	3.56E-48	postive
NFAT5	AC005632.3	0.767033502	8.46E-105	postive
MALT1	AC005632.3	0.516568828	7.91E-38	postive
PIK3R1	AC005632.3	0.583125415	4.71E-50	postive
PIK3CA	AC005632.3	0.672182369	1.35E-71	postive
RASGRP3	AC005632.3	0.566952806	7.98E-47	postive
PLXNC1	AC005632.3	0.504130548	7.86E-36	postive
GNRH1	AC005632.3	0.692574955	1.22E-77	postive
IL6ST	AC005632.3	0.576352944	1.12E-48	postive
ACVR2A	AC005632.3	0.534370375	7.79E-41	postive
ANGPTL1	AC005632.3	0.744143846	1.90E-95	postive
BMPR2	AC005632.3	0.64212736	1.60E-63	postive
CRLF3	AC005632.3	0.639473281	7.48E-63	postive
IL18R1	AC005632.3	0.528484047	8.05E-40	postive
NR2C2	AC005632.3	0.624619857	3.19E-59	postive
RORA	AC005632.3	0.788758654	9.99E-115	postive
SOS1	AC005632.3	0.599073889	2.03E-53	postive
SOS2	AC005632.3	0.505544161	4.71E-36	postive
BRAF	AC005632.3	0.70252249	8.97E-81	postive
PTPRC	AC005632.3	0.500016255	3.45E-35	postive
ITK	AC005632.3	0.541712142	3.97E-42	postive
CD28	AC005632.3	0.511464326	5.34E-37	postive
CBL	AC005632.3	0.573668143	3.83E-48	postive
CBLB	AC005632.3	0.723451665	8.14E-88	postive
PDK1	AC005632.3	0.622021036	1.31E-58	postive
TRAJ1	AC005632.3	0.674438827	3.06E-72	postive
TRAJ2	AC005632.3	0.689625911	9.83E-77	postive
TRAJ3	AC005632.3	0.726242951	8.36E-89	postive
TRAJ5	AC005632.3	0.728188448	1.68E-89	postive
TRAJ6	AC005632.3	0.719335042	2.22E-86	postive
TRAJ8	AC005632.3	0.656611232	2.69E-67	postive
TRAJ10	AC005632.3	0.721377747	4.34E-87	postive
TRAJ12	AC005632.3	0.623200665	6.92E-59	postive
TRAJ13	AC005632.3	0.691039963	3.63E-77	postive
TRAJ14	AC005632.3	0.688026316	3.01E-76	postive
TRAJ16	AC005632.3	0.737768854	5.09E-93	postive
TRAJ17	AC005632.3	0.684113531	4.52E-75	postive

TRAJ18	AC005632.3	0.691814315	2.10E-77	postive
TRAJ21	AC005632.3	0.734208394	1.08E-91	postive
TRAJ31	AC005632.3	0.757158318	1.23E-100	postive
TRAJ37	AC005632.3	0.717004192	1.41E-85	postive
TRAJ38	AC005632.3	0.802581989	1.12E-121	postive
TRAJ39	AC005632.3	0.754995522	9.42E-100	postive
LTB4R	AC135050.3	0.521034098	1.45E-38	postive
TRBV30	LINC00941	0.605289987	8.77E-55	postive
CREB1	OCIAD1-AS1	0.657409103	1.64E-67	postive
UBR1	OCIAD1-AS1	0.507293639	2.48E-36	postive
IL15	OCIAD1-AS1	0.504886114	5.98E-36	postive
CYLD	OCIAD1-AS1	0.529295618	5.85E-40	postive
DDX17	OCIAD1-AS1	0.604145441	1.57E-54	postive
NFAT5	OCIAD1-AS1	0.746710277	1.91E-96	postive
MALT1	OCIAD1-AS1	0.507892673	2.00E-36	postive
PIK3R1	OCIAD1-AS1	0.504625975	6.57E-36	postive
PIK3CA	OCIAD1-AS1	0.54454735	1.23E-42	postive
GNRH1	OCIAD1-AS1	0.70681623	3.62E-82	postive
IL6ST	OCIAD1-AS1	0.52811738	9.29E-40	postive
ACVR2A	OCIAD1-AS1	0.574076927	3.18E-48	postive
ANGPTL1	OCIAD1-AS1	0.638704055	1.17E-62	postive
BMPR2	OCIAD1-AS1	0.57984513	2.20E-49	postive
CRLF3	OCIAD1-AS1	0.518327964	4.06E-38	postive
NR2C1	OCIAD1-AS1	0.507533444	2.28E-36	postive
NR2C2	OCIAD1-AS1	0.620737324	2.63E-58	postive
RORA	OCIAD1-AS1	0.740920128	3.28E-94	postive
SOS1	OCIAD1-AS1	0.567918357	5.18E-47	postive
SOS2	OCIAD1-AS1	0.524241119	4.21E-39	postive
BRAF	OCIAD1-AS1	0.677287057	4.61E-73	postive
CBLB	OCIAD1-AS1	0.614207129	8.58E-57	postive
PDK1	OCIAD1-AS1	0.509379287	1.16E-36	postive
TRAJ1	OCIAD1-AS1	0.638320886	1.45E-62	postive
TRAJ2	OCIAD1-AS1	0.614260779	8.34E-57	postive
TRAJ3	OCIAD1-AS1	0.666170741	6.63E-70	postive
TRAJ5	OCIAD1-AS1	0.68940091	1.15E-76	postive
TRAJ6	OCIAD1-AS1	0.674320658	3.31E-72	postive
TRAJ8	OCIAD1-AS1	0.631023378	9.18E-61	postive
TRAJ10	OCIAD1-AS1	0.687118931	5.67E-76	postive
TRAJ12	OCIAD1-AS1	0.591824484	7.25E-52	postive
TRAJ13	OCIAD1-AS1	0.66917196	9.60E-71	postive
TRAJ14	OCIAD1-AS1	0.641264782	2.65E-63	postive
TRAJ16	OCIAD1-AS1	0.667163166	3.51E-70	postive
TRAJ17	OCIAD1-AS1	0.643521464	7.07E-64	postive
TRAJ18	OCIAD1-AS1	0.623778493	5.05E-59	postive
TRAJ21	OCIAD1-AS1	0.684470103	3.54E-75	postive
TRAJ31	OCIAD1-AS1	0.726658044	5.94E-89	postive
TRAJ37	OCIAD1-AS1	0.712133157	6.27E-84	postive
TRAJ38	OCIAD1-AS1	0.725993635	1.03E-88	postive
TRAJ39	OCIAD1-AS1	0.702002753	1.32E-80	postive
DDX17	AL162274.2	0.532195092	1.86E-40	postive
NFAT5	AL162274.2	0.542366063	3.04E-42	postive
GNRH1	AL162274.2	0.695358572	1.67E-78	postive
NR2C2	AL162274.2	0.576888092	8.71E-49	postive
RORA	AL162274.2	0.570579586	1.56E-47	postive
BRAF	AL162274.2	0.530329638	3.89E-40	postive
CREB1	AC073569.2	0.559361006	2.28E-45	postive
UBR1	AC073569.2	0.512403711	3.77E-37	postive
ZC3HAV1L	AC073569.2	0.578614873	3.91E-49	postive
LMBR1	AC073569.2	0.533200837	1.24E-40	postive
IREB2	AC073569.2	0.584710945	2.22E-50	postive
NFAT5	AC073569.2	0.577350186	7.03E-49	postive
PIK3CA	AC073569.2	0.578218585	4.70E-49	postive
BMPR2	AC073569.2	0.512606962	3.49E-37	postive
RORA	AC073569.2	0.513440361	2.56E-37	postive
SOS1	AC073569.2	0.524775363	3.42E-39	postive

BRAF	AC073569.2	0.555225158	1.36E-44	postive
CBL	AC073569.2	0.543298651	2.07E-42	postive
TRAJ3	AC073569.2	0.549499165	1.56E-43	postive
TRAJ5	AC073569.2	0.525825466	2.28E-39	postive
TRAJ6	AC073569.2	0.537018453	2.69E-41	postive
TRAJ8	AC073569.2	0.532700262	1.52E-40	postive
TRAJ10	AC073569.2	0.524180436	4.31E-39	postive
TRAJ13	AC073569.2	0.539199018	1.11E-41	postive
TRAJ14	AC073569.2	0.514420942	1.77E-37	postive
TRAJ16	AC073569.2	0.51643997	8.30E-38	postive
TRAJ17	AC073569.2	0.519074164	3.06E-38	postive
TRAJ21	AC073569.2	0.55076005	9.15E-44	postive
TRAJ31	AC073569.2	0.530876605	3.13E-40	postive
TRAJ37	AC073569.2	0.533911296	9.37E-41	postive
TRAJ38	AC073569.2	0.55747288	5.17E-45	postive
TRAJ39	AC073569.2	0.558817744	2.88E-45	postive
ELANE	MIR223HG	0.527930665	1.00E-39	postive
AZU1	MIR223HG	0.630667767	1.12E-60	postive
CREB1	AC115989.1	0.644476474	4.03E-64	postive
UBR1	AC115989.1	0.580289157	1.79E-49	postive
IREB2	AC115989.1	0.54697187	4.50E-43	postive
DDX17	AC115989.1	0.527970817	9.84E-40	postive
NFAT5	AC115989.1	0.719953402	1.36E-86	postive
AKT3	AC115989.1	0.59217822	6.10E-52	postive
IGHD4-4	AC115989.1	0.54528295	9.09E-43	postive
GNRH1	AC115989.1	0.654985605	7.30E-67	postive
RABEP1	AC115989.1	0.525787154	2.31E-39	postive
ACVR2A	AC115989.1	0.571658444	9.57E-48	postive
ANGPTL1	AC115989.1	0.577215803	7.48E-49	postive
BMPR1A	AC115989.1	0.518883141	3.29E-38	postive
BMPR2	AC115989.1	0.572400238	6.83E-48	postive
CRLF3	AC115989.1	0.582294832	6.98E-50	postive
NR2C2	AC115989.1	0.611397723	3.74E-56	postive
RORA	AC115989.1	0.621763999	1.51E-58	postive
SOS1	AC115989.1	0.586735008	8.47E-51	postive
SOS2	AC115989.1	0.500789674	2.62E-35	postive
BRAF	AC115989.1	0.733328226	2.28E-91	postive
TRAJ1	AC115989.1	0.698963726	1.23E-79	postive
TRAJ2	AC115989.1	0.726587845	6.30E-89	postive
TRAJ3	AC115989.1	0.761096951	2.84E-102	postive
TRAJ5	AC115989.1	0.674703164	2.57E-72	postive
TRAJ6	AC115989.1	0.742426824	8.70E-95	postive
TRAJ8	AC115989.1	0.824745455	4.68E-134	postive
TRAJ10	AC115989.1	0.667306495	3.20E-70	postive
TRAJ12	AC115989.1	0.621973638	1.35E-58	postive
TRAJ13	AC115989.1	0.763535936	2.66E-103	postive
TRAJ14	AC115989.1	0.747192267	1.24E-96	postive
TRAJ16	AC115989.1	0.69751637	3.52E-79	postive
TRAJ17	AC115989.1	0.678854269	1.61E-73	postive
TRAJ18	AC115989.1	0.559734272	1.93E-45	postive
TRAJ21	AC115989.1	0.740636113	4.20E-94	postive
TRAJ31	AC115989.1	0.717069806	1.34E-85	postive
TRAJ37	AC115989.1	0.74013887	6.49E-94	postive
TRAJ38	AC115989.1	0.737005518	9.84E-93	postive
TRAJ39	AC115989.1	0.767798192	3.95E-105	postive
PLXNA3	AL645608.8	0.598033126	3.41E-53	postive
CREB1	AC004477.3	0.665788094	8.46E-70	postive
UBR1	AC004477.3	0.50682864	2.95E-36	postive
ZC3HAV1	AC004477.3	0.562431448	5.93E-46	postive
MAPK8	AC004477.3	0.512925423	3.10E-37	postive
IREB2	AC004477.3	0.513958701	2.11E-37	postive
DDX17	AC004477.3	0.519708443	2.40E-38	postive
NFAT5	AC004477.3	0.689011911	1.51E-76	postive
PIK3CA	AC004477.3	0.600801047	8.53E-54	postive
GNRH1	AC004477.3	0.668492862	1.49E-70	postive

RABEP1	AC004477.3	0.517316414	5.96E-38	postive
ACVR2A	AC004477.3	0.537372667	2.33E-41	postive
ANGPTL1	AC004477.3	0.624807201	2.88E-59	postive
BMPR2	AC004477.3	0.615655006	3.99E-57	postive
CRLF3	AC004477.3	0.539944858	8.19E-42	postive
NR2C2	AC004477.3	0.624968658	2.63E-59	postive
RORA	AC004477.3	0.665932167	7.72E-70	postive
SOS1	AC004477.3	0.648934346	2.86E-65	postive
SOS2	AC004477.3	0.507216039	2.56E-36	postive
BRAF	AC004477.3	0.681447957	2.79E-74	postive
CBL	AC004477.3	0.560731735	1.25E-45	postive
CBLB	AC004477.3	0.621187899	2.06E-58	postive
TRAJ1	AC004477.3	0.601813494	5.12E-54	postive
TRAJ2	AC004477.3	0.557199169	5.82E-45	postive
TRAJ3	AC004477.3	0.619458154	5.24E-58	postive
TRAJ5	AC004477.3	0.659255757	5.22E-68	postive
TRAJ6	AC004477.3	0.64522776	2.59E-64	postive
TRAJ8	AC004477.3	0.626675685	1.03E-59	postive
TRAJ10	AC004477.3	0.630319169	1.36E-60	postive
TRAJ12	AC004477.3	0.587427315	6.08E-51	postive
TRAJ13	AC004477.3	0.653395025	1.93E-66	postive
TRAJ14	AC004477.3	0.622349574	1.10E-58	postive
TRAJ16	AC004477.3	0.653016641	2.43E-66	postive
TRAJ17	AC004477.3	0.628446327	3.87E-60	postive
TRAJ18	AC004477.3	0.577367546	6.97E-49	postive
TRAJ21	AC004477.3	0.656480401	2.91E-67	postive
TRAJ31	AC004477.3	0.70535195	1.09E-81	postive
TRAJ37	AC004477.3	0.654223752	1.16E-66	postive
TRAJ38	AC004477.3	0.6916076	2.43E-77	postive
TRAJ39	AC004477.3	0.661491792	1.29E-68	postive
DDX17	AL117379.1	0.565408172	1.59E-46	postive
NFAT5	AL117379.1	0.590292528	1.53E-51	postive
LTB4R2	AL117379.1	0.519688464	2.42E-38	postive
GNRH1	AL117379.1	0.741463362	2.03E-94	postive
ANGPTL1	AL117379.1	0.531239805	2.71E-40	postive
NR2C1	AL117379.1	0.522146329	9.44E-39	postive
NR2C2	AL117379.1	0.635586157	6.97E-62	postive
RORA	AL117379.1	0.550131731	1.19E-43	postive
BRAF	AL117379.1	0.656388773	3.08E-67	postive
CBLB	AL117379.1	0.511035931	6.26E-37	postive
TRAJ1	AL117379.1	0.560252923	1.54E-45	postive
TRAJ2	AL117379.1	0.565348827	1.63E-46	postive
TRAJ3	AL117379.1	0.62276139	8.79E-59	postive
TRAJ5	AL117379.1	0.558404907	3.45E-45	postive
TRAJ6	AL117379.1	0.576340273	1.12E-48	postive
TRAJ8	AL117379.1	0.601228585	6.88E-54	postive
TRAJ10	AL117379.1	0.531777994	2.19E-40	postive
TRAJ13	AL117379.1	0.577931432	5.37E-49	postive
TRAJ14	AL117379.1	0.569735668	2.29E-47	postive
TRAJ16	AL117379.1	0.555108757	1.43E-44	postive
TRAJ17	AL117379.1	0.537368124	2.33E-41	postive
TRAJ21	AL117379.1	0.585992969	1.21E-50	postive
TRAJ31	AL117379.1	0.57230723	7.13E-48	postive
TRAJ37	AL117379.1	0.569616412	2.41E-47	postive
TRAJ38	AL117379.1	0.600779611	8.63E-54	postive
TRAJ39	AL117379.1	0.602559775	3.51E-54	postive
PTX3	AP000695.1	0.646050569	1.59E-64	postive
SEMA6B	AP000695.1	0.579648768	2.41E-49	postive
SEMA7A	AP000695.1	0.723501768	7.82E-88	postive
GREM1	AP000695.1	0.576252996	1.17E-48	postive
TNFRSF8	AP000695.1	0.606218214	5.46E-55	postive
CREB1	AC138207.4	0.601257748	6.78E-54	postive
ZC3HAV1	AC138207.4	0.517447275	5.67E-38	postive
CYLD	AC138207.4	0.517319874	5.95E-38	postive
NFKBIZ	AC138207.4	0.530137888	4.20E-40	postive

DDX17	AC138207.4	0.567936533	5.14E-47	postive
JAK2	AC138207.4	0.551645509	6.28E-44	postive
NFAT5	AC138207.4	0.653839242	1.47E-66	postive
PIK3CA	AC138207.4	0.561751777	8.00E-46	postive
RASGRP3	AC138207.4	0.549256852	1.72E-43	postive
LTB4R2	AC138207.4	0.543114958	2.23E-42	postive
PLXNC1	AC138207.4	0.51238301	3.80E-37	postive
GNRH1	AC138207.4	0.636215437	4.86E-62	postive
ANGPTL1	AC138207.4	0.596294349	8.08E-53	postive
BMPR2	AC138207.4	0.559270954	2.37E-45	postive
CRLF3	AC138207.4	0.643733104	6.25E-64	postive
NR2C2	AC138207.4	0.617344892	1.62E-57	postive
RORA	AC138207.4	0.636901886	3.29E-62	postive
SOS1	AC138207.4	0.586999497	7.46E-51	postive
SOS2	AC138207.4	0.505585898	4.63E-36	postive
BRAF	AC138207.4	0.684057386	4.70E-75	postive
CBL	AC138207.4	0.594673606	1.80E-52	postive
CBLB	AC138207.4	0.665303354	1.15E-69	postive
TRAJ1	AC138207.4	0.625863483	1.61E-59	postive
TRAJ2	AC138207.4	0.644092509	5.06E-64	postive
TRAJ3	AC138207.4	0.661306096	1.45E-68	postive
TRAJ5	AC138207.4	0.657177731	1.89E-67	postive
TRAJ6	AC138207.4	0.635939147	5.70E-62	postive
TRAJ8	AC138207.4	0.645598224	2.08E-64	postive
TRAJ10	AC138207.4	0.603248901	2.48E-54	postive
TRAJ12	AC138207.4	0.551167204	7.70E-44	postive
TRAJ13	AC138207.4	0.644128845	4.95E-64	postive
TRAJ14	AC138207.4	0.642239644	1.50E-63	postive
TRAJ16	AC138207.4	0.65323638	2.13E-66	postive
TRAJ17	AC138207.4	0.604336352	1.43E-54	postive
TRAJ18	AC138207.4	0.574945184	2.13E-48	postive
TRAJ21	AC138207.4	0.660286606	2.74E-68	postive
TRAJ31	AC138207.4	0.65347108	1.84E-66	postive
TRAJ37	AC138207.4	0.636182829	4.96E-62	postive
TRAJ38	AC138207.4	0.6933691	6.95E-78	postive
TRAJ39	AC138207.4	0.677513969	3.96E-73	postive
CREB1	AC010226.1	0.692813619	1.03E-77	postive
UBR1	AC010226.1	0.579708742	2.35E-49	postive
IL15	AC010226.1	0.625749268	1.72E-59	postive
CYLD	AC010226.1	0.688828842	1.72E-76	postive
DDX17	AC010226.1	0.573149532	4.85E-48	postive
PIK3CG	AC010226.1	0.564488686	2.39E-46	postive
JAK2	AC010226.1	0.656058331	3.78E-67	postive
TXK	AC010226.1	0.518272918	4.15E-38	postive
NFAT5	AC010226.1	0.819844694	3.58E-131	postive
NFATC3	AC010226.1	0.558035322	4.05E-45	postive
PIK3R1	AC010226.1	0.625872462	1.60E-59	postive
PIK3CA	AC010226.1	0.577555038	6.39E-49	postive
RASGRP3	AC010226.1	0.543108905	2.24E-42	postive
PLXNC1	AC010226.1	0.510185167	8.58E-37	postive
GNRH1	AC010226.1	0.688291303	2.50E-76	postive
IL6ST	AC010226.1	0.595592139	1.14E-52	postive
IL7	AC010226.1	0.51764178	5.27E-38	postive
RABEP1	AC010226.1	0.557781611	4.52E-45	postive
ACVR2A	AC010226.1	0.564393952	2.49E-46	postive
ANGPTL1	AC010226.1	0.683717013	5.94E-75	postive
BMPR2	AC010226.1	0.611088805	4.40E-56	postive
CRLF3	AC010226.1	0.596978336	5.76E-53	postive
NR2C2	AC010226.1	0.611768518	3.09E-56	postive
RORA	AC010226.1	0.785619094	3.20E-113	postive
SOS1	AC010226.1	0.554842045	1.61E-44	postive
SOS2	AC010226.1	0.53674544	3.00E-41	postive
BRAF	AC010226.1	0.670522766	4.00E-71	postive
PTPRC	AC010226.1	0.51422117	1.91E-37	postive
ITK	AC010226.1	0.541296199	4.71E-42	postive

CD28	AC010226.1	0.560671967	1.28E-45	postive
CBL	AC010226.1	0.508638862	1.52E-36	postive
CBLB	AC010226.1	0.610447088	6.15E-56	postive
RASGRP1	AC010226.1	0.511525858	5.22E-37	postive
PDK1	AC010226.1	0.519255903	2.85E-38	postive
TRAJ1	AC010226.1	0.731391651	1.16E-90	postive
TRAJ2	AC010226.1	0.726480663	6.88E-89	postive
TRAJ3	AC010226.1	0.78847971	1.36E-114	postive
TRAJ5	AC010226.1	0.730596998	2.27E-90	postive
TRAJ6	AC010226.1	0.794152524	2.25E-117	postive
TRAJ8	AC010226.1	0.704043548	2.90E-81	postive
TRAJ10	AC010226.1	0.793514055	4.67E-117	postive
TRAJ12	AC010226.1	0.721659139	3.46E-87	postive
TRAJ13	AC010226.1	0.737602007	5.88E-93	postive
TRAJ14	AC010226.1	0.743570382	3.16E-95	postive
TRAJ16	AC010226.1	0.756880024	1.60E-100	postive
TRAJ17	AC010226.1	0.729777689	4.49E-90	postive
TRAJ18	AC010226.1	0.770227947	3.44E-106	postive
TRAJ21	AC010226.1	0.773094079	1.86E-107	postive
TRAJ31	AC010226.1	0.778977939	4.06E-110	postive
TRAJ37	AC010226.1	0.783984853	1.90E-112	postive
TRAJ38	AC010226.1	0.817906894	4.67E-130	postive
TRAJ39	AC010226.1	0.784590593	9.85E-113	postive
CREB1	LINC00861	0.65327263	2.08E-66	postive
CIITA	LINC00861	0.618482527	8.84E-58	postive
IL15	LINC00861	0.619510877	5.09E-58	postive
CYLD	LINC00861	0.72762249	2.69E-89	postive
IL7R	LINC00861	0.566147428	1.14E-46	postive
DDX17	LINC00861	0.612147575	2.53E-56	postive
CD40LG	LINC00861	0.538572836	1.43E-41	postive
TRIM22	LINC00861	0.512813966	3.23E-37	postive
PIK3CG	LINC00861	0.627851507	5.38E-60	postive
CCR4	LINC00861	0.538472419	1.49E-41	postive
JAK2	LINC00861	0.694246373	3.71E-78	postive
TXK	LINC00861	0.570370334	1.72E-47	postive
PPP3CC	LINC00861	0.527279405	1.29E-39	postive
NFAT5	LINC00861	0.761742322	1.52E-102	postive
NFATC2	LINC00861	0.610375302	6.38E-56	postive
PIK3R1	LINC00861	0.604063928	1.64E-54	postive
PIK3CA	LINC00861	0.546919674	4.60E-43	postive
RASGRP3	LINC00861	0.665183534	1.24E-69	postive
PRKCB	LINC00861	0.598251122	3.06E-53	postive
IGHD6-6	LINC00861	0.551013859	8.21E-44	postive
PLXNC1	LINC00861	0.624758421	2.95E-59	postive
GNRH1	LINC00861	0.653278229	2.07E-66	postive
IL6ST	LINC00861	0.548795247	2.09E-43	postive
RABEP1	LINC00861	0.520359617	1.87E-38	postive
TNFSF8	LINC00861	0.601204681	6.96E-54	postive
ACVR2A	LINC00861	0.5274482	1.21E-39	postive
ANGPTL1	LINC00861	0.613302525	1.38E-56	postive
BMPR2	LINC00861	0.577334848	7.08E-49	postive
CRLF3	LINC00861	0.634694325	1.16E-61	postive
IL18RAP	LINC00861	0.532316728	1.77E-40	postive
NR2C2	LINC00861	0.627353493	7.08E-60	postive
RORA	LINC00861	0.76755163	5.05E-105	postive
LCP2	LINC00861	0.514690604	1.60E-37	postive
SOS1	LINC00861	0.585761022	1.35E-50	postive
SOS2	LINC00861	0.512276911	3.95E-37	postive
BRAF	LINC00861	0.641222438	2.71E-63	postive
SH2D1A	LINC00861	0.570297605	1.77E-47	postive
CD3G	LINC00861	0.517161153	6.32E-38	postive
PTPRC	LINC00861	0.67066112	3.65E-71	postive
ITK	LINC00861	0.774680992	3.63E-108	postive
CD28	LINC00861	0.750068935	9.04E-98	postive
ICOS	LINC00861	0.641793606	1.94E-63	postive

CTLA4	LINC00861	0.536240343	3.68E-41	postive
CBL	LINC00861	0.563724119	3.35E-46	postive
CBLB	LINC00861	0.630497071	1.23E-60	postive
RASGRP1	LINC00861	0.539629027	9.31E-42	postive
TRAJ1	LINC00861	0.779239415	3.08E-110	postive
TRAJ2	LINC00861	0.802653286	1.03E-121	postive
TRAJ3	LINC00861	0.810577173	5.93E-126	postive
TRAJ5	LINC00861	0.742167862	1.09E-94	postive
TRAJ6	LINC00861	0.758412763	3.73E-101	postive
TRAJ8	LINC00861	0.709996612	3.24E-83	postive
TRAJ10	LINC00861	0.73544831	3.74E-92	postive
TRAJ12	LINC00861	0.681311213	3.07E-74	postive
TRAJ13	LINC00861	0.726185444	8.76E-89	postive
TRAJ14	LINC00861	0.723762185	6.33E-88	postive
TRAJ16	LINC00861	0.748063621	5.61E-97	postive
TRAJ17	LINC00861	0.69771231	3.05E-79	postive
TRAJ18	LINC00861	0.748624517	3.37E-97	postive
TRAJ21	LINC00861	0.735265124	4.38E-92	postive
TRAJ31	LINC00861	0.748582128	3.51E-97	postive
TRAJ37	LINC00861	0.772322094	4.10E-107	postive
TRAJ38	LINC00861	0.786884628	7.97E-114	postive
TRAJ39	LINC00861	0.750687863	5.12E-98	postive
TRBC1	LINC00861	0.569292502	2.79E-47	postive
RFXAP	AC073655.2	0.500654771	2.75E-35	postive
DDX17	AC073655.2	0.617223385	1.73E-57	postive
NFAT5	AC073655.2	0.613449114	1.28E-56	postive
GNRH1	AC073655.2	0.670004327	5.60E-71	postive
ANGPTL1	AC073655.2	0.500872377	2.54E-35	postive
NR2C1	AC073655.2	0.554968559	1.52E-44	postive
NR2C2	AC073655.2	0.659607457	4.19E-68	postive
RORA	AC073655.2	0.569750448	2.27E-47	postive
BRAF	AC073655.2	0.621344835	1.89E-58	postive
CBLB	AC073655.2	0.508401648	1.66E-36	postive
TRAJ1	AC073655.2	0.5740406	3.23E-48	postive
TRAJ2	AC073655.2	0.539089645	1.16E-41	postive
TRAJ3	AC073655.2	0.584033259	3.07E-50	postive
TRAJ5	AC073655.2	0.551671331	6.22E-44	postive
TRAJ6	AC073655.2	0.580275819	1.80E-49	postive
TRAJ8	AC073655.2	0.546058485	6.58E-43	postive
TRAJ10	AC073655.2	0.547840588	3.13E-43	postive
TRAJ12	AC073655.2	0.57034946	1.73E-47	postive
TRAJ13	AC073655.2	0.544392268	1.32E-42	postive
TRAJ14	AC073655.2	0.569278508	2.81E-47	postive
TRAJ16	AC073655.2	0.58424187	2.78E-50	postive
TRAJ17	AC073655.2	0.532292508	1.79E-40	postive
TRAJ18	AC073655.2	0.560300068	1.51E-45	postive
TRAJ21	AC073655.2	0.577426972	6.79E-49	postive
TRAJ31	AC073655.2	0.573314794	4.50E-48	postive
TRAJ37	AC073655.2	0.571192753	1.18E-47	postive
TRAJ38	AC073655.2	0.613963062	9.75E-57	postive
TRAJ39	AC073655.2	0.584064593	3.02E-50	postive
CREB1	AC009054.2	0.659544065	4.36E-68	postive
UBR1	AC009054.2	0.536403468	3.44E-41	postive
IL15	AC009054.2	0.514699312	1.60E-37	postive
CYLD	AC009054.2	0.553310424	3.09E-44	postive
DDX17	AC009054.2	0.520930378	1.51E-38	postive
JAK2	AC009054.2	0.530608732	3.48E-40	postive
NFAT5	AC009054.2	0.836855676	1.39E-141	postive
NFATC3	AC009054.2	0.509073826	1.29E-36	postive
PIK3R1	AC009054.2	0.52359247	5.41E-39	postive
PIK3CA	AC009054.2	0.556448869	8.05E-45	postive
AKT3	AC009054.2	0.527807746	1.05E-39	postive
GNRH1	AC009054.2	0.668277325	1.71E-70	postive
IL6ST	AC009054.2	0.538298826	1.60E-41	postive
ACVR2A	AC009054.2	0.53362178	1.05E-40	postive

ANGPTL1	AC009054.2	0.639849009	6.02E-63	postive
BMPR2	AC009054.2	0.576427675	1.08E-48	postive
CRLF3	AC009054.2	0.588436039	3.74E-51	postive
NR2C2	AC009054.2	0.545260317	9.18E-43	postive
RORA	AC009054.2	0.759484817	1.34E-101	postive
SOS1	AC009054.2	0.511151854	6.00E-37	postive
BRAF	AC009054.2	0.658626327	7.72E-68	postive
CBL	AC009054.2	0.501704136	1.89E-35	postive
CBLB	AC009054.2	0.569624522	2.40E-47	postive
TRAJ1	AC009054.2	0.689879931	8.22E-77	postive
TRAJ2	AC009054.2	0.715558874	4.38E-85	postive
TRAJ3	AC009054.2	0.759044523	2.04E-101	postive
TRAJ5	AC009054.2	0.70602261	6.58E-82	postive
TRAJ6	AC009054.2	0.774367222	5.02E-108	postive
TRAJ8	AC009054.2	0.731253206	1.31E-90	postive
TRAJ10	AC009054.2	0.769039607	1.14E-105	postive
TRAJ12	AC009054.2	0.628615825	3.52E-60	postive
TRAJ13	AC009054.2	0.765214566	5.12E-104	postive
TRAJ14	AC009054.2	0.722565152	1.67E-87	postive
TRAJ16	AC009054.2	0.742374169	9.12E-95	postive
TRAJ17	AC009054.2	0.699123139	1.09E-79	postive
TRAJ18	AC009054.2	0.695389473	1.64E-78	postive
TRAJ21	AC009054.2	0.779083343	3.63E-110	postive
TRAJ31	AC009054.2	0.770604755	2.35E-106	postive
TRAJ37	AC009054.2	0.808391739	9.17E-125	postive
TRAJ38	AC009054.2	0.787633029	3.49E-114	postive
TRAJ39	AC009054.2	0.792247295	1.98E-116	postive
CREB1	ZKSCAN2-DT	0.566142685	1.15E-46	postive
RFXAP	ZKSCAN2-DT	0.56451302	2.37E-46	postive
CYLD	ZKSCAN2-DT	0.521567504	1.18E-38	postive
DDX17	ZKSCAN2-DT	0.690507717	5.29E-77	postive
NFAT5	ZKSCAN2-DT	0.691841441	2.06E-77	postive
LTB4R2	ZKSCAN2-DT	0.580818888	1.40E-49	postive
GNRH1	ZKSCAN2-DT	0.754430626	1.60E-99	postive
ACVR2A	ZKSCAN2-DT	0.548352795	2.52E-43	postive
ANGPTL1	ZKSCAN2-DT	0.559058105	2.60E-45	postive
NR2C1	ZKSCAN2-DT	0.530843465	3.17E-40	postive
NR2C2	ZKSCAN2-DT	0.707954739	1.53E-82	postive
RORA	ZKSCAN2-DT	0.659830384	3.65E-68	postive
SOS1	ZKSCAN2-DT	0.553706414	2.61E-44	postive
BRAF	ZKSCAN2-DT	0.671155313	2.65E-71	postive
CBLB	ZKSCAN2-DT	0.559903884	1.80E-45	postive
TRAJ1	ZKSCAN2-DT	0.659403301	4.76E-68	postive
TRAJ2	ZKSCAN2-DT	0.643913562	5.62E-64	postive
TRAJ3	ZKSCAN2-DT	0.692970622	9.23E-78	postive
TRAJ5	ZKSCAN2-DT	0.657958144	1.17E-67	postive
TRAJ6	ZKSCAN2-DT	0.662646958	6.22E-69	postive
TRAJ8	ZKSCAN2-DT	0.67347222	5.80E-72	postive
TRAJ10	ZKSCAN2-DT	0.626552162	1.10E-59	postive
TRAJ12	ZKSCAN2-DT	0.592423062	5.42E-52	postive
TRAJ13	ZKSCAN2-DT	0.658211299	9.99E-68	postive
TRAJ14	ZKSCAN2-DT	0.654703387	8.68E-67	postive
TRAJ16	ZKSCAN2-DT	0.654526069	9.67E-67	postive
TRAJ17	ZKSCAN2-DT	0.632019513	5.25E-61	postive
TRAJ18	ZKSCAN2-DT	0.577655073	6.10E-49	postive
TRAJ21	ZKSCAN2-DT	0.665883772	7.96E-70	postive
TRAJ31	ZKSCAN2-DT	0.666872448	4.23E-70	postive
TRAJ37	ZKSCAN2-DT	0.667119269	3.61E-70	postive
TRAJ38	ZKSCAN2-DT	0.695777901	1.24E-78	postive
TRAJ39	ZKSCAN2-DT	0.686652396	7.84E-76	postive
DDX17	AC090589.3	0.657734339	1.34E-67	postive
IRF9	AC090589.3	0.557661564	4.76E-45	postive
NFAT5	AC090589.3	0.558463584	3.36E-45	postive
LTB4R2	AC090589.3	0.601125818	7.25E-54	postive
GNRH1	AC090589.3	0.701084718	2.59E-80	postive

NR2C1	AC090589.3	0.587717354	5.29E-51	postive
NR2C2	AC090589.3	0.683444054	7.16E-75	postive
RORA	AC090589.3	0.526000385	2.13E-39	postive
BRAF	AC090589.3	0.506746323	3.04E-36	postive
CREB1	XIST	0.516619589	7.76E-38	postive
CYLD	XIST	0.543669545	1.77E-42	postive
NFAT5	XIST	0.654773829	8.31E-67	postive
IL6ST	XIST	0.515301069	1.27E-37	postive
RORA	XIST	0.659628378	4.14E-68	postive
BRAF	XIST	0.531567689	2.38E-40	postive
TRAJ1	XIST	0.583770609	3.47E-50	postive
TRAJ2	XIST	0.665961467	7.58E-70	postive
TRAJ3	XIST	0.644055633	5.17E-64	postive
TRAJ5	XIST	0.597444091	4.57E-53	postive
TRAJ6	XIST	0.618998226	6.71E-58	postive
TRAJ8	XIST	0.662097292	8.80E-69	postive
TRAJ10	XIST	0.616695922	2.29E-57	postive
TRAJ12	XIST	0.545464869	8.43E-43	postive
TRAJ13	XIST	0.625511984	1.95E-59	postive
TRAJ14	XIST	0.608527094	1.66E-55	postive
TRAJ16	XIST	0.678725641	1.76E-73	postive
TRAJ17	XIST	0.631794465	5.96E-61	postive
TRAJ18	XIST	0.605546189	7.70E-55	postive
TRAJ21	XIST	0.63066175	1.12E-60	postive
TRAJ31	XIST	0.646514348	1.21E-64	postive
TRAJ37	XIST	0.678635594	1.87E-73	postive
TRAJ38	XIST	0.659612352	4.18E-68	postive
TRAJ39	XIST	0.669638304	7.10E-71	postive
CREB1	PSPC1-AS2	0.744875985	9.89E-96	postive
RFXAP	PSPC1-AS2	0.603573812	2.10E-54	postive
UBR1	PSPC1-AS2	0.621666176	1.59E-58	postive
ZC3HAV1	PSPC1-AS2	0.559228329	2.41E-45	postive
ZC3HAV1L	PSPC1-AS2	0.502458315	1.44E-35	postive
IL15	PSPC1-AS2	0.571513609	1.02E-47	postive
CYLD	PSPC1-AS2	0.602417182	3.78E-54	postive
EIF2AK2	PSPC1-AS2	0.507959911	1.95E-36	postive
MAPK8	PSPC1-AS2	0.553423935	2.95E-44	postive
LMBR1	PSPC1-AS2	0.514939355	1.46E-37	postive
IREB2	PSPC1-AS2	0.588703839	3.29E-51	postive
DDX17	PSPC1-AS2	0.701545066	1.85E-80	postive
JAK2	PSPC1-AS2	0.60236977	3.87E-54	postive
TXK	PSPC1-AS2	0.573214972	4.71E-48	postive
NFAT5	PSPC1-AS2	0.856954882	1.49E-155	postive
MALT1	PSPC1-AS2	0.523634336	5.32E-39	postive
PIK3R1	PSPC1-AS2	0.597072911	5.49E-53	postive
PIK3CA	PSPC1-AS2	0.631078365	8.90E-61	postive
AKT3	PSPC1-AS2	0.549556147	1.52E-43	postive
RASGRP3	PSPC1-AS2	0.517872384	4.83E-38	postive
IGHD4-4	PSPC1-AS2	0.527331568	1.26E-39	postive
IGHD6-6	PSPC1-AS2	0.556441509	8.07E-45	postive
LTB4R2	PSPC1-AS2	0.516998115	6.72E-38	postive
GNRH1	PSPC1-AS2	0.816962281	1.62E-129	postive
IL6ST	PSPC1-AS2	0.598604115	2.56E-53	postive
RABEP1	PSPC1-AS2	0.564315098	2.58E-46	postive
ACVR2A	PSPC1-AS2	0.654074151	1.28E-66	postive
ANGPTL1	PSPC1-AS2	0.720206138	1.11E-86	postive
BMPR1A	PSPC1-AS2	0.536159698	3.80E-41	postive
BMPR2	PSPC1-AS2	0.676895213	5.99E-73	postive
CRLF3	PSPC1-AS2	0.649843038	1.66E-65	postive
NR2C1	PSPC1-AS2	0.573168114	4.81E-48	postive
NR2C2	PSPC1-AS2	0.759187441	1.78E-101	postive
RORA	PSPC1-AS2	0.811731543	1.38E-126	postive
SOS1	PSPC1-AS2	0.675988322	1.10E-72	postive
SOS2	PSPC1-AS2	0.579267649	2.88E-49	postive
BRAF	PSPC1-AS2	0.796130434	2.29E-118	postive

ITK	PSPC1-AS2	0.515052709	1.40E-37	postive
CBL	PSPC1-AS2	0.598914591	2.20E-53	postive
CBLB	PSPC1-AS2	0.673380039	6.16E-72	postive
RASGRP1	PSPC1-AS2	0.501288627	2.19E-35	postive
PDK1	PSPC1-AS2	0.567175647	7.23E-47	postive
TRAJ1	PSPC1-AS2	0.793495158	4.77E-117	postive
TRAJ2	PSPC1-AS2	0.782707958	7.57E-112	postive
TRAJ3	PSPC1-AS2	0.844566549	1.04E-146	postive
TRAJ5	PSPC1-AS2	0.796192396	2.13E-118	postive
TRAJ6	PSPC1-AS2	0.824693856	5.02E-134	postive
TRAJ8	PSPC1-AS2	0.818718956	1.60E-130	postive
TRAJ10	PSPC1-AS2	0.802031791	2.17E-121	postive
TRAJ12	PSPC1-AS2	0.719847009	1.48E-86	postive
TRAJ13	PSPC1-AS2	0.81325999	1.96E-127	postive
TRAJ14	PSPC1-AS2	0.799347908	5.30E-120	postive
TRAJ16	PSPC1-AS2	0.819924686	3.21E-131	postive
TRAJ17	PSPC1-AS2	0.788671266	1.10E-114	postive
TRAJ18	PSPC1-AS2	0.742487553	8.25E-95	postive
TRAJ21	PSPC1-AS2	0.830864244	8.73E-138	postive
TRAJ31	PSPC1-AS2	0.834946955	2.34E-140	postive
TRAJ37	PSPC1-AS2	0.836673611	1.82E-141	postive
TRAJ38	PSPC1-AS2	0.859624301	1.44E-157	postive
TRAJ39	PSPC1-AS2	0.855124871	3.41E-154	postive
CREB1	RPS6KA2-IT1	0.596988199	5.73E-53	postive
NFAT5	RPS6KA2-IT1	0.530368616	3.83E-40	postive
PIK3CA	RPS6KA2-IT1	0.519602366	2.50E-38	postive
ANGPTL1	RPS6KA2-IT1	0.557909979	4.28E-45	postive
BMPR2	RPS6KA2-IT1	0.550325875	1.10E-43	postive
NR2C2	RPS6KA2-IT1	0.58441671	2.56E-50	postive
RORA	RPS6KA2-IT1	0.525817412	2.28E-39	postive
SOS1	RPS6KA2-IT1	0.599474518	1.66E-53	postive
BRAF	RPS6KA2-IT1	0.660183387	2.92E-68	postive
CBLB	RPS6KA2-IT1	0.531470882	2.48E-40	postive
TRAJ1	RPS6KA2-IT1	0.51884612	3.34E-38	postive
TRAJ2	RPS6KA2-IT1	0.520278527	1.93E-38	postive
TRAJ3	RPS6KA2-IT1	0.591845094	7.18E-52	postive
TRAJ5	RPS6KA2-IT1	0.544040018	1.52E-42	postive
TRAJ6	RPS6KA2-IT1	0.551282174	7.33E-44	postive
TRAJ8	RPS6KA2-IT1	0.572852711	5.56E-48	postive
TRAJ10	RPS6KA2-IT1	0.524895755	3.27E-39	postive
TRAJ13	RPS6KA2-IT1	0.551905597	5.63E-44	postive
TRAJ14	RPS6KA2-IT1	0.554754446	1.67E-44	postive
TRAJ16	RPS6KA2-IT1	0.543552576	1.86E-42	postive
TRAJ17	RPS6KA2-IT1	0.546546263	5.37E-43	postive
TRAJ21	RPS6KA2-IT1	0.558264145	3.67E-45	postive
TRAJ31	RPS6KA2-IT1	0.570505073	1.62E-47	postive
TRAJ37	RPS6KA2-IT1	0.525563779	2.52E-39	postive
TRAJ38	RPS6KA2-IT1	0.596980264	5.75E-53	postive
TRAJ39	RPS6KA2-IT1	0.568707259	3.63E-47	postive
AGER	AL590226.1	0.773817259	8.85E-108	postive
CCL14	AL590226.1	0.574639315	2.45E-48	postive
SEMA3G	AL590226.1	0.666870595	4.23E-70	postive
EDNRB	AL590226.1	0.648918622	2.88E-65	postive
VEGFD	AL590226.1	0.676594487	7.33E-73	postive
ACVRL1	AL590226.1	0.613084114	1.55E-56	postive
CALCRL	AL590226.1	0.562241166	6.45E-46	postive
IL3RA	AL590226.1	0.517671332	5.21E-38	postive
S1PR1	AL590226.1	0.66718947	3.45E-70	postive
TEK	AL590226.1	0.651259817	7.05E-66	postive
TIE1	AL590226.1	0.508511641	1.59E-36	postive
VIPR1	AL590226.1	0.61852207	8.66E-58	postive
THBS1	LINC01614	0.517290472	6.02E-38	postive
NOX4	LINC01614	0.69755507	3.42E-79	postive
VCAM1	LINC01614	0.558929063	2.75E-45	postive
TNFSF4	LINC01614	0.68925548	1.27E-76	postive

FGF1	LINC01614	0.510966437	6.43E-37	postive
GREM1	LINC01614	0.58662376	8.93E-51	postive
INHBA	LINC01614	0.679794411	8.56E-74	postive
ANGPTL2	LINC01614	0.54192781	3.63E-42	postive
CREB1	AC053513.2	0.815542289	1.03E-128	postive
RFXAP	AC053513.2	0.539571175	9.53E-42	postive
UBR1	AC053513.2	0.677049667	5.41E-73	postive
ZC3HAV1	AC053513.2	0.571626266	9.72E-48	postive
ZC3HAV1L	AC053513.2	0.541712736	3.97E-42	postive
IL15	AC053513.2	0.58129219	1.12E-49	postive
CYLD	AC053513.2	0.62419315	4.03E-59	postive
EIF2AK2	AC053513.2	0.538044771	1.77E-41	postive
MAPK8	AC053513.2	0.587177541	6.85E-51	postive
TLR1	AC053513.2	0.513037055	2.98E-37	postive
NFKBIZ	AC053513.2	0.513104477	2.90E-37	postive
LMBR1	AC053513.2	0.580585775	1.56E-49	postive
LIMS1	AC053513.2	0.518811877	3.38E-38	postive
IREB2	AC053513.2	0.634452422	1.33E-61	postive
DDX17	AC053513.2	0.655401201	5.66E-67	postive
PIK3CG	AC053513.2	0.541379886	4.55E-42	postive
JAK2	AC053513.2	0.67345615	5.86E-72	postive
TXK	AC053513.2	0.602785403	3.14E-54	postive
NFAT5	AC053513.2	0.872769354	3.87E-168	postive
MALT1	AC053513.2	0.553016685	3.51E-44	postive
PIK3R1	AC053513.2	0.631281137	7.95E-61	postive
PIK3CA	AC053513.2	0.699702691	7.15E-80	postive
PIK3CB	AC053513.2	0.523677859	5.23E-39	postive
AKT3	AC053513.2	0.551978037	5.46E-44	postive
RASGRP3	AC053513.2	0.540384433	6.84E-42	postive
GNRH1	AC053513.2	0.800344239	1.63E-120	postive
IL6ST	AC053513.2	0.612700372	1.89E-56	postive
RABEP1	AC053513.2	0.608226609	1.94E-55	postive
ACVR2A	AC053513.2	0.646818577	1.01E-64	postive
ANGPTL1	AC053513.2	0.775589552	1.42E-108	postive
BMPR1A	AC053513.2	0.568227373	4.51E-47	postive
BMPR2	AC053513.2	0.740117741	6.62E-94	postive
CRLF3	AC053513.2	0.671054817	2.83E-71	postive
NR1D2	AC053513.2	0.526479515	1.76E-39	postive
NR2C1	AC053513.2	0.539105568	1.15E-41	postive
NR2C2	AC053513.2	0.741496239	1.98E-94	postive
RORA	AC053513.2	0.834147321	7.55E-140	postive
SOS1	AC053513.2	0.706534536	4.48E-82	postive
SOS2	AC053513.2	0.595518023	1.19E-52	postive
BRAF	AC053513.2	0.793974715	2.75E-117	postive
TEC	AC053513.2	0.511846967	4.63E-37	postive
CBL	AC053513.2	0.630617868	1.15E-60	postive
CBLB	AC053513.2	0.76178098	1.47E-102	postive
PDK1	AC053513.2	0.605677652	7.20E-55	postive
TRAJ1	AC053513.2	0.726610375	6.18E-89	postive
TRAJ2	AC053513.2	0.742785334	6.34E-95	postive
TRAJ3	AC053513.2	0.777096518	2.94E-109	postive
TRAJ5	AC053513.2	0.74998203	9.78E-98	postive
TRAJ6	AC053513.2	0.791318536	5.66E-116	postive
TRAJ8	AC053513.2	0.727936547	2.07E-89	postive
TRAJ10	AC053513.2	0.772331294	4.06E-107	postive
TRAJ12	AC053513.2	0.681619595	2.49E-74	postive
TRAJ13	AC053513.2	0.751222079	3.14E-98	postive
TRAJ14	AC053513.2	0.736535475	1.47E-92	postive
TRAJ16	AC053513.2	0.770563207	2.45E-106	postive
TRAJ17	AC053513.2	0.707082481	2.96E-82	postive
TRAJ18	AC053513.2	0.708255748	1.22E-82	postive
TRAJ21	AC053513.2	0.772190964	4.69E-107	postive
TRAJ31	AC053513.2	0.797921258	2.84E-119	postive
TRAJ37	AC053513.2	0.787710168	3.20E-114	postive
TRAJ38	AC053513.2	0.848845452	1.13E-149	postive

TRAJ39	AC053513.2	0.791670031	3.80E-116	postive
CREB1	AL353804.1	0.795802147	3.35E-118	postive
RFXAP	AL353804.1	0.55589261	1.02E-44	postive
UBR1	AL353804.1	0.683378044	7.49E-75	postive
ZC3HAV1	AL353804.1	0.564418593	2.47E-46	postive
IL15	AL353804.1	0.54957977	1.51E-43	postive
CYLD	AL353804.1	0.692865835	9.95E-78	postive
EIF2AK2	AL353804.1	0.54981496	1.36E-43	postive
MAPK8	AL353804.1	0.553624087	2.71E-44	postive
LMBR1	AL353804.1	0.551527108	6.61E-44	postive
IREB2	AL353804.1	0.616312265	2.81E-57	postive
DDX17	AL353804.1	0.637829764	1.93E-62	postive
PIK3CG	AL353804.1	0.570708206	1.47E-47	postive
JAK2	AL353804.1	0.646130213	1.52E-64	postive
TXK	AL353804.1	0.584565385	2.38E-50	postive
NFAT5	AL353804.1	0.927089906	2.69E-229	postive
NFATC3	AL353804.1	0.600192937	1.16E-53	postive
MALT1	AL353804.1	0.548513196	2.36E-43	postive
PIK3R1	AL353804.1	0.689911835	8.04E-77	postive
PIK3CA	AL353804.1	0.691440931	2.73E-77	postive
PIK3CB	AL353804.1	0.503813504	8.82E-36	postive
AKT3	AL353804.1	0.532283121	1.79E-40	postive
RASGRP3	AL353804.1	0.534469246	7.49E-41	postive
IGHD6-6	AL353804.1	0.560330547	1.49E-45	postive
PLXNC1	AL353804.1	0.518870277	3.31E-38	postive
GNRH1	AL353804.1	0.720038795	1.27E-86	postive
IL6ST	AL353804.1	0.69932516	9.43E-80	postive
RABEP1	AL353804.1	0.570800229	1.41E-47	postive
ACVR2A	AL353804.1	0.658257037	9.71E-68	postive
ANGPTL1	AL353804.1	0.731450655	1.11E-90	postive
BMPR1A	AL353804.1	0.574217036	2.98E-48	postive
BMPR2	AL353804.1	0.744765207	1.09E-95	postive
CRLF3	AL353804.1	0.652519325	3.29E-66	postive
NR1D2	AL353804.1	0.546314336	5.92E-43	postive
NR2C2	AL353804.1	0.704291743	2.41E-81	postive
NR3C1	AL353804.1	0.50956416	1.08E-36	postive
RORA	AL353804.1	0.903754548	1.48E-198	postive
SOS1	AL353804.1	0.680921019	4.00E-74	postive
SOS2	AL353804.1	0.596772928	6.37E-53	postive
BRAF	AL353804.1	0.811550981	1.73E-126	postive
ITK	AL353804.1	0.535271663	5.43E-41	postive
CD28	AL353804.1	0.543045191	2.29E-42	postive
CBL	AL353804.1	0.574022002	3.26E-48	postive
CBLB	AL353804.1	0.68474062	2.94E-75	postive
RASGRP1	AL353804.1	0.529395414	5.62E-40	postive
PDK1	AL353804.1	0.54360417	1.82E-42	postive
TRAJ1	AL353804.1	0.801228938	5.68E-121	postive
TRAJ2	AL353804.1	0.8385131	1.16E-142	postive
TRAJ3	AL353804.1	0.862092851	1.81E-159	postive
TRAJ5	AL353804.1	0.839899247	1.42E-143	postive
TRAJ6	AL353804.1	0.869210468	3.65E-165	postive
TRAJ8	AL353804.1	0.851330654	1.95E-151	postive
TRAJ10	AL353804.1	0.851275893	2.13E-151	postive
TRAJ12	AL353804.1	0.787386027	4.58E-114	postive
TRAJ13	AL353804.1	0.860690451	2.20E-158	postive
TRAJ14	AL353804.1	0.843045265	1.13E-145	postive
TRAJ16	AL353804.1	0.87171605	3.00E-167	postive
TRAJ17	AL353804.1	0.822048076	1.85E-132	postive
TRAJ18	AL353804.1	0.825023566	3.19E-134	postive
TRAJ21	AL353804.1	0.883955495	4.17E-178	postive
TRAJ31	AL353804.1	0.897831113	5.30E-192	postive
TRAJ37	AL353804.1	0.907765806	3.05E-203	postive
TRAJ38	AL353804.1	0.925158278	2.20E-226	postive
TRAJ39	AL353804.1	0.908363169	5.86E-204	postive
CREB1	ARHGEF38-IT1	0.714279675	1.19E-84	postive

PSMD6	ARHGEF38-IT1	0.529270584	5.91E-40	postive
UBR1	ARHGEF38-IT1	0.595392084	1.26E-52	postive
ZC3HAV1	ARHGEF38-IT1	0.523552567	5.49E-39	postive
IL15	ARHGEF38-IT1	0.52577249	2.32E-39	postive
CYLD	ARHGEF38-IT1	0.512642707	3.45E-37	postive
EIF2AK2	ARHGEF38-IT1	0.571554121	1.00E-47	postive
LMBR1	ARHGEF38-IT1	0.544338479	1.34E-42	postive
IREB2	ARHGEF38-IT1	0.566404692	1.02E-46	postive
DDX17	ARHGEF38-IT1	0.548362436	2.51E-43	postive
JAK2	ARHGEF38-IT1	0.604052106	1.65E-54	postive
TXK	ARHGEF38-IT1	0.519157256	2.96E-38	postive
NFAT5	ARHGEF38-IT1	0.726178428	8.81E-89	postive
PIK3R1	ARHGEF38-IT1	0.550719545	9.30E-44	postive
PIK3CA	ARHGEF38-IT1	0.62509463	2.46E-59	postive
PIK3CB	ARHGEF38-IT1	0.525984179	2.14E-39	postive
RASGRP3	ARHGEF38-IT1	0.531904798	2.08E-40	postive
PLXNC1	ARHGEF38-IT1	0.522484141	8.29E-39	postive
GNRH1	ARHGEF38-IT1	0.681459548	2.77E-74	postive
IL6ST	ARHGEF38-IT1	0.509612151	1.06E-36	postive
RABEP1	ARHGEF38-IT1	0.507463018	2.34E-36	postive
ACVR2A	ARHGEF38-IT1	0.60609679	5.81E-55	postive
ANGPTL1	ARHGEF38-IT1	0.674435792	3.07E-72	postive
BMPR2	ARHGEF38-IT1	0.638894028	1.05E-62	postive
CRLF3	ARHGEF38-IT1	0.608470725	1.71E-55	postive
NR2C2	ARHGEF38-IT1	0.69047261	5.42E-77	postive
RORA	ARHGEF38-IT1	0.689344978	1.20E-76	postive
SOS1	ARHGEF38-IT1	0.680273217	6.20E-74	postive
SOS2	ARHGEF38-IT1	0.558276824	3.65E-45	postive
BRAF	ARHGEF38-IT1	0.759820984	9.71E-102	postive
TEC	ARHGEF38-IT1	0.514552984	1.69E-37	postive
CBL	ARHGEF38-IT1	0.532642228	1.55E-40	postive
CBLB	ARHGEF38-IT1	0.665531249	9.97E-70	postive
RASGRP1	ARHGEF38-IT1	0.52024459	1.96E-38	postive
PDK1	ARHGEF38-IT1	0.520147347	2.03E-38	postive
TRAJ1	ARHGEF38-IT1	0.801561875	3.82E-121	postive
TRAJ2	ARHGEF38-IT1	0.629869537	1.75E-60	postive
TRAJ3	ARHGEF38-IT1	0.737052225	9.45E-93	postive
TRAJ5	ARHGEF38-IT1	0.855480745	1.86E-154	postive
TRAJ6	ARHGEF38-IT1	0.790866282	9.42E-116	postive
TRAJ8	ARHGEF38-IT1	0.799775844	3.20E-120	postive
TRAJ10	ARHGEF38-IT1	0.753192725	5.07E-99	postive
TRAJ12	ARHGEF38-IT1	0.812232116	7.28E-127	postive
TRAJ13	ARHGEF38-IT1	0.798748964	1.07E-119	postive
TRAJ14	ARHGEF38-IT1	0.819946076	3.12E-131	postive
TRAJ16	ARHGEF38-IT1	0.791417119	5.06E-116	postive
TRAJ17	ARHGEF38-IT1	0.786503591	1.21E-113	postive
TRAJ18	ARHGEF38-IT1	0.714739761	8.30E-85	postive
TRAJ21	ARHGEF38-IT1	0.814289299	5.22E-128	postive
TRAJ31	ARHGEF38-IT1	0.834946519	2.34E-140	postive
TRAJ37	ARHGEF38-IT1	0.769461815	7.45E-106	postive
TRAJ38	ARHGEF38-IT1	0.84011463	1.02E-143	postive
TRAJ39	ARHGEF38-IT1	0.833422254	2.18E-139	postive
DDX17	AC004908.1	0.615838987	3.62E-57	postive
GNRH1	AC004908.1	0.650234492	1.31E-65	postive
NR2C1	AC004908.1	0.504443555	7.02E-36	postive
NR2C2	AC004908.1	0.586629387	8.91E-51	postive
RORA	AC004908.1	0.524035487	4.56E-39	postive
CREB1	AL049840.4	0.734950912	5.73E-92	postive
UBR1	AL049840.4	0.620169382	3.57E-58	postive
ZC3HAV1	AL049840.4	0.647764728	5.75E-65	postive
ZC3HAV1L	AL049840.4	0.566266675	1.08E-46	postive
IL15	AL049840.4	0.535503533	4.95E-41	postive
CYLD	AL049840.4	0.593010614	4.06E-52	postive
EIF2AK2	AL049840.4	0.512660323	3.42E-37	postive
TRAF3	AL049840.4	0.547066674	4.32E-43	postive

LMBR1	AL049840.4	0.555358009	1.29E-44	postive
IREB2	AL049840.4	0.562896188	4.83E-46	postive
DDX17	AL049840.4	0.651605655	5.72E-66	postive
PIK3CG	AL049840.4	0.534061462	8.82E-41	postive
JAK2	AL049840.4	0.613063479	1.57E-56	postive
TXK	AL049840.4	0.569573924	2.46E-47	postive
NFAT5	AL049840.4	0.751329162	2.84E-98	postive
MALT1	AL049840.4	0.513182011	2.82E-37	postive
PIK3R1	AL049840.4	0.576105751	1.25E-48	postive
PIK3CA	AL049840.4	0.598857531	2.26E-53	postive
AKT3	AL049840.4	0.546021481	6.69E-43	postive
RASGRP3	AL049840.4	0.507157252	2.61E-36	postive
IGHD4-4	AL049840.4	0.557324779	5.51E-45	postive
IGHD6-6	AL049840.4	0.524782183	3.41E-39	postive
LTB4R2	AL049840.4	0.516012706	9.75E-38	postive
GNRH1	AL049840.4	0.759533815	1.28E-101	postive
IL6ST	AL049840.4	0.517618841	5.32E-38	postive
RABEP1	AL049840.4	0.602182903	4.25E-54	postive
ACVR2A	AL049840.4	0.565431355	1.57E-46	postive
ANGPTL1	AL049840.4	0.647029524	8.90E-65	postive
BMPR2	AL049840.4	0.653204781	2.17E-66	postive
CRLF3	AL049840.4	0.599934378	1.32E-53	postive
NR2C1	AL049840.4	0.512087412	4.24E-37	postive
NR2C2	AL049840.4	0.728846295	9.75E-90	postive
RORA	AL049840.4	0.687992492	3.08E-76	postive
SOS1	AL049840.4	0.642321669	1.43E-63	postive
SOS2	AL049840.4	0.591267094	9.51E-52	postive
BRAF	AL049840.4	0.748621911	3.38E-97	postive
CBL	AL049840.4	0.67789807	3.07E-73	postive
CBLB	AL049840.4	0.630216615	1.44E-60	postive
PDK1	AL049840.4	0.515511574	1.18E-37	postive
TRAJ1	AL049840.4	0.669850733	6.18E-71	postive
TRAJ2	AL049840.4	0.691065591	3.57E-77	postive
TRAJ3	AL049840.4	0.742626836	7.30E-95	postive
TRAJ5	AL049840.4	0.627357216	7.07E-60	postive
TRAJ6	AL049840.4	0.696556858	7.05E-79	postive
TRAJ8	AL049840.4	0.652972848	2.49E-66	postive
TRAJ10	AL049840.4	0.655355636	5.82E-67	postive
TRAJ12	AL049840.4	0.59082601	1.18E-51	postive
TRAJ13	AL049840.4	0.646215498	1.44E-64	postive
TRAJ14	AL049840.4	0.644003437	5.33E-64	postive
TRAJ16	AL049840.4	0.638332963	1.44E-62	postive
TRAJ17	AL049840.4	0.61246136	2.15E-56	postive
TRAJ18	AL049840.4	0.612242142	2.41E-56	postive
TRAJ21	AL049840.4	0.668807906	1.22E-70	postive
TRAJ31	AL049840.4	0.653533701	1.77E-66	postive
TRAJ37	AL049840.4	0.666024204	7.28E-70	postive
TRAJ38	AL049840.4	0.70696896	3.23E-82	postive
TRAJ39	AL049840.4	0.686057705	1.18E-75	postive
CREB1	AL138963.1	0.802162715	1.86E-121	postive
RFXAP	AL138963.1	0.517800469	4.96E-38	postive
UBR1	AL138963.1	0.683234734	8.26E-75	postive
ZC3HAV1	AL138963.1	0.572936984	5.35E-48	postive
IL15	AL138963.1	0.552504093	4.36E-44	postive
CYLD	AL138963.1	0.610671889	5.47E-56	postive
EIF2AK2	AL138963.1	0.571813437	8.92E-48	postive
MAPK8	AL138963.1	0.580206209	1.86E-49	postive
LMBR1	AL138963.1	0.578682188	3.79E-49	postive
SP1	AL138963.1	0.51540454	1.23E-37	postive
LIMS1	AL138963.1	0.521644917	1.15E-38	postive
IREB2	AL138963.1	0.635015879	9.64E-62	postive
DDX17	AL138963.1	0.625149587	2.38E-59	postive
PIK3CG	AL138963.1	0.522669667	7.72E-39	postive
JAK2	AL138963.1	0.643822287	5.93E-64	postive
TXK	AL138963.1	0.649333421	2.25E-65	postive

NFAT5	AL138963.1	0.837947648	2.71E-142	postive
MALT1	AL138963.1	0.536702372	3.05E-41	postive
PIK3R1	AL138963.1	0.644913047	3.12E-64	postive
PIK3CA	AL138963.1	0.720689177	7.53E-87	postive
PIK3CB	AL138963.1	0.55769055	4.70E-45	postive
AKT3	AL138963.1	0.532278484	1.80E-40	postive
RASGRP3	AL138963.1	0.515378529	1.24E-37	postive
IGHD6-6	AL138963.1	0.518950072	3.21E-38	postive
ROBO2	AL138963.1	0.561306278	9.73E-46	postive
GNRH1	AL138963.1	0.782642453	8.13E-112	postive
IL6ST	AL138963.1	0.61654958	2.48E-57	postive
IL7	AL138963.1	0.500132547	3.31E-35	postive
RABEP1	AL138963.1	0.573654058	3.85E-48	postive
ACVR2A	AL138963.1	0.660117023	3.05E-68	postive
ANGPTL1	AL138963.1	0.771217631	1.26E-106	postive
BMPR1A	AL138963.1	0.581082516	1.23E-49	postive
BMPR2	AL138963.1	0.785831116	2.54E-113	postive
CRLF3	AL138963.1	0.640431354	4.29E-63	postive
NR1D2	AL138963.1	0.553057741	3.45E-44	postive
NR2C2	AL138963.1	0.697673405	3.14E-79	postive
RORA	AL138963.1	0.834604159	3.87E-140	postive
SOS1	AL138963.1	0.695193365	1.88E-78	postive
SOS2	AL138963.1	0.567804625	5.45E-47	postive
BRAF	AL138963.1	0.793059062	7.85E-117	postive
CBL	AL138963.1	0.598868605	2.25E-53	postive
CBLB	AL138963.1	0.740516906	4.67E-94	postive
RASGRP1	AL138963.1	0.502848366	1.25E-35	postive
PDK1	AL138963.1	0.592315777	5.71E-52	postive
TRAJ1	AL138963.1	0.684434523	3.63E-75	postive
TRAJ2	AL138963.1	0.691581414	2.48E-77	postive
TRAJ3	AL138963.1	0.747174011	1.26E-96	postive
TRAJ5	AL138963.1	0.729715081	4.73E-90	postive
TRAJ6	AL138963.1	0.755834777	4.29E-100	postive
TRAJ8	AL138963.1	0.712308426	5.48E-84	postive
TRAJ10	AL138963.1	0.719433974	2.05E-86	postive
TRAJ12	AL138963.1	0.68803856	2.99E-76	postive
TRAJ13	AL138963.1	0.732435419	4.84E-91	postive
TRAJ14	AL138963.1	0.710590251	2.05E-83	postive
TRAJ16	AL138963.1	0.751270104	3.00E-98	postive
TRAJ17	AL138963.1	0.699353301	9.24E-80	postive
TRAJ18	AL138963.1	0.699681195	7.27E-80	postive
TRAJ21	AL138963.1	0.765677302	3.24E-104	postive
TRAJ31	AL138963.1	0.789870499	2.88E-115	postive
TRAJ37	AL138963.1	0.753425383	4.08E-99	postive
TRAJ38	AL138963.1	0.824693573	5.02E-134	postive
TRAJ39	AL138963.1	0.773453451	1.29E-107	postive
IFI30	MMP2-AS1	0.540914331	5.51E-42	postive
CCL18	MMP2-AS1	0.548473413	2.40E-43	postive
FGR	MMP2-AS1	0.51363495	2.38E-37	postive
PIK3R5	MMP2-AS1	0.500807539	2.60E-35	postive
NR1H3	MMP2-AS1	0.530336831	3.88E-40	postive
CREB1	AP000240.1	0.627200453	7.71E-60	postive
DDX17	AP000240.1	0.529629847	5.13E-40	postive
JAK2	AP000240.1	0.537566566	2.15E-41	postive
NFAT5	AP000240.1	0.662424425	7.16E-69	postive
PIK3CA	AP000240.1	0.58432674	2.67E-50	postive
GNRH1	AP000240.1	0.6949731	2.21E-78	postive
ACVR2A	AP000240.1	0.535511141	4.93E-41	postive
ANGPTL1	AP000240.1	0.67230684	1.25E-71	postive
BMPR2	AP000240.1	0.614505018	7.33E-57	postive
CRLF3	AP000240.1	0.531868745	2.11E-40	postive
NR2C2	AP000240.1	0.578065801	5.04E-49	postive
RORA	AP000240.1	0.675985861	1.10E-72	postive
SOS1	AP000240.1	0.564192993	2.73E-46	postive
BRAF	AP000240.1	0.620623234	2.80E-58	postive

CBLB	AP000240.1	0.694445447	3.22E-78	postive
PDK1	AP000240.1	0.515483657	1.19E-37	postive
TRAJ1	AP000240.1	0.539574938	9.52E-42	postive
TRAJ2	AP000240.1	0.552780129	3.88E-44	postive
TRAJ3	AP000240.1	0.565000579	1.91E-46	postive
TRAJ5	AP000240.1	0.585823647	1.31E-50	postive
TRAJ6	AP000240.1	0.592598421	4.97E-52	postive
TRAJ8	AP000240.1	0.537696664	2.04E-41	postive
TRAJ10	AP000240.1	0.582190772	7.33E-50	postive
TRAJ12	AP000240.1	0.509463908	1.12E-36	postive
TRAJ13	AP000240.1	0.564832016	2.05E-46	postive
TRAJ14	AP000240.1	0.553847982	2.46E-44	postive
TRAJ16	AP000240.1	0.622972007	7.84E-59	postive
TRAJ17	AP000240.1	0.546526525	5.42E-43	postive
TRAJ18	AP000240.1	0.533258115	1.22E-40	postive
TRAJ21	AP000240.1	0.586160396	1.11E-50	postive
TRAJ31	AP000240.1	0.641308264	2.58E-63	postive
TRAJ37	AP000240.1	0.579877723	2.17E-49	postive
TRAJ38	AP000240.1	0.681636937	2.46E-74	postive
TRAJ39	AP000240.1	0.602776461	3.15E-54	postive
NFKBIZ	ADAMTSL4-AS2	0.520584042	1.72E-38	postive
DDX17	ADAMTSL4-AS2	0.522514639	8.20E-39	postive
NFAT5	ADAMTSL4-AS2	0.600506511	9.89E-54	postive
LTB4R2	ADAMTSL4-AS2	0.520546749	1.74E-38	postive
GNRH1	ADAMTSL4-AS2	0.554572111	1.80E-44	postive
ANGPTL1	ADAMTSL4-AS2	0.525682418	2.41E-39	postive
NR2C2	ADAMTSL4-AS2	0.58414815	2.90E-50	postive
RORA	ADAMTSL4-AS2	0.597878898	3.68E-53	postive
BRAF	ADAMTSL4-AS2	0.508474557	1.61E-36	postive
CBLB	ADAMTSL4-AS2	0.523407017	5.81E-39	postive
TRAJ1	ADAMTSL4-AS2	0.568077485	4.82E-47	postive
TRAJ3	ADAMTSL4-AS2	0.58899752	2.86E-51	postive
TRAJ5	ADAMTSL4-AS2	0.597367767	4.74E-53	postive
TRAJ6	ADAMTSL4-AS2	0.57777911	5.76E-49	postive
TRAJ8	ADAMTSL4-AS2	0.542135163	3.34E-42	postive
TRAJ10	ADAMTSL4-AS2	0.604775916	1.14E-54	postive
TRAJ12	ADAMTSL4-AS2	0.507933206	1.97E-36	postive
TRAJ13	ADAMTSL4-AS2	0.559682225	1.98E-45	postive
TRAJ14	ADAMTSL4-AS2	0.555671934	1.12E-44	postive
TRAJ16	ADAMTSL4-AS2	0.611520734	3.51E-56	postive
TRAJ17	ADAMTSL4-AS2	0.604737821	1.16E-54	postive
TRAJ18	ADAMTSL4-AS2	0.572586741	6.28E-48	postive
TRAJ21	ADAMTSL4-AS2	0.597924971	3.60E-53	postive
TRAJ31	ADAMTSL4-AS2	0.586776652	8.30E-51	postive
TRAJ37	ADAMTSL4-AS2	0.567546823	6.12E-47	postive
TRAJ38	ADAMTSL4-AS2	0.607105711	3.46E-55	postive
TRAJ39	ADAMTSL4-AS2	0.61520276	5.07E-57	postive
A2M	LINC01936	0.718771819	3.47E-86	postive
AGER	LINC01936	0.52864285	7.56E-40	postive
SLIT2	LINC01936	0.634606406	1.22E-61	postive
EDNRB	LINC01936	0.51541615	1.22E-37	postive
VEGFD	LINC01936	0.703886589	3.26E-81	postive
GDF10	LINC01936	0.581680465	9.32E-50	postive
LTBP2	LINC01936	0.566467692	9.91E-47	postive
ANGPT1	LINC01936	0.641254241	2.66E-63	postive
CALCRL	LINC01936	0.53950404	9.80E-42	postive
S1PR1	LINC01936	0.537649949	2.08E-41	postive
TEK	LINC01936	0.537515048	2.20E-41	postive
CREB1	PPP3CB-AS1	0.680875163	4.12E-74	postive
RFXAP	PPP3CB-AS1	0.525224739	2.87E-39	postive
UBR1	PPP3CB-AS1	0.639261691	8.45E-63	postive
ZC3HAV1	PPP3CB-AS1	0.508744503	1.46E-36	postive
CYLD	PPP3CB-AS1	0.613673637	1.14E-56	postive
MAPK8	PPP3CB-AS1	0.523923856	4.76E-39	postive
IREB2	PPP3CB-AS1	0.543289707	2.07E-42	postive

DDX17	PPP3CB-AS1	0.732092477	6.46E-91	postive
JAK2	PPP3CB-AS1	0.553891845	2.41E-44	postive
TXK	PPP3CB-AS1	0.59252914	5.14E-52	postive
PPP3CB	PPP3CB-AS1	0.51466984	1.62E-37	postive
NFAT5	PPP3CB-AS1	0.785626222	3.18E-113	postive
PIK3R1	PPP3CB-AS1	0.60550361	7.87E-55	postive
PIK3CA	PPP3CB-AS1	0.533666438	1.03E-40	postive
ROBO2	PPP3CB-AS1	0.562818778	5.00E-46	postive
GNRH1	PPP3CB-AS1	0.758685366	2.88E-101	postive
IL6ST	PPP3CB-AS1	0.591947952	6.83E-52	postive
RABEP1	PPP3CB-AS1	0.540095753	7.70E-42	postive
ACVR2A	PPP3CB-AS1	0.602679161	3.31E-54	postive
ANGPTL1	PPP3CB-AS1	0.60726765	3.18E-55	postive
BMPR1A	PPP3CB-AS1	0.534215826	8.29E-41	postive
BMPR2	PPP3CB-AS1	0.652471866	3.38E-66	postive
CRLF3	PPP3CB-AS1	0.5247178	3.50E-39	postive
NR1D2	PPP3CB-AS1	0.5529355	3.63E-44	postive
NR2C1	PPP3CB-AS1	0.545237133	9.26E-43	postive
NR2C2	PPP3CB-AS1	0.71456826	9.49E-85	postive
NR3C2	PPP3CB-AS1	0.518700108	3.53E-38	postive
RORA	PPP3CB-AS1	0.790760714	1.06E-115	postive
SOS1	PPP3CB-AS1	0.599003522	2.10E-53	postive
BRAF	PPP3CB-AS1	0.716075622	2.92E-85	postive
CBLB	PPP3CB-AS1	0.610908821	4.83E-56	postive
TRAJ1	PPP3CB-AS1	0.595756528	1.05E-52	postive
TRAJ2	PPP3CB-AS1	0.655091381	6.84E-67	postive
TRAJ3	PPP3CB-AS1	0.65045371	1.15E-65	postive
TRAJ5	PPP3CB-AS1	0.620235621	3.45E-58	postive
TRAJ6	PPP3CB-AS1	0.644652958	3.64E-64	postive
TRAJ8	PPP3CB-AS1	0.591519205	8.41E-52	postive
TRAJ10	PPP3CB-AS1	0.607637128	2.63E-55	postive
TRAJ12	PPP3CB-AS1	0.619644972	4.74E-58	postive
TRAJ13	PPP3CB-AS1	0.62615197	1.37E-59	postive
TRAJ14	PPP3CB-AS1	0.604492188	1.32E-54	postive
TRAJ16	PPP3CB-AS1	0.617825148	1.26E-57	postive
TRAJ17	PPP3CB-AS1	0.55699028	6.37E-45	postive
TRAJ18	PPP3CB-AS1	0.63622353	4.84E-62	postive
TRAJ21	PPP3CB-AS1	0.648765247	3.16E-65	postive
TRAJ31	PPP3CB-AS1	0.673574734	5.42E-72	postive
TRAJ37	PPP3CB-AS1	0.686872281	6.73E-76	postive
TRAJ38	PPP3CB-AS1	0.703795686	3.48E-81	postive
TRAJ39	PPP3CB-AS1	0.648467924	3.78E-65	postive
IRF3	PTOV1-AS1	0.500256299	3.17E-35	postive
GNRH1	AL035071.1	0.517527506	5.50E-38	postive
UCN3	AL713965.1	0.508514393	1.59E-36	postive
CREB1	AC011468.5	0.581448401	1.04E-49	postive
UBR1	AC011468.5	0.524493987	3.82E-39	postive
IREB2	AC011468.5	0.500596735	2.81E-35	postive
DDX17	AC011468.5	0.590134203	1.65E-51	postive
TXK	AC011468.5	0.538210781	1.66E-41	postive
NFAT5	AC011468.5	0.645141932	2.72E-64	postive
PIK3R1	AC011468.5	0.503833407	8.75E-36	postive
PIK3CA	AC011468.5	0.505910874	4.12E-36	postive
GNRH1	AC011468.5	0.755161525	8.07E-100	postive
IL6ST	AC011468.5	0.538383211	1.55E-41	postive
ACVR2A	AC011468.5	0.548966424	1.95E-43	postive
ANGPTL1	AC011468.5	0.571284791	1.13E-47	postive
BMPR2	AC011468.5	0.5742321	2.96E-48	postive
CRLF3	AC011468.5	0.50095813	2.47E-35	postive
NR2C1	AC011468.5	0.500177321	3.26E-35	postive
NR2C2	AC011468.5	0.654988209	7.29E-67	postive
RORA	AC011468.5	0.686080071	1.17E-75	postive
SOS1	AC011468.5	0.52583791	2.26E-39	postive
BRAF	AC011468.5	0.637785784	1.98E-62	postive
TEC	AC011468.5	0.52981001	4.77E-40	postive

CBLB	AC011468.5	0.595007056	1.53E-52	postive
PDK1	AC011468.5	0.502871248	1.24E-35	postive
TRAJ3	AC011468.5	0.514535558	1.70E-37	postive
TRAJ5	AC011468.5	0.558605979	3.16E-45	postive
TRAJ6	AC011468.5	0.516525815	8.04E-38	postive
TRAJ13	AC011468.5	0.505065115	5.60E-36	postive
TRAJ16	AC011468.5	0.512023573	4.34E-37	postive
TRAJ21	AC011468.5	0.537936423	1.85E-41	postive
TRAJ31	AC011468.5	0.559607568	2.04E-45	postive
TRAJ37	AC011468.5	0.550525116	1.01E-43	postive
TRAJ38	AC011468.5	0.572529911	6.44E-48	postive
TRAJ39	AC011468.5	0.58541319	1.59E-50	postive
GAL	SNHG1	0.529579182	5.23E-40	postive
GNRH1	LINC01876	0.594082716	2.40E-52	postive
TEC	LINC01876	0.587547024	5.74E-51	postive
IRF9	MMP25-AS1	0.536817548	2.91E-41	postive
ZAP70	MMP25-AS1	0.578530702	4.06E-49	postive
CREB1	AC024933.1	0.732634337	4.09E-91	postive
UBR1	AC024933.1	0.578341054	4.44E-49	postive
ZC3HAV1	AC024933.1	0.520922128	1.51E-38	postive
IL15	AC024933.1	0.50053994	2.86E-35	postive
CYLD	AC024933.1	0.561475687	9.03E-46	postive
MAPK8	AC024933.1	0.504570045	6.70E-36	postive
LMBR1	AC024933.1	0.512799352	3.25E-37	postive
IREB2	AC024933.1	0.544918572	1.06E-42	postive
DDX17	AC024933.1	0.581537538	9.96E-50	postive
JAK2	AC024933.1	0.583976504	3.15E-50	postive
TXK	AC024933.1	0.520630719	1.69E-38	postive
NFAT5	AC024933.1	0.776236437	7.22E-109	postive
PIK3R1	AC024933.1	0.551190566	7.62E-44	postive
PIK3CA	AC024933.1	0.667257018	3.30E-70	postive
PIK3CB	AC024933.1	0.524558761	3.72E-39	postive
GNRH1	AC024933.1	0.70320664	5.40E-81	postive
IL6ST	AC024933.1	0.562187395	6.61E-46	postive
ACVR2A	AC024933.1	0.624812503	2.87E-59	postive
ANGPTL1	AC024933.1	0.705024172	1.39E-81	postive
BMPR1A	AC024933.1	0.504472657	6.94E-36	postive
BMPR2	AC024933.1	0.693060574	8.66E-78	postive
CRLF3	AC024933.1	0.566932671	8.06E-47	postive
NR2C2	AC024933.1	0.654392395	1.05E-66	postive
RORA	AC024933.1	0.764569038	9.67E-104	postive
SOS1	AC024933.1	0.650556349	1.08E-65	postive
SOS2	AC024933.1	0.526149857	2.01E-39	postive
BRAF	AC024933.1	0.720057138	1.25E-86	postive
CBL	AC024933.1	0.563599997	3.54E-46	postive
CBLB	AC024933.1	0.691534157	2.56E-77	postive
PDK1	AC024933.1	0.507113026	2.65E-36	postive
TRAJ1	AC024933.1	0.683341498	7.68E-75	postive
TRAJ2	AC024933.1	0.66395081	2.73E-69	postive
TRAJ3	AC024933.1	0.691535663	2.56E-77	postive
TRAJ5	AC024933.1	0.738001554	4.16E-93	postive
TRAJ6	AC024933.1	0.726141955	9.08E-89	postive
TRAJ8	AC024933.1	0.714475842	1.02E-84	postive
TRAJ10	AC024933.1	0.710401876	2.37E-83	postive
TRAJ12	AC024933.1	0.647822249	5.55E-65	postive
TRAJ13	AC024933.1	0.728974915	8.76E-90	postive
TRAJ14	AC024933.1	0.702673552	8.02E-81	postive
TRAJ16	AC024933.1	0.752231134	1.24E-98	postive
TRAJ17	AC024933.1	0.700955146	2.85E-80	postive
TRAJ18	AC024933.1	0.645348949	2.41E-64	postive
TRAJ21	AC024933.1	0.740518844	4.66E-94	postive
TRAJ31	AC024933.1	0.776797772	4.02E-109	postive
TRAJ37	AC024933.1	0.728844648	9.76E-90	postive
TRAJ38	AC024933.1	0.795848745	3.18E-118	postive
TRAJ39	AC024933.1	0.747816077	7.03E-97	postive

CREB1	AL031775.2	0.698479518	1.75E-79	postive
RFXAP	AL031775.2	0.538619892	1.40E-41	postive
UBR1	AL031775.2	0.586674308	8.72E-51	postive
ZC3HAV1	AL031775.2	0.521349813	1.28E-38	postive
CYLD	AL031775.2	0.543714992	1.74E-42	postive
EIF2AK2	AL031775.2	0.513584658	2.43E-37	postive
MAPK8	AL031775.2	0.523664943	5.26E-39	postive
NFKBIZ	AL031775.2	0.50164478	1.93E-35	postive
IREB2	AL031775.2	0.535382484	5.19E-41	postive
DDX17	AL031775.2	0.682715714	1.18E-74	postive
JAK2	AL031775.2	0.574307871	2.86E-48	postive
TXK	AL031775.2	0.544532618	1.24E-42	postive
NFAT5	AL031775.2	0.781420746	3.02E-111	postive
MALT1	AL031775.2	0.500385894	3.03E-35	postive
PIK3R1	AL031775.2	0.56523905	1.71E-46	postive
PIK3CA	AL031775.2	0.581399305	1.06E-49	postive
LTB4R2	AL031775.2	0.506735261	3.05E-36	postive
GNRH1	AL031775.2	0.793317133	5.84E-117	postive
IL6ST	AL031775.2	0.55685794	6.74E-45	postive
RABEP1	AL031775.2	0.526044544	2.09E-39	postive
ACVR2A	AL031775.2	0.616873166	2.09E-57	postive
ANGPTL1	AL031775.2	0.70296644	6.45E-81	postive
BMPR2	AL031775.2	0.616416963	2.66E-57	postive
CRLF3	AL031775.2	0.596381597	7.74E-53	postive
NR2C1	AL031775.2	0.555736203	1.09E-44	postive
NR2C2	AL031775.2	0.74778226	7.24E-97	postive
RORA	AL031775.2	0.761486778	1.95E-102	postive
SOS1	AL031775.2	0.647738289	5.84E-65	postive
SOS2	AL031775.2	0.553480703	2.88E-44	postive
BRAF	AL031775.2	0.783297885	4.01E-112	postive
CBL	AL031775.2	0.525264667	2.83E-39	postive
CBLB	AL031775.2	0.679375033	1.14E-73	postive
PDK1	AL031775.2	0.524192496	4.29E-39	postive
TRAJ1	AL031775.2	0.72344443	8.19E-88	postive
TRAJ2	AL031775.2	0.704810886	1.63E-81	postive
TRAJ3	AL031775.2	0.743107231	4.77E-95	postive
TRAJ5	AL031775.2	0.754343482	1.73E-99	postive
TRAJ6	AL031775.2	0.750476878	6.22E-98	postive
TRAJ8	AL031775.2	0.736628266	1.36E-92	postive
TRAJ10	AL031775.2	0.722698207	1.50E-87	postive
TRAJ12	AL031775.2	0.676023105	1.07E-72	postive
TRAJ13	AL031775.2	0.74251287	8.07E-95	postive
TRAJ14	AL031775.2	0.74258699	7.56E-95	postive
TRAJ16	AL031775.2	0.752109467	1.38E-98	postive
TRAJ17	AL031775.2	0.707098877	2.93E-82	postive
TRAJ18	AL031775.2	0.676066932	1.04E-72	postive
TRAJ21	AL031775.2	0.763848255	1.96E-103	postive
TRAJ31	AL031775.2	0.776605296	4.92E-109	postive
TRAJ37	AL031775.2	0.747543068	9.00E-97	postive
TRAJ38	AL031775.2	0.810991893	3.51E-126	postive
TRAJ39	AL031775.2	0.779885317	1.55E-110	postive
CREB1	ATP1A1-AS1	0.640828447	3.41E-63	postive
UBR1	ATP1A1-AS1	0.570178153	1.87E-47	postive
MAPK8	ATP1A1-AS1	0.50932122	1.18E-36	postive
IREB2	ATP1A1-AS1	0.545003052	1.02E-42	postive
DDX17	ATP1A1-AS1	0.547506504	3.60E-43	postive
TXK	ATP1A1-AS1	0.534991322	6.08E-41	postive
NFAT5	ATP1A1-AS1	0.60557308	7.59E-55	postive
PIK3R1	ATP1A1-AS1	0.614830467	6.17E-57	postive
IGHD4-4	ATP1A1-AS1	0.555842454	1.04E-44	postive
GNRH1	ATP1A1-AS1	0.659558893	4.32E-68	postive
IL6ST	ATP1A1-AS1	0.594412165	2.04E-52	postive
ACVR2A	ATP1A1-AS1	0.549078049	1.86E-43	postive
ANGPTL1	ATP1A1-AS1	0.644406384	4.20E-64	postive
BMPR2	ATP1A1-AS1	0.60726619	3.19E-55	postive

NR2C2	ATP1A1-AS1	0.648346394	4.06E-65	postive
RORA	ATP1A1-AS1	0.7112468	1.24E-83	postive
SOS1	ATP1A1-AS1	0.534784648	6.60E-41	postive
BRAF	ATP1A1-AS1	0.705509124	9.68E-82	postive
CBLB	ATP1A1-AS1	0.559802305	1.88E-45	postive
PDK1	ATP1A1-AS1	0.512495043	3.64E-37	postive
TRAJ3	ATP1A1-AS1	0.528453156	8.14E-40	postive
TRAJ5	ATP1A1-AS1	0.541408153	4.50E-42	postive
TRAJ21	ATP1A1-AS1	0.514892533	1.49E-37	postive
TRAJ31	ATP1A1-AS1	0.552035536	5.32E-44	postive
TRAJ37	ATP1A1-AS1	0.545778295	7.40E-43	postive
TRAJ38	ATP1A1-AS1	0.582756051	5.61E-50	postive
TRAJ39	ATP1A1-AS1	0.559698805	1.96E-45	postive
DDX17	AC095057.3	0.608610004	1.59E-55	postive
NFAT5	AC095057.3	0.511724999	4.85E-37	postive
GNRH1	AC095057.3	0.613353226	1.34E-56	postive
NR2C1	AC095057.3	0.555074352	1.45E-44	postive
NR2C2	AC095057.3	0.553766576	2.55E-44	postive
CREB1	AC009090.3	0.62050834	2.98E-58	postive
UBR1	AC009090.3	0.58321528	4.52E-50	postive
CYLD	AC009090.3	0.53022755	4.05E-40	postive
DDX17	AC009090.3	0.666608842	5.01E-70	postive
JAK2	AC009090.3	0.516173682	9.18E-38	postive
TXK	AC009090.3	0.627984765	4.99E-60	postive
NFAT5	AC009090.3	0.686665951	7.76E-76	postive
PIK3R1	AC009090.3	0.571052705	1.26E-47	postive
PIK3CA	AC009090.3	0.531285105	2.66E-40	postive
ROBO2	AC009090.3	0.563204772	4.22E-46	postive
GNRH1	AC009090.3	0.776800235	4.01E-109	postive
IL6ST	AC009090.3	0.592364043	5.57E-52	postive
ACVR2A	AC009090.3	0.532585779	1.59E-40	postive
ANGPTL1	AC009090.3	0.62373644	5.17E-59	postive
BMPR2	AC009090.3	0.620482972	3.02E-58	postive
NR2C1	AC009090.3	0.514844658	1.51E-37	postive
NR2C2	AC009090.3	0.662572125	6.52E-69	postive
NR3C2	AC009090.3	0.537696327	2.04E-41	postive
RORA	AC009090.3	0.71244191	4.94E-84	postive
SOS1	AC009090.3	0.502385519	1.48E-35	postive
BRAF	AC009090.3	0.65992916	3.43E-68	postive
CBLB	AC009090.3	0.597182843	5.20E-53	postive
TRAJ3	AC009090.3	0.529467563	5.46E-40	postive
TRAJ5	AC009090.3	0.554641731	1.75E-44	postive
TRAJ6	AC009090.3	0.520780964	1.59E-38	postive
TRAJ13	AC009090.3	0.513152993	2.85E-37	postive
TRAJ16	AC009090.3	0.501134712	2.31E-35	postive
TRAJ18	AC009090.3	0.517341045	5.91E-38	postive
TRAJ21	AC009090.3	0.549411757	1.62E-43	postive
TRAJ31	AC009090.3	0.56424535	2.66E-46	postive
TRAJ37	AC009090.3	0.53993093	8.23E-42	postive
TRAJ38	AC009090.3	0.583841428	3.36E-50	postive
TRAJ39	AC009090.3	0.55239079	4.58E-44	postive
CREB1	AC010834.3	0.75408148	2.22E-99	postive
RFXAP	AC010834.3	0.521801236	1.08E-38	postive
UBR1	AC010834.3	0.604124579	1.59E-54	postive
ZC3HAV1	AC010834.3	0.594056485	2.43E-52	postive
ZC3HAV1L	AC010834.3	0.573661787	3.84E-48	postive
IL15	AC010834.3	0.538732026	1.34E-41	postive
CYLD	AC010834.3	0.539486799	9.87E-42	postive
EIF2AK2	AC010834.3	0.541666412	4.05E-42	postive
MAPK8	AC010834.3	0.573062263	5.05E-48	postive
LMBR1	AC010834.3	0.588035998	4.54E-51	postive
IREB2	AC010834.3	0.608783501	1.46E-55	postive
DDX17	AC010834.3	0.589152657	2.65E-51	postive
JAK2	AC010834.3	0.545977961	6.81E-43	postive
PTK2	AC010834.3	0.529343727	5.74E-40	postive

TXK	AC010834.3	0.516393834	8.45E-38	postive
PPP3CB	AC010834.3	0.506435127	3.40E-36	postive
NFAT5	AC010834.3	0.766052812	2.24E-104	postive
MALT1	AC010834.3	0.512502691	3.63E-37	postive
PIK3R1	AC010834.3	0.607200785	3.29E-55	postive
PIK3CA	AC010834.3	0.637910798	1.84E-62	postive
PIK3CB	AC010834.3	0.504174942	7.74E-36	postive
AKT3	AC010834.3	0.624159189	4.10E-59	postive
IGHD4-4	AC010834.3	0.545671305	7.73E-43	postive
GMFB	AC010834.3	0.500178841	3.26E-35	postive
GNRH1	AC010834.3	0.688193175	2.68E-76	postive
IL6ST	AC010834.3	0.600317688	1.09E-53	postive
RABEP1	AC010834.3	0.568508956	3.97E-47	postive
ACVR2A	AC010834.3	0.625804898	1.66E-59	postive
ANGPTL1	AC010834.3	0.752590705	8.86E-99	postive
BMPR1A	AC010834.3	0.546213296	6.17E-43	postive
BMPR2	AC010834.3	0.668165124	1.84E-70	postive
CRLF3	AC010834.3	0.642615609	1.20E-63	postive
NR2C2	AC010834.3	0.698689078	1.50E-79	postive
RORA	AC010834.3	0.740575463	4.43E-94	postive
SOS1	AC010834.3	0.704422377	2.18E-81	postive
SOS2	AC010834.3	0.613606204	1.18E-56	postive
BRAF	AC010834.3	0.837202253	8.27E-142	postive
CBL	AC010834.3	0.625987773	1.50E-59	postive
CBLB	AC010834.3	0.650454032	1.15E-65	postive
PDK1	AC010834.3	0.548713903	2.17E-43	postive
TRAJ1	AC010834.3	0.739403056	1.23E-93	postive
TRAJ2	AC010834.3	0.722284242	2.09E-87	postive
TRAJ3	AC010834.3	0.794009576	2.65E-117	postive
TRAJ5	AC010834.3	0.805596926	2.89E-123	postive
TRAJ6	AC010834.3	0.774353113	5.09E-108	postive
TRAJ8	AC010834.3	0.838854775	6.91E-143	postive
TRAJ10	AC010834.3	0.733801719	1.52E-91	postive
TRAJ12	AC010834.3	0.666700945	4.72E-70	postive
TRAJ13	AC010834.3	0.830374804	1.76E-137	postive
TRAJ14	AC010834.3	0.803190157	5.39E-122	postive
TRAJ16	AC010834.3	0.756875985	1.60E-100	postive
TRAJ17	AC010834.3	0.765285716	4.77E-104	postive
TRAJ18	AC010834.3	0.640130214	5.11E-63	postive
TRAJ21	AC010834.3	0.821317822	4.97E-132	postive
TRAJ31	AC010834.3	0.810971539	3.60E-126	postive
TRAJ37	AC010834.3	0.785137433	5.42E-113	postive
TRAJ38	AC010834.3	0.818587332	1.90E-130	postive
TRAJ39	AC010834.3	0.836420973	2.65E-141	postive
TKFC	BX537318.1	0.546578769	5.30E-43	postive
CREB1	AC073487.1	0.777657233	1.63E-109	postive
RFXAP	AC073487.1	0.558422814	3.42E-45	postive
UBR1	AC073487.1	0.651874804	4.86E-66	postive
ZC3HAV1	AC073487.1	0.569830243	2.19E-47	postive
ZC3HAV1L	AC073487.1	0.516306591	8.73E-38	postive
IL15	AC073487.1	0.575551015	1.61E-48	postive
CYLD	AC073487.1	0.594669871	1.80E-52	postive
EIF2AK2	AC073487.1	0.545364292	8.79E-43	postive
MAPK8	AC073487.1	0.596294477	8.08E-53	postive
LMBR1	AC073487.1	0.538511997	1.47E-41	postive
IREB2	AC073487.1	0.613427662	1.29E-56	postive
DDX17	AC073487.1	0.691266718	3.09E-77	postive
JAK2	AC073487.1	0.625943814	1.54E-59	postive
TXK	AC073487.1	0.611703453	3.19E-56	postive
NFAT5	AC073487.1	0.84761608	8.23E-149	postive
MALT1	AC073487.1	0.537615664	2.11E-41	postive
PIK3R1	AC073487.1	0.606848048	3.95E-55	postive
PIK3CA	AC073487.1	0.66279496	5.67E-69	postive
PIK3CB	AC073487.1	0.515001531	1.43E-37	postive
AKT3	AC073487.1	0.531170954	2.79E-40	postive

RASGRP3	AC073487.1	0.533126993	1.28E-40	postive
IGHD6-6	AC073487.1	0.51535848	1.25E-37	postive
LTB4R2	AC073487.1	0.518838001	3.35E-38	postive
GNRH1	AC073487.1	0.832121887	1.43E-138	postive
IL6ST	AC073487.1	0.586654642	8.80E-51	postive
RABEP1	AC073487.1	0.569881263	2.14E-47	postive
ACVR2A	AC073487.1	0.663992063	2.66E-69	postive
ANGPTL1	AC073487.1	0.759779257	1.01E-101	postive
BMPR1A	AC073487.1	0.563770653	3.29E-46	postive
BMPR2	AC073487.1	0.710240391	2.69E-83	postive
CRLF3	AC073487.1	0.66092944	1.83E-68	postive
NR2C1	AC073487.1	0.576358161	1.11E-48	postive
NR2C2	AC073487.1	0.768897538	1.31E-105	postive
RORA	AC073487.1	0.812564845	4.77E-127	postive
SOS1	AC073487.1	0.701754706	1.58E-80	postive
SOS2	AC073487.1	0.593984742	2.52E-52	postive
BRAF	AC073487.1	0.818416373	2.38E-130	postive
TEC	AC073487.1	0.501682086	1.90E-35	postive
CBL	AC073487.1	0.608128722	2.04E-55	postive
CBLB	AC073487.1	0.743711139	2.79E-95	postive
PDK1	AC073487.1	0.581872138	8.51E-50	postive
TRAJ1	AC073487.1	0.750788001	4.67E-98	postive
TRAJ2	AC073487.1	0.764992641	6.37E-104	postive
TRAJ3	AC073487.1	0.806041556	1.67E-123	postive
TRAJ5	AC073487.1	0.764849514	7.34E-104	postive
TRAJ6	AC073487.1	0.800937068	8.05E-121	postive
TRAJ8	AC073487.1	0.7711366	1.37E-106	postive
TRAJ10	AC073487.1	0.769406078	7.88E-106	postive
TRAJ12	AC073487.1	0.72424747	4.27E-88	postive
TRAJ13	AC073487.1	0.773481827	1.25E-107	postive
TRAJ14	AC073487.1	0.771476146	9.71E-107	postive
TRAJ16	AC073487.1	0.795082568	7.70E-118	postive
TRAJ17	AC073487.1	0.742908652	5.69E-95	postive
TRAJ18	AC073487.1	0.725020963	2.27E-88	postive
TRAJ21	AC073487.1	0.801967683	2.35E-121	postive
TRAJ31	AC073487.1	0.813589179	1.28E-127	postive
TRAJ37	AC073487.1	0.793191751	6.74E-117	postive
TRAJ38	AC073487.1	0.859156604	3.27E-157	postive
TRAJ39	AC073487.1	0.814106936	6.60E-128	postive
ZYX	AC093673.1	0.578209555	4.72E-49	postive
TNFRSF12A	AC093673.1	0.551068695	8.02E-44	postive
CREB1	AL158212.3	0.537659863	2.07E-41	postive
ZC3HAV1	AL158212.3	0.591793752	7.36E-52	postive
MAVS	AL158212.3	0.53980729	8.66E-42	postive
MAPK8	AL158212.3	0.504702974	6.39E-36	postive
IREB2	AL158212.3	0.506915832	2.85E-36	postive
DDX17	AL158212.3	0.612017282	2.71E-56	postive
NFAT5	AL158212.3	0.566840747	8.39E-47	postive
GNRH1	AL158212.3	0.665592418	9.59E-70	postive
ACVR2A	AL158212.3	0.516889866	7.00E-38	postive
ANGPTL1	AL158212.3	0.51633133	8.65E-38	postive
NR2C2	AL158212.3	0.706360967	5.10E-82	postive
RORA	AL158212.3	0.583167476	4.62E-50	postive
SOS1	AL158212.3	0.563873488	3.14E-46	postive
BRAF	AL158212.3	0.57056527	1.57E-47	postive
CBL	AL158212.3	0.57015571	1.89E-47	postive
CBLB	AL158212.3	0.551534588	6.59E-44	postive
TRAJ3	AL158212.3	0.517257928	6.09E-38	postive
TRAJ5	AL158212.3	0.526548617	1.72E-39	postive
TRAJ38	AL158212.3	0.510052172	9.01E-37	postive
TRAJ39	AL158212.3	0.518675663	3.56E-38	postive
UBR1	AL445493.3	0.504898769	5.95E-36	postive
DDX17	AL445493.3	0.527794497	1.05E-39	postive
NFAT5	AL445493.3	0.562956747	4.71E-46	postive
IGHD6-6	AL445493.3	0.516179917	9.16E-38	postive

GNRH1	AL445493.3	0.651286141	6.94E-66	postive
NR2C2	AL445493.3	0.549050879	1.88E-43	postive
RORA	AL445493.3	0.53098553	3.00E-40	postive
BRAF	AL445493.3	0.511459744	5.35E-37	postive
TEC	AL445493.3	0.585059444	1.88E-50	postive
NFKBIZ	AC123595.1	0.561240955	1.00E-45	postive
TRAJ5	AC123595.1	0.565633483	1.44E-46	postive
CREB1	AC069023.1	0.589374036	2.38E-51	postive
IL15	AC069023.1	0.644933393	3.08E-64	postive
JAK2	AC069023.1	0.57723839	7.40E-49	postive
NFAT5	AC069023.1	0.615244314	4.96E-57	postive
PIK3CA	AC069023.1	0.580938874	1.32E-49	postive
GNRH1	AC069023.1	0.502182587	1.59E-35	postive
ANGPTL1	AC069023.1	0.680255035	6.27E-74	postive
BMPR2	AC069023.1	0.516491836	8.14E-38	postive
IL18R1	AC069023.1	0.55360807	2.72E-44	postive
RORA	AC069023.1	0.609209812	1.17E-55	postive
BRAF	AC069023.1	0.564987582	1.92E-46	postive
CBL	AC069023.1	0.531513188	2.43E-40	postive
CBLB	AC069023.1	0.657537172	1.52E-67	postive
PDK1	AC069023.1	0.545578245	8.04E-43	postive
TRAJ1	AC069023.1	0.512133177	4.17E-37	postive
TRAJ2	AC069023.1	0.578648375	3.85E-49	postive
TRAJ3	AC069023.1	0.655461254	5.45E-67	postive
TRAJ5	AC069023.1	0.527152408	1.36E-39	postive
TRAJ6	AC069023.1	0.615295286	4.83E-57	postive
TRAJ10	AC069023.1	0.63970928	6.53E-63	postive
TRAJ12	AC069023.1	0.552152724	5.07E-44	postive
TRAJ13	AC069023.1	0.51610433	9.42E-38	postive
TRAJ14	AC069023.1	0.575623361	1.56E-48	postive
TRAJ16	AC069023.1	0.596105615	8.87E-53	postive
TRAJ17	AC069023.1	0.567610354	5.95E-47	postive
TRAJ18	AC069023.1	0.660305233	2.71E-68	postive
TRAJ21	AC069023.1	0.607656659	2.61E-55	postive
TRAJ31	AC069023.1	0.577281694	7.26E-49	postive
TRAJ37	AC069023.1	0.56269566	5.28E-46	postive
TRAJ38	AC069023.1	0.672727112	9.46E-72	postive
TRAJ39	AC069023.1	0.588896122	3.00E-51	postive
PLAU	LINC00973	0.52256485	8.04E-39	postive
FAM3C	LINC00973	0.519986774	2.16E-38	postive
PLXNB3	U52111.1	0.636288777	4.67E-62	postive
CREB1	AC090948.1	0.782764244	7.13E-112	postive
RFXAP	AC090948.1	0.54510534	9.79E-43	postive
UBR1	AC090948.1	0.656791766	2.40E-67	postive
ZC3HAV1	AC090948.1	0.585010789	1.93E-50	postive
IL15	AC090948.1	0.614787674	6.31E-57	postive
CYLD	AC090948.1	0.669616907	7.20E-71	postive
EIF2AK2	AC090948.1	0.568323359	4.32E-47	postive
MAPK8	AC090948.1	0.552774834	3.89E-44	postive
LMBR1	AC090948.1	0.545178928	9.49E-43	postive
IREB2	AC090948.1	0.599011809	2.09E-53	postive
DDX17	AC090948.1	0.670106563	5.24E-71	postive
PIK3CG	AC090948.1	0.567462153	6.36E-47	postive
JAK2	AC090948.1	0.670498582	4.06E-71	postive
TXK	AC090948.1	0.641319378	2.56E-63	postive
NFAT5	AC090948.1	0.873709084	6.13E-169	postive
NFATC3	AC090948.1	0.502781659	1.28E-35	postive
MALT1	AC090948.1	0.552949856	3.61E-44	postive
PIK3R1	AC090948.1	0.688704799	1.87E-76	postive
PIK3CA	AC090948.1	0.684675086	3.07E-75	postive
PIK3CB	AC090948.1	0.524684412	3.54E-39	postive
AKT3	AC090948.1	0.534179649	8.41E-41	postive
RASGRP3	AC090948.1	0.574043183	3.23E-48	postive
IGHD6-6	AC090948.1	0.564650924	2.23E-46	postive
PLXNC1	AC090948.1	0.540127486	7.60E-42	postive

GNRH1	AC090948.1	0.769099418	1.07E-105	postive
IL6ST	AC090948.1	0.667077114	3.71E-70	postive
RABEP1	AC090948.1	0.570553912	1.58E-47	postive
ACVR2A	AC090948.1	0.642242108	1.50E-63	postive
ANGPTL1	AC090948.1	0.780738027	6.27E-111	postive
BMPR1A	AC090948.1	0.532211723	1.84E-40	postive
BMPR2	AC090948.1	0.735117601	4.97E-92	postive
CRLF3	AC090948.1	0.65085212	9.02E-66	postive
LIFR	AC090948.1	0.513376842	2.62E-37	postive
NR1D2	AC090948.1	0.525010405	3.12E-39	postive
NR2C2	AC090948.1	0.750325465	7.14E-98	postive
RORA	AC090948.1	0.87766069	2.24E-172	postive
SOS1	AC090948.1	0.700076937	5.44E-80	postive
SOS2	AC090948.1	0.589067511	2.76E-51	postive
BRAF	AC090948.1	0.838034123	2.38E-142	postive
ITK	AC090948.1	0.60257629	3.49E-54	postive
TEC	AC090948.1	0.503187229	1.11E-35	postive
CD28	AC090948.1	0.551807661	5.87E-44	postive
CBL	AC090948.1	0.621439581	1.80E-58	postive
CBLB	AC090948.1	0.749813509	1.14E-97	postive
RASGRP1	AC090948.1	0.574246092	2.94E-48	postive
PDK1	AC090948.1	0.571326979	1.11E-47	postive
TRAJ1	AC090948.1	0.800971799	7.72E-121	postive
TRAJ2	AC090948.1	0.799435582	4.78E-120	postive
TRAJ3	AC090948.1	0.86175086	3.34E-159	postive
TRAJ5	AC090948.1	0.845263659	3.48E-147	postive
TRAJ6	AC090948.1	0.843405849	6.42E-146	postive
TRAJ8	AC090948.1	0.819709096	4.28E-131	postive
TRAJ10	AC090948.1	0.812572711	4.72E-127	postive
TRAJ12	AC090948.1	0.788046061	2.21E-114	postive
TRAJ13	AC090948.1	0.835912161	5.63E-141	postive
TRAJ14	AC090948.1	0.83658474	2.07E-141	postive
TRAJ16	AC090948.1	0.83830637	1.58E-142	postive
TRAJ17	AC090948.1	0.806570797	8.74E-124	postive
TRAJ18	AC090948.1	0.812071112	8.94E-127	postive
TRAJ21	AC090948.1	0.872753885	3.99E-168	postive
TRAJ31	AC090948.1	0.876184664	4.44E-171	postive
TRAJ37	AC090948.1	0.862367391	1.11E-159	postive
TRAJ38	AC090948.1	0.907367849	9.08E-203	postive
TRAJ39	AC090948.1	0.885769202	8.09E-180	postive
CREB1	AC093788.1	0.73454126	8.12E-92	postive
RFXAP	AC093788.1	0.53737289	2.33E-41	postive
UBR1	AC093788.1	0.595649023	1.11E-52	postive
ZC3HAV1	AC093788.1	0.538690749	1.36E-41	postive
IL15	AC093788.1	0.561437017	9.19E-46	postive
CYLD	AC093788.1	0.596996566	5.70E-53	postive
EIF2AK2	AC093788.1	0.526698718	1.62E-39	postive
MAPK8	AC093788.1	0.550162917	1.18E-43	postive
LMBR1	AC093788.1	0.505212547	5.31E-36	postive
IREB2	AC093788.1	0.552177862	5.01E-44	postive
DDX17	AC093788.1	0.704446038	2.14E-81	postive
JAK2	AC093788.1	0.617679847	1.36E-57	postive
TXK	AC093788.1	0.569517953	2.52E-47	postive
NFAT5	AC093788.1	0.845822696	1.44E-147	postive
MALT1	AC093788.1	0.511801137	4.71E-37	postive
PIK3R1	AC093788.1	0.591316342	9.29E-52	postive
PIK3CA	AC093788.1	0.618554562	8.51E-58	postive
AKT3	AC093788.1	0.51531387	1.27E-37	postive
RASGRP3	AC093788.1	0.520726192	1.63E-38	postive
GNRH1	AC093788.1	0.807981902	1.53E-124	postive
IL6ST	AC093788.1	0.589725889	2.01E-51	postive
RABEP1	AC093788.1	0.539538193	9.66E-42	postive
ACVR2A	AC093788.1	0.620343184	3.25E-58	postive
ANGPTL1	AC093788.1	0.731394383	1.16E-90	postive
BMPR1A	AC093788.1	0.504686495	6.43E-36	postive

BMPR2	AC093788.1	0.653998054	1.34E-66	postive
CRLF3	AC093788.1	0.636489695	4.16E-62	postive
NR2C1	AC093788.1	0.547762726	3.23E-43	postive
NR2C2	AC093788.1	0.739586352	1.05E-93	postive
RORA	AC093788.1	0.807696621	2.17E-124	postive
SOS1	AC093788.1	0.678684316	1.81E-73	postive
SOS2	AC093788.1	0.549489908	1.56E-43	postive
BRAF	AC093788.1	0.807626879	2.37E-124	postive
ITK	AC093788.1	0.510014151	9.14E-37	postive
CBL	AC093788.1	0.576515241	1.03E-48	postive
CBLB	AC093788.1	0.724075509	4.91E-88	postive
PDK1	AC093788.1	0.540452319	6.65E-42	postive
TRAJ1	AC093788.1	0.783901266	2.08E-112	postive
TRAJ2	AC093788.1	0.783280104	4.08E-112	postive
TRAJ3	AC093788.1	0.81391833	8.41E-128	postive
TRAJ5	AC093788.1	0.814721814	2.99E-128	postive
TRAJ6	AC093788.1	0.822872998	6.06E-133	postive
TRAJ8	AC093788.1	0.805073177	5.48E-123	postive
TRAJ10	AC093788.1	0.789343775	5.20E-115	postive
TRAJ12	AC093788.1	0.757588216	8.17E-101	postive
TRAJ13	AC093788.1	0.822729362	7.36E-133	postive
TRAJ14	AC093788.1	0.818478382	2.20E-130	postive
TRAJ16	AC093788.1	0.814961115	2.19E-128	postive
TRAJ17	AC093788.1	0.768525477	1.91E-105	postive
TRAJ18	AC093788.1	0.754561215	1.42E-99	postive
TRAJ21	AC093788.1	0.841508459	1.21E-144	postive
TRAJ31	AC093788.1	0.849739493	2.65E-150	postive
TRAJ37	AC093788.1	0.835072942	1.94E-140	postive
TRAJ38	AC093788.1	0.880546854	5.80E-175	postive
TRAJ39	AC093788.1	0.847279628	1.41E-148	postive
CREB1	AC068790.2	0.799436977	4.77E-120	postive
RFXAP	AC068790.2	0.535390515	5.18E-41	postive
UBR1	AC068790.2	0.66821024	1.79E-70	postive
ZC3HAV1	AC068790.2	0.581977712	8.10E-50	postive
ZC3HAV1L	AC068790.2	0.531307819	2.64E-40	postive
IL15	AC068790.2	0.566701323	8.93E-47	postive
CYLD	AC068790.2	0.611368774	3.80E-56	postive
EIF2AK2	AC068790.2	0.559368235	2.27E-45	postive
MAPK8	AC068790.2	0.569424451	2.63E-47	postive
LMBR1	AC068790.2	0.590571206	1.33E-51	postive
IREB2	AC068790.2	0.633950467	1.76E-61	postive
DDX17	AC068790.2	0.615681766	3.93E-57	postive
PIK3CG	AC068790.2	0.525233473	2.86E-39	postive
JAK2	AC068790.2	0.639542048	7.19E-63	postive
TXK	AC068790.2	0.562264368	6.39E-46	postive
PPP3CB	AC068790.2	0.502789397	1.28E-35	postive
NFAT5	AC068790.2	0.880788197	3.50E-175	postive
NFATC3	AC068790.2	0.535497434	4.96E-41	postive
MALT1	AC068790.2	0.537130966	2.57E-41	postive
PIK3R1	AC068790.2	0.627944784	5.11E-60	postive
PIK3CA	AC068790.2	0.685931688	1.29E-75	postive
PIK3CB	AC068790.2	0.535612457	4.73E-41	postive
AKT3	AC068790.2	0.552405093	4.55E-44	postive
RASGRP3	AC068790.2	0.531026437	2.95E-40	postive
IGHD4-4	AC068790.2	0.524564886	3.71E-39	postive
IGHD6-6	AC068790.2	0.504826027	6.11E-36	postive
PLXNC1	AC068790.2	0.524838766	3.34E-39	postive
GNRH1	AC068790.2	0.766537371	1.38E-104	postive
IL6ST	AC068790.2	0.612517627	2.08E-56	postive
RABEP1	AC068790.2	0.587279799	6.52E-51	postive
ACVR2A	AC068790.2	0.67665687	7.03E-73	postive
ANGPTL1	AC068790.2	0.745016382	8.72E-96	postive
BMPR1A	AC068790.2	0.57697579	8.36E-49	postive
BMPR2	AC068790.2	0.717835217	7.30E-86	postive
CRLF3	AC068790.2	0.68373956	5.85E-75	postive

NR1D2	AC068790.2	0.509051187	1.30E-36	postive
NR2C2	AC068790.2	0.729333076	6.51E-90	postive
RORA	AC068790.2	0.820659658	1.20E-131	postive
SOS1	AC068790.2	0.72304173	1.13E-87	postive
SOS2	AC068790.2	0.614122964	8.96E-57	postive
BRAF	AC068790.2	0.824983037	3.37E-134	postive
CBL	AC068790.2	0.605155994	9.39E-55	postive
CBLB	AC068790.2	0.67764647	3.63E-73	postive
RASGRP1	AC068790.2	0.536201087	3.74E-41	postive
PDK1	AC068790.2	0.571924884	8.48E-48	postive
TRAJ1	AC068790.2	0.845579135	2.12E-147	postive
TRAJ2	AC068790.2	0.769452883	7.52E-106	postive
TRAJ3	AC068790.2	0.854563216	8.82E-154	postive
TRAJ5	AC068790.2	0.884754936	7.40E-179	postive
TRAJ6	AC068790.2	0.884520272	1.23E-178	postive
TRAJ8	AC068790.2	0.886956894	5.89E-181	postive
TRAJ10	AC068790.2	0.858730705	6.89E-157	postive
TRAJ12	AC068790.2	0.815714837	8.24E-129	postive
TRAJ13	AC068790.2	0.888672748	1.27E-182	postive
TRAJ14	AC068790.2	0.873672174	6.59E-169	postive
TRAJ16	AC068790.2	0.869914318	9.58E-166	postive
TRAJ17	AC068790.2	0.853888028	2.75E-153	postive
TRAJ18	AC068790.2	0.784707539	8.67E-113	postive
TRAJ21	AC068790.2	0.896816965	6.40E-191	postive
TRAJ31	AC068790.2	0.912522488	4.38E-209	postive
TRAJ37	AC068790.2	0.891842012	9.00E-186	postive
TRAJ38	AC068790.2	0.918689613	3.55E-217	postive
TRAJ39	AC068790.2	0.918804182	2.47E-217	postive
CREB1	AL133330.1	0.765429238	4.14E-104	postive
RFXAP	AL133330.1	0.509248582	1.21E-36	postive
UBR1	AL133330.1	0.598501272	2.70E-53	postive
ZC3HAV1	AL133330.1	0.568259095	4.45E-47	postive
TLR4	AL133330.1	0.521138088	1.39E-38	postive
IL15	AL133330.1	0.674647379	2.67E-72	postive
CYLD	AL133330.1	0.667669068	2.53E-70	postive
EIF2AK2	AL133330.1	0.556737648	7.10E-45	postive
TLR1	AL133330.1	0.515680271	1.11E-37	postive
NFKBIZ	AL133330.1	0.502364786	1.49E-35	postive
LMBR1	AL133330.1	0.541447757	4.43E-42	postive
LIMS1	AL133330.1	0.52085207	1.55E-38	postive
IREB2	AL133330.1	0.549746773	1.40E-43	postive
DDX17	AL133330.1	0.61871025	7.83E-58	postive
PIK3CG	AL133330.1	0.583614558	3.74E-50	postive
JAK2	AL133330.1	0.694614905	2.85E-78	postive
TXK	AL133330.1	0.581231738	1.15E-49	postive
NFAT5	AL133330.1	0.843829827	3.31E-146	postive
MALT1	AL133330.1	0.561235657	1.00E-45	postive
PIK3R1	AL133330.1	0.644609918	3.73E-64	postive
PIK3CA	AL133330.1	0.712149042	6.19E-84	postive
PIK3CB	AL133330.1	0.559204041	2.44E-45	postive
RASGRP3	AL133330.1	0.576527643	1.03E-48	postive
IGHD6-6	AL133330.1	0.538419506	1.52E-41	postive
PLXNC1	AL133330.1	0.56617863	1.13E-46	postive
GNRH1	AL133330.1	0.7026249	8.31E-81	postive
IL6ST	AL133330.1	0.616441922	2.63E-57	postive
IL7	AL133330.1	0.553971948	2.33E-44	postive
RABEP1	AL133330.1	0.52855702	7.82E-40	postive
ACVR2A	AL133330.1	0.609108944	1.23E-55	postive
ANGPTL1	AL133330.1	0.764637415	9.04E-104	postive
BMPR2	AL133330.1	0.683803626	5.59E-75	postive
CRLF3	AL133330.1	0.626932205	8.94E-60	postive
IL18R1	AL133330.1	0.537075176	2.63E-41	postive
NR2C2	AL133330.1	0.704902329	1.52E-81	postive
RORA	AL133330.1	0.82567384	1.30E-134	postive
SOS1	AL133330.1	0.66020338	2.89E-68	postive

SOS2	AL133330.1	0.600718971	8.89E-54	postive
BRAF	AL133330.1	0.766112834	2.11E-104	postive
PTPRC	AL133330.1	0.527160552	1.35E-39	postive
ITK	AL133330.1	0.582646382	5.91E-50	postive
CD28	AL133330.1	0.544752747	1.13E-42	postive
CBL	AL133330.1	0.633214048	2.68E-61	postive
CBLB	AL133330.1	0.69356476	6.04E-78	postive
RASGRP1	AL133330.1	0.566397998	1.02E-46	postive
PDK1	AL133330.1	0.57394362	3.38E-48	postive
TRAJ1	AL133330.1	0.821741806	2.80E-132	postive
TRAJ2	AL133330.1	0.753849696	2.75E-99	postive
TRAJ3	AL133330.1	0.864948779	1.03E-161	postive
TRAJ5	AL133330.1	0.866859663	3.02E-163	postive
TRAJ6	AL133330.1	0.861025458	1.21E-158	postive
TRAJ8	AL133330.1	0.811478211	1.90E-126	postive
TRAJ10	AL133330.1	0.873005899	2.44E-168	postive
TRAJ12	AL133330.1	0.800506159	1.34E-120	postive
TRAJ13	AL133330.1	0.836208193	3.63E-141	postive
TRAJ14	AL133330.1	0.844599409	9.92E-147	postive
TRAJ16	AL133330.1	0.873248852	1.51E-168	postive
TRAJ17	AL133330.1	0.865662091	2.77E-162	postive
TRAJ18	AL133330.1	0.861592802	4.42E-159	postive
TRAJ21	AL133330.1	0.888924901	7.20E-183	postive
TRAJ31	AL133330.1	0.87824634	6.77E-173	postive
TRAJ37	AL133330.1	0.84847264	2.07E-149	postive
TRAJ38	AL133330.1	0.898114276	2.63E-192	postive
TRAJ39	AL133330.1	0.893792011	9.27E-188	postive
NFAT5	AC004596.1	0.533459788	1.12E-40	postive
PIK3CA	AC004596.1	0.508729998	1.47E-36	postive
ANGPTL1	AC004596.1	0.500151433	3.29E-35	postive
NR2C2	AC004596.1	0.554088613	2.22E-44	postive
SOS1	AC004596.1	0.530636255	3.45E-40	postive
BRAF	AC004596.1	0.5079976	1.92E-36	postive
TRAJ1	AC004596.1	0.641169802	2.80E-63	postive
TRAJ3	AC004596.1	0.608324961	1.85E-55	postive
TRAJ5	AC004596.1	0.643713797	6.32E-64	postive
TRAJ6	AC004596.1	0.619121025	6.28E-58	postive
TRAJ8	AC004596.1	0.646826699	1.00E-64	postive
TRAJ10	AC004596.1	0.616278849	2.86E-57	postive
TRAJ12	AC004596.1	0.605426391	8.18E-55	postive
TRAJ13	AC004596.1	0.616632815	2.37E-57	postive
TRAJ14	AC004596.1	0.626692847	1.02E-59	postive
TRAJ16	AC004596.1	0.655426538	5.57E-67	postive
TRAJ17	AC004596.1	0.683006565	9.66E-75	postive
TRAJ18	AC004596.1	0.577575084	6.34E-49	postive
TRAJ21	AC004596.1	0.640283657	4.68E-63	postive
TRAJ31	AC004596.1	0.619316353	5.65E-58	postive
TRAJ37	AC004596.1	0.570177066	1.87E-47	postive
TRAJ38	AC004596.1	0.614371611	7.86E-57	postive
TRAJ39	AC004596.1	0.65719411	1.87E-67	postive
HLA-G	AL109615.3	0.557317901	5.53E-45	postive
CREB1	AC130650.2	0.728368108	1.45E-89	postive
UBR1	AC130650.2	0.58769999	5.33E-51	postive
ZC3HAV1	AC130650.2	0.512917187	3.11E-37	postive
ZC3HAV1L	AC130650.2	0.537727683	2.02E-41	postive
IL15	AC130650.2	0.605767034	6.87E-55	postive
CYLD	AC130650.2	0.522436446	8.45E-39	postive
EIF2AK2	AC130650.2	0.505008703	5.72E-36	postive
MAPK8	AC130650.2	0.55932453	2.31E-45	postive
NFKBIZ	AC130650.2	0.517261738	6.09E-38	postive
LMBR1	AC130650.2	0.564323938	2.57E-46	postive
IREB2	AC130650.2	0.59275368	4.61E-52	postive
DDX17	AC130650.2	0.53315324	1.27E-40	postive
JAK2	AC130650.2	0.57514197	1.95E-48	postive
TXK	AC130650.2	0.539398081	1.02E-41	postive

NFAT5	AC130650.2	0.752861614	6.89E-99	postive
MALT1	AC130650.2	0.518444457	3.89E-38	postive
PIK3R1	AC130650.2	0.624217054	3.97E-59	postive
PIK3CA	AC130650.2	0.636499838	4.13E-62	postive
PIK3CB	AC130650.2	0.511916024	4.52E-37	postive
RASGRP3	AC130650.2	0.500442038	2.97E-35	postive
IGHD4-4	AC130650.2	0.540585262	6.30E-42	postive
GNRH1	AC130650.2	0.668456219	1.53E-70	postive
IL6ST	AC130650.2	0.639027652	9.68E-63	postive
ACVR2A	AC130650.2	0.588157338	4.28E-51	postive
ANGPTL1	AC130650.2	0.744449603	1.45E-95	postive
BMPR1A	AC130650.2	0.527659616	1.11E-39	postive
BMPR2	AC130650.2	0.678476274	2.08E-73	postive
CRLF3	AC130650.2	0.596075988	9.00E-53	postive
NR2C2	AC130650.2	0.649939355	1.56E-65	postive
RORA	AC130650.2	0.815544744	1.03E-128	postive
SOS1	AC130650.2	0.620472597	3.04E-58	postive
SOS2	AC130650.2	0.538710968	1.35E-41	postive
BRAF	AC130650.2	0.778471399	6.94E-110	postive
ITK	AC130650.2	0.502357728	1.49E-35	postive
CBL	AC130650.2	0.536917009	2.80E-41	postive
CBLB	AC130650.2	0.689421681	1.13E-76	postive
PDK1	AC130650.2	0.600265156	1.12E-53	postive
TRAJ1	AC130650.2	0.60914458	1.21E-55	postive
TRAJ2	AC130650.2	0.648821416	3.06E-65	postive
TRAJ3	AC130650.2	0.694742344	2.60E-78	postive
TRAJ5	AC130650.2	0.734137678	1.14E-91	postive
TRAJ6	AC130650.2	0.68086953	4.14E-74	postive
TRAJ8	AC130650.2	0.649966339	1.54E-65	postive
TRAJ10	AC130650.2	0.698828982	1.35E-79	postive
TRAJ12	AC130650.2	0.613623647	1.17E-56	postive
TRAJ13	AC130650.2	0.684008185	4.86E-75	postive
TRAJ14	AC130650.2	0.667202436	3.42E-70	postive
TRAJ16	AC130650.2	0.66240842	7.23E-69	postive
TRAJ17	AC130650.2	0.65861392	7.78E-68	postive
TRAJ18	AC130650.2	0.635869825	5.93E-62	postive
TRAJ21	AC130650.2	0.7138436	1.67E-84	postive
TRAJ31	AC130650.2	0.765783318	2.92E-104	postive
TRAJ37	AC130650.2	0.740201758	6.15E-94	postive
TRAJ38	AC130650.2	0.778588116	6.13E-110	postive
TRAJ39	AC130650.2	0.742062092	1.20E-94	postive
CREB1	AC005519.1	0.608888335	1.38E-55	postive
RFXAP	AC005519.1	0.5775553	6.39E-49	postive
CYLD	AC005519.1	0.507350315	2.43E-36	postive
NFKBIZ	AC005519.1	0.53801608	1.79E-41	postive
DDX17	AC005519.1	0.730528848	2.40E-90	postive
IRF9	AC005519.1	0.569232435	2.87E-47	postive
JAK2	AC005519.1	0.524201329	4.27E-39	postive
TXK	AC005519.1	0.562851212	4.93E-46	postive
NFAT5	AC005519.1	0.658908082	6.48E-68	postive
PIK3R1	AC005519.1	0.512472625	3.67E-37	postive
PIK3CA	AC005519.1	0.514086947	2.01E-37	postive
LTB4R2	AC005519.1	0.636391939	4.40E-62	postive
GNRH1	AC005519.1	0.78724528	5.35E-114	postive
RABEP1	AC005519.1	0.510346293	8.08E-37	postive
ACVR2A	AC005519.1	0.546560362	5.34E-43	postive
ANGPTL1	AC005519.1	0.65186467	4.89E-66	postive
BMPR2	AC005519.1	0.532400614	1.71E-40	postive
CRLF3	AC005519.1	0.511116825	6.08E-37	postive
NR2C1	AC005519.1	0.621552882	1.69E-58	postive
NR2C2	AC005519.1	0.73484672	6.26E-92	postive
RORA	AC005519.1	0.667628177	2.60E-70	postive
SOS1	AC005519.1	0.581246004	1.14E-49	postive
SOS2	AC005519.1	0.533372248	1.16E-40	postive
BRAF	AC005519.1	0.655161529	6.55E-67	postive

ITK	AC005519.1	0.501048046	2.39E-35	postive
CBL	AC005519.1	0.565410869	1.59E-46	postive
CBLB	AC005519.1	0.630520522	1.22E-60	postive
PDK1	AC005519.1	0.519294721	2.81E-38	postive
TRAJ1	AC005519.1	0.573808411	3.59E-48	postive
TRAJ2	AC005519.1	0.562153234	6.71E-46	postive
TRAJ3	AC005519.1	0.611619667	3.33E-56	postive
TRAJ5	AC005519.1	0.574917621	2.16E-48	postive
TRAJ6	AC005519.1	0.563074044	4.47E-46	postive
TRAJ8	AC005519.1	0.514279066	1.87E-37	postive
TRAJ10	AC005519.1	0.544744385	1.14E-42	postive
TRAJ13	AC005519.1	0.533153226	1.27E-40	postive
TRAJ14	AC005519.1	0.525087188	3.03E-39	postive
TRAJ16	AC005519.1	0.547102352	4.26E-43	postive
TRAJ17	AC005519.1	0.51654591	7.98E-38	postive
TRAJ18	AC005519.1	0.534450332	7.55E-41	postive
TRAJ21	AC005519.1	0.563389389	3.89E-46	postive
TRAJ31	AC005519.1	0.567174447	7.23E-47	postive
TRAJ37	AC005519.1	0.569544902	2.49E-47	postive
TRAJ38	AC005519.1	0.60740236	2.97E-55	postive
TRAJ39	AC005519.1	0.577550408	6.41E-49	postive
CXCR6	DTNB-AS1	0.522715422	7.59E-39	postive
CD79A	DTNB-AS1	0.543614739	1.81E-42	postive
CD79B	DTNB-AS1	0.511970928	4.43E-37	postive
IGHG1	DTNB-AS1	0.58030281	1.78E-49	postive
IGHV3-15	DTNB-AS1	0.508423247	1.64E-36	postive
IGHV3-21	DTNB-AS1	0.579298447	2.84E-49	postive
IGHV3-23	DTNB-AS1	0.580532377	1.60E-49	postive
IGHV3-30	DTNB-AS1	0.500629181	2.77E-35	postive
IGHV3-74	DTNB-AS1	0.527265998	1.30E-39	postive
IGHV5-51	DTNB-AS1	0.526533982	1.73E-39	postive
IGKC	DTNB-AS1	0.698880203	1.30E-79	postive
IGKJ5	DTNB-AS1	0.697395779	3.84E-79	postive
IGKV1-12	DTNB-AS1	0.512132145	4.17E-37	postive
IGKV1-39	DTNB-AS1	0.511012877	6.32E-37	postive
IGKV1-5	DTNB-AS1	0.665570656	9.72E-70	postive
IGKV1-9	DTNB-AS1	0.521609142	1.16E-38	postive
IGKV1D-39	DTNB-AS1	0.520515846	1.76E-38	postive
IGKV2D-28	DTNB-AS1	0.512459979	3.69E-37	postive
IGKV3-11	DTNB-AS1	0.597139951	5.31E-53	postive
IGKV3-15	DTNB-AS1	0.620840205	2.49E-58	postive
IGKV3-20	DTNB-AS1	0.657056193	2.04E-67	postive
IGKV3-7	DTNB-AS1	0.510023738	9.11E-37	postive
IGKV3D-11	DTNB-AS1	0.534581914	7.16E-41	postive
IGKV3D-20	DTNB-AS1	0.51318288	2.82E-37	postive
IGKV4-1	DTNB-AS1	0.636786951	3.51E-62	postive
IGLC2	DTNB-AS1	0.593946012	2.57E-52	postive
IGLC3	DTNB-AS1	0.608753744	1.48E-55	postive
IGLV1-44	DTNB-AS1	0.577109037	7.86E-49	postive
IGLV2-14	DTNB-AS1	0.55749078	5.13E-45	postive
IGLV3-1	DTNB-AS1	0.53176962	2.20E-40	postive
IGLV3-21	DTNB-AS1	0.537918951	1.87E-41	postive
IGLV3-25	DTNB-AS1	0.525422472	2.66E-39	postive
IGLV6-57	DTNB-AS1	0.556948977	6.48E-45	postive
IGLV8-61	DTNB-AS1	0.575236809	1.87E-48	postive
PNOC	DTNB-AS1	0.58542067	1.59E-50	postive
TNFSF13B	DTNB-AS1	0.561697043	8.19E-46	postive
TNFRSF17	DTNB-AS1	0.792567108	1.37E-116	postive
CD48	DTNB-AS1	0.627814158	5.49E-60	postive
SH2D1A	DTNB-AS1	0.600702446	8.97E-54	postive
PDK1	DTNB-AS1	0.631367107	7.57E-61	postive
TRAV16	DTNB-AS1	0.526463308	1.77E-39	postive
CREB1	AC009120.2	0.50608813	3.86E-36	postive
RFXAP	AC009120.2	0.515634061	1.12E-37	postive
CYLD	AC009120.2	0.562474478	5.82E-46	postive

DDX17	AC009120.2	0.726268593	8.18E-89	postive
NFAT5	AC009120.2	0.744378915	1.54E-95	postive
NFATC3	AC009120.2	0.541048789	5.21E-42	postive
LTB4R2	AC009120.2	0.504150754	7.80E-36	postive
GNRH1	AC009120.2	0.77658055	5.04E-109	postive
ACVR2A	AC009120.2	0.505236472	5.26E-36	postive
NR2C1	AC009120.2	0.579811603	2.24E-49	postive
NR2C2	AC009120.2	0.691068137	3.56E-77	postive
RORA	AC009120.2	0.671408424	2.24E-71	postive
BRAF	AC009120.2	0.535393637	5.17E-41	postive
TRAJ1	AC009120.2	0.63787273	1.88E-62	postive
TRAJ2	AC009120.2	0.58589021	1.27E-50	postive
TRAJ3	AC009120.2	0.634063277	1.66E-61	postive
TRAJ5	AC009120.2	0.60187344	4.97E-54	postive
TRAJ6	AC009120.2	0.634610155	1.21E-61	postive
TRAJ8	AC009120.2	0.574452893	2.67E-48	postive
TRAJ10	AC009120.2	0.638005789	1.74E-62	postive
TRAJ12	AC009120.2	0.584605555	2.34E-50	postive
TRAJ13	AC009120.2	0.602767894	3.16E-54	postive
TRAJ14	AC009120.2	0.579886833	2.16E-49	postive
TRAJ16	AC009120.2	0.628677559	3.40E-60	postive
TRAJ17	AC009120.2	0.587888197	4.87E-51	postive
TRAJ18	AC009120.2	0.622516452	1.00E-58	postive
TRAJ21	AC009120.2	0.617791909	1.28E-57	postive
TRAJ31	AC009120.2	0.634406214	1.36E-61	postive
TRAJ37	AC009120.2	0.67241417	1.16E-71	postive
TRAJ38	AC009120.2	0.637756487	2.01E-62	postive
TRAJ39	AC009120.2	0.632735629	3.51E-61	postive
CREB1	AL049840.5	0.517398286	5.78E-38	postive
ZC3HAV1	AL049840.5	0.537052046	2.65E-41	postive
TRAF3	AL049840.5	0.575390096	1.74E-48	postive
DDX17	AL049840.5	0.540693054	6.03E-42	postive
LTB4R2	AL049840.5	0.523123611	6.48E-39	postive
GNRH1	AL049840.5	0.572869438	5.52E-48	postive
NR2C2	AL049840.5	0.578758906	3.65E-49	postive
BRAF	AL049840.5	0.513463417	2.54E-37	postive
CBL	AL049840.5	0.570419925	1.68E-47	postive
DDX17	AC048341.2	0.609078971	1.25E-55	postive
TXK	AC048341.2	0.550093531	1.21E-43	postive
NFAT5	AC048341.2	0.589682772	2.05E-51	postive
GNRH1	AC048341.2	0.792427901	1.61E-116	postive
NR2C1	AC048341.2	0.512639739	3.45E-37	postive
NR2C2	AC048341.2	0.651666051	5.51E-66	postive
RORA	AC048341.2	0.591356503	9.11E-52	postive
BRAF	AC048341.2	0.568978634	3.22E-47	postive
TEC	AC048341.2	0.597924015	3.60E-53	postive
CBLB	AC048341.2	0.538731625	1.34E-41	postive
TRAJ1	AC048341.2	0.513207885	2.79E-37	postive
TRAJ3	AC048341.2	0.521396899	1.26E-38	postive
TRAJ5	AC048341.2	0.524928741	3.22E-39	postive
TRAJ37	AC048341.2	0.501360102	2.13E-35	postive
TRAJ38	AC048341.2	0.524551577	3.73E-39	postive
TRAJ39	AC048341.2	0.554224578	2.09E-44	postive
IRF9	AC020907.4	0.533073031	1.31E-40	postive
NFAT5	AC018645.3	0.544504078	1.26E-42	postive
TRAJ6	AC018645.3	0.516513637	8.07E-38	postive
TRAJ8	AC018645.3	0.511303053	5.67E-37	postive
TRAJ31	AC018645.3	0.510123823	8.78E-37	postive
TRAJ37	AC018645.3	0.520328838	1.90E-38	postive
TRAJ38	AC018645.3	0.506568362	3.24E-36	postive
CREB1	DNM3OS	0.766989856	8.84E-105	postive
UBR1	DNM3OS	0.575737162	1.48E-48	postive
ZC3HAV1	DNM3OS	0.51012084	8.79E-37	postive
IL15	DNM3OS	0.565482716	1.54E-46	postive
CYLD	DNM3OS	0.607328078	3.09E-55	postive

EIF2AK2	DNM3OS	0.501629812	1.94E-35	postive
PDGFRA	DNM3OS	0.554361231	1.97E-44	postive
TLR1	DNM3OS	0.538606793	1.41E-41	postive
LMBR1	DNM3OS	0.531450493	2.50E-40	postive
LIMS1	DNM3OS	0.560151728	1.61E-45	postive
IREB2	DNM3OS	0.546533244	5.40E-43	postive
DDX17	DNM3OS	0.572900971	5.44E-48	postive
PIK3CG	DNM3OS	0.564286525	2.62E-46	postive
JAK2	DNM3OS	0.686585621	8.21E-76	postive
TXK	DNM3OS	0.615061773	5.46E-57	postive
NFAT5	DNM3OS	0.775130282	2.28E-108	postive
MALT1	DNM3OS	0.522910316	7.04E-39	postive
PIK3R1	DNM3OS	0.623616341	5.52E-59	postive
PIK3CA	DNM3OS	0.674358113	3.23E-72	postive
AKT3	DNM3OS	0.508945601	1.36E-36	postive
RASGRP3	DNM3OS	0.583362326	4.21E-50	postive
PLXNC1	DNM3OS	0.615589101	4.13E-57	postive
FGF7	DNM3OS	0.547887945	3.07E-43	postive
GNRH1	DNM3OS	0.687654861	3.90E-76	postive
IL6ST	DNM3OS	0.587512082	5.83E-51	postive
IL7	DNM3OS	0.509802922	9.88E-37	postive
RABEP1	DNM3OS	0.522723335	7.56E-39	postive
ACVR2A	DNM3OS	0.646235247	1.43E-64	postive
ANGPTL1	DNM3OS	0.715570104	4.34E-85	postive
BMPR1A	DNM3OS	0.51865558	3.59E-38	postive
BMPR2	DNM3OS	0.712991117	3.23E-84	postive
CRLF3	DNM3OS	0.631084793	8.87E-61	postive
NR2C2	DNM3OS	0.643716675	6.31E-64	postive
RORA	DNM3OS	0.783219715	4.36E-112	postive
SOS1	DNM3OS	0.63613133	5.10E-62	postive
SOS2	DNM3OS	0.580746864	1.44E-49	postive
BRAF	DNM3OS	0.709356534	5.27E-83	postive
CD28	DNM3OS	0.523189452	6.32E-39	postive
CBL	DNM3OS	0.553521407	2.83E-44	postive
CBLB	DNM3OS	0.725690761	1.31E-88	postive
RASGRP1	DNM3OS	0.508875153	1.39E-36	postive
PDK1	DNM3OS	0.567940732	5.13E-47	postive
TRAJ1	DNM3OS	0.708771678	8.23E-83	postive
TRAJ2	DNM3OS	0.686937615	6.43E-76	postive
TRAJ3	DNM3OS	0.709325681	5.40E-83	postive
TRAJ5	DNM3OS	0.755424821	6.30E-100	postive
TRAJ6	DNM3OS	0.747857808	6.77E-97	postive
TRAJ8	DNM3OS	0.694293484	3.59E-78	postive
TRAJ10	DNM3OS	0.738794941	2.09E-93	postive
TRAJ12	DNM3OS	0.685705938	1.51E-75	postive
TRAJ13	DNM3OS	0.72255647	1.68E-87	postive
TRAJ14	DNM3OS	0.719362189	2.17E-86	postive
TRAJ16	DNM3OS	0.753987973	2.42E-99	postive
TRAJ17	DNM3OS	0.693639967	5.73E-78	postive
TRAJ18	DNM3OS	0.701429675	2.01E-80	postive
TRAJ21	DNM3OS	0.74469406	1.16E-95	postive
TRAJ31	DNM3OS	0.78039862	9.00E-111	postive
TRAJ37	DNM3OS	0.736835623	1.14E-92	postive
TRAJ38	DNM3OS	0.8160286	5.48E-129	postive
TRAJ39	DNM3OS	0.756184595	3.08E-100	postive
DDX17	AC093227.1	0.599648521	1.52E-53	postive
NFAT5	AC093227.1	0.511471731	5.33E-37	postive
GNRH1	AC093227.1	0.596874764	6.06E-53	postive
IL6ST	AC093227.1	0.504734337	6.32E-36	postive
NR2C1	AC093227.1	0.514524123	1.71E-37	postive
NR2C2	AC093227.1	0.604234646	1.50E-54	postive
RORA	AC093227.1	0.54622943	6.13E-43	postive
BRAF	AC093227.1	0.547219033	4.06E-43	postive
CREB1	AC020913.3	0.749803811	1.15E-97	postive
UBR1	AC020913.3	0.577250138	7.36E-49	postive

ZC3HAV1	AC020913.3	0.559543885	2.10E-45	postive
ZC3HAV1L	AC020913.3	0.511214667	5.86E-37	postive
IL15	AC020913.3	0.627164772	7.86E-60	postive
CYLD	AC020913.3	0.573314913	4.50E-48	postive
EIF2AK2	AC020913.3	0.535252919	5.47E-41	postive
MAPK8	AC020913.3	0.536154782	3.81E-41	postive
TLR1	AC020913.3	0.547456776	3.67E-43	postive
NFKBIZ	AC020913.3	0.540056155	7.82E-42	postive
LMBR1	AC020913.3	0.573164348	4.82E-48	postive
LIMS1	AC020913.3	0.547653069	3.38E-43	postive
IREB2	AC020913.3	0.561846845	7.67E-46	postive
DDX17	AC020913.3	0.570926645	1.33E-47	postive
PIK3CG	AC020913.3	0.562601183	5.51E-46	postive
JAK2	AC020913.3	0.675919764	1.15E-72	postive
TXK	AC020913.3	0.579830952	2.22E-49	postive
NFAT5	AC020913.3	0.765481404	3.94E-104	postive
MALT1	AC020913.3	0.550801681	8.99E-44	postive
PIK3R1	AC020913.3	0.612027457	2.69E-56	postive
PIK3CA	AC020913.3	0.713629911	1.97E-84	postive
PIK3CB	AC020913.3	0.53808966	1.74E-41	postive
AKT3	AC020913.3	0.521131722	1.39E-38	postive
RASGRP3	AC020913.3	0.548630605	2.24E-43	postive
GNRH1	AC020913.3	0.68439454	3.73E-75	postive
IL6ST	AC020913.3	0.599770132	1.43E-53	postive
RABEP1	AC020913.3	0.529032425	6.49E-40	postive
ACVR2A	AC020913.3	0.564137918	2.79E-46	postive
ANGPTL1	AC020913.3	0.819422627	6.27E-131	postive
BMPR2	AC020913.3	0.674399282	3.14E-72	postive
CRLF3	AC020913.3	0.646132498	1.52E-64	postive
IL18R1	AC020913.3	0.541211461	4.88E-42	postive
NR2C2	AC020913.3	0.658962725	6.26E-68	postive
RORA	AC020913.3	0.796803623	1.05E-118	postive
SOS1	AC020913.3	0.655673051	4.79E-67	postive
SOS2	AC020913.3	0.583095052	4.78E-50	postive
BRAF	AC020913.3	0.760832489	3.67E-102	postive
ITK	AC020913.3	0.546498505	5.48E-43	postive
CBL	AC020913.3	0.638161561	1.59E-62	postive
CBLB	AC020913.3	0.766896111	9.70E-105	postive
RASGRP1	AC020913.3	0.510974391	6.41E-37	postive
PDK1	AC020913.3	0.632584315	3.82E-61	postive
TRAJ1	AC020913.3	0.686060557	1.18E-75	postive
TRAJ2	AC020913.3	0.661823218	1.05E-68	postive
TRAJ3	AC020913.3	0.728751495	1.05E-89	postive
TRAJ5	AC020913.3	0.791352138	5.45E-116	postive
TRAJ6	AC020913.3	0.738465778	2.79E-93	postive
TRAJ8	AC020913.3	0.692177325	1.62E-77	postive
TRAJ10	AC020913.3	0.748670111	3.24E-97	postive
TRAJ12	AC020913.3	0.634858106	1.05E-61	postive
TRAJ13	AC020913.3	0.745662917	4.89E-96	postive
TRAJ14	AC020913.3	0.728122957	1.78E-89	postive
TRAJ16	AC020913.3	0.748507614	3.75E-97	postive
TRAJ17	AC020913.3	0.723415529	8.38E-88	postive
TRAJ18	AC020913.3	0.69840658	1.84E-79	postive
TRAJ21	AC020913.3	0.782438015	1.01E-111	postive
TRAJ31	AC020913.3	0.785733376	2.83E-113	postive
TRAJ37	AC020913.3	0.733010548	2.98E-91	postive
TRAJ38	AC020913.3	0.820003979	2.89E-131	postive
TRAJ39	AC020913.3	0.790168259	2.07E-115	postive
ZC3HAV1	LINC02035	0.507646503	2.18E-36	postive
DDX17	LINC02035	0.517404011	5.77E-38	postive
PIK3CA	LINC02035	0.523355753	5.93E-39	postive
GSK3B	LINC02035	0.556260585	8.73E-45	postive
GNRH1	LINC02035	0.535567334	4.82E-41	postive
BMPR2	LINC02035	0.549203802	1.76E-43	postive
NR2C2	LINC02035	0.61852119	8.66E-58	postive

RORA	LINC02035	0.535833917	4.33E-41	postive
SOS1	LINC02035	0.524635908	3.61E-39	postive
BRAF	LINC02035	0.541704921	3.98E-42	postive
CBLB	LINC02035	0.628394842	3.98E-60	postive
DDX17	AC009022.1	0.640564454	3.98E-63	postive
NFAT5	AC009022.1	0.597341304	4.81E-53	postive
GNRH1	AC009022.1	0.69034708	5.92E-77	postive
NR2C1	AC009022.1	0.541996315	3.53E-42	postive
NR2C2	AC009022.1	0.63043808	1.27E-60	postive
RORA	AC009022.1	0.53777769	1.98E-41	postive
CAMP	AL139260.1	0.630748538	1.07E-60	postive
LTB4R2	AC026471.4	0.501900689	1.76E-35	postive
CHGA	AL662844.4	0.526841352	1.53E-39	postive
GRP	AL662844.4	0.547093655	4.27E-43	postive

Supplementary Table S2. DEirlncRNAs between LUAD and normal controls.

gene	conMean	treatMean	logFC	pValue	fdr
LANCL1-AS1	3.907684383	0.378268854	-3.368830049	1.45E-34	1.21E-31
PCAT19	6.474671692	1.324507045	-2.289351523	1.96E-33	3.37E-31
MIR3945HG	5.146101133	0.498959062	-3.366486453	2.09E-33	3.37E-31
FENDRR	9.043970353	0.687331329	-3.717878637	2.42E-33	3.37E-31
AC093110.1	10.10275924	1.241613177	-3.024461693	1.98E-33	3.37E-31
AL590226.1	6.109808294	0.516321034	-3.564786836	1.94E-33	3.37E-31
SMIM25	22.76153469	4.551109321	-2.322307688	8.09E-33	9.63E-31
LINC02014	0.08493322	1.40999085	4.053213045	1.66E-32	1.73E-30
LINC02154	4.25089949	0.430478803	-3.303754043	3.66E-32	3.39E-30
AC026369.3	8.662691499	0.968303583	-3.161284003	7.69E-32	6.41E-30
PARAL1	5.824633046	0.804344043	-2.856282538	3.59E-31	2.72E-29
AC245041.2	4.152364412	0.775287238	-2.421130239	1.56E-30	1.00E-28
AP001189.3	3.510269676	0.691992542	-2.342753476	1.46E-29	8.11E-28
AL035409.1	1.683952264	0.381474512	-2.142192669	8.47E-29	3.92E-27
LINC01936	4.920278576	1.008363826	-2.286723731	1.83E-28	8.04E-27
SNHG4	0.288007961	1.519905113	2.399800663	4.88E-27	1.69E-25
AL157838.1	0.134484062	0.902373158	2.746288946	7.66E-27	2.45E-25
LINC01614	0.232574542	3.240205365	3.800320163	6.77E-25	1.71E-23
AC099850.4	1.16935025	6.587926904	2.494117429	2.11E-24	5.17E-23
AL645608.8	0.030710075	0.574176057	4.224709223	6.48E-24	1.50E-22
LINC00942	0.038498878	3.959325442	6.684294443	1.31E-22	2.66E-21
AL096865.1	0.109627975	0.574192309	2.388918015	3.16E-22	5.85E-21
AC016773.2	0.202776671	0.920454836	2.182455254	3.26E-22	5.91E-21
LINC01607	0.197957364	1.175331401	2.569805959	4.03E-22	7.14E-21
LINC02544	0.0875849	1.16641065	3.735249821	5.04E-22	8.75E-21
U52111.1	0.123317368	0.664095791	2.429015352	1.38E-20	1.99E-19
AC013457.1	5.024030841	0.644786925	-2.961950926	1.96E-20	2.72E-19
LINC02362	0.153430472	1.077997084	2.812696334	3.36E-20	4.52E-19
AC022211.2	0.22026138	1.025491049	2.219026434	5.76E-19	6.85E-18
AC138393.3	0.115124378	0.754191534	2.711737595	6.42E-19	7.37E-18
SCAT2	0.18088244	0.876437493	2.276598841	6.46E-19	7.37E-18
AL031058.1	0.412759422	1.918358337	2.216499179	1.31E-18	1.42E-17
AL109615.3	0.251374571	2.202404117	3.131168594	4.68E-18	4.70E-17
UBE2Q1-AS1	0.154821817	0.644113838	2.056706904	1.01E-17	9.79E-17
ITGB1-DT	0.042921051	0.577216305	3.749354737	1.50E-17	1.43E-16
LINC01765	3.239791511	0.661854509	-2.291314956	2.39E-16	2.10E-15
LUCAT1	0.189314032	2.060883055	3.444409389	3.89E-16	3.27E-15
AC007014.2	0.135486707	0.608573281	2.167279684	7.69E-16	6.21E-15
AC133644.1	0.148761888	0.690118498	2.213839141	6.74E-15	4.53E-14
AL512413.1	0.193378662	0.927181663	2.261423424	7.48E-15	4.90E-14
AL606489.1	0.248377764	1.041898022	2.06860615	9.24E-15	6.01E-14
AL022322.1	0.261066336	1.122059246	2.103660512	1.40E-14	8.87E-14
LINC01842	0.05771041	0.720279625	3.641653617	1.90E-14	1.18E-13
DTNB-AS1	0.272264508	1.470631616	2.433355075	5.18E-14	3.08E-13
SRGAP3-AS2	7.542064767	1.778157051	-2.084576788	1.79E-13	9.88E-13
AP003119.2	0.223084572	0.931607486	2.062131486	1.90E-13	1.04E-12
LINC01559	0.007734279	0.718201636	6.536978319	1.94E-13	1.05E-12
AC026368.1	0.149315375	1.05422264	2.819744947	7.13E-13	3.60E-12
AL109761.1	0.071475759	0.574128913	3.005848777	1.00E-12	4.98E-12
AC006017.1	0.134381931	0.559846353	2.058691773	1.01E-12	4.99E-12
AL049836.1	0.17516206	0.795974864	2.184032551	1.23E-12	5.92E-12
AC004884.2	0.098256482	0.949087101	3.271916004	3.23E-12	1.39E-11
LINC00582	0.137186391	0.61352392	2.160982221	4.39E-12	1.83E-11
AC011511.5	3.15623519	0.596838012	-2.402793387	9.68E-12	3.88E-11
AC004264.1	0.185876733	0.852461131	2.197287862	1.16E-11	4.57E-11
LINC01214	0.013550286	1.247734997	6.524844469	1.94E-11	7.41E-11
NAALADL2-AS2	0.0096358	0.66734983	6.113894927	7.08E-11	2.51E-10
LINC01843	0.232567497	1.136238848	2.288544748	8.23E-11	2.89E-10
AC114488.1	0.111529678	0.549409291	2.300453648	8.90E-11	3.12E-10
AC073316.2	0.040015216	0.702553393	4.133987281	1.39E-10	4.74E-10
HAGLROS	0.130349858	0.79936151	2.6164591	3.22E-10	1.04E-09
LINC02195	0.321205763	1.303775645	2.021125947	6.94E-10	2.14E-09

AC124319.1	0.42085797	1.712462258	2.024666849	1.15E-09	3.39E-09
EGOT	0.083098433	0.579936232	2.803001105	1.92E-09	5.50E-09
LINC01876	0.141652072	0.821091543	2.535191367	4.81E-09	1.30E-08
LINC02036	0.26695629	1.121228082	2.070404338	8.17E-09	2.17E-08
LINC00973	0.023189351	1.881659676	6.342399468	8.63E-09	2.27E-08
AP001429.1	0.077090394	0.820343798	3.41160565	1.86E-08	4.70E-08
HIF1A-AS3	0.301159254	2.231400565	2.889351021	6.46E-08	1.55E-07
AC109446.3	0.187765842	1.473432643	2.972174572	1.61E-07	3.66E-07
AL391427.1	0.124606486	2.061937761	4.048549711	2.18E-07	4.87E-07
AC026355.2	0.367021774	1.632179135	2.152861842	9.02E-07	1.86E-06
AL022313.2	0.10780521	0.885589017	3.038210426	2.08E-06	4.04E-06
PABPC4-AS1	0.191310203	0.807843339	2.078161727	3.70E-06	6.87E-06
MIR9-3HG	0.095720663	0.660890983	2.787510022	6.59E-06	1.20E-05
LINC01116	0.320240344	1.336840964	2.061600872	9.78E-06	1.74E-05
LINC01655	0.46463157	2.083850103	2.165092415	1.77E-05	3.07E-05
AL136115.2	0.155345571	0.704417792	2.180950242	3.62E-05	6.05E-05
AP003119.1	0.055839642	0.724147311	3.696921609	4.56E-05	7.57E-05
AC131971.1	0.054539341	0.678924051	3.63788102	4.79E-05	7.92E-05
LINC00513	0.182436058	1.151758696	2.658375683	5.26E-05	8.62E-05
LINC00941	0.070513865	0.685954439	3.282133901	6.17E-05	0.000100331
AC010547.2	0.087667212	0.546457654	2.640000429	7.28E-05	0.000117934
AC083809.1	0.162554798	5.545109489	5.092217898	0.000203825	0.000318549
DPP10-AS1	0.163251565	1.765552186	3.434950738	0.00022123	0.000344457
AL353804.2	0.144804156	0.679296237	2.229937851	0.000542889	0.000796174
AC063965.2	0.205726719	1.125974984	2.452373696	0.00787465	0.010107217
AC037198.1	0.447695843	2.687739724	2.585802609	0.012661272	0.015907752
MACC1-AS1	0.035264644	0.623943437	4.14512088	0.019508651	0.023863005
AL162724.1	0.119378596	0.577587709	2.274495847	0.020141882	0.024457999
LINC00958	0.17907592	1.045149722	2.545066374	0.030537315	0.036287565

Supplementary Table S3. Coexpression analysis between lncRNAs and immune-related genes in LUSC.

immuneGene	lncRNA	cor	pvalue	Regulation
HGF	AC022733.1	0.633786802	9.58E-58	postive
IGLV3-22	AC144831.1	0.640736431	2.28E-59	postive
WNT5A	LINC02561	0.535875957	1.17E-38	postive
OSGIN1	LINC02561	0.549036553	7.27E-41	postive
NTF4	AC008687.3	0.601225644	1.17E-50	postive
IGKV6D-21	LYPLAL1-AS1	0.555680137	5.12E-42	postive
FGFR3	FEZF1-AS1	0.553839314	1.07E-41	postive
IKBKB	AC090739.1	0.72651419	1.65E-83	postive
SFTPD	LINC02489	0.524571254	7.74E-37	postive
SFTPA1	LINC02489	0.531564407	5.90E-38	postive
SFTPA2	LINC02489	0.518827952	6.13E-36	postive
DLL4	LINC02489	0.501243559	2.72E-33	postive
ARRB1	LINC02489	0.592495594	6.77E-49	postive
C5	LINC02489	0.584468601	2.55E-47	postive
S100A6	BX470102.1	0.555501542	5.51E-42	postive
CD1C	AC093278.2	0.575194148	1.49E-45	postive
CD1E	AC093278.2	0.520523553	3.34E-36	postive
CD4	AC093278.2	0.671537367	4.21E-67	postive
CD74	AC093278.2	0.634029251	8.42E-58	postive
CTSS	AC093278.2	0.574926262	1.67E-45	postive
HLA-DMA	AC093278.2	0.628320988	1.70E-56	postive
HLA-DMB	AC093278.2	0.55080785	3.60E-41	postive
HLA-DOA	AC093278.2	0.609463902	2.25E-52	postive
HLA-DPA1	AC093278.2	0.578118485	4.18E-46	postive
HLA-DPB1	AC093278.2	0.660147336	3.90E-64	postive
HLA-DQA2	AC093278.2	0.520064425	3.94E-36	postive
CIITA	AC093278.2	0.519873645	4.21E-36	postive
IFI30	AC093278.2	0.517051957	1.15E-35	postive
SFTPD	AC093278.2	0.563367909	2.21E-43	postive
COLEC12	AC093278.2	0.566278796	6.57E-44	postive
A2M	AC093278.2	0.668748408	2.31E-66	postive
CYBB	AC093278.2	0.567051312	4.75E-44	postive
TLR7	AC093278.2	0.568043389	3.13E-44	postive
CYLD	AC093278.2	0.5300893	1.02E-37	postive
DLL4	AC093278.2	0.553957724	1.02E-41	postive
IL7R	AC093278.2	0.556566924	3.58E-42	postive
CD40LG	AC093278.2	0.636371723	2.41E-58	postive
TRIM22	AC093278.2	0.61197427	6.59E-53	postive
PDCD1	AC093278.2	0.500010608	4.12E-33	postive
ANXA6	AC093278.2	0.586032671	1.27E-47	postive
CCR8	AC093278.2	0.534584678	1.91E-38	postive
CCR5	AC093278.2	0.600131283	1.95E-50	postive
CCL23	AC093278.2	0.576857149	7.23E-46	postive
CCR1	AC093278.2	0.548548594	8.81E-41	postive
CXCR4	AC093278.2	0.522716499	1.52E-36	postive
CCR4	AC093278.2	0.667546329	4.77E-66	postive
FGR	AC093278.2	0.656146525	4.00E-63	postive
CD86	AC093278.2	0.524549345	7.80E-37	postive
BTK	AC093278.2	0.693497245	3.27E-73	postive
PIK3R5	AC093278.2	0.614691934	1.72E-53	postive
INPP5D	AC093278.2	0.597555737	6.53E-50	postive
LILRB3	AC093278.2	0.559885426	9.27E-43	postive
RASGRP3	AC093278.2	0.661943259	1.35E-64	postive

PRKCB	AC093278.2	0.6339847	8.62E-58	postive
C3	AC093278.2	0.569019928	2.07E-44	postive
SEMA3G	AC093278.2	0.541935667	1.16E-39	postive
CCRL2	AC093278.2	0.544734464	3.92E-40	postive
CMKLR1	AC093278.2	0.58067605	1.36E-46	postive
CX3CR1	AC093278.2	0.512788188	5.17E-35	postive
CXCR3	AC093278.2	0.525611589	5.29E-37	postive
ACKR1	AC093278.2	0.677374413	1.13E-68	postive
PLXNC1	AC093278.2	0.597285505	7.40E-50	postive
ADA2	AC093278.2	0.59734602	7.20E-50	postive
GMFG	AC093278.2	0.587639578	6.15E-48	postive
IL16	AC093278.2	0.643267673	5.69E-60	postive
IL6ST	AC093278.2	0.522091194	1.90E-36	postive
TNFSF12	AC093278.2	0.567895363	3.33E-44	postive
TNFSF14	AC093278.2	0.524776087	7.18E-37	postive
ACVRL1	AC093278.2	0.70948773	5.11E-78	postive
APLNR	AC093278.2	0.585866935	1.36E-47	postive
CSF1R	AC093278.2	0.563259654	2.31E-43	postive
ENG	AC093278.2	0.660426033	3.31E-64	postive
FLT1	AC093278.2	0.51850268	6.88E-36	postive
FLT4	AC093278.2	0.639264227	5.07E-59	postive
IL10RA	AC093278.2	0.672804839	1.93E-67	postive
IL12RB1	AC093278.2	0.549451692	6.17E-41	postive
IL2RG	AC093278.2	0.524371934	8.32E-37	postive
IL3RA	AC093278.2	0.671362786	4.69E-67	postive
NPR1	AC093278.2	0.621722858	5.06E-55	postive
S1PR1	AC093278.2	0.739475064	5.62E-88	postive
TEK	AC093278.2	0.720173869	2.04E-81	postive
TGFBR2	AC093278.2	0.565381857	9.55E-44	postive
TIE1	AC093278.2	0.801259058	1.30E-113	postive
TNFRSF1B	AC093278.2	0.667216639	5.83E-66	postive
ICAM2	AC093278.2	0.535596629	1.30E-38	postive
ITGAL	AC093278.2	0.623433459	2.11E-55	postive
ITGB2	AC093278.2	0.557897418	2.09E-42	postive
NCR3	AC093278.2	0.536249385	1.02E-38	postive
CD247	AC093278.2	0.595885058	1.42E-49	postive
LCP2	AC093278.2	0.596679113	9.82E-50	postive
HCST	AC093278.2	0.522245103	1.80E-36	postive
CD3E	AC093278.2	0.599380099	2.78E-50	postive
PTPRC	AC093278.2	0.670955716	6.02E-67	postive
ITK	AC093278.2	0.669775833	1.24E-66	postive
GRAP2	AC093278.2	0.58235246	6.51E-47	postive
CD28	AC093278.2	0.652709873	2.87E-62	postive
ICOS	AC093278.2	0.584576627	2.43E-47	postive
CTLA4	AC093278.2	0.506318927	4.86E-34	postive
TRAC	AC093278.2	0.575188204	1.49E-45	postive
TRAV2	AC093278.2	0.51297237	4.85E-35	postive
TRAV8-3	AC093278.2	0.562061606	3.79E-43	postive
TRAV8-6	AC093278.2	0.541565844	1.34E-39	postive
TRAV9-2	AC093278.2	0.542944953	7.86E-40	postive
TRAV13-1	AC093278.2	0.535926141	1.15E-38	postive
TRBC1	AC093278.2	0.583732982	3.53E-47	postive
TRBC2	AC093278.2	0.545424353	3.00E-40	postive
TRBJ2-7	AC093278.2	0.507971358	2.75E-34	postive
TRBV3-1	AC093278.2	0.520125246	3.85E-36	postive
TRBV5-1	AC093278.2	0.527948734	2.25E-37	postive

TRBV18	AC093278.2	0.562727953	2.88E-43	postive
TRBV19	AC093278.2	0.548937638	7.56E-41	postive
TRBV20-1	AC093278.2	0.568434943	2.65E-44	postive
TRBV28	AC093278.2	0.57194117	6.00E-45	postive
TRBV29-1	AC093278.2	0.551265715	3.00E-41	postive
DDX17	AL355488.1	0.565156439	1.05E-43	postive
PLXNB1	AL355488.1	0.522356566	1.73E-36	postive
GNRH1	AL355488.1	0.608933892	2.91E-52	postive
NR2C1	AL355488.1	0.520475751	3.40E-36	postive
TNFRSF25	AL355488.1	0.500798269	3.16E-33	postive
PLCG1	AL355488.1	0.501544951	2.46E-33	postive
PSMD3	AC005288.1	0.628580016	1.48E-56	postive
FCER1G	PRKAR1B-AS1	0.511298154	8.70E-35	postive
HLA-DMB	PRKAR1B-AS1	0.567202472	4.46E-44	postive
CXCL10	PRKAR1B-AS1	0.504421718	9.28E-34	postive
CCL4	PRKAR1B-AS1	0.50930215	1.74E-34	postive
CCL3	PRKAR1B-AS1	0.538210455	4.83E-39	postive
CD86	PRKAR1B-AS1	0.521402937	2.43E-36	postive
NFKBIE	PRKAR1B-AS1	0.512204328	6.35E-35	postive
EBI3	PRKAR1B-AS1	0.572024595	5.79E-45	postive
GMFG	PRKAR1B-AS1	0.573400411	3.21E-45	postive
IL12RB1	PRKAR1B-AS1	0.533928276	2.44E-38	postive
TYROBP	PRKAR1B-AS1	0.520145899	3.82E-36	postive
LCK	PRKAR1B-AS1	0.592218726	7.69E-49	postive
LCP2	PRKAR1B-AS1	0.519429147	4.94E-36	postive
HCST	PRKAR1B-AS1	0.556304396	3.98E-42	postive
CD48	PRKAR1B-AS1	0.520577294	3.28E-36	postive
CD3D	PRKAR1B-AS1	0.543817436	5.60E-40	postive
ICOS	PRKAR1B-AS1	0.570205697	1.26E-44	postive
TRBC2	PRKAR1B-AS1	0.520485684	3.38E-36	postive
CD4	AC008972.2	0.609446444	2.27E-52	postive
CD74	AC008972.2	0.529115733	1.46E-37	postive
CTSS	AC008972.2	0.590891989	1.41E-48	postive
FCGRT	AC008972.2	0.54912878	7.01E-41	postive
HLA-DMA	AC008972.2	0.534632602	1.87E-38	postive
HLA-DMB	AC008972.2	0.526053641	4.51E-37	postive
HLA-DPA1	AC008972.2	0.50843982	2.34E-34	postive
HLA-DPB1	AC008972.2	0.543437297	6.49E-40	postive
LGMN	AC008972.2	0.572119548	5.56E-45	postive
CYBB	AC008972.2	0.580132111	1.73E-46	postive
TLR7	AC008972.2	0.553784494	1.10E-41	postive
TLR8	AC008972.2	0.518221298	7.60E-36	postive
CD14	AC008972.2	0.533874838	2.49E-38	postive
CCR5	AC008972.2	0.53169872	5.61E-38	postive
CCR1	AC008972.2	0.576676073	7.82E-46	postive
FGR	AC008972.2	0.578397369	3.70E-46	postive
CD86	AC008972.2	0.571542178	7.11E-45	postive
BTK	AC008972.2	0.536937031	7.84E-39	postive
PIK3R5	AC008972.2	0.531787347	5.43E-38	postive
LILRB3	AC008972.2	0.560673242	6.71E-43	postive
C5AR1	AC008972.2	0.54006821	2.38E-39	postive
CCRL2	AC008972.2	0.584158766	2.92E-47	postive
CMKLR1	AC008972.2	0.566032365	7.28E-44	postive
PLXNC1	AC008972.2	0.562143238	3.66E-43	postive
ADA2	AC008972.2	0.51086229	1.01E-34	postive
C3AR1	AC008972.2	0.581737964	8.54E-47	postive

CSF1R	AC008972.2	0.586587426	9.87E-48	postive
CSF2RA	AC008972.2	0.512305167	6.13E-35	postive
ENG	AC008972.2	0.548830726	7.88E-41	postive
IL10RA	AC008972.2	0.561737929	4.33E-43	postive
TNFRSF1B	AC008972.2	0.586334814	1.11E-47	postive
ITGB2	AC008972.2	0.593275801	4.73E-49	postive
TYROBP	AC008972.2	0.578621835	3.35E-46	postive
LCP2	AC008972.2	0.55641569	3.81E-42	postive
HCST	AC008972.2	0.532328474	4.44E-38	postive
PTPRC	AC008972.2	0.529760849	1.15E-37	postive
PCSK1	NEBL-AS1	0.526140266	4.36E-37	postive
CHGA	NEBL-AS1	0.659082211	7.27E-64	postive
GHRH	NEBL-AS1	0.660340659	3.48E-64	postive
SCG2	NEBL-AS1	0.655460035	5.94E-63	postive
TRH	NEBL-AS1	0.637557569	1.27E-58	postive
NR0B2	NEBL-AS1	0.519035934	5.69E-36	postive
THBS1	AP001434.1	0.537902135	5.43E-39	postive
PDGFRB	AP001434.1	0.584884295	2.11E-47	postive
EDNRA	AP001434.1	0.503759493	1.16E-33	postive
PLXNC1	AP001434.1	0.505748916	5.90E-34	postive
CCN2	AP001434.1	0.619121993	1.89E-54	postive
FGF7	AP001434.1	0.531632243	5.75E-38	postive
GREM1	AP001434.1	0.604974775	1.96E-51	postive
INHBA	AP001434.1	0.547421599	1.37E-40	postive
PDGFRL	AP001434.1	0.547339649	1.42E-40	postive
ANGPTL2	AP001434.1	0.642823706	7.27E-60	postive
IL1R1	AP001434.1	0.509833838	1.45E-34	postive
GNRH1	AL117379.1	0.600358275	1.76E-50	postive
OGFR	AL117379.1	0.538852411	3.78E-39	postive
LMBR1L	AC010542.6	0.523169064	1.29E-36	postive
GNRH1	AC010542.6	0.59168568	9.81E-49	postive
TNFRSF14	AC010542.6	0.520892816	2.92E-36	postive
TNFRSF25	AC010542.6	0.5688961	2.18E-44	postive
CTSL	AC023043.1	0.500002407	4.13E-33	postive
NFYB	TMPO-AS1	0.53175485	5.50E-38	postive
TNFRSF10B	AC107959.3	0.614708696	1.71E-53	postive
ZYX	AC093673.1	0.561000712	5.86E-43	postive
DDX17	AC015871.3	0.559581338	1.05E-42	postive
GNRH1	AC015871.3	0.547159639	1.52E-40	postive
PDGFRB	HECW2-AS1	0.504008298	1.07E-33	postive
PLXNC1	HECW2-AS1	0.549628114	5.75E-41	postive
GMFG	HECW2-AS1	0.529139525	1.45E-37	postive
CD48	HECW2-AS1	0.515528371	1.98E-35	postive
PSMC4	NKX2-1-AS1	0.516780082	1.27E-35	postive
ARRB1	NKX2-1-AS1	0.50633339	4.83E-34	postive
AKT2	NKX2-1-AS1	0.557873584	2.11E-42	postive
GRP	NKX2-1-AS1	0.587966309	5.31E-48	postive
ANGPTL1	NKX2-1-AS1	0.742068798	6.66E-89	postive
PROC	AL109615.3	0.547934886	1.12E-40	postive
CALCA	AL109615.3	0.530989203	7.31E-38	postive
THPO	AL109615.3	0.531715963	5.58E-38	postive
IREB2	OIP5-AS1	0.521587884	2.28E-36	postive
DDX17	AC002553.1	0.517254131	1.07E-35	postive
PLXNA3	AC002553.1	0.506003294	5.41E-34	postive
PLXNB1	AC002553.1	0.520496351	3.37E-36	postive
GNRH1	AC002553.1	0.610072514	1.67E-52	postive

GNRH1	LINC02604	0.585648034	1.50E-47	postive
OGFR	LINC02604	0.518263759	7.49E-36	postive
TNFRSF25	LINC02604	0.543218941	7.07E-40	postive
NR2C1	AC007938.3	0.566635541	5.66E-44	postive
PLCG1	AC007938.3	0.557003994	3.00E-42	postive
NFKBIB	AC022144.1	0.510987963	9.70E-35	postive
GNRH1	MED8-AS1	0.537851311	5.54E-39	postive
NFYB	AC127024.5	0.517933711	8.42E-36	postive
AZGP1	HOTAIR	0.760163504	1.09E-95	postive
S100A13	HOTAIR	0.515359093	2.10E-35	postive
S100A1	HOTAIR	0.593378752	4.51E-49	postive
DDX17	AC138956.2	0.565738531	8.23E-44	postive
NR2C2	AC138956.2	0.6081778	4.19E-52	postive
PIK3CG	AC007996.1	0.550333299	4.35E-41	postive
ANGPT1	AC007996.1	0.614527721	1.87E-53	postive
PLXNB1	ASMTL-AS1	0.518666174	6.49E-36	postive
GNRH1	ASMTL-AS1	0.611374168	8.84E-53	postive
TNFRSF25	ASMTL-AS1	0.511895668	7.07E-35	postive
DDX17	AC137932.1	0.504214642	9.95E-34	postive
NR2C2	AC137932.1	0.545199891	3.27E-40	postive
WNT5A	AC010731.2	0.537933738	5.37E-39	postive
GNRH1	AC009118.3	0.558561352	1.59E-42	postive
GNRH1	AL031775.2	0.517100574	1.13E-35	postive
DDX17	ERVK13-1	0.501328553	2.64E-33	postive
NR2C2	ERVK13-1	0.517757293	8.97E-36	postive
DDX17	AL135999.1	0.573751744	2.76E-45	postive
IRF9	AL135999.1	0.666063432	1.17E-65	postive
GNRH1	AL135999.1	0.506241605	4.99E-34	postive
NRTN	AC015802.3	0.526759759	3.48E-37	postive
CDC42	LINC00339	0.519090426	5.58E-36	postive
IRF9	AL136295.7	0.53984425	2.59E-39	postive
GNRH1	AL136295.7	0.64449382	2.90E-60	postive
TNFRSF25	AL136295.7	0.537496893	6.34E-39	postive
SFTPD	RBPMS-AS1	0.517786429	8.88E-36	postive
SFTPA1	RBPMS-AS1	0.553942929	1.03E-41	postive
SFTPA2	RBPMS-AS1	0.547707749	1.23E-40	postive
TFRC	LINC00885	0.528012343	2.20E-37	postive
CD1C	AC004687.1	0.682883781	3.43E-70	postive
HLA-DOB	AC004687.1	0.672576515	2.23E-67	postive
LTA	AC004687.1	0.762682144	1.11E-96	postive
CIITA	AC004687.1	0.553298251	1.33E-41	postive
CETP	AC004687.1	0.814427444	2.85E-120	postive
CD40LG	AC004687.1	0.782198168	8.18E-105	postive
BACH2	AC004687.1	0.510573259	1.12E-34	postive
PDCD1	AC004687.1	0.639277201	5.03E-59	postive
CCR7	AC004687.1	0.641827513	1.25E-59	postive
CCL21	AC004687.1	0.866342844	7.25E-153	postive
CXCR4	AC004687.1	0.502771743	1.62E-33	postive
CD79A	AC004687.1	0.717704323	1.28E-80	postive
CD79B	AC004687.1	0.910645435	4.64E-194	postive
BTK	AC004687.1	0.658692684	9.13E-64	postive
VAV1	AC004687.1	0.52167199	2.21E-36	postive
RAC2	AC004687.1	0.533800651	2.56E-38	postive
CD19	AC004687.1	0.916147466	1.19E-200	postive
CR2	AC004687.1	0.858422689	4.50E-147	postive
PIK3CD	AC004687.1	0.612139558	6.07E-53	postive

INPP5D	AC004687.1	0.728903459	2.59E-84	postive
CD22	AC004687.1	0.928696134	1.51E-217	postive
CD72	AC004687.1	0.852638858	4.62E-143	postive
PTPN6	AC004687.1	0.613231899	3.55E-53	postive
RASGRP3	AC004687.1	0.661998876	1.31E-64	postive
PLCG2	AC004687.1	0.657256407	2.10E-63	postive
PRKCB	AC004687.1	0.68628026	3.83E-71	postive
IGLJ1	AC004687.1	0.808922256	2.00E-117	postive
FLT3LG	AC004687.1	0.535866783	1.18E-38	postive
GMFG	AC004687.1	0.521814902	2.10E-36	postive
IL16	AC004687.1	0.7189728	5.00E-81	postive
LTB	AC004687.1	0.725035694	5.13E-83	postive
IL10RA	AC004687.1	0.511501271	8.11E-35	postive
IL12RB1	AC004687.1	0.603736233	3.54E-51	postive
IL21R	AC004687.1	0.500014957	4.11E-33	postive
TNFRSF13C	AC004687.1	0.899228674	1.19E-181	postive
ITGAL	AC004687.1	0.709876137	3.87E-78	postive
LCK	AC004687.1	0.669235149	1.72E-66	postive
NCR3	AC004687.1	0.775183715	8.51E-102	postive
CD247	AC004687.1	0.629891359	7.48E-57	postive
ZAP70	AC004687.1	0.824674654	7.82E-126	postive
CD3D	AC004687.1	0.517129897	1.12E-35	postive
CD3E	AC004687.1	0.620911705	7.63E-55	postive
PTPRC	AC004687.1	0.560553244	7.05E-43	postive
ITK	AC004687.1	0.558510075	1.63E-42	postive
GRAP2	AC004687.1	0.617195462	4.96E-54	postive
CD28	AC004687.1	0.561747124	4.31E-43	postive
ICOS	AC004687.1	0.543467917	6.42E-40	postive
CTLA4	AC004687.1	0.55177151	2.46E-41	postive
TRAC	AC004687.1	0.517229403	1.08E-35	postive
TRAV8-3	AC004687.1	0.591615864	1.01E-48	postive
TRBC1	AC004687.1	0.688646251	8.16E-72	postive
TRBC2	AC004687.1	0.669325634	1.63E-66	postive
TRBJ2-1	AC004687.1	0.613056042	3.87E-53	postive
TRBJ2-2	AC004687.1	0.722891074	2.63E-82	postive
TRBJ2-3	AC004687.1	0.734025998	4.57E-86	postive
TRBJ2-7	AC004687.1	0.776026586	3.74E-102	postive
TRBV2	AC004687.1	0.640622677	2.42E-59	postive
TRBV3-1	AC004687.1	0.562300212	3.43E-43	postive
TRBV5-1	AC004687.1	0.630037791	6.92E-57	postive
TRBV18	AC004687.1	0.58308803	4.70E-47	postive
TRBV28	AC004687.1	0.696447283	4.49E-74	postive
TRBV29-1	AC004687.1	0.541001785	1.66E-39	postive
GNRH1	AC233728.1	0.527466921	2.68E-37	postive
PRDX1	AC114489.2	0.501062736	2.89E-33	postive
OSGIN1	AC114489.2	0.577866119	4.66E-46	postive
IGF1R	IRAIN	0.541971818	1.14E-39	postive
PSMC4	AC093227.1	0.571174119	8.32E-45	postive
AKT2	AC093227.1	0.578670456	3.28E-46	postive
GRP	AC093227.1	0.512441989	5.84E-35	postive
ANGPTL1	AC093227.1	0.548157757	1.03E-40	postive
LMBR1L	ZNF32-AS2	0.519870128	4.22E-36	postive
DDX17	ZNF32-AS2	0.557882423	2.10E-42	postive
PLXNB1	ZNF32-AS2	0.527628321	2.53E-37	postive
GNRH1	ZNF32-AS2	0.541661151	1.29E-39	postive
NR2C2	ZNF32-AS2	0.563896917	1.77E-43	postive

PLCG1	ZNF32-AS2	0.593129106	5.06E-49	postive
PAK1	KCTD21-AS1	0.5081079	2.63E-34	postive
S100A3	AC083801.2	0.571518795	7.18E-45	postive
LGR6	AC083801.2	0.592594433	6.47E-49	postive
SEMA5B	LINC01786	0.519966749	4.08E-36	postive
GNRH1	NEAT1	0.576611868	8.04E-46	postive
GIPR	NEAT1	0.546041437	2.36E-40	postive
SLC22A17	AC004540.1	0.531122284	6.95E-38	postive
CHGA	AC004540.1	0.614084613	2.33E-53	postive
GHRH	AC004540.1	0.611028504	1.05E-52	postive
KL	AC004540.1	0.538884184	3.74E-39	postive
SCG2	AC004540.1	0.628493457	1.55E-56	postive
TRH	AC004540.1	0.594483789	2.71E-49	postive
NR0B2	AC004540.1	0.553368566	1.30E-41	postive
LMBR1L	AC132872.3	0.545277891	3.18E-40	postive
PLXNA3	AC132872.3	0.521155653	2.66E-36	postive
PLXNB1	AC132872.3	0.510149649	1.30E-34	postive
GNRH1	AC132872.3	0.602892956	5.29E-51	postive
SH3BP2	NOP14-AS1	0.527446613	2.70E-37	postive
B2M	PCED1B-AS1	0.522084945	1.90E-36	postive
CD1C	PCED1B-AS1	0.689550689	4.50E-72	postive
CD1D	PCED1B-AS1	0.613455093	3.18E-53	postive
CD4	PCED1B-AS1	0.621864577	4.71E-55	postive
CD8A	PCED1B-AS1	0.547792738	1.19E-40	postive
CD74	PCED1B-AS1	0.697800388	1.79E-74	postive
CTSS	PCED1B-AS1	0.587151648	7.66E-48	postive
HLA-DMA	PCED1B-AS1	0.730694369	6.37E-85	postive
HLA-DMB	PCED1B-AS1	0.740308323	2.84E-88	postive
HLA-DOA	PCED1B-AS1	0.649265712	2.02E-61	postive
HLA-DOB	PCED1B-AS1	0.8747438	1.97E-159	postive
HLA-DPA1	PCED1B-AS1	0.612221639	5.83E-53	postive
HLA-DPB1	PCED1B-AS1	0.702594809	6.60E-76	postive
HLA-DQA1	PCED1B-AS1	0.53309438	3.33E-38	postive
HLA-DQA2	PCED1B-AS1	0.582711247	5.56E-47	postive
HLA-DQB1	PCED1B-AS1	0.504152296	1.02E-33	postive
HLA-DRA	PCED1B-AS1	0.586053264	1.25E-47	postive
HLA-DRB1	PCED1B-AS1	0.544065905	5.09E-40	postive
HLA-E	PCED1B-AS1	0.577532602	5.39E-46	postive
HLA-F	PCED1B-AS1	0.504433207	9.24E-34	postive
LTA	PCED1B-AS1	0.785500452	2.84E-106	postive
CIITA	PCED1B-AS1	0.639615579	4.19E-59	postive
IFI30	PCED1B-AS1	0.608510815	3.57E-52	postive
RBP5	PCED1B-AS1	0.600581205	1.58E-50	postive
CETP	PCED1B-AS1	0.679904887	2.29E-69	postive
CYBB	PCED1B-AS1	0.541588519	1.33E-39	postive
ISG20	PCED1B-AS1	0.637653992	1.21E-58	postive
TLR7	PCED1B-AS1	0.535993171	1.12E-38	postive
CYLD	PCED1B-AS1	0.570439093	1.14E-44	postive
CCL4	PCED1B-AS1	0.512509418	5.70E-35	postive
IL7R	PCED1B-AS1	0.529613592	1.22E-37	postive
CD40LG	PCED1B-AS1	0.876015122	1.82E-160	postive
TRIM22	PCED1B-AS1	0.59287524	5.69E-49	postive
PDCD1	PCED1B-AS1	0.798413054	3.07E-112	postive
ANXA6	PCED1B-AS1	0.607443662	5.98E-52	postive
CCR7	PCED1B-AS1	0.588081942	5.04E-48	postive
CCR8	PCED1B-AS1	0.525929941	4.71E-37	postive

CCL21	PCED1B-AS1	0.706917231	3.18E-77	postive
CCR5	PCED1B-AS1	0.601980741	8.16E-51	postive
CXCR4	PCED1B-AS1	0.744018153	1.32E-89	postive
CXCR6	PCED1B-AS1	0.581258782	1.06E-46	postive
CCR4	PCED1B-AS1	0.733855141	5.23E-86	postive
FGR	PCED1B-AS1	0.547198711	1.50E-40	postive
CD86	PCED1B-AS1	0.545397423	3.03E-40	postive
CD79A	PCED1B-AS1	0.887004034	6.50E-170	postive
CD79B	PCED1B-AS1	0.789428879	4.82E-108	postive
BTK	PCED1B-AS1	0.897576774	5.60E-180	postive
VAV1	PCED1B-AS1	0.712060651	8.02E-79	postive
RAC2	PCED1B-AS1	0.638862368	6.30E-59	postive
PPP3CC	PCED1B-AS1	0.524907826	6.84E-37	postive
NFATC2	PCED1B-AS1	0.511909038	7.03E-35	postive
CD19	PCED1B-AS1	0.776360197	2.70E-102	postive
CR2	PCED1B-AS1	0.673216174	1.50E-67	postive
PIK3R5	PCED1B-AS1	0.614254529	2.14E-53	postive
PIK3CD	PCED1B-AS1	0.593602668	4.07E-49	postive
INPP5D	PCED1B-AS1	0.809319019	1.26E-117	postive
CD22	PCED1B-AS1	0.673326451	1.40E-67	postive
CD72	PCED1B-AS1	0.802554622	3.03E-114	postive
PTPN6	PCED1B-AS1	0.62061599	8.87E-55	postive
FCGR2B	PCED1B-AS1	0.647493295	5.46E-61	postive
RASGRP3	PCED1B-AS1	0.7887184	1.01E-107	postive
PLCG2	PCED1B-AS1	0.744412062	9.49E-90	postive
PRKCB	PCED1B-AS1	0.82535217	3.26E-126	postive
IGHA1	PCED1B-AS1	0.591720932	9.65E-49	postive
IGHA2	PCED1B-AS1	0.534839015	1.73E-38	postive
IGHD1-1	PCED1B-AS1	0.540407316	2.09E-39	postive
IGHD6-25	PCED1B-AS1	0.602288317	7.05E-51	postive
IGHG1	PCED1B-AS1	0.606075819	1.16E-51	postive
IGHG2	PCED1B-AS1	0.600098334	1.98E-50	postive
IGHG3	PCED1B-AS1	0.579368991	2.42E-46	postive
IGHG4	PCED1B-AS1	0.525738549	5.05E-37	postive
IGHJ2	PCED1B-AS1	0.522801825	1.47E-36	postive
IGHJ3	PCED1B-AS1	0.604690537	2.25E-51	postive
IGHV1-46	PCED1B-AS1	0.536283495	1.00E-38	postive
IGHV1-69	PCED1B-AS1	0.501809234	2.25E-33	postive
IGHV2-5	PCED1B-AS1	0.504783288	8.20E-34	postive
IGHV3-15	PCED1B-AS1	0.527296236	2.86E-37	postive
IGHV3-23	PCED1B-AS1	0.556004291	4.49E-42	postive
IGHV3-30	PCED1B-AS1	0.540850828	1.76E-39	postive
IGHV3-33	PCED1B-AS1	0.53416025	2.23E-38	postive
IGHV3-7	PCED1B-AS1	0.517577014	9.56E-36	postive
IGHV4-28	PCED1B-AS1	0.5913279	1.15E-48	postive
IGHV4-34	PCED1B-AS1	0.555691314	5.10E-42	postive
IGHV4-39	PCED1B-AS1	0.501078811	2.88E-33	postive
IGHV4-59	PCED1B-AS1	0.547987071	1.10E-40	postive
IGHV4-61	PCED1B-AS1	0.525193457	6.17E-37	postive
IGHV5-51	PCED1B-AS1	0.580296846	1.61E-46	postive
IGKC	PCED1B-AS1	0.667189837	5.92E-66	postive
IGKJ5	PCED1B-AS1	0.700243038	3.36E-75	postive
IGKV1-12	PCED1B-AS1	0.513288956	4.34E-35	postive
IGKV1-39	PCED1B-AS1	0.560627997	6.83E-43	postive
IGKV1-5	PCED1B-AS1	0.583511829	3.90E-47	postive
IGKV1-8	PCED1B-AS1	0.564647387	1.30E-43	postive

IGKV1D-39	PCED1B-AS1	0.520989661	2.82E-36	postive
IGKV1D-8	PCED1B-AS1	0.565689637	8.40E-44	postive
IGKV3-11	PCED1B-AS1	0.630195265	6.38E-57	postive
IGKV3-15	PCED1B-AS1	0.607254988	6.55E-52	postive
IGKV3-20	PCED1B-AS1	0.629341291	9.97E-57	postive
IGKV3-7	PCED1B-AS1	0.527838253	2.34E-37	postive
IGKV3D-11	PCED1B-AS1	0.558281647	1.78E-42	postive
IGKV3D-15	PCED1B-AS1	0.537575254	6.15E-39	postive
IGKV3D-7	PCED1B-AS1	0.526884878	3.32E-37	postive
IGKV4-1	PCED1B-AS1	0.540596587	1.94E-39	postive
IGLC2	PCED1B-AS1	0.658245684	1.18E-63	postive
IGLC3	PCED1B-AS1	0.575005935	1.61E-45	postive
IGLC6	PCED1B-AS1	0.526684264	3.58E-37	postive
IGLJ1	PCED1B-AS1	0.813454685	9.21E-120	postive
IGLJ2	PCED1B-AS1	0.629739846	8.09E-57	postive
IGLJ3	PCED1B-AS1	0.661268994	2.02E-64	postive
IGLV1-40	PCED1B-AS1	0.597721453	6.04E-50	postive
IGLV1-44	PCED1B-AS1	0.568956118	2.13E-44	postive
IGLV1-51	PCED1B-AS1	0.546219357	2.20E-40	postive
IGLV2-14	PCED1B-AS1	0.541991765	1.14E-39	postive
IGLV2-23	PCED1B-AS1	0.509944906	1.39E-34	postive
IGLV3-1	PCED1B-AS1	0.530029223	1.04E-37	postive
IGLV3-21	PCED1B-AS1	0.507017516	3.82E-34	postive
IGLV3-25	PCED1B-AS1	0.53275532	3.78E-38	postive
IGLV4-69	PCED1B-AS1	0.504619047	8.67E-34	postive
CCRL2	PCED1B-AS1	0.501707385	2.33E-33	postive
CMKLR1	PCED1B-AS1	0.525733074	5.07E-37	postive
CXCR3	PCED1B-AS1	0.662227391	1.15E-64	postive
CYSLTR1	PCED1B-AS1	0.506385363	4.75E-34	postive
ACKR1	PCED1B-AS1	0.534338753	2.09E-38	postive
PLXNC1	PCED1B-AS1	0.561976829	3.92E-43	postive
ADA2	PCED1B-AS1	0.622174935	4.02E-55	postive
FLT3LG	PCED1B-AS1	0.611083199	1.02E-52	postive
GMFG	PCED1B-AS1	0.836759312	7.07E-133	postive
IL16	PCED1B-AS1	0.908828884	5.62E-192	postive
LTB	PCED1B-AS1	0.644783962	2.47E-60	postive
TNFSF12	PCED1B-AS1	0.564437238	1.42E-43	postive
TNFSF13B	PCED1B-AS1	0.504869166	7.97E-34	postive
CSF2RA	PCED1B-AS1	0.501504104	2.49E-33	postive
IL10RA	PCED1B-AS1	0.846159973	9.10E-139	postive
IL12RB1	PCED1B-AS1	0.826008069	1.39E-126	postive
IL2RB	PCED1B-AS1	0.556281403	4.02E-42	postive
IL18RAP	PCED1B-AS1	0.584837774	2.16E-47	postive
IL21R	PCED1B-AS1	0.650122318	1.25E-61	postive
IL2RG	PCED1B-AS1	0.654864346	8.38E-63	postive
IL3RA	PCED1B-AS1	0.62197123	4.46E-55	postive
NR1H3	PCED1B-AS1	0.535778018	1.22E-38	postive
S1PR1	PCED1B-AS1	0.53329895	3.09E-38	postive
S1PR2	PCED1B-AS1	0.513766531	3.67E-35	postive
TNFRSF13C	PCED1B-AS1	0.673108049	1.60E-67	postive
TNFRSF14	PCED1B-AS1	0.577399701	5.71E-46	postive
TNFRSF17	PCED1B-AS1	0.729577599	1.53E-84	postive
TNFRSF1B	PCED1B-AS1	0.671813029	3.56E-67	postive
ICAM2	PCED1B-AS1	0.6537432	1.59E-62	postive
ITGAL	PCED1B-AS1	0.87183523	4.17E-157	postive
ITGB2	PCED1B-AS1	0.564235914	1.54E-43	postive

TYROBP	PCED1B-AS1	0.512493207	5.74E-35	postive
LCK	PCED1B-AS1	0.778251158	4.21E-103	postive
NCR3	PCED1B-AS1	0.867847133	5.22E-154	postive
CD247	PCED1B-AS1	0.84875545	1.83E-140	postive
ZAP70	PCED1B-AS1	0.80611227	5.24E-116	postive
LCP2	PCED1B-AS1	0.654568186	9.93E-63	postive
HCST	PCED1B-AS1	0.703732203	2.99E-76	postive
CD48	PCED1B-AS1	0.69829239	1.28E-74	postive
SH2D1A	PCED1B-AS1	0.708330816	1.17E-77	postive
CD3D	PCED1B-AS1	0.80535166	1.26E-115	postive
CD3E	PCED1B-AS1	0.876365597	9.41E-161	postive
CD3G	PCED1B-AS1	0.631448759	3.30E-57	postive
PTPRC	PCED1B-AS1	0.847329724	1.58E-139	postive
ITK	PCED1B-AS1	0.84428079	1.47E-137	postive
GRAP2	PCED1B-AS1	0.786441461	1.08E-106	postive
CD28	PCED1B-AS1	0.845148608	4.09E-138	postive
ICOS	PCED1B-AS1	0.767089211	1.90E-98	postive
CTLA4	PCED1B-AS1	0.747624294	6.34E-91	postive
TRAC	PCED1B-AS1	0.798735188	2.15E-112	postive
TRAV2	PCED1B-AS1	0.693126603	4.19E-73	postive
TRAV4	PCED1B-AS1	0.663163342	6.58E-65	postive
TRAV8-3	PCED1B-AS1	0.803961831	6.15E-115	postive
TRAV8-6	PCED1B-AS1	0.734503735	3.12E-86	postive
TRAV9-2	PCED1B-AS1	0.757350376	1.35E-94	postive
TRAV12-1	PCED1B-AS1	0.639067699	5.64E-59	postive
TRAV12-2	PCED1B-AS1	0.612437124	5.25E-53	postive
TRAV12-3	PCED1B-AS1	0.628920249	1.24E-56	postive
TRAV13-1	PCED1B-AS1	0.664442388	3.07E-65	postive
TRAV21	PCED1B-AS1	0.616554841	6.83E-54	postive
TRBC1	PCED1B-AS1	0.871690678	5.43E-157	postive
TRBC2	PCED1B-AS1	0.898499661	6.58E-181	postive
TRBJ2-1	PCED1B-AS1	0.785298866	3.49E-106	postive
TRBJ2-2	PCED1B-AS1	0.754217628	2.15E-93	postive
TRBJ2-3	PCED1B-AS1	0.779908569	8.13E-104	postive
TRBJ2-7	PCED1B-AS1	0.825970535	1.46E-126	postive
TRBV2	PCED1B-AS1	0.787639155	3.12E-107	postive
TRBV3-1	PCED1B-AS1	0.765588133	7.67E-98	postive
TRBV4-1	PCED1B-AS1	0.52015038	3.82E-36	postive
TRBV5-1	PCED1B-AS1	0.825820771	1.77E-126	postive
TRBV5-4	PCED1B-AS1	0.727728673	6.44E-84	postive
TRBV5-6	PCED1B-AS1	0.714147841	1.76E-79	postive
TRBV6-1	PCED1B-AS1	0.71861132	6.55E-81	postive
TRBV6-5	PCED1B-AS1	0.746384845	1.81E-90	postive
TRBV7-9	PCED1B-AS1	0.658986354	7.69E-64	postive
TRBV9	PCED1B-AS1	0.559279085	1.19E-42	postive
TRBV10-3	PCED1B-AS1	0.66167161	1.59E-64	postive
TRBV12-4	PCED1B-AS1	0.502935221	1.54E-33	postive
TRBV18	PCED1B-AS1	0.824232825	1.38E-125	postive
TRBV19	PCED1B-AS1	0.746963288	1.11E-90	postive
TRBV20-1	PCED1B-AS1	0.770204837	1.01E-99	postive
TRBV28	PCED1B-AS1	0.896938133	2.44E-179	postive
TRBV29-1	PCED1B-AS1	0.800954969	1.83E-113	postive
LMBR1L	AL360181.2	0.511452265	8.25E-35	postive
IRF3	SNHG12	0.50202853	2.09E-33	postive
GNRH1	SNHG12	0.598139847	4.97E-50	postive
TNFRSF25	SNHG12	0.592844655	5.77E-49	postive

B2M	AL590764.1	0.588824638	3.60E-48	postive
CD1C	AL590764.1	0.629288656	1.02E-56	postive
CD1D	AL590764.1	0.616669493	6.45E-54	postive
CD4	AL590764.1	0.616378397	7.46E-54	postive
CD8A	AL590764.1	0.582900074	5.11E-47	postive
CD74	AL590764.1	0.683730022	1.99E-70	postive
CTSS	AL590764.1	0.668574282	2.56E-66	postive
FCER1G	AL590764.1	0.655102386	7.30E-63	postive
HLA-DMA	AL590764.1	0.742524563	4.57E-89	postive
HLA-DMB	AL590764.1	0.819340424	6.79E-123	postive
HLA-DOA	AL590764.1	0.603278921	4.40E-51	postive
HLA-DOB	AL590764.1	0.731158927	4.42E-85	postive
HLA-DPA1	AL590764.1	0.635901251	3.10E-58	postive
HLA-DPB1	AL590764.1	0.710003918	3.53E-78	postive
HLA-DQA1	AL590764.1	0.541167577	1.56E-39	postive
HLA-DQA2	AL590764.1	0.575410566	1.35E-45	postive
HLA-DQB1	AL590764.1	0.526230316	4.22E-37	postive
HLA-DRA	AL590764.1	0.676665465	1.76E-68	postive
HLA-DRB1	AL590764.1	0.606525053	9.31E-52	postive
HLA-E	AL590764.1	0.545134892	3.36E-40	postive
LTA	AL590764.1	0.686296263	3.79E-71	postive
CIITA	AL590764.1	0.540638313	1.91E-39	postive
IFI30	AL590764.1	0.602190638	7.38E-51	postive
RBP5	AL590764.1	0.531323575	6.45E-38	postive
CETP	AL590764.1	0.571389854	7.59E-45	postive
CYBB	AL590764.1	0.584594818	2.41E-47	postive
ISG20	AL590764.1	0.603293349	4.37E-51	postive
TLR7	AL590764.1	0.579664402	2.13E-46	postive
TLR8	AL590764.1	0.591278431	1.18E-48	postive
CCL4	AL590764.1	0.614713306	1.71E-53	postive
CD40LG	AL590764.1	0.777017641	1.42E-102	postive
TRIM22	AL590764.1	0.618800202	2.22E-54	postive
PDCD1	AL590764.1	0.702456316	7.27E-76	postive
ANXA6	AL590764.1	0.554130135	9.56E-42	postive
CCL21	AL590764.1	0.547143977	1.53E-40	postive
CCL3	AL590764.1	0.530777892	7.90E-38	postive
CCR5	AL590764.1	0.621912102	4.59E-55	postive
CCR1	AL590764.1	0.532075614	4.88E-38	postive
CXCR4	AL590764.1	0.683438914	2.40E-70	postive
CXCR6	AL590764.1	0.661496804	1.76E-64	postive
CCR4	AL590764.1	0.675596998	3.43E-68	postive
FGR	AL590764.1	0.585845178	1.38E-47	postive
CD86	AL590764.1	0.622216035	3.94E-55	postive
CD79A	AL590764.1	0.695006473	1.19E-73	postive
CD79B	AL590764.1	0.640427217	2.69E-59	postive
BTK	AL590764.1	0.862467242	5.49E-150	postive
VAV1	AL590764.1	0.658652375	9.34E-64	postive
RAC2	AL590764.1	0.588657292	3.88E-48	postive
CD19	AL590764.1	0.613478552	3.14E-53	postive
CR2	AL590764.1	0.535103056	1.57E-38	postive
PIK3R5	AL590764.1	0.549747119	5.49E-41	postive
INPP5D	AL590764.1	0.696279719	5.03E-74	postive
CD22	AL590764.1	0.521364203	2.47E-36	postive
CD72	AL590764.1	0.736673653	5.46E-87	postive
PTPN6	AL590764.1	0.571931439	6.02E-45	postive
LILRB3	AL590764.1	0.516732678	1.29E-35	postive

FCGR2B	AL590764.1	0.6783247	6.21E-69	postive
RASGRP3	AL590764.1	0.704297443	2.01E-76	postive
PLCG2	AL590764.1	0.600175852	1.91E-50	postive
PRKCB	AL590764.1	0.725549931	3.46E-83	postive
IGHA1	AL590764.1	0.500586477	3.39E-33	postive
IGHD6-25	AL590764.1	0.509995079	1.37E-34	postive
IGHV3-30	AL590764.1	0.507368982	3.39E-34	postive
IGHV3-7	AL590764.1	0.50979779	1.47E-34	postive
IGKC	AL590764.1	0.56684004	5.19E-44	postive
IGKJ5	AL590764.1	0.566506664	5.97E-44	postive
IGKV1-5	AL590764.1	0.513548425	3.97E-35	postive
IGKV1-8	AL590764.1	0.514891777	2.47E-35	postive
IGKV3-11	AL590764.1	0.5566402	3.47E-42	postive
IGKV3-20	AL590764.1	0.537028017	7.57E-39	postive
IGLC2	AL590764.1	0.555167067	6.30E-42	postive
IGLJ1	AL590764.1	0.61104546	1.04E-52	postive
CCRL2	AL590764.1	0.617011825	5.43E-54	postive
CMKLR1	AL590764.1	0.568803658	2.27E-44	postive
CXCR3	AL590764.1	0.632442989	1.95E-57	postive
CYSLTR1	AL590764.1	0.508043264	2.69E-34	postive
ACKR1	AL590764.1	0.502041168	2.08E-33	postive
PLXNC1	AL590764.1	0.588621734	3.95E-48	postive
ADA2	AL590764.1	0.574007024	2.48E-45	postive
FLT3LG	AL590764.1	0.536829775	8.16E-39	postive
GMFG	AL590764.1	0.916274372	8.30E-201	postive
IL16	AL590764.1	0.783330447	2.60E-105	postive
LTB	AL590764.1	0.52614328	4.36E-37	postive
TNFSF12	AL590764.1	0.575197198	1.48E-45	postive
TNFSF13B	AL590764.1	0.611166161	9.79E-53	postive
C3AR1	AL590764.1	0.500022351	4.10E-33	postive
CSF1R	AL590764.1	0.507684925	3.04E-34	postive
CSF2RA	AL590764.1	0.510903595	9.98E-35	postive
IL10RA	AL590764.1	0.766353099	3.77E-98	postive
IL12RB1	AL590764.1	0.769073572	2.96E-99	postive
IL2RB	AL590764.1	0.512291414	6.16E-35	postive
IL18RAP	AL590764.1	0.629638798	8.53E-57	postive
IL21R	AL590764.1	0.565890757	7.72E-44	postive
IL2RG	AL590764.1	0.60636276	1.01E-51	postive
IL3RA	AL590764.1	0.583422724	4.05E-47	postive
NR1H3	AL590764.1	0.66864597	2.46E-66	postive
S1PR1	AL590764.1	0.510033319	1.35E-34	postive
TNFRSF13C	AL590764.1	0.503834104	1.13E-33	postive
TNFRSF14	AL590764.1	0.518653082	6.52E-36	postive
TNFRSF17	AL590764.1	0.676903115	1.52E-68	postive
TNFRSF1B	AL590764.1	0.641760724	1.30E-59	postive
ICAM2	AL590764.1	0.623737802	1.81E-55	postive
ITGAL	AL590764.1	0.768633678	4.47E-99	postive
ITGB2	AL590764.1	0.578365215	3.75E-46	postive
TYROBP	AL590764.1	0.664857624	2.40E-65	postive
LCK	AL590764.1	0.727590655	7.17E-84	postive
NCR3	AL590764.1	0.797643912	7.16E-112	postive
CD247	AL590764.1	0.787265585	4.60E-107	postive
ZAP70	AL590764.1	0.685984505	4.64E-71	postive
LCP2	AL590764.1	0.675123704	4.60E-68	postive
HCST	AL590764.1	0.794011356	3.71E-110	postive
CD48	AL590764.1	0.841058409	1.60E-135	postive

CD244	AL590764.1	0.562669241	2.95E-43	postive
SH2D1A	AL590764.1	0.736843376	4.76E-87	postive
GZMB	AL590764.1	0.520633657	3.21E-36	postive
CD3D	AL590764.1	0.845921219	1.30E-138	postive
CD3E	AL590764.1	0.799748648	7.01E-113	postive
CD3G	AL590764.1	0.634213408	7.64E-58	postive
PTPRC	AL590764.1	0.811630923	8.17E-119	postive
ITK	AL590764.1	0.751058426	3.35E-92	postive
GRAP2	AL590764.1	0.71831458	8.16E-81	postive
CD28	AL590764.1	0.751253824	2.83E-92	postive
ICOS	AL590764.1	0.763607247	4.76E-97	postive
CTLA4	AL590764.1	0.712964957	4.16E-79	postive
TRAC	AL590764.1	0.740275501	2.92E-88	postive
TRAV2	AL590764.1	0.697659174	1.97E-74	postive
TRAV4	AL590764.1	0.681998716	6.04E-70	postive
TRAV8-3	AL590764.1	0.733423033	7.38E-86	postive
TRAV8-6	AL590764.1	0.699098775	7.37E-75	postive
TRAV9-2	AL590764.1	0.727475294	7.84E-84	postive
TRAV12-1	AL590764.1	0.599626161	2.48E-50	postive
TRAV12-2	AL590764.1	0.639894044	3.60E-59	postive
TRAV12-3	AL590764.1	0.592759637	6.00E-49	postive
TRAV13-1	AL590764.1	0.662477328	9.88E-65	postive
TRAV21	AL590764.1	0.582460212	6.21E-47	postive
TRBC1	AL590764.1	0.784632213	6.91E-106	postive
TRBC2	AL590764.1	0.842408982	2.27E-136	postive
TRBJ2-1	AL590764.1	0.668236889	3.15E-66	postive
TRBJ2-2	AL590764.1	0.655605442	5.47E-63	postive
TRBJ2-3	AL590764.1	0.717789971	1.21E-80	postive
TRBJ2-7	AL590764.1	0.677174118	1.28E-68	postive
TRBV2	AL590764.1	0.705309519	9.89E-77	postive
TRBV3-1	AL590764.1	0.700236013	3.37E-75	postive
TRBV4-1	AL590764.1	0.525624943	5.27E-37	postive
TRBV5-1	AL590764.1	0.771205598	3.92E-100	postive
TRBV5-4	AL590764.1	0.657694562	1.63E-63	postive
TRBV5-6	AL590764.1	0.655963376	4.45E-63	postive
TRBV6-1	AL590764.1	0.662958911	7.43E-65	postive
TRBV6-5	AL590764.1	0.670062504	1.04E-66	postive
TRBV7-9	AL590764.1	0.670063806	1.04E-66	postive
TRBV9	AL590764.1	0.638803478	6.50E-59	postive
TRBV10-3	AL590764.1	0.607803062	5.03E-52	postive
TRBV12-4	AL590764.1	0.586795126	8.99E-48	postive
TRBV18	AL590764.1	0.76549248	8.38E-98	postive
TRBV19	AL590764.1	0.675988231	2.69E-68	postive
TRBV20-1	AL590764.1	0.729815709	1.27E-84	postive
TRBV28	AL590764.1	0.806926962	2.04E-116	postive
TRBV29-1	AL590764.1	0.731835815	2.59E-85	postive
NOS2	C5orf66-AS1	0.502916493	1.55E-33	postive
CD1C	AC004585.1	0.561808021	4.21E-43	postive
CD1D	AC004585.1	0.567083896	4.69E-44	postive
CD74	AC004585.1	0.522370425	1.72E-36	postive
HLA-DMA	AC004585.1	0.567612608	3.75E-44	postive
HLA-DMB	AC004585.1	0.614157306	2.25E-53	postive
HLA-DOB	AC004585.1	0.667290944	5.57E-66	postive
HLA-DPB1	AC004585.1	0.527894473	2.29E-37	postive
HLA-F	AC004585.1	0.504276236	9.75E-34	postive
LTA	AC004585.1	0.721481331	7.63E-82	postive

CIITA	AC004585.1	0.541752944	1.24E-39	postive
IFI30	AC004585.1	0.587349337	7.01E-48	postive
RBP5	AC004585.1	0.523832595	1.01E-36	postive
CETP	AC004585.1	0.613677583	2.85E-53	postive
CD40LG	AC004585.1	0.736525143	6.16E-87	postive
TRIM22	AC004585.1	0.506346165	4.81E-34	postive
PDCD1	AC004585.1	0.699917425	4.20E-75	postive
CCR7	AC004585.1	0.570445522	1.13E-44	postive
CCL21	AC004585.1	0.60536441	1.63E-51	postive
CXCR4	AC004585.1	0.60167183	9.44E-51	postive
CCR4	AC004585.1	0.596143277	1.26E-49	postive
CD79A	AC004585.1	0.648178447	3.72E-61	postive
CD79B	AC004585.1	0.678689381	4.94E-69	postive
BTK	AC004585.1	0.719646625	3.02E-81	postive
VAV1	AC004585.1	0.59347668	4.32E-49	postive
RAC2	AC004585.1	0.60016281	1.92E-50	postive
CD19	AC004585.1	0.671848592	3.48E-67	postive
CR2	AC004585.1	0.612432789	5.26E-53	postive
PIK3CD	AC004585.1	0.538306793	4.66E-39	postive
INPP5D	AC004585.1	0.677988021	7.68E-69	postive
CD22	AC004585.1	0.609765216	1.94E-52	postive
CD72	AC004585.1	0.74794027	4.84E-91	postive
PTPN6	AC004585.1	0.547087921	1.56E-40	postive
RASGRP3	AC004585.1	0.628658104	1.42E-56	postive
PLCG2	AC004585.1	0.548330569	9.60E-41	postive
PRKCB	AC004585.1	0.69267269	5.68E-73	postive
IGLJ1	AC004585.1	0.587159734	7.63E-48	postive
CXCR3	AC004585.1	0.54525404	3.20E-40	postive
FLT3LG	AC004585.1	0.555003765	6.73E-42	postive
GMFG	AC004585.1	0.723575684	1.56E-82	postive
IL16	AC004585.1	0.712138469	7.58E-79	postive
LTB	AC004585.1	0.591024821	1.33E-48	postive
IL10RA	AC004585.1	0.634982438	5.07E-58	postive
IL12RB1	AC004585.1	0.716529591	3.06E-80	postive
IL18RAP	AC004585.1	0.517248429	1.07E-35	postive
IL21R	AC004585.1	0.551811447	2.42E-41	postive
IL2RG	AC004585.1	0.518134048	7.84E-36	postive
IL3RA	AC004585.1	0.503972988	1.08E-33	postive
NR1H3	AC004585.1	0.553780624	1.10E-41	postive
TNFRSF13C	AC004585.1	0.580810798	1.29E-46	postive
TNFRSF1B	AC004585.1	0.541520305	1.36E-39	postive
ITGAL	AC004585.1	0.725049937	5.07E-83	postive
LCK	AC004585.1	0.716150995	4.05E-80	postive
NCR3	AC004585.1	0.77592169	4.15E-102	postive
CD247	AC004585.1	0.719401233	3.63E-81	postive
ZAP70	AC004585.1	0.71536647	7.20E-80	postive
LCP2	AC004585.1	0.528890502	1.59E-37	postive
HCST	AC004585.1	0.629854288	7.62E-57	postive
CD48	AC004585.1	0.675602928	3.42E-68	postive
SH2D1A	AC004585.1	0.656378547	3.50E-63	postive
CD3D	AC004585.1	0.747377817	7.81E-91	postive
CD3E	AC004585.1	0.740789058	1.91E-88	postive
CD3G	AC004585.1	0.537066558	7.46E-39	postive
PTPRC	AC004585.1	0.725860444	2.72E-83	postive
ITK	AC004585.1	0.707757021	1.75E-77	postive
GRAP2	AC004585.1	0.67223775	2.74E-67	postive

CD28	AC004585.1	0.710702414	2.14E-78	postive
ICOS	AC004585.1	0.650611089	9.47E-62	postive
CTLA4	AC004585.1	0.681813697	6.80E-70	postive
TRAC	AC004585.1	0.669346146	1.61E-66	postive
TRAV2	AC004585.1	0.613179744	3.64E-53	postive
TRAV4	AC004585.1	0.584160223	2.92E-47	postive
TRAV8-3	AC004585.1	0.69666966	3.86E-74	postive
TRAV8-6	AC004585.1	0.638842134	6.37E-59	postive
TRAV9-2	AC004585.1	0.681878254	6.52E-70	postive
TRAV12-1	AC004585.1	0.530230212	9.68E-38	postive
TRAV12-2	AC004585.1	0.523072162	1.33E-36	postive
TRAV12-3	AC004585.1	0.538925823	3.68E-39	postive
TRAV13-1	AC004585.1	0.524613417	7.62E-37	postive
TRBC1	AC004585.1	0.736306879	7.34E-87	postive
TRBC2	AC004585.1	0.792708732	1.50E-109	postive
TRBJ2-1	AC004585.1	0.664318571	3.31E-65	postive
TRBJ2-2	AC004585.1	0.675010696	4.94E-68	postive
TRBJ2-3	AC004585.1	0.747309568	8.27E-91	postive
TRBJ2-7	AC004585.1	0.699666947	4.99E-75	postive
TRBV2	AC004585.1	0.671808917	3.57E-67	postive
TRBV3-1	AC004585.1	0.660532668	3.11E-64	postive
TRBV5-1	AC004585.1	0.718355322	7.92E-81	postive
TRBV5-4	AC004585.1	0.581713288	8.64E-47	postive
TRBV5-6	AC004585.1	0.59813046	4.99E-50	postive
TRBV6-1	AC004585.1	0.58224393	6.83E-47	postive
TRBV6-5	AC004585.1	0.624695748	1.11E-55	postive
TRBV7-9	AC004585.1	0.589169483	3.08E-48	postive
TRBV9	AC004585.1	0.510276048	1.24E-34	postive
TRBV10-3	AC004585.1	0.571973859	5.92E-45	postive
TRBV18	AC004585.1	0.733972414	4.76E-86	postive
TRBV19	AC004585.1	0.617393388	4.49E-54	postive
TRBV20-1	AC004585.1	0.649474609	1.80E-61	postive
TRBV28	AC004585.1	0.774232521	2.14E-101	postive
TRBV29-1	AC004585.1	0.685447858	6.57E-71	postive
LMBR1L	AC090589.3	0.563604732	2.00E-43	postive
DDX17	AC090589.3	0.592768288	5.97E-49	postive
PLXNB1	AC090589.3	0.591749221	9.53E-49	postive
GNRH1	AC090589.3	0.608053141	4.45E-52	postive
NR2C2	AC090589.3	0.535996635	1.12E-38	postive
TNFRSF25	AC090589.3	0.539037516	3.53E-39	postive
CTSS	AC026369.3	0.516685572	1.31E-35	postive
HLA-DMA	AC026369.3	0.560522751	7.14E-43	postive
HLA-DMB	AC026369.3	0.500804934	3.15E-33	postive
HLA-DPB1	AC026369.3	0.562194819	3.59E-43	postive
HLA-DQA2	AC026369.3	0.513698765	3.76E-35	postive
SLC11A1	AC026369.3	0.519789048	4.34E-36	postive
MARCO	AC026369.3	0.663269861	6.17E-65	postive
AGER	AC026369.3	0.703292591	4.06E-76	postive
CCL18	AC026369.3	0.52541164	5.70E-37	postive
CCL23	AC026369.3	0.586859681	8.73E-48	postive
FGR	AC026369.3	0.580287619	1.62E-46	postive
CCRL2	AC026369.3	0.523397838	1.18E-36	postive
VEGFD	AC026369.3	0.613893261	2.56E-53	postive
GMFG	AC026369.3	0.57153234	7.14E-45	postive
ACVRL1	AC026369.3	0.516300511	1.50E-35	postive
IL3RA	AC026369.3	0.613700798	2.82E-53	postive

TYROBP	AC026369.3	0.524942213	6.76E-37	postive
CD4	AC108134.3	0.518638565	6.55E-36	postive
CD74	AC108134.3	0.545379657	3.05E-40	postive
CTSS	AC108134.3	0.543354259	6.70E-40	postive
HLA-DMA	AC108134.3	0.619964281	1.23E-54	postive
HLA-DMB	AC108134.3	0.554164569	9.43E-42	postive
HLA-DOA	AC108134.3	0.503565171	1.24E-33	postive
HLA-DPB1	AC108134.3	0.594631329	2.54E-49	postive
IFI30	AC108134.3	0.671240469	5.05E-67	postive
SLC11A1	AC108134.3	0.617539169	4.17E-54	postive
MARCO	AC108134.3	0.528040554	2.17E-37	postive
CD40LG	AC108134.3	0.56868128	2.39E-44	postive
TRIM22	AC108134.3	0.568567671	2.51E-44	postive
AGER	AC108134.3	0.546620086	1.88E-40	postive
CCL23	AC108134.3	0.539492316	2.96E-39	postive
CCR4	AC108134.3	0.513028676	4.76E-35	postive
FGR	AC108134.3	0.674093357	8.72E-68	postive
BTK	AC108134.3	0.652474383	3.29E-62	postive
VAV1	AC108134.3	0.523796973	1.02E-36	postive
PIK3R5	AC108134.3	0.550113028	4.75E-41	postive
INPP5D	AC108134.3	0.520034901	3.98E-36	postive
LILRB3	AC108134.3	0.630097387	6.71E-57	postive
RASGRP3	AC108134.3	0.560260596	7.95E-43	postive
C5AR1	AC108134.3	0.54425381	4.73E-40	postive
CCRL2	AC108134.3	0.599660281	2.44E-50	postive
GMFG	AC108134.3	0.615377845	1.23E-53	postive
IL16	AC108134.3	0.532196698	4.66E-38	postive
TNFSF12	AC108134.3	0.507780124	2.94E-34	postive
TNFSF14	AC108134.3	0.506164293	5.12E-34	postive
ACVRL1	AC108134.3	0.536765108	8.37E-39	postive
IL10RA	AC108134.3	0.617376569	4.53E-54	postive
IL12RB1	AC108134.3	0.536990427	7.68E-39	postive
IL3RA	AC108134.3	0.684544436	1.18E-70	postive
NR1H3	AC108134.3	0.533690112	2.67E-38	postive
S1PR1	AC108134.3	0.527299669	2.85E-37	postive
TIE1	AC108134.3	0.54465823	4.04E-40	postive
TNFRSF14	AC108134.3	0.530872737	7.63E-38	postive
TNFRSF1B	AC108134.3	0.517936004	8.42E-36	postive
ITGAL	AC108134.3	0.606727843	8.45E-52	postive
ITGB2	AC108134.3	0.52673362	3.51E-37	postive
TYROBP	AC108134.3	0.526671144	3.59E-37	postive
CD247	AC108134.3	0.570714529	1.01E-44	postive
ZAP70	AC108134.3	0.553683011	1.14E-41	postive
LCP2	AC108134.3	0.525222753	6.10E-37	postive
HCST	AC108134.3	0.542114045	1.08E-39	postive
CD3E	AC108134.3	0.521713099	2.18E-36	postive
PTPRC	AC108134.3	0.577800567	4.80E-46	postive
ITK	AC108134.3	0.587910261	5.44E-48	postive
GRAP2	AC108134.3	0.56336347	2.21E-43	postive
CD28	AC108134.3	0.524192429	8.88E-37	postive
ICOS	AC108134.3	0.521218887	2.60E-36	postive
CTLA4	AC108134.3	0.550497222	4.08E-41	postive
TRAV8-3	AC108134.3	0.503153557	1.43E-33	postive
TRBC1	AC108134.3	0.565951756	7.53E-44	postive
TRBC2	AC108134.3	0.528570979	1.79E-37	postive
TRBJ2-2	AC108134.3	0.5103175	1.22E-34	postive

TRBJ2-7	AC108134.3	0.547046446	1.59E-40	postive
TRBV18	AC108134.3	0.50566883	6.06E-34	postive
TRBV28	AC108134.3	0.539789305	2.65E-39	postive
NRTN	CASC15	0.550736514	3.71E-41	postive
DDX17	AL391834.1	0.512861292	5.04E-35	postive
GNRH1	AL391834.1	0.517977802	8.29E-36	postive
NFYC	AL604028.1	0.553207607	1.38E-41	postive
DDX17	AL022322.1	0.528607384	1.76E-37	postive
BIRC5	SNHG30	0.518088732	7.97E-36	postive
DDX17	AL021707.3	0.734541275	3.03E-86	postive
PLXNB1	AL021707.3	0.53997512	2.46E-39	postive
NR2C2	AL021707.3	0.511739708	7.46E-35	postive
S100A12	AC008735.4	0.616210074	8.11E-54	postive
NOS2	AC008735.4	0.628576348	1.49E-56	postive
NPR3	AC008735.4	0.535711922	1.25E-38	postive
GNRH1	AC108673.3	0.527894541	2.29E-37	postive
TNFRSF25	AC108673.3	0.513583823	3.92E-35	postive
FABP7	HOXC-AS1	0.564041162	1.67E-43	postive
DDX17	Z83843.1	0.577873897	4.65E-46	postive
ACVR2B	ACVR2B-AS1	0.537338223	6.73E-39	postive
CHGA	U91328.1	0.512366746	6.00E-35	postive
GHRH	U91328.1	0.513728209	3.72E-35	postive
SCG2	U91328.1	0.520332918	3.58E-36	postive
TK2	AL133355.1	0.512384267	5.96E-35	postive
SEM1	SLC12A9-AS1	0.505067181	7.45E-34	postive
SHC2	DICER1-AS1	0.538858771	3.77E-39	postive
GNRH1	MHENCR	0.562446528	3.23E-43	postive
TNFRSF25	MHENCR	0.540267141	2.20E-39	postive
CD1C	MIR155HG	0.567109861	4.63E-44	postive
CD1D	MIR155HG	0.520129058	3.85E-36	postive
CD8A	MIR155HG	0.558479059	1.65E-42	postive
CD74	MIR155HG	0.580336084	1.58E-46	postive
HLA-DMA	MIR155HG	0.624335549	1.33E-55	postive
HLA-DMB	MIR155HG	0.684710748	1.06E-70	postive
HLA-DOA	MIR155HG	0.573484315	3.10E-45	postive
HLA-DOB	MIR155HG	0.708860544	7.99E-78	postive
HLA-DPA1	MIR155HG	0.50615853	5.13E-34	postive
HLA-DPB1	MIR155HG	0.589281608	2.93E-48	postive
HLA-DQA2	MIR155HG	0.513924799	3.47E-35	postive
HLA-DRA	MIR155HG	0.52224993	1.79E-36	postive
IFNG	MIR155HG	0.501302399	2.67E-33	postive
LTA	MIR155HG	0.771426435	3.17E-100	postive
CIITA	MIR155HG	0.669689758	1.30E-66	postive
IFI30	MIR155HG	0.60887887	2.98E-52	postive
RBP5	MIR155HG	0.532429631	4.27E-38	postive
CETP	MIR155HG	0.631277549	3.61E-57	postive
ISG20	MIR155HG	0.512517473	5.69E-35	postive
CCL4	MIR155HG	0.57750891	5.45E-46	postive
CD40LG	MIR155HG	0.772130844	1.62E-100	postive
TRIM22	MIR155HG	0.570811727	9.70E-45	postive
PDCD1	MIR155HG	0.766907488	2.25E-98	postive
CCR7	MIR155HG	0.545614101	2.78E-40	postive
CCL21	MIR155HG	0.622457438	3.48E-55	postive
CCR5	MIR155HG	0.541483229	1.38E-39	postive
CXCR4	MIR155HG	0.665275567	1.87E-65	postive
CXCR6	MIR155HG	0.545611777	2.79E-40	postive

CCR4	MIR155HG	0.597301359	7.35E-50	postive
CD79A	MIR155HG	0.642896542	6.98E-60	postive
CD79B	MIR155HG	0.707003064	2.99E-77	postive
BTK	MIR155HG	0.744495494	8.85E-90	postive
VAV1	MIR155HG	0.614112995	2.30E-53	postive
RAC2	MIR155HG	0.553661976	1.15E-41	postive
CD19	MIR155HG	0.703035144	4.86E-76	postive
CR2	MIR155HG	0.630219953	6.29E-57	postive
PIK3CD	MIR155HG	0.595610301	1.61E-49	postive
INPP5D	MIR155HG	0.702558201	6.77E-76	postive
CD22	MIR155HG	0.661421013	1.84E-64	postive
CD72	MIR155HG	0.805869032	6.93E-116	postive
PTPN6	MIR155HG	0.564440495	1.41E-43	postive
RASGRP3	MIR155HG	0.681671854	7.44E-70	postive
PLCG2	MIR155HG	0.559214411	1.22E-42	postive
PRKCB	MIR155HG	0.694825929	1.34E-73	postive
IGLJ1	MIR155HG	0.644929797	2.27E-60	postive
CXCR3	MIR155HG	0.584672494	2.32E-47	postive
FLT3LG	MIR155HG	0.616771255	6.13E-54	postive
GMFG	MIR155HG	0.752079021	1.39E-92	postive
IL16	MIR155HG	0.716535996	3.05E-80	postive
LTB	MIR155HG	0.63217645	2.25E-57	postive
IL10RA	MIR155HG	0.662546068	9.48E-65	postive
IL12RB1	MIR155HG	0.755358541	7.90E-94	postive
IL18RAP	MIR155HG	0.61417104	2.23E-53	postive
IL21R	MIR155HG	0.603058212	4.89E-51	postive
IL2RG	MIR155HG	0.580850834	1.26E-46	postive
IL3RA	MIR155HG	0.511010762	9.62E-35	postive
NR1H3	MIR155HG	0.627561391	2.52E-56	postive
TNFRSF13C	MIR155HG	0.642334584	9.50E-60	postive
TNFRSF14	MIR155HG	0.576454149	8.61E-46	postive
TNFRSF1B	MIR155HG	0.535582769	1.31E-38	postive
ICAM2	MIR155HG	0.528204236	2.05E-37	postive
ITGAL	MIR155HG	0.759416417	2.14E-95	postive
LCK	MIR155HG	0.778675118	2.77E-103	postive
NCR3	MIR155HG	0.786174691	1.42E-106	postive
CD247	MIR155HG	0.775107074	9.17E-102	postive
ZAP70	MIR155HG	0.789912789	2.90E-108	postive
LCP2	MIR155HG	0.567146062	4.57E-44	postive
HCST	MIR155HG	0.651666122	5.21E-62	postive
CD48	MIR155HG	0.727338235	8.71E-84	postive
SH2D1A	MIR155HG	0.718203762	8.86E-81	postive
CD3D	MIR155HG	0.799417947	1.01E-112	postive
CD3E	MIR155HG	0.777387106	9.87E-103	postive
CD3G	MIR155HG	0.590935638	1.38E-48	postive
PTPRC	MIR155HG	0.740622557	2.19E-88	postive
ITK	MIR155HG	0.737788568	2.22E-87	postive
GRAP2	MIR155HG	0.713691414	2.45E-79	postive
CD28	MIR155HG	0.683981038	1.69E-70	postive
ICOS	MIR155HG	0.746882165	1.19E-90	postive
CTLA4	MIR155HG	0.74524571	4.72E-90	postive
TRAC	MIR155HG	0.771812293	2.20E-100	postive
TRAV2	MIR155HG	0.653487149	1.84E-62	postive
TRAV4	MIR155HG	0.642918796	6.90E-60	postive
TRAV8-3	MIR155HG	0.761768207	2.55E-96	postive
TRAV8-6	MIR155HG	0.627671876	2.38E-56	postive

TRAV9-2	MIR155HG	0.672116068	2.95E-67	postive
TRAV12-1	MIR155HG	0.585449916	1.64E-47	postive
TRAV12-2	MIR155HG	0.600382239	1.74E-50	postive
TRAV12-3	MIR155HG	0.572457345	4.81E-45	postive
TRAV13-1	MIR155HG	0.595901901	1.41E-49	postive
TRAV21	MIR155HG	0.501529948	2.47E-33	postive
TRBC1	MIR155HG	0.781300721	2.02E-104	postive
TRBC2	MIR155HG	0.825406283	3.04E-126	postive
TRBJ2-1	MIR155HG	0.672589876	2.21E-67	postive
TRBJ2-2	MIR155HG	0.685483284	6.42E-71	postive
TRBJ2-3	MIR155HG	0.749580862	1.19E-91	postive
TRBJ2-7	MIR155HG	0.73816815	1.63E-87	postive
TRBV2	MIR155HG	0.733520602	6.83E-86	postive
TRBV3-1	MIR155HG	0.697490107	2.21E-74	postive
TRBV5-1	MIR155HG	0.752866559	7.00E-93	postive
TRBV5-4	MIR155HG	0.614741015	1.68E-53	postive
TRBV5-6	MIR155HG	0.612782524	4.43E-53	postive
TRBV6-1	MIR155HG	0.627272861	2.93E-56	postive
TRBV6-5	MIR155HG	0.65138644	6.10E-62	postive
TRBV7-9	MIR155HG	0.62713317	3.15E-56	postive
TRBV9	MIR155HG	0.601363201	1.09E-50	postive
TRBV10-3	MIR155HG	0.61610659	8.54E-54	postive
TRBV12-4	MIR155HG	0.546932169	1.66E-40	postive
TRBV18	MIR155HG	0.733720899	5.82E-86	postive
TRBV19	MIR155HG	0.617072068	5.27E-54	postive
TRBV20-1	MIR155HG	0.674924083	5.21E-68	postive
TRBV28	MIR155HG	0.801940645	6.05E-114	postive
TRBV29-1	MIR155HG	0.677323253	1.17E-68	postive
CALCA	AL024508.1	0.557323854	2.63E-42	postive
THPO	AL024508.1	0.524564877	7.75E-37	postive
XCL1	AL139089.1	0.521428878	2.41E-36	postive
GNRH1	AL139089.1	0.528320684	1.96E-37	postive
GNRH1	AP001107.4	0.520174631	3.78E-36	postive
DDX17	AC084018.1	0.596498223	1.07E-49	postive
PLXNB1	AC084018.1	0.55423088	9.18E-42	postive
GNRH1	AC084018.1	0.517028013	1.16E-35	postive
NR2C2	AC084018.1	0.500198728	3.86E-33	postive
DDX17	LINC01355	0.528588477	1.78E-37	postive
PLXNB1	LINC01355	0.511672768	7.64E-35	postive
GNRH1	LINC01355	0.647129556	6.70E-61	postive
NR2C1	LINC01355	0.505124523	7.30E-34	postive
TNFRSF25	LINC01355	0.594488128	2.71E-49	postive
PLCG1	LINC01355	0.557685138	2.27E-42	postive
CD1C	FAM30A	0.632034553	2.42E-57	postive
CD1D	FAM30A	0.554125809	9.58E-42	postive
HLA-DMA	FAM30A	0.509216414	1.79E-34	postive
HLA-DOB	FAM30A	0.829465334	1.47E-128	postive
LTA	FAM30A	0.69600253	6.06E-74	postive
CIITA	FAM30A	0.551266191	3.00E-41	postive
RBP5	FAM30A	0.532214337	4.63E-38	postive
CETP	FAM30A	0.677555688	1.01E-68	postive
ISG20	FAM30A	0.560628488	6.83E-43	postive
CD40LG	FAM30A	0.822598947	1.12E-124	postive
PDCD1	FAM30A	0.678045732	7.40E-69	postive
CCR7	FAM30A	0.584487395	2.52E-47	postive
CCL21	FAM30A	0.711853854	9.31E-79	postive

CXCR4	FAM30A	0.665197334	1.96E-65	postive
CCR4	FAM30A	0.63468678	5.93E-58	postive
CD79A	FAM30A	0.902243004	8.92E-185	postive
CD79B	FAM30A	0.807757452	7.80E-117	postive
BTK	FAM30A	0.763228597	6.74E-97	postive
VAV1	FAM30A	0.582061684	7.40E-47	postive
RAC2	FAM30A	0.52126629	2.56E-36	postive
CD19	FAM30A	0.831882184	5.75E-130	postive
CR2	FAM30A	0.716480036	3.18E-80	postive
PIK3R5	FAM30A	0.502656496	1.69E-33	postive
PIK3CD	FAM30A	0.53889783	3.72E-39	postive
INPP5D	FAM30A	0.738518356	1.23E-87	postive
CD22	FAM30A	0.723455476	1.71E-82	postive
CD72	FAM30A	0.748294298	3.58E-91	postive
PTPN6	FAM30A	0.541228144	1.52E-39	postive
RASGRP3	FAM30A	0.711826164	9.50E-79	postive
PLCG2	FAM30A	0.762121533	1.85E-96	postive
PRKCB	FAM30A	0.747791349	5.50E-91	postive
IGHA1	FAM30A	0.574893024	1.69E-45	postive
IGHA2	FAM30A	0.588302734	4.56E-48	postive
IGHD1-1	FAM30A	0.621170789	6.69E-55	postive
IGHD6-25	FAM30A	0.645252368	1.90E-60	postive
IGHG1	FAM30A	0.542692803	8.66E-40	postive
IGHG2	FAM30A	0.640831171	2.16E-59	postive
IGHG3	FAM30A	0.604698628	2.24E-51	postive
IGHG4	FAM30A	0.58532576	1.74E-47	postive
IGHJ2	FAM30A	0.57215344	5.48E-45	postive
IGHJ3	FAM30A	0.594010328	3.38E-49	postive
IGHV1-2	FAM30A	0.539000948	3.58E-39	postive
IGHV1-46	FAM30A	0.520157298	3.81E-36	postive
IGHV1-69	FAM30A	0.569794968	1.49E-44	postive
IGHV3-15	FAM30A	0.520054545	3.95E-36	postive
IGHV3-23	FAM30A	0.51184658	7.19E-35	postive
IGHV3-35	FAM30A	0.511225404	8.93E-35	postive
IGHV3-7	FAM30A	0.505670431	6.06E-34	postive
IGHV4-28	FAM30A	0.57980594	2.00E-46	postive
IGHV4-31	FAM30A	0.536025656	1.11E-38	postive
IGHV4-34	FAM30A	0.525290373	5.95E-37	postive
IGHV4-61	FAM30A	0.555912782	4.66E-42	postive
IGHV5-51	FAM30A	0.548216699	1.00E-40	postive
IGKC	FAM30A	0.618200112	3.00E-54	postive
IGKJ5	FAM30A	0.760712536	6.64E-96	postive
IGKV1-13	FAM30A	0.512647491	5.44E-35	postive
IGKV1-39	FAM30A	0.51562528	1.91E-35	postive
IGKV1-5	FAM30A	0.541062007	1.62E-39	postive
IGKV1D-39	FAM30A	0.558827993	1.43E-42	postive
IGKV1D-42	FAM30A	0.518749595	6.30E-36	postive
IGKV1D-8	FAM30A	0.522933563	1.40E-36	postive
IGKV3-11	FAM30A	0.556939025	3.08E-42	postive
IGKV3-15	FAM30A	0.552774964	1.65E-41	postive
IGKV3-20	FAM30A	0.604724552	2.21E-51	postive
IGKV3-7	FAM30A	0.581032262	1.17E-46	postive
IGKV3D-11	FAM30A	0.570328955	1.19E-44	postive
IGKV3D-15	FAM30A	0.555015686	6.70E-42	postive
IGKV3D-7	FAM30A	0.53028689	9.48E-38	postive
IGLC2	FAM30A	0.605541023	1.49E-51	postive

IGLC3	FAM30A	0.540274612	2.20E-39	postive
IGLC6	FAM30A	0.53068015	8.20E-38	postive
IGLJ1	FAM30A	0.902539177	4.34E-185	postive
IGLJ2	FAM30A	0.573514797	3.06E-45	postive
IGLJ3	FAM30A	0.648085787	3.92E-61	postive
IGLV1-40	FAM30A	0.601358248	1.09E-50	postive
IGLV1-44	FAM30A	0.598566942	4.07E-50	postive
IGLV2-14	FAM30A	0.505677323	6.05E-34	postive
FLT3LG	FAM30A	0.508742845	2.11E-34	postive
GMFG	FAM30A	0.612706466	4.60E-53	postive
IL16	FAM30A	0.838477744	6.33E-134	postive
LTB	FAM30A	0.61332178	3.39E-53	postive
IL10RA	FAM30A	0.709213657	6.21E-78	postive
IL12RB1	FAM30A	0.670021533	1.06E-66	postive
IL21R	FAM30A	0.529437185	1.30E-37	postive
TNFRSF13C	FAM30A	0.756344829	3.31E-94	postive
TNFRSF14	FAM30A	0.502689715	1.67E-33	postive
TNFRSF17	FAM30A	0.661277512	2.01E-64	postive
ITGAL	FAM30A	0.751228815	2.89E-92	postive
LCK	FAM30A	0.647019555	7.12E-61	postive
NCR3	FAM30A	0.782819879	4.36E-105	postive
CD247	FAM30A	0.683001669	3.18E-70	postive
ZAP70	FAM30A	0.757844581	8.72E-95	postive
CD48	FAM30A	0.502507463	1.78E-33	postive
SH2D1A	FAM30A	0.51807657	8.01E-36	postive
CD3D	FAM30A	0.595065842	2.07E-49	postive
CD3E	FAM30A	0.692523691	6.27E-73	postive
PTPRC	FAM30A	0.679204103	3.57E-69	postive
ITK	FAM30A	0.736937218	4.42E-87	postive
GRAP2	FAM30A	0.649427526	1.85E-61	postive
CD28	FAM30A	0.734079853	4.37E-86	postive
ICOS	FAM30A	0.596615717	1.01E-49	postive
CTLA4	FAM30A	0.645711222	1.47E-60	postive
TRAC	FAM30A	0.596100342	1.28E-49	postive
TRAV2	FAM30A	0.503090897	1.46E-33	postive
TRAV8-3	FAM30A	0.664175893	3.60E-65	postive
TRAV8-6	FAM30A	0.560885342	6.15E-43	postive
TRAV9-2	FAM30A	0.590296785	1.85E-48	postive
TRBC1	FAM30A	0.725220072	4.45E-83	postive
TRBC2	FAM30A	0.724971951	5.38E-83	postive
TRBJ2-1	FAM30A	0.673376172	1.36E-67	postive
TRBJ2-2	FAM30A	0.707813318	1.69E-77	postive
TRBJ2-3	FAM30A	0.69140488	1.32E-72	postive
TRBJ2-7	FAM30A	0.760099869	1.16E-95	postive
TRBV2	FAM30A	0.677887388	8.18E-69	postive
TRBV3-1	FAM30A	0.615761655	1.01E-53	postive
TRBV5-1	FAM30A	0.691961795	9.12E-73	postive
TRBV5-4	FAM30A	0.62894305	1.23E-56	postive
TRBV5-6	FAM30A	0.56535289	9.67E-44	postive
TRBV6-1	FAM30A	0.609603201	2.10E-52	postive
TRBV6-5	FAM30A	0.592990495	5.40E-49	postive
TRBV10-3	FAM30A	0.506783606	4.14E-34	postive
TRBV18	FAM30A	0.695954289	6.27E-74	postive
TRBV19	FAM30A	0.588223215	4.73E-48	postive
TRBV20-1	FAM30A	0.598676308	3.87E-50	postive
TRBV28	FAM30A	0.728180446	4.54E-84	postive

TRBV29-1	FAM30A	0.670622468	7.38E-67	postive
CTF1	CD2BP2-DT	0.536201444	1.04E-38	postive
SFTPD	AC005479.2	0.505034653	7.53E-34	postive
SFTPD	AC079467.1	0.578855596	3.03E-46	postive
SFTPA1	AC079467.1	0.57781009	4.78E-46	postive
SFTPA2	AC079467.1	0.599598365	2.51E-50	postive
AGER	AC079467.1	0.543934424	5.35E-40	postive
NPR1	AC079467.1	0.511705045	7.55E-35	postive
TEK	AC079467.1	0.568853845	2.22E-44	postive
TNFRSF10C	AC079467.1	0.505725943	5.95E-34	postive
IGLV8-61	AC004982.2	0.505299144	6.88E-34	postive
SCTR	AC004982.2	0.543726897	5.80E-40	postive
TPT1	LINC00324	0.50793464	2.79E-34	postive
GMFG	LINC00324	0.511747854	7.44E-35	postive
CD48	LINC00324	0.569503791	1.69E-44	postive
NR2C2	AP003392.1	0.540479462	2.03E-39	postive
IL27RA	LINC01315	0.538926586	3.68E-39	postive
PLXNB1	LINC00106	0.518030407	8.14E-36	postive
GNRH1	LINC00106	0.544650493	4.05E-40	postive
NCK1	NCK1-DT	0.669543731	1.42E-66	postive
GNRH1	AC024075.2	0.505092058	7.38E-34	postive
DDX17	RAD51-AS1	0.607904611	4.79E-52	postive
PLXNB1	RAD51-AS1	0.534398731	2.04E-38	postive
GNRH1	RAD51-AS1	0.59439628	2.83E-49	postive
NR2C1	RAD51-AS1	0.561256445	5.28E-43	postive
NR2C2	RAD51-AS1	0.504404455	9.33E-34	postive
TNFRSF25	RAD51-AS1	0.532583763	4.03E-38	postive
B2M	LINC01871	0.52621759	4.24E-37	postive
CD8A	LINC01871	0.78605863	1.60E-106	postive
FCER1G	LINC01871	0.647139666	6.66E-61	postive
HLA-DMB	LINC01871	0.529353118	1.34E-37	postive
HLA-DRA	LINC01871	0.516287542	1.51E-35	postive
IFNG	LINC01871	0.828298368	6.90E-128	postive
CXCL9	LINC01871	0.570644734	1.04E-44	postive
RBP4	LINC01871	0.578636144	3.33E-46	postive
ORM2	LINC01871	0.513849967	3.57E-35	postive
IRF1	LINC01871	0.542664216	8.76E-40	postive
CCL5	LINC01871	0.560734295	6.54E-43	postive
TLR8	LINC01871	0.512439521	5.85E-35	postive
GNLY	LINC01871	0.585782953	1.42E-47	postive
APOM	LINC01871	0.646435923	9.86E-61	postive
PLA2G2A	LINC01871	0.652644458	2.98E-62	postive
CCL4	LINC01871	0.733496754	6.96E-86	postive
PDCD1	LINC01871	0.513435067	4.13E-35	postive
FASLG	LINC01871	0.701264137	1.66E-75	postive
APOH	LINC01871	0.623165715	2.42E-55	postive
CCL3	LINC01871	0.561946747	3.97E-43	postive
CCL4L2	LINC01871	0.508201153	2.54E-34	postive
XCL2	LINC01871	0.815564048	7.16E-121	postive
CXCR6	LINC01871	0.788987171	7.65E-108	postive
CXCR3	LINC01871	0.530312745	9.39E-38	postive
AGT	LINC01871	0.600901856	1.36E-50	postive
GMFG	LINC01871	0.618454116	2.64E-54	postive
IL12RB1	LINC01871	0.515377668	2.08E-35	postive
IL18RAP	LINC01871	0.769789473	1.50E-99	postive
NR1H3	LINC01871	0.619597516	1.48E-54	postive

CD247	LINC01871	0.550482054	4.10E-41	postive
HCST	LINC01871	0.611222788	9.52E-53	postive
CD48	LINC01871	0.700991874	2.00E-75	postive
CD244	LINC01871	0.745413345	4.10E-90	postive
SH2D1A	LINC01871	0.648812515	2.61E-61	postive
GZMB	LINC01871	0.811432207	1.03E-118	postive
PRF1	LINC01871	0.708473149	1.05E-77	postive
CD3D	LINC01871	0.799837789	6.35E-113	postive
CD3E	LINC01871	0.550056916	4.85E-41	postive
CD3G	LINC01871	0.541305484	1.48E-39	postive
ICOS	LINC01871	0.502903904	1.55E-33	postive
CTLA4	LINC01871	0.506444116	4.65E-34	postive
TRAC	LINC01871	0.531826005	5.35E-38	postive
TRAV4	LINC01871	0.634649391	6.05E-58	postive
TRAV12-1	LINC01871	0.501815404	2.24E-33	postive
TRAV12-2	LINC01871	0.592778155	5.95E-49	postive
TRBC2	LINC01871	0.595199328	1.95E-49	postive
TRBV7-9	LINC01871	0.63099964	4.18E-57	postive
TRBV9	LINC01871	0.773585575	4.00E-101	postive
TRBV10-3	LINC01871	0.532644103	3.94E-38	postive
TRBV12-4	LINC01871	0.668495568	2.69E-66	postive
TRBV28	LINC01871	0.522110184	1.89E-36	postive
TRDC	LINC01871	0.677736501	8.99E-69	postive
LMBR1L	AL513218.1	0.555826028	4.83E-42	postive
GNRH1	AL513218.1	0.616563682	6.80E-54	postive
TNFRSF25	AL513218.1	0.597143846	7.91E-50	postive
PLCG1	AL513218.1	0.519722014	4.45E-36	postive
OSGIN1	AL365181.3	0.512891887	4.99E-35	postive
DDX17	AC012615.6	0.557099166	2.88E-42	postive
PLXNB1	AC012615.6	0.588411769	4.34E-48	postive
GNRH1	AC012615.6	0.588457763	4.25E-48	postive
TNFRSF25	AC012615.6	0.593983164	3.42E-49	postive
CD4	AP002954.1	0.614597611	1.81E-53	postive
CD74	AP002954.1	0.630273547	6.12E-57	postive
CTSS	AP002954.1	0.619514896	1.55E-54	postive
FCER1G	AP002954.1	0.636216382	2.62E-58	postive
HLA-DMA	AP002954.1	0.634594351	6.23E-58	postive
HLA-DMB	AP002954.1	0.605105976	1.84E-51	postive
HLA-DOA	AP002954.1	0.513230032	4.43E-35	postive
HLA-DPA1	AP002954.1	0.606819244	8.08E-52	postive
HLA-DPB1	AP002954.1	0.651161838	6.93E-62	postive
HLA-DQA1	AP002954.1	0.526172553	4.31E-37	postive
HLA-DQB1	AP002954.1	0.527390029	2.76E-37	postive
HLA-DRA	AP002954.1	0.581095086	1.13E-46	postive
HLA-DRB1	AP002954.1	0.587475107	6.62E-48	postive
CYBB	AP002954.1	0.620537186	9.23E-55	postive
TLR7	AP002954.1	0.598191643	4.85E-50	postive
TLR8	AP002954.1	0.608102705	4.35E-52	postive
MSR1	AP002954.1	0.57469787	1.84E-45	postive
CCL4	AP002954.1	0.521319587	2.51E-36	postive
MARCO	AP002954.1	0.529855936	1.11E-37	postive
CD14	AP002954.1	0.586906318	8.55E-48	postive
CCR5	AP002954.1	0.569534025	1.67E-44	postive
CCR1	AP002954.1	0.600621679	1.55E-50	postive
FGR	AP002954.1	0.548457446	9.13E-41	postive
CD86	AP002954.1	0.597982542	5.35E-50	postive

BTK	AP002954.1	0.535937984	1.14E-38	postive
LILRB3	AP002954.1	0.555310194	5.95E-42	postive
C5AR1	AP002954.1	0.526068064	4.48E-37	postive
CCRL2	AP002954.1	0.606662214	8.72E-52	postive
CMKLR1	AP002954.1	0.611973396	6.59E-53	postive
CXCR3	AP002954.1	0.508452722	2.33E-34	postive
PLXNC1	AP002954.1	0.520978213	2.84E-36	postive
GMFG	AP002954.1	0.550499228	4.07E-41	postive
TNFSF12	AP002954.1	0.616319413	7.68E-54	postive
TNFSF13	AP002954.1	0.514363106	2.98E-35	postive
TNFSF13B	AP002954.1	0.588704614	3.80E-48	postive
ACVRL1	AP002954.1	0.515563884	1.95E-35	postive
C3AR1	AP002954.1	0.624662812	1.13E-55	postive
CSF1R	AP002954.1	0.569223372	1.90E-44	postive
CSF2RA	AP002954.1	0.516569497	1.37E-35	postive
ENG	AP002954.1	0.539107442	3.43E-39	postive
IL10RA	AP002954.1	0.543147444	7.26E-40	postive
IL3RA	AP002954.1	0.512861214	5.04E-35	postive
TNFRSF1B	AP002954.1	0.526680187	3.58E-37	postive
ITGB2	AP002954.1	0.581072069	1.15E-46	postive
TYROBP	AP002954.1	0.666537977	8.76E-66	postive
FCGR3A	AP002954.1	0.638520068	7.58E-59	postive
LCP2	AP002954.1	0.574218216	2.26E-45	postive
HCST	AP002954.1	0.617690785	3.87E-54	postive
PTPRC	AP002954.1	0.50404909	1.05E-33	postive
GNRH1	AP001160.1	0.614200868	2.20E-53	postive
NR2C1	AP001160.1	0.548437787	9.20E-41	postive
TNFRSF25	AP001160.1	0.549039324	7.26E-41	postive
PIK3R5	AC002091.1	0.511807595	7.29E-35	postive
CD8B	LINC02404	0.751064407	3.34E-92	postive
DEFA5	LINC02404	0.960111704	7.10E-279	postive
PENK	LINC02404	0.734116387	4.25E-86	postive
CST4	LINC02404	0.70040145	3.01E-75	postive
DKK1	LINC02404	0.643434109	5.19E-60	postive
FGF3	LINC02404	0.555127704	6.40E-42	postive
TAC1	LINC02404	0.563389486	2.19E-43	postive
CXCL2	LINC01936	0.546134404	2.27E-40	postive
COLEC12	LINC01936	0.522692176	1.53E-36	postive
A2M	LINC01936	0.661505976	1.75E-64	postive
SFTPA1	LINC01936	0.537855573	5.53E-39	postive
MSR1	LINC01936	0.58012608	1.74E-46	postive
SLC11A1	LINC01936	0.589982573	2.13E-48	postive
MARCO	LINC01936	0.648530184	3.06E-61	postive
AGER	LINC01936	0.684319527	1.36E-70	postive
CCL23	LINC01936	0.582418142	6.32E-47	postive
SEMA3G	LINC01936	0.529913075	1.09E-37	postive
C5AR1	LINC01936	0.534855935	1.72E-38	postive
CX3CR1	LINC01936	0.543924812	5.37E-40	postive
AGRP	LINC01936	0.707664675	1.87E-77	postive
VEGFD	LINC01936	0.682807208	3.60E-70	postive
GDF10	LINC01936	0.608607457	3.40E-52	postive
RETN	LINC01936	0.721422501	7.97E-82	postive
TNFSF12	LINC01936	0.538767103	3.91E-39	postive
TNFSF13	LINC01936	0.574129557	2.35E-45	postive
ACVRL1	LINC01936	0.651914844	4.52E-62	postive
ENG	LINC01936	0.518606373	6.63E-36	postive

IL3RA	LINC01936	0.54457075	4.18E-40	postive
NPR1	LINC01936	0.527285799	2.87E-37	postive
TEK	LINC01936	0.537612061	6.07E-39	postive
TIE1	LINC01936	0.514489826	2.85E-35	postive
B2M	USP30-AS1	0.640676763	2.35E-59	postive
CD4	USP30-AS1	0.580246874	1.65E-46	postive
CD8A	USP30-AS1	0.708091025	1.38E-77	postive
CD74	USP30-AS1	0.767011322	2.04E-98	postive
CTSS	USP30-AS1	0.68554156	6.18E-71	postive
FCER1G	USP30-AS1	0.608042925	4.48E-52	postive
HLA-A	USP30-AS1	0.681282585	9.54E-70	postive
HLA-B	USP30-AS1	0.707066045	2.86E-77	postive
HLA-C	USP30-AS1	0.663887534	4.28E-65	postive
HLA-DMA	USP30-AS1	0.760162308	1.09E-95	postive
HLA-DMB	USP30-AS1	0.709672608	4.47E-78	postive
HLA-DOA	USP30-AS1	0.634931485	5.21E-58	postive
HLA-DOB	USP30-AS1	0.572417768	4.89E-45	postive
HLA-DPA1	USP30-AS1	0.701001953	1.99E-75	postive
HLA-DPB1	USP30-AS1	0.737840072	2.13E-87	postive
HLA-DQA1	USP30-AS1	0.612758679	4.48E-53	postive
HLA-DQA2	USP30-AS1	0.538874699	3.75E-39	postive
HLA-DQB1	USP30-AS1	0.573683851	2.85E-45	postive
HLA-DRA	USP30-AS1	0.641958463	1.17E-59	postive
HLA-DRB1	USP30-AS1	0.654805252	8.66E-63	postive
HLA-DRB5	USP30-AS1	0.514472529	2.87E-35	postive
HLA-E	USP30-AS1	0.718615516	6.53E-81	postive
HLA-F	USP30-AS1	0.738669488	1.08E-87	postive
HLA-H	USP30-AS1	0.571006421	8.93E-45	postive
IFNG	USP30-AS1	0.629447312	9.43E-57	postive
LTA	USP30-AS1	0.542419467	9.62E-40	postive
CIITA	USP30-AS1	0.584142497	2.94E-47	postive
PSMB8	USP30-AS1	0.568616594	2.46E-44	postive
PSME1	USP30-AS1	0.55294663	1.54E-41	postive
PSME2	USP30-AS1	0.617507727	4.24E-54	postive
TAP1	USP30-AS1	0.670402644	8.43E-67	postive
TAP2	USP30-AS1	0.620838787	7.92E-55	postive
IFI30	USP30-AS1	0.573236041	3.45E-45	postive
TAPBPL	USP30-AS1	0.555616477	5.26E-42	postive
CXCL9	USP30-AS1	0.62113813	6.81E-55	postive
CYBB	USP30-AS1	0.579148559	2.67E-46	postive
ISG20	USP30-AS1	0.5998886	2.19E-50	postive
STAT1	USP30-AS1	0.624442712	1.26E-55	postive
IRF1	USP30-AS1	0.779701525	9.99E-104	postive
IL15	USP30-AS1	0.581044013	1.16E-46	postive
PLAAT4	USP30-AS1	0.542978946	7.75E-40	postive
CCL5	USP30-AS1	0.562753855	2.85E-43	postive
TLR8	USP30-AS1	0.579387538	2.40E-46	postive
GNLY	USP30-AS1	0.51843507	7.05E-36	postive
CCL4	USP30-AS1	0.70401773	2.45E-76	postive
CD40LG	USP30-AS1	0.568909931	2.17E-44	postive
TRIM22	USP30-AS1	0.683054516	3.07E-70	postive
PDCD1	USP30-AS1	0.717886734	1.12E-80	postive
FASLG	USP30-AS1	0.709566372	4.83E-78	postive
GBP2	USP30-AS1	0.616515715	6.96E-54	postive
CCR5	USP30-AS1	0.715245488	7.87E-80	postive
CCR1	USP30-AS1	0.529845225	1.12E-37	postive

CXCR6	USP30-AS1	0.679546112	2.87E-69	postive
FGR	USP30-AS1	0.54738112	1.39E-40	postive
CD86	USP30-AS1	0.568033418	3.14E-44	postive
BTK	USP30-AS1	0.628468183	1.57E-56	postive
VAV1	USP30-AS1	0.582517619	6.05E-47	postive
RAC2	USP30-AS1	0.538166479	4.91E-39	postive
INPP5D	USP30-AS1	0.555302084	5.97E-42	postive
CD72	USP30-AS1	0.52903163	1.51E-37	postive
PTPN6	USP30-AS1	0.563475863	2.11E-43	postive
RASGRP3	USP30-AS1	0.513686283	3.78E-35	postive
PRKCB	USP30-AS1	0.533376509	3.00E-38	postive
CCRL2	USP30-AS1	0.563947836	1.74E-43	postive
CMKLR1	USP30-AS1	0.586915679	8.51E-48	postive
CXCR3	USP30-AS1	0.717623279	1.36E-80	postive
ADA2	USP30-AS1	0.530631376	8.35E-38	postive
FLT3LG	USP30-AS1	0.631222216	3.72E-57	postive
GMFG	USP30-AS1	0.652444371	3.34E-62	postive
IL16	USP30-AS1	0.561409256	4.96E-43	postive
SECTM1	USP30-AS1	0.560689237	6.66E-43	postive
TNFSF12	USP30-AS1	0.542019171	1.12E-39	postive
TNFSF13B	USP30-AS1	0.585085075	1.93E-47	postive
IL10RA	USP30-AS1	0.670000466	1.08E-66	postive
IL12RB1	USP30-AS1	0.753744367	3.25E-93	postive
IL15RA	USP30-AS1	0.657901875	1.45E-63	postive
IL2RB	USP30-AS1	0.569879888	1.44E-44	postive
IL18RAP	USP30-AS1	0.581342247	1.02E-46	postive
IL21R	USP30-AS1	0.564928018	1.15E-43	postive
IL2RG	USP30-AS1	0.617636033	3.98E-54	postive
IL3RA	USP30-AS1	0.532900296	3.58E-38	postive
NR1H3	USP30-AS1	0.639512783	4.43E-59	postive
TNFRSF14	USP30-AS1	0.591379113	1.13E-48	postive
TNFRSF1B	USP30-AS1	0.614425836	1.97E-53	postive
ICAM2	USP30-AS1	0.50285212	1.58E-33	postive
ITGAL	USP30-AS1	0.693423283	3.44E-73	postive
ITGB2	USP30-AS1	0.593388355	4.49E-49	postive
TYROBP	USP30-AS1	0.58875604	3.71E-48	postive
LCK	USP30-AS1	0.631154562	3.85E-57	postive
FCGR3A	USP30-AS1	0.542178409	1.06E-39	postive
NCR3	USP30-AS1	0.537265262	6.92E-39	postive
CD247	USP30-AS1	0.742723235	3.87E-89	postive
ZAP70	USP30-AS1	0.593131133	5.06E-49	postive
LCP2	USP30-AS1	0.666743377	7.75E-66	postive
HCST	USP30-AS1	0.702690932	6.17E-76	postive
CD48	USP30-AS1	0.533444962	2.92E-38	postive
SH2D1A	USP30-AS1	0.612934632	4.11E-53	postive
GZMB	USP30-AS1	0.592381912	7.13E-49	postive
PRF1	USP30-AS1	0.67060556	7.45E-67	postive
CD3D	USP30-AS1	0.713806816	2.25E-79	postive
CD3E	USP30-AS1	0.760730677	6.54E-96	postive
CD3G	USP30-AS1	0.598679089	3.86E-50	postive
PTPRC	USP30-AS1	0.651666861	5.20E-62	postive
ITK	USP30-AS1	0.644474228	2.93E-60	postive
GRAP2	USP30-AS1	0.643071938	6.34E-60	postive
CD28	USP30-AS1	0.537561799	6.18E-39	postive
ICOS	USP30-AS1	0.66852958	2.63E-66	postive
CTLA4	USP30-AS1	0.646661126	8.70E-61	postive

TRAC	USP30-AS1	0.728070743	4.94E-84	postive
TRAV2	USP30-AS1	0.625025478	9.34E-56	postive
TRAV4	USP30-AS1	0.613735756	2.77E-53	postive
TRAV8-3	USP30-AS1	0.657585217	1.74E-63	postive
TRAV8-6	USP30-AS1	0.624321962	1.34E-55	postive
TRAV9-2	USP30-AS1	0.573000654	3.81E-45	postive
TRAV12-1	USP30-AS1	0.538039815	5.16E-39	postive
TRAV12-2	USP30-AS1	0.613312233	3.41E-53	postive
TRAV12-3	USP30-AS1	0.593447597	4.37E-49	postive
TRAV13-1	USP30-AS1	0.661006022	2.35E-64	postive
TRAV21	USP30-AS1	0.560564934	7.01E-43	postive
TRBC1	USP30-AS1	0.679820072	2.42E-69	postive
TRBC2	USP30-AS1	0.733445322	7.25E-86	postive
TRBJ2-1	USP30-AS1	0.613265395	3.49E-53	postive
TRBJ2-2	USP30-AS1	0.554012919	1.00E-41	postive
TRBJ2-3	USP30-AS1	0.528091597	2.13E-37	postive
TRBJ2-7	USP30-AS1	0.608731126	3.21E-52	postive
TRBV2	USP30-AS1	0.569061551	2.04E-44	postive
TRBV3-1	USP30-AS1	0.574698461	1.84E-45	postive
TRBV4-1	USP30-AS1	0.502886289	1.56E-33	postive
TRBV4-2	USP30-AS1	0.529498128	1.27E-37	postive
TRBV5-1	USP30-AS1	0.63256457	1.83E-57	postive
TRBV5-4	USP30-AS1	0.561328297	5.12E-43	postive
TRBV5-6	USP30-AS1	0.58746375	6.65E-48	postive
TRBV6-1	USP30-AS1	0.591339582	1.15E-48	postive
TRBV6-5	USP30-AS1	0.665313074	1.83E-65	postive
TRBV7-9	USP30-AS1	0.599273302	2.92E-50	postive
TRBV9	USP30-AS1	0.525092235	6.40E-37	postive
TRBV10-3	USP30-AS1	0.535429861	1.39E-38	postive
TRBV18	USP30-AS1	0.595126769	2.02E-49	postive
TRBV19	USP30-AS1	0.618914418	2.09E-54	postive
TRBV20-1	USP30-AS1	0.586392609	1.08E-47	postive
TRBV28	USP30-AS1	0.705905138	6.50E-77	postive
TRBV29-1	USP30-AS1	0.586373231	1.09E-47	postive
CD74	AL157394.1	0.563773492	1.87E-43	postive
HLA-DOA	AL157394.1	0.535074307	1.58E-38	postive
ICAM1	AL157394.1	0.504446269	9.20E-34	postive
CIITA	AL157394.1	0.602098595	7.71E-51	postive
RELB	AL157394.1	0.634052477	8.32E-58	postive
RFX5	AL157394.1	0.507725926	3.00E-34	postive
TAPBP	AL157394.1	0.55459316	7.94E-42	postive
APOBEC3G	AL157394.1	0.51516924	2.24E-35	postive
IL15	AL157394.1	0.59157173	1.03E-48	postive
CD40	AL157394.1	0.504316552	9.61E-34	postive
CCL5	AL157394.1	0.530275141	9.52E-38	postive
TLR1	AL157394.1	0.53778828	5.67E-39	postive
STING1	AL157394.1	0.501236974	2.73E-33	postive
APOBEC3H	AL157394.1	0.508328962	2.43E-34	postive
VCAM1	AL157394.1	0.657989115	1.37E-63	postive
CCR5	AL157394.1	0.502225411	1.95E-33	postive
JAK2	AL157394.1	0.574240049	2.24E-45	postive
NFKBIE	AL157394.1	0.582397268	6.38E-47	postive
IL15RA	AL157394.1	0.570952636	9.14E-45	postive
IL18R1	AL157394.1	0.506073365	5.28E-34	postive
IL21R	AL157394.1	0.623883619	1.68E-55	postive
IL2RG	AL157394.1	0.673462433	1.29E-67	postive

SSTR2	AL157394.1	0.567085828	4.68E-44	postive
TNFRSF14	AL157394.1	0.549794064	5.39E-41	postive
TNFRSF1B	AL157394.1	0.599511561	2.61E-50	postive
TNFRSF9	AL157394.1	0.605531384	1.50E-51	postive
FAS	AL157394.1	0.859778664	4.86E-148	postive
PLXNB1	AC002128.1	0.5160048	1.67E-35	postive
MC1R	AC002128.1	0.522416078	1.69E-36	postive
PAK5	AL109976.1	0.552472504	1.86E-41	postive
HGF	AC091849.2	0.676148011	2.43E-68	postive
TAFA5	AC091849.2	0.501150556	2.81E-33	postive
AZGP1	AP000851.2	0.559609803	1.04E-42	postive
S100A1	AP000851.2	0.645745688	1.45E-60	postive
PLCG1	SNHG11	0.533922651	2.44E-38	postive
IRF3	LENG8-AS1	0.502118992	2.02E-33	postive
DDX17	LENG8-AS1	0.500849462	3.11E-33	postive
CD4	AC090559.1	0.871381653	9.51E-157	postive
CD74	AC090559.1	0.735161844	1.84E-86	postive
CTSS	AC090559.1	0.768023102	7.93E-99	postive
FCER1G	AC090559.1	0.661280608	2.00E-64	postive
FCGRT	AC090559.1	0.572353375	5.03E-45	postive
HLA-DMA	AC090559.1	0.68367667	2.06E-70	postive
HLA-DMB	AC090559.1	0.686990892	2.41E-71	postive
HLA-DOA	AC090559.1	0.67284618	1.88E-67	postive
HLA-DPA1	AC090559.1	0.754373036	1.88E-93	postive
HLA-DPB1	AC090559.1	0.763200946	6.91E-97	postive
HLA-DQA1	AC090559.1	0.644353523	3.13E-60	postive
HLA-DQB1	AC090559.1	0.585498326	1.61E-47	postive
HLA-DRA	AC090559.1	0.641792154	1.28E-59	postive
HLA-DRB1	AC090559.1	0.652694095	2.90E-62	postive
HLA-DRB5	AC090559.1	0.542682976	8.69E-40	postive
HLA-E	AC090559.1	0.527526445	2.63E-37	postive
LGMN	AC090559.1	0.608399012	3.77E-52	postive
IFI30	AC090559.1	0.517569468	9.59E-36	postive
CD209	AC090559.1	0.558911029	1.38E-42	postive
CXCL16	AC090559.1	0.602574634	6.15E-51	postive
COLEC12	AC090559.1	0.571553555	7.08E-45	postive
TLR4	AC090559.1	0.542613535	8.93E-40	postive
A2M	AC090559.1	0.639592996	4.24E-59	postive
TLR2	AC090559.1	0.521229466	2.59E-36	postive
CYBB	AC090559.1	0.849297736	8.03E-141	postive
TLR7	AC090559.1	0.78873732	9.93E-108	postive
LYZ	AC090559.1	0.561590089	4.60E-43	postive
CYLD	AC090559.1	0.513065185	4.70E-35	postive
TLR8	AC090559.1	0.745466083	3.92E-90	postive
TK2	AC090559.1	0.501396758	2.58E-33	postive
MSR1	AC090559.1	0.740991321	1.62E-88	postive
SLC11A1	AC090559.1	0.651560737	5.53E-62	postive
MARCO	AC090559.1	0.623662285	1.88E-55	postive
IL7R	AC090559.1	0.574017618	2.47E-45	postive
CD14	AC090559.1	0.70694979	3.11E-77	postive
TRIM22	AC090559.1	0.593691628	3.91E-49	postive
AQP9	AC090559.1	0.633957018	8.75E-58	postive
ANXA6	AC090559.1	0.580335637	1.58E-46	postive
VIM	AC090559.1	0.543220512	7.06E-40	postive
SLC29A3	AC090559.1	0.519285268	5.20E-36	postive
CCR8	AC090559.1	0.616212374	8.10E-54	postive

CCR5	AC090559.1	0.704031905	2.42E-76	postive
CCL23	AC090559.1	0.566765008	5.36E-44	postive
CCR1	AC090559.1	0.776373797	2.67E-102	postive
CCR4	AC090559.1	0.613275658	3.47E-53	postive
FGR	AC090559.1	0.782755398	4.66E-105	postive
CD86	AC090559.1	0.750437901	5.72E-92	postive
BTK	AC090559.1	0.70902805	7.09E-78	postive
VAV1	AC090559.1	0.602998917	5.03E-51	postive
PIK3R5	AC090559.1	0.735613902	1.28E-86	postive
INPP5D	AC090559.1	0.54564898	2.75E-40	postive
LILRB3	AC090559.1	0.804486295	3.38E-115	postive
FCGR2B	AC090559.1	0.62532893	7.99E-56	postive
RASGRP3	AC090559.1	0.558904298	1.38E-42	postive
PRKCB	AC090559.1	0.572934111	3.92E-45	postive
C5AR1	AC090559.1	0.793213603	8.73E-110	postive
CCRL2	AC090559.1	0.717266942	1.78E-80	postive
CMKLR1	AC090559.1	0.789640203	3.86E-108	postive
CX3CR1	AC090559.1	0.647959833	4.21E-61	postive
CXCR3	AC090559.1	0.561312594	5.16E-43	postive
FPR1	AC090559.1	0.650264251	1.15E-61	postive
FPR2	AC090559.1	0.506134492	5.17E-34	postive
PLXNC1	AC090559.1	0.774946599	1.07E-101	postive
PLXND1	AC090559.1	0.538969926	3.62E-39	postive
PTAFR	AC090559.1	0.628068055	1.94E-56	postive
ADA2	AC090559.1	0.722534229	3.44E-82	postive
CSF1	AC090559.1	0.534337534	2.09E-38	postive
GMFG	AC090559.1	0.584045355	3.07E-47	postive
IL16	AC090559.1	0.588996282	3.33E-48	postive
IL6ST	AC090559.1	0.502491726	1.79E-33	postive
TNFSF12	AC090559.1	0.699508358	5.57E-75	postive
TNFSF13	AC090559.1	0.705417208	9.17E-77	postive
TNFSF13B	AC090559.1	0.614129084	2.28E-53	postive
ACVRL1	AC090559.1	0.702830672	5.60E-76	postive
C3AR1	AC090559.1	0.805635114	9.07E-116	postive
CSF1R	AC090559.1	0.843925825	2.48E-137	postive
CSF2RA	AC090559.1	0.635224129	4.46E-58	postive
ENG	AC090559.1	0.723924093	1.20E-82	postive
FLT4	AC090559.1	0.574121544	2.36E-45	postive
IL10RA	AC090559.1	0.77948459	1.24E-103	postive
IL12RB1	AC090559.1	0.600260613	1.84E-50	postive
IL2RG	AC090559.1	0.505327857	6.81E-34	postive
IL3RA	AC090559.1	0.670268927	9.15E-67	postive
NRP1	AC090559.1	0.535003256	1.63E-38	postive
S1PR1	AC090559.1	0.536924684	7.88E-39	postive
TEK	AC090559.1	0.550315962	4.38E-41	postive
TGFBR2	AC090559.1	0.627625691	2.44E-56	postive
TIE1	AC090559.1	0.599289277	2.90E-50	postive
TNFRSF1B	AC090559.1	0.750469016	5.57E-92	postive
ICAM2	AC090559.1	0.563529257	2.06E-43	postive
ITGAL	AC090559.1	0.627432298	2.69E-56	postive
ITGB2	AC090559.1	0.78311924	3.22E-105	postive
TYROBP	AC090559.1	0.734703358	2.66E-86	postive
FCGR3A	AC090559.1	0.709341331	5.67E-78	postive
CD247	AC090559.1	0.549144747	6.96E-41	postive
LCP2	AC090559.1	0.785609934	2.54E-106	postive
HCST	AC090559.1	0.612065843	6.30E-53	postive

CD3E	AC090559.1	0.527763239	2.41E-37	postive
PTPRC	AC090559.1	0.726903388	1.22E-83	postive
ITK	AC090559.1	0.599042571	3.26E-50	postive
GRAP2	AC090559.1	0.522963878	1.39E-36	postive
CD28	AC090559.1	0.638724169	6.79E-59	postive
ICOS	AC090559.1	0.587199102	7.50E-48	postive
TRAC	AC090559.1	0.509646298	1.54E-34	postive
TRAV8-6	AC090559.1	0.55908501	1.29E-42	postive
TRAV9-2	AC090559.1	0.513858091	3.56E-35	postive
TRAV13-1	AC090559.1	0.537160044	7.20E-39	postive
TRBC1	AC090559.1	0.552900807	1.57E-41	postive
TRBV6-5	AC090559.1	0.50485298	8.01E-34	postive
TRBV19	AC090559.1	0.506241154	4.99E-34	postive
TRBV20-1	AC090559.1	0.528457512	1.86E-37	postive
TRBV29-1	AC090559.1	0.528394819	1.91E-37	postive
PNOC	STARD4-AS1	0.746148591	2.21E-90	postive
AGTR1	STARD4-AS1	0.609380664	2.34E-52	postive
RARA	RARA-AS1	0.62759206	2.48E-56	postive
SEMA3B	LMNTD2-AS1	0.579015529	2.82E-46	postive
NRTN	LMNTD2-AS1	0.58025637	1.64E-46	postive
VIM	MIR100HG	0.506137583	5.17E-34	postive
NPY	MIR100HG	0.568764269	2.31E-44	postive
GNRH1	SCAT2	0.601830227	8.76E-51	postive
NR2C1	SCAT2	0.565905276	7.68E-44	postive
TNFRSF25	SCAT2	0.529730858	1.17E-37	postive
PROCR	AL161431.1	0.513837144	3.58E-35	postive
VGF	AL161431.1	0.882053268	1.53E-165	postive
CD4	HLA-DQB1-AS1	0.514667079	2.68E-35	postive
CD74	HLA-DQB1-AS1	0.646494744	9.54E-61	postive
CTSS	HLA-DQB1-AS1	0.511154027	9.15E-35	postive
HLA-B	HLA-DQB1-AS1	0.523168378	1.29E-36	postive
HLA-DMA	HLA-DQB1-AS1	0.684167663	1.50E-70	postive
HLA-DMB	HLA-DQB1-AS1	0.611451738	8.51E-53	postive
HLA-DOA	HLA-DQB1-AS1	0.636049662	2.87E-58	postive
HLA-DOB	HLA-DQB1-AS1	0.520582093	3.27E-36	postive
HLA-DPA1	HLA-DQB1-AS1	0.585306881	1.75E-47	postive
HLA-DPB1	HLA-DQB1-AS1	0.632962397	1.48E-57	postive
HLA-DQA1	HLA-DQB1-AS1	0.591595825	1.02E-48	postive
HLA-DQB1	HLA-DQB1-AS1	0.733002503	1.03E-85	postive
HLA-DRA	HLA-DQB1-AS1	0.567949297	3.26E-44	postive
HLA-DRB1	HLA-DQB1-AS1	0.648161532	3.76E-61	postive
HLA-DRB5	HLA-DQB1-AS1	0.66762612	4.55E-66	postive
HLA-E	HLA-DQB1-AS1	0.524983439	6.66E-37	postive
HLA-F	HLA-DQB1-AS1	0.555941117	4.61E-42	postive
CIITA	HLA-DQB1-AS1	0.634811824	5.55E-58	postive
IFI30	HLA-DQB1-AS1	0.658807889	8.53E-64	postive
CYBB	HLA-DQB1-AS1	0.502115761	2.03E-33	postive
CD40LG	HLA-DQB1-AS1	0.562223587	3.54E-43	postive
TRIM22	HLA-DQB1-AS1	0.570983569	9.02E-45	postive
PDCD1	HLA-DQB1-AS1	0.541110911	1.59E-39	postive
FGR	HLA-DQB1-AS1	0.533838191	2.52E-38	postive
CD86	HLA-DQB1-AS1	0.500250278	3.80E-33	postive
BTK	HLA-DQB1-AS1	0.620972701	7.40E-55	postive
VAV1	HLA-DQB1-AS1	0.54322223	7.06E-40	postive
PIK3R5	HLA-DQB1-AS1	0.530412313	9.05E-38	postive
INPP5D	HLA-DQB1-AS1	0.60537176	1.62E-51	postive

CD72	HLA-DQB1-AS1	0.523104308	1.32E-36	postive
LILRB3	HLA-DQB1-AS1	0.527068676	3.11E-37	postive
RASGRP3	HLA-DQB1-AS1	0.55390903	1.05E-41	postive
PRKCB	HLA-DQB1-AS1	0.546781363	1.76E-40	postive
GMFG	HLA-DQB1-AS1	0.568578734	2.50E-44	postive
IL16	HLA-DQB1-AS1	0.564988406	1.13E-43	postive
IL10RA	HLA-DQB1-AS1	0.609863678	1.85E-52	postive
IL12RB1	HLA-DQB1-AS1	0.631920979	2.57E-57	postive
IL21R	HLA-DQB1-AS1	0.525708027	5.11E-37	postive
IL2RG	HLA-DQB1-AS1	0.506818027	4.09E-34	postive
IL3RA	HLA-DQB1-AS1	0.60495988	1.97E-51	postive
TNFRSF14	HLA-DQB1-AS1	0.538199834	4.85E-39	postive
TNFRSF1B	HLA-DQB1-AS1	0.588731596	3.76E-48	postive
ITGAL	HLA-DQB1-AS1	0.624909453	9.92E-56	postive
NCR3	HLA-DQB1-AS1	0.508356632	2.41E-34	postive
CD247	HLA-DQB1-AS1	0.592982245	5.42E-49	postive
ZAP70	HLA-DQB1-AS1	0.596504034	1.07E-49	postive
LCP2	HLA-DQB1-AS1	0.536552585	9.07E-39	postive
HCST	HLA-DQB1-AS1	0.53964873	2.79E-39	postive
CD3D	HLA-DQB1-AS1	0.506626395	4.37E-34	postive
CD3E	HLA-DQB1-AS1	0.574677009	1.86E-45	postive
PTPRC	HLA-DQB1-AS1	0.613918194	2.53E-53	postive
ITK	HLA-DQB1-AS1	0.608295479	3.96E-52	postive
GRAP2	HLA-DQB1-AS1	0.575137398	1.52E-45	postive
CD28	HLA-DQB1-AS1	0.555666005	5.15E-42	postive
ICOS	HLA-DQB1-AS1	0.597110257	8.03E-50	postive
CTLA4	HLA-DQB1-AS1	0.560516303	7.15E-43	postive
TRAC	HLA-DQB1-AS1	0.536263841	1.01E-38	postive
TRAV8-3	HLA-DQB1-AS1	0.529732285	1.16E-37	postive
TRBC1	HLA-DQB1-AS1	0.601490858	1.03E-50	postive
TRBC2	HLA-DQB1-AS1	0.57940355	2.38E-46	postive
TRBJ2-1	HLA-DQB1-AS1	0.588404473	4.35E-48	postive
TRBJ2-2	HLA-DQB1-AS1	0.545871091	2.52E-40	postive
TRBJ2-3	HLA-DQB1-AS1	0.508331398	2.43E-34	postive
TRBJ2-7	HLA-DQB1-AS1	0.632273912	2.14E-57	postive
TRBV6-5	HLA-DQB1-AS1	0.51007695	1.33E-34	postive
TRBV18	HLA-DQB1-AS1	0.505361135	6.74E-34	postive
TRBV19	HLA-DQB1-AS1	0.504402835	9.34E-34	postive
TRBV28	HLA-DQB1-AS1	0.565484903	9.15E-44	postive
TRBV29-1	HLA-DQB1-AS1	0.528700641	1.70E-37	postive
CMTM7	AC121757.1	0.50475012	8.30E-34	postive
DDX17	ALG13-AS1	0.520502859	3.36E-36	postive
VGF	GAS6-DT	0.729512955	1.61E-84	postive
PSMD3	AC009283.1	0.542367138	9.82E-40	postive
NR0B2	AC007541.1	0.536024881	1.11E-38	postive
SFTPD	LHFPL3-AS2	0.635136557	4.67E-58	postive
A2M	LHFPL3-AS2	0.506198959	5.06E-34	postive
SFTPA1	LHFPL3-AS2	0.623956518	1.62E-55	postive
SFTPA2	LHFPL3-AS2	0.614812967	1.62E-53	postive
AGER	LHFPL3-AS2	0.5425454	9.17E-40	postive
VEGFD	LHFPL3-AS2	0.520304366	3.61E-36	postive
AGTR2	LHFPL3-AS2	0.622663186	3.13E-55	postive
NPR1	LHFPL3-AS2	0.571289173	7.92E-45	postive
ROBO2	DUBR	0.526217521	4.24E-37	postive
SEMA5A	SNHG18	0.521594839	2.27E-36	postive
FOS	NR4A1AS	0.599914242	2.16E-50	postive

NR4A1	NR4A1AS	0.632829684	1.59E-57	postive
CD1C	AC145098.1	0.650684672	9.08E-62	postive
CD1D	AC145098.1	0.569255193	1.88E-44	postive
CD4	AC145098.1	0.676927097	1.49E-68	postive
CD74	AC145098.1	0.645913613	1.32E-60	postive
CTSS	AC145098.1	0.567341554	4.21E-44	postive
HLA-DMA	AC145098.1	0.665769982	1.39E-65	postive
HLA-DMB	AC145098.1	0.651944706	4.44E-62	postive
HLA-DOA	AC145098.1	0.660620799	2.95E-64	postive
HLA-DOB	AC145098.1	0.700201629	3.46E-75	postive
HLA-DPA1	AC145098.1	0.568937946	2.15E-44	postive
HLA-DPB1	AC145098.1	0.670338645	8.77E-67	postive
HLA-DQA2	AC145098.1	0.528625703	1.75E-37	postive
LTA	AC145098.1	0.729850128	1.23E-84	postive
CIITA	AC145098.1	0.646379534	1.02E-60	postive
IFI30	AC145098.1	0.620076891	1.16E-54	postive
RBP5	AC145098.1	0.555916852	4.66E-42	postive
CETP	AC145098.1	0.716454605	3.24E-80	postive
CYBB	AC145098.1	0.602982978	5.07E-51	postive
TLR7	AC145098.1	0.580822282	1.28E-46	postive
CYLD	AC145098.1	0.571958431	5.95E-45	postive
IL7R	AC145098.1	0.536039302	1.10E-38	postive
CD40LG	AC145098.1	0.834299619	2.13E-131	postive
TRIM22	AC145098.1	0.580693963	1.35E-46	postive
PIK3CG	AC145098.1	0.505221639	7.06E-34	postive
PDCD1	AC145098.1	0.712562231	5.57E-79	postive
ANXA6	AC145098.1	0.582087476	7.32E-47	postive
CCR7	AC145098.1	0.579772965	2.03E-46	postive
CCR8	AC145098.1	0.52888909	1.59E-37	postive
CCL21	AC145098.1	0.688252501	1.06E-71	postive
CCR5	AC145098.1	0.576140133	9.87E-46	postive
CCR1	AC145098.1	0.537599852	6.10E-39	postive
CXCR4	AC145098.1	0.688310066	1.02E-71	postive
CCR4	AC145098.1	0.707973834	1.50E-77	postive
FGR	AC145098.1	0.655121141	7.23E-63	postive
CD86	AC145098.1	0.541913504	1.17E-39	postive
CD79A	AC145098.1	0.747704507	5.92E-91	postive
CD79B	AC145098.1	0.718559651	6.80E-81	postive
BTK	AC145098.1	0.867677857	7.03E-154	postive
VAV1	AC145098.1	0.681177741	1.02E-69	postive
RAC2	AC145098.1	0.620964799	7.43E-55	postive
NFATC2	AC145098.1	0.507731174	2.99E-34	postive
CD19	AC145098.1	0.720272342	1.89E-81	postive
CR2	AC145098.1	0.651325689	6.32E-62	postive
PIK3R5	AC145098.1	0.676771159	1.65E-68	postive
PIK3CD	AC145098.1	0.615898881	9.47E-54	postive
INPP5D	AC145098.1	0.811500836	9.54E-119	postive
CD22	AC145098.1	0.665402293	1.73E-65	postive
CD72	AC145098.1	0.762831814	9.68E-97	postive
PTPN6	AC145098.1	0.6422222	1.01E-59	postive
LILRB3	AC145098.1	0.609216206	2.53E-52	postive
FCGR2B	AC145098.1	0.552201915	2.07E-41	postive
RASGRP3	AC145098.1	0.808452744	3.47E-117	postive
PLCG2	AC145098.1	0.715632844	5.92E-80	postive
PRKCB	AC145098.1	0.809030762	1.76E-117	postive
IGKJ5	AC145098.1	0.548316795	9.65E-41	postive

IGLJ1	AC145098.1	0.733581116	6.51E-86	postive
C5AR1	AC145098.1	0.541607399	1.32E-39	postive
CCRL2	AC145098.1	0.550075493	4.82E-41	postive
CMKLR1	AC145098.1	0.60476749	2.16E-51	postive
CXCR3	AC145098.1	0.564151491	1.59E-43	postive
ACKR1	AC145098.1	0.533837088	2.52E-38	postive
PLXNC1	AC145098.1	0.576942626	6.97E-46	postive
ADA2	AC145098.1	0.639843055	3.70E-59	postive
FLT3LG	AC145098.1	0.511840907	7.20E-35	postive
GMFG	AC145098.1	0.672177172	2.84E-67	postive
IL16	AC145098.1	0.862156155	9.27E-150	postive
LTB	AC145098.1	0.626966183	3.43E-56	postive
TNFSF12	AC145098.1	0.553689763	1.14E-41	postive
TNFSF14	AC145098.1	0.52804951	2.17E-37	postive
ACVRL1	AC145098.1	0.522575703	1.59E-36	postive
CSF1R	AC145098.1	0.576261649	9.36E-46	postive
ENG	AC145098.1	0.551000284	3.34E-41	postive
FLT4	AC145098.1	0.553343143	1.31E-41	postive
IL10RA	AC145098.1	0.846056267	1.06E-138	postive
IL12RB1	AC145098.1	0.764061627	3.14E-97	postive
IL21R	AC145098.1	0.629159041	1.10E-56	postive
IL2RG	AC145098.1	0.594412613	2.80E-49	postive
IL3RA	AC145098.1	0.664534431	2.91E-65	postive
S1PR1	AC145098.1	0.54465034	4.05E-40	postive
S1PR2	AC145098.1	0.548413473	9.29E-41	postive
TIE1	AC145098.1	0.561529018	4.72E-43	postive
TNFRSF13C	AC145098.1	0.642732135	7.64E-60	postive
TNFRSF14	AC145098.1	0.563775772	1.86E-43	postive
TNFRSF1B	AC145098.1	0.708470437	1.06E-77	postive
ICAM2	AC145098.1	0.548372862	9.44E-41	postive
ITGAL	AC145098.1	0.852396432	6.74E-143	postive
ITGB2	AC145098.1	0.647805389	4.59E-61	postive
LCK	AC145098.1	0.679402642	3.15E-69	postive
NCR3	AC145098.1	0.74192065	7.53E-89	postive
CD247	AC145098.1	0.753336364	4.65E-93	postive
ZAP70	AC145098.1	0.748713773	2.51E-91	postive
LCP2	AC145098.1	0.667633841	4.53E-66	postive
HCST	AC145098.1	0.580081014	1.77E-46	postive
SH2D1A	AC145098.1	0.515049654	2.34E-35	postive
CD3D	AC145098.1	0.589053335	3.25E-48	postive
CD3E	AC145098.1	0.73457736	2.94E-86	postive
CD3G	AC145098.1	0.503058684	1.47E-33	postive
PTPRC	AC145098.1	0.821890493	2.75E-124	postive
ITK	AC145098.1	0.784493005	7.96E-106	postive
GRAP2	AC145098.1	0.748492668	3.03E-91	postive
CD28	AC145098.1	0.803264501	1.36E-114	postive
ICOS	AC145098.1	0.702148789	9.00E-76	postive
CTLA4	AC145098.1	0.678133709	7.01E-69	postive
TRAC	AC145098.1	0.650139855	1.24E-61	postive
TRAV2	AC145098.1	0.587392521	6.87E-48	postive
TRAV4	AC145098.1	0.53108714	7.05E-38	postive
TRAV8-3	AC145098.1	0.703039046	4.85E-76	postive
TRAV8-6	AC145098.1	0.653071774	2.34E-62	postive
TRAV9-2	AC145098.1	0.671839948	3.50E-67	postive
TRAV12-1	AC145098.1	0.545686934	2.71E-40	postive
TRAV12-3	AC145098.1	0.529207971	1.41E-37	postive

TRAV13-1	AC145098.1	0.584936182	2.07E-47	postive
TRAV21	AC145098.1	0.52155104	2.31E-36	postive
TRBC1	AC145098.1	0.789233615	5.91E-108	postive
TRBC2	AC145098.1	0.724182558	9.84E-83	postive
TRBJ2-1	AC145098.1	0.718468162	7.28E-81	postive
TRBJ2-2	AC145098.1	0.69702159	3.04E-74	postive
TRBJ2-3	AC145098.1	0.735412606	1.51E-86	postive
TRBJ2-7	AC145098.1	0.782572506	5.61E-105	postive
TRBV2	AC145098.1	0.698097308	1.46E-74	postive
TRBV3-1	AC145098.1	0.655850082	4.75E-63	postive
TRBV5-1	AC145098.1	0.709798868	4.09E-78	postive
TRBV5-4	AC145098.1	0.625739085	6.47E-56	postive
TRBV5-6	AC145098.1	0.615587285	1.11E-53	postive
TRBV6-1	AC145098.1	0.598345176	4.51E-50	postive
TRBV6-5	AC145098.1	0.612035127	6.39E-53	postive
TRBV7-9	AC145098.1	0.539462209	3.00E-39	postive
TRBV10-3	AC145098.1	0.543392531	6.61E-40	postive
TRBV18	AC145098.1	0.737555189	2.68E-87	postive
TRBV19	AC145098.1	0.631871268	2.64E-57	postive
TRBV20-1	AC145098.1	0.670544245	7.74E-67	postive
TRBV28	AC145098.1	0.750878125	3.92E-92	postive
TRBV29-1	AC145098.1	0.712543893	5.65E-79	postive
PLCG1	ZNF528-AS1	0.581858411	8.10E-47	postive
PIK3CA	AC090425.3	0.507365639	3.39E-34	postive
CXCR4	AC007384.1	0.536924928	7.87E-39	postive
IGLV2-14	AC007384.1	0.500273912	3.77E-33	postive
DEFA6	LINC02178	0.76431458	2.49E-97	postive
SERPIND1	LINC02178	0.734822174	2.42E-86	postive
MAPK14	LINC02178	0.53691674	7.90E-39	postive
BMP4	LINC02178	0.61975727	1.37E-54	postive
CHGB	LINC02178	0.758272024	5.96E-95	postive
FGF3	LINC02178	0.749155014	1.72E-91	postive
TNFRSF19	LINC02178	0.5805838	1.42E-46	postive
PLXNB1	AC002553.2	0.525192487	6.17E-37	postive
GNRH1	AC002553.2	0.637039917	1.68E-58	postive
LMBR1L	AL591895.1	0.531536914	5.96E-38	postive
DDX17	AL591895.1	0.517562343	9.61E-36	postive
GNRH1	AL591895.1	0.573833283	2.67E-45	postive
TNFSF8	AL391069.2	0.613516447	3.08E-53	postive
PLXNB1	AC021078.1	0.50638755	4.74E-34	postive
GNRH1	AC021078.1	0.592540475	6.63E-49	postive
DDX17	PAN3-AS1	0.5027232	1.65E-33	postive
CD4	TBX5-AS1	0.512478271	5.77E-35	postive
COLEC12	TBX5-AS1	0.622797548	2.93E-55	postive
A2M	TBX5-AS1	0.597508848	6.67E-50	postive
PDGFRB	TBX5-AS1	0.591495295	1.07E-48	postive
ANXA6	TBX5-AS1	0.538670903	4.05E-39	postive
PLXNC1	TBX5-AS1	0.521081247	2.73E-36	postive
CLEC11A	TBX5-AS1	0.517296972	1.06E-35	postive
LTBP2	TBX5-AS1	0.59127795	1.18E-48	postive
ACVRL1	TBX5-AS1	0.614947278	1.52E-53	postive
ANGPTL2	TBX5-AS1	0.571737676	6.54E-45	postive
ENG	TBX5-AS1	0.544187917	4.85E-40	postive
FLT4	TBX5-AS1	0.609710096	1.99E-52	postive
TIE1	TBX5-AS1	0.529737073	1.16E-37	postive
IGHD6-25	AL022067.1	0.506437979	4.66E-34	postive

IGHG2	AL022067.1	0.528018554	2.19E-37	postive
IGHG3	AL022067.1	0.504926357	7.81E-34	postive
IGHJ2	AL022067.1	0.50547567	6.48E-34	postive
IGHV4-31	AL022067.1	0.524186991	8.89E-37	postive
IGKJ5	AL022067.1	0.503099355	1.45E-33	postive
IGLV1-40	AL022067.1	0.500622232	3.35E-33	postive
IGLV1-44	AL022067.1	0.524011035	9.48E-37	postive
OSGIN1	AL033397.1	0.541643583	1.30E-39	postive
CXCL2	MIR223HG	0.526822624	3.40E-37	postive
SLC11A1	MIR223HG	0.557461867	2.49E-42	postive
AQP9	MIR223HG	0.534828101	1.74E-38	postive
AGER	MIR223HG	0.51234143	6.05E-35	postive
CXCR1	MIR223HG	0.678272327	6.42E-69	postive
FGR	MIR223HG	0.511657893	7.68E-35	postive
LILRB3	MIR223HG	0.6104409	1.40E-52	postive
CCRL2	MIR223HG	0.547054889	1.58E-40	postive
FPR1	MIR223HG	0.598789185	3.67E-50	postive
FPR2	MIR223HG	0.608656346	3.33E-52	postive
CXCR2	MIR223HG	0.598621019	3.97E-50	postive
CMTM2	MIR223HG	0.658993568	7.66E-64	postive
CSF3R	MIR223HG	0.708032619	1.44E-77	postive
FCGR3B	MIR223HG	0.662556904	9.42E-65	postive
PTGS2	AL158166.1	0.527439662	2.71E-37	postive
IL1RL1	AL158166.1	0.662693209	8.69E-65	postive
CDK4	AL158166.1	0.624538089	1.20E-55	postive
DDX17	AC008870.2	0.528037854	2.18E-37	postive
PLXNB1	AC008870.2	0.557130764	2.85E-42	postive
GNRH1	AC008870.2	0.635498623	3.85E-58	postive
NR2C1	AC008870.2	0.516386795	1.46E-35	postive
TNFRSF25	AC008870.2	0.520127876	3.85E-36	postive
VTN	GLIS2-AS1	0.550852945	3.54E-41	postive
CHGA	GLIS2-AS1	0.648041885	4.02E-61	postive
GHRH	GLIS2-AS1	0.649633227	1.64E-61	postive
SCG2	GLIS2-AS1	0.648713018	2.76E-61	postive
TRH	GLIS2-AS1	0.646977097	7.29E-61	postive
GNRH1	AC011462.4	0.532775478	3.75E-38	postive
NR5A1	AC092916.1	0.518440863	7.03E-36	postive
NRTN	UNC5B-AS1	0.529702001	1.18E-37	postive
INHA	AL513304.1	0.505036675	7.52E-34	postive
OSGIN1	AL590666.2	0.515795669	1.80E-35	postive
APOM	AC008915.3	0.564304375	1.50E-43	postive
PLA2G2A	AC008915.3	0.559514348	1.08E-42	postive
APOH	AC008915.3	0.553446185	1.26E-41	postive
XCL2	AC008915.3	0.551036594	3.29E-41	postive
AGT	AC008915.3	0.650365632	1.09E-61	postive
NOX4	LINC01614	0.50886225	2.02E-34	postive
F2R	LINC01614	0.509703083	1.51E-34	postive
PDGFRB	LINC01614	0.640670484	2.36E-59	postive
TNFSF4	LINC01614	0.553784312	1.10E-41	postive
EDNRA	LINC01614	0.548439306	9.20E-41	postive
PLXNC1	LINC01614	0.532654614	3.93E-38	postive
CCN2	LINC01614	0.58968708	2.44E-48	postive
GREM1	LINC01614	0.67308412	1.63E-67	postive
INHBA	LINC01614	0.54951473	6.02E-41	postive
PDGFRL	LINC01614	0.652427252	3.38E-62	postive
ANGPTL2	LINC01614	0.604568513	2.38E-51	postive

DDX17	AL049840.2	0.500255211	3.79E-33	postive
PLXNA3	AL049840.2	0.509659043	1.54E-34	postive
NRTN	AC074389.2	0.501032721	2.92E-33	postive
DDX17	PSMA3-AS1	0.619910532	1.27E-54	postive
IRF9	PSMA3-AS1	0.504703787	8.43E-34	postive
GNRH1	PSMA3-AS1	0.641032904	1.94E-59	postive
PRDX2	MAPKAPK5-AS1	0.51735897	1.03E-35	postive
BIRC5	MAPKAPK5-AS1	0.505991811	5.43E-34	postive
TPT1	SNHG29	0.607299522	6.41E-52	postive
PTHLH	AC008011.2	0.824526363	9.47E-126	postive
PAEP	AC083809.1	0.889445481	3.81E-172	postive
BMP5	AC083809.1	0.673648507	1.15E-67	postive
IL17RE	MIR1915HG	0.589480633	2.68E-48	postive
SH3BP2	AL158212.3	0.500929187	3.02E-33	postive
GNRH1	AC048341.2	0.596195692	1.23E-49	postive
PLXNB1	AC020558.2	0.520767241	3.06E-36	postive
CIITA	AP001062.1	0.50229431	1.91E-33	postive
TSLP	LINC00626	0.569951487	1.40E-44	postive
IRF3	AC010326.3	0.556723267	3.36E-42	postive
GNRH1	AC010326.3	0.558691191	1.51E-42	postive
TNFRSF25	AC010326.3	0.52587533	4.81E-37	postive
DEFB4A	PARAL1	0.539973001	2.47E-39	postive
DCK	UBA6-AS1	0.525337975	5.85E-37	postive
CD40LG	HCG27	0.517043238	1.16E-35	postive
CD19	HCG27	0.516575592	1.36E-35	postive
CD72	HCG27	0.53414729	2.25E-38	postive
IGLJ1	HCG27	0.533448034	2.92E-38	postive
TNFRSF13C	HCG27	0.509580039	1.58E-34	postive
TNFRSF25	HCG27	0.51819511	7.68E-36	postive
ITGAL	HCG27	0.500670886	3.30E-33	postive
ZAP70	HCG27	0.565829195	7.92E-44	postive
TRBC1	HCG27	0.50805788	2.67E-34	postive
TRBJ2-2	HCG27	0.511186866	9.05E-35	postive
TRBJ2-7	HCG27	0.557213802	2.75E-42	postive
NOD1	GARS1-DT	0.552685856	1.71E-41	postive
DDX17	GARS1-DT	0.546398368	2.05E-40	postive
GNRH1	GARS1-DT	0.621552308	5.52E-55	postive
TNFRSF25	GARS1-DT	0.521436625	2.40E-36	postive
HGF	LINC02866	0.663202319	6.43E-65	postive
PCSK1	LINC02866	0.530528946	8.67E-38	postive
CD19	AL353622.1	0.5222144	1.82E-36	postive
IGLJ1	AL353622.1	0.541041359	1.64E-39	postive
GNRH1	AL353622.1	0.583711154	3.57E-47	postive
TNFRSF13C	AL353622.1	0.525526532	5.46E-37	postive
TNFRSF14	AL353622.1	0.544334144	4.58E-40	postive
TNFRSF25	AL353622.1	0.569788127	1.50E-44	postive
ZAP70	AL353622.1	0.577169248	6.32E-46	postive
TRBJ2-7	AL353622.1	0.535752754	1.23E-38	postive
CD1C	LINC02576	0.526440261	3.91E-37	postive
CD1D	LINC02576	0.528059139	2.16E-37	postive
HLA-DMA	LINC02576	0.504187692	1.00E-33	postive
HLA-DMB	LINC02576	0.520040667	3.97E-36	postive
HLA-DOB	LINC02576	0.799769535	6.85E-113	postive
LTA	LINC02576	0.629406019	9.64E-57	postive
CETP	LINC02576	0.547905413	1.13E-40	postive
ISG20	LINC02576	0.578620731	3.36E-46	postive

CD40LG	LINC02576	0.745287093	4.56E-90	postive
PIK3CG	LINC02576	0.528507326	1.83E-37	postive
PDCD1	LINC02576	0.641188203	1.78E-59	postive
CCL21	LINC02576	0.596367108	1.14E-49	postive
CXCR4	LINC02576	0.681563631	7.98E-70	postive
CCR4	LINC02576	0.594053499	3.31E-49	postive
CD79A	LINC02576	0.86043237	1.65E-148	postive
CD79B	LINC02576	0.715665685	5.78E-80	postive
BTK	LINC02576	0.7228069	2.80E-82	postive
VAV1	LINC02576	0.61578493	1.00E-53	postive
CD19	LINC02576	0.717705133	1.28E-80	postive
CR2	LINC02576	0.600447379	1.68E-50	postive
PIK3R5	LINC02576	0.506157739	5.13E-34	postive
INPP5D	LINC02576	0.699188575	6.93E-75	postive
CD22	LINC02576	0.591043653	1.31E-48	postive
CD72	LINC02576	0.672000382	3.17E-67	postive
FCGR2B	LINC02576	0.502333343	1.88E-33	postive
RASGRP3	LINC02576	0.666389076	9.59E-66	postive
PLCG2	LINC02576	0.726826047	1.29E-83	postive
PRKCB	LINC02576	0.707493015	2.12E-77	postive
IGHA1	LINC02576	0.650109101	1.26E-61	postive
IGHA2	LINC02576	0.614654583	1.76E-53	postive
IGHD1-1	LINC02576	0.535705615	1.25E-38	postive
IGHD6-25	LINC02576	0.718608388	6.56E-81	postive
IGHG1	LINC02576	0.664385589	3.18E-65	postive
IGHG2	LINC02576	0.703266089	4.14E-76	postive
IGHG3	LINC02576	0.662742477	8.44E-65	postive
IGHG4	LINC02576	0.615309102	1.27E-53	postive
IGHJ2	LINC02576	0.633232881	1.29E-57	postive
IGHJ3	LINC02576	0.670247008	9.27E-67	postive
IGHV1-2	LINC02576	0.580951963	1.21E-46	postive
IGHV1-24	LINC02576	0.527633086	2.53E-37	postive
IGHV1-46	LINC02576	0.62105516	7.10E-55	postive
IGHV1-69	LINC02576	0.629203513	1.07E-56	postive
IGHV2-70	LINC02576	0.552473584	1.86E-41	postive
IGHV3-11	LINC02576	0.54453058	4.25E-40	postive
IGHV3-15	LINC02576	0.588668761	3.86E-48	postive
IGHV3-21	LINC02576	0.572863862	4.04E-45	postive
IGHV3-23	LINC02576	0.647898744	4.36E-61	postive
IGHV3-30	LINC02576	0.593508033	4.25E-49	postive
IGHV3-33	LINC02576	0.532245097	4.58E-38	postive
IGHV3-35	LINC02576	0.563291261	2.28E-43	postive
IGHV3-49	LINC02576	0.525711569	5.11E-37	postive
IGHV3-53	LINC02576	0.558142366	1.89E-42	postive
IGHV3-66	LINC02576	0.572647889	4.44E-45	postive
IGHV3-7	LINC02576	0.563442993	2.14E-43	postive
IGHV3-74	LINC02576	0.541663652	1.29E-39	postive
IGHV4-28	LINC02576	0.681846584	6.66E-70	postive
IGHV4-31	LINC02576	0.583059056	4.76E-47	postive
IGHV4-34	LINC02576	0.611381155	8.81E-53	postive
IGHV4-39	LINC02576	0.540555042	1.97E-39	postive
IGHV4-59	LINC02576	0.620126069	1.14E-54	postive
IGHV4-61	LINC02576	0.608079976	4.40E-52	postive
IGHV5-51	LINC02576	0.664561964	2.86E-65	postive
IGKC	LINC02576	0.730897313	5.43E-85	postive
IGKJ5	LINC02576	0.802859339	2.15E-114	postive

IGKV1-12	LINC02576	0.516307826	1.50E-35	postive
IGKV1-13	LINC02576	0.546108693	2.30E-40	postive
IGKV1-17	LINC02576	0.50025377	3.79E-33	postive
IGKV1-27	LINC02576	0.531497978	6.05E-38	postive
IGKV1-33	LINC02576	0.511863053	7.15E-35	postive
IGKV1-39	LINC02576	0.638106323	9.48E-59	postive
IGKV1-5	LINC02576	0.668104459	3.41E-66	postive
IGKV1-8	LINC02576	0.602815887	5.49E-51	postive
IGKV1D-39	LINC02576	0.635795886	3.28E-58	postive
IGKV1D-42	LINC02576	0.595507237	1.69E-49	postive
IGKV1D-8	LINC02576	0.632060287	2.39E-57	postive
IGKV3-11	LINC02576	0.699454012	5.78E-75	postive
IGKV3-15	LINC02576	0.668549123	2.60E-66	postive
IGKV3-20	LINC02576	0.700721069	2.42E-75	postive
IGKV3-7	LINC02576	0.623692009	1.85E-55	postive
IGKV3D-11	LINC02576	0.657794143	1.54E-63	postive
IGKV3D-15	LINC02576	0.630193541	6.38E-57	postive
IGKV3D-7	LINC02576	0.559680298	1.01E-42	postive
IGKV4-1	LINC02576	0.608727822	3.21E-52	postive
IGLC2	LINC02576	0.709072158	6.87E-78	postive
IGLC3	LINC02576	0.623813306	1.74E-55	postive
IGLC6	LINC02576	0.575507054	1.30E-45	postive
IGLJ1	LINC02576	0.822182388	1.90E-124	postive
IGLJ2	LINC02576	0.63683341	1.88E-58	postive
IGLJ3	LINC02576	0.701212577	1.72E-75	postive
IGLV1-36	LINC02576	0.549629547	5.75E-41	postive
IGLV1-40	LINC02576	0.661163922	2.14E-64	postive
IGLV1-44	LINC02576	0.685245363	7.49E-71	postive
IGLV1-50	LINC02576	0.525704841	5.12E-37	postive
IGLV1-51	LINC02576	0.585078983	1.94E-47	postive
IGLV2-11	LINC02576	0.548663132	8.42E-41	postive
IGLV2-14	LINC02576	0.634690314	5.92E-58	postive
IGLV2-23	LINC02576	0.57114144	8.43E-45	postive
IGLV3-1	LINC02576	0.589096688	3.18E-48	postive
IGLV3-19	LINC02576	0.509026671	1.91E-34	postive
IGLV3-21	LINC02576	0.51759145	9.51E-36	postive
IGLV3-25	LINC02576	0.60468915	2.25E-51	postive
IGLV4-69	LINC02576	0.57730179	5.96E-46	postive
IGLV6-57	LINC02576	0.526706124	3.55E-37	postive
ADA2	LINC02576	0.51487267	2.49E-35	postive
GMFG	LINC02576	0.619923443	1.26E-54	postive
IL16	LINC02576	0.788602483	1.14E-107	postive
LTB	LINC02576	0.506918979	3.95E-34	postive
IL10RA	LINC02576	0.682393304	4.69E-70	postive
IL12RB1	LINC02576	0.65270656	2.88E-62	postive
TNFRSF13C	LINC02576	0.624878486	1.01E-55	postive
TNFRSF17	LINC02576	0.781762864	1.27E-104	postive
ICAM2	LINC02576	0.530871011	7.63E-38	postive
ITGAL	LINC02576	0.687124396	2.21E-71	postive
LCK	LINC02576	0.622577013	3.27E-55	postive
NCR3	LINC02576	0.719939582	2.43E-81	postive
CD247	LINC02576	0.643250449	5.75E-60	postive
ZAP70	LINC02576	0.661696143	1.57E-64	postive
CD48	LINC02576	0.544466284	4.35E-40	postive
SH2D1A	LINC02576	0.54872601	8.22E-41	postive
CD3D	LINC02576	0.619607232	1.48E-54	postive

CD3E	LINC02576	0.667342726	5.40E-66	postive
PTPRC	LINC02576	0.654281	1.17E-62	postive
ITK	LINC02576	0.692900457	4.88E-73	postive
GRAP2	LINC02576	0.623029695	2.60E-55	postive
CD28	LINC02576	0.6829656	3.25E-70	postive
ICOS	LINC02576	0.572932066	3.93E-45	postive
CTLA4	LINC02576	0.609771074	1.93E-52	postive
TRAC	LINC02576	0.595351417	1.82E-49	postive
TRAV2	LINC02576	0.514895499	2.47E-35	postive
TRAV4	LINC02576	0.511649795	7.70E-35	postive
TRAV8-3	LINC02576	0.637957358	1.03E-58	postive
TRAV8-6	LINC02576	0.573206193	3.49E-45	postive
TRAV9-2	LINC02576	0.613652618	2.88E-53	postive
TRBC1	LINC02576	0.688175543	1.11E-71	postive
TRBC2	LINC02576	0.696630401	3.96E-74	postive
TRBJ2-1	LINC02576	0.627304541	2.88E-56	postive
TRBJ2-2	LINC02576	0.594603786	2.57E-49	postive
TRBJ2-3	LINC02576	0.633832604	9.35E-58	postive
TRBJ2-7	LINC02576	0.671834998	3.51E-67	postive
TRBV2	LINC02576	0.638890482	6.21E-59	postive
TRBV3-1	LINC02576	0.611862279	6.96E-53	postive
TRBV5-1	LINC02576	0.672175498	2.85E-67	postive
TRBV5-4	LINC02576	0.636797198	1.92E-58	postive
TRBV5-6	LINC02576	0.563076212	2.49E-43	postive
TRBV6-1	LINC02576	0.6158194	9.85E-54	postive
TRBV6-5	LINC02576	0.606991299	7.44E-52	postive
TRBV18	LINC02576	0.669795339	1.22E-66	postive
TRBV19	LINC02576	0.566569149	5.81E-44	postive
TRBV20-1	LINC02576	0.619089977	1.92E-54	postive
TRBV28	LINC02576	0.690171103	2.99E-72	postive
TRBV29-1	LINC02576	0.637805284	1.12E-58	postive
AKT1	AL049840.5	0.511590258	7.86E-35	postive
GNRH1	AP000254.2	0.578721974	3.21E-46	postive
TNFRSF25	AP000254.2	0.542260334	1.02E-39	postive
CD4	IRF1-AS1	0.523418965	1.18E-36	postive
CD8A	IRF1-AS1	0.612022681	6.43E-53	postive
CD74	IRF1-AS1	0.70694312	3.12E-77	postive
CTSS	IRF1-AS1	0.546752447	1.78E-40	postive
HLA-B	IRF1-AS1	0.552279277	2.01E-41	postive
HLA-DMA	IRF1-AS1	0.659765629	4.87E-64	postive
HLA-DMB	IRF1-AS1	0.611731434	7.42E-53	postive
HLA-DOA	IRF1-AS1	0.69263929	5.81E-73	postive
HLA-DOB	IRF1-AS1	0.652260728	3.71E-62	postive
HLA-DPA1	IRF1-AS1	0.617369117	4.55E-54	postive
HLA-DPB1	IRF1-AS1	0.647644292	5.02E-61	postive
HLA-DQA1	IRF1-AS1	0.566696039	5.51E-44	postive
HLA-DQA2	IRF1-AS1	0.553332072	1.32E-41	postive
HLA-DRA	IRF1-AS1	0.543645893	5.99E-40	postive
HLA-DRB1	IRF1-AS1	0.526406558	3.96E-37	postive
HLA-E	IRF1-AS1	0.554784662	7.35E-42	postive
HLA-F	IRF1-AS1	0.605472583	1.54E-51	postive
LTA	IRF1-AS1	0.570631199	1.05E-44	postive
CIITA	IRF1-AS1	0.825139588	4.29E-126	postive
RFX5	IRF1-AS1	0.580344063	1.58E-46	postive
TAP2	IRF1-AS1	0.550943937	3.41E-41	postive
IFI30	IRF1-AS1	0.624001803	1.58E-55	postive

ERAP1	IRF1-AS1	0.52228102	1.77E-36	postive
RBP5	IRF1-AS1	0.523377817	1.19E-36	postive
ISG20	IRF1-AS1	0.543540752	6.24E-40	postive
IRF1	IRF1-AS1	0.70953483	4.94E-78	postive
IL15	IRF1-AS1	0.550002153	4.96E-41	postive
DHX58	IRF1-AS1	0.536246026	1.02E-38	postive
CCL5	IRF1-AS1	0.522035757	1.94E-36	postive
CCL4	IRF1-AS1	0.600102599	1.98E-50	postive
CD40LG	IRF1-AS1	0.649268386	2.02E-61	postive
TRIM22	IRF1-AS1	0.730743759	6.13E-85	postive
PDCD1	IRF1-AS1	0.72117523	9.61E-82	postive
FASLG	IRF1-AS1	0.586245142	1.15E-47	postive
ANXA6	IRF1-AS1	0.517659921	9.29E-36	postive
CCR8	IRF1-AS1	0.511345005	8.56E-35	postive
CCR5	IRF1-AS1	0.673594158	1.19E-67	postive
CXCR4	IRF1-AS1	0.632759619	1.65E-57	postive
CXCR6	IRF1-AS1	0.567924568	3.29E-44	postive
CCR4	IRF1-AS1	0.556063705	4.39E-42	postive
CD79A	IRF1-AS1	0.517756465	8.97E-36	postive
BTK	IRF1-AS1	0.6268095	3.72E-56	postive
VAV1	IRF1-AS1	0.620153328	1.12E-54	postive
RAC2	IRF1-AS1	0.519098745	5.56E-36	postive
PIK3R5	IRF1-AS1	0.586115141	1.22E-47	postive
PIK3CD	IRF1-AS1	0.550048993	4.87E-41	postive
INPP5D	IRF1-AS1	0.66998133	1.09E-66	postive
CD72	IRF1-AS1	0.529469263	1.28E-37	postive
PTPN6	IRF1-AS1	0.519440546	4.92E-36	postive
RASGRP3	IRF1-AS1	0.582476385	6.16E-47	postive
PRKCB	IRF1-AS1	0.589664373	2.46E-48	postive
IGLJ1	IRF1-AS1	0.512244382	6.26E-35	postive
SEMA4D	IRF1-AS1	0.522093229	1.90E-36	postive
CMKLR1	IRF1-AS1	0.530334923	9.32E-38	postive
CXCR3	IRF1-AS1	0.698412682	1.18E-74	postive
ADA2	IRF1-AS1	0.534653428	1.86E-38	postive
FLT3LG	IRF1-AS1	0.62832812	1.69E-56	postive
GMFG	IRF1-AS1	0.57983857	1.97E-46	postive
IL16	IRF1-AS1	0.633337151	1.22E-57	postive
IL10RA	IRF1-AS1	0.705121347	1.13E-76	postive
IL12RB1	IRF1-AS1	0.748673265	2.59E-91	postive
IL15RA	IRF1-AS1	0.609514642	2.19E-52	postive
IL2RB	IRF1-AS1	0.538996692	3.58E-39	postive
IL18RAP	IRF1-AS1	0.612339824	5.51E-53	postive
IL21R	IRF1-AS1	0.688882116	6.99E-72	postive
IL2RG	IRF1-AS1	0.713822859	2.23E-79	postive
IL3RA	IRF1-AS1	0.585823191	1.39E-47	postive
NR1H3	IRF1-AS1	0.514026178	3.35E-35	postive
TNFRSF14	IRF1-AS1	0.677560399	1.00E-68	postive
TNFRSF1B	IRF1-AS1	0.628280254	1.73E-56	postive
ICAM2	IRF1-AS1	0.540291372	2.18E-39	postive
ITGAL	IRF1-AS1	0.724159425	1.00E-82	postive
LCK	IRF1-AS1	0.636452108	2.31E-58	postive
NCR3	IRF1-AS1	0.542596449	8.99E-40	postive
CD247	IRF1-AS1	0.734880099	2.31E-86	postive
ZAP70	IRF1-AS1	0.697613111	2.03E-74	postive
LCP2	IRF1-AS1	0.623290104	2.28E-55	postive
HCST	IRF1-AS1	0.557945171	2.05E-42	postive

SH2D1A	IRF1-AS1	0.607055528	7.21E-52	postive
GZMB	IRF1-AS1	0.509732477	1.50E-34	postive
PRF1	IRF1-AS1	0.574122585	2.36E-45	postive
CD3D	IRF1-AS1	0.643888098	4.04E-60	postive
CD3E	IRF1-AS1	0.722789621	2.84E-82	postive
CD3G	IRF1-AS1	0.554524061	8.16E-42	postive
PTPRC	IRF1-AS1	0.685233787	7.54E-71	postive
ITK	IRF1-AS1	0.738347486	1.41E-87	postive
GRAP2	IRF1-AS1	0.717423908	1.58E-80	postive
CD28	IRF1-AS1	0.610614349	1.28E-52	postive
ICOS	IRF1-AS1	0.641043751	1.93E-59	postive
CTLA4	IRF1-AS1	0.667168463	6.00E-66	postive
TRAC	IRF1-AS1	0.697066297	2.95E-74	postive
TRAV2	IRF1-AS1	0.562218473	3.55E-43	postive
TRAV4	IRF1-AS1	0.603888017	3.29E-51	postive
TRAV8-3	IRF1-AS1	0.693713438	2.83E-73	postive
TRAV8-6	IRF1-AS1	0.609471496	2.24E-52	postive
TRAV9-2	IRF1-AS1	0.600781078	1.44E-50	postive
TRAV12-1	IRF1-AS1	0.542434709	9.57E-40	postive
TRAV12-2	IRF1-AS1	0.559879869	9.29E-43	postive
TRAV12-3	IRF1-AS1	0.533287925	3.10E-38	postive
TRAV13-1	IRF1-AS1	0.595186363	1.96E-49	postive
TRAV21	IRF1-AS1	0.511136866	9.21E-35	postive
TRBC1	IRF1-AS1	0.730851446	5.63E-85	postive
TRBC2	IRF1-AS1	0.69929307	6.45E-75	postive
TRBJ2-1	IRF1-AS1	0.66424032	3.47E-65	postive
TRBJ2-2	IRF1-AS1	0.558230385	1.82E-42	postive
TRBJ2-3	IRF1-AS1	0.595698113	1.55E-49	postive
TRBJ2-7	IRF1-AS1	0.685110078	8.17E-71	postive
TRBV2	IRF1-AS1	0.563975337	1.72E-43	postive
TRBV3-1	IRF1-AS1	0.569549079	1.66E-44	postive
TRBV5-1	IRF1-AS1	0.602153385	7.52E-51	postive
TRBV5-4	IRF1-AS1	0.596612301	1.01E-49	postive
TRBV5-6	IRF1-AS1	0.576809035	7.39E-46	postive
TRBV6-1	IRF1-AS1	0.568000918	3.19E-44	postive
TRBV6-5	IRF1-AS1	0.639429041	4.63E-59	postive
TRBV7-9	IRF1-AS1	0.549553641	5.92E-41	postive
TRBV9	IRF1-AS1	0.508311279	2.45E-34	postive
TRBV18	IRF1-AS1	0.627204945	3.03E-56	postive
TRBV19	IRF1-AS1	0.642798587	7.37E-60	postive
TRBV20-1	IRF1-AS1	0.606338691	1.02E-51	postive
TRBV28	IRF1-AS1	0.684925413	9.21E-71	postive
TRBV29-1	IRF1-AS1	0.629032647	1.17E-56	postive
CHGA	RPARP-AS1	0.608581181	3.45E-52	postive
GHRH	RPARP-AS1	0.6069462	7.60E-52	postive
SCG2	RPARP-AS1	0.602002378	8.07E-51	postive
TRH	RPARP-AS1	0.594672923	2.49E-49	postive
NR0B2	RPARP-AS1	0.523255803	1.25E-36	postive
TPT1	ZFAS1	0.605969215	1.22E-51	postive
NR0B2	AL031847.1	0.528224891	2.03E-37	postive
LMBR1L	AC006435.2	0.529153586	1.44E-37	postive
DDX17	AC006435.2	0.572259139	5.24E-45	postive
PLXNB1	AC006435.2	0.601737331	9.15E-51	postive
GNRH1	AC006435.2	0.640686406	2.34E-59	postive
NR2C1	AC006435.2	0.522133009	1.87E-36	postive
TNFRSF25	AC006435.2	0.591327367	1.16E-48	postive

PLCG1	AC006435.2	0.503940552	1.09E-33	postive
DDX17	LINC01089	0.529446233	1.29E-37	postive
GNRH1	LINC01089	0.506367942	4.77E-34	postive
NR2C1	LINC01089	0.503217003	1.40E-33	postive
TNFRSF25	LINC01089	0.532675	3.90E-38	postive
CD79A	AC012181.1	0.501619258	2.40E-33	postive
BTK	AC012181.1	0.52753355	2.62E-37	postive
FCGR2B	AC012181.1	0.509908413	1.41E-34	postive
IGHA1	AC012181.1	0.511970502	6.89E-35	postive
IGHA2	AC012181.1	0.526177489	4.31E-37	postive
IGHD6-25	AC012181.1	0.563674101	1.94E-43	postive
IGHG2	AC012181.1	0.557149361	2.83E-42	postive
IGHG3	AC012181.1	0.538495317	4.34E-39	postive
IGHJ2	AC012181.1	0.500643258	3.33E-33	postive
IGHV1-69	AC012181.1	0.522058226	1.92E-36	postive
IGHV4-28	AC012181.1	0.515272038	2.16E-35	postive
IGHV4-31	AC012181.1	0.50147599	2.52E-33	postive
IGKC	AC012181.1	0.54892381	7.60E-41	postive
IGKJ5	AC012181.1	0.666437905	9.31E-66	postive
IGKV1-5	AC012181.1	0.507177097	3.62E-34	postive
IGKV1D-39	AC012181.1	0.520086912	3.90E-36	postive
IGKV3-15	AC012181.1	0.525246182	6.05E-37	postive
IGKV3-20	AC012181.1	0.536512053	9.21E-39	postive
IGKV3-7	AC012181.1	0.501187868	2.77E-33	postive
IGKV3D-11	AC012181.1	0.52548731	5.54E-37	postive
IGLC2	AC012181.1	0.541831129	1.21E-39	postive
IGLJ1	AC012181.1	0.515619714	1.91E-35	postive
IGLJ3	AC012181.1	0.520807546	3.02E-36	postive
IGLV1-40	AC012181.1	0.54435936	4.54E-40	postive
IGLV1-44	AC012181.1	0.54112638	1.58E-39	postive
IGLV2-14	AC012181.1	0.522771339	1.49E-36	postive
IL16	AC012181.1	0.503126547	1.44E-33	postive
IL10RA	AC012181.1	0.568296721	2.81E-44	postive
TNFRSF17	AC012181.1	0.563616462	1.99E-43	postive
ITK	AC012181.1	0.545688306	2.71E-40	postive
CTLA4	AC012181.1	0.540973163	1.68E-39	postive
PLXNB1	AC027796.4	0.538257051	4.75E-39	postive
GNRH1	AC027796.4	0.512713236	5.31E-35	postive
RELA	AP000759.1	0.512408604	5.91E-35	postive
DDX17	AC127024.4	0.515276647	2.16E-35	postive
GNRH1	AC127024.4	0.509583382	1.58E-34	postive
GNRH1	AP006621.2	0.536007827	1.11E-38	postive
GNRH1	AGBL5-IT1	0.59254421	6.62E-49	postive
TNFRSF25	AGBL5-IT1	0.51272011	5.30E-35	postive
NR2C1	AC079907.1	0.563654549	1.96E-43	postive
CD8B	AC104958.2	0.755001084	1.08E-93	postive
DEFA5	AC104958.2	0.868138855	3.13E-154	postive
PENK	AC104958.2	0.661034858	2.31E-64	postive
CST4	AC104958.2	0.645060263	2.12E-60	postive
BMP4	AC104958.2	0.557680644	2.28E-42	postive
DKK1	AC104958.2	0.575985218	1.06E-45	postive
FGF3	AC104958.2	0.648732747	2.73E-61	postive
TAC1	AC104958.2	0.557111776	2.87E-42	postive
FGFRL1	AC104958.2	0.520557218	3.30E-36	postive
PTPN6	AC005840.4	0.504330418	9.57E-34	postive
GNRH1	AC005840.4	0.5230375	1.35E-36	postive

PROC	AL135999.3	0.652135142	3.99E-62	postive
ARRB1	AL135999.3	0.660975332	2.40E-64	postive
C5	AL135999.3	0.681014102	1.13E-69	postive
CALCA	AL135999.3	0.587406512	6.83E-48	postive
THPO	AL135999.3	0.601439706	1.05E-50	postive
RELB	AC108868.1	0.539215035	3.29E-39	postive
NFKB1	AC108868.1	0.514116906	3.25E-35	postive
IKBKB	AC108868.1	0.709396151	5.45E-78	postive
IGHD1-26	AC108868.1	0.511702457	7.56E-35	postive
CSF2RB	AC108868.1	0.544298869	4.65E-40	postive
IFI30	SH3BP5-AS1	0.554072582	9.79E-42	postive
LMBR1L	SH3BP5-AS1	0.576549039	8.27E-46	postive
DDX17	SH3BP5-AS1	0.584751198	2.24E-47	postive
CD40LG	SH3BP5-AS1	0.538381087	4.53E-39	postive
INPP5D	SH3BP5-AS1	0.51165695	7.68E-35	postive
IGLJ1	SH3BP5-AS1	0.502738028	1.64E-33	postive
GNRH1	SH3BP5-AS1	0.637570496	1.27E-58	postive
GIPR	SH3BP5-AS1	0.533975488	2.40E-38	postive
NR2C2	SH3BP5-AS1	0.636612142	2.12E-58	postive
TNFRSF13C	SH3BP5-AS1	0.506487441	4.58E-34	postive
TNFRSF14	SH3BP5-AS1	0.541600462	1.32E-39	postive
TNFRSF25	SH3BP5-AS1	0.509994341	1.37E-34	postive
ITGAL	SH3BP5-AS1	0.510303872	1.23E-34	postive
ZAP70	SH3BP5-AS1	0.568992529	2.10E-44	postive
ITK	SH3BP5-AS1	0.520364749	3.53E-36	postive
TRBJ2-7	SH3BP5-AS1	0.564656208	1.29E-43	postive
B2M	LINC01943	0.573658322	2.88E-45	postive
CD8A	LINC01943	0.658207225	1.21E-63	postive
CD74	LINC01943	0.533822531	2.54E-38	postive
CTSS	LINC01943	0.571006246	8.93E-45	postive
FCER1G	LINC01943	0.706637175	3.88E-77	postive
HLA-DMA	LINC01943	0.522954301	1.39E-36	postive
HLA-DMB	LINC01943	0.659003982	7.61E-64	postive
HLA-DPA1	LINC01943	0.545386337	3.04E-40	postive
HLA-DPB1	LINC01943	0.536572087	9.00E-39	postive
HLA-DQA1	LINC01943	0.512405789	5.91E-35	postive
HLA-DRA	LINC01943	0.624120726	1.49E-55	postive
HLA-DRB1	LINC01943	0.564092139	1.63E-43	postive
IFNG	LINC01943	0.643995813	3.81E-60	postive
CXCL9	LINC01943	0.536822959	8.18E-39	postive
CCL5	LINC01943	0.512380219	5.97E-35	postive
TLR8	LINC01943	0.571377821	7.63E-45	postive
APOM	LINC01943	0.553378234	1.29E-41	postive
PLA2G2A	LINC01943	0.56110717	5.61E-43	postive
TPT1	LINC01943	0.506728167	4.22E-34	postive
CCL4	LINC01943	0.662031034	1.29E-64	postive
FASLG	LINC01943	0.52872706	1.69E-37	postive
APOH	LINC01943	0.528797312	1.64E-37	postive
CCL3	LINC01943	0.598769658	3.70E-50	postive
CCR5	LINC01943	0.562774536	2.82E-43	postive
XCL2	LINC01943	0.710493924	2.48E-78	postive
CXCR6	LINC01943	0.736494911	6.31E-87	postive
CD86	LINC01943	0.534398343	2.04E-38	postive
FCGR2B	LINC01943	0.523940726	9.73E-37	postive
CXCR3	LINC01943	0.573003091	3.81E-45	postive
PLXNC1	LINC01943	0.527028688	3.15E-37	postive

AGT	LINC01943	0.549142345	6.97E-41	postive
GMFG	LINC01943	0.735726497	1.17E-86	postive
TNFSF13B	LINC01943	0.614966893	1.50E-53	postive
IL10RA	LINC01943	0.502264829	1.93E-33	postive
IL12RB1	LINC01943	0.555010537	6.71E-42	postive
IL18RAP	LINC01943	0.602483122	6.43E-51	postive
IL2RG	LINC01943	0.502942922	1.53E-33	postive
NR1H3	LINC01943	0.638473593	7.78E-59	postive
TYROBP	LINC01943	0.594198345	3.10E-49	postive
LCK	LINC01943	0.549793705	5.39E-41	postive
CD247	LINC01943	0.536562717	9.03E-39	postive
LCP2	LINC01943	0.553780676	1.10E-41	postive
HCST	LINC01943	0.701234597	1.69E-75	postive
CD48	LINC01943	0.784447382	8.34E-106	postive
CD244	LINC01943	0.612055012	6.33E-53	postive
SH2D1A	LINC01943	0.67713602	1.31E-68	postive
GZMB	LINC01943	0.649020129	2.32E-61	postive
PRF1	LINC01943	0.504077944	1.04E-33	postive
CD3D	LINC01943	0.793700966	5.17E-110	postive
CD3E	LINC01943	0.592310307	7.37E-49	postive
CD3G	LINC01943	0.532280909	4.52E-38	postive
PTPRC	LINC01943	0.521614014	2.26E-36	postive
ICOS	LINC01943	0.613943218	2.50E-53	postive
CTLA4	LINC01943	0.554921257	6.96E-42	postive
TRAC	LINC01943	0.612982467	4.01E-53	postive
TRAV2	LINC01943	0.590388216	1.77E-48	postive
TRAV4	LINC01943	0.677391211	1.12E-68	postive
TRAV8-3	LINC01943	0.508593624	2.22E-34	postive
TRAV8-6	LINC01943	0.548559073	8.77E-41	postive
TRAV9-2	LINC01943	0.534087837	2.30E-38	postive
TRAV12-1	LINC01943	0.501933876	2.16E-33	postive
TRAV12-2	LINC01943	0.636458795	2.30E-58	postive
TRAV13-1	LINC01943	0.527576722	2.58E-37	postive
TRBC1	LINC01943	0.511215657	8.96E-35	postive
TRBC2	LINC01943	0.645220318	1.94E-60	postive
TRBV2	LINC01943	0.506309368	4.87E-34	postive
TRBV3-1	LINC01943	0.517451971	1.00E-35	postive
TRBV5-1	LINC01943	0.522530577	1.62E-36	postive
TRBV6-5	LINC01943	0.541973814	1.14E-39	postive
TRBV7-9	LINC01943	0.595308481	1.85E-49	postive
TRBV9	LINC01943	0.72162424	6.85E-82	postive
TRBV12-4	LINC01943	0.688165079	1.12E-71	postive
TRBV20-1	LINC01943	0.583293062	4.29E-47	postive
TRBV28	LINC01943	0.56657123	5.81E-44	postive
TRDC	LINC01943	0.527994924	2.21E-37	postive
LMBR1L	AC008760.1	0.515018329	2.37E-35	postive
GNRH1	AC008760.1	0.640690664	2.33E-59	postive
GIPR	AC008760.1	0.525067613	6.46E-37	postive
TNFRSF25	AC008760.1	0.553685293	1.14E-41	postive
CD4	AC138207.5	0.727956819	5.40E-84	postive
CD74	AC138207.5	0.673323957	1.40E-67	postive
CTSS	AC138207.5	0.660842503	2.59E-64	postive
FCER1G	AC138207.5	0.681028875	1.12E-69	postive
FCGRT	AC138207.5	0.509816842	1.46E-34	postive
HLA-DMA	AC138207.5	0.686504231	3.31E-71	postive
HLA-DMB	AC138207.5	0.657369065	1.97E-63	postive

HLA-DOA	AC138207.5	0.587481888	6.60E-48	postive
HLA-DPA1	AC138207.5	0.648464578	3.17E-61	postive
HLA-DPB1	AC138207.5	0.681352158	9.13E-70	postive
HLA-DQA1	AC138207.5	0.549416171	6.26E-41	postive
HLA-DQB1	AC138207.5	0.51033532	1.22E-34	postive
HLA-DRA	AC138207.5	0.563186674	2.38E-43	postive
HLA-DRB1	AC138207.5	0.578629963	3.34E-46	postive
LGMN	AC138207.5	0.607508907	5.79E-52	postive
IFI30	AC138207.5	0.536935707	7.84E-39	postive
CYBB	AC138207.5	0.692008243	8.84E-73	postive
TLR7	AC138207.5	0.706523543	4.20E-77	postive
TLR8	AC138207.5	0.61959155	1.49E-54	postive
MSR1	AC138207.5	0.567232338	4.40E-44	postive
CCL4	AC138207.5	0.536335695	9.84E-39	postive
CD14	AC138207.5	0.648434465	3.23E-61	postive
TRIM22	AC138207.5	0.513358727	4.24E-35	postive
CCR5	AC138207.5	0.66786207	3.95E-66	postive
CCR1	AC138207.5	0.680396085	1.68E-69	postive
CXCR6	AC138207.5	0.541028911	1.64E-39	postive
CCR4	AC138207.5	0.511323086	8.63E-35	postive
FGR	AC138207.5	0.627230713	2.99E-56	postive
CD86	AC138207.5	0.636625321	2.10E-58	postive
RNASE2	AC138207.5	0.509966922	1.38E-34	postive
BTK	AC138207.5	0.636105082	2.78E-58	postive
VAV1	AC138207.5	0.510674994	1.08E-34	postive
PIK3R5	AC138207.5	0.538397022	4.50E-39	postive
LILRB3	AC138207.5	0.642835094	7.22E-60	postive
FCGR2B	AC138207.5	0.574216906	2.26E-45	postive
RASGRP3	AC138207.5	0.527159432	3.01E-37	postive
C5AR1	AC138207.5	0.573612651	2.93E-45	postive
CCRL2	AC138207.5	0.648186572	3.71E-61	postive
CMKLR1	AC138207.5	0.70209372	9.35E-76	postive
CXCR3	AC138207.5	0.57268187	4.37E-45	postive
PLXNC1	AC138207.5	0.703480382	3.56E-76	postive
ADA2	AC138207.5	0.586176039	1.19E-47	postive
CSF1	AC138207.5	0.507916249	2.81E-34	postive
GMFG	AC138207.5	0.598810454	3.63E-50	postive
IL16	AC138207.5	0.516088915	1.62E-35	postive
TNFSF12	AC138207.5	0.68715391	2.17E-71	postive
TNFSF13B	AC138207.5	0.604017617	3.10E-51	postive
ACVRL1	AC138207.5	0.527355092	2.80E-37	postive
C3AR1	AC138207.5	0.65054005	9.86E-62	postive
CSF1R	AC138207.5	0.697674633	1.95E-74	postive
CSF2RA	AC138207.5	0.518352459	7.26E-36	postive
ENG	AC138207.5	0.628554197	1.50E-56	postive
IL10RA	AC138207.5	0.669395683	1.56E-66	postive
IL12RB1	AC138207.5	0.562766317	2.83E-43	postive
IL3RA	AC138207.5	0.524355755	8.37E-37	postive
NR1H3	AC138207.5	0.575802472	1.14E-45	postive
TIE1	AC138207.5	0.535533525	1.33E-38	postive
TNFRSF1B	AC138207.5	0.64732933	5.99E-61	postive
ITGAL	AC138207.5	0.557049097	2.94E-42	postive
ITGB2	AC138207.5	0.661584596	1.67E-64	postive
TYROBP	AC138207.5	0.675568629	3.49E-68	postive
FCGR3A	AC138207.5	0.617789033	3.68E-54	postive
CD247	AC138207.5	0.528539564	1.81E-37	postive

LCP2	AC138207.5	0.689716795	4.03E-72	postive
HCST	AC138207.5	0.642267854	9.86E-60	postive
CD3D	AC138207.5	0.501291954	2.68E-33	postive
CD3E	AC138207.5	0.535582171	1.31E-38	postive
PTPRC	AC138207.5	0.617477944	4.30E-54	postive
ITK	AC138207.5	0.512882276	5.01E-35	postive
GRAP2	AC138207.5	0.517281064	1.06E-35	postive
CD28	AC138207.5	0.522932118	1.40E-36	postive
ICOS	AC138207.5	0.519915383	4.15E-36	postive
TRAC	AC138207.5	0.506989255	3.86E-34	postive
TRAV8-6	AC138207.5	0.51089958	1.00E-34	postive
TRBC1	AC138207.5	0.514759971	2.59E-35	postive
TRBC2	AC138207.5	0.50979727	1.47E-34	postive
TRBV20-1	AC138207.5	0.503828414	1.13E-33	postive
TRBV28	AC138207.5	0.505425287	6.59E-34	postive
PLXNA3	AC245140.2	0.536554173	9.06E-39	postive
CD4	SMIM25	0.631955103	2.53E-57	postive
CD74	SMIM25	0.567236715	4.39E-44	postive
CTSS	SMIM25	0.666282008	1.02E-65	postive
FCER1G	SMIM25	0.617181135	4.99E-54	postive
FCGRT	SMIM25	0.530508903	8.73E-38	postive
HLA-DMA	SMIM25	0.613698167	2.82E-53	postive
HLA-DMB	SMIM25	0.567124801	4.61E-44	postive
HLA-DOA	SMIM25	0.500752733	3.21E-33	postive
HLA-DPA1	SMIM25	0.560921594	6.06E-43	postive
HLA-DPB1	SMIM25	0.627685731	2.36E-56	postive
HLA-DRA	SMIM25	0.536438492	9.47E-39	postive
HLA-DRB1	SMIM25	0.562118997	3.70E-43	postive
HLA-DRB5	SMIM25	0.506275592	4.93E-34	postive
LGMN	SMIM25	0.544288109	4.67E-40	postive
CXCL16	SMIM25	0.610468046	1.38E-52	postive
CXCL2	SMIM25	0.607113051	7.01E-52	postive
SFTPD	SMIM25	0.519619676	4.62E-36	postive
COLEC12	SMIM25	0.540453517	2.05E-39	postive
A2M	SMIM25	0.634867455	5.39E-58	postive
SFTPA1	SMIM25	0.53403907	2.34E-38	postive
CYBB	SMIM25	0.644476733	2.92E-60	postive
TLR7	SMIM25	0.553952941	1.03E-41	postive
TLR8	SMIM25	0.610251405	1.53E-52	postive
MSR1	SMIM25	0.750293811	6.48E-92	postive
SLC11A1	SMIM25	0.802282237	4.12E-114	postive
MARCO	SMIM25	0.739196767	7.05E-88	postive
CD14	SMIM25	0.550664219	3.82E-41	postive
AQP9	SMIM25	0.736081494	8.80E-87	postive
AGER	SMIM25	0.631660779	2.95E-57	postive
CCL18	SMIM25	0.518776125	6.24E-36	postive
CCL23	SMIM25	0.674252836	7.90E-68	postive
CCR1	SMIM25	0.680446734	1.62E-69	postive
FGR	SMIM25	0.76958236	1.83E-99	postive
CD86	SMIM25	0.582585685	5.87E-47	postive
BTK	SMIM25	0.577485345	5.51E-46	postive
PIK3R5	SMIM25	0.540658757	1.90E-39	postive
LILRB3	SMIM25	0.799836044	6.36E-113	postive
FCGR2B	SMIM25	0.503500904	1.27E-33	postive
C5AR1	SMIM25	0.777476722	9.03E-103	postive
CCRL2	SMIM25	0.812907021	1.78E-119	postive

CMKLR1	SMIM25	0.567396545	4.11E-44	postive
FPR1	SMIM25	0.617968343	3.37E-54	postive
FPR2	SMIM25	0.566197243	6.79E-44	postive
PLXNC1	SMIM25	0.52838234	1.92E-37	postive
PTAFR	SMIM25	0.51678457	1.27E-35	postive
AGRP	SMIM25	0.653518691	1.81E-62	postive
ADA2	SMIM25	0.518769457	6.25E-36	postive
VEGFD	SMIM25	0.612769394	4.46E-53	postive
GMFG	SMIM25	0.554631726	7.82E-42	postive
RETN	SMIM25	0.715649834	5.85E-80	postive
TNFSF12	SMIM25	0.607591164	5.57E-52	postive
TNFSF13	SMIM25	0.657560463	1.76E-63	postive
ACVRL1	SMIM25	0.668231987	3.15E-66	postive
C3AR1	SMIM25	0.632434887	1.96E-57	postive
CSF1R	SMIM25	0.55278941	1.64E-41	postive
CSF3R	SMIM25	0.585619484	1.52E-47	postive
ENG	SMIM25	0.599759271	2.33E-50	postive
IL10RA	SMIM25	0.533633243	2.72E-38	postive
IL3RA	SMIM25	0.628363846	1.66E-56	postive
NPR1	SMIM25	0.521513172	2.34E-36	postive
S1PR1	SMIM25	0.517020303	1.17E-35	postive
TEK	SMIM25	0.535359312	1.42E-38	postive
TGFBR2	SMIM25	0.537233871	7.00E-39	postive
TIE1	SMIM25	0.549295508	6.56E-41	postive
TNFRSF1B	SMIM25	0.532445294	4.25E-38	postive
ITGB2	SMIM25	0.6080698	4.42E-52	postive
TYROBP	SMIM25	0.752275083	1.17E-92	postive
FCGR3A	SMIM25	0.575960498	1.07E-45	postive
LCP2	SMIM25	0.592664912	6.26E-49	postive
HCST	SMIM25	0.528602626	1.77E-37	postive
IFI30	AC116914.2	0.570543708	1.09E-44	postive
DDX17	AC116914.2	0.507193709	3.60E-34	postive
CD40LG	AC116914.2	0.520631835	3.21E-36	postive
IRF9	AC116914.2	0.512953816	4.88E-35	postive
PDCD1	AC116914.2	0.511828709	7.23E-35	postive
CD19	AC116914.2	0.500334262	3.69E-33	postive
CD72	AC116914.2	0.528680905	1.72E-37	postive
IGLJ1	AC116914.2	0.532742856	3.80E-38	postive
GNRH1	AC116914.2	0.597552387	6.54E-50	postive
TNFRSF14	AC116914.2	0.544640726	4.07E-40	postive
ITGAL	AC116914.2	0.526583642	3.71E-37	postive
ZAP70	AC116914.2	0.612203833	5.89E-53	postive
ITK	AC116914.2	0.527674651	2.49E-37	postive
TRBC1	AC116914.2	0.513937025	3.46E-35	postive
TRBJ2-7	AC116914.2	0.589147833	3.11E-48	postive
NPPC	LINC02351	0.509789011	1.47E-34	postive
HLA-DOB	AC090948.3	0.587321903	7.09E-48	postive
LTA	AC090948.3	0.625559097	7.10E-56	postive
CIITA	AC090948.3	0.548918869	7.61E-41	postive
IFI30	AC090948.3	0.534694727	1.83E-38	postive
RBP5	AC090948.3	0.522313107	1.75E-36	postive
CETP	AC090948.3	0.613845076	2.62E-53	postive
CD40LG	AC090948.3	0.688004484	1.24E-71	postive
PDCD1	AC090948.3	0.639138432	5.43E-59	postive
CCL21	AC090948.3	0.609649099	2.05E-52	postive
CD79A	AC090948.3	0.595451715	1.74E-49	postive

CD79B	AC090948.3	0.665868384	1.31E-65	postive
BTK	AC090948.3	0.563701687	1.92E-43	postive
CD19	AC090948.3	0.694183975	2.07E-73	postive
CR2	AC090948.3	0.619037659	1.97E-54	postive
PIK3CD	AC090948.3	0.50166724	2.36E-33	postive
INPP5D	AC090948.3	0.621573873	5.46E-55	postive
CD22	AC090948.3	0.659140297	7.03E-64	postive
CD72	AC090948.3	0.680422952	1.65E-69	postive
PTPN6	AC090948.3	0.551076602	3.24E-41	postive
RASGRP3	AC090948.3	0.589573226	2.57E-48	postive
PLCG2	AC090948.3	0.555848148	4.79E-42	postive
PRKCB	AC090948.3	0.579860087	1.95E-46	postive
IGLJ1	AC090948.3	0.668862859	2.15E-66	postive
FLT3LG	AC090948.3	0.547544034	1.31E-40	postive
GNRH1	AC090948.3	0.538818545	3.83E-39	postive
IL16	AC090948.3	0.620459285	9.60E-55	postive
LTB	AC090948.3	0.58180791	8.28E-47	postive
IL10RA	AC090948.3	0.531269713	6.58E-38	postive
IL12RB1	AC090948.3	0.568656118	2.42E-44	postive
TNFRSF13C	AC090948.3	0.698947827	8.17E-75	postive
TNFRSF14	AC090948.3	0.555015004	6.70E-42	postive
ITGAL	AC090948.3	0.674283749	7.75E-68	postive
LCK	AC090948.3	0.585605206	1.53E-47	postive
NCR3	AC090948.3	0.627670303	2.38E-56	postive
CD247	AC090948.3	0.610427634	1.40E-52	postive
ZAP70	AC090948.3	0.774930378	1.09E-101	postive
CD3E	AC090948.3	0.604302916	2.70E-51	postive
PTPRC	AC090948.3	0.532179357	4.69E-38	postive
ITK	AC090948.3	0.618621438	2.43E-54	postive
GRAP2	AC090948.3	0.609801626	1.91E-52	postive
CD28	AC090948.3	0.533625712	2.73E-38	postive
CTLA4	AC090948.3	0.582008056	7.58E-47	postive
TRAC	AC090948.3	0.502445703	1.81E-33	postive
TRAV8-3	AC090948.3	0.579873074	1.94E-46	postive
TRBC1	AC090948.3	0.678087676	7.21E-69	postive
TRBC2	AC090948.3	0.621119709	6.87E-55	postive
TRBJ2-1	AC090948.3	0.596487878	1.07E-49	postive
TRBJ2-2	AC090948.3	0.658519757	1.01E-63	postive
TRBJ2-3	AC090948.3	0.630825742	4.58E-57	postive
TRBJ2-7	AC090948.3	0.743769222	1.62E-89	postive
TRBV2	AC090948.3	0.561268079	5.25E-43	postive
TRBV3-1	AC090948.3	0.509717433	1.51E-34	postive
TRBV5-1	AC090948.3	0.543327835	6.77E-40	postive
TRBV18	AC090948.3	0.551625127	2.60E-41	postive
TRBV28	AC090948.3	0.660936518	2.45E-64	postive
PSMD11	AC125257.1	0.584609435	2.39E-47	postive
PSME3	AC125257.1	0.537355902	6.69E-39	postive
NR2C1	NFYC-AS1	0.53019701	9.80E-38	postive
NR2C2	NFYC-AS1	0.530651018	8.28E-38	postive
PLCG1	NFYC-AS1	0.524794894	7.13E-37	postive
IRF9	AL157871.2	0.552159106	2.10E-41	postive
DDX17	STAG3L5P-PVRIG2P-I	0.524125465	9.10E-37	postive
PLXNB1	STAG3L5P-PVRIG2P-I	0.561218819	5.36E-43	postive
GNRH1	STAG3L5P-PVRIG2P-I	0.607058754	7.20E-52	postive
TNFRSF25	STAG3L5P-PVRIG2P-I	0.539031283	3.53E-39	postive
SHC2	AC007383.1	0.501056946	2.90E-33	postive

IRF3	PTOV1-AS1	0.606774105	8.26E-52	postive
PLCG1	AC012360.3	0.577173715	6.30E-46	postive
SFTPD	AC092071.1	0.563173021	2.39E-43	postive
AGTR2	AC092071.1	0.605507041	1.52E-51	postive
NPR1	AC092071.1	0.510815087	1.03E-34	postive
AGER	AC093110.1	0.62548604	7.37E-56	postive
VEGFD	AC093110.1	0.53698645	7.69E-39	postive
NPR1	AC093110.1	0.502423686	1.83E-33	postive
BPIFB1	ACBD3-AS1	0.577102694	6.50E-46	postive
BPIFA1	ACBD3-AS1	0.523535595	1.13E-36	postive
SCGB3A1	ACBD3-AS1	0.527887841	2.30E-37	postive
IRF3	AC245884.8	0.556802623	3.25E-42	postive
GNRH1	AC245884.8	0.627645991	2.41E-56	postive
TNFRSF25	AC245884.8	0.545429581	2.99E-40	postive
ALB	AC139887.2	0.569766513	1.51E-44	postive
FGFR3	AC139887.2	0.784332421	9.38E-106	postive
HLA-DOB	DTNB-AS1	0.630228579	6.26E-57	postive
ISG20	DTNB-AS1	0.545573964	2.83E-40	postive
CD79A	DTNB-AS1	0.630506102	5.42E-57	postive
BTK	DTNB-AS1	0.516582099	1.36E-35	postive
FCGR2B	DTNB-AS1	0.544310905	4.63E-40	postive
IGHA1	DTNB-AS1	0.697457911	2.26E-74	postive
IGHA2	DTNB-AS1	0.672565459	2.24E-67	postive
IGHD	DTNB-AS1	0.53252263	4.13E-38	postive
IGHD6-25	DTNB-AS1	0.669570751	1.40E-66	postive
IGHG1	DTNB-AS1	0.705865421	6.69E-77	postive
IGHG2	DTNB-AS1	0.757085268	1.71E-94	postive
IGHG3	DTNB-AS1	0.700370418	3.08E-75	postive
IGHG4	DTNB-AS1	0.606641251	8.81E-52	postive
IGHJ2	DTNB-AS1	0.653056195	2.36E-62	postive
IGHJ3	DTNB-AS1	0.709424432	5.34E-78	postive
IGHV1-2	DTNB-AS1	0.58280007	5.34E-47	postive
IGHV1-24	DTNB-AS1	0.581605752	9.06E-47	postive
IGHV1-46	DTNB-AS1	0.633216406	1.30E-57	postive
IGHV1-69	DTNB-AS1	0.65797825	1.38E-63	postive
IGHV2-5	DTNB-AS1	0.533495784	2.87E-38	postive
IGHV2-70	DTNB-AS1	0.614946857	1.52E-53	postive
IGHV3-11	DTNB-AS1	0.548140757	1.03E-40	postive
IGHV3-15	DTNB-AS1	0.57025408	1.23E-44	postive
IGHV3-20	DTNB-AS1	0.504614648	8.69E-34	postive
IGHV3-21	DTNB-AS1	0.582291484	6.69E-47	postive
IGHV3-23	DTNB-AS1	0.631852426	2.67E-57	postive
IGHV3-30	DTNB-AS1	0.602963893	5.12E-51	postive
IGHV3-33	DTNB-AS1	0.635047683	4.90E-58	postive
IGHV3-35	DTNB-AS1	0.583918627	3.25E-47	postive
IGHV3-43	DTNB-AS1	0.574370408	2.12E-45	postive
IGHV3-53	DTNB-AS1	0.553774072	1.10E-41	postive
IGHV3-66	DTNB-AS1	0.573097632	3.66E-45	postive
IGHV3-7	DTNB-AS1	0.541340515	1.46E-39	postive
IGHV3-73	DTNB-AS1	0.508483519	2.31E-34	postive
IGHV3-74	DTNB-AS1	0.570695142	1.02E-44	postive
IGHV4-28	DTNB-AS1	0.702414256	7.48E-76	postive
IGHV4-31	DTNB-AS1	0.584629147	2.37E-47	postive
IGHV4-34	DTNB-AS1	0.612531924	5.01E-53	postive
IGHV4-39	DTNB-AS1	0.539628957	2.81E-39	postive
IGHV4-59	DTNB-AS1	0.682317313	4.93E-70	postive

IGHV4-61	DTNB-AS1	0.595935204	1.39E-49	postive
IGHV5-51	DTNB-AS1	0.702936497	5.20E-76	postive
IGKC	DTNB-AS1	0.851828905	1.63E-142	postive
IGKJ5	DTNB-AS1	0.806985688	1.91E-116	postive
IGKV1-12	DTNB-AS1	0.536431732	9.49E-39	postive
IGKV1-13	DTNB-AS1	0.629763714	7.99E-57	postive
IGKV1-16	DTNB-AS1	0.505232831	7.04E-34	postive
IGKV1-17	DTNB-AS1	0.627449471	2.67E-56	postive
IGKV1-27	DTNB-AS1	0.57847889	3.57E-46	postive
IGKV1-33	DTNB-AS1	0.665299065	1.84E-65	postive
IGKV1-39	DTNB-AS1	0.692996886	4.57E-73	postive
IGKV1-5	DTNB-AS1	0.722954848	2.50E-82	postive
IGKV1-8	DTNB-AS1	0.64617345	1.14E-60	postive
IGKV1-9	DTNB-AS1	0.535926345	1.15E-38	postive
IGKV1D-12	DTNB-AS1	0.510661695	1.09E-34	postive
IGKV1D-33	DTNB-AS1	0.533457462	2.91E-38	postive
IGKV1D-39	DTNB-AS1	0.708582472	9.75E-78	postive
IGKV1D-42	DTNB-AS1	0.716921782	2.29E-80	postive
IGKV1D-8	DTNB-AS1	0.753375101	4.49E-93	postive
IGKV2-28	DTNB-AS1	0.548656241	8.44E-41	postive
IGKV3-11	DTNB-AS1	0.76357521	4.90E-97	postive
IGKV3-15	DTNB-AS1	0.793786099	4.72E-110	postive
IGKV3-20	DTNB-AS1	0.774327011	1.95E-101	postive
IGKV3-7	DTNB-AS1	0.682204408	5.30E-70	postive
IGKV3D-11	DTNB-AS1	0.748517207	2.96E-91	postive
IGKV3D-15	DTNB-AS1	0.692954311	4.71E-73	postive
IGKV3D-20	DTNB-AS1	0.619255227	1.76E-54	postive
IGKV3D-7	DTNB-AS1	0.590905723	1.40E-48	postive
IGKV4-1	DTNB-AS1	0.707830329	1.67E-77	postive
IGKV6-21	DTNB-AS1	0.504964194	7.71E-34	postive
IGLC2	DTNB-AS1	0.827899469	1.17E-127	postive
IGLC3	DTNB-AS1	0.770789623	5.82E-100	postive
IGLC6	DTNB-AS1	0.695599757	7.96E-74	postive
IGLJ1	DTNB-AS1	0.526131771	4.38E-37	postive
IGLJ2	DTNB-AS1	0.728964168	2.47E-84	postive
IGLJ3	DTNB-AS1	0.7489655	2.02E-91	postive
IGLV1-36	DTNB-AS1	0.576134932	9.89E-46	postive
IGLV1-40	DTNB-AS1	0.72403028	1.10E-82	postive
IGLV1-44	DTNB-AS1	0.667014964	6.58E-66	postive
IGLV1-50	DTNB-AS1	0.587818896	5.67E-48	postive
IGLV1-51	DTNB-AS1	0.655779381	4.95E-63	postive
IGLV2-11	DTNB-AS1	0.579025751	2.81E-46	postive
IGLV2-14	DTNB-AS1	0.646910875	7.57E-61	postive
IGLV2-23	DTNB-AS1	0.581338834	1.02E-46	postive
IGLV2-33	DTNB-AS1	0.620579945	9.03E-55	postive
IGLV2-8	DTNB-AS1	0.501026602	2.93E-33	postive
IGLV3-1	DTNB-AS1	0.701176747	1.76E-75	postive
IGLV3-10	DTNB-AS1	0.583922878	3.24E-47	postive
IGLV3-16	DTNB-AS1	0.510147223	1.30E-34	postive
IGLV3-19	DTNB-AS1	0.574738875	1.81E-45	postive
IGLV3-21	DTNB-AS1	0.662003484	1.31E-64	postive
IGLV3-25	DTNB-AS1	0.68455099	1.17E-70	postive
IGLV3-9	DTNB-AS1	0.538451958	4.41E-39	postive
IGLV4-69	DTNB-AS1	0.599131768	3.12E-50	postive
IGLV5-48	DTNB-AS1	0.535737764	1.23E-38	postive
IGLV6-57	DTNB-AS1	0.582454759	6.22E-47	postive

GMFG	DTNB-AS1	0.568228883	2.90E-44	postive
IL16	DTNB-AS1	0.540697009	1.87E-39	postive
IL10RA	DTNB-AS1	0.510139469	1.30E-34	postive
TNFRSF17	DTNB-AS1	0.905553821	2.49E-188	postive
ICAM2	DTNB-AS1	0.50261765	1.71E-33	postive
CD48	DTNB-AS1	0.506975368	3.88E-34	postive
CD3D	DTNB-AS1	0.523448273	1.16E-36	postive
TRBV5-4	DTNB-AS1	0.502628386	1.70E-33	postive
TRBV6-1	DTNB-AS1	0.550165153	4.65E-41	postive
TRBV20-1	DTNB-AS1	0.500071816	4.03E-33	postive
CALCA	HOXB-AS3	0.616986224	5.50E-54	postive
THPO	HOXB-AS3	0.5123532	6.02E-35	postive
HLA-DOB	AC012236.1	0.719596568	3.14E-81	postive
ISG20	AC012236.1	0.611909161	6.80E-53	postive
CD40LG	AC012236.1	0.613597886	2.96E-53	postive
PDCD1	AC012236.1	0.558000083	2.00E-42	postive
ANXA6	AC012236.1	0.500034854	4.08E-33	postive
CXCR4	AC012236.1	0.582342993	6.54E-47	postive
CCR4	AC012236.1	0.547106125	1.55E-40	postive
CD79A	AC012236.1	0.766083446	4.85E-98	postive
CD79B	AC012236.1	0.501842282	2.22E-33	postive
BTK	AC012236.1	0.620303349	1.04E-54	postive
VAV1	AC012236.1	0.509838892	1.44E-34	postive
CD19	AC012236.1	0.504469752	9.13E-34	postive
INPP5D	AC012236.1	0.537616456	6.06E-39	postive
FCGR2B	AC012236.1	0.556836111	3.21E-42	postive
RASGRP3	AC012236.1	0.556225137	4.11E-42	postive
PLCG2	AC012236.1	0.609946929	1.78E-52	postive
PRKCB	AC012236.1	0.561258827	5.27E-43	postive
IGHA1	AC012236.1	0.821936067	2.60E-124	postive
IGHA2	AC012236.1	0.723619234	1.51E-82	postive
IGHD	AC012236.1	0.513331032	4.28E-35	postive
IGHD2-2	AC012236.1	0.533753776	2.60E-38	postive
IGHD6-25	AC012236.1	0.781746618	1.29E-104	postive
IGHG1	AC012236.1	0.803321855	1.27E-114	postive
IGHG2	AC012236.1	0.815976659	4.32E-121	postive
IGHG3	AC012236.1	0.787371064	4.12E-107	postive
IGHG4	AC012236.1	0.679574191	2.82E-69	postive
IGHJ2	AC012236.1	0.736261042	7.62E-87	postive
IGHJ3	AC012236.1	0.821153428	6.99E-124	postive
IGHV1-2	AC012236.1	0.632858086	1.57E-57	postive
IGHV1-24	AC012236.1	0.662829218	8.02E-65	postive
IGHV1-46	AC012236.1	0.741131229	1.44E-88	postive
IGHV1-69	AC012236.1	0.712550883	5.62E-79	postive
IGHV2-26	AC012236.1	0.565660842	8.50E-44	postive
IGHV2-5	AC012236.1	0.649700117	1.58E-61	postive
IGHV2-70	AC012236.1	0.665327744	1.81E-65	postive
IGHV3-11	AC012236.1	0.619047358	1.96E-54	postive
IGHV3-13	AC012236.1	0.528646425	1.74E-37	postive
IGHV3-15	AC012236.1	0.672376001	2.52E-67	postive
IGHV3-20	AC012236.1	0.575970291	1.06E-45	postive
IGHV3-21	AC012236.1	0.65395351	1.41E-62	postive
IGHV3-23	AC012236.1	0.774776797	1.26E-101	postive
IGHV3-30	AC012236.1	0.71022585	3.01E-78	postive
IGHV3-33	AC012236.1	0.641259948	1.71E-59	postive
IGHV3-35	AC012236.1	0.681697371	7.32E-70	postive

IGHV3-38	AC012236.1	0.557772924	2.19E-42	postive
IGHV3-43	AC012236.1	0.58034402	1.58E-46	postive
IGHV3-48	AC012236.1	0.535680189	1.26E-38	postive
IGHV3-49	AC012236.1	0.595621671	1.60E-49	postive
IGHV3-53	AC012236.1	0.628283764	1.73E-56	postive
IGHV3-66	AC012236.1	0.669987148	1.09E-66	postive
IGHV3-7	AC012236.1	0.610121134	1.63E-52	postive
IGHV3-72	AC012236.1	0.573940601	2.55E-45	postive
IGHV3-74	AC012236.1	0.614024712	2.40E-53	postive
IGHV4-28	AC012236.1	0.806808049	2.35E-116	postive
IGHV4-31	AC012236.1	0.608862803	3.01E-52	postive
IGHV4-34	AC012236.1	0.653006648	2.43E-62	postive
IGHV4-39	AC012236.1	0.652647065	2.98E-62	postive
IGHV4-59	AC012236.1	0.703406973	3.75E-76	postive
IGHV4-61	AC012236.1	0.671542397	4.20E-67	postive
IGHV5-51	AC012236.1	0.744801874	6.85E-90	postive
IGKC	AC012236.1	0.882116048	1.35E-165	postive
IGKJ5	AC012236.1	0.843943426	2.42E-137	postive
IGKV1-12	AC012236.1	0.584009454	3.12E-47	postive
IGKV1-13	AC012236.1	0.63226951	2.14E-57	postive
IGKV1-16	AC012236.1	0.543970874	5.28E-40	postive
IGKV1-17	AC012236.1	0.5489081	7.65E-41	postive
IGKV1-27	AC012236.1	0.669965716	1.10E-66	postive
IGKV1-33	AC012236.1	0.668022482	3.58E-66	postive
IGKV1-39	AC012236.1	0.771187651	3.99E-100	postive
IGKV1-5	AC012236.1	0.750743178	4.40E-92	postive
IGKV1-8	AC012236.1	0.70311861	4.58E-76	postive
IGKV1-9	AC012236.1	0.505025921	7.55E-34	postive
IGKV1D-12	AC012236.1	0.661095866	2.23E-64	postive
IGKV1D-13	AC012236.1	0.528678858	1.72E-37	postive
IGKV1D-33	AC012236.1	0.610316074	1.48E-52	postive
IGKV1D-39	AC012236.1	0.754511036	1.66E-93	postive
IGKV1D-42	AC012236.1	0.738505194	1.24E-87	postive
IGKV1D-43	AC012236.1	0.509346524	1.71E-34	postive
IGKV1D-8	AC012236.1	0.747350275	7.99E-91	postive
IGKV2-28	AC012236.1	0.578304879	3.85E-46	postive
IGKV2D-28	AC012236.1	0.556101985	4.32E-42	postive
IGKV3-11	AC012236.1	0.835012013	8.00E-132	postive
IGKV3-15	AC012236.1	0.833274679	8.68E-131	postive
IGKV3-20	AC012236.1	0.79483061	1.53E-110	postive
IGKV3-7	AC012236.1	0.766607532	2.98E-98	postive
IGKV3D-11	AC012236.1	0.763291217	6.36E-97	postive
IGKV3D-15	AC012236.1	0.72824611	4.31E-84	postive
IGKV3D-20	AC012236.1	0.541175322	1.55E-39	postive
IGKV3D-7	AC012236.1	0.681953549	6.22E-70	postive
IGKV4-1	AC012236.1	0.708198675	1.28E-77	postive
IGKV5-2	AC012236.1	0.538632913	4.11E-39	postive
IGLC2	AC012236.1	0.852809297	3.54E-143	postive
IGLC3	AC012236.1	0.720575398	1.51E-81	postive
IGLC6	AC012236.1	0.632825091	1.60E-57	postive
IGLC7	AC012236.1	0.509314518	1.73E-34	postive
IGLJ1	AC012236.1	0.699384774	6.06E-75	postive
IGLJ2	AC012236.1	0.746874803	1.20E-90	postive
IGLJ3	AC012236.1	0.749279159	1.55E-91	postive
IGLV1-36	AC012236.1	0.691762453	1.04E-72	postive
IGLV1-40	AC012236.1	0.761715908	2.68E-96	postive

IGLV1-44	AC012236.1	0.773507422	4.31E-101	postive
IGLV1-50	AC012236.1	0.614766131	1.66E-53	postive
IGLV1-51	AC012236.1	0.677203092	1.26E-68	postive
IGLV2-11	AC012236.1	0.639867862	3.65E-59	postive
IGLV2-14	AC012236.1	0.682479535	4.44E-70	postive
IGLV2-23	AC012236.1	0.617464396	4.33E-54	postive
IGLV2-33	AC012236.1	0.619224079	1.79E-54	postive
IGLV2-8	AC012236.1	0.52733399	2.82E-37	postive
IGLV3-1	AC012236.1	0.692522925	6.28E-73	postive
IGLV3-10	AC012236.1	0.531795806	5.41E-38	postive
IGLV3-16	AC012236.1	0.501053228	2.90E-33	postive
IGLV3-19	AC012236.1	0.622401991	3.58E-55	postive
IGLV3-21	AC012236.1	0.620811784	8.03E-55	postive
IGLV3-25	AC012236.1	0.703866804	2.72E-76	postive
IGLV3-27	AC012236.1	0.573332922	3.31E-45	postive
IGLV3-9	AC012236.1	0.538596066	4.17E-39	postive
IGLV4-69	AC012236.1	0.663749021	4.64E-65	postive
IGLV6-57	AC012236.1	0.609172703	2.59E-52	postive
IGLV9-49	AC012236.1	0.556527399	3.64E-42	postive
ADA2	AC012236.1	0.568646471	2.43E-44	postive
GMFG	AC012236.1	0.540909566	1.72E-39	postive
IL16	AC012236.1	0.681175666	1.02E-69	postive
IL10RA	AC012236.1	0.661162064	2.15E-64	postive
IL12RB1	AC012236.1	0.560038722	8.70E-43	postive
TNFRSF17	AC012236.1	0.847269991	1.73E-139	postive
ICAM2	AC012236.1	0.558452611	1.66E-42	postive
ITGAL	AC012236.1	0.574526415	1.98E-45	postive
NCR3	AC012236.1	0.536905744	7.93E-39	postive
CD247	AC012236.1	0.551722019	2.51E-41	postive
ZAP70	AC012236.1	0.504409503	9.32E-34	postive
CD3D	AC012236.1	0.521719785	2.17E-36	postive
CD3E	AC012236.1	0.593487077	4.30E-49	postive
PTPRC	AC012236.1	0.560551275	7.05E-43	postive
ITK	AC012236.1	0.616018789	8.92E-54	postive
GRAP2	AC012236.1	0.50653066	4.52E-34	postive
CD28	AC012236.1	0.600940314	1.33E-50	postive
CTLA4	AC012236.1	0.520685218	3.15E-36	postive
TRAC	AC012236.1	0.522935836	1.40E-36	postive
TRAV8-3	AC012236.1	0.512044095	6.71E-35	postive
TRAV9-2	AC012236.1	0.503840364	1.13E-33	postive
TRBC1	AC012236.1	0.580473033	1.49E-46	postive
TRBC2	AC012236.1	0.591774972	9.41E-49	postive
TRBJ2-1	AC012236.1	0.521353193	2.48E-36	postive
TRBJ2-7	AC012236.1	0.545807137	2.58E-40	postive
TRBV2	AC012236.1	0.508597705	2.22E-34	postive
TRBV3-1	AC012236.1	0.514207098	3.15E-35	postive
TRBV5-1	AC012236.1	0.545951728	2.44E-40	postive
TRBV5-4	AC012236.1	0.561734807	4.33E-43	postive
TRBV5-6	AC012236.1	0.506968115	3.89E-34	postive
TRBV6-1	AC012236.1	0.572300829	5.14E-45	postive
TRBV6-5	AC012236.1	0.518434052	7.05E-36	postive
TRBV18	AC012236.1	0.572988358	3.83E-45	postive
TRBV19	AC012236.1	0.510745105	1.05E-34	postive
TRBV20-1	AC012236.1	0.566755091	5.38E-44	postive
TRBV28	AC012236.1	0.594877683	2.26E-49	postive
TRBV29-1	AC012236.1	0.544780105	3.85E-40	postive

FGFR1	AC087623.1	0.73596908	9.63E-87	postive
VGF	LINC02657	0.746822846	1.25E-90	postive
SKIV2L	HCG18	0.622721071	3.04E-55	postive
DAXX	HCG18	0.583366074	4.16E-47	postive
TRIM27	HCG18	0.610815205	1.16E-52	postive
RXRB	HCG18	0.55818073	1.86E-42	postive
GNRH1	AC234582.1	0.524877509	6.92E-37	postive
GIPR	AC234582.1	0.563823447	1.83E-43	postive
IRF9	U62317.2	0.568261575	2.86E-44	postive
TYMP	U62317.2	0.604757951	2.17E-51	postive
BPIFB1	ANKRD44-AS1	0.523197065	1.27E-36	postive
BPIFA1	ANKRD44-AS1	0.500760544	3.20E-33	postive
SCGB3A1	ANKRD44-AS1	0.643782591	4.29E-60	postive
CHGA	AC026471.2	0.553788695	1.10E-41	postive
GHRH	AC026471.2	0.554589124	7.95E-42	postive
SCG2	AC026471.2	0.567912859	3.31E-44	postive
TRH	AC026471.2	0.544223743	4.79E-40	postive
GNRH1	AC004918.1	0.514388531	2.95E-35	postive
TNFRSF25	AC004918.1	0.522109197	1.89E-36	postive
RBP4	AC060780.1	0.62879478	1.33E-56	postive
RBP2	AC060780.1	0.549070928	7.17E-41	postive
APOM	AC060780.1	0.711785349	9.78E-79	postive
PLA2G2A	AC060780.1	0.710811049	1.98E-78	postive
APOH	AC060780.1	0.647219842	6.37E-61	postive
VTN	AC060780.1	0.553821021	1.08E-41	postive
XCL2	AC060780.1	0.622511817	3.38E-55	postive
SEMA4G	AC060780.1	0.54002941	2.41E-39	postive
AGT	AC060780.1	0.671952457	3.27E-67	postive
LEPR	AC060780.1	0.563088214	2.48E-43	postive
GNRH1	AC018653.3	0.500475677	3.52E-33	postive
PLCG1	AL356299.3	0.501842765	2.22E-33	postive
CHGA	AP001160.3	0.592243534	7.60E-49	postive
GHRH	AP001160.3	0.593192712	4.92E-49	postive
SCG2	AP001160.3	0.584119308	2.97E-47	postive
TRH	AP001160.3	0.56627204	6.58E-44	postive
SEMA6C	AC011005.4	0.506048662	5.33E-34	postive
PLXNB1	AC011005.4	0.569597812	1.62E-44	postive
DDX17	RUSC1-AS1	0.509627975	1.55E-34	postive
GNRH1	RUSC1-AS1	0.537234431	7.00E-39	postive
DDX17	AC109460.2	0.547809363	1.18E-40	postive
IRF9	AC109460.2	0.512925809	4.93E-35	postive
PLXNB1	AC109460.2	0.553935016	1.03E-41	postive
GNRH1	AC109460.2	0.54794348	1.12E-40	postive
RABEP2	AC109460.2	0.542181609	1.05E-39	postive
NR2C2	AC109460.2	0.501762407	2.28E-33	postive
TNFRSF25	AC109460.2	0.500831471	3.12E-33	postive
CXCL14	AC114296.1	0.583220024	4.43E-47	postive
CD1C	AC008105.3	0.511208404	8.98E-35	postive
HLA-DMA	AC008105.3	0.554503128	8.23E-42	postive
HLA-DOB	AC008105.3	0.635746346	3.37E-58	postive
HLA-F	AC008105.3	0.561567436	4.64E-43	postive
LTA	AC008105.3	0.631943459	2.54E-57	postive
CIITA	AC008105.3	0.597636279	6.29E-50	postive
IFI30	AC008105.3	0.67441636	7.14E-68	postive
RBP5	AC008105.3	0.511706309	7.55E-35	postive
CETP	AC008105.3	0.61050225	1.35E-52	postive

CD40LG	AC008105.3	0.715865859	4.99E-80	postive
TRIM22	AC008105.3	0.519021511	5.72E-36	postive
PDCD1	AC008105.3	0.6693959	1.56E-66	postive
CCR7	AC008105.3	0.511561465	7.94E-35	postive
CCL21	AC008105.3	0.594911736	2.23E-49	postive
CXCR4	AC008105.3	0.526027946	4.55E-37	postive
CCR4	AC008105.3	0.520736941	3.09E-36	postive
CD79A	AC008105.3	0.59368707	3.92E-49	postive
CD79B	AC008105.3	0.642472821	8.81E-60	postive
BTK	AC008105.3	0.65733073	2.02E-63	postive
VAV1	AC008105.3	0.554382006	8.64E-42	postive
RAC2	AC008105.3	0.515300727	2.14E-35	postive
CD19	AC008105.3	0.669925987	1.13E-66	postive
CR2	AC008105.3	0.596247088	1.20E-49	postive
PIK3CD	AC008105.3	0.516902442	1.22E-35	postive
INPP5D	AC008105.3	0.697019947	3.04E-74	postive
CD22	AC008105.3	0.617942319	3.41E-54	postive
CD72	AC008105.3	0.691887329	9.58E-73	postive
PTPN6	AC008105.3	0.62174247	5.01E-55	postive
RASGRP3	AC008105.3	0.63363647	1.04E-57	postive
PLCG2	AC008105.3	0.537928968	5.38E-39	postive
PRKCB	AC008105.3	0.608115913	4.32E-52	postive
IGLJ1	AC008105.3	0.65604124	4.25E-63	postive
FLT3LG	AC008105.3	0.555764006	4.95E-42	postive
GMFG	AC008105.3	0.577331417	5.89E-46	postive
IL16	AC008105.3	0.650406952	1.06E-61	postive
LTB	AC008105.3	0.584915848	2.08E-47	postive
TNFSF14	AC008105.3	0.593765508	3.78E-49	postive
IL10RA	AC008105.3	0.609923738	1.80E-52	postive
IL12RB1	AC008105.3	0.653780882	1.56E-62	postive
IL18RAP	AC008105.3	0.526901197	3.30E-37	postive
IL21R	AC008105.3	0.506835504	4.07E-34	postive
IL2RG	AC008105.3	0.510219605	1.27E-34	postive
IL3RA	AC008105.3	0.539436072	3.03E-39	postive
TNFRSF13C	AC008105.3	0.61779274	3.68E-54	postive
TNFRSF14	AC008105.3	0.651336799	6.28E-62	postive
TNFRSF1B	AC008105.3	0.503509087	1.26E-33	postive
ITGAL	AC008105.3	0.706425523	4.51E-77	postive
LCK	AC008105.3	0.625815967	6.22E-56	postive
NCR3	AC008105.3	0.666328831	9.94E-66	postive
CD247	AC008105.3	0.673114156	1.60E-67	postive
ZAP70	AC008105.3	0.77256762	1.07E-100	postive
CD3D	AC008105.3	0.575870849	1.11E-45	postive
CD3E	AC008105.3	0.65380003	1.54E-62	postive
PTPRC	AC008105.3	0.61425389	2.14E-53	postive
ITK	AC008105.3	0.662833511	8.00E-65	postive
GRAP2	AC008105.3	0.65600487	4.34E-63	postive
CD28	AC008105.3	0.590133397	1.99E-48	postive
ICOS	AC008105.3	0.578329155	3.81E-46	postive
CTLA4	AC008105.3	0.634308909	7.26E-58	postive
TRAC	AC008105.3	0.561938747	3.98E-43	postive
TRAV8-3	AC008105.3	0.628473454	1.57E-56	postive
TRAV8-6	AC008105.3	0.526536562	3.78E-37	postive
TRBC1	AC008105.3	0.692814059	5.17E-73	postive
TRBC2	AC008105.3	0.701602996	1.31E-75	postive
TRBJ2-1	AC008105.3	0.615450026	1.18E-53	postive

TRBJ2-2	AC008105.3	0.707445446	2.19E-77	postive
TRBJ2-3	AC008105.3	0.671045386	5.69E-67	postive
TRBJ2-7	AC008105.3	0.760996321	5.14E-96	postive
TRBV2	AC008105.3	0.56446859	1.40E-43	postive
TRBV3-1	AC008105.3	0.542895701	8.01E-40	postive
TRBV5-1	AC008105.3	0.577896368	4.60E-46	postive
TRBV6-5	AC008105.3	0.512708061	5.32E-35	postive
TRBV18	AC008105.3	0.621174375	6.68E-55	postive
TRBV19	AC008105.3	0.52514812	6.27E-37	postive
TRBV28	AC008105.3	0.704150951	2.23E-76	postive
TRBV29-1	AC008105.3	0.554618027	7.86E-42	postive
NPY	KC877982.1	0.610357454	1.45E-52	postive
IGLV3-22	LINC01705	0.510146351	1.30E-34	postive
CD1C	AC243960.1	0.699159279	7.07E-75	postive
CD1D	AC243960.1	0.609926931	1.79E-52	postive
HLA-DMA	AC243960.1	0.576389303	8.86E-46	postive
HLA-DMB	AC243960.1	0.640379132	2.77E-59	postive
HLA-DOB	AC243960.1	0.783530106	2.12E-105	postive
HLA-DPB1	AC243960.1	0.508643564	2.18E-34	postive
LTA	AC243960.1	0.801775144	7.28E-114	postive
CIITA	AC243960.1	0.589236987	2.99E-48	postive
IFI30	AC243960.1	0.533072602	3.36E-38	postive
RBP5	AC243960.1	0.573428482	3.17E-45	postive
CETP	AC243960.1	0.771768015	2.29E-100	postive
ISG20	AC243960.1	0.521208007	2.61E-36	postive
CD40LG	AC243960.1	0.858482189	4.08E-147	postive
PDCD1	AC243960.1	0.759703074	1.65E-95	postive
CCR7	AC243960.1	0.617288413	4.73E-54	postive
CCL21	AC243960.1	0.777489827	8.92E-103	postive
CXCR4	AC243960.1	0.650850042	8.27E-62	postive
CCR4	AC243960.1	0.621655678	5.23E-55	postive
CD79A	AC243960.1	0.811632614	8.15E-119	postive
CD79B	AC243960.1	0.874164424	5.80E-159	postive
BTK	AC243960.1	0.799715742	7.27E-113	postive
VAV1	AC243960.1	0.615848196	9.71E-54	postive
RAC2	AC243960.1	0.579428502	2.36E-46	postive
CD19	AC243960.1	0.868259055	2.53E-154	postive
CR2	AC243960.1	0.78676166	7.74E-107	postive
PIK3CD	AC243960.1	0.58061083	1.40E-46	postive
INPP5D	AC243960.1	0.772608768	1.02E-100	postive
CD22	AC243960.1	0.808620031	2.85E-117	postive
CD72	AC243960.1	0.893573142	4.83E-176	postive
PTPN6	AC243960.1	0.615883275	9.54E-54	postive
RASGRP3	AC243960.1	0.726146761	2.18E-83	postive
PLCG2	AC243960.1	0.703947082	2.57E-76	postive
PRKCB	AC243960.1	0.778912171	2.19E-103	postive
IGHD1-1	AC243960.1	0.517218252	1.09E-35	postive
IGKJ5	AC243960.1	0.531459108	6.14E-38	postive
IGLJ1	AC243960.1	0.803139205	1.56E-114	postive
CXCR3	AC243960.1	0.513107464	4.63E-35	postive
FLT3LG	AC243960.1	0.573110179	3.64E-45	postive
GMFG	AC243960.1	0.786098001	1.54E-106	postive
IL16	AC243960.1	0.833298126	8.40E-131	postive
LTB	AC243960.1	0.684160154	1.51E-70	postive
IL10RA	AC243960.1	0.678270544	6.43E-69	postive
IL12RB1	AC243960.1	0.749236882	1.60E-91	postive

IL18RAP	AC243960.1	0.564913786	1.16E-43	postive
IL21R	AC243960.1	0.576227671	9.50E-46	postive
IL2RG	AC243960.1	0.516001679	1.67E-35	postive
NR1H3	AC243960.1	0.51680612	1.26E-35	postive
TNFRSF13C	AC243960.1	0.802497645	3.23E-114	postive
TNFRSF14	AC243960.1	0.535341406	1.43E-38	postive
TNFRSF17	AC243960.1	0.537465698	6.41E-39	postive
ITGAL	AC243960.1	0.798358697	3.26E-112	postive
LCK	AC243960.1	0.782463574	6.26E-105	postive
NCR3	AC243960.1	0.904042264	1.08E-186	postive
CD247	AC243960.1	0.779064673	1.88E-103	postive
ZAP70	AC243960.1	0.851081769	5.19E-142	postive
HCST	AC243960.1	0.611642136	7.75E-53	postive
CD48	AC243960.1	0.770409921	8.35E-100	postive
SH2D1A	AC243960.1	0.697098163	2.89E-74	postive
CD3D	AC243960.1	0.806276748	4.33E-116	postive
CD3E	AC243960.1	0.782735523	4.75E-105	postive
CD3G	AC243960.1	0.573884287	2.61E-45	postive
PTPRC	AC243960.1	0.757977151	7.75E-95	postive
ITK	AC243960.1	0.76275415	1.04E-96	postive
GRAP2	AC243960.1	0.72214224	4.63E-82	postive
CD28	AC243960.1	0.739572464	5.19E-88	postive
ICOS	AC243960.1	0.70937948	5.52E-78	postive
CTLA4	AC243960.1	0.713440572	2.94E-79	postive
TRAC	AC243960.1	0.688446642	9.30E-72	postive
TRAV2	AC243960.1	0.61435902	2.03E-53	postive
TRAV4	AC243960.1	0.635829692	3.22E-58	postive
TRAV8-3	AC243960.1	0.74458241	8.23E-90	postive
TRAV8-6	AC243960.1	0.610685506	1.24E-52	postive
TRAV9-2	AC243960.1	0.662649845	8.92E-65	postive
TRAV12-1	AC243960.1	0.522652406	1.55E-36	postive
TRAV12-2	AC243960.1	0.523928052	9.77E-37	postive
TRAV12-3	AC243960.1	0.526357269	4.03E-37	postive
TRAV13-1	AC243960.1	0.521473097	2.37E-36	postive
TRBC1	AC243960.1	0.813870139	5.58E-120	postive
TRBC2	AC243960.1	0.84715574	2.05E-139	postive
TRBJ2-1	AC243960.1	0.710813679	1.97E-78	postive
TRBJ2-2	AC243960.1	0.781028082	2.65E-104	postive
TRBJ2-3	AC243960.1	0.824180878	1.48E-125	postive
TRBJ2-7	AC243960.1	0.812538872	2.76E-119	postive
TRBV2	AC243960.1	0.757573033	1.11E-94	postive
TRBV3-1	AC243960.1	0.716341225	3.52E-80	postive
TRBV5-1	AC243960.1	0.781795417	1.23E-104	postive
TRBV5-4	AC243960.1	0.635070412	4.84E-58	postive
TRBV5-6	AC243960.1	0.596498196	1.07E-49	postive
TRBV6-1	AC243960.1	0.629129128	1.11E-56	postive
TRBV6-5	AC243960.1	0.643149749	6.07E-60	postive
TRBV7-9	AC243960.1	0.608896222	2.96E-52	postive
TRBV9	AC243960.1	0.576931047	7.00E-46	postive
TRBV10-3	AC243960.1	0.600440405	1.69E-50	postive
TRBV12-4	AC243960.1	0.560631434	6.82E-43	postive
TRBV18	AC243960.1	0.747497144	7.06E-91	postive
TRBV19	AC243960.1	0.619508478	1.55E-54	postive
TRBV20-1	AC243960.1	0.642425325	9.04E-60	postive
TRBV28	AC243960.1	0.822628251	1.08E-124	postive
TRBV29-1	AC243960.1	0.688796743	7.39E-72	postive

SHC3	AL353150.1	0.726402623	1.79E-83	postive
THPO	AC016394.3	0.538623969	4.13E-39	postive
OSGIN1	ARHGEF26-AS1	0.520110178	3.87E-36	postive
BPIFA1	WDR86-AS1	0.53368914	2.67E-38	postive
MC1R	AL162586.1	0.501348315	2.63E-33	postive
SLC22A17	MEG3	0.662703256	8.64E-65	postive
PCSK1	MEG3	0.635243009	4.41E-58	postive
VTN	MEG3	0.738195605	1.59E-87	postive
CHGA	MEG3	0.947546084	7.80E-250	postive
GHRH	MEG3	0.949967942	7.82E-255	postive
GRP	MEG3	0.642105454	1.08E-59	postive
SCG2	MEG3	0.938002949	3.25E-232	postive
TRH	MEG3	0.927225705	2.05E-215	postive
IL20RA	MEG3	0.596145407	1.26E-49	postive
NR0B2	MEG3	0.723571875	1.57E-82	postive
SSTR1	MEG3	0.542259224	1.02E-39	postive
SHC2	MEG3	0.546165948	2.24E-40	postive
GNRH1	AC232271.1	0.580244561	1.65E-46	postive
COLEC12	AC104083.1	0.557327003	2.63E-42	postive
PDGFRB	AC104083.1	0.663275222	6.16E-65	postive
EDNRA	AC104083.1	0.552836084	1.61E-41	postive
PLXNC1	AC104083.1	0.500177074	3.89E-33	postive
TGFB3	AC104083.1	0.559670262	1.01E-42	postive
CXCL2	AL162511.1	0.597380835	7.08E-50	postive
SFTPD	AL162511.1	0.686707158	2.90E-71	postive
A2M	AL162511.1	0.571930802	6.03E-45	postive
SFTPA1	AL162511.1	0.690010087	3.32E-72	postive
SFTPA2	AL162511.1	0.666643422	8.23E-66	postive
MARCO	AL162511.1	0.515508568	1.99E-35	postive
ARRB1	AL162511.1	0.540351722	2.13E-39	postive
AGER	AL162511.1	0.708096663	1.38E-77	postive
CCL23	AL162511.1	0.60119463	1.18E-50	postive
AGRP	AL162511.1	0.53900905	3.56E-39	postive
VEGFD	AL162511.1	0.643554942	4.86E-60	postive
RETN	AL162511.1	0.56460911	1.32E-43	postive
ACVRL1	AL162511.1	0.521556495	2.30E-36	postive
NPR1	AL162511.1	0.589870594	2.24E-48	postive
S1PR1	AL162511.1	0.504259322	9.80E-34	postive
SCTR	AL162511.1	0.507485752	3.25E-34	postive
TEK	AL162511.1	0.537875536	5.49E-39	postive
SRC	NORAD	0.537490885	6.35E-39	postive
GNRH1	AC092119.2	0.615757552	1.02E-53	postive
RXRB	Z84485.1	0.518367368	7.22E-36	postive
TMSB10	CYTOR	0.510193892	1.28E-34	postive
TPT1	CYTOR	0.544338007	4.58E-40	postive
CKLF	CYTOR	0.514696469	2.65E-35	postive
TNFRSF10A	TNFRSF10A-AS1	0.872968089	5.27E-158	postive
TNFRSF10B	TNFRSF10A-AS1	0.646811542	8.00E-61	postive
SYTL1	AL354836.1	0.512545121	5.63E-35	postive
PLXNB1	AL354836.1	0.642992159	6.62E-60	postive
GNRH1	AL354836.1	0.584871117	2.13E-47	postive
TNFRSF25	AL354836.1	0.557243824	2.72E-42	postive
THPO	AC026689.1	0.60576649	1.34E-51	postive
PLXNB1	ZKSCAN2-DT	0.502514935	1.77E-33	postive
GNRH1	ZKSCAN2-DT	0.532447995	4.24E-38	postive
RABEP2	ZKSCAN2-DT	0.520766996	3.06E-36	postive

COLEC12	MAGI2-AS3	0.576783851	7.47E-46	postive
A2M	MAGI2-AS3	0.528002198	2.20E-37	postive
VIM	MAGI2-AS3	0.526327665	4.08E-37	postive
FLT4	MAGI2-AS3	0.530005315	1.05E-37	postive
S1PR1	MAGI2-AS3	0.516109938	1.61E-35	postive
PPIA	SNHG15	0.604247057	2.78E-51	postive
HLA-DOB	AC012181.2	0.500934834	3.02E-33	postive
CD40LG	AC012181.2	0.511547263	7.98E-35	postive
CD79A	AC012181.2	0.532576038	4.05E-38	postive
BTK	AC012181.2	0.541540571	1.35E-39	postive
FCGR2B	AC012181.2	0.502388018	1.85E-33	postive
IGHA1	AC012181.2	0.503212034	1.40E-33	postive
IGHA2	AC012181.2	0.503270871	1.37E-33	postive
IGHD6-25	AC012181.2	0.547805685	1.18E-40	postive
IGHG2	AC012181.2	0.536747062	8.42E-39	postive
IGHG3	AC012181.2	0.522755306	1.49E-36	postive
IGHV4-28	AC012181.2	0.527904105	2.29E-37	postive
IGKC	AC012181.2	0.536894622	7.97E-39	postive
IGKJ5	AC012181.2	0.639652613	4.11E-59	postive
IGKV1D-39	AC012181.2	0.504160228	1.01E-33	postive
IGKV3-15	AC012181.2	0.520893883	2.92E-36	postive
IGKV3-20	AC012181.2	0.521876805	2.05E-36	postive
IGKV3-7	AC012181.2	0.503744533	1.17E-33	postive
IGLC2	AC012181.2	0.533508638	2.85E-38	postive
IGLJ1	AC012181.2	0.538174919	4.90E-39	postive
IGLJ2	AC012181.2	0.515251121	2.18E-35	postive
IGLJ3	AC012181.2	0.505164699	7.20E-34	postive
IGLV1-40	AC012181.2	0.51221552	6.32E-35	postive
IGLV2-14	AC012181.2	0.506113404	5.21E-34	postive
IL16	AC012181.2	0.539275781	3.22E-39	postive
IL10RA	AC012181.2	0.597497115	6.71E-50	postive
TNFRSF17	AC012181.2	0.548899019	7.67E-41	postive
ITK	AC012181.2	0.5392197	3.29E-39	postive
CTLA4	AC012181.2	0.503046211	1.48E-33	postive
PLXNB1	ZNF436-AS1	0.50627225	4.93E-34	postive
PLCG1	ZNF436-AS1	0.596515371	1.06E-49	postive
GSK3B	AC092910.3	0.511045201	9.50E-35	postive
PRKCQ	PRKCQ-AS1	0.80632772	4.09E-116	postive
LMBR1L	AC006042.1	0.506745461	4.19E-34	postive
GIPR	AC006042.1	0.54642949	2.03E-40	postive
LMBR1L	AL132989.1	0.509037505	1.91E-34	postive
DDX17	AL132989.1	0.623942405	1.63E-55	postive
IRF9	AL132989.1	0.535245272	1.49E-38	postive
PLXNB1	AL132989.1	0.522043122	1.93E-36	postive
GNRH1	AL132989.1	0.613261483	3.50E-53	postive
NR2C2	AL132989.1	0.549936843	5.09E-41	postive
TNFRSF25	AL132989.1	0.512371719	5.99E-35	postive
CD1C	LINC01857	0.645652478	1.52E-60	postive
CD1D	LINC01857	0.541812632	1.22E-39	postive
CD4	LINC01857	0.521762913	2.14E-36	postive
CD74	LINC01857	0.579354806	2.44E-46	postive
CTSS	LINC01857	0.553101911	1.44E-41	postive
HLA-DMA	LINC01857	0.626240132	4.99E-56	postive
HLA-DMB	LINC01857	0.680807748	1.29E-69	postive
HLA-DOA	LINC01857	0.577835096	4.73E-46	postive
HLA-DOB	LINC01857	0.706376125	4.67E-77	postive

HLA-DPA1	LINC01857	0.506168808	5.11E-34	postive
HLA-DPB1	LINC01857	0.596585719	1.03E-49	postive
HLA-DQA2	LINC01857	0.541826268	1.21E-39	postive
HLA-DRA	LINC01857	0.50277972	1.62E-33	postive
LTA	LINC01857	0.781558984	1.56E-104	postive
CIITA	LINC01857	0.558417581	1.69E-42	postive
IFI30	LINC01857	0.582508218	6.08E-47	postive
RBP5	LINC01857	0.677076018	1.36E-68	postive
CETP	LINC01857	0.761588722	3.00E-96	postive
CD40LG	LINC01857	0.79796863	5.01E-112	postive
PDCD1	LINC01857	0.687611968	1.61E-71	postive
CCR7	LINC01857	0.564029732	1.68E-43	postive
CCL21	LINC01857	0.704357497	1.93E-76	postive
CXCR4	LINC01857	0.64757993	5.21E-61	postive
CCR4	LINC01857	0.615213728	1.33E-53	postive
FGR	LINC01857	0.546551658	1.93E-40	postive
CD79A	LINC01857	0.707093899	2.81E-77	postive
CD79B	LINC01857	0.779404981	1.34E-103	postive
BTK	LINC01857	0.815941023	4.52E-121	postive
VAV1	LINC01857	0.630943053	4.31E-57	postive
RAC2	LINC01857	0.54470868	3.96E-40	postive
CD19	LINC01857	0.764598037	1.91E-97	postive
CR2	LINC01857	0.727755808	6.31E-84	postive
PIK3R5	LINC01857	0.501542179	2.46E-33	postive
PIK3CD	LINC01857	0.539020286	3.55E-39	postive
INPP5D	LINC01857	0.717779695	1.21E-80	postive
CD22	LINC01857	0.732888297	1.13E-85	postive
CD72	LINC01857	0.830152422	5.88E-129	postive
PTPN6	LINC01857	0.62075705	8.26E-55	postive
RASGRP3	LINC01857	0.751018249	3.47E-92	postive
PLCG2	LINC01857	0.62899946	1.19E-56	postive
PRKCB	LINC01857	0.756518266	2.83E-94	postive
IGLJ1	LINC01857	0.659244376	6.61E-64	postive
CXCR3	LINC01857	0.51343026	4.13E-35	postive
ADA2	LINC01857	0.505353253	6.75E-34	postive
GMFG	LINC01857	0.706286298	4.97E-77	postive
IL16	LINC01857	0.766028397	5.10E-98	postive
LTB	LINC01857	0.641444938	1.55E-59	postive
IL10RA	LINC01857	0.701411233	1.50E-75	postive
IL12RB1	LINC01857	0.707069395	2.86E-77	postive
IL21R	LINC01857	0.637129016	1.61E-58	postive
IL2RG	LINC01857	0.581924155	7.87E-47	postive
IL3RA	LINC01857	0.539364895	3.11E-39	postive
NR1H3	LINC01857	0.589485741	2.67E-48	postive
TNFRSF13C	LINC01857	0.703422226	3.71E-76	postive
TNFRSF1B	LINC01857	0.600520109	1.63E-50	postive
ITGAL	LINC01857	0.766838997	2.40E-98	postive
ITGB2	LINC01857	0.511130235	9.23E-35	postive
LCK	LINC01857	0.701986544	1.01E-75	postive
NCR3	LINC01857	0.820617266	1.37E-123	postive
CD247	LINC01857	0.720138969	2.09E-81	postive
ZAP70	LINC01857	0.734146597	4.15E-86	postive
LCP2	LINC01857	0.523683469	1.07E-36	postive
HCST	LINC01857	0.623817892	1.74E-55	postive
CD48	LINC01857	0.661987303	1.32E-64	postive
SH2D1A	LINC01857	0.609221078	2.53E-52	postive

CD3D	LINC01857	0.674492804	6.81E-68	postive
CD3E	LINC01857	0.743537595	1.97E-89	postive
CD3G	LINC01857	0.538364582	4.56E-39	postive
PTPRC	LINC01857	0.7451986	4.91E-90	postive
ITK	LINC01857	0.720338936	1.80E-81	postive
GRAP2	LINC01857	0.665232637	1.92E-65	postive
CD28	LINC01857	0.75107935	3.29E-92	postive
ICOS	LINC01857	0.685317483	7.14E-71	postive
CTLA4	LINC01857	0.651278792	6.49E-62	postive
TRAC	LINC01857	0.682032882	5.91E-70	postive
TRAV2	LINC01857	0.648943571	2.42E-61	postive
TRAV4	LINC01857	0.543141149	7.28E-40	postive
TRAV8-3	LINC01857	0.70826831	1.22E-77	postive
TRAV8-6	LINC01857	0.638058928	9.73E-59	postive
TRAV9-2	LINC01857	0.653555168	1.77E-62	postive
TRAV12-1	LINC01857	0.568963277	2.12E-44	postive
TRAV12-2	LINC01857	0.515351347	2.10E-35	postive
TRAV12-3	LINC01857	0.518159772	7.77E-36	postive
TRAV13-1	LINC01857	0.548983019	7.42E-41	postive
TRBC1	LINC01857	0.742802177	3.63E-89	postive
TRBC2	LINC01857	0.775860162	4.40E-102	postive
TRBJ2-1	LINC01857	0.680796653	1.30E-69	postive
TRBJ2-2	LINC01857	0.726465968	1.71E-83	postive
TRBJ2-3	LINC01857	0.712107315	7.75E-79	postive
TRBJ2-7	LINC01857	0.725135111	4.75E-83	postive
TRBV2	LINC01857	0.726540583	1.61E-83	postive
TRBV3-1	LINC01857	0.675454189	3.75E-68	postive
TRBV5-1	LINC01857	0.749084561	1.83E-91	postive
TRBV5-4	LINC01857	0.595108738	2.03E-49	postive
TRBV5-6	LINC01857	0.593891189	3.57E-49	postive
TRBV6-1	LINC01857	0.582471573	6.18E-47	postive
TRBV6-5	LINC01857	0.595199601	1.95E-49	postive
TRBV7-9	LINC01857	0.599239433	2.97E-50	postive
TRBV10-3	LINC01857	0.57893251	2.93E-46	postive
TRBV18	LINC01857	0.708569315	9.84E-78	postive
TRBV19	LINC01857	0.609328723	2.40E-52	postive
TRBV20-1	LINC01857	0.664641699	2.73E-65	postive
TRBV28	LINC01857	0.769944794	1.30E-99	postive
TRBV29-1	LINC01857	0.679842629	2.38E-69	postive
IGKV2D-29	AC078802.1	0.660234318	3.70E-64	postive
IGLV7-43	AC078802.1	0.605935735	1.24E-51	postive
CTSB	AC025857.2	0.603374975	4.21E-51	postive
TRAF3	AL049840.4	0.513086587	4.66E-35	postive
AKT1	AL049840.4	0.55046129	4.14E-41	postive
VGF	LINC02677	0.614778248	1.65E-53	postive
CD8A	TRBV11-2	0.534927255	1.67E-38	postive
FASLG	TRBV11-2	0.500607174	3.37E-33	postive
CXCR6	TRBV11-2	0.543859447	5.51E-40	postive
IL12RB1	TRBV11-2	0.509069633	1.89E-34	postive
IL18RAP	TRBV11-2	0.508911595	1.99E-34	postive
CD247	TRBV11-2	0.554368722	8.69E-42	postive
CD3D	TRBV11-2	0.573658103	2.88E-45	postive
CD3E	TRBV11-2	0.570168442	1.28E-44	postive
PTPRC	TRBV11-2	0.515023329	2.36E-35	postive
ITK	TRBV11-2	0.501657686	2.37E-33	postive
TRAC	TRBV11-2	0.570880996	9.42E-45	postive

TRAV8-3	TRBV11-2	0.505195565	7.13E-34	postive
TRAV8-6	TRBV11-2	0.594208801	3.08E-49	postive
TRAV9-2	TRBV11-2	0.510167524	1.29E-34	postive
TRAV13-1	TRBV11-2	0.547264936	1.46E-40	postive
TRAV19	TRBV11-2	0.511818961	7.26E-35	postive
TRAV21	TRBV11-2	0.70266244	6.30E-76	postive
TRBC2	TRBV11-2	0.563199507	2.37E-43	postive
TRBV5-1	TRBV11-2	0.510521987	1.14E-34	postive
TRBV5-6	TRBV11-2	0.510383269	1.20E-34	postive
TRBV7-9	TRBV11-2	0.515894626	1.74E-35	postive
TRBV11-2	TRBV11-2	1	0	postive
TRBV12-4	TRBV11-2	0.501028221	2.92E-33	postive
TRBV18	TRBV11-2	0.527731356	2.44E-37	postive
TRBV20-1	TRBV11-2	0.515911253	1.73E-35	postive
TRBV28	TRBV11-2	0.537238913	6.99E-39	postive
FGFR3	CTBP1-DT	0.715608026	6.03E-80	postive
TNFRSF25	AC139100.2	0.506558701	4.47E-34	postive
CHGA	LINC00957	0.643594564	4.76E-60	postive
GHRH	LINC00957	0.649872099	1.44E-61	postive
SCG2	LINC00957	0.63117388	3.81E-57	postive
TRH	LINC00957	0.628946582	1.22E-56	postive
NR0B2	LINC00957	0.565787858	8.06E-44	postive
CD8A	DBH-AS1	0.543627146	6.03E-40	postive
HLA-DMA	DBH-AS1	0.513020305	4.77E-35	postive
HLA-DOB	DBH-AS1	0.569529794	1.67E-44	postive
LTA	DBH-AS1	0.60363253	3.72E-51	postive
CIITA	DBH-AS1	0.607292421	6.43E-52	postive
IFI30	DBH-AS1	0.596852685	9.06E-50	postive
CETP	DBH-AS1	0.539694832	2.74E-39	postive
CD40LG	DBH-AS1	0.636500891	2.25E-58	postive
TRIM22	DBH-AS1	0.519752419	4.40E-36	postive
PDCD1	DBH-AS1	0.74337884	2.25E-89	postive
FASLG	DBH-AS1	0.535297722	1.46E-38	postive
CCL21	DBH-AS1	0.514929538	2.44E-35	postive
CCR5	DBH-AS1	0.501003194	2.95E-33	postive
CD79A	DBH-AS1	0.505649707	6.10E-34	postive
CD79B	DBH-AS1	0.553115135	1.44E-41	postive
BTK	DBH-AS1	0.568567411	2.51E-44	postive
VAV1	DBH-AS1	0.503014635	1.50E-33	postive
CD19	DBH-AS1	0.565002481	1.12E-43	postive
PIK3CD	DBH-AS1	0.518516892	6.84E-36	postive
INPP5D	DBH-AS1	0.645248423	1.91E-60	postive
CD22	DBH-AS1	0.529951856	1.07E-37	postive
CD72	DBH-AS1	0.646000083	1.26E-60	postive
PTPN6	DBH-AS1	0.578861158	3.02E-46	postive
RASGRP3	DBH-AS1	0.547402164	1.38E-40	postive
PRKCB	DBH-AS1	0.578795113	3.11E-46	postive
IGLJ1	DBH-AS1	0.564346311	1.47E-43	postive
CXCR3	DBH-AS1	0.544232932	4.77E-40	postive
FLT3LG	DBH-AS1	0.574689593	1.85E-45	postive
IL16	DBH-AS1	0.593922856	3.51E-49	postive
LTB	DBH-AS1	0.505704259	5.99E-34	postive
IL10RA	DBH-AS1	0.604756375	2.18E-51	postive
IL12RB1	DBH-AS1	0.666147524	1.11E-65	postive
IL18RAP	DBH-AS1	0.512795478	5.16E-35	postive
IL21R	DBH-AS1	0.519211297	5.34E-36	postive

TNFRSF13C	DBH-AS1	0.5297259	1.17E-37	postive
TNFRSF14	DBH-AS1	0.611729323	7.43E-53	postive
ITGAL	DBH-AS1	0.721251952	9.07E-82	postive
LCK	DBH-AS1	0.672498989	2.33E-67	postive
NCR3	DBH-AS1	0.583703879	3.58E-47	postive
CD247	DBH-AS1	0.698105467	1.45E-74	postive
ZAP70	DBH-AS1	0.762030643	2.01E-96	postive
SH2D1A	DBH-AS1	0.528162054	2.08E-37	postive
CD3D	DBH-AS1	0.600909773	1.35E-50	postive
CD3E	DBH-AS1	0.690151311	3.03E-72	postive
PTPRC	DBH-AS1	0.607257445	6.54E-52	postive
ITK	DBH-AS1	0.674539082	6.62E-68	postive
GRAP2	DBH-AS1	0.685660603	5.72E-71	postive
CD28	DBH-AS1	0.542898284	8.00E-40	postive
ICOS	DBH-AS1	0.550941349	3.42E-41	postive
CTLA4	DBH-AS1	0.670149654	9.84E-67	postive
TRAC	DBH-AS1	0.602107358	7.68E-51	postive
TRAV4	DBH-AS1	0.543068888	7.49E-40	postive
TRAV8-3	DBH-AS1	0.70624191	5.13E-77	postive
TRAV8-6	DBH-AS1	0.532637405	3.95E-38	postive
TRBC1	DBH-AS1	0.714218998	1.67E-79	postive
TRBC2	DBH-AS1	0.701407788	1.50E-75	postive
TRBJ2-1	DBH-AS1	0.675891273	2.85E-68	postive
TRBJ2-2	DBH-AS1	0.652307425	3.62E-62	postive
TRBJ2-3	DBH-AS1	0.645272433	1.88E-60	postive
TRBJ2-7	DBH-AS1	0.727141121	1.01E-83	postive
TRBV2	DBH-AS1	0.589132406	3.13E-48	postive
TRBV3-1	DBH-AS1	0.529171802	1.43E-37	postive
TRBV5-1	DBH-AS1	0.584057815	3.06E-47	postive
TRBV5-6	DBH-AS1	0.510199122	1.28E-34	postive
TRBV6-5	DBH-AS1	0.569219358	1.91E-44	postive
TRBV18	DBH-AS1	0.5860944	1.23E-47	postive
TRBV19	DBH-AS1	0.558278017	1.79E-42	postive
TRBV20-1	DBH-AS1	0.535229282	1.49E-38	postive
TRBV28	DBH-AS1	0.706206596	5.26E-77	postive
TRBV29-1	DBH-AS1	0.56464625	1.30E-43	postive
TGFB2	TGFB2-AS1	0.934450777	2.31E-226	postive
CIITA	WAKMAR2	0.534068271	2.31E-38	postive
TNFAIP3	WAKMAR2	0.72450647	7.68E-83	postive
TYK2	WAKMAR2	0.59213512	7.99E-49	postive
PIK3CD	WAKMAR2	0.51040879	1.19E-34	postive
TNFRSF4	WAKMAR2	0.584740405	2.25E-47	postive
CD1C	LINC01781	0.631569826	3.10E-57	postive
CD1D	LINC01781	0.602568737	6.17E-51	postive
HLA-DMA	LINC01781	0.564634305	1.30E-43	postive
HLA-DMB	LINC01781	0.519538373	4.75E-36	postive
HLA-DOB	LINC01781	0.759553796	1.89E-95	postive
HLA-DPB1	LINC01781	0.517416669	1.01E-35	postive
LTA	LINC01781	0.610314564	1.48E-52	postive
IFI30	LINC01781	0.505677204	6.05E-34	postive
RBP5	LINC01781	0.501991565	2.11E-33	postive
CETP	LINC01781	0.590582748	1.62E-48	postive
ISG20	LINC01781	0.516050971	1.64E-35	postive
CD40LG	LINC01781	0.748153085	4.04E-91	postive
PDCD1	LINC01781	0.603189801	4.59E-51	postive
CCR7	LINC01781	0.547046817	1.59E-40	postive

CCL21	LINC01781	0.583280534	4.32E-47	postive
CXCR4	LINC01781	0.646148327	1.16E-60	postive
CCR4	LINC01781	0.644861526	2.36E-60	postive
CD79A	LINC01781	0.792281059	2.36E-109	postive
CD79B	LINC01781	0.676935516	1.49E-68	postive
BTK	LINC01781	0.73121627	4.23E-85	postive
VAV1	LINC01781	0.556905331	3.12E-42	postive
CD19	LINC01781	0.693662985	2.93E-73	postive
CR2	LINC01781	0.61686417	5.85E-54	postive
PIK3R5	LINC01781	0.503204736	1.40E-33	postive
INPP5D	LINC01781	0.674556403	6.55E-68	postive
CD22	LINC01781	0.574821564	1.74E-45	postive
CD72	LINC01781	0.636637914	2.09E-58	postive
FCGR2B	LINC01781	0.512057982	6.68E-35	postive
RASGRP3	LINC01781	0.655893628	4.63E-63	postive
PLCG2	LINC01781	0.65962574	5.29E-64	postive
PRKCB	LINC01781	0.709356596	5.61E-78	postive
IGHA1	LINC01781	0.562994032	2.58E-43	postive
IGHA2	LINC01781	0.50696149	3.90E-34	postive
IGHD1-1	LINC01781	0.549972575	5.02E-41	postive
IGHD6-25	LINC01781	0.575539033	1.28E-45	postive
IGHG1	LINC01781	0.50512908	7.29E-34	postive
IGHG2	LINC01781	0.519360176	5.07E-36	postive
IGHG3	LINC01781	0.524985157	6.65E-37	postive
IGHJ2	LINC01781	0.508711755	2.13E-34	postive
IGHJ3	LINC01781	0.551385472	2.86E-41	postive
IGHV4-28	LINC01781	0.528343306	1.94E-37	postive
IGHV4-34	LINC01781	0.534549208	1.93E-38	postive
IGKC	LINC01781	0.583181548	4.51E-47	postive
IGKJ5	LINC01781	0.630863675	4.49E-57	postive
IGKV1-5	LINC01781	0.511328069	8.61E-35	postive
IGKV1D-8	LINC01781	0.506173987	5.10E-34	postive
IGKV3-11	LINC01781	0.547345446	1.41E-40	postive
IGKV3-15	LINC01781	0.505964728	5.48E-34	postive
IGKV3-20	LINC01781	0.545137273	3.35E-40	postive
IGKV3-7	LINC01781	0.502524006	1.77E-33	postive
IGKV3D-11	LINC01781	0.522107109	1.89E-36	postive
IGKV3D-15	LINC01781	0.501379383	2.60E-33	postive
IGLC2	LINC01781	0.572490617	4.74E-45	postive
IGLJ1	LINC01781	0.652856498	2.64E-62	postive
IGLJ3	LINC01781	0.558065559	1.95E-42	postive
IGLV1-40	LINC01781	0.544723381	3.94E-40	postive
IGLV1-44	LINC01781	0.556561369	3.59E-42	postive
ACKR1	LINC01781	0.635516663	3.81E-58	postive
GMFG	LINC01781	0.652345905	3.54E-62	postive
IL16	LINC01781	0.763849868	3.81E-97	postive
IL10RA	LINC01781	0.647395783	5.77E-61	postive
IL12RB1	LINC01781	0.577481987	5.51E-46	postive
S1PR1	LINC01781	0.607012483	7.36E-52	postive
TIE1	LINC01781	0.509597107	1.57E-34	postive
TNFRSF13C	LINC01781	0.618958798	2.05E-54	postive
TNFRSF17	LINC01781	0.602197089	7.36E-51	postive
TNFRSF1B	LINC01781	0.506985472	3.86E-34	postive
ICAM2	LINC01781	0.538443751	4.42E-39	postive
ITGAL	LINC01781	0.660173264	3.84E-64	postive
LCK	LINC01781	0.568648653	2.43E-44	postive

NCR3	LINC01781	0.784131316	1.15E-105	postive
CD247	LINC01781	0.638292214	8.58E-59	postive
ZAP70	LINC01781	0.636541486	2.20E-58	postive
HCST	LINC01781	0.505010625	7.59E-34	postive
CD48	LINC01781	0.524909917	6.84E-37	postive
SH2D1A	LINC01781	0.5156769	1.87E-35	postive
CD3D	LINC01781	0.572150323	5.49E-45	postive
CD3E	LINC01781	0.65241264	3.41E-62	postive
PTPRC	LINC01781	0.66277673	8.27E-65	postive
ITK	LINC01781	0.730279926	8.82E-85	postive
GRAP2	LINC01781	0.584899564	2.10E-47	postive
CD28	LINC01781	0.718486319	7.18E-81	postive
ICOS	LINC01781	0.521314556	2.51E-36	postive
CTLA4	LINC01781	0.555343428	5.87E-42	postive
TRAC	LINC01781	0.568364851	2.73E-44	postive
TRAV2	LINC01781	0.501449607	2.54E-33	postive
TRAV8-3	LINC01781	0.592813742	5.85E-49	postive
TRAV8-6	LINC01781	0.528646695	1.74E-37	postive
TRAV9-2	LINC01781	0.576981599	6.85E-46	postive
TRBC1	LINC01781	0.657951983	1.40E-63	postive
TRBC2	LINC01781	0.685045436	8.52E-71	postive
TRBJ2-1	LINC01781	0.61616445	8.30E-54	postive
TRBJ2-2	LINC01781	0.627051939	3.28E-56	postive
TRBJ2-3	LINC01781	0.603977352	3.16E-51	postive
TRBJ2-7	LINC01781	0.66411576	3.73E-65	postive
TRBV2	LINC01781	0.601095296	1.24E-50	postive
TRBV3-1	LINC01781	0.586852927	8.76E-48	postive
TRBV5-1	LINC01781	0.6583014	1.15E-63	postive
TRBV5-4	LINC01781	0.604120337	2.95E-51	postive
TRBV5-6	LINC01781	0.535626256	1.29E-38	postive
TRBV6-1	LINC01781	0.547619638	1.27E-40	postive
TRBV6-5	LINC01781	0.579775425	2.03E-46	postive
TRBV18	LINC01781	0.650504121	1.01E-61	postive
TRBV19	LINC01781	0.569900121	1.43E-44	postive
TRBV20-1	LINC01781	0.582148257	7.13E-47	postive
TRBV28	LINC01781	0.685780716	5.29E-71	postive
TRBV29-1	LINC01781	0.627342065	2.82E-56	postive
DDX17	AC095057.3	0.528356785	1.94E-37	postive
GNRH1	AC095057.3	0.517857607	8.66E-36	postive
GNRH1	AC009065.9	0.518280582	7.45E-36	postive
DDX17	AL022328.3	0.628008734	2.00E-56	postive
GNRH1	AL022328.3	0.535019594	1.62E-38	postive
GNRH1	AC005899.7	0.527601261	2.56E-37	postive
IL17RE	AL035446.1	0.510244885	1.25E-34	postive
DDX17	INE1	0.521922612	2.02E-36	postive
PLXNB1	INE1	0.531818319	5.37E-38	postive
GNRH1	INE1	0.55664818	3.46E-42	postive
TNFRSF25	INE1	0.503151869	1.43E-33	postive
PLCG1	INE1	0.514736939	2.61E-35	postive
CALCA	LINC00261	0.72864057	3.17E-84	postive
THPO	LINC00261	0.558664192	1.53E-42	postive
NR0B2	LINC00261	0.558095185	1.92E-42	postive
TMSB4Y	AC011297.1	0.56397865	1.71E-43	postive
TYMP	U62317.1	0.538762009	3.92E-39	postive
DEFA6	LINC00920	0.842565386	1.81E-136	postive
SERPIND1	LINC00920	0.766597657	3.00E-98	postive

MAPK14	LINC00920	0.573332083	3.31E-45	postive
BMP4	LINC00920	0.636398164	2.38E-58	postive
CHGB	LINC00920	0.718284202	8.35E-81	postive
FGF3	LINC00920	0.534082423	2.30E-38	postive
TNFRSF19	LINC00920	0.589564446	2.58E-48	postive
PCSK1	AC090152.1	0.540676041	1.88E-39	postive
NR0B2	AC090152.1	0.58291934	5.07E-47	postive
GNRH1	AC103691.1	0.580254147	1.64E-46	postive
OGFR	AC103691.1	0.506260906	4.95E-34	postive
TNFRSF25	AC103691.1	0.501533383	2.47E-33	postive
HLA-DOB	AL662844.4	0.524073393	9.27E-37	postive
LTA	AL662844.4	0.564662283	1.29E-43	postive
CETP	AL662844.4	0.594818997	2.32E-49	postive
CD40LG	AL662844.4	0.615504598	1.15E-53	postive
PDCD1	AL662844.4	0.534870232	1.71E-38	postive
CCL21	AL662844.4	0.599533042	2.59E-50	postive
CD79A	AL662844.4	0.551431072	2.81E-41	postive
CD79B	AL662844.4	0.664362357	3.22E-65	postive
BTK	AL662844.4	0.522907499	1.41E-36	postive
CD19	AL662844.4	0.672171389	2.86E-67	postive
CR2	AL662844.4	0.642922577	6.88E-60	postive
INPP5D	AL662844.4	0.574411744	2.08E-45	postive
CD22	AL662844.4	0.658917225	8.01E-64	postive
CD72	AL662844.4	0.653107062	2.29E-62	postive
PTPN6	AL662844.4	0.521140755	2.67E-36	postive
RASGRP3	AL662844.4	0.53301256	3.44E-38	postive
PLCG2	AL662844.4	0.51362135	3.87E-35	postive
PRKCB	AL662844.4	0.551011462	3.32E-41	postive
IGLJ1	AL662844.4	0.608482775	3.62E-52	postive
IL16	AL662844.4	0.559913332	9.16E-43	postive
LTB	AL662844.4	0.502698115	1.66E-33	postive
TNFRSF13C	AL662844.4	0.69313441	4.17E-73	postive
ITGAL	AL662844.4	0.568427001	2.66E-44	postive
LCK	AL662844.4	0.51500455	2.38E-35	postive
NCR3	AL662844.4	0.597638754	6.28E-50	postive
ZAP70	AL662844.4	0.624059299	1.53E-55	postive
GRAP2	AL662844.4	0.522880198	1.43E-36	postive
TRBC1	AL662844.4	0.541613872	1.31E-39	postive
TRBC2	AL662844.4	0.516407562	1.45E-35	postive
TRBJ2-1	AL662844.4	0.525423414	5.67E-37	postive
TRBJ2-2	AL662844.4	0.588618599	3.95E-48	postive
TRBJ2-3	AL662844.4	0.534536284	1.94E-38	postive
TRBJ2-7	AL662844.4	0.62435601	1.32E-55	postive
TRBV28	AL662844.4	0.530587322	8.48E-38	postive
DDX17	AC005104.1	0.558984948	1.34E-42	postive
PLXNB1	AC005104.1	0.518186702	7.70E-36	postive
GNRH1	AC005104.1	0.654267354	1.18E-62	postive
TNFRSF25	AC005104.1	0.529001715	1.53E-37	postive
CMTM8	AC097639.1	0.719545669	3.26E-81	postive
FGFR3	AC019205.1	0.551493796	2.74E-41	postive
IFI30	AL117336.1	0.633885436	9.09E-58	postive
B2M	PSMB8-AS1	0.665027483	2.17E-65	postive
CD8A	PSMB8-AS1	0.565014492	1.11E-43	postive
CD74	PSMB8-AS1	0.608128043	4.30E-52	postive
CTSS	PSMB8-AS1	0.530115564	1.01E-37	postive
HLA-A	PSMB8-AS1	0.688194394	1.10E-71	postive

HLA-B	PSMB8-AS1	0.72604979	2.35E-83	postive
HLA-C	PSMB8-AS1	0.706088906	5.71E-77	postive
HLA-DMA	PSMB8-AS1	0.697915279	1.65E-74	postive
HLA-DMB	PSMB8-AS1	0.671987251	3.20E-67	postive
HLA-DOA	PSMB8-AS1	0.547600994	1.28E-40	postive
HLA-DOB	PSMB8-AS1	0.68356141	2.22E-70	postive
HLA-DPA1	PSMB8-AS1	0.556944095	3.07E-42	postive
HLA-DPB1	PSMB8-AS1	0.611518572	8.24E-53	postive
HLA-DQB1	PSMB8-AS1	0.546995498	1.62E-40	postive
HLA-DRA	PSMB8-AS1	0.599918549	2.16E-50	postive
HLA-DRB1	PSMB8-AS1	0.577958118	4.48E-46	postive
HLA-DRB5	PSMB8-AS1	0.516744512	1.29E-35	postive
HLA-E	PSMB8-AS1	0.72589588	2.65E-83	postive
HLA-F	PSMB8-AS1	0.742802591	3.63E-89	postive
HLA-H	PSMB8-AS1	0.600433913	1.69E-50	postive
LTA	PSMB8-AS1	0.565501135	9.09E-44	postive
CIITA	PSMB8-AS1	0.51963595	4.59E-36	postive
PSMB8	PSMB8-AS1	0.715096834	8.78E-80	postive
PSME1	PSMB8-AS1	0.505268937	6.95E-34	postive
PSME2	PSMB8-AS1	0.511457609	8.23E-35	postive
TAP1	PSMB8-AS1	0.639299581	4.97E-59	postive
TAP2	PSMB8-AS1	0.613572274	3.00E-53	postive
IFI30	PSMB8-AS1	0.551469865	2.77E-41	postive
ISG20	PSMB8-AS1	0.632090702	2.35E-57	postive
IRF1	PSMB8-AS1	0.615629204	1.08E-53	postive
CYLD	PSMB8-AS1	0.523162477	1.29E-36	postive
CCL4	PSMB8-AS1	0.534755737	1.79E-38	postive
CD40LG	PSMB8-AS1	0.64903537	2.30E-61	postive
TRIM22	PSMB8-AS1	0.669733767	1.27E-66	postive
PDCD1	PSMB8-AS1	0.68925675	5.46E-72	postive
FASLG	PSMB8-AS1	0.534186116	2.21E-38	postive
GBP2	PSMB8-AS1	0.516213344	1.55E-35	postive
CCR5	PSMB8-AS1	0.518916254	5.94E-36	postive
CXCR4	PSMB8-AS1	0.568823548	2.25E-44	postive
CXCR6	PSMB8-AS1	0.564526627	1.36E-43	postive
CCR4	PSMB8-AS1	0.542350146	9.89E-40	postive
CD79A	PSMB8-AS1	0.591103653	1.28E-48	postive
CD79B	PSMB8-AS1	0.532002207	5.01E-38	postive
BTK	PSMB8-AS1	0.674297569	7.69E-68	postive
VAV1	PSMB8-AS1	0.587171183	7.59E-48	postive
RAC2	PSMB8-AS1	0.511500736	8.11E-35	postive
CD19	PSMB8-AS1	0.525409486	5.70E-37	postive
INPP5D	PSMB8-AS1	0.616139567	8.40E-54	postive
CD72	PSMB8-AS1	0.606036353	1.18E-51	postive
PTPN6	PSMB8-AS1	0.554295554	8.95E-42	postive
FCGR2B	PSMB8-AS1	0.536708241	8.55E-39	postive
RASGRP3	PSMB8-AS1	0.574173178	2.31E-45	postive
PLCG2	PSMB8-AS1	0.50028553	3.75E-33	postive
PRKCB	PSMB8-AS1	0.585799463	1.40E-47	postive
IGKJ5	PSMB8-AS1	0.525150206	6.27E-37	postive
IGLJ1	PSMB8-AS1	0.554892499	7.04E-42	postive
CXCR3	PSMB8-AS1	0.5734061	3.21E-45	postive
FLT3LG	PSMB8-AS1	0.583257601	4.36E-47	postive
GMFG	PSMB8-AS1	0.697590823	2.06E-74	postive
IL16	PSMB8-AS1	0.651901713	4.55E-62	postive
TNFSF12	PSMB8-AS1	0.515768395	1.82E-35	postive

TNFSF13B	PSMB8-AS1	0.506823492	4.08E-34	postive
IL10RA	PSMB8-AS1	0.643210328	5.88E-60	postive
IL12RB1	PSMB8-AS1	0.689488839	4.69E-72	postive
IL15RA	PSMB8-AS1	0.540165162	2.29E-39	postive
IL18RAP	PSMB8-AS1	0.588046086	5.12E-48	postive
IL2RG	PSMB8-AS1	0.520055592	3.95E-36	postive
TNFRSF14	PSMB8-AS1	0.581139258	1.11E-46	postive
TNFRSF17	PSMB8-AS1	0.564635603	1.30E-43	postive
TNFRSF1B	PSMB8-AS1	0.551761087	2.47E-41	postive
ICAM2	PSMB8-AS1	0.52441164	8.20E-37	postive
ITGAL	PSMB8-AS1	0.664275146	3.40E-65	postive
LCK	PSMB8-AS1	0.627869231	2.15E-56	postive
NCR3	PSMB8-AS1	0.629959166	7.21E-57	postive
CD247	PSMB8-AS1	0.707061534	2.87E-77	postive
ZAP70	PSMB8-AS1	0.634779738	5.65E-58	postive
LCP2	PSMB8-AS1	0.563387852	2.19E-43	postive
HCST	PSMB8-AS1	0.610131214	1.62E-52	postive
CD48	PSMB8-AS1	0.576693924	7.76E-46	postive
SH2D1A	PSMB8-AS1	0.612000236	6.50E-53	postive
PRF1	PSMB8-AS1	0.507798327	2.92E-34	postive
CD3D	PSMB8-AS1	0.696415811	4.58E-74	postive
CD3E	PSMB8-AS1	0.711115208	1.59E-78	postive
CD3G	PSMB8-AS1	0.547514451	1.32E-40	postive
PTPRC	PSMB8-AS1	0.663220502	6.36E-65	postive
ITK	PSMB8-AS1	0.667745339	4.23E-66	postive
GRAP2	PSMB8-AS1	0.651994281	4.32E-62	postive
CD28	PSMB8-AS1	0.614022147	2.40E-53	postive
ICOS	PSMB8-AS1	0.640325439	2.85E-59	postive
CTLA4	PSMB8-AS1	0.650295017	1.13E-61	postive
TRAC	PSMB8-AS1	0.645599632	1.57E-60	postive
TRAV2	PSMB8-AS1	0.568120289	3.03E-44	postive
TRAV4	PSMB8-AS1	0.570664505	1.03E-44	postive
TRAV8-3	PSMB8-AS1	0.651368563	6.16E-62	postive
TRAV8-6	PSMB8-AS1	0.603404257	4.15E-51	postive
TRAV9-2	PSMB8-AS1	0.576882131	7.15E-46	postive
TRAV12-1	PSMB8-AS1	0.525102194	6.38E-37	postive
TRAV12-2	PSMB8-AS1	0.504817299	8.11E-34	postive
TRAV12-3	PSMB8-AS1	0.556133808	4.26E-42	postive
TRAV13-1	PSMB8-AS1	0.540822322	1.78E-39	postive
TRBC1	PSMB8-AS1	0.680121188	2.00E-69	postive
TRBC2	PSMB8-AS1	0.737539591	2.71E-87	postive
TRBJ2-1	PSMB8-AS1	0.627788609	2.24E-56	postive
TRBJ2-2	PSMB8-AS1	0.62753482	2.55E-56	postive
TRBJ2-3	PSMB8-AS1	0.607321281	6.34E-52	postive
TRBJ2-7	PSMB8-AS1	0.657156662	2.23E-63	postive
TRBV2	PSMB8-AS1	0.600219457	1.87E-50	postive
TRBV3-1	PSMB8-AS1	0.580104699	1.75E-46	postive
TRBV5-1	PSMB8-AS1	0.639634404	4.15E-59	postive
TRBV5-4	PSMB8-AS1	0.544083088	5.05E-40	postive
TRBV5-6	PSMB8-AS1	0.565849431	7.86E-44	postive
TRBV6-1	PSMB8-AS1	0.587761822	5.82E-48	postive
TRBV6-5	PSMB8-AS1	0.638472762	7.78E-59	postive
TRBV7-9	PSMB8-AS1	0.56654214	5.88E-44	postive
TRBV10-3	PSMB8-AS1	0.505914576	5.58E-34	postive
TRBV18	PSMB8-AS1	0.637487757	1.32E-58	postive
TRBV19	PSMB8-AS1	0.590517108	1.67E-48	postive

TRBV20-1	PSMB8-AS1	0.578963161	2.89E-46	postive
TRBV28	PSMB8-AS1	0.707268304	2.48E-77	postive
TRBV29-1	PSMB8-AS1	0.611462855	8.47E-53	postive
GNRH1	AC011481.1	0.515725916	1.84E-35	postive
BMP8B	AL603839.3	0.515559529	1.95E-35	postive
CD1C	AL731567.1	0.517508324	9.80E-36	postive
CD74	AL731567.1	0.525831402	4.89E-37	postive
HLA-DMA	AL731567.1	0.573467676	3.12E-45	postive
HLA-DOA	AL731567.1	0.50453946	8.91E-34	postive
HLA-DOB	AL731567.1	0.566976945	4.90E-44	postive
HLA-DPB1	AL731567.1	0.520064572	3.94E-36	postive
HLA-DQA2	AL731567.1	0.501560344	2.44E-33	postive
LTA	AL731567.1	0.54548411	2.93E-40	postive
CIITA	AL731567.1	0.586433347	1.06E-47	postive
IFI30	AL731567.1	0.655236929	6.76E-63	postive
RBP5	AL731567.1	0.542444192	9.53E-40	postive
CETP	AL731567.1	0.543341629	6.74E-40	postive
SLC11A1	AL731567.1	0.547813254	1.18E-40	postive
CD40LG	AL731567.1	0.647269578	6.19E-61	postive
TRIM22	AL731567.1	0.534168893	2.23E-38	postive
PDCD1	AL731567.1	0.568618423	2.46E-44	postive
CCL21	AL731567.1	0.528544648	1.81E-37	postive
CXCR4	AL731567.1	0.537872948	5.49E-39	postive
FGR	AL731567.1	0.581310024	1.03E-46	postive
CD79B	AL731567.1	0.545623061	2.78E-40	postive
BTK	AL731567.1	0.676040874	2.60E-68	postive
VAV1	AL731567.1	0.584105043	2.99E-47	postive
RAC2	AL731567.1	0.50210896	2.03E-33	postive
CD19	AL731567.1	0.564514506	1.37E-43	postive
CR2	AL731567.1	0.512288751	6.16E-35	postive
PIK3R5	AL731567.1	0.602311731	6.97E-51	postive
PIK3CD	AL731567.1	0.516278889	1.52E-35	postive
INPP5D	AL731567.1	0.676973635	1.45E-68	postive
CD22	AL731567.1	0.529055944	1.50E-37	postive
CD72	AL731567.1	0.611650828	7.72E-53	postive
PTPN6	AL731567.1	0.586892591	8.60E-48	postive
LILRB3	AL731567.1	0.544789743	3.84E-40	postive
RASGRP3	AL731567.1	0.64849505	3.12E-61	postive
PLCG2	AL731567.1	0.534097436	2.29E-38	postive
PRKCB	AL731567.1	0.606891537	7.81E-52	postive
IGLJ1	AL731567.1	0.57829249	3.87E-46	postive
FLT3LG	AL731567.1	0.532912875	3.57E-38	postive
GMFG	AL731567.1	0.562731711	2.87E-43	postive
IL16	AL731567.1	0.610202245	1.57E-52	postive
LTB	AL731567.1	0.534843408	1.73E-38	postive
TNFSF14	AL731567.1	0.532642658	3.95E-38	postive
FLT4	AL731567.1	0.500654635	3.32E-33	postive
IL10RA	AL731567.1	0.602583892	6.13E-51	postive
IL12RB1	AL731567.1	0.605711563	1.38E-51	postive
IL21R	AL731567.1	0.532291402	4.50E-38	postive
IL3RA	AL731567.1	0.63269423	1.71E-57	postive
TNFRSF13C	AL731567.1	0.516996612	1.18E-35	postive
TNFRSF14	AL731567.1	0.621652616	5.24E-55	postive
TNFRSF1B	AL731567.1	0.544569537	4.18E-40	postive
ITGAL	AL731567.1	0.657431701	1.90E-63	postive
LCK	AL731567.1	0.537778328	5.70E-39	postive

NCR3	AL731567.1	0.579560198	2.23E-46	postive
CD247	AL731567.1	0.598804805	3.64E-50	postive
ZAP70	AL731567.1	0.672016061	3.14E-67	postive
CD3E	AL731567.1	0.566626428	5.68E-44	postive
PTPRC	AL731567.1	0.590286741	1.86E-48	postive
ITK	AL731567.1	0.603440962	4.08E-51	postive
GRAP2	AL731567.1	0.613721318	2.79E-53	postive
CD28	AL731567.1	0.556002	4.50E-42	postive
ICOS	AL731567.1	0.546950966	1.65E-40	postive
CTLA4	AL731567.1	0.544788222	3.84E-40	postive
TRAV8-3	AL731567.1	0.552434991	1.89E-41	postive
TRBC1	AL731567.1	0.63574774	3.37E-58	postive
TRBC2	AL731567.1	0.576964172	6.90E-46	postive
TRBJ2-1	AL731567.1	0.571973547	5.92E-45	postive
TRBJ2-2	AL731567.1	0.585171913	1.86E-47	postive
TRBJ2-3	AL731567.1	0.55773165	2.23E-42	postive
TRBJ2-7	AL731567.1	0.670663449	7.19E-67	postive
TRBV2	AL731567.1	0.504661651	8.55E-34	postive
TRBV5-1	AL731567.1	0.502630795	1.70E-33	postive
TRBV18	AL731567.1	0.510466246	1.16E-34	postive
TRBV28	AL731567.1	0.604179817	2.87E-51	postive
PAK5	SOX2-OT	0.621757598	4.97E-55	postive
DDX17	AL139287.1	0.540143437	2.31E-39	postive
PLXNB1	AL139287.1	0.548243754	9.93E-41	postive
GNRH1	AL139287.1	0.535252679	1.48E-38	postive
TNFRSF25	AL139287.1	0.58502793	1.98E-47	postive
PLCG1	AL139287.1	0.527864725	2.32E-37	postive
CCL8	AC018926.2	0.558091695	1.93E-42	postive
CCL3	AC018926.2	0.517533304	9.71E-36	postive
NR2C1	AC079684.2	0.520770508	3.06E-36	postive
DDX17	AL021707.8	0.674507158	6.75E-68	postive
GNRH1	AL021707.8	0.58022408	1.66E-46	postive
GNRH1	AC007038.1	0.665631343	1.51E-65	postive
NR2C1	AC007038.1	0.500659619	3.31E-33	postive
TNFRSF25	AC007038.1	0.594829648	2.31E-49	postive
DDX17	AL031186.1	0.504169097	1.01E-33	postive
PLXNB1	AL031186.1	0.514190625	3.16E-35	postive
PAEP	AL049870.3	0.545994711	2.40E-40	postive
B2M	LINC01094	0.602410718	6.65E-51	postive
CD4	LINC01094	0.560831608	6.29E-43	postive
CTSS	LINC01094	0.599614027	2.49E-50	postive
FCER1G	LINC01094	0.639687475	4.03E-59	postive
HLA-DMB	LINC01094	0.635839791	3.21E-58	postive
HLA-DPA1	LINC01094	0.551361011	2.89E-41	postive
HLA-DQA1	LINC01094	0.520940671	2.87E-36	postive
HLA-DQB1	LINC01094	0.540462602	2.04E-39	postive
HLA-DRA	LINC01094	0.627288259	2.90E-56	postive
HLA-DRB1	LINC01094	0.622711168	3.06E-55	postive
HLA-DRB5	LINC01094	0.52345897	1.16E-36	postive
CCL8	LINC01094	0.635443804	3.96E-58	postive
TLR4	LINC01094	0.546178676	2.23E-40	postive
CYBB	LINC01094	0.663350316	5.89E-65	postive
TLR7	LINC01094	0.681017624	1.13E-69	postive
TLR8	LINC01094	0.629570786	8.84E-57	postive
MSR1	LINC01094	0.742329883	5.36E-89	postive
CD14	LINC01094	0.712861935	4.48E-79	postive

AQP9	LINC01094	0.568894778	2.19E-44	postive
CCL3	LINC01094	0.537562091	6.18E-39	postive
CCR1	LINC01094	0.690434604	2.51E-72	postive
IL10	LINC01094	0.659295735	6.42E-64	postive
CD86	LINC01094	0.749994843	8.37E-92	postive
RNASE2	LINC01094	0.62179596	4.87E-55	postive
FCGR2B	LINC01094	0.662830133	8.01E-65	postive
C5AR1	LINC01094	0.519929108	4.13E-36	postive
CCRL2	LINC01094	0.591675551	9.85E-49	postive
CMKLR1	LINC01094	0.599105956	3.16E-50	postive
FPR1	LINC01094	0.676278387	2.24E-68	postive
FPR2	LINC01094	0.564261196	1.52E-43	postive
PLXNC1	LINC01094	0.576969365	6.89E-46	postive
GMFG	LINC01094	0.529504802	1.27E-37	postive
OSM	LINC01094	0.54110651	1.60E-39	postive
TNFSF13B	LINC01094	0.705923125	6.42E-77	postive
C3AR1	LINC01094	0.751258566	2.82E-92	postive
CSF1R	LINC01094	0.617340173	4.61E-54	postive
CSF2RA	LINC01094	0.5260549	4.50E-37	postive
TYROBP	LINC01094	0.711204785	1.49E-78	postive
FCGR3A	LINC01094	0.769819899	1.46E-99	postive
LCP2	LINC01094	0.613338148	3.37E-53	postive
HCST	LINC01094	0.534251809	2.16E-38	postive
RHOA	LINC01094	0.550710609	3.75E-41	postive
TNFRSF10A	AC100861.1	0.664996015	2.21E-65	postive
TNFRSF10B	AC100861.1	0.50011869	3.97E-33	postive
KRAS	RASSF8-AS1	0.568201126	2.93E-44	postive
PSMD10	AL035425.4	0.537422527	6.52E-39	postive
DDX17	AL354733.3	0.606645357	8.79E-52	postive
PLXNB1	AL354733.3	0.502334918	1.88E-33	postive
GNRH1	AL354733.3	0.502648871	1.69E-33	postive
NR2C2	AL354733.3	0.50715269	3.65E-34	postive
DDX17	AC009022.1	0.527173102	2.99E-37	postive
GNRH1	AC009022.1	0.606484125	9.50E-52	postive
TNFRSF25	AC009022.1	0.517463929	9.96E-36	postive
DDX17	AP000442.1	0.519290183	5.19E-36	postive
GNRH1	AP000442.1	0.566624604	5.68E-44	postive
DDX17	AC010834.3	0.562799151	2.79E-43	postive
NR2C2	AC010834.3	0.572548354	4.63E-45	postive
PLCG1	AC010834.3	0.521632597	2.24E-36	postive
DDX17	AC004148.1	0.542181841	1.05E-39	postive
PLXNB1	AC004148.1	0.583294863	4.29E-47	postive
GNRH1	AC004148.1	0.616853214	5.88E-54	postive
NR2C1	AC004148.1	0.558448121	1.67E-42	postive
TNFRSF25	AC004148.1	0.532013043	4.99E-38	postive
PLCG1	AC004148.1	0.504651635	8.58E-34	postive
GNRH1	AC004253.1	0.557960951	2.03E-42	postive
TNFRSF25	AC004253.1	0.504407484	9.32E-34	postive
LTA	AL844908.1	0.54559379	2.81E-40	postive
CETP	AL844908.1	0.524727033	7.31E-37	postive
CD40LG	AL844908.1	0.544628015	4.09E-40	postive
CCL21	AL844908.1	0.57960108	2.19E-46	postive
CD79B	AL844908.1	0.600495053	1.65E-50	postive
CD19	AL844908.1	0.613465392	3.16E-53	postive
CR2	AL844908.1	0.591788813	9.36E-49	postive
INPP5D	AL844908.1	0.563297625	2.27E-43	postive

CD22	AL844908.1	0.611779786	7.25E-53	postive
CD72	AL844908.1	0.586260236	1.14E-47	postive
RASGRP3	AL844908.1	0.545211838	3.26E-40	postive
IGLJ1	AL844908.1	0.566707655	5.49E-44	postive
LTB	AL844908.1	0.531725727	5.56E-38	postive
TNFRSF13C	AL844908.1	0.602725002	5.73E-51	postive
ITGAL	AL844908.1	0.523982788	9.58E-37	postive
NCR3	AL844908.1	0.511015098	9.60E-35	postive
ZAP70	AL844908.1	0.57200236	5.84E-45	postive
TRBJ2-2	AL844908.1	0.542374468	9.79E-40	postive
TRBJ2-3	AL844908.1	0.527097277	3.07E-37	postive
TRBJ2-7	AL844908.1	0.551068386	3.25E-41	postive
TRBV28	AL844908.1	0.501332466	2.64E-33	postive
LMBR1L	CCDC18-AS1	0.559055911	1.30E-42	postive
DDX17	CCDC18-AS1	0.579357852	2.43E-46	postive
GNRH1	CCDC18-AS1	0.586242908	1.15E-47	postive
TNFRSF25	CCDC18-AS1	0.505691987	6.02E-34	postive
B2M	HCP5	0.662901001	7.69E-65	postive
HLA-A	HCP5	0.745445449	3.99E-90	postive
HLA-B	HCP5	0.741115088	1.46E-88	postive
HLA-C	HCP5	0.769272664	2.45E-99	postive
HLA-E	HCP5	0.596824035	9.18E-50	postive
HLA-F	HCP5	0.664278942	3.39E-65	postive
HLA-H	HCP5	0.604841895	2.09E-51	postive
PSMB8	HCP5	0.783801961	1.61E-105	postive
PSME1	HCP5	0.548575138	8.72E-41	postive
TAP1	HCP5	0.742735309	3.83E-89	postive
TAP2	HCP5	0.53378774	2.57E-38	postive
STAT1	HCP5	0.522662976	1.55E-36	postive
IRF1	HCP5	0.54747718	1.34E-40	postive
LMBR1L	AC024060.2	0.513617334	3.87E-35	postive
GNRH1	AC024060.2	0.720052918	2.23E-81	postive
NR2C1	AC024060.2	0.516866698	1.23E-35	postive
TNFRSF25	AC024060.2	0.579622308	2.17E-46	postive
LMBR1L	AL031600.1	0.511364629	8.50E-35	postive
DDX17	AL031600.1	0.545547417	2.86E-40	postive
IRF9	AL031600.1	0.504499822	9.03E-34	postive
GNRH1	AL031600.1	0.536952243	7.79E-39	postive
NR2C2	AL031600.1	0.503359049	1.33E-33	postive
GNRH1	AC011498.6	0.507704668	3.02E-34	postive
DDX17	AL021707.2	0.573681431	2.85E-45	postive
OSGIN1	AC092115.4	0.554321607	8.85E-42	postive
IFI30	AP002807.1	0.502949346	1.53E-33	postive
GNRH1	AP002807.1	0.594631097	2.54E-49	postive
OGFR	AP002807.1	0.568030105	3.15E-44	postive
TNFRSF14	AP002807.1	0.545893505	2.50E-40	postive
TNFRSF25	AP002807.1	0.564676856	1.28E-43	postive
CXCL2	SFTA1P	0.709458861	5.21E-78	postive
SFTPD	SFTA1P	0.736879503	4.63E-87	postive
A2M	SFTA1P	0.655620576	5.42E-63	postive
SFTPA1	SFTA1P	0.766083406	4.85E-98	postive
SFTPA2	SFTA1P	0.740725456	2.02E-88	postive
SLC11A1	SFTA1P	0.611555113	8.09E-53	postive
MARCO	SFTA1P	0.616511431	6.98E-54	postive
AGER	SFTA1P	0.856810117	6.15E-146	postive
CCL23	SFTA1P	0.699543381	5.43E-75	postive

FGR	SFTA1P	0.507310846	3.46E-34	postive
SEMA3G	SFTA1P	0.567427071	4.06E-44	postive
C5AR1	SFTA1P	0.524311831	8.50E-37	postive
CCRL2	SFTA1P	0.507222723	3.56E-34	postive
EDNRB	SFTA1P	0.57818208	4.06E-46	postive
AGRP	SFTA1P	0.660368675	3.42E-64	postive
VEGFD	SFTA1P	0.789082434	6.92E-108	postive
GDF10	SFTA1P	0.551565799	2.67E-41	postive
RETN	SFTA1P	0.675996965	2.67E-68	postive
SCGB3A1	SFTA1P	0.531175283	6.82E-38	postive
ACVRL1	SFTA1P	0.626076698	5.43E-56	postive
CSF3R	SFTA1P	0.539178529	3.34E-39	postive
ENG	SFTA1P	0.5180236	8.16E-36	postive
IL3RA	SFTA1P	0.574919094	1.67E-45	postive
NPR1	SFTA1P	0.675311445	4.10E-68	postive
S1PR1	SFTA1P	0.591810414	9.26E-49	postive
SCTR	SFTA1P	0.535079676	1.58E-38	postive
TEK	SFTA1P	0.618871477	2.14E-54	postive
TIE1	SFTA1P	0.574748043	1.80E-45	postive
TNFRSF14	PRKCZ-AS1	0.510829845	1.02E-34	postive
LMBR1L	AL139349.1	0.554161113	9.44E-42	postive
GNRH1	AL139349.1	0.58812885	4.93E-48	postive
OGFR	AL139349.1	0.512922973	4.94E-35	postive
TNFRSF25	AL139349.1	0.503229815	1.39E-33	postive
NRTN	AC107952.2	0.802300266	4.03E-114	postive
IFI30	AL021578.1	0.51711457	1.13E-35	postive
SDC4	AL021578.1	0.527998925	2.21E-37	postive
CD19	AF131215.6	0.508039069	2.69E-34	postive
TNFRSF13C	AF131215.6	0.521036662	2.78E-36	postive
ZAP70	AF131215.6	0.509239465	1.78E-34	postive
DDX17	LINC00641	0.525983023	4.62E-37	postive
GNRH1	LINC00641	0.528668257	1.73E-37	postive
AZGP1	LINC01138	0.611785084	7.23E-53	postive
PNOC	LINC01138	0.560479994	7.26E-43	postive
SYTL1	AL390719.2	0.524201441	8.85E-37	postive
PLXNB1	AL390719.2	0.549486773	6.08E-41	postive
GNRH1	AL390719.2	0.541381924	1.44E-39	postive
TNFRSF25	AL390719.2	0.582609691	5.81E-47	postive
LRSAM1	SLC25A25-AS1	0.501569526	2.44E-33	postive
CTSE	C8orf34-AS1	0.753409619	4.36E-93	postive
SFTPD	C8orf34-AS1	0.807850453	7.00E-117	postive
COLEC12	C8orf34-AS1	0.614157802	2.25E-53	postive
A2M	C8orf34-AS1	0.584410646	2.61E-47	postive
SFTPA1	C8orf34-AS1	0.638229103	8.87E-59	postive
SFTPA2	C8orf34-AS1	0.647123238	6.72E-61	postive
LTF	C8orf34-AS1	0.542924436	7.92E-40	postive
DMBT1	C8orf34-AS1	0.6507024	8.99E-62	postive
CCL23	C8orf34-AS1	0.548746603	8.15E-41	postive
C3	C8orf34-AS1	0.583196158	4.48E-47	postive
ACKR1	C8orf34-AS1	0.628272213	1.74E-56	postive
AGTR2	C8orf34-AS1	0.567482017	3.96E-44	postive
CSF3R	C8orf34-AS1	0.635920824	3.07E-58	postive
NPR1	C8orf34-AS1	0.620359233	1.01E-54	postive
S1PR1	C8orf34-AS1	0.593207095	4.88E-49	postive
SCTR	C8orf34-AS1	0.724097368	1.05E-82	postive
TEK	C8orf34-AS1	0.588542319	4.09E-48	postive

TIE1	C8orf34-AS1	0.621048869	7.12E-55	postive
B2M	CARD8-AS1	0.566155053	6.92E-44	postive
CTSS	CARD8-AS1	0.550444616	4.16E-41	postive
FCER1G	CARD8-AS1	0.556640349	3.47E-42	postive
FCGRT	CARD8-AS1	0.542369886	9.81E-40	postive
HLA-DMA	CARD8-AS1	0.527801244	2.37E-37	postive
HLA-DMB	CARD8-AS1	0.709774444	4.16E-78	postive
HLA-DRA	CARD8-AS1	0.578762028	3.16E-46	postive
HLA-DRB1	CARD8-AS1	0.502298142	1.91E-33	postive
TLR7	CARD8-AS1	0.515027545	2.36E-35	postive
TLR8	CARD8-AS1	0.518994707	5.77E-36	postive
TPT1	CARD8-AS1	0.505487005	6.45E-34	postive
MSR1	CARD8-AS1	0.55843251	1.68E-42	postive
CCL3	CARD8-AS1	0.507449833	3.29E-34	postive
CXCR6	CARD8-AS1	0.545996178	2.40E-40	postive
IL10	CARD8-AS1	0.611367449	8.87E-53	postive
CD86	CARD8-AS1	0.579447409	2.34E-46	postive
BTK	CARD8-AS1	0.621284136	6.32E-55	postive
CD72	CARD8-AS1	0.503350361	1.33E-33	postive
FCGR2B	CARD8-AS1	0.586503848	1.02E-47	postive
CCRL2	CARD8-AS1	0.567059185	4.73E-44	postive
FPR1	CARD8-AS1	0.581741301	8.53E-47	postive
GMFG	CARD8-AS1	0.755316381	8.19E-94	postive
IL16	CARD8-AS1	0.520456355	3.42E-36	postive
TNFSF13B	CARD8-AS1	0.575510643	1.30E-45	postive
IL10RA	CARD8-AS1	0.517073878	1.14E-35	postive
IL12RB1	CARD8-AS1	0.539457105	3.00E-39	postive
IL18RAP	CARD8-AS1	0.533590956	2.77E-38	postive
NR1H3	CARD8-AS1	0.501890908	2.19E-33	postive
ICAM2	CARD8-AS1	0.525590919	5.33E-37	postive
TYROBP	CARD8-AS1	0.629047018	1.16E-56	postive
LCK	CARD8-AS1	0.506586471	4.43E-34	postive
NCR3	CARD8-AS1	0.528867856	1.60E-37	postive
LCP2	CARD8-AS1	0.547922262	1.13E-40	postive
HCST	CARD8-AS1	0.596507438	1.06E-49	postive
CD48	CARD8-AS1	0.740381554	2.67E-88	postive
SH2D1A	CARD8-AS1	0.546379069	2.07E-40	postive
CD3D	CARD8-AS1	0.633716048	9.95E-58	postive
CD3E	CARD8-AS1	0.506971871	3.88E-34	postive
PTPRC	CARD8-AS1	0.591989118	8.54E-49	postive
CD28	CARD8-AS1	0.513456151	4.10E-35	postive
ICOS	CARD8-AS1	0.608313581	3.93E-52	postive
TRAV2	CARD8-AS1	0.529679733	1.19E-37	postive
TRAV4	CARD8-AS1	0.505498534	6.43E-34	postive
TRAV8-3	CARD8-AS1	0.501489948	2.50E-33	postive
TRAV9-2	CARD8-AS1	0.501887456	2.19E-33	postive
TRBC1	CARD8-AS1	0.501188512	2.77E-33	postive
TRBC2	CARD8-AS1	0.545922132	2.47E-40	postive
TRBV5-1	CARD8-AS1	0.536020918	1.11E-38	postive
TRBV20-1	CARD8-AS1	0.518122786	7.88E-36	postive
VTN	AL356215.1	0.525770446	5.00E-37	postive
CHGA	AL356215.1	0.694941939	1.24E-73	postive
GHRH	AL356215.1	0.700000369	3.97E-75	postive
SCG2	AL356215.1	0.697957398	1.61E-74	postive
TRH	AL356215.1	0.673561199	1.21E-67	postive
DDX17	TTC28-AS1	0.538377843	4.53E-39	postive

NRTN	NALT1	0.531928475	5.15E-38	postive
IFI30	LINC-PINT	0.579146144	2.67E-46	postive
GIPR	LINC-PINT	0.531929138	5.15E-38	postive
GNRH1	AC011472.1	0.504990242	7.64E-34	postive
MC1R	AC011472.1	0.517548501	9.66E-36	postive
DDX17	AC129510.1	0.530044053	1.04E-37	postive
PLXNB1	AC129510.1	0.585120524	1.90E-47	postive
GNRH1	AC129510.1	0.531145226	6.89E-38	postive
CD4	AL357033.4	0.5733512	3.28E-45	postive
CD74	AL357033.4	0.636240747	2.59E-58	postive
CTSS	AL357033.4	0.509177231	1.82E-34	postive
HLA-A	AL357033.4	0.503432244	1.30E-33	postive
HLA-DMA	AL357033.4	0.660342541	3.48E-64	postive
HLA-DMB	AL357033.4	0.54136265	1.45E-39	postive
HLA-DOA	AL357033.4	0.584096423	3.00E-47	postive
HLA-DOB	AL357033.4	0.574405377	2.09E-45	postive
HLA-DPA1	AL357033.4	0.551386403	2.86E-41	postive
HLA-DPB1	AL357033.4	0.624699417	1.10E-55	postive
HLA-E	AL357033.4	0.619441962	1.60E-54	postive
HLA-F	AL357033.4	0.525998494	4.60E-37	postive
CIITA	AL357033.4	0.502092958	2.04E-33	postive
ISG20	AL357033.4	0.514084171	3.29E-35	postive
CD40LG	AL357033.4	0.602032337	7.96E-51	postive
TRIM22	AL357033.4	0.565025423	1.11E-43	postive
PDCD1	AL357033.4	0.622770244	2.97E-55	postive
CCR5	AL357033.4	0.545165319	3.32E-40	postive
CCR4	AL357033.4	0.528321461	1.96E-37	postive
FGR	AL357033.4	0.501122392	2.83E-33	postive
CD79A	AL357033.4	0.559473762	1.10E-42	postive
BTK	AL357033.4	0.623239018	2.34E-55	postive
VAV1	AL357033.4	0.559516715	1.08E-42	postive
RAC2	AL357033.4	0.534008448	2.37E-38	postive
PIK3R5	AL357033.4	0.539763491	2.67E-39	postive
INPP5D	AL357033.4	0.622926225	2.74E-55	postive
RASGRP3	AL357033.4	0.593190456	4.92E-49	postive
PLCG2	AL357033.4	0.519155935	5.45E-36	postive
PRKCB	AL357033.4	0.5766051	8.07E-46	postive
IGHJ3	AL357033.4	0.505130273	7.29E-34	postive
C3	AL357033.4	0.520158121	3.81E-36	postive
CXCR3	AL357033.4	0.569343331	1.81E-44	postive
ADA2	AL357033.4	0.541858062	1.20E-39	postive
GMFG	AL357033.4	0.548632256	8.52E-41	postive
IL16	AL357033.4	0.643738814	4.39E-60	postive
TNFSF12	AL357033.4	0.521587362	2.28E-36	postive
ENG	AL357033.4	0.534058672	2.32E-38	postive
IL10RA	AL357033.4	0.647582893	5.20E-61	postive
IL12RB1	AL357033.4	0.619412099	1.63E-54	postive
TIE1	AL357033.4	0.502115872	2.03E-33	postive
TNFRSF14	AL357033.4	0.531452541	6.15E-38	postive
TNFRSF17	AL357033.4	0.507544622	3.19E-34	postive
TNFRSF1B	AL357033.4	0.605397381	1.60E-51	postive
ICAM2	AL357033.4	0.529180477	1.43E-37	postive
ITGAL	AL357033.4	0.625614885	6.90E-56	postive
ITGB2	AL357033.4	0.519163384	5.43E-36	postive
LCK	AL357033.4	0.529641299	1.20E-37	postive
NCR3	AL357033.4	0.535802595	1.20E-38	postive

CD247	AL357033.4	0.628932047	1.23E-56	postive
ZAP70	AL357033.4	0.51958581	4.67E-36	postive
LCP2	AL357033.4	0.541642833	1.30E-39	postive
HCST	AL357033.4	0.523751209	1.04E-36	postive
CD3D	AL357033.4	0.530459874	8.89E-38	postive
CD3E	AL357033.4	0.623921389	1.65E-55	postive
PTPRC	AL357033.4	0.594799243	2.35E-49	postive
ITK	AL357033.4	0.604783994	2.15E-51	postive
GRAP2	AL357033.4	0.555760478	4.96E-42	postive
CD28	AL357033.4	0.564382656	1.45E-43	postive
ICOS	AL357033.4	0.537967087	5.30E-39	postive
TRAC	AL357033.4	0.558306472	1.77E-42	postive
TRAV8-3	AL357033.4	0.523358337	1.20E-36	postive
TRAV8-6	AL357033.4	0.513912499	3.49E-35	postive
TRAV13-1	AL357033.4	0.506531371	4.51E-34	postive
TRBC1	AL357033.4	0.577161369	6.34E-46	postive
TRBC2	AL357033.4	0.586719658	9.30E-48	postive
TRBJ2-7	AL357033.4	0.550978366	3.37E-41	postive
TRBV2	AL357033.4	0.503004322	1.50E-33	postive
TRBV5-1	AL357033.4	0.537646504	5.99E-39	postive
TRBV5-4	AL357033.4	0.520048879	3.96E-36	postive
TRBV5-6	AL357033.4	0.528660081	1.73E-37	postive
TRBV6-1	AL357033.4	0.512262726	6.22E-35	postive
TRBV6-5	AL357033.4	0.519464399	4.88E-36	postive
TRBV18	AL357033.4	0.535036331	1.61E-38	postive
TRBV19	AL357033.4	0.538839716	3.80E-39	postive
TRBV20-1	AL357033.4	0.504384335	9.40E-34	postive
TRBV28	AL357033.4	0.601064564	1.26E-50	postive
TRBV29-1	AL357033.4	0.536384282	9.66E-39	postive
GNRH1	AC005674.2	0.534248607	2.16E-38	postive
FABP7	AL022316.1	0.558596669	1.57E-42	postive
MAP3K14	AL022316.1	0.742654664	4.10E-89	postive
TRGJP2	AL022316.1	0.672046989	3.08E-67	postive
TRGC2	AL022316.1	0.658235343	1.19E-63	postive
FGFR1	AC069120.1	0.71468427	1.19E-79	postive
IRF3	AC008735.2	0.544258218	4.72E-40	postive
DDX17	AC008735.2	0.507086413	3.73E-34	postive
PLXNB1	AC008735.2	0.524420966	8.17E-37	postive
GNRH1	AC008735.2	0.516058048	1.64E-35	postive
GNRH1	Z69706.1	0.514693148	2.65E-35	postive
PLXNB1	AC037459.2	0.539000773	3.58E-39	postive
GNRH1	AC037459.2	0.703964712	2.54E-76	postive
NR2C2	AC037459.2	0.524546581	7.80E-37	postive
TNFRSF25	AC037459.2	0.518391623	7.16E-36	postive
HLA-DOB	LINC02362	0.562370875	3.33E-43	postive
CD79A	LINC02362	0.552998783	1.50E-41	postive
FCGR2B	LINC02362	0.555346392	5.86E-42	postive
IGHA1	LINC02362	0.747683655	6.02E-91	postive
IGHA2	LINC02362	0.724865953	5.84E-83	postive
IGHD	LINC02362	0.502221553	1.96E-33	postive
IGHD6-25	LINC02362	0.751248929	2.84E-92	postive
IGHG1	LINC02362	0.706073923	5.77E-77	postive
IGHG2	LINC02362	0.7617335	2.63E-96	postive
IGHG3	LINC02362	0.70673322	3.62E-77	postive
IGHG4	LINC02362	0.562207662	3.57E-43	postive
IGHJ2	LINC02362	0.60109712	1.24E-50	postive

IGHJ3	LINC02362	0.650797352	8.52E-62	postive
IGHV1-2	LINC02362	0.612895052	4.19E-53	postive
IGHV1-24	LINC02362	0.548267449	9.84E-41	postive
IGHV1-46	LINC02362	0.69624465	5.15E-74	postive
IGHV1-69	LINC02362	0.659615833	5.32E-64	postive
IGHV2-5	LINC02362	0.611000153	1.06E-52	postive
IGHV2-70	LINC02362	0.565833951	7.91E-44	postive
IGHV3-11	LINC02362	0.516244783	1.53E-35	postive
IGHV3-15	LINC02362	0.590308153	1.84E-48	postive
IGHV3-21	LINC02362	0.594171214	3.13E-49	postive
IGHV3-23	LINC02362	0.648799895	2.63E-61	postive
IGHV3-30	LINC02362	0.59749602	6.71E-50	postive
IGHV3-33	LINC02362	0.617535057	4.18E-54	postive
IGHV3-35	LINC02362	0.597199955	7.70E-50	postive
IGHV3-43	LINC02362	0.599937337	2.14E-50	postive
IGHV3-48	LINC02362	0.540617274	1.93E-39	postive
IGHV3-49	LINC02362	0.554737234	7.49E-42	postive
IGHV3-53	LINC02362	0.654103522	1.30E-62	postive
IGHV3-66	LINC02362	0.629771914	7.96E-57	postive
IGHV3-7	LINC02362	0.633602082	1.06E-57	postive
IGHV3-74	LINC02362	0.608395303	3.77E-52	postive
IGHV4-28	LINC02362	0.720694638	1.38E-81	postive
IGHV4-31	LINC02362	0.51048726	1.15E-34	postive
IGHV4-34	LINC02362	0.606071519	1.16E-51	postive
IGHV4-39	LINC02362	0.638071848	9.66E-59	postive
IGHV4-59	LINC02362	0.640408349	2.72E-59	postive
IGHV4-61	LINC02362	0.608250144	4.05E-52	postive
IGHV5-51	LINC02362	0.662811217	8.11E-65	postive
IGKC	LINC02362	0.823429772	3.87E-125	postive
IGKJ5	LINC02362	0.819380583	6.46E-123	postive
IGKV1-12	LINC02362	0.532120313	4.80E-38	postive
IGKV1-13	LINC02362	0.602309083	6.98E-51	postive
IGKV1-16	LINC02362	0.567765059	3.52E-44	postive
IGKV1-17	LINC02362	0.538035269	5.17E-39	postive
IGKV1-27	LINC02362	0.559455344	1.10E-42	postive
IGKV1-33	LINC02362	0.657051943	2.37E-63	postive
IGKV1-39	LINC02362	0.696013062	6.02E-74	postive
IGKV1-5	LINC02362	0.694848876	1.32E-73	postive
IGKV1-8	LINC02362	0.630030416	6.95E-57	postive
IGKV1-9	LINC02362	0.513939484	3.46E-35	postive
IGKV1D-13	LINC02362	0.505427268	6.59E-34	postive
IGKV1D-33	LINC02362	0.512516301	5.69E-35	postive
IGKV1D-39	LINC02362	0.698209369	1.35E-74	postive
IGKV1D-42	LINC02362	0.695719965	7.34E-74	postive
IGKV1D-8	LINC02362	0.727489576	7.75E-84	postive
IGKV2-28	LINC02362	0.53619902	1.04E-38	postive
IGKV3-11	LINC02362	0.729126837	2.17E-84	postive
IGKV3-15	LINC02362	0.771271725	3.68E-100	postive
IGKV3-20	LINC02362	0.755419293	7.48E-94	postive
IGKV3-7	LINC02362	0.711981269	8.49E-79	postive
IGKV3D-11	LINC02362	0.706341428	4.78E-77	postive
IGKV3D-15	LINC02362	0.658513957	1.01E-63	postive
IGKV3D-20	LINC02362	0.510259356	1.25E-34	postive
IGKV3D-7	LINC02362	0.625816225	6.22E-56	postive
IGKV4-1	LINC02362	0.658641877	9.40E-64	postive
IGLC2	LINC02362	0.809113793	1.60E-117	postive

IGLC3	LINC02362	0.696892951	3.32E-74	postive
IGLC6	LINC02362	0.618860434	2.15E-54	postive
IGLJ1	LINC02362	0.532707652	3.85E-38	postive
IGLJ2	LINC02362	0.697729135	1.88E-74	postive
IGLJ3	LINC02362	0.736343373	7.13E-87	postive
IGLV1-36	LINC02362	0.595918101	1.40E-49	postive
IGLV1-40	LINC02362	0.701069752	1.90E-75	postive
IGLV1-44	LINC02362	0.627234211	2.99E-56	postive
IGLV1-50	LINC02362	0.570749843	9.96E-45	postive
IGLV1-51	LINC02362	0.629882064	7.51E-57	postive
IGLV10-54	LINC02362	0.538773627	3.90E-39	postive
IGLV2-11	LINC02362	0.579433795	2.35E-46	postive
IGLV2-14	LINC02362	0.64512447	2.04E-60	postive
IGLV2-23	LINC02362	0.562669699	2.95E-43	postive
IGLV2-33	LINC02362	0.571883794	6.15E-45	postive
IGLV2-8	LINC02362	0.537692218	5.89E-39	postive
IGLV3-1	LINC02362	0.659099868	7.20E-64	postive
IGLV3-10	LINC02362	0.594308515	2.94E-49	postive
IGLV3-16	LINC02362	0.513618193	3.87E-35	postive
IGLV3-19	LINC02362	0.559369206	1.14E-42	postive
IGLV3-21	LINC02362	0.559969458	8.95E-43	postive
IGLV3-25	LINC02362	0.637723985	1.17E-58	postive
IGLV3-27	LINC02362	0.513463494	4.08E-35	postive
IGLV3-9	LINC02362	0.520532311	3.33E-36	postive
IGLV4-69	LINC02362	0.60108087	1.25E-50	postive
IGLV6-57	LINC02362	0.53680413	8.24E-39	postive
IGLV8-61	LINC02362	0.503115977	1.45E-33	postive
IL10RA	LINC02362	0.525876832	4.81E-37	postive
IL18RAP	LINC02362	0.511838278	7.21E-35	postive
TNFRSF17	LINC02362	0.82024054	2.20E-123	postive
TRBV6-1	LINC02362	0.53292112	3.56E-38	postive
DDX17	AL021707.6	0.657190576	2.19E-63	postive
PLXNB1	AL021707.6	0.534190234	2.21E-38	postive
GNRH1	AL021707.6	0.641432891	1.56E-59	postive
TNFRSF25	AL021707.6	0.603041008	4.93E-51	postive
PSMD2	KCNMB2-AS1	0.516819296	1.25E-35	postive
PIK3CA	KCNMB2-AS1	0.594345249	2.89E-49	postive
CD8A	LINC02195	0.711802263	9.66E-79	postive
FCER1G	LINC02195	0.575122728	1.53E-45	postive
IFNG	LINC02195	0.742601114	4.28E-89	postive
RBP4	LINC02195	0.627476062	2.63E-56	postive
ORM2	LINC02195	0.501856674	2.21E-33	postive
PGC	LINC02195	0.503840691	1.13E-33	postive
CCL5	LINC02195	0.511978148	6.87E-35	postive
APOM	LINC02195	0.707081969	2.83E-77	postive
PLA2G2A	LINC02195	0.710287475	2.88E-78	postive
CCL4	LINC02195	0.634079794	8.20E-58	postive
FASLG	LINC02195	0.555086192	6.51E-42	postive
APOH	LINC02195	0.668751715	2.30E-66	postive
CCL3	LINC02195	0.529404422	1.31E-37	postive
XCL2	LINC02195	0.825323926	3.38E-126	postive
CXCR6	LINC02195	0.676869673	1.55E-68	postive
SEMA4G	LINC02195	0.500477844	3.52E-33	postive
AGT	LINC02195	0.649868636	1.44E-61	postive
GMFG	LINC02195	0.534128263	2.26E-38	postive
IL18RAP	LINC02195	0.623991588	1.59E-55	postive

LEPR	LINC02195	0.510249266	1.25E-34	postive
NR1H3	LINC02195	0.519208123	5.35E-36	postive
HCST	LINC02195	0.538884179	3.74E-39	postive
CD48	LINC02195	0.662698532	8.66E-65	postive
CD244	LINC02195	0.679958581	2.21E-69	postive
SH2D1A	LINC02195	0.555421017	5.69E-42	postive
GZMB	LINC02195	0.760256407	1.00E-95	postive
PRF1	LINC02195	0.612402888	5.34E-53	postive
CD3D	LINC02195	0.713459339	2.90E-79	postive
TRAV4	LINC02195	0.536251487	1.02E-38	postive
TRAV12-2	LINC02195	0.54018771	2.27E-39	postive
TRBV7-9	LINC02195	0.533386381	2.99E-38	postive
TRBV9	LINC02195	0.753439363	4.25E-93	postive
TRBV12-4	LINC02195	0.665303399	1.84E-65	postive
TRDC	LINC02195	0.607849386	4.92E-52	postive
DDX17	LINC00893	0.565558929	8.87E-44	postive
PLXNA3	LINC00893	0.558200279	1.84E-42	postive
PLXNB1	LINC00893	0.520496329	3.37E-36	postive
GNRH1	LINC00893	0.547433211	1.37E-40	postive
AQP9	AC245128.3	0.534846154	1.73E-38	postive
FPR1	AC245128.3	0.556658348	3.45E-42	postive
FPR2	AC245128.3	0.534566957	1.92E-38	postive
MUC5AC	AL357093.2	0.601016774	1.29E-50	postive
BPIFB1	AL357093.2	0.737015928	4.14E-87	postive
BPIFA1	AL357093.2	0.590452036	1.72E-48	postive
DES	AL357093.2	0.510029156	1.35E-34	postive
SCGB3A1	AL357093.2	0.683947982	1.73E-70	postive
IRF9	AL512791.1	0.561075563	5.69E-43	postive
CD1C	AC018755.4	0.586500072	1.03E-47	postive
CD1D	AC018755.4	0.57871528	3.22E-46	postive
CD4	AC018755.4	0.635987485	2.96E-58	postive
CD74	AC018755.4	0.630327422	5.95E-57	postive
CTSS	AC018755.4	0.560603807	6.90E-43	postive
FCER1G	AC018755.4	0.524691156	7.41E-37	postive
HLA-DMA	AC018755.4	0.667048017	6.45E-66	postive
HLA-DMB	AC018755.4	0.655641029	5.36E-63	postive
HLA-DOA	AC018755.4	0.588626615	3.94E-48	postive
HLA-DOB	AC018755.4	0.566704173	5.49E-44	postive
HLA-DPA1	AC018755.4	0.557123226	2.86E-42	postive
HLA-DPB1	AC018755.4	0.651557364	5.54E-62	postive
HLA-DRA	AC018755.4	0.501082226	2.87E-33	postive
LTA	AC018755.4	0.630133502	6.58E-57	postive
CIITA	AC018755.4	0.542161803	1.06E-39	postive
IFI30	AC018755.4	0.63915686	5.37E-59	postive
PTGDS	AC018755.4	0.539293239	3.20E-39	postive
RBP5	AC018755.4	0.512784813	5.18E-35	postive
CETP	AC018755.4	0.627106665	3.19E-56	postive
CYBB	AC018755.4	0.554997928	6.75E-42	postive
TLR8	AC018755.4	0.51126219	8.81E-35	postive
IL7R	AC018755.4	0.514693069	2.65E-35	postive
CD40LG	AC018755.4	0.7197568	2.78E-81	postive
TRIM22	AC018755.4	0.558775162	1.46E-42	postive
PDCD1	AC018755.4	0.646730529	8.37E-61	postive
ANXA6	AC018755.4	0.504371874	9.44E-34	postive
CCL21	AC018755.4	0.586597865	9.82E-48	postive
CCR5	AC018755.4	0.551967883	2.27E-41	postive

CCR1	AC018755.4	0.507595983	3.13E-34	postive
CXCR4	AC018755.4	0.605212698	1.75E-51	postive
CCR4	AC018755.4	0.6464869	9.58E-61	postive
FGR	AC018755.4	0.659331327	6.29E-64	postive
CD86	AC018755.4	0.542070073	1.10E-39	postive
CD79A	AC018755.4	0.552037806	2.21E-41	postive
CD79B	AC018755.4	0.595163588	1.98E-49	postive
BTK	AC018755.4	0.758359141	5.51E-95	postive
VAV1	AC018755.4	0.565070621	1.09E-43	postive
RAC2	AC018755.4	0.543585582	6.13E-40	postive
NFATC2	AC018755.4	0.503136848	1.44E-33	postive
CD19	AC018755.4	0.58819134	4.79E-48	postive
CR2	AC018755.4	0.550181001	4.62E-41	postive
PIK3R5	AC018755.4	0.606011766	1.19E-51	postive
PIK3CD	AC018755.4	0.519686738	4.51E-36	postive
INPP5D	AC018755.4	0.688560186	8.63E-72	postive
CD22	AC018755.4	0.55765759	2.30E-42	postive
CD72	AC018755.4	0.711022483	1.70E-78	postive
PTPN6	AC018755.4	0.525331221	5.87E-37	postive
LILRB3	AC018755.4	0.616780159	6.10E-54	postive
FCGR2B	AC018755.4	0.525662187	5.20E-37	postive
RASGRP3	AC018755.4	0.684256543	1.42E-70	postive
PLCG2	AC018755.4	0.532508686	4.15E-38	postive
PRKCB	AC018755.4	0.70723144	2.55E-77	postive
IGLJ1	AC018755.4	0.503112412	1.45E-33	postive
C5AR1	AC018755.4	0.516297996	1.51E-35	postive
CCRL2	AC018755.4	0.563978913	1.71E-43	postive
CMKLR1	AC018755.4	0.54747414	1.34E-40	postive
CXCR3	AC018755.4	0.539218136	3.29E-39	postive
ACKR1	AC018755.4	0.54615205	2.26E-40	postive
PLXNC1	AC018755.4	0.547770128	1.20E-40	postive
ADA2	AC018755.4	0.535370168	1.42E-38	postive
GMFG	AC018755.4	0.71312262	3.71E-79	postive
IL16	AC018755.4	0.71114648	1.55E-78	postive
LTB	AC018755.4	0.556451424	3.75E-42	postive
TNFSF12	AC018755.4	0.532796782	3.72E-38	postive
TNFSF14	AC018755.4	0.523489835	1.15E-36	postive
ACVRL1	AC018755.4	0.510785307	1.04E-34	postive
CSF1R	AC018755.4	0.549695921	5.60E-41	postive
CSF3R	AC018755.4	0.524350887	8.38E-37	postive
IL10RA	AC018755.4	0.730109079	1.01E-84	postive
IL12RB1	AC018755.4	0.658107913	1.28E-63	postive
IL18RAP	AC018755.4	0.500005405	4.12E-33	postive
IL21R	AC018755.4	0.528564186	1.79E-37	postive
IL2RG	AC018755.4	0.570293316	1.21E-44	postive
IL3RA	AC018755.4	0.61200022	6.50E-53	postive
NR1H3	AC018755.4	0.537591836	6.11E-39	postive
S1PR1	AC018755.4	0.553822786	1.08E-41	postive
TIE1	AC018755.4	0.544860658	3.74E-40	postive
TNFRSF13C	AC018755.4	0.538878274	3.75E-39	postive
TNFRSF14	AC018755.4	0.536268358	1.01E-38	postive
TNFRSF1B	AC018755.4	0.713543095	2.73E-79	postive
ITGAL	AC018755.4	0.730069409	1.04E-84	postive
ITGB2	AC018755.4	0.57076502	9.90E-45	postive
TYROBP	AC018755.4	0.525319445	5.89E-37	postive
LCK	AC018755.4	0.611397039	8.74E-53	postive

NCR3	AC018755.4	0.714774529	1.11E-79	postive
CD247	AC018755.4	0.708372411	1.13E-77	postive
ZAP70	AC018755.4	0.655306592	6.49E-63	postive
LCP2	AC018755.4	0.614469888	1.92E-53	postive
HCST	AC018755.4	0.656930315	2.54E-63	postive
CD48	AC018755.4	0.575250102	1.45E-45	postive
SH2D1A	AC018755.4	0.552014992	2.23E-41	postive
CD3D	AC018755.4	0.638771753	6.62E-59	postive
CD3E	AC018755.4	0.70848076	1.05E-77	postive
CD3G	AC018755.4	0.520697049	3.14E-36	postive
PTPRC	AC018755.4	0.75346018	4.17E-93	postive
ITK	AC018755.4	0.710800499	1.99E-78	postive
GRAP2	AC018755.4	0.624350414	1.32E-55	postive
CD28	AC018755.4	0.711657508	1.07E-78	postive
ICOS	AC018755.4	0.623259545	2.31E-55	postive
CTLA4	AC018755.4	0.591161019	1.25E-48	postive
TRAC	AC018755.4	0.629390877	9.71E-57	postive
TRAV2	AC018755.4	0.53008664	1.02E-37	postive
TRAV4	AC018755.4	0.53889403	3.72E-39	postive
TRAV8-3	AC018755.4	0.638103191	9.50E-59	postive
TRAV8-6	AC018755.4	0.564932148	1.15E-43	postive
TRAV9-2	AC018755.4	0.591197882	1.23E-48	postive
TRAV12-1	AC018755.4	0.504771582	8.24E-34	postive
TRAV13-1	AC018755.4	0.526167266	4.32E-37	postive
TRBC1	AC018755.4	0.689597773	4.36E-72	postive
TRBC2	AC018755.4	0.710658659	2.20E-78	postive
TRBJ2-1	AC018755.4	0.605653374	1.42E-51	postive
TRBJ2-2	AC018755.4	0.610459258	1.38E-52	postive
TRBJ2-3	AC018755.4	0.637370644	1.41E-58	postive
TRBJ2-7	AC018755.4	0.651415674	6.00E-62	postive
TRBV2	AC018755.4	0.596362693	1.14E-49	postive
TRBV3-1	AC018755.4	0.56993938	1.41E-44	postive
TRBV5-1	AC018755.4	0.664551244	2.88E-65	postive
TRBV5-4	AC018755.4	0.506009457	5.40E-34	postive
TRBV5-6	AC018755.4	0.531977182	5.06E-38	postive
TRBV6-5	AC018755.4	0.523534312	1.13E-36	postive
TRBV7-9	AC018755.4	0.571191301	8.26E-45	postive
TRBV10-3	AC018755.4	0.503090489	1.46E-33	postive
TRBV18	AC018755.4	0.631667716	2.94E-57	postive
TRBV19	AC018755.4	0.560272206	7.91E-43	postive
TRBV20-1	AC018755.4	0.610999188	1.06E-52	postive
TRBV28	AC018755.4	0.700570293	2.68E-75	postive
TRBV29-1	AC018755.4	0.640672386	2.36E-59	postive
IFI30	AC069281.2	0.517834883	8.73E-36	postive
CD40LG	AC069281.2	0.51605423	1.64E-35	postive
CD79B	AC069281.2	0.513406318	4.17E-35	postive
CD19	AC069281.2	0.550741655	3.70E-41	postive
INPP5D	AC069281.2	0.516962474	1.19E-35	postive
CD22	AC069281.2	0.525592544	5.33E-37	postive
CD72	AC069281.2	0.522503618	1.64E-36	postive
PTPN6	AC069281.2	0.522238762	1.80E-36	postive
GNRH1	AC069281.2	0.556521604	3.65E-42	postive
RABEP2	AC069281.2	0.509362747	1.70E-34	postive
OGFR	AC069281.2	0.509740847	1.49E-34	postive
TNFRSF13C	AC069281.2	0.556937693	3.08E-42	postive
TNFRSF14	AC069281.2	0.562144726	3.66E-43	postive

TNFRSF25	AC069281.2	0.541323585	1.47E-39	postive
ITGAL	AC069281.2	0.502022842	2.09E-33	postive
ZAP70	AC069281.2	0.602144722	7.55E-51	postive
TRBJ2-2	AC069281.2	0.544898056	3.68E-40	postive
TRBJ2-7	AC069281.2	0.590966127	1.36E-48	postive
PSMD11	AC145207.8	0.540375125	2.11E-39	postive
OSGIN1	AC145207.8	0.629164649	1.09E-56	postive
ANGPTL1	AC093010.2	0.502797753	1.61E-33	postive
TIE1	AC093010.2	0.500323773	3.71E-33	postive
WNT5A	AC121764.1	0.516386426	1.46E-35	postive
B2M	CCR5AS	0.532413174	4.30E-38	postive
CD4	CCR5AS	0.581505524	9.46E-47	postive
CD74	CCR5AS	0.555283061	6.01E-42	postive
CTSS	CCR5AS	0.652318404	3.59E-62	postive
FCER1G	CCR5AS	0.653844041	1.50E-62	postive
HLA-DMA	CCR5AS	0.569102802	2.00E-44	postive
HLA-DMB	CCR5AS	0.633853678	9.25E-58	postive
HLA-DPA1	CCR5AS	0.540892068	1.73E-39	postive
HLA-DPB1	CCR5AS	0.564333247	1.48E-43	postive
HLA-DRA	CCR5AS	0.565984587	7.43E-44	postive
HLA-DRB1	CCR5AS	0.542274687	1.02E-39	postive
CYBB	CCR5AS	0.627180796	3.07E-56	postive
TLR7	CCR5AS	0.556231682	4.10E-42	postive
TLR8	CCR5AS	0.615966452	9.15E-54	postive
MSR1	CCR5AS	0.581121051	1.12E-46	postive
CCL4	CCR5AS	0.506288201	4.91E-34	postive
CD14	CCR5AS	0.571303481	7.87E-45	postive
AQP9	CCR5AS	0.5641732	1.58E-43	postive
CCR5	CCR5AS	0.548022094	1.08E-40	postive
CCR1	CCR5AS	0.64580876	1.40E-60	postive
CXCR6	CCR5AS	0.50380376	1.14E-33	postive
FGR	CCR5AS	0.556276418	4.03E-42	postive
CD86	CCR5AS	0.658412985	1.07E-63	postive
BTK	CCR5AS	0.532668615	3.91E-38	postive
LILRB3	CCR5AS	0.593099833	5.13E-49	postive
FCGR2B	CCR5AS	0.572396858	4.94E-45	postive
CCRL2	CCR5AS	0.733259269	8.40E-86	postive
CMKLR1	CCR5AS	0.570642154	1.04E-44	postive
FPR1	CCR5AS	0.561331944	5.12E-43	postive
PLXNC1	CCR5AS	0.574797019	1.76E-45	postive
GMFG	CCR5AS	0.592089884	8.15E-49	postive
IL32	CCR5AS	0.532590526	4.02E-38	postive
TNFSF13B	CCR5AS	0.689713326	4.04E-72	postive
C3AR1	CCR5AS	0.623285485	2.28E-55	postive
CSF1R	CCR5AS	0.54920777	6.79E-41	postive
CSF2RA	CCR5AS	0.555540651	5.42E-42	postive
IL10RA	CCR5AS	0.513774884	3.66E-35	postive
NR1H3	CCR5AS	0.552985442	1.51E-41	postive
TNFRSF1B	CCR5AS	0.547468333	1.35E-40	postive
ITGB2	CCR5AS	0.555318875	5.93E-42	postive
TYROBP	CCR5AS	0.683683557	2.05E-70	postive
FCGR3A	CCR5AS	0.586054586	1.25E-47	postive
LCP2	CCR5AS	0.633823646	9.39E-58	postive
HCST	CCR5AS	0.594763584	2.39E-49	postive
PTPRC	CCR5AS	0.516784526	1.27E-35	postive
RHOA	CCR5AS	0.540986095	1.67E-39	postive

ICOS	CCR5AS	0.506481554	4.59E-34	postive
LMBR1L	AL365330.1	0.549905384	5.15E-41	postive
IRF3	AL365330.1	0.51998118	4.06E-36	postive
DDX17	AL365330.1	0.539235632	3.27E-39	postive
PLXNB1	AL365330.1	0.53126152	6.60E-38	postive
GNRH1	AL365330.1	0.625764023	6.39E-56	postive
NR2C2	AL365330.1	0.541844907	1.20E-39	postive
TNFRSF25	AL365330.1	0.655463504	5.93E-63	postive
PLCG1	AL365330.1	0.559489082	1.09E-42	postive
CD1C	LINC00926	0.713946486	2.04E-79	postive
HLA-DOB	LINC00926	0.699355265	6.18E-75	postive
LTA	LINC00926	0.800581545	2.77E-113	postive
CIITA	LINC00926	0.559067844	1.29E-42	postive
RBP5	LINC00926	0.508144312	2.59E-34	postive
CETP	LINC00926	0.850619568	1.06E-141	postive
CD40LG	LINC00926	0.806247846	4.48E-116	postive
BACH2	LINC00926	0.56088131	6.16E-43	postive
PDCD1	LINC00926	0.637701792	1.18E-58	postive
CCR7	LINC00926	0.666960753	6.80E-66	postive
CCL21	LINC00926	0.899553922	5.55E-182	postive
CXCR4	LINC00926	0.570958641	9.12E-45	postive
CD79A	LINC00926	0.773099276	6.39E-101	postive
CD79B	LINC00926	0.959392393	5.65E-277	postive
BTK	LINC00926	0.677053871	1.38E-68	postive
VAV1	LINC00926	0.508661234	2.17E-34	postive
RAC2	LINC00926	0.535183193	1.52E-38	postive
CD19	LINC00926	0.974948767	0	postive
CR2	LINC00926	0.930567461	2.49E-220	postive
PIK3CD	LINC00926	0.614883338	1.57E-53	postive
INPP5D	LINC00926	0.750065595	7.88E-92	postive
CD22	LINC00926	0.976393582	0	postive
CD72	LINC00926	0.880019965	8.42E-164	postive
PTPN6	LINC00926	0.591685561	9.81E-49	postive
RASGRP3	LINC00926	0.696387979	4.67E-74	postive
PLCG2	LINC00926	0.694639977	1.52E-73	postive
PRKCB	LINC00926	0.724419945	8.21E-83	postive
IGLJ1	LINC00926	0.819395265	6.34E-123	postive
FLT3LG	LINC00926	0.510468846	1.16E-34	postive
GMFG	LINC00926	0.503236281	1.39E-33	postive
IL16	LINC00926	0.745594684	3.52E-90	postive
LTB	LINC00926	0.759913503	1.37E-95	postive
IL10RA	LINC00926	0.509825125	1.45E-34	postive
IL12RB1	LINC00926	0.587038475	8.06E-48	postive
IL21R	LINC00926	0.512332894	6.07E-35	postive
TNFRSF13C	LINC00926	0.956434606	1.67E-269	postive
ITGAL	LINC00926	0.711252117	1.44E-78	postive
LCK	LINC00926	0.671810052	3.56E-67	postive
NCR3	LINC00926	0.824808269	6.58E-126	postive
CD247	LINC00926	0.614436449	1.96E-53	postive
ZAP70	LINC00926	0.818734219	1.45E-122	postive
CD3E	LINC00926	0.617416595	4.44E-54	postive
PTPRC	LINC00926	0.584154227	2.93E-47	postive
ITK	LINC00926	0.596873715	8.97E-50	postive
GRAP2	LINC00926	0.598668155	3.88E-50	postive
CD28	LINC00926	0.61938713	1.65E-54	postive
ICOS	LINC00926	0.524369433	8.32E-37	postive

CTLA4	LINC00926	0.537155783	7.21E-39	postive
TRAC	LINC00926	0.512857281	5.05E-35	postive
TRAV8-3	LINC00926	0.60600234	1.20E-51	postive
TRBC1	LINC00926	0.683195334	2.81E-70	postive
TRBC2	LINC00926	0.665124642	2.05E-65	postive
TRBJ2-1	LINC00926	0.640267468	2.94E-59	postive
TRBJ2-2	LINC00926	0.750880633	3.91E-92	postive
TRBJ2-3	LINC00926	0.731354419	3.79E-85	postive
TRBJ2-7	LINC00926	0.78714915	5.18E-107	postive
TRBV2	LINC00926	0.65546463	5.93E-63	postive
TRBV3-1	LINC00926	0.578089104	4.23E-46	postive
TRBV5-1	LINC00926	0.658643966	9.39E-64	postive
TRBV18	LINC00926	0.605480186	1.54E-51	postive
TRBV28	LINC00926	0.685195176	7.73E-71	postive
TRBV29-1	LINC00926	0.558119341	1.91E-42	postive
DDX17	AC009120.3	0.538386073	4.52E-39	postive
NR2C2	AC009120.3	0.556240727	4.08E-42	postive
CACYBP	AL392172.1	0.509990448	1.37E-34	postive
NENF	AL392172.1	0.554000115	1.01E-41	postive
LRSAM1	AL445222.2	0.540195764	2.26E-39	postive
NOX4	LINC02544	0.50670956	4.25E-34	postive
PDGFRB	LINC02544	0.57459628	1.92E-45	postive
CLEC11A	LINC02544	0.509872192	1.43E-34	postive
SCT	LINC02544	0.597675502	6.17E-50	postive
ANGPTL2	LINC02544	0.521793519	2.11E-36	postive
SEMA6D	KDM4A-AS1	0.513605386	3.89E-35	postive
ARTN	KDM4A-AS1	0.637104174	1.63E-58	postive
MUC5AC	ELN-AS1	0.620155383	1.12E-54	postive
BPIFB1	ELN-AS1	0.688935899	6.74E-72	postive
BPIFA1	ELN-AS1	0.561619831	4.54E-43	postive
DES	ELN-AS1	0.612921379	4.14E-53	postive
SCGB3A1	ELN-AS1	0.555432888	5.66E-42	postive
GIPR	ELN-AS1	0.532890709	3.60E-38	postive
LTA	AL683807.1	0.524441157	8.11E-37	postive
CETP	AL683807.1	0.555200964	6.22E-42	postive
CD40LG	AL683807.1	0.571098834	8.59E-45	postive
PDCD1	AL683807.1	0.552799906	1.63E-41	postive
CCL21	AL683807.1	0.515958284	1.70E-35	postive
CD79A	AL683807.1	0.512941172	4.91E-35	postive
CD79B	AL683807.1	0.554807576	7.28E-42	postive
BTK	AL683807.1	0.507970308	2.75E-34	postive
CD19	AL683807.1	0.572780122	4.19E-45	postive
CR2	AL683807.1	0.525707162	5.11E-37	postive
INPP5D	AL683807.1	0.519988744	4.04E-36	postive
CD22	AL683807.1	0.544818514	3.80E-40	postive
CD72	AL683807.1	0.566353439	6.36E-44	postive
PRKCB	AL683807.1	0.505647863	6.11E-34	postive
IGLJ1	AL683807.1	0.5531119	1.44E-41	postive
IL16	AL683807.1	0.545573584	2.83E-40	postive
TNFRSF13C	AL683807.1	0.542987973	7.73E-40	postive
ITGAL	AL683807.1	0.552600449	1.76E-41	postive
LCK	AL683807.1	0.515266266	2.17E-35	postive
NCR3	AL683807.1	0.544884704	3.70E-40	postive
CD247	AL683807.1	0.51637619	1.46E-35	postive
ZAP70	AL683807.1	0.600109261	1.97E-50	postive
CD3E	AL683807.1	0.506466689	4.62E-34	postive

ITK	AL683807.1	0.53712484	7.30E-39	postive
TRAV8-3	AL683807.1	0.500817997	3.14E-33	postive
TRBC1	AL683807.1	0.5407202	1.85E-39	postive
TRBC2	AL683807.1	0.523776203	1.03E-36	postive
TRBJ2-1	AL683807.1	0.510676231	1.08E-34	postive
TRBJ2-2	AL683807.1	0.537955901	5.32E-39	postive
TRBJ2-3	AL683807.1	0.526829748	3.39E-37	postive
TRBJ2-7	AL683807.1	0.58417641	2.90E-47	postive
TRBV28	AL683807.1	0.514568507	2.77E-35	postive
ANGPTL4	AL355312.4	0.555805504	4.87E-42	postive
GNRH1	AP003352.1	0.549897122	5.17E-41	postive
NR2C1	AP003352.1	0.51176497	7.40E-35	postive
TNFRSF25	AP003352.1	0.524802009	7.11E-37	postive
MUC5AC	LINC02166	0.527777511	2.39E-37	postive
BPIFB1	LINC02166	0.606736826	8.41E-52	postive
BPIFA1	LINC02166	0.55801784	1.99E-42	postive
DES	LINC02166	0.502301048	1.90E-33	postive
SCGB3A1	LINC02166	0.519183253	5.40E-36	postive
AZGP1	CCNT2-AS1	0.569102362	2.00E-44	postive
DDX17	AL022328.1	0.620264037	1.06E-54	postive
PLXNB1	AL022328.1	0.577640059	5.15E-46	postive
NR2C2	AL022328.1	0.50547822	6.47E-34	postive
DDX17	ATP1B3-AS1	0.502375567	1.86E-33	postive
CD1C	AL133371.2	0.623219852	2.36E-55	postive
CD1D	AL133371.2	0.531486333	6.07E-38	postive
CD4	AL133371.2	0.725678989	3.13E-83	postive
CD74	AL133371.2	0.640113659	3.20E-59	postive
CTSS	AL133371.2	0.595206968	1.94E-49	postive
HLA-DMA	AL133371.2	0.642881091	7.04E-60	postive
HLA-DMB	AL133371.2	0.646153598	1.15E-60	postive
HLA-DOA	AL133371.2	0.597675814	6.17E-50	postive
HLA-DOB	AL133371.2	0.554510255	8.21E-42	postive
HLA-DPA1	AL133371.2	0.62034005	1.02E-54	postive
HLA-DPB1	AL133371.2	0.657870514	1.47E-63	postive
HLA-DRA	AL133371.2	0.526137553	4.37E-37	postive
HLA-DRB1	AL133371.2	0.509342061	1.72E-34	postive
LTA	AL133371.2	0.515958408	1.70E-35	postive
IFI30	AL133371.2	0.53019839	9.80E-38	postive
COLEC12	AL133371.2	0.526929286	3.27E-37	postive
A2M	AL133371.2	0.559394315	1.13E-42	postive
CYBB	AL133371.2	0.655215979	6.84E-63	postive
TLR7	AL133371.2	0.686827266	2.68E-71	postive
CYLD	AL133371.2	0.557079199	2.91E-42	postive
TLR8	AL133371.2	0.535413475	1.39E-38	postive
MSR1	AL133371.2	0.529821094	1.13E-37	postive
IL7R	AL133371.2	0.593425984	4.42E-49	postive
IFNAR2	AL133371.2	0.503143379	1.43E-33	postive
CD40LG	AL133371.2	0.648334388	3.41E-61	postive
CD14	AL133371.2	0.542648397	8.81E-40	postive
TRIM22	AL133371.2	0.564143424	1.60E-43	postive
PDCD1	AL133371.2	0.538605468	4.16E-39	postive
ANXA6	AL133371.2	0.591421256	1.11E-48	postive
CCR8	AL133371.2	0.527487163	2.66E-37	postive
CCR5	AL133371.2	0.573394085	3.22E-45	postive
CCL23	AL133371.2	0.51433145	3.01E-35	postive
CCR1	AL133371.2	0.599428685	2.72E-50	postive

CXCR4	AL133371.2	0.562412265	3.28E-43	postive
CCR4	AL133371.2	0.700869534	2.18E-75	postive
FGR	AL133371.2	0.592758856	6.00E-49	postive
CD86	AL133371.2	0.640987307	1.99E-59	postive
CD79A	AL133371.2	0.573400287	3.21E-45	postive
BTK	AL133371.2	0.77090962	5.19E-100	postive
VAV1	AL133371.2	0.576348057	9.02E-46	postive
NFATC2	AL133371.2	0.515830815	1.78E-35	postive
PIK3R5	AL133371.2	0.614036801	2.38E-53	postive
INPP5D	AL133371.2	0.63189277	2.61E-57	postive
CD72	AL133371.2	0.548644416	8.48E-41	postive
LILRB3	AL133371.2	0.571305982	7.86E-45	postive
FCGR2B	AL133371.2	0.631898311	2.60E-57	postive
RASGRP3	AL133371.2	0.6708429	6.45E-67	postive
PLCG2	AL133371.2	0.531869344	5.27E-38	postive
PRKCB	AL133371.2	0.683759192	1.95E-70	postive
IGKJ5	AL133371.2	0.509629501	1.55E-34	postive
C3	AL133371.2	0.538528867	4.28E-39	postive
C5AR1	AL133371.2	0.570593881	1.06E-44	postive
CCRL2	AL133371.2	0.545342355	3.10E-40	postive
CMKLR1	AL133371.2	0.616058149	8.75E-54	postive
CX3CR1	AL133371.2	0.522253356	1.79E-36	postive
CXCR3	AL133371.2	0.50124704	2.72E-33	postive
ACKR1	AL133371.2	0.552915253	1.56E-41	postive
FPR1	AL133371.2	0.536641446	8.77E-39	postive
PLXNC1	AL133371.2	0.653151722	2.23E-62	postive
ADA2	AL133371.2	0.620396306	9.91E-55	postive
GMFG	AL133371.2	0.665802946	1.36E-65	postive
IL16	AL133371.2	0.726756724	1.37E-83	postive
IL6ST	AL133371.2	0.582568871	5.92E-47	postive
TNFSF12	AL133371.2	0.602810237	5.50E-51	postive
TNFSF13	AL133371.2	0.503089058	1.46E-33	postive
TNFSF13B	AL133371.2	0.542558004	9.12E-40	postive
ACVRL1	AL133371.2	0.585096142	1.92E-47	postive
C3AR1	AL133371.2	0.609557386	2.15E-52	postive
CSF1R	AL133371.2	0.663529513	5.29E-65	postive
CSF2RA	AL133371.2	0.570628045	1.05E-44	postive
ENG	AL133371.2	0.605549523	1.49E-51	postive
FLT4	AL133371.2	0.536577364	8.98E-39	postive
IL10RA	AL133371.2	0.753628266	3.60E-93	postive
IL12RB1	AL133371.2	0.627522793	2.57E-56	postive
IL3RA	AL133371.2	0.600492719	1.65E-50	postive
S1PR1	AL133371.2	0.64612878	1.17E-60	postive
TEK	AL133371.2	0.55972473	9.90E-43	postive
TGFBR2	AL133371.2	0.586310148	1.12E-47	postive
TIE1	AL133371.2	0.612119931	6.13E-53	postive
TNFRSF1B	AL133371.2	0.696321263	4.89E-74	postive
ICAM2	AL133371.2	0.568274828	2.84E-44	postive
ITGAL	AL133371.2	0.663834643	4.41E-65	postive
ITGB2	AL133371.2	0.572773311	4.20E-45	postive
TYROBP	AL133371.2	0.556488753	3.69E-42	postive
LCK	AL133371.2	0.527032955	3.15E-37	postive
FCGR3A	AL133371.2	0.534953646	1.66E-38	postive
NCR3	AL133371.2	0.61697577	5.53E-54	postive
CD247	AL133371.2	0.624035796	1.55E-55	postive
ZAP70	AL133371.2	0.506130802	5.18E-34	postive

LCP2	AL133371.2	0.672076988	3.03E-67	postive
HCST	AL133371.2	0.589031749	3.28E-48	postive
CD3D	AL133371.2	0.529196974	1.42E-37	postive
CD3E	AL133371.2	0.628290716	1.72E-56	postive
PTPRC	AL133371.2	0.760829347	5.98E-96	postive
ITK	AL133371.2	0.703072557	4.73E-76	postive
GRAP2	AL133371.2	0.573329086	3.31E-45	postive
CD28	AL133371.2	0.743884577	1.47E-89	postive
ICOS	AL133371.2	0.630934522	4.32E-57	postive
CTLA4	AL133371.2	0.55550438	5.50E-42	postive
TRAC	AL133371.2	0.592168629	7.86E-49	postive
TRAV2	AL133371.2	0.52044349	3.44E-36	postive
TRAV8-3	AL133371.2	0.596714236	9.66E-50	postive
TRAV8-6	AL133371.2	0.587200241	7.49E-48	postive
TRAV9-2	AL133371.2	0.623239457	2.33E-55	postive
TRAV12-1	AL133371.2	0.52308731	1.33E-36	postive
TRAV13-1	AL133371.2	0.55570955	5.06E-42	postive
TRAV21	AL133371.2	0.548832391	7.88E-41	postive
TRBC1	AL133371.2	0.613741935	2.76E-53	postive
TRBC2	AL133371.2	0.611029223	1.05E-52	postive
TRBJ2-1	AL133371.2	0.555656893	5.17E-42	postive
TRBJ2-2	AL133371.2	0.535051873	1.60E-38	postive
TRBJ2-3	AL133371.2	0.505790671	5.82E-34	postive
TRBJ2-7	AL133371.2	0.566690793	5.53E-44	postive
TRBV2	AL133371.2	0.56404072	1.67E-43	postive
TRBV3-1	AL133371.2	0.554439935	8.44E-42	postive
TRBV5-1	AL133371.2	0.606611531	8.93E-52	postive
TRBV5-4	AL133371.2	0.563585216	2.02E-43	postive
TRBV5-6	AL133371.2	0.533585662	2.77E-38	postive
TRBV6-1	AL133371.2	0.533004713	3.45E-38	postive
TRBV6-5	AL133371.2	0.53187783	5.25E-38	postive
TRBV7-9	AL133371.2	0.506202395	5.05E-34	postive
TRBV18	AL133371.2	0.634637625	6.09E-58	postive
TRBV19	AL133371.2	0.535255197	1.48E-38	postive
TRBV20-1	AL133371.2	0.631578629	3.08E-57	postive
TRBV28	AL133371.2	0.603929412	3.23E-51	postive
TRBV29-1	AL133371.2	0.639400418	4.71E-59	postive
DDX17	AC009120.2	0.598529871	4.14E-50	postive
PLXNB1	AC009120.2	0.530452135	8.92E-38	postive
GNRH1	AC009120.2	0.611497324	8.32E-53	postive
MC1R	AC009120.2	0.510897112	1.00E-34	postive
CHGA	AL162171.1	0.510811354	1.03E-34	postive
GHRH	AL162171.1	0.510350091	1.21E-34	postive
SCG2	AL162171.1	0.509410258	1.68E-34	postive
TNFRSF19	AC004540.2	0.640903192	2.08E-59	postive
GNRH1	AC138028.4	0.615065927	1.43E-53	postive
TNFRSF25	AC138028.4	0.575692376	1.20E-45	postive
CYSLTR2	LINC02167	0.552429815	1.89E-41	postive
RELB	LINC02384	0.54551565	2.89E-40	postive
RFX5	LINC02384	0.572002124	5.84E-45	postive
VCAM1	LINC02384	0.517393669	1.02E-35	postive
CD70	LINC02384	0.525675982	5.17E-37	postive
IL21R	LINC02384	0.503754673	1.16E-33	postive
SSTR2	LINC02384	0.52372403	1.05E-36	postive
TNFRSF9	LINC02384	0.54213983	1.07E-39	postive
MAP3K14	LINC02384	0.686463391	3.40E-71	postive

TRGJP2	LINC02384	0.585542417	1.58E-47	postive
TRGC2	LINC02384	0.622434192	3.52E-55	postive
PTGS2	LINC01436	0.563860338	1.80E-43	postive
IL1RL1	LINC01436	0.68298392	3.21E-70	postive
CDK4	LINC01436	0.624622936	1.15E-55	postive
OSGIN1	DLGAP1-AS2	0.529921095	1.09E-37	postive
GNRH1	AC008610.1	0.540939424	1.70E-39	postive
TNFRSF25	AC008610.1	0.503362709	1.33E-33	postive
HLA-DOB	AL357992.1	0.503710979	1.18E-33	postive
LTA	AL357992.1	0.541256704	1.51E-39	postive
CETP	AL357992.1	0.526853142	3.36E-37	postive
CD40LG	AL357992.1	0.575742853	1.17E-45	postive
PDCD1	AL357992.1	0.537566361	6.17E-39	postive
CCL21	AL357992.1	0.534253141	2.16E-38	postive
CD79A	AL357992.1	0.515525637	1.98E-35	postive
CD79B	AL357992.1	0.575399804	1.36E-45	postive
BTK	AL357992.1	0.509151946	1.83E-34	postive
CD19	AL357992.1	0.595928992	1.39E-49	postive
CR2	AL357992.1	0.51510966	2.29E-35	postive
INPP5D	AL357992.1	0.53090421	7.54E-38	postive
CD22	AL357992.1	0.55293664	1.54E-41	postive
CD72	AL357992.1	0.616982023	5.52E-54	postive
RASGRP3	AL357992.1	0.501116926	2.84E-33	postive
IGLJ1	AL357992.1	0.566182345	6.84E-44	postive
IL16	AL357992.1	0.542493169	9.35E-40	postive
LTB	AL357992.1	0.538979796	3.60E-39	postive
IL12RB1	AL357992.1	0.542107824	1.09E-39	postive
TNFRSF13C	AL357992.1	0.547606489	1.28E-40	postive
ITGAL	AL357992.1	0.549377876	6.35E-41	postive
LCK	AL357992.1	0.571937148	6.01E-45	postive
NCR3	AL357992.1	0.56937145	1.79E-44	postive
CD247	AL357992.1	0.536029627	1.11E-38	postive
ZAP70	AL357992.1	0.630916117	4.37E-57	postive
CD3D	AL357992.1	0.519030401	5.70E-36	postive
CD3E	AL357992.1	0.537756115	5.74E-39	postive
ITK	AL357992.1	0.511644968	7.71E-35	postive
GRAP2	AL357992.1	0.505598135	6.21E-34	postive
CTLA4	AL357992.1	0.537093836	7.39E-39	postive
TRAV8-3	AL357992.1	0.517179468	1.10E-35	postive
TRBC1	AL357992.1	0.548953195	7.51E-41	postive
TRBC2	AL357992.1	0.590541883	1.65E-48	postive
TRBJ2-1	AL357992.1	0.52189998	2.04E-36	postive
TRBJ2-2	AL357992.1	0.556343589	3.92E-42	postive
TRBJ2-3	AL357992.1	0.566575793	5.80E-44	postive
TRBJ2-7	AL357992.1	0.582442742	6.26E-47	postive
TRBV5-1	AL357992.1	0.543124007	7.33E-40	postive
TRBV28	AL357992.1	0.557437577	2.51E-42	postive
NRTN	HHIP-AS1	0.500018212	4.11E-33	postive
GNRH1	AC084824.5	0.559366874	1.15E-42	postive
SOS1	SOS1-IT1	0.570231042	1.24E-44	postive
SEMA3B	AC105020.1	0.51470476	2.64E-35	postive
GNRH1	AC105020.1	0.57578624	1.15E-45	postive
HLA-DOB	LINC00582	0.562939248	2.64E-43	postive
ISG20	LINC00582	0.586714896	9.32E-48	postive
CD79A	LINC00582	0.596963726	8.60E-50	postive
FCGR2B	LINC00582	0.577266846	6.05E-46	postive

IGHA1	LINC00582	0.74107717	1.51E-88	postive
IGHA2	LINC00582	0.717901339	1.11E-80	postive
IGHD6-25	LINC00582	0.682461434	4.49E-70	postive
IGHG1	LINC00582	0.805595189	9.50E-116	postive
IGHG2	LINC00582	0.773403981	4.77E-101	postive
IGHG3	LINC00582	0.742954973	3.19E-89	postive
IGHG4	LINC00582	0.636227058	2.61E-58	postive
IGHJ2	LINC00582	0.713239065	3.41E-79	postive
IGHJ3	LINC00582	0.814744996	1.94E-120	postive
IGHV1-2	LINC00582	0.58915131	3.11E-48	postive
IGHV1-24	LINC00582	0.671334058	4.77E-67	postive
IGHV1-46	LINC00582	0.697303174	2.51E-74	postive
IGHV1-69	LINC00582	0.679911619	2.28E-69	postive
IGHV2-26	LINC00582	0.536001774	1.12E-38	postive
IGHV2-5	LINC00582	0.595896794	1.41E-49	postive
IGHV2-70	LINC00582	0.672384102	2.51E-67	postive
IGHV3-11	LINC00582	0.592885608	5.66E-49	postive
IGHV3-15	LINC00582	0.670538295	7.76E-67	postive
IGHV3-20	LINC00582	0.62468804	1.11E-55	postive
IGHV3-21	LINC00582	0.638105674	9.48E-59	postive
IGHV3-23	LINC00582	0.731434839	3.56E-85	postive
IGHV3-30	LINC00582	0.706274927	5.01E-77	postive
IGHV3-33	LINC00582	0.644763271	2.49E-60	postive
IGHV3-35	LINC00582	0.661436236	1.83E-64	postive
IGHV3-43	LINC00582	0.536329336	9.87E-39	postive
IGHV3-49	LINC00582	0.530763491	7.95E-38	postive
IGHV3-53	LINC00582	0.533244968	3.15E-38	postive
IGHV3-66	LINC00582	0.593717645	3.86E-49	postive
IGHV3-7	LINC00582	0.585536453	1.58E-47	postive
IGHV3-72	LINC00582	0.558032232	1.97E-42	postive
IGHV3-73	LINC00582	0.516590478	1.36E-35	postive
IGHV3-74	LINC00582	0.585008206	2.00E-47	postive
IGHV4-28	LINC00582	0.736218403	7.88E-87	postive
IGHV4-31	LINC00582	0.632597032	1.80E-57	postive
IGHV4-34	LINC00582	0.617550075	4.15E-54	postive
IGHV4-39	LINC00582	0.577650686	5.12E-46	postive
IGHV4-59	LINC00582	0.725320311	4.12E-83	postive
IGHV4-61	LINC00582	0.638811972	6.47E-59	postive
IGHV5-51	LINC00582	0.737321615	3.24E-87	postive
IGKC	LINC00582	0.899122708	1.53E-181	postive
IGKJ5	LINC00582	0.779708	9.92E-104	postive
IGKV1-12	LINC00582	0.621088382	6.98E-55	postive
IGKV1-13	LINC00582	0.658599454	9.64E-64	postive
IGKV1-16	LINC00582	0.583184139	4.50E-47	postive
IGKV1-17	LINC00582	0.605449465	1.56E-51	postive
IGKV1-27	LINC00582	0.616465964	7.14E-54	postive
IGKV1-33	LINC00582	0.648726254	2.74E-61	postive
IGKV1-39	LINC00582	0.75376254	3.20E-93	postive
IGKV1-5	LINC00582	0.762853464	9.49E-97	postive
IGKV1-8	LINC00582	0.696152827	5.48E-74	postive
IGKV1-9	LINC00582	0.520311122	3.60E-36	postive
IGKV1D-12	LINC00582	0.68992629	3.51E-72	postive
IGKV1D-13	LINC00582	0.551615589	2.61E-41	postive
IGKV1D-33	LINC00582	0.664363263	3.22E-65	postive
IGKV1D-39	LINC00582	0.740469752	2.49E-88	postive
IGKV1D-42	LINC00582	0.702070552	9.50E-76	postive

IGKV1D-43	LINC00582	0.513635484	3.85E-35	postive
IGKV1D-8	LINC00582	0.767844894	9.37E-99	postive
IGKV2-28	LINC00582	0.580211043	1.67E-46	postive
IGKV2D-28	LINC00582	0.560670478	6.72E-43	postive
IGKV3-11	LINC00582	0.841642147	6.89E-136	postive
IGKV3-15	LINC00582	0.829411873	1.58E-128	postive
IGKV3-20	LINC00582	0.804547	3.15E-115	postive
IGKV3-7	LINC00582	0.73177584	2.72E-85	postive
IGKV3D-11	LINC00582	0.786190409	1.40E-106	postive
IGKV3D-15	LINC00582	0.726203754	2.09E-83	postive
IGKV3D-20	LINC00582	0.59182248	9.21E-49	postive
IGKV3D-7	LINC00582	0.644605706	2.72E-60	postive
IGKV4-1	LINC00582	0.693973032	2.38E-73	postive
IGKV5-2	LINC00582	0.53012282	1.01E-37	postive
IGLC2	LINC00582	0.861048718	5.92E-149	postive
IGLC3	LINC00582	0.754187525	2.21E-93	postive
IGLC6	LINC00582	0.695225007	1.03E-73	postive
IGLC7	LINC00582	0.501334803	2.64E-33	postive
IGLJ2	LINC00582	0.698451279	1.15E-74	postive
IGLJ3	LINC00582	0.705824889	6.88E-77	postive
IGLV1-36	LINC00582	0.629255344	1.04E-56	postive
IGLV1-40	LINC00582	0.785101622	4.27E-106	postive
IGLV1-44	LINC00582	0.786953114	6.35E-107	postive
IGLV1-50	LINC00582	0.637271547	1.49E-58	postive
IGLV1-51	LINC00582	0.715800804	5.24E-80	postive
IGLV10-54	LINC00582	0.522230214	1.81E-36	postive
IGLV2-11	LINC00582	0.664001537	4.00E-65	postive
IGLV2-14	LINC00582	0.692336098	7.11E-73	postive
IGLV2-18	LINC00582	0.524444525	8.10E-37	postive
IGLV2-23	LINC00582	0.617872762	3.53E-54	postive
IGLV2-33	LINC00582	0.553022223	1.49E-41	postive
IGLV3-1	LINC00582	0.720085545	2.18E-81	postive
IGLV3-10	LINC00582	0.559777407	9.68E-43	postive
IGLV3-16	LINC00582	0.515829229	1.78E-35	postive
IGLV3-19	LINC00582	0.642605597	8.19E-60	postive
IGLV3-21	LINC00582	0.647645575	5.02E-61	postive
IGLV3-25	LINC00582	0.690849601	1.91E-72	postive
IGLV3-27	LINC00582	0.558528197	1.61E-42	postive
IGLV3-9	LINC00582	0.514838351	2.52E-35	postive
IGLV4-69	LINC00582	0.640128938	3.17E-59	postive
IGLV6-57	LINC00582	0.619466611	1.59E-54	postive
IGLV9-49	LINC00582	0.585696244	1.47E-47	postive
GMFG	LINC00582	0.53074901	7.99E-38	postive
IL16	LINC00582	0.502919285	1.54E-33	postive
TNFRSF17	LINC00582	0.88928738	5.33E-172	postive
ICAM2	LINC00582	0.526556295	3.75E-37	postive
DDX17	AL355075.2	0.595889054	1.42E-49	postive
IRF9	AL355075.2	0.585728521	1.45E-47	postive
GNRH1	AL355075.2	0.548184934	1.02E-40	postive
CTSE	LINC02471	0.710249872	2.96E-78	postive
CXCL2	LINC02471	0.509189702	1.81E-34	postive
SFTPD	LINC02471	0.759859756	1.43E-95	postive
COLEC12	LINC02471	0.507708419	3.01E-34	postive
A2M	LINC02471	0.524930296	6.79E-37	postive
SFTPA1	LINC02471	0.794586977	1.99E-110	postive
SFTPA2	LINC02471	0.792651214	1.59E-109	postive

DMBT1	LINC02471	0.653493421	1.84E-62	postive
CCL23	LINC02471	0.64071033	2.31E-59	postive
ACKR1	LINC02471	0.501033744	2.92E-33	postive
AGTR2	LINC02471	0.558750696	1.47E-42	postive
CSF3R	LINC02471	0.518047544	8.09E-36	postive
NPR1	LINC02471	0.548160332	1.03E-40	postive
S1PR1	LINC02471	0.517790311	8.87E-36	postive
SCTR	LINC02471	0.567627045	3.73E-44	postive
TEK	LINC02471	0.521263615	2.56E-36	postive
GPI	AC002116.2	0.528427359	1.89E-37	postive
VGF	PDE2A-AS2	0.762904421	9.06E-97	postive
CD1C	ITGB2-AS1	0.698458028	1.14E-74	postive
HLA-DOB	ITGB2-AS1	0.701151382	1.80E-75	postive
LTA	ITGB2-AS1	0.808194156	4.69E-117	postive
CIITA	ITGB2-AS1	0.598849156	3.57E-50	postive
RBP5	ITGB2-AS1	0.511421539	8.34E-35	postive
CETP	ITGB2-AS1	0.823525628	3.42E-125	postive
CD40LG	ITGB2-AS1	0.813155821	1.32E-119	postive
BACH2	ITGB2-AS1	0.518346969	7.27E-36	postive
PDCD1	ITGB2-AS1	0.674470064	6.91E-68	postive
CCR7	ITGB2-AS1	0.657906909	1.44E-63	postive
CCL21	ITGB2-AS1	0.886987092	6.73E-170	postive
CXCR4	ITGB2-AS1	0.571204595	8.21E-45	postive
CD79A	ITGB2-AS1	0.74529318	4.53E-90	postive
CD79B	ITGB2-AS1	0.934451668	2.30E-226	postive
BTK	ITGB2-AS1	0.691273002	1.44E-72	postive
VAV1	ITGB2-AS1	0.548899729	7.67E-41	postive
RAC2	ITGB2-AS1	0.571722767	6.58E-45	postive
CD19	ITGB2-AS1	0.946190513	3.87E-247	postive
CR2	ITGB2-AS1	0.886668728	1.30E-169	postive
PIK3CD	ITGB2-AS1	0.634491244	6.59E-58	postive
INPP5D	ITGB2-AS1	0.756156878	3.90E-94	postive
CD22	ITGB2-AS1	0.951531521	3.41E-258	postive
CD72	ITGB2-AS1	0.891639166	3.38E-174	postive
PTPN6	ITGB2-AS1	0.644012573	3.78E-60	postive
RASGRP3	ITGB2-AS1	0.695610784	7.90E-74	postive
PLCG2	ITGB2-AS1	0.66786415	3.94E-66	postive
PRKCB	ITGB2-AS1	0.717471095	1.53E-80	postive
IGLJ1	ITGB2-AS1	0.81193647	5.68E-119	postive
FLT3LG	ITGB2-AS1	0.554111578	9.63E-42	postive
GMFG	ITGB2-AS1	0.54240241	9.69E-40	postive
IL16	ITGB2-AS1	0.738779353	9.91E-88	postive
LTB	ITGB2-AS1	0.775112367	9.12E-102	postive
IL10RA	ITGB2-AS1	0.546214254	2.20E-40	postive
IL12RB1	ITGB2-AS1	0.645595369	1.57E-60	postive
IL21R	ITGB2-AS1	0.556913714	3.11E-42	postive
TNFRSF13C	ITGB2-AS1	0.922895346	2.22E-209	postive
ITGAL	ITGB2-AS1	0.748309909	3.54E-91	postive
LCK	ITGB2-AS1	0.702787422	5.77E-76	postive
NCR3	ITGB2-AS1	0.810209017	4.41E-118	postive
CD247	ITGB2-AS1	0.657361424	1.98E-63	postive
ZAP70	ITGB2-AS1	0.844027639	2.13E-137	postive
CD3D	ITGB2-AS1	0.541633905	1.30E-39	postive
CD3E	ITGB2-AS1	0.66216585	1.19E-64	postive
PTPRC	ITGB2-AS1	0.615070043	1.43E-53	postive
ITK	ITGB2-AS1	0.612438818	5.24E-53	postive

GRAP2	ITGB2-AS1	0.640118783	3.19E-59	postive
CD28	ITGB2-AS1	0.624226555	1.41E-55	postive
ICOS	ITGB2-AS1	0.573973554	2.51E-45	postive
CTLA4	ITGB2-AS1	0.587220781	7.42E-48	postive
TRAC	ITGB2-AS1	0.556945532	3.07E-42	postive
TRAV8-3	ITGB2-AS1	0.64319797	5.92E-60	postive
TRAV9-2	ITGB2-AS1	0.518111228	7.91E-36	postive
TRBC1	ITGB2-AS1	0.720146808	2.08E-81	postive
TRBC2	ITGB2-AS1	0.705686013	7.59E-77	postive
TRBJ2-1	ITGB2-AS1	0.671257379	5.00E-67	postive
TRBJ2-2	ITGB2-AS1	0.772807038	8.47E-101	postive
TRBJ2-3	ITGB2-AS1	0.750418112	5.82E-92	postive
TRBJ2-7	ITGB2-AS1	0.805881957	6.83E-116	postive
TRBV2	ITGB2-AS1	0.664621022	2.76E-65	postive
TRBV3-1	ITGB2-AS1	0.599229149	2.98E-50	postive
TRBV5-1	ITGB2-AS1	0.674495317	6.80E-68	postive
TRBV5-4	ITGB2-AS1	0.501462884	2.53E-33	postive
TRBV18	ITGB2-AS1	0.636404804	2.37E-58	postive
TRBV19	ITGB2-AS1	0.509215153	1.79E-34	postive
TRBV28	ITGB2-AS1	0.729254795	1.97E-84	postive
TRBV29-1	ITGB2-AS1	0.57198241	5.89E-45	postive
DEFA5	AC073365.1	0.520284635	3.64E-36	postive
GNRH1	LINC01004	0.609679326	2.02E-52	postive
PCSK2	AL589843.1	0.580750736	1.32E-46	postive
AKT2	AL589843.1	0.502935907	1.54E-33	postive
GRP	AL589843.1	0.672864834	1.86E-67	postive
ANGPTL1	AL589843.1	0.61836063	2.77E-54	postive
HSPA6	AC109322.1	0.546048311	2.35E-40	postive
RAF1	FGD5-AS1	0.531920261	5.17E-38	postive
DDX17	AL022328.2	0.631962889	2.52E-57	postive
PLXNB1	AL022328.2	0.587521967	6.48E-48	postive
GNRH1	AL022328.2	0.568318863	2.79E-44	postive
TNFRSF25	AL022328.2	0.515663221	1.88E-35	postive
DDX17	AP000692.1	0.526192385	4.28E-37	postive
PLXNB1	AP000692.1	0.541192835	1.54E-39	postive
GNRH1	AP000692.1	0.529115402	1.46E-37	postive
PSMC1	FOXN3-AS1	0.545844942	2.54E-40	postive
TNFRSF25	AC092171.4	0.517515175	9.78E-36	postive
DDX17	CR936218.1	0.585366906	1.70E-47	postive
PLXNB1	CR936218.1	0.558643811	1.54E-42	postive
GNRH1	CR936218.1	0.516074558	1.63E-35	postive
NR2C2	CR936218.1	0.551668222	2.56E-41	postive
WNT5A	LINC01508	0.508945584	1.97E-34	postive
IGLV8-61	AC005332.3	0.525705815	5.12E-37	postive
IDO1	AC007991.2	0.612838358	4.31E-53	postive
HLA-DMA	TNFRSF14-AS1	0.505141583	7.26E-34	postive
CIITA	TNFRSF14-AS1	0.546779951	1.77E-40	postive
IFI30	TNFRSF14-AS1	0.593387325	4.50E-49	postive
LMBR1L	TNFRSF14-AS1	0.504501445	9.03E-34	postive
CD40LG	TNFRSF14-AS1	0.549592132	5.84E-41	postive
TRIM22	TNFRSF14-AS1	0.511393912	8.42E-35	postive
PDCD1	TNFRSF14-AS1	0.547457741	1.35E-40	postive
BTK	TNFRSF14-AS1	0.504409964	9.31E-34	postive
INPP5D	TNFRSF14-AS1	0.553876678	1.06E-41	postive
PTPN6	TNFRSF14-AS1	0.523955794	9.67E-37	postive
RASGRP3	TNFRSF14-AS1	0.507331778	3.43E-34	postive

FLT3LG	TNFRSF14-AS1	0.513366568	4.23E-35	postive
IL16	TNFRSF14-AS1	0.509767617	1.48E-34	postive
LTB	TNFRSF14-AS1	0.514778326	2.57E-35	postive
TNFSF14	TNFRSF14-AS1	0.535470443	1.36E-38	postive
GIPR	TNFRSF14-AS1	0.591081604	1.29E-48	postive
IL10RA	TNFRSF14-AS1	0.505265384	6.96E-34	postive
IL12RB1	TNFRSF14-AS1	0.531337256	6.42E-38	postive
TNFRSF14	TNFRSF14-AS1	0.85936327	9.63E-148	postive
TNFRSF1B	TNFRSF14-AS1	0.501820217	2.24E-33	postive
ITGAL	TNFRSF14-AS1	0.554476534	8.32E-42	postive
CD247	TNFRSF14-AS1	0.555151892	6.34E-42	postive
ZAP70	TNFRSF14-AS1	0.606873769	7.87E-52	postive
CD3E	TNFRSF14-AS1	0.500438364	3.57E-33	postive
ITK	TNFRSF14-AS1	0.535879653	1.17E-38	postive
GRAP2	TNFRSF14-AS1	0.51360626	3.89E-35	postive
TRBC1	TNFRSF14-AS1	0.514399315	2.94E-35	postive
TRBC2	TNFRSF14-AS1	0.513417722	4.15E-35	postive
TRBJ2-2	TNFRSF14-AS1	0.504803234	8.15E-34	postive
TRBJ2-7	TNFRSF14-AS1	0.583893935	3.29E-47	postive
TRBV28	TNFRSF14-AS1	0.502534645	1.76E-33	postive
VGF	AC006504.5	0.580686262	1.36E-46	postive
IGHA1	AC087752.3	0.508844488	2.04E-34	postive
IGHA2	AC087752.3	0.556212081	4.13E-42	postive
IGHD6-25	AC087752.3	0.579894574	1.92E-46	postive
IGHG2	AC087752.3	0.544301035	4.64E-40	postive
IGHG3	AC087752.3	0.505956227	5.50E-34	postive
IGKJ5	AC087752.3	0.573265387	3.40E-45	postive
IGKV3-7	AC087752.3	0.534090755	2.29E-38	postive
BMP5	AL139393.3	0.56078075	6.42E-43	postive
EED	RAB30-DT	0.529697786	1.18E-37	postive
DDX17	AC245060.2	0.558413676	1.69E-42	postive
CD320	AC008946.1	0.513387972	4.19E-35	postive
GNRH1	LINC00342	0.559378585	1.14E-42	postive
GIPR	LINC00342	0.517929064	8.44E-36	postive
PROC	AC114810.1	0.65359219	1.74E-62	postive
CALCA	AC114810.1	0.734596713	2.90E-86	postive
THPO	AC114810.1	0.654397421	1.10E-62	postive
BMP8B	AL031985.3	0.54173237	1.25E-39	postive
PIK3CG	AL138767.3	0.556662178	3.44E-42	postive
MAP3K14	AL138767.3	0.54227625	1.02E-39	postive
TRGJP2	AL138767.3	0.575648169	1.22E-45	postive
TRGC2	AL138767.3	0.584548716	2.46E-47	postive
B2M	LINC02446	0.509805041	1.46E-34	postive
CD8A	LINC02446	0.836452624	1.08E-132	postive
CTSS	LINC02446	0.501218554	2.74E-33	postive
FCER1G	LINC02446	0.630302872	6.03E-57	postive
HLA-DMB	LINC02446	0.505604813	6.20E-34	postive
IFNG	LINC02446	0.871491787	7.79E-157	postive
TAP1	LINC02446	0.50891168	1.99E-34	postive
CXCL9	LINC02446	0.729498677	1.63E-84	postive
STAT1	LINC02446	0.547313696	1.43E-40	postive
IRF1	LINC02446	0.600509776	1.63E-50	postive
CCL5	LINC02446	0.649491072	1.78E-61	postive
TLR8	LINC02446	0.554565838	8.03E-42	postive
GNLY	LINC02446	0.588554738	4.07E-48	postive
CCL4	LINC02446	0.762439253	1.39E-96	postive

PDCD1	LINC02446	0.510803857	1.03E-34	postive
FASLG	LINC02446	0.748292462	3.59E-91	postive
GBP2	LINC02446	0.570739586	1.00E-44	postive
CCL3	LINC02446	0.539867472	2.57E-39	postive
CCR5	LINC02446	0.592176219	7.84E-49	postive
XCL2	LINC02446	0.710108322	3.27E-78	postive
CXCR6	LINC02446	0.761504127	3.24E-96	postive
CXCR3	LINC02446	0.617656463	3.94E-54	postive
GMFG	LINC02446	0.517515013	9.78E-36	postive
SECTM1	LINC02446	0.522305726	1.76E-36	postive
TNFSF13B	LINC02446	0.542401832	9.69E-40	postive
IL12RB1	LINC02446	0.573101048	3.65E-45	postive
IL18RAP	LINC02446	0.694167128	2.09E-73	postive
NR1H3	LINC02446	0.611629818	7.80E-53	postive
CD247	LINC02446	0.54328684	6.88E-40	postive
LCP2	LINC02446	0.538096294	5.05E-39	postive
HCST	LINC02446	0.604169767	2.88E-51	postive
CD48	LINC02446	0.588235594	4.70E-48	postive
CD244	LINC02446	0.69211796	8.22E-73	postive
SH2D1A	LINC02446	0.61955498	1.52E-54	postive
GZMB	LINC02446	0.828385393	6.15E-128	postive
PRF1	LINC02446	0.775492888	6.30E-102	postive
CD3D	LINC02446	0.72028596	1.87E-81	postive
CD3E	LINC02446	0.590860271	1.43E-48	postive
CD3G	LINC02446	0.567593616	3.78E-44	postive
GRAP2	LINC02446	0.510469242	1.16E-34	postive
TRAC	LINC02446	0.562261734	3.49E-43	postive
TRAV4	LINC02446	0.627360372	2.80E-56	postive
TRAV8-3	LINC02446	0.505267692	6.95E-34	postive
TRAV8-6	LINC02446	0.527367021	2.78E-37	postive
TRAV12-2	LINC02446	0.613866698	2.59E-53	postive
TRAV12-3	LINC02446	0.507831602	2.89E-34	postive
TRBC1	LINC02446	0.509223799	1.79E-34	postive
TRBC2	LINC02446	0.586068601	1.25E-47	postive
TRBV4-1	LINC02446	0.501366299	2.61E-33	postive
TRBV6-5	LINC02446	0.538676204	4.05E-39	postive
TRBV7-9	LINC02446	0.591753625	9.51E-49	postive
TRBV9	LINC02446	0.726411042	1.78E-83	postive
TRBV12-4	LINC02446	0.574362461	2.13E-45	postive
TRBV19	LINC02446	0.501587694	2.42E-33	postive
TRBV28	LINC02446	0.529111365	1.46E-37	postive
TRDC	LINC02446	0.558330148	1.75E-42	postive
NFKBIB	AC002398.1	0.530508228	8.74E-38	postive
PDIA2	AC108488.1	0.65294637	2.51E-62	postive
CRLF1	AC108488.1	0.568812935	2.26E-44	postive
SLC22A17	AC004908.1	0.561900128	4.05E-43	postive
VTN	AC004908.1	0.554295662	8.95E-42	postive
CHGA	AC004908.1	0.727609357	7.06E-84	postive
GHRH	AC004908.1	0.729369863	1.80E-84	postive
GRP	AC004908.1	0.605277165	1.70E-51	postive
SCG2	AC004908.1	0.708400083	1.11E-77	postive
TRH	AC004908.1	0.705577965	8.19E-77	postive
NR0B2	AC004908.1	0.571083216	8.65E-45	postive
SHC2	AC004908.1	0.544862019	3.73E-40	postive
GNRH1	AC016773.2	0.585881174	1.35E-47	postive
NR2C1	AC016773.2	0.512396436	5.93E-35	postive

TNFRSF25	AC016773.2	0.589794139	2.32E-48	postive
PLCG1	AC016773.2	0.521357288	2.47E-36	postive
PCSK1	AC004034.1	0.57063937	1.04E-44	postive
VTN	AC004034.1	0.617510784	4.23E-54	postive
CHGA	AC004034.1	0.799240158	1.23E-112	postive
GHRH	AC004034.1	0.799051914	1.52E-112	postive
GRP	AC004034.1	0.54295291	7.83E-40	postive
SCG2	AC004034.1	0.784257219	1.01E-105	postive
TRH	AC004034.1	0.776700137	1.94E-102	postive
NR0B2	AC004034.1	0.710846364	1.93E-78	postive
SEMA3B	KRT7-AS	0.585277245	1.77E-47	postive
PSMC4	DRAIC	0.527724401	2.44E-37	postive
PROC	DRAIC	0.575465222	1.32E-45	postive
CALCA	DRAIC	0.66072264	2.78E-64	postive
GRP	DRAIC	0.622217101	3.93E-55	postive
THPO	DRAIC	0.730632114	6.69E-85	postive
NR0B2	DRAIC	0.591295749	1.17E-48	postive
MUC5AC	SRGAP3-AS2	0.609501467	2.21E-52	postive
BPIFB1	SRGAP3-AS2	0.679354699	3.24E-69	postive
BPIFA1	SRGAP3-AS2	0.575104671	1.54E-45	postive
DES	SRGAP3-AS2	0.550745038	3.70E-41	postive
SCGB3A1	SRGAP3-AS2	0.649024491	2.32E-61	postive
AZGP1	AC020765.2	0.548552462	8.80E-41	postive
PRDX2	AC020765.2	0.547993598	1.10E-40	postive
C3	AC008760.2	0.6249301	9.81E-56	postive
CMTM7	AC008760.2	0.554363927	8.71E-42	postive
CXCL2	AC008268.1	0.616859966	5.86E-54	postive
SFTPD	AC008268.1	0.537209088	7.07E-39	postive
A2M	AC008268.1	0.57299239	3.83E-45	postive
SFTPA1	AC008268.1	0.664952964	2.27E-65	postive
SFTPA2	AC008268.1	0.624092898	1.51E-55	postive
SLC11A1	AC008268.1	0.551390471	2.86E-41	postive
MARCO	AC008268.1	0.666202439	1.07E-65	postive
AGER	AC008268.1	0.918229472	2.91E-203	postive
CCL23	AC008268.1	0.622546447	3.33E-55	postive
SEMA3G	AC008268.1	0.526057648	4.50E-37	postive
EDNRB	AC008268.1	0.575873218	1.11E-45	postive
AGRP	AC008268.1	0.743032469	3.00E-89	postive
VEGFD	AC008268.1	0.859984401	3.46E-148	postive
GDF10	AC008268.1	0.608533292	3.53E-52	postive
RETN	AC008268.1	0.730454685	7.69E-85	postive
SCGB3A1	AC008268.1	0.510682587	1.08E-34	postive
ACVRL1	AC008268.1	0.565282659	9.96E-44	postive
IL3RA	AC008268.1	0.553260972	1.36E-41	postive
NPR1	AC008268.1	0.541668895	1.29E-39	postive
TEK	AC008268.1	0.532532767	4.11E-38	postive
CTSE	LRRK2-DT	0.642126008	1.07E-59	postive
CXCL2	LRRK2-DT	0.596160672	1.25E-49	postive
SFTPD	LRRK2-DT	0.855826016	2.99E-145	postive
COLEC12	LRRK2-DT	0.652437841	3.36E-62	postive
A2M	LRRK2-DT	0.691512377	1.23E-72	postive
SFTPA1	LRRK2-DT	0.827883635	1.19E-127	postive
SFTPA2	LRRK2-DT	0.817967631	3.74E-122	postive
PGC	LRRK2-DT	0.53534001	1.43E-38	postive
SLC11A1	LRRK2-DT	0.510023651	1.35E-34	postive
DMBT1	LRRK2-DT	0.700330802	3.16E-75	postive

DES	LRRK2-DT	0.54014721	2.31E-39	postive
AGER	LRRK2-DT	0.677684562	9.29E-69	postive
CCL23	LRRK2-DT	0.706881302	3.26E-77	postive
C3	LRRK2-DT	0.507302872	3.46E-34	postive
SEMA3G	LRRK2-DT	0.542322386	9.99E-40	postive
C5AR1	LRRK2-DT	0.509545094	1.60E-34	postive
ACKR1	LRRK2-DT	0.638303098	8.53E-59	postive
EDNRB	LRRK2-DT	0.532433993	4.27E-38	postive
VEGFD	LRRK2-DT	0.592013803	8.44E-49	postive
ACVRL1	LRRK2-DT	0.596909694	8.82E-50	postive
AGTR2	LRRK2-DT	0.654927006	8.08E-63	postive
CSF3R	LRRK2-DT	0.611549239	8.11E-53	postive
ENG	LRRK2-DT	0.512651731	5.43E-35	postive
IL3RA	LRRK2-DT	0.504671311	8.52E-34	postive
NPR1	LRRK2-DT	0.718631943	6.45E-81	postive
S1PR1	LRRK2-DT	0.67569746	3.22E-68	postive
SCTR	LRRK2-DT	0.70709782	2.80E-77	postive
TEK	LRRK2-DT	0.684024158	1.65E-70	postive
TGFBR2	LRRK2-DT	0.539573919	2.87E-39	postive
TIE1	LRRK2-DT	0.627449186	2.67E-56	postive
PLCG1	AC107375.1	0.550304457	4.40E-41	postive
PTPN11	AC026367.3	0.5453503	3.09E-40	postive
CKLF	GAPLINC	0.508221892	2.53E-34	postive
CTSS	MMP2-AS1	0.603408812	4.14E-51	postive
FCER1G	MMP2-AS1	0.517254519	1.07E-35	postive
IFI30	MMP2-AS1	0.561727173	4.35E-43	postive
CYBB	MMP2-AS1	0.539951349	2.49E-39	postive
CHIT1	MMP2-AS1	0.620553433	9.15E-55	postive
TLR8	MMP2-AS1	0.521603311	2.26E-36	postive
CCR5	MMP2-AS1	0.590851266	1.44E-48	postive
FGR	MMP2-AS1	0.605328844	1.65E-51	postive
LILRB3	MMP2-AS1	0.527269926	2.89E-37	postive
CCRL2	MMP2-AS1	0.500831695	3.12E-33	postive
CMKLR1	MMP2-AS1	0.503112836	1.45E-33	postive
IL10RA	MMP2-AS1	0.533614208	2.74E-38	postive
NR1H3	MMP2-AS1	0.611180799	9.72E-53	postive
ITGB2	MMP2-AS1	0.602438581	6.56E-51	postive
LCP2	MMP2-AS1	0.517319171	1.05E-35	postive
CTLA4	MMP2-AS1	0.502511931	1.77E-33	postive
FGFR3	AC107464.2	0.500729098	3.23E-33	postive
PLXNB1	AC073957.3	0.528007864	2.20E-37	postive
CD4	AL365361.1	0.568862273	2.22E-44	postive
CD74	AL365361.1	0.55594584	4.60E-42	postive
CTSS	AL365361.1	0.507233307	3.55E-34	postive
HLA-DMA	AL365361.1	0.574260968	2.22E-45	postive
HLA-DMB	AL365361.1	0.519122361	5.51E-36	postive
HLA-DOA	AL365361.1	0.547102067	1.56E-40	postive
HLA-DOB	AL365361.1	0.569278884	1.86E-44	postive
HLA-DPA1	AL365361.1	0.531118339	6.96E-38	postive
HLA-DPB1	AL365361.1	0.576272716	9.32E-46	postive
LTA	AL365361.1	0.503942342	1.09E-33	postive
CYBB	AL365361.1	0.531378718	6.32E-38	postive
TLR7	AL365361.1	0.503773928	1.16E-33	postive
CD40LG	AL365361.1	0.606962972	7.54E-52	postive
PDCD1	AL365361.1	0.553910142	1.04E-41	postive
CCR5	AL365361.1	0.522776511	1.48E-36	postive

CXCR4	AL365361.1	0.510243424	1.26E-34	postive
CCR4	AL365361.1	0.59177882	9.40E-49	postive
CD79A	AL365361.1	0.595092423	2.05E-49	postive
BTK	AL365361.1	0.701662113	1.26E-75	postive
VAV1	AL365361.1	0.556481684	3.70E-42	postive
PIK3R5	AL365361.1	0.545256322	3.20E-40	postive
INPP5D	AL365361.1	0.605651591	1.42E-51	postive
RASGRP3	AL365361.1	0.592992538	5.39E-49	postive
PLCG2	AL365361.1	0.524106915	9.16E-37	postive
PRKCB	AL365361.1	0.612012163	6.47E-53	postive
IGHA1	AL365361.1	0.509458246	1.65E-34	postive
IGHG1	AL365361.1	0.522157367	1.85E-36	postive
IGHJ3	AL365361.1	0.538539622	4.26E-39	postive
IGHV1-46	AL365361.1	0.504240729	9.87E-34	postive
IGHV2-70	AL365361.1	0.554659059	7.73E-42	postive
IGHV3-23	AL365361.1	0.528410971	1.90E-37	postive
IGHV3-30	AL365361.1	0.508220361	2.53E-34	postive
IGHV4-28	AL365361.1	0.541158499	1.56E-39	postive
IGHV4-34	AL365361.1	0.51787882	8.59E-36	postive
IGHV4-59	AL365361.1	0.510102463	1.32E-34	postive
IGHV5-51	AL365361.1	0.53832385	4.63E-39	postive
IGKC	AL365361.1	0.549483049	6.09E-41	postive
IGKJ5	AL365361.1	0.587106691	7.81E-48	postive
IGKV1-39	AL365361.1	0.516310926	1.50E-35	postive
IGKV1-5	AL365361.1	0.540768229	1.82E-39	postive
IGKV1-8	AL365361.1	0.525399286	5.72E-37	postive
IGKV3-11	AL365361.1	0.549836672	5.30E-41	postive
IGKV3-15	AL365361.1	0.514583046	2.76E-35	postive
IGKV3-20	AL365361.1	0.554973666	6.81E-42	postive
IGLC2	AL365361.1	0.535675301	1.26E-38	postive
IGLJ1	AL365361.1	0.517133653	1.12E-35	postive
IGLJ2	AL365361.1	0.503719225	1.18E-33	postive
IGLJ3	AL365361.1	0.51047588	1.16E-34	postive
IGLV1-40	AL365361.1	0.531791299	5.42E-38	postive
IGLV1-51	AL365361.1	0.54450207	4.29E-40	postive
CXCR3	AL365361.1	0.527893498	2.29E-37	postive
PLXNC1	AL365361.1	0.548967088	7.47E-41	postive
ADA2	AL365361.1	0.578252424	3.94E-46	postive
GMFG	AL365361.1	0.583047011	4.79E-47	postive
IL16	AL365361.1	0.663685289	4.82E-65	postive
CSF1R	AL365361.1	0.508729161	2.12E-34	postive
ENG	AL365361.1	0.587122613	7.76E-48	postive
IL10RA	AL365361.1	0.672866841	1.86E-67	postive
IL12RB1	AL365361.1	0.571196819	8.24E-45	postive
IL3RA	AL365361.1	0.577389767	5.74E-46	postive
PTGER2	AL365361.1	0.55189127	2.34E-41	postive
S1PR1	AL365361.1	0.5239096	9.84E-37	postive
TGFBR2	AL365361.1	0.549565407	5.90E-41	postive
TNFRSF17	AL365361.1	0.566616187	5.70E-44	postive
TNFRSF1B	AL365361.1	0.551807997	2.42E-41	postive
ICAM2	AL365361.1	0.666954999	6.82E-66	postive
ITGAL	AL365361.1	0.644134146	3.53E-60	postive
LCK	AL365361.1	0.517193027	1.10E-35	postive
NCR3	AL365361.1	0.558725635	1.49E-42	postive
CD247	AL365361.1	0.580969303	1.20E-46	postive
ZAP70	AL365361.1	0.538984219	3.60E-39	postive

LCP2	AL365361.1	0.540909722	1.72E-39	postive
FYN	AL365361.1	0.507664145	3.06E-34	postive
CD3D	AL365361.1	0.507861078	2.86E-34	postive
CD3E	AL365361.1	0.58494774	2.06E-47	postive
PTPRC	AL365361.1	0.665295921	1.85E-65	postive
ITK	AL365361.1	0.649520746	1.75E-61	postive
GRAP2	AL365361.1	0.570620519	1.05E-44	postive
CD28	AL365361.1	0.629361938	9.86E-57	postive
ICOS	AL365361.1	0.527973576	2.23E-37	postive
TRAC	AL365361.1	0.538361836	4.56E-39	postive
TRAV8-3	AL365361.1	0.549531009	5.98E-41	postive
TRAV8-6	AL365361.1	0.509999234	1.37E-34	postive
TRAV9-2	AL365361.1	0.537838865	5.57E-39	postive
TRBC1	AL365361.1	0.593531445	4.21E-49	postive
TRBC2	AL365361.1	0.56588854	7.73E-44	postive
TRBJ2-3	AL365361.1	0.513793813	3.64E-35	postive
TRBJ2-7	AL365361.1	0.534585461	1.90E-38	postive
TRBV2	AL365361.1	0.521221273	2.60E-36	postive
TRBV3-1	AL365361.1	0.509077274	1.88E-34	postive
TRBV5-1	AL365361.1	0.530848612	7.70E-38	postive
TRBV5-4	AL365361.1	0.528203654	2.05E-37	postive
TRBV6-1	AL365361.1	0.5074542	3.29E-34	postive
TRBV6-5	AL365361.1	0.537784926	5.68E-39	postive
TRBV18	AL365361.1	0.572036283	5.76E-45	postive
TRBV19	AL365361.1	0.552065353	2.19E-41	postive
TRBV20-1	AL365361.1	0.553317936	1.32E-41	postive
TRBV28	AL365361.1	0.581864412	8.08E-47	postive
TRBV29-1	AL365361.1	0.559834707	9.46E-43	postive
TNFRSF25	H1FX-AS1	0.521523379	2.33E-36	postive
GNRH1	AC004908.2	0.558631934	1.55E-42	postive
DDX17	AC015849.3	0.559505959	1.08E-42	postive
NR2C2	AC015849.3	0.558703874	1.50E-42	postive
SP1	AC073046.1	0.502851315	1.58E-33	postive
IREB2	AC073046.1	0.506904831	3.97E-34	postive
DEFA6	AC106795.2	0.522754912	1.49E-36	postive
VTN	AC106795.2	0.502211523	1.96E-33	postive
CHGA	AC106795.2	0.651058728	7.35E-62	postive
CHGB	AC106795.2	0.667096234	6.26E-66	postive
GHRH	AC106795.2	0.653188988	2.19E-62	postive
KL	AC106795.2	0.584276809	2.77E-47	postive
SCG2	AC106795.2	0.649131894	2.18E-61	postive
TRH	AC106795.2	0.633550891	1.09E-57	postive
NR0B2	AC106795.2	0.514759406	2.59E-35	postive
MC1R	AC105219.1	0.52871188	1.70E-37	postive
IGLV8-61	AC009133.1	0.503380595	1.32E-33	postive
SCTR	AC009133.1	0.636306261	2.50E-58	postive
S100A1	AL022328.4	0.514395035	2.95E-35	postive
CD4	AL357033.3	0.597287771	7.40E-50	postive
CD74	AL357033.3	0.594702326	2.45E-49	postive
CTSS	AL357033.3	0.512639986	5.45E-35	postive
HLA-DMA	AL357033.3	0.594318018	2.93E-49	postive
HLA-DMB	AL357033.3	0.513818371	3.61E-35	postive
HLA-DOA	AL357033.3	0.56631173	6.48E-44	postive
HLA-DPA1	AL357033.3	0.530980937	7.33E-38	postive
HLA-DPB1	AL357033.3	0.592984915	5.41E-49	postive
HLA-E	AL357033.3	0.559412515	1.12E-42	postive

CYBB	AL357033.3	0.505458003	6.52E-34	postive
TLR7	AL357033.3	0.515348075	2.11E-35	postive
CYLD	AL357033.3	0.501824861	2.24E-33	postive
CD40LG	AL357033.3	0.548074047	1.06E-40	postive
TRIM22	AL357033.3	0.542882443	8.05E-40	postive
PDCD1	AL357033.3	0.569939189	1.41E-44	postive
CCR5	AL357033.3	0.543155847	7.24E-40	postive
CCR4	AL357033.3	0.574259384	2.22E-45	postive
FGR	AL357033.3	0.504241288	9.86E-34	postive
BTK	AL357033.3	0.600217496	1.88E-50	postive
VAV1	AL357033.3	0.526276165	4.15E-37	postive
PIK3R5	AL357033.3	0.542391353	9.73E-40	postive
INPP5D	AL357033.3	0.593883117	3.58E-49	postive
FCGR2B	AL357033.3	0.500363575	3.66E-33	postive
RASGRP3	AL357033.3	0.562474421	3.19E-43	postive
PRKCB	AL357033.3	0.555171221	6.29E-42	postive
C3	AL357033.3	0.523150685	1.30E-36	postive
CMKLR1	AL357033.3	0.504672626	8.52E-34	postive
CXCR3	AL357033.3	0.53684048	8.13E-39	postive
PLXNC1	AL357033.3	0.51420632	3.15E-35	postive
ADA2	AL357033.3	0.536570157	9.01E-39	postive
IL16	AL357033.3	0.610394933	1.43E-52	postive
CSF1R	AL357033.3	0.546560028	1.92E-40	postive
ENG	AL357033.3	0.548602844	8.62E-41	postive
IL10RA	AL357033.3	0.644703799	2.58E-60	postive
IL12RB1	AL357033.3	0.576556498	8.24E-46	postive
S1PR1	AL357033.3	0.517224722	1.08E-35	postive
TGFBR2	AL357033.3	0.514258701	3.09E-35	postive
TIE1	AL357033.3	0.525886687	4.79E-37	postive
TNFRSF1B	AL357033.3	0.612289544	5.64E-53	postive
ICAM2	AL357033.3	0.533995463	2.38E-38	postive
ITGAL	AL357033.3	0.590653671	1.57E-48	postive
ITGB2	AL357033.3	0.536591181	8.94E-39	postive
CD247	AL357033.3	0.58416895	2.91E-47	postive
LCP2	AL357033.3	0.559295159	1.18E-42	postive
CD3E	AL357033.3	0.56040513	7.49E-43	postive
PTPRC	AL357033.3	0.610539178	1.33E-52	postive
ITK	AL357033.3	0.607580373	5.60E-52	postive
GRAP2	AL357033.3	0.522115998	1.88E-36	postive
CD28	AL357033.3	0.58093696	1.22E-46	postive
ICOS	AL357033.3	0.508145776	2.59E-34	postive
TRAC	AL357033.3	0.507561914	3.17E-34	postive
TRAV8-3	AL357033.3	0.513042059	4.73E-35	postive
TRAV8-6	AL357033.3	0.519168378	5.42E-36	postive
TRBC1	AL357033.3	0.547619114	1.27E-40	postive
TRBC2	AL357033.3	0.520704969	3.13E-36	postive
TRBJ2-7	AL357033.3	0.505215618	7.08E-34	postive
TRBV5-6	AL357033.3	0.513343477	4.26E-35	postive
TRBV18	AL357033.3	0.518627724	6.58E-36	postive
TRBV28	AL357033.3	0.529274855	1.38E-37	postive
TRBV29-1	AL357033.3	0.545272901	3.18E-40	postive
DDX17	AC114730.3	0.523557384	1.12E-36	postive
PLXNB1	AC114730.3	0.501627379	2.39E-33	postive
GNRH1	AC114730.3	0.581049995	1.16E-46	postive
TNFRSF25	AC114730.3	0.510451118	1.17E-34	postive
GNRH1	AL390728.6	0.583533819	3.86E-47	postive

GNRH1	AC020907.4	0.584164816	2.91E-47	postive
PLCG1	ADNP-AS1	0.516028208	1.66E-35	postive
ALB	AC139887.1	0.608520992	3.55E-52	postive
FGFR3	AC139887.1	0.847123445	2.15E-139	postive
EDN3	DLX6-AS1	0.572453571	4.82E-45	postive
FGF19	DLX6-AS1	0.546053948	2.35E-40	postive
DDX17	AC108010.1	0.51702784	1.16E-35	postive
NR2C2	AC108010.1	0.582880988	5.15E-47	postive
CD4	AP001189.3	0.572021222	5.80E-45	postive
THBS1	AP001189.3	0.564381368	1.45E-43	postive
SFTPD	AP001189.3	0.52817157	2.07E-37	postive
COLEC12	AP001189.3	0.645886847	1.34E-60	postive
A2M	AP001189.3	0.71882003	5.60E-81	postive
LRP1	AP001189.3	0.600874755	1.38E-50	postive
TLR7	AP001189.3	0.500155976	3.92E-33	postive
ELN	AP001189.3	0.508544224	2.26E-34	postive
DLL4	AP001189.3	0.53215901	4.73E-38	postive
PDGFRB	AP001189.3	0.6884071	9.54E-72	postive
ANXA6	AP001189.3	0.598575925	4.05E-50	postive
VIM	AP001189.3	0.510249135	1.25E-34	postive
LILRB3	AP001189.3	0.513927058	3.47E-35	postive
C3	AP001189.3	0.622197375	3.97E-55	postive
SEMA3G	AP001189.3	0.595050619	2.09E-49	postive
SEMA6B	AP001189.3	0.545419733	3.00E-40	postive
C5AR1	AP001189.3	0.560148298	8.32E-43	postive
ACKR1	AP001189.3	0.529733471	1.16E-37	postive
PLXNC1	AP001189.3	0.593522666	4.23E-49	postive
PLXND1	AP001189.3	0.560405948	7.49E-43	postive
CCN2	AP001189.3	0.519570526	4.70E-36	postive
FGF7	AP001189.3	0.532202195	4.65E-38	postive
LTBP2	AP001189.3	0.619282046	1.74E-54	postive
OGN	AP001189.3	0.596802693	9.27E-50	postive
TNFSF12	AP001189.3	0.50109166	2.86E-33	postive
ACVRL1	AP001189.3	0.715400672	7.03E-80	postive
ANGPTL2	AP001189.3	0.548027345	1.08E-40	postive
APLNR	AP001189.3	0.56408974	1.64E-43	postive
CSF1R	AP001189.3	0.520155748	3.81E-36	postive
ENG	AP001189.3	0.681876828	6.53E-70	postive
FLT1	AP001189.3	0.58811639	4.96E-48	postive
FLT4	AP001189.3	0.63232601	2.08E-57	postive
IL1R1	AP001189.3	0.607244471	6.58E-52	postive
IL3RA	AP001189.3	0.513679426	3.79E-35	postive
NPR1	AP001189.3	0.623043873	2.58E-55	postive
S1PR1	AP001189.3	0.628497022	1.55E-56	postive
TEK	AP001189.3	0.684476981	1.23E-70	postive
TGFBR2	AP001189.3	0.61184223	7.03E-53	postive
TIE1	AP001189.3	0.72667758	1.45E-83	postive
TNFRSF1B	AP001189.3	0.535068253	1.59E-38	postive
SEMA3B	AC092535.5	0.5202067	3.74E-36	postive
NRTN	AC092535.5	0.825037595	4.89E-126	postive
PDGFA	AC092535.5	0.542376413	9.79E-40	postive
CD74	AC004847.1	0.50071172	3.25E-33	postive
CTSE	AC004847.1	0.516088951	1.62E-35	postive
DDX17	ANKRD10-IT1	0.512645447	5.44E-35	postive
TSLP	SOCS2-AS1	0.590428059	1.74E-48	postive
PSMC4	SLC25A21-AS1	0.562230157	3.53E-43	postive

AKT2	SLC25A21-AS1	0.597026518	8.35E-50	postive
GRP	SLC25A21-AS1	0.59498787	2.15E-49	postive
ANGPTL1	SLC25A21-AS1	0.705924266	6.42E-77	postive
AZGP1	LINC00623	0.630887252	4.43E-57	postive
IL17RB	LINC00623	0.503734987	1.17E-33	postive
CXCL2	AC013457.1	0.63047191	5.51E-57	postive
MARCO	AC013457.1	0.535619894	1.29E-38	postive
AGER	AC013457.1	0.604020157	3.09E-51	postive
AGRP	AC013457.1	0.77820284	4.41E-103	postive
VEGFD	AC013457.1	0.691371904	1.35E-72	postive
GDF10	AC013457.1	0.558848231	1.42E-42	postive
RETN	AC013457.1	0.773025687	6.86E-101	postive
DDX17	AL928654.2	0.527915658	2.28E-37	postive
IRF9	AL928654.2	0.518329444	7.32E-36	postive
PLXNB1	AL928654.2	0.586691926	9.41E-48	postive
GNRH1	AL928654.2	0.53095593	7.40E-38	postive
GNRH1	AC005261.1	0.510228448	1.26E-34	postive
PSMD10	AL035425.2	0.807647775	8.86E-117	postive
COLEC10	AL035425.2	0.545110217	3.39E-40	postive
PSMD11	SAMD12-AS1	0.536879932	8.01E-39	postive
ZC3HAV1L	WNT5A-AS1	0.500563532	3.42E-33	postive
WNT5A	WNT5A-AS1	0.869123984	5.46E-155	postive
OSGIN1	WNT5A-AS1	0.602830877	5.45E-51	postive
CHGA	AC069224.2	0.625970022	5.74E-56	postive
GHRH	AC069224.2	0.623333674	2.22E-55	postive
SCG2	AC069224.2	0.632199574	2.22E-57	postive
TRH	AC069224.2	0.604365021	2.62E-51	postive
RELB	LINC00839	0.632223371	2.19E-57	postive
RFX5	LINC00839	0.505589245	6.23E-34	postive
VCAM1	LINC00839	0.558051546	1.96E-42	postive
CD70	LINC00839	0.577277145	6.03E-46	postive
CSF2RB	LINC00839	0.517165225	1.11E-35	postive
SSTR2	LINC00839	0.526575504	3.72E-37	postive
TNFRSF9	LINC00839	0.56065333	6.76E-43	postive
FAS	LINC00839	0.504536656	8.92E-34	postive
GNRH1	LINC00115	0.564651078	1.30E-43	postive
TNFRSF25	LINC00115	0.553347157	1.31E-41	postive
NENF	AC008443.5	0.520721274	3.11E-36	postive
LMBR1L	PTOV1-AS2	0.501490385	2.50E-33	postive
IRF3	PTOV1-AS2	0.655575571	5.56E-63	postive
DDX17	PTOV1-AS2	0.542251845	1.03E-39	postive
IRF9	PTOV1-AS2	0.520760141	3.07E-36	postive
PLXNB1	PTOV1-AS2	0.586215417	1.17E-47	postive
GNRH1	PTOV1-AS2	0.668897799	2.11E-66	postive
TNFRSF25	PTOV1-AS2	0.650441327	1.04E-61	postive
PLCG1	PTOV1-AS2	0.524515033	7.90E-37	postive
PDIA2	AC120498.2	0.698189641	1.37E-74	postive
IGHV3-48	AC120498.2	0.621792911	4.88E-55	postive
IGKV2D-30	AC120498.2	0.762901453	9.09E-97	postive
FGF18	AC120498.2	0.547080525	1.57E-40	postive
CRLF1	AC120498.2	0.63323042	1.29E-57	postive
LMBR1L	AL109811.2	0.500697948	3.27E-33	postive
DDX17	AL109811.2	0.500191478	3.87E-33	postive
GNRH1	AL109811.2	0.624365899	1.31E-55	postive
NR2C1	AL109811.2	0.557094686	2.89E-42	postive
TNFRSF25	AL109811.2	0.657235793	2.13E-63	postive

THBS1	AC037198.1	0.750485697	5.49E-92	postive
GNRH1	AC011468.1	0.546026182	2.37E-40	postive
DEFA6	AC104823.1	0.720373139	1.75E-81	postive
SERPIND1	AC104823.1	0.653209407	2.16E-62	postive
BMP4	AC104823.1	0.851486357	2.78E-142	postive
CHGB	AC104823.1	0.62223088	3.91E-55	postive
FGF3	AC104823.1	0.591255642	1.19E-48	postive
TNFRSF19	AC104823.1	0.55089994	3.47E-41	postive
DDX17	AL031714.1	0.514119694	3.24E-35	postive
PLXNB1	AL031714.1	0.562798745	2.79E-43	postive
GNRH1	AL031714.1	0.579677008	2.11E-46	postive
PLCG1	AL031714.1	0.525196216	6.16E-37	postive
AGTR2	MIR22HG	0.518982163	5.80E-36	postive
GNRH1	MIR222HG	0.531656912	5.70E-38	postive
PLXNB1	AC145423.3	0.523781941	1.03E-36	postive
AZGP1	AC245297.4	0.686673821	2.96E-71	postive
S100A13	AC245297.4	0.612870405	4.24E-53	postive
S100A1	AC245297.4	0.598727363	3.78E-50	postive
NFKBIB	LINC01534	0.519913442	4.16E-36	postive
PAK4	LINC01534	0.507729621	2.99E-34	postive
PLXNB1	AC018690.1	0.520146825	3.82E-36	postive
DDX17	AC120053.1	0.514126347	3.24E-35	postive
GNRH1	AC120053.1	0.623612075	1.93E-55	postive
TNFRSF25	AC120053.1	0.578611041	3.37E-46	postive
FABP7	AC011445.2	0.709402207	5.43E-78	postive
MAP3K14	AC011445.2	0.57833273	3.81E-46	postive
TRGJP2	AC011445.2	0.586068282	1.25E-47	postive
NR2C2	AC108449.2	0.564912987	1.16E-43	postive
AGER	FENDRR	0.637764621	1.14E-58	postive
SEMA3G	FENDRR	0.51927202	5.23E-36	postive
AGRP	FENDRR	0.522054441	1.92E-36	postive
VEGFD	FENDRR	0.631044467	4.08E-57	postive

Supplementary Table S4. DEirlncRNAs between LUSC and normal controls.

gene	conMean	treatMean	logFC	pValue	fdr
AC008268.1	17.97189968	0.235803219	-6.252015694	9.24E-33	4.01E-30
LHFPL3-AS2	9.889351147	0.597335291	-4.049263001	2.56E-30	2.22E-28
AC093110.1	8.663289026	0.713895122	-3.6011308	2.41E-30	2.22E-28
SFTA1P	82.0998836	3.723053453	-4.462821942	1.41E-30	2.22E-28
LRRK2-DT	10.04287541	0.802720563	-3.645130725	2.30E-30	2.22E-28
AL133355.1	4.440150717	0.934169489	-2.248852417	5.19E-30	3.30E-28
AC079467.1	5.439518647	0.330383794	-4.041264163	7.61E-30	3.30E-28
SMIM25	21.5404181	2.843117839	-2.921500452	6.50E-30	3.30E-28
AL162511.1	5.526684508	0.299971033	-4.203519162	7.57E-30	3.30E-28
FENDRR	8.2117461	0.553763408	-3.89034739	5.78E-30	3.30E-28
LINC02489	4.748758146	0.349808864	-3.762911529	1.06E-29	3.85E-28
AC026369.3	8.088500286	0.486493739	-4.055379091	9.86E-30	3.85E-28
RBPMS-AS1	4.651686561	0.708888109	-2.714124054	2.02E-29	5.86E-28
AL135999.3	3.738084543	0.246754304	-3.921152042	1.98E-29	5.86E-28
AP001189.3	4.510417375	0.538882986	-3.065216997	1.86E-29	5.86E-28
TMPO-AS1	0.253465	1.536741822	2.60001636	2.23E-29	6.04E-28
PARAL1	5.153048508	0.456779252	-3.495857147	4.78E-29	1.22E-27
TBX5-AS1	4.617314047	0.800211646	-2.52860033	7.31E-29	1.76E-27
KCNMB2-AS1	0.031997689	6.13426142	7.58277815	9.32E-29	2.13E-27
LINC01936	4.615948303	0.469579397	-3.297186051	1.21E-28	2.63E-27
LINC00261	4.484046571	0.460473082	-3.283612532	1.30E-28	2.68E-27
MAGI2-AS3	3.070080048	0.723527227	-2.085157059	1.83E-28	3.61E-27
LINC02471	4.700365676	0.441110506	-3.413560972	2.27E-28	4.29E-27
AC093278.2	3.727527145	0.751083728	-2.311173211	3.18E-28	5.75E-27
C8orf34-AS1	8.535276717	1.219767762	-2.806831435	6.16E-28	1.07E-26
AC090559.1	5.170396539	1.11719815	-2.210389841	8.55E-28	1.43E-26
HHIP-AS1	5.187326461	0.893806956	-2.53695599	5.33E-27	8.26E-26
DLX6-AS1	0.002955418	0.55722749	7.558760644	6.78E-27	1.01E-25
AL021707.2	0.22911712	0.947122752	2.047466152	7.45E-27	1.04E-25
AC109322.1	0.390786706	1.620021418	2.051559592	7.23E-27	1.04E-25
KDM4A-AS1	0.226349979	1.664349261	2.878331137	1.33E-26	1.75E-25
NKX2-1-AS1	2.440831027	0.482308769	-2.33934348	2.17E-26	2.78E-25
LINC02657	0.016357312	1.510662088	6.52910151	2.69E-26	3.33E-25
AC013457.1	9.499853925	0.369619034	-4.683794371	3.95E-26	4.76E-25
AC004540.2	2.170536189	0.486900056	-2.156353904	5.07E-26	5.94E-25
AL365361.1	5.003992047	1.102129834	-2.182785309	1.09E-25	1.22E-24
AC091849.2	2.774724431	0.610486532	-2.184313125	1.76E-25	1.91E-24
AC092071.1	3.415481808	0.29202789	-3.547911042	4.11E-25	4.25E-24
SLC12A9-AS1	0.278723219	1.66504229	2.578653723	1.39E-24	1.34E-23
ELN-AS1	5.599897985	0.880300466	-2.669332608	2.13E-24	2.01E-23
AC007996.1	0.509760005	2.922228464	2.519178882	2.96E-24	2.73E-23
LINC00626	0.011249591	2.787789274	7.953105137	4.54E-24	4.10E-23
AC114489.2	0.010319197	0.991628743	6.586397483	1.20E-23	1.00E-22
AC002116.2	0.293920383	1.230210995	2.065408458	3.30E-23	2.70E-22
C5orf66-AS1	0.006506729	2.229707835	8.420706522	3.52E-23	2.83E-22
SCAT2	0.163819699	1.053665888	2.685236718	6.46E-23	5.10E-22
AC083801.2	0.005372032	0.699601799	7.024922224	4.82E-22	3.61E-21
FEZF1-AS1	0.035483737	2.493097971	6.134637827	6.46E-22	4.59E-21
AL356299.3	0.103728017	0.66779285	2.686595021	9.97E-22	6.98E-21
MIR223HG	3.142687829	0.711666783	-2.142725167	1.26E-21	8.70E-21
LINC01705	0.064405469	0.889271029	3.787368082	2.24E-21	1.52E-20
AC018755.4	3.489320783	0.772036014	-2.176206181	3.00E-21	2.00E-20
AC079684.2	0.249417688	1.04324684	2.064444867	6.79E-21	4.40E-20
AP002954.1	2.014410891	0.448570395	-2.166951675	1.49E-20	9.49E-20
AC011005.4	0.060025303	0.691293127	3.52565489	1.82E-20	1.15E-19

AC078802.1	0.101793142	0.642920211	2.658999335	6.46E-20	3.60E-19
SOX2-OT	0.047648805	2.507356329	5.717583207	9.30E-20	5.11E-19
ANKRD44-AS1	5.836838608	0.818507915	-2.834118904	2.76E-19	1.48E-18
AC092115.4	0.06828315	0.640282403	3.229106851	3.29E-19	1.74E-18
HOTAIR	0.004664609	0.60790999	7.025957754	5.72E-19	2.99E-18
HOXC-AS1	0.043346456	0.919748343	4.407253209	8.24E-19	4.21E-18
AL109615.3	0.251825095	1.662720028	2.723051302	5.86E-18	2.83E-17
AC106795.2	0.13854982	0.776711253	2.486973532	8.98E-18	4.28E-17
LINC01614	0.427010494	3.371895969	2.981216598	1.28E-17	6.02E-17
AC114296.1	0.028109591	0.624826137	4.47432043	1.32E-17	6.18E-17
AC073365.1	0.007400879	2.405457037	8.344398636	1.59E-17	7.24E-17
AC004034.1	0.13675741	0.862690127	2.657223441	1.72E-17	7.78E-17
LINC00885	0.528358097	4.05052498	2.938520946	2.09E-17	9.26E-17
U62317.1	1.235710468	5.781975229	2.226221675	3.34E-17	1.43E-16
LINC02544	0.144971799	0.971933065	2.745084682	4.23E-17	1.78E-16
SRGAP3-AS2	6.690086829	0.665923031	-3.328597593	1.18E-16	4.85E-16
AC022144.1	0.318742882	1.34264407	2.074611874	1.73E-16	6.89E-16
AC092171.4	0.140211215	0.589432329	2.071724437	2.04E-16	8.05E-16
LINC02178	0.00885968	1.221783554	7.107518353	2.19E-15	7.86E-15
AL390719.2	0.700068469	3.6343276	2.376120536	4.06E-15	1.43E-14
AL033397.1	0.033626605	7.268181371	7.755847491	4.98E-15	1.74E-14
AC105219.1	0.10129101	0.857096212	3.08095102	2.85E-14	9.51E-14
AL022322.1	0.264256792	1.152791591	2.125119262	4.77E-14	1.53E-13
KC877982.1	0.00629298	2.07199719	8.363063062	6.90E-14	2.19E-13
AL357093.2	3.597925876	0.634936066	-2.502482231	2.46E-13	7.42E-13
AC120498.2	2.325971623	0.576883065	-2.011482678	3.46E-13	1.04E-12
AC010731.2	0.035205442	1.103984745	4.970777963	7.80E-13	2.27E-12
WNT5A-AS1	0.655488962	3.435324907	2.389803161	8.04E-12	2.18E-11
LINC02561	0.189024727	2.788566877	3.882876997	3.40E-11	8.89E-11
IRAIN	0.118642867	0.55586703	2.228114445	4.33E-11	1.12E-10
AL109976.1	0.18974725	0.915297945	2.270162467	6.36E-11	1.60E-10
LINC02677	0.052424421	1.591829683	4.924303157	1.18E-10	2.91E-10
UNC5B-AS1	0.328077589	1.473781433	2.16741363	1.99E-10	4.80E-10
AL590666.2	0.284263646	2.976202592	3.388171225	5.84E-10	1.38E-09
SAMD12-AS1	0.236524034	1.25340741	2.405796733	1.01E-09	2.33E-09
AC008011.2	0.155871199	1.493354055	3.260129969	1.54E-09	3.45E-09
AL161431.1	0.09989542	5.083847877	5.669358522	1.81E-09	3.98E-09
AL022316.1	0.117816595	0.62242853	2.401365423	8.20E-09	1.67E-08
AL365181.3	0.269823947	4.158310609	3.945907226	1.49E-08	2.95E-08
LINC02404	0.001527236	0.657254788	8.749386036	2.17E-08	4.18E-08
AC145207.8	0.299931	1.882101667	2.649642011	6.15E-08	1.12E-07
AC069120.1	0.020963124	0.623735292	4.895008287	6.24E-08	1.13E-07
AL035425.2	0.031240127	0.727449861	4.541375609	1.86E-07	3.26E-07
AP000851.2	0.03254026	0.622009285	4.256638422	2.76E-06	4.41E-06
AC083809.1	0.156290223	1.130354044	2.854475285	0.000228241	0.000317489
AL035425.4	0.02827214	1.653716427	5.870186949	0.001566743	0.002035828

Supplementary Table S5. The overlapping DEirlncRNAs between LUAD and LUSC.

AC013457.1
AC026369.3
AC083809.1
AC093110.1
AL022322.1
AL109615.3
AP001189.3
FENDRR
LINC01614
LINC01936
LINC02544
PARAL1
SCAT2
SMIM25
SRGAP3-AS2

Supplementary Table S6. Survival-related DEirlncRNA pairs in LUAD.

lncRNA pairs	HR	HR.95L	HR.95H	pvalue
LINC00941|AL590226.1	2.164666847	1.599183073	2.930110151	5.76E-07
ITGB1-DT|AC026355.2	2.217265146	1.607212101	3.058877371	1.23E-06
LINC02362|LINC00941	0.438211316	0.312807675	0.613888894	1.61E-06
ITGB1-DT|AL590226.1	2.071415853	1.534253916	2.796645062	1.99E-06
AC026355.2|LINC00941	0.453346028	0.324662217	0.63303523	3.42E-06
SNHG4|LINC01116	0.499935291	0.369760272	0.675938748	6.64E-06
LINC01116|DTNB-AS1	1.981266932	1.46968337	2.670928132	7.24E-06
LINC02036|AL049836.1	0.506036205	0.375629544	0.68171592	7.47E-06
ITGB1-DT|LINC02036	2.042930287	1.493444663	2.794589087	7.85E-06
ITGB1-DT|FENDRR	1.979103382	1.466533977	2.670821309	8.06E-06
LINC02036|LINC00941	0.487337317	0.353699607	0.67146713	1.10E-05
AC026355.2|AL049836.1	0.526986281	0.391725845	0.708951283	2.31E-05
PCAT19|LINC01116	0.51920729	0.383009338	0.703837176	2.42E-05
ITGB1-DT|LANCL1-AS1	1.904128909	1.411141074	2.569344036	2.52E-05
LINC00941|DTNB-AS1	2.00607003	1.447245086	2.780674127	2.93E-05
LINC02014|LINC00941	0.46001966	0.319439623	0.66246662	3.01E-05
LINC02195|LINC01116	0.529212236	0.392110389	0.714251903	3.19E-05
AC026355.2|AL606489.1	0.530008721	0.392181636	0.716273326	3.60E-05
AC026369.3|LINC01116	0.540239426	0.400558704	0.728628875	5.48E-05
ITGB1-DT|AC109446.3	1.844930669	1.370063007	2.484388788	5.49E-05
AL157838.1|LINC00941	0.508589741	0.36480368	0.709048564	6.66E-05
FENDRR|LINC00941	0.536672595	0.394630864	0.729840214	7.26E-05
AC026369.3|LINC00941	0.538882738	0.396167534	0.733009599	8.19E-05
AC109446.3|LINC00941	0.543565061	0.401327539	0.736214056	8.20E-05
AC026369.3|LINC01843	0.543813144	0.401097523	0.737308806	8.78E-05
AL606489.1|AC006017.1	1.984660156	1.407833031	2.797828896	9.14E-05
ITGB1-DT|AL157838.1	1.874161981	1.365804334	2.571732308	9.98E-05
LINC00942|LANCL1-AS1	1.869526519	1.363340064	2.563651945	0.00010281
AC004264.1|LINC02036	1.803071869	1.338777608	2.428385525	0.000104209
LINC02036|AL606489.1	0.553527899	0.410458627	0.74646533	0.000105984
LINC01843|LINC01936	1.79161708	1.330499407	2.412546555	0.000122623
LINC01843|LINC02362	1.792079101	1.329778875	2.415098904	0.00012699
AC138393.3|LINC00941	0.543027125	0.397299201	0.74220753	0.000128164
LINC01559|AC109446.3	1.881912081	1.359637821	2.60480624	0.000137683
ITGB1-DT|AC007014.2	1.810606396	1.334237387	2.457055658	0.000138314
AC026369.3|HIF1A-AS3	0.559529234	0.414728002	0.754887448	0.00014455
LINC00582|LINC01116	0.545648393	0.39921013	0.745803139	0.000144962
LINC01843|DTNB-AS1	1.7641723	1.312282678	2.371671865	0.000169912
AC026355.2|HIF1A-AS3	0.562765942	0.416741884	0.759956024	0.000176181
AC026355.2|AC026368.1	0.564224379	0.417842258	0.761888354	0.000187979
AC026355.2|LINC01116	0.569883528	0.423417322	0.767014525	0.000207287
AL606489.1|AL590226.1	1.895737421	1.349959135	2.662169746	0.000222367
LINC00941|U52111.1	1.806227615	1.315436951	2.480132699	0.000257456
LINC01559|FENDRR	1.808769986	1.314967359	2.488007661	0.000269314
LINC00958|LINC01116	0.534478538	0.381382053	0.749031857	0.000274581
AC026355.2|LINC01876	0.577470477	0.429063184	0.777209893	0.000291248
AL606489.1|LINC01936	1.740300279	1.289357996	2.348955893	0.000293666
AC026369.3|LINC01559	0.553179345	0.400858022	0.763380976	0.000314544
LINC00513|ITGB1-DT	0.573929233	0.423969999	0.776929419	0.000326263
AC026369.3|AC245041.2	0.577373062	0.427837431	0.779173648	0.000328748
AC004264.1|LINC00941	0.568467181	0.417326289	0.774345984	0.000341383
LINC02036|LINC01116	0.58063923	0.430993018	0.782244494	0.00035022
AC099850.4|SMIM25	1.782036474	1.291520308	2.458849448	0.000435785
LINC00942|AL590226.1	1.711181633	1.268027756	2.309210163	0.00044341
AC006017.1|LINC00941	0.578409898	0.426062929	0.785231448	0.000447917
LINC01843|PCAT19	1.724634174	1.272080855	2.338187093	0.000448645
LINC01116|LINC02154	2.049136402	1.371453832	3.061685269	0.000462227
ITGB1-DT|AC063965.2	1.697848509	1.261398275	2.285312749	0.000479939
AC114488.1|ITGB1-DT	0.589664162	0.437709802	0.794370658	0.000512662
ITGB1-DT|AC131971.1	1.854918847	1.307480206	2.631568657	0.000535329

ITGB1-DT|EGOT	1.690806662	1.255122754	2.277727147	0.000551048
LINC01843|LINC02036	1.688056508	1.253877364	2.272578528	0.000557936
AC026355.2|AC245041.2	0.587514047	0.434352972	0.794682614	0.00055805
AC037198.1|LINC01936	1.696205182	1.256115968	2.290482798	0.000565036
LINC02036|LINC00973	0.56585384	0.409359009	0.782175452	0.00056621
LINC02195|LINC00941	0.559660416	0.402123739	0.778913928	0.000578794
LINC01116|LANCL1-AS1	1.865962356	1.307743589	2.662460395	0.000583223
LINC02014|AL049836.1	0.571398524	0.415069198	0.786606847	0.000599703
AC011511.5|LINC01116	0.589691428	0.435963236	0.797626846	0.000609766
LINC01843|AC093110.1	1.685704589	1.250362812	2.272620342	0.000612963
LINC02362|LINC01116	0.596961926	0.444277351	0.802119533	0.000619493
ITGB1-DT|AC004264.1	1.708842984	1.257128824	2.322868022	0.000624051
AP003119.2|ITGB1-DT	0.556958426	0.3974226	0.780536103	0.000676563
AL031058.1|AC026355.2	1.809794125	1.283658362	2.551578265	0.000712295
LINC00942|LINC01936	1.718182391	1.254182735	2.353844177	0.000751286
AC022211.2|LINC00941	0.571953924	0.412959467	0.792163197	0.00077387
AL157838.1|AL606489.1	0.584777012	0.427661303	0.799614443	0.00077737
ITGB1-DT|AL096865.1	1.697124377	1.246245441	2.31112673	0.000787443
LINC01843|AL590226.1	1.718417435	1.252422593	2.357797199	0.000794877
LINC01843|AC026355.2	1.660446569	1.234320682	2.233684366	0.000804425
ITGB1-DT|U52111.1	1.696193531	1.243517265	2.313657057	0.000850112
ITGB1-DT|AC138393.3	1.675012038	1.236753224	2.268573287	0.000859273
LINC02154|LINC00941	0.60264395	0.447146248	0.812216881	0.00088136
AC026369.3|LINC00942	0.602571922	0.446959989	0.812361129	0.000889373
PARAL1|LINC01116	0.586372675	0.427905663	0.803525038	0.000897589
AL136115.2|FENDRR	1.657180873	1.227016552	2.238151099	0.000987027
SCAT2|LINC00941	0.574889721	0.413490604	0.799288275	0.000993338
LINC00941|AL645608.8	1.652727159	1.225388188	2.229095309	0.00099627
AC138393.3|AL606489.1	0.555862582	0.391675451	0.788875611	0.00101035
FENDRR|HIF1A-AS3	0.593946104	0.435246215	0.810511298	0.001021618
AP003119.1|AC026355.2	1.696784415	1.234654696	2.331888714	0.00111653
PCAT19|AL606489.1	0.607448016	0.450096833	0.81980824	0.001118595
LINC00958|HIF1A-AS3	0.572081635	0.408528104	0.801113543	0.001151102
LINC02195|LINC00942	0.603224215	0.444610581	0.818422839	0.001165371
FENDRR|LINC00942	0.609855463	0.452159244	0.822550218	0.001196702
AC133644.1|AC026355.2	1.632249087	1.210203718	2.201478181	0.001327882
AC245041.2|LINC01936	1.700745606	1.229501478	2.352608492	0.001336464
ITGB1-DT|AC026368.1	1.640857361	1.212428176	2.220678248	0.0013383
LINC01116|AC093110.1	1.63580612	1.210364983	2.210789061	0.001363484
AC011511.5|LINC00941	0.597801455	0.436310652	0.819064531	0.001363693
AC026355.2|LINC01614	0.55633892	0.388406382	0.796879271	0.001381761
LINC01843|LINC02195	1.621152233	1.203858076	2.183093353	0.001463465
AC026355.2|SNHG4	0.595052192	0.432159962	0.819342703	0.001468057
LINC01843|SNHG4	1.641643018	1.209230356	2.228683548	0.001483083
AC109446.3|LINC00942	0.617725679	0.458795011	0.831711343	0.001502523
LINC00582|LINC00941	0.606199438	0.444983111	0.825824057	0.001507925
LINC01843|LINC00582	1.666623308	1.215327421	2.285501999	0.001522614
LINC02362|LINC00942	0.612457351	0.451673923	0.830475232	0.001601977
ITGB1-DT|AL109761.1	1.611251489	1.197037608	2.168796822	0.00165415
LINC02036|AL162724.1	0.56506489	0.395701803	0.806916541	0.001688538
LINC01843|EGOT	1.772284935	1.239721851	2.533627916	0.001698431
LINC01843|AL645608.8	1.696141958	1.21918312	2.359692728	0.001710397
LINC01116|AL590226.1	1.62980303	1.201068811	2.211578462	0.001710651
HIF1A-AS3|LINC01936	1.613016987	1.196381473	2.174744311	0.001712312
UBE2Q1-AS1|LINC00941	0.606605221	0.442596691	0.831388715	0.001882779
ITGB1-DT|AC037198.1	1.621972971	1.192858849	2.205454836	0.002037029
ITGB1-DT|UBE2Q1-AS1	1.628326025	1.194512501	2.219688485	0.002039765
ITGB1-DT|PARAL1	1.60083319	1.187106902	2.158749896	0.002040564
AL136115.2|AC026355.2	1.722543236	1.218102548	2.435882928	0.002098486
AC011511.5|ITGB1-DT	0.620635481	0.457943555	0.841126371	0.00210198
ITGB1-DT|AC006017.1	1.599164624	1.184356714	2.159254441	0.002181468
LINC01607|LINC00941	0.569341932	0.396973644	0.816553543	0.002202607

AP003119.2|LINC00941	0.601354885	0.434137316	0.832979992	0.002218902
LINC01843|PARAL1	1.63472093	1.192730378	2.240500089	0.002244681
LINC01842|DTNB-AS1	1.589227719	1.180308458	2.139817541	0.002271591
LINC01559|AC004884.2	1.626120939	1.189947698	2.222172716	0.002277437
LINC00958|LINC01559	0.61749367	0.452799606	0.842090911	0.002320458
AL136115.2|AC011511.5	1.610212622	1.184101229	2.189664722	0.002385823
LINC00958|LINC01843	0.581466146	0.409706631	0.825231651	0.002402867
AC245041.2|AL590226.1	1.588110087	1.1774796	2.141942541	0.00244309
AC026355.2|UBE2Q1-AS1	0.627708056	0.464366417	0.848505381	0.002459802
AL606489.1|AL645608.8	1.660089862	1.195290556	2.305630488	0.002491567
FENDRR|LINC01116	0.620545275	0.455323635	0.845720294	0.002521074
LINC01559|SRGAP3-AS2	1.602684489	1.179997332	2.176782524	0.002531867
AC138393.3|LINC00942	0.632226023	0.468933303	0.852380802	0.002632332
AC138393.3|LINC01116	0.619359939	0.453255978	0.846335742	0.002636088
AC133644.1|LINC02362	1.58916812	1.172975857	2.153032647	0.002792397
UBE2Q1-AS1|AL606489.1	0.621407462	0.454785391	0.84907572	0.002815447
ITGB1-DT|SCAT2	1.640289191	1.185288215	2.269953076	0.002831623
LINC01559|LINC00582	1.623425192	1.180856681	2.23186217	0.00284869
AL049836.1|LINC01936	1.567173038	1.165718444	2.106882106	0.002925027
AC037198.1|LANCL1-AS1	1.718651181	1.202468826	2.456414517	0.002961094
PABPC4-AS1|ITGB1-DT	0.638002519	0.4740908	0.858584925	0.003013724
LANCL1-AS1|AC022211.2	0.517749611	0.334985543	0.800227549	0.003044983
LINC01843|U52111.1	1.576687347	1.166316883	2.131447317	0.003074536
LINC01116|AC124319.1	1.564912191	1.16246307	2.106690723	0.00315262
AL136115.2|AC026369.3	1.610340912	1.172076773	2.212481224	0.003286433
LINC02036|LINC00942	0.627010878	0.459087276	0.856357085	0.003335971
ITGB1-DT|AL645608.8	1.558079451	1.157684734	2.096953951	0.003432077
AC114488.1|LINC00941	0.64045216	0.475117911	0.863320368	0.003448864
AC026369.3|LUCAT1	0.628730709	0.46067253	0.858098278	0.003451507
AC010547.2|AL590226.1	1.604779007	1.167797221	2.205276407	0.003540644
LINC01765|LINC01116	0.596012557	0.420910301	0.843958837	0.003546764
LINC02362|LINC01614	0.580410359	0.402534225	0.836888302	0.003572491
LUCAT1|AL590226.1	1.606686766	1.167547752	2.210995105	0.003603696
PARAL1|AL606489.1	0.619095447	0.448227962	0.855098756	0.003614711
AC010547.2|DTNB-AS1	1.650885442	1.177734533	2.314123146	0.003621467
AC133644.1|AC006017.1	1.586354513	1.162187768	2.165330519	0.003651554
AL096865.1|LINC00941	0.617149621	0.445608355	0.854727364	0.003676495
AC073316.2|AC022211.2	0.613813794	0.441150668	0.854055997	0.003778667
AC026355.2|LUCAT1	0.64468048	0.478932407	0.86779035	0.003789773
LINC02544|LINC00941	0.61995609	0.448270345	0.857396788	0.003853059
AC026369.3|ITGB1-DT	0.638687979	0.470626947	0.866763659	0.004004936
FENDRR|AL606489.1	0.622239224	0.449857986	0.860675289	0.004150568
LINC00958|LUCAT1	0.607954908	0.432550651	0.854487606	0.004164859
FENDRR|AC026368.1	0.642577136	0.474542574	0.870112396	0.004242471
FENDRR|LUCAT1	0.629066654	0.457646565	0.864695346	0.004295642
LINC01559|AL109761.1	1.586044099	1.155079676	2.177802914	0.004356352
AP001429.1|ITGB1-DT	0.641882305	0.473240389	0.870620732	0.004359923
AC011511.5|LINC01614	0.548269603	0.36258413	0.829047749	0.00439158
ITGB1-DT|AP001189.3	1.61716754	1.160996521	2.252574237	0.004471436
LINC01607|AL606489.1	0.645019326	0.476688253	0.872792498	0.004486661
AC073316.2|LINC01842	0.635600351	0.464691212	0.869368295	0.00456822
AP001189.3|LINC00941	0.61206823	0.43584578	0.859541459	0.004602478
LANCL1-AS1|AL049836.1	0.589340591	0.408787584	0.849640122	0.004611451
AC026355.2|AC007014.2	0.647836148	0.479656936	0.87498302	0.004642669
AC010547.2|LANCL1-AS1	1.53605149	1.141136645	2.067635099	0.004645136
AC010547.2|FENDRR	1.571997402	1.14902754	2.150667191	0.004674614
LINC01842|LINC02036	1.538414166	1.141264286	2.073768691	0.004695059
AC022211.2|DTNB-AS1	1.537689734	1.140750703	2.072748858	0.004737664
LINC00942|AC037198.1	1.533120579	1.139551102	2.062618085	0.004757601
PABPC4-AS1|AL606489.1	0.585253431	0.403460276	0.848959859	0.004761239
HIF1A-AS3|AL590226.1	1.555798853	1.14405821	2.115722828	0.004831948
AC010547.2|AC006017.1	1.557510059	1.144304812	2.119922557	0.00484837

LANCL1-AS1|LINC00941	0.652516938	0.484590154	0.878636	0.004919349
AC006017.1|U52111.1	0.628704025	0.454763315	0.869174664	0.004978145
AL157838.1|LINC00942	0.646218078	0.476395551	0.876577885	0.005003889
LINC01116|LINC01936	1.528139088	1.135702577	2.05618013	0.005105662
LINC02362|AL049836.1	0.656135205	0.488299662	0.881658214	0.005181391
AC026369.3|AC133644.1	0.655502226	0.487477656	0.881441771	0.005187895
AC124319.1|LINC00941	0.621231017	0.444912784	0.867423886	0.005189726
PARAL1|LINC00941	0.654150236	0.485654748	0.881104391	0.005223257
AC114488.1|LINC01843	0.612918203	0.434473692	0.864652409	0.005298267
LINC01843|LINC02014	1.555181995	1.139900059	2.121757096	0.005334745
AL590226.1|LINC01614	0.563555426	0.376177529	0.844268182	0.005422222
LINC01559|LANCL1-AS1	1.569786915	1.142180951	2.1574786	0.005447218
AC109446.3|HIF1A-AS3	0.638507928	0.465092723	0.876582999	0.005525993
LINC01843|LINC02154	1.704323881	1.169309693	2.484132225	0.005542913
AL606489.1|LANCL1-AS1	1.739717626	1.176366327	2.572852816	0.005544508
AP003119.2|AL590226.1	1.589081038	1.145179218	2.205050971	0.005588242
AC109446.3|LINC01116	0.638743255	0.465124303	0.877169701	0.005609918
AL606489.1|DTNB-AS1	1.521548771	1.130406433	2.04803387	0.005632121
AC133644.1|LINC01936	1.534515502	1.132999305	2.078322394	0.005661705
AC026369.3|AL109615.3	0.624945622	0.447520855	0.872712469	0.005796997
AC010547.2|LINC02036	1.603966418	1.146361288	2.244238616	0.005833266
LINC00958|LINC01614	0.551596616	0.361137041	0.842502409	0.005905264
AC063965.2|LINC00941	0.659788581	0.490610435	0.887304756	0.005942155
PARAL1|LINC00973	0.656347002	0.486152777	0.886123472	0.005970736
LINC00958|LINC00942	0.652426698	0.480976074	0.884993288	0.006044063
LINC01614|LINC01936	1.639881276	1.151958831	2.334467629	0.006050011
LINC02036|UBE2Q1-AS1	0.657357973	0.486888942	0.887511437	0.006160815
LINC02036|AL353804.2	0.630582146	0.453333498	0.87713316	0.006171135
AL353804.2|LINC00941	0.659415115	0.489320669	0.888636678	0.006226212
LINC01559|AC004264.1	1.588489354	1.139996796	2.213425896	0.006256288
LINC00582|LINC00942	0.659243139	0.488866919	0.888997597	0.0063098
AL606489.1|AC022211.2	1.510578261	1.123282779	2.031408943	0.006350101
ITGB1-DT|LINC01655	1.546106649	1.1297874	2.11583681	0.006481638
SRGAP3-AS2|LINC01116	0.643007702	0.467858059	0.883727226	0.006492287
AL512413.1|LINC00941	0.628251887	0.449531894	0.878025429	0.006496331
LINC02036|AC004884.2	0.631753388	0.453773715	0.879540463	0.006523641
LINC02036|EGOT	0.63078614	0.452569987	0.879181488	0.006526877
LINC01842|LINC02362	1.506602906	1.12061312	2.025545013	0.006646794
AC011511.5|AL606489.1	0.647444946	0.472901837	0.886410088	0.00668306
AL353804.2|AC006017.1	1.562567578	1.13178226	2.157320821	0.006683371
LINC00942|AC006017.1	1.508885548	1.120764765	2.031412538	0.006699231
AC114488.1|LINC01116	0.638720095	0.461667958	0.883672675	0.006796813
AC114488.1|LINC01559	0.648463013	0.473737503	0.887631393	0.006848802
ITGB1-DT|MIR3945HG	1.529836291	1.124007586	2.082191533	0.006866826
ITGB1-DT|LINC02544	1.544900221	1.126090013	2.119472391	0.007017219
AL136115.2|AC138393.3	1.552094487	1.127483206	2.136614792	0.007022907
LINC02362|AC022211.2	0.662068797	0.490515806	0.893620728	0.007038964
AL031058.1|LINC01936	1.570080455	1.130456341	2.180670358	0.007111856
LINC01936|LINC00973	0.631079191	0.451281839	0.882510463	0.007135272
AC073316.2|EGOT	0.661581874	0.489393284	0.894353459	0.00723403
LINC01843|FENDRR	1.535340198	1.122623948	2.099785532	0.007273108
LINC02036|LINC01214	0.641576589	0.46376971	0.887553693	0.007354361
LINC01843|LINC01765	1.596511666	1.133880169	2.247900236	0.007369803
FENDRR|AC245041.2	0.661742501	0.489177012	0.895183394	0.007401625
LINC02544|LINC02362	1.506494083	1.115666148	2.034232575	0.007489859
LINC02014|AC026355.2	1.562188194	1.126469478	2.166443034	0.00750073
PARAL1|LINC01842	0.662985933	0.490492308	0.896141163	0.007513523
AC083809.1|LINC01765	1.524086458	1.118763192	2.076256663	0.007553638
LINC01843|AC007014.2	1.53173516	1.119872344	2.095071472	0.007619597
LINC02036|LUCAT1	0.668243644	0.496867842	0.898729058	0.007671827
LINC02362|HIF1A-AS3	0.667577734	0.495780894	0.898905213	0.007766435
LINC02362|AL606489.1	0.666543487	0.494382724	0.898656444	0.00779364

AL096865.1|LINC02036	1.512090958	1.11450683	2.05150745	0.007896918
AC026368.1|LINC00941	0.657269379	0.482112765	0.896062225	0.007954058
LINC02036|LINC01876	0.666176807	0.493355557	0.89953692	0.00802727
AC026355.2|AL109615.3	0.653527218	0.47717207	0.8950604	0.008028866
AC114488.1|LINC01936	1.537744981	1.118653985	2.113843654	0.008034209
HIF1A-AS3|LANCL1-AS1	1.633907397	1.136277217	2.34947365	0.008064478
AL109761.1|LINC00942	0.66182587	0.487240461	0.898967793	0.008251182
UBE2Q1-AS1|LINC01936	1.498151538	1.109778699	2.022437475	0.00828313
FENDRR|AL109615.3	0.612841026	0.426066239	0.881492333	0.008288755
LINC01765|AL606489.1	0.595846406	0.405494558	0.875555373	0.008370785
AC083809.1|AL590226.1	1.488719617	1.107045004	2.00198374	0.008467724
LINC02036|AC026368.1	0.671449756	0.499039782	0.903424519	0.008519563
AP001189.3|LINC01614	0.591362388	0.399626771	0.875090206	0.008607378
AP003119.2|AC026369.3	1.4955271	1.107554488	2.019405213	0.008623253
AC016773.2|AL606489.1	0.672126453	0.499605531	0.904221312	0.008659465
LINC01559|PARAL1	1.530310693	1.113436106	2.103264665	0.008736779
LINC01842|AL590226.1	1.486364578	1.104406916	2.00042179	0.008915793
AL031058.1|PCAT19	1.503298311	1.107111407	2.041263235	0.009003821
AC011511.5|LINC01936	1.498067532	1.106018525	2.029085662	0.00903052
LINC00958|LINC00941	0.671576846	0.497773364	0.906065877	0.00917315
LINC00513|LINC00942	0.672349676	0.498625679	0.906600092	0.009244459
AC013457.1|AL109615.3	0.511272546	0.308426545	0.847526327	0.009281765
AC007014.2|AL606489.1	0.64242172	0.460220092	0.896757168	0.009314242
AL022322.1|AL606489.1	0.672892085	0.499074802	0.907246281	0.00936572
LINC00582|AL049836.1	0.670379017	0.495714081	0.906587173	0.009411046
LINC01559|LINC01765	1.485461642	1.100539862	2.005012602	0.009709458
AL109761.1|AL606489.1	0.62453026	0.437102106	0.89232708	0.009718082
AL022322.1|LINC00941	0.643606252	0.460813868	0.898907425	0.009732533
LINC01843|AL096865.1	1.496890283	1.101846513	2.033568643	0.009869641
LINC02036|AC007014.2	0.664099501	0.48661946	0.906310132	0.009879202
LINC01842|EGOT	1.489149326	1.099103253	2.017613639	0.010176449
ITGB1-DT|AL162724.1	1.504550382	1.101216519	2.055610149	0.010303097
LINC01607|LINC01843	0.671037856	0.49471045	0.910212839	0.010323416
LINC02154|HIF1A-AS3	0.625210931	0.436615183	0.895270536	0.010350817
LINC01765|AC022211.2	0.614679832	0.423279399	0.892628597	0.010567181
AL049836.1|DTNB-AS1	1.474299845	1.094501838	1.985889797	0.010646281
LINC01843|AL157838.1	1.476901299	1.094931208	1.99212282	0.010650949
LINC02362|SCAT2	0.67610415	0.500578752	0.913176638	0.010704507
FENDRR|AL049836.1	0.671225539	0.494076151	0.911891261	0.010774295
LINC00513|LINC00941	0.671982211	0.494747357	0.912708448	0.010938849
LINC01843|LINC02544	1.471703356	1.092599309	1.982346821	0.011000045
AP001429.1|AL590226.1	1.522031861	1.100133488	2.10572718	0.011207485
AC063965.2|LINC00942	0.674002782	0.496552182	0.914868099	0.011383238
MIR3945HG|AL606489.1	0.647911182	0.462576936	0.907500716	0.011584272
AL136115.2|AL590226.1	1.482391222	1.091837791	2.012646708	0.011632176
AC026355.2|MIR3945HG	0.672418262	0.494011192	0.915255214	0.011639836
AL512413.1|ITGB1-DT	0.650581276	0.465649037	0.908959243	0.011756105
AC026369.3|EGOT	0.673074062	0.494641007	0.915873709	0.011764597
PABPC4-AS1|LINC00941	0.679269849	0.502661887	0.917928213	0.011822534
AC026355.2|AC022211.2	0.683842398	0.508316919	0.919978083	0.012036437
LUCAT1|LINC02362	1.474367095	1.088851431	1.996377349	0.012059596
AC133644.1|LINC00942	0.684362582	0.508767952	0.920561411	0.012171961
LINC00958|AC133644.1	0.659868992	0.476494164	0.913814103	0.01233134
AP001429.1|LINC00973	0.684565587	0.508638878	0.921341375	0.012401198
AC073316.2|LINC00941	0.684335226	0.508205813	0.921505993	0.012475363
AC037198.1|LINC00941	0.660959769	0.477630682	0.914656099	0.012483224
AL096865.1|AL606489.1	0.666959821	0.485200218	0.916807921	0.012595074
LINC00942|DTNB-AS1	1.484880373	1.088311057	2.025955454	0.012638467
AL606489.1|SCAT2	1.476851285	1.086566452	2.007322897	0.012767412
AC007014.2|LINC00941	0.662688008	0.479204307	0.916426229	0.012860191
AL031058.1|AC016773.2	1.565995631	1.09903182	2.231366073	0.013040952
LINC00958|LINC01842	0.679152932	0.500228621	0.922075797	0.013139385

LINC00513|AL606489.1	0.660576109	0.47596524	0.91679131	0.013158289
AC133644.1|LINC02036	1.461628527	1.082661931	1.973245654	0.01318889
LINC01842|LINC02154	1.507877999	1.089703875	2.086526542	0.013198907
AL512413.1|LANCL1-AS1	1.585258332	1.101038799	2.282429993	0.013228997
LINC00513|AC026355.2	1.465177214	1.082599891	1.982952598	0.013360894
LINC00582|HIF1A-AS3	0.676662203	0.496509465	0.922181286	0.013402887
LINC02544|AC026355.2	1.457914917	1.080734956	1.966731893	0.013576193
LINC00958|AL022322.1	0.61580761	0.419003973	0.905048727	0.013595182
ITGB1-DT|LINC01765	1.455319165	1.080153507	1.960789702	0.013630039
LINC01842|LINC01765	1.488444508	1.085144833	2.041632588	0.013634327
AL035409.1|AL096865.1	1.4796908	1.083753097	2.020280145	0.013655913
LINC02544|LINC02036	1.452946543	1.078924248	1.956628243	0.013885592
AC010547.2|SRGAP3-AS2	1.460968664	1.080098742	1.976142878	0.013895551
AL035409.1|AC109446.3	1.456984907	1.07942737	1.966602921	0.013916776
FENDRR|LINC00973	0.673916617	0.492051544	0.923000063	0.013921883
LINC01936|AL109615.3	0.679235372	0.499034852	0.924505952	0.013932434
LINC00582|AL109615.3	0.633721833	0.440032896	0.912666677	0.014246014
AC010547.2|LINC02154	1.450147056	1.077142958	1.952318835	0.014294231
AC026369.3|AL035409.1	0.678277818	0.496735884	0.926167835	0.014583384
LINC02036|AC124319.1	0.686429345	0.507379164	0.92866495	0.01469221
AC073316.2|LINC00942	0.684057474	0.504219058	0.928038361	0.014694126
LINC01116|AC037198.1	1.461218482	1.077065357	1.982386155	0.01481004
LANCL1-AS1|AL109615.3	0.587990383	0.383586977	0.901314984	0.014821376
AC083809.1|AC109446.3	1.44418291	1.074175783	1.941641499	0.014942386
AC073316.2|ITGB1-DT	0.692527677	0.514951516	0.931339298	0.015077002
AC026355.2|LINC01842	0.688052074	0.508786469	0.930480043	0.015188675
AC026368.1|LANCL1-AS1	1.501970328	1.081497077	2.085918598	0.015202995
LINC01607|AL049836.1	0.675167132	0.491546072	0.927381343	0.015287416
ITGB1-DT|LINC02154	1.449377901	1.073813835	1.956294686	0.015293001
LUCAT1|AL353804.2	1.601824718	1.094563564	2.344169414	0.015306843
LINC01843|AC013457.1	1.656759239	1.101109462	2.492805005	0.015433503
AC011511.5|LINC01559	0.659014047	0.470133305	0.923779511	0.015517691
LINC01559|AL590226.1	1.490550575	1.078695198	2.059655981	0.015560292
AC073316.2|LINC01559	0.691624882	0.512953116	0.932531576	0.015603263
LINC02036|HIF1A-AS3	0.691088188	0.512209289	0.932436984	0.015619064
AL606489.1|LINC02154	1.596979397	1.092465279	2.334484439	0.015670889
PCAT19|AL049836.1	0.672961293	0.488035787	0.927958387	0.01568946
LINC01843|AC109446.3	1.487756237	1.077530527	2.05415862	0.015794118
AC016773.2|LINC01116	0.692548783	0.513620443	0.933809825	0.015994984
AC073316.2|HIF1A-AS3	0.672637334	0.487129677	0.928789611	0.016010516
ITGB1-DT|SRGAP3-AS2	1.440867808	1.070240069	1.93984518	0.016066935
LINC01843|AC006017.1	1.473805729	1.074472499	2.021553208	0.016152249
LINC01116|LINC01655	1.438291748	1.069566179	1.934132916	0.016173556
LINC02362|MIR9-3HG	0.663280303	0.474648788	0.926876401	0.016184255
LINC01559|EGOT	1.469073814	1.073301862	2.010783683	0.016320537
LINC01842|LANCL1-AS1	1.459981807	1.071924431	1.98852346	0.016369984
AC016773.2|LUCAT1	0.696056959	0.517656914	0.935938993	0.016477468
HIF1A-AS3|DTNB-AS1	1.442392058	1.069147321	1.94593842	0.01650293
UBE2Q1-AS1|LINC02362	1.440733658	1.068685994	1.942304367	0.016583222
LINC02036|LINC01614	0.677206053	0.492229081	0.93169635	0.016638272
AC011511.5|LUCAT1	0.682706719	0.499461201	0.933182524	0.016681948
LINC00958|LINC01876	0.683629354	0.500488702	0.933785503	0.016822538
LINC01842|SNHG4	1.470371788	1.071901426	2.016970164	0.016824867
LINC01842|AL353804.2	1.461225297	1.070668744	1.994248345	0.016834307
LUCAT1|AC109446.3	1.484443672	1.072924954	2.053799764	0.01708336
LINC01559|LINC01876	1.476683063	1.071903938	2.034317434	0.01708962
LINC00958|AC010547.2	0.696924337	0.517646323	0.938292247	0.017324438
AL606489.1|AC037198.1	1.450649063	1.067731883	1.970890574	0.01735573
AC010547.2|AC138393.3	1.462224345	1.068706772	2.000642357	0.017530687
LINC01607|LINC01116	0.692759518	0.51164163	0.937991988	0.017598476
AC010547.2|U52111.1	1.489803388	1.071424103	2.071555165	0.017781554
LINC01559|AC138393.3	1.493717589	1.071873618	2.081581445	0.01779345

AC010547.2|AL353804.2	1.433699858	1.064050237	1.93176526	0.017882396
LINC02362|AL109615.3	0.678672623	0.492360595	0.935486175	0.017921037
LINC02195|MIR9-3HG	0.67795624	0.491135341	0.935841152	0.018121543
AC026355.2|AP001189.3	0.698440827	0.518602664	0.940642273	0.018136544
LINC00942|U52111.1	1.434512747	1.062524454	1.936733611	0.01847523
HIF1A-AS3|LINC00941	0.677629988	0.490055846	0.93700015	0.018597531
UBE2Q1-AS1|AC093110.1	1.495611124	1.069102065	2.092272298	0.018770138
AP001429.1|LINC02036	1.526909857	1.072527785	2.1737933	0.018850401
SNHG4|AL606489.1	0.688277044	0.503802885	0.94029888	0.018942716
LINC01607|LINC01876	0.687941292	0.503214376	0.940480328	0.019047251
AC026355.2|LINC00942	0.687171773	0.502003654	0.940640655	0.019182724
LUCAT1|LANCL1-AS1	1.520125985	1.070520249	2.158560768	0.019239286
LINC01607|LINC02036	1.454205065	1.062190153	1.990898112	0.019470086
AC016773.2|LINC00942	0.681457328	0.493880865	0.940275525	0.01954986
LUCAT1|LINC02154	1.566590571	1.073248357	2.286708387	0.020002965
AC011511.5|AL109615.3	0.659355348	0.464046446	0.936866294	0.020134553
AL035409.1|LINC02362	1.478042019	1.062215593	2.056652364	0.020446671
PARAL1|AC010547.2	0.6975627	0.514373412	0.945993142	0.020495038
AL031058.1|LINC01655	1.433122601	1.056722067	1.943595628	0.020619971
PARAL1|LINC01614	0.612337095	0.404235416	0.927570182	0.020623194
LINC01842|AC006017.1	1.424855311	1.054860342	1.924626965	0.020992231
LINC01843|MIR3945HG	1.44525291	1.056819229	1.976455307	0.021110922
AL353804.2|LINC00942	0.693620885	0.508155194	0.946777554	0.021195649
AC133644.1|AL353804.2	1.498329901	1.062213678	2.113503658	0.021231322
AC026368.1|AL606489.1	0.686104366	0.497961253	0.945332991	0.021236796
LINC00958|AL109615.3	0.621379011	0.414430868	0.931667751	0.021309045
AC114488.1|DTNB-AS1	1.451113808	1.056829417	1.992498743	0.021355028
PCAT19|LINC00942	0.670607954	0.47688739	0.94302143	0.021604258
AC073316.2|LINC01116	0.683063172	0.493400305	0.945632365	0.021629609
LINC01842|LINC01655	1.422846193	1.052967042	1.922653995	0.021676869
LUCAT1|LINC02195	1.41808308	1.052087796	1.911399057	0.021828884
NAALADL2-AS2|LINC00941	0.669708734	0.475402926	0.943430853	0.021846631
AC026355.2|AL353804.2	0.656687432	0.458321504	0.94090803	0.021910673
AL590226.1|AL109615.3	0.667086173	0.471871096	0.943062557	0.021915147
LINC02362|AC037198.1	0.706303749	0.524583305	0.950973813	0.021951513
ITGB1-DT|AC245041.2	1.416538299	1.051556929	1.90819983	0.021982686
AC004264.1|FENDRR	1.425396806	1.052138162	1.931073436	0.022134861
LINC01765|LINC00941	0.703828034	0.520727742	0.951310755	0.022333251
AC026369.3|AL031058.1	0.67959799	0.487772624	0.946862135	0.022451343
PCAT19|AC026355.2	1.449882191	1.05334003	1.995707281	0.022682977
AC093110.1|AL049836.1	0.699407362	0.514060404	0.951582058	0.022852502
AC010547.2|LINC01655	1.48169507	1.0554709	2.080038664	0.023091839
LINC02014|LINC01936	1.441433945	1.050726625	1.977423784	0.023407347
AC013457.1|LINC00942	0.682641565	0.490708724	0.949645856	0.023407755
ITGB1-DT|AC004884.2	1.409272252	1.047166779	1.896592138	0.023566447
LINC01116|HAGLROS	1.446181568	1.050688632	1.990543214	0.023616835
AP003119.2|LINC02154	1.528310968	1.058448726	2.20675254	0.023633748
AL353804.2|AL590226.1	1.426019235	1.04859665	1.93928796	0.023667372
LINC01116|U52111.1	1.415885014	1.047507619	1.913809825	0.023707001
AL512413.1|AL590226.1	1.439351164	1.049727717	1.973589665	0.023740994
AL136115.2|ITGB1-DT	0.710142472	0.527671502	0.955712651	0.023889415
LINC01842|AL022313.2	1.418247755	1.047192042	1.920781113	0.023949509
ITGB1-DT|AC022211.2	1.45410709	1.050411712	2.012951115	0.024047242
MIR3945HG|LINC01936	1.439935912	1.048959169	1.976640743	0.024090628
AL096865.1|HIF1A-AS3	0.707303533	0.5233386	0.955936152	0.024248157
AL391427.1|ITGB1-DT	0.710400122	0.527623027	0.95649414	0.024255349
AL590226.1|LINC00973	0.6978822	0.51013972	0.954717983	0.024461912
[truncated: 56,853 more chars]
